# Substance abuse among new patients attending main government hospitals in Malaysia from 2018–2021: A comparison between before and during COVID-19 pandemic

**DOI:** 10.1371/journal.pone.0309422

**Published:** 2024-10-24

**Authors:** Nor Asiah Muhamad, Norliza Chemi, Nur Hasnah Ma’amor, Izzah Athirah Rosli, Fatin Norhasny Leman, Mohd Fadzli Mohamad Isa, Mohammad Zabri Johari, Norni Abdullah, Nor Ashikin Ibrahim, Huan-Keat Chan, Muhammad Radzi Abu Hassan, on behalf of My Substance Abuse Study Group

**Affiliations:** 1 Sector for Evidence-Based Healthcare, National Institutes of Health, Ministry of Health, Selangor, Malaysia; 2 Department of Psychiatry, Kajang Hospital, Ministry of Health, Selangor, Malaysia; 3 Department of Psychiatry, Kuala Lumpur Hospital, Ministry of Health, Kuala Lumpur, Malaysia; 4 Institute for Behavioural Health Research, National Institutes of Health, Ministry of Health, Selangor, Malaysia; 5 Department of Psychiatry, Tengku Ampuan Rahimah Hospital, Ministry of Health, Selangor, Malaysia; 6 National Centre of Excellence for Mental Health, Cyberjaya, Malaysia; 7 Clinical Research Centre, Sultanah Bahiyah Hospital, Ministry of Health, Kedah, Malaysia; Monash University, MALAYSIA

## Abstract

**Background:**

Substance abuse admission to health facilities following the pandemic is often met with challenges. COVID-19 is causing an insurmountable psychosocial impact on the whole of mankind. Marginalized communities, particularly those with substance use disorders (SUDs), are also likely to suffer from greater psychosocial burden.

**Objectives:**

This study sought to evaluate substance abuse trends before and during the pandemic.

**Methods:**

A cross-sectional study was conducted among patients attending selected government hospitals in Malaysia. Data from the year 2018 to 2021 was utilized.

**Results:**

A total of 9,606 patients consisting of 7881 males and 1725 females were identified. Most of the patients involved with substance abuse from 2018 to 2021 were males, aged between 26 and 44 years old, Malays, high school students, singles, workers of private sectors and those residing in urban areas. The most abused substances over the four years were tobacco (61.8%), followed by amphetamine-type stimulants (ATS) (43.1%), alcohol (39.7%), cannabis (17.2%), opioids (13.0%), and kratom (8.8%). Those who worked in the private sector and were self-employed or unemployed were more associated with substance abuse during the pandemic compared to those who worked in the government sector. Those with a history of psychiatric illness were more associated with abuse of substances during the pandemic than those without the history (adjusted OR: 1.18, 95% CI 1.09–1.29, *p* <0.001).

**Conclusions:**

Targeted exploration of factors affecting substance abuse in Malaysia is essential. The results of this study assist in identifying variations in substance abuse treatment characteristics for those admitted to treatment in Malaysia.

## Introduction

Substance use disorders (SUDs) are a public health concern worldwide [1, 2]. They are defined as maladaptive patterns of substance use that cause clinically significant impairment or distress that occurs in both younger and older populations [3, 4]. SUDs are conditions where abuse or dependence on illicit drugs, with or without alcohol, is prevalent and associated with numerous adverse health and social consequences [5]. According to the Diagnostic and Statistical Manual of Mental Disorders, Fifth Edition (DSM-5), SUDs involve recurring symptoms caused by taking substances continuously despite its harmful effects [6]. SUDs include both substance dependence and substance abuse, that affect a person’s brain and behaviour, leading to an inability to control the use of substances such as illicit drugs and alcohol [7, 8]. SUDs have been associated with violent behaviour while under the influence of various drugs [9]. Additionally, SUDs can result in various types of mental illnesses, such as mood, anxiety, and sleep issues [10].

According to the World Drug Report 2021, several reasons have been identified as contributing to the aetiology of substance abuse: (1) epigenetic/genetic/personality traits, (2) family discordance/economic stress/domestic violence/poor interpersonal relationships, (3) psychopathologies such as social anxiety, depressive episodes, and post-traumatic stress disorder, (4) alleviation effects of substance withdrawal, (5) seeking enjoyment/getting high/increased energy/self-confidence, and (6) peer pressure [11–13].

The relationship between drug use and psychiatric disorders is complex and multifaceted [14]. Individuals with a history of substance use are at an increased risk of developing psychiatric symptoms or disorders, and vice versa [15]. Some individuals may use drugs as a way to cope with or self-medicate underlying psychiatric symptoms or distress [16]. For example, someone with anxiety may turn to alcohol or benzodiazepines to alleviate their symptoms temporarily. When individuals with psychiatric disorders and substance use problems seek treatment, the presence of illicit drug use can complicate treatment outcomes in several ways, including interfering with medications, which reduces their effectiveness, increasing the risk of relapse, leading to a cycle of instability and non-compliance, and reducing one’s motivation and commitment to treatment [17].

The COVID-19 pandemic has posed multiple challenges to deliveries in healthcare and public health, necessitating the adaptation of different strategies to minimize the pandemic’s impact [18]. Due to these circumstances, various restrictions were imposed by governments worldwide to address the threats to public health, which affected the patterns of drug abuse around the world [19]. In Malaysia, the first three cases of COVID-19 were recorded in January 2020, which triggered the mandated Movement Control Order (MCO) on March 18 [20]. Although the MCO showed improvement in decreasing and minimizing the spread of COVID-19 cases in Malaysia, it had a profound impact on mental health, including stress, anxiety, depression, and a sense of well-being, possibly due to external factors such as loneliness or loss of social support [21–23].

Individuals suffering from SUD are more likely to be at risk for SARS-CoV-2 infection due to various factors related to their medical background, psychological and psychosocial status, which are exacerbated by difficulty in accessing treatment and adherence [24–27]. As a result, these individuals are more likely to turn to alternative substances because of their increased negative emotions, such as boredom, fear, anxiety, and psychological distress, as well as their limited physical contact and minimal access to treatment and rehabilitation [19, 28–30]. This may result in an increased trend of substance misuse during the COVID-19 pandemic, which is related to their psychological and mental situations [19, 30].

Individuals with moderate to severe SUD are an important risk group and could suffer a major impact, as they may be associated with certain clinical and demographic characteristics, such as chronic respiratory diseases, other non-communicable diseases, and immunosuppression [31]. Therefore, it is important to measure the impact of COVID-19 on substance users and to suggest specific strategies.

Trend analyses are invaluable for supporting evidence and decision-making, and this is achievable through epidemiologic approaches. Examining trends provides essential information for actions such as risk needs assessment, program planning, program evaluation, and policy development activities [32]. Therefore, this study aimed to evaluate substance abuse trends among patients attending government hospitals in Malaysia before and during the COVID-19 pandemic.

## Materials and methods

### Study setting, design and period

A retrospective cross-sectional study was conducted from August until December 2021 at 16 selected government hospitals which are the tertiary hospitals in Malaysia namely; (1) Hospital Tuanku Fauziah, Perlis, (2) Hospital Sultanah Bahiyah, Kedah, (3) Hospital Pulau Pinang, (4) Hospital Raja Permaisuri Bainun, Perak, (5) Hospital Tengku Ampuan Rahimah, Klang, (6) Hospital Kajang, (7) Hospital Tuanku Ja’afar, Negeri Sembilan, (8) Hospital Melaka, (9) Hospital Sultanah Aminah, Johor, (10) Hospital Putrajaya, (11) Hospital Kuala Lumpur, (12) Hospital Tengku Ampuan Afzan, Pahang, (13) Hospital Sultanah Nur Zahirah, Terengganu, (14) Hospital Raja Perempuan Zainab II, Kelantan, (15) Hospital Umum Sarawak, (16) Hospital Queen Elizabeth, Sabah. All these hospitals are the major referral hospitals with addiction psychiatrist. These hospitals are located in the urban vicinity in the 14 states of Malaysia. For states with more than one main government hospitals, a simple random sampling method was used to select one hospital to participate in the study. Patients from suburban or rural are either referred from districts hospitals or primary health clinics to these hospitals when the need arise.

### Data source and study population

We retrieved patients’ medical records from hospital registry from the selected hospitals in Malaysia from January 2018 to December 2021. We screened all registered patients from psychiatric clinics in the selected hospitals and identified new patients without prior registration. We included patients aged 18 years old and above, and primarily diagnosed with SUD between 2018 to 2021 by a psychiatrist in charged. The report of urine drug test result was referred to confirm the drug use.

### Data collection

A standardized data collection form was used to collect the data by trained investigators. The form was divided into two sections. The first section contained information on sociodemographic profiles such as age, gender, ethnicity, education level, marital status, employment, place of residence, and history of psychiatric illness. The second section contained information on the substance abuse profiles, including the types of substance abuse (tobacco, alcohol, cannabis, amphetamine-type stimulants (ATS), inhalants, sedative or hypnotics, hallucinogens, opioids, kratom or others), reason on substance use initiation, and duration/length of abuse.

### Statistical analysis

Data was analysed using SPSS version 26.0. As data was normally distributed, parametric tests were used to determine the trend of substance abuse before and during the pandemic as well as association between substance abuse and sociodemographic profiles. Any categorical data were presented with frequencies and percentages, meanwhile mean and standard deviation (SD) were reported for continuous data. Pearson’s chi-square test was used to determine the difference in distribution of substance abuse before and during pandemic. Wald test of multivariate logistic regression was used to determine any potential predictors of substance abuse before and during pandemic using the ‘Enter’ method. Only new admission records were included; therefore, multiple admissions were excluded. Since the sample size is large, statistically significant effect was set at 0.05 as the threshold for significance.

### Ethics

The study was approved by the Medical Research & Ethics Committee (MREC), Ministry of Health Malaysia with the grant number NMMR-21-1275-60439 IIR. Informed consent form was not obtained since the data were extracted from patient’s medical record. Prior to analysis, data were deidentified because MREC permits the use of secondary data without subject identification.

## Results

A total of 9,606 new patients with SUD from the year 2018 to 2021 were identified, of which 82% were males and 18% were females **(Table 1)**. The mean age was 35.4 (SD 12.1) years old, ranging from 10 to 92 years old. Majority of the patients aged between 26 to 44 years old (56%), Malays (57%), high school students (43%), singles (46%), worked in private sectors (36%) or unemployed (35%), and resided in urban areas (64%). About 63% of the SUD patients had a history of psychiatric illness, of which 15% had depression, 17% had schizophrenia, 5% had anxiety, 5% had mania or bipolar, 21% had psychosis and 43% had other psychiatric illness. **Table 2** shows the substance abuse profiles of the patients. Over half of the patients are smokers (61.8%) and almost 40% consumed alcohol. The top three most abused substances were ATS (43.1%), followed by cannabis (17.2%) and opioids (13.0%). Most of the patients were poly-substance users (56.8%). The mean duration for substance use was 12.1 years. Peer pressure, relaxation and joy seeking were the most common reasons for patients to take substance for their first time.

**Table 1:** Sociodemographic profiles of SUD patients. (N = 9,606).

Variables	Before pandemic		During pandemic		Total
	2018	2019	2020	2021	
	N = 2,354	N = 2,433	N = 2,883	N = 1,936	N = 9,606
**Gender**					
Female	378 (16.1)	463 (19.0)	531 (18.4)	353 (18.2)	1725 (18.0)
Male	1976 (83.9)	1970 (81.0)	2352 (81.6)	1583 (81.8)	7881 (82.0)
**Age groups**					
<18	38 (1.6)	42 (1.7)	48 (1.7)	28 (1.4)	156 (1.6)
18–25	471 (20.0)	582 (23.9)	601 (20.8)	408 (21.1)	2062 (21.5)
26–44	1327 (56.4)	1327 (54.5)	1657 (57.5)	1111 (57.4)	5422 (56.4)
45–59	403 (17.1)	385 (15.8)	456 (15.8)	324 (16.7)	1568 (16.3)
60 and above	115 (4.9)	97 (4.0)	121 (4.2)	65 (3.4)	398 (4.1)
**Ethnic groups**					
Malay	1275 (54.2)	1407 (57.8)	1606 (55.7)	1164 (60.1)	5452 (56.8)
Chinese	434 (18.4)	453 (18.6)	496 (17.2)	254 (13.1)	1637 (17.0)
Indian	378 (16.1)	327 (13.4)	494 (17.1)	347 (17.9)	1546 (16.1)
Others	267 (11.3)	246 (10.1)	287 (10.0)	171 (8.8)	971 (10.1)
**Education level**					
No formal education	675 (28.7)	625 (25.7)	763 (26.5)	489 (25.3)	2552 (26.6)
Primary	287 (12.2)	270 (11.1)	307 (10.6)	188 (9.7)	1052 (11.0)
Secondary	1003 (42.6)	1011 (41.6)	1231 (42.7)	867 (44.8)	4112 (42.8)
Tertiary	389 (16.5)	527 (21.7)	582 (20.2)	392 (20.2)	1890 (19.7)
**Marital status**					
Single	1111 (47.2)	1123 (46.2)	1264 (43.8)	876 (45.2)	4374 (45.5)
Married	702 (29.8)	747 (30.7)	888 (30.8)	582 (30.1)	2919 (30.4)
Others	541 (23.0)	563 (23.1)	731 (25.4)	478 (24.7)	2313 (24.1)
**Occupation**					
Government	93 (4.0)	124 (5.1)	100 (3.5)	67 (3.5)	384 (4.0)
Private	831 (35.3)	830 (34.1)	1055 (36.6)	745 (38.5)	3461 (36.0)
Self-employed	271 (11.5)	274 (11.3)	313 (10.9)	225 (11.6)	1083 (11.3)
Others	369 (15.7)	340 (14.0)	378 (13.1)	210 (10.8)	1297 (13.5)
Unemployed	790 (33.6)	865 (35.6)	1037 (36.0)	689 (35.6)	3381 (35.2)
**Place of residence**					
Urban	1445 (61.4)	1511 (62.1)	1850 (64.2)	1360 (70.2)	6166 (64.2)
Rural	909 (38.6)	922 (37.9)	1033 (35.8)	576 (29.8)	3440 (35.8)
**History of psychiatric illness**	1444 (61.3)	1501 (61.7)	1860 (64.5)	1281 (66.2)	6086 (63.4)
Depression	218 (15.1)	278 (18.5)	274 (14.7)	175 (13.7)	945 (15.5)
Schizophrenia	334 (23.1)	58 (3.9)	352 (18.9)	272 (21.2)	1016 (16.7)
Anxiety	61 (4.2)	94 (6.3)	101 (5.4)	59 (4.6)	315 (5.2)
Mania/Bipolar	58 (4.0)	78 (5.2)	88 (4.7)	62 (4.8)	286 (4.7)
Psychosis	305 (21.1)	285 (19.0)	414 (22.3)	273 (21.3)	1277 (21.0)
Others	607 (42.0)	595 (39.6)	818 (44.0)	569 (44.0)	2584 (42.5)

Data is presented in numbers (percentage).

**Table 2:** Substance abuse profiles of SUD patients. (N = 9,606).

Variables	Before pandemic		During pandemic		Total
	2018	2019	2020	2021	
	N = 2,354	N = 2,433	N = 2,883	N = 1,936	N = 9,606
**Types of substance**					
Tobacco	1430 (60.7)	1536 (63.1)	1734 (60.1)	1240 (64.0)	5940 (61.8)
Alcohol	966 (41.0)	956 (39.3)	1156 (40.1)	739 (38.2)	3817 (39.7)
Cannabis	443 (18.8)	386 (15.9)	446 (15.5)	381 (19.7)	1656 (17.2)
ATS	1023 (43.5)	1001 (41.1)	1254 (43.5)	863 (44.6)	4141 (43.1)
Inhalant	100 (4.2)	84 (3.5)	80 (2.8)	43 (2.2)	307 (3.2)
Sedative/hypnotics	181 (7.7)	177 (7.3)	132 (4.6)	76 (3.9)	566 (5.9)
Hallucinogens	66 (2.8)	52 (2.1)	66 (2.3)	38 (2.0)	222 (2.3)
Opioids	337 (14.3)	316 (13.0)	368 (12.8)	223 (11.5)	1244 (13.0)
Kratom	185 (7.9)	217 (8.9)	273 (9.5)	166 (8.6)	841 (8.8)
Others	144 (6.1)	144 (5.9)	183 (6.3)	83 (4.3)	554 (5.8)
**Number of substances abused at a time**					
1	993 (42.2)	1069 (43.9)	1288 (44.7)	795 (41.1)	4145 (43.2)
≥2	1361 (57.8)	1364 (56.1)	1595 (55.3)	1141 (58.9)	5461 (56.8)
**Reason for initiating substance use**					
Health	57 (2.4)	58 (2.4)	49 (1.70)	30 (1.5)	194 (2.0)
Family issue	100 (4.2)	98 (4.0)	98 (3.4)	68 (3.5)	364 (3.8)
Job problem	73 (3.1)	80 (3.3)	71 (2.5)	45 (2.3)	269 (2.8)
Relationship	48 (2.0)	79 (3.2)	67 (2.3)	47 (2.4)	241 (2.5)
Relaxation	243 (10.3)	234 (9.6)	223 (7.7)	183 (9.5)	883 (9.2)
Joy seeking	173 (7.3)	159 (6.5)	189 (6.6)	124 (6.4)	645 (6.7)
Peer pressure	476 (20.2)	472 (19.4)	515 (17.9)	394 (20.4)	1857 (19.3)
Financial problem	25 (1.1)	19 (0.8)	19 (0.7)	12 (0.6)	75 (0.8)
Others	106 (4.5)	136 (5.6)	150 (5.2)	114 (5.9)	506 (5.3)

Data is presented in numbers (percentage).

### Sociodemographic characteristics of SUD patients before and during the COVID-19 pandemic

Overall, there was an increase of 0.4% in the frequency of SUD patients during the COVID-19 pandemic, of which 5.5% increased from 2018 to 2020, and a decrease of 9.8% in 2021. **Table 3** shows the trend of SUD patients before and during the pandemic. There were significant differences observed in terms of the distributions of SUD patients between ethnic groups, education level, occupation, place of residence, and history of psychiatric illness. Based on ethnic groups, Malays and Indians were observed to have an increase of 1.5% and 2.8%, respectively during the pandemic, while Chinese had a decrement of 2.9%. An increase trend of SUD also was also seen in patients with secondary and tertiary level of education with 1.4% and 1.1%, respectively. There were also slight increases in SUD among those who worked in the private sectors with 2.7%, and those who were unemployed with 1.2%. Besides that, those who lived in urban were also seen to have an increase trend of SUD by 4.8% during the pandemic. An increment of 3.7% in SUD patients with psychiatric illness history was also observed during the pandemic. Schizophrenia had the highest increment with about 5% compared to the other types of psychiatric illness.

**Table 3:** SUD patients before and during the COVID-19 pandemic. (N = 9,606).

Variables	Before pandemic	During pandemic	*p*-value^a^
	N = 4,787	N = 4,819	
**Gender**			0.322
Female	841 (17.6)	884 (18.3)	
Male	3946 (82.4)	3935 (81.7)	
**Age groups**			0.280
<18	80 (1.7)	76 (1.6)	
18–25	1053 (22.0)	1009 (20.9)	
26–44	2654 (55.4)	2768 (57.4)	
45–59	788 (16.5)	780 (16.2)	
60 and above	212 (4.4)	186 (3.9)	
**Ethnic groups**			**<0.001**
Malay	2682 (56.0)	2770 (57.5)	
Chinese	887 (18.5)	750 (15.6)	
Indian	705 (14.7)	841 (17.5)	
Others	513 (10.7)	458 (9.5)	
**Education level**			**0.047**
No formal education	1300 (27.2)	1252 (26.0)	
Primary	557 (11.6)	495 (10.3)	
Secondary	2014 (42.1)	2098 (43.5)	
Tertiary	916 (19.1)	974 (20.2)	
**Marital status**			0.109
Single	2234 (46.7)	2140 (44.4)	
Married	1449 (30.3)	1470 (30.5)	
Others	1104 (23.0)	1209 (25.1)	
**Occupation**			**<0.001**
Government	217 (4.5)	167 (3.5)	
Private	1661 (34.7)	1800 (37.4)	
Self-employed	545 (11.4)	538 (11.2)	
Others	709 (14.8)	588 (12.2)	
Unemployed	1655 (34.6)	1726 (35.8)	
**Place of residence**			**<0.001**
Urban	2956 (61.8)	3210 (66.6)	
Rural	1831 (38.2)	1609 (33.4)	
**History of psychiatric illness**	2945 (61.5)	3141 (65.2)	**<0.001**
Depression	496 (10.4)	450 (9.3)	0.092
Schizophrenia	392 (8.2)	624 (12.9)	**<0.001**
Anxiety	155 (3.2)	161 (3.3)	0.777
Mania/Bipolar	136 (2.8)	150 (3.1)	0.433
Psychosis	590 (12.3)	687 (14.3)	**0.005**
Others	1203 (25.1)	1390 (28.8)	**<0.001**

Data is presented in numbers (percentage).

^a^Pearson’s Chi-Square test, significant at *p* <0.05 (bold)

### Substance abuse among SUD patients before and during the COVID-19 pandemic

**Fig 1:**
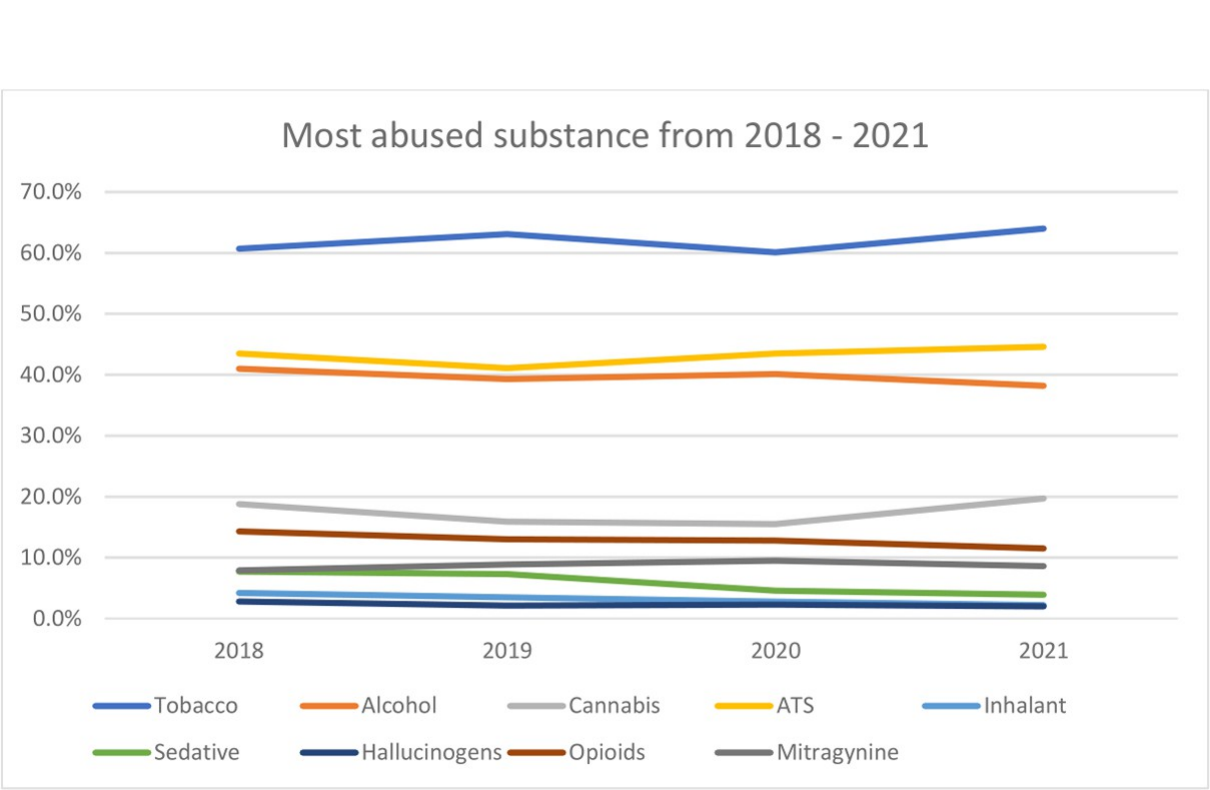
Types of substance abuse from 2018 to 2021.

Among the substances abused by patients, the use of inhalants, sedatives, and opioids were found to have significant differences between before and during the COVID-19 pandemic **(Table 4)**. A decrement of 1.2%, 3.2%, and 1.3% was observed in the use of the three substances during the pandemic, respectively. The trend of substance use over the four-years period is shown in **Fig 1**. Overall, tobacco, ATS, and kratom had increased trend, while others had decreased trend. Although, the findings for the use ATS and kratom were not statistically significant, but their increase trend during the pandemic were notable. ATS recorded an increase by 1.6% during the pandemic, while kratom use had an increment of 0.7%.

**Table 4:** Substance abuse among SUD patients before and during the COVID-19 pandemic. (N = 9,606).

Variables	Before pandemic	During pandemic	*p*-value^a^
	N = 4,787	N = 4,819	
**Types of substance**			
Tobacco	2966 (62.0)	2974 (61.7)	0.804
Alcohol	1922 (40.2)	1895 (39.3)	0.408
Cannabis	829 (17.3)	827 (17.2)	0.839
ATS	2024 (42.3)	2117 (43.9)	0.103
Inhalant	184 (3.8)	123 (2.6)	**<0.001**
Sedative or hypnotics	358 (7.5)	208 (4.3)	**<0.001**
Hallucinogens	118 (2.5)	104 (2.2)	0.317
Opioids	653 (13.6)	591 (12.3)	**0.044**
Kratom	402 (8.4)	439 (9.1)	0.217
Others	288 (6.0)	266 (5.5)	0.297
**Number of substances abused at a time**			
1	2062 (43.1)	2083 (43.2)	0.882
≥2	2725 (56.9)	2736 (56.8)	
**Reason for initiating substance use**			
Health	115 (2.4)	79 (1.6)	**0.008**
Family issue	198 (4.1)	166 (3.4)	0.076
Job problem	153 (3.2)	116 (2.4)	**0.019**
Relationship	127 (2.7)	114 (2.4)	0.368
Relaxation	477 (10.0)	406 (8.4)	**0.009**
Joy seeking	332 (6.9)	313 (6.5)	0.389
Peer pressure	948 (19.8)	909 (18.9)	0.243
Financial problem	44 (0.9)	31 (0.6)	0.125
Others	242 (5.1)	264 (5.5)	0.353

Data is presented in numbers (percentage).

^a^Pearson’s Chi-Square test, significant at *p* <0.05 (bold)

### Potential predictors of substance abuse during the COVID-19 pandemic

Further analysis to examine the potential predictors of substance abuse during the COVID-19 pandemic was performed using multivariate logistic regression analysis **(Table 5)**. Based on the findings, those who worked in the private sector (adjusted OR 1.423, 95% CI 1.147–1.767, *p* = 0.001), were self-employed (aOR 1.356, 95% CI 1.069–1.722, *p* = 0.012) or unemployed (aOR 1.367, 95% CI 1.101–1.698, *p* = 0.005) were more associated with substance abuse during the pandemic compared to those who worked in the government sector. Not only that, those who resides in urban area had higher chance to abuse substances than those who resides in rural area (aOR 1.192, 95% CI 1.091–1.302), *p*<0.001). Furthermore, those who had a history of psychiatric illness had higher risk to abuse substances during the pandemic than those without psychiatric history (aOR 1.184, 95% CI 1.086–1.291, *p*<0.001).

**Table 5:** Logistic regression on the likelihood of substance abuse during the pandemic (N = 9,606).

Variables	Before pandemic	During pandemic	Adjusted OR^a^ (95% CI)	*p*-value^b^
	N = 4,787 (%)	N = 4,819 (%)		
**Ethnic groups**				
Malay (ref)	2682 (56.0)	2770 (57.5)	1.000	
Chinese	887 (18.5)	750 (15.6)	0.780 (0.696–0.874)	**<0.001**
Indian	705 (14.7)	841 (17.5)	1.116 (0.992–1.256)	0.068
Others	513 (10.7)	458 (9.5)	0.848 (0.737–0.976)	**0.022**
**Education level**				
No formal education (ref)	1300 (27.2)	1252 (26.0)	1.000	
Primary	557 (11.6)	495 (10.3)	0.903 (0.778–1.048)	0.178
Secondary	2014 (42.1)	2098 (43.5)	1.042 (0.938–1.156)	0.443
Tertiary	916 (19.1)	974 (20.2)	1.080 (0.953–1.223)	0.228
**Occupation**				
Government (ref)	217 (4.5)	167 (3.5)	1.000	
Private	1661 (34.7)	1800 (37.4)	1.423 (1.147–1.767)	**0.001**
Self-employed	545 (11.4)	538 (11.2)	1.356 (1.069–1.722)	**0.012**
Others	709 (14.8)	588 (12.2)	1.078 (0.850–1.367)	0.534
Unemployed	1655 (34.6)	1726 (35.8)	1.367 (1.101–1.698)	**0.005**
**Place of residence**				
Rural (ref)	1831 (38.2)	1609 (33.4)	1.000	
Urban	2956 (61.8)	3210 (66.6)	1.192 (1.091–1.302)	**<0.001**
**History of psychiatric illness**				
No (ref)	1842 (38.5)	1678 (34.8)	1.000	
Yes	2945 (61.5)	3141 (65.2)	1.184 (1.086–1.291)	**<0.001**

^a^adjusted for gender, age groups, and marital status

^b^Wald test from multivariate logistic regression analysis using ‘Enter’ method, significant at *p* <0.05 (bold)

## Discussion

This study demonstrated the trend of SUD among new patients attending psychiatric facilities in the 16 main government hospitals in Malaysia from 2018 to 2021. The years 2018 to 2019 were considered as the period before the pandemic, while 2020 to 2021 was considered as the period during the pandemic. The most abused substances over the four-year period were tobacco, ATS, alcohol, cannabis, opioids, and kratom. Prominent increased trends during the COVID-19 pandemic were evident among Malays, Indians, those who with secondary or tertiary level of education, those working in the private sector, the unemployed, those residing in urban areas, and those with a history of psychiatric illness. Decreased trends were found for the use of inhalants, sedatives, and opioids. This study also found that those working in the private sector, self-employed individuals, the unemployed, those residing in urban areas, and those with a history of psychiatric illness were significant potential predictors of substance abuse during the COVID-19 pandemic.

From the current findings, it was notable that there was an increase in substance abuse among patients attending psychiatric facilities during the COVID-19 pandemic. Due to the unprecedented effects of the pandemic, many individuals experienced significant burdens, eventually triggering them to take drugs [33]. Furthermore, another major consequence of COVID-19 was the economic downturn, which resulted in salary and employment losses worldwide, leading to the emergence and rise of mental health disorders, including depression, stress, anxiety, and more [34]. It was also identified that there was a 10% decrease in substance abuse from 2020 to 2021. This finding could be due to the limited availability of substances, as restrictions for non-essential industries were imposed during the COVID-19 pandemic. As reported by the Malay Mail, alcoholic beverage factories were not allowed to operate during the pandemic as they were not considered essential [35].

Furthermore, the prevalence of drug use offenses during lockdown periods can be influenced by various factors, including the implementation of movement control orders (MCOs). Movement restrictions imposed during lockdowns can affect individuals’ ability to access substance abuse treatment services [36]. Limited mobility, the closure of treatment facilities, or reduced availability of in-person counselling sessions may create barriers for individuals seeking help for SUDs [37, 38]. Besides that, patients with SUDs may face heightened stigma or confidentiality concerns during lockdown periods, especially if they fear being identified or judged by others in their community [37, 39]. This fear may deter them from openly seeking treatment or disclosing their substance use issues.

Based on the overall profiles of SUD patients, apart from smoking, ATS was found to be the most abused substance, with its highest use reaching about 45% in 2021, followed by cannabis and opioids. A previous study conducted in 2013 reported that although opioids remained as the main abused drug, the trend of ATS was identified as a growing problem in Malaysia [40]. Subsequently, ATS was identified as the most used drug in 2017, surpassing opioid consumption [41]. According to statistics on drug addiction from 2016 to 2020, the use of ATS had increased from 2018 among Malaysians [42]. The consumption of ATS, which are synthetic psychostimulants, can activate the central nervous system to release dopamine, adrenaline, noradrenaline, and serotonin, leading users to experience excitement, heightened arousal, euphoria, and improved alertness [43]. With regard to that, ATS addiction has been associated with increased engagement in sexual risk behaviours. A study conducted in Malaysia found that drug use during sex work and servicing multiple clients per day were notably linked with current ATS usage [44]. Apart from that, ATS drugs are known to enhance concentration, focus, and alertness [45]. This may also explain why people consume ATS—to stay awake for extended periods, boost productivity, or perform better academically or at work.

Apart from that, the rising trend of kratom use was also notable. Kratom (*Mitragyna speciosa*), also known as ‘ketum’ or ‘biak-biak’, is a tropical plant indigenous to Southeast Asia, with mitragynine being one of the psychoactive compounds found abundantly in it [46]. Historically, in Malaysia, kratom has been used for its herbal properties to treat pain, diarrhoea, cough, and other common ailments [47]. Its consumption in the form of kratom drinks is common among heavy laborers, like rubber tappers, farmers, peasants, and machine operators, to combat fatigue and boost work performance. Kratom is also popular as a social drink, particularly among men. Unlike in the United States, where kratom is easily accessible through online purchases at reasonable prices [48, 49], in Malaysia, kratom is readily cultivated and available in local eateries and sold by illegal traders at low costs [46]. This accessibility explains why one may get addicted to kratom, despite its use, including buying and distributing are prohibited and regulated under the Poisons Act of 1952 [50].

In this study, the popularity of opioid use was found to have decreased, and this finding may be associated with the increased use of ATS and kratom. The increased supply of ATS has led to drop in prices across Southeast Asia, making them more affordable and popular. Consequently, more drug users are switching from opioids to ATS [44]. Besides that, former opioid users are more inclined to use kratom for its mood-elevating effects [47]. They also reported that kratom helps manage pain and alleviates opioid withdrawal symptoms [47, 51]. This explains the declined in opioid use and the rise in the use of ATS and kratom.

Trends in substance use by demographic characteristics were also analysed in this study. The results indicated that males had a higher number of new substance abuse cases compared to females. This is consistent with previous findings reported in the USA [50]. The higher prevalence among males could be attributed to high-risk behaviour, risky decision-making, and greater exposure to drugs and alcohol at a young age due to peer pressure [52]. Although the number of females using substances was lower than that of males, the increased trend of female SUD patients during the pandemic was notable. This finding aligns with a previous study that reported an increase in drug or alcohol use among women during the COVID-19 pandemic [53]. The study suggested that this increase was primarily due to higher anxiety levels in women, greater changes in daily life, and increased mental health-related distress compared to men. Women faced greater challenges, including increased unemployment, heightened childcare responsibilities, and intimate partner violence, which had gotten worse during the pandemic.

Substance use was most prevalent among individuals aged 26 to 44 years. This is consistent with previous findings that showed the highest prevalence of drug use among those aged 25 to 30 years old [54]. Another study reported an increased use of drugs among adults older than 30 years old during the COVID-19 pandemic [55]. Besides that, this study observed that individuals aged 36 to 45 years were more associated with substance abuse during the pandemic compared to those aged 19 to 25 years old. However, many studies have described that younger age groups reported higher levels of mental problems and loneliness during the pandemic, which led to greater involvement in substance use [56–58].

Furthermore, the trend of SUD patients with a history of psychiatric illness increased during the pandemic. As mentioned earlier, the COVID-19 pandemic had brought many challenges which affected numerous individuals. Lockdowns and social isolation measures are believed to have contributed to increased stress, misery, anxiety, and mental health challenges, which may have led some individuals to abuse substances as a means of coping with feelings of isolation and boredom, resulting in a higher incidence of drug-related offenses [33, 59]. Several studies also indicated that the pandemic placed significant strain on individuals, particularly those with existing mental health issues [60, 61]. According to these studies, some people turned to drugs as a way to escape their troubles and concerns, as the effects of substances could temporarily improve their mood. As of June 2020, 13% of Americans reported initiating or increasing substance use to cope with stress or emotions caused by the COVID-19 pandemic [62]. In addition to that, individuals with a history of psychiatric disorder were reported to be more vulnerable to substance abuse [63]. This evidence is consistent with our finding that those with a history of mental health problems are more likely to abuse substances than those without such conditions.

As mentioned earlier, the challenges, stresses, and burdens associated with the COVID-19 pandemic can significantly impact individuals’ mental well-being, potentially triggering substance abuse as a coping mechanism. Psychological help, such as therapy, counselling, and mental health support services, plays a crucial role in promoting mental health wellness [64, 65]. Although the implementation of lockdown during the pandemic created barriers for individuals seeking treatment, access to psychological therapies should still be maintained [66]. For example, monitoring psychosocial needs and providing psychosocial care can be effectively managed through telemedicine. Such support can yield positive outcomes from interventions such as emphatic listening, psychoeducation, and supportive therapy [65].

Understanding the preliminary findings on the trends of substance abuse in this study can provide valuable insights for treatment providers and inform the design of interventions targeting vulnerable groups of psychiatric patients with SUDs in Malaysia. For example, treatment providers can identify specific prevalent substances to tailor their treatment approaches and interventions. Besides that, monitoring drug use trends allows for early identification of patterns, guiding healthcare providers in implementing proactive screening measures to detect SUDs at an early stage. Furthermore, interventions offering support, education, and prevention strategies can be designed to specifically target high-risk groups, such as males, adults, and individuals with a history of mental health disorders. Data on substance use trends from this study can inform the development and implementation of evidence-based interventions. Last but not least, the study findings may foster collaborative efforts between healthcare providers, public health agencies, law enforcement, and community organizations, leading to comprehensive interventions that address treatment, prevention, harm reduction, and community support initiatives.

### Strength and limitations

This study is a multi-centre study which utilised a large sample population. It does not only evaluate substance abuse data at the height of pandemic in Malaysia, but also pre-pandemic. However, some limitations of our study should be noted. The first limitation is the retrospective cross-sectional design: we identified associations but could not infer cause and effect where the data depends on the efficiency of record reporting by the physicians. Secondly, we were unable to retrieve many variables which may influence the findings, such as previous medical history or length of the substance use prior to attending psychiatric facilities and therefore were not included in the analysis. Thirdly, the data on substance used included in this study was mainly based on self-report from patients, doctors and retrospective data collection from available notes in patients’ medical records. These factors may therefore also account for our findings.

## Conclusion

This study showed that there was a growing trend of substance abuse during the COVID-19 pandemic in Malaysia. Apart from taking drugs to cope with social isolation, and underlying mental health problems, we believe the contributing factors are due to gender, age, peer pressure, family history of substance abuse, social influence, past childhood abuse, interpersonal trauma, and lack of family support. Therefore, targeted exploration of factors affecting substance abuse in Malaysia is essential to decrease the trend. To the best of our knowledge, the current study is the first approach to investigate the trend of substance abuse before and during the COVID-19. The preliminary findings from this study may assist in identifying variations in substance abuse treatment or another alternative approach for those suffering from SUDs in Malaysia. By leveraging insights from the drug use trends in Malaysia, treatment providers could enhance the ability to deliver targeted, evidence-based interventions that address the unique needs of vulnerable groups of patients with SUDs. This approach contributes to improved outcomes, reduced relapse rates, and a more effective response to substance use challenges at both individual and population levels, and therefore will help in developing effective measures to reduce the trend of the substance abuse and decreased population-wide burdens in terms of morbidity, mortality, and economic costs. Besides that, dissemination of these trends can contribute to the current, though limited, substance abuse management which is specific to Malaysia. Furthermore, results from this study can guide policy makers by providing current, baseline, population-based evidence for future guidance.

## Supporting information

S1 Raw data (PDF)
