# Supplementary material for: Substance abuse among new patients attending main government hospitals in Malaysia from 2018–2021: A comparison between before and during COVID-19 pandemic
Source: PLoS One. 2024 Oct 24;19(10):e0309422. doi: 10.1371/journal.pone.0309422 (PMC11500913; doi:10.1371/journal.pone.0309422)
Supplement: S1 Raw data — (PDF) [file pone.0309422.s001.pdf]

No	Patient ID	Gender	Ethnic group	Year of diagnosis	Age	Marital status	Education level	Occupation	Place of residence	History of psy illness	Tobacco	Alcohol	Cannabis	ATS	Inhalant	Sedative	Hallucinogens	Opioids	Kratom
1	HKB0004	Male	Malay	2018	28	Single	Tertiary	Unemployed	Urban	Yes	No	No	No	Yes	No	No	No	No	No
2	HKB0008	Male	Malay	2020	40	Single	No formal education	Private	Urban	Yes	No	No	No	Yes	No	No	No	No	No
3	HKB0011	Male	Malay	2019	27	Single	Tertiary	Private	Urban	Yes	No	No	No	Yes	No	No	No	No	No
4	HKB0045	Male	Malay	2020	35	Single	No formal education	Unemployed	Urban	Yes	No	Yes	No	Yes	No	No	No	No	Yes
5	HKB0046	Male	Malay	2019	21	Single	Tertiary	Unemployed	Urban	Yes	Yes	No	Yes	No	No	Yes	No	No	Yes
6	HKB0048	Male	Malay	2018	30	Others	No formal education	Unemployed	Urban	Yes	Yes	Yes	No	Yes	No	Yes	No	No	Yes
7	HKB0049	Male	Malay	2021	48	Single	No formal education	Self-employed	Rural	Yes	No	No	No	Yes	No	No	No	Yes	Yes
8	HKB0050	Male	Malay	2019	41	Others	Tertiary	Self-employed	Urban	Yes	No	Yes	No	Yes	No	No	No	Yes	Yes
9	HKB0053	Male	Malay	2018	17	Single	Secondary	Unemployed	Urban	Yes	Yes	No	No	No	No	No	No	Yes	Yes
10	HKB0054	Male	Malay	2019	18	Single	Secondary	Others	Rural	Yes	Yes	No	No	No	No	No	No	No	Yes
11	HKB0055	Male	Malay	2019	18	Single	Primary	Unemployed	Rural	Yes	Yes	No	No	Yes	No	No	No	No	No
12	HKB0062	Male	Malay	2019	31	Single	Tertiary	Others	Rural	Yes	No	Yes	No	Yes	No	Yes	No	No	Yes
13	HKB0063	Male	Malay	2020	42	Others	No formal education	Unemployed	Rural	Yes	No	Yes	Yes	Yes	No	No	No	Yes	Yes
14	HKB0066	Male	Malay	2020	28	Married	No formal education	Private	Urban	Yes	No	No	No	Yes	No	No	No	No	No
15	HKB0068	Male	Malay	2019	21	Single	Secondary	Self-employed	Rural	Yes	No	No	No	Yes	No	No	No	Yes	Yes
16	HKB0070	Female	Malay	2020	39	Single	Tertiary	Unemployed	Urban	Yes	Yes	Yes	No	Yes	No	No	No	No	No
17	HKB0071	Male	Malay	2020	24	Single	No formal education	Unemployed	Urban	Yes	No	No	No	Yes	No	No	No	No	No
18	HKB0072	Male	Malay	2018	17	Single	Secondary	Unemployed	Urban	Yes	Yes	No	No	No	No	No	No	Yes	Yes
19	HKB0073	Male	Malay	2018	23	Single	Primary	Private	Urban	Yes	No	No	No	Yes	No	No	No	No	No
20	HKB0074	Male	Malay	2018	33	Others	Tertiary	Self-employed	Rural	Yes	No	No	No	Yes	No	No	No	No	Yes
21	HKB0075	Male	Malay	2019	26	Single	Tertiary	Private	Urban	Yes	No	No	No	Yes	No	No	No	No	No
22	HKB0076	Male	Malay	2018	36	Single	No formal education	Private	Rural	Yes	No	No	No	Yes	No	No	No	No	No
23	HKB0079	Male	Malay	2018	32	Others	Secondary	Self-employed	Rural	Yes	No	No	No	Yes	No	No	No	No	No
24	HKB0080	Female	Malay	2021	24	Single	Secondary	Unemployed	Rural	Yes	No	No	No	Yes	No	No	No	No	Yes
25	HKB0081	Male	Malay	2018	17	Single	Secondary	Unemployed	Urban	Yes	No	No	No	No	No	No	No	No	Yes
26	HKB0082	Male	Malay	2019	32	Others	Tertiary	Unemployed	Urban	Yes	Yes	No	Yes	Yes	No	No	No	Yes	No
27	HKB0083	Female	Malay	2020	28	Single	No formal education	Unemployed	Urban	Yes	Yes	No	No	Yes	No	No	No	No	No
28	HKB0084	Male	Malay	2020	30	Single	Tertiary	Private	Urban	Yes	No	No	Yes	Yes	No	No	No	No	No
29	HKB0085	Male	Malay	2018	38	Single	Secondary	Private	Rural	Yes	No	No	No	No	No	No	No	No	Yes
30	HKB0086	Male	Malay	2018	22	Single	Secondary	Private	Urban	Yes	No	No	No	Yes	No	No	No	No	No
31	HKB0088	Male	Malay	2021	30	Single	Secondary	Self-employed	Rural	Yes	No	No	Yes	Yes	No	No	No	No	Yes
32	HKB0091	Male	Malay	2018	31	Single	No formal education	Self-employed	Rural	Yes	No	No	Yes	Yes	Yes	No	No	No	No

No	Patient ID	Gender	Ethnic group	Year of diagnosis	Age	Marital status	Education level	Occupation	Place of residence	History of psy illness	Tobacco	Alcohol	Cannabis	ATS	Inhalant	Sedative	Hallucinogens	Opioids	Kratom
33	HKB0092	Male	Malay	2019	27	Single	No formal education	Self-employed	Rural	Yes	Yes	No	No	Yes	No	Yes	No	No	No
34	HKB0093	Male	Malay	2018	57	Single	No formal education	Unemployed	Rural	Yes	No	No	No	Yes	No	No	No	No	No
35	HKB0095	Male	Malay	2020	36	Single	No formal education	Unemployed	Urban	Yes	No	No	Yes	Yes	No	No	No	No	No
36	HKB0096	Female	Malay	2020	30	Married	No formal education	Unemployed	Rural	Yes	No	No	No	Yes	No	No	No	No	No
37	HKB0097	Male	Malay	2019	33	Single	Tertiary	Unemployed	Urban	Yes	No	No	No	Yes	No	No	No	No	No
38	HKB0098	Male	Malay	2018	39	Others	Secondary	Self-employed	Urban	Yes	Yes	No	No	Yes	No	Yes	No	No	No
39	HKB0099	Male	Malay	2021	41	Married	Primary	Others	Rural	Yes	Yes	No	Yes	Yes	No	No	No	Yes	No
40	HKB0100	Male	Malay	2021	38	Others	Secondary	Self-employed	Urban	Yes	No	No	No	Yes	No	Yes	No	Yes	No
41	HKB0101	Male	Malay	2020	28	Single	No formal education	Unemployed	Urban	Yes	No	No	Yes	Yes	No	No	No	No	Yes
42	HKB0102	Male	Malay	2018	18	Single	Secondary	Unemployed	Urban	Yes	No	No	Yes	No	No	No	No	No	Yes
43	HKB0103	Male	Malay	2020	33	Single	No formal education	Unemployed	Rural	Yes	No	No	No	Yes	No	No	No	No	No
44	HKB0104	Male	Malay	2021	26	Married	Primary	Private	Rural	Yes	No	No	No	No	No	Yes	No	Yes	Yes
45	HKB0105	Male	Malay	2019	34	Single	No formal education	Private	Rural	Yes	Yes	Yes	No	Yes	No	No	No	Yes	Yes
46	HKB0110	Male	Malay	2021	25	Married	Secondary	Self-employed	Urban	Yes	Yes	No	No	Yes	No	No	No	Yes	Yes
47	HKB0111	Male	Malay	2019	26	Single	Secondary	Private	Rural	Yes	No	Yes	Yes	Yes	Yes	No	No	Yes	No
48	HKB0112	Male	Malay	2020	31	Single	No formal education	Private	Rural	Yes	No	No	No	Yes	No	No	No	No	No
49	HKB0113	Male	Malay	2019	29	Single	Secondary	Private	Rural	Yes	Yes	No	No	Yes	No	No	No	No	Yes
50	HKB0114	Male	Malay	2018	27	Single	No formal education	Private	Rural	Yes	No	No	No	Yes	No	Yes	No	No	No
51	HKB0115	Male	Malay	2019	20	Single	Secondary	Unemployed	Rural	Yes	Yes	No	No	Yes	No	No	No	No	Yes
52	HKB0119	Male	Malay	2018	22	Single	Secondary	Unemployed	Urban	Yes	No	Yes	Yes	Yes	No	No	No	No	Yes
53	HKB0120	Female	Malay	2019	28	Others	Secondary	Unemployed	Urban	Yes	Yes	No	No	Yes	No	No	No	No	No
54	HKB0121	Male	Malay	2020	46	Single	Secondary	Unemployed	Rural	Yes	Yes	No	Yes	Yes	No	No	No	Yes	No
55	HKB0122	Male	Malay	2020	53	Married	No formal education	Unemployed	Rural	Yes	No	Yes	Yes	Yes	No	No	No	No	No
56	HKB0123	Male	Malay	2020	42	Married	Secondary	Unemployed	Rural	Yes	Yes	Yes	No	Yes	No	No	No	Yes	No
57	HKB0124	Male	Malay	2020	34	Married	Secondary	Unemployed	Rural	Yes	Yes	No	No	Yes	No	No	No	No	No
58	HKB0125	Male	Malay	2019	40	Married	No formal education	Unemployed	Rural	Yes	No	No	Yes	No	No	No	No	Yes	No
59	HKB0126	Male	Malay	2019	34	Single	No formal education	Unemployed	Rural	Yes	Yes	No	No	Yes	Yes	No	No	No	Yes
60	HKB0130	Male	Malay	2018	17	Single	Secondary	Unemployed	Urban	Yes	Yes	No	No	No	No	No	No	No	Yes

No	Patient ID	Gender	Ethnic group	Year of diagnosis	Age	Marital status	Education level	Occupation	Place of residence	History of psy illness	Tobacco	Alcohol	Cannabis	ATS	Inhalant	Sedative	Hallucinogens	Opioids	Kratom
61	HKB0133	Male	Malay	2020	25	Single	Secondary	Unemployed	Rural	Yes	No	No	No	Yes	No	No	No	No	No
62	HKB0136	Male	Malay	2020	28	Single	Secondary	Unemployed	Rural	Yes	Yes	No	No	Yes	No	No	No	Yes	Yes
63	HKB0137	Male	Malay	2018	44	Single	Secondary	Self-employed	Rural	Yes	Yes	Yes	No	Yes	No	No	No	Yes	Yes
64	HKB0138	Male	Malay	2021	22	Single	Secondary	Self-employed	Rural	Yes	Yes	No	Yes	Yes	No	No	No	No	Yes
65	HKB0141	Male	Malay	2020	23	Single	No formal education	Unemployed	Rural	Yes	No	No	No	Yes	No	No	No	No	No
66	HKB0142	Male	Malay	2018	36	Single	No formal education	Private	Rural	Yes	No	No	No	Yes	No	No	No	No	No
67	HKB0144	Male	Malay	2018	40	Single	No formal education	Self-employed	Rural	Yes	Yes	No	Yes	Yes	No	No	No	Yes	No
68	HKB0145	Male	Malay	2021	29	Single	Secondary	Self-employed	Rural	Yes	Yes	No	No	Yes	No	No	No	No	No
69	HKB0146	Male	Malay	2019	24	Single	No formal education	Private	Rural	Yes	No	No	No	Yes	No	No	No	No	No
70	HKB0148	Male	Malay	2020	36	Others	Tertiary	Self-employed	Rural	Yes	No	No	No	Yes	No	No	No	No	No
71	HKB0149	Male	Malay	2021	44	Married	Tertiary	Self-employed	Rural	Yes	No	No	No	Yes	No	No	No	No	No
72	HKB0151	Male	Malay	2021	29	Single	Secondary	Unemployed	Rural	Yes	Yes	No	No	Yes	Yes	No	Yes	No	No
73	HKB0152	Male	Malay	2021	23	Single	Tertiary	Others	Rural	Yes	Yes	No	No	No	No	Yes	No	No	Yes
74	HKB0153	Male	Malay	2020	40	Married	No formal education	Private	Urban	Yes	Yes	No	No	Yes	No	No	No	Yes	Yes
75	HKB0154	Male	Malay	2018	24	Single	Secondary	Private	Rural	Yes	Yes	No	No	Yes	No	Yes	No	No	Yes
76	HKB0157	Male	Malay	2020	25	Married	Secondary	Private	Rural	Yes	Yes	No	No	No	No	Yes	No	No	Yes
77	HKB0158	Female	Malay	2021	46	Others	No formal education	Self-employed	Rural	Yes	Yes	No	No	Yes	No	No	No	No	Yes
78	HKB0159	Male	Malay	2018	23	Single	Tertiary	Unemployed	Rural	Yes	Yes	No	No	Yes	No	No	No	No	No
79	HKB0160	Female	Malay	2020	38	Married	No formal education	Unemployed	Urban	Yes	Yes	No	No	Yes	No	No	No	No	No
80	HKB0161	Male	Malay	2021	24	Single	Tertiary	Private	Rural	Yes	Yes	No	Yes	No	No	No	No	No	No
81	HKB0164	Female	Malay	2020	24	Single	Secondary	Self-employed	Rural	Yes	No	No	No	Yes	No	No	No	No	No
82	HKB0166	Female	Malay	2020	29	Single	Secondary	Unemployed	Rural	Yes	No	No	No	Yes	No	No	No	No	No
83	HKB0168	Male	Malay	2018	20	Single	Secondary	Private	Urban	Yes	No	No	No	No	No	No	No	No	Yes
84	HKB0169	Male	Others	2021	30	Single	Secondary	Self-employed	Rural	Yes	No	No	No	Yes	No	No	No	No	No
85	HKB0171	Male	Malay	2021	29	Single	Secondary	Private	Urban	Yes	Yes	Yes	Yes	Yes	Yes	No	No	Yes	Yes
86	HKB0173	Male	Malay	2021	27	Married	Secondary	Self-employed	Urban	No	No	No	No	No	No	No	No	No	Yes
87	HKB0174	Male	Malay	2020	35	Single	Secondary	Self-employed	Rural	Yes	No	No	No	Yes	No	No	No	No	No
88	HKB0175	Male	Malay	2021	29	Single	Tertiary	Private	Urban	Yes	Yes	No	Yes	Yes	No	Yes	No	Yes	Yes
89	HKB0176	Male	Malay	2021	40	Married	Tertiary	Government	Urban	Yes	No	No	No	Yes	No	No	No	No	No
90	HKB0177	Male	Malay	2019	44	Others	No formal education	Others	Urban	Yes	No	Yes	No	Yes	No	No	No	No	No
91	HKB0179	Male	Malay	2019	40	Single	Secondary	Unemployed	Urban	Yes	Yes	No	Yes	Yes	No	No	No	No	Yes
92	HKB0180	Male	Malay	2021	39	Married	Secondary	Unemployed	Rural	Yes	Yes	No	No	Yes	No	No	No	No	No
93	HKB0181	Male	Malay	2020	29	Single	Secondary	Unemployed	Urban	Yes	Yes	No	Yes	Yes	No	No	No	Yes	No
94	HKB0185	Male	Malay	2020	36	Single	No formal education	Private	Urban	Yes	No	No	Yes	Yes	No	No	No	No	No

No	Patient ID	Gender	Ethnic group	Year of diagnosis	Age	Marital status	Education level	Occupation	Place of residence	History of psy illness	Tobacco	Alcohol	Cannabis	ATS	Inhalant	Sedative	Hallucinogens	Opioids	Kratom
95	HKB0186	Male	Malay	2020	31	Single	Secondary	Unemployed	Urban	Yes	No	No	No	Yes	No	No	No	No	Yes
96	HKB0187	Male	Malay	2019	24	Single	No formal education	Unemployed	Urban	Yes	Yes	No	No	Yes	Yes	No	No	No	No
97	HKB0188	Male	Malay	2018	28	Others	Secondary	Self-employed	Rural	Yes	No	No	No	Yes	No	No	No	No	Yes
98	HKB0189	Male	Malay	2020	30	Single	No formal education	Private	Rural	Yes	No	No	Yes	Yes	No	No	No	Yes	Yes
99	HKB0190	Male	Malay	2018	36	Single	No formal education	Private	Rural	Yes	No	No	Yes	Yes	No	No	No	Yes	Yes
100	HKB0192	Male	Malay	2018	22	Married	Secondary	Self-employed	Rural	Yes	Yes	No	No	No	No	No	No	Yes	Yes
101	HKB0193	Male	Malay	2019	28	Single	Secondary	Unemployed	Rural	Yes	No	No	No	Yes	No	No	No	No	Yes
102	HKB0194	Male	Malay	2020	20	Single	Tertiary	Unemployed	Rural	Yes	No	No	Yes	No	No	No	Yes	No	No
103	HKB0195	Male	Malay	2019	24	Single	Tertiary	Unemployed	Urban	Yes	Yes	Yes	Yes	No	No	No	No	Yes	Yes
104	HKB0196	Male	Malay	2021	20	Single	Secondary	Private	Urban	Yes	Yes	No	No	Yes	No	Yes	No	No	Yes
105	HKB0197	Male	Malay	2020	17	Single	Secondary	Private	Rural	Yes	Yes	No	No	No	No	No	No	No	Yes
106	HKB0198	Male	Malay	2019	33	Single	Secondary	Unemployed	Urban	Yes	Yes	Yes	Yes	Yes	No	No	No	No	No
107	HKB0199	Male	Malay	2020	33	Single	No formal education	Self-employed	Urban	Yes	No	No	Yes	Yes	No	No	No	No	Yes
108	HKB0200	Male	Malay	2020	26	Single	Secondary	Unemployed	Rural	Yes	No	No	No	Yes	No	No	No	No	Yes
109	HKB0202	Male	Malay	2018	30	Others	Secondary	Unemployed	Rural	Yes	No	No	No	Yes	No	No	No	Yes	No
110	HKB0207	Male	Malay	2018	33	Single	Tertiary	Private	Urban	Yes	Yes	No	No	No	No	No	No	No	No
111	HKB0212	Male	Malay	2021	22	Single	No formal education	Unemployed	Urban	Yes	Yes	No	Yes	Yes	No	No	No	Yes	Yes
112	HKB0222	Male	Malay	2019	34	Single	No formal education	Unemployed	Rural	Yes	No	No	No	Yes	No	Yes	No	No	No
113	HKB0225	Male	Malay	2018	34	Single	No formal education	Unemployed	Urban	Yes	Yes	No	No	No	Yes	No	No	No	No
114	HKB0226	Male	Malay	2020	44	Single	Secondary	Self-employed	Rural	Yes	No	No	No	Yes	No	No	No	No	Yes
115	HKB0228	Male	Malay	2018	53	Others	No formal education	Others	Urban	Yes	No	No	No	No	No	No	No	Yes	No
116	HKB0240	Male	Malay	2019	44	Single	Primary	Unemployed	Rural	Yes	Yes	No	No	Yes	No	No	No	No	No
117	HKB0241	Male	Malay	2018	34	Single	Secondary	Unemployed	Rural	Yes	Yes	No	No	Yes	No	No	No	No	No
118	HKB0244	Male	Malay	2019	33	Single	Secondary	Unemployed	Rural	Yes	No	No	No	Yes	No	No	No	No	Yes
119	HKB0246	Male	Malay	2019	46	Single	No formal education	Unemployed	Urban	Yes	No	No	No	Yes	No	No	No	Yes	No
120	HKB0247	Male	Malay	2018	20	Single	Secondary	Self-employed	Urban	Yes	No	No	No	Yes	No	No	No	Yes	No
121	HKB0250	Male	Malay	2019	42	Married	No formal education	Unemployed	Rural	Yes	No	No	No	Yes	No	No	No	No	No
122	HKB0251	Male	Malay	2019	36	Single	No formal education	Others	Urban	Yes	No	No	No	Yes	No	No	No	Yes	No
123	HKB0257	Male	Malay	2020	33	Single	No formal education	Unemployed	Rural	Yes	No	No	No	Yes	No	No	No	No	No
124	HKB0260	Male	Malay	2020	27	Single	No formal education	Private	Rural	Yes	No	No	No	No	No	Yes	No	No	No

No	Patient ID	Gender	Ethnic group	Year of diagnosis	Age	Marital status	Education level	Occupation	Place of residence	History of psy illness	Tobacco	Alcohol	Cannabis	ATS	Inhalant	Sedative	Hallucinogens	Opioids	Kratom
125	HKB0264	Male	Malay	2019	30	Others	No formal education	Others	Rural	Yes	No	No	No	Yes	No	No	No	No	No
126	HKB0266	Male	Malay	2019	41	Single	No formal education	Unemployed	Rural	Yes	Yes	No	No	Yes	No	No	No	No	No
127	HKB0272	Male	Malay	2021	38	Single	No formal education	Unemployed	Rural	Yes	No	No	No	Yes	No	No	No	No	Yes
128	HKB0273	Male	Malay	2018	57	Others	No formal education	Unemployed	Rural	Yes	No	No	Yes	No	No	No	No	No	No
129	HKB0278	Female	Malay	2018	33	Single	No formal education	Others	Rural	Yes	No	No	No	No	No	No	No	Yes	No
130	HKB0280	Female	Malay	2020	32	Single	Primary	Unemployed	Rural	Yes	Yes	No	No	Yes	No	No	No	No	No
131	HKB0282	Male	Malay	2020	33	Single	Secondary	Self-employed	Urban	Yes	Yes	No	No	Yes	No	No	No	No	No
132	HKB0286	Male	Malay	2018	34	Married	No formal education	Others	Rural	Yes	No	No	No	Yes	No	No	No	No	Yes
133	HKB0295	Male	Malay	2019	42	Others	No formal education	Others	Rural	Yes	No	No	No	Yes	Yes	No	No	Yes	No
134	HKB0300	Male	Malay	2019	25	Single	Secondary	Unemployed	Rural	Yes	No	No	No	Yes	No	No	No	Yes	No
135	HKB0302	Male	Malay	2019	27	Single	No formal education	Others	Rural	Yes	No	No	No	Yes	No	No	No	No	No
136	HKB0303	Male	Malay	2018	24	Single	Secondary	Unemployed	Rural	Yes	No	No	No	Yes	No	No	No	No	No
137	HKB0305	Male	Malay	2019	34	Others	No formal education	Others	Rural	Yes	No	No	No	Yes	No	No	No	No	No
138	HKB0311	Male	Malay	2019	40	Others	No formal education	Private	Urban	Yes	No	No	No	Yes	No	Yes	No	No	No
139	HKB0313	Male	Malay	2020	30	Others	Secondary	Others	Rural	Yes	No	No	No	Yes	No	No	No	No	No
140	HKB0315	Male	Chinese	2020	34	Single	Tertiary	Government	Rural	Yes	No	No	No	Yes	No	No	No	No	No
141	HKB0316	Male	Malay	2021	45	Others	No formal education	Unemployed	Urban	Yes	No	No	No	Yes	No	No	No	No	No
142	HKB0327	Male	Malay	2019	44	Others	No formal education	Others	Urban	Yes	No	No	No	Yes	No	No	No	No	Yes
143	HKB0328	Male	Malay	2020	28	Others	No formal education	Others	Urban	Yes	No	No	No	Yes	No	No	No	No	No
144	HKB0338	Male	Indian	2020	33	Single	Secondary	Self-employed	Urban	Yes	No	Yes	No	No	No	No	No	No	No
145	HKB0339	Male	Malay	2018	33	Others	No formal education	Others	Rural	Yes	No	No	No	Yes	No	No	No	No	No
146	HKB0347	Male	Malay	2020	28	Others	Secondary	Unemployed	Urban	Yes	Yes	No	No	Yes	No	No	No	Yes	Yes
147	HKB0348	Male	Malay	2020	25	Others	No formal education	Others	Urban	Yes	No	No	No	No	No	No	No	No	Yes
148	HKB0359	Male	Malay	2019	30	Single	Tertiary	Private	Rural	Yes	Yes	No	No	Yes	No	Yes	No	No	No
149	HKB0362	Male	Malay	2018	43	Others	No formal education	Others	Rural	Yes	No	No	No	Yes	No	No	No	No	No
150	HKB0365	Male	Malay	2019	37	Single	Secondary	Unemployed	Rural	Yes	No	Yes	No	Yes	No	No	No	No	Yes
151	HKB0370	Male	Malay	2019	19	Others	Tertiary	Unemployed	Rural	Yes	No	No	No	Yes	No	No	No	No	No
152	HKB0383	Male	Malay	2018	35	Others	Secondary	Unemployed	Rural	Yes	No	No	No	Yes	No	No	No	No	No

No	Patient ID	Gender	Ethnic group	Year of diagnosis	Age	Marital status	Education level	Occupation	Place of residence	History of psy illness	Tobacco	Alcohol	Cannabis	ATS	Inhalant	Sedative	Hallucinogens	Opioids	Kratom
153	HKB0388	Male	Malay	2021	21	Single	Secondary	Unemployed	Rural	Yes	No	No	No	Yes	No	No	No	No	No
154	HKB0389	Male	Malay	2021	35	Single	Tertiary	Unemployed	Urban	Yes	No	No	Yes	No	No	No	No	No	No
155	HKB0394	Male	Malay	2020	33	Others	No formal education	Self-employed	Rural	Yes	No	No	Yes	Yes	No	No	No	No	Yes
156	HKB0395	Male	Malay	2018	39	Single	No formal education	Unemployed	Rural	Yes	No	No	No	Yes	No	No	No	No	No
157	HKB0396	Male	Malay	2019	36	Married	Tertiary	Unemployed	Rural	Yes	Yes	No	No	No	No	No	No	No	No
158	HKB0402	Male	Malay	2018	29	Single	No formal education	Others	Rural	Yes	No	No	No	No	No	Yes	No	No	No
159	HKB0403	Male	Malay	2018	26	Single	Secondary	Others	Rural	Yes	No	No	No	Yes	No	No	No	No	No
160	HKB0408	Male	Malay	2020	19	Single	Secondary	Self-employed	Rural	Yes	No	No	No	No	No	No	No	Yes	Yes
161	HKB0410	Male	Malay	2021	40	Married	Secondary	Self-employed	Rural	Yes	No	No	No	Yes	No	No	No	No	Yes
162	HKB0412	Male	Others	2021	42	Single	No formal education	Unemployed	Rural	Yes	No	Yes	No	No	No	No	No	No	No
163	HKB0414	Male	Malay	2018	31	Single	Secondary	Unemployed	Rural	Yes	Yes	No	No	No	No	No	No	No	No
164	HKB0415	Male	Malay	2018	27	Married	Tertiary	Others	Rural	Yes	Yes	No	No	No	No	No	No	No	No
165	HKB0416	Male	Chinese	2021	44	Married	No formal education	Others	Rural	Yes	No	No	No	Yes	No	Yes	No	No	No
166	HKB0419	Male	Malay	2021	32	Single	No formal education	Others	Rural	Yes	No	No	No	Yes	No	No	No	No	No
167	HKB0420	Male	Malay	2018	50	Married	No formal education	Self-employed	Urban	Yes	No	No	No	Yes	No	No	No	No	No
168	HKB0422	Male	Malay	2020	37	Married	No formal education	Others	Rural	Yes	No	No	No	Yes	No	No	No	No	No
169	HKB0427	Male	Malay	2019	40	Single	No formal education	Others	Rural	Yes	No	No	No	Yes	No	No	No	No	No
170	HKB0433	Male	Malay	2019	23	Single	Secondary	Private	Rural	Yes	Yes	No	No	No	No	No	No	No	No
171	HKB0436	Male	Malay	2019	33	Others	No formal education	Government	Rural	Yes	No	No	No	Yes	No	No	No	No	No
172	HKB0437	Male	Malay	2021	28	Single	No formal education	Others	Rural	Yes	No	No	No	Yes	No	No	No	No	No
173	HKB0438	Male	Malay	2020	34	Others	No formal education	Private	Rural	Yes	No	No	No	Yes	No	No	No	No	No
174	HKB0441	Male	Malay	2018	25	Single	Secondary	Unemployed	Rural	Yes	Yes	No	No	Yes	No	No	No	No	No
175	HKB0454	Male	Malay	2018	57	Others	No formal education	Others	Rural	Yes	No	No	No	Yes	No	No	No	Yes	No
176	HKB0455	Male	Malay	2020	33	Others	No formal education	Private	Rural	Yes	No	No	No	Yes	No	No	No	No	No
177	HKB0457	Male	Malay	2018	35	Others	No formal education	Others	Rural	Yes	No	No	No	Yes	No	No	No	No	No
178	HKB0460	Male	Others	2019	29	Others	No formal education	Others	Rural	Yes	No	No	No	Yes	No	No	No	No	No
179	HKB0462	Male	Malay	2018	40	Married	No formal education	Others	Rural	Yes	No	No	No	No	No	No	No	No	Yes

No	Patient ID	Gender	Ethnic group	Year of diagnosis	Age	Marital status	Education level	Occupation	Place of residence	History of psy illness	Tobacco	Alcohol	Cannabis	ATS	Inhalant	Sedative	Hallucinogens	Opioids	Kratom
180	HKB0465	Male	Malay	2021	46	Single	No formal education	Unemployed	Rural	Yes	Yes	No	Yes	No	No	No	No	No	Yes
181	HKB0468	Male	Malay	2020	44	Others	No formal education	Self-employed	Rural	Yes	No	No	No	Yes	No	No	No	No	No
182	HKB0476	Male	Malay	2019	31	Single	Tertiary	Private	Urban	Yes	No	No	No	Yes	No	No	No	No	Yes
183	HKB0477	Male	Indian	2019	32	Single	Tertiary	Government	Rural	Yes	No	Yes	No	No	No	No	No	No	No
184	HKB0481	Male	Malay	2018	20	Single	Secondary	Others	Rural	Yes	No	No	No	Yes	No	No	No	No	No
185	HKB0485	Male	Malay	2020	40	Single	Tertiary	Self-employed	Rural	Yes	No	No	No	Yes	No	No	No	Yes	No
186	HKB0486	Male	Chinese	2019	39	Single	Tertiary	Self-employed	Urban	Yes	No	Yes	No	No	No	No	No	No	No
187	HKB0489	Male	Malay	2019	16	Single	Secondary	Unemployed	Rural	Yes	Yes	No	No	No	No	No	No	No	No
188	HKB0489	Male	Malay	2019	15	Single	Secondary	Unemployed	Urban	Yes	Yes	No	No	Yes	No	No	No	No	Yes
189	HKB0491	Male	Malay	2018	28	Single	No formal education	Self-employed	Rural	Yes	Yes	Yes	No	No	No	No	No	No	No
190	HKB0493	Male	Malay	2019	29	Single	No formal education	Others	Rural	Yes	Yes	No	No	Yes	No	No	No	No	No
191	HKB0494	Male	Malay	2021	52	Others	Secondary	Private	Rural	Yes	Yes	No	No	No	No	No	No	No	No
192	HKB0496	Male	Malay	2018	36	Single	Secondary	Self-employed	Rural	Yes	Yes	No	No	No	No	No	No	No	No
193	HKB0497	Male	Malay	2018	32	Single	Tertiary	Private	Rural	Yes	Yes	No	No	No	No	No	No	No	No
194	HKB0498	Male	Chinese	2019	40	Others	No formal education	Others	Urban	Yes	No	Yes	No	No	No	No	No	No	No
195	HKB0503	Male	Malay	2020	16	Single	No formal education	Others	Rural	Yes	Yes	Yes	No	Yes	No	No	No	No	Yes
196	HKB0505	Male	Malay	2020	32	Single	Tertiary	Unemployed	Urban	Yes	No	No	No	Yes	No	No	No	No	Yes
197	HKB0509	Male	Malay	2018	35	Single	Secondary	Others	Rural	Yes	Yes	No	No	No	No	No	No	No	No
198	HKB0510	Male	Malay	2018	44	Single	Secondary	Self-employed	Rural	Yes	Yes	No	No	No	No	No	No	No	No
199	HKB0512	Male	Malay	2019	20	Single	Secondary	Others	Rural	Yes	No	No	No	No	No	No	No	No	Yes
200	HKB0516	Male	Malay	2018	27	Single	Secondary	Private	Urban	Yes	No	No	No	Yes	No	No	No	No	No
201	HKB0519	Male	Malay	2018	41	Married	Secondary	Private	Rural	Yes	No	No	No	Yes	No	No	No	No	No
202	HKB0521	Male	Malay	2018	41	Married	Secondary	Unemployed	Rural	Yes	No	No	No	Yes	No	No	No	No	No
203	HKB0526	Male	Malay	2019	33	Single	No formal education	Others	Urban	Yes	No	No	No	Yes	No	No	No	No	No
204	HKB0528	Male	Malay	2018	56	Married	No formal education	Private	Rural	Yes	Yes	No	No	No	No	No	No	No	No
205	HKB0529	Male	Malay	2018	35	Single	No formal education	Unemployed	Rural	Yes	No	No	No	Yes	No	No	No	No	No
206	HKB0530	Male	Malay	2018	33	Others	Secondary	Private	Urban	Yes	No	No	No	No	Yes	No	No	No	No
207	HKB0532	Male	Indian	2018	26	Single	No formal education	Private	Urban	Yes	No	No	Yes	No	No	No	No	No	No
208	HKB0536	Male	Malay	2019	30	Married	Primary	Private	Urban	Yes	No	No	Yes	Yes	Yes	No	No	No	No
209	HKB0537	Male	Malay	2020	50	Others	Secondary	Private	Rural	Yes	Yes	No	No	Yes	No	No	No	No	Yes
210	HKB0542	Male	Malay	2021	24	Single	Secondary	Unemployed	Rural	Yes	No	No	No	Yes	No	No	No	No	No
211	HKB0543	Male	Malay	2021	29	Others	No formal education	Others	Rural	Yes	No	No	No	No	No	No	No	No	Yes

No	Patient ID	Gender	Ethnic group	Year of diagnosis	Age	Marital status	Education level	Occupation	Place of residence	History of psy illness	Tobacco	Alcohol	Cannabis	ATS	Inhalant	Sedative	Hallucinogens	Opioids	Kratom
212	HKB0549	Male	Malay	2018	54	Married	No formal education	Others	Urban	Yes	No	No	Yes	Yes	No	No	No	Yes	No
213	HKB0552	Male	Malay	2020	22	Single	Secondary	Unemployed	Rural	Yes	Yes	No	No	No	No	No	No	No	No
214	HKB0554	Male	Malay	2019	27	Single	Secondary	Self-employed	Rural	Yes	Yes	No	No	No	No	No	No	No	No
215	HKB0555	Female	Malay	2020	31	Others	No formal education	Unemployed	Rural	Yes	No	No	No	Yes	No	No	No	No	No
216	HKB0556	Male	Malay	2018	29	Single	No formal education	Self-employed	Rural	Yes	Yes	No	Yes	No	No	Yes	No	No	No
217	HKB0558	Male	Malay	2020	43	Others	No formal education	Private	Rural	Yes	No	Yes	No	Yes	No	No	No	No	Yes
218	HKB0559	Male	Malay	2020	36	Married	No formal education	Others	Rural	Yes	No	No	No	Yes	No	No	No	No	Yes
219	HKB0560	Male	Malay	2018	21	Single	Secondary	Private	Rural	Yes	No	No	No	Yes	No	No	No	No	Yes
220	HKB0561	Male	Malay	2018	21	Single	Tertiary	Unemployed	Rural	Yes	Yes	No	No	No	No	No	No	No	No
221	HKB0564	Male	Malay	2018	41	Married	Tertiary	Unemployed	Rural	Yes	No	No	No	Yes	No	No	No	No	No
222	HKB0566	Male	Malay	2021	26	Married	No formal education	Private	Rural	Yes	No	No	No	No	No	No	No	No	Yes
223	HKB0567	Male	Malay	2018	23	Single	Secondary	Unemployed	Urban	Yes	No	No	Yes	No	No	No	No	No	Yes
224	HKB0568	Male	Malay	2019	21	Single	Secondary	Private	Urban	Yes	No	No	No	Yes	No	No	No	No	No
225	HKB0569	Male	Malay	2021	30	Single	Tertiary	Unemployed	Rural	Yes	Yes	No	No	Yes	No	No	No	No	Yes
226	HKB0573	Male	Malay	2021	24	Single	No formal education	Private	Rural	Yes	Yes	No	Yes	Yes	No	No	No	No	No
227	HKB0574	Male	Malay	2019	43	Others	No formal education	Others	Urban	Yes	No	No	No	No	No	No	No	No	No
228	HKB0582	Male	Malay	2018	39	Single	Primary	Unemployed	Urban	Yes	No	No	No	Yes	Yes	No	Yes	No	No
229	HKB0591	Male	Malay	2020	25	Single	No formal education	Unemployed	Rural	Yes	No	No	No	Yes	No	No	No	No	No
230	HKB0592	Male	Malay	2020	40	Others	No formal education	Others	Rural	Yes	No	No	No	Yes	No	No	No	No	No
231	HKB0593	Male	Malay	2018	44	Married	Tertiary	Unemployed	Rural	Yes	Yes	No	Yes	Yes	No	No	No	No	No
232	HKB0598	Male	Malay	2019	32	Single	No formal education	Others	Rural	Yes	No	No	No	Yes	No	No	No	No	No
233	HKB0599	Male	Malay	2019	23	Single	Secondary	Self-employed	Rural	Yes	No	No	No	Yes	No	No	No	No	Yes
234	HKB0599	Male	Malay	2019	22	Single	Secondary	Self-employed	Rural	Yes	No	No	Yes	Yes	No	No	No	No	Yes
235	HKB0600	Male	Malay	2019	17	Single	Secondary	Unemployed	Rural	Yes	Yes	No	No	No	No	No	No	No	No
236	HKB0601	Male	Malay	2021	38	Married	No formal education	Unemployed	Rural	Yes	No	No	No	Yes	No	No	No	Yes	Yes
237	HKB0605	Male	Malay	2021	28	Single	Secondary	Self-employed	Rural	Yes	Yes	No	No	No	No	No	No	No	No
238	HKB0612	Male	Malay	2020	21	Single	No formal education	Unemployed	Rural	Yes	No	No	No	Yes	No	No	No	No	No
239	HKB0615	Male	Malay	2020	35	Others	Tertiary	Private	Urban	Yes	No	No	No	Yes	Yes	No	No	No	No
240	HKB0617	Male	Chinese	2018	17	Single	Secondary	Unemployed	Urban	Yes	Yes	Yes	No	No	No	No	No	Yes	Yes
241	HKB0619	Male	Malay	2020	32	Single	Secondary	Unemployed	Rural	Yes	No	No	No	Yes	No	No	No	No	No
242	HKB0622	Male	Malay	2018	31	Others	Tertiary	Private	Urban	Yes	Yes	No	No	No	No	No	No	No	No

No	Patient ID	Gender	Ethnic group	Year of diagnosis	Age	Marital status	Education level	Occupation	Place of residence	History of psy illness	Tobacco	Alcohol	Cannabis	ATS	Inhalant	Sedative	Hallucinogens	Opioids	Kratom
243	HKB0623	Male	Malay	2018	24	Single	Secondary	Unemployed	Urban	Yes	Yes	No	No	Yes	No	No	No	No	No
244	HKB0628	Male	Malay	2019	39	Married	No formal education	Others	Rural	Yes	Yes	No	No	Yes	No	No	No	No	No
245	HKB0632	Female	Malay	2020	21	Others	Tertiary	Unemployed	Rural	Yes	Yes	No	No	No	No	No	No	No	No
246	HKB0634	Male	Chinese	2019	49	Married	Secondary	Private	Rural	Yes	No	Yes	No	No	No	No	No	No	No
247	HKB0637	Male	Malay	2018	36	Others	Secondary	Others	Rural	Yes	No	No	No	Yes	No	No	No	No	No
248	HKB0639	Male	Malay	2020	35	Married	Secondary	Self-employed	Rural	Yes	Yes	No	No	Yes	No	No	No	No	No
249	HKB0642	Male	Malay	2019	38	Single	Tertiary	Government	Rural	Yes	Yes	No	No	No	No	No	No	No	No
250	HKB0646	Male	Malay	2020	28	Single	No formal education	Unemployed	Rural	Yes	No	No	No	Yes	No	No	No	No	No
251	HKB0648	Male	Malay	2020	24	Single	No formal education	Private	Rural	Yes	No	No	No	Yes	No	No	No	No	Yes
252	HKB0649	Male	Malay	2019	33	Married	Tertiary	Government	Urban	Yes	No	No	No	Yes	No	No	No	No	No
253	HKB0654	Male	Malay	2021	27	Married	Primary	Unemployed	Urban	Yes	No	No	No	No	No	No	No	Yes	Yes
254	HKB0656	Male	Malay	2019	32	Single	No formal education	Others	Rural	Yes	No	No	No	Yes	No	No	No	No	No
255	HKB0658	Male	Malay	2021	62	Married	No formal education	Unemployed	Rural	Yes	No	No	Yes	Yes	No	No	No	No	No
256	HKB0659	Male	Malay	2021	47	Married	Secondary	Private	Urban	Yes	No	No	No	Yes	No	No	No	No	No
257	HKB0660	Male	Malay	2021	35	Others	Secondary	Unemployed	Rural	Yes	No	No	No	Yes	No	No	No	No	No
258	HKB0665	Male	Malay	2018	31	Married	No formal education	Private	Rural	Yes	No	No	No	Yes	No	No	No	No	No
259	HKB0667	Male	Malay	2019	49	Married	Secondary	Others	Rural	Yes	No	No	No	Yes	No	No	No	Yes	No
260	HKB0668	Male	Malay	2020	27	Single	Tertiary	Unemployed	Rural	Yes	Yes	No	Yes	No	No	No	No	No	No
261	HKB0669	Male	Malay	2019	25	Married	No formal education	Unemployed	Rural	Yes	No	No	No	No	No	No	No	No	Yes
262	HKB0670	Male	Malay	2019	38	Married	Secondary	Self-employed	Rural	Yes	Yes	No	No	No	No	No	No	No	No
263	HKB0670	Male	Malay	2019	37	Married	Secondary	Self-employed	Rural	Yes	Yes	Yes	No	Yes	No	No	No	No	No
264	HKB0672	Male	Malay	2021	22	Single	Secondary	Unemployed	Rural	Yes	Yes	No	Yes	Yes	No	No	Yes	No	No
265	HKB0673	Male	Malay	2021	26	Single	Tertiary	Private	Rural	Yes	Yes	No	No	No	No	No	No	No	No
266	HKB0682	Male	Malay	2019	31	Single	No formal education	Unemployed	Rural	Yes	Yes	No	No	Yes	No	No	No	No	No
267	HKB0683	Male	Malay	2018	20	Single	Secondary	Unemployed	Rural	No	Yes	No	No	No	No	No	No	No	No
268	HKB0688	Male	Malay	2018	34	Others	No formal education	Others	Rural	Yes	No	No	No	Yes	No	No	No	No	No
269	HKB0691	Male	Malay	2019	18	Single	Tertiary	Unemployed	Rural	Yes	Yes	No	No	Yes	No	No	No	No	Yes
270	HKB0693	Male	Malay	2019	44	Married	Secondary	Self-employed	Rural	Yes	Yes	No	No	No	No	No	No	No	No
271	HKB0695	Male	Malay	2020	23	Others	Tertiary	Others	Urban	Yes	Yes	No	No	No	No	No	No	No	No
272	HKB0700	Male	Malay	2019	27	Single	No formal education	Others	Rural	Yes	Yes	No	No	Yes	No	No	No	No	No
273	HKB0701	Male	Malay	2020	20	Single	Secondary	Self-employed	Rural	Yes	No	No	No	Yes	No	Yes	No	No	Yes
274	HKB0706	Male	Chinese	2018	57	Married	No formal education	Self-employed	Rural	Yes	No	Yes	No	No	No	No	No	No	No
275	HKB0707	Male	Indian	2020	26	Single	Secondary	Unemployed	Urban	Yes	No	Yes	Yes	Yes	No	Yes	No	No	No

No	Patient ID	Gender	Ethnic group	Year of diagnosis	Age	Marital status	Education level	Occupation	Place of residence	History of psy illness	Tobacco	Alcohol	Cannabis	ATS	Inhalant	Sedative	Hallucinogens	Opioids	Kratom
276	HKB0711	Male	Malay	2019	55	Married	Secondary	Government	Rural	Yes	Yes	No	No	No	No	No	No	No	No
277	HKB0715	Male	Malay	2018	37	Others	No formal education	Unemployed	Rural	Yes	No	No	No	Yes	No	No	No	No	Yes
278	HKB0717	Male	Malay	2018	20	Single	No formal education	Others	Rural	Yes	No	No	No	Yes	No	No	No	No	Yes
279	HKB0718	Male	Malay	2018	29	Others	Secondary	Others	Urban	Yes	No	No	No	Yes	No	No	No	No	No
280	HKB0722	Male	Malay	2020	18	Single	Secondary	Unemployed	Rural	Yes	Yes	No	No	No	No	No	No	No	Yes
281	HKB0723	Male	Malay	2019	35	Others	No formal education	Others	Rural	Yes	No	No	No	No	No	No	No	Yes	No
282	HKB0726	Male	Malay	2021	29	Others	No formal education	Others	Rural	Yes	No	No	No	Yes	No	No	No	No	No
283	HKB0733	Male	Malay	2020	34	Others	Primary	Unemployed	Rural	Yes	No	No	No	Yes	No	No	No	No	No
284	HKB0735	Male	Malay	2018	33	Single	Secondary	Unemployed	Rural	Yes	Yes	No	No	Yes	No	No	No	Yes	Yes
285	HKB0737	Male	Malay	2018	30	Others	No formal education	Others	Rural	Yes	Yes	No	Yes	No	No	No	No	No	No
286	HKB0738	Male	Malay	2021	31	Single	No formal education	Unemployed	Rural	Yes	No	No	No	Yes	No	No	No	No	No
287	HKB0739	Male	Malay	2021	22	Married	Secondary	Self-employed	Rural	Yes	Yes	No	No	No	No	No	No	No	Yes
288	HKB0741	Male	Malay	2020	18	Single	Secondary	Self-employed	Urban	Yes	No	No	No	Yes	No	No	No	No	No
289	HKB0742	Male	Malay	2021	26	Single	No formal education	Unemployed	Rural	Yes	Yes	No	No	No	No	No	No	No	No
290	HKB0744	Male	Chinese	2020	27	Single	Tertiary	Self-employed	Rural	Yes	No	Yes	No	No	No	No	No	No	No
291	HKB0751	Male	Malay	2018	31	Others	Tertiary	Private	Urban	Yes	No	No	No	Yes	No	No	No	No	No
292	HKB0752	Male	Malay	2020	28	Single	Secondary	Private	Rural	Yes	No	No	No	Yes	No	No	No	No	No
293	HKB0753	Male	Malay	2020	22	Single	Secondary	Self-employed	Rural	Yes	No	No	No	Yes	No	No	No	No	Yes
294	HKB0758	Male	Malay	2019	49	Others	No formal education	Others	Rural	Yes	No	No	Yes	No	No	No	No	No	No
295	HKB0759	Male	Malay	2021	34	Married	Secondary	Private	Urban	Yes	Yes	No	No	No	No	No	No	No	No
296	HKB0766	Male	Malay	2021	30	Others	No formal education	Unemployed	Rural	Yes	Yes	No	No	No	No	No	No	No	No
297	HKB0776	Male	Malay	2018	36	Single	Secondary	Unemployed	Urban	Yes	Yes	No	No	No	No	No	No	No	No
298	HKB0778	Male	Malay	2018	28	Single	Primary	Unemployed	Rural	Yes	Yes	No	No	No	No	No	No	No	No
299	HKB0779	Male	Malay	2019	34	Single	No formal education	Unemployed	Rural	Yes	Yes	Yes	No	Yes	No	No	No	No	Yes
300	HKB0780	Male	Malay	2020	43	Married	Secondary	Private	Rural	Yes	No	No	No	Yes	No	No	No	No	No
301	HKB0781	Male	Malay	2019	42	Married	No formal education	Private	Rural	Yes	No	No	No	Yes	No	No	No	No	No
302	HKB0787	Male	Malay	2020	43	Married	Secondary	Unemployed	Urban	Yes	No	No	Yes	Yes	No	No	No	No	Yes
303	HKB0790	Male	Malay	2018	28	Others	No formal education	Others	Rural	Yes	No	No	No	No	No	No	No	Yes	No
304	HKB0791	Male	Malay	2018	24	Others	No formal education	Others	Rural	Yes	No	Yes	No	Yes	No	No	No	No	Yes
305	HKB0793	Male	Malay	2020	29	Single	No formal education	Others	Urban	Yes	Yes	No	No	Yes	No	No	No	No	No

No	Patient ID	Gender	Ethnic group	Year of diagnosis	Age	Marital status	Education level	Occupation	Place of residence	History of psy illness	Tobacco	Alcohol	Cannabis	ATS	Inhalant	Sedative	Hallucinogens	Opioids	Kratom
306	HKB0796	Male	Others	2021	25	Single	Secondary	Private	Urban	Yes	Yes	No	No	No	No	No	No	No	No
307	HKB0810	Male	Malay	2020	41	Others	No formal education	Unemployed	Rural	Yes	No	Yes	No	No	No	No	No	No	Yes
308	HKB0811	Male	Malay	2019	34	Single	Secondary	Unemployed	Rural	Yes	No	No	No	Yes	No	No	No	No	No
309	HKB0816	Male	Malay	2019	32	Married	Secondary	Self-employed	Rural	Yes	Yes	No	Yes	Yes	No	No	No	No	No
310	HKB0817	Male	Malay	2019	28	Single	Secondary	Private	Urban	Yes	No	No	No	Yes	No	No	No	No	Yes
311	HKB0818	Male	Malay	2020	25	Single	No formal education	Unemployed	Rural	Yes	No	No	No	Yes	No	No	No	No	No
312	HKB0821	Male	Malay	2018	23	Single	No formal education	Unemployed	Rural	Yes	No	No	No	No	No	No	No	Yes	Yes
313	HKB0825	Male	Malay	2018	21	Single	No formal education	Unemployed	Rural	Yes	No	No	No	No	No	No	No	Yes	Yes
314	HKB0827	Male	Malay	2019	33	Others	Secondary	Others	Urban	Yes	No	No	Yes	Yes	No	No	No	Yes	No
315	HKB0831	Male	Malay	2020	23	Single	No formal education	Others	Rural	Yes	No	No	No	No	No	No	No	No	Yes
316	HKB0834	Male	Malay	2020	25	Single	No formal education	Others	Urban	Yes	No	No	No	Yes	No	No	No	No	Yes
317	HKB0835	Male	Malay	2019	28	Married	Tertiary	Private	Urban	Yes	Yes	No	No	No	No	No	No	No	No
318	HKB0837	Male	Malay	2018	24	Others	No formal education	Private	Rural	Yes	No	No	No	No	No	No	No	No	Yes
319	HKB0840	Male	Malay	2019	36	Married	No formal education	Others	Rural	Yes	Yes	No	No	No	No	No	No	No	No
320	HKB0842	Male	Malay	2020	38	Others	No formal education	Private	Urban	Yes	Yes	No	Yes	Yes	No	No	No	Yes	Yes
321	HKB0850	Male	Malay	2020	44	Married	No formal education	Others	Rural	Yes	No	Yes	No	No	No	No	No	No	No
322	HKB0851	Male	Malay	2020	20	Single	Secondary	Unemployed	Rural	Yes	No	No	No	No	No	No	No	No	Yes
323	HKB0852	Male	Malay	2018	17	Single	Secondary	Unemployed	Urban	Yes	No	No	No	No	No	No	No	Yes	Yes
324	HKB0853	Male	Malay	2021	23	Single	Secondary	Self-employed	Rural	Yes	Yes	No	No	No	No	Yes	No	No	Yes
325	HKB0854	Male	Malay	2018	21	Single	No formal education	Self-employed	Rural	Yes	Yes	No	No	No	No	No	No	Yes	Yes
326	HKB0855	Male	Malay	2020	32	Married	Secondary	Self-employed	Rural	Yes	No	No	No	Yes	No	No	No	No	No
327	HKB0856	Male	Malay	2018	20	Single	Secondary	Self-employed	Rural	Yes	No	No	No	Yes	No	No	No	Yes	Yes
328	HKB0857	Male	Malay	2018	20	Single	Secondary	Unemployed	Rural	Yes	No	No	No	Yes	No	No	No	No	No
329	HKB0865	Male	Malay	2021	46	Married	No formal education	Self-employed	Rural	Yes	Yes	No	No	No	No	No	No	No	No
330	HKB0866	Male	Malay	2018	25	Others	No formal education	Others	Urban	Yes	No	No	No	Yes	No	No	No	No	No
331	HKB0868	Male	Malay	2019	30	Married	Secondary	Self-employed	Rural	Yes	Yes	No	No	No	No	No	No	No	No
332	HKB0869	Male	Malay	2018	35	Single	Tertiary	Government	Rural	Yes	No	No	No	Yes	No	No	No	No	No
333	HKB0874	Male	Malay	2019	34	Married	No formal education	Others	Rural	Yes	No	No	No	Yes	No	No	No	No	No
334	HKB0881	Male	Malay	2021	24	Single	Tertiary	Government	Urban	Yes	No	No	Yes	No	No	No	No	No	No

No	Patient ID	Gender	Ethnic group	Year of diagnosis	Age	Marital status	Education level	Occupation	Place of residence	History of psy illness	Tobacco	Alcohol	Cannabis	ATS	Inhalant	Sedative	Hallucinogens	Opioids	Kratom
335	HKB0882	Male	Malay	2019	29	Single	No formal education	Unemployed	Urban	Yes	Yes	No	No	Yes	No	No	No	No	Yes
336	HKB0884	Male	Malay	2018	28	Others	No formal education	Private	Rural	Yes	No	No	No	Yes	No	No	No	No	No
337	HKB0887	Male	Malay	2020	23	Single	Secondary	Private	Rural	Yes	No	No	No	Yes	No	No	No	No	No
338	HKB0890	Male	Malay	2021	25	Single	Secondary	Unemployed	Rural	Yes	No	No	No	No	No	No	No	No	Yes
339	HKB0891	Male	Malay	2020	19	Single	Secondary	Private	Urban	Yes	Yes	No	No	No	No	No	No	No	No
340	HKB0893	Male	Malay	2019	29	Single	Secondary	Private	Rural	Yes	No	No	No	Yes	No	No	No	No	Yes
341	HKB0894	Male	Malay	2020	37	Single	Tertiary	Self-employed	Rural	Yes	No	Yes	No	Yes	No	No	No	No	Yes
342	HKB0895	Male	Malay	2019	20	Single	No formal education	Private	Rural	Yes	Yes	No	No	Yes	No	No	No	No	No
343	HKB0897	Male	Malay	2019	24	Married	Secondary	Self-employed	Rural	Yes	No	No	No	Yes	No	No	No	No	No
344	HKB0898	Male	Malay	2018	21	Single	Secondary	Government	Rural	Yes	Yes	No	No	No	No	No	No	No	No
345	HKB0900	Male	Malay	2020	30	Single	No formal education	Unemployed	Rural	Yes	No	No	No	Yes	No	No	No	No	Yes
346	HKB0901	Male	Malay	2018	27	Others	Secondary	Self-employed	Urban	Yes	No	No	No	Yes	No	No	No	No	No
347	HKB0903	Male	Malay	2018	55	Others	Secondary	Unemployed	Rural	Yes	Yes	No	No	No	No	No	No	No	No
348	HKB0904	Male	Malay	2018	32	Others	Secondary	Unemployed	Rural	Yes	Yes	No	No	No	No	No	No	No	No
349	HKB0905	Male	Malay	2019	38	Single	No formal education	Self-employed	Rural	Yes	No	No	No	No	No	No	No	No	Yes
350	HKB0908	Male	Malay	2019	24	Single	Secondary	Others	Rural	Yes	Yes	No	No	No	No	No	No	No	No
351	HKB0910	Male	Malay	2018	23	Married	No formal education	Others	Rural	No	No	No	No	Yes	No	No	No	No	No
352	HKB0913	Male	Malay	2018	26	Others	Secondary	Unemployed	Urban	Yes	Yes	Yes	No	Yes	No	No	No	No	No
353	HKB0916	Male	Malay	2018	41	Others	No formal education	Unemployed	Rural	Yes	Yes	No	No	No	No	No	No	No	No
354	HKB0917	Male	Malay	2021	20	Single	No formal education	Others	Rural	No	Yes	No	No	No	No	No	No	No	No
355	HKB0921	Male	Malay	2018	21	Single	Secondary	Unemployed	Urban	Yes	Yes	No	No	No	No	No	No	No	No
356	HKB0923	Male	Malay	2019	44	Others	No formal education	Private	Urban	Yes	No	No	No	Yes	No	No	No	No	No
357	HKB0925	Male	Malay	2020	27	Others	No formal education	Private	Rural	Yes	No	Yes	No	Yes	No	No	No	Yes	Yes
358	HKB0927	Male	Malay	2019	25	Others	Secondary	Others	Rural	Yes	No	No	No	Yes	No	No	No	No	Yes
359	HKB0934	Male	Malay	2019	47	Single	Primary	Private	Rural	Yes	Yes	No	No	No	No	No	No	No	No
360	HKB0937	Male	Malay	2019	28	Others	No formal education	Others	Rural	Yes	No	No	No	No	No	No	No	No	Yes
361	HKB0939	Male	Malay	2020	27	Others	No formal education	Unemployed	Rural	Yes	No	No	No	Yes	No	No	No	No	No
362	HKB0940	Male	Malay	2018	55	Others	No formal education	Others	Rural	No	Yes	No	No	No	No	No	No	No	No
363	HKB0943	Male	Malay	2019	44	Others	Primary	Unemployed	Rural	Yes	No	No	No	Yes	No	No	No	No	No

No	Patient ID	Gender	Ethnic group	Year of diagnosis	Age	Marital status	Education level	Occupation	Place of residence	History of psy illness	Tobacco	Alcohol	Cannabis	ATS	Inhalant	Sedative	Hallucinogens	Opioids	Kratom
364	HKB0944	Male	Malay	2020	37	Others	No formal education	Others	Rural	Yes	No	No	No	Yes	No	No	No	No	No
365	HKB0949	Male	Malay	2019	21	Single	Tertiary	Unemployed	Urban	Yes	Yes	No	No	Yes	No	No	No	No	Yes
366	HKB0950	Male	Malay	2018	30	Single	No formal education	Unemployed	Rural	Yes	No	No	No	No	No	No	No	No	Yes
367	HKB0955	Male	Malay	2018	37	Others	No formal education	Government	Rural	Yes	No	No	No	Yes	No	No	No	No	No
368	HKB0956	Male	Malay	2018	32	Married	Secondary	Others	Rural	Yes	No	No	No	No	No	No	No	No	Yes
369	HKB0958	Male	Malay	2019	28	Married	Tertiary	Government	Rural	Yes	Yes	No	No	No	No	No	No	No	No
370	HKB0960	Male	Malay	2018	40	Others	No formal education	Others	Urban	Yes	Yes	No	No	No	No	No	No	Yes	No
371	HKB0961	Male	Malay	2020	37	Others	No formal education	Others	Rural	Yes	No	No	No	Yes	No	No	No	No	No
372	HKB0962	Male	Malay	2019	23	Single	No formal education	Others	Rural	Yes	No	No	Yes	Yes	No	No	No	Yes	Yes
373	HKB0963	Male	Malay	2018	39	Others	No formal education	Others	Urban	Yes	No	No	No	No	No	No	No	No	Yes
374	HKB0974	Male	Malay	2021	25	Single	Secondary	Unemployed	Rural	Yes	No	No	No	Yes	No	Yes	No	No	Yes
375	HKB0979	Male	Malay	2018	18	Single	Secondary	Unemployed	Rural	Yes	No	No	No	Yes	Yes	No	No	Yes	Yes
376	HKB0981	Male	Malay	2019	35	Married	No formal education	Others	Urban	Yes	Yes	No	No	No	No	No	No	No	No
377	HKB0987	Female	Others	2018	31	Married	Tertiary	Government	Urban	Yes	Yes	Yes	No	No	No	No	No	No	No
378	HKB0989	Male	Malay	2020	42	Others	No formal education	Unemployed	Rural	Yes	No	No	No	No	No	No	No	Yes	No
379	HKB1000	Male	Malay	2021	20	Single	No formal education	Self-employed	Rural	Yes	Yes	No	No	Yes	No	No	No	No	No
380	HKB1003	Male	Malay	2018	32	Married	Secondary	Self-employed	Rural	Yes	No	No	No	Yes	No	No	No	No	No
381	HKB1005	Male	Malay	2020	22	Others	Tertiary	Others	Urban	Yes	Yes	Yes	Yes	No	No	No	No	No	Yes
382	HKB1006	Male	Malay	2020	39	Married	No formal education	Unemployed	Rural	Yes	No	No	No	Yes	No	Yes	No	No	No
383	HKB1011	Male	Malay	2018	40	Single	Secondary	Self-employed	Rural	Yes	No	No	No	Yes	No	No	No	No	No
384	HKB1015	Male	Malay	2020	40	Married	No formal education	Others	Rural	Yes	No	No	No	Yes	No	No	No	No	No
385	HKB1016	Male	Malay	2018	27	Others	No formal education	Others	Rural	Yes	No	No	No	Yes	No	No	No	Yes	No
386	HKB1017	Male	Malay	2018	31	Others	No formal education	Others	Rural	Yes	No	No	No	Yes	No	No	No	Yes	No
387	HKB1020	Male	Malay	2019	27	Others	Tertiary	Government	Rural	Yes	Yes	No	No	Yes	No	No	No	No	No
388	HKB1024	Male	Malay	2018	29	Married	No formal education	Private	Urban	No	Yes	No	No	No	No	No	No	No	No
389	HKB1025	Male	Malay	2021	36	Married	Primary	Government	Rural	Yes	Yes	No	No	Yes	No	No	No	No	No
390	HKB1026	Male	Malay	2018	27	Single	No formal education	Unemployed	Rural	Yes	No	No	No	Yes	No	No	No	No	No
391	HKB1027	Male	Malay	2020	22	Single	Tertiary	Unemployed	Rural	Yes	Yes	No	No	No	No	No	No	No	No

No	Patient ID	Gender	Ethnic group	Year of diagnosis	Age	Marital status	Education level	Occupation	Place of residence	History of psy illness	Tobacco	Alcohol	Cannabis	ATS	Inhalant	Sedative	Hallucinogens	Opioids	Kratom
392	HKB1035	Male	Malay	2020	18	Single	Secondary	Private	Rural	Yes	Yes	No	No	No	No	No	No	No	Yes
393	HKB1036	Male	Malay	2020	27	Single	Tertiary	Self-employed	Rural	Yes	No	No	No	Yes	No	No	No	Yes	Yes
394	HKB1038	Male	Malay	2020	33	Married	No formal education	Others	Rural	Yes	No	No	No	Yes	No	No	No	No	No
395	HKB1042	Male	Malay	2020	30	Single	Primary	Private	Rural	Yes	No	No	No	No	No	No	No	No	Yes
396	HKB1043	Male	Malay	2019	34	Others	Secondary	Others	Urban	Yes	Yes	No	No	No	No	No	No	No	No
397	HKB1050	Male	Malay	2021	31	Others	No formal education	Unemployed	Urban	Yes	No	No	No	Yes	No	No	No	No	No
398	HKB1057	Male	Malay	2019	34	Others	Tertiary	Government	Rural	Yes	No	No	No	Yes	No	No	No	No	No
399	HKB1059	Male	Malay	2019	47	Married	No formal education	Self-employed	Rural	No	Yes	No	No	No	No	No	No	No	No
400	HKB1061	Male	Malay	2019	22	Single	Secondary	Unemployed	Rural	Yes	No	No	No	Yes	No	No	No	No	No
401	HKB1062	Male	Malay	2021	24	Single	Secondary	Unemployed	Rural	Yes	No	No	No	Yes	No	No	No	No	No
402	HKB1064	Male	Malay	2019	22	Single	Tertiary	Private	Urban	Yes	No	Yes	Yes	Yes	No	No	No	No	No
403	HKB1069	Female	Malay	2019	35	Married	Tertiary	Unemployed	Rural	Yes	Yes	No	Yes	No	No	No	No	No	No
404	HKB1070	Female	Malay	2019	48	Others	No formal education	Others	Rural	Yes	No	No	No	Yes	No	No	No	No	No
405	HKB1071	Female	Malay	2019	21	Single	Secondary	Self-employed	Rural	Yes	Yes	No	No	Yes	No	No	No	No	No
406	HKB1072	Female	Malay	2020	27	Others	Tertiary	Self-employed	Rural	Yes	No	No	Yes	Yes	No	No	No	No	No
407	HKB1074	Female	Malay	2018	23	Single	Tertiary	Unemployed	Rural	Yes	Yes	No	No	No	No	No	No	No	No
408	HKB1079	Male	Malay	2018	43	Married	No formal education	Private	Rural	Yes	No	No	No	No	No	No	No	No	Yes
409	HKB1080	Male	Malay	2021	28	Married	No formal education	Unemployed	Rural	Yes	No	No	Yes	Yes	No	No	No	No	Yes
410	HKB1081	Male	Malay	2019	42	Others	No formal education	Unemployed	Rural	Yes	Yes	No	No	No	No	No	No	No	No
411	HKB1082	Male	Malay	2019	21	Single	No formal education	Others	Rural	Yes	No	No	No	Yes	No	No	No	No	No
412	HKB1090	Male	Malay	2021	27	Single	No formal education	Others	Rural	Yes	Yes	No	No	No	No	No	No	No	No
413	HKB1091	Male	Malay	2019	23	Single	No formal education	Others	Rural	Yes	No	No	Yes	Yes	No	No	No	Yes	Yes
414	HKB1092	Male	Malay	2020	23	Single	Primary	Private	Rural	Yes	No	No	No	Yes	No	No	No	Yes	Yes
415	HKB1093	Male	Malay	2020	45	Married	No formal education	Private	Urban	Yes	No	No	No	Yes	No	No	No	No	No
416	HKB1098	Female	Malay	2018	19	Single	Secondary	Unemployed	Rural	Yes	Yes	No	No	No	No	No	No	No	No
417	HKB1099	Female	Malay	2018	35	Married	No formal education	Unemployed	Rural	Yes	Yes	No	No	No	No	No	No	Yes	No
418	HKB1100	Male	Malay	2020	24	Single	Tertiary	Unemployed	Urban	Yes	No	No	Yes	No	No	No	No	No	No
419	HKB1101	Male	Malay	2019	34	Single	Secondary	Self-employed	Rural	Yes	Yes	No	No	Yes	No	No	No	No	No
420	HKB1102	Male	Malay	2020	26	Single	Secondary	Unemployed	Rural	Yes	No	No	No	Yes	No	No	No	No	No
421	HKJ0008	Male	Malay	2018	49	Others	Secondary	Others	Urban	Yes	No	No	No	No	No	No	No	Yes	No
422	HKJ0013	Male	Chinese	2018	29	Others	No formal education	Others	Urban	Yes	No	No	No	Yes	No	No	No	No	No

No	Patient ID	Gender	Ethnic group	Year of diagnosis	Age	Marital status	Education level	Occupation	Place of residence	History of psy illness	Tobacco	Alcohol	Cannabis	ATS	Inhalant	Sedative	Hallucinogens	Opioids	Kratom
423	HKJ0016	Male	Malay	2018	23	Single	Primary	Unemployed	Rural	No	No	No	No	Yes	No	No	No	No	No
424	HKJ0024	Male	Malay	2018	47	Others	Secondary	Others	Urban	Yes	Yes	No	No	No	No	No	No	No	No
425	HKJ0031	Male	Malay	2018	25	Single	Secondary	Unemployed	Urban	Yes	No	No	Yes	No	No	No	No	No	No
426	HKJ0035	Male	Chinese	2018	29	Others	No formal education	Self-employed	Urban	Yes	No	No	No	Yes	No	No	No	No	No
427	HKJ0040	Male	Malay	2018	16	Single	Secondary	Others	Urban	Yes	Yes	No	No	No	No	No	No	No	No
428	HKJ0041	Male	Malay	2018	52	Others	Secondary	Private	Urban	Yes	Yes	No	No	No	No	No	No	No	No
429	HKJ0042	Male	Malay	2018	27	Single	Secondary	Others	Urban	Yes	No	No	No	Yes	No	No	No	No	No
430	HKJ0044	Male	Chinese	2018	33	Married	Tertiary	Private	Urban	No	No	Yes	No	No	No	No	No	No	No
431	HKJ0047	Female	Malay	2018	36	Others	Tertiary	Self-employed	Urban	Yes	Yes	No	No	No	No	No	No	No	No
432	HKJ0049	Male	Chinese	2018	72	Single	Primary	Others	Urban	Yes	Yes	No	No	No	No	No	No	No	No
433	HKJ0050	Male	Malay	2018	25	Single	No formal education	Private	Rural	Yes	Yes	Yes	No	Yes	No	No	Yes	No	No
434	HKJ0052	Male	Malay	2018	28	Married	Secondary	Others	Urban	Yes	Yes	No	No	No	No	No	No	No	No
435	HKJ0062	Male	Indian	2018	29	Single	No formal education	Others	Urban	Yes	No	No	No	Yes	No	No	No	No	No
436	HKJ0067	Male	Malay	2018	22	Single	Tertiary	Self-employed	Urban	Yes	Yes	No	No	No	No	No	No	No	No
437	HKJ0069	Female	Chinese	2018	40	Others	Secondary	Private	Urban	Yes	No	No	No	Yes	No	No	No	No	No
438	HKJ0071	Male	Chinese	2018	34	Married	No formal education	Others	Urban	Yes	Yes	Yes	No	No	No	No	No	No	No
439	HKJ0073	Male	Indian	2018	26	Single	No formal education	Private	Urban	Yes	Yes	Yes	No	No	No	No	No	No	No
440	HKJ0074	Female	Malay	2018	35	Others	No formal education	Others	Urban	Yes	Yes	No	No	No	No	No	No	No	No
441	HKJ0076	Male	Chinese	2018	36	Single	Secondary	Private	Urban	Yes	No	No	No	No	No	No	No	No	No
442	HKJ0079	Female	Malay	2018	71	Married	Primary	Self-employed	Urban	Yes	Yes	No	No	No	No	No	No	No	No
443	HKJ0080	Male	Malay	2018	38	Single	Secondary	Private	Urban	Yes	Yes	No	No	No	No	No	No	No	No
444	HKJ0081	Male	Malay	2018	43	Others	Tertiary	Private	Urban	Yes	Yes	Yes	No	No	No	No	No	No	No
445	HKJ0082	Female	Malay	2018	17	Others	Tertiary	Others	Urban	Yes	Yes	No	No	No	No	No	No	No	No
446	HKJ0083	Male	Malay	2018	22	Others	Secondary	Private	Urban	Yes	Yes	No	No	No	No	No	No	No	Yes
447	HKJ0085	Female	Others	2018	36	Married	Secondary	Unemployed	Urban	Yes	No	Yes	No	No	No	No	No	No	No
448	HKJ0087	Male	Indian	2018	56	Others	Tertiary	Private	Urban	Yes	No	Yes	No	No	No	No	No	No	No
449	HKJ0090	Male	Malay	2018	31	Single	No formal education	Others	Urban	No	Yes	No	No	Yes	No	No	No	No	No
450	HKJ0091	Male	Malay	2018	37	Single	Secondary	Unemployed	Urban	Yes	No	No	No	No	No	No	No	Yes	No
451	HKJ0092	Male	Malay	2018	29	Single	Tertiary	Private	Urban	Yes	No	Yes	No	No	No	No	No	No	No
452	HKJ0093	Male	Chinese	2018	21	Single	Tertiary	Others	Urban	Yes	Yes	Yes	No	No	No	No	No	No	No
453	HKJ0096	Male	Malay	2018	36	Single	Primary	Others	Urban	Yes	No	No	Yes	Yes	No	No	No	No	No
454	HKJ0097	Male	Chinese	2018	55	Married	Tertiary	Private	Urban	Yes	No	Yes	No	No	No	No	No	No	No
455	HKJ0098	Male	Others	2018	47	Others	No formal education	Others	Urban	Yes	Yes	No	No	No	No	No	No	No	No
456	HKJ0103	Male	Malay	2018	30	Single	No formal education	Private	Urban	Yes	Yes	No	No	No	No	No	No	No	No

No	Patient ID	Gender	Ethnic group	Year of diagnosis	Age	Marital status	Education level	Occupation	Place of residence	History of psy illness	Tobacco	Alcohol	Cannabis	ATS	Inhalant	Sedative	Hallucinogens	Opioids	Kratom
457	HKJ0104	Female	Chinese	2018	48	Single	No formal education	Self-employed	Urban	Yes	Yes	Yes	No	No	No	No	No	No	No
458	HKJ0106	Male	Malay	2018	55	Married	Secondary	Private	Urban	Yes	Yes	Yes	No	No	No	No	No	No	No
459	HKJ0107	Male	Malay	2018	55	Single	Secondary	Others	Urban	Yes	No	No	No	Yes	No	No	No	Yes	No
460	HKJ0108	Male	Malay	2018	31	Single	Secondary	Unemployed	Urban	Yes	Yes	No	No	No	No	No	No	No	No
461	HKJ0109	Male	Malay	2018	35	Others	Secondary	Unemployed	Urban	Yes	Yes	No	No	No	No	No	No	No	No
462	HKJ0111	Female	Malay	2018	14	Single	Secondary	Unemployed	Urban	No	Yes	No	No	No	No	No	No	No	No
463	HKJ0112	Female	Malay	2018	21	Single	Tertiary	Unemployed	Urban	No	No	Yes	Yes	Yes	No	No	No	Yes	No
464	HKJ0115	Male	Malay	2018	27	Single	Secondary	Private	Urban	No	Yes	No	Yes	Yes	No	No	No	No	No
465	HKJ0116	Male	Others	2018	41	Married	No formal education	Private	Urban	No	Yes	No	No	No	No	No	No	No	No
466	HKJ0118	Female	Others	2018	56	Others	No formal education	Private	Urban	Yes	Yes	Yes	No	No	No	No	No	No	No
467	HKJ0127	Male	Indian	2018	42	Single	Secondary	Unemployed	Urban	Yes	No	Yes	No	No	No	No	No	No	No
468	HKJ0128	Male	Malay	2018	34	Single	No formal education	Others	Urban	Yes	No	No	No	Yes	No	No	No	No	No
469	HKJ0129	Male	Chinese	2018	33	Others	Secondary	Private	Urban	Yes	No	No	Yes	Yes	No	No	No	No	No
470	HKJ0130	Female	Others	2018	27	Others	Tertiary	Others	Urban	Yes	No	No	No	No	No	No	No	No	Yes
471	HKJ0131	Male	Malay	2018	33	Married	No formal education	Others	Urban	Yes	Yes	No	No	No	No	No	No	No	No
472	HKJ0131	Male	Indian	2018	52	Married	Secondary	Unemployed	Urban	No	Yes	Yes	No	No	No	No	No	No	No
473	HKJ0134	Male	Indian	2018	64	Married	Tertiary	Others	Urban	Yes	Yes	Yes	No	No	No	No	No	No	No
474	HKJ0135	Male	Malay	2018	57	Others	Secondary	Private	Urban	No	Yes	No	No	No	No	No	No	No	No
475	HKJ0138	Male	Malay	2018	32	Single	Tertiary	Self-employed	Urban	Yes	Yes	Yes	No	No	No	No	No	No	No
476	HKJ0140	Male	Malay	2018	39	Married	No formal education	Private	Urban	Yes	Yes	No	No	No	No	No	No	No	No
477	HKJ0141	Male	Malay	2018	23	Single	Tertiary	Self-employed	Urban	Yes	Yes	No	No	No	No	No	No	No	No
478	HKJ0143	Female	Malay	2018	21	Others	Tertiary	Others	Urban	Yes	No	No	Yes	No	No	No	No	No	No
479	HKJ0144	Male	Chinese	2018	34	Single	Secondary	Unemployed	Urban	Yes	Yes	No	No	No	No	No	No	No	No
480	HKJ0145	Male	Others	2018	33	Single	No formal education	Others	Urban	Yes	Yes	No	No	No	No	No	No	No	No
481	HKJ0146	Male	Indian	2018	53	Others	Tertiary	Private	Urban	Yes	Yes	Yes	No	No	No	No	No	No	No
482	HKJ0148	Male	Indian	2018	70	Others	Secondary	Others	Urban	Yes	No	Yes	No	No	No	No	No	No	No
483	HKJ0151	Male	Chinese	2018	32	Married	Secondary	Private	Urban	Yes	Yes	No	Yes	No	No	No	No	No	No
484	HKJ0157	Male	Others	2018	38	Single	No formal education	Others	Urban	No	No	Yes	No	No	No	No	No	No	No
485	HKJ0158	Male	Malay	2018	22	Others	Tertiary	Others	Urban	Yes	Yes	No	No	No	No	No	No	No	No
486	HKJ0160	Female	Chinese	2018	31	Single	Secondary	Self-employed	Urban	Yes	Yes	Yes	No	No	No	No	No	No	No
487	HKJ0161	Male	Indian	2018	41	Others	Primary	Private	Urban	Yes	No	Yes	No	No	No	No	No	No	No
488	HKJ0162	Male	Chinese	2018	39	Single	Secondary	Others	Urban	Yes	Yes	No	No	No	No	No	No	No	No
489	HKJ0165	Male	Chinese	2018	36	Single	Primary	Unemployed	Urban	Yes	Yes	No	No	Yes	No	No	No	No	No
490	HKJ0166	Male	Malay	2018	20	Single	Secondary	Unemployed	Urban	Yes	Yes	No	No	No	No	No	No	No	No
491	HKJ0170	Female	Malay	2021	29	Others	Secondary	Self-employed	Urban	Yes	Yes	No	No	No	No	No	No	No	No
492	HKJ0172	Female	Chinese	2021	30	Single	Tertiary	Private	Urban	Yes	No	Yes	No	No	No	No	No	No	No

No	Patient ID	Gender	Ethnic group	Year of diagnosis	Age	Marital status	Education level	Occupation	Place of residence	History of psy illness	Tobacco	Alcohol	Cannabis	ATS	Inhalant	Sedative	Hallucinogens	Opioids	Kratom
493	HKJ0175	Male	Malay	2021	44	Married	Tertiary	Government	Urban	Yes	Yes	No	No	No	No	No	No	No	No
494	HKJ0177	Female	Malay	2021	28	Single	Tertiary	Private	Urban	Yes	Yes	Yes	No	No	No	No	No	No	No
495	HKJ0178	Female	Malay	2021	29	Single	Tertiary	Private	Urban	Yes	Yes	No	No	No	No	No	No	No	No
496	HKJ0179	Male	Malay	2018	22	Single	Tertiary	Private	Urban	Yes	Yes	No	No	No	No	No	No	No	No
497	HKJ0182	Male	Malay	2021	41	Others	Secondary	Private	Rural	No	Yes	No	No	No	No	No	No	No	No
498	HKJ0183	Female	Malay	2020	17	Others	Secondary	Private	Urban	Yes	Yes	No	No	No	No	No	No	No	No
499	HKJ0184	Female	Malay	2021	27	Single	Secondary	Unemployed	Urban	Yes	No	Yes	No	No	No	No	No	No	No
500	HKJ0185	Male	Malay	2019	30	Single	Tertiary	Private	Urban	Yes	Yes	No	No	No	No	No	No	No	No
501	HKJ0187	Male	Malay	2021	24	Married	Secondary	Private	Urban	Yes	No	No	Yes	Yes	No	No	No	No	Yes
502	HKJ0191	Male	Others	2018	39	Others	No formal education	Others	Urban	Yes	No	No	No	No	No	No	No	Yes	No
503	HKJ0192	Male	Malay	2021	29	Married	Secondary	Private	Urban	Yes	Yes	No	No	No	No	No	No	No	No
504	HKJ0193	Male	Indian	2018	24	Single	Secondary	Unemployed	Urban	Yes	Yes	No	No	No	No	No	No	No	No
505	HKJ0195	Male	Malay	2021	30	Single	Tertiary	Unemployed	Urban	Yes	Yes	No	No	Yes	No	No	No	No	No
506	HKJ0199	Male	Chinese	2021	55	Married	No formal education	Others	Urban	Yes	Yes	Yes	No	No	No	No	No	No	No
507	HKJ0203	Male	Malay	2021	31	Married	Secondary	Unemployed	Urban	Yes	No	No	No	Yes	No	No	No	No	No
508	HKJ0204	Male	Malay	2021	29	Single	Secondary	Private	Urban	Yes	Yes	No	No	Yes	No	No	No	No	No
509	HKJ0206	Male	Malay	2020	39	Others	Tertiary	Self-employed	Urban	Yes	No	No	No	Yes	No	No	No	No	No
510	HKJ0207	Male	Malay	2018	39	Married	Tertiary	Private	Urban	Yes	Yes	No	Yes	No	No	No	No	No	No
511	HKJ0208	Female	Malay	2021	43	Others	Tertiary	Self-employed	Urban	Yes	Yes	No	No	Yes	No	No	No	No	No
512	HKJ0210	Female	Malay	2021	31	Others	Secondary	Unemployed	Urban	Yes	Yes	Yes	No	Yes	No	No	No	No	No
513	HKJ0212	Male	Malay	2020	37	Others	Tertiary	Private	Urban	Yes	No	No	No	Yes	No	No	No	No	No
514	HKJ0213	Female	Malay	2021	23	Married	Secondary	Private	Urban	Yes	Yes	No	No	Yes	No	No	No	No	No
515	HKJ0214	Female	Malay	2021	29	Single	No formal education	Others	Urban	Yes	No	No	No	Yes	No	No	No	No	No
516	HKJ0215	Male	Malay	2018	42	Others	Secondary	Others	Urban	Yes	Yes	No	No	Yes	No	No	No	No	No
517	HKJ0216	Male	Chinese	2018	47	Single	Primary	Private	Urban	Yes	No	Yes	Yes	No	No	No	No	No	No
518	HKJ0217	Male	Malay	2018	31	Others	Tertiary	Others	Urban	Yes	Yes	Yes	No	Yes	No	No	No	No	No
519	HKJ0218	Male	Indian	2021	72	Others	No formal education	Self-employed	Urban	Yes	No	Yes	No	No	No	No	No	No	No
520	HKJ0221	Male	Malay	2021	32	Others	No formal education	Others	Urban	Yes	No	No	No	Yes	No	No	No	Yes	No
521	HKJ0224	Male	Indian	2020	55	Married	Secondary	Unemployed	Urban	Yes	No	Yes	No	No	No	No	No	No	No
522	HKJ0225	Male	Malay	2019	39	Married	Secondary	Unemployed	Urban	Yes	Yes	No	No	Yes	No	No	No	No	No
523	HKJ0227	Male	Malay	2020	27	Single	Tertiary	Private	Urban	Yes	No	Yes	No	Yes	No	No	No	No	No
524	HKJ0228	Female	Malay	2018	21	Single	Secondary	Unemployed	Urban	Yes	Yes	No	Yes	Yes	No	No	No	No	No
525	HKJ0229	Male	Malay	2018	46	Married	Secondary	Others	Urban	Yes	Yes	No	Yes	Yes	No	No	No	No	No
526	HKJ0233	Male	Chinese	2019	50	Married	Primary	Unemployed	Urban	Yes	No	No	No	Yes	No	Yes	No	No	No
527	HKJ0236	Male	Malay	2020	39	Married	Secondary	Government	Urban	Yes	Yes	No	No	No	No	No	No	No	Yes
528	HKJ0237	Male	Chinese	2019	36	Others	No formal education	Others	Urban	Yes	Yes	Yes	No	Yes	No	No	No	No	No
529	HKJ0238	Male	Malay	2021	22	Single	Tertiary	Others	Urban	Yes	Yes	No	No	No	No	No	No	No	No
530	HKJ0240	Male	Chinese	2019	70	Others	Secondary	Private	Urban	Yes	No	Yes	No	No	No	No	No	No	No

No	Patient ID	Gender	Ethnic group	Year of diagnosis	Age	Marital status	Education level	Occupation	Place of residence	History of psy illness	Tobacco	Alcohol	Cannabis	ATS	Inhalant	Sedative	Hallucinogens	Opioids	Kratom
531	HKJ0241	Male	Chinese	2019	23	Others	No formal education	Others	Urban	No	No	Yes	Yes	Yes	No	No	No	No	No
532	HKJ0242	Male	Malay	2019	25	Others	Tertiary	Private	Urban	No	Yes	No	No	No	No	No	No	No	No
533	HKJ0243	Male	Malay	2021	51	Married	Secondary	Unemployed	Urban	No	No	No	Yes	No	No	No	No	Yes	No
534	HKJ0244	Male	Malay	2021	39	Others	No formal education	Unemployed	Urban	No	No	No	No	Yes	No	No	No	No	No
535	HKJ0245	Female	Malay	2020	32	Married	Tertiary	Self-employed	Urban	Yes	Yes	Yes	No	No	No	No	No	No	No
536	HKJ0246	Male	Others	2020	38	Single	Secondary	Others	Urban	Yes	Yes	No	No	No	No	No	No	Yes	No
537	HKJ0248	Male	Chinese	2020	52	Single	No formal education	Private	Urban	No	Yes	No	No	Yes	No	No	No	No	No
538	HKJ0249	Male	Indian	2020	55	Married	No formal education	Private	Urban	No	No	Yes	No	No	No	No	No	No	No
539	HKJ0251	Male	Chinese	2020	35	Married	Tertiary	Private	Urban	Yes	No	Yes	No	No	No	No	No	No	No
540	HKJ0252	Male	Malay	2018	33	Single	Tertiary	Unemployed	Urban	Yes	Yes	No	No	Yes	No	No	No	No	No
541	HKJ0253	Male	Malay	2020	26	Single	Tertiary	Private	Urban	No	No	No	No	Yes	No	No	No	No	No
542	HKJ0254	Female	Malay	2020	32	Married	Tertiary	Private	Urban	Yes	No	No	No	Yes	No	No	No	No	No
543	HKJ0255	Female	Malay	2021	27	Married	Tertiary	Private	Urban	Yes	Yes	No	No	No	No	No	No	No	No
544	HKJ0256	Female	Chinese	2019	23	Others	Tertiary	Others	Urban	Yes	No	Yes	No	No	No	No	No	No	No
545	HKJ0257	Female	Chinese	2019	44	Others	No formal education	Private	Urban	Yes	No	Yes	No	No	No	No	No	No	No
546	HKJ0258	Female	Malay	2019	31	Others	Tertiary	Others	Urban	Yes	No	No	Yes	No	No	No	No	No	No
547	HKJ0260	Male	Chinese	2021	23	Single	Tertiary	Private	Urban	Yes	No	Yes	No	No	No	No	No	No	No
548	HKJ0261	Male	Chinese	2020	47	Others	Primary	Others	Urban	Yes	Yes	No	No	No	No	No	No	No	No
549	HKJ0264	Male	Malay	2019	30	Others	Secondary	Self-employed	Urban	Yes	Yes	No	No	No	No	No	No	No	No
550	HKJ0265	Male	Malay	2019	25	Others	Tertiary	Private	Urban	Yes	Yes	No	No	No	No	No	No	No	No
551	HKJ0266	Male	Indian	2019	69	Others	Tertiary	Unemployed	Urban	Yes	Yes	No	No	No	No	No	No	No	No
552	HKJ0267	Male	Malay	2020	18	Others	Tertiary	Others	Urban	Yes	No	Yes	No	No	No	No	No	No	No
553	HKJ0268	Female	Chinese	2019	36	Married	Secondary	Others	Urban	Yes	No	Yes	No	No	No	No	No	No	No
554	HKJ0270	Female	Malay	2019	21	Others	Tertiary	Others	Urban	Yes	Yes	Yes	No	No	No	No	No	No	No
555	HKJ0271	Female	Malay	2021	24	Single	Tertiary	Private	Urban	Yes	No	Yes	No	No	No	No	No	No	No
556	HKJ0274	Female	Indian	2021	15	Others	Secondary	Others	Urban	Yes	No	Yes	No	No	No	No	No	No	No
557	HKJ0277	Female	Chinese	2020	21	Others	Tertiary	Others	Urban	No	No	Yes	No	No	No	No	No	No	No
558	HKJ0278	Female	Indian	2020	39	Married	Secondary	Private	Urban	Yes	Yes	Yes	No	No	No	No	No	No	No
559	HKJ0280	Male	Indian	2021	52	Married	Secondary	Private	Urban	Yes	Yes	No	No	No	No	No	No	No	No
560	HKJ0281	Male	Malay	2021	18	Single	Tertiary	Unemployed	Urban	Yes	No	No	Yes	No	No	No	No	No	No
561	HKJ0283	Female	Others	2020	48	Married	Secondary	Private	Urban	No	No	Yes	No	No	No	No	No	No	No
562	HKJ0284	Male	Malay	2021	19	Single	Secondary	Private	Urban	Yes	Yes	No	No	No	No	No	No	No	No
563	HKJ0287	Male	Malay	2019	15	Single	Secondary	Unemployed	Urban	Yes	Yes	No	No	No	No	No	No	No	No
564	HKJ0288	Male	Malay	2020	27	Single	Tertiary	Others	Urban	No	No	No	Yes	No	No	No	No	No	No
565	HKJ0289	Male	Malay	2020	23	Single	Tertiary	Private	Urban	Yes	Yes	Yes	No	No	No	No	No	No	Yes
566	HKJ0291	Male	Chinese	2021	50	Married	Secondary	Private	Urban	No	No	No	No	No	No	No	No	Yes	No
567	HKJ0293	Male	Malay	2020	31	Married	Tertiary	Private	Urban	No	Yes	No	No	Yes	No	No	No	No	Yes
568	HKJ0297	Male	Malay	2019	49	Others	No formal education	Private	Urban	No	No	No	No	Yes	No	No	No	No	No

No	Patient ID	Gender	Ethnic group	Year of diagnosis	Age	Marital status	Education level	Occupation	Place of residence	History of psy illness	Tobacco	Alcohol	Cannabis	ATS	Inhalant	Sedative	Hallucinogens	Opioids	Kratom
569	HKJ0298	Male	Malay	2021	26	Single	Secondary	Private	Urban	No	No	No	No	Yes	No	No	No	No	No
570	HKJ0299	Male	Indian	2020	38	Others	Secondary	Private	Urban	Yes	Yes	Yes	No	No	No	No	No	No	No
571	HKJ0300	Male	Malay	2019	43	Single	Secondary	Private	Urban	Yes	Yes	Yes	No	No	No	No	No	Yes	No
572	HKJ0301	Male	Chinese	2018	48	Others	Secondary	Others	Urban	Yes	No	No	No	No	No	No	No	Yes	No
573	HKJ0303	Male	Chinese	2021	54	Married	Primary	Private	Urban	Yes	No	Yes	No	No	No	No	No	No	No
574	HKJ0304	Male	Malay	2020	29	Married	Secondary	Private	Urban	Yes	Yes	No	No	No	No	No	No	No	No
575	HKJ0305	Male	Malay	2021	28	Married	Tertiary	Private	Urban	Yes	Yes	No	No	No	No	No	No	No	No
576	HKJ0306	Male	Malay	2021	22	Single	Tertiary	Unemployed	Urban	Yes	Yes	No	No	No	No	No	No	No	No
577	HKJ0308	Male	Indian	2021	28	Single	Secondary	Unemployed	Urban	Yes	Yes	No	No	No	No	No	No	No	No
578	HKJ0310	Male	Chinese	2021	22	Others	Tertiary	Private	Urban	Yes	No	Yes	No	No	No	No	No	No	No
579	HKJ0311	Male	Malay	2021	36	Married	Tertiary	Private	Urban	Yes	Yes	No	No	No	No	No	No	No	No
580	HKJ0312	Male	Indian	2020	20	Others	Secondary	Others	Urban	Yes	Yes	Yes	No	No	No	No	No	No	No
581	HKJ0314	Male	Malay	2019	21	Single	Tertiary	Others	Urban	Yes	Yes	Yes	No	Yes	No	No	No	No	No
582	HKJ0315	Male	Malay	2021	27	Single	Tertiary	Private	Urban	Yes	Yes	No	No	No	No	No	No	No	No
583	HKJ0316	Male	Malay	2021	18	Single	Secondary	Private	Urban	Yes	Yes	No	No	No	No	No	No	No	No
584	HKJ0317	Female	Chinese	2021	35	Married	Tertiary	Unemployed	Urban	Yes	No	Yes	No	No	No	No	No	No	No
585	HKJ0318	Male	Malay	2021	35	Others	Tertiary	Self-employed	Urban	Yes	Yes	Yes	No	Yes	No	No	No	No	Yes
586	HKJ0319	Male	Chinese	2020	45	Others	Secondary	Unemployed	Urban	Yes	No	No	No	Yes	No	No	No	No	No
587	HKJ0320	Male	Malay	2020	49	Married	Secondary	Unemployed	Urban	Yes	Yes	No	No	Yes	No	No	No	No	No
588	HKJ0321	Female	Malay	2020	37	Others	Secondary	Private	Urban	Yes	Yes	No	No	No	No	No	No	No	No
589	HKJ0322	Female	Malay	2018	31	Others	Secondary	Private	Urban	No	Yes	No	No	No	No	No	No	No	No
590	HKJ0324	Male	Malay	2020	27	Single	Secondary	Private	Urban	Yes	Yes	No	No	No	No	No	No	No	No
591	HKJ0326	Male	Malay	2021	48	Others	Secondary	Private	Urban	Yes	Yes	No	No	No	No	No	No	No	No
592	HKJ0327	Male	Chinese	2021	55	Married	Tertiary	Private	Urban	Yes	Yes	No	No	No	No	No	No	No	No
593	HKJ0328	Male	Malay	2019	33	Others	Tertiary	Private	Urban	Yes	Yes	No	Yes	No	No	No	No	No	Yes
594	HKJ0329	Female	Malay	2020	32	Married	Tertiary	Private	Urban	Yes	Yes	No	No	No	No	No	No	No	No
595	HKJ0330	Female	Malay	2020	19	Others	Tertiary	Others	Urban	Yes	Yes	No	No	No	No	No	No	No	No
596	HKJ0331	Female	Malay	2021	18	Single	Tertiary	Others	Urban	Yes	Yes	No	No	No	No	No	No	No	No
597	HKJ0332	Female	Chinese	2019	22	Single	Tertiary	Others	Urban	Yes	No	Yes	No	No	No	No	No	No	No
598	HKJ0334	Male	Others	2019	36	Married	Primary	Others	Urban	Yes	No	Yes	No	No	No	No	No	No	No
599	HKJ0335	Male	Malay	2020	35	Married	Tertiary	Unemployed	Urban	Yes	Yes	No	No	No	No	No	No	No	No
600	HKJ0336	Male	Malay	2020	37	Married	Secondary	Private	Urban	Yes	No	Yes	Yes	Yes	Yes	No	No	No	No
601	HKJ0337	Male	Chinese	2019	74	Others	Primary	Others	Urban	Yes	No	Yes	No	No	No	No	No	No	No
602	HKJ0338	Male	Chinese	2020	28	Others	Tertiary	Private	Urban	Yes	No	Yes	No	No	No	No	No	No	No
603	HKJ0339	Male	Chinese	2020	21	Single	Tertiary	Private	Urban	Yes	Yes	Yes	No	No	No	No	No	No	Yes
604	HKJ0340	Male	Malay	2019	40	Married	Primary	Others	Urban	Yes	Yes	No	No	No	No	No	No	No	No
605	HKJ0342	Male	Indian	2019	37	Others	Secondary	Private	Urban	Yes	Yes	No	No	No	No	No	No	No	No
606	HKJ0343	Male	Malay	2019	19	Single	Tertiary	Others	Urban	Yes	Yes	No	No	No	No	No	No	No	No
607	HKJ0345	Male	Malay	2019	38	Married	Secondary	Private	Urban	Yes	Yes	Yes	Yes	Yes	No	No	No	No	Yes
608	HKJ0348	Male	Malay	2020	26	Others	Tertiary	Others	Urban	Yes	No	No	Yes	No	No	No	No	No	No
609	HKJ0349	Male	Malay	2020	26	Others	Tertiary	Private	Urban	Yes	Yes	No	No	No	No	No	No	No	No
610	HKJ0353	Male	Malay	2021	25	Single	Secondary	Private	Urban	Yes	No	No	Yes	Yes	No	No	No	No	No
611	HKJ0354	Female	Malay	2021	29	Single	Tertiary	Private	Urban	Yes	Yes	No	No	No	No	No	No	No	No
612	HKJ0355	Female	Malay	2020	21	Others	Tertiary	Private	Urban	Yes	Yes	No	No	No	No	No	No	No	No

No	Patient ID	Gender	Ethnic group	Year of diagnosis	Age	Marital status	Education level	Occupation	Place of residence	History of psy illness	Tobacco	Alcohol	Cannabis	ATS	Inhalant	Sedative	Hallucinogens	Opioids	Kratom
613	HKJ0358	Female	Malay	2020	32	Married	Tertiary	Private	Urban	Yes	No	Yes	No	No	No	No	No	No	No
614	HKJ0359	Male	Malay	2020	20	Others	Secondary	Private	Urban	Yes	Yes	Yes	No	Yes	No	No	No	No	No
615	HKJ0360	Male	Malay	2021	39	Others	Secondary	Private	Urban	Yes	Yes	No	No	No	No	No	No	No	No
616	HKJ0362	Male	Chinese	2019	54	Married	Primary	Unemployed	Urban	Yes	No	No	No	Yes	No	No	No	No	No
617	HKJ0364	Male	Malay	2020	30	Others	Tertiary	Others	Urban	Yes	Yes	No	No	No	No	No	No	No	No
618	HKJ0365	Male	Malay	2021	31	Single	Secondary	Private	Urban	Yes	Yes	Yes	Yes	Yes	No	No	No	No	No
619	HKJ0366	Male	Malay	2020	29	Married	Secondary	Private	Urban	Yes	No	Yes	No	No	No	No	No	No	No
620	HKJ0367	Male	Malay	2020	28	Single	Tertiary	Self-employed	Urban	Yes	No	No	No	Yes	No	No	No	No	Yes
621	HKJ0368	Male	Malay	2020	24	Others	Secondary	Private	Urban	Yes	Yes	No	No	No	No	No	No	No	No
622	HKJ0369	Male	Malay	2021	34	Married	No formal education	Others	Urban	No	Yes	No	No	No	No	No	No	No	No
623	HKJ0372	Male	Chinese	2021	52	Single	Secondary	Unemployed	Urban	Yes	No	No	No	No	No	No	No	Yes	No
624	HKJ0374	Female	Indian	2021	21	Single	Tertiary	Private	Urban	Yes	No	Yes	No	No	No	No	No	No	No
625	HKJ0375	Male	Malay	2019	40	Married	Primary	Private	Urban	Yes	No	No	No	No	No	Yes	No	No	No
626	HKJ0376	Male	Malay	2019	30	Single	Primary	Private	Urban	Yes	Yes	No	No	No	No	No	No	No	No
627	HKJ0378	Male	Malay	2021	38	Others	Secondary	Self-employed	Urban	Yes	Yes	No	No	No	No	No	No	No	Yes
628	HKJ0379	Male	Malay	2020	28	Married	Secondary	Private	Urban	Yes	Yes	Yes	No	No	No	No	No	No	No
629	HKJ0381	Female	Chinese	2021	30	Others	Tertiary	Unemployed	Urban	Yes	No	Yes	No	No	No	No	No	No	No
630	HKJ0383	Male	Chinese	2020	56	Single	No formal education	Self-employed	Urban	Yes	Yes	No	No	No	No	No	No	No	No
631	HKJ0384	Male	Malay	2020	24	Others	Tertiary	Private	Urban	Yes	Yes	No	No	No	No	No	No	No	No
632	HKJ0385	Male	Chinese	2019	35	Single	Secondary	Private	Urban	Yes	No	Yes	No	No	No	No	No	No	No
633	HKJ0387	Female	Chinese	2020	43	Married	Secondary	Unemployed	Urban	Yes	No	Yes	No	No	No	No	No	No	No
634	HKJ0389	Male	Malay	2020	34	Single	Secondary	Private	Urban	Yes	Yes	No	No	No	No	No	No	No	No
635	HKJ0391	Male	Malay	2021	27	Single	Tertiary	Unemployed	Urban	Yes	No	No	No	No	No	No	No	No	Yes
636	HKJ0392	Male	Chinese	2020	36	Single	Primary	Others	Urban	Yes	Yes	No	No	No	No	No	No	No	No
637	HKJ0393	Male	Chinese	2019	23	Single	Tertiary	Others	Urban	Yes	Yes	No	No	No	No	No	No	No	No
638	HKJ0394	Male	Indian	2020	23	Single	Secondary	Private	Urban	Yes	No	Yes	No	No	No	No	No	No	No
639	HKJ0395	Male	Malay	2020	35	Others	Tertiary	Private	Urban	Yes	Yes	No	No	No	No	No	Yes	No	No
640	HKJ0396	Male	Malay	2020	28	Single	Tertiary	Unemployed	Urban	Yes	Yes	No	No	No	No	No	No	No	Yes
641	HKJ0397	Female	Others	2020	24	Single	Secondary	Others	Urban	Yes	Yes	No	Yes	No	No	No	No	No	No
642	HKJ0398	Female	Malay	2021	26	Single	Tertiary	Others	Urban	Yes	No	No	Yes	No	No	No	No	No	No
643	HKJ0399	Female	Malay	2021	23	Others	Tertiary	Private	Urban	Yes	Yes	Yes	No	No	No	No	No	No	No
644	HKJ0400	Male	Malay	2019	47	Married	Tertiary	Others	Urban	Yes	Yes	No	No	No	No	No	No	No	No
645	HKJ0401	Male	Malay	2021	59	Married	Tertiary	Private	Urban	Yes	Yes	No	No	No	No	No	No	No	No
646	HKJ0403	Male	Chinese	2019	48	Married	Secondary	Private	Urban	Yes	Yes	Yes	No	No	No	No	No	No	No
647	HKJ0404	Male	Malay	2019	24	Others	Secondary	Private	Urban	Yes	No	Yes	Yes	No	No	No	No	No	No
648	HKJ0405	Male	Malay	2020	28	Others	Secondary	Private	Urban	Yes	Yes	Yes	No	No	No	No	No	No	Yes
649	HKJ0407	Male	Indian	2021	24	Others	Secondary	Unemployed	Urban	Yes	Yes	Yes	No	No	No	No	No	No	No
650	HKJ0408	Male	Indian	2020	20	Single	Secondary	Private	Urban	Yes	Yes	Yes	No	No	No	No	No	No	No
651	HKJ0409	Female	Malay	2021	25	Others	Tertiary	Private	Urban	Yes	Yes	No	No	No	No	No	No	No	No
652	HKJ0410	Female	Chinese	2020	44	Married	Secondary	Private	Urban	Yes	Yes	No	No	No	No	No	No	No	No
653	HKJ0411	Female	Malay	2020	25	Others	Secondary	Others	Urban	Yes	Yes	No	No	No	No	No	No	No	No
654	HKJ0412	Female	Chinese	2020	31	Others	Tertiary	Others	Urban	Yes	No	Yes	No	No	No	No	No	No	No

No	Patient ID	Gender	Ethnic group	Year of diagnosis	Age	Marital status	Education level	Occupation	Place of residence	History of psy illness	Tobacco	Alcohol	Cannabis	ATS	Inhalant	Sedative	Hallucinogens	Opioids	Kratom
655	HKJ0413	Male	Chinese	2020	54	Married	Tertiary	Private	Urban	Yes	Yes	No	No	No	No	No	No	No	No
656	HKJ0414	Female	Indian	2021	28	Married	Tertiary	Private	Urban	No	Yes	No	No	No	No	No	No	No	No
657	HKJ0415	Male	Chinese	2021	61	Married	Secondary	Private	Urban	Yes	Yes	Yes	No	No	No	No	No	No	No
658	HKJ0416	Female	Malay	2020	39	Others	Secondary	Private	Urban	Yes	Yes	No	No	No	No	No	No	No	No
659	HKJ0417	Male	Malay	2021	30	Others	Secondary	Private	Urban	Yes	Yes	No	Yes	Yes	No	No	No	No	Yes
660	HKJ0418	Female	Malay	2020	37	Others	Secondary	Unemployed	Urban	Yes	Yes	No	No	No	No	No	No	No	No
661	HKJ0419	Female	Chinese	2019	32	Married	Secondary	Unemployed	Urban	Yes	Yes	Yes	No	No	No	No	No	No	No
662	HKJ0420	Male	Chinese	2020	27	Single	Tertiary	Unemployed	Urban	Yes	No	No	No	Yes	No	No	No	No	No
663	HKJ0421	Male	Indian	2019	23	Others	Tertiary	Others	Urban	No	No	Yes	No	No	No	No	No	No	No
664	HKJ0422	Male	Chinese	2020	48	Others	Secondary	Private	Urban	Yes	No	Yes	No	No	No	No	No	No	No
665	HKJ0423	Male	Malay	2020	44	Single	Secondary	Self-employed	Urban	Yes	Yes	No	No	No	No	No	No	No	No
666	HKJ0424	Male	Indian	2021	40	Married	Tertiary	Unemployed	Urban	No	Yes	Yes	No	No	No	No	No	No	No
667	HKJ0425	Male	Malay	2019	32	Married	Secondary	Government	Urban	No	Yes	No	No	No	No	No	No	No	No
668	HKJ0426	Male	Malay	2020	32	Single	Secondary	Private	Urban	Yes	Yes	No	No	No	No	No	No	No	No
669	HKJ0429	Male	Malay	2020	58	Married	Secondary	Private	Urban	Yes	Yes	No	No	No	No	No	No	No	No
670	HKJ0429	Male	Malay	2020	59	Married	Secondary	Private	Urban	Yes	Yes	No	No	No	No	No	No	No	No
671	HKJ0430	Male	Malay	2020	24	Others	Secondary	Self-employed	Urban	Yes	Yes	Yes	No	No	No	No	No	No	No
672	HKJ0431	Male	Malay	2019	32	Others	Primary	Private	Urban	Yes	No	Yes	No	No	No	No	No	No	No
673	HKJ0433	Female	Malay	2020	23	Single	Tertiary	Unemployed	Urban	Yes	Yes	No	No	No	No	No	No	No	No
674	HKJ0434	Female	Malay	2020	28	Single	Tertiary	Self-employed	Urban	Yes	No	Yes	No	No	No	No	No	No	No
675	HKJ0435	Female	Chinese	2020	70	Others	Secondary	Private	Urban	Yes	No	Yes	No	No	No	No	No	No	No
676	HKJ0436	Female	Malay	2020	18	Single	Secondary	Unemployed	Urban	Yes	Yes	No	No	No	No	No	No	No	No
677	HKJ0437	Female	Malay	2019	19	Single	Tertiary	Others	Urban	Yes	Yes	Yes	No	No	No	No	No	No	No
678	HKJ0438	Male	Chinese	2021	29	Others	Tertiary	Private	Urban	Yes	No	No	No	Yes	No	No	No	No	No
679	HKJ0440	Male	Malay	2020	31	Others	Secondary	Private	Urban	Yes	Yes	No	No	No	No	No	No	No	No
680	HKJ0441	Female	Malay	2020	20	Others	Tertiary	Others	Urban	Yes	Yes	No	No	No	No	No	No	No	No
681	HKJ0442	Male	Malay	2020	22	Others	Secondary	Private	Urban	Yes	No	Yes	Yes	Yes	No	No	No	No	No
682	HKJ0443	Male	Malay	2020	42	Single	Secondary	Unemployed	Urban	Yes	Yes	No	No	No	No	No	No	No	No
683	HKJ0444	Male	Malay	2021	35	Married	Secondary	Private	Urban	No	Yes	Yes	Yes	Yes	No	No	No	No	No
684	HKJ0445	Male	Indian	2021	81	Married	No formal education	Others	Urban	Yes	Yes	No	No	No	No	No	No	No	No
685	HKJ0446	Male	Indian	2020	79	Married	Secondary	Others	Urban	Yes	Yes	No	No	No	No	No	No	No	No
686	HKJ0447	Male	Malay	2020	18	Single	Secondary	Unemployed	Urban	Yes	Yes	No	No	No	No	No	No	No	No
687	HKJ0448	Male	Malay	2020	17	Single	Tertiary	Others	Urban	No	Yes	No	No	No	No	No	No	No	No
688	HKJ0449	Male	Others	2020	31	Single	No formal education	Private	Urban	Yes	Yes	Yes	No	No	No	No	No	No	No
689	HKJ0450	Male	Malay	2019	43	Others	Secondary	Unemployed	Urban	Yes	Yes	No	No	No	No	No	No	No	No
691	HKJ0452	Male	Malay	2020	27	Single	Tertiary	Private	Urban	Yes	Yes	No	Yes	No	No	No	No	Yes	Yes
690	HKJ0452	Male	Malay	2021	35	Married	Tertiary	Government	Urban	Yes	Yes	No	No	No	No	No	No	No	No
692	HKJ0453	Male	Malay	2020	53	Married	Tertiary	Others	Urban	Yes	Yes	No	No	No	No	No	No	No	No
693	HKJ0454	Male	Malay	2021	45	Married	Secondary	Private	Urban	Yes	Yes	No	No	No	No	No	No	No	No
694	HKJ0455	Male	Chinese	2019	43	Single	Tertiary	Others	Urban	Yes	Yes	No	No	No	No	No	No	No	No
695	HKJ0456	Male	Indian	2021	56	Married	Secondary	Private	Urban	Yes	No	Yes	No	No	No	No	No	No	No

No	Patient ID	Gender	Ethnic group	Year of diagnosis	Age	Marital status	Education level	Occupation	Place of residence	History of psy illness	Tobacco	Alcohol	Cannabis	ATS	Inhalant	Sedative	Hallucinogens	Opioids	Kratom
696	HKJ0457	Male	Chinese	2020	30	Single	Tertiary	Private	Urban	Yes	Yes	Yes	No	No	No	No	No	No	No
697	HKJ0458	Male	Malay	2020	45	Married	Secondary	Self-employed	Urban	Yes	Yes	No	No	No	No	No	No	No	No
698	HKJ0459	Female	Malay	2020	22	Others	Secondary	Others	Urban	Yes	No	Yes	No	No	No	No	No	No	No
699	HKJ0460	Female	Chinese	2020	37	Others	Secondary	Unemployed	Urban	Yes	Yes	No	No	Yes	No	No	No	No	No
700	HKJ0461	Female	Indian	2020	27	Single	Tertiary	Private	Urban	Yes	No	Yes	No	No	No	No	No	No	No
702	HKJ0463	Male	Malay	2019	45	Single	Tertiary	Unemployed	Urban	No	Yes	No	No	No	No	No	No	No	No
701	HKJ0463	Male	Malay	2020	58	Others	Tertiary	Private	Urban	Yes	Yes	No	No	No	No	No	No	No	No
703	HKJ0464	Male	Indian	2019	51	Married	Secondary	Private	Urban	Yes	Yes	Yes	No	No	No	No	No	No	No
704	HKJ0466	Male	Malay	2019	19	Single	Tertiary	Others	Urban	Yes	No	Yes	No	No	No	No	No	No	Yes
705	HKJ0467	Male	Chinese	2021	20	Others	Tertiary	Others	Urban	Yes	No	Yes	No	No	No	No	No	No	No
706	HKJ0468	Male	Chinese	2019	15	Single	Secondary	Others	Urban	Yes	No	Yes	No	No	No	No	No	No	No
707	HKJ0469	Female	Chinese	2021	41	Married	Secondary	Private	Urban	Yes	Yes	No	No	No	No	No	No	No	No
708	HKJ0470	Female	Malay	2021	24	Others	Tertiary	Private	Urban	Yes	Yes	No	No	No	No	No	No	No	No
709	HKJ0471	Female	Malay	2019	24	Others	Tertiary	Unemployed	Urban	Yes	Yes	No	No	No	No	No	No	No	No
710	HKJ0472	Female	Malay	2021	24	Others	Secondary	Private	Urban	Yes	No	No	Yes	No	No	No	No	No	No
711	HKJ0473	Female	Malay	2021	16	Others	Secondary	Others	Urban	Yes	Yes	No	No	No	No	No	No	No	No
712	HKJ0474	Male	Others	2019	29	Single	Tertiary	Private	Urban	Yes	Yes	No	No	No	No	No	No	No	No
713	HKJ0475	Male	Chinese	2020	23	Others	Secondary	Unemployed	Urban	Yes	Yes	Yes	Yes	Yes	No	No	No	No	No
714	HKJ0476	Male	Malay	2020	23	Single	Tertiary	Others	Urban	Yes	No	Yes	No	No	No	No	No	No	No
715	HKJ0477	Male	Malay	2020	41	Married	Primary	Private	Urban	Yes	Yes	No	No	No	No	No	No	No	No
716	HKJ0478	Male	Indian	2021	35	Single	Tertiary	Private	Urban	Yes	No	Yes	Yes	No	No	No	No	No	No
717	HKJ0479	Female	Malay	2021	23	Single	Secondary	Private	Urban	Yes	Yes	No	No	No	No	No	No	No	No
718	HKJ0480	Female	Malay	2021	18	Married	Primary	Private	Urban	Yes	Yes	No	No	No	No	No	Yes	No	No
719	HKJ0481	Female	Malay	2020	38	Married	Secondary	Private	Urban	Yes	Yes	No	No	No	No	No	No	No	No
720	HKJ0482	Male	Malay	2020	38	Single	Tertiary	Private	Urban	Yes	Yes	No	No	No	No	No	No	No	No
721	HKJ0483	Male	Chinese	2019	78	Others	Secondary	Unemployed	Urban	Yes	No	Yes	No	No	No	Yes	No	No	No
722	HKJ0484	Male	Malay	2019	24	Others	Tertiary	Others	Urban	Yes	Yes	No	No	No	No	No	No	No	No
723	HKJ0485	Male	Malay	2021	25	Others	Secondary	Private	Urban	Yes	Yes	Yes	No	No	No	No	Yes	No	No
724	HKJ0486	Female	Malay	2021	16	Others	Secondary	Others	Urban	Yes	Yes	No	No	No	No	No	No	No	No
725	HKJ0487	Male	Chinese	2019	20	Single	Tertiary	Others	Urban	Yes	No	Yes	No	No	No	No	No	No	No
726	HKJ0488	Male	Chinese	2019	49	Single	Secondary	Private	Urban	Yes	No	Yes	No	No	No	No	No	No	No
727	HKJ0489	Male	Malay	2021	42	Married	Tertiary	Self-employed	Urban	Yes	Yes	No	No	No	No	No	No	No	No
728	HKJ0490	Male	Chinese	2019	35	Single	Primary	Others	Urban	Yes	No	No	No	Yes	No	No	No	No	No
729	HKJ0491	Male	Indian	2020	32	Single	Secondary	Private	Urban	Yes	No	Yes	No	No	No	No	No	No	No
730	HKJ0492	Male	Chinese	2020	43	Single	Secondary	Others	Urban	Yes	Yes	No	No	No	No	No	No	No	No
731	HKJ0493	Male	Chinese	2021	48	Single	Secondary	Self-employed	Urban	Yes	No	Yes	No	No	No	No	No	No	No
732	HKJ0494	Female	Chinese	2020	24	Single	Tertiary	Others	Urban	Yes	No	Yes	No	No	No	No	No	No	No
733	HKJ0495	Female	Others	2020	26	Others	Secondary	Unemployed	Urban	Yes	No	Yes	No	No	No	No	No	No	No
734	HKJ0496	Male	Chinese	2019	41	Others	Primary	Private	Urban	Yes	No	No	No	Yes	No	No	No	No	No
735	HKJ0497	Male	Others	2020	23	Others	No formal education	Private	Urban	Yes	No	Yes	No	No	No	No	No	No	No
736	HKJ0498	Male	Malay	2021	47	Others	Secondary	Unemployed	Urban	Yes	Yes	No	No	No	No	No	No	No	No
737	HKJ0499	Male	Indian	2019	72	Others	No formal education	Others	Urban	Yes	No	Yes	No	No	No	No	No	No	No

No	Patient ID	Gender	Ethnic group	Year of diagnosis	Age	Marital status	Education level	Occupation	Place of residence	History of psy illness	Tobacco	Alcohol	Cannabis	ATS	Inhalant	Sedative	Hallucinogens	Opioids	Kratom
738	HKJ0500	Male	Malay	2019	21	Others	Secondary	Others	Urban	Yes	Yes	No	No	No	No	No	No	No	No
739	HKJ0501	Male	Chinese	2020	25	Single	Secondary	Private	Urban	Yes	No	Yes	No	No	No	No	No	No	No
740	HKJ0502	Male	Malay	2021	25	Single	Secondary	Private	Urban	Yes	Yes	No	No	No	No	No	No	No	No
741	HKJ0503	Male	Malay	2020	22	Others	Secondary	Private	Urban	Yes	Yes	No	No	Yes	No	No	No	No	Yes
742	HKJ0504	Male	Malay	2020	37	Others	Secondary	Self-employed	Urban	Yes	No	No	No	Yes	No	No	No	No	No
743	HKJ0505	Male	Malay	2021	32	Married	Tertiary	Private	Urban	Yes	Yes	No	No	No	No	No	No	No	No
744	HKJ0506	Male	Chinese	2020	70	Others	Tertiary	Others	Urban	Yes	Yes	Yes	No	No	No	No	No	No	No
745	HKJ0507	Male	Malay	2021	29	Single	Tertiary	Self-employed	Urban	Yes	No	No	No	Yes	No	No	No	No	No
746	HKJ0508	Male	Indian	2020	38	Married	Secondary	Others	Urban	Yes	No	Yes	No	No	No	No	No	No	No
747	HKJ0509	Male	Malay	2019	44	Married	Secondary	Self-employed	Urban	Yes	Yes	No	No	No	No	No	No	No	No
748	HKJ0510	Male	Malay	2020	26	Single	Secondary	Private	Urban	Yes	No	No	Yes	No	No	No	No	No	No
749	HKJ0511	Male	Malay	2020	29	Single	Secondary	Private	Urban	Yes	Yes	No	No	No	No	No	No	No	No
750	HKJ0512	Female	Malay	2020	22	Others	Tertiary	Others	Urban	Yes	No	Yes	No	No	No	No	No	No	No
751	HKJ0513	Female	Malay	2020	26	Others	Tertiary	Others	Urban	Yes	Yes	No	No	No	No	No	No	No	No
752	HKJ0516	Male	Malay	2021	44	Married	Tertiary	Private	Urban	No	Yes	No	No	No	No	No	No	No	No
753	HKJ0522	Male	Malay	2020	24	Others	Secondary	Unemployed	Urban	Yes	No	No	Yes	Yes	No	No	No	No	No
754	HKJ0523	Male	Malay	2020	24	Others	Tertiary	Private	Urban	Yes	No	Yes	Yes	No	No	No	No	No	No
755	HKJ0524	Male	Chinese	2019	34	Single	Secondary	Unemployed	Urban	Yes	Yes	No	No	No	No	No	No	No	No
756	HKJ0525	Male	Malay	2021	26	Single	Tertiary	Unemployed	Urban	Yes	No	No	No	No	No	No	No	No	Yes
757	HKJ0526	Male	Malay	2021	50	Married	Secondary	Self-employed	Urban	Yes	Yes	No	No	No	No	No	No	No	No
758	HKJ0527	Male	Malay	2021	37	Married	Tertiary	Private	Urban	Yes	Yes	No	No	No	No	No	No	No	No
759	HKJ0528	Male	Malay	2019	36	Others	Tertiary	Unemployed	Urban	Yes	Yes	No	No	No	No	No	No	No	No
760	HKJ0529	Male	Malay	2020	41	Single	Tertiary	Private	Urban	Yes	Yes	No	No	No	No	No	No	No	No
761	HKJ0530	Male	Indian	2021	39	Others	No formal education	Others	Urban	Yes	No	No	Yes	Yes	No	No	No	Yes	No
762	HKJ0531	Male	Malay	2021	34	Others	No formal education	Unemployed	Urban	Yes	Yes	No	No	Yes	No	No	No	No	No
763	HKJ0532	Male	Malay	2021	42	Others	Tertiary	Private	Urban	Yes	Yes	No	No	No	No	No	No	No	No
764	HKJ0533	Male	Malay	2020	39	Others	Secondary	Private	Urban	Yes	No	No	No	Yes	No	No	No	No	No
765	HKJ0534	Male	Malay	2019	20	Single	Tertiary	Others	Urban	Yes	Yes	No	No	No	No	No	No	No	No
766	HKJ0535	Male	Indian	2019	76	Others	Secondary	Others	Urban	Yes	No	Yes	No	No	No	No	No	No	No
767	HKJ0536	Female	Malay	2019	44	Married	Tertiary	Government	Urban	Yes	Yes	No	No	No	No	No	No	No	No
768	HKJ0537	Male	Malay	2021	18	Single	Secondary	Others	Urban	Yes	No	No	Yes	No	No	No	No	No	No
769	HKJ0538	Male	Malay	2020	33	Married	Tertiary	Private	Urban	Yes	Yes	No	No	No	No	No	No	No	No
770	HKJ0539	Male	Chinese	2019	60	Married	Secondary	Unemployed	Urban	Yes	No	Yes	No	No	No	No	No	No	No
771	HKJ0540	Male	Chinese	2021	38	Single	Secondary	Unemployed	Urban	Yes	Yes	No	No	No	No	No	No	No	No
772	HKJ0541	Male	Malay	2020	33	Single	Secondary	Unemployed	Urban	Yes	No	No	No	Yes	No	No	No	No	No
773	HKJ0542	Female	Malay	2021	31	Married	Tertiary	Private	Urban	Yes	No	Yes	No	No	No	No	No	No	No
774	HKJ0554	Male	Indian	2021	37	Married	Tertiary	Others	Urban	Yes	No	Yes	No	No	No	No	No	No	No
775	HKJ0560	Male	Malay	2021	35	Others	Tertiary	Private	Urban	Yes	No	No	Yes	No	No	No	No	No	No
776	HKJ0562	Male	Chinese	2020	55	Single	No formal education	Unemployed	Urban	Yes	Yes	No	No	No	No	No	No	No	No
777	HKJ0563	Male	Others	2019	28	Single	Tertiary	Unemployed	Urban	Yes	No	No	No	Yes	No	No	No	No	No
778	HKJ0564	Male	Chinese	2020	53	Single	Primary	Unemployed	Urban	Yes	Yes	No	No	No	No	No	No	No	No

No	Patient ID	Gender	Ethnic group	Year of diagnosis	Age	Marital status	Education level	Occupation	Place of residence	History of psy illness	Tobacco	Alcohol	Cannabis	ATS	Inhalant	Sedative	Hallucinogens	Opioids	Kratom
779	HKJ0565	Male	Chinese	2019	59	Married	No formal education	Others	Urban	Yes	No	Yes	No	No	No	No	No	No	No
780	HKJ0566	Male	Malay	2019	36	Others	Secondary	Private	Urban	Yes	Yes	No	No	No	No	No	No	No	No
781	HKJ0567	Male	Indian	2019	36	Married	Tertiary	Others	Urban	Yes	No	Yes	No	No	No	No	No	No	No
782	HKJ0568	Male	Malay	2021	31	Single	Tertiary	Government	Urban	Yes	No	No	Yes	Yes	No	No	No	No	No
783	HKJ0569	Male	Malay	2019	18	Others	Tertiary	Others	Urban	Yes	Yes	No	No	No	No	No	No	No	No
784	HKJ0570	Female	Chinese	2019	28	Married	Secondary	Unemployed	Urban	Yes	No	Yes	No	No	No	No	No	No	No
785	HKJ0571	Female	Chinese	2020	59	Married	Primary	Unemployed	Urban	Yes	No	Yes	No	No	No	No	No	No	No
786	HKJ0572	Female	Others	2021	69	Others	Tertiary	Others	Urban	Yes	No	Yes	No	No	No	No	No	No	No
787	HKJ0573	Male	Indian	2020	42	Married	Secondary	Private	Urban	Yes	Yes	Yes	No	No	No	No	No	No	No
788	HKJ0574	Male	Chinese	2021	41	Others	Primary	Private	Urban	Yes	Yes	No	No	No	No	No	No	No	No
789	HKJ0575	Male	Malay	2020	39	Single	Secondary	Private	Urban	Yes	No	No	Yes	No	No	No	No	No	No
790	HKJ0576	Female	Chinese	2021	77	Others	Secondary	Others	Urban	Yes	No	Yes	No	No	No	No	No	No	No
791	HKJ0577	Male	Malay	2020	42	Married	Tertiary	Private	Urban	No	Yes	No	No	No	No	No	No	No	No
792	HKJ0578	Male	Malay	2019	23	Single	Secondary	Private	Urban	Yes	No	No	No	Yes	No	No	No	Yes	Yes
793	HKJ0579	Male	Others	2021	41	Single	Secondary	Private	Urban	Yes	Yes	No	No	No	No	No	No	No	No
794	HKJ0580	Male	Malay	2020	45	Married	No formal education	Self-employed	Urban	No	Yes	No	No	No	No	No	No	No	No
795	HKJ0581	Male	Chinese	2019	22	Single	Secondary	Private	Urban	Yes	Yes	No	No	No	No	No	No	No	No
796	HKJ0585	Female	Malay	2021	29	Single	Secondary	Private	Urban	Yes	No	Yes	No	No	No	No	No	No	No
797	HKJ0586	Female	Malay	2019	21	Others	Tertiary	Others	Urban	Yes	No	Yes	No	No	No	No	No	No	No
798	HKJ0587	Female	Malay	2021	30	Others	Tertiary	Private	Urban	Yes	Yes	No	No	No	No	No	No	No	No
799	HKJ0588	Male	Malay	2019	28	Married	Secondary	Unemployed	Urban	Yes	No	No	No	Yes	No	No	No	No	No
800	HKJ0589	Male	Malay	2021	76	Others	Primary	Unemployed	Urban	Yes	Yes	No	No	No	No	No	No	No	No
801	HKJ0590	Female	Malay	2021	19	Single	Tertiary	Others	Urban	Yes	Yes	Yes	No	No	No	No	No	No	No
802	HKJ0591	Male	Chinese	2020	34	Others	Secondary	Private	Urban	Yes	No	No	No	Yes	No	No	No	No	No
803	HKJ0592	Male	Malay	2020	36	Married	Secondary	Self-employed	Urban	Yes	Yes	No	No	No	No	No	No	No	Yes
804	HKJ0593	Male	Chinese	2019	32	Single	Tertiary	Unemployed	Urban	Yes	No	Yes	No	No	No	No	No	No	No
805	HKJ0594	Male	Indian	2019	32	Single	Secondary	Private	Urban	Yes	No	Yes	No	No	No	No	No	No	No
806	HKJ0603	Male	Chinese	2020	41	Married	Secondary	Private	Urban	Yes	No	Yes	No	No	No	No	No	No	No
807	HKJ0604	Male	Indian	2021	31	Married	Secondary	Private	Urban	Yes	No	Yes	No	No	No	No	No	No	No
808	HKJ0605	Male	Malay	2021	32	Married	Tertiary	Private	Urban	Yes	Yes	No	No	No	No	No	No	No	No
809	HKJ0606	Male	Malay	2021	26	Others	Secondary	Private	Urban	Yes	Yes	No	No	No	No	No	No	No	No
810	HKJ0607	Male	Malay	2020	17	Others	Secondary	Others	Urban	Yes	Yes	No	No	No	No	No	No	No	No
811	HKJ0608	Male	Malay	2020	19	Others	Tertiary	Others	Urban	Yes	Yes	Yes	No	No	No	No	No	No	No
812	HKJ0609	Male	Chinese	2019	19	Others	Secondary	Self-employed	Urban	No	Yes	No	No	No	No	No	No	No	No
813	HKJ0610	Female	Indian	2020	21	Others	Secondary	Private	Urban	Yes	No	Yes	No	No	No	No	No	No	No
814	HKJ0611	Male	Malay	2021	29	Married	Secondary	Private	Urban	Yes	Yes	No	No	No	No	No	No	No	No
815	HKJ0613	Male	Malay	2021	35	Others	Secondary	Private	Urban	Yes	Yes	No	No	No	No	No	No	No	No
816	HKJ0614	Female	Others	2021	25	Married	Secondary	Unemployed	Urban	Yes	No	Yes	No	No	No	No	No	No	No
817	HKJ0615	Female	Chinese	2019	22	Others	Tertiary	Others	Urban	Yes	No	Yes	No	No	No	No	No	No	No
818	HKJ0616	Female	Malay	2020	21	Others	Tertiary	Others	Urban	Yes	No	Yes	No	Yes	No	No	No	No	Yes
819	HKJ0621	Male	Malay	2021	24	Single	Tertiary	Unemployed	Urban	Yes	Yes	No	No	No	No	No	No	No	No
820	HKJ0622	Male	Malay	2021	30	Married	Secondary	Private	Urban	Yes	Yes	No	No	No	No	No	No	No	No

No	Patient ID	Gender	Ethnic group	Year of diagnosis	Age	Marital status	Education level	Occupation	Place of residence	History of psy illness	Tobacco	Alcohol	Cannabis	ATS	Inhalant	Sedative	Hallucinogens	Opioids	Kratom
821	HKJ0623	Male	Indian	2020	58	Married	Primary	Private	Urban	Yes	Yes	No	No	No	No	No	No	No	No
822	HKJ0624	Male	Malay	2021	30	Single	Tertiary	Others	Urban	Yes	Yes	No	No	No	No	No	No	No	No
823	HKJ0625	Male	Malay	2021	54	Married	Secondary	Private	Urban	Yes	Yes	No	No	No	No	No	No	No	No
824	HKJ0626	Male	Indian	2020	38	Married	Secondary	Private	Urban	Yes	Yes	Yes	No	No	No	No	No	No	No
825	HKJ0627	Male	Malay	2019	17	Single	Secondary	Others	Urban	Yes	Yes	No	No	No	No	No	No	No	No
826	HKJ0628	Male	Malay	2020	33	Single	Tertiary	Self-employed	Urban	Yes	No	No	No	Yes	No	No	No	No	No
827	HKJ0629	Male	Malay	2019	25	Others	No formal education	Others	Urban	Yes	Yes	No	No	No	No	No	No	No	No
828	HKJ0632	Female	Malay	2020	27	Married	Tertiary	Private	Urban	Yes	No	Yes	No	No	No	No	No	No	No
829	HKJ0633	Female	Malay	2021	24	Single	Tertiary	Others	Urban	Yes	Yes	No	No	No	No	No	No	No	No
830	HKJ0634	Female	Indian	2021	60	Married	Primary	Others	Urban	Yes	No	Yes	No	No	No	No	No	No	No
831	HKJ0636	Male	Malay	2020	22	Single	Tertiary	Unemployed	Urban	Yes	Yes	No	No	No	No	No	No	No	No
832	HKJ0637	Male	Malay	2019	37	Married	Secondary	Unemployed	Urban	Yes	Yes	No	No	No	No	No	No	No	No
833	HKJ0638	Male	Indian	2019	22	Single	Secondary	Private	Urban	Yes	Yes	Yes	No	No	No	No	No	No	No
834	HKJ0639	Male	Chinese	2020	24	Others	Secondary	Others	Urban	Yes	Yes	No	No	No	No	No	No	No	No
835	HKJ0640	Male	Malay	2019	15	Others	No formal education	Others	Urban	Yes	Yes	No	No	No	No	No	No	No	No
836	HKJ0641	Male	Indian	2020	42	Married	Tertiary	Private	Urban	Yes	Yes	Yes	No	No	No	No	No	No	No
837	HKJ0642	Male	Malay	2021	27	Single	Secondary	Others	Urban	Yes	Yes	No	No	No	No	No	No	No	No
838	HKJ0643	Male	Chinese	2019	22	Others	Secondary	Private	Urban	Yes	No	Yes	No	No	No	No	No	No	No
839	HKJ0644	Male	Malay	2021	33	Single	Secondary	Self-employed	Urban	Yes	No	Yes	No	No	No	No	No	No	No
840	HKJ0645	Male	Malay	2019	23	Single	Secondary	Self-employed	Urban	Yes	Yes	No	No	No	No	No	No	No	No
841	HKJ0646	Male	Malay	2021	22	Others	Secondary	Private	Urban	Yes	Yes	No	No	No	No	No	No	No	No
842	HKJ0647	Male	Chinese	2019	38	Others	Secondary	Unemployed	Urban	Yes	Yes	No	No	No	No	No	No	No	No
843	HKJ0648	Male	Malay	2020	29	Married	Secondary	Private	Urban	Yes	Yes	No	No	No	No	No	No	No	No
844	HKJ0649	Male	Malay	2021	24	Single	Tertiary	Unemployed	Urban	Yes	No	Yes	No	No	No	No	No	No	No
845	HKJ0650	Male	Indian	2021	41	Married	Primary	Private	Urban	Yes	Yes	Yes	No	No	No	No	No	No	No
846	HKJ0673	Male	Malay	2018	26	Single	Secondary	Private	Urban	Yes	Yes	No	No	No	No	No	No	No	No
847	HKJ0687	Male	Chinese	2021	28	Single	Tertiary	Private	Urban	Yes	Yes	Yes	No	No	No	No	No	No	No
848	HKJ0688	Male	Indian	2019	35	Married	Primary	Others	Urban	Yes	No	Yes	No	No	No	No	No	No	No
849	HKJ0689	Male	Indian	2020	49	Single	Secondary	Unemployed	Urban	Yes	Yes	No	No	No	No	No	No	No	No
850	HKJ0690	Male	Malay	2020	28	Others	Secondary	Unemployed	Urban	Yes	Yes	No	No	No	No	No	No	No	No
851	HKJ0691	Male	Malay	2020	34	Others	Tertiary	Government	Urban	Yes	Yes	No	No	No	No	No	No	No	No
852	HKJ0694	Male	Malay	2021	34	Single	No formal education	Government	Urban	Yes	Yes	No	No	No	No	No	No	No	No
853	HKJ0695	Female	Malay	2020	40	Married	Tertiary	Private	Urban	Yes	Yes	No	No	No	No	No	No	No	No
854	HKJ0696	Female	Malay	2019	33	Married	Secondary	Private	Urban	Yes	Yes	No	No	No	No	No	No	No	No
855	HKJ0697	Male	Malay	2021	17	Single	Secondary	Others	Urban	Yes	Yes	No	No	No	No	No	No	No	No
856	HKJ0698	Male	Malay	2020	17	Single	Primary	Self-employed	Urban	Yes	Yes	Yes	No	No	No	No	No	No	No
857	HKJ0699	Male	Chinese	2020	23	Others	Tertiary	Others	Urban	Yes	No	Yes	No	No	No	No	No	No	Yes
858	HKJ0700	Male	Malay	2021	32	Single	Tertiary	Private	Urban	Yes	Yes	No	No	Yes	No	No	No	No	No
859	HKJ0701	Female	Chinese	2019	34	Married	Primary	Self-employed	Urban	Yes	Yes	Yes	No	No	No	No	No	No	No
860	HKJ0702	Female	Chinese	2020	66	Married	Primary	Unemployed	Urban	Yes	No	Yes	No	No	No	No	No	No	No
861	HKJ0703	Female	Malay	2021	26	Single	Tertiary	Others	Urban	Yes	Yes	No	No	No	No	No	No	No	No

No	Patient ID	Gender	Ethnic group	Year of diagnosis	Age	Marital status	Education level	Occupation	Place of residence	History of psy illness	Tobacco	Alcohol	Cannabis	ATS	Inhalant	Sedative	Hallucinogens	Opioids	Kratom
862	HKJ0704	Female	Others	2020	43	Married	Secondary	Private	Urban	Yes	No	Yes	No	No	No	No	No	No	No
863	HKJ0705	Female	Malay	2019	21	Others	Tertiary	Private	Urban	Yes	Yes	Yes	No	Yes	No	No	No	No	No
864	HKJ0706	Female	Malay	2019	40	Others	No formal education	Private	Urban	Yes	Yes	No	No	No	No	No	No	No	No
865	HKJ0707	Female	Malay	2021	23	Single	Tertiary	Others	Urban	Yes	Yes	No	No	No	No	No	No	No	No
866	HKJ0708	Female	Chinese	2020	40	Others	Tertiary	Private	Urban	Yes	No	Yes	No	No	No	No	No	No	No
867	HKJ0709	Female	Malay	2021	17	Single	Primary	Others	Urban	Yes	Yes	No	Yes	Yes	Yes	No	No	No	No
868	HKJ0710	Female	Malay	2019	33	Married	Tertiary	Government	Urban	Yes	No	No	Yes	No	No	No	No	No	No
869	HKJ0711	Female	Chinese	2021	33	Others	Tertiary	Private	Urban	Yes	Yes	No	No	No	No	No	No	No	No
870	HKJ0712	Female	Chinese	2019	24	Single	Tertiary	Others	Urban	Yes	Yes	Yes	No	No	No	No	No	No	No
871	HKJ0713	Female	Chinese	2020	36	Single	Tertiary	Government	Urban	Yes	No	Yes	No	No	No	No	No	No	No
872	HKJ0714	Female	Chinese	2020	32	Others	Tertiary	Private	Urban	Yes	Yes	Yes	No	No	No	No	No	No	No
873	HKJ0715	Male	Chinese	2019	41	Single	Tertiary	Unemployed	Urban	Yes	Yes	No	No	No	No	No	No	No	No
874	HKJ0719	Male	Malay	2019	46	Married	Secondary	Private	Urban	Yes	Yes	No	No	No	No	No	No	No	No
875	HKJ0720	Male	Malay	2021	25	Single	Tertiary	Others	Urban	Yes	Yes	No	No	No	No	No	No	No	No
876	HKJ0721	Male	Indian	2020	36	Others	Primary	Private	Urban	Yes	Yes	Yes	No	No	No	No	No	No	No
877	HKJ0722	Male	Malay	2019	25	Others	Primary	Others	Urban	Yes	Yes	No	No	No	Yes	No	No	No	No
878	HKJ0723	Male	Malay	2019	22	Single	Tertiary	Others	Urban	Yes	Yes	No	Yes	No	No	No	No	Yes	No
879	HKJ0724	Male	Indian	2019	22	Others	Tertiary	Private	Urban	Yes	Yes	Yes	No	No	No	No	No	No	No
880	HKJ0725	Male	Malay	2021	31	Others	Secondary	Private	Urban	Yes	Yes	No	No	Yes	No	No	No	Yes	No
881	HKJ0726	Male	Malay	2020	28	Others	Tertiary	Self-employed	Urban	Yes	Yes	No	No	No	No	No	No	No	No
882	HKJ0727	Male	Chinese	2021	58	Single	Secondary	Private	Urban	Yes	No	Yes	No	No	No	No	No	No	No
883	HKJ0728	Male	Indian	2020	54	Married	No formal education	Others	Urban	Yes	No	Yes	No	No	No	No	No	No	No
884	HKJ0729	Male	Chinese	2019	82	Others	Secondary	Others	Urban	Yes	No	Yes	No	No	No	No	No	No	No
885	HKJ0742	Female	Malay	2021	22	Married	Tertiary	Private	Urban	Yes	Yes	No	No	No	No	No	No	No	No
886	HKJ0743	Female	Malay	2020	41	Married	Secondary	Private	Urban	Yes	Yes	Yes	No	No	No	No	No	No	No
887	HKJ0744	Female	Malay	2021	37	Married	Secondary	Unemployed	Urban	Yes	No	No	No	No	No	No	No	Yes	No
888	HKJ0745	Male	Malay	2019	25	Single	Tertiary	Private	Urban	Yes	Yes	No	No	No	No	No	No	No	No
889	HKJ0746	Male	Malay	2020	33	Single	Primary	Others	Urban	Yes	Yes	No	No	No	No	No	No	No	No
890	HKJ0747	Male	Chinese	2020	33	Others	No formal education	Others	Urban	Yes	No	No	No	No	No	Yes	No	No	No
891	HKJ0748	Male	Chinese	2020	34	Others	Tertiary	Unemployed	Urban	Yes	Yes	Yes	No	No	No	No	No	No	No
892	HKJ0749	Male	Malay	2020	31	Married	No formal education	Private	Urban	Yes	Yes	No	No	No	No	No	No	No	No
893	HKJ0750	Male	Chinese	2021	49	Others	Primary	Others	Urban	Yes	Yes	No	Yes	No	No	No	No	No	No
894	HKJ0751	Female	Malay	2021	28	Single	Tertiary	Unemployed	Urban	Yes	Yes	No	Yes	Yes	No	No	No	No	No
895	HKJ0752	Male	Indian	2021	24	Single	Tertiary	Unemployed	Urban	Yes	No	Yes	No	No	No	No	No	No	No
896	HKJ0753	Male	Malay	2021	47	Others	Secondary	Unemployed	Urban	Yes	Yes	No	No	No	No	No	No	No	Yes
897	HKJ0754	Male	Indian	2020	24	Others	Secondary	Unemployed	Urban	No	Yes	Yes	No	Yes	No	No	No	No	No
898	HKJ0755	Male	Malay	2021	23	Single	Tertiary	Self-employed	Urban	Yes	Yes	No	No	No	No	No	No	No	No
899	HKJ0756	Male	Malay	2019	34	Others	No formal education	Private	Urban	Yes	Yes	No	No	Yes	No	No	No	No	No
900	HKJ0758	Male	Malay	2021	49	Others	Tertiary	Private	Urban	Yes	Yes	No	No	No	No	No	No	No	No

No	Patient ID	Gender	Ethnic group	Year of diagnosis	Age	Marital status	Education level	Occupation	Place of residence	History of psy illness	Tobacco	Alcohol	Cannabis	ATS	Inhalant	Sedative	Hallucinogens	Opioids	Kratom
901	HKJ0759	Female	Malay	2019	25	Others	Secondary	Others	Urban	Yes	Yes	No	No	No	No	No	No	No	No
902	HKJ0760	Male	Malay	2020	40	Married	Secondary	Private	Urban	Yes	No	No	No	Yes	No	No	No	No	No
903	HKJ0761	Female	Others	2021	22	Others	Tertiary	Private	Urban	No	No	Yes	No	No	No	No	No	No	No
904	HKJ0762	Male	Indian	2020	23	Single	No formal education	Unemployed	Urban	Yes	Yes	No	No	No	No	No	No	No	No
905	HKJ0763	Female	Chinese	2021	69	Single	Secondary	Unemployed	Urban	Yes	No	Yes	No	No	No	No	No	No	No
906	HKJ0764	Female	Malay	2020	20	Single	Secondary	Private	Urban	Yes	Yes	No	No	No	No	No	No	No	No
907	HKJ0766	Male	Malay	2021	26	Single	Primary	Self-employed	Urban	Yes	No	Yes	Yes	No	No	No	No	No	No
908	HKJ0767	Female	Malay	2021	21	Married	Secondary	Unemployed	Urban	Yes	Yes	No	No	No	No	No	No	No	No
909	HKJ0768	Female	Indian	2020	29	Single	No formal education	Unemployed	Urban	Yes	No	Yes	No	No	No	No	No	No	No
910	HKJ0770	Female	Chinese	2020	18	Others	Tertiary	Others	Urban	Yes	No	Yes	No	No	No	No	No	No	No
911	HKJ0771	Male	Others	2020	27	Single	No formal education	Private	Urban	No	No	Yes	No	No	No	No	No	No	No
912	HKJ0772	Male	Malay	2021	28	Others	Tertiary	Unemployed	Urban	Yes	Yes	Yes	No	No	No	No	No	No	No
913	HKJ0774	Female	Malay	2021	27	Others	Secondary	Unemployed	Urban	Yes	No	No	No	Yes	No	No	No	No	No
914	HKJ0775	Male	Malay	2021	32	Married	Tertiary	Self-employed	Urban	Yes	Yes	No	No	No	No	No	No	No	No
915	HKJ0776	Male	Chinese	2021	29	Single	Secondary	Private	Urban	Yes	No	No	No	No	No	No	No	No	No
916	HKJ0777	Male	Chinese	2020	42	Married	Secondary	Others	Urban	Yes	Yes	No	No	No	No	No	No	No	No
917	HKJ0778	Male	Indian	2019	45	Others	Primary	Others	Urban	Yes	Yes	No	No	No	Yes	No	No	No	No
918	HKJ0779	Male	Malay	2021	29	Others	No formal education	Others	Urban	Yes	Yes	Yes	No	No	No	No	No	No	No
919	HKJ0780	Male	Malay	2021	35	Married	Tertiary	Private	Urban	Yes	Yes	No	No	Yes	No	No	No	No	No
920	HKJ0782	Male	Malay	2020	32	Married	Tertiary	Private	Urban	Yes	Yes	No	No	No	No	No	No	No	No
921	HKJ0784	Female	Malay	2021	28	Married	Tertiary	Private	Urban	Yes	Yes	Yes	No	No	No	No	No	No	No
922	HKJ0785	Female	Indian	2020	22	Others	Tertiary	Others	Urban	Yes	No	Yes	No	No	No	No	No	No	No
923	HKJ0786	Female	Indian	2020	19	Single	Tertiary	Others	Urban	Yes	No	Yes	No	No	No	No	No	No	No
924	HKJ0787	Male	Malay	2021	32	Others	Tertiary	Private	Urban	Yes	Yes	No	No	No	No	No	No	No	No
925	HKJ0788	Male	Chinese	2019	79	Married	Primary	Private	Urban	Yes	No	Yes	No	No	No	No	No	No	No
926	HKJ0789	Male	Malay	2020	18	Single	Primary	Unemployed	Urban	Yes	Yes	Yes	No	No	No	No	No	No	No
927	HKJ0791	Female	Malay	2021	26	Single	Secondary	Unemployed	Urban	Yes	No	No	No	Yes	No	No	No	No	No
928	HKJ0792	Male	Chinese	2020	36	Single	Secondary	Private	Urban	Yes	Yes	Yes	No	No	No	No	No	No	No
929	HKJ0793	Female	Chinese	2020	24	Single	Tertiary	Others	Urban	Yes	No	Yes	No	No	No	No	No	No	No
930	HKJ0794	Male	Malay	2020	23	Single	Tertiary	Others	Urban	Yes	Yes	No	No	Yes	No	No	No	No	No
931	HKJ0795	Male	Others	2020	37	Others	Primary	Private	Urban	Yes	Yes	No	No	No	No	No	No	No	No
932	HKJ0796	Female	Chinese	2020	42	Married	Tertiary	Private	Urban	Yes	No	Yes	No	No	No	No	No	No	No
933	HKJ0797	Male	Malay	2019	18	Others	Secondary	Others	Urban	Yes	Yes	No	No	No	No	No	No	No	No
934	HKJ0798	Male	Chinese	2020	40	Others	No formal education	Others	Urban	Yes	No	No	No	Yes	No	No	No	No	No
935	HKJ0799	Male	Malay	2021	31	Married	Secondary	Private	Urban	Yes	Yes	No	No	No	No	No	No	No	No
936	HKJ0800	Male	Chinese	2021	42	Married	Primary	Private	Urban	Yes	Yes	Yes	No	No	No	No	No	No	No
937	HKJ0801	Male	Indian	2021	46	Married	Primary	Private	Urban	Yes	Yes	Yes	No	Yes	No	No	No	No	No
938	HKJ0802	Male	Malay	2020	34	Others	Tertiary	Unemployed	Urban	Yes	Yes	No	Yes	No	No	No	No	No	No
939	HKJ0803	Male	Indian	2018	27	Single	Tertiary	Unemployed	Urban	Yes	No	Yes	Yes	No	No	No	No	No	No

No	Patient ID	Gender	Ethnic group	Year of diagnosis	Age	Marital status	Education level	Occupation	Place of residence	History of psy illness	Tobacco	Alcohol	Cannabis	ATS	Inhalant	Sedative	Hallucinogens	Opioids	Kratom
940	HKJ0804	Male	Malay	2018	27	Single	Secondary	Unemployed	Urban	Yes	Yes	No	No	No	No	No	No	No	No
941	HKJ0805	Male	Malay	2020	28	Others	Tertiary	Others	Urban	Yes	Yes	No	No	No	No	No	No	No	No
942	HKJ0806	Male	Malay	2020	32	Single	Secondary	Unemployed	Urban	Yes	No	Yes	No	No	No	No	No	No	No
943	HKJ0807	Male	Chinese	2020	43	Married	Primary	Private	Urban	Yes	No	Yes	No	No	No	No	No	No	No
944	HKJ0808	Female	Indian	2019	22	Single	Secondary	Unemployed	Urban	Yes	No	No	Yes	Yes	No	No	No	No	No
945	HKJ0809	Male	Malay	2021	33	Single	Secondary	Private	Urban	Yes	Yes	No	No	No	No	No	No	No	No
946	HKJ0810	Male	Malay	2020	25	Others	No formal education	Others	Urban	Yes	No	No	No	No	No	No	No	No	No
947	HKJ0811	Male	Indian	2019	29	Single	No formal education	Unemployed	Urban	Yes	No	Yes	No	No	No	No	No	No	No
948	HKJ0812	Male	Others	2020	29	Others	Primary	Unemployed	Urban	Yes	Yes	No	No	No	No	No	No	No	No
949	HKJ0813	Male	Indian	2019	55	Married	Secondary	Self-employed	Urban	Yes	No	No	Yes	No	No	No	No	Yes	No
950	HKJ0814	Male	Malay	2021	31	Others	No formal education	Unemployed	Urban	Yes	No	No	No	Yes	No	No	No	No	No
951	HKJ0815	Male	Malay	2019	46	Others	No formal education	Others	Urban	Yes	No	No	Yes	No	No	No	No	No	Yes
952	HKJ0816	Male	Chinese	2021	46	Others	No formal education	Unemployed	Urban	Yes	No	No	No	Yes	No	No	No	Yes	No
953	HKJ0819	Male	Chinese	2020	35	Single	Primary	Self-employed	Urban	Yes	Yes	Yes	No	Yes	No	No	No	No	No
954	HKJ0820	Female	Chinese	2020	42	Married	Primary	Private	Urban	Yes	No	Yes	No	No	No	No	No	No	No
955	HKJ0821	Male	Malay	2021	24	Single	Tertiary	Private	Urban	Yes	Yes	No	No	No	No	No	No	No	No
956	HKJ0822	Male	Malay	2019	40	Married	Secondary	Others	Urban	Yes	Yes	No	No	No	No	No	No	Yes	No
957	HKJ0823	Female	Malay	2020	19	Single	Tertiary	Others	Urban	Yes	Yes	No	No	No	No	No	No	No	No
958	HKJ0824	Male	Chinese	2020	37	Others	Primary	Self-employed	Urban	Yes	No	Yes	No	Yes	No	No	No	No	No
959	HKJ0825	Male	Chinese	2018	48	Single	Secondary	Unemployed	Urban	Yes	Yes	Yes	No	No	No	No	No	No	No
960	HKJ0826	Male	Malay	2018	53	Married	Secondary	Private	Urban	Yes	Yes	No	No	No	No	No	No	No	No
961	HKJ0827	Male	Malay	2019	41	Married	Secondary	Unemployed	Urban	Yes	Yes	No	No	No	No	No	No	Yes	No
962	HKJ0828	Male	Malay	2020	29	Single	Tertiary	Private	Urban	Yes	No	No	No	Yes	No	No	No	No	Yes
963	HKJ0829	Male	Indian	2021	36	Single	Secondary	Private	Urban	Yes	No	Yes	No	No	No	No	No	No	No
964	HKJ0830	Male	Chinese	2019	33	Others	Tertiary	Unemployed	Urban	Yes	No	Yes	No	No	No	No	No	No	No
965	HKJ0831	Female	Malay	2020	29	Single	Tertiary	Others	Urban	Yes	Yes	No	No	Yes	No	No	No	No	No
966	HKJ0832	Male	Malay	2019	31	Married	No formal education	Private	Urban	Yes	No	No	No	Yes	No	No	No	No	No
967	HKJ0833	Male	Malay	2018	37	Others	Secondary	Unemployed	Urban	Yes	Yes	No	No	No	No	No	No	No	No
968	HKJ0834	Male	Malay	2019	40	Others	Tertiary	Private	Urban	Yes	Yes	No	No	Yes	No	No	No	No	No
969	HKJ0835	Male	Malay	2019	31	Single	Secondary	Private	Urban	Yes	No	No	No	Yes	No	No	No	No	No
970	HKJ0836	Female	Malay	2021	34	Others	Secondary	Unemployed	Urban	Yes	No	No	No	No	No	No	No	Yes	No
971	HKJ0837	Male	Indian	2021	45	Others	Primary	Unemployed	Urban	Yes	Yes	No	No	No	No	No	No	No	No
972	HKJ0838	Male	Malay	2021	39	Married	No formal education	Others	Urban	Yes	Yes	No	No	Yes	No	No	No	No	No
973	HKJ0839	Male	Malay	2019	29	Single	Tertiary	Unemployed	Urban	Yes	Yes	No	No	No	No	No	No	No	No
974	HKJ0840	Female	Malay	2018	25	Others	Secondary	Private	Urban	Yes	Yes	No	No	Yes	No	No	No	No	No
975	HKL0004	Male	Indian	2018	69	Married	No formal education	Unemployed	Urban	Yes	No	Yes	No	No	No	No	No	No	No

No	Patient ID	Gender	Ethnic group	Year of diagnosis	Age	Marital status	Education level	Occupation	Place of residence	History of psy illness	Tobacco	Alcohol	Cannabis	ATS	Inhalant	Sedative	Hallucinogens	Opioids	Kratom
976	HKL0009	Female	Chinese	2019	55	Married	No formal education	Others	Urban	No	No	No	No	No	No	Yes	No	No	No
977	HKL0010	Male	Indian	2018	64	Married	No formal education	Others	Urban	No	No	Yes	No	No	No	No	No	No	No
978	HKL0012	Female	Indian	2018	62	Married	Secondary	Others	Urban	No	No	No	No	No	No	Yes	No	No	No
979	HKL0017	Male	Malay	2018	58	Married	No formal education	Private	Rural	Yes	Yes	Yes	No	No	No	No	No	No	No
980	HKL0030	Female	Malay	2018	52	Others	No formal education	Unemployed	Urban	Yes	Yes	No	No	Yes	No	No	No	Yes	No
981	HKL0049	Male	Malay	2018	46	Married	No formal education	Unemployed	Urban	Yes	No	No	No	Yes	No	No	No	Yes	Yes
982	HKL0055	Male	Malay	2018	44	Others	Secondary	Unemployed	Urban	Yes	Yes	No	No	No	No	No	No	No	No
983	HKL0056	Male	Malay	2018	44	Single	No formal education	Unemployed	Urban	Yes	No	No	No	Yes	No	No	No	Yes	No
984	HKL0058	Male	Indian	2018	42	Others	No formal education	Others	Urban	Yes	Yes	Yes	No	No	No	No	No	No	No
985	HKL0062	Male	Chinese	2018	47	Single	No formal education	Private	Urban	Yes	Yes	No	No	No	No	No	No	No	No
986	HKL0068	Male	Indian	2018	40	Married	Secondary	Private	Urban	Yes	No	Yes	Yes	No	No	No	No	No	No
987	HKL0081	Male	Indian	2018	37	Others	No formal education	Private	Urban	Yes	Yes	Yes	No	No	No	No	No	No	No
988	HKL0082	Male	Indian	2018	33	Single	No formal education	Unemployed	Urban	Yes	Yes	Yes	Yes	Yes	No	Yes	No	No	No
989	HKL0084	Female	Chinese	2018	37	Others	No formal education	Others	Urban	Yes	Yes	No	No	Yes	No	No	No	No	No
990	HKL0087	Male	Malay	2018	35	Single	Secondary	Private	Urban	Yes	Yes	No	No	Yes	No	No	No	No	No
991	HKL0105	Female	Malay	2018	33	Married	No formal education	Others	Urban	Yes	Yes	No	No	Yes	No	No	No	Yes	No
992	HKL0108	Female	Others	2018	32	Married	Primary	Self-employed	Urban	Yes	Yes	Yes	Yes	Yes	No	No	No	No	No
993	HKL0112	Male	Malay	2018	31	Others	No formal education	Self-employed	Urban	Yes	Yes	Yes	Yes	Yes	Yes	Yes	Yes	Yes	Yes
994	HKL0113	Male	Malay	2018	32	Married	Tertiary	Unemployed	Urban	Yes	Yes	No	No	No	No	No	No	No	No
995	HKL0121	Male	Indian	2018	31	Married	Primary	Unemployed	Urban	Yes	No	No	No	No	No	No	No	Yes	No
996	HKL0144	Male	Malay	2018	27	Married	Secondary	Private	Urban	Yes	Yes	No	No	Yes	No	No	No	No	Yes
997	HKL0154	Male	Malay	2018	26	Single	No formal education	Government	Urban	No	Yes	No	No	Yes	No	No	No	No	No
998	HKL0170	Male	Others	2018	22	Married	Secondary	Government	Urban	No	Yes	Yes	No	Yes	No	No	No	No	No
999	HKL0173	Male	Indian	2018	24	Single	Tertiary	Government	Rural	No	No	No	No	No	No	Yes	No	No	No
1000	HKL0175	Male	Indian	2018	23	Single	Secondary	Private	Rural	Yes	Yes	Yes	No	No	No	No	No	No	No
1001	HKL0176	Male	Malay	2018	20	Single	Secondary	Others	Urban	Yes	No	No	Yes	No	No	No	No	No	No
1002	HKL0186	Male	Malay	2018	35	Single	No formal education	Government	Urban	No	Yes	No	No	Yes	No	No	No	No	No
1003	HKL0187	Male	Malay	2019	19	Single	Secondary	Unemployed	Urban	Yes	No	No	Yes	No	No	No	No	No	No
1004	HKL0188	Female	Malay	2018	18	Single	Secondary	Private	Urban	Yes	Yes	No	No	No	No	No	No	No	No

No	Patient ID	Gender	Ethnic group	Year of diagnosis	Age	Marital status	Education level	Occupation	Place of residence	History of psy illness	Tobacco	Alcohol	Cannabis	ATS	Inhalant	Sedative	Hallucinogens	Opioids	Kratom
1005	HKL0189	Female	Malay	2019	19	Single	Secondary	Unemployed	Urban	No	Yes	No	No	Yes	No	No	No	No	No
1006	HKL0191	Male	Malay	2019	19	Single	Secondary	Unemployed	Urban	Yes	No	No	Yes	Yes	No	Yes	No	Yes	Yes
1007	HKL0192	Male	Indian	2018	18	Single	Secondary	Unemployed	Urban	Yes	No	Yes	Yes	No	No	No	No	No	No
1008	HKL0193	Male	Malay	2018	18	Single	Secondary	Unemployed	Urban	Yes	Yes	Yes	No	Yes	No	No	No	No	No
1009	HKL0194	Male	Malay	2019	18	Single	Secondary	Unemployed	Urban	Yes	Yes	No	No	Yes	No	No	No	No	No
1010	HKL0197	Male	Malay	2019	17	Single	Secondary	Unemployed	Urban	Yes	No	No	Yes	No	No	No	No	No	No
1011	HKL0198	Male	Malay	2019	18	Single	Secondary	Unemployed	Urban	No	Yes	No	No	Yes	No	No	No	No	Yes
1012	HKL0199	Female	Malay	2019	17	Single	Secondary	Unemployed	Rural	No	Yes	No	Yes	Yes	No	No	No	No	No
1013	HKL0203	Male	Malay	2020	25	Single	Primary	Private	Urban	No	Yes	No	No	Yes	No	No	No	Yes	No
1014	HKL0204	Female	Chinese	2018	76	Married	No formal education	Unemployed	Urban	No	No	No	No	No	No	Yes	No	No	No
1015	HKL0209	Female	Malay	2020	34	Married	Secondary	Unemployed	Urban	Yes	Yes	Yes	No	Yes	No	No	No	Yes	No
1016	HKL0211	Male	Malay	2019	17	Single	Secondary	Unemployed	Urban	No	Yes	No	Yes	No	No	No	No	No	No
1017	HKL0214	Male	Indian	2019	65	Single	Tertiary	Unemployed	Urban	No	Yes	No	Yes	Yes	No	No	No	Yes	No
1018	HKL0215	Male	Malay	2019	35	Others	Secondary	Private	Rural	Yes	Yes	No	No	Yes	Yes	No	No	No	No
1019	HKL0217	Male	Malay	2020	22	Single	No formal education	Unemployed	Urban	No	No	No	No	Yes	No	No	No	No	No
1020	HKL0218	Male	Malay	2021	32	Others	Secondary	Government	Rural	Yes	No	No	No	Yes	No	No	No	No	No
1021	HKL0219	Male	Malay	2021	42	Others	Tertiary	Unemployed	Urban	No	No	No	Yes	Yes	No	No	No	No	No
1022	HKL0222	Male	Chinese	2020	58	Married	Tertiary	Unemployed	Urban	Yes	No	No	Yes	Yes	No	No	No	No	No
1023	HKL0224	Male	Chinese	2020	61	Married	No formal education	Unemployed	Urban	No	Yes	Yes	No	No	No	No	No	No	No
1024	HKL0225	Male	Malay	2019	41	Married	Secondary	Government	Rural	No	No	No	No	Yes	No	No	No	No	No
1025	HKL0226	Male	Malay	2021	23	Single	Secondary	Self-employed	Urban	Yes	Yes	No	No	Yes	No	No	No	No	No
1026	HKL0227	Male	Others	2020	26	Others	Secondary	Others	Urban	No	Yes	Yes	No	Yes	No	No	No	No	No
1027	HKL0228	Male	Malay	2020	45	Single	No formal education	Others	Urban	Yes	Yes	No	No	No	No	No	No	No	No
1028	HKL0229	Male	Malay	2020	25	Single	Tertiary	Others	Urban	Yes	Yes	No	Yes	No	No	No	No	No	Yes
1029	HKL0230	Male	Malay	2019	40	Single	No formal education	Unemployed	Urban	Yes	Yes	No	No	No	No	No	No	Yes	No
1030	HKL0231	Male	Malay	2019	35	Single	No formal education	Others	Urban	Yes	Yes	No	No	Yes	No	No	No	No	No
1031	HKL0238	Male	Others	2020	26	Others	Secondary	Private	Urban	Yes	Yes	Yes	No	Yes	No	No	No	No	No
1032	HKL0240	Female	Chinese	2018	34	Others	No formal education	Unemployed	Urban	Yes	Yes	No	No	No	No	No	No	No	No
1033	HKL0241	Female	Others	2018	33	Single	Tertiary	Unemployed	Urban	Yes	Yes	Yes	No	No	No	No	No	No	No
1034	HKL0242	Female	Malay	2019	41	Others	No formal education	Others	Urban	Yes	Yes	No	No	Yes	No	No	No	No	No
1035	HKL0243	Male	Malay	2018	32	Single	Secondary	Unemployed	Urban	Yes	Yes	No	No	No	No	No	No	No	No
1036	HKL0244	Male	Malay	2021	27	Married	Secondary	Unemployed	Urban	Yes	Yes	No	No	No	No	No	No	No	No
1037	HKL0246	Male	Chinese	2020	46	Married	No formal education	Unemployed	Urban	No	No	No	No	Yes	No	No	No	No	No
1038	HKL0247	Male	Indian	2019	44	Married	No formal education	Private	Urban	No	Yes	Yes	No	No	No	No	No	No	No

No	Patient ID	Gender	Ethnic group	Year of diagnosis	Age	Marital status	Education level	Occupation	Place of residence	History of psy illness	Tobacco	Alcohol	Cannabis	ATS	Inhalant	Sedative	Hallucinogens	Opioids	Kratom
1039	HKL0248	Male	Chinese	2020	28	Single	Tertiary	Self-employed	Urban	Yes	No	Yes	No	No	No	No	No	No	No
1040	HKL0252	Male	Malay	2021	23	Others	Secondary	Unemployed	Urban	No	No	No	No	Yes	No	No	No	No	No
1041	HKL0253	Male	Malay	2021	24	Single	Tertiary	Private	Urban	No	No	No	No	Yes	No	No	No	No	No
1042	HKL0254	Male	Malay	2021	46	Others	No formal education	Unemployed	Urban	No	Yes	No	No	No	No	Yes	No	Yes	No
1043	HKL0256	Male	Malay	2018	23	Single	No formal education	Others	Urban	No	No	No	Yes	Yes	No	No	No	No	No
1044	HKL0257	Male	Malay	2020	36	Married	Tertiary	Unemployed	Urban	No	Yes	No	Yes	Yes	No	No	No	No	No
1045	HKL0259	Male	Malay	2019	35	Married	No formal education	Government	Urban	Yes	No	No	No	Yes	No	No	No	No	No
1046	HKL0262	Male	Others	2020	31	Others	Secondary	Unemployed	Urban	Yes	Yes	No	No	Yes	No	No	No	No	No
1047	HKL0267	Male	Malay	2019	65	Married	No formal education	Others	Urban	Yes	No	No	No	No	No	Yes	No	No	No
1048	HKL0268	Male	Malay	2021	24	Others	Secondary	Others	Rural	Yes	Yes	No	Yes	Yes	No	No	No	No	No
1049	HKL0272	Male	Others	2018	65	Single	No formal education	Unemployed	Urban	Yes	No	Yes	No	No	No	No	No	No	No
1050	HKL0273	Male	Chinese	2018	39	Single	No formal education	Unemployed	Urban	Yes	No	No	No	Yes	No	No	No	No	No
1051	HKL0275	Male	Indian	2021	34	Others	Tertiary	Private	Urban	No	Yes	No	No	No	No	No	No	No	No
1052	HKL0276	Male	Malay	2019	37	Married	Secondary	Government	Urban	No	Yes	No	No	Yes	No	No	No	No	Yes
1053	HKL0277	Male	Malay	2019	32	Others	Secondary	Government	Urban	Yes	Yes	No	No	Yes	No	No	No	No	No
1054	HKL0278	Male	Indian	2021	49	Married	No formal education	Self-employed	Urban	No	Yes	Yes	No	Yes	No	No	No	No	No
1055	HKL0279	Female	Malay	2018	22	Single	Tertiary	Private	Urban	Yes	No	Yes	Yes	Yes	No	No	No	No	No
1056	HKL0280	Female	Malay	2018	19	Single	Secondary	Unemployed	Urban	Yes	Yes	No	No	No	No	No	No	No	No
1057	HKL0282	Female	Others	2018	33	Married	No formal education	Others	Urban	No	No	Yes	No	No	No	No	No	No	No
1058	HKL0284	Female	Malay	2021	24	Single	Tertiary	Unemployed	Urban	Yes	No	No	Yes	No	No	No	No	No	No
1059	HKL0285	Female	Malay	2019	24	Single	Tertiary	Private	Urban	Yes	Yes	Yes	No	No	No	No	No	No	No
1060	HKL0286	Female	Malay	2020	32	Single	Tertiary	Unemployed	Urban	No	No	No	No	Yes	No	No	No	No	No
1061	HKL0287	Male	Malay	2018	34	Single	Secondary	Government	Urban	No	Yes	No	No	Yes	No	No	No	No	No
1062	HKL0288	Male	Indian	2018	43	Others	No formal education	Private	Urban	No	Yes	Yes	No	No	No	No	No	No	No
1063	HKL0289	Male	Malay	2020	26	Others	Tertiary	Private	Rural	No	Yes	No	No	No	No	No	No	No	Yes
1064	HKL0290	Male	Malay	2018	25	Single	Tertiary	Unemployed	Urban	Yes	Yes	Yes	Yes	Yes	No	No	No	No	No
1065	HKL0291	Male	Indian	2021	46	Married	No formal education	Self-employed	Urban	Yes	No	Yes	No	Yes	No	No	No	No	No
1066	HKL0292	Male	Chinese	2020	67	Married	Secondary	Others	Urban	Yes	No	Yes	No	No	No	No	No	No	No
1067	HKL0293	Male	Indian	2021	40	Others	Primary	Self-employed	Urban	Yes	Yes	Yes	No	No	No	No	No	No	No
1068	HKL0294	Male	Malay	2021	30	Others	No formal education	Private	Urban	Yes	No	Yes	No	Yes	Yes	No	No	No	No
1069	HKL0295	Male	Chinese	2018	34	Others	Primary	Self-employed	Urban	Yes	No	No	No	Yes	No	No	No	No	No
1070	HKL0296	Male	Malay	2018	30	Married	Secondary	Self-employed	Urban	Yes	No	No	Yes	No	No	No	No	No	No

No	Patient ID	Gender	Ethnic group	Year of diagnosis	Age	Marital status	Education level	Occupation	Place of residence	History of psy illness	Tobacco	Alcohol	Cannabis	ATS	Inhalant	Sedative	Hallucinogens	Opioids	Kratom
1071	HKL0300	Male	Malay	2021	23	Single	Tertiary	Unemployed	Urban	Yes	No	Yes	Yes	No	No	No	No	No	No
1072	HKL0301	Male	Chinese	2021	32	Single	No formal education	Private	Urban	Yes	Yes	No	No	No	No	No	No	No	No
1073	HKL0302	Male	Malay	2019	26	Others	Secondary	Unemployed	Urban	Yes	Yes	No	No	Yes	No	No	No	No	No
1074	HKL0319	Male	Malay	2021	37	Single	No formal education	Unemployed	Rural	Yes	No	No	No	Yes	No	No	No	Yes	No
1075	HKL0320	Male	Malay	2020	46	Single	Secondary	Unemployed	Rural	No	No	No	No	Yes	No	No	No	Yes	Yes
1076	HKL0321	Male	Malay	2020	27	Single	No formal education	Private	Rural	Yes	No	No	No	Yes	No	No	No	No	No
1077	HKL0333	Male	Malay	2021	55	Others	No formal education	Unemployed	Urban	Yes	No	No	No	Yes	No	No	No	No	No
1078	HKL0334	Male	Chinese	2019	42	Others	Secondary	Private	Urban	Yes	No	Yes	Yes	No	No	No	No	No	No
1079	HKL0335	Male	Malay	2018	22	Single	Tertiary	Private	Urban	Yes	No	No	Yes	No	No	No	No	No	No
1080	HKL0338	Male	Malay	2020	26	Others	Tertiary	Private	Urban	Yes	No	No	No	No	No	No	No	No	Yes
1081	HKL0339	Male	Indian	2018	43	Others	No formal education	Private	Rural	Yes	No	Yes	No	No	No	No	No	No	No
1082	HKL0340	Male	Chinese	2020	27	Others	Secondary	Private	Rural	Yes	No	No	Yes	No	No	No	Yes	No	No
1083	HKL0342	Male	Malay	2018	58	Single	Secondary	Unemployed	Urban	No	No	No	No	No	No	No	No	Yes	No
1084	HKL0349	Male	Indian	2018	59	Married	No formal education	Government	Urban	No	No	Yes	Yes	No	No	No	No	No	No
1085	HKL0351	Male	Malay	2018	59	Others	Secondary	Government	Urban	No	Yes	No	No	No	No	No	No	Yes	No
1086	HKL0353	Male	Malay	2019	58	Married	Secondary	Unemployed	Urban	Yes	Yes	No	No	No	No	No	No	No	No
1087	HKL0355	Male	Malay	2020	21	Single	Tertiary	Unemployed	Urban	No	No	No	Yes	No	No	No	No	No	No
1088	HKL0366	Male	Others	2019	21	Single	Tertiary	Unemployed	Urban	Yes	No	No	Yes	No	No	No	No	No	No
1089	HKL0367	Female	Others	2018	30	Others	No formal education	Unemployed	Rural	Yes	Yes	Yes	No	Yes	No	No	No	No	No
1090	HKL0368	Female	Malay	2020	24	Others	Secondary	Unemployed	Urban	Yes	Yes	No	No	Yes	No	No	No	No	No
1091	HKL0370	Female	Indian	2020	28	Others	Secondary	Self-employed	Urban	Yes	Yes	Yes	No	Yes	No	No	No	No	No
1092	HKL0383	Female	Indian	2019	37	Married	No formal education	Others	Urban	No	Yes	No	No	Yes	No	No	No	Yes	No
1093	HKL0386	Male	Others	2021	45	Others	No formal education	Private	Urban	Yes	No	No	No	Yes	No	No	No	No	No
1094	HKL0387	Female	Malay	2020	23	Single	No formal education	Others	Urban	Yes	No	No	No	Yes	No	No	No	No	No
1095	HKL0388	Female	Malay	2018	40	Married	No formal education	Others	Urban	Yes	No	No	No	Yes	No	No	No	No	No
1096	HKL0390	Female	Malay	2018	52	Others	No formal education	Unemployed	Urban	Yes	Yes	No	No	Yes	No	No	No	Yes	No
1097	HKL0391	Female	Indian	2020	58	Others	Primary	Others	Urban	Yes	Yes	No	No	Yes	No	No	No	No	No
1098	HKL0392	Female	Malay	2019	57	Married	No formal education	Others	Urban	Yes	No	No	No	No	No	Yes	No	No	No

No	Patient ID	Gender	Ethnic group	Year of diagnosis	Age	Marital status	Education level	Occupation	Place of residence	History of psy illness	Tobacco	Alcohol	Cannabis	ATS	Inhalant	Sedative	Hallucinogens	Opioids	Kratom
1099	HKL0393	Male	Others	2019	33	Married	No formal education	Private	Urban	Yes	No	No	No	Yes	No	No	No	No	No
1100	HKL0402	Male	Malay	2019	19	Others	Secondary	Private	Urban	Yes	Yes	No	No	No	No	No	No	No	No
1101	HKL0404	Male	Malay	2021	24	Others	Secondary	Private	Urban	Yes	Yes	No	No	No	No	No	No	No	No
1102	HKL0406	Male	Indian	2018	35	Single	Secondary	Private	Urban	Yes	No	No	Yes	Yes	No	Yes	Yes	No	No
1103	HKL0407	Male	Chinese	2020	35	Single	Secondary	Unemployed	Urban	Yes	Yes	No	No	Yes	No	No	No	No	No
1104	HKL0410	Male	Malay	2021	40	Others	Secondary	Others	Urban	Yes	Yes	No	No	Yes	No	No	No	Yes	No
1105	HKL0424	Male	Chinese	2018	59	Married	No formal education	Private	Urban	Yes	No	No	No	No	No	Yes	No	No	No
1106	HKL0427	Male	Malay	2018	36	Single	Tertiary	Unemployed	Rural	Yes	Yes	No	No	Yes	No	No	No	No	No
1107	HKL0428	Male	Malay	2020	32	Others	Tertiary	Unemployed	Urban	Yes	Yes	No	No	Yes	No	No	No	No	No
1108	HKL0430	Male	Malay	2018	26	Single	No formal education	Others	Urban	Yes	Yes	No	Yes	Yes	No	No	No	No	No
1109	HKL0433	Male	Indian	2020	35	Single	No formal education	Unemployed	Urban	Yes	No	Yes	No	No	No	No	No	No	No
1110	HKL0435	Male	Indian	2018	35	Others	No formal education	Others	Urban	Yes	No	Yes	No	No	No	No	No	No	No
1111	HKL0436	Male	Malay	2018	25	Single	Tertiary	Unemployed	Urban	Yes	No	No	Yes	No	No	No	No	No	No
1112	HKL0437	Male	Malay	2020	29	Married	Tertiary	Private	Urban	Yes	No	No	No	Yes	No	No	No	No	No
1113	HKL0446	Male	Malay	2019	36	Married	Tertiary	Private	Urban	Yes	Yes	No	No	Yes	No	No	No	No	No
1114	HKL0448	Male	Indian	2019	59	Others	No formal education	Others	Urban	Yes	No	Yes	No	No	No	No	No	No	No
1115	HKL0450	Male	Malay	2019	43	Single	No formal education	Unemployed	Urban	Yes	No	No	No	Yes	No	No	No	No	No
1116	HKL0452	Male	Indian	2019	59	Married	Secondary	Others	Urban	Yes	No	Yes	No	No	No	No	No	No	No
1117	HKL0453	Male	Malay	2019	29	Single	Secondary	Private	Urban	Yes	No	No	Yes	Yes	No	No	No	No	No
1118	HKL0455	Male	Malay	2019	26	Single	No formal education	Private	Urban	Yes	No	No	No	Yes	No	No	No	No	No
1119	HKL0468	Male	Others	2020	20	Single	Tertiary	Unemployed	Urban	Yes	Yes	No	Yes	No	No	No	No	No	No
1120	HKL0469	Male	Indian	2018	56	Married	No formal education	Others	Urban	Yes	No	Yes	No	No	No	No	No	No	No
1121	HKL0472	Male	Indian	2018	54	Others	No formal education	Others	Urban	Yes	No	Yes	No	No	No	No	No	No	No
1122	HKL0475	Male	Indian	2021	41	Single	Secondary	Private	Urban	Yes	No	Yes	No	No	No	No	No	No	No
1123	HKL0486	Male	Malay	2018	22	Single	Tertiary	Unemployed	Urban	No	No	Yes	Yes	Yes	No	No	No	Yes	No
1124	HKL0487	Male	Indian	2020	48	Single	Secondary	Unemployed	Urban	No	No	No	Yes	Yes	No	No	No	No	No
1125	HKL0488	Male	Malay	2019	24	Single	Tertiary	Self-employed	Urban	No	Yes	No	No	Yes	No	No	No	No	No
1126	HKL0489	Male	Malay	2018	36	Single	No formal education	Self-employed	Urban	No	No	No	No	Yes	No	No	No	No	No
1127	HKL0490	Male	Malay	2020	25	Single	No formal education	Unemployed	Urban	No	Yes	No	No	No	No	No	No	No	Yes
1128	HKL0491	Female	Malay	2019	40	Married	Secondary	Others	Urban	Yes	No	No	No	Yes	No	No	No	No	No
1129	HKL0493	Female	Malay	2020	24	Married	Primary	Unemployed	Urban	Yes	No	No	No	Yes	No	No	No	No	No

No	Patient ID	Gender	Ethnic group	Year of diagnosis	Age	Marital status	Education level	Occupation	Place of residence	History of psy illness	Tobacco	Alcohol	Cannabis	ATS	Inhalant	Sedative	Hallucinogens	Opioids	Kratom
1130	HKL0494	Female	Malay	2021	33	Others	Secondary	Unemployed	Urban	Yes	Yes	No	No	Yes	No	No	No	No	No
1131	HKL0496	Female	Others	2019	24	Single	Secondary	Private	Urban	Yes	No	Yes	No	No	No	No	No	No	No
1132	HKL0497	Female	Indian	2020	29	Others	Secondary	Unemployed	Urban	Yes	Yes	No	No	Yes	No	No	No	Yes	No
1133	HKL0498	Female	Malay	2018	24	Married	Secondary	Private	Urban	Yes	No	No	No	Yes	No	No	No	No	No
1134	HKL0501	Male	Chinese	2018	36	Married	Secondary	Private	Urban	Yes	Yes	No	No	Yes	No	No	No	No	No
1135	HKL0502	Male	Malay	2019	23	Single	Tertiary	Unemployed	Urban	Yes	No	No	No	Yes	No	No	No	No	No
1136	HKL0503	Male	Malay	2019	32	Married	Secondary	Self-employed	Urban	Yes	Yes	No	Yes	Yes	No	No	No	No	No
1137	HKL0505	Male	Malay	2018	30	Others	No formal education	Unemployed	Rural	Yes	No	No	No	Yes	No	No	No	No	No
1138	HKL0507	Male	Malay	2021	25	Others	No formal education	Private	Urban	Yes	No	No	No	No	No	No	No	No	Yes
1139	HKL0509	Male	Indian	2018	41	Others	Tertiary	Unemployed	Urban	Yes	No	Yes	No	No	No	No	No	No	No
1140	HKL0511	Male	Indian	2019	58	Married	No formal education	Unemployed	Urban	No	Yes	Yes	No	No	No	No	No	Yes	No
1141	HKL0513	Male	Indian	2018	24	Single	Secondary	Self-employed	Urban	No	No	Yes	No	No	No	No	No	No	No
1142	HKL0514	Male	Indian	2021	24	Single	Tertiary	Self-employed	Urban	No	Yes	Yes	No	Yes	No	No	No	No	No
1143	HKL0515	Male	Indian	2020	58	Single	No formal education	Private	Urban	Yes	Yes	Yes	No	No	No	No	No	No	No
1144	HKL0516	Male	Malay	2018	27	Single	Tertiary	Government	Urban	No	Yes	Yes	Yes	No	No	No	No	No	No
1145	HKL0518	Male	Malay	2021	32	Single	No formal education	Others	Urban	No	Yes	No	No	Yes	No	No	No	No	No
1146	HKL0521	Male	Indian	2020	30	Single	No formal education	Private	Urban	No	Yes	No	Yes	No	No	No	No	No	No
1147	HKL0522	Female	Malay	2021	34	Single	No formal education	Others	Urban	Yes	No	No	No	Yes	No	No	No	No	No
1148	HKL0525	Male	Malay	2021	23	Single	Tertiary	Unemployed	Urban	No	No	No	Yes	No	No	No	No	No	No
1149	HKL0527	Male	Malay	2020	26	Single	Tertiary	Self-employed	Urban	Yes	No	No	Yes	No	No	No	No	No	No
1150	HKL0528	Male	Indian	2018	27	Others	Secondary	Private	Urban	No	No	Yes	No	No	No	No	No	No	No
1151	HKL0529	Male	Chinese	2018	46	Single	Tertiary	Private	Urban	Yes	Yes	No	No	No	No	No	No	No	No
1152	HKL0530	Male	Indian	2019	38	Single	Secondary	Unemployed	Urban	Yes	Yes	Yes	No	Yes	No	No	No	No	No
1153	HKL0533	Male	Malay	2020	25	Married	Secondary	Unemployed	Urban	Yes	Yes	No	No	Yes	No	No	No	No	No
1154	HKL0534	Male	Malay	2020	21	Single	Secondary	Unemployed	Urban	Yes	No	No	Yes	No	No	No	No	No	No
1155	HKL0535	Male	Malay	2019	37	Married	No formal education	Unemployed	Rural	Yes	No	No	No	Yes	No	No	No	No	No
1156	HKL0536	Male	Malay	2019	28	Single	Tertiary	Unemployed	Urban	Yes	No	No	Yes	Yes	No	Yes	No	Yes	No
1157	HKL0540	Male	Others	2020	25	Single	No formal education	Unemployed	Urban	Yes	No	No	No	Yes	No	No	No	Yes	No
1158	HKL0541	Male	Malay	2020	22	Single	No formal education	Private	Urban	Yes	No	No	Yes	No	No	No	No	No	No
1159	HKL0542	Male	Malay	2019	39	Others	Tertiary	Unemployed	Rural	Yes	Yes	No	No	Yes	No	No	No	No	No
1160	HKL0543	Male	Malay	2020	21	Single	Secondary	Unemployed	Urban	Yes	Yes	No	No	Yes	No	No	No	No	No

No	Patient ID	Gender	Ethnic group	Year of diagnosis	Age	Marital status	Education level	Occupation	Place of residence	History of psy illness	Tobacco	Alcohol	Cannabis	ATS	Inhalant	Sedative	Hallucinogens	Opioids	Kratom
1161	HKL0545	Male	Indian	2018	45	Others	No formal education	Others	Urban	Yes	No	Yes	No	No	No	No	No	No	No
1162	HKL0546	Male	Malay	2021	32	Married	No formal education	Private	Urban	Yes	Yes	No	Yes	No	No	No	No	No	Yes
1163	HKL0548	Male	Malay	2021	25	Others	Primary	Private	Urban	Yes	No	Yes	Yes	Yes	No	Yes	No	No	No
1164	HKL0550	Male	Chinese	2020	46	Single	No formal education	Unemployed	Urban	Yes	No	No	No	Yes	No	No	No	No	No
1165	HKL0552	Male	Malay	2021	42	Others	Secondary	Unemployed	Urban	Yes	Yes	No	No	Yes	No	No	No	No	No
1166	HKL0553	Male	Indian	2021	28	Married	No formal education	Private	Urban	No	Yes	Yes	No	No	No	No	No	No	No
1167	HKL0554	Male	Indian	2018	28	Single	No formal education	Unemployed	Urban	Yes	Yes	No	No	No	No	No	No	No	No
1168	HKL0555	Male	Chinese	2020	65	Married	No formal education	Others	Urban	No	Yes	Yes	No	No	No	No	No	No	No
1169	HKL0556	Male	Chinese	2018	42	Others	No formal education	Unemployed	Urban	No	No	No	No	Yes	No	No	No	No	No
1170	HKL0559	Male	Malay	2021	36	Single	Secondary	Government	Urban	Yes	Yes	No	No	No	No	No	No	No	No
1171	HKL0560	Male	Indian	2021	25	Single	No formal education	Private	Urban	No	No	Yes	No	No	No	No	No	No	No
1172	HKL0561	Male	Malay	2020	57	Married	Secondary	Government	Urban	Yes	No	No	No	Yes	No	No	No	No	No
1173	HKL0562	Male	Malay	2020	38	Married	Tertiary	Unemployed	Urban	Yes	Yes	No	No	No	No	No	No	No	No
1174	HKL0563	Male	Malay	2020	33	Married	No formal education	Private	Urban	No	Yes	No	No	Yes	No	No	No	No	No
1175	HKL0564	Male	Chinese	2019	26	Single	Secondary	Self-employed	Urban	No	Yes	No	No	Yes	No	No	No	No	No
1176	HKL0565	Male	Indian	2021	64	Others	No formal education	Self-employed	Urban	No	No	Yes	No	No	No	No	No	No	No
1177	HKL0566	Male	Malay	2018	26	Single	Tertiary	Government	Urban	No	Yes	No	No	Yes	No	No	No	No	No
1178	HKL0567	Male	Indian	2019	18	Single	No formal education	Unemployed	Urban	Yes	Yes	No	No	No	No	No	No	No	No
1179	HKL0568	Male	Malay	2018	44	Married	No formal education	Self-employed	Urban	No	No	No	No	Yes	No	No	No	No	No
1180	HKL0569	Male	Malay	2018	29	Married	Secondary	Private	Urban	Yes	Yes	No	No	Yes	No	No	No	No	No
1181	HKL0573	Male	Malay	2021	29	Others	Secondary	Unemployed	Urban	Yes	No	No	No	Yes	No	No	No	No	No
1182	HKL0576	Male	Indian	2018	31	Single	No formal education	Unemployed	Urban	Yes	No	No	Yes	Yes	No	No	No	No	No
1183	HKL0584	Male	Malay	2019	19	Others	No formal education	Others	Urban	Yes	No	No	No	No	Yes	No	No	No	No
1184	HKL0588	Female	Indian	2018	37	Married	No formal education	Private	Urban	Yes	Yes	Yes	No	No	No	No	No	No	No
1185	HKL0590	Male	Indian	2018	31	Single	Secondary	Private	Urban	Yes	Yes	Yes	No	No	No	No	No	No	No
1186	HKL0593	Male	Chinese	2018	21	Single	Tertiary	Unemployed	Urban	Yes	Yes	Yes	No	No	No	No	No	No	No
1187	HKL0594	Male	Indian	2021	30	Single	Secondary	Unemployed	Urban	Yes	Yes	Yes	Yes	Yes	No	No	No	No	No
1188	HKL0595	Male	Indian	2018	31	Married	No formal education	Self-employed	Urban	Yes	Yes	Yes	Yes	No	No	No	No	No	No

No	Patient ID	Gender	Ethnic group	Year of diagnosis	Age	Marital status	Education level	Occupation	Place of residence	History of psy illness	Tobacco	Alcohol	Cannabis	ATS	Inhalant	Sedative	Hallucinogens	Opioids	Kratom
1189	HKL0596	Male	Malay	2021	31	Single	No formal education	Unemployed	Urban	Yes	Yes	No	No	Yes	No	No	No	No	No
1190	HKL0597	Male	Chinese	2019	41	Others	No formal education	Self-employed	Urban	Yes	Yes	Yes	No	Yes	No	No	No	No	No
1191	HKL0599	Male	Indian	2018	26	Single	Secondary	Unemployed	Urban	No	Yes	Yes	Yes	Yes	No	No	No	No	No
1192	HKL0600	Male	Indian	2018	30	Single	No formal education	Unemployed	Urban	Yes	Yes	No	No	No	No	No	No	No	No
1193	HKL0601	Male	Malay	2020	21	Single	Secondary	Private	Urban	No	No	No	No	Yes	No	No	No	No	No
1194	HKL0602	Male	Malay	2020	44	Married	No formal education	Unemployed	Urban	Yes	No	No	No	Yes	No	No	No	No	No
1195	HKL0605	Male	Indian	2020	52	Single	Secondary	Unemployed	Urban	Yes	No	Yes	No	No	No	No	No	No	No
1196	HKL0607	Male	Malay	2021	28	Single	No formal education	Private	Urban	No	Yes	No	No	No	No	No	No	No	Yes
1197	HKL0609	Male	Indian	2020	49	Single	Secondary	Private	Urban	No	Yes	Yes	No	No	No	No	No	No	No
1198	HKL0611	Male	Indian	2019	54	Single	No formal education	Private	Urban	Yes	Yes	Yes	No	No	No	No	No	No	No
1199	HKL0613	Male	Malay	2021	23	Single	Secondary	Private	Urban	No	Yes	Yes	Yes	Yes	No	No	No	No	No
1200	HKL0614	Male	Chinese	2018	50	Single	No formal education	Private	Urban	Yes	No	Yes	No	No	No	No	No	No	No
1201	HKL0616	Male	Malay	2021	24	Single	Tertiary	Private	Urban	No	Yes	No	Yes	No	No	No	No	No	No
1202	HKL0618	Male	Indian	2020	44	Single	Secondary	Unemployed	Urban	No	Yes	No	No	Yes	No	No	No	No	No
1203	HKL0619	Male	Malay	2021	28	Single	Primary	Unemployed	Urban	Yes	Yes	No	No	Yes	No	No	No	No	Yes
1204	HKL0622	Male	Malay	2020	54	Single	No formal education	Private	Urban	No	No	Yes	No	Yes	No	No	No	Yes	No
1205	HKL0625	Male	Chinese	2020	64	Single	No formal education	Unemployed	Urban	No	No	No	No	Yes	No	No	No	No	No
1206	HKL0629	Male	Chinese	2019	41	Others	No formal education	Private	Urban	No	Yes	Yes	Yes	Yes	No	No	No	No	No
1207	HKL0630	Male	Chinese	2018	64	Single	No formal education	Others	Urban	Yes	No	Yes	No	No	No	No	No	No	No
1208	HKL0631	Male	Others	2018	24	Married	No formal education	Private	Urban	Yes	Yes	No	No	No	No	No	No	Yes	No
1209	HKL0632	Female	Others	2020	39	Others	No formal education	Others	Urban	Yes	No	No	Yes	Yes	No	No	No	No	No
1210	HKL0634	Female	Chinese	2020	60	Single	No formal education	Others	Urban	Yes	No	Yes	No	No	No	No	No	No	No
1211	HKL0636	Male	Malay	2020	24	Others	No formal education	Others	Urban	Yes	Yes	No	No	No	No	No	No	No	No
1212	HKL0637	Male	Chinese	2018	41	Others	Secondary	Unemployed	Urban	Yes	No	No	No	No	No	Yes	No	No	No
1213	HKL0638	Male	Indian	2020	46	Married	No formal education	Unemployed	Urban	Yes	No	Yes	No	No	No	No	No	No	No
1214	HKL0642	Male	Chinese	2018	70	Married	No formal education	Others	Urban	No	No	Yes	No	No	No	No	No	No	No
1215	HKL0643	Male	Malay	2020	54	Others	Tertiary	Private	Urban	No	Yes	No	No	No	No	No	No	No	No

No	Patient ID	Gender	Ethnic group	Year of diagnosis	Age	Marital status	Education level	Occupation	Place of residence	History of psy illness	Tobacco	Alcohol	Cannabis	ATS	Inhalant	Sedative	Hallucinogens	Opioids	Kratom
1216	HKL0646	Male	Malay	2018	26	Single	Tertiary	Private	Urban	No	Yes	No	Yes	No	No	No	No	No	No
1217	HKL0647	Male	Malay	2019	21	Single	Tertiary	Unemployed	Urban	Yes	Yes	No	No	No	No	No	No	No	No
1218	HKL0649	Male	Malay	2019	29	Married	Secondary	Others	Urban	No	Yes	Yes	No	Yes	No	No	No	No	No
1219	HKL0653	Male	Malay	2020	30	Single	Secondary	Unemployed	Urban	Yes	Yes	No	No	Yes	No	No	No	No	No
1220	HKL0656	Male	Malay	2018	34	Single	No formal education	Government	Urban	No	Yes	No	No	Yes	No	No	No	No	No
1221	HKL0657	Male	Others	2020	54	Single	No formal education	Unemployed	Urban	No	Yes	Yes	No	Yes	No	No	No	Yes	No
1222	HKL0658	Male	Indian	2018	60	Married	No formal education	Self-employed	Urban	No	Yes	Yes	No	Yes	No	No	No	No	No
1223	HKL0659	Male	Others	2020	16	Single	Secondary	Unemployed	Urban	Yes	Yes	No	No	Yes	Yes	No	No	No	No
1224	HKL0660	Male	Malay	2021	19	Single	Secondary	Unemployed	Urban	No	Yes	No	Yes	No	No	No	No	No	No
1225	HKL0661	Male	Indian	2020	45	Single	No formal education	Unemployed	Urban	No	No	Yes	No	No	No	No	No	No	No
1226	HKL0663	Male	Indian	2020	31	Married	Secondary	Unemployed	Urban	No	No	No	Yes	No	No	No	No	No	No
1227	HKL0664	Male	Malay	2020	31	Married	No formal education	Private	Urban	No	No	No	No	Yes	No	No	No	No	No
1228	HKL0666	Male	Indian	2019	52	Married	No formal education	Others	Urban	No	No	Yes	No	No	No	No	No	No	No
1229	HKL0669	Male	Malay	2020	42	Single	Secondary	Unemployed	Urban	No	Yes	Yes	Yes	Yes	No	No	No	No	No
1230	HKL0670	Male	Malay	2019	22	Single	Secondary	Unemployed	Urban	Yes	Yes	Yes	No	No	No	No	No	No	No
1231	HKL0671	Male	Malay	2020	34	Others	Primary	Unemployed	Urban	Yes	Yes	No	No	Yes	No	No	No	No	No
1232	HKL0672	Male	Malay	2021	33	Single	No formal education	Self-employed	Urban	No	Yes	Yes	Yes	Yes	No	No	No	No	Yes
1233	HKL0675	Male	Malay	2018	20	Single	Secondary	Unemployed	Urban	No	Yes	Yes	No	Yes	No	No	No	No	No
1234	HKL0677	Female	Others	2021	31	Others	No formal education	Private	Urban	No	Yes	No	No	Yes	No	No	No	No	No
1235	HKL0678	Male	Others	2020	35	Single	Tertiary	Private	Urban	No	No	No	No	Yes	No	No	No	No	No
1236	HKL0680	Male	Indian	2019	56	Married	No formal education	Unemployed	Urban	No	Yes	Yes	No	No	No	No	No	No	No
1237	HKL0681	Male	Malay	2021	33	Single	Primary	Self-employed	Rural	No	Yes	No	No	Yes	No	No	No	No	No
1238	HKL0682	Male	Malay	2020	33	Single	No formal education	Private	Urban	Yes	No	No	No	No	No	Yes	No	No	No
1239	HKL0683	Male	Malay	2019	38	Others	No formal education	Unemployed	Urban	Yes	Yes	No	No	Yes	No	No	No	No	No
1240	HKL0685	Male	Malay	2019	36	Single	Secondary	Unemployed	Urban	No	Yes	No	No	Yes	No	No	No	Yes	No
1241	HKL0687	Male	Malay	2018	33	Single	Secondary	Unemployed	Rural	No	Yes	No	No	Yes	No	No	No	No	No
1242	HKL0688	Male	Malay	2019	18	Single	Secondary	Unemployed	Urban	Yes	Yes	No	No	No	No	No	No	No	No
1243	HKL0690	Male	Malay	2020	34	Married	Tertiary	Private	Urban	Yes	Yes	No	No	No	No	No	No	No	No
1244	HKL0693	Male	Malay	2019	30	Single	No formal education	Unemployed	Urban	Yes	Yes	No	No	Yes	No	No	No	No	No
1245	HKL0696	Male	Chinese	2018	47	Married	No formal education	Unemployed	Urban	Yes	No	Yes	No	No	No	No	No	No	No
1246	HKL0697	Male	Malay	2018	20	Single	Secondary	Unemployed	Urban	No	Yes	No	No	Yes	No	No	No	No	No

No	Patient ID	Gender	Ethnic group	Year of diagnosis	Age	Marital status	Education level	Occupation	Place of residence	History of psy illness	Tobacco	Alcohol	Cannabis	ATS	Inhalant	Sedative	Hallucinogens	Opioids	Kratom
1247	HKL0698	Male	Malay	2018	22	Others	Secondary	Private	Urban	No	Yes	No	Yes	No	No	No	No	No	Yes
1248	HKL0699	Male	Indian	2019	37	Single	No formal education	Unemployed	Urban	No	No	Yes	No	Yes	No	No	No	No	Yes
1249	HKL0700	Male	Malay	2021	43	Single	Secondary	Self-employed	Urban	Yes	Yes	No	No	Yes	No	No	No	No	No
1250	HKL0702	Male	Malay	2019	24	Single	Secondary	Unemployed	Urban	No	Yes	No	Yes	No	No	No	No	No	Yes
1251	HKL0707	Female	Others	2021	36	Married	No formal education	Unemployed	Rural	Yes	Yes	No	No	Yes	No	No	No	No	No
1252	HKL0708	Male	Malay	2020	28	Single	No formal education	Unemployed	Urban	No	Yes	No	No	Yes	No	No	No	Yes	No
1253	HKL0709	Male	Indian	2020	40	Single	No formal education	Unemployed	Urban	No	No	No	No	Yes	No	No	No	No	No
1254	HKL0710	Male	Malay	2021	21	Single	Secondary	Private	Urban	Yes	Yes	No	Yes	No	No	No	No	No	No
1255	HKL0711	Male	Indian	2019	69	Single	No formal education	Others	Urban	No	Yes	Yes	No	No	No	No	No	No	No
1256	HKL0712	Male	Indian	2021	50	Married	Primary	Private	Urban	Yes	No	Yes	No	No	No	No	No	No	No
1257	HKL0715	Male	Malay	2018	58	Married	Secondary	Unemployed	Urban	Yes	Yes	No	No	No	No	No	No	No	No
1258	HKL0716	Male	Malay	2018	23	Single	Secondary	Others	Urban	Yes	Yes	Yes	Yes	No	No	No	No	No	No
1259	HKL0717	Male	Indian	2021	39	Others	No formal education	Private	Urban	Yes	Yes	Yes	No	No	No	No	No	No	No
1260	HKL0719	Male	Indian	2018	43	Single	No formal education	Unemployed	Urban	No	Yes	Yes	No	No	No	No	No	No	No
1261	HKL0720	Male	Malay	2021	37	Married	No formal education	Government	Urban	No	Yes	No	No	No	No	No	No	No	No
1262	HKL0721	Male	Malay	2019	25	Single	Secondary	Unemployed	Urban	No	Yes	Yes	No	No	No	No	No	No	No
1263	HKL0723	Male	Indian	2020	40	Single	No formal education	Unemployed	Urban	Yes	No	Yes	No	No	No	No	No	No	No
1264	HKL0725	Male	Malay	2021	33	Married	Tertiary	Government	Urban	No	Yes	No	No	No	No	No	No	No	No
1265	HKL0726	Male	Malay	2020	42	Others	No formal education	Government	Urban	Yes	Yes	No	Yes	Yes	No	No	No	No	No
1266	HKL0727	Male	Chinese	2018	54	Others	Tertiary	Unemployed	Urban	Yes	Yes	No	No	No	No	No	No	No	No
1267	HKL0728	Male	Indian	2021	25	Others	Secondary	Unemployed	Urban	Yes	Yes	Yes	No	No	No	No	No	No	No
1268	HKL0729	Male	Malay	2018	50	Single	No formal education	Private	Urban	No	No	Yes	No	Yes	No	No	No	No	No
1269	HKL0731	Male	Indian	2021	39	Single	No formal education	Private	Urban	Yes	No	No	No	No	No	No	No	Yes	No
1270	HKL0732	Male	Malay	2020	33	Others	Tertiary	Private	Urban	Yes	No	No	No	Yes	No	No	No	No	No
1271	HKL0736	Male	Malay	2020	37	Others	No formal education	Self-employed	Urban	Yes	No	No	No	Yes	No	No	No	No	No
1272	HKL0742	Male	Malay	2019	37	Single	No formal education	Private	Urban	Yes	No	No	No	Yes	No	No	No	Yes	No
1273	HKL0746	Male	Malay	2020	43	Married	No formal education	Private	Urban	No	Yes	No	Yes	Yes	No	No	No	No	No
1274	HKL0747	Male	Chinese	2018	57	Married	Primary	Self-employed	Urban	No	Yes	Yes	No	No	No	No	No	No	No
1275	HKL0748	Male	Malay	2020	37	Married	Tertiary	Unemployed	Urban	Yes	Yes	No	No	No	No	No	No	No	No

No	Patient ID	Gender	Ethnic group	Year of diagnosis	Age	Marital status	Education level	Occupation	Place of residence	History of psy illness	Tobacco	Alcohol	Cannabis	ATS	Inhalant	Sedative	Hallucinogens	Opioids	Kratom
1276	HKL0750	Male	Malay	2020	36	Single	Primary	Others	Urban	Yes	Yes	No	No	No	No	No	No	No	No
1277	HKL0751	Male	Malay	2021	24	Single	Tertiary	Private	Urban	Yes	Yes	No	No	No	No	No	No	No	No
1278	HKL0755	Male	Indian	2020	69	Others	No formal education	Government	Urban	No	Yes	Yes	No	No	No	No	No	No	No
1279	HKL0759	Male	Malay	2020	21	Single	Secondary	Unemployed	Urban	No	Yes	No	No	No	No	No	No	No	No
1280	HKL0761	Female	Malay	2021	56	Others	No formal education	Others	Urban	No	No	No	No	Yes	No	No	No	Yes	No
1281	HKL0762	Male	Malay	2018	17	Single	Secondary	Unemployed	Urban	No	Yes	No	No	No	No	No	No	No	No
1282	HKL0763	Male	Indian	2020	18	Single	Secondary	Unemployed	Urban	Yes	Yes	Yes	No	No	No	No	No	No	No
1283	HKL0764	Male	Malay	2019	19	Single	Secondary	Unemployed	Urban	Yes	Yes	Yes	No	No	No	No	No	No	No
1284	HKL0766	Male	Malay	2018	57	Others	Primary	Others	Urban	Yes	Yes	No	No	No	No	No	No	No	No
1285	HKL0768	Male	Malay	2020	24	Married	No formal education	Self-employed	Urban	No	No	No	No	Yes	No	No	No	No	No
1286	HKL0769	Male	Indian	2021	25	Single	Tertiary	Private	Urban	Yes	No	Yes	No	No	No	No	No	No	No
1287	HKL0770	Male	Malay	2019	40	Married	Tertiary	Self-employed	Urban	Yes	Yes	No	Yes	Yes	No	No	No	No	No
1288	HKL0771	Male	Indian	2020	28	Married	Secondary	Unemployed	Urban	No	Yes	Yes	No	Yes	No	No	No	No	No
1289	HKL0774	Male	Malay	2020	25	Single	Secondary	Private	Urban	No	No	No	No	Yes	No	No	No	No	No
1290	HKL0776	Male	Malay	2020	30	Single	Secondary	Private	Urban	No	Yes	Yes	No	Yes	No	No	No	No	No
1291	HKL0777	Male	Malay	2021	22	Single	Tertiary	Unemployed	Urban	No	Yes	No	No	No	No	No	No	No	No
1292	HKL0778	Male	Malay	2018	27	Single	Secondary	Others	Urban	No	No	No	No	Yes	No	No	No	No	Yes
1293	HKL0779	Male	Malay	2020	61	Others	Secondary	Others	Urban	Yes	Yes	No	No	No	No	No	No	Yes	No
1294	HKL0781	Male	Malay	2018	28	Single	Secondary	Unemployed	Urban	Yes	Yes	No	No	No	Yes	No	No	No	No
1295	HKL0783	Male	Malay	2021	20	Single	Secondary	Private	Urban	Yes	Yes	No	No	No	No	No	No	No	Yes
1296	HKL0784	Male	Malay	2019	19	Single	Secondary	Unemployed	Urban	Yes	Yes	No	No	No	No	No	No	No	No
1297	HKL0786	Male	Others	2018	19	Single	No formal education	Others	Urban	Yes	Yes	No	Yes	No	No	No	No	No	No
1298	HKL0794	Male	Malay	2019	35	Others	Tertiary	Government	Urban	Yes	Yes	No	No	No	No	No	No	No	No
1299	HKL0795	Male	Malay	2020	41	Single	No formal education	Unemployed	Urban	No	Yes	No	No	Yes	No	No	No	Yes	No
1300	HKL0797	Male	Malay	2020	27	Single	Primary	Self-employed	Rural	Yes	Yes	No	Yes	No	No	No	No	No	No
1301	HKL0801	Female	Indian	2021	56	Single	No formal education	Others	Urban	No	Yes	Yes	No	No	No	No	No	No	No
1302	HKL0806	Male	Others	2018	30	Single	No formal education	Private	Urban	No	No	No	No	Yes	No	No	No	No	No
1303	HKL0807	Female	Indian	2020	44	Married	No formal education	Unemployed	Rural	Yes	Yes	Yes	No	No	No	No	No	No	No
1304	HKL0811	Male	Malay	2021	40	Single	Secondary	Self-employed	Urban	No	No	No	No	Yes	No	No	No	No	No
1305	HKL0816	Male	Malay	2018	36	Single	Secondary	Unemployed	Urban	No	Yes	No	No	No	No	No	No	No	No
1306	HKL0817	Male	Malay	2021	29	Married	Secondary	Government	Urban	Yes	Yes	No	No	No	No	No	No	No	Yes
1307	HKL0820	Male	Malay	2019	22	Single	Tertiary	Unemployed	Urban	No	Yes	No	No	Yes	No	No	No	No	Yes
1308	HKL0829	Male	Malay	2020	31	Married	Secondary	Unemployed	Urban	No	No	No	Yes	Yes	No	No	No	No	No
1309	HKL0835	Male	Malay	2021	30	Married	No formal education	Private	Urban	No	No	No	No	Yes	No	No	No	No	Yes

No	Patient ID	Gender	Ethnic group	Year of diagnosis	Age	Marital status	Education level	Occupation	Place of residence	History of psy illness	Tobacco	Alcohol	Cannabis	ATS	Inhalant	Sedative	Hallucinogens	Opioids	Kratom
1310	HKL0840	Male	Chinese	2020	55	Married	No formal education	Unemployed	Urban	No	Yes	No	No	No	No	No	No	Yes	No
1311	HKL0849	Male	Malay	2020	55	Married	Secondary	Unemployed	Rural	No	Yes	No	No	Yes	No	No	No	Yes	No
1312	HKL0853	Male	Malay	2021	58	Single	No formal education	Unemployed	Rural	No	No	No	No	Yes	No	No	No	Yes	No
1313	HKL0854	Male	Others	2019	45	Single	Secondary	Private	Urban	Yes	Yes	No	No	No	No	No	No	No	No
1314	HKL0855	Male	Indian	2020	18	Single	Secondary	Unemployed	Urban	No	Yes	Yes	No	No	No	No	No	No	No
1315	HKL0859	Male	Malay	2018	39	Single	No formal education	Others	Urban	Yes	No	No	No	No	No	No	No	Yes	No
1316	HKL0861	Male	Malay	2021	29	Others	No formal education	Unemployed	Urban	No	Yes	No	No	Yes	No	No	No	No	No
1317	HKL0865	Male	Malay	2020	21	Single	Secondary	Private	Rural	No	Yes	Yes	No	Yes	No	No	No	No	No
1318	HKL0874	Male	Indian	2019	42	Single	Secondary	Private	Urban	No	No	Yes	No	No	No	No	No	No	No
1319	HKL0877	Male	Malay	2019	37	Married	Secondary	Government	Urban	No	Yes	No	No	No	No	No	No	No	No
1320	HKL0879	Male	Indian	2018	44	Married	No formal education	Self-employed	Urban	No	Yes	Yes	No	No	No	No	No	No	No
1321	HKL0881	Male	Malay	2021	23	Others	Tertiary	Government	Urban	Yes	Yes	Yes	Yes	Yes	No	No	No	No	No
1322	HKL0882	Male	Malay	2019	28	Single	Tertiary	Private	Urban	Yes	No	No	Yes	Yes	No	No	No	No	No
1323	HKL0884	Male	Indian	2019	56	Married	No formal education	Unemployed	Urban	No	Yes	Yes	No	No	No	No	No	No	No
1324	HKL0886	Male	Others	2021	53	Married	No formal education	Self-employed	Urban	Yes	No	Yes	No	No	No	No	No	No	No
1325	HKL0891	Male	Indian	2020	29	Single	Tertiary	Others	Urban	No	Yes	Yes	No	Yes	No	No	No	No	No
1326	HKL0896	Male	Indian	2021	35	Single	No formal education	Unemployed	Urban	No	Yes	Yes	No	No	No	No	No	No	No
1327	HKL0900	Male	Malay	2019	24	Single	Secondary	Unemployed	Urban	No	Yes	No	No	No	No	No	No	Yes	No
1328	HKL0903	Male	Malay	2019	26	Single	Tertiary	Unemployed	Rural	Yes	Yes	No	Yes	No	No	No	No	No	No
1329	HKL0904	Male	Indian	2019	56	Married	Secondary	Self-employed	Urban	No	Yes	Yes	No	No	No	No	No	No	No
1330	HKL0907	Male	Malay	2021	59	Others	No formal education	Self-employed	Urban	No	Yes	No	No	Yes	No	No	No	No	No
1331	HKL0914	Male	Chinese	2021	31	Single	No formal education	Others	Urban	Yes	No	Yes	No	No	No	No	No	No	No
1332	HKL0915	Male	Malay	2021	18	Single	Secondary	Unemployed	Urban	Yes	Yes	No	No	No	No	No	No	No	No
1333	HKL0916	Male	Malay	2020	38	Others	No formal education	Self-employed	Urban	No	Yes	No	Yes	Yes	No	No	No	No	No
1334	HKL0919	Male	Chinese	2018	46	Single	No formal education	Unemployed	Urban	No	No	No	No	Yes	No	No	No	No	No
1335	HKL0922	Male	Chinese	2021	53	Married	Tertiary	Government	Urban	Yes	Yes	Yes	No	No	No	No	No	No	No
1336	HKL0933	Male	Others	2021	46	Single	No formal education	Others	Urban	Yes	No	No	No	Yes	No	No	No	Yes	No
1337	HKL0934	Male	Malay	2021	19	Others	Tertiary	Unemployed	Urban	Yes	Yes	No	No	No	No	No	No	No	No
1338	HKL0936	Male	Indian	2018	49	Married	No formal education	Private	Urban	No	No	Yes	Yes	Yes	No	No	No	No	No
1339	HKL0940	Male	Malay	2021	38	Single	Secondary	Unemployed	Urban	No	Yes	No	No	No	No	No	No	No	No

No	Patient ID	Gender	Ethnic group	Year of diagnosis	Age	Marital status	Education level	Occupation	Place of residence	History of psy illness	Tobacco	Alcohol	Cannabis	ATS	Inhalant	Sedative	Hallucinogens	Opioids	Kratom
1340	HKL0945	Male	Indian	2020	42	Others	Secondary	Unemployed	Urban	No	No	Yes	No	No	No	No	No	No	No
1341	HKL0958	Male	Malay	2018	46	Married	No formal education	Unemployed	Urban	No	Yes	No	No	Yes	No	No	No	No	No
1342	HKL0959	Male	Indian	2021	54	Married	No formal education	Self-employed	Urban	Yes	Yes	No	Yes	No	No	No	No	No	Yes
1343	HKL0960	Male	Indian	2021	34	Married	No formal education	Private	Urban	No	Yes	Yes	No	No	No	No	No	No	No
1344	HKL0961	Male	Malay	2021	38	Others	No formal education	Self-employed	Urban	No	Yes	No	Yes	No	No	No	No	No	No
1345	HKL0962	Male	Others	2019	36	Others	Secondary	Private	Urban	Yes	Yes	Yes	No	Yes	No	No	No	No	No
1346	HKL0964	Male	Chinese	2021	67	Married	No formal education	Private	Urban	Yes	No	No	No	Yes	No	No	No	No	No
1347	HKL0965	Female	Malay	2020	67	Married	No formal education	Unemployed	Urban	Yes	Yes	No	No	No	No	No	No	No	No
1348	HKL0969	Female	Chinese	2021	32	Married	No formal education	Private	Urban	No	Yes	No	No	Yes	No	No	No	No	No
1349	HKL0970	Female	Malay	2019	19	Single	Secondary	Unemployed	Urban	No	Yes	No	No	Yes	No	No	No	No	No
1350	HKL0977	Male	Malay	2019	22	Single	Tertiary	Unemployed	Urban	Yes	Yes	No	No	No	No	No	No	No	No
1351	HKL0980	Male	Malay	2019	49	Single	Secondary	Unemployed	Urban	No	No	No	No	Yes	No	No	No	Yes	No
1352	HKL0981	Male	Malay	2019	30	Married	Tertiary	Private	Urban	Yes	No	Yes	Yes	No	No	No	No	No	No
1353	HKL0982	Male	Indian	2021	19	Single	Secondary	Unemployed	Urban	Yes	Yes	Yes	Yes	No	Yes	No	No	No	No
1354	HKL0983	Female	Others	2020	29	Others	No formal education	Unemployed	Urban	No	No	No	No	Yes	No	No	No	No	No
1355	HKL0986	Male	Others	2021	35	Others	No formal education	Unemployed	Urban	No	Yes	Yes	Yes	Yes	No	No	No	No	No
1356	HKL0988	Male	Malay	2018	28	Single	No formal education	Unemployed	Rural	Yes	Yes	No	No	No	No	No	No	No	Yes
1357	HKL0995	Male	Malay	2020	39	Others	No formal education	Others	Urban	No	Yes	No	Yes	No	No	No	No	No	Yes
1358	HKL1003	Male	Malay	2020	21	Single	Tertiary	Unemployed	Rural	Yes	No	No	No	Yes	No	No	No	No	No
1359	HKL1004	Male	Indian	2020	39	Married	Secondary	Private	Urban	No	Yes	Yes	No	No	No	No	No	No	No
1360	HKL1017	Male	Indian	2019	41	Others	No formal education	Unemployed	Urban	Yes	Yes	Yes	No	No	No	No	No	No	No
1361	HKL1018	Male	Malay	2018	49	Others	Secondary	Unemployed	Urban	Yes	Yes	No	Yes	Yes	No	No	No	No	No
1362	HKL1023	Male	Malay	2018	34	Single	No formal education	Unemployed	Urban	Yes	Yes	Yes	Yes	Yes	Yes	No	No	No	No
1363	HKL1024	Male	Indian	2021	29	Single	No formal education	Unemployed	Urban	Yes	Yes	Yes	Yes	No	No	No	No	No	No
1364	HKL1025	Male	Indian	2019	33	Married	No formal education	Private	Urban	No	No	No	No	Yes	No	No	No	No	No
1365	HKL1026	Male	Indian	2019	56	Single	No formal education	Unemployed	Urban	No	Yes	Yes	No	No	No	No	No	No	No
1366	HKL1027	Male	Malay	2021	37	Married	Secondary	Government	Rural	No	Yes	No	No	Yes	No	No	No	No	No

No	Patient ID	Gender	Ethnic group	Year of diagnosis	Age	Marital status	Education level	Occupation	Place of residence	History of psy illness	Tobacco	Alcohol	Cannabis	ATS	Inhalant	Sedative	Hallucinogens	Opioids	Kratom
1367	HKL1028	Male	Malay	2020	35	Married	No formal education	Private	Rural	Yes	Yes	No	No	Yes	No	No	No	No	No
1368	HKL1030	Male	Malay	2020	31	Single	Tertiary	Unemployed	Urban	No	Yes	No	No	Yes	No	No	No	No	No
1369	HKL1031	Male	Indian	2020	58	Married	Secondary	Unemployed	Urban	No	Yes	Yes	No	No	No	No	No	No	No
1370	HKL1032	Female	Others	2021	43	Single	No formal education	Private	Urban	Yes	Yes	No	No	No	No	No	No	No	No
1371	HKL1033	Male	Indian	2020	47	Married	No formal education	Self-employed	Urban	No	Yes	No	Yes	Yes	No	No	No	No	No
1372	HKL1034	Male	Malay	2018	37	Married	No formal education	Self-employed	Urban	No	Yes	Yes	No	No	No	No	No	No	No
1373	HKL1035	Male	Malay	2019	37	Single	No formal education	Private	Urban	Yes	Yes	No	No	Yes	No	No	No	No	No
1374	HKL1037	Male	Chinese	2020	32	Single	Secondary	Others	Urban	No	No	No	No	Yes	No	No	No	No	No
1375	HKL1039	Male	Indian	2019	53	Single	Secondary	Unemployed	Urban	No	No	No	No	Yes	No	No	No	Yes	No
1376	HKL1042	Male	Malay	2020	23	Single	Tertiary	Unemployed	Urban	No	Yes	Yes	Yes	Yes	No	No	No	No	No
1377	HKL1043	Male	Malay	2018	23	Single	Tertiary	Private	Urban	No	Yes	No	Yes	Yes	No	No	No	No	No
1378	HKL1045	Male	Indian	2021	30	Married	No formal education	Private	Urban	No	Yes	No	Yes	Yes	No	No	No	No	No
1379	HKL1047	Male	Chinese	2019	59	Married	No formal education	Others	Urban	Yes	No	Yes	No	No	No	No	No	No	No
1380	HKL1052	Male	Malay	2021	23	Single	Tertiary	Unemployed	Urban	Yes	Yes	Yes	Yes	No	No	No	No	No	No
1381	HKL1053	Male	Malay	2018	23	Single	No formal education	Self-employed	Urban	No	No	Yes	No	Yes	No	No	No	No	Yes
1382	HKL1055	Male	Malay	2018	41	Single	No formal education	Self-employed	Urban	Yes	Yes	Yes	No	No	No	No	No	No	No
1383	HKL1057	Male	Malay	2018	24	Single	Secondary	Unemployed	Urban	No	Yes	No	Yes	No	No	No	No	No	Yes
1384	HKL1063	Male	Malay	2019	22	Single	Tertiary	Private	Rural	No	Yes	No	Yes	No	No	No	No	No	No
1385	HKL1066	Male	Malay	2020	56	Others	No formal education	Unemployed	Urban	No	Yes	No	No	No	No	No	No	Yes	No
1386	HKL1067	Male	Malay	2021	39	Married	Primary	Unemployed	Urban	Yes	Yes	No	Yes	No	No	No	No	No	No
1387	HKL1068	Male	Malay	2021	19	Single	Secondary	Self-employed	Rural	Yes	Yes	No	No	No	No	No	No	No	No
1388	HKL1069	Male	Malay	2019	30	Others	No formal education	Unemployed	Urban	Yes	Yes	No	Yes	Yes	No	No	No	Yes	No
1389	HKL1071	Male	Malay	2019	23	Single	Tertiary	Unemployed	Rural	Yes	No	No	Yes	No	No	No	No	No	Yes
1390	HKL1073	Female	Indian	2019	39	Married	No formal education	Unemployed	Urban	No	No	Yes	No	Yes	No	No	No	No	No
1391	HKL1076	Male	Malay	2019	40	Single	No formal education	Unemployed	Rural	No	No	Yes	No	Yes	No	No	No	No	No
1392	HKL1077	Male	Malay	2020	48	Others	Primary	Unemployed	Urban	Yes	Yes	No	Yes	Yes	No	No	No	No	No
1393	HKL1078	Male	Malay	2019	21	Single	Secondary	Unemployed	Urban	No	No	No	Yes	No	No	No	No	No	Yes
1394	HKL1079	Male	Malay	2020	39	Others	No formal education	Unemployed	Urban	Yes	Yes	No	No	Yes	No	No	No	Yes	No
1395	HKL1080	Male	Malay	2020	27	Single	Secondary	Unemployed	Urban	No	Yes	No	No	Yes	No	No	No	No	No

No	Patient ID	Gender	Ethnic group	Year of diagnosis	Age	Marital status	Education level	Occupation	Place of residence	History of psy illness	Tobacco	Alcohol	Cannabis	ATS	Inhalant	Sedative	Hallucinogens	Opioids	Kratom
1396	HKL1084	Male	Malay	2021	67	Single	Secondary	Unemployed	Urban	No	Yes	No	Yes	Yes	No	No	No	Yes	No
1397	HKL1088	Male	Others	2021	31	Married	No formal education	Government	Urban	Yes	No	Yes	No	No	No	No	No	No	No
1398	HKL1094	Male	Indian	2019	48	Married	No formal education	Unemployed	Urban	No	Yes	Yes	No	No	No	No	No	No	No
1399	HKL1095	Male	Indian	2021	23	Others	Secondary	Unemployed	Urban	No	Yes	Yes	Yes	No	No	No	No	No	No
1400	HKL1096	Male	Malay	2018	41	Single	No formal education	Private	Urban	No	Yes	No	No	Yes	No	No	No	Yes	No
1401	HKL1097	Male	Indian	2020	22	Single	Secondary	Unemployed	Urban	Yes	Yes	Yes	No	No	No	No	No	No	No
1402	HKL1098	Male	Chinese	2021	44	Others	Secondary	Private	Urban	Yes	Yes	No	No	Yes	No	No	No	No	No
1403	HKL1100	Male	Indian	2020	30	Single	Tertiary	Private	Urban	No	No	No	Yes	No	No	No	No	No	No
1404	HKL1101	Male	Indian	2021	58	Married	Tertiary	Self-employed	Urban	No	No	Yes	No	No	No	No	No	No	No
1405	HKL1104	Male	Malay	2020	30	Single	Tertiary	Government	Urban	Yes	Yes	No	No	Yes	No	No	No	No	No
1406	HKL1105	Male	Malay	2020	45	Married	Primary	Unemployed	Urban	No	No	Yes	No	No	No	No	No	No	No
1407	HKL1107	Male	Indian	2018	81	Married	No formal education	Others	Urban	No	No	Yes	No	No	No	No	No	No	No
1408	HKL1108	Male	Indian	2018	76	Others	No formal education	Others	Urban	No	No	Yes	No	No	No	No	No	No	No
1409	HKL1109	Male	Chinese	2018	73	Married	No formal education	Others	Urban	No	Yes	Yes	No	No	No	Yes	No	No	No
1410	HKL1115	Male	Malay	2021	31	Others	No formal education	Unemployed	Urban	Yes	No	No	No	Yes	No	No	No	No	No
1411	HKL1116	Female	Indian	2021	46	Married	No formal education	Unemployed	Urban	Yes	No	Yes	No	No	No	No	No	No	No
1412	HKL1117	Male	Indian	2018	57	Married	No formal education	Private	Rural	Yes	Yes	Yes	No	No	No	No	No	No	No
1413	HKL1118	Male	Malay	2020	25	Single	Tertiary	Unemployed	Urban	Yes	Yes	No	Yes	No	No	No	No	No	Yes
1414	HKL1121	Male	Indian	2020	42	Single	No formal education	Unemployed	Urban	No	No	No	No	Yes	No	No	No	Yes	No
1415	HKL1122	Male	Indian	2020	61	Single	Secondary	Unemployed	Urban	No	No	Yes	No	No	No	No	No	No	No
1416	HKL1123	Male	Malay	2020	55	Others	Primary	Private	Urban	No	No	No	No	Yes	No	No	No	Yes	No
1417	HKL1135	Male	Malay	2019	45	Married	Secondary	Government	Rural	No	Yes	No	No	No	No	No	No	Yes	No
1418	HM0001	Male	Malay	2020	18	Single	Secondary	Others	Rural	Yes	Yes	No	No	No	No	No	No	No	No
1419	HM0002	Male	Malay	2021	19	Single	Tertiary	Private	Rural	Yes	Yes	No	No	No	No	No	No	No	No
1420	HM0003	Female	Malay	2020	20	Single	Tertiary	Unemployed	Rural	No	Yes	Yes	No	Yes	No	No	No	No	No
1421	HM0004	Male	Malay	2020	19	Single	Tertiary	Unemployed	Rural	Yes	Yes	No	No	No	No	No	No	No	No
1422	HM0005	Female	Malay	2018	15	Single	Secondary	Unemployed	Rural	No	Yes	No	No	No	No	No	No	No	No
1423	HM0006	Male	Malay	2021	21	Single	Secondary	Unemployed	Rural	Yes	Yes	No	No	No	No	No	No	No	No
1424	HM0008	Male	Malay	2020	18	Single	Secondary	Unemployed	Rural	Yes	Yes	Yes	Yes	Yes	Yes	Yes	No	Yes	No
1425	HM0009	Male	Malay	2019	19	Single	Secondary	Unemployed	Rural	No	Yes	No	No	No	No	No	No	No	No
1426	HM0010	Female	Malay	2020	20	Single	Tertiary	Unemployed	Rural	No	No	No	Yes	No	No	No	No	No	No
1427	HM0011	Male	Malay	2020	19	Single	Secondary	Unemployed	Rural	No	No	Yes	No	No	No	No	No	No	No
1428	HM0012	Male	Malay	2021	20	Single	Secondary	Unemployed	Rural	No	Yes	No	No	Yes	No	No	No	No	No

No	Patient ID	Gender	Ethnic group	Year of diagnosis	Age	Marital status	Education level	Occupation	Place of residence	History of psy illness	Tobacco	Alcohol	Cannabis	ATS	Inhalant	Sedative	Hallucinogens	Opioids	Kratom
1429	HM0014	Male	Malay	2021	20	Single	Tertiary	Unemployed	Rural	No	Yes	No	No	No	No	No	No	No	No
1430	HM0015	Male	Malay	2021	20	Single	Tertiary	Unemployed	Rural	No	Yes	Yes	Yes	Yes	No	No	No	No	No
1431	HM0016	Female	Malay	2020	16	Single	Secondary	Unemployed	Rural	No	Yes	No	No	No	No	No	No	No	No
1432	HM0017	Male	Malay	2018	17	Single	Secondary	Unemployed	Urban	No	Yes	No	No	No	No	No	No	No	No
1433	HM0019	Male	Malay	2019	18	Single	Secondary	Private	Rural	No	Yes	No	No	No	No	No	No	No	No
1434	HM0020	Female	Malay	2021	20	Single	Tertiary	Unemployed	Rural	No	Yes	No	No	No	No	No	No	No	No
1435	HM0021	Female	Malay	2018	15	Single	Secondary	Others	Rural	No	Yes	Yes	No	Yes	No	Yes	No	No	No
1436	HM0022	Female	Malay	2018	18	Single	Secondary	Unemployed	Rural	No	Yes	No	Yes	Yes	No	No	No	No	No
1437	HM0023	Male	Malay	2018	18	Single	Secondary	Unemployed	Rural	No	Yes	No	No	No	Yes	No	No	No	No
1438	HM0024	Male	Indian	2020	55	Single	Primary	Unemployed	Urban	Yes	Yes	No	No	No	No	No	No	No	No
1439	HM0025	Male	Malay	2020	19	Single	Secondary	Unemployed	Rural	No	No	Yes	No	Yes	No	No	No	No	No
1440	HM0026	Male	Others	2018	25	Single	Tertiary	Others	Urban	Yes	Yes	Yes	No	No	No	No	No	No	No
1441	HM0027	Male	Malay	2019	16	Single	Secondary	Private	Rural	No	Yes	No	No	No	No	No	No	No	No
1442	HM0028	Female	Others	2018	18	Single	Tertiary	Unemployed	Urban	No	No	Yes	No	No	No	No	No	No	No
1443	HM0029	Female	Malay	2021	21	Single	No formal education	Private	Rural	No	Yes	No	No	No	No	No	No	No	No
1444	HM0030	Female	Malay	2020	16	Single	Secondary	Unemployed	Rural	No	Yes	No	No	No	No	No	No	No	No
1445	HM0031	Male	Chinese	2020	20	Single	Secondary	Private	Urban	No	No	Yes	No	No	No	No	No	No	No
1446	HM0032	Female	Malay	2021	20	Single	Secondary	Unemployed	Rural	No	Yes	No	Yes	No	No	No	No	No	No
1447	HM0033	Male	Malay	2019	19	Single	Secondary	Private	Rural	No	Yes	No	No	No	No	No	No	No	No
1448	HM0034	Male	Malay	2020	20	Single	Tertiary	Private	Rural	No	Yes	Yes	Yes	Yes	No	No	No	No	No
1449	HM0035	Female	Malay	2020	20	Single	Secondary	Private	Rural	No	Yes	No	No	No	No	No	No	No	No
1450	HM0036	Male	Malay	2019	19	Single	Secondary	Self-employed	Rural	No	Yes	No	No	No	No	No	No	No	No
1451	HM0037	Male	Malay	2021	17	Single	Secondary	Private	Urban	No	Yes	No	Yes	No	No	No	No	No	No
1452	HM0038	Female	Malay	2019	18	Single	Primary	Self-employed	Rural	No	Yes	No	No	No	No	No	No	No	No
1453	HM0039	Male	Malay	2018	18	Single	Secondary	Others	Rural	No	Yes	No	No	No	No	No	No	No	No
1454	HM0040	Female	Malay	2021	20	Single	Secondary	Private	Rural	No	No	No	No	No	No	Yes	No	No	No
1455	HM0041	Female	Malay	2021	21	Single	Secondary	Private	Rural	No	Yes	No	No	No	No	Yes	No	No	No
1456	HM0042	Male	Indian	2020	16	Single	Secondary	Unemployed	Rural	No	Yes	No	No	No	No	No	No	No	No
1457	HM0043	Female	Indian	2019	16	Single	Secondary	Unemployed	Rural	No	Yes	Yes	No	No	No	No	No	No	No
1458	HM0044	Female	Indian	2020	18	Single	Secondary	Unemployed	Rural	No	Yes	Yes	No	No	No	No	No	No	No
1459	HM0045	Male	Malay	2018	17	Single	Tertiary	Unemployed	Rural	No	Yes	No	No	No	No	No	No	No	Yes
1460	HM0046	Female	Malay	2020	17	Single	Secondary	Private	Rural	No	No	No	No	Yes	No	Yes	No	No	No
1461	HM0047	Male	Malay	2019	17	Single	Secondary	Unemployed	Rural	No	Yes	No	No	No	No	No	No	No	No
1462	HM0048	Male	Malay	2020	20	Single	Tertiary	Private	Rural	No	Yes	No	Yes	No	No	No	No	No	No
1463	HM0049	Female	Malay	2019	16	Single	Secondary	Unemployed	Rural	No	Yes	No	No	No	No	No	No	No	No
1464	HM0050	Male	Malay	2020	16	Single	Secondary	Unemployed	Rural	No	Yes	No	Yes	No	No	No	No	No	No
1465	HM0051	Male	Malay	2020	19	Single	Secondary	Private	Rural	No	No	Yes	No	No	No	No	No	No	No
1466	HM0052	Male	Malay	2021	20	Single	Secondary	Private	Urban	No	Yes	No	No	No	No	Yes	No	No	No

No	Patient ID	Gender	Ethnic group	Year of diagnosis	Age	Marital status	Education level	Occupation	Place of residence	History of psy illness	Tobacco	Alcohol	Cannabis	ATS	Inhalant	Sedative	Hallucinogens	Opioids	Kratom
1467	HM0053	Male	Malay	2020	19	Single	Secondary	Unemployed	Rural	No	Yes	Yes	No	Yes	No	No	No	No	No
1468	HM0054	Female	Malay	2020	19	Single	Tertiary	Private	Rural	No	No	No	No	No	No	Yes	No	No	No
1469	HM0055	Male	Malay	2019	18	Single	Tertiary	Unemployed	Rural	No	Yes	No	No	No	No	No	No	No	No
1470	HM0056	Female	Malay	2021	21	Single	Secondary	Private	Rural	No	Yes	No	No	No	No	No	No	No	No
1471	HM0057	Female	Malay	2018	15	Single	Secondary	Unemployed	Rural	No	Yes	Yes	No	Yes	Yes	No	No	No	No
1472	HM0058	Male	Malay	2020	20	Single	Tertiary	Unemployed	Rural	No	Yes	No	Yes	No	No	No	No	No	No
1473	HM0059	Male	Malay	2019	19	Single	Secondary	Unemployed	Urban	No	Yes	No	No	No	No	No	No	No	No
1474	HM0060	Male	Malay	2019	15	Single	Secondary	Unemployed	Rural	No	Yes	No	No	No	Yes	No	No	No	No
1475	HM0061	Male	Malay	2019	18	Single	Secondary	Unemployed	Rural	No	Yes	No	No	Yes	No	No	No	No	No
1476	HM0062	Male	Malay	2021	20	Single	Tertiary	Unemployed	Rural	No	Yes	No	No	No	No	Yes	No	No	Yes
1477	HM0063	Male	Malay	2021	20	Single	Tertiary	Unemployed	Rural	No	Yes	No	No	No	No	No	No	No	No
1478	HM0065	Male	Malay	2021	21	Single	Tertiary	Unemployed	Rural	No	Yes	No	No	No	No	No	No	No	No
1479	HM0066	Male	Malay	2021	20	Single	Secondary	Private	Rural	No	Yes	No	Yes	Yes	No	Yes	No	No	No
1480	HM0067	Male	Malay	2021	19	Single	Tertiary	Unemployed	Rural	No	Yes	No	No	Yes	No	No	No	No	No
1481	HM0068	Male	Malay	2021	21	Single	Tertiary	Unemployed	Rural	No	Yes	No	No	No	No	No	No	No	No
1482	HM0069	Male	Malay	2019	16	Single	Secondary	Unemployed	Rural	No	Yes	Yes	No	No	No	No	No	No	No
1483	HM0070	Male	Malay	2020	19	Single	Secondary	Private	Rural	No	Yes	No	No	No	No	Yes	No	No	No
1484	HM0071	Male	Malay	2018	18	Single	Secondary	Unemployed	Rural	No	Yes	No	No	Yes	No	No	No	No	No
1485	HM0072	Female	Malay	2019	19	Single	Secondary	Unemployed	Rural	No	Yes	Yes	No	Yes	No	No	No	No	No
1486	HM0073	Male	Malay	2021	21	Single	Tertiary	Unemployed	Rural	No	Yes	No	No	No	No	No	No	No	No
1487	HM0074	Male	Indian	2021	20	Single	Secondary	Private	Urban	No	No	Yes	No	No	No	No	No	No	No
1488	HM0075	Female	Malay	2020	36	Married	Secondary	Private	Rural	No	Yes	No	No	No	No	No	No	No	No
1489	HM0076	Male	Malay	2018	45	Single	Secondary	Private	Rural	No	Yes	No	No	No	No	No	Yes	No	No
1490	HM0077	Female	Malay	2020	49	Others	No formal education	Private	Rural	No	Yes	No	No	No	No	No	No	Yes	No
1491	HM0078	Male	Malay	2021	40	Single	Secondary	Unemployed	Rural	Yes	Yes	No	No	No	No	No	No	No	No
1492	HM0079	Male	Malay	2020	27	Single	Secondary	Private	Rural	No	Yes	No	No	No	No	No	No	No	Yes
1493	HM0080	Male	Malay	2021	31	Married	Tertiary	Government	Rural	No	Yes	No	No	No	No	No	No	No	No
1494	HM0081	Male	Malay	2018	38	Single	Tertiary	Unemployed	Rural	No	Yes	No	No	No	No	No	No	No	No
1495	HM0084	Male	Malay	2019	30	Single	Tertiary	Self-employed	Rural	No	Yes	No	No	No	No	No	No	No	No
1496	HM0085	Male	Malay	2019	42	Others	No formal education	Others	Rural	Yes	No	No	No	No	No	No	No	Yes	No
1497	HM0087	Female	Chinese	2018	52	Others	Primary	Private	Urban	No	No	Yes	No	Yes	No	No	No	No	No
1498	HM0088	Male	Chinese	2021	50	Married	No formal education	Private	Rural	No	Yes	Yes	No	No	No	No	No	No	No
1499	HM0090	Male	Malay	2020	25	Single	Secondary	Unemployed	Rural	No	Yes	No	No	Yes	No	No	No	No	No
1500	HM0091	Male	Malay	2018	39	Married	Tertiary	Others	Rural	No	Yes	No	No	No	No	No	No	No	No
1501	HM0092	Male	Chinese	2018	72	Married	Secondary	Others	Rural	No	No	Yes	No	No	No	No	No	No	No
1502	HM0094	Male	Malay	2018	57	Married	Secondary	Others	Rural	Yes	Yes	Yes	No	No	No	No	No	No	No
1503	HM0095	Male	Malay	2021	27	Single	Secondary	Private	Rural	No	No	No	No	No	Yes	No	No	No	No
1504	HM0096	Male	Malay	2019	30	Single	Secondary	Unemployed	Rural	No	Yes	No	Yes	Yes	No	No	No	Yes	No
1505	HM0097	Male	Malay	2021	53	Married	Secondary	Unemployed	Rural	No	Yes	No	No	No	No	No	No	No	No
1506	HM0098	Male	Malay	2021	38	Married	No formal education	Private	Rural	No	Yes	No	No	No	No	No	No	No	No

No	Patient ID	Gender	Ethnic group	Year of diagnosis	Age	Marital status	Education level	Occupation	Place of residence	History of psy illness	Tobacco	Alcohol	Cannabis	ATS	Inhalant	Sedative	Hallucinogens	Opioids	Kratom
1507	HM0099	Female	Chinese	2018	53	Married	Primary	Self-employed	Rural	No	No	Yes	No	No	No	No	No	No	No
1508	HM0100	Male	Malay	2019	38	Married	No formal education	Government	Rural	No	Yes	No	No	Yes	No	No	No	No	No
1509	HM0101	Male	Malay	2018	28	Married	Tertiary	Private	Rural	No	Yes	No	No	Yes	No	No	No	No	No
1510	HM0102	Male	Chinese	2020	54	Others	Secondary	Unemployed	Rural	No	No	Yes	No	No	No	No	No	No	No
1511	HM0103	Male	Chinese	2018	45	Single	Primary	Private	Rural	No	Yes	No	No	No	No	No	No	No	No
1512	HM0105	Male	Malay	2020	48	Single	Secondary	Unemployed	Rural	Yes	Yes	No	No	No	No	No	No	No	No
1513	HM0107	Male	Malay	2019	24	Single	No formal education	Others	Rural	No	Yes	No	No	No	No	No	No	No	No
1514	HM0108	Male	Chinese	2019	50	Single	Secondary	Unemployed	Rural	No	Yes	No	No	No	No	No	No	No	No
1515	HM0109	Male	Malay	2018	48	Single	No formal education	Others	Rural	No	Yes	No	No	No	No	No	Yes	No	No
1516	HM0111	Female	Malay	2018	34	Single	No formal education	Unemployed	Rural	No	No	No	No	Yes	No	No	No	No	No
1517	HM0112	Male	Malay	2018	78	Others	Primary	Others	Rural	No	Yes	No	No	No	No	No	No	No	No
1518	HM0113	Male	Malay	2019	48	Married	No formal education	Unemployed	Rural	No	Yes	No	No	No	No	No	No	No	No
1519	HM0114	Male	Chinese	2019	25	Married	No formal education	Others	Rural	No	Yes	No	No	No	No	No	No	No	No
1520	HM0115	Male	Others	2018	24	Single	Tertiary	Unemployed	Rural	Yes	Yes	Yes	No	No	No	No	No	No	No
1521	HM0116	Male	Malay	2018	27	Single	Secondary	Private	Rural	Yes	Yes	No	No	No	No	No	No	No	No
1522	HM0117	Male	Malay	2018	46	Married	Secondary	Private	Rural	No	Yes	No	No	No	No	No	No	No	No
1523	HM0119	Male	Malay	2020	32	Single	Secondary	Unemployed	Rural	No	Yes	No	No	Yes	No	No	No	No	No
1524	HM0120	Male	Malay	2020	54	Married	Tertiary	Private	Rural	No	Yes	No	No	No	No	No	No	No	No
1525	HM0121	Male	Malay	2021	46	Married	Tertiary	Private	Rural	No	Yes	No	No	No	No	No	No	No	No
1526	HM0122	Male	Chinese	2019	48	Married	Primary	Unemployed	Rural	No	Yes	Yes	No	No	No	No	No	No	No
1527	HM0123	Male	Malay	2020	23	Single	Secondary	Private	Rural	No	Yes	No	No	No	No	No	No	No	No
1528	HM0124	Male	Malay	2019	39	Single	Secondary	Unemployed	Rural	No	Yes	Yes	No	Yes	No	No	No	No	No
1529	HM0125	Female	Chinese	2019	64	Married	Secondary	Private	Rural	Yes	Yes	No	No	No	No	No	No	No	No
1530	HM0128	Male	Malay	2019	25	Married	No formal education	Others	Rural	No	No	No	No	Yes	No	No	No	No	No
1531	HM0129	Male	Malay	2021	36	Single	Tertiary	Self-employed	Rural	Yes	Yes	No	No	No	No	No	No	No	No
1532	HM0130	Female	Chinese	2018	38	Single	Tertiary	Unemployed	Rural	No	Yes	No	No	No	No	No	No	No	No
1533	HM0133	Male	Chinese	2021	34	Single	Secondary	Private	Rural	No	Yes	Yes	No	No	No	No	No	No	No
1534	HM0135	Male	Malay	2018	22	Single	Secondary	Unemployed	Rural	No	Yes	No	No	No	No	No	No	No	No
1535	HM0138	Male	Malay	2019	32	Married	Tertiary	Others	Rural	No	Yes	No	No	Yes	No	No	Yes	No	No
1536	HM0139	Female	Chinese	2020	54	Others	Secondary	Others	Rural	No	No	Yes	No	No	No	No	Yes	Yes	No
1537	HM0140	Male	Malay	2018	70	Married	Tertiary	Others	Rural	No	Yes	No	No	No	No	No	No	No	No
1538	HM0141	Male	Malay	2018	30	Single	No formal education	Private	Rural	Yes	Yes	No	Yes	No	No	No	No	Yes	No
1539	HM0142	Male	Chinese	2019	38	Single	Tertiary	Private	Rural	No	No	Yes	No	No	No	No	No	No	No
1540	HM0143	Male	Malay	2019	30	Single	Secondary	Others	Rural	No	Yes	No	No	No	No	No	No	No	No
1541	HM0145	Male	Malay	2018	49	Married	No formal education	Unemployed	Rural	No	Yes	No	No	No	No	No	No	No	No

No	Patient ID	Gender	Ethnic group	Year of diagnosis	Age	Marital status	Education level	Occupation	Place of residence	History of psy illness	Tobacco	Alcohol	Cannabis	ATS	Inhalant	Sedative	Hallucinogens	Opioids	Kratom
1542	HM0146	Male	Malay	2018	48	Single	No formal education	Unemployed	Rural	Yes	No	No	No	Yes	No	No	No	No	No
1543	HM0147	Female	Chinese	2020	73	Married	No formal education	Others	Rural	No	No	Yes	No	No	No	No	No	No	No
1544	HM0148	Male	Chinese	2019	27	Single	No formal education	Others	Rural	No	No	Yes	No	No	No	No	No	No	No
1545	HM0153	Male	Malay	2018	27	Others	Tertiary	Government	Rural	No	Yes	No	No	No	No	No	No	No	No
1546	HM0154	Male	Malay	2019	52	Single	Primary	Private	Rural	Yes	Yes	Yes	No	No	No	No	No	No	No
1547	HM0156	Male	Chinese	2020	68	Married	No formal education	Unemployed	Rural	No	No	Yes	No	No	No	No	No	No	No
1548	HM0159	Female	Malay	2019	40	Others	Tertiary	Government	Rural	No	Yes	No	No	No	No	No	No	No	No
1549	HM0160	Male	Indian	2020	45	Married	Tertiary	Government	Rural	No	No	Yes	No	No	No	No	No	No	No
1550	HM0162	Male	Chinese	2019	33	Single	Tertiary	Private	Rural	No	Yes	No	No	No	No	Yes	No	No	No
1551	HM0163	Male	Malay	2020	43	Married	Secondary	Government	Rural	No	Yes	Yes	No	No	No	No	No	No	No
1552	HM0164	Male	Malay	2018	34	Single	Secondary	Government	Rural	No	Yes	No	No	Yes	Yes	No	No	Yes	No
1553	HM0165	Male	Malay	2019	29	Others	Tertiary	Unemployed	Rural	No	Yes	Yes	No	Yes	No	No	No	No	No
1554	HM0167	Male	Malay	2018	64	Married	Secondary	Others	Rural	No	Yes	No	No	No	No	No	No	No	No
1555	HM0168	Male	Indian	2021	31	Single	Secondary	Unemployed	Rural	Yes	Yes	Yes	No	Yes	No	No	No	No	No
1556	HM0170	Male	Malay	2019	51	Others	Secondary	Private	Rural	No	Yes	No	No	No	No	No	No	No	No
1557	HM0171	Male	Malay	2021	51	Married	Secondary	Unemployed	Rural	No	Yes	No	No	No	No	No	No	No	No
1558	HM0172	Female	Others	2021	32	Single	Secondary	Others	Rural	No	No	Yes	No	No	No	No	No	No	No
1559	HM0173	Female	Chinese	2018	66	Single	Primary	Others	Rural	No	Yes	No	No	No	No	No	No	No	No
1560	HM0174	Male	Chinese	2018	37	Single	Secondary	Private	Rural	No	Yes	No	No	No	No	No	No	Yes	No
1561	HM0175	Male	Malay	2019	18	Single	Secondary	Unemployed	Rural	No	Yes	No	No	No	No	No	No	No	No
1562	HM0177	Female	Malay	2021	31	Single	Secondary	Private	Rural	Yes	Yes	No	No	No	No	No	No	No	No
1563	HM0178	Male	Chinese	2018	37	Single	Secondary	Private	Urban	No	Yes	No	No	No	No	No	No	No	No
1564	HM0179	Male	Chinese	2018	21	Single	Tertiary	Private	Urban	No	Yes	Yes	No	No	No	No	No	No	No
1565	HM0180	Male	Chinese	2018	44	Married	Tertiary	Private	Rural	No	No	Yes	No	No	No	No	No	No	No
1566	HM0182	Male	Others	2018	26	Single	Tertiary	Private	Rural	Yes	No	Yes	No	No	No	No	No	No	No
1567	HM0183	Male	Malay	2020	32	Single	Secondary	Private	Urban	No	Yes	Yes	No	No	No	No	No	No	No
1568	HM0184	Male	Chinese	2020	41	Married	Tertiary	Private	Rural	No	Yes	Yes	No	No	No	No	No	No	No
1569	HM0185	Male	Chinese	2019	43	Others	Secondary	Private	Rural	No	Yes	Yes	No	No	No	No	No	No	No
1570	HM0186	Male	Indian	2020	50	Married	Secondary	Private	Rural	No	Yes	Yes	No	No	No	No	No	No	No
1571	HM0187	Male	Malay	2020	28	Married	Secondary	Private	Rural	No	Yes	No	No	No	No	No	No	No	No
1572	HM0188	Male	Indian	2020	42	Single	Tertiary	Private	Rural	Yes	No	Yes	No	No	No	No	No	No	No
1573	HM0189	Male	Malay	2021	46	Others	No formal education	Government	Rural	No	Yes	No	Yes	No	No	No	No	No	No
1574	HM0190	Male	Chinese	2018	47	Others	Tertiary	Private	Rural	No	No	Yes	No	No	No	No	No	No	No
1575	HM0191	Male	Indian	2019	59	Married	Secondary	Private	Rural	No	Yes	No	No	No	No	No	No	No	No
1576	HM0192	Male	Malay	2019	33	Married	Tertiary	Private	Rural	No	Yes	Yes	No	Yes	No	No	No	Yes	No
1577	HM0193	Male	Malay	2018	45	Others	Secondary	Others	Rural	No	Yes	No	No	No	No	No	No	No	No
1578	HM0197	Male	Indian	2018	65	Married	Secondary	Others	Rural	No	Yes	No	No	No	No	No	No	No	No
1579	HM0198	Male	Malay	2018	25	Single	Secondary	Unemployed	Rural	No	Yes	No	No	Yes	No	No	No	No	No
1580	HM0199	Male	Malay	2018	31	Married	Tertiary	Private	Rural	No	Yes	No	No	No	No	No	No	No	No

No	Patient ID	Gender	Ethnic group	Year of diagnosis	Age	Marital status	Education level	Occupation	Place of residence	History of psy illness	Tobacco	Alcohol	Cannabis	ATS	Inhalant	Sedative	Hallucinogens	Opioids	Kratom
1581	HM0204	Male	Others	2020	42	Single	No formal education	Others	Urban	No	Yes	No	No	Yes	No	No	No	Yes	No
1582	HM0205	Female	Malay	2018	35	Single	No formal education	Unemployed	Rural	No	Yes	No	No	No	No	No	No	No	No
1583	HM0206	Male	Malay	2019	27	Single	Primary	Unemployed	Rural	No	Yes	No	No	No	No	No	No	No	No
1584	HM0208	Male	Chinese	2020	53	Single	Primary	Unemployed	Rural	No	Yes	No	No	Yes	No	No	No	No	No
1585	HM0209	Male	Indian	2020	35	Married	No formal education	Private	Rural	No	Yes	No	No	No	No	No	No	No	No
1586	HM0211	Male	Malay	2020	29	Others	Secondary	Private	Rural	No	Yes	No	Yes	Yes	Yes	No	No	Yes	Yes
1587	HM0212	Male	Malay	2018	32	Others	No formal education	Unemployed	Rural	No	No	No	No	Yes	No	No	No	No	No
1588	HM0213	Male	Malay	2019	75	Married	No formal education	Self-employed	Rural	No	Yes	Yes	No	No	No	No	No	No	No
1589	HM0214	Male	Chinese	2019	78	Married	Secondary	Private	Rural	No	No	Yes	No	No	No	No	No	No	No
1590	HM0215	Male	Malay	2019	32	Married	Secondary	Private	Rural	No	Yes	No	No	No	No	No	No	No	No
1591	HM0217	Male	Malay	2020	33	Married	Secondary	Self-employed	Rural	No	Yes	No	No	No	No	No	No	No	No
1592	HM0218	Male	Malay	2021	26	Single	Secondary	Government	Rural	No	Yes	No	No	No	No	Yes	No	Yes	No
1593	HM0219	Male	Malay	2018	33	Married	Secondary	Private	Rural	No	Yes	No	No	No	No	Yes	No	No	No
1594	HM0221	Male	Malay	2018	33	Married	Secondary	Government	Rural	No	Yes	Yes	No	No	No	No	No	No	No
1595	HM0222	Male	Malay	2019	21	Single	Secondary	Unemployed	Rural	No	Yes	No	No	No	No	No	No	No	No
1596	HM0222	Male	Malay	2019	22	Single	Secondary	Unemployed	Rural	No	Yes	No	No	No	No	No	No	No	No
1597	HM0223	Male	Chinese	2020	34	Single	Tertiary	Unemployed	Rural	No	No	No	No	No	No	Yes	No	No	No
1598	HM0225	Male	Malay	2020	35	Married	Secondary	Unemployed	Rural	No	Yes	No	No	No	No	No	No	No	No
1599	HM0226	Male	Malay	2020	21	Single	Tertiary	Unemployed	Rural	No	Yes	Yes	No	No	No	No	No	No	No
1600	HM0227	Male	Malay	2019	21	Single	Secondary	Unemployed	Rural	No	Yes	No	No	No	No	No	No	No	No
1601	HM0230	Female	Malay	2019	21	Married	Secondary	Private	Rural	No	No	Yes	No	No	No	No	No	No	No
1602	HM0234	Male	Indian	2018	55	Married	Secondary	Private	Rural	No	Yes	Yes	No	No	No	No	No	Yes	No
1603	HM0236	Female	Malay	2018	22	Others	No formal education	Others	Urban	No	Yes	No	No	Yes	No	No	No	No	No
1604	HM0237	Male	Malay	2018	58	Married	Secondary	Unemployed	Rural	No	Yes	No	No	No	No	No	No	No	No
1605	HM0238	Male	Malay	2019	33	Single	No formal education	Unemployed	Rural	Yes	Yes	Yes	No	Yes	No	No	No	No	Yes
1606	HM0241	Male	Malay	2018	40	Married	Secondary	Self-employed	Rural	No	No	No	No	Yes	No	No	No	Yes	No
1607	HM0242	Male	Malay	2018	84	Married	Primary	Unemployed	Rural	No	Yes	No	No	No	No	No	No	No	No
1608	HM0243	Female	Chinese	2020	69	Married	Secondary	Others	Rural	No	No	Yes	No	No	No	No	No	No	No
1609	HM0245	Male	Malay	2020	23	Single	Secondary	Unemployed	Rural	No	Yes	Yes	No	Yes	Yes	No	No	No	No
1610	HM0247	Male	Malay	2020	25	Single	Tertiary	Others	Rural	No	Yes	No	No	No	No	No	No	No	No
1611	HM0248	Male	Malay	2020	47	Single	Secondary	Private	Rural	No	Yes	No	Yes	Yes	No	No	No	Yes	No
1612	HM0249	Male	Chinese	2019	43	Single	Tertiary	Private	Rural	No	Yes	No	No	No	No	No	No	No	No
1613	HM0250	Male	Malay	2020	37	Married	Tertiary	Self-employed	Rural	No	Yes	No	No	No	No	No	No	No	No
1614	HM0251	Male	Malay	2019	29	Single	Primary	Private	Rural	No	Yes	No	No	No	No	No	No	No	No
1615	HM0252	Male	Malay	2018	30	Single	Secondary	Unemployed	Rural	No	Yes	No	Yes	Yes	Yes	No	No	No	No
1616	HM0253	Male	Malay	2019	38	Single	No formal education	Private	Rural	No	Yes	No	No	Yes	No	No	No	No	No

No	Patient ID	Gender	Ethnic group	Year of diagnosis	Age	Marital status	Education level	Occupation	Place of residence	History of psy illness	Tobacco	Alcohol	Cannabis	ATS	Inhalant	Sedative	Hallucinogens	Opioids	Kratom
1617	HM0254	Male	Malay	2018	39	Married	Tertiary	Others	Rural	No	Yes	No	Yes	Yes	No	No	No	No	No
1618	HM0255	Male	Chinese	2019	49	Single	Secondary	Private	Rural	No	Yes	No	No	No	No	No	No	No	No
1619	HM0257	Male	Malay	2019	22	Single	Tertiary	Unemployed	Rural	No	No	No	No	Yes	No	No	No	No	No
1620	HM0258	Female	Malay	2020	33	Single	Secondary	Unemployed	Rural	No	Yes	Yes	No	No	No	No	No	No	No
1621	HM0260	Male	Malay	2020	32	Married	Tertiary	Private	Rural	No	Yes	No	No	No	No	No	No	No	No
1622	HM0261	Male	Chinese	2018	24	Single	Tertiary	Self-employed	Rural	No	Yes	Yes	No	No	No	No	No	No	No
1623	HM0263	Male	Chinese	2018	34	Single	Secondary	Self-employed	Rural	No	Yes	Yes	No	No	No	No	No	No	No
1624	HM0265	Male	Malay	2018	36	Married	Tertiary	Private	Rural	No	Yes	No	No	No	No	No	No	No	No
1625	HM0266	Male	Malay	2018	49	Married	Secondary	Private	Rural	No	Yes	No	No	No	No	No	No	No	No
1626	HM0269	Male	Chinese	2018	34	Single	Secondary	Unemployed	Rural	No	Yes	Yes	No	No	No	No	No	No	No
1627	HM0270	Male	Malay	2018	57	Married	Secondary	Private	Rural	No	Yes	No	No	No	No	No	No	No	No
1628	HM0271	Male	Malay	2018	37	Single	Secondary	Private	Rural	No	Yes	Yes	No	No	No	No	No	Yes	No
1629	HM0272	Male	Malay	2019	58	Single	Secondary	Unemployed	Rural	No	Yes	No	No	No	No	No	No	No	No
1630	HM0273	Female	Chinese	2019	46	Others	Secondary	Private	Rural	No	No	Yes	No	No	No	No	No	No	No
1631	HM0274	Female	Malay	2021	34	Single	Secondary	Private	Rural	No	Yes	No	No	No	No	No	No	No	No
1632	HM0275	Male	Malay	2018	32	Others	Secondary	Government	Rural	No	Yes	No	No	Yes	No	No	No	No	No
1633	HM0276	Female	Malay	2020	41	Married	Tertiary	Unemployed	Rural	No	No	Yes	No	No	No	No	No	No	No
1634	HM0277	Male	Others	2019	27	Single	Secondary	Private	Rural	No	Yes	No	No	No	No	No	No	No	No
1635	HM0278	Male	Malay	2020	45	Married	Secondary	Private	Rural	No	No	Yes	No	No	No	No	No	No	No
1636	HM0279	Male	Malay	2018	23	Single	No formal education	Others	Rural	No	No	No	No	Yes	No	No	No	No	No
1637	HM0280	Male	Chinese	2020	61	Others	Primary	Private	Rural	No	Yes	Yes	No	Yes	No	No	No	No	No
1638	HM0281	Male	Malay	2020	32	Single	Secondary	Private	Rural	Yes	No	No	No	Yes	No	No	No	Yes	No
1639	HM0282	Male	Malay	2021	37	Single	Secondary	Government	Rural	No	Yes	No	No	No	No	No	No	No	No
1640	HM0283	Male	Chinese	2020	44	Others	Secondary	Unemployed	Rural	No	Yes	Yes	No	Yes	No	No	No	No	No
1641	HM0284	Male	Chinese	2020	53	Single	Secondary	Unemployed	Rural	No	Yes	No	No	No	No	No	No	No	No
1642	HM0285	Male	Malay	2019	46	Others	Primary	Unemployed	Rural	No	Yes	Yes	No	No	No	No	No	No	No
1643	HM0286	Male	Chinese	2021	30	Married	Secondary	Private	Rural	No	Yes	Yes	No	No	No	No	No	No	No
1644	HM0287	Male	Indian	2018	34	Single	Tertiary	Unemployed	Rural	No	Yes	Yes	No	No	No	No	No	No	No
1645	HM0288	Male	Malay	2021	63	Married	Primary	Unemployed	Rural	No	Yes	No	No	No	No	No	No	No	No
1646	HM0289	Male	Malay	2019	26	Married	Tertiary	Private	Rural	No	Yes	No	No	No	No	No	No	No	No
1647	HM0290	Female	Malay	2021	22	Single	Secondary	Private	Rural	No	Yes	No	No	No	No	No	No	No	No
1648	HM0292	Male	Malay	2018	25	Married	Secondary	Government	Rural	No	Yes	No	No	No	No	No	No	No	Yes
1649	HM0293	Male	Malay	2021	22	Single	No formal education	Unemployed	Rural	No	No	No	No	Yes	No	No	No	No	No
1650	HM0296	Male	Malay	2020	26	Single	Tertiary	Private	Rural	No	Yes	No	Yes	Yes	No	No	No	No	No
1651	HM0297	Male	Malay	2019	30	Single	No formal education	Self-employed	Rural	No	Yes	No	No	Yes	No	No	No	No	No
1652	HM0298	Male	Malay	2019	47	Married	Secondary	Unemployed	Rural	No	Yes	No	No	No	No	No	No	No	No
1653	HM0299	Male	Chinese	2018	21	Single	Secondary	Private	Rural	No	No	Yes	No	No	No	No	No	No	No
1654	HM0300	Male	Chinese	2018	58	Others	Secondary	Unemployed	Rural	No	Yes	Yes	No	No	No	No	No	No	No
1655	HM0301	Male	Chinese	2020	36	Married	Tertiary	Others	Rural	No	No	Yes	No	No	No	No	No	No	No
1656	HM0302	Male	Malay	2019	37	Others	No formal education	Others	Rural	No	No	No	Yes	Yes	No	No	No	No	No

No	Patient ID	Gender	Ethnic group	Year of diagnosis	Age	Marital status	Education level	Occupation	Place of residence	History of psy illness	Tobacco	Alcohol	Cannabis	ATS	Inhalant	Sedative	Hallucinogens	Opioids	Kratom
1657	HM0303	Female	Malay	2021	41	Single	Secondary	Unemployed	Rural	No	Yes	No	No	Yes	No	No	No	No	No
1658	HM0304	Male	Malay	2018	22	Single	Secondary	Unemployed	Rural	No	Yes	No	Yes	No	No	No	No	No	Yes
1659	HM0305	Male	Chinese	2018	63	Single	Secondary	Others	Rural	Yes	Yes	No	No	No	No	No	No	No	No
1660	HM0306	Male	Chinese	2019	35	Others	Secondary	Private	Rural	Yes	No	Yes	No	No	No	No	No	No	No
1661	HM0308	Male	Malay	2019	38	Others	Tertiary	Self-employed	Rural	No	Yes	Yes	No	No	No	No	No	No	No
1662	HM0309	Male	Chinese	2021	45	Others	Secondary	Private	Rural	Yes	Yes	Yes	No	No	No	No	No	Yes	No
1663	HM0310	Male	Malay	2020	29	Married	Secondary	Government	Rural	No	No	No	No	Yes	No	No	No	No	No
1664	HM0312	Male	Chinese	2021	54	Married	Tertiary	Private	Rural	Yes	No	Yes	No	No	No	No	No	No	No
1665	HM0313	Male	Malay	2020	27	Single	Secondary	Unemployed	Rural	No	No	No	No	Yes	No	No	No	No	No
1666	HM0315	Male	Chinese	2020	38	Others	Secondary	Private	Rural	No	Yes	No	Yes	Yes	No	No	No	No	No
1667	HM0317	Male	Malay	2018	21	Single	No formal education	Others	Rural	No	Yes	No	No	Yes	No	No	No	Yes	No
1668	HM0319	Male	Malay	2018	28	Single	No formal education	Unemployed	Rural	No	Yes	No	No	No	No	No	No	No	Yes
1669	HM0320	Male	Malay	2018	37	Single	Primary	Unemployed	Rural	No	Yes	No	No	No	No	No	No	No	No
1670	HM0321	Male	Malay	2019	26	Single	Tertiary	Government	Rural	No	No	Yes	No	No	No	No	No	No	No
1671	HM0323	Male	Malay	2020	22	Single	No formal education	Others	Rural	No	Yes	No	No	No	No	No	No	No	No
1672	HM0324	Male	Malay	2019	53	Married	No formal education	Government	Rural	No	Yes	No	No	No	No	No	No	No	No
1673	HM0325	Male	Malay	2021	34	Others	Secondary	Private	Rural	No	Yes	No	No	Yes	No	No	No	No	No
1674	HM0327	Male	Chinese	2018	76	Married	Primary	Unemployed	Rural	No	No	Yes	No	No	No	No	No	No	No
1675	HM0328	Male	Malay	2021	34	Single	Primary	Government	Rural	No	Yes	No	No	No	No	No	No	No	No
1676	HM0329	Male	Chinese	2018	51	Others	Secondary	Private	Rural	No	Yes	No	No	No	No	No	No	No	No
1677	HM0330	Male	Malay	2018	40	Others	Secondary	Private	Rural	No	Yes	No	Yes	Yes	No	Yes	No	No	No
1678	HM0332	Male	Chinese	2020	74	Married	Primary	Self-employed	Rural	No	No	Yes	No	No	No	No	No	No	No
1679	HM0333	Male	Malay	2018	28	Married	No formal education	Government	Rural	No	No	No	No	No	No	Yes	No	No	No
1680	HM0334	Male	Malay	2018	40	Married	No formal education	Self-employed	Rural	No	Yes	No	No	No	No	No	No	No	No
1681	HM0335	Male	Chinese	2018	83	Married	No formal education	Unemployed	Rural	No	No	Yes	No	No	No	No	No	No	No
1682	HM0336	Male	Malay	2018	19	Single	Secondary	Unemployed	Rural	No	Yes	No	No	No	No	No	No	No	No
1683	HM0338	Male	Malay	2019	38	Married	Tertiary	Private	Rural	No	Yes	No	No	Yes	No	No	No	No	No
1684	HM0339	Male	Chinese	2018	34	Married	Secondary	Private	Rural	No	Yes	Yes	No	No	No	No	No	No	No
1685	HM0340	Male	Malay	2018	25	Single	Secondary	Private	Rural	No	Yes	No	No	No	No	No	No	No	No
1686	HM0340	Male	Malay	2018	27	Single	Secondary	Private	Rural	Yes	Yes	No	Yes	No	No	No	No	No	No

No	Patient ID	Gender	Ethnic group	Year of diagnosis	Age	Marital status	Education level	Occupation	Place of residence	History of psy illness	Tobacco	Alcohol	Cannabis	ATS	Inhalant	Sedative	Hallucinogens	Opioids	Kratom
1687	HM0342	Male	Malay	2018	29	Single	Tertiary	Unemployed	Rural	No	No	No	No	Yes	No	No	No	No	No
1688	HM0344	Male	Indian	2020	32	Single	Secondary	Unemployed	Rural	No	Yes	No	No	No	No	No	No	No	No
1689	HM0345	Male	Malay	2019	48	Married	Secondary	Private	Rural	No	Yes	No	No	No	No	No	No	No	No
1690	HM0346	Male	Chinese	2021	49	Others	No formal education	Private	Rural	No	Yes	Yes	No	No	No	No	No	No	No
1691	HM0347	Male	Chinese	2021	44	Single	Secondary	Private	Rural	No	Yes	No	No	No	No	No	No	No	No
1692	HM0348	Male	Malay	2019	45	Others	Secondary	Self-employed	Rural	Yes	Yes	No	Yes	No	No	No	No	No	No
1693	HM0350	Male	Malay	2020	34	Married	Secondary	Private	Rural	No	Yes	No	No	No	No	No	No	No	No
1694	HM0351	Male	Others	2020	44	Married	Tertiary	Others	Rural	No	Yes	No	No	No	No	No	No	No	No
1695	HM0353	Male	Malay	2018	38	Married	No formal education	Private	Rural	No	Yes	No	No	No	Yes	No	No	No	No
1696	HM0354	Male	Malay	2019	26	Married	Secondary	Private	Rural	Yes	Yes	No	No	No	No	No	No	No	No
1697	HM0355	Male	Malay	2018	40	Single	Secondary	Private	Rural	Yes	Yes	Yes	No	No	No	No	No	No	No
1698	HM0356	Male	Malay	2019	32	Married	Tertiary	Private	Rural	No	No	No	Yes	No	No	No	No	No	No
1699	HM0357	Male	Indian	2021	23	Others	Secondary	Private	Rural	No	Yes	Yes	No	No	No	No	No	No	No
1700	HM0360	Male	Malay	2020	39	Married	Secondary	Government	Rural	No	Yes	No	No	No	No	No	No	No	No
1701	HM0361	Male	Malay	2019	36	Married	Tertiary	Unemployed	Rural	No	No	No	Yes	Yes	No	No	No	No	No
1702	HM0363	Male	Chinese	2019	55	Single	Secondary	Private	Rural	No	Yes	Yes	No	No	No	No	No	No	No
1703	HM0364	Male	Indian	2018	35	Others	Tertiary	Private	Rural	No	Yes	Yes	Yes	Yes	No	No	No	No	No
1704	HM0366	Female	Chinese	2020	28	Single	Tertiary	Private	Rural	No	Yes	Yes	Yes	Yes	No	No	No	No	No
1705	HM0368	Male	Malay	2019	53	Single	Primary	Private	Rural	No	Yes	No	No	No	No	No	No	No	No
1706	HM0369	Male	Malay	2019	24	Single	No formal education	Self-employed	Rural	No	Yes	No	No	Yes	No	No	No	No	No
1707	HM0370	Male	Malay	2018	30	Married	Tertiary	Government	Rural	No	Yes	No	No	No	No	No	No	No	No
1708	HM0371	Female	Malay	2019	64	Married	No formal education	Others	Rural	No	Yes	No	No	No	No	No	No	No	No
1709	HM0372	Male	Malay	2018	34	Single	Secondary	Others	Rural	No	Yes	No	No	No	No	No	No	No	No
1710	HM0373	Male	Chinese	2020	60	Married	Tertiary	Others	Rural	Yes	No	Yes	No	No	No	No	No	No	No
1711	HM0374	Female	Chinese	2019	35	Single	No formal education	Private	Rural	Yes	Yes	Yes	No	No	No	No	No	No	No
1712	HM0375	Male	Malay	2018	81	Married	Primary	Others	Rural	No	Yes	No	No	No	No	No	No	No	No
1713	HM0377	Male	Malay	2020	31	Single	Secondary	Private	Rural	No	Yes	No	No	No	No	No	No	No	No
1714	HM0381	Male	Chinese	2018	39	Single	Secondary	Unemployed	Rural	No	Yes	Yes	No	No	No	No	No	No	No
1715	HM0382	Male	Malay	2018	51	Single	Secondary	Private	Rural	No	Yes	No	No	Yes	No	No	No	No	No
1716	HM0384	Male	Chinese	2019	32	Single	Secondary	Unemployed	Rural	No	No	No	No	Yes	No	No	No	No	No
1717	HM0385	Female	Malay	2020	25	Married	Tertiary	Private	Rural	Yes	No	No	No	Yes	No	No	No	No	No
1718	HM0386	Male	Malay	2018	27	Single	Secondary	Unemployed	Rural	No	Yes	Yes	Yes	Yes	No	No	No	No	No
1719	HM0387	Male	Malay	2020	43	Single	Secondary	Private	Rural	No	No	No	No	No	No	No	No	Yes	No
1720	HM0388	Male	Malay	2018	33	Single	Secondary	Unemployed	Rural	No	Yes	No	No	Yes	Yes	No	No	No	No
1721	HM0391	Female	Malay	2021	51	Others	Secondary	Unemployed	Rural	No	Yes	No	No	Yes	No	No	No	No	No
1722	HM0392	Male	Malay	2018	23	Others	Secondary	Private	Rural	No	Yes	No	No	Yes	No	No	No	No	No
1723	HM0393	Female	Chinese	2018	41	Married	Secondary	Unemployed	Rural	No	Yes	No	No	Yes	No	No	No	No	No
1724	HM0394	Male	Indian	2018	30	Married	Secondary	Unemployed	Rural	No	Yes	Yes	Yes	Yes	No	No	No	Yes	No

No	Patient ID	Gender	Ethnic group	Year of diagnosis	Age	Marital status	Education level	Occupation	Place of residence	History of psy illness	Tobacco	Alcohol	Cannabis	ATS	Inhalant	Sedative	Hallucinogens	Opioids	Kratom
1725	HM0395	Male	Malay	2020	47	Single	No formal education	Self-employed	Rural	No	Yes	No	No	Yes	No	No	No	Yes	No
1726	HM0396	Male	Malay	2018	35	Married	No formal education	Unemployed	Rural	No	Yes	No	No	Yes	No	No	No	No	No
1727	HM0397	Male	Malay	2020	41	Others	Secondary	Unemployed	Rural	No	No	No	No	Yes	No	No	No	Yes	No
1728	HM0398	Female	Malay	2020	31	Single	Tertiary	Private	Rural	No	No	No	No	Yes	No	No	No	No	No
1729	HM0399	Male	Malay	2020	33	Single	Tertiary	Private	Rural	No	Yes	No	No	No	No	No	No	No	No
1730	HM0400	Female	Indian	2019	24	Single	Secondary	Others	Rural	No	No	No	No	No	No	No	No	Yes	No
1731	HM0401	Male	Malay	2020	21	Single	Secondary	Private	Rural	No	Yes	No	No	Yes	No	No	No	No	No
1732	HM0402	Male	Malay	2021	30	Single	Tertiary	Others	Rural	No	Yes	No	No	Yes	No	No	No	Yes	No
1733	HM0406	Female	Malay	2021	27	Married	Primary	Others	Rural	No	Yes	No	No	Yes	No	No	No	No	No
1734	HM0407	Male	Chinese	2018	51	Married	Tertiary	Private	Rural	No	No	Yes	Yes	No	No	No	No	No	No
1735	HM0408	Male	Chinese	2021	43	Single	No formal education	Others	Rural	No	Yes	Yes	No	Yes	No	No	No	No	No
1736	HM0409	Male	Malay	2020	29	Single	Tertiary	Government	Rural	No	Yes	No	Yes	No	No	No	No	No	No
1737	HM0410	Male	Malay	2018	33	Others	No formal education	Government	Rural	Yes	No	No	Yes	No	No	No	No	No	No
1738	HM0411	Male	Malay	2018	34	Single	No formal education	Others	Rural	No	No	No	No	Yes	No	No	No	Yes	No
1739	HM0412	Male	Malay	2019	21	Single	Secondary	Others	Rural	No	No	No	Yes	Yes	No	No	No	No	Yes
1740	HM0413	Male	Malay	2019	33	Single	Secondary	Unemployed	Rural	No	Yes	No	No	Yes	No	No	No	No	No
1741	HM0414	Female	Malay	2021	42	Married	Secondary	Private	Rural	No	No	No	No	Yes	No	No	No	No	No
1742	HM0415	Male	Malay	2019	34	Single	Secondary	Unemployed	Rural	No	No	No	Yes	Yes	No	Yes	No	No	Yes
1743	HM0416	Male	Chinese	2018	35	Married	No formal education	Self-employed	Rural	No	Yes	No	No	No	No	Yes	No	No	No
1744	HM0417	Male	Malay	2021	33	Single	Secondary	Others	Rural	No	Yes	No	No	Yes	No	No	No	No	No
1745	HM0418	Male	Malay	2019	37	Married	Tertiary	Private	Rural	No	Yes	No	No	Yes	No	No	No	No	No
1746	HM0419	Male	Malay	2018	33	Married	No formal education	Others	Rural	No	Yes	No	Yes	Yes	No	No	No	No	No
1747	HM0423	Male	Malay	2018	24	Single	Secondary	Self-employed	Rural	No	Yes	No	No	Yes	No	No	No	No	No
1748	HM0424	Male	Malay	2019	32	Others	Secondary	Private	Rural	Yes	Yes	No	No	Yes	No	No	No	No	No
1749	HM0425	Male	Malay	2019	41	Single	Tertiary	Self-employed	Rural	No	No	No	No	Yes	No	No	No	No	No
1750	HM0426	Male	Malay	2019	22	Single	Tertiary	Unemployed	Rural	Yes	Yes	Yes	No	No	No	No	No	No	Yes
1751	HM0427	Male	Malay	2020	33	Single	Secondary	Unemployed	Rural	No	Yes	No	No	Yes	No	No	No	No	No
1752	HM0428	Male	Malay	2020	25	Single	Secondary	Private	Rural	No	Yes	No	No	Yes	No	No	No	No	No
1753	HM0429	Male	Chinese	2020	37	Others	Secondary	Unemployed	Rural	No	Yes	Yes	No	No	No	No	No	No	No
1754	HM0430	Male	Chinese	2019	32	Others	No formal education	Others	Rural	Yes	Yes	Yes	No	Yes	No	No	No	No	No
1755	HM0431	Male	Malay	2020	47	Others	No formal education	Others	Rural	No	Yes	No	No	Yes	No	No	No	No	No
1756	HM0432	Male	Malay	2018	47	Single	Secondary	Self-employed	Rural	No	Yes	No	No	Yes	No	No	No	No	No
1757	HM0433	Male	Others	2021	55	Single	Secondary	Private	Rural	No	Yes	Yes	No	No	No	No	No	No	No
1758	HM0434	Male	Malay	2018	21	Single	Secondary	Private	Rural	No	Yes	Yes	Yes	Yes	Yes	No	No	No	No

No	Patient ID	Gender	Ethnic group	Year of diagnosis	Age	Marital status	Education level	Occupation	Place of residence	History of psy illness	Tobacco	Alcohol	Cannabis	ATS	Inhalant	Sedative	Hallucinogens	Opioids	Kratom
1759	HM0437	Male	Chinese	2019	23	Single	Tertiary	Unemployed	Rural	No	No	Yes	No	No	No	No	No	No	No
1760	HM0439	Male	Chinese	2018	48	Single	Secondary	Self-employed	Rural	No	Yes	No	No	Yes	No	No	No	No	No
1761	HM0441	Male	Malay	2018	51	Single	Secondary	Private	Rural	No	Yes	No	No	Yes	No	No	No	No	No
1762	HM0442	Male	Malay	2019	41	Others	Tertiary	Government	Rural	No	Yes	No	No	No	No	No	No	No	No
1763	HM0443	Male	Malay	2020	23	Single	Secondary	Private	Rural	No	No	Yes	No	Yes	No	No	No	No	No
1764	HM0445	Male	Indian	2020	49	Others	Tertiary	Private	Rural	No	Yes	Yes	No	No	No	No	No	No	No
1765	HM0447	Male	Malay	2021	51	Others	No formal education	Others	Rural	Yes	Yes	No	No	No	No	No	No	Yes	No
1766	HM0448	Male	Malay	2020	35	Single	Secondary	Private	Rural	No	Yes	No	No	No	No	No	No	No	No
1767	HM0449	Male	Malay	2018	42	Single	Primary	Others	Rural	No	No	No	Yes	Yes	No	No	No	Yes	No
1768	HM0452	Male	Malay	2018	30	Married	Secondary	Government	Rural	No	Yes	No	No	No	No	No	No	No	No
1769	HM0453	Male	Chinese	2019	45	Married	Secondary	Private	Rural	No	Yes	Yes	No	No	No	No	No	No	No
1770	HM0455	Male	Chinese	2019	34	Single	Secondary	Private	Rural	Yes	Yes	No	Yes	Yes	No	No	No	No	No
1771	HM0456	Male	Malay	2021	39	Married	Tertiary	Government	Rural	No	Yes	No	No	No	No	No	No	No	No
1772	HM0458	Male	Chinese	2021	30	Others	Secondary	Private	Urban	No	Yes	No	No	No	No	No	No	No	No
1773	HM0461	Female	Chinese	2020	42	Others	Secondary	Unemployed	Rural	No	Yes	No	Yes	Yes	No	No	No	No	No
1774	HM0462	Male	Malay	2020	32	Single	Secondary	Private	Rural	No	Yes	No	No	No	No	No	No	No	No
1775	HM0463	Male	Malay	2020	24	Single	Secondary	Private	Rural	No	Yes	No	No	No	No	No	No	No	No
1776	HM0464	Male	Malay	2019	60	Married	Primary	Unemployed	Rural	No	Yes	No	No	No	No	No	No	No	No
1777	HM0465	Male	Indian	2018	29	Married	Secondary	Private	Rural	No	Yes	Yes	No	Yes	No	No	No	No	No
1778	HM0468	Male	Malay	2019	34	Single	Secondary	Private	Rural	No	Yes	No	No	Yes	No	No	No	Yes	No
1779	HM0469	Male	Malay	2019	21	Single	Tertiary	Unemployed	Rural	Yes	Yes	No	Yes	No	No	No	No	No	No
1780	HM0470	Male	Chinese	2021	44	Married	Tertiary	Private	Urban	No	Yes	No	No	No	No	No	No	No	No
1781	HM0471	Female	Chinese	2018	23	Single	Secondary	Others	Rural	Yes	Yes	No	Yes	Yes	No	No	No	No	No
1782	HM0472	Male	Indian	2019	28	Single	Primary	Unemployed	Rural	No	Yes	No	No	No	No	No	No	No	No
1783	HM0473	Male	Chinese	2019	61	Married	Secondary	Others	Rural	Yes	Yes	No	No	No	No	No	No	No	No
1784	HM0475	Male	Chinese	2020	38	Others	Secondary	Private	Rural	Yes	Yes	No	No	Yes	No	No	No	No	No
1785	HM0476	Male	Malay	2019	67	Single	Secondary	Others	Rural	Yes	Yes	No	No	No	No	No	No	No	No
1786	HM0477	Female	Malay	2018	25	Single	Secondary	Unemployed	Rural	No	No	No	No	Yes	No	No	No	No	No
1787	HM0478	Female	Chinese	2018	56	Married	Tertiary	Government	Rural	No	No	Yes	No	No	No	No	No	No	No
1788	HM0479	Male	Chinese	2020	48	Single	Secondary	Unemployed	Rural	Yes	Yes	Yes	No	No	No	No	No	No	No
1789	HM0480	Male	Malay	2020	45	Others	Secondary	Private	Rural	Yes	Yes	No	Yes	Yes	No	No	No	No	No
1790	HM0481	Male	Chinese	2018	39	Single	Secondary	Self-employed	Rural	No	No	Yes	No	No	No	No	No	No	No
1791	HM0482	Male	Indian	2020	23	Married	Secondary	Unemployed	Rural	Yes	No	No	No	Yes	No	No	No	No	No
1792	HM0483	Male	Malay	2018	20	Single	Secondary	Private	Rural	No	Yes	Yes	Yes	No	No	No	No	No	Yes
1793	HM0484	Male	Malay	2018	24	Single	Secondary	Others	Rural	No	No	No	No	Yes	No	No	No	No	No
1794	HM0485	Male	Chinese	2018	66	Single	No formal education	Others	Rural	No	Yes	Yes	No	No	No	No	No	No	No
1795	HM0486	Male	Malay	2019	22	Single	Secondary	Others	Rural	No	Yes	No	No	No	No	No	No	No	No
1796	HM0488	Male	Malay	2018	38	Single	Secondary	Unemployed	Rural	No	Yes	No	No	No	No	No	No	No	No

No	Patient ID	Gender	Ethnic group	Year of diagnosis	Age	Marital status	Education level	Occupation	Place of residence	History of psy illness	Tobacco	Alcohol	Cannabis	ATS	Inhalant	Sedative	Hallucinogens	Opioids	Kratom
1797	HM0489	Female	Malay	2021	24	Single	Secondary	Unemployed	Rural	No	Yes	No	Yes	No	No	No	No	No	No
1798	HM0490	Male	Malay	2021	25	Single	Tertiary	Private	Rural	No	Yes	No	Yes	No	No	No	No	No	No
1799	HM0492	Male	Malay	2018	24	Others	Tertiary	Others	Rural	No	Yes	Yes	Yes	No	No	No	No	No	No
1800	HM0493	Male	Malay	2018	31	Single	Secondary	Self-employed	Rural	No	Yes	No	No	Yes	No	No	No	No	No
1801	HM0494	Male	Malay	2021	34	Married	Secondary	Government	Rural	No	Yes	No	No	No	No	No	No	No	No
1802	HM0495	Male	Malay	2018	44	Others	No formal education	Unemployed	Rural	Yes	No	No	Yes	No	No	No	No	No	No
1803	HM0496	Male	Malay	2020	58	Married	Secondary	Unemployed	Rural	No	Yes	No	No	No	No	No	No	No	No
1804	HM0497	Male	Malay	2019	28	Single	Tertiary	Private	Rural	No	No	Yes	No	No	No	No	No	No	No
1805	HM0499	Female	Chinese	2019	53	Married	Tertiary	Unemployed	Rural	No	No	Yes	No	No	No	No	No	No	No
1806	HM0500	Male	Malay	2021	34	Married	Secondary	Government	Rural	No	Yes	No	No	No	No	No	No	No	No
1807	HM0501	Male	Malay	2021	35	Single	Secondary	Private	Rural	No	Yes	Yes	No	Yes	No	No	No	No	No
1808	HM0502	Male	Indian	2018	32	Others	No formal education	Others	Rural	No	Yes	Yes	Yes	No	No	No	No	No	No
1809	HM0503	Male	Malay	2021	32	Single	Tertiary	Private	Rural	Yes	No	No	No	Yes	No	No	No	No	No
1810	HM0504	Male	Malay	2018	28	Single	Secondary	Government	Rural	No	Yes	No	No	No	No	No	No	No	Yes
1811	HM0505	Male	Malay	2020	34	Single	Secondary	Unemployed	Rural	No	No	No	No	Yes	No	No	No	No	No
1812	HM0506	Male	Malay	2021	26	Single	Tertiary	Private	Rural	No	Yes	No	No	No	No	No	No	No	Yes
1813	HM0507	Male	Chinese	2020	36	Single	Secondary	Private	Rural	No	Yes	Yes	No	No	No	No	No	No	No
1814	HM0508	Male	Indian	2020	52	Married	Primary	Unemployed	Rural	No	Yes	Yes	No	No	No	No	No	No	No
1815	HM0510	Male	Malay	2021	27	Single	Secondary	Unemployed	Rural	No	Yes	No	No	Yes	No	No	No	No	No
1816	HM0511	Male	Chinese	2018	37	Single	Tertiary	Self-employed	Rural	Yes	No	Yes	No	No	No	No	No	No	No
1817	HM0514	Male	Malay	2018	28	Married	Tertiary	Government	Rural	No	Yes	No	No	No	No	No	No	No	No
1818	HM0515	Male	Malay	2019	24	Single	Secondary	Unemployed	Rural	No	Yes	No	No	No	No	No	No	Yes	No
1819	HM0517	Female	Chinese	2020	43	Others	No formal education	Unemployed	Rural	Yes	No	Yes	No	No	No	No	No	No	No
1820	HM0519	Male	Malay	2018	40	Others	No formal education	Others	Rural	Yes	No	No	No	Yes	No	No	No	Yes	No
1821	HM0520	Male	Chinese	2019	69	Single	Primary	Private	Rural	No	Yes	No	No	No	No	No	No	No	No
1822	HM0521	Male	Chinese	2019	35	Single	Secondary	Private	Rural	No	Yes	Yes	No	No	No	No	No	No	No
1823	HM0522	Male	Malay	2018	44	Others	No formal education	Others	Rural	Yes	No	No	No	Yes	No	No	No	No	No
1824	HM0523	Male	Chinese	2018	74	Others	Tertiary	Private	Rural	No	Yes	No	No	No	No	No	No	No	No
1825	HM0524	Male	Indian	2018	59	Married	Secondary	Self-employed	Rural	No	No	Yes	No	No	No	No	No	No	No
1826	HM0525	Male	Others	2021	37	Single	No formal education	Others	Urban	No	Yes	Yes	No	No	No	No	No	No	No
1827	HM0526	Female	Malay	2019	36	Married	No formal education	Others	Rural	No	No	No	No	Yes	No	No	No	No	No
1828	HM0528	Male	Chinese	2018	33	Single	No formal education	Unemployed	Rural	No	Yes	Yes	No	No	No	No	No	No	No
1829	HM0529	Female	Malay	2019	27	Married	Tertiary	Private	Rural	No	Yes	No	No	No	No	No	No	No	No

No	Patient ID	Gender	Ethnic group	Year of diagnosis	Age	Marital status	Education level	Occupation	Place of residence	History of psy illness	Tobacco	Alcohol	Cannabis	ATS	Inhalant	Sedative	Hallucinogens	Opioids	Kratom
1830	HM0530	Male	Chinese	2018	68	Single	Primary	Unemployed	Rural	No	No	Yes	No	No	No	No	No	No	No
1831	HM0531	Male	Indian	2018	52	Others	No formal education	Private	Rural	No	Yes	No	No	No	No	No	No	No	No
1832	HM0532	Male	Malay	2019	36	Others	Secondary	Private	Rural	No	Yes	No	No	No	No	No	No	No	No
1833	HM0534	Female	Malay	2018	31	Others	Secondary	Unemployed	Rural	No	Yes	Yes	No	No	No	No	No	No	No
1834	HM0535	Male	Malay	2020	35	Others	Secondary	Private	Rural	No	Yes	Yes	Yes	No	No	No	No	No	No
1835	HM0536	Male	Malay	2019	31	Others	Secondary	Unemployed	Rural	No	Yes	No	No	Yes	No	No	No	No	No
1836	HM0537	Male	Chinese	2020	65	Single	Secondary	Unemployed	Rural	No	Yes	No	No	No	No	No	No	No	No
1837	HM0538	Male	Malay	2020	42	Others	Secondary	Private	Rural	No	No	No	No	No	No	No	No	Yes	No
1838	HM0539	Male	Malay	2018	22	Single	Secondary	Unemployed	Rural	No	Yes	No	No	No	No	No	No	No	No
1839	HM0540	Male	Chinese	2020	37	Single	Secondary	Private	Rural	Yes	Yes	No	No	No	No	No	No	No	No
1840	HM0541	Male	Malay	2019	28	Single	Secondary	Government	Rural	No	Yes	No	No	No	No	No	No	No	No
1841	HM0543	Male	Chinese	2019	38	Single	No formal education	Unemployed	Rural	No	Yes	Yes	No	No	No	No	No	No	No
1842	HM0544	Male	Chinese	2018	36	Married	No formal education	Private	Rural	No	No	Yes	No	No	No	No	No	No	No
1843	HM0545	Male	Malay	2020	52	Others	Secondary	Private	Rural	No	Yes	No	No	No	No	No	No	Yes	No
1844	HM0546	Male	Malay	2019	23	Single	Tertiary	Others	Rural	No	Yes	No	No	No	No	No	No	No	No
1845	HM0548	Male	Chinese	2021	53	Married	Tertiary	Private	Rural	No	No	Yes	No	No	No	No	No	No	No
1846	HM0550	Male	Malay	2020	54	Single	Secondary	Private	Rural	No	Yes	No	No	No	No	No	No	No	No
1847	HM0552	Male	Malay	2019	46	Others	Tertiary	Private	Rural	No	Yes	No	No	No	No	No	No	No	No
1848	HM0553	Male	Malay	2020	38	Married	Secondary	Private	Rural	No	Yes	No	No	No	No	No	No	No	Yes
1849	HM0554	Male	Malay	2020	22	Single	Tertiary	Unemployed	Rural	Yes	Yes	No	No	No	No	No	No	No	No
1850	HM0555	Male	Malay	2020	50	Single	Secondary	Others	Rural	Yes	Yes	No	No	No	No	No	No	No	No
1851	HM0556	Male	Malay	2019	50	Married	No formal education	Others	Rural	No	Yes	No	No	No	No	No	No	No	No
1852	HM0557	Male	Malay	2019	36	Single	Tertiary	Self-employed	Rural	No	Yes	No	No	No	No	No	No	No	No
1853	HM0558	Male	Malay	2018	38	Single	No formal education	Others	Rural	No	No	No	No	Yes	No	No	No	No	No
1854	HM0559	Female	Chinese	2021	49	Single	Secondary	Private	Urban	Yes	No	Yes	No	No	No	No	No	No	No
1855	HM0560	Female	Malay	2021	49	Single	Secondary	Private	Rural	No	Yes	No	No	No	No	No	No	No	No
1856	HM0561	Female	Malay	2020	23	Others	Primary	Unemployed	Rural	No	Yes	No	No	Yes	No	No	No	No	No
1857	HM0562	Male	Malay	2021	29	Single	Tertiary	Self-employed	Rural	No	Yes	Yes	No	No	No	No	No	No	No
1858	HM0563	Male	Indian	2020	51	Married	Primary	Unemployed	Rural	No	Yes	No	No	No	No	No	No	No	No
1859	HM0564	Female	Chinese	2019	23	Others	Secondary	Private	Rural	No	Yes	No	No	Yes	No	No	Yes	No	No
1860	HM0565	Male	Malay	2020	30	Single	Secondary	Self-employed	Rural	No	Yes	No	No	Yes	No	No	No	Yes	No
1861	HM0566	Male	Malay	2020	26	Single	Tertiary	Unemployed	Rural	No	Yes	No	No	Yes	No	No	No	No	No
1862	HM0567	Male	Malay	2019	34	Married	Tertiary	Private	Rural	No	Yes	No	No	No	No	No	No	No	No
1863	HM0568	Male	Malay	2021	27	Others	Tertiary	Private	Rural	No	Yes	Yes	No	No	No	No	No	No	No
1864	HM0569	Male	Indian	2018	67	Married	Secondary	Others	Rural	No	Yes	No	No	No	No	No	No	No	No
1865	HM0570	Male	Indian	2021	28	Others	Tertiary	Self-employed	Rural	No	Yes	Yes	No	No	No	No	No	No	No
1866	HM0571	Male	Malay	2019	35	Married	Secondary	Private	Rural	No	Yes	No	No	No	No	No	No	No	No
1867	HM0572	Male	Malay	2018	52	Others	Secondary	Self-employed	Rural	No	Yes	No	No	No	No	No	No	No	No

No	Patient ID	Gender	Ethnic group	Year of diagnosis	Age	Marital status	Education level	Occupation	Place of residence	History of psy illness	Tobacco	Alcohol	Cannabis	ATS	Inhalant	Sedative	Hallucinogens	Opioids	Kratom
1868	HM0574	Male	Malay	2018	31	Married	No formal education	Government	Rural	No	Yes	No	No	No	No	No	No	No	No
1869	HM0575	Male	Malay	2018	22	Single	Secondary	Private	Rural	No	Yes	No	No	No	No	No	No	No	Yes
1870	HM0577	Male	Chinese	2018	27	Single	Secondary	Unemployed	Rural	No	No	Yes	No	No	No	No	No	No	No
1871	HM0578	Male	Malay	2018	22	Single	Primary	Private	Rural	No	Yes	No	No	Yes	No	No	No	No	No
1872	HM0579	Male	Malay	2019	50	Single	Secondary	Others	Rural	Yes	Yes	No	No	No	No	No	No	No	No
1873	HM0580	Male	Malay	2019	35	Married	Tertiary	Government	Rural	No	Yes	No	No	Yes	No	No	No	No	No
1874	HM0581	Male	Malay	2019	32	Married	Secondary	Private	Rural	No	Yes	No	No	Yes	No	No	No	No	No
1875	HM0582	Male	Malay	2020	30	Others	No formal education	Others	Rural	No	Yes	No	No	Yes	No	No	No	No	No
1876	HM0583	Male	Malay	2021	27	Others	No formal education	Private	Rural	No	No	Yes	No	No	No	No	No	No	No
1877	HM0584	Male	Chinese	2018	44	Single	Secondary	Others	Rural	Yes	Yes	Yes	No	No	No	No	No	No	No
1878	HM0589	Male	Chinese	2018	47	Married	Tertiary	Private	Rural	No	Yes	No	No	No	No	No	No	No	No
1879	HM0592	Male	Chinese	2020	31	Single	Secondary	Unemployed	Rural	No	No	No	No	Yes	No	No	No	No	No
1880	HM0593	Male	Malay	2020	24	Others	Secondary	Private	Rural	No	No	No	No	Yes	No	No	No	No	No
1881	HM0594	Male	Malay	2020	31	Single	Secondary	Unemployed	Rural	No	Yes	No	No	No	No	No	No	No	No
1882	HM0595	Male	Malay	2020	21	Single	No formal education	Unemployed	Rural	No	Yes	No	No	Yes	No	No	No	No	No
1883	HM0597	Female	Malay	2019	25	Single	No formal education	Unemployed	Rural	No	Yes	No	No	No	No	No	No	No	No
1884	HM0598	Male	Malay	2021	34	Single	Tertiary	Unemployed	Rural	Yes	Yes	No	No	No	No	No	No	No	No
1885	HM0600	Female	Malay	2018	70	Others	No formal education	Unemployed	Rural	Yes	Yes	No	No	No	No	No	No	No	No
1886	HM0601	Male	Malay	2019	28	Single	Tertiary	Private	Rural	No	Yes	No	No	No	No	No	No	No	No
1887	HM0604	Male	Indian	2020	43	Married	Secondary	Unemployed	Rural	No	No	Yes	No	No	No	No	No	No	No
1888	HM0605	Male	Malay	2019	23	Single	Secondary	Private	Rural	No	Yes	No	No	No	No	No	No	No	No
1889	HM0606	Female	Malay	2019	38	Single	Secondary	Private	Rural	No	Yes	No	No	Yes	No	No	No	No	No
1890	HM0607	Male	Malay	2019	22	Single	Tertiary	Unemployed	Rural	Yes	No	Yes	No	No	No	No	No	No	No
1891	HM0608	Male	Malay	2021	23	Single	Tertiary	Unemployed	Rural	No	Yes	No	No	No	No	No	No	No	No
1892	HM0609	Male	Chinese	2018	49	Married	Secondary	Unemployed	Rural	Yes	Yes	No	No	No	No	No	No	No	No
1893	HM0610	Male	Malay	2019	22	Others	Secondary	Unemployed	Rural	No	No	No	No	Yes	No	No	No	No	No
1894	HM0612	Male	Malay	2020	24	Others	Tertiary	Unemployed	Rural	No	Yes	No	No	No	No	No	No	No	No
1895	HM0613	Male	Chinese	2019	33	Married	No formal education	Unemployed	Rural	No	Yes	No	No	Yes	No	No	No	No	No
1896	HM0616	Male	Malay	2018	64	Married	Primary	Others	Rural	No	Yes	No	No	No	No	No	No	No	No
1897	HM0618	Male	Malay	2018	27	Single	Tertiary	Unemployed	Rural	No	Yes	No	No	No	No	No	No	No	No
1898	HM0619	Male	Malay	2018	27	Single	Tertiary	Private	Rural	No	Yes	Yes	No	No	No	No	No	No	No
1899	HM0621	Male	Chinese	2019	26	Single	No formal education	Private	Rural	No	No	Yes	No	No	No	No	No	No	No
1900	HM0622	Male	Malay	2019	28	Others	No formal education	Self-employed	Rural	Yes	Yes	No	No	No	No	No	No	No	No

No	Patient ID	Gender	Ethnic group	Year of diagnosis	Age	Marital status	Education level	Occupation	Place of residence	History of psy illness	Tobacco	Alcohol	Cannabis	ATS	Inhalant	Sedative	Hallucinogens	Opioids	Kratom
1901	HM0623	Male	Malay	2018	25	Single	Primary	Unemployed	Rural	No	No	No	No	Yes	No	No	No	No	No
1902	HM0624	Male	Chinese	2020	50	Single	Primary	Self-employed	Rural	No	Yes	No	No	No	No	No	No	No	No
1903	HM0626	Male	Malay	2020	29	Others	Secondary	Unemployed	Rural	No	Yes	No	No	Yes	No	No	No	No	Yes
1904	HM0627	Male	Malay	2020	24	Single	Secondary	Private	Rural	Yes	Yes	No	No	No	No	No	No	No	No
1905	HM0628	Female	Malay	2018	45	Married	Secondary	Private	Rural	No	Yes	No	No	No	No	No	No	No	No
1906	HM0630	Male	Indian	2020	29	Married	Secondary	Private	Rural	No	Yes	Yes	Yes	Yes	No	No	No	No	No
1907	HM0631	Male	Malay	2019	21	Others	Tertiary	Private	Rural	No	Yes	No	No	Yes	No	No	No	No	Yes
1908	HM0632	Male	Malay	2020	55	Married	Secondary	Government	Rural	No	Yes	No	No	No	No	No	No	No	No
1909	HM0633	Male	Malay	2018	39	Others	Tertiary	Private	Rural	Yes	No	No	No	Yes	No	No	No	No	No
1910	HM0634	Male	Malay	2018	27	Married	Secondary	Private	Rural	No	Yes	Yes	No	Yes	No	No	No	No	No
1911	HM0635	Male	Malay	2018	49	Married	No formal education	Private	Rural	No	Yes	No	No	No	No	No	No	No	No
1912	HM0636	Male	Malay	2021	43	Married	Tertiary	Unemployed	Rural	No	Yes	Yes	No	Yes	No	No	No	No	No
1913	HM0638	Male	Indian	2020	45	Married	No formal education	Self-employed	Rural	No	No	Yes	No	No	No	No	No	No	No
1914	HM0639	Male	Malay	2019	60	Married	No formal education	Unemployed	Rural	No	Yes	No	No	No	No	No	No	No	No
1915	HM0641	Male	Others	2018	25	Single	Tertiary	Unemployed	Rural	No	Yes	No	No	No	No	No	No	No	No
1916	HM0642	Male	Malay	2020	31	Married	Tertiary	Self-employed	Rural	No	Yes	No	No	No	No	No	No	No	No
1917	HM0643	Male	Malay	2020	38	Married	No formal education	Others	Rural	No	Yes	No	No	Yes	No	No	No	No	No
1918	HM0644	Male	Malay	2019	31	Others	Primary	Unemployed	Rural	No	Yes	No	No	No	No	No	No	No	No
1919	HM0645	Male	Indian	2018	30	Single	Secondary	Others	Rural	No	No	No	Yes	Yes	No	No	No	No	No
1920	HM0646	Male	Indian	2019	35	Married	Secondary	Private	Rural	No	Yes	Yes	No	No	No	No	No	No	No
1921	HM0647	Male	Malay	2018	63	Married	No formal education	Others	Rural	No	Yes	No	No	Yes	No	No	No	No	No
1922	HM0649	Male	Malay	2021	56	Married	Secondary	Private	Rural	No	Yes	No	No	No	No	No	No	No	No
1923	HM0650	Male	Indian	2018	21	Single	Secondary	Private	Rural	No	Yes	No	No	No	No	No	No	Yes	No
1924	HM0652	Male	Malay	2018	40	Married	Secondary	Unemployed	Rural	No	Yes	Yes	Yes	No	No	No	No	No	No
1925	HM0653	Male	Chinese	2020	27	Single	Tertiary	Private	Rural	No	Yes	Yes	No	No	No	No	No	No	No
1926	HM0655	Male	Malay	2018	21	Single	Tertiary	Private	Rural	No	Yes	No	Yes	No	No	Yes	No	No	No
1927	HM0656	Female	Malay	2019	35	Married	Tertiary	Private	Urban	No	Yes	No	No	No	No	No	No	No	No
1928	HM0657	Male	Malay	2018	27	Single	Secondary	Private	Rural	No	Yes	Yes	No	No	No	No	No	No	No
1929	HM0658	Male	Malay	2019	33	Married	No formal education	Self-employed	Rural	No	No	No	No	Yes	No	No	No	No	Yes
1930	HM0659	Male	Malay	2019	28	Single	Tertiary	Government	Rural	No	Yes	No	No	Yes	No	No	No	No	No
1931	HM0660	Male	Malay	2019	36	Single	Tertiary	Unemployed	Rural	No	Yes	Yes	No	No	No	No	No	No	No
1932	HM0661	Male	Chinese	2021	47	Single	Primary	Unemployed	Rural	No	Yes	No	No	No	No	No	No	No	No
1933	HM0663	Male	Malay	2018	22	Single	Tertiary	Unemployed	Rural	No	Yes	No	No	No	No	No	No	No	No
1934	HM0664	Male	Indian	2020	30	Single	Tertiary	Self-employed	Rural	No	Yes	Yes	No	No	No	No	No	No	No

No	Patient ID	Gender	Ethnic group	Year of diagnosis	Age	Marital status	Education level	Occupation	Place of residence	History of psy illness	Tobacco	Alcohol	Cannabis	ATS	Inhalant	Sedative	Hallucinogens	Opioids	Kratom
1935	HM0665	Male	Malay	2018	67	Married	Primary	Self-employed	Rural	No	Yes	No	No	No	No	No	No	No	No
1936	HM0666	Male	Malay	2020	27	Single	Secondary	Unemployed	Rural	No	Yes	No	No	Yes	No	No	No	No	No
1937	HM0669	Male	Malay	2020	29	Single	No formal education	Others	Rural	No	Yes	No	No	No	No	No	No	No	No
1938	HM0670	Male	Malay	2019	59	Single	Secondary	Unemployed	Rural	No	Yes	Yes	No	No	No	No	No	No	No
1939	HM0671	Male	Malay	2019	23	Single	Secondary	Private	Rural	Yes	Yes	No	No	No	No	No	No	No	No
1940	HM0672	Male	Malay	2018	38	Single	Secondary	Private	Rural	No	Yes	No	No	No	No	No	No	No	No
1941	HM0674	Male	Chinese	2018	50	Single	Secondary	Self-employed	Rural	No	No	Yes	No	No	No	No	No	No	No
1942	HM0675	Male	Malay	2021	22	Single	Tertiary	Unemployed	Rural	No	Yes	No	Yes	No	No	No	No	No	Yes
1943	HM0676	Male	Malay	2018	31	Single	Secondary	Private	Rural	Yes	Yes	Yes	Yes	No	No	No	No	No	No
1944	HM0678	Male	Indian	2018	26	Others	Tertiary	Private	Rural	No	Yes	Yes	Yes	No	No	No	No	No	No
1945	HM0679	Male	Malay	2020	29	Single	Secondary	Private	Rural	No	Yes	No	No	No	No	No	No	No	No
1946	HM0680	Male	Malay	2018	39	Married	Secondary	Unemployed	Rural	Yes	Yes	No	No	No	No	No	No	No	No
1947	HM0681	Male	Malay	2021	24	Single	Secondary	Private	Rural	No	Yes	No	No	Yes	No	No	No	No	No
1948	HM0683	Male	Chinese	2020	32	Single	No formal education	Others	Rural	No	Yes	Yes	No	No	No	No	No	No	No
1949	HM0685	Female	Chinese	2018	24	Single	Secondary	Unemployed	Rural	No	Yes	Yes	No	No	No	No	No	No	No
1950	HM0688	Male	Indian	2018	68	Married	Secondary	Others	Rural	No	Yes	No	No	No	No	No	No	No	No
1951	HM0690	Male	Malay	2018	40	Single	Secondary	Self-employed	Rural	No	Yes	No	No	Yes	No	No	No	No	No
1952	HM0691	Male	Malay	2019	41	Others	Secondary	Self-employed	Rural	No	Yes	No	No	No	Yes	No	No	No	No
1953	HM0692	Female	Chinese	2020	26	Single	Tertiary	Private	Rural	No	No	Yes	No	No	No	No	No	No	No
1954	HM0693	Male	Malay	2018	23	Single	Secondary	Private	Rural	No	Yes	No	No	No	No	No	No	No	No
1955	HM0695	Female	Malay	2018	37	Married	Secondary	Private	Rural	No	Yes	No	No	No	No	No	No	No	No
1956	HM0699	Male	Malay	2018	31	Married	Tertiary	Private	Rural	No	Yes	No	No	No	No	No	No	No	No
1957	HM0700	Male	Malay	2018	44	Single	Secondary	Unemployed	Rural	No	Yes	Yes	Yes	Yes	Yes	No	No	Yes	Yes
1958	HM0701	Male	Malay	2021	40	Others	Tertiary	Private	Urban	Yes	Yes	No	No	No	No	No	No	No	No
1959	HM0702	Female	Malay	2021	28	Single	Secondary	Private	Rural	Yes	Yes	No	No	No	No	No	No	No	No
1960	HM0703	Male	Indian	2019	34	Single	Secondary	Private	Rural	No	Yes	Yes	No	No	No	No	No	No	No
1961	HM0704	Male	Malay	2018	28	Married	Tertiary	Others	Rural	No	Yes	No	No	No	No	No	No	No	No
1962	HM0707	Male	Malay	2021	25	Single	Secondary	Unemployed	Rural	No	Yes	No	No	Yes	No	No	No	No	No
1963	HM0708	Male	Chinese	2021	58	Married	No formal education	Private	Rural	No	Yes	Yes	No	No	No	No	No	No	No
1964	HM0709	Male	Malay	2018	35	Single	Secondary	Private	Rural	No	Yes	No	Yes	Yes	Yes	No	No	No	No
1965	HM0710	Male	Indian	2021	28	Single	Secondary	Private	Rural	No	Yes	Yes	No	No	No	No	No	No	No
1966	HM0711	Male	Chinese	2020	24	Single	Secondary	Private	Urban	Yes	Yes	Yes	No	No	No	No	No	No	No
1967	HM0712	Male	Malay	2020	33	Single	Tertiary	Private	Rural	No	Yes	No	Yes	Yes	No	No	No	No	No
1968	HM0713	Female	Indian	2020	32	Married	Secondary	Private	Rural	No	Yes	Yes	No	No	No	No	No	No	No
1969	HM0715	Male	Malay	2021	32	Single	Tertiary	Self-employed	Rural	No	Yes	No	No	No	No	No	No	No	No
1970	HM0716	Male	Indian	2019	25	Single	Tertiary	Self-employed	Rural	No	Yes	Yes	No	No	No	No	No	No	No
1971	HM0717	Male	Malay	2019	42	Married	Tertiary	Government	Rural	No	Yes	No	No	No	No	No	No	No	No
1972	HM0718	Male	Malay	2020	39	Single	Secondary	Self-employed	Rural	No	Yes	No	No	Yes	No	No	No	Yes	No
1973	HM0719	Male	Malay	2019	25	Single	Secondary	Private	Rural	No	Yes	No	No	No	No	No	No	No	No

No	Patient ID	Gender	Ethnic group	Year of diagnosis	Age	Marital status	Education level	Occupation	Place of residence	History of psy illness	Tobacco	Alcohol	Cannabis	ATS	Inhalant	Sedative	Hallucinogens	Opioids	Kratom
1974	HM0721	Male	Malay	2019	45	Others	No formal education	Others	Rural	No	Yes	Yes	No	No	No	No	No	No	No
1975	HM0723	Male	Malay	2021	27	Married	Tertiary	Self-employed	Urban	No	Yes	No	No	No	No	No	No	No	Yes
1976	HM0724	Male	Malay	2019	20	Single	Secondary	Self-employed	Rural	No	Yes	No	No	No	No	No	No	No	No
1977	HM0725	Male	Malay	2020	37	Single	Secondary	Private	Rural	Yes	Yes	No	No	No	No	No	No	No	No
1978	HM0726	Male	Chinese	2020	42	Married	Tertiary	Unemployed	Rural	No	No	Yes	No	No	No	No	No	No	No
1979	HM0729	Male	Indian	2018	37	Married	Secondary	Private	Rural	Yes	Yes	Yes	No	No	No	No	No	No	No
1980	HM0730	Male	Indian	2020	25	Single	Tertiary	Private	Urban	No	Yes	Yes	No	No	No	No	No	No	No
1981	HM0732	Male	Malay	2019	33	Married	Secondary	Self-employed	Rural	No	Yes	No	No	No	No	No	No	No	No
1982	HM0733	Male	Chinese	2018	56	Married	No formal education	Self-employed	Rural	No	Yes	Yes	No	No	No	No	No	No	No
1983	HM0735	Male	Malay	2020	30	Married	Tertiary	Private	Urban	No	Yes	No	No	No	No	No	No	No	No
1984	HM0736	Male	Malay	2021	38	Single	Tertiary	Private	Rural	No	Yes	No	No	No	No	No	No	No	No
1985	HM0737	Male	Malay	2019	33	Single	Secondary	Unemployed	Rural	Yes	Yes	No	No	No	No	No	No	No	No
1986	HM0738	Male	Malay	2021	41	Married	Secondary	Unemployed	Rural	No	Yes	No	No	No	No	No	No	No	No
1987	HM0739	Female	Malay	2018	32	Others	Secondary	Private	Rural	No	Yes	No	No	Yes	No	No	No	Yes	No
1988	HM0740	Male	Chinese	2021	30	Married	Secondary	Private	Urban	Yes	Yes	No	No	Yes	No	No	No	No	No
1989	HM0741	Male	Malay	2020	30	Others	Secondary	Private	Rural	No	Yes	Yes	No	Yes	No	No	No	Yes	No
1990	HM0742	Male	Malay	2018	33	Married	Tertiary	Private	Rural	No	Yes	No	No	Yes	No	No	No	No	No
1991	HM0743	Male	Malay	2018	33	Married	Secondary	Government	Rural	Yes	Yes	No	Yes	Yes	No	No	No	No	No
1992	HM0744	Male	Others	2018	36	Married	Secondary	Private	Rural	No	Yes	No	No	No	No	No	No	No	No
1993	HM0745	Male	Indian	2018	31	Others	Secondary	Private	Rural	No	Yes	No	No	No	No	No	No	No	No
1994	HM0746	Male	Chinese	2020	23	Single	Secondary	Private	Urban	No	Yes	Yes	No	Yes	No	No	No	No	No
1995	HM0747	Male	Malay	2019	20	Single	Secondary	Others	Rural	No	Yes	No	No	Yes	No	No	No	No	No
1996	HM0749	Male	Malay	2021	38	Others	Secondary	Government	Rural	No	Yes	No	No	No	No	No	No	No	No
1997	HM0750	Male	Chinese	2020	27	Single	Tertiary	Government	Rural	No	No	Yes	No	No	No	No	No	No	No
1998	HM0752	Male	Malay	2019	35	Single	Secondary	Private	Rural	No	Yes	Yes	Yes	Yes	No	No	No	Yes	Yes
1999	HM0753	Male	Malay	2018	33	Married	Tertiary	Government	Rural	No	No	No	No	Yes	No	No	No	No	No
2000	HM0754	Male	Malay	2020	44	Married	Secondary	Government	Urban	No	Yes	No	No	No	No	No	No	No	No
2001	HM0756	Male	Chinese	2018	26	Single	Tertiary	Government	Rural	No	No	Yes	No	No	No	No	No	No	No
2002	HM0757	Male	Indian	2021	25	Single	Secondary	Unemployed	Rural	No	No	Yes	No	No	No	No	No	No	No
2003	HM0758	Male	Chinese	2018	67	Married	Secondary	Others	Rural	No	Yes	Yes	No	No	No	No	No	No	No
2004	HM0760	Male	Malay	2019	29	Married	No formal education	Government	Rural	No	Yes	No	No	No	No	No	No	No	No
2005	HM0761	Male	Indian	2019	28	Married	Secondary	Unemployed	Rural	No	Yes	Yes	No	No	No	No	No	No	No
2006	HM0762	Male	Malay	2020	48	Married	Secondary	Private	Urban	No	Yes	No	No	No	No	No	No	No	No
2007	HM0763	Female	Malay	2020	24	Married	Secondary	Private	Rural	Yes	Yes	No	Yes	No	No	No	No	No	No
2008	HM0764	Female	Malay	2019	24	Married	Primary	Private	Rural	No	Yes	No	No	Yes	No	No	No	No	No
2009	HM0767	Male	Malay	2018	35	Single	Tertiary	Private	Rural	No	Yes	No	No	No	No	No	No	No	No
2010	HM0768	Male	Chinese	2019	71	Married	Primary	Others	Rural	No	Yes	Yes	No	No	No	No	No	No	No

No	Patient ID	Gender	Ethnic group	Year of diagnosis	Age	Marital status	Education level	Occupation	Place of residence	History of psy illness	Tobacco	Alcohol	Cannabis	ATS	Inhalant	Sedative	Hallucinogens	Opioids	Kratom
2011	HM0769	Female	Chinese	2019	54	Married	Primary	Self-employed	Rural	No	No	Yes	No	No	No	No	No	No	No
2012	HM0770	Male	Chinese	2018	51	Married	Secondary	Others	Rural	No	Yes	Yes	Yes	Yes	No	No	No	Yes	No
2013	HM0771	Male	Indian	2018	34	Married	Secondary	Private	Rural	No	No	Yes	Yes	Yes	No	No	No	No	No
2014	HM0772	Male	Chinese	2018	33	Single	Secondary	Unemployed	Rural	No	Yes	Yes	Yes	No	No	No	No	No	No
2015	HM0774	Male	Malay	2019	36	Single	Tertiary	Private	Rural	No	No	No	Yes	Yes	No	No	No	No	No
2016	HM0775	Male	Malay	2018	34	Single	Secondary	Unemployed	Rural	No	Yes	No	No	No	No	No	No	No	No
2017	HM0776	Male	Malay	2018	44	Married	Secondary	Private	Rural	No	Yes	No	No	No	No	No	No	No	No
2018	HM0779	Male	Chinese	2018	68	Married	Primary	Private	Rural	No	No	Yes	No	No	No	No	No	No	No
2019	HM0781	Male	Malay	2019	31	Single	Primary	Private	Rural	No	Yes	No	No	Yes	Yes	No	No	No	Yes
2020	HM0782	Female	Chinese	2021	25	Single	Tertiary	Private	Rural	No	No	Yes	No	No	No	No	No	No	No
2021	HM0783	Male	Chinese	2018	37	Others	No formal education	Private	Rural	Yes	Yes	No	No	Yes	No	No	No	No	No
2022	HM0784	Male	Malay	2020	25	Married	Secondary	Self-employed	Rural	No	Yes	No	No	No	No	No	Yes	No	No
2023	HM0785	Male	Malay	2020	28	Others	Tertiary	Government	Rural	No	Yes	No	No	No	No	No	No	No	No
2024	HM0786	Male	Malay	2019	43	Single	Secondary	Unemployed	Rural	No	Yes	No	No	No	No	No	No	No	No
2025	HM0787	Male	Malay	2019	29	Single	Secondary	Private	Rural	No	Yes	No	Yes	Yes	No	No	No	No	No
2026	HM0788	Male	Malay	2021	59	Single	Primary	Unemployed	Rural	No	Yes	No	No	No	No	No	No	No	No
2027	HM0789	Male	Chinese	2020	37	Single	Tertiary	Private	Rural	No	Yes	Yes	No	Yes	No	No	No	No	No
2028	HM0790	Male	Indian	2020	32	Married	Secondary	Unemployed	Rural	Yes	No	Yes	No	No	No	No	No	No	No
2029	HM0791	Female	Chinese	2020	42	Married	Secondary	Private	Rural	No	No	Yes	No	No	No	No	No	No	No
2030	HM0792	Male	Malay	2018	29	Others	Tertiary	Private	Rural	No	Yes	No	No	No	No	No	No	No	No
2031	HM0793	Male	Malay	2020	31	Others	Primary	Private	Rural	Yes	Yes	No	No	No	No	No	No	No	No
2032	HM0794	Male	Malay	2020	49	Married	Secondary	Private	Rural	No	Yes	No	No	No	No	No	No	No	No
2033	HM0795	Male	Chinese	2020	37	Others	Secondary	Private	Rural	Yes	Yes	No	No	Yes	No	No	No	No	No
2034	HM0796	Male	Indian	2018	44	Married	Secondary	Private	Rural	No	Yes	Yes	Yes	No	No	No	No	No	No
2035	HM0797	Male	Indian	2018	29	Married	Tertiary	Private	Rural	No	Yes	No	No	No	No	No	No	No	No
2036	HM0798	Male	Chinese	2020	32	Single	Tertiary	Self-employed	Rural	No	Yes	Yes	No	No	No	No	No	No	No
2037	HM0800	Female	Malay	2021	27	Others	Secondary	Unemployed	Rural	No	Yes	No	No	No	No	No	No	No	No
2038	HM0801	Male	Malay	2020	33	Others	Secondary	Private	Rural	No	Yes	No	No	Yes	No	No	No	No	No
2039	HM0802	Male	Malay	2019	25	Single	Tertiary	Private	Rural	No	Yes	No	No	No	Yes	No	No	No	No
2040	HM0803	Male	Malay	2019	40	Single	Secondary	Private	Rural	No	No	No	No	No	No	No	No	Yes	No
2041	HM0805	Male	Malay	2020	36	Married	Secondary	Private	Rural	No	Yes	No	No	No	No	No	No	No	No
2042	HM0806	Male	Malay	2018	20	Single	Secondary	Private	Rural	No	No	No	No	Yes	No	No	No	No	No
2043	HM0807	Male	Chinese	2018	48	Single	Secondary	Private	Rural	No	Yes	No	No	Yes	No	No	No	No	No
2044	HM0809	Male	Malay	2018	29	Others	Secondary	Self-employed	Rural	No	Yes	No	No	Yes	No	No	No	Yes	No
2045	HM0811	Male	Malay	2018	34	Single	Tertiary	Others	Rural	Yes	Yes	No	No	Yes	No	No	No	No	No
2046	HM0812	Male	Malay	2021	36	Married	Tertiary	Private	Rural	No	Yes	No	Yes	No	No	No	Yes	No	No
2047	HM0813	Male	Chinese	2019	23	Others	Tertiary	Others	Rural	Yes	Yes	Yes	No	No	No	No	No	No	No
2048	HM0814	Male	Chinese	2021	46	Others	Secondary	Self-employed	Urban	No	Yes	Yes	No	Yes	No	No	No	No	No
2049	HM0815	Female	Malay	2020	30	Married	Secondary	Private	Rural	No	Yes	No	No	No	No	No	No	No	No
2050	HM0816	Male	Malay	2019	22	Others	Secondary	Unemployed	Rural	No	Yes	No	No	No	No	No	No	No	No

No	Patient ID	Gender	Ethnic group	Year of diagnosis	Age	Marital status	Education level	Occupation	Place of residence	History of psy illness	Tobacco	Alcohol	Cannabis	ATS	Inhalant	Sedative	Hallucinogens	Opioids	Kratom
2051	HM0817	Male	Malay	2020	36	Single	Secondary	Unemployed	Rural	No	Yes	No	Yes	Yes	No	No	No	No	No
2052	HM0819	Male	Malay	2018	26	Single	Secondary	Private	Rural	No	Yes	No	Yes	Yes	No	No	No	No	No
2053	HM0820	Female	Malay	2018	23	Others	Secondary	Private	Rural	No	No	No	No	Yes	No	No	No	No	No
2054	HM0821	Male	Chinese	2019	35	Married	Secondary	Self-employed	Rural	No	No	No	No	Yes	No	No	No	No	No
2055	HM0823	Male	Malay	2020	29	Single	Secondary	Private	Rural	No	Yes	No	No	Yes	No	No	No	No	No
2056	HM0824	Male	Malay	2021	53	Single	Primary	Private	Rural	No	No	No	No	Yes	No	No	No	No	No
2057	HM0825	Male	Indian	2020	37	Married	Primary	Private	Rural	No	Yes	No	No	No	No	No	No	No	No
2058	HM0826	Male	Chinese	2018	40	Others	Secondary	Unemployed	Urban	Yes	Yes	Yes	No	Yes	No	No	No	No	No
2059	HM0827	Male	Malay	2019	56	Married	Secondary	Unemployed	Rural	Yes	Yes	No	No	No	No	No	No	No	No
2060	HM0828	Male	Malay	2019	62	Others	No formal education	Others	Rural	No	Yes	No	No	No	No	No	No	No	No
2061	HM0829	Male	Malay	2020	28	Single	Tertiary	Private	Rural	Yes	Yes	Yes	Yes	No	No	No	No	No	No
2062	HM0830	Male	Malay	2020	34	Single	Secondary	Private	Rural	No	Yes	Yes	No	No	No	No	No	No	No
2063	HM0834	Male	Malay	2018	50	Married	Tertiary	Self-employed	Rural	Yes	Yes	Yes	Yes	Yes	No	No	No	No	No
2064	HM0835	Male	Malay	2018	19	Single	Secondary	Private	Rural	No	Yes	Yes	Yes	Yes	No	No	No	No	No
2065	HM0836	Male	Malay	2020	37	Others	No formal education	Others	Rural	No	Yes	No	No	No	No	No	No	No	No
2066	HM0837	Male	Malay	2021	36	Others	Secondary	Private	Rural	No	Yes	No	No	No	No	No	No	No	No
2067	HM0838	Male	Malay	2018	21	Single	Secondary	Private	Rural	No	Yes	No	No	No	No	No	No	No	No
2068	HM0839	Male	Malay	2020	26	Others	Secondary	Self-employed	Rural	No	No	No	No	Yes	No	No	No	Yes	No
2069	HM0840	Female	Chinese	2018	38	Married	Secondary	Unemployed	Rural	No	No	Yes	No	No	No	No	No	No	No
2070	HM0841	Female	Chinese	2018	58	Single	Secondary	Self-employed	Rural	No	No	Yes	No	No	No	No	No	No	No
2071	HM0843	Male	Chinese	2020	72	Married	Tertiary	Others	Rural	No	No	Yes	No	No	No	No	No	No	No
2072	HM0846	Male	Malay	2018	23	Single	Secondary	Unemployed	Rural	No	Yes	No	No	No	No	No	No	No	No
2073	HM0847	Male	Malay	2018	33	Single	Secondary	Private	Rural	No	Yes	No	No	Yes	No	No	No	Yes	No
2074	HM0848	Male	Malay	2019	36	Others	No formal education	Private	Rural	No	Yes	No	No	No	No	No	No	No	No
2075	HM0849	Male	Indian	2021	32	Married	Secondary	Private	Rural	Yes	No	Yes	No	Yes	No	No	No	No	No
2076	HM0850	Male	Chinese	2020	52	Married	Secondary	Unemployed	Rural	No	No	Yes	No	No	No	No	No	No	No
2077	HM0851	Male	Malay	2018	41	Married	Tertiary	Private	Urban	No	Yes	No	No	No	No	No	No	No	No
2078	HM0852	Male	Malay	2020	22	Single	Tertiary	Unemployed	Rural	No	Yes	No	No	No	No	No	No	No	No
2079	HM0853	Male	Chinese	2018	53	Married	Secondary	Private	Rural	No	Yes	No	No	No	No	No	No	No	No
2080	HM0854	Male	Malay	2020	33	Single	Primary	Private	Rural	Yes	Yes	No	No	No	No	No	No	Yes	No
2081	HM0856	Male	Malay	2020	21	Single	Tertiary	Government	Rural	Yes	Yes	No	No	No	No	No	No	No	No
2082	HM0857	Male	Malay	2020	31	Single	Tertiary	Self-employed	Rural	Yes	Yes	Yes	No	No	No	No	No	No	No
2083	HM0858	Female	Malay	2020	32	Married	Secondary	Private	Rural	No	Yes	No	No	No	No	No	No	No	No
2084	HM0860	Male	Malay	2019	24	Married	Tertiary	Unemployed	Rural	Yes	Yes	No	No	No	No	No	No	No	No
2085	HM0861	Male	Malay	2018	23	Others	Secondary	Private	Rural	No	No	No	No	Yes	No	No	No	No	No
2086	HM0862	Male	Malay	2019	32	Single	Secondary	Private	Rural	No	No	Yes	Yes	Yes	Yes	No	No	Yes	No
2087	HM0863	Male	Malay	2021	40	Others	Secondary	Others	Rural	Yes	Yes	No	No	Yes	No	No	No	Yes	No

No	Patient ID	Gender	Ethnic group	Year of diagnosis	Age	Marital status	Education level	Occupation	Place of residence	History of psy illness	Tobacco	Alcohol	Cannabis	ATS	Inhalant	Sedative	Hallucinogens	Opioids	Kratom
2088	HM0864	Male	Malay	2021	22	Single	Secondary	Private	Rural	No	Yes	No	No	Yes	No	No	No	No	No
2089	HM0865	Male	Malay	2018	23	Single	Secondary	Private	Rural	No	Yes	No	No	Yes	No	No	No	No	No
2090	HM0866	Male	Indian	2018	24	Single	Secondary	Unemployed	Rural	No	Yes	No	No	Yes	No	No	No	No	No
2091	HM0867	Male	Malay	2019	26	Single	Tertiary	Self-employed	Rural	Yes	Yes	Yes	Yes	No	No	No	No	No	No
2092	HM0868	Male	Malay	2019	21	Single	Tertiary	Unemployed	Rural	No	Yes	No	No	No	No	No	No	No	No
2093	HM0869	Male	Malay	2021	52	Others	Primary	Private	Rural	Yes	Yes	No	No	No	No	No	No	No	No
2094	HM0871	Male	Malay	2018	42	Single	Secondary	Private	Urban	Yes	Yes	No	No	Yes	No	No	No	Yes	No
2095	HM0872	Male	Malay	2019	67	Married	Primary	Others	Rural	No	Yes	No	No	No	No	No	No	No	No
2096	HM0873	Male	Chinese	2018	59	Married	Tertiary	Others	Rural	Yes	Yes	Yes	No	No	No	No	No	No	No
2097	HM0874	Male	Malay	2018	21	Single	Secondary	Private	Rural	Yes	Yes	No	No	No	No	No	No	No	No
2098	HM0875	Male	Chinese	2020	45	Single	No formal education	Private	Urban	Yes	Yes	No	Yes	No	No	No	No	No	No
2099	HM0876	Male	Malay	2020	47	Others	No formal education	Unemployed	Rural	Yes	Yes	Yes	No	Yes	No	No	No	No	No
2100	HM0877	Male	Chinese	2018	37	Single	Secondary	Private	Rural	Yes	Yes	No	No	No	No	No	No	No	No
2101	HM0879	Male	Malay	2019	37	Married	Tertiary	Others	Rural	No	Yes	No	No	No	No	No	No	No	No
2102	HM0880	Male	Indian	2020	41	Married	Tertiary	Government	Urban	No	Yes	No	No	No	No	No	No	No	No
2103	HM0881	Female	Malay	2019	20	Single	Tertiary	Private	Rural	No	Yes	No	No	No	No	No	No	No	No
2104	HM0882	Male	Malay	2020	23	Single	Secondary	Private	Rural	No	Yes	Yes	No	No	No	No	No	No	No
2105	HM0883	Male	Chinese	2018	58	Others	Secondary	Self-employed	Rural	No	Yes	Yes	No	No	No	No	No	No	No
2106	HM0884	Male	Malay	2018	54	Single	No formal education	Private	Rural	No	Yes	No	No	No	No	No	No	No	No
2107	HM0885	Male	Malay	2020	38	Married	Tertiary	Private	Rural	No	Yes	No	No	No	No	No	No	No	No
2108	HM0886	Male	Chinese	2018	38	Single	Secondary	Private	Rural	No	Yes	Yes	No	Yes	No	No	No	No	No
2109	HM0888	Male	Others	2018	19	Single	Secondary	Self-employed	Rural	No	No	Yes	Yes	No	No	No	No	Yes	No
2110	HM0889	Female	Malay	2020	46	Single	Secondary	Unemployed	Rural	No	No	No	No	No	No	No	No	Yes	No
2111	HM0890	Male	Malay	2021	62	Single	Secondary	Unemployed	Rural	No	No	Yes	No	No	No	No	No	No	No
2112	HM0891	Male	Malay	2020	21	Single	Tertiary	Private	Rural	No	Yes	No	No	No	No	No	No	No	No
2113	HM0892	Male	Malay	2019	51	Married	Tertiary	Others	Rural	No	Yes	No	No	No	No	No	No	No	No
2114	HM0893	Male	Malay	2018	61	Married	Secondary	Private	Rural	No	Yes	No	No	No	No	No	No	No	No
2115	HM0894	Male	Indian	2018	25	Single	No formal education	Private	Rural	No	No	Yes	No	No	No	No	No	No	No
2116	HM0895	Male	Malay	2018	27	Single	Tertiary	Others	Rural	No	Yes	No	No	No	No	No	No	No	No
2117	HM0896	Male	Malay	2021	32	Single	Tertiary	Others	Rural	No	Yes	No	No	No	No	No	No	No	No
2118	HM0897	Male	Malay	2020	31	Others	No formal education	Others	Rural	No	No	No	No	Yes	No	No	No	No	No
2119	HM0898	Male	Malay	2020	21	Single	Secondary	Private	Rural	No	Yes	No	No	No	No	No	No	No	No
2120	HM0899	Male	Indian	2018	35	Married	Secondary	Self-employed	Rural	No	Yes	Yes	No	Yes	No	No	No	No	No
2121	HM0900	Male	Malay	2020	31	Married	Tertiary	Government	Rural	No	Yes	No	No	No	No	No	No	No	No
2122	HM0901	Male	Indian	2019	39	Single	Primary	Private	Rural	No	Yes	No	No	Yes	No	No	No	Yes	No
2123	HM0903	Male	Chinese	2020	68	Married	Secondary	Others	Rural	No	No	Yes	No	No	No	No	No	No	No
2124	HM0904	Male	Chinese	2018	52	Others	Secondary	Self-employed	Rural	Yes	Yes	Yes	No	No	No	No	No	No	No

No	Patient ID	Gender	Ethnic group	Year of diagnosis	Age	Marital status	Education level	Occupation	Place of residence	History of psy illness	Tobacco	Alcohol	Cannabis	ATS	Inhalant	Sedative	Hallucinogens	Opioids	Kratom
2125	HM0905	Male	Malay	2020	24	Single	No formal education	Self-employed	Rural	Yes	Yes	No	No	No	No	No	No	No	No
2126	HM0907	Male	Malay	2019	33	Married	Secondary	Others	Rural	No	Yes	No	No	No	No	No	No	No	No
2127	HM0908	Male	Malay	2020	27	Single	Primary	Others	Rural	No	Yes	No	No	No	No	No	No	No	No
2128	HM0910	Female	Chinese	2019	35	Married	Tertiary	Unemployed	Rural	Yes	No	Yes	No	No	No	No	No	No	No
2129	HM0911	Male	Malay	2018	60	Married	Secondary	Private	Rural	No	Yes	No	No	No	No	No	No	No	No
2130	HM0912	Male	Chinese	2020	63	Married	No formal education	Private	Rural	No	Yes	Yes	No	No	No	No	No	No	No
2131	HM0913	Male	Malay	2019	57	Married	Secondary	Self-employed	Rural	No	Yes	No	No	No	No	No	No	No	No
2132	HM0914	Male	Chinese	2020	45	Others	Secondary	Private	Rural	No	Yes	No	No	No	No	No	No	No	No
2133	HM0915	Female	Malay	2020	21	Single	Tertiary	Others	Urban	No	Yes	No	No	No	No	No	No	No	No
2134	HM0918	Male	Malay	2019	60	Married	No formal education	Others	Rural	Yes	Yes	No	No	No	No	No	No	No	No
2135	HM0919	Male	Indian	2020	47	Others	Secondary	Unemployed	Rural	No	Yes	Yes	No	No	No	No	No	No	No
2136	HM0920	Male	Indian	2018	57	Married	Secondary	Private	Rural	No	No	Yes	No	No	No	No	No	No	No
2137	HM0921	Male	Malay	2018	23	Single	Secondary	Unemployed	Rural	No	No	No	Yes	Yes	No	No	No	Yes	No
2138	HM0922	Male	Malay	2020	40	Married	Tertiary	Government	Urban	No	Yes	No	No	No	No	No	No	No	No
2139	HM0923	Male	Chinese	2020	40	Single	Secondary	Unemployed	Rural	No	Yes	Yes	Yes	Yes	No	No	No	Yes	No
2140	HM0924	Male	Malay	2019	52	Married	No formal education	Private	Rural	Yes	Yes	No	No	No	No	No	No	No	No
2141	HM0925	Male	Malay	2020	36	Married	Tertiary	Private	Rural	No	Yes	No	No	No	No	No	No	No	No
2142	HM0926	Male	Malay	2018	19	Single	Primary	Unemployed	Rural	No	Yes	No	No	No	No	No	No	No	No
2143	HM0927	Male	Malay	2018	35	Others	Secondary	Unemployed	Rural	No	No	No	No	Yes	No	No	No	Yes	No
2144	HM0928	Female	Malay	2019	33	Others	Secondary	Government	Urban	No	Yes	Yes	No	No	No	No	No	No	No
2145	HM0929	Male	Malay	2020	27	Single	Secondary	Unemployed	Rural	No	Yes	Yes	Yes	Yes	No	No	No	No	Yes
2146	HM0930	Male	Malay	2019	60	Married	Secondary	Others	Rural	No	Yes	No	No	No	No	No	No	No	No
2147	HM0931	Male	Malay	2021	46	Married	Secondary	Self-employed	Rural	No	Yes	No	No	No	No	No	No	No	No
2148	HM0932	Male	Malay	2020	24	Single	Secondary	Unemployed	Rural	No	No	Yes	No	Yes	No	No	No	No	No
2149	HM0933	Female	Chinese	2020	25	Single	Tertiary	Government	Rural	No	No	Yes	No	No	No	No	No	No	No
2150	HM0935	Male	Malay	2019	25	Others	Tertiary	Private	Rural	Yes	Yes	No	Yes	No	No	No	No	No	No
2151	HM0936	Male	Malay	2018	26	Married	Tertiary	Private	Rural	No	Yes	No	No	No	No	No	No	No	No
2152	HM0937	Female	Malay	2019	25	Others	Secondary	Unemployed	Rural	Yes	Yes	No	No	Yes	No	No	No	No	No
2153	HM0938	Male	Malay	2019	39	Married	Secondary	Self-employed	Rural	Yes	Yes	Yes	No	Yes	No	No	No	Yes	No
2154	HM0939	Male	Malay	2020	24	Single	Secondary	Private	Rural	No	Yes	No	Yes	No	No	No	No	Yes	No
2155	HM0940	Female	Chinese	2018	33	Others	Primary	Unemployed	Urban	No	Yes	No	No	Yes	No	No	No	No	No
2156	HM0941	Male	Malay	2018	38	Married	Tertiary	Government	Urban	No	Yes	No	No	No	No	No	No	No	No
2157	HM0942	Male	Malay	2020	24	Single	Secondary	Private	Rural	Yes	Yes	No	No	No	No	No	No	No	No
2158	HM0943	Male	Malay	2018	24	Single	Secondary	Private	Rural	Yes	Yes	No	No	No	No	No	No	No	No
2159	HM0944	Male	Malay	2019	38	Married	Tertiary	Government	Rural	No	Yes	No	No	No	No	No	No	No	No
2160	HM0945	Female	Malay	2019	35	Others	Secondary	Private	Rural	No	No	Yes	No	No	No	No	No	No	No
2161	HM0946	Male	Malay	2020	25	Others	Tertiary	Government	Rural	No	Yes	No	No	No	No	No	No	No	No
2162	HM0947	Male	Malay	2020	33	Others	Secondary	Private	Rural	No	Yes	No	No	No	No	No	No	No	No
2163	HM0948	Female	Indian	2021	24	Single	Tertiary	Unemployed	Rural	Yes	No	Yes	No	No	No	No	No	No	No
2164	HM0949	Male	Malay	2019	58	Others	Secondary	Unemployed	Rural	No	No	Yes	No	No	No	No	No	No	No

No	Patient ID	Gender	Ethnic group	Year of diagnosis	Age	Marital status	Education level	Occupation	Place of residence	History of psy illness	Tobacco	Alcohol	Cannabis	ATS	Inhalant	Sedative	Hallucinogens	Opioids	Kratom
2165	HM0950	Male	Malay	2020	55	Married	Tertiary	Others	Rural	No	Yes	No	No	No	No	No	No	No	No
2166	HM0951	Male	Malay	2021	63	Single	Primary	Unemployed	Rural	Yes	Yes	Yes	No	No	No	No	No	No	No
2167	HM0952	Male	Malay	2019	22	Single	Secondary	Private	Urban	No	Yes	No	Yes	No	No	No	No	No	No
2168	HM0953	Male	Malay	2020	32	Single	Secondary	Private	Rural	Yes	Yes	Yes	No	No	No	No	No	No	No
2169	HM0954	Male	Malay	2020	30	Single	Secondary	Unemployed	Rural	No	Yes	No	No	No	No	No	No	No	No
2170	HM0956	Male	Malay	2021	36	Single	Secondary	Private	Rural	No	No	Yes	Yes	No	No	No	No	No	No
2171	HM0957	Male	Indian	2019	21	Others	Primary	Private	Rural	No	Yes	Yes	No	No	No	No	No	No	No
2172	HM0958	Male	Malay	2019	31	Married	Tertiary	Private	Urban	No	Yes	No	No	No	No	No	No	No	Yes
2173	HM0959	Male	Chinese	2020	51	Single	Secondary	Unemployed	Rural	Yes	Yes	No	No	No	No	No	No	No	No
2174	HM0960	Male	Malay	2018	36	Married	Secondary	Private	Rural	No	Yes	No	No	No	No	No	No	No	No
2175	HM0961	Male	Indian	2018	53	Married	Secondary	Private	Rural	No	Yes	Yes	No	No	No	No	Yes	No	No
2176	HM0962	Male	Malay	2019	33	Married	Secondary	Government	Rural	No	Yes	No	No	No	No	No	No	No	No
2177	HM0964	Male	Malay	2018	27	Single	Tertiary	Government	Rural	No	Yes	No	Yes	No	No	No	No	No	No
2178	HM0965	Female	Chinese	2018	38	Others	Secondary	Private	Rural	No	Yes	Yes	No	No	No	No	No	No	No
2179	HM0965	Female	Chinese	2018	40	Others	Secondary	Private	Rural	No	Yes	Yes	No	No	No	No	No	No	No
2180	HM0966	Male	Malay	2021	35	Single	Secondary	Others	Rural	No	Yes	No	No	No	No	No	No	No	No
2181	HM0967	Male	Malay	2018	29	Others	No formal education	Others	Rural	No	Yes	No	No	No	No	No	No	No	No
2182	HM0969	Male	Malay	2018	34	Married	No formal education	Private	Rural	Yes	Yes	No	No	Yes	No	No	No	No	No
2183	HM0970	Male	Others	2020	30	Single	Secondary	Private	Rural	No	Yes	No	No	Yes	No	No	No	No	No
2184	HM0971	Male	Malay	2018	53	Married	Secondary	Private	Rural	No	Yes	No	No	No	No	No	No	No	No
2185	HM0972	Male	Malay	2020	26	Married	Secondary	Unemployed	Rural	No	Yes	No	Yes	No	No	No	No	No	No
2186	HM0973	Male	Malay	2020	34	Married	Primary	Private	Rural	No	Yes	No	No	Yes	No	No	No	No	No
2187	HM0974	Male	Malay	2020	24	Single	Tertiary	Private	Urban	No	Yes	Yes	No	No	No	No	No	No	No
2188	HM0975	Male	Malay	2020	62	Married	Secondary	Private	Rural	Yes	Yes	No	No	No	No	No	No	No	No
2189	HM0976	Male	Others	2018	35	Single	Secondary	Private	Rural	No	Yes	No	No	Yes	No	No	No	No	No
2190	HM0977	Male	Indian	2020	51	Single	Secondary	Unemployed	Rural	No	Yes	No	No	Yes	No	No	No	Yes	No
2191	HM0978	Female	Malay	2020	25	Single	Secondary	Unemployed	Rural	No	Yes	No	No	No	No	No	No	No	No
2192	HM0979	Male	Malay	2018	33	Married	Tertiary	Private	Rural	No	Yes	No	No	No	No	No	No	No	No
2193	HM0980	Male	Chinese	2019	49	Single	No formal education	Unemployed	Rural	Yes	Yes	No	No	No	No	No	No	No	No
2194	HM0981	Male	Malay	2021	41	Others	Secondary	Private	Rural	No	Yes	No	No	No	No	No	No	No	No
2195	HM0982	Male	Malay	2020	29	Single	Secondary	Private	Rural	No	Yes	Yes	No	Yes	No	No	No	No	No
2196	HM0983	Male	Malay	2020	24	Single	Secondary	Private	Rural	No	Yes	Yes	No	Yes	No	No	No	No	No
2197	HM0984	Male	Indian	2019	25	Single	Secondary	Private	Rural	No	Yes	Yes	No	No	No	No	No	No	No
2198	HM0985	Male	Others	2019	24	Others	Tertiary	Others	Rural	No	Yes	No	No	No	No	No	No	No	No
2199	HM0986	Male	Malay	2021	61	Single	Secondary	Unemployed	Rural	No	Yes	Yes	No	No	No	No	No	No	No
2200	HM0988	Male	Chinese	2018	35	Single	Primary	Unemployed	Rural	Yes	Yes	No	No	Yes	No	No	No	No	No
2201	HM0989	Male	Malay	2020	44	Married	Secondary	Self-employed	Rural	No	Yes	No	No	No	No	No	No	No	No
2202	HM0990	Male	Malay	2019	60	Others	No formal education	Self-employed	Rural	Yes	Yes	No	No	No	No	No	No	No	No
2203	HM0991	Female	Malay	2019	31	Others	Secondary	Private	Urban	No	No	Yes	Yes	Yes	No	No	No	No	No
2204	HM0992	Male	Malay	2019	35	Single	Tertiary	Self-employed	Rural	No	Yes	No	No	No	No	No	No	No	No

No	Patient ID	Gender	Ethnic group	Year of diagnosis	Age	Marital status	Education level	Occupation	Place of residence	History of psy illness	Tobacco	Alcohol	Cannabis	ATS	Inhalant	Sedative	Hallucinogens	Opioids	Kratom
2205	HM0993	Male	Malay	2020	28	Single	Tertiary	Private	Rural	No	Yes	No	No	No	No	No	No	No	No
2206	HM0994	Male	Malay	2018	27	Single	Secondary	Self-employed	Rural	No	Yes	Yes	No	Yes	No	No	No	No	No
2207	HM0996	Male	Indian	2019	58	Married	No formal education	Unemployed	Rural	No	Yes	No	No	Yes	No	No	No	No	No
2208	HM0997	Male	Chinese	2018	49	Others	Secondary	Unemployed	Urban	Yes	Yes	No	No	No	No	No	No	No	No
2209	HM0998	Male	Malay	2021	32	Single	Primary	Self-employed	Rural	Yes	No	No	No	Yes	No	No	No	Yes	No
2210	HM0999	Male	Chinese	2020	26	Single	Tertiary	Private	Urban	No	Yes	Yes	No	Yes	No	No	No	No	No
2211	HM1000	Male	Malay	2021	22	Single	Tertiary	Unemployed	Rural	No	Yes	No	No	No	No	No	No	No	No
2212	HM1001	Male	Malay	2019	24	Others	Secondary	Private	Urban	No	Yes	No	No	No	No	No	No	No	No
2213	HM1002	Male	Indian	2020	27	Married	Primary	Unemployed	Rural	No	Yes	Yes	No	No	No	No	No	No	No
2214	HM1003	Male	Malay	2018	33	Married	Secondary	Government	Rural	No	Yes	No	No	No	No	No	No	No	No
2215	HM1004	Male	Malay	2019	61	Married	No formal education	Private	Rural	No	Yes	No	No	No	No	No	No	No	No
2216	HM1005	Male	Chinese	2019	20	Single	Tertiary	Unemployed	Urban	No	Yes	Yes	No	No	No	No	No	No	No
2217	HM1006	Male	Indian	2018	35	Married	Secondary	Private	Rural	No	Yes	No	No	No	No	No	No	No	No
2218	HM1007	Male	Malay	2019	20	Single	Tertiary	Unemployed	Rural	Yes	Yes	No	No	No	No	No	No	No	No
2219	HM1008	Male	Malay	2019	42	Single	No formal education	Private	Rural	No	Yes	No	No	No	No	No	No	No	No
2220	HM1009	Female	Malay	2018	25	Married	No formal education	Unemployed	Rural	No	Yes	Yes	No	Yes	No	No	No	No	No
2221	HM1010	Male	Chinese	2019	56	Single	Primary	Unemployed	Rural	No	Yes	No	No	No	No	No	No	No	No
2222	HM1011	Female	Malay	2018	33	Others	Secondary	Unemployed	Rural	Yes	No	No	No	Yes	No	No	No	Yes	No
2223	HM1012	Male	Malay	2021	25	Single	Secondary	Private	Rural	No	Yes	No	No	No	No	No	No	No	No
2224	HM1013	Male	Chinese	2019	37	Single	Secondary	Private	Rural	No	No	Yes	No	No	No	No	No	No	No
2225	HM1014	Male	Malay	2019	21	Single	Secondary	Private	Urban	No	Yes	No	Yes	Yes	No	No	No	No	No
2226	HM1015	Male	Malay	2019	23	Single	Tertiary	Unemployed	Rural	No	Yes	Yes	No	Yes	No	No	No	No	No
2227	HM1016	Male	Indian	2021	45	Others	Primary	Private	Urban	Yes	Yes	Yes	No	No	No	No	No	No	No
2228	HM1017	Female	Malay	2021	31	Single	Secondary	Private	Rural	No	Yes	Yes	No	Yes	No	No	No	No	No
2229	HM1018	Female	Malay	2019	32	Married	Secondary	Private	Rural	Yes	Yes	No	No	No	No	No	No	No	No
2230	HM1019	Male	Indian	2021	31	Single	Tertiary	Private	Rural	Yes	Yes	Yes	No	No	No	No	No	No	No
2231	HM1020	Male	Malay	2021	24	Single	Secondary	Private	Rural	No	No	No	Yes	Yes	No	No	No	Yes	No
2232	HM1021	Male	Indian	2018	24	Single	Secondary	Private	Rural	Yes	No	Yes	No	No	No	No	No	No	No
2233	HM1022	Male	Malay	2019	23	Single	Primary	Private	Urban	No	Yes	No	No	Yes	No	No	No	Yes	No
2234	HM1023	Female	Chinese	2020	56	Single	Tertiary	Unemployed	Rural	No	Yes	No	No	No	No	No	No	No	No
2235	HM1024	Male	Indian	2021	46	Others	No formal education	Private	Rural	No	No	Yes	No	No	No	No	No	No	No
2236	HM1025	Male	Malay	2020	34	Single	Secondary	Unemployed	Rural	No	Yes	No	Yes	Yes	Yes	No	No	No	Yes
2237	HM1026	Male	Malay	2020	37	Married	Secondary	Unemployed	Urban	Yes	Yes	No	No	Yes	No	No	No	No	No
2238	HM1027	Male	Malay	2019	46	Married	Tertiary	Others	Rural	No	Yes	No	No	No	No	No	No	No	No
2239	HM1028	Male	Malay	2021	22	Single	Secondary	Private	Rural	Yes	Yes	No	No	No	No	No	No	No	No
2240	HM1030	Male	Malay	2019	21	Single	Tertiary	Private	Rural	No	Yes	No	No	No	No	No	No	No	No
2241	HM1031	Male	Malay	2020	29	Single	Tertiary	Private	Rural	No	Yes	No	No	No	No	No	No	No	No
2242	HM1032	Male	Malay	2020	45	Others	Secondary	Others	Rural	No	Yes	No	No	No	No	No	No	No	No

No	Patient ID	Gender	Ethnic group	Year of diagnosis	Age	Marital status	Education level	Occupation	Place of residence	History of psy illness	Tobacco	Alcohol	Cannabis	ATS	Inhalant	Sedative	Hallucinogens	Opioids	Kratom
2243	HM1033	Male	Malay	2019	40	Married	No formal education	Private	Rural	No	Yes	No	No	Yes	No	No	No	No	No
2244	HM1034	Female	Chinese	2018	23	Single	Tertiary	Unemployed	Rural	No	Yes	Yes	Yes	No	No	No	No	No	No
2245	HM1035	Male	Indian	2021	39	Others	Secondary	Self-employed	Rural	No	Yes	Yes	No	No	No	No	No	No	No
2246	HM1036	Male	Malay	2019	20	Single	Tertiary	Unemployed	Rural	No	Yes	No	No	Yes	No	No	No	No	No
2247	HM1037	Male	Malay	2020	26	Single	Secondary	Self-employed	Rural	Yes	Yes	No	No	No	No	No	No	No	No
2248	HM1038	Male	Malay	2020	25	Single	Secondary	Others	Rural	No	Yes	No	No	Yes	Yes	No	No	Yes	No
2249	HM1039	Male	Malay	2020	23	Single	Tertiary	Unemployed	Rural	Yes	No	Yes	No	Yes	No	No	No	No	No
2250	HM1040	Male	Malay	2019	22	Single	Tertiary	Others	Rural	No	Yes	No	Yes	No	No	No	No	No	No
2251	HM1041	Male	Malay	2021	28	Single	Tertiary	Government	Rural	No	Yes	No	Yes	No	No	No	No	No	No
2252	HM1042	Male	Malay	2021	42	Single	Secondary	Unemployed	Rural	No	Yes	No	No	No	No	No	No	Yes	No
2253	HM1043	Male	Malay	2019	22	Single	Tertiary	Private	Rural	No	Yes	No	No	No	No	No	No	No	No
2254	HM1044	Male	Malay	2018	26	Single	Secondary	Private	Rural	No	Yes	No	No	Yes	No	No	No	Yes	No
2255	HM1045	Male	Others	2019	24	Single	Secondary	Private	Rural	No	Yes	Yes	No	No	No	No	No	No	No
2256	HM1046	Male	Malay	2018	23	Single	Secondary	Private	Rural	No	Yes	Yes	No	No	No	No	No	No	No
2257	HM1047	Male	Chinese	2020	32	Single	Tertiary	Government	Rural	No	No	Yes	No	No	No	No	No	No	No
2258	HM1048	Male	Malay	2018	20	Others	Secondary	Private	Urban	No	Yes	Yes	No	No	No	No	No	No	No
2259	HM1049	Male	Malay	2019	53	Others	No formal education	Unemployed	Rural	No	Yes	No	No	No	No	No	No	No	No
2260	HM1050	Male	Malay	2018	33	Others	Secondary	Private	Urban	No	Yes	No	No	No	No	No	Yes	No	No
2261	HM1051	Female	Malay	2019	22	Single	Tertiary	Others	Rural	Yes	Yes	No	No	No	No	No	No	No	No
2262	HM1052	Male	Malay	2019	52	Married	Secondary	Unemployed	Rural	No	Yes	No	No	No	No	No	No	No	No
2263	HM1053	Female	Malay	2018	15	Single	Secondary	Unemployed	Rural	No	Yes	No	No	No	No	No	No	No	No
2264	HM1054	Male	Malay	2020	19	Others	Tertiary	Unemployed	Rural	No	Yes	No	No	No	No	No	No	No	No
2265	HM1055	Female	Malay	2020	20	Others	Tertiary	Unemployed	Urban	No	Yes	Yes	No	Yes	No	No	No	No	No
2266	HM1056	Female	Malay	2020	17	Single	Secondary	Unemployed	Rural	No	Yes	No	No	No	No	No	No	No	No
2267	HM1058	Male	Malay	2021	21	Single	Secondary	Unemployed	Rural	Yes	Yes	No	No	No	No	No	No	No	No
2268	HM1059	Male	Malay	2021	20	Single	Tertiary	Unemployed	Rural	No	No	No	No	Yes	No	No	No	No	No
2269	HM1060	Female	Malay	2021	17	Single	Secondary	Unemployed	Rural	No	Yes	No	No	No	No	No	No	No	No
2270	HM1061	Male	Malay	2021	17	Single	Tertiary	Unemployed	Rural	No	Yes	No	No	No	No	No	No	No	No
2271	HM1062	Female	Malay	2021	15	Single	Secondary	Unemployed	Rural	No	Yes	No	No	No	No	No	No	No	No
2272	HM1063	Female	Malay	2021	19	Single	Tertiary	Unemployed	Rural	No	Yes	No	No	No	No	No	No	No	No
2273	HM1064	Male	Malay	2020	19	Single	Primary	Unemployed	Rural	No	No	No	No	Yes	No	No	No	No	No
2274	HM1065	Male	Malay	2021	15	Single	Secondary	Others	Rural	No	Yes	No	No	Yes	No	No	No	No	No
2275	HM1066	Female	Chinese	2018	54	Married	Primary	Private	Rural	No	No	Yes	No	No	No	No	No	No	No
2276	HM1068	Male	Chinese	2021	40	Single	Secondary	Private	Rural	Yes	No	Yes	No	No	No	No	No	No	No
2277	HM1070	Male	Malay	2019	17	Single	Secondary	Unemployed	Rural	No	Yes	No	No	No	No	No	No	No	No
2278	HM1080	Male	Malay	2018	46	Married	Secondary	Self-employed	Urban	No	No	No	No	No	No	No	No	Yes	No
2279	HM1093	Male	Malay	2018	48	Married	Secondary	Self-employed	Rural	No	No	No	No	No	No	No	No	Yes	No
2280	HM1116	Male	Malay	2021	62	Others	No formal education	Unemployed	Rural	No	No	No	No	No	No	No	No	Yes	No
2281	HM1117	Male	Malay	2020	49	Single	No formal education	Others	Rural	No	No	No	No	Yes	No	No	No	Yes	No
2282	HM1120	Male	Chinese	2020	57	Married	Secondary	Unemployed	Rural	No	Yes	No	No	Yes	No	No	No	Yes	No

No	Patient ID	Gender	Ethnic group	Year of diagnosis	Age	Marital status	Education level	Occupation	Place of residence	History of psy illness	Tobacco	Alcohol	Cannabis	ATS	Inhalant	Sedative	Hallucinogens	Opioids	Kratom
2283	HM1122	Male	Malay	2019	44	Married	Tertiary	Government	Rural	No	No	No	No	No	No	No	No	Yes	No
2284	HM1159	Female	Malay	2021	24	Single	Tertiary	Unemployed	Rural	Yes	Yes	Yes	No	No	No	No	No	No	No
2285	HM1160	Male	Chinese	2019	35	Single	Primary	Private	Rural	No	Yes	Yes	No	No	No	No	No	No	No
2286	HM1161	Male	Malay	2019	40	Single	Secondary	Private	Rural	No	Yes	Yes	No	No	No	No	No	No	No
2287	HM1163	Male	Chinese	2020	39	Others	Tertiary	Government	Rural	No	Yes	Yes	No	No	No	No	No	No	No
2288	HM1165	Male	Chinese	2018	41	Single	Tertiary	Private	Rural	Yes	No	Yes	No	No	No	No	No	No	No
2289	HM1166	Male	Chinese	2018	58	Single	Tertiary	Private	Rural	No	No	Yes	No	No	No	No	No	No	No
2290	HM1167	Male	Indian	2020	32	Married	Primary	Private	Rural	No	Yes	Yes	No	No	No	No	No	No	No
2291	HM1168	Male	Malay	2018	21	Married	Secondary	Private	Rural	No	Yes	No	No	No	No	No	No	No	No
2292	HM1171	Male	Malay	2020	43	Married	Secondary	Unemployed	Rural	No	Yes	No	No	No	No	No	No	No	No
2293	HM1172	Male	Others	2021	25	Single	Secondary	Private	Urban	No	Yes	No	Yes	No	No	No	No	No	No
2294	HM1173	Male	Chinese	2018	54	Married	Primary	Self-employed	Rural	No	Yes	Yes	No	No	No	No	No	No	No
2295	HM1175	Male	Malay	2019	20	Others	Secondary	Private	Rural	No	Yes	No	No	Yes	No	No	No	No	No
2296	HM1177	Female	Chinese	2021	25	Others	Secondary	Private	Rural	No	No	Yes	No	No	No	Yes	No	No	No
2297	HM1178	Male	Malay	2018	20	Single	Primary	Private	Rural	No	Yes	No	No	No	No	No	No	No	No
2298	HM1179	Male	Indian	2019	25	Married	Secondary	Others	Rural	No	No	Yes	No	No	No	No	No	No	No
2299	HM1180	Male	Chinese	2021	51	Married	No formal education	Private	Rural	Yes	No	Yes	No	No	No	No	No	No	No
2300	HM1181	Male	Malay	2020	30	Married	Secondary	Private	Rural	No	Yes	No	No	No	No	No	No	No	No
2301	HM1182	Female	Indian	2021	25	Single	Secondary	Private	Rural	No	No	Yes	No	No	No	No	No	No	No
2302	HM1183	Female	Malay	2019	24	Single	Secondary	Private	Rural	No	Yes	No	No	No	No	No	No	No	No
2303	HM1184	Female	Chinese	2019	76	Married	Primary	Private	Rural	No	No	Yes	No	No	No	No	No	No	No
2304	HM1185	Female	Chinese	2018	32	Married	Secondary	Private	Rural	No	No	Yes	No	No	No	No	No	No	No
2305	HM1186	Male	Malay	2021	43	Married	Tertiary	Private	Rural	No	Yes	No	No	No	No	No	No	No	No
2306	HM1188	Male	Malay	2018	24	Single	Secondary	Others	Rural	No	Yes	No	No	No	No	No	No	No	No
2307	HM1189	Male	Malay	2018	53	Married	No formal education	Others	Urban	No	Yes	No	No	No	No	Yes	No	No	No
2308	HM1190	Male	Malay	2019	23	Married	Secondary	Private	Rural	No	Yes	No	Yes	No	No	No	No	No	No
2309	HM1191	Male	Indian	2019	44	Married	Tertiary	Private	Urban	No	Yes	No	No	No	No	No	No	No	No
2310	HM1192	Male	Malay	2020	24	Others	No formal education	Others	Rural	No	Yes	No	No	No	No	No	No	No	No
2311	HM1193	Female	Malay	2021	31	Married	Secondary	Private	Rural	No	No	No	No	Yes	No	No	No	No	No
2312	HM1196	Male	Malay	2021	25	Single	Secondary	Private	Rural	No	Yes	Yes	Yes	No	No	No	No	No	No
2313	HM1198	Female	Malay	2020	22	Single	Tertiary	Private	Rural	No	No	No	No	Yes	No	No	No	No	No
2314	HM1199	Female	Chinese	2019	27	Single	Tertiary	Private	Rural	No	No	Yes	No	No	No	No	No	No	No
2315	HM1200	Male	Malay	2019	32	Single	Secondary	Private	Rural	Yes	Yes	No	No	No	No	No	No	No	No
2316	HM1201	Male	Chinese	2018	27	Married	Secondary	Private	Rural	No	Yes	Yes	No	No	No	No	No	No	No
2317	HM1202	Male	Malay	2018	24	Single	Secondary	Private	Rural	No	Yes	No	No	No	No	No	No	No	No
2318	HM1203	Male	Malay	2019	39	Single	No formal education	Private	Rural	Yes	Yes	No	No	No	No	No	No	No	No
2319	HM1204	Male	Malay	2018	24	Single	Secondary	Private	Rural	No	Yes	No	No	Yes	No	Yes	No	No	Yes

No	Patient ID	Gender	Ethnic group	Year of diagnosis	Age	Marital status	Education level	Occupation	Place of residence	History of psy illness	Tobacco	Alcohol	Cannabis	ATS	Inhalant	Sedative	Hallucinogens	Opioids	Kratom
2320	HM1205	Male	Malay	2020	52	Married	Secondary	Private	Rural	No	Yes	Yes	No	No	No	No	No	No	No
2321	HM1206	Male	Chinese	2020	51	Married	No formal education	Others	Rural	No	Yes	Yes	No	No	No	No	No	No	No
2322	HM1208	Male	Chinese	2020	41	Single	Primary	Private	Rural	No	Yes	No	No	No	No	No	No	No	No
2323	HM1209	Female	Chinese	2018	30	Married	Secondary	Private	Rural	No	No	Yes	No	No	No	No	No	No	No
2324	HM1210	Male	Malay	2020	34	Single	Secondary	Unemployed	Rural	No	Yes	No	No	No	No	No	No	No	No
2325	HM1212	Female	Malay	2019	25	Others	Tertiary	Private	Rural	No	Yes	No	No	No	No	No	No	No	No
2326	HM1213	Male	Chinese	2021	33	Others	Secondary	Private	Rural	No	No	Yes	No	No	No	No	No	No	No
2327	HM1214	Male	Chinese	2020	62	Married	Secondary	Others	Rural	No	No	Yes	No	No	No	No	No	No	No
2328	HM1215	Female	Malay	2019	55	Others	No formal education	Unemployed	Rural	No	Yes	No	No	No	No	No	No	No	No
2329	HM1216	Male	Malay	2021	43	Married	Tertiary	Private	Rural	No	Yes	No	Yes	No	No	No	No	No	Yes
2330	HM1217	Male	Malay	2018	25	Single	Secondary	Government	Rural	No	Yes	No	No	No	No	No	No	No	No
2331	HM1221	Male	Malay	2018	27	Single	Secondary	Private	Rural	Yes	Yes	Yes	No	No	No	No	No	No	No
2332	HM1222	Male	Indian	2020	52	Married	Secondary	Unemployed	Rural	No	Yes	Yes	No	No	No	No	No	No	No
2333	HM1223	Male	Malay	2021	28	Single	Tertiary	Private	Rural	No	Yes	Yes	No	No	No	No	No	No	No
2334	HM1224	Female	Malay	2020	22	Married	Tertiary	Self-employed	Rural	No	Yes	Yes	No	No	No	No	No	No	No
2335	HM1225	Male	Chinese	2021	43	Married	Secondary	Unemployed	Rural	Yes	Yes	Yes	No	No	No	No	No	No	No
2336	HM1226	Male	Malay	2020	37	Single	Secondary	Unemployed	Rural	No	Yes	No	No	Yes	No	No	No	Yes	No
2337	HM1227	Male	Malay	2021	22	Single	Secondary	Others	Rural	No	Yes	No	No	No	No	No	No	No	No
2338	HM1229	Male	Malay	2020	48	Married	Secondary	Others	Rural	No	Yes	No	No	No	No	No	No	No	No
2339	HM1230	Male	Malay	2021	30	Single	Secondary	Others	Rural	No	Yes	No	No	Yes	No	No	No	No	No
2340	HM1232	Male	Malay	2021	23	Single	Secondary	Unemployed	Rural	No	Yes	No	No	No	No	No	No	No	No
2341	HM1233	Female	Malay	2019	30	Married	Secondary	Others	Rural	No	Yes	Yes	No	No	No	No	No	No	No
2342	HM1234	Male	Malay	2020	62	Married	Tertiary	Private	Rural	No	Yes	No	No	No	No	No	No	No	No
2343	HM1235	Male	Chinese	2020	32	Single	Tertiary	Unemployed	Rural	Yes	Yes	No	No	No	No	No	No	No	No
2344	HM1236	Female	Malay	2020	52	Single	Tertiary	Private	Rural	No	Yes	No	No	No	No	No	No	No	No
2345	HM1237	Male	Chinese	2020	28	Single	Tertiary	Private	Urban	No	No	Yes	No	No	No	No	No	No	No
2346	HM1238	Male	Chinese	2020	58	Single	No formal education	Self-employed	Rural	No	No	No	No	No	No	No	No	Yes	No
2347	HM1239	Male	Malay	2020	43	Married	Secondary	Private	Rural	No	Yes	No	No	No	No	No	No	No	No
2348	HM1240	Female	Malay	2020	39	Others	Tertiary	Private	Urban	No	No	No	No	Yes	No	No	No	No	No
2349	HM1242	Male	Malay	2019	29	Single	Secondary	Private	Rural	No	Yes	No	Yes	Yes	No	No	No	No	Yes
2350	HM1243	Male	Malay	2019	53	Married	Secondary	Private	Rural	No	Yes	No	No	No	No	No	No	No	No
2351	HM1244	Female	Malay	2019	59	Others	Secondary	Others	Rural	No	Yes	No	No	No	No	No	No	No	No
2352	HM1246	Female	Others	2021	37	Others	No formal education	Private	Rural	No	No	Yes	No	No	No	No	No	No	No
2353	HM1247	Male	Malay	2020	25	Single	Tertiary	Private	Rural	No	Yes	No	No	No	No	No	No	No	No
2354	HM1249	Male	Malay	2019	28	Others	Tertiary	Private	Rural	Yes	Yes	No	No	No	No	No	No	No	No
2355	HM1250	Male	Malay	2021	25	Single	Secondary	Self-employed	Rural	No	Yes	No	No	No	No	No	No	No	No

No	Patient ID	Gender	Ethnic group	Year of diagnosis	Age	Marital status	Education level	Occupation	Place of residence	History of psy illness	Tobacco	Alcohol	Cannabis	ATS	Inhalant	Sedative	Hallucinogens	Opioids	Kratom
2356	HM1251	Male	Malay	2019	51	Single	Secondary	Private	Rural	No	Yes	No	No	No	No	No	No	Yes	No
2357	HM1252	Male	Malay	2019	53	Married	Tertiary	Self-employed	Rural	No	No	No	No	Yes	No	No	No	No	No
2358	HM1253	Female	Malay	2021	24	Single	Tertiary	Private	Urban	No	Yes	No	No	No	No	No	No	No	No
2359	HM1254	Male	Malay	2021	40	Married	Secondary	Government	Rural	No	Yes	No	No	No	No	No	No	No	No
2360	HM1256	Male	Malay	2021	22	Single	Tertiary	Unemployed	Rural	Yes	Yes	No	No	No	No	No	No	No	No
2361	HM1257	Male	Malay	2018	42	Married	No formal education	Self-employed	Rural	No	Yes	Yes	Yes	Yes	No	No	No	No	No
2362	HM1258	Male	Chinese	2021	49	Married	Secondary	Private	Urban	No	Yes	No	No	No	No	No	No	No	No
2363	HM1259	Male	Malay	2018	20	Single	Secondary	Private	Rural	No	Yes	Yes	No	No	No	No	No	No	No
2364	HM1260	Male	Indian	2020	53	Others	Secondary	Private	Rural	Yes	No	Yes	No	No	No	No	No	No	No
2365	HM1261	Male	Malay	2021	59	Married	Secondary	Others	Urban	Yes	Yes	No	No	No	No	No	No	No	No
2366	HM1262	Male	Malay	2018	24	Single	Secondary	Private	Rural	No	Yes	No	No	Yes	Yes	No	No	No	No
2367	HM1263	Male	Malay	2020	31	Others	Secondary	Unemployed	Rural	No	Yes	No	No	No	No	No	No	No	No
2368	HP0002	Male	Malay	2020	29	Others	Secondary	Private	Urban	Yes	Yes	Yes	Yes	Yes	No	No	No	No	No
2369	HP0004	Male	Malay	2020	27	Single	Tertiary	Others	Urban	Yes	Yes	No	Yes	No	No	No	No	No	No
2370	HP0005	Male	Malay	2020	45	Married	Tertiary	Self-employed	Urban	Yes	No	No	No	Yes	No	No	No	No	No
2371	HP0006	Male	Malay	2021	20	Single	Secondary	Private	Urban	Yes	Yes	No	No	Yes	No	No	No	No	No
2372	HP0007	Male	Malay	2019	39	Married	Tertiary	Self-employed	Urban	Yes	Yes	No	Yes	No	No	No	No	No	No
2373	HP0009	Male	Malay	2018	26	Single	Tertiary	Private	Urban	Yes	Yes	No	No	No	No	No	No	No	No
2374	HP0010	Female	Malay	2019	27	Single	Tertiary	Private	Urban	Yes	Yes	No	Yes	No	No	No	No	No	No
2375	HP0011	Male	Malay	2019	28	Single	Tertiary	Unemployed	Urban	Yes	Yes	Yes	No	Yes	No	No	No	No	No
2376	HP0012	Female	Malay	2018	23	Single	Secondary	Private	Urban	Yes	Yes	Yes	Yes	No	No	No	No	No	No
2377	HP0014	Male	Malay	2021	21	Single	Tertiary	Private	Urban	Yes	Yes	Yes	Yes	No	No	No	No	No	No
2378	HP0015	Male	Malay	2019	35	Single	No formal education	Others	Urban	Yes	No	Yes	No	Yes	No	No	No	No	No
2379	HP0016	Male	Malay	2021	24	Single	Tertiary	Private	Urban	Yes	No	No	No	Yes	No	Yes	No	No	No
2380	HP0018	Male	Malay	2020	22	Single	Tertiary	Unemployed	Urban	Yes	No	No	Yes	No	No	No	No	No	No
2381	HP0019	Male	Malay	2019	30	Single	Tertiary	Unemployed	Urban	Yes	Yes	No	No	No	No	No	No	No	No
2382	HP0020	Male	Malay	2020	24	Others	Tertiary	Private	Urban	Yes	No	No	Yes	No	No	No	No	No	No
2383	HP0023	Female	Malay	2019	22	Single	Secondary	Private	Urban	No	Yes	Yes	Yes	Yes	No	No	No	No	No
2384	HP0024	Female	Malay	2021	36	Single	Tertiary	Self-employed	Urban	Yes	Yes	No	No	No	No	No	No	No	No
2385	HP0025	Male	Malay	2021	28	Single	Tertiary	Private	Urban	Yes	Yes	Yes	No	No	No	No	Yes	No	No
2386	HP0027	Male	Malay	2020	34	Married	No formal education	Government	Rural	Yes	No	No	No	Yes	No	No	No	No	Yes
2387	HP0028	Male	Malay	2021	34	Married	Secondary	Self-employed	Urban	Yes	Yes	No	Yes	No	No	No	No	No	Yes
2388	HP0029	Male	Malay	2019	34	Single	No formal education	Private	Urban	Yes	Yes	No	No	No	Yes	No	No	No	No
2389	HP0030	Male	Malay	2020	29	Single	Tertiary	Private	Rural	Yes	Yes	No	Yes	No	No	No	No	No	No
2390	HP0031	Male	Malay	2019	24	Single	Tertiary	Unemployed	Urban	Yes	No	No	Yes	No	No	No	No	No	No
2391	HP0033	Male	Indian	2019	29	Single	No formal education	Unemployed	Urban	Yes	Yes	Yes	Yes	Yes	No	No	No	No	No

No	Patient ID	Gender	Ethnic group	Year of diagnosis	Age	Marital status	Education level	Occupation	Place of residence	History of psy illness	Tobacco	Alcohol	Cannabis	ATS	Inhalant	Sedative	Hallucinogens	Opioids	Kratom
2392	HP0034	Male	Malay	2020	47	Married	No formal education	Government	Urban	Yes	Yes	No	Yes	No	No	No	No	No	No
2393	HP0035	Male	Malay	2020	26	Single	Secondary	Unemployed	Urban	Yes	Yes	No	No	No	No	No	No	No	No
2394	HP0036	Male	Malay	2018	32	Married	No formal education	Private	Urban	Yes	Yes	No	No	No	No	No	No	No	No
2395	HP0037	Male	Malay	2021	36	Single	Tertiary	Private	Urban	Yes	No	No	Yes	No	No	No	No	No	No
2396	HP0037	Male	Malay	2019	31	Single	Tertiary	Unemployed	Urban	Yes	Yes	No	No	No	No	No	No	Yes	No
2397	HP0040	Male	Malay	2020	27	Single	Tertiary	Private	Urban	Yes	Yes	No	Yes	No	No	No	No	No	No
2398	HP0041	Male	Malay	2019	30	Single	Secondary	Unemployed	Urban	Yes	Yes	No	Yes	No	No	No	No	No	No
2399	HP0042	Male	Malay	2018	27	Single	Tertiary	Private	Urban	Yes	Yes	No	Yes	No	No	No	No	No	Yes
2400	HP0045	Female	Malay	2019	25	Single	Tertiary	Unemployed	Urban	Yes	Yes	No	No	No	No	No	No	No	No
2401	HP0046	Female	Malay	2021	20	Single	Tertiary	Unemployed	Urban	Yes	Yes	Yes	Yes	No	No	No	No	No	No
2402	HP0048	Male	Malay	2019	30	Single	Tertiary	Self-employed	Urban	Yes	No	No	No	Yes	No	No	No	No	No
2403	HP0051	Male	Chinese	2019	45	Others	Tertiary	Self-employed	Urban	Yes	No	Yes	No	No	No	No	No	No	No
2404	HP0052	Male	Indian	2018	52	Single	Secondary	Unemployed	Urban	Yes	Yes	Yes	No	No	No	No	No	No	No
2405	HP0054	Male	Malay	2021	29	Married	Secondary	Others	Urban	No	Yes	No	No	No	No	No	No	No	No
2406	HP0055	Male	Malay	2019	21	Single	Tertiary	Unemployed	Urban	Yes	Yes	No	No	No	No	No	No	No	No
2407	HP0056	Male	Malay	2019	25	Single	Tertiary	Private	Rural	Yes	Yes	No	No	No	No	No	No	No	No
2408	HP0057	Male	Malay	2020	26	Single	Secondary	Unemployed	Urban	Yes	Yes	No	No	No	No	No	No	No	No
2409	HP0058	Male	Malay	2020	59	Married	No formal education	Government	Urban	No	Yes	No	No	No	No	No	No	No	No
2410	HP0059	Female	Malay	2019	24	Single	Tertiary	Private	Urban	No	No	Yes	No	No	No	No	No	No	No
2411	HP0060	Female	Malay	2020	35	Married	Secondary	Private	Urban	Yes	Yes	No	No	No	No	No	No	No	No
2412	HP0061	Female	Malay	2019	19	Single	No formal education	Private	Urban	No	Yes	Yes	No	No	No	No	No	No	No
2413	HP0062	Female	Malay	2021	30	Single	Tertiary	Private	Urban	Yes	Yes	No	No	No	No	No	No	No	No
2414	HP0066	Male	Chinese	2018	23	Single	Secondary	Private	Urban	Yes	Yes	No	No	No	No	No	No	No	No
2415	HP0067	Male	Malay	2019	34	Single	Tertiary	Government	Urban	Yes	Yes	Yes	No	No	No	No	No	No	No
2416	HP0068	Male	Others	2020	21	Others	Tertiary	Unemployed	Urban	Yes	No	Yes	No	No	No	No	No	No	No
2417	HP0069	Male	Chinese	2019	38	Others	Secondary	Private	Rural	Yes	Yes	Yes	No	No	No	No	No	No	No
2418	HP0070	Male	Malay	2019	50	Married	Secondary	Government	Urban	Yes	Yes	No	No	No	No	No	No	No	No
2419	HP0071	Male	Malay	2020	41	Married	Secondary	Self-employed	Rural	Yes	Yes	No	No	No	No	No	No	No	No
2420	HP0072	Male	Malay	2019	33	Married	Tertiary	Private	Urban	Yes	Yes	No	No	No	No	No	No	No	No
2421	HP0073	Male	Malay	2020	22	Single	Tertiary	Unemployed	Urban	Yes	Yes	No	No	No	No	No	No	No	No
2422	HP0075	Male	Indian	2019	59	Married	Tertiary	Self-employed	Urban	Yes	Yes	Yes	No	No	No	No	No	No	No
2423	HP0077	Female	Chinese	2019	25	Others	Tertiary	Government	Urban	Yes	Yes	Yes	No	No	No	No	No	No	No
2424	HP0078	Male	Malay	2019	34	Single	Secondary	Unemployed	Urban	Yes	Yes	No	No	No	No	No	No	No	No
2425	HP0080	Male	Malay	2020	36	Single	No formal education	Private	Urban	Yes	Yes	Yes	Yes	No	No	No	No	No	No
2426	HP0081	Male	Malay	2020	33	Single	Tertiary	Self-employed	Urban	Yes	No	Yes	Yes	No	No	No	No	No	No
2427	HP0082	Male	Malay	2019	34	Married	No formal education	Government	Urban	Yes	Yes	No	No	No	No	No	No	No	No
2428	HP0083	Male	Malay	2020	67	Married	Tertiary	Others	Urban	Yes	Yes	No	No	No	No	No	No	No	No
2429	HP0084	Male	Malay	2020	21	Single	Tertiary	Unemployed	Urban	Yes	Yes	No	No	No	No	No	No	No	No

No	Patient ID	Gender	Ethnic group	Year of diagnosis	Age	Marital status	Education level	Occupation	Place of residence	History of psy illness	Tobacco	Alcohol	Cannabis	ATS	Inhalant	Sedative	Hallucinogens	Opioids	Kratom
2430	HP0085	Male	Malay	2018	20	Single	Tertiary	Unemployed	Urban	Yes	Yes	No	No	No	No	No	No	No	No
2431	HP0086	Male	Malay	2019	30	Single	Tertiary	Unemployed	Urban	Yes	Yes	No	No	Yes	No	No	No	No	No
2432	HP0087	Male	Malay	2019	20	Single	Tertiary	Unemployed	Rural	Yes	Yes	No	No	No	No	No	No	No	No
2433	HP0088	Male	Malay	2019	24	Single	Secondary	Unemployed	Urban	Yes	Yes	No	No	No	No	No	No	No	No
2434	HP0089	Male	Malay	2020	26	Single	Tertiary	Unemployed	Rural	Yes	Yes	No	No	No	No	No	No	No	No
2435	HP0090	Female	Malay	2019	28	Single	Tertiary	Unemployed	Urban	Yes	Yes	No	No	No	No	No	No	No	No
2436	HP0091	Female	Malay	2019	23	Single	No formal education	Unemployed	Rural	Yes	Yes	Yes	No	No	No	No	No	No	No
2437	HP0092	Female	Malay	2019	20	Others	Secondary	Private	Urban	Yes	Yes	Yes	No	No	No	No	No	No	No
2438	HP0093	Female	Malay	2020	38	Single	Tertiary	Private	Urban	Yes	No	Yes	No	Yes	No	No	No	No	No
2439	HP0094	Male	Malay	2019	30	Single	Secondary	Private	Urban	Yes	Yes	Yes	No	No	No	No	No	No	No
2440	HP0095	Male	Indian	2019	52	Others	No formal education	Government	Urban	Yes	Yes	Yes	No	No	No	No	No	No	No
2441	HP0096	Female	Malay	2020	22	Single	Tertiary	Private	Urban	Yes	Yes	Yes	Yes	No	No	No	No	No	No
2442	HP0097	Female	Malay	2019	20	Single	Tertiary	Unemployed	Urban	Yes	No	No	Yes	Yes	No	No	No	No	No
2443	HP0098	Male	Malay	2019	26	Others	Tertiary	Private	Urban	Yes	Yes	No	No	No	No	No	No	No	No
2444	HP0099	Male	Chinese	2021	42	Others	No formal education	Private	Urban	Yes	No	Yes	No	No	No	No	No	No	No
2445	HP0100	Male	Malay	2020	31	Married	Tertiary	Private	Urban	Yes	Yes	No	No	No	No	No	No	No	No
2446	HP0102	Male	Malay	2021	18	Single	Secondary	Unemployed	Urban	Yes	Yes	No	No	No	No	No	No	No	No
2447	HP0105	Female	Chinese	2019	61	Married	No formal education	Private	Urban	Yes	No	Yes	No	No	No	No	No	No	No
2448	HP0108	Male	Malay	2018	22	Single	Secondary	Unemployed	Urban	Yes	Yes	No	No	No	No	No	No	No	No
2449	HP0109	Male	Chinese	2018	64	Married	No formal education	Others	Rural	Yes	Yes	Yes	No	No	No	No	No	No	No
2450	HP0110	Female	Chinese	2019	18	Single	Tertiary	Unemployed	Urban	Yes	No	Yes	No	No	No	No	No	No	No
2451	HP0111	Male	Malay	2019	18	Single	Tertiary	Unemployed	Rural	Yes	Yes	No	No	No	No	No	No	No	No
2452	HP0118	Female	Malay	2019	42	Single	Tertiary	Private	Urban	Yes	Yes	No	No	No	No	No	No	No	No
2453	HP0119	Male	Others	2020	41	Others	No formal education	Unemployed	Rural	Yes	Yes	No	No	No	No	No	No	No	No
2454	HP0120	Male	Malay	2019	38	Others	No formal education	Unemployed	Rural	Yes	Yes	No	No	Yes	No	No	No	No	No
2455	HP0121	Male	Indian	2019	48	Single	No formal education	Private	Urban	Yes	No	Yes	No	No	No	No	No	No	No
2456	HP0122	Female	Malay	2018	38	Others	No formal education	Unemployed	Rural	Yes	Yes	No	No	Yes	No	No	No	No	No
2457	HP0123	Male	Malay	2019	27	Single	Secondary	Self-employed	Urban	Yes	Yes	No	No	Yes	No	No	No	No	No
2458	HP0124	Male	Malay	2019	18	Single	Tertiary	Unemployed	Urban	Yes	No	No	Yes	No	No	No	No	No	Yes
2459	HP0126	Male	Malay	2020	19	Single	Tertiary	Unemployed	Urban	Yes	No	No	Yes	No	No	No	No	No	No
2460	HP0127	Female	Malay	2020	22	Single	Tertiary	Unemployed	Urban	Yes	Yes	No	No	No	No	No	No	No	No
2461	HP0129	Male	Chinese	2019	29	Single	Tertiary	Unemployed	Rural	Yes	No	Yes	No	Yes	No	No	No	No	No
2462	HP0130	Male	Malay	2019	49	Single	Secondary	Unemployed	Urban	Yes	Yes	No	No	No	No	No	No	No	No

No	Patient ID	Gender	Ethnic group	Year of diagnosis	Age	Marital status	Education level	Occupation	Place of residence	History of psy illness	Tobacco	Alcohol	Cannabis	ATS	Inhalant	Sedative	Hallucinogens	Opioids	Kratom
2463	HP0136	Male	Chinese	2019	42	Single	No formal education	Private	Urban	Yes	No	Yes	No	No	No	No	No	No	No
2464	HP0140	Male	Malay	2019	20	Single	Tertiary	Unemployed	Rural	Yes	Yes	No	No	No	No	No	No	No	No
2465	HP0141	Female	Malay	2020	19	Others	Tertiary	Unemployed	Rural	Yes	Yes	No	No	No	No	No	No	No	No
2466	HP0142	Female	Malay	2019	23	Others	Tertiary	Unemployed	Urban	Yes	Yes	Yes	No	No	No	No	No	No	No
2467	HP0143	Female	Malay	2019	21	Single	Tertiary	Unemployed	Urban	Yes	Yes	No	No	No	No	No	No	No	No
2468	HP0145	Male	Malay	2020	31	Others	Secondary	Private	Rural	Yes	Yes	Yes	No	No	No	No	No	No	No
2469	HP0146	Male	Malay	2019	53	Married	No formal education	Government	Urban	Yes	Yes	No	No	No	No	No	No	No	No
2470	HP0148	Female	Malay	2019	37	Married	Tertiary	Self-employed	Urban	Yes	Yes	No	No	No	No	No	No	No	No
2471	HP0149	Female	Malay	2018	30	Married	Tertiary	Private	Urban	Yes	Yes	No	No	No	No	No	No	No	No
2472	HPP0001	Female	Chinese	2019	50	Married	No formal education	Private	Urban	No	Yes	Yes	No	No	No	No	No	No	No
2473	HPP0002	Female	Malay	2020	19	Single	Secondary	Others	Urban	No	Yes	No	No	No	No	No	No	No	No
2474	HPP0003	Female	Indian	2019	59	Others	No formal education	Unemployed	Urban	No	No	No	No	No	No	Yes	No	No	No
2475	HPP0004	Female	Chinese	2021	19	Single	Secondary	Others	Urban	Yes	No	Yes	No	No	No	No	No	No	No
2476	HPP0007	Female	Others	2019	21	Single	Secondary	Unemployed	Urban	No	Yes	Yes	Yes	No	No	No	No	No	No
2477	HPP0008	Female	Malay	2019	33	Others	No formal education	Unemployed	Rural	No	No	No	No	Yes	No	No	No	No	No
2478	HPP0010	Male	Malay	2018	38	Single	Tertiary	Unemployed	Urban	No	Yes	No	No	Yes	No	No	No	No	No
2479	HPP0011	Male	Malay	2019	27	Single	Primary	Unemployed	Urban	No	Yes	No	Yes	Yes	No	No	No	No	Yes
2480	HPP0013	Male	Malay	2019	23	Single	No formal education	Others	Urban	No	Yes	No	Yes	No	No	No	No	No	No
2481	HPP0014	Male	Indian	2020	43	Single	Secondary	Private	Urban	Yes	Yes	Yes	No	Yes	No	No	No	Yes	No
2482	HPP0016	Male	Malay	2018	28	Married	Secondary	Government	Urban	No	Yes	No	Yes	Yes	No	No	No	No	Yes
2483	HPP0017	Male	Chinese	2020	42	Single	No formal education	Others	Urban	No	No	No	Yes	No	No	No	No	Yes	No
2484	HPP0019	Male	Chinese	2018	49	Others	Primary	Private	Urban	No	No	No	No	Yes	No	Yes	No	No	No
2485	HPP0020	Male	Chinese	2018	39	Others	No formal education	Self-employed	Urban	Yes	No	No	No	Yes	No	No	No	No	No
2486	HPP0022	Male	Malay	2019	23	Single	Primary	Private	Urban	No	No	No	Yes	Yes	No	No	No	Yes	No
2487	HPP0024	Male	Malay	2019	22	Single	Secondary	Private	Urban	No	No	No	No	Yes	No	No	No	No	No
2488	HPP0025	Male	Malay	2020	27	Single	Tertiary	Private	Urban	No	Yes	No	Yes	No	No	No	No	No	Yes
2489	HPP0026	Male	Malay	2018	30	Married	Secondary	Self-employed	Urban	No	Yes	Yes	No	Yes	No	No	No	No	Yes
2490	HPP0027	Male	Malay	2020	21	Single	Tertiary	Private	Urban	No	Yes	No	Yes	No	No	No	No	No	No
2491	HPP0028	Male	Malay	2018	36	Married	No formal education	Others	Urban	No	Yes	No	No	No	No	No	No	No	Yes
2492	HPP0029	Male	Malay	2018	29	Married	Secondary	Private	Urban	No	Yes	No	No	No	No	No	No	No	No
2493	HPP0030	Female	Chinese	2018	43	Married	No formal education	Self-employed	Urban	No	Yes	Yes	No	No	No	No	No	No	No
2494	HPP0031	Female	Malay	2021	32	Single	Tertiary	Private	Urban	Yes	No	Yes	No	No	No	No	No	No	No

No	Patient ID	Gender	Ethnic group	Year of diagnosis	Age	Marital status	Education level	Occupation	Place of residence	History of psy illness	Tobacco	Alcohol	Cannabis	ATS	Inhalant	Sedative	Hallucinogens	Opioids	Kratom
2495	HPP0032	Female	Malay	2019	21	Single	No formal education	Private	Urban	No	Yes	No	No	No	No	No	No	No	No
2496	HPP0040	Male	Chinese	2018	49	Others	Primary	Private	Urban	No	Yes	Yes	Yes	No	No	No	No	No	No
2497	HPP0043	Male	Indian	2021	39	Others	No formal education	Unemployed	Urban	No	No	No	Yes	No	No	No	No	No	No
2498	HPP0048	Male	Chinese	2020	44	Others	No formal education	Others	Urban	Yes	Yes	No	No	No	No	No	No	No	No
2499	HPP0051	Male	Malay	2018	38	Others	No formal education	Others	Urban	No	No	No	No	No	No	No	No	No	Yes
2500	HPP0052	Male	Indian	2020	48	Married	No formal education	Unemployed	Urban	No	No	Yes	No	No	No	No	No	No	No
2501	HPP0056	Female	Malay	2018	22	Others	Primary	Others	Urban	No	No	No	No	Yes	No	No	No	No	No
2502	HPP0063	Male	Malay	2019	28	Single	Tertiary	Government	Urban	No	Yes	No	No	Yes	No	No	No	No	No
2503	HPP0064	Male	Chinese	2018	42	Single	No formal education	Unemployed	Urban	Yes	Yes	No	No	Yes	No	No	No	No	No
2504	HPP0065	Male	Malay	2018	46	Single	Primary	Private	Urban	No	Yes	No	No	No	No	No	No	No	No
2505	HPP0066	Male	Chinese	2018	43	Single	No formal education	Private	Urban	No	No	No	No	Yes	No	No	No	Yes	No
2506	HPP0067	Male	Chinese	2019	53	Others	Tertiary	Private	Urban	No	Yes	Yes	No	Yes	No	No	No	No	No
2507	HPP0068	Male	Indian	2020	47	Married	No formal education	Unemployed	Urban	No	Yes	No	No	No	No	No	No	No	No
2508	HPP0070	Male	Malay	2018	33	Single	Tertiary	Unemployed	Urban	No	Yes	No	Yes	Yes	No	No	No	No	Yes
2509	HPP0074	Male	Malay	2018	39	Others	No formal education	Private	Urban	Yes	Yes	No	No	Yes	No	No	No	No	Yes
2510	HPP0075	Male	Chinese	2019	49	Others	Primary	Unemployed	Urban	No	Yes	Yes	Yes	No	No	No	No	No	No
2511	HPP0077	Male	Chinese	2019	41	Married	Tertiary	Self-employed	Urban	No	Yes	No	Yes	Yes	No	Yes	No	No	No
2512	HPP0079	Male	Indian	2018	49	Single	Primary	Unemployed	Urban	No	Yes	Yes	Yes	Yes	No	No	No	Yes	No
2513	HPP0080	Female	Malay	2020	26	Married	Primary	Unemployed	Urban	No	Yes	No	No	Yes	No	No	No	No	No
2514	HPP0081	Female	Chinese	2021	22	Single	Secondary	Unemployed	Urban	No	No	Yes	Yes	No	No	No	No	No	No
2515	HPP0083	Female	Malay	2021	25	Others	Primary	Private	Urban	No	Yes	Yes	No	No	No	No	Yes	No	No
2516	HPP0085	Male	Malay	2018	31	Single	Primary	Unemployed	Urban	Yes	No	No	No	Yes	No	No	No	Yes	Yes
2517	HPP0087	Male	Malay	2020	25	Single	Primary	Unemployed	Urban	No	Yes	No	No	Yes	No	No	No	No	Yes
2518	HPP0089	Male	Malay	2018	26	Single	Secondary	Others	Urban	No	Yes	Yes	Yes	No	No	No	No	No	No
2519	HPP0090	Male	Chinese	2020	31	Single	Tertiary	Unemployed	Urban	Yes	No	No	Yes	Yes	No	No	No	No	No
2520	HPP0091	Male	Indian	2021	33	Married	No formal education	Others	Urban	Yes	No	No	No	No	No	No	No	Yes	No
2521	HPP0092	Male	Malay	2020	25	Single	Primary	Unemployed	Urban	No	Yes	No	Yes	Yes	No	No	No	Yes	Yes
2522	HPP0093	Male	Indian	2019	26	Single	Tertiary	Private	Urban	Yes	No	Yes	No	No	No	No	No	No	No

No	Patient ID	Gender	Ethnic group	Year of diagnosis	Age	Marital status	Education level	Occupation	Place of residence	History of psy illness	Tobacco	Alcohol	Cannabis	ATS	Inhalant	Sedative	Hallucinogens	Opioids	Kratom
2523	HPP0094	Male	Chinese	2020	40	Single	Secondary	Unemployed	Urban	No	Yes	No	No	Yes	No	No	No	Yes	No
2524	HPP0095	Male	Chinese	2021	32	Single	Primary	Private	Urban	No	Yes	Yes	No	No	No	No	No	No	No
2525	HPP0096	Male	Malay	2018	22	Single	Secondary	Private	Urban	No	Yes	No	No	Yes	No	Yes	No	No	No
2526	HPP0097	Male	Indian	2020	24	Married	Secondary	Private	Urban	No	No	Yes	No	No	No	No	No	No	No
2527	HPP0098	Male	Malay	2018	34	Others	Primary	Unemployed	Urban	Yes	Yes	No	Yes	Yes	No	No	No	Yes	No
2528	HPP0099	Male	Indian	2019	43	Others	Primary	Private	Urban	No	Yes	No	Yes	No	No	No	No	No	No
2529	HPP0100	Male	Malay	2018	28	Single	Secondary	Private	Urban	No	Yes	Yes	Yes	Yes	No	No	No	No	No
2530	HPP0101	Male	Malay	2021	32	Single	Secondary	Self-employed	Urban	No	No	No	No	Yes	No	No	No	Yes	No
2531	HPP0102	Male	Indian	2018	27	Single	Secondary	Private	Urban	No	Yes	Yes	Yes	Yes	No	No	No	No	No
2532	HPP0103	Male	Chinese	2020	27	Others	Secondary	Private	Urban	No	Yes	Yes	Yes	Yes	Yes	No	No	No	No
2533	HPP0104	Female	Others	2020	30	Single	No formal education	Unemployed	Urban	No	No	No	No	Yes	No	No	No	No	No
2534	HPP0107	Female	Malay	2020	22	Single	Tertiary	Unemployed	Urban	Yes	Yes	Yes	Yes	No	No	No	Yes	No	Yes
2535	HPP0108	Male	Malay	2020	48	Married	Primary	Self-employed	Urban	No	Yes	No	No	No	No	No	No	No	Yes
2536	HPP0109	Male	Indian	2019	25	Single	Primary	Unemployed	Urban	No	Yes	No	Yes	Yes	No	No	No	No	Yes
2537	HPP0110	Male	Malay	2019	23	Single	Primary	Unemployed	Urban	No	Yes	No	Yes	Yes	No	No	No	Yes	No
2538	HPP0111	Male	Malay	2021	29	Single	Secondary	Government	Urban	No	Yes	No	Yes	Yes	No	No	No	No	No
2539	HPP0112	Male	Malay	2019	54	Single	Secondary	Private	Urban	No	Yes	No	No	No	No	No	No	Yes	No
2540	HPP0113	Male	Malay	2019	32	Others	Tertiary	Private	Urban	No	Yes	Yes	No	No	No	No	No	No	No
2541	HPP0114	Male	Indian	2019	58	Married	No formal education	Others	Urban	No	No	Yes	No	No	No	No	No	No	No
2542	HPP0117	Male	Indian	2021	25	Single	Tertiary	Unemployed	Urban	No	Yes	Yes	Yes	No	No	No	No	No	No
2543	HPP0118	Male	Malay	2018	23	Single	Secondary	Unemployed	Urban	No	Yes	No	No	Yes	No	No	No	No	Yes
2544	HPP0120	Male	Chinese	2019	31	Single	Primary	Private	Urban	No	Yes	Yes	No	No	No	No	No	No	No
2545	HPP0121	Female	Indian	2018	31	Others	No formal education	Unemployed	Urban	No	Yes	Yes	No	Yes	No	No	No	Yes	No
2546	HPP0122	Female	Malay	2019	21	Single	Secondary	Unemployed	Urban	No	Yes	Yes	No	No	No	No	No	No	No
2547	HPP0123	Female	Indian	2018	27	Single	Secondary	Unemployed	Urban	No	No	No	No	Yes	No	No	No	No	No
2548	HPP0124	Male	Others	2020	30	Single	No formal education	Unemployed	Urban	Yes	No	No	No	Yes	No	No	No	No	No
2549	HPP0125	Male	Malay	2021	28	Single	Secondary	Unemployed	Urban	Yes	Yes	No	Yes	Yes	Yes	No	No	Yes	No
2550	HPP0126	Male	Malay	2019	19	Single	Primary	Self-employed	Urban	No	Yes	No	No	Yes	No	No	No	No	No
2551	HPP0127	Male	Malay	2018	32	Single	Secondary	Others	Urban	No	Yes	No	Yes	Yes	No	Yes	No	Yes	Yes
2552	HPP0128	Male	Chinese	2019	44	Single	Tertiary	Unemployed	Urban	No	Yes	No	No	Yes	No	No	No	No	No
2553	HPP0129	Male	Malay	2020	59	Married	Primary	Private	Urban	No	Yes	No	No	Yes	No	No	No	Yes	No
2554	HPP0131	Male	Malay	2021	27	Single	Primary	Private	Urban	No	Yes	No	No	Yes	No	No	No	No	Yes
2555	HPP0132	Male	Indian	2019	42	Married	Primary	Government	Urban	No	Yes	Yes	Yes	No	No	No	No	No	No
2556	HPP0135	Male	Malay	2019	28	Married	Secondary	Government	Urban	No	Yes	No	Yes	No	No	No	No	No	Yes
2557	HPP0138	Male	Malay	2019	26	Single	Secondary	Unemployed	Urban	No	Yes	No	No	Yes	Yes	No	No	No	No
2558	HPP0139	Male	Malay	2018	20	Single	Secondary	Unemployed	Urban	No	No	No	Yes	No	No	No	No	No	No

No	Patient ID	Gender	Ethnic group	Year of diagnosis	Age	Marital status	Education level	Occupation	Place of residence	History of psy illness	Tobacco	Alcohol	Cannabis	ATS	Inhalant	Sedative	Hallucinogens	Opioids	Kratom
2559	HPP0140	Male	Chinese	2021	37	Single	Primary	Unemployed	Urban	Yes	No	No	Yes	No	Yes	No	No	No	No
2560	HPP0141	Male	Malay	2021	34	Married	No formal education	Private	Urban	No	Yes	No	No	Yes	No	No	No	No	Yes
2561	HPP0145	Female	Indian	2020	34	Married	Primary	Unemployed	Urban	No	No	No	No	Yes	No	No	No	No	No
2562	HPP0146	Female	Malay	2019	23	Single	Secondary	Unemployed	Urban	No	No	Yes	Yes	No	No	No	No	No	No
2563	HPP0148	Male	Malay	2019	45	Others	No formal education	Unemployed	Urban	No	No	No	No	Yes	No	No	No	No	No
2564	HPP0149	Male	Chinese	2021	52	Married	No formal education	Unemployed	Urban	No	Yes	Yes	No	Yes	No	No	No	No	No
2565	HPP0151	Male	Malay	2020	44	Married	No formal education	Others	Rural	Yes	Yes	Yes	Yes	No	No	No	No	Yes	No
2566	HPP0152	Male	Malay	2021	25	Single	Primary	Unemployed	Urban	No	Yes	No	No	Yes	No	No	No	No	No
2567	HPP0154	Male	Others	2021	34	Single	Primary	Private	Urban	No	Yes	No	No	Yes	No	No	No	No	No
2568	HPP0156	Female	Malay	2020	25	Married	Secondary	Private	Urban	No	No	No	No	Yes	No	No	No	No	No
2569	HPP0157	Male	Chinese	2021	54	Others	No formal education	Others	Urban	Yes	Yes	No	No	Yes	No	No	No	Yes	No
2570	HPP0160	Male	Malay	2020	23	Single	Primary	Unemployed	Urban	No	Yes	No	No	No	No	No	No	No	No
2571	HPP0162	Male	Chinese	2018	42	Single	Primary	Unemployed	Urban	No	Yes	No	Yes	Yes	No	No	No	No	No
2572	HPP0163	Male	Chinese	2019	57	Married	No formal education	Private	Urban	No	Yes	Yes	Yes	No	No	No	No	No	No
2573	HPP0165	Male	Malay	2018	30	Others	Secondary	Unemployed	Urban	No	Yes	No	Yes	Yes	No	No	No	No	No
2574	HPP0167	Male	Malay	2019	37	Single	Secondary	Self-employed	Urban	Yes	Yes	Yes	No	No	No	No	No	Yes	No
2575	HPP0168	Male	Indian	2019	34	Single	Primary	Private	Urban	No	Yes	Yes	No	No	No	No	No	No	No
2576	HPP0169	Male	Chinese	2019	49	Married	Primary	Private	Urban	No	Yes	No	No	Yes	No	No	No	No	No
2577	HPP0171	Male	Indian	2018	21	Single	Secondary	Unemployed	Urban	No	No	No	No	Yes	No	No	No	Yes	No
2578	HPP0172	Male	Chinese	2021	54	Single	Primary	Private	Urban	No	Yes	Yes	No	No	No	No	No	Yes	No
2579	HPP0173	Male	Indian	2021	29	Others	Primary	Unemployed	Urban	No	Yes	Yes	Yes	Yes	No	No	No	No	No
2580	HPP0175	Male	Chinese	2019	42	Single	Primary	Private	Urban	No	Yes	No	No	Yes	No	No	No	No	No
2581	HPP0179	Male	Malay	2019	38	Single	No formal education	Unemployed	Rural	No	No	No	No	Yes	No	No	No	Yes	Yes
2582	HPP0180	Male	Malay	2020	22	Single	Secondary	Unemployed	Urban	No	Yes	Yes	Yes	No	No	No	No	No	No
2583	HPP0181	Male	Indian	2021	24	Single	Primary	Self-employed	Urban	No	Yes	No	Yes	No	No	No	No	No	No
2584	HPP0182	Male	Chinese	2019	55	Single	Primary	Unemployed	Urban	No	Yes	No	No	Yes	No	No	No	Yes	No
2585	HPP0183	Male	Indian	2019	56	Married	No formal education	Unemployed	Urban	Yes	No	Yes	No	No	No	No	No	Yes	No
2586	HPP0184	Male	Malay	2020	42	Single	Primary	Unemployed	Urban	No	No	No	No	No	Yes	No	No	No	No
2587	HPP0186	Male	Malay	2019	24	Single	Secondary	Unemployed	Urban	No	No	No	Yes	Yes	No	No	No	No	No
2588	HPP0188	Male	Malay	2019	54	Married	Secondary	Government	Urban	No	No	No	No	Yes	No	Yes	No	No	No

No	Patient ID	Gender	Ethnic group	Year of diagnosis	Age	Marital status	Education level	Occupation	Place of residence	History of psy illness	Tobacco	Alcohol	Cannabis	ATS	Inhalant	Sedative	Hallucinogens	Opioids	Kratom
2589	HPP0192	Male	Chinese	2020	53	Others	No formal education	Unemployed	Urban	No	No	No	No	No	No	No	No	Yes	No
2590	HPP0193	Male	Malay	2018	38	Single	Secondary	Others	Urban	No	Yes	No	No	No	No	No	No	No	Yes
2591	HPP0194	Male	Chinese	2019	54	Married	Primary	Private	Urban	No	Yes	Yes	Yes	No	No	No	No	Yes	No
2592	HPP0196	Male	Others	2018	20	Single	No formal education	Others	Urban	No	No	No	Yes	Yes	No	No	No	Yes	No
2593	HPP0198	Male	Malay	2019	30	Single	Secondary	Private	Urban	No	Yes	Yes	Yes	Yes	Yes	Yes	No	No	No
2594	HPP0199	Female	Chinese	2019	41	Married	Tertiary	Private	Urban	Yes	Yes	Yes	No	No	No	Yes	No	No	No
2595	HPP0201	Male	Indian	2020	43	Single	Primary	Unemployed	Urban	No	No	No	Yes	Yes	No	No	No	Yes	No
2596	HPP0202	Male	Indian	2020	32	Single	Secondary	Private	Urban	No	Yes	Yes	No	No	No	No	No	No	No
2597	HPP0203	Male	Indian	2020	29	Single	No formal education	Private	Urban	No	Yes	Yes	No	No	No	No	No	No	No
2598	HPP0204	Male	Indian	2018	41	Married	No formal education	Private	Urban	No	No	Yes	No	No	No	No	No	No	No
2599	HPP0205	Male	Indian	2018	49	Single	Primary	Unemployed	Urban	No	Yes	Yes	Yes	No	No	No	No	Yes	No
2600	HPP0206	Male	Malay	2020	42	Others	Primary	Unemployed	Urban	Yes	Yes	No	No	No	No	No	No	No	No
2601	HPP0207	Male	Malay	2019	38	Others	No formal education	Private	Urban	No	Yes	No	Yes	Yes	No	No	No	No	No
2602	HPP0208	Female	Others	2019	56	Married	No formal education	Private	Urban	No	No	No	No	Yes	No	No	No	No	No
2603	HPP0209	Male	Indian	2018	55	Married	Primary	Unemployed	Urban	No	No	Yes	No	No	No	No	No	No	No
2604	HPP0210	Male	Malay	2019	26	Single	Secondary	Unemployed	Urban	No	Yes	No	No	No	No	No	No	No	No
2605	HPP0211	Male	Indian	2019	24	Single	Primary	Private	Urban	Yes	No	Yes	No	No	No	No	No	No	No
2606	HPP0212	Male	Others	2021	28	Married	Secondary	Government	Urban	No	Yes	Yes	No	No	No	No	No	No	Yes
2607	HPP0213	Male	Malay	2018	35	Single	Secondary	Self-employed	Urban	No	No	No	No	Yes	No	Yes	No	No	No
2608	HPP0215	Male	Malay	2021	28	Single	Secondary	Private	Urban	No	No	No	Yes	No	No	No	No	No	No
2609	HPP0216	Male	Malay	2021	50	Others	No formal education	Unemployed	Urban	No	No	No	No	Yes	No	No	No	Yes	No
2610	HPP0217	Female	Others	2020	32	Married	No formal education	Private	Urban	No	No	No	Yes	No	No	No	No	No	No
2611	HPP0218	Male	Malay	2020	43	Single	Secondary	Private	Urban	No	Yes	Yes	No	No	No	No	No	Yes	Yes
2612	HPP0219	Female	Others	2020	21	Others	No formal education	Private	Urban	No	No	No	No	Yes	No	No	No	No	No
2613	HPP0221	Male	Malay	2020	53	Married	Secondary	Government	Urban	No	No	No	No	Yes	No	No	No	No	No
2614	HPP0222	Male	Malay	2021	33	Others	No formal education	Others	Urban	No	No	No	Yes	No	No	No	No	No	No
2615	HPP0223	Female	Malay	2018	27	Single	Primary	Private	Urban	No	Yes	No	No	Yes	No	No	No	No	No
2616	HPP0224	Male	Malay	2019	48	Others	Primary	Unemployed	Urban	No	No	No	Yes	Yes	No	No	No	No	No
2617	HPP0225	Male	Indian	2018	50	Single	Primary	Private	Urban	No	No	No	Yes	No	No	No	No	No	No
2618	HPP0226	Female	Chinese	2021	37	Others	Primary	Self-employed	Urban	Yes	Yes	No	No	No	No	No	No	No	No
2619	HPP0228	Male	Others	2021	19	Others	No formal education	Unemployed	Urban	No	Yes	No	No	Yes	No	No	No	No	No

No	Patient ID	Gender	Ethnic group	Year of diagnosis	Age	Marital status	Education level	Occupation	Place of residence	History of psy illness	Tobacco	Alcohol	Cannabis	ATS	Inhalant	Sedative	Hallucinogens	Opioids	Kratom
2620	HPP0230	Male	Indian	2021	38	Single	No formal education	Private	Urban	No	Yes	No	Yes	Yes	No	No	No	Yes	Yes
2621	HPP0231	Male	Chinese	2020	55	Others	No formal education	Unemployed	Urban	No	No	No	No	Yes	No	No	No	No	No
2622	HPP0233	Female	Indian	2021	32	Others	No formal education	Unemployed	Urban	No	No	No	Yes	Yes	Yes	No	No	Yes	No
2623	HPP0234	Male	Malay	2021	28	Single	Secondary	Self-employed	Urban	No	No	No	Yes	Yes	Yes	No	No	Yes	Yes
2624	HPP0236	Female	Malay	2019	28	Others	No formal education	Unemployed	Urban	No	No	No	No	Yes	No	No	No	No	No
2625	HPP0237	Male	Chinese	2019	43	Others	No formal education	Private	Urban	No	Yes	Yes	No	No	No	No	No	No	No
2626	HPP0238	Female	Malay	2019	34	Others	No formal education	Unemployed	Urban	No	No	No	No	Yes	No	No	No	No	No
2627	HPP0239	Male	Indian	2020	40	Married	Secondary	Private	Urban	No	Yes	Yes	No	No	No	No	No	No	No
2628	HPP0240	Male	Malay	2018	37	Others	Primary	Private	Urban	Yes	Yes	No	No	No	No	No	No	No	No
2629	HPP0241	Male	Indian	2019	23	Others	Tertiary	Unemployed	Urban	No	Yes	Yes	Yes	No	No	No	No	No	No
2630	HPP0244	Male	Malay	2021	27	Single	Primary	Private	Urban	No	No	No	No	Yes	No	No	No	Yes	Yes
2631	HPP0245	Male	Indian	2018	39	Single	Tertiary	Government	Urban	Yes	Yes	Yes	No	Yes	No	No	No	No	No
2632	HPP0246	Female	Chinese	2018	43	Others	Tertiary	Unemployed	Urban	No	Yes	Yes	No	No	No	No	No	No	No
2633	HPP0247	Male	Malay	2019	27	Single	Primary	Private	Urban	No	No	No	No	Yes	No	No	No	No	No
2634	HPP0248	Female	Malay	2018	21	Married	Tertiary	Private	Urban	Yes	Yes	No	No	Yes	No	No	No	No	No
2635	HPP0250	Male	Chinese	2019	56	Single	Primary	Others	Urban	Yes	No	No	No	Yes	No	No	No	No	No
2636	HPP0251	Female	Chinese	2019	32	Others	Primary	Private	Urban	No	No	Yes	No	Yes	No	No	No	No	No
2637	HPP0252	Male	Malay	2021	34	Others	Tertiary	Unemployed	Urban	Yes	No	No	No	Yes	No	No	No	No	No
2638	HPP0253	Female	Malay	2021	30	Single	Primary	Unemployed	Urban	No	Yes	No	Yes	Yes	No	No	No	No	No
2639	HPP0256	Male	Malay	2018	39	Single	Tertiary	Unemployed	Urban	Yes	Yes	Yes	Yes	Yes	No	No	No	No	No
2640	HPP0258	Female	Malay	2019	18	Single	Primary	Unemployed	Urban	No	Yes	No	No	Yes	No	No	No	No	No
2641	HPP0259	Male	Malay	2018	21	Single	Primary	Private	Urban	No	Yes	No	Yes	Yes	No	No	No	No	No
2642	HPP0260	Male	Malay	2018	28	Single	No formal education	Unemployed	Urban	No	No	No	Yes	Yes	No	No	No	Yes	Yes
2643	HPP0261	Male	Malay	2020	21	Single	Primary	Self-employed	Urban	No	Yes	No	No	Yes	No	No	No	No	No
2644	HPP0262	Male	Indian	2019	36	Single	Secondary	Private	Urban	No	No	Yes	No	No	No	No	No	No	No
2645	HPP0264	Male	Chinese	2018	38	Married	Primary	Self-employed	Urban	No	No	No	No	Yes	No	No	No	No	No
2646	HPP0265	Male	Malay	2018	34	Single	Primary	Unemployed	Urban	No	No	No	Yes	Yes	No	No	No	Yes	Yes
2647	HPP0266	Male	Indian	2019	23	Married	Primary	Private	Urban	No	Yes	Yes	No	No	No	No	No	No	No
2648	HPP0268	Male	Indian	2021	58	Married	Primary	Unemployed	Urban	Yes	Yes	Yes	No	No	No	No	No	No	No
2649	HPP0269	Male	Malay	2019	44	Single	No formal education	Unemployed	Urban	No	No	No	No	Yes	No	No	No	Yes	No
2650	HPP0271	Male	Others	2020	44	Married	No formal education	Private	Urban	Yes	No	No	Yes	No	No	No	No	No	No
2651	HPP0272	Male	Malay	2018	29	Single	Primary	Unemployed	Urban	No	No	No	No	Yes	No	No	No	No	No
2652	HPP0273	Male	Malay	2018	38	Single	Primary	Private	Urban	No	Yes	No	No	No	No	No	No	No	No
2653	HPP0276	Male	Chinese	2018	38	Others	Secondary	Self-employed	Urban	No	Yes	Yes	Yes	Yes	No	No	No	No	No

No	Patient ID	Gender	Ethnic group	Year of diagnosis	Age	Marital status	Education level	Occupation	Place of residence	History of psy illness	Tobacco	Alcohol	Cannabis	ATS	Inhalant	Sedative	Hallucinogens	Opioids	Kratom
2654	HPP0278	Female	Malay	2019	34	Married	No formal education	Unemployed	Urban	No	No	No	No	No	No	No	No	Yes	No
2655	HPP0280	Male	Indian	2020	49	Single	Secondary	Private	Urban	No	Yes	No	No	No	No	No	No	Yes	No
2656	HPP0281	Male	Chinese	2020	50	Single	Primary	Unemployed	Urban	No	Yes	No	No	Yes	No	No	No	Yes	No
2657	HPP0282	Male	Malay	2019	32	Single	Primary	Private	Urban	No	Yes	No	No	Yes	No	No	No	No	No
2658	HPP0283	Male	Malay	2020	44	Married	Primary	Private	Urban	No	No	No	No	No	No	No	No	Yes	No
2659	HPP0284	Male	Malay	2020	23	Single	Secondary	Unemployed	Urban	No	No	No	No	Yes	No	No	No	No	No
2660	HPP0287	Male	Malay	2021	28	Married	Secondary	Private	Urban	No	Yes	No	No	No	No	No	No	No	No
2661	HPP0290	Male	Malay	2018	22	Single	Secondary	Private	Urban	No	No	No	Yes	Yes	No	No	No	No	No
2662	HPP0291	Male	Malay	2020	36	Others	Secondary	Unemployed	Urban	No	No	No	No	No	No	No	No	No	No
2663	HPP0294	Female	Malay	2021	28	Married	Tertiary	Private	Urban	No	Yes	Yes	Yes	No	No	No	No	Yes	No
2664	HPP0295	Female	Chinese	2019	47	Single	Primary	Unemployed	Urban	Yes	Yes	Yes	No	Yes	No	No	No	No	No
2665	HPP0296	Male	Chinese	2020	52	Married	Secondary	Unemployed	Urban	No	Yes	No	Yes	No	No	No	No	No	No
2666	HPP0297	Male	Chinese	2019	49	Single	Secondary	Unemployed	Urban	No	No	No	No	No	No	No	No	Yes	No
2667	HPP0298	Male	Malay	2021	36	Married	Secondary	Private	Urban	No	Yes	No	Yes	Yes	No	No	No	No	Yes
2668	HPP0299	Male	Malay	2019	25	Others	Secondary	Unemployed	Urban	No	Yes	No	Yes	Yes	No	No	No	No	No
2669	HPP0300	Male	Malay	2020	25	Single	Secondary	Unemployed	Urban	No	Yes	No	No	No	No	No	No	No	Yes
2670	HPP0301	Male	Indian	2021	32	Others	Primary	Unemployed	Urban	No	Yes	Yes	Yes	No	No	No	No	No	No
2671	HPP0303	Male	Indian	2021	30	Married	Secondary	Government	Urban	No	Yes	No	Yes	No	No	No	No	No	No
2672	HPP0304	Male	Indian	2020	37	Others	Primary	Unemployed	Urban	No	Yes	No	No	Yes	No	No	No	No	No
2673	HPP0305	Male	Indian	2019	24	Single	Primary	Private	Urban	No	No	No	No	No	No	No	No	No	No
2674	HPP0306	Male	Chinese	2020	41	Single	No formal education	Unemployed	Urban	No	Yes	No	No	No	No	No	No	No	No
2675	HPP0308	Male	Malay	2018	34	Single	Secondary	Unemployed	Urban	No	No	No	No	No	No	No	No	No	Yes
2676	HPP0310	Male	Chinese	2019	33	Married	Primary	Self-employed	Urban	Yes	Yes	Yes	No	No	No	No	No	No	No
2677	HPP0311	Male	Malay	2019	22	Single	Primary	Unemployed	Urban	Yes	Yes	No	No	Yes	No	No	No	No	No
2678	HPP0316	Male	Indian	2021	40	Others	No formal education	Others	Urban	No	No	No	Yes	No	No	No	No	No	No
2679	HPP0317	Male	Chinese	2018	41	Single	Secondary	Private	Urban	No	No	No	Yes	Yes	No	No	No	No	No
2680	HPP0320	Male	Indian	2021	29	Single	Tertiary	Private	Urban	No	Yes	Yes	No	No	No	No	No	No	No
2681	HPP0321	Male	Malay	2018	58	Single	No formal education	Self-employed	Urban	Yes	No	No	No	No	No	No	No	Yes	No
2682	HPP0322	Male	Malay	2019	25	Single	Secondary	Private	Urban	No	Yes	No	Yes	Yes	No	No	No	No	No
2683	HPP0323	Female	Indian	2020	20	Married	Primary	Private	Urban	No	No	No	No	Yes	No	No	No	No	No
2684	HPP0325	Female	Chinese	2019	21	Single	Tertiary	Unemployed	Urban	No	Yes	Yes	No	No	No	No	No	No	No
2685	HPP0326	Male	Others	2019	22	Single	No formal education	Unemployed	Urban	No	No	No	No	Yes	No	No	No	No	No
2686	HPP0328	Male	Malay	2020	32	Single	Primary	Unemployed	Urban	No	No	No	Yes	No	Yes	No	No	No	Yes
2687	HPP0329	Male	Malay	2019	19	Others	Tertiary	Government	Urban	No	No	No	No	Yes	No	No	No	No	No
2688	HPP0330	Male	Malay	2020	46	Others	Secondary	Unemployed	Urban	No	Yes	No	No	Yes	No	No	No	Yes	Yes

No	Patient ID	Gender	Ethnic group	Year of diagnosis	Age	Marital status	Education level	Occupation	Place of residence	History of psy illness	Tobacco	Alcohol	Cannabis	ATS	Inhalant	Sedative	Hallucinogens	Opioids	Kratom
2689	HPP0331	Male	Malay	2019	37	Single	Secondary	Unemployed	Urban	No	Yes	Yes	Yes	Yes	No	No	No	No	Yes
2690	HPP0332	Male	Malay	2018	59	Single	Tertiary	Private	Urban	Yes	Yes	No	Yes	Yes	No	No	No	Yes	No
2691	HPP0334	Male	Malay	2020	41	Others	Secondary	Private	Urban	No	No	No	No	Yes	No	No	No	No	No
2692	HPP0335	Male	Chinese	2018	42	Others	No formal education	Private	Urban	No	Yes	No	No	Yes	No	No	No	No	No
2693	HPP0336	Male	Chinese	2018	38	Others	Primary	Unemployed	Urban	No	Yes	No	No	Yes	No	No	No	No	No
2694	HPP0337	Male	Indian	2020	48	Married	Secondary	Private	Urban	No	Yes	No	No	No	No	No	No	Yes	No
2695	HPP0338	Male	Others	2020	32	Single	Primary	Unemployed	Urban	No	No	No	No	Yes	No	No	No	No	No
2696	HPP0339	Male	Malay	2020	24	Single	Secondary	Unemployed	Urban	Yes	Yes	No	Yes	Yes	No	No	No	Yes	Yes
2697	HPP0340	Male	Malay	2018	28	Married	Secondary	Self-employed	Urban	No	No	No	Yes	No	No	No	No	No	No
2698	HPP0341	Male	Malay	2019	22	Single	Secondary	Unemployed	Urban	No	Yes	Yes	Yes	No	No	No	No	No	No
2699	HPP0342	Female	Malay	2019	22	Single	Secondary	Private	Urban	No	No	No	No	Yes	No	No	No	No	No
2700	HPP0343	Female	Malay	2020	35	Married	Primary	Self-employed	Urban	No	Yes	No	No	Yes	No	No	No	No	No
2701	HPP0344	Female	Chinese	2019	36	Single	Secondary	Private	Urban	No	Yes	No	No	Yes	No	No	No	No	No
2702	HPP0345	Male	Indian	2021	29	Others	Tertiary	Government	Urban	No	Yes	Yes	Yes	No	No	No	No	No	No
2703	HPP0346	Male	Malay	2019	41	Married	Secondary	Private	Urban	No	Yes	No	No	No	No	No	No	No	Yes
2704	HPP0348	Male	Chinese	2018	37	Married	Primary	Unemployed	Urban	Yes	Yes	No	No	No	No	No	No	No	No
2705	HPP0349	Male	Chinese	2018	39	Single	Secondary	Private	Urban	Yes	No	No	No	Yes	No	No	No	No	No
2706	HPP0352	Male	Malay	2020	30	Single	Secondary	Unemployed	Urban	No	Yes	No	No	Yes	No	No	No	No	No
2707	HPP0354	Female	Others	2021	28	Married	No formal education	Unemployed	Urban	No	No	Yes	No	Yes	No	No	No	No	No
2708	HPP0355	Male	Malay	2018	24	Single	Secondary	Private	Urban	No	Yes	No	No	No	No	No	No	No	No
2709	HPP0357	Male	Malay	2018	44	Others	No formal education	Others	Urban	No	Yes	No	No	No	No	No	No	Yes	No
2710	HPP0358	Male	Malay	2020	25	Single	Secondary	Private	Urban	No	Yes	Yes	Yes	Yes	No	No	No	Yes	Yes
2711	HPP0359	Male	Malay	2018	35	Single	Tertiary	Unemployed	Urban	No	No	No	No	No	No	No	No	Yes	No
2712	HPP0360	Male	Malay	2018	35	Married	Secondary	Private	Urban	No	No	No	No	Yes	No	No	No	No	No
2713	HPP0361	Male	Malay	2018	21	Others	No formal education	Others	Urban	Yes	No	No	No	No	No	No	No	No	Yes
2714	HPP0362	Male	Malay	2020	35	Single	Secondary	Private	Urban	No	Yes	No	No	No	No	No	No	No	No
2715	HPP0371	Male	Indian	2020	38	Others	Primary	Unemployed	Urban	No	Yes	Yes	No	No	No	No	No	No	No
2716	HPP0372	Male	Malay	2018	38	Others	Secondary	Private	Urban	Yes	No	No	No	No	No	No	No	Yes	No
2717	HPP0391	Female	Chinese	2018	32	Others	Secondary	Unemployed	Urban	No	No	No	No	Yes	No	Yes	No	No	No
2718	HPP0394	Male	Others	2018	30	Single	No formal education	Private	Urban	Yes	No	Yes	No	No	No	No	No	No	No
2719	HPP0395	Male	Chinese	2019	33	Single	Secondary	Unemployed	Urban	No	Yes	No	No	No	No	No	No	No	No
2720	HPP0397	Male	Malay	2019	55	Others	Secondary	Unemployed	Urban	No	Yes	No	No	No	No	No	No	No	Yes
2721	HPP0398	Male	Malay	2019	31	Married	No formal education	Private	Urban	No	No	No	No	Yes	No	No	No	No	No
2722	HPP0399	Male	Indian	2019	30	Married	Primary	Private	Urban	No	Yes	No	Yes	No	No	No	No	No	No
2723	HPP0400	Male	Malay	2020	31	Married	Secondary	Government	Urban	No	Yes	No	No	No	No	No	No	No	No
2724	HPP0401	Male	Indian	2021	51	Single	Secondary	Others	Urban	No	No	No	No	Yes	No	No	No	Yes	No

No	Patient ID	Gender	Ethnic group	Year of diagnosis	Age	Marital status	Education level	Occupation	Place of residence	History of psy illness	Tobacco	Alcohol	Cannabis	ATS	Inhalant	Sedative	Hallucinogens	Opioids	Kratom
2725	HPP0404	Male	Indian	2018	34	Married	Secondary	Private	Urban	No	No	No	Yes	Yes	No	No	No	No	No
2726	HPP0405	Male	Indian	2018	41	Single	Primary	Private	Urban	No	No	No	Yes	Yes	No	No	No	No	No
2727	HPP0406	Male	Malay	2018	47	Single	Secondary	Unemployed	Urban	No	No	No	No	Yes	No	No	No	No	No
2728	HPP0409	Female	Malay	2018	22	Single	Tertiary	Unemployed	Urban	Yes	Yes	No	Yes	No	No	No	No	No	No
2729	HPP0411	Female	Chinese	2020	41	Others	Tertiary	Private	Urban	No	No	No	No	Yes	No	No	No	No	No
2730	HPP0413	Male	Chinese	2020	48	Others	Secondary	Private	Urban	No	No	No	No	Yes	No	No	No	Yes	No
2731	HPP0414	Male	Indian	2020	44	Single	Secondary	Private	Urban	No	Yes	Yes	No	Yes	No	No	No	Yes	No
2732	HPP0415	Male	Malay	2018	26	Single	Secondary	Private	Urban	No	Yes	No	Yes	Yes	No	No	No	No	No
2733	HPP0416	Male	Chinese	2020	40	Married	Primary	Unemployed	Urban	No	No	No	No	Yes	No	No	No	No	No
2734	HPP0417	Male	Indian	2020	26	Single	Secondary	Private	Urban	No	Yes	No	No	Yes	No	No	No	No	No
2735	HPP0421	Male	Malay	2019	43	Single	Secondary	Unemployed	Urban	No	Yes	No	Yes	Yes	No	No	No	Yes	Yes
2736	HPP0423	Female	Malay	2018	37	Married	Tertiary	Government	Urban	No	Yes	Yes	Yes	No	No	No	No	No	No
2737	HPP0424	Male	Malay	2019	38	Others	Secondary	Private	Urban	No	Yes	No	Yes	Yes	No	No	No	No	Yes
2738	HPP0425	Female	Chinese	2018	42	Others	Secondary	Unemployed	Urban	Yes	No	Yes	No	No	No	No	No	No	No
2739	HPP0426	Male	Indian	2021	25	Others	Tertiary	Self-employed	Urban	Yes	Yes	No	Yes	No	No	No	No	No	No
2740	HPP0427	Male	Malay	2021	38	Single	Secondary	Unemployed	Urban	No	Yes	No	No	Yes	No	No	No	No	No
2741	HPP0430	Male	Malay	2020	29	Single	Secondary	Private	Urban	No	Yes	No	No	Yes	No	No	No	No	No
2742	HPP0431	Male	Malay	2020	40	Married	Secondary	Private	Urban	Yes	Yes	No	No	No	No	No	No	No	No
2743	HPP0433	Male	Indian	2019	38	Single	Secondary	Unemployed	Urban	No	Yes	No	No	No	No	No	No	No	No
2744	HPP0435	Male	Malay	2019	39	Married	No formal education	Government	Urban	No	Yes	No	No	Yes	No	No	No	No	Yes
2745	HPP0436	Female	Malay	2021	40	Single	Tertiary	Unemployed	Urban	No	No	No	Yes	No	No	No	No	No	No
2746	HPP0437	Male	Malay	2020	53	Married	Secondary	Private	Urban	No	No	No	Yes	No	No	No	No	Yes	No
2747	HPP0438	Male	Malay	2021	35	Married	Secondary	Self-employed	Urban	No	Yes	No	No	No	No	No	No	No	No
2748	HPP0440	Male	Malay	2018	54	Single	Secondary	Government	Urban	No	Yes	No	No	No	No	No	No	No	No
2749	HPP0443	Female	Malay	2021	41	Married	Secondary	Unemployed	Urban	No	Yes	No	No	No	No	No	No	No	No
2750	HPP0444	Female	Malay	2021	30	Married	No formal education	Unemployed	Urban	No	Yes	No	No	Yes	No	No	No	No	No
2751	HPP0445	Male	Others	2018	31	Single	Secondary	Unemployed	Urban	No	Yes	No	No	Yes	No	No	No	No	Yes
2752	HPP0448	Male	Chinese	2019	30	Single	Secondary	Private	Urban	No	Yes	Yes	No	No	No	No	No	No	No
2753	HPP0449	Male	Malay	2019	29	Others	No formal education	Others	Urban	No	No	No	No	Yes	No	No	No	No	No
2754	HPP0450	Male	Malay	2020	26	Single	Tertiary	Private	Urban	Yes	Yes	No	Yes	No	No	No	No	No	No
2755	HPP0454	Male	Malay	2021	27	Single	Tertiary	Private	Urban	Yes	Yes	No	No	No	No	No	No	No	No
2756	HPP0455	Male	Chinese	2020	32	Single	Secondary	Unemployed	Urban	Yes	Yes	No	Yes	Yes	No	No	No	Yes	No
2757	HPP0457	Female	Indian	2020	24	Single	Tertiary	Self-employed	Urban	Yes	Yes	Yes	No	No	No	No	No	No	No
2758	HPP0460	Male	Malay	2020	33	Single	Secondary	Unemployed	Urban	No	Yes	Yes	No	Yes	No	No	No	Yes	Yes
2759	HPP0476	Male	Malay	2020	36	Single	Secondary	Private	Urban	Yes	Yes	No	No	No	No	No	No	No	No
2760	HPP0478	Female	Chinese	2020	36	Married	Tertiary	Private	Urban	Yes	Yes	Yes	No	No	No	No	No	No	No
2761	HPP0479	Female	Chinese	2020	40	Single	Primary	Unemployed	Urban	No	No	Yes	No	No	No	No	No	No	No
2762	HPP0480	Female	Malay	2019	33	Others	Tertiary	Private	Urban	No	Yes	Yes	Yes	No	No	No	No	No	No
2763	HPP0481	Female	Indian	2020	28	Married	Tertiary	Unemployed	Urban	No	No	Yes	No	No	No	No	No	No	No

No	Patient ID	Gender	Ethnic group	Year of diagnosis	Age	Marital status	Education level	Occupation	Place of residence	History of psy illness	Tobacco	Alcohol	Cannabis	ATS	Inhalant	Sedative	Hallucinogens	Opioids	Kratom
2764	HPP0482	Female	Indian	2019	26	Single	Tertiary	Unemployed	Urban	No	No	Yes	No	No	No	No	No	No	No
2765	HPP0483	Female	Chinese	2020	21	Single	Tertiary	Unemployed	Urban	Yes	No	Yes	No	No	No	No	No	No	No
2766	HPP0484	Male	Chinese	2020	19	Single	Tertiary	Unemployed	Urban	Yes	No	Yes	No	No	No	No	No	No	No
2767	HPP0485	Male	Indian	2019	19	Single	Tertiary	Unemployed	Urban	No	Yes	Yes	Yes	No	No	No	No	No	No
2768	HPP0486	Male	Malay	2019	18	Single	Secondary	Unemployed	Urban	No	No	No	Yes	No	No	No	No	No	Yes
2769	HPP0487	Female	Others	2020	28	Married	Tertiary	Self-employed	Urban	No	No	No	Yes	Yes	No	No	No	No	No
2770	HPP0488	Male	Others	2020	26	Married	Primary	Private	Urban	Yes	Yes	No	No	No	No	No	No	No	No
2771	HPP0490	Male	Malay	2019	38	Married	Secondary	Private	Urban	No	Yes	No	No	No	No	No	No	No	No
2772	HPP0492	Male	Chinese	2018	24	Single	Tertiary	Private	Urban	No	No	Yes	No	No	No	No	No	No	No
2773	HPP0494	Male	Malay	2018	32	Single	Secondary	Unemployed	Urban	No	Yes	Yes	No	No	No	No	No	No	No
2774	HPP0495	Male	Malay	2020	31	Married	Secondary	Private	Urban	Yes	Yes	No	No	No	No	No	No	No	No
2775	HPP0496	Male	Indian	2020	33	Married	Secondary	Private	Urban	No	Yes	Yes	No	No	No	No	No	No	No
2776	HPP0499	Male	Indian	2021	34	Married	Tertiary	Private	Urban	No	Yes	Yes	No	No	No	No	No	No	No
2777	HPP0502	Male	Indian	2018	39	Single	Secondary	Unemployed	Urban	No	Yes	Yes	No	No	No	No	No	No	No
2778	HPP0503	Male	Malay	2020	24	Single	Tertiary	Unemployed	Urban	No	Yes	No	Yes	No	No	No	No	No	No
2779	HPP0504	Male	Chinese	2020	51	Married	Secondary	Unemployed	Urban	No	Yes	Yes	No	No	No	No	No	No	No
2780	HPP0505	Male	Chinese	2020	31	Single	Tertiary	Private	Urban	No	No	Yes	No	No	No	No	No	No	No
2781	HPP0506	Male	Malay	2020	32	Married	Secondary	Private	Urban	No	No	No	Yes	No	No	No	No	No	No
2782	HPP0508	Male	Chinese	2018	47	Single	Secondary	Private	Urban	No	Yes	No	No	No	No	No	No	No	No
2783	HPP0509	Male	Malay	2018	22	Single	Primary	Private	Urban	No	No	No	Yes	No	No	No	No	No	No
2784	HPP0511	Male	Indian	2021	37	Single	Secondary	Private	Urban	No	Yes	Yes	Yes	No	No	No	No	No	No
2785	HPP0512	Male	Indian	2021	34	Single	No formal education	Unemployed	Urban	No	Yes	Yes	Yes	No	No	No	No	No	No
2786	HPP0513	Male	Malay	2019	37	Single	Tertiary	Private	Urban	Yes	Yes	Yes	No	No	No	No	No	Yes	Yes
2787	HPP0514	Male	Indian	2018	57	Single	Secondary	Unemployed	Urban	No	Yes	Yes	No	No	No	No	No	No	No
2788	HPP0558	Male	Malay	2018	58	Married	Secondary	Private	Urban	No	Yes	No	No	No	No	No	No	Yes	No
2789	HPP0561	Male	Chinese	2020	38	Single	Secondary	Private	Urban	Yes	Yes	Yes	No	No	No	No	No	No	No
2790	HPP0562	Male	Malay	2020	35	Single	Secondary	Unemployed	Urban	No	Yes	No	No	Yes	No	No	No	Yes	Yes
2791	HPP0563	Female	Chinese	2020	46	Married	Tertiary	Others	Urban	Yes	Yes	Yes	No	No	No	No	No	No	No
2792	HPP0566	Male	Malay	2019	20	Single	Secondary	Unemployed	Urban	No	Yes	No	No	No	Yes	No	No	No	No
2793	HPP0567	Female	Chinese	2019	19	Single	Tertiary	Unemployed	Urban	No	No	Yes	No	No	No	No	No	No	No
2794	HPP0568	Female	Chinese	2019	19	Single	Tertiary	Unemployed	Urban	No	No	Yes	No	No	No	No	No	No	No
2795	HPP0569	Male	Malay	2021	19	Single	Secondary	Private	Urban	No	No	Yes	No	No	No	No	No	No	No
2796	HPP0571	Female	Malay	2021	38	Single	Primary	Unemployed	Urban	No	Yes	Yes	No	Yes	No	No	No	No	Yes
2797	HPP0572	Male	Chinese	2018	60	Single	No formal education	Unemployed	Urban	Yes	Yes	No	No	No	No	No	No	No	No
2798	HPP0573	Male	Chinese	2018	54	Others	No formal education	Others	Urban	No	No	No	No	No	No	No	No	No	No
2799	HPP0575	Male	Indian	2021	29	Single	No formal education	Unemployed	Urban	Yes	Yes	Yes	No	No	No	No	No	No	No
2800	HPP0576	Female	Malay	2021	31	Single	Tertiary	Private	Urban	No	No	Yes	No	No	No	No	No	No	No
2801	HPP0577	Male	Malay	2019	37	Others	Tertiary	Others	Urban	No	Yes	No	Yes	Yes	No	No	No	No	No
2802	HPP0578	Female	Malay	2020	32	Married	Tertiary	Private	Urban	No	Yes	Yes	No	No	No	No	No	No	No

No	Patient ID	Gender	Ethnic group	Year of diagnosis	Age	Marital status	Education level	Occupation	Place of residence	History of psy illness	Tobacco	Alcohol	Cannabis	ATS	Inhalant	Sedative	Hallucinogens	Opioids	Kratom
2803	HPP0579	Female	Chinese	2019	28	Single	Tertiary	Private	Urban	Yes	No	Yes	No	No	No	No	No	No	No
2804	HPP0581	Female	Chinese	2020	48	Single	Tertiary	Private	Urban	Yes	No	Yes	No	No	No	No	No	No	No
2805	HPP0582	Male	Malay	2019	34	Married	No formal education	Unemployed	Urban	No	Yes	No	Yes	Yes	No	No	No	No	Yes
2806	HPP0583	Female	Chinese	2020	58	Others	Primary	Self-employed	Urban	No	Yes	No	No	No	No	No	No	No	No
2807	HPP0584	Male	Indian	2021	28	Single	Secondary	Private	Urban	No	Yes	Yes	Yes	No	No	No	No	No	No
2808	HPP0585	Male	Chinese	2020	33	Single	Secondary	Private	Urban	No	Yes	No	No	No	No	No	No	No	No
2809	HPP0586	Male	Malay	2018	31	Married	Secondary	Private	Urban	No	Yes	Yes	No	Yes	No	No	No	No	No
2810	HPP0587	Male	Chinese	2021	54	Others	Secondary	Unemployed	Urban	No	Yes	No	No	No	No	No	No	No	No
2811	HPP0589	Male	Indian	2019	24	Single	Primary	Unemployed	Urban	No	Yes	Yes	Yes	Yes	No	No	No	No	No
2812	HPP0590	Male	Malay	2020	32	Single	Secondary	Unemployed	Urban	No	Yes	No	Yes	Yes	No	No	No	No	No
2813	HPP0592	Male	Malay	2019	52	Married	Secondary	Private	Urban	No	Yes	No	No	No	No	No	No	No	No
2814	HPP0593	Male	Chinese	2018	19	Single	Secondary	Unemployed	Urban	No	Yes	No	No	No	No	No	No	No	No
2815	HPP0595	Male	Malay	2018	34	Married	Secondary	Private	Urban	No	Yes	No	No	No	No	No	No	No	No
2816	HPP0596	Male	Chinese	2020	39	Single	Secondary	Unemployed	Urban	No	Yes	Yes	No	No	No	No	No	No	No
2817	HPP0597	Male	Chinese	2019	22	Single	Secondary	Private	Urban	No	No	Yes	No	No	No	No	No	No	No
2818	HPP0598	Male	Indian	2019	32	Single	Tertiary	Government	Urban	No	Yes	Yes	No	No	No	No	No	No	No
2819	HPP0599	Male	Chinese	2018	23	Single	Secondary	Unemployed	Urban	No	Yes	No	No	No	No	No	No	No	No
2820	HPP0600	Male	Chinese	2019	40	Single	Secondary	Unemployed	Urban	Yes	Yes	No	No	No	No	No	No	No	No
2821	HPP0601	Male	Malay	2018	27	Married	Secondary	Self-employed	Urban	No	Yes	No	No	No	No	No	No	No	No
2822	HPP0603	Male	Malay	2018	53	Single	No formal education	Unemployed	Urban	No	No	No	No	No	No	No	No	Yes	No
2823	HPP0605	Male	Malay	2021	37	Single	Secondary	Private	Urban	No	Yes	No	Yes	Yes	No	No	No	Yes	No
2824	HPP0607	Male	Chinese	2018	29	Single	Tertiary	Private	Urban	No	No	Yes	No	No	No	No	No	No	No
2825	HPP0609	Female	Chinese	2020	38	Others	Tertiary	Private	Urban	No	Yes	No	No	No	No	No	No	No	No
2826	HPP0610	Female	Indian	2019	52	Married	No formal education	Others	Urban	Yes	No	No	No	No	No	Yes	No	No	Yes
2827	HPP0611	Male	Malay	2019	35	Single	Secondary	Private	Urban	No	Yes	No	No	Yes	No	No	No	Yes	No
2828	HPP0612	Female	Chinese	2018	50	Others	Tertiary	Private	Urban	No	Yes	Yes	Yes	No	No	No	No	No	No
2829	HPP0613	Male	Chinese	2018	28	Single	Tertiary	Private	Urban	Yes	Yes	Yes	No	No	No	No	No	No	No
2830	HPP0614	Male	Indian	2019	35	Married	Secondary	Private	Urban	No	Yes	Yes	No	No	No	No	No	No	No
2831	HPP0615	Male	Malay	2019	38	Married	Secondary	Government	Urban	No	Yes	Yes	No	No	No	No	No	No	No
2832	HPP0616	Male	Indian	2021	27	Single	No formal education	Unemployed	Urban	No	Yes	Yes	No	No	No	No	No	No	No
2833	HPP0617	Male	Malay	2021	30	Married	Secondary	Private	Urban	No	Yes	No	Yes	Yes	No	No	No	No	No
2834	HPP0619	Male	Malay	2018	31	Single	Tertiary	Private	Urban	No	Yes	No	No	No	No	No	No	No	No
2835	HPP0620	Male	Chinese	2019	29	Single	Tertiary	Unemployed	Urban	No	No	Yes	No	No	No	No	No	No	No
2836	HPP0622	Male	Malay	2018	22	Single	Secondary	Private	Urban	No	Yes	No	No	Yes	No	No	No	No	No
2837	HPP0624	Female	Malay	2019	29	Others	Secondary	Private	Urban	No	No	No	No	Yes	No	No	No	No	No
2838	HPP0626	Female	Indian	2018	21	Others	Tertiary	Unemployed	Urban	Yes	Yes	Yes	No	No	No	No	No	No	No
2839	HPP0627	Female	Chinese	2019	49	Others	Tertiary	Unemployed	Urban	Yes	No	Yes	No	No	No	No	No	No	No
2840	HPP0628	Male	Chinese	2020	20	Single	Tertiary	Unemployed	Urban	No	No	Yes	No	No	No	No	No	No	No
2841	HPP0629	Female	Chinese	2018	20	Single	Tertiary	Unemployed	Urban	No	Yes	Yes	No	No	No	No	No	No	No
2842	HPP0630	Male	Chinese	2019	18	Single	Secondary	Unemployed	Urban	No	No	Yes	No	No	No	No	No	No	No

No	Patient ID	Gender	Ethnic group	Year of diagnosis	Age	Marital status	Education level	Occupation	Place of residence	History of psy illness	Tobacco	Alcohol	Cannabis	ATS	Inhalant	Sedative	Hallucinogens	Opioids	Kratom
2843	HPP0631	Male	Others	2019	38	Single	No formal education	Unemployed	Urban	No	Yes	No	No	No	No	No	No	No	No
2844	HPP0632	Female	Chinese	2019	30	Married	Tertiary	Private	Urban	No	No	Yes	No	No	No	No	No	No	No
2845	HPP0636	Male	Indian	2019	43	Married	Secondary	Private	Urban	No	Yes	Yes	No	No	No	No	No	No	No
2846	HPP0637	Male	Chinese	2020	51	Single	Secondary	Unemployed	Urban	Yes	Yes	Yes	No	No	No	No	No	No	No
2847	HPP0639	Male	Malay	2020	28	Married	Secondary	Unemployed	Urban	No	Yes	Yes	No	Yes	No	No	No	No	No
2848	HPP0640	Male	Malay	2021	46	Others	Secondary	Private	Urban	Yes	Yes	Yes	No	Yes	No	No	No	Yes	No
2849	HPP0641	Male	Malay	2020	24	Single	Tertiary	Private	Urban	No	Yes	No	No	No	No	No	No	No	No
2850	HPP0642	Male	Chinese	2019	50	Married	Secondary	Self-employed	Urban	No	Yes	Yes	Yes	Yes	No	No	No	No	No
2851	HPP0643	Male	Indian	2020	42	Single	Secondary	Unemployed	Urban	No	Yes	No	No	No	No	No	No	No	No
2852	HPP0644	Male	Malay	2018	34	Single	Secondary	Unemployed	Urban	No	Yes	No	Yes	No	No	No	No	No	No
2853	HPP0647	Male	Malay	2020	45	Single	Secondary	Unemployed	Urban	No	Yes	No	No	Yes	No	No	No	Yes	No
2854	HPP0648	Male	Chinese	2021	50	Others	Secondary	Self-employed	Urban	No	Yes	Yes	No	No	No	No	No	No	No
2855	HPP0649	Male	Others	2020	28	Single	No formal education	Unemployed	Urban	Yes	Yes	No	No	No	No	No	No	No	No
2856	HPP0651	Male	Chinese	2020	27	Single	Secondary	Unemployed	Urban	No	Yes	Yes	No	No	No	No	No	No	No
2857	HPP0652	Male	Malay	2018	34	Single	Tertiary	Private	Urban	No	No	No	Yes	No	No	No	No	No	No
2858	HPP0653	Male	Chinese	2018	51	Married	Primary	Private	Urban	No	Yes	Yes	No	No	No	No	No	No	No
2859	HPP0655	Male	Indian	2020	26	Single	Tertiary	Private	Urban	No	Yes	Yes	Yes	Yes	No	No	No	No	No
2860	HPP0659	Male	Others	2020	26	Married	Tertiary	Government	Urban	No	Yes	Yes	No	No	No	No	No	No	No
2861	HPP0661	Male	Malay	2020	56	Single	Secondary	Unemployed	Urban	Yes	Yes	No	No	No	No	No	No	No	No
2862	HPP0662	Male	Malay	2020	28	Single	Secondary	Self-employed	Urban	No	Yes	No	Yes	No	No	No	No	Yes	No
2863	HPP0664	Male	Others	2018	26	Single	No formal education	Others	Urban	No	Yes	No	No	Yes	No	No	No	No	No
2864	HPP0666	Female	Indian	2021	39	Single	Secondary	Private	Urban	Yes	No	Yes	No	No	No	No	No	No	No
2865	HPP0667	Male	Chinese	2019	24	Single	Tertiary	Unemployed	Urban	No	No	Yes	No	No	No	No	No	No	No
2866	HPP0669	Male	Malay	2019	41	Single	No formal education	Unemployed	Urban	No	Yes	No	Yes	No	No	No	No	Yes	No
2867	HPP0671	Male	Chinese	2018	56	Married	Secondary	Unemployed	Urban	No	No	Yes	No	No	No	No	No	No	No
2868	HPP0674	Male	Others	2021	48	Married	Secondary	Private	Urban	No	Yes	No	No	No	No	No	No	No	No
2869	HPP0675	Male	Malay	2018	57	Married	Secondary	Unemployed	Urban	No	Yes	No	Yes	No	No	No	No	No	No
2870	HPP0676	Male	Others	2019	38	Married	Tertiary	Private	Urban	Yes	Yes	Yes	No	No	No	No	No	No	No
2871	HPP0677	Male	Malay	2021	48	Others	Primary	Unemployed	Urban	No	Yes	No	Yes	Yes	No	No	No	No	No
2872	HPP0678	Male	Others	2018	40	Married	Tertiary	Private	Urban	No	Yes	Yes	No	No	No	No	No	No	No
2873	HPP0679	Male	Malay	2019	48	Single	Primary	Private	Urban	No	No	No	No	No	No	No	No	Yes	No
2874	HPP0691	Male	Chinese	2018	51	Married	Secondary	Private	Urban	Yes	No	No	No	Yes	No	No	No	Yes	No
2875	HPP0699	Male	Chinese	2019	50	Single	Secondary	Self-employed	Urban	Yes	No	Yes	No	Yes	No	Yes	No	Yes	No
2876	HPP0700	Male	Chinese	2021	51	Single	Primary	Private	Urban	No	No	No	Yes	Yes	No	No	No	Yes	No
2877	HPP0701	Male	Malay	2021	43	Single	Secondary	Private	Urban	No	No	No	Yes	Yes	No	No	No	Yes	No
2878	HPP0702	Male	Malay	2021	32	Single	Tertiary	Private	Urban	No	No	No	No	No	No	No	No	Yes	No
2879	HPP0703	Male	Indian	2021	60	Married	Secondary	Others	Urban	No	No	No	No	Yes	No	No	No	Yes	No

No	Patient ID	Gender	Ethnic group	Year of diagnosis	Age	Marital status	Education level	Occupation	Place of residence	History of psy illness	Tobacco	Alcohol	Cannabis	ATS	Inhalant	Sedative	Hallucinogens	Opioids	Kratom
2880	HPP0704	Male	Indian	2018	72	Married	Primary	Unemployed	Urban	Yes	Yes	Yes	No	No	No	No	No	Yes	No
2881	HPP0705	Male	Indian	2021	49	Married	Secondary	Private	Urban	No	No	No	No	No	No	No	No	Yes	No
2882	HPP0706	Male	Malay	2021	37	Single	Secondary	Private	Urban	No	No	No	No	Yes	No	No	No	Yes	No
2883	HPP0707	Male	Indian	2018	27	Others	Secondary	Unemployed	Urban	Yes	Yes	Yes	Yes	Yes	No	No	No	Yes	No
2884	HPP0708	Male	Indian	2021	56	Single	Secondary	Unemployed	Urban	No	No	No	No	No	No	No	No	Yes	No
2885	HPP0709	Male	Malay	2021	50	Married	Secondary	Private	Urban	No	No	No	Yes	Yes	No	No	No	Yes	No
2886	HPP0710	Male	Indian	2018	56	Married	Secondary	Unemployed	Urban	Yes	Yes	Yes	No	No	No	No	No	Yes	No
2887	HPP0711	Male	Indian	2021	43	Single	Secondary	Private	Urban	No	No	No	No	No	No	No	No	Yes	No
2888	HPP0713	Male	Indian	2019	52	Single	Secondary	Private	Urban	No	No	No	Yes	Yes	No	No	No	Yes	No
2889	HPP0723	Male	Malay	2019	38	Single	Secondary	Private	Urban	Yes	No	No	No	No	No	No	No	Yes	No
2890	HPP0727	Male	Indian	2020	48	Married	Secondary	Private	Urban	No	Yes	Yes	Yes	No	No	No	No	No	No
2891	HPP0731	Male	Malay	2021	54	Single	Secondary	Unemployed	Urban	No	Yes	No	No	No	No	No	No	No	No
2892	HPP0733	Male	Chinese	2018	55	Single	Primary	Private	Urban	No	Yes	No	No	No	No	No	No	No	No
2893	HPP0734	Male	Chinese	2020	54	Married	Primary	Self-employed	Urban	No	Yes	No	No	No	No	No	No	No	No
2894	HPP0735	Male	Malay	2019	22	Single	Secondary	Private	Urban	No	Yes	No	No	No	No	No	No	No	No
2895	HPP0736	Male	Malay	2020	28	Single	Secondary	Private	Urban	No	Yes	No	Yes	No	No	No	No	No	Yes
2896	HPP0737	Female	Chinese	2021	23	Single	Secondary	Unemployed	Urban	No	No	Yes	No	No	No	No	No	No	No
2897	HPP0738	Male	Malay	2021	29	Single	Secondary	Private	Urban	No	Yes	Yes	No	No	No	No	No	No	Yes
2898	HPP0739	Female	Indian	2020	31	Married	Secondary	Unemployed	Urban	No	No	Yes	No	No	No	No	No	No	No
2899	HPP0740	Female	Malay	2018	24	Single	Tertiary	Private	Urban	No	No	Yes	No	No	No	No	No	No	No
2900	HPP0741	Male	Chinese	2019	23	Single	Secondary	Unemployed	Urban	No	Yes	Yes	No	Yes	No	No	No	No	No
2901	HPP0743	Female	Malay	2018	45	Married	Secondary	Unemployed	Urban	No	Yes	No	No	No	No	No	No	No	No
2902	HPP0744	Female	Chinese	2018	44	Married	Tertiary	Private	Urban	No	No	Yes	No	No	No	No	No	No	No
2903	HPP0746	Male	Malay	2020	34	Married	Tertiary	Government	Urban	No	Yes	No	No	No	No	No	No	No	Yes
2904	HPP0748	Male	Chinese	2019	50	Married	No formal education	Unemployed	Urban	No	No	Yes	No	No	No	No	No	No	No
2905	HPP0749	Female	Chinese	2019	43	Married	Secondary	Unemployed	Urban	No	No	Yes	No	No	No	No	No	No	No
2906	HPP0750	Male	Indian	2019	24	Single	No formal education	Unemployed	Urban	No	Yes	Yes	Yes	No	No	No	No	No	No
2907	HPP0751	Male	Indian	2018	25	Single	Primary	Private	Urban	No	Yes	No	No	No	No	No	No	No	No
2908	HPP0754	Male	Indian	2018	29	Single	Secondary	Private	Urban	No	Yes	Yes	Yes	Yes	No	No	No	No	No
2909	HPP0755	Male	Malay	2018	38	Single	Secondary	Private	Urban	No	Yes	No	Yes	No	No	No	No	No	No
2910	HPP0756	Male	Chinese	2019	57	Single	Primary	Private	Urban	No	No	No	No	Yes	No	No	No	Yes	No
2911	HPP0757	Male	Malay	2018	26	Single	Secondary	Unemployed	Urban	No	No	No	No	No	No	No	No	No	Yes
2912	HPP0758	Male	Malay	2019	26	Married	Tertiary	Self-employed	Urban	No	Yes	Yes	No	No	No	No	No	No	Yes
2913	HPP0759	Male	Malay	2019	35	Single	Secondary	Private	Urban	No	Yes	No	No	No	No	No	No	No	No
2914	HPP0760	Male	Chinese	2020	65	Married	Secondary	Unemployed	Urban	No	Yes	No	No	No	No	No	No	No	No
2915	HPP0762	Male	Malay	2020	58	Married	Secondary	Private	Urban	No	Yes	No	No	No	No	No	No	No	No
2916	HPP0763	Male	Chinese	2019	69	Married	No formal education	Unemployed	Urban	No	Yes	No	No	No	No	No	No	No	No
2917	HPP0764	Male	Malay	2021	45	Others	No formal education	Private	Urban	No	Yes	Yes	No	Yes	No	No	No	Yes	No

No	Patient ID	Gender	Ethnic group	Year of diagnosis	Age	Marital status	Education level	Occupation	Place of residence	History of psy illness	Tobacco	Alcohol	Cannabis	ATS	Inhalant	Sedative	Hallucinogens	Opioids	Kratom
2918	HPP0765	Male	Malay	2020	33	Married	Secondary	Private	Urban	No	Yes	No	No	No	No	No	No	No	No
2919	HPP0767	Male	Chinese	2019	50	Others	No formal education	Private	Urban	No	Yes	Yes	No	No	No	No	No	No	No
2920	HPP0769	Male	Chinese	2018	41	Single	Primary	Unemployed	Urban	Yes	Yes	No	No	No	No	No	No	No	No
2921	HPP0770	Male	Malay	2021	27	Single	Tertiary	Government	Urban	No	Yes	Yes	No	No	No	No	No	No	No
2922	HPP0771	Male	Malay	2018	29	Single	Tertiary	Private	Urban	No	Yes	No	No	No	No	No	No	No	No
2923	HPP0773	Male	Indian	2021	28	Single	Secondary	Private	Urban	No	Yes	Yes	No	Yes	No	No	No	No	No
2924	HPP0774	Male	Malay	2018	44	Married	Tertiary	Government	Urban	Yes	Yes	No	No	No	No	No	No	No	No
2925	HPP0775	Male	Malay	2018	22	Single	Secondary	Private	Urban	No	No	No	No	Yes	No	No	No	No	No
2926	HPP0776	Male	Chinese	2018	40	Single	Secondary	Private	Urban	No	Yes	Yes	No	No	No	No	No	No	No
2927	HPP0777	Male	Indian	2019	35	Single	Secondary	Private	Urban	No	Yes	Yes	No	No	No	No	No	No	No
2928	HPP0780	Male	Chinese	2018	45	Married	Secondary	Self-employed	Urban	No	No	Yes	No	No	No	No	No	No	No
2929	HPP0782	Female	Chinese	2019	48	Single	Secondary	Private	Urban	No	No	No	No	No	No	Yes	No	No	No
2930	HPP0783	Female	Malay	2019	22	Single	Tertiary	Unemployed	Urban	No	Yes	No	Yes	No	No	No	No	No	No
2931	HPP0784	Female	Malay	2018	35	Others	No formal education	Others	Urban	No	No	No	No	Yes	No	No	No	No	No
2932	HPP0786	Female	Chinese	2018	38	Married	Tertiary	Unemployed	Urban	No	Yes	No	No	No	No	No	No	No	No
2933	HPP0788	Male	Malay	2018	33	Single	Secondary	Unemployed	Urban	Yes	Yes	No	No	No	No	No	No	No	No
2934	HPP0789	Male	Malay	2020	20	Single	Secondary	Unemployed	Urban	No	Yes	No	Yes	No	No	No	No	No	No
2935	HPP0790	Male	Malay	2018	28	Single	Tertiary	Government	Urban	No	Yes	No	No	No	No	No	No	No	No
2936	HPP0791	Male	Malay	2018	33	Married	Secondary	Private	Urban	No	No	No	No	Yes	No	No	No	No	No
2937	HPP0792	Male	Malay	2018	43	Married	Secondary	Private	Urban	No	Yes	No	No	No	No	No	No	No	No
2938	HPP0793	Male	Malay	2020	25	Married	Secondary	Private	Urban	No	Yes	No	No	No	No	No	No	No	No
2939	HPP0795	Male	Indian	2020	23	Others	Tertiary	Unemployed	Urban	No	Yes	Yes	No	No	No	No	No	No	No
2940	HPP0796	Male	Indian	2021	29	Others	Secondary	Private	Urban	No	Yes	Yes	No	No	No	No	No	No	No
2941	HPP0797	Male	Chinese	2019	43	Married	Secondary	Unemployed	Urban	No	No	Yes	Yes	Yes	No	No	No	No	No
2942	HPP0799	Male	Chinese	2020	57	Married	Secondary	Private	Urban	Yes	Yes	No	No	No	No	No	No	No	No
2943	HPP0800	Male	Chinese	2018	50	Married	Secondary	Private	Urban	No	Yes	Yes	No	No	No	No	No	No	No
2944	HPP0801	Male	Indian	2021	33	Single	Secondary	Private	Urban	No	Yes	Yes	No	Yes	No	No	No	No	No
2945	HPP0802	Male	Indian	2020	31	Single	Secondary	Private	Urban	No	No	Yes	No	No	No	No	No	No	No
2946	HPP0804	Male	Malay	2020	38	Married	Secondary	Unemployed	Urban	No	Yes	No	Yes	No	No	No	No	No	Yes
2947	HPP0805	Male	Indian	2020	35	Single	Tertiary	Unemployed	Urban	Yes	Yes	No	No	No	No	No	No	No	No
2948	HPP0807	Male	Indian	2019	19	Others	Secondary	Private	Urban	No	Yes	Yes	No	No	No	No	No	No	No
2949	HPP0808	Male	Malay	2019	43	Single	Secondary	Private	Urban	Yes	Yes	No	No	No	No	No	No	No	No
2950	HPP0809	Male	Chinese	2019	25	Single	Secondary	Private	Urban	No	Yes	Yes	No	Yes	No	No	No	No	No
2951	HPP0810	Male	Malay	2019	22	Single	Secondary	Unemployed	Urban	No	No	No	No	No	No	No	No	No	Yes
2952	HPP0811	Male	Indian	2021	40	Married	Tertiary	Private	Urban	No	No	Yes	No	No	No	No	No	No	No
2953	HPP0812	Male	Indian	2021	49	Married	Secondary	Private	Urban	No	Yes	Yes	Yes	Yes	No	No	No	No	No
2954	HPP0813	Male	Chinese	2020	49	Married	Secondary	Private	Urban	No	Yes	No	No	No	No	No	No	No	No
2955	HPP0816	Male	Malay	2018	56	Married	Secondary	Others	Urban	Yes	Yes	Yes	Yes	No	No	No	No	No	No
2956	HPP0817	Male	Malay	2018	32	Single	No formal education	Unemployed	Urban	No	No	No	No	Yes	No	No	No	No	Yes
2957	HPP0819	Male	Indian	2018	57	Single	Primary	Private	Urban	No	No	Yes	No	No	No	No	No	No	No

No	Patient ID	Gender	Ethnic group	Year of diagnosis	Age	Marital status	Education level	Occupation	Place of residence	History of psy illness	Tobacco	Alcohol	Cannabis	ATS	Inhalant	Sedative	Hallucinogens	Opioids	Kratom
2958	HPP0820	Male	Malay	2018	51	Others	No formal education	Unemployed	Urban	Yes	Yes	No	No	No	No	Yes	No	No	No
2959	HPP0821	Male	Malay	2018	41	Others	Tertiary	Unemployed	Urban	No	Yes	No	No	Yes	No	No	No	No	No
2960	HPP0822	Female	Chinese	2019	30	Others	Secondary	Private	Urban	No	Yes	Yes	No	No	No	No	No	No	No
2961	HPP0823	Male	Chinese	2018	59	Others	Primary	Unemployed	Urban	Yes	Yes	Yes	No	No	No	No	No	No	No
2962	HPP0824	Female	Malay	2020	22	Single	Tertiary	Unemployed	Urban	No	No	No	Yes	No	No	No	No	No	No
2963	HPP0826	Female	Chinese	2018	21	Single	Tertiary	Unemployed	Urban	Yes	No	Yes	No	No	No	No	No	No	No
2964	HPP0827	Male	Indian	2020	53	Single	Secondary	Private	Urban	Yes	Yes	Yes	No	No	No	No	No	No	No
2965	HPP0829	Male	Malay	2020	35	Single	Secondary	Private	Urban	No	Yes	Yes	No	No	No	No	No	No	No
2966	HPP0830	Male	Malay	2018	56	Married	Secondary	Government	Urban	No	Yes	No	No	No	No	No	No	No	No
2967	HPP0832	Male	Malay	2018	29	Single	Secondary	Unemployed	Urban	No	Yes	Yes	No	No	No	No	No	No	No
2968	HPP0833	Male	Indian	2020	24	Single	Tertiary	Private	Urban	No	No	Yes	No	No	No	No	No	No	No
2969	HPP0834	Female	Chinese	2020	27	Married	Secondary	Unemployed	Urban	No	No	Yes	No	No	No	No	No	No	No
2970	HPP0836	Male	Malay	2021	25	Single	Secondary	Unemployed	Urban	No	Yes	No	No	No	No	No	No	No	No
2971	HPP0837	Male	Chinese	2021	34	Married	Secondary	Private	Urban	No	Yes	No	No	No	No	No	No	No	No
2972	HPP0838	Male	Chinese	2021	69	Married	Secondary	Self-employed	Urban	No	Yes	Yes	No	No	No	No	No	No	No
2973	HPP0839	Female	Indian	2020	29	Married	Tertiary	Private	Urban	No	No	Yes	No	No	No	No	No	No	No
2974	HPP0840	Male	Indian	2020	33	Single	No formal education	Unemployed	Urban	No	Yes	Yes	No	No	No	No	No	No	No
2975	HPP0841	Female	Chinese	2020	25	Single	Tertiary	Unemployed	Urban	No	No	Yes	No	No	No	No	No	No	No
2976	HPP0842	Male	Indian	2018	29	Single	Secondary	Private	Urban	No	Yes	Yes	Yes	No	No	No	No	Yes	No
2977	HPP0843	Male	Chinese	2018	42	Single	Secondary	Private	Urban	No	Yes	Yes	Yes	No	No	No	No	No	No
2978	HPP0844	Male	Malay	2020	41	Others	No formal education	Private	Urban	No	Yes	No	No	Yes	No	No	No	Yes	No
2979	HPP0845	Male	Malay	2021	35	Married	Secondary	Self-employed	Urban	No	Yes	Yes	Yes	Yes	No	No	No	No	Yes
2980	HPP0848	Male	Chinese	2019	30	Single	Secondary	Self-employed	Urban	No	No	Yes	No	No	No	No	No	No	No
2981	HPP0849	Female	Chinese	2018	44	Single	Primary	Unemployed	Urban	Yes	Yes	No	No	No	No	No	No	No	No
2982	HPP0850	Male	Chinese	2020	29	Single	Secondary	Private	Urban	No	Yes	No	No	No	No	No	No	No	No
2983	HPP0851	Male	Malay	2019	35	Single	Secondary	Unemployed	Urban	No	Yes	No	No	No	No	No	No	No	No
2984	HPP0854	Female	Chinese	2021	41	Married	Secondary	Private	Urban	No	No	Yes	No	No	No	No	No	No	No
2985	HPP0855	Male	Chinese	2020	36	Others	Tertiary	Private	Urban	No	No	Yes	No	No	No	No	No	No	No
2986	HPP0857	Male	Malay	2018	55	Single	Secondary	Private	Urban	Yes	No	No	No	No	No	No	No	Yes	No
2987	HPP0858	Male	Malay	2018	21	Single	Tertiary	Unemployed	Urban	No	Yes	No	No	No	No	No	No	No	No
2988	HPP0859	Female	Indian	2021	26	Single	Tertiary	Unemployed	Urban	No	No	Yes	No	No	No	No	No	No	No
2989	HPP0860	Male	Malay	2018	21	Single	Secondary	Unemployed	Urban	No	Yes	Yes	No	No	No	No	No	No	No
2990	HPP0861	Female	Chinese	2019	22	Single	Tertiary	Unemployed	Urban	No	No	Yes	No	No	No	No	No	No	No
2991	HPP0862	Male	Others	2020	58	Married	Tertiary	Others	Urban	Yes	Yes	No	No	No	No	No	No	No	No
2992	HPP0863	Male	Malay	2021	19	Single	Secondary	Unemployed	Urban	No	Yes	No	Yes	Yes	Yes	No	No	No	Yes
2993	HPP0864	Male	Others	2021	22	Single	Secondary	Private	Urban	No	Yes	No	No	Yes	No	No	No	No	No
2994	HPP0865	Male	Others	2020	28	Married	Tertiary	Private	Urban	No	Yes	No	No	Yes	No	No	No	No	No
2995	HPP0866	Male	Malay	2018	20	Single	Secondary	Private	Urban	No	Yes	No	No	No	No	No	No	No	No
2996	HPP0867	Female	Chinese	2019	47	Others	Secondary	Private	Urban	Yes	Yes	Yes	No	No	No	No	No	No	No
2997	HPP0868	Male	Chinese	2019	52	Married	Secondary	Private	Urban	No	No	No	No	Yes	No	No	No	No	No
2998	HPP0870	Male	Malay	2020	24	Single	Secondary	Private	Urban	No	Yes	No	No	No	No	No	No	No	No

No	Patient ID	Gender	Ethnic group	Year of diagnosis	Age	Marital status	Education level	Occupation	Place of residence	History of psy illness	Tobacco	Alcohol	Cannabis	ATS	Inhalant	Sedative	Hallucinogens	Opioids	Kratom
2999	HPP0871	Male	Malay	2021	26	Others	Secondary	Private	Urban	No	Yes	Yes	Yes	No	No	No	No	No	No
3000	HPP0872	Female	Chinese	2019	43	Married	Secondary	Private	Urban	Yes	No	Yes	No	No	No	No	No	No	No
3001	HPP0873	Male	Malay	2021	23	Single	Secondary	Private	Urban	No	Yes	No	Yes	Yes	No	No	No	No	Yes
3002	HPP0874	Male	Malay	2019	46	Married	Secondary	Government	Urban	No	Yes	Yes	Yes	Yes	No	No	No	No	No
3003	HPP0875	Male	Malay	2021	31	Single	Secondary	Private	Urban	No	Yes	No	No	No	No	No	No	No	No
3004	HPP0876	Male	Malay	2019	22	Single	Secondary	Unemployed	Urban	No	Yes	No	Yes	Yes	No	No	No	No	No
3005	HPP0877	Male	Chinese	2018	35	Married	Tertiary	Private	Urban	No	Yes	No	No	No	No	No	No	No	No
3006	HPP0878	Male	Chinese	2021	50	Single	Secondary	Private	Urban	No	No	Yes	No	No	No	No	No	No	No
3007	HPP0879	Male	Chinese	2020	59	Married	Secondary	Self-employed	Urban	Yes	No	Yes	No	No	No	No	No	No	No
3008	HPP0880	Male	Malay	2019	24	Single	Secondary	Private	Urban	No	Yes	Yes	No	Yes	No	No	No	No	No
3009	HPP0881	Male	Malay	2018	22	Single	Tertiary	Unemployed	Urban	No	Yes	No	Yes	Yes	No	No	No	No	No
3010	HPP0882	Male	Chinese	2019	49	Married	Tertiary	Private	Urban	No	Yes	No	No	No	No	No	No	No	No
3011	HPP0886	Male	Malay	2020	27	Single	Secondary	Government	Urban	No	Yes	No	No	No	No	No	No	No	No
3012	HPP0887	Male	Chinese	2020	48	Single	Tertiary	Self-employed	Urban	No	Yes	Yes	No	No	No	No	No	No	No
3013	HPP0888	Male	Chinese	2018	20	Single	Secondary	Private	Urban	No	No	Yes	No	No	No	No	No	No	No
3014	HPP0890	Female	Chinese	2020	19	Single	Secondary	Unemployed	Urban	No	No	Yes	No	No	No	No	No	No	No
3015	HPP0891	Female	Chinese	2019	47	Married	Secondary	Unemployed	Urban	No	No	Yes	No	No	No	No	No	No	No
3016	HPP0893	Male	Malay	2018	25	Single	Tertiary	Unemployed	Urban	No	Yes	Yes	No	No	No	No	No	No	No
3017	HPP0895	Male	Malay	2021	30	Others	Tertiary	Private	Urban	No	Yes	No	No	No	No	No	No	No	No
3018	HPP0896	Male	Chinese	2021	59	Married	Secondary	Unemployed	Urban	No	No	Yes	No	No	No	No	No	No	No
3019	HPP0897	Male	Chinese	2020	26	Married	Tertiary	Private	Urban	Yes	No	No	No	Yes	No	No	No	No	No
3020	HPP0898	Female	Chinese	2018	45	Single	Tertiary	Private	Urban	No	No	Yes	No	No	No	No	No	No	No
3021	HPP0900	Female	Chinese	2020	24	Others	Secondary	Private	Urban	No	No	Yes	No	No	No	No	No	No	No
3022	HPP0901	Male	Chinese	2018	29	Others	Secondary	Self-employed	Urban	No	No	Yes	No	No	No	No	No	No	No
3023	HPP0902	Female	Indian	2019	31	Single	Secondary	Private	Urban	No	No	Yes	No	No	No	No	No	No	No
3024	HPP0903	Female	Chinese	2021	32	Single	Secondary	Self-employed	Urban	No	No	Yes	No	No	No	No	No	No	No
3025	HPP0906	Female	Chinese	2018	23	Single	Tertiary	Unemployed	Urban	No	No	Yes	No	No	No	No	No	No	No
3026	HPP0907	Female	Malay	2020	51	Married	Secondary	Unemployed	Urban	No	Yes	Yes	No	No	No	No	No	No	No
3027	HPP0908	Male	Chinese	2018	30	Others	Primary	Unemployed	Urban	Yes	No	No	No	Yes	No	No	No	No	No
3028	HPP0909	Male	Chinese	2018	41	Married	Secondary	Private	Urban	No	Yes	Yes	No	No	No	No	No	No	No
3029	HPP0910	Male	Indian	2020	23	Single	Secondary	Others	Urban	No	Yes	Yes	No	Yes	No	No	No	No	No
3030	HPP0911	Male	Chinese	2019	25	Single	Tertiary	Private	Urban	No	No	Yes	No	No	No	No	No	No	No
3031	HPP0912	Male	Chinese	2018	55	Others	No formal education	Others	Urban	No	No	No	No	No	No	No	No	Yes	No
3032	HPP0913	Male	Chinese	2018	27	Single	Secondary	Private	Urban	No	Yes	No	No	No	No	No	No	No	No
3033	HPP0914	Male	Chinese	2019	41	Single	Tertiary	Private	Urban	Yes	No	Yes	No	No	No	No	No	No	No
3034	HPP0915	Male	Chinese	2018	47	Married	Secondary	Self-employed	Urban	No	Yes	No	No	No	No	No	No	No	No
3035	HPP0916	Male	Malay	2018	29	Married	Secondary	Private	Urban	No	Yes	No	No	Yes	No	No	No	Yes	Yes
3036	HPP0918	Male	Indian	2019	26	Single	No formal education	Unemployed	Urban	No	No	No	Yes	Yes	No	No	No	No	No
3037	HPP0919	Male	Malay	2020	34	Others	Tertiary	Private	Urban	No	Yes	No	No	No	No	No	No	No	No
3038	HPP0920	Female	Indian	2020	28	Single	Tertiary	Government	Urban	No	No	Yes	No	No	No	No	No	No	No
3039	HPP0921	Male	Malay	2021	35	Married	Tertiary	Government	Urban	No	Yes	No	No	No	No	No	No	No	No
3040	HPP0922	Female	Indian	2018	53	Married	Secondary	Unemployed	Urban	No	No	Yes	No	No	No	No	No	No	No

No	Patient ID	Gender	Ethnic group	Year of diagnosis	Age	Marital status	Education level	Occupation	Place of residence	History of psy illness	Tobacco	Alcohol	Cannabis	ATS	Inhalant	Sedative	Hallucinogens	Opioids	Kratom
3041	HPP0927	Male	Indian	2020	33	Single	Tertiary	Private	Urban	Yes	No	Yes	No	No	No	No	No	No	No
3042	HPP0929	Male	Chinese	2021	25	Single	Secondary	Unemployed	Urban	Yes	No	Yes	No	No	No	No	No	No	No
3043	HPP0930	Female	Malay	2021	33	Married	Tertiary	Private	Urban	No	Yes	Yes	No	Yes	No	No	No	No	No
3044	HPP0932	Male	Malay	2019	32	Single	Secondary	Unemployed	Urban	Yes	Yes	No	No	No	No	No	No	No	No
3045	HPP0933	Male	Malay	2018	21	Single	Secondary	Private	Urban	No	Yes	No	No	No	No	No	No	No	No
3046	HPP0934	Male	Malay	2020	26	Single	Secondary	Private	Urban	Yes	Yes	No	No	No	No	No	No	No	No
3047	HPP0936	Male	Chinese	2018	41	Single	Secondary	Unemployed	Urban	No	Yes	Yes	Yes	Yes	No	No	No	Yes	No
3048	HPP0937	Female	Indian	2021	21	Single	Secondary	Private	Urban	Yes	Yes	Yes	No	No	No	No	No	No	No
3049	HPP0938	Male	Malay	2020	31	Single	Secondary	Unemployed	Urban	No	Yes	No	No	Yes	No	No	No	No	Yes
3050	HPP0939	Male	Malay	2020	23	Single	Secondary	Government	Urban	No	Yes	No	No	No	No	No	No	No	No
3051	HPP0940	Female	Chinese	2018	21	Single	Tertiary	Unemployed	Urban	No	No	Yes	No	No	No	No	No	No	No
3052	HPP0941	Male	Malay	2018	21	Single	Secondary	Unemployed	Urban	No	Yes	Yes	Yes	Yes	No	No	No	No	No
3053	HPP0942	Female	Chinese	2020	52	Single	Tertiary	Unemployed	Urban	Yes	Yes	Yes	No	No	No	No	No	No	No
3054	HPP0943	Female	Indian	2019	22	Single	Tertiary	Private	Urban	No	Yes	No	No	No	No	No	No	No	No
3055	HPP0944	Male	Chinese	2019	38	Single	Tertiary	Private	Urban	No	No	Yes	No	No	No	No	No	No	No
3056	HPP0945	Male	Chinese	2019	43	Single	Primary	Others	Urban	No	No	Yes	Yes	No	No	No	No	No	No
3057	HPP0946	Male	Malay	2021	30	Married	Secondary	Government	Urban	No	Yes	No	No	Yes	No	No	No	No	Yes
3058	HPP0947	Male	Malay	2018	44	Married	Secondary	Private	Urban	No	Yes	Yes	No	No	No	No	No	No	Yes
3059	HPP0948	Male	Chinese	2019	21	Single	Secondary	Unemployed	Urban	No	Yes	No	No	No	No	No	No	No	No
3060	HPP0949	Male	Malay	2021	35	Married	Secondary	Private	Urban	No	Yes	No	No	No	No	No	No	No	No
3061	HPP0950	Male	Chinese	2019	48	Single	Secondary	Private	Urban	No	Yes	Yes	No	Yes	No	No	No	Yes	No
3062	HPP0952	Male	Indian	2021	38	Single	Tertiary	Private	Urban	No	No	Yes	No	No	No	No	No	No	No
3063	HPP0953	Male	Malay	2021	23	Single	Secondary	Unemployed	Urban	No	Yes	No	No	No	No	No	No	No	No
3064	HPP0954	Male	Malay	2020	37	Others	Secondary	Unemployed	Urban	No	Yes	No	No	No	No	No	No	No	No
3065	HPP0955	Male	Indian	2020	41	Married	Secondary	Unemployed	Urban	No	Yes	No	Yes	Yes	No	No	No	No	No
3066	HPP0956	Male	Chinese	2020	25	Single	Secondary	Private	Urban	No	No	Yes	No	No	No	No	No	No	No
3067	HPP0957	Male	Malay	2020	31	Single	Secondary	Unemployed	Urban	No	Yes	No	No	Yes	No	No	No	No	No
3068	HPP0958	Male	Malay	2020	56	Married	Secondary	Private	Urban	No	Yes	No	No	No	No	No	No	No	No
3069	HPP0960	Male	Malay	2020	38	Single	Secondary	Private	Urban	No	Yes	No	No	Yes	No	No	No	No	No
3070	HPP0961	Male	Malay	2019	32	Married	Secondary	Private	Urban	Yes	Yes	No	No	No	No	No	No	Yes	No
3071	HPP0962	Male	Malay	2018	33	Single	Secondary	Private	Urban	No	Yes	No	Yes	Yes	No	No	No	No	No
3072	HPP0963	Male	Malay	2018	33	Single	Secondary	Private	Urban	No	No	No	Yes	No	No	No	No	No	No
3073	HPP0964	Male	Chinese	2018	28	Single	Secondary	Private	Urban	No	Yes	Yes	No	No	No	No	No	No	No
3074	HPP0965	Male	Malay	2019	31	Single	Secondary	Private	Urban	No	Yes	No	No	No	No	No	No	No	No
3075	HPP0966	Female	Chinese	2018	18	Single	Secondary	Unemployed	Urban	No	Yes	Yes	No	No	No	No	No	No	No
3076	HPP0967	Female	Chinese	2019	27	Married	Secondary	Private	Urban	No	No	Yes	No	No	No	No	No	No	No
3077	HPP0968	Male	Others	2020	25	Single	Primary	Private	Urban	No	Yes	Yes	No	No	No	No	No	No	No
3078	HPP0969	Female	Chinese	2019	21	Single	Tertiary	Private	Urban	No	No	Yes	No	No	No	No	No	No	No
3079	HPP0970	Female	Indian	2020	59	Others	No formal education	Private	Urban	No	No	No	No	No	No	Yes	No	No	No
3080	HPP0972	Female	Indian	2020	24	Others	Tertiary	Unemployed	Urban	No	No	Yes	No	No	No	No	No	No	No
3081	HPP0973	Female	Others	2020	46	Others	Secondary	Private	Urban	Yes	Yes	No	No	No	No	No	No	No	No
3082	HPP0975	Female	Chinese	2021	44	Others	Secondary	Unemployed	Urban	No	Yes	Yes	No	No	No	No	No	No	No

No	Patient ID	Gender	Ethnic group	Year of diagnosis	Age	Marital status	Education level	Occupation	Place of residence	History of psy illness	Tobacco	Alcohol	Cannabis	ATS	Inhalant	Sedative	Hallucinogens	Opioids	Kratom
3083	HPP0976	Male	Malay	2021	50	Others	Secondary	Others	Urban	No	Yes	No	No	Yes	No	No	No	No	No
3084	HPP0977	Male	Malay	2021	32	Married	Secondary	Others	Urban	No	Yes	No	Yes	No	No	No	No	No	No
3085	HPP0978	Male	Chinese	2019	55	Married	Secondary	Others	Urban	No	Yes	Yes	No	No	No	No	No	No	No
3086	HPP0979	Male	Malay	2018	37	Married	Secondary	Private	Urban	No	Yes	No	Yes	No	No	No	No	No	Yes
3087	HPP0980	Male	Malay	2019	40	Married	Tertiary	Government	Urban	No	Yes	No	No	No	No	No	No	No	No
3088	HPP0981	Male	Malay	2021	29	Married	Tertiary	Private	Urban	No	Yes	No	No	No	No	No	No	No	Yes
3089	HPP0982	Male	Chinese	2019	49	Married	Secondary	Private	Urban	Yes	No	Yes	No	No	No	No	No	No	No
3090	HPP0984	Male	Chinese	2018	41	Single	Secondary	Private	Urban	No	Yes	No	No	Yes	No	No	No	Yes	No
3091	HPP0986	Male	Indian	2021	52	Others	No formal education	Others	Urban	No	No	Yes	Yes	Yes	No	No	No	Yes	No
3092	HPP0987	Male	Chinese	2018	37	Married	Tertiary	Self-employed	Urban	No	Yes	Yes	No	No	No	No	No	No	No
3093	HPP0988	Male	Chinese	2019	39	Single	Secondary	Self-employed	Urban	No	Yes	Yes	No	Yes	No	No	No	No	No
3094	HPP0990	Male	Chinese	2020	44	Married	Tertiary	Private	Urban	Yes	Yes	Yes	No	No	No	No	No	No	No
3095	HPP0991	Male	Indian	2019	59	Others	Primary	Unemployed	Urban	Yes	Yes	Yes	Yes	No	No	No	No	Yes	No
3096	HPP0992	Male	Chinese	2021	26	Single	Secondary	Private	Urban	No	Yes	No	Yes	Yes	No	No	No	No	No
3097	HPP0993	Male	Chinese	2020	32	Others	Tertiary	Self-employed	Urban	No	No	Yes	No	No	No	No	No	No	No
3098	HPP0994	Male	Indian	2021	39	Married	Secondary	Unemployed	Urban	No	Yes	No	No	No	No	No	No	No	No
3099	HPP0996	Male	Malay	2020	36	Married	Secondary	Government	Urban	No	Yes	Yes	Yes	Yes	No	No	No	No	No
3100	HPP0998	Male	Malay	2021	24	Single	Secondary	Unemployed	Urban	Yes	No	No	No	Yes	No	No	No	No	Yes
3101	HPP0999	Male	Chinese	2020	27	Single	Tertiary	Private	Urban	No	No	Yes	No	No	No	No	No	No	No
3102	HPP1000	Male	Chinese	2021	32	Single	Tertiary	Private	Urban	No	Yes	Yes	No	Yes	No	No	No	No	No
3103	HPP1001	Male	Malay	2021	38	Others	No formal education	Others	Urban	No	No	No	No	Yes	No	No	No	Yes	No
3104	HPP1002	Male	Others	2020	33	Others	Tertiary	Private	Urban	Yes	Yes	Yes	No	No	No	No	No	No	No
3105	HPP1003	Male	Indian	2020	34	Single	Secondary	Private	Urban	No	Yes	Yes	No	No	No	No	No	No	No
3106	HPP1004	Male	Chinese	2019	27	Single	Tertiary	Private	Urban	No	Yes	Yes	No	No	No	No	No	No	No
3107	HPP1005	Female	Chinese	2020	30	Single	Tertiary	Private	Urban	No	No	Yes	No	No	No	No	No	No	No
3108	HPP1006	Female	Malay	2020	23	Married	Secondary	Private	Urban	No	No	No	No	Yes	No	No	No	No	No
3109	HPP1007	Female	Chinese	2018	22	Single	Tertiary	Unemployed	Urban	No	No	Yes	No	No	No	No	No	No	No
3110	HPP1008	Male	Malay	2020	44	Single	Tertiary	Private	Urban	No	Yes	No	No	No	No	No	No	No	No
3111	HPP1009	Male	Malay	2020	36	Single	Secondary	Private	Urban	No	Yes	Yes	No	Yes	No	No	No	No	No
3112	HPP1011	Male	Chinese	2020	26	Single	Tertiary	Private	Urban	No	Yes	Yes	No	No	No	No	No	No	No
3113	HPP1012	Male	Chinese	2018	19	Single	Tertiary	Unemployed	Urban	No	Yes	Yes	No	No	No	No	No	No	No
3114	HPP1013	Male	Indian	2020	46	Single	Secondary	Unemployed	Urban	No	Yes	No	Yes	Yes	No	No	No	Yes	Yes
3115	HPP1014	Male	Malay	2019	29	Single	Primary	Private	Urban	No	Yes	No	Yes	Yes	No	No	No	No	No
3116	HPP1015	Male	Indian	2018	37	Married	Secondary	Private	Urban	No	Yes	No	No	No	No	No	No	No	No
3117	HPP1016	Male	Malay	2019	33	Single	Secondary	Private	Urban	No	Yes	Yes	No	Yes	No	No	No	No	No
3118	HPP1017	Male	Chinese	2019	20	Single	Tertiary	Unemployed	Urban	No	Yes	Yes	No	No	No	No	No	No	No
3119	HPP1018	Male	Malay	2020	30	Single	Tertiary	Unemployed	Urban	Yes	No	No	No	Yes	No	No	No	No	No
3120	HPP1019	Male	Indian	2018	32	Single	Tertiary	Government	Urban	No	Yes	Yes	No	No	No	No	No	No	No
3121	HPP1021	Male	Malay	2018	30	Single	Tertiary	Private	Urban	No	Yes	Yes	No	No	No	No	No	No	No
3122	HPP1022	Male	Indian	2020	21	Single	No formal education	Others	Urban	No	No	No	Yes	Yes	No	No	No	No	No

No	Patient ID	Gender	Ethnic group	Year of diagnosis	Age	Marital status	Education level	Occupation	Place of residence	History of psy illness	Tobacco	Alcohol	Cannabis	ATS	Inhalant	Sedative	Hallucinogens	Opioids	Kratom
3123	HPP1023	Male	Chinese	2021	29	Single	Tertiary	Private	Urban	No	No	Yes	No	No	No	No	No	No	No
3124	HPP1024	Male	Indian	2018	33	Single	Tertiary	Unemployed	Urban	Yes	Yes	Yes	Yes	No	No	No	No	No	No
3125	HPP1025	Male	Malay	2021	54	Single	No formal education	Unemployed	Urban	No	No	No	No	Yes	No	No	No	No	No
3126	HPP1027	Male	Malay	2018	27	Single	Tertiary	Government	Urban	No	Yes	No	No	No	No	No	No	No	No
3127	HPP1029	Female	Indian	2020	19	Married	Secondary	Private	Urban	No	No	Yes	No	No	No	No	No	No	No
3128	HPP1031	Female	Chinese	2020	31	Single	Tertiary	Unemployed	Urban	Yes	Yes	Yes	No	No	No	No	No	No	No
3129	HPP1032	Female	Chinese	2021	43	Single	Secondary	Unemployed	Urban	Yes	Yes	No	No	No	No	No	No	No	No
3130	HPP1033	Female	Chinese	2019	21	Single	Tertiary	Private	Urban	No	No	Yes	No	No	No	No	No	No	No
3131	HPP1034	Female	Indian	2021	28	Single	Tertiary	Unemployed	Urban	No	Yes	Yes	No	No	No	No	No	No	No
3132	HPP1035	Female	Malay	2020	25	Single	Secondary	Private	Urban	No	Yes	No	No	Yes	No	No	No	No	No
3133	HPP1036	Female	Indian	2018	26	Others	Secondary	Private	Urban	No	No	Yes	No	No	No	No	No	No	No
3134	HPP1037	Female	Others	2021	40	Married	Secondary	Self-employed	Urban	No	No	No	No	Yes	No	No	No	No	No
3135	HPP1038	Female	Malay	2020	21	Single	Secondary	Unemployed	Urban	No	No	No	Yes	Yes	No	No	No	No	No
3136	HPP1039	Male	Malay	2018	17	Single	Tertiary	Private	Urban	No	Yes	No	No	Yes	No	No	No	No	No
3137	HPP1040	Male	Indian	2019	19	Single	Secondary	Private	Urban	No	No	Yes	No	No	No	No	No	No	No
3138	HPP1041	Female	Indian	2020	24	Others	Secondary	Unemployed	Urban	Yes	No	Yes	No	No	No	No	No	No	No
3139	HPP1042	Male	Malay	2020	24	Married	Secondary	Private	Urban	No	No	No	No	Yes	No	No	No	No	No
3140	HPP1043	Male	Chinese	2020	32	Single	Secondary	Private	Urban	No	No	Yes	No	No	No	No	No	No	No
3141	HPP1044	Male	Indian	2020	30	Married	Tertiary	Private	Urban	No	No	Yes	No	No	No	No	No	No	No
3142	HPP1047	Male	Malay	2019	50	Married	No formal education	Unemployed	Urban	No	Yes	No	Yes	Yes	No	No	No	No	No
3143	HPP1048	Male	Indian	2020	38	Single	Tertiary	Private	Urban	No	Yes	Yes	No	Yes	No	No	No	No	No
3144	HPP1049	Male	Malay	2019	49	Single	Secondary	Unemployed	Urban	No	Yes	No	No	Yes	No	No	No	Yes	No
3145	HPP1050	Male	Malay	2020	22	Single	Secondary	Unemployed	Urban	No	Yes	No	No	Yes	Yes	No	No	Yes	No
3146	HPP1052	Male	Chinese	2021	40	Others	Secondary	Unemployed	Urban	No	Yes	No	No	No	No	No	No	No	No
3147	HPP1054	Male	Indian	2021	48	Married	Secondary	Private	Urban	No	No	Yes	No	No	No	No	No	No	No
3148	HPP1055	Female	Others	2021	24	Others	Primary	Private	Urban	No	No	Yes	No	No	No	No	No	No	No
3149	HPP1056	Male	Malay	2021	38	Others	Secondary	Unemployed	Urban	No	Yes	No	No	Yes	No	No	No	Yes	No
3150	HPP1057	Female	Malay	2018	33	Others	Secondary	Private	Urban	No	No	No	No	No	No	No	No	Yes	No
3151	HPP1058	Male	Malay	2018	31	Married	Secondary	Private	Urban	No	Yes	No	No	No	No	No	No	No	No
3152	HPP1059	Male	Malay	2018	33	Single	No formal education	Others	Urban	Yes	Yes	No	No	Yes	No	No	No	No	No
3153	HPP1060	Female	Malay	2018	37	Married	Secondary	Private	Urban	No	Yes	Yes	No	No	No	No	No	No	No
3154	HPP1062	Male	Chinese	2021	49	Single	Secondary	Self-employed	Rural	No	No	No	No	Yes	No	No	No	No	No
3155	HPP1063	Male	Chinese	2020	45	Single	Secondary	Private	Urban	No	Yes	Yes	Yes	Yes	No	No	No	No	No
3156	HPP1064	Male	Chinese	2018	33	Married	Tertiary	Self-employed	Urban	No	No	Yes	No	No	No	No	No	No	No
3157	HPP1065	Female	Chinese	2021	23	Others	Secondary	Private	Urban	No	No	Yes	No	No	No	No	No	No	No
3158	HPP1067	Male	Malay	2021	22	Single	Tertiary	Unemployed	Urban	No	Yes	No	No	No	No	No	No	No	No
3159	HPP1069	Male	Indian	2019	46	Single	Tertiary	Unemployed	Urban	No	No	Yes	No	No	No	No	No	No	No
3160	HPP1070	Female	Indian	2019	34	Married	Primary	Unemployed	Urban	Yes	No	Yes	No	Yes	No	No	No	No	No
3161	HPP1071	Male	Chinese	2019	19	Single	Secondary	Others	Urban	No	No	Yes	No	No	No	No	No	No	No
3162	HPP1073	Male	Chinese	2021	33	Single	Secondary	Unemployed	Urban	No	Yes	Yes	No	Yes	No	No	No	No	No
3163	HPP1075	Male	Indian	2021	33	Single	Secondary	Private	Urban	No	Yes	Yes	Yes	Yes	No	No	No	Yes	No

No	Patient ID	Gender	Ethnic group	Year of diagnosis	Age	Marital status	Education level	Occupation	Place of residence	History of psy illness	Tobacco	Alcohol	Cannabis	ATS	Inhalant	Sedative	Hallucinogens	Opioids	Kratom
3164	HPP1077	Male	Indian	2019	47	Married	Secondary	Private	Urban	No	No	Yes	No	No	No	No	No	No	No
3165	HPP1079	Male	Malay	2019	59	Others	Tertiary	Private	Urban	Yes	Yes	Yes	No	No	No	No	No	No	No
3166	HPP1080	Male	Malay	2020	36	Single	No formal education	Unemployed	Urban	No	No	No	Yes	Yes	No	No	No	No	No
3167	HPP1081	Male	Malay	2020	35	Others	Tertiary	Unemployed	Urban	No	Yes	No	No	Yes	No	No	No	No	No
3168	HPP1082	Male	Indian	2019	41	Married	Tertiary	Unemployed	Urban	Yes	No	Yes	No	No	No	No	No	No	No
3169	HPP1083	Male	Chinese	2020	38	Married	Secondary	Private	Urban	No	No	Yes	No	No	No	No	No	No	No
3170	HPP1084	Male	Chinese	2019	33	Married	Secondary	Private	Urban	No	Yes	Yes	Yes	Yes	No	No	No	No	No
3171	HPP1085	Male	Chinese	2018	37	Single	Tertiary	Private	Urban	Yes	Yes	No	No	No	No	No	No	No	No
3172	HPP1086	Male	Chinese	2018	46	Married	Primary	Private	Urban	Yes	No	No	No	Yes	No	No	No	No	No
3173	HPP1087	Male	Chinese	2018	51	Others	Tertiary	Private	Urban	No	No	Yes	No	No	No	No	No	No	No
3174	HPP1089	Male	Malay	2018	27	Single	Secondary	Unemployed	Urban	No	Yes	No	No	No	No	No	No	No	No
3175	HPP1091	Male	Indian	2020	52	Married	Secondary	Private	Urban	No	Yes	Yes	No	No	No	No	No	No	No
3176	HPP1092	Male	Malay	2018	30	Single	Secondary	Private	Urban	No	No	No	Yes	No	No	No	No	No	No
3177	HPP1094	Male	Indian	2021	49	Married	Tertiary	Unemployed	Urban	Yes	Yes	Yes	No	No	No	No	No	No	No
3178	HPP1095	Male	Malay	2021	31	Married	Secondary	Private	Urban	No	No	No	Yes	No	No	No	No	No	No
3179	HPP1096	Male	Indian	2020	48	Married	Secondary	Private	Urban	No	No	Yes	No	No	No	No	No	No	No
3180	HPP1097	Male	Chinese	2020	55	Married	Secondary	Private	Urban	Yes	Yes	No	No	No	No	No	No	No	No
3181	HPP1098	Male	Others	2020	20	Single	Tertiary	Unemployed	Urban	No	No	Yes	No	No	No	No	No	No	No
3182	HPP1099	Male	Chinese	2021	24	Single	Tertiary	Private	Urban	No	No	No	Yes	No	No	No	No	No	No
3183	HPP1100	Female	Indian	2019	31	Married	Secondary	Unemployed	Urban	No	Yes	Yes	No	No	No	No	No	No	No
3184	HPP1101	Male	Indian	2018	33	Single	Secondary	Private	Urban	No	Yes	Yes	No	No	No	No	No	No	No
3185	HPP1102	Male	Others	2020	75	Married	Tertiary	Others	Urban	No	No	Yes	No	No	No	No	No	No	No
3186	HPP1103	Male	Malay	2018	23	Single	Secondary	Private	Urban	No	Yes	No	No	No	No	No	No	No	Yes
3187	HPP1104	Female	Chinese	2019	25	Single	Secondary	Private	Urban	No	No	Yes	No	No	No	No	No	No	No
3188	HPP1105	Female	Malay	2021	22	Single	Tertiary	Private	Urban	No	Yes	Yes	No	No	No	No	No	No	No
3189	HPP1106	Female	Chinese	2020	45	Others	Tertiary	Private	Urban	No	Yes	No	No	No	No	No	No	No	No
3190	HPP1107	Female	Malay	2021	38	Married	Secondary	Private	Urban	No	Yes	No	No	No	No	No	No	No	No
3191	HPP1108	Female	Malay	2020	24	Single	Tertiary	Private	Urban	Yes	Yes	No	Yes	No	No	No	No	No	No
3192	HPP1110	Female	Malay	2020	23	Single	Secondary	Private	Urban	Yes	No	Yes	No	No	No	No	No	No	No
3193	HPP1111	Female	Others	2018	21	Single	Tertiary	Unemployed	Urban	No	No	Yes	No	No	No	No	No	No	No
3194	HPP1112	Male	Chinese	2019	19	Single	Secondary	Unemployed	Urban	No	No	Yes	No	No	No	No	No	No	No
3195	HPP1113	Male	Indian	2021	20	Single	Secondary	Private	Urban	No	Yes	Yes	No	No	No	No	No	No	No
3196	HPP1114	Male	Malay	2019	19	Single	No formal education	Unemployed	Urban	Yes	No	No	No	Yes	No	No	No	No	Yes
3197	HPP1115	Male	Indian	2021	24	Single	Secondary	Private	Urban	No	Yes	No	No	No	No	No	No	No	No
3198	HPP1117	Male	Malay	2018	28	Married	Tertiary	Private	Urban	No	Yes	No	No	No	No	No	No	No	No
3199	HPP1118	Male	Malay	2021	41	Single	Secondary	Private	Urban	No	Yes	No	Yes	Yes	No	No	No	No	No
3200	HPP1119	Male	Chinese	2020	50	Single	Secondary	Private	Urban	No	Yes	Yes	Yes	No	No	No	No	No	No
3201	HPP1120	Male	Malay	2020	44	Others	Secondary	Private	Urban	No	Yes	No	No	No	No	No	No	No	No
3202	HPP1122	Male	Chinese	2018	51	Others	No formal education	Private	Urban	Yes	Yes	No	No	Yes	No	No	No	No	No
3203	HPP1123	Male	Chinese	2021	45	Married	Secondary	Self-employed	Urban	Yes	No	Yes	No	No	No	No	No	No	No

No	Patient ID	Gender	Ethnic group	Year of diagnosis	Age	Marital status	Education level	Occupation	Place of residence	History of psy illness	Tobacco	Alcohol	Cannabis	ATS	Inhalant	Sedative	Hallucinogens	Opioids	Kratom
3204	HPP1124	Male	Chinese	2019	48	Single	Secondary	Unemployed	Urban	No	Yes	Yes	No	No	No	No	No	No	No
3205	HPP1125	Male	Chinese	2018	35	Single	Tertiary	Unemployed	Urban	Yes	Yes	Yes	No	No	No	No	No	No	No
3206	HPP1127	Male	Chinese	2020	40	Single	Secondary	Unemployed	Urban	Yes	Yes	No	No	No	No	No	No	No	No
3207	HPP1128	Male	Malay	2019	56	Others	Secondary	Unemployed	Urban	No	Yes	No	No	No	No	Yes	No	No	No
3208	HPP1133	Male	Malay	2019	58	Married	Secondary	Unemployed	Urban	No	Yes	No	No	No	No	No	No	No	No
3209	HPP1134	Male	Indian	2021	22	Single	Secondary	Private	Urban	No	Yes	No	No	No	No	No	No	No	No
3210	HPP1136	Male	Chinese	2019	38	Single	Primary	Private	Urban	Yes	Yes	No	No	No	No	No	No	No	No
3211	HPP1137	Male	Malay	2020	46	Others	Secondary	Private	Urban	No	Yes	No	Yes	No	No	No	No	No	Yes
3212	HPP1139	Male	Indian	2020	48	Married	Primary	Private	Urban	No	Yes	Yes	No	No	No	No	No	No	No
3213	HPP1140	Male	Indian	2018	44	Single	No formal education	Unemployed	Urban	No	Yes	Yes	Yes	No	No	No	No	No	No
3214	HPP1143	Female	Chinese	2020	38	Married	Tertiary	Unemployed	Urban	No	No	Yes	No	No	No	No	No	No	No
3215	HPP1144	Female	Chinese	2018	32	Others	No formal education	Unemployed	Urban	No	No	Yes	No	No	No	No	No	No	No
3216	HPP1145	Female	Indian	2020	35	Single	Tertiary	Unemployed	Urban	No	Yes	Yes	No	Yes	No	No	No	No	No
3217	HPP1146	Female	Indian	2020	36	Married	Secondary	Unemployed	Urban	No	No	Yes	No	No	No	No	No	Yes	No
3218	HPP1147	Female	Others	2018	34	Single	No formal education	Self-employed	Urban	No	No	No	No	Yes	No	No	No	No	No
3219	HPP1149	Male	Malay	2020	32	Single	Secondary	Private	Urban	Yes	Yes	Yes	No	No	No	No	No	No	No
3220	HPP1151	Male	Malay	2018	19	Single	Secondary	Unemployed	Urban	Yes	Yes	Yes	No	Yes	No	No	No	No	Yes
3221	HPP1155	Male	Chinese	2018	40	Married	Secondary	Private	Urban	No	Yes	No	No	No	No	No	No	No	No
3222	HPP11557	Male	Malay	2018	22	Single	Tertiary	Private	Urban	No	Yes	Yes	No	No	No	No	No	No	No
3223	HPP1156	Male	Malay	2018	28	Married	Secondary	Government	Urban	No	Yes	No	No	No	No	No	No	No	No
3224	HPP1157	Female	Others	2019	23	Single	Secondary	Private	Urban	No	No	Yes	No	No	No	No	No	No	No
3225	HPP1160	Male	Malay	2020	19	Single	Secondary	Private	Urban	No	Yes	No	Yes	No	No	No	No	No	Yes
3226	HPP1161	Male	Chinese	2020	27	Single	Secondary	Unemployed	Urban	No	Yes	No	No	Yes	No	No	No	Yes	No
3227	HPP1164	Male	Chinese	2020	56	Married	Secondary	Others	Urban	Yes	No	Yes	No	No	No	No	No	No	No
3228	HPP1165	Male	Malay	2019	51	Married	Secondary	Private	Urban	Yes	Yes	No	No	No	No	No	No	No	No
3229	HPP1166	Male	Indian	2020	44	Others	Secondary	Private	Urban	No	Yes	No	No	Yes	No	No	No	No	No
3230	HPP1168	Male	Chinese	2018	26	Single	Secondary	Unemployed	Urban	No	Yes	No	Yes	Yes	No	No	No	No	No
3231	HPP1169	Male	Malay	2019	28	Single	Tertiary	Self-employed	Urban	No	Yes	Yes	No	No	No	No	No	No	No
3232	HPP1170	Male	Chinese	2020	34	Single	Secondary	Self-employed	Urban	No	Yes	No	No	No	No	No	No	No	No
3233	HPP1171	Male	Chinese	2018	24	Single	Secondary	Unemployed	Urban	Yes	Yes	Yes	No	Yes	No	No	No	Yes	No
3234	HPP1172	Male	Chinese	2021	49	Married	Secondary	Others	Urban	Yes	Yes	No	No	No	No	No	No	No	No
3235	HPP1173	Male	Malay	2020	39	Married	Tertiary	Private	Urban	No	No	Yes	No	No	No	No	No	No	No
3236	HPP1174	Male	Chinese	2020	25	Single	No formal education	Private	Urban	No	Yes	No	Yes	No	No	No	No	Yes	No
3237	HPP1175	Male	Chinese	2021	49	Married	Secondary	Self-employed	Urban	No	Yes	No	No	No	No	No	No	No	No
3238	HPP1176	Male	Chinese	2018	52	Single	Primary	Unemployed	Urban	No	No	No	No	Yes	No	Yes	No	Yes	No
3239	HPP1177	Male	Chinese	2020	27	Single	Tertiary	Private	Urban	No	No	Yes	No	No	No	No	No	No	No
3240	HPP1178	Female	Others	2021	42	Others	Secondary	Private	Urban	Yes	No	Yes	No	No	No	No	No	No	No
3241	HPP1179	Male	Indian	2020	38	Married	Tertiary	Unemployed	Urban	No	No	Yes	No	Yes	No	No	No	No	No

No	Patient ID	Gender	Ethnic group	Year of diagnosis	Age	Marital status	Education level	Occupation	Place of residence	History of psy illness	Tobacco	Alcohol	Cannabis	ATS	Inhalant	Sedative	Hallucinogens	Opioids	Kratom
3242	HPP1180	Female	Chinese	2019	21	Single	Tertiary	Unemployed	Urban	No	No	Yes	No	No	No	No	No	No	No
3243	HPP1183	Female	Chinese	2019	32	Married	Secondary	Unemployed	Urban	Yes	No	Yes	No	No	No	No	No	No	No
3244	HPP1185	Female	Chinese	2021	37	Others	Tertiary	Private	Urban	Yes	No	Yes	No	No	No	No	No	No	No
3245	HPP1186	Female	Malay	2020	29	Married	Secondary	Self-employed	Urban	No	Yes	No	No	No	No	No	No	No	No
3246	HPP1187	Female	Indian	2019	52	Married	Secondary	Private	Urban	No	No	Yes	No	No	No	No	No	No	No
3247	HPP1188	Female	Chinese	2018	46	Married	Secondary	Private	Urban	Yes	Yes	Yes	No	No	No	No	No	No	No
3248	HPP1189	Female	Malay	2020	35	Married	Primary	Unemployed	Urban	No	Yes	No	No	Yes	No	No	No	Yes	No
3249	HPP1190	Female	Chinese	2018	44	Others	Secondary	Private	Urban	Yes	Yes	Yes	No	No	No	No	No	No	No
3250	HPP1192	Male	Chinese	2018	42	Single	Secondary	Unemployed	Urban	Yes	No	Yes	No	No	No	No	No	No	No
3251	HPP1195	Male	Others	2019	54	Single	No formal education	Others	Urban	Yes	Yes	Yes	No	No	No	No	No	No	No
3252	HPP1196	Male	Chinese	2019	36	Single	Tertiary	Private	Urban	Yes	Yes	No	No	No	No	No	No	No	No
3253	HPP1198	Male	Chinese	2018	55	Others	Secondary	Unemployed	Urban	No	Yes	No	No	No	No	No	No	No	No
3254	HPP1201	Male	Chinese	2020	35	Married	No formal education	Unemployed	Urban	Yes	Yes	No	No	No	No	No	No	No	No
3255	HPP1202	Male	Chinese	2018	40	Married	Tertiary	Private	Urban	No	Yes	Yes	No	No	No	No	No	No	No
3256	HPP1203	Male	Indian	2018	24	Single	Secondary	Private	Urban	No	No	Yes	No	No	No	No	No	No	No
3257	HPP1205	Male	Malay	2018	19	Single	Secondary	Unemployed	Urban	No	Yes	No	No	No	No	No	No	No	No
3258	HPP1206	Male	Chinese	2021	35	Single	No formal education	Unemployed	Urban	No	No	Yes	No	No	No	No	No	No	No
3259	HPP1208	Male	Malay	2021	50	Married	Tertiary	Private	Urban	Yes	Yes	No	No	No	No	No	No	No	No
3260	HPP1209	Male	Chinese	2018	39	Single	Tertiary	Private	Urban	No	No	Yes	No	No	No	No	No	No	No
3261	HPP1210	Male	Indian	2021	48	Single	No formal education	Private	Urban	No	Yes	No	Yes	No	No	No	No	Yes	No
3262	HPP1211	Male	Indian	2021	56	Married	Secondary	Private	Urban	Yes	No	Yes	No	No	No	No	No	No	No
3263	HPP1213	Male	Chinese	2021	39	Married	Tertiary	Private	Urban	Yes	No	Yes	No	No	No	No	No	No	No
3264	HPP1214	Male	Malay	2020	40	Single	No formal education	Unemployed	Urban	Yes	No	No	Yes	No	No	No	No	Yes	Yes
3265	HPP1218	Male	Indian	2021	56	Married	Secondary	Private	Urban	Yes	No	Yes	No	No	No	No	No	No	No
3266	HPP1220	Male	Indian	2021	41	Others	Secondary	Unemployed	Urban	No	Yes	Yes	Yes	Yes	No	No	No	No	Yes
3267	HPP1222	Male	Malay	2020	32	Married	Secondary	Private	Urban	Yes	Yes	No	No	No	No	No	No	No	Yes
3268	HPP1224	Male	Indian	2019	35	Single	Secondary	Unemployed	Urban	Yes	Yes	Yes	No	No	No	No	No	No	No
3269	HPP1226	Male	Chinese	2019	24	Single	Tertiary	Private	Urban	No	Yes	Yes	No	Yes	No	No	No	No	No
3270	HPP1228	Male	Indian	2020	49	Single	Secondary	Unemployed	Urban	Yes	No	Yes	No	No	No	No	No	No	No
3271	HPP1229	Female	Chinese	2020	20	Single	Secondary	Private	Urban	No	Yes	Yes	No	No	No	No	No	No	No
3272	HPP1230	Female	Chinese	2020	52	Married	Secondary	Private	Urban	Yes	Yes	No	No	No	No	No	No	No	No
3273	HPP1231	Female	Chinese	2018	24	Single	Tertiary	Unemployed	Urban	Yes	Yes	Yes	No	No	No	No	No	No	No
3274	HPP1232	Female	Chinese	2021	51	Married	Secondary	Private	Urban	No	Yes	No	No	No	No	No	No	No	No
3275	HPP1233	Female	Chinese	2020	41	Single	Secondary	Unemployed	Urban	Yes	Yes	Yes	No	No	No	No	No	No	No
3276	HPP1234	Female	Chinese	2019	21	Single	Tertiary	Unemployed	Urban	Yes	No	Yes	No	No	No	No	No	No	No
3277	HPP1236	Female	Chinese	2020	49	Others	Secondary	Private	Urban	No	No	Yes	No	No	No	No	No	No	No
3278	HPP1237	Female	Indian	2020	23	Married	Secondary	Private	Urban	No	No	Yes	No	No	No	No	No	No	No
3279	HPP1238	Female	Malay	2020	36	Married	Secondary	Private	Urban	No	No	Yes	No	No	No	No	No	No	No

No	Patient ID	Gender	Ethnic group	Year of diagnosis	Age	Marital status	Education level	Occupation	Place of residence	History of psy illness	Tobacco	Alcohol	Cannabis	ATS	Inhalant	Sedative	Hallucinogens	Opioids	Kratom
3280	HPP1240	Female	Indian	2019	33	Others	Secondary	Private	Urban	No	No	Yes	No	No	No	No	No	No	No
3281	HPP1241	Male	Chinese	2018	18	Single	Tertiary	Unemployed	Urban	Yes	No	Yes	Yes	No	No	No	No	No	No
3282	HPP1242	Male	Chinese	2018	55	Others	Secondary	Private	Urban	No	Yes	No	No	No	No	No	No	No	No
3283	HPP1243	Male	Malay	2018	49	Single	Primary	Private	Urban	No	Yes	No	No	No	No	No	No	Yes	No
3284	HPP1244	Male	Malay	2021	43	Single	Secondary	Unemployed	Urban	No	Yes	No	No	Yes	No	No	No	No	Yes
3285	HPP1245	Male	Chinese	2018	47	Single	Secondary	Unemployed	Urban	No	Yes	No	Yes	Yes	No	No	No	Yes	No
3286	HPP1251	Male	Chinese	2018	26	Single	Tertiary	Private	Urban	Yes	No	Yes	No	No	No	No	No	No	No
3287	HPP1252	Male	Malay	2019	34	Married	Tertiary	Government	Urban	No	Yes	No	No	No	No	No	No	No	No
3288	HPP1253	Male	Chinese	2021	34	Married	Primary	Private	Urban	No	Yes	Yes	No	No	No	No	No	No	No
3289	HPP1256	Female	Chinese	2021	41	Others	Primary	Private	Urban	Yes	Yes	Yes	No	No	No	No	No	No	No
3290	HPP1257	Male	Malay	2018	31	Single	Secondary	Private	Urban	No	Yes	Yes	Yes	No	No	No	No	No	No
3291	HPP1259	Male	Malay	2018	24	Single	Secondary	Private	Urban	No	Yes	No	No	Yes	Yes	No	No	No	No
3292	HPP1260	Male	Chinese	2018	54	Married	Secondary	Private	Urban	Yes	Yes	Yes	No	No	No	No	No	No	No
3293	HPP1262	Male	Malay	2021	55	Others	Tertiary	Government	Urban	Yes	Yes	No	No	No	No	No	No	No	No
3294	HPP1265	Male	Indian	2020	30	Single	Secondary	Private	Urban	No	Yes	No	No	Yes	No	No	No	No	No
3295	HPP1266	Male	Indian	2019	36	Single	Secondary	Private	Urban	No	Yes	Yes	No	No	No	No	No	No	Yes
3296	HPP1267	Male	Chinese	2018	57	Single	Primary	Unemployed	Urban	No	Yes	No	No	No	No	No	No	No	No
3297	HPP1268	Male	Malay	2019	32	Single	Tertiary	Private	Urban	No	Yes	No	Yes	Yes	No	No	No	No	Yes
3298	HPP1270	Male	Chinese	2020	56	Married	Secondary	Self-employed	Urban	No	Yes	No	No	No	No	No	No	No	No
3299	HPP1271	Male	Malay	2019	36	Married	Secondary	Private	Urban	No	Yes	No	No	Yes	No	No	No	No	No
3300	HPP1273	Male	Indian	2018	32	Single	Primary	Unemployed	Urban	No	Yes	No	No	No	No	No	No	No	No
3301	HPP1274	Male	Chinese	2021	55	Married	Tertiary	Private	Urban	No	Yes	Yes	No	No	No	No	No	No	No
3302	HPP1275	Male	Malay	2020	54	Married	Primary	Others	Urban	No	Yes	No	No	No	No	No	No	No	No
3303	HPP1276	Male	Malay	2021	22	Single	Secondary	Private	Urban	No	Yes	No	No	No	No	No	No	No	No
3304	HPP1277	Male	Indian	2019	21	Single	Tertiary	Unemployed	Urban	Yes	No	Yes	No	No	No	No	No	No	No
3305	HPP1278	Male	Indian	2021	28	Single	Secondary	Private	Urban	No	Yes	Yes	No	No	No	No	No	No	No
3306	HPP1279	Female	Others	2021	36	Married	Primary	Private	Urban	Yes	Yes	Yes	No	No	No	No	No	No	No
3307	HPP1281	Female	Others	2018	38	Married	Secondary	Unemployed	Urban	No	No	Yes	No	No	No	No	No	No	No
3308	HPP1282	Female	Malay	2018	28	Single	Tertiary	Unemployed	Urban	No	Yes	No	No	Yes	No	No	No	No	No
3309	HPP1283	Female	Chinese	2018	27	Single	Tertiary	Unemployed	Urban	No	Yes	No	Yes	No	No	No	No	No	No
3310	HPP1284	Female	Chinese	2021	37	Married	Secondary	Self-employed	Urban	No	Yes	Yes	No	No	No	No	No	No	No
3311	HPP1285	Female	Malay	2019	26	Single	Tertiary	Private	Urban	No	Yes	No	No	No	No	No	No	No	No
3312	HPP1286	Female	Indian	2020	23	Single	Secondary	Private	Urban	No	No	Yes	No	No	No	No	No	No	No
3313	HPP1288	Male	Malay	2020	26	Single	Tertiary	Private	Urban	No	Yes	No	No	No	No	No	No	No	No
3314	HPP1289	Male	Chinese	2021	49	Others	Secondary	Private	Urban	Yes	No	Yes	No	No	No	No	No	No	No
3315	HPP1290	Male	Malay	2018	44	Married	Tertiary	Private	Urban	No	Yes	No	No	No	No	No	No	No	No
3316	HPP1291	Male	Chinese	2019	51	Single	No formal education	Unemployed	Urban	No	No	No	No	No	No	No	No	Yes	No
3317	HPP1292	Male	Malay	2020	33	Married	Secondary	Private	Urban	No	Yes	No	No	No	No	No	No	No	No
3318	HPP1295	Male	Chinese	2020	49	Married	Secondary	Unemployed	Urban	Yes	Yes	No	No	No	No	No	No	No	No
3319	HPP1296	Male	Indian	2018	29	Single	Tertiary	Private	Urban	Yes	Yes	Yes	No	Yes	No	No	No	No	No

No	Patient ID	Gender	Ethnic group	Year of diagnosis	Age	Marital status	Education level	Occupation	Place of residence	History of psy illness	Tobacco	Alcohol	Cannabis	ATS	Inhalant	Sedative	Hallucinogens	Opioids	Kratom
3320	HPP1297	Male	Malay	2019	35	Others	No formal education	Private	Urban	No	Yes	Yes	No	No	No	No	No	No	No
3321	HPP1298	Male	Chinese	2019	43	Others	Tertiary	Unemployed	Urban	No	No	Yes	No	No	No	No	No	No	No
3322	HPP1299	Male	Malay	2019	20	Single	Tertiary	Unemployed	Urban	No	No	Yes	No	No	No	No	No	No	No
3323	HPP1300	Male	Chinese	2018	27	Single	No formal education	Government	Urban	No	Yes	No	No	No	No	No	No	No	No
3324	HPP1301	Male	Malay	2021	31	Married	Tertiary	Private	Urban	No	No	No	Yes	No	No	No	No	No	No
3325	HPP1302	Male	Chinese	2019	31	Single	Secondary	Private	Urban	No	No	Yes	No	No	No	No	No	No	No
3326	HPP1303	Male	Malay	2020	42	Others	No formal education	Others	Urban	No	No	No	No	Yes	No	No	No	No	No
3327	HPP1304	Female	Chinese	2019	18	Single	Tertiary	Private	Urban	Yes	Yes	Yes	No	No	No	No	No	No	No
3328	HPP1305	Male	Malay	2021	23	Others	Secondary	Unemployed	Urban	No	Yes	No	No	No	No	No	No	No	No
3329	HPP1306	Female	Others	2019	22	Single	No formal education	Others	Urban	No	Yes	No	No	Yes	No	No	No	No	No
3330	HPP1307	Male	Malay	2020	57	Married	Tertiary	Private	Urban	No	Yes	No	No	No	No	No	No	No	No
3331	HPP1308	Female	Chinese	2018	53	Married	Secondary	Unemployed	Urban	Yes	No	Yes	No	No	No	No	No	No	No
3332	HPP1309	Male	Chinese	2018	28	Single	Tertiary	Private	Urban	No	No	Yes	No	No	No	No	No	No	No
3333	HPP1310	Male	Indian	2018	45	Others	Tertiary	Private	Urban	No	Yes	Yes	No	No	No	No	No	No	No
3334	HPP1311	Male	Chinese	2021	30	Single	Tertiary	Unemployed	Urban	No	No	Yes	No	No	No	No	No	No	No
3335	HPP1312	Female	Chinese	2021	28	Single	Tertiary	Unemployed	Urban	No	No	Yes	No	No	No	No	No	No	No
3336	HPP1313	Male	Indian	2018	44	Married	Tertiary	Private	Urban	No	No	Yes	No	No	No	No	No	No	No
3337	HPP1314	Female	Chinese	2019	32	Married	Secondary	Self-employed	Urban	No	No	Yes	No	No	No	No	No	No	No
3338	HPP1315	Male	Malay	2020	20	Single	Secondary	Private	Urban	No	Yes	No	No	Yes	No	No	No	No	No
3339	HPP1318	Male	Chinese	2018	21	Single	Tertiary	Unemployed	Urban	No	No	Yes	No	No	No	No	No	No	No
3340	HPP1319	Female	Chinese	2019	37	Single	Secondary	Private	Urban	No	No	Yes	No	No	No	No	No	No	No
3341	HPP1320	Female	Chinese	2020	59	Single	Secondary	Private	Urban	Yes	Yes	No	No	No	No	No	No	No	No
3342	HPP1321	Male	Malay	2020	24	Single	Secondary	Private	Urban	No	Yes	No	No	No	No	No	No	No	Yes
3343	HPP1324	Female	Chinese	2020	23	Single	Tertiary	Private	Urban	No	No	Yes	No	No	No	No	No	No	No
3344	HPP1326	Female	Chinese	2019	28	Single	Tertiary	Self-employed	Urban	No	No	Yes	No	No	No	No	No	No	No
3345	HPP1327	Male	Malay	2020	25	Single	Secondary	Unemployed	Urban	No	Yes	No	Yes	No	No	No	No	No	Yes
3346	HPP1328	Male	Malay	2021	29	Single	Secondary	Unemployed	Urban	Yes	Yes	No	Yes	No	No	No	No	Yes	No
3347	HPP1329	Male	Chinese	2019	46	Married	Secondary	Private	Urban	Yes	No	Yes	No	No	No	No	No	No	No
3348	HPP1331	Female	Chinese	2019	31	Others	Secondary	Private	Urban	No	No	Yes	No	No	No	No	No	No	No
3349	HPP1332	Male	Indian	2020	54	Others	No formal education	Unemployed	Urban	No	Yes	Yes	No	Yes	No	No	No	Yes	Yes
3350	HPP1333	Male	Malay	2019	24	Single	Tertiary	Private	Urban	No	Yes	Yes	No	No	No	No	No	No	No
3351	HPP1334	Female	Chinese	2020	52	Others	Secondary	Unemployed	Urban	No	No	Yes	No	No	No	No	No	No	No
3352	HPP1335	Male	Malay	2020	47	Single	Secondary	Unemployed	Urban	No	Yes	No	No	No	No	No	No	No	No
3353	HPP1336	Female	Chinese	2019	37	Others	Secondary	Private	Urban	No	Yes	Yes	No	No	No	No	No	No	No
3354	HPP1337	Male	Chinese	2019	42	Single	Tertiary	Private	Urban	No	No	Yes	No	No	No	No	No	No	No
3355	HPP1338	Female	Malay	2019	22	Single	Secondary	Private	Urban	No	No	No	No	No	No	No	No	No	No
3356	HPP1341	Male	Chinese	2020	51	Single	No formal education	Others	Urban	Yes	No	Yes	No	Yes	No	No	No	Yes	No

No	Patient ID	Gender	Ethnic group	Year of diagnosis	Age	Marital status	Education level	Occupation	Place of residence	History of psy illness	Tobacco	Alcohol	Cannabis	ATS	Inhalant	Sedative	Hallucinogens	Opioids	Kratom
3357	HPP1343	Male	Indian	2019	40	Married	Tertiary	Government	Urban	No	Yes	No	No	No	No	No	No	No	No
3358	HPP1344	Male	Malay	2021	32	Single	Tertiary	Private	Urban	No	Yes	No	No	No	No	No	No	No	No
3359	HPP1345	Male	Malay	2018	29	Married	Tertiary	Private	Urban	No	Yes	No	No	No	No	No	No	No	No
3360	HPP1346	Male	Indian	2021	27	Single	Tertiary	Private	Urban	No	Yes	No	Yes	No	No	No	No	No	No
3361	HPP1347	Male	Malay	2021	44	Others	Secondary	Private	Urban	No	Yes	No	No	No	No	No	No	No	No
3362	HPP1349	Male	Indian	2018	42	Married	No formal education	Private	Urban	No	Yes	No	Yes	Yes	No	No	No	No	No
3363	HPP1350	Male	Malay	2018	32	Married	Tertiary	Private	Urban	Yes	Yes	Yes	No	No	No	No	No	No	No
3364	HPP1352	Male	Indian	2019	30	Married	Secondary	Self-employed	Urban	No	Yes	Yes	Yes	No	No	No	No	No	No
3365	HPP1354	Male	Indian	2020	32	Single	Primary	Private	Urban	No	Yes	No	No	Yes	No	No	No	No	No
3366	HPP1357	Male	Chinese	2019	28	Single	Tertiary	Unemployed	Urban	No	No	Yes	No	No	No	No	No	No	No
3367	HPP1358	Male	Malay	2020	29	Others	Secondary	Private	Urban	No	Yes	No	No	No	No	No	No	No	Yes
3368	HPP1359	Male	Indian	2019	30	Single	Tertiary	Unemployed	Urban	Yes	No	Yes	No	No	No	No	No	No	No
3369	HPP1361	Male	Malay	2020	29	Single	Secondary	Private	Urban	No	Yes	No	No	No	No	No	No	No	Yes
3370	HPP1362	Male	Chinese	2019	46	Others	Secondary	Private	Urban	No	Yes	No	No	No	No	No	No	No	No
3371	HPP1363	Male	Indian	2018	56	Married	No formal education	Private	Urban	Yes	No	Yes	No	No	No	No	No	No	No
3372	HPP1364	Male	Malay	2018	48	Married	Secondary	Private	Urban	No	Yes	No	No	No	No	No	No	No	No
3373	HPP1365	Male	Chinese	2019	24	Single	Secondary	Private	Urban	Yes	No	Yes	No	No	No	No	No	No	No
3374	HPP1367	Male	Indian	2020	23	Single	Secondary	Private	Urban	No	No	No	Yes	Yes	No	No	No	No	No
3375	HPP1368	Male	Others	2021	37	Married	No formal education	Private	Urban	No	No	Yes	No	No	No	No	No	No	No
3376	HPP1369	Female	Malay	2018	22	Single	Secondary	Private	Urban	No	No	No	No	No	No	Yes	No	No	No
3377	HPP1370	Female	Chinese	2018	44	Single	Secondary	Private	Urban	No	No	Yes	No	No	No	No	No	No	No
3378	HPP1372	Female	Others	2020	20	Single	Secondary	Private	Urban	No	No	Yes	No	No	No	No	No	No	No
3379	HPP1374	Female	Chinese	2020	40	Married	No formal education	Unemployed	Urban	No	No	Yes	No	No	No	No	No	No	No
3380	HPP1375	Male	Indian	2019	27	Married	Tertiary	Private	Urban	No	Yes	No	Yes	No	No	No	No	No	No
3381	HPP1376	Male	Malay	2019	34	Single	Tertiary	Private	Urban	No	Yes	No	No	No	No	No	No	No	No
3382	HPP1377	Male	Malay	2018	42	Single	Secondary	Private	Urban	No	Yes	No	No	No	No	No	No	No	No
3383	HPP1378	Male	Malay	2018	24	Single	Secondary	Unemployed	Urban	No	No	No	No	No	No	No	No	No	Yes
3384	HPP1379	Male	Malay	2021	34	Single	Secondary	Private	Urban	Yes	Yes	No	No	No	No	No	No	No	No
3385	HPP1380	Male	Malay	2020	29	Married	Secondary	Private	Urban	No	Yes	No	No	No	No	No	No	No	No
3386	HPP1381	Male	Indian	2019	31	Single	Secondary	Private	Urban	No	Yes	No	No	No	Yes	No	No	No	No
3387	HPP1382	Male	Malay	2018	56	Married	Primary	Unemployed	Urban	No	Yes	No	No	No	No	No	No	No	No
3388	HPP1384	Male	Malay	2018	38	Single	Secondary	Private	Urban	No	Yes	No	No	No	No	No	No	No	No
3389	HPP1385	Male	Chinese	2018	36	Single	Secondary	Unemployed	Urban	No	Yes	Yes	No	Yes	No	No	No	No	No
3390	HPP1387	Female	Malay	2020	22	Others	Tertiary	Private	Urban	No	Yes	Yes	No	No	No	No	No	No	No
3391	HPP1388	Female	Malay	2020	21	Single	Secondary	Private	Urban	No	Yes	No	No	No	No	No	No	No	No
3392	HPP1389	Male	Indian	2021	21	Single	Primary	Private	Urban	No	Yes	Yes	Yes	Yes	No	No	No	No	No
3393	HPP1390	Female	Indian	2019	38	Single	Tertiary	Private	Urban	No	No	Yes	No	No	No	No	No	No	No
3394	HPP1391	Male	Indian	2021	21	Married	Secondary	Private	Urban	No	No	Yes	Yes	Yes	No	No	No	No	No
3395	HPP1392	Female	Chinese	2019	56	Single	Secondary	Private	Urban	No	No	Yes	No	No	No	No	No	No	No

No	Patient ID	Gender	Ethnic group	Year of diagnosis	Age	Marital status	Education level	Occupation	Place of residence	History of psy illness	Tobacco	Alcohol	Cannabis	ATS	Inhalant	Sedative	Hallucinogens	Opioids	Kratom
3396	HPP1394	Male	Malay	2019	32	Others	No formal education	Unemployed	Urban	No	No	Yes	No	Yes	No	No	No	No	No
3397	HPP1395	Male	Indian	2020	32	Single	Secondary	Private	Urban	No	Yes	Yes	Yes	Yes	No	No	No	Yes	No
3398	HPP1396	Male	Chinese	2019	44	Married	Tertiary	Private	Urban	No	Yes	Yes	No	No	No	No	No	No	No
3399	HPP1397	Male	Indian	2020	27	Single	Tertiary	Private	Urban	No	Yes	Yes	No	No	No	No	No	No	No
3400	HPP1398	Male	Indian	2019	28	Others	Secondary	Government	Urban	No	Yes	No	No	No	No	No	No	No	No
3401	HPP1399	Male	Chinese	2019	43	Married	Secondary	Private	Urban	No	No	Yes	No	No	No	No	No	No	No
3402	HPP1401	Male	Chinese	2020	36	Married	Secondary	Self-employed	Urban	No	Yes	Yes	Yes	Yes	No	No	No	No	No
3403	HPP1402	Male	Malay	2020	21	Single	No formal education	Unemployed	Urban	No	No	No	No	No	No	No	No	Yes	Yes
3404	HPP1403	Male	Indian	2019	46	Others	Secondary	Private	Urban	No	Yes	No	No	No	No	No	No	No	No
3405	HPP1404	Male	Chinese	2019	47	Single	Secondary	Self-employed	Urban	No	No	Yes	No	No	No	No	No	No	No
3406	HPP1405	Male	Chinese	2019	37	Others	Tertiary	Private	Urban	No	Yes	Yes	Yes	No	No	No	No	No	No
3407	HPP1407	Male	Indian	2020	34	Single	Tertiary	Unemployed	Urban	No	Yes	Yes	No	Yes	No	No	No	No	No
3408	HPP1408	Male	Chinese	2020	50	Others	No formal education	Private	Urban	No	No	Yes	Yes	No	No	No	No	No	No
3409	HPP1409	Female	Malay	2020	26	Others	Secondary	Unemployed	Urban	No	No	No	No	Yes	No	No	No	No	No
3410	HPP1411	Male	Indian	2018	22	Single	Tertiary	Private	Urban	No	Yes	Yes	Yes	Yes	No	No	No	No	No
3411	HPP1412	Female	Chinese	2018	34	Married	Secondary	Unemployed	Urban	No	No	Yes	No	No	No	No	No	No	No
3412	HPP1413	Female	Indian	2020	37	Married	Tertiary	Government	Urban	No	No	Yes	No	No	No	No	No	No	No
3413	HPP1414	Female	Chinese	2021	39	Single	Tertiary	Private	Urban	Yes	Yes	No	No	No	No	No	No	No	No
3414	HPP1415	Female	Malay	2021	43	Others	Tertiary	Self-employed	Urban	No	Yes	Yes	No	No	No	No	No	No	No
3415	HPP1416	Female	Indian	2019	30	Single	Secondary	Unemployed	Urban	No	Yes	No	No	Yes	No	No	No	No	No
3416	HPP1417	Female	Indian	2020	23	Single	Tertiary	Private	Urban	No	Yes	Yes	Yes	Yes	No	No	No	No	No
3417	HPP1418	Male	Chinese	2019	18	Single	Secondary	Unemployed	Urban	No	Yes	No	No	No	No	No	No	No	No
3418	HPP1419	Female	Others	2021	22	Single	Tertiary	Private	Urban	No	No	Yes	Yes	No	No	No	No	No	No
3419	HPP1420	Male	Others	2019	51	Single	No formal education	Unemployed	Urban	No	Yes	Yes	No	No	No	Yes	No	No	No
3420	HPP1421	Male	Indian	2020	26	Single	Secondary	Unemployed	Urban	No	Yes	No	Yes	No	No	No	No	No	No
3421	HPP1422	Male	Chinese	2020	56	Single	Secondary	Unemployed	Urban	No	No	No	Yes	No	No	No	No	No	No
3422	HPP1423	Male	Malay	2019	29	Single	Secondary	Private	Urban	No	Yes	Yes	No	No	No	No	No	No	No
3423	HPP1424	Male	Malay	2018	26	Single	Secondary	Private	Urban	No	Yes	No	No	No	No	No	No	No	No
3424	HPP1425	Male	Malay	2019	30	Married	Secondary	Private	Urban	No	Yes	No	No	No	No	No	No	No	No
3425	HPP1426	Male	Others	2020	51	Married	Tertiary	Private	Urban	No	Yes	No	No	No	No	No	No	No	No
3426	HPP1427	Male	Indian	2018	12	Married	Secondary	Unemployed	Urban	No	Yes	Yes	Yes	No	No	No	No	No	No
3427	HPP1429	Male	Chinese	2021	48	Single	Primary	Private	Urban	Yes	Yes	Yes	No	No	No	No	No	No	No
3428	HPP1430	Male	Indian	2021	34	Others	No formal education	Unemployed	Urban	No	Yes	Yes	Yes	No	No	No	No	No	No
3429	HPP1431	Female	Chinese	2018	22	Single	Tertiary	Private	Urban	No	Yes	Yes	No	No	No	No	No	No	No
3430	HPP1433	Female	Chinese	2018	40	Married	Secondary	Unemployed	Urban	No	No	No	No	Yes	No	No	No	No	No
3431	HPP1435	Male	Malay	2021	40	Married	Tertiary	Government	Urban	No	Yes	No	No	No	No	No	No	No	No
3432	HPP1437	Male	Malay	2021	36	Married	No formal education	Government	Urban	Yes	Yes	No	No	No	No	No	No	No	No
3433	HPP1438	Male	Chinese	2018	38	Single	Primary	Self-employed	Urban	Yes	Yes	Yes	Yes	Yes	No	No	No	No	No

No	Patient ID	Gender	Ethnic group	Year of diagnosis	Age	Marital status	Education level	Occupation	Place of residence	History of psy illness	Tobacco	Alcohol	Cannabis	ATS	Inhalant	Sedative	Hallucinogens	Opioids	Kratom
3434	HPP1439	Male	Malay	2020	22	Others	Tertiary	Private	Urban	No	Yes	No	No	Yes	No	No	No	No	No
3435	HPP1441	Male	Malay	2020	33	Married	Secondary	Private	Urban	No	Yes	No	No	Yes	No	Yes	No	No	No
3436	HPP1442	Male	Chinese	2021	36	Single	Secondary	Unemployed	Urban	No	Yes	No	Yes	Yes	No	No	No	No	No
3437	HPP1443	Male	Indian	2021	35	Single	No formal education	Others	Urban	No	Yes	Yes	No	No	No	No	No	No	No
3438	HPP1444	Male	Indian	2021	41	Single	Primary	Private	Urban	No	Yes	Yes	No	No	No	No	No	No	No
3439	HPP1445	Male	Malay	2021	45	Married	Secondary	Private	Urban	No	Yes	No	No	No	No	No	No	No	No
3440	HPP1446	Female	Malay	2018	20	Single	Tertiary	Unemployed	Urban	No	No	Yes	No	No	No	No	No	No	No
3441	HPP1447	Male	Malay	2020	30	Married	Tertiary	Private	Urban	No	Yes	No	No	No	No	No	No	No	No
3442	HPP1448	Female	Malay	2021	50	Married	Secondary	Unemployed	Urban	No	Yes	No	No	No	No	No	No	No	No
3443	HPP1449	Female	Chinese	2018	22	Others	Secondary	Private	Urban	No	No	Yes	No	No	No	No	No	No	No
3444	HPP1451	Female	Indian	2018	27	Married	Secondary	Unemployed	Urban	No	No	No	No	No	No	No	No	Yes	No
3445	HPP1452	Female	Malay	2018	23	Others	Secondary	Private	Urban	Yes	Yes	No	No	No	No	No	No	No	No
3446	HPP1453	Male	Malay	2020	19	Single	Secondary	Private	Urban	No	Yes	Yes	No	No	No	No	No	No	No
3447	HPP1454	Male	Indian	2020	33	Single	No formal education	Others	Urban	No	No	No	No	No	No	No	No	Yes	No
3448	HPP1455	Male	Others	2019	31	Married	Secondary	Self-employed	Urban	No	Yes	Yes	Yes	Yes	No	No	No	Yes	No
3449	HPP1456	Male	Chinese	2019	24	Single	Tertiary	Private	Urban	No	No	Yes	No	No	No	No	No	No	No
3450	HPP1457	Male	Chinese	2020	42	Single	Primary	Unemployed	Urban	Yes	Yes	Yes	No	No	No	No	No	No	No
3451	HPP1460	Male	Indian	2018	27	Single	Secondary	Private	Urban	No	Yes	Yes	No	No	No	No	No	No	No
3452	HPP1461	Male	Malay	2020	29	Single	Secondary	Unemployed	Urban	No	Yes	Yes	No	Yes	No	No	No	No	No
3453	HPP1462	Male	Malay	2020	31	Single	Secondary	Private	Urban	No	Yes	Yes	Yes	Yes	Yes	No	No	Yes	Yes
3454	HPP1463	Male	Chinese	2020	32	Married	Secondary	Private	Urban	No	Yes	Yes	No	Yes	No	No	No	No	No
3455	HPP1464	Male	Indian	2020	43	Others	Secondary	Private	Urban	Yes	Yes	Yes	No	Yes	No	No	No	No	No
3456	HPP1465	Male	Chinese	2019	52	Married	Secondary	Unemployed	Urban	No	Yes	No	No	No	No	No	No	No	No
3457	HPP1466	Male	Chinese	2021	40	Single	Tertiary	Private	Urban	No	Yes	Yes	No	No	No	No	No	No	No
3458	HPP1467	Male	Indian	2019	24	Single	Tertiary	Private	Urban	No	Yes	Yes	No	No	No	No	No	No	No
3459	HPP1468	Male	Malay	2020	50	Married	Secondary	Private	Urban	No	Yes	No	No	No	No	No	No	No	No
3460	HPP1469	Male	Chinese	2020	34	Single	Tertiary	Unemployed	Urban	No	Yes	Yes	No	Yes	No	No	No	No	No
3461	HPP1470	Male	Malay	2021	29	Single	Secondary	Private	Urban	No	Yes	No	Yes	Yes	No	No	No	No	No
3462	HPP1471	Male	Chinese	2019	40	Married	Tertiary	Unemployed	Urban	No	Yes	No	No	No	No	No	No	No	No
3463	HPP1473	Male	Indian	2018	43	Others	Secondary	Private	Urban	No	No	Yes	No	No	No	No	No	No	No
3464	HPP1474	Male	Malay	2020	27	Single	Secondary	Private	Urban	No	Yes	Yes	No	No	No	No	No	No	No
3465	HPP1475	Female	Chinese	2019	19	Single	Tertiary	Unemployed	Urban	No	Yes	Yes	No	No	No	No	No	No	No
3466	HPP1476	Female	Chinese	2021	25	Single	Tertiary	Private	Urban	No	No	Yes	No	No	No	No	No	No	No
3467	HPP1477	Male	Chinese	2021	54	Married	Secondary	Unemployed	Urban	No	Yes	Yes	No	No	No	No	No	No	No
3468	HPP1478	Male	Chinese	2020	22	Single	Tertiary	Unemployed	Urban	No	No	Yes	No	No	No	No	No	No	No
3469	HPP1479	Male	Malay	2018	44	Married	Secondary	Private	Urban	No	Yes	Yes	No	Yes	No	No	No	No	Yes
3470	HPP1480	Male	Others	2020	29	Single	Tertiary	Private	Urban	No	Yes	Yes	Yes	No	No	No	No	No	No
3471	HPP1481	Male	Indian	2020	23	Single	Tertiary	Unemployed	Urban	Yes	No	Yes	No	No	No	No	No	No	No
3472	HPP1482	Male	Chinese	2020	56	Others	Tertiary	Unemployed	Urban	Yes	No	Yes	No	No	No	No	No	No	No
3473	HPP1483	Male	Others	2020	45	Single	Secondary	Private	Urban	No	Yes	Yes	No	No	No	No	No	No	No
3474	HPP1484	Male	Chinese	2020	57	Single	Secondary	Private	Urban	Yes	Yes	Yes	No	No	No	No	No	No	No
3475	HPP1485	Male	Malay	2020	28	Single	Secondary	Unemployed	Urban	Yes	No	No	Yes	Yes	Yes	No	No	Yes	Yes

No	Patient ID	Gender	Ethnic group	Year of diagnosis	Age	Marital status	Education level	Occupation	Place of residence	History of psy illness	Tobacco	Alcohol	Cannabis	ATS	Inhalant	Sedative	Hallucinogens	Opioids	Kratom
3476	HPP1486	Female	Chinese	2018	32	Single	Tertiary	Unemployed	Urban	No	No	Yes	No	No	No	No	No	No	No
3477	HPP1487	Female	Indian	2021	38	Married	Secondary	Unemployed	Urban	No	No	Yes	No	No	No	No	No	No	No
3478	HPP1488	Male	Others	2018	41	Married	Secondary	Private	Urban	No	No	Yes	No	No	No	No	No	No	No
3479	HPP1489	Female	Others	2020	48	Married	Tertiary	Government	Urban	No	No	Yes	No	No	No	No	No	No	No
3480	HPP1490	Male	Malay	2019	41	Married	Secondary	Private	Urban	No	Yes	No	No	No	No	No	No	No	No
3481	HPP1491	Male	Malay	2020	38	Single	Tertiary	Unemployed	Urban	No	Yes	No	No	Yes	No	No	No	No	No
3482	HPP1492	Male	Malay	2018	28	Single	Secondary	Unemployed	Urban	No	Yes	No	No	Yes	No	No	No	No	Yes
3483	HPP1494	Male	Chinese	2019	40	Single	Tertiary	Private	Urban	No	No	Yes	No	No	No	No	No	No	No
3484	HPP1495	Female	Indian	2018	50	Married	Secondary	Private	Urban	No	No	Yes	No	No	No	No	No	No	No
3485	HPP1497	Male	Chinese	2019	37	Single	Secondary	Private	Urban	No	No	Yes	No	No	No	No	No	No	No
3486	HPP1500	Male	Malay	2018	45	Single	Secondary	Unemployed	Urban	No	Yes	No	No	No	No	No	No	No	No
3487	HPP1501	Male	Indian	2018	45	Others	Secondary	Private	Urban	Yes	Yes	No	No	Yes	No	No	No	No	No
3488	HPP1502	Male	Indian	2018	32	Single	Secondary	Private	Urban	No	Yes	Yes	No	No	No	No	No	No	No
3489	HPP1503	Male	Indian	2018	51	Single	Secondary	Private	Urban	No	Yes	No	Yes	No	No	No	No	No	No
3490	HPP1504	Male	Malay	2021	27	Married	Tertiary	Private	Urban	No	Yes	No	Yes	Yes	No	No	No	No	No
3491	HPP1505	Male	Malay	2021	36	Others	Secondary	Private	Urban	No	Yes	No	Yes	Yes	No	No	No	Yes	Yes
3492	HPP1506	Male	Indian	2018	53	Married	No formal education	Private	Urban	No	Yes	No	Yes	No	No	No	No	No	No
3493	HPP1507	Male	Malay	2018	26	Single	Secondary	Unemployed	Urban	No	No	No	Yes	Yes	No	No	No	Yes	No
3494	HPP1510	Male	Others	2020	43	Married	Tertiary	Self-employed	Urban	No	Yes	No	No	No	No	No	No	No	No
3495	HPP1511	Male	Chinese	2018	28	Others	Tertiary	Private	Urban	No	No	No	No	Yes	No	No	No	No	No
3496	HPP1512	Male	Malay	2019	58	Others	Secondary	Others	Urban	No	Yes	Yes	No	No	No	No	No	No	No
3497	HPP1514	Female	Chinese	2020	36	Married	Secondary	Private	Urban	No	No	Yes	No	No	No	No	No	No	No
3498	HPP1515	Female	Chinese	2019	26	Single	Secondary	Private	Urban	No	No	Yes	No	No	No	No	No	No	No
3499	HPP1516	Female	Malay	2019	28	Married	Tertiary	Unemployed	Urban	No	Yes	No	No	No	No	No	No	Yes	No
3500	HPP1517	Female	Malay	2018	25	Married	Tertiary	Government	Urban	No	No	No	No	No	No	Yes	No	No	No
3501	HPP1518	Female	Malay	2018	18	Single	Secondary	Private	Urban	No	Yes	No	No	No	No	No	No	No	No
3502	HPP1519	Male	Malay	2020	36	Married	Secondary	Private	Urban	No	Yes	No	No	No	No	No	No	No	No
3503	HPP1521	Male	Malay	2021	58	Others	Secondary	Private	Urban	No	Yes	No	No	No	No	No	No	No	No
3504	HPP1522	Male	Chinese	2020	28	Single	Primary	Private	Urban	No	Yes	No	No	No	No	No	No	No	No
3505	HPP1523	Male	Malay	2018	22	Single	Tertiary	Unemployed	Urban	No	Yes	No	Yes	No	No	No	No	No	No
3506	HPP1524	Male	Malay	2021	51	Single	Secondary	Unemployed	Urban	No	Yes	Yes	No	No	No	No	No	No	No
3507	HPP1526	Male	Chinese	2019	29	Married	Tertiary	Private	Urban	No	Yes	No	No	No	No	No	No	No	No
3508	HPP1527	Male	Chinese	2019	29	Single	No formal education	Others	Urban	No	No	No	No	Yes	No	No	No	No	No
3509	HPP1530	Male	Chinese	2019	56	Married	Secondary	Unemployed	Urban	No	Yes	No	No	No	No	No	No	No	No
3510	HPP1531	Male	Malay	2020	54	Others	Secondary	Private	Urban	No	Yes	No	No	No	No	No	No	No	No
3511	HPP1532	Male	Chinese	2021	44	Married	Secondary	Private	Urban	No	Yes	No	No	No	No	No	No	No	No
3512	HPP1533	Male	Malay	2020	32	Married	Tertiary	Private	Urban	No	Yes	No	No	No	No	No	No	No	No
3513	HPP1534	Male	Chinese	2021	47	Single	Tertiary	Self-employed	Urban	No	Yes	No	No	No	No	No	No	No	No
3514	HPP1535	Male	Malay	2019	35	Single	No formal education	Private	Urban	No	Yes	No	No	Yes	No	No	No	No	No
3515	HPP1536	Male	Chinese	2021	25	Single	Secondary	Private	Urban	No	No	Yes	No	No	No	No	No	No	No

No	Patient ID	Gender	Ethnic group	Year of diagnosis	Age	Marital status	Education level	Occupation	Place of residence	History of psy illness	Tobacco	Alcohol	Cannabis	ATS	Inhalant	Sedative	Hallucinogens	Opioids	Kratom
3516	HPP1538	Male	Malay	2019	52	Others	No formal education	Others	Urban	No	No	No	No	Yes	No	No	No	Yes	No
3517	HPP1539	Male	Indian	2021	29	Single	Secondary	Self-employed	Urban	No	Yes	Yes	No	No	No	No	No	No	No
3518	HPP1540	Male	Chinese	2018	24	Single	Tertiary	Private	Urban	No	Yes	Yes	No	No	No	No	No	No	No
3519	HPP1541	Male	Malay	2020	32	Single	Secondary	Unemployed	Urban	No	No	Yes	Yes	Yes	Yes	No	No	Yes	Yes
3520	HPP1542	Male	Indian	2018	19	Single	Secondary	Private	Urban	No	Yes	No	No	No	No	No	No	No	No
3521	HPP1543	Male	Chinese	2019	33	Married	Tertiary	Private	Urban	No	No	Yes	No	No	No	No	No	No	No
3522	HPP1544	Female	Chinese	2019	19	Single	Secondary	Unemployed	Urban	No	No	Yes	No	No	No	No	No	No	No
3523	HPP1545	Female	Chinese	2019	29	Single	Secondary	Private	Urban	No	No	Yes	No	No	No	No	No	No	No
3524	HPP1546	Female	Chinese	2018	20	Single	Tertiary	Unemployed	Urban	No	No	Yes	No	No	No	No	No	No	No
3525	HPP1547	Female	Others	2019	22	Single	Secondary	Private	Urban	No	Yes	Yes	No	No	No	No	No	No	No
3526	HPP1548	Male	Malay	2018	26	Others	No formal education	Others	Urban	No	Yes	No	No	No	No	No	No	No	No
3527	HPP1549	Male	Chinese	2021	35	Single	Secondary	Unemployed	Urban	No	Yes	No	No	No	No	No	No	No	No
3528	HPP1550	Male	Chinese	2021	53	Single	Secondary	Private	Urban	No	Yes	Yes	No	No	No	No	No	No	No
3529	HPP1551	Male	Chinese	2020	42	Single	Secondary	Unemployed	Urban	No	Yes	Yes	No	No	No	No	No	No	No
3530	HPP1552	Male	Indian	2020	50	Single	Primary	Private	Urban	No	Yes	Yes	No	Yes	No	No	No	No	No
3531	HPP1554	Male	Chinese	2020	29	Single	Tertiary	Private	Urban	No	No	Yes	No	No	No	No	No	No	No
3532	HPP1555	Male	Malay	2021	23	Single	No formal education	Private	Urban	No	Yes	No	No	No	No	No	No	No	No
3533	HPP1556	Male	Malay	2019	20	Single	Secondary	Private	Urban	No	Yes	No	No	No	No	No	No	No	No
3534	HPP1558	Male	Indian	2020	23	Single	Secondary	Private	Urban	No	Yes	Yes	Yes	No	No	No	No	No	No
3535	HPP1560	Male	Malay	2018	30	Married	Tertiary	Private	Urban	Yes	Yes	No	No	No	No	No	No	No	Yes
3536	HPP1561	Male	Chinese	2019	24	Single	Tertiary	Private	Urban	Yes	No	Yes	No	No	No	No	No	No	No
3537	HPP1562	Male	Malay	2018	44	Others	No formal education	Private	Urban	Yes	Yes	No	No	No	No	No	No	No	No
3538	HPP1563	Male	Chinese	2019	29	Single	Secondary	Unemployed	Urban	No	Yes	Yes	No	No	No	No	No	Yes	No
3539	HPP1564	Male	Malay	2021	27	Single	Secondary	Private	Urban	No	Yes	No	No	Yes	No	No	No	No	No
3540	HPP1566	Male	Chinese	2021	34	Others	Secondary	Private	Urban	No	No	No	No	Yes	No	No	No	No	No
3541	HPP1568	Male	Indian	2018	55	Single	Primary	Private	Urban	No	No	Yes	No	No	No	No	No	No	No
3542	HPP1569	Male	Chinese	2019	30	Single	Tertiary	Private	Urban	No	No	Yes	No	No	No	No	No	No	No
3543	HPP1570	Male	Malay	2021	28	Married	Secondary	Private	Urban	No	Yes	No	No	No	No	No	No	No	No
3544	HPP1571	Male	Malay	2019	30	Single	Primary	Unemployed	Urban	Yes	Yes	No	No	No	No	No	No	No	No
3545	HPP1572	Male	Malay	2020	21	Single	Secondary	Private	Urban	No	Yes	Yes	No	No	No	No	No	No	Yes
3546	HPP1580	Male	Malay	2018	22	Single	Secondary	Private	Urban	Yes	Yes	No	No	No	No	No	No	Yes	No
3547	HPP1583	Male	Indian	2018	49	Single	Secondary	Private	Urban	Yes	No	No	No	No	No	No	No	Yes	No
3548	HPP1587	Male	Malay	2020	54	Single	No formal education	Others	Urban	Yes	No	No	No	No	No	No	No	Yes	No
3549	HPP1590	Male	Chinese	2019	48	Single	Secondary	Private	Urban	Yes	No	No	No	No	No	No	No	Yes	No
3550	HPP1597	Male	Indian	2020	55	Single	Secondary	Private	Urban	Yes	No	No	Yes	Yes	No	No	No	Yes	No
3551	HQE0013	Male	Others	2018	50	Married	No formal education	Government	Urban	Yes	Yes	Yes	No	No	No	No	No	No	No
3552	HQE0014	Male	Others	2018	46	Single	No formal education	Self-employed	Rural	Yes	Yes	Yes	No	No	No	No	No	No	No

No	Patient ID	Gender	Ethnic group	Year of diagnosis	Age	Marital status	Education level	Occupation	Place of residence	History of psy illness	Tobacco	Alcohol	Cannabis	ATS	Inhalant	Sedative	Hallucinogens	Opioids	Kratom
3553	HQE0017	Female	Others	2018	19	Single	Tertiary	Unemployed	Urban	Yes	No	Yes	No	No	No	No	No	No	No
3554	HQE0022	Male	Others	2020	35	Others	Secondary	Unemployed	Rural	Yes	Yes	Yes	No	Yes	No	No	No	No	No
3555	HQE0024	Male	Others	2020	25	Single	Tertiary	Unemployed	Rural	Yes	Yes	No	No	No	No	No	No	No	No
3556	HQE0025	Female	Others	2018	29	Married	Secondary	Unemployed	Rural	Yes	No	No	No	No	No	No	No	Yes	No
3557	HQE0026	Female	Others	2020	34	Married	Secondary	Government	Urban	Yes	Yes	No	No	No	No	No	No	No	No
3558	HQE0027	Male	Others	2018	28	Single	Secondary	Unemployed	Urban	Yes	No	Yes	No	No	No	No	No	No	No
3559	HQE0028	Male	Others	2018	35	Single	Primary	Private	Rural	Yes	Yes	No	No	No	No	No	No	No	No
3560	HQE0029	Male	Others	2019	44	Married	Tertiary	Government	Urban	Yes	Yes	Yes	No	Yes	No	No	No	No	No
3561	HQE0031	Female	Others	2019	19	Others	Tertiary	Unemployed	Urban	Yes	Yes	No	No	No	No	No	No	No	No
3562	HQE0032	Male	Others	2019	22	Married	Secondary	Unemployed	Rural	Yes	Yes	Yes	No	No	No	No	No	No	No
3563	HQE0033	Female	Others	2018	18	Single	Tertiary	Unemployed	Urban	Yes	Yes	Yes	No	No	No	No	No	No	No
3564	HQE0034	Male	Others	2019	31	Single	Tertiary	Unemployed	Urban	Yes	Yes	No	No	No	No	No	No	No	No
3565	HQE0035	Male	Chinese	2019	81	Married	No formal education	Others	Urban	No	Yes	No	No	No	No	No	No	No	No
3566	HQE0036	Male	Others	2019	40	Single	No formal education	Private	Rural	Yes	No	Yes	No	No	No	No	No	No	No
3567	HQE0038	Male	Others	2018	53	Single	No formal education	Self-employed	Rural	Yes	No	Yes	No	No	No	No	No	No	No
3568	HQE0039	Male	Others	2018	29	Single	No formal education	Self-employed	Rural	Yes	No	Yes	No	No	No	No	No	No	No
3569	HQE0040	Male	Others	2021	56	Married	Secondary	Private	Rural	Yes	No	Yes	No	No	No	No	No	No	No
3570	HQE0041	Male	Others	2020	31	Married	Secondary	Self-employed	Rural	Yes	No	Yes	No	No	No	No	No	No	No
3571	HQE0042	Male	Others	2019	31	Single	Secondary	Private	Rural	Yes	Yes	Yes	No	No	No	No	No	No	No
3572	HQE0043	Male	Others	2020	21	Others	No formal education	Unemployed	Rural	Yes	Yes	Yes	No	No	No	No	No	No	No
3573	HQE0044	Male	Others	2018	29	Single	Secondary	Unemployed	Rural	Yes	Yes	Yes	No	No	No	No	No	No	No
3574	HQE0045	Male	Others	2021	34	Single	No formal education	Private	Urban	Yes	Yes	Yes	No	No	No	No	No	No	No
3575	HQE0047	Male	Others	2019	26	Single	Tertiary	Private	Rural	Yes	Yes	Yes	No	No	No	No	No	No	No
3576	HQE0051	Male	Others	2021	46	Single	Secondary	Government	Rural	Yes	Yes	Yes	No	Yes	No	No	No	No	No
3577	HQE0053	Female	Others	2021	35	Others	Tertiary	Private	Rural	Yes	Yes	Yes	Yes	Yes	No	No	No	No	No
3578	HQE0054	Male	Others	2021	21	Single	Tertiary	Unemployed	Urban	Yes	Yes	Yes	No	Yes	No	No	No	No	No
3579	HQE0055	Male	Others	2018	28	Married	Tertiary	Government	Urban	Yes	Yes	Yes	No	Yes	No	No	No	No	No

No	Patient ID	Gender	Ethnic group	Year of diagnosis	Age	Marital status	Education level	Occupation	Place of residence	History of psy illness	Tobacco	Alcohol	Cannabis	ATS	Inhalant	Sedative	Hallucinogens	Opioids	Kratom
3580	HQE0056	Male	Others	2018	33	Others	Secondary	Private	Rural	Yes	Yes	Yes	No	Yes	No	No	No	No	No
3581	HQE0057	Male	Others	2020	56	Married	No formal education	Private	Rural	Yes	Yes	Yes	No	No	No	No	No	No	No
3582	HQE0058	Male	Others	2020	56	Single	Tertiary	Others	Rural	Yes	Yes	Yes	No	No	No	No	No	No	No
3583	HQE0059	Male	Others	2020	50	Married	No formal education	Self-employed	Rural	Yes	Yes	Yes	No	No	No	No	No	No	No
3584	HQE0060	Male	Chinese	2018	39	Single	Tertiary	Private	Rural	Yes	Yes	Yes	No	No	No	No	No	No	No
3585	HQE0061	Female	Malay	2020	40	Married	No formal education	Unemployed	Rural	Yes	Yes	No	No	Yes	No	No	No	No	No
3586	HQE0062	Male	Others	2020	67	Married	No formal education	Others	Rural	Yes	No	Yes	No	No	No	No	No	No	No
3587	HQE0064	Male	Chinese	2019	29	Single	Tertiary	Government	Urban	Yes	Yes	No	No	Yes	No	No	No	No	No
3588	HQE0065	Male	Others	2021	57	Single	No formal education	Unemployed	Rural	Yes	Yes	Yes	No	Yes	No	No	No	No	No
3589	HQE0066	Male	Others	2019	63	Married	No formal education	Others	Rural	No	Yes	Yes	No	No	No	No	No	No	No
3590	HQE0067	Male	Others	2018	34	Single	Primary	Others	Rural	Yes	Yes	Yes	No	No	No	No	No	No	No
3591	HQE0068	Male	Others	2018	40	Married	Primary	Private	Rural	Yes	Yes	Yes	No	No	No	No	No	No	No
3592	HQE0069	Female	Chinese	2018	30	Others	Secondary	Unemployed	Urban	Yes	Yes	Yes	No	Yes	No	No	No	No	No
3593	HQE0070	Female	Malay	2019	24	Others	Tertiary	Private	Urban	Yes	Yes	Yes	No	No	No	No	No	No	No
3594	HQE0073	Male	Others	2019	30	Married	No formal education	Private	Urban	Yes	Yes	Yes	No	Yes	No	No	No	No	No
3595	HQE0075	Male	Others	2020	54	Single	Primary	Unemployed	Rural	Yes	Yes	Yes	No	No	No	No	No	No	No
3596	HQE0076	Male	Chinese	2020	47	Married	Secondary	Private	Rural	Yes	Yes	Yes	No	No	No	No	No	No	No
3597	HQE0077	Male	Others	2018	39	Single	No formal education	Private	Rural	Yes	Yes	No	No	Yes	No	No	No	No	No
3598	HQE0078	Male	Others	2020	29	Single	No formal education	Private	Rural	Yes	Yes	Yes	No	No	No	No	No	No	No
3599	HQE0079	Male	Chinese	2019	45	Married	Secondary	Private	Rural	Yes	Yes	Yes	No	No	No	No	No	No	No
3600	HQE0080	Male	Chinese	2020	61	Married	Secondary	Self-employed	Urban	Yes	Yes	Yes	No	No	No	No	No	No	No
3601	HQE0081	Male	Chinese	2018	43	Married	Secondary	Unemployed	Urban	Yes	Yes	Yes	No	No	No	No	No	No	No
3602	HQE0082	Male	Others	2018	31	Others	Secondary	Government	Urban	Yes	Yes	Yes	No	No	No	No	No	No	No
3603	HQE0083	Male	Others	2018	45	Single	Primary	Private	Rural	Yes	Yes	Yes	No	No	No	No	No	No	No
3604	HQE0084	Male	Others	2020	38	Married	Secondary	Unemployed	Rural	Yes	Yes	No	No	No	No	No	No	No	No
3605	HQE0085	Male	Others	2018	68	Single	No formal education	Others	Rural	Yes	Yes	No	No	No	No	No	No	No	No

No	Patient ID	Gender	Ethnic group	Year of diagnosis	Age	Marital status	Education level	Occupation	Place of residence	History of psy illness	Tobacco	Alcohol	Cannabis	ATS	Inhalant	Sedative	Hallucinogens	Opioids	Kratom
3606	HQE0086	Male	Others	2019	35	Others	Tertiary	Unemployed	Urban	Yes	Yes	Yes	Yes	Yes	No	No	No	No	No
3607	HQE0087	Male	Others	2019	48	Married	Secondary	Self-employed	Rural	Yes	Yes	Yes	No	No	No	No	No	No	No
3608	HQE0088	Male	Others	2019	61	Married	Secondary	Others	Rural	Yes	No	Yes	No	No	No	No	No	No	No
3609	HQE0089	Male	Others	2019	46	Married	Secondary	Private	Rural	Yes	Yes	Yes	No	No	No	No	No	No	No
3610	HQE0091	Male	Others	2018	18	Single	Secondary	Unemployed	Rural	Yes	Yes	No	No	No	No	No	No	No	No
3611	HQE0092	Male	Others	2019	48	Married	Secondary	Others	Rural	Yes	Yes	No	No	No	No	No	No	No	No
3612	HQE0093	Male	Chinese	2020	29	Single	Secondary	Unemployed	Rural	Yes	Yes	Yes	No	Yes	No	No	No	No	No
3613	HQE0094	Male	Others	2019	28	Single	Primary	Unemployed	Rural	Yes	Yes	Yes	No	Yes	No	No	No	No	No
3614	HQE0095	Male	Others	2020	25	Single	No formal education	Private	Rural	Yes	Yes	Yes	No	Yes	No	No	No	No	No
3615	HQE0096	Male	Chinese	2018	70	Single	Secondary	Unemployed	Rural	Yes	Yes	Yes	No	No	No	No	No	No	No
3616	HQE0097	Male	Others	2018	41	Married	Primary	Unemployed	Rural	Yes	Yes	Yes	No	Yes	No	No	No	No	No
3617	HQE0098	Male	Others	2019	55	Married	No formal education	Unemployed	Rural	Yes	Yes	Yes	No	Yes	No	No	No	No	No
3618	HQE0099	Male	Others	2019	29	Single	Tertiary	Unemployed	Rural	Yes	Yes	Yes	No	No	No	No	No	No	No
3619	HQE0100	Male	Others	2020	43	Single	No formal education	Private	Rural	Yes	Yes	Yes	No	No	No	No	No	No	No
3620	HQE0101	Male	Others	2019	34	Others	No formal education	Private	Rural	Yes	Yes	Yes	No	No	No	No	No	No	No
3621	HQE0102	Male	Chinese	2020	30	Others	Secondary	Private	Urban	Yes	Yes	Yes	Yes	Yes	No	No	No	No	No
3622	HQE0103	Male	Others	2020	44	Single	No formal education	Private	Rural	Yes	Yes	Yes	No	No	No	No	No	No	No
3623	HQE0104	Male	Others	2019	62	Single	No formal education	Others	Rural	Yes	Yes	Yes	No	No	No	No	No	No	No
3624	HQE0106	Male	Others	2020	29	Others	Secondary	Unemployed	Rural	Yes	Yes	No	No	Yes	No	No	No	No	No
3625	HQE0107	Female	Others	2021	23	Single	Secondary	Private	Urban	Yes	Yes	Yes	No	No	No	No	No	No	No
3626	HQE0108	Female	Others	2020	36	Married	No formal education	Unemployed	Rural	Yes	Yes	Yes	No	Yes	No	No	No	No	No
3627	HQE0109	Male	Chinese	2021	56	Single	No formal education	Unemployed	Rural	Yes	Yes	Yes	Yes	Yes	No	No	No	Yes	No
3628	HQE0110	Male	Others	2020	57	Married	Primary	Self-employed	Rural	Yes	Yes	Yes	No	No	No	No	No	No	No
3629	HQE0111	Male	Others	2020	31	Others	Secondary	Unemployed	Rural	Yes	Yes	No	No	Yes	No	No	No	No	No
3630	HQE0112	Male	Chinese	2019	31	Single	Primary	Private	Rural	Yes	Yes	Yes	No	Yes	No	No	No	No	No
3631	HQE0113	Male	Others	2019	32	Married	Primary	Self-employed	Rural	Yes	Yes	Yes	No	No	No	No	No	No	No
3632	HQE0114	Male	Others	2019	37	Single	Secondary	Unemployed	Urban	Yes	Yes	Yes	No	No	No	No	No	No	No
3633	HQE0115	Male	Chinese	2020	25	Single	No formal education	Private	Urban	Yes	Yes	Yes	Yes	No	No	No	No	No	No
3634	HQE0116	Male	Others	2020	50	Married	No formal education	Unemployed	Rural	Yes	Yes	Yes	No	No	No	No	No	No	No

No	Patient ID	Gender	Ethnic group	Year of diagnosis	Age	Marital status	Education level	Occupation	Place of residence	History of psy illness	Tobacco	Alcohol	Cannabis	ATS	Inhalant	Sedative	Hallucinogens	Opioids	Kratom
3635	HQE0118	Male	Others	2019	41	Single	Secondary	Private	Rural	Yes	Yes	Yes	No	No	No	No	No	No	No
3636	HQE0119	Male	Malay	2018	49	Married	Tertiary	Government	Urban	Yes	Yes	No	No	No	No	No	No	No	No
3637	HQE0120	Female	Chinese	2019	28	Single	Secondary	Unemployed	Urban	Yes	Yes	No	No	No	No	No	No	No	No
3638	HQE0121	Female	Malay	2019	20	Single	Tertiary	Private	Urban	Yes	Yes	Yes	No	No	No	No	No	No	No
3639	HQE0122	Male	Others	2018	29	Single	No formal education	Private	Rural	Yes	No	Yes	No	No	No	No	No	No	No
3640	HQE0122	Male	Others	2018	49	Married	No formal education	Private	Urban	Yes	Yes	Yes	No	No	No	No	No	No	No
3641	HQE0124	Female	Others	2018	25	Others	Primary	Private	Rural	Yes	Yes	No	Yes	No	No	No	No	No	No
3642	HQE0125	Male	Chinese	2018	52	Others	Secondary	Private	Rural	Yes	Yes	Yes	No	No	No	No	No	No	No
3643	HQE0126	Male	Malay	2018	32	Married	Secondary	Self-employed	Rural	Yes	Yes	Yes	No	No	No	No	No	No	No
3644	HQE0127	Male	Others	2020	37	Married	No formal education	Private	Rural	Yes	Yes	Yes	Yes	Yes	No	No	No	No	No
3645	HQE0128	Male	Others	2019	22	Others	No formal education	Private	Rural	Yes	Yes	Yes	No	No	No	No	No	No	No
3646	HQE0129	Male	Others	2020	57	Married	Secondary	Government	Rural	Yes	Yes	Yes	No	No	No	No	No	No	No
3647	HQE0130	Female	Chinese	2018	28	Others	Secondary	Private	Urban	Yes	Yes	Yes	No	No	No	No	No	No	No
3648	HQE0131	Female	Others	2021	51	Others	No formal education	Unemployed	Rural	Yes	No	Yes	No	No	No	No	No	No	No
3649	HQE0133	Male	Others	2021	45	Single	Secondary	Self-employed	Rural	Yes	Yes	Yes	No	No	No	No	No	No	No
3650	HQE0134	Male	Others	2018	54	Others	No formal education	Unemployed	Rural	Yes	Yes	Yes	No	No	No	No	No	No	No
3651	HQE0135	Male	Others	2019	26	Married	No formal education	Private	Rural	Yes	Yes	No	No	Yes	No	No	No	No	No
3652	HQE0136	Male	Others	2019	43	Married	Primary	Private	Rural	Yes	Yes	Yes	No	No	No	No	No	No	No
3653	HQE0137	Male	Others	2019	29	Married	Secondary	Private	Rural	Yes	Yes	Yes	No	Yes	No	No	No	No	No
3654	HQE0143	Male	Others	2018	54	Married	No formal education	Others	Rural	Yes	Yes	Yes	No	No	No	No	No	No	No
3655	HQE0144	Male	Others	2021	26	Married	Primary	Private	Rural	Yes	Yes	Yes	No	Yes	No	No	No	Yes	Yes
3656	HQE0145	Female	Others	2018	21	Others	Tertiary	Unemployed	Rural	Yes	Yes	Yes	No	No	No	No	No	No	No
3657	HQE0146	Female	Indian	2018	51	Others	Tertiary	Private	Urban	Yes	No	Yes	No	No	No	No	No	No	No
3658	HQE0147	Female	Others	2019	21	Others	Primary	Private	Rural	Yes	Yes	Yes	No	No	No	No	No	No	No
3659	HQE0148	Female	Chinese	2019	22	Single	Primary	Private	Rural	Yes	No	Yes	No	No	No	No	No	No	No
3660	HQE0149	Female	Others	2020	20	Others	Secondary	Private	Rural	Yes	Yes	Yes	No	No	No	No	No	No	No
3661	HQE0150	Female	Others	2020	25	Others	Secondary	Private	Rural	Yes	Yes	Yes	No	No	No	No	No	No	No
3662	HQE0151	Male	Malay	2020	36	Married	Secondary	Government	Rural	Yes	Yes	No	No	No	No	No	No	No	No
3663	HQE0152	Male	Indian	2018	59	Others	Secondary	Self-employed	Rural	Yes	No	Yes	No	No	No	No	No	No	No

No	Patient ID	Gender	Ethnic group	Year of diagnosis	Age	Marital status	Education level	Occupation	Place of residence	History of psy illness	Tobacco	Alcohol	Cannabis	ATS	Inhalant	Sedative	Hallucinogens	Opioids	Kratom
3664	HQE0153	Male	Others	2018	26	Single	Tertiary	Private	Rural	Yes	Yes	Yes	No	No	No	No	No	No	No
3665	HQE0154	Male	Malay	2020	27	Single	Secondary	Unemployed	Rural	Yes	Yes	Yes	Yes	Yes	No	No	No	No	No
3666	HQE0155	Male	Others	2019	24	Single	Tertiary	Private	Urban	Yes	Yes	Yes	Yes	Yes	No	No	No	No	No
3667	HQE0156	Male	Others	2019	47	Married	Primary	Private	Rural	Yes	No	Yes	No	No	No	No	No	No	No
3668	HQE0157	Male	Others	2020	30	Married	Secondary	Private	Rural	Yes	Yes	Yes	No	No	No	No	No	No	No
3669	HQE0158	Male	Others	2019	55	Single	Secondary	Private	Rural	Yes	Yes	Yes	No	No	No	No	No	No	No
3670	HQE0160	Male	Others	2019	71	Others	Tertiary	Private	Urban	Yes	Yes	Yes	No	No	No	No	No	No	No
3671	HQE0161	Female	Others	2019	20	Others	No formal education	Private	Rural	Yes	Yes	Yes	No	Yes	No	No	No	No	No
3672	HQE0162	Female	Chinese	2018	35	Married	Tertiary	Private	Urban	Yes	Yes	Yes	No	No	No	No	No	No	No
3673	HQE0163	Female	Others	2019	30	Married	Secondary	Unemployed	Rural	Yes	Yes	Yes	No	No	No	No	No	No	No
3674	HQE0165	Male	Others	2019	30	Married	Tertiary	Self-employed	Rural	Yes	Yes	Yes	No	No	No	No	No	No	No
3675	HQE0166	Male	Chinese	2019	39	Married	Secondary	Private	Rural	Yes	No	Yes	No	No	No	No	No	No	No
3676	HQE0167	Male	Malay	2020	30	Others	Tertiary	Government	Urban	Yes	Yes	Yes	Yes	No	No	No	Yes	No	No
3677	HQE0168	Male	Indian	2020	26	Single	No formal education	Self-employed	Rural	Yes	Yes	Yes	No	Yes	No	No	No	No	No
3678	HQE0169	Male	Others	2020	31	Single	No formal education	Private	Rural	Yes	Yes	Yes	No	Yes	Yes	No	No	No	No
3679	HQE0170	Male	Others	2019	21	Single	Primary	Private	Rural	Yes	Yes	No	No	Yes	No	No	No	No	No
3680	HQE0171	Male	Chinese	2021	48	Married	No formal education	Private	Rural	Yes	Yes	Yes	No	Yes	No	No	No	No	No
3681	HQE0172	Male	Others	2020	38	Married	Tertiary	Private	Rural	Yes	Yes	Yes	Yes	No	No	No	No	No	No
3682	HQE0174	Male	Others	2021	25	Single	Secondary	Private	Rural	Yes	Yes	No	No	Yes	No	No	No	No	No
3683	HQE0175	Male	Others	2020	46	Others	Primary	Unemployed	Rural	Yes	Yes	Yes	No	Yes	No	No	No	No	No
3684	HQE0176	Male	Others	2019	32	Married	Primary	Unemployed	Urban	Yes	Yes	No	No	No	No	No	No	No	No
3685	HQE0177	Male	Malay	2018	49	Married	Primary	Private	Urban	Yes	Yes	No	Yes	Yes	No	No	No	No	No
3686	HQE0178	Male	Others	2018	23	Single	Primary	Unemployed	Rural	Yes	Yes	No	No	Yes	No	No	No	No	No
3687	HQE0180	Male	Others	2018	40	Married	No formal education	Others	Rural	Yes	Yes	Yes	Yes	Yes	No	No	No	No	No
3688	HQE0181	Male	Others	2019	24	Single	Primary	Unemployed	Rural	Yes	Yes	No	No	No	No	No	No	No	No
3689	HQE0182	Male	Others	2020	18	Single	Secondary	Unemployed	Urban	Yes	Yes	No	No	No	No	No	No	No	No
3690	HQE0183	Male	Others	2021	53	Married	Primary	Others	Rural	Yes	Yes	Yes	No	Yes	No	No	No	No	No
3691	HQE0184	Male	Chinese	2021	45	Married	Primary	Unemployed	Rural	Yes	Yes	Yes	No	No	No	No	No	No	No
3692	HQE0185	Male	Others	2020	31	Others	Secondary	Unemployed	Rural	Yes	Yes	Yes	No	Yes	No	No	No	No	No
3693	HQE0186	Male	Malay	2018	22	Single	Secondary	Unemployed	Rural	Yes	Yes	No	No	Yes	No	No	No	No	No
3694	HQE0188	Male	Others	2018	55	Married	No formal education	Private	Rural	Yes	Yes	Yes	No	Yes	No	No	No	No	No
3695	HQE0189	Male	Others	2020	16	Single	No formal education	Private	Rural	Yes	Yes	Yes	No	Yes	No	No	No	No	No

No	Patient ID	Gender	Ethnic group	Year of diagnosis	Age	Marital status	Education level	Occupation	Place of residence	History of psy illness	Tobacco	Alcohol	Cannabis	ATS	Inhalant	Sedative	Hallucinogens	Opioids	Kratom
3696	HQE0190	Male	Others	2018	42	Single	Secondary	Unemployed	Rural	Yes	Yes	No	No	No	No	No	No	No	No
3697	HQE0191	Male	Others	2019	41	Others	Secondary	Government	Rural	Yes	Yes	Yes	No	Yes	No	No	No	No	No
3698	HQE0192	Male	Others	2019	28	Single	Primary	Private	Rural	Yes	Yes	Yes	No	Yes	No	No	No	No	No
3699	HQE0193	Male	Others	2019	32	Single	Secondary	Unemployed	Rural	Yes	Yes	Yes	No	Yes	No	No	No	No	No
3700	HQE0195	Male	Others	2018	30	Married	Secondary	Private	Rural	Yes	Yes	No	No	Yes	No	No	No	No	No
3701	HQE0196	Male	Others	2019	19	Single	No formal education	Unemployed	Rural	Yes	Yes	No	No	Yes	No	No	No	No	No
3702	HQE0197	Male	Others	2019	25	Single	Primary	Self-employed	Rural	Yes	Yes	Yes	No	Yes	No	No	No	No	No
3703	HQE0198	Female	Others	2019	23	Others	No formal education	Private	Urban	Yes	Yes	Yes	No	No	No	No	No	No	No
3704	HQE0199	Male	Others	2019	29	Single	No formal education	Others	Rural	Yes	Yes	Yes	No	Yes	No	No	No	No	No
3705	HQE0202	Male	Others	2019	19	Single	No formal education	Private	Rural	Yes	Yes	No	No	Yes	No	No	No	No	No
3706	HQE0203	Male	Others	2020	36	Married	Primary	Private	Rural	Yes	Yes	No	Yes	No	No	No	No	No	No
3707	HQE0204	Male	Others	2021	21	Single	No formal education	Unemployed	Rural	Yes	Yes	No	No	Yes	No	No	No	No	No
3708	HQE0205	Male	Others	2018	50	Others	Primary	Private	Urban	Yes	Yes	Yes	No	Yes	No	No	No	No	No
3709	HQE0206	Male	Others	2019	39	Others	Secondary	Private	Rural	Yes	Yes	Yes	No	Yes	No	No	No	No	No
3710	HQE0207	Male	Others	2020	34	Single	Primary	Private	Rural	Yes	Yes	Yes	No	Yes	No	No	No	No	No
3711	HQE0208	Male	Others	2018	45	Married	Primary	Private	Rural	Yes	Yes	No	No	No	No	No	No	No	No
3712	HQE0209	Male	Others	2020	28	Others	Secondary	Private	Rural	Yes	Yes	Yes	No	No	No	No	No	No	No
3713	HQE0210	Male	Others	2020	31	Married	No formal education	Private	Rural	Yes	Yes	Yes	No	Yes	No	No	No	No	No
3714	HQE0211	Female	Others	2018	22	Single	No formal education	Unemployed	Rural	Yes	Yes	Yes	Yes	No	No	No	No	No	No
3715	HQE0212	Female	Others	2019	23	Single	Tertiary	Private	Rural	Yes	Yes	Yes	Yes	No	No	No	No	No	No
3716	HQE0214	Male	Others	2020	23	Single	No formal education	Private	Urban	Yes	Yes	Yes	No	Yes	No	No	No	No	No
3717	HQE0216	Male	Others	2019	32	Others	Primary	Private	Urban	Yes	Yes	Yes	No	Yes	No	No	No	No	No
3718	HQE0217	Female	Others	2018	38	Others	Secondary	Private	Rural	Yes	Yes	Yes	No	Yes	No	No	No	No	No
3719	HQE0218	Male	Others	2018	41	Others	No formal education	Unemployed	Rural	Yes	Yes	Yes	No	No	No	No	No	No	No
3720	HQE0219	Male	Others	2018	26	Others	Secondary	Private	Rural	Yes	Yes	Yes	Yes	No	No	No	No	No	No
3721	HQE0220	Male	Others	2020	23	Single	Tertiary	Private	Rural	Yes	Yes	Yes	No	No	No	No	No	No	No
3722	HQE0221	Male	Others	2018	41	Married	Tertiary	Unemployed	Rural	Yes	Yes	Yes	No	Yes	No	No	No	No	No
3723	HQE0224	Male	Others	2021	29	Married	No formal education	Private	Rural	Yes	Yes	Yes	No	No	No	No	No	No	No
3724	HQE0225	Male	Others	2018	32	Others	Secondary	Unemployed	Rural	Yes	Yes	Yes	No	Yes	No	No	No	No	No
3725	HQE0226	Male	Others	2021	50	Married	Tertiary	Private	Rural	Yes	No	No	No	No	No	No	No	No	Yes
3726	HQE0229	Male	Indian	2020	54	Married	Secondary	Government	Urban	Yes	Yes	Yes	No	Yes	No	No	No	No	No

No	Patient ID	Gender	Ethnic group	Year of diagnosis	Age	Marital status	Education level	Occupation	Place of residence	History of psy illness	Tobacco	Alcohol	Cannabis	ATS	Inhalant	Sedative	Hallucinogens	Opioids	Kratom
3727	HQE0232	Male	Others	2020	23	Married	Secondary	Unemployed	Urban	Yes	Yes	No	No	No	No	No	No	No	No
3728	HQE0233	Male	Others	2019	23	Single	Primary	Unemployed	Rural	Yes	Yes	No	No	Yes	Yes	No	No	No	No
3729	HQE0234	Male	Others	2021	40	Single	Tertiary	Private	Urban	Yes	No	Yes	No	No	No	No	No	No	No
3730	HQE0235	Male	Others	2020	21	Single	Tertiary	Private	Rural	Yes	Yes	Yes	No	Yes	No	No	No	No	No
3731	HQE0236	Male	Others	2020	41	Married	Secondary	Private	Rural	Yes	Yes	Yes	No	No	No	No	No	No	No
3732	HQE0237	Female	Others	2021	22	Single	Secondary	Unemployed	Rural	Yes	Yes	Yes	No	No	No	No	No	No	No
3733	HQE0238	Female	Others	2020	24	Single	Secondary	Unemployed	Urban	Yes	Yes	Yes	No	No	No	No	No	No	No
3734	HQE0240	Male	Others	2018	59	Others	Secondary	Unemployed	Urban	Yes	Yes	Yes	No	No	No	No	No	No	No
3735	HQE0241	Male	Others	2019	20	Single	Tertiary	Unemployed	Rural	Yes	Yes	Yes	Yes	No	No	No	No	No	No
3736	HQE0242	Male	Others	2019	29	Single	Tertiary	Private	Urban	Yes	Yes	Yes	No	Yes	No	No	No	No	No
3737	HQE0243	Male	Others	2020	36	Married	No formal education	Private	Rural	Yes	Yes	Yes	No	No	No	No	No	No	No
3738	HQE0244	Male	Chinese	2020	19	Single	Tertiary	Self-employed	Urban	Yes	Yes	Yes	No	No	No	No	No	No	No
3739	HQE0245	Male	Others	2018	27	Single	Primary	Private	Rural	Yes	Yes	Yes	No	No	No	No	No	No	No
3740	HQE0248	Male	Others	2020	22	Single	No formal education	Unemployed	Rural	Yes	Yes	Yes	No	Yes	No	No	No	No	No
3741	HQE0251	Male	Others	2019	49	Others	Secondary	Private	Rural	Yes	Yes	No	No	Yes	No	No	No	No	No
3742	HQE0252	Male	Others	2018	27	Single	No formal education	Unemployed	Rural	Yes	Yes	No	No	Yes	Yes	No	No	No	No
3743	HQE0253	Male	Others	2020	29	Single	Secondary	Unemployed	Rural	Yes	Yes	Yes	No	No	No	No	No	No	No
3744	HQE0255	Male	Others	2018	35	Single	Secondary	Unemployed	Rural	Yes	Yes	Yes	No	Yes	No	No	No	No	No
3745	HQE0256	Male	Chinese	2019	28	Others	Tertiary	Self-employed	Rural	Yes	Yes	Yes	No	No	No	No	No	No	No
3746	HQE0257	Male	Others	2019	28	Single	Tertiary	Private	Rural	Yes	Yes	No	No	Yes	No	No	No	No	No
3747	HQE0258	Male	Others	2019	32	Others	No formal education	Private	Rural	Yes	Yes	Yes	No	Yes	No	No	No	No	No
3748	HQE0259	Male	Others	2018	29	Others	No formal education	Unemployed	Rural	Yes	Yes	Yes	No	Yes	No	No	No	No	No
3749	HQE0260	Male	Others	2019	19	Single	No formal education	Unemployed	Rural	Yes	Yes	No	No	Yes	No	No	No	No	No
3750	HQE0262	Male	Others	2018	23	Single	No formal education	Private	Rural	Yes	Yes	No	No	No	No	No	No	No	No
3751	HQE0263	Male	Others	2018	27	Married	No formal education	Private	Rural	Yes	Yes	No	No	No	No	No	No	No	No
3752	HQE0264	Male	Others	2020	26	Single	Primary	Unemployed	Rural	Yes	Yes	Yes	No	Yes	No	No	No	No	No
3753	HQE0266	Female	Others	2018	18	Others	No formal education	Unemployed	Rural	Yes	No	Yes	No	Yes	No	No	No	No	No
3754	HQE0268	Male	Others	2020	44	Married	No formal education	Unemployed	Rural	Yes	Yes	Yes	No	No	No	No	No	No	No
3755	HQE0269	Male	Malay	2021	24	Single	Tertiary	Government	Rural	Yes	Yes	Yes	Yes	No	No	No	No	No	No
3756	HQE0270	Male	Others	2018	18	Single	Secondary	Unemployed	Rural	Yes	Yes	No	No	No	Yes	No	No	No	No
3757	HQE0271	Male	Others	2021	40	Married	Tertiary	Government	Rural	Yes	Yes	Yes	No	Yes	No	No	No	No	No
3758	HQE0272	Female	Others	2019	25	Single	Secondary	Private	Urban	Yes	Yes	Yes	Yes	No	No	No	No	No	No

No	Patient ID	Gender	Ethnic group	Year of diagnosis	Age	Marital status	Education level	Occupation	Place of residence	History of psy illness	Tobacco	Alcohol	Cannabis	ATS	Inhalant	Sedative	Hallucinogens	Opioids	Kratom
3759	HQE0273	Female	Others	2019	23	Single	Secondary	Private	Rural	Yes	Yes	Yes	Yes	No	No	No	No	No	No
3760	HQE0274	Female	Others	2019	23	Others	Primary	Private	Rural	Yes	Yes	Yes	No	Yes	No	No	No	No	No
3761	HQE0275	Male	Others	2018	24	Single	Secondary	Private	Rural	Yes	Yes	Yes	No	No	No	No	No	No	No
3762	HQE0276	Female	Others	2020	39	Married	Primary	Private	Rural	Yes	No	Yes	No	No	No	No	No	No	No
3763	HQE0277	Female	Others	2019	14	Single	Secondary	Unemployed	Rural	Yes	Yes	Yes	No	No	No	No	No	No	No
3764	HQE0278	Female	Others	2019	49	Married	Tertiary	Private	Rural	Yes	Yes	Yes	No	No	No	No	No	No	No
3765	HQE0279	Male	Others	2020	35	Single	No formal education	Unemployed	Rural	Yes	Yes	Yes	No	Yes	No	No	No	No	No
3766	HQE0281	Male	Chinese	2018	38	Married	Secondary	Private	Rural	Yes	Yes	Yes	No	No	No	No	No	No	No
3767	HQE0282	Male	Chinese	2021	50	Others	Secondary	Private	Rural	Yes	Yes	Yes	No	No	No	No	No	No	No
3768	HQE0283	Male	Others	2020	39	Married	Secondary	Government	Rural	Yes	Yes	No	No	No	No	No	No	No	No
3769	HQE0285	Male	Chinese	2018	21	Single	Tertiary	Unemployed	Rural	Yes	Yes	Yes	No	No	No	No	No	No	No
3770	HQE0286	Male	Others	2018	26	Single	Secondary	Unemployed	Rural	Yes	Yes	Yes	No	Yes	No	No	No	No	No
3771	HQE0287	Male	Others	2019	51	Single	Tertiary	Private	Rural	Yes	Yes	Yes	No	No	No	No	No	No	No
3772	HQE0289	Male	Others	2020	53	Married	No formal education	Private	Rural	Yes	Yes	Yes	No	No	No	No	No	No	No
3773	HQE0290	Male	Others	2021	78	Married	No formal education	Others	Rural	Yes	Yes	Yes	No	No	No	No	No	No	No
3774	HQE0291	Male	Indian	2019	57	Married	Secondary	Unemployed	Rural	Yes	No	Yes	No	No	No	No	No	No	No
3775	HQE0292	Male	Chinese	2020	37	Single	Tertiary	Private	Rural	Yes	Yes	Yes	No	No	No	No	No	No	No
3776	HQE0293	Male	Others	2020	47	Married	Tertiary	Private	Urban	Yes	Yes	Yes	No	No	No	No	No	No	No
3777	HQE0295	Male	Others	2020	52	Married	Secondary	Others	Rural	Yes	Yes	Yes	No	No	No	No	No	No	No
3778	HQE0296	Male	Others	2020	34	Single	No formal education	Private	Rural	Yes	Yes	Yes	No	No	No	No	No	No	No
3779	HQE0299	Male	Others	2020	46	Single	No formal education	Unemployed	Rural	Yes	Yes	Yes	No	No	No	No	No	No	No
3780	HQE0300	Male	Others	2019	20	Single	Tertiary	Government	Urban	Yes	Yes	Yes	No	No	No	No	No	No	No
3781	HQE0301	Female	Others	2021	30	Single	Tertiary	Government	Rural	Yes	No	Yes	No	No	No	No	No	No	No
3782	HQE0303	Male	Others	2018	23	Single	Primary	Private	Rural	Yes	Yes	Yes	No	No	No	No	No	No	No
3783	HQE0304	Male	Others	2021	47	Single	Secondary	Private	Rural	Yes	Yes	Yes	No	No	No	No	No	No	No
3784	HQE0305	Male	Others	2018	39	Married	Tertiary	Private	Rural	Yes	Yes	No	No	Yes	No	No	No	No	No
3785	HQE0306	Female	Chinese	2021	21	Others	Secondary	Private	Rural	Yes	No	Yes	No	No	No	No	No	No	No
3786	HQE0308	Female	Malay	2021	21	Single	Tertiary	Unemployed	Rural	Yes	Yes	Yes	No	No	No	No	No	No	No
3787	HQE0309	Male	Others	2019	24	Single	Secondary	Private	Rural	Yes	Yes	No	No	No	No	No	No	No	No
3788	HQE0310	Male	Others	2020	19	Single	No formal education	Unemployed	Rural	Yes	Yes	Yes	No	Yes	Yes	Yes	No	No	No
3789	HQE0311	Male	Others	2018	52	Married	Tertiary	Private	Rural	Yes	Yes	Yes	No	No	No	No	No	No	No
3790	HQE0312	Male	Others	2019	30	Single	No formal education	Private	Rural	Yes	Yes	Yes	No	No	No	No	No	No	No

No	Patient ID	Gender	Ethnic group	Year of diagnosis	Age	Marital status	Education level	Occupation	Place of residence	History of psy illness	Tobacco	Alcohol	Cannabis	ATS	Inhalant	Sedative	Hallucinogens	Opioids	Kratom
3791	HQE0314	Male	Others	2018	30	Single	No formal education	Private	Rural	Yes	No	Yes	No	No	No	No	No	No	No
3792	HQE0315	Female	Others	2021	27	Married	Secondary	Private	Rural	Yes	No	Yes	No	No	No	Yes	No	No	No
3793	HQE0316	Female	Others	2021	31	Married	Secondary	Private	Rural	Yes	Yes	Yes	No	No	No	No	No	No	No
3794	HQE0317	Female	Malay	2021	23	Single	Tertiary	Private	Rural	Yes	Yes	Yes	No	No	No	No	No	No	No
3795	HQE0318	Female	Others	2021	21	Single	Tertiary	Private	Rural	Yes	Yes	No	No	No	No	No	No	No	No
3796	HQE0319	Female	Chinese	2020	25	Married	Tertiary	Government	Rural	Yes	No	Yes	No	No	No	Yes	No	No	No
3797	HQE0321	Female	Others	2019	26	Married	Secondary	Private	Rural	Yes	No	Yes	No	No	No	No	No	No	No
3798	HQE0322	Female	Others	2020	13	Single	Secondary	Unemployed	Rural	Yes	Yes	Yes	No	No	No	No	No	No	No
3799	HQE0324	Male	Others	2020	48	Married	Tertiary	Government	Rural	Yes	Yes	Yes	No	No	No	No	No	No	No
3800	HQE0325	Male	Chinese	2019	55	Married	Tertiary	Government	Rural	Yes	No	Yes	No	No	No	No	No	No	No
3801	HQE0328	Male	Others	2018	59	Married	Secondary	Private	Rural	Yes	Yes	No	No	No	No	No	No	No	No
3802	HQE0331	Male	Others	2020	45	Married	Tertiary	Private	Rural	Yes	Yes	Yes	Yes	Yes	No	No	No	No	No
3803	HQE0333	Male	Others	2021	17	Single	Secondary	Unemployed	Rural	Yes	Yes	No	No	No	No	No	No	No	No
3804	HQE0335	Male	Others	2019	28	Married	Secondary	Government	Rural	Yes	Yes	Yes	No	No	No	No	No	No	No
3805	HQE0336	Male	Others	2021	54	Married	Secondary	Government	Rural	Yes	Yes	Yes	No	No	No	No	No	No	No
3806	HQE0337	Male	Others	2020	38	Married	Secondary	Government	Rural	Yes	Yes	Yes	No	No	No	No	No	No	No
3807	HQE0338	Female	Others	2018	32	Married	Secondary	Private	Rural	Yes	Yes	Yes	No	No	No	No	No	No	No
3808	HQE0340	Male	Others	2021	27	Others	Tertiary	Private	Rural	Yes	Yes	Yes	No	No	No	No	No	No	No
3809	HQE0341	Male	Others	2021	27	Single	Tertiary	Unemployed	Urban	Yes	No	Yes	No	No	No	No	No	No	No
3810	HQE0343	Male	Others	2020	43	Married	No formal education	Private	Rural	Yes	Yes	Yes	No	No	No	No	No	No	No
3811	HQE0344	Male	Others	2020	39	Married	Tertiary	Government	Rural	Yes	Yes	Yes	No	Yes	No	No	No	No	No
3812	HQE0345	Male	Others	2019	69	Married	Secondary	Others	Rural	Yes	No	Yes	No	Yes	No	No	No	No	No
3813	HQE0348	Male	Others	2020	42	Single	No formal education	Unemployed	Rural	Yes	No	Yes	No	No	No	No	No	No	No
3814	HQE0349	Male	Others	2019	59	Married	Tertiary	Others	Rural	Yes	Yes	Yes	No	No	No	No	No	No	No
3815	HQE0350	Male	Others	2019	30	Single	Secondary	Private	Rural	Yes	Yes	Yes	No	No	No	No	No	No	No
3816	HQE0351	Male	Others	2019	59	Married	Primary	Self-employed	Rural	Yes	No	Yes	No	No	No	No	No	No	No
3817	HQE0352	Male	Indian	2020	51	Married	Secondary	Private	Rural	Yes	Yes	No	No	Yes	No	No	No	No	No
3818	HQE0353	Male	Others	2019	55	Single	Primary	Self-employed	Rural	Yes	Yes	Yes	No	No	No	No	No	No	No
3819	HQE0354	Male	Others	2020	25	Single	Secondary	Private	Rural	Yes	Yes	Yes	No	Yes	No	No	No	No	No
3820	HQE0355	Male	Others	2019	42	Others	No formal education	Self-employed	Rural	Yes	Yes	Yes	No	No	No	No	No	No	No
3821	HQE0356	Male	Others	2020	49	Married	Secondary	Private	Rural	Yes	Yes	Yes	No	No	No	No	No	No	No
3822	HQE0358	Female	Others	2019	20	Single	Tertiary	Private	Rural	Yes	No	Yes	No	No	No	No	No	No	No

No	Patient ID	Gender	Ethnic group	Year of diagnosis	Age	Marital status	Education level	Occupation	Place of residence	History of psy illness	Tobacco	Alcohol	Cannabis	ATS	Inhalant	Sedative	Hallucinogens	Opioids	Kratom
3823	HQE0359	Female	Others	2021	34	Married	No formal education	Others	Rural	Yes	Yes	No	No	No	No	No	No	No	No
3824	HQE0361	Female	Others	2020	39	Married	Tertiary	Private	Rural	Yes	No	Yes	No	No	No	No	No	No	No
3825	HQE0363	Female	Others	2018	31	Others	No formal education	Self-employed	Rural	Yes	Yes	No	No	Yes	No	No	No	No	No
3826	HQE0364	Female	Others	2019	27	Married	Secondary	Unemployed	Rural	Yes	No	Yes	No	No	No	No	No	No	No
3827	HQE0365	Female	Others	2019	24	Single	Tertiary	Private	Rural	Yes	Yes	Yes	No	No	No	No	No	No	No
3828	HQE0366	Female	Others	2018	37	Married	No formal education	Private	Rural	Yes	Yes	No	No	No	No	No	No	No	No
3829	HQE0367	Female	Others	2018	23	Single	Tertiary	Private	Rural	Yes	Yes	Yes	No	No	No	No	No	No	No
3830	HQE0368	Male	Others	2020	39	Married	Secondary	Self-employed	Rural	Yes	Yes	No	No	No	No	No	No	No	No
3831	HQE0369	Male	Chinese	2020	42	Married	Tertiary	Self-employed	Rural	Yes	No	Yes	No	No	No	No	No	No	No
3832	HQE0371	Male	Others	2018	46	Married	Tertiary	Self-employed	Rural	Yes	Yes	No	No	No	No	No	No	No	No
3833	HQE0374	Female	Chinese	2019	36	Single	Secondary	Private	Rural	Yes	Yes	Yes	No	No	No	No	No	No	No
3834	HQE0375	Male	Others	2021	23	Single	Tertiary	Unemployed	Rural	Yes	Yes	No	No	No	No	No	No	No	No
3835	HQE0382	Female	Others	2018	20	Others	Secondary	Private	Rural	Yes	No	Yes	No	No	No	No	No	No	No
3836	HQE0383	Female	Others	2019	20	Single	Secondary	Private	Rural	Yes	Yes	No	No	No	No	No	No	No	No
3837	HQE0385	Female	Others	2019	26	Others	Secondary	Private	Rural	Yes	No	Yes	No	No	No	No	No	No	No
3838	HQE0387	Female	Chinese	2018	27	Others	Tertiary	Private	Urban	Yes	Yes	Yes	No	No	No	No	No	No	No
3839	HQE0388	Female	Chinese	2018	19	Others	Secondary	Private	Rural	Yes	Yes	Yes	No	No	No	No	No	No	No
3840	HQE0389	Female	Chinese	2020	15	Single	Secondary	Unemployed	Rural	Yes	No	Yes	No	No	No	No	No	No	No
3841	HQE0390	Female	Others	2020	28	Others	Tertiary	Government	Rural	Yes	Yes	No	No	No	No	No	No	No	No
3842	HQE0391	Female	Others	2021	43	Others	No formal education	Self-employed	Rural	Yes	Yes	Yes	No	No	No	No	No	No	No
3843	HQE0392	Female	Chinese	2019	30	Others	No formal education	Private	Urban	Yes	Yes	Yes	No	No	No	No	No	No	No
3844	HQE0393	Female	Chinese	2020	36	Married	Tertiary	Government	Rural	Yes	No	Yes	No	No	No	No	No	No	No
3845	HQE0394	Female	Others	2018	27	Single	Secondary	Private	Rural	Yes	Yes	Yes	No	No	No	No	No	No	No
3846	HQE0395	Female	Chinese	2020	17	Single	Secondary	Unemployed	Urban	Yes	Yes	Yes	No	No	No	No	No	No	No
3847	HQE0396	Female	Others	2019	24	Others	Secondary	Private	Rural	Yes	Yes	Yes	No	No	No	No	No	No	No
3848	HQE0397	Female	Malay	2019	23	Single	Tertiary	Unemployed	Rural	Yes	No	Yes	No	No	No	No	No	No	No
3849	HQE0398	Female	Others	2018	14	Single	Secondary	Unemployed	Rural	Yes	Yes	No	No	No	No	No	No	No	No
3850	HQE0399	Female	Others	2020	35	Single	Secondary	Private	Rural	Yes	No	Yes	No	No	No	No	No	No	No
3851	HQE0401	Female	Chinese	2020	44	Married	Tertiary	Private	Rural	Yes	No	Yes	No	No	No	No	No	No	No
3852	HQE0402	Female	Others	2019	26	Others	No formal education	Private	Rural	Yes	Yes	Yes	No	No	No	No	No	No	No
3853	HQE0403	Female	Others	2018	46	Married	Secondary	Government	Rural	Yes	Yes	No	No	No	No	No	No	No	No
3854	HQE0404	Female	Chinese	2018	55	Married	No formal education	Others	Rural	Yes	Yes	No	No	No	No	No	No	No	No
3855	HQE0406	Male	Malay	2018	36	Married	Secondary	Private	Rural	Yes	Yes	No	No	No	No	No	No	No	No
3856	HQE0407	Male	Others	2018	44	Single	No formal education	Private	Rural	Yes	No	Yes	No	No	No	No	No	No	No
3857	HQE0408	Female	Chinese	2020	75	Married	Secondary	Unemployed	Rural	Yes	No	Yes	No	No	No	No	No	No	No
3858	HQE0409	Male	Others	2018	34	Married	Secondary	Private	Rural	Yes	No	Yes	No	No	No	No	No	No	No

No	Patient ID	Gender	Ethnic group	Year of diagnosis	Age	Marital status	Education level	Occupation	Place of residence	History of psy illness	Tobacco	Alcohol	Cannabis	ATS	Inhalant	Sedative	Hallucinogens	Opioids	Kratom
3859	HQE0410	Male	Others	2019	36	Married	Secondary	Private	Rural	Yes	No	Yes	No	No	No	No	No	No	No
3860	HQE0412	Male	Others	2019	67	Married	Secondary	Others	Rural	Yes	Yes	No	No	No	No	No	No	No	No
3861	HQE0415	Male	Chinese	2019	22	Single	Secondary	Unemployed	Rural	Yes	No	Yes	No	No	No	No	No	No	No
3862	HQE0416	Male	Others	2018	24	Married	Secondary	Private	Urban	Yes	No	Yes	No	No	No	No	No	No	No
3863	HQE0420	Male	Others	2018	24	Others	No formal education	Self-employed	Rural	Yes	Yes	No	No	No	No	No	No	No	No
3864	HQE0421	Male	Others	2020	26	Married	No formal education	Private	Rural	Yes	Yes	No	No	Yes	No	No	No	No	No
3865	HQE0423	Male	Others	2020	22	Single	No formal education	Unemployed	Rural	Yes	Yes	No	No	No	Yes	No	No	No	No
3866	HQE0424	Male	Others	2020	50	Single	No formal education	Unemployed	Rural	Yes	Yes	Yes	No	No	No	No	No	No	No
3867	HQE0425	Male	Chinese	2019	19	Single	No formal education	Private	Rural	Yes	Yes	Yes	No	No	No	No	No	No	No
3868	HQE0426	Male	Chinese	2019	59	Others	Secondary	Unemployed	Rural	Yes	No	Yes	No	No	No	No	No	No	No
3869	HQE0428	Male	Others	2020	39	Others	Tertiary	Unemployed	Rural	Yes	No	Yes	No	No	No	No	No	No	No
3870	HQE0429	Male	Malay	2019	25	Single	Tertiary	Private	Urban	Yes	Yes	Yes	No	No	No	No	No	No	No
3871	HQE0430	Male	Chinese	2020	58	Married	Primary	Self-employed	Rural	Yes	No	Yes	No	No	No	No	No	No	No
3872	HQE0431	Male	Others	2020	21	Single	Tertiary	Unemployed	Rural	Yes	Yes	Yes	No	No	No	No	No	No	No
3873	HQE0432	Male	Others	2020	78	Others	No formal education	Others	Rural	Yes	Yes	No	No	No	No	No	No	No	No
3874	HQE0433	Male	Chinese	2020	71	Married	No formal education	Others	Rural	Yes	Yes	No	No	No	No	No	No	No	No
3875	HQE0434	Male	Others	2019	54	Others	Secondary	Self-employed	Rural	Yes	Yes	Yes	No	No	No	No	No	No	No
3876	HQE0435	Male	Others	2018	61	Married	Tertiary	Others	Rural	Yes	No	Yes	No	No	No	No	No	No	No
3877	HQE0436	Male	Others	2018	31	Married	Tertiary	Government	Rural	Yes	Yes	No	No	No	No	No	No	No	No
3878	HQE0437	Male	Others	2020	36	Married	Tertiary	Private	Rural	Yes	Yes	Yes	No	Yes	No	No	No	No	No
3879	HQE0438	Male	Chinese	2019	67	Married	Secondary	Others	Rural	Yes	Yes	Yes	No	No	No	No	No	No	No
3880	HQE0439	Male	Others	2018	24	Single	Secondary	Private	Rural	Yes	Yes	Yes	Yes	Yes	No	No	No	No	No
3881	HQE0440	Male	Others	2018	72	Married	No formal education	Unemployed	Rural	Yes	Yes	Yes	No	No	No	No	No	No	No
3882	HQE0441	Male	Others	2018	29	Married	Tertiary	Private	Rural	Yes	Yes	No	No	No	No	No	No	No	No
3883	HQE0442	Male	Chinese	2018	37	Married	Tertiary	Private	Rural	Yes	Yes	Yes	No	No	No	No	No	No	No
3884	HQE0443	Male	Malay	2019	36	Married	Secondary	Private	Rural	Yes	Yes	Yes	Yes	Yes	No	No	No	No	No
3885	HQE0444	Male	Others	2019	33	Others	Tertiary	Unemployed	Urban	Yes	Yes	Yes	No	No	No	No	No	No	No
3886	HQE0445	Male	Others	2019	22	Single	Primary	Private	Rural	Yes	No	Yes	No	No	No	No	No	No	No
3887	HQE0446	Male	Others	2020	29	Single	No formal education	Unemployed	Rural	Yes	Yes	Yes	No	Yes	Yes	No	No	No	No
3888	HQE0447	Male	Others	2019	26	Single	Secondary	Private	Urban	Yes	Yes	Yes	No	No	No	No	No	No	No
3889	HQE0448	Male	Others	2019	30	Single	No formal education	Self-employed	Rural	Yes	Yes	Yes	No	No	No	No	No	No	No
3890	HQE0449	Male	Others	2019	46	Single	Tertiary	Private	Rural	Yes	Yes	Yes	No	No	No	No	No	No	No
3891	HQE0450	Male	Others	2020	10	Single	Primary	Unemployed	Rural	Yes	Yes	No	No	No	No	No	No	No	No

No	Patient ID	Gender	Ethnic group	Year of diagnosis	Age	Marital status	Education level	Occupation	Place of residence	History of psy illness	Tobacco	Alcohol	Cannabis	ATS	Inhalant	Sedative	Hallucinogens	Opioids	Kratom
3892	HQE0451	Male	Chinese	2018	47	Others	No formal education	Unemployed	Rural	Yes	Yes	Yes	No	Yes	No	No	No	No	No
3893	HQE0452	Male	Indian	2019	41	Married	No formal education	Self-employed	Rural	Yes	Yes	Yes	No	No	No	Yes	No	No	No
3894	HQE0453	Female	Others	2018	37	Married	Tertiary	Government	Rural	Yes	Yes	Yes	No	No	No	No	No	No	No
3895	HQE0455	Female	Others	2019	22	Single	Secondary	Private	Rural	Yes	No	Yes	No	No	No	No	No	No	No
3896	HQE0456	Female	Others	2020	20	Single	Tertiary	Private	Rural	Yes	No	Yes	No	No	No	No	No	No	No
3897	HQE0457	Female	Chinese	2020	22	Single	Secondary	Private	Rural	Yes	No	Yes	No	No	No	No	No	No	No
3898	HQE0459	Female	Others	2020	24	Married	Secondary	Unemployed	Rural	Yes	Yes	Yes	No	No	No	No	No	No	No
3899	HQE0460	Female	Others	2020	22	Married	Secondary	Unemployed	Rural	Yes	No	Yes	No	No	No	No	No	No	No
3900	HQE0461	Female	Others	2019	19	Single	Secondary	Private	Rural	Yes	Yes	Yes	No	No	No	No	No	No	No
3901	HQE0462	Female	Others	2018	29	Others	Primary	Private	Rural	Yes	Yes	No	No	No	No	No	No	No	No
3902	HQE0463	Female	Others	2020	23	Single	Tertiary	Private	Rural	Yes	No	Yes	No	No	No	No	No	No	No
3903	HQE0464	Female	Others	2018	25	Single	Tertiary	Private	Rural	Yes	Yes	Yes	No	No	No	No	No	No	No
3904	HQE0465	Female	Chinese	2020	30	Married	Secondary	Self-employed	Urban	Yes	Yes	Yes	No	No	No	No	No	No	No
3905	HQE0466	Female	Others	2019	24	Single	Tertiary	Private	Rural	Yes	Yes	Yes	No	No	No	No	No	No	No
3906	HQE0467	Female	Others	2018	22	Single	Secondary	Private	Rural	Yes	Yes	Yes	No	No	No	No	No	No	No
3907	HQE0468	Female	Others	2020	16	Single	Secondary	Unemployed	Urban	Yes	No	Yes	No	No	No	No	No	No	No
3908	HQE0469	Female	Others	2018	25	Single	Tertiary	Unemployed	Rural	Yes	Yes	No	No	No	No	No	No	No	No
3909	HQE0470	Female	Others	2018	30	Others	Primary	Private	Rural	Yes	Yes	Yes	No	No	No	No	No	No	No
3910	HQE0471	Female	Others	2019	27	Single	Tertiary	Private	Rural	Yes	No	Yes	No	No	No	No	No	No	No
3911	HQE0472	Female	Others	2018	23	Single	Tertiary	Private	Urban	Yes	Yes	Yes	No	No	No	No	No	No	No
3912	HQE0473	Female	Others	2018	24	Married	Primary	Unemployed	Rural	Yes	Yes	Yes	No	No	No	No	No	No	No
3913	HQE0474	Female	Others	2020	32	Married	Primary	Unemployed	Rural	Yes	No	Yes	No	No	No	No	No	No	No
3914	HQE0475	Female	Others	2018	68	Married	Tertiary	Others	Rural	Yes	No	Yes	No	No	No	No	No	No	No
3915	HQE0476	Female	Chinese	2019	46	Married	Primary	Unemployed	Rural	Yes	No	Yes	No	No	No	No	No	No	No
3916	HQE0477	Female	Others	2019	28	Married	Tertiary	Government	Rural	Yes	No	Yes	No	No	No	No	No	No	No
3917	HQE0478	Female	Others	2020	49	Others	Secondary	Self-employed	Rural	Yes	No	Yes	No	No	No	No	No	No	No
3918	HQE0479	Female	Others	2019	24	Single	Tertiary	Private	Urban	Yes	No	Yes	No	No	No	No	No	No	No
3919	HQE0480	Female	Others	2019	21	Others	Secondary	Unemployed	Rural	Yes	No	Yes	No	No	No	No	No	No	No
3920	HQE0481	Female	Others	2020	29	Married	Primary	Unemployed	Rural	Yes	Yes	Yes	No	No	No	No	No	No	No
3921	HQE0482	Female	Chinese	2020	25	Married	Secondary	Unemployed	Urban	Yes	No	Yes	No	No	No	No	No	No	No
3922	HQE0483	Female	Others	2020	15	Single	Primary	Private	Rural	Yes	Yes	Yes	No	No	No	No	No	No	No
3923	HQE0484	Female	Others	2020	32	Others	Tertiary	Private	Urban	Yes	No	Yes	No	No	No	No	No	No	No
3924	HQE0485	Female	Others	2020	48	Others	Primary	Unemployed	Rural	Yes	No	Yes	No	No	No	No	No	No	No
3925	HQE0486	Female	Others	2020	41	Married	Secondary	Government	Rural	Yes	No	Yes	No	No	No	No	No	No	No
3926	HQE0487	Female	Chinese	2019	22	Single	Tertiary	Unemployed	Rural	Yes	No	Yes	No	No	No	No	No	No	No
3927	HQE0489	Male	Others	2019	46	Married	Tertiary	Self-employed	Rural	Yes	Yes	Yes	No	No	No	No	No	No	No
3928	HQE0492	Female	Others	2018	20	Others	Secondary	Unemployed	Rural	Yes	No	Yes	No	No	No	No	No	No	No
3929	HQE0493	Female	Others	2018	28	Others	Tertiary	Private	Rural	Yes	No	Yes	No	No	No	No	No	No	No
3930	HQE0494	Female	Others	2018	26	Married	No formal education	Unemployed	Rural	Yes	Yes	No	No	Yes	No	No	No	No	No
3931	HQE0495	Female	Others	2019	19	Single	Secondary	Unemployed	Rural	Yes	No	Yes	No	No	No	No	No	No	No
3932	HQE0496	Female	Others	2019	18	Married	Secondary	Unemployed	Rural	Yes	Yes	No	No	No	No	No	No	No	No

No	Patient ID	Gender	Ethnic group	Year of diagnosis	Age	Marital status	Education level	Occupation	Place of residence	History of psy illness	Tobacco	Alcohol	Cannabis	ATS	Inhalant	Sedative	Hallucinogens	Opioids	Kratom
3933	HQE0497	Female	Others	2018	56	Married	No formal education	Unemployed	Rural	Yes	No	Yes	No	No	No	No	No	No	No
3934	HQE0498	Female	Chinese	2020	33	Others	Secondary	Self-employed	Rural	Yes	Yes	Yes	No	No	No	No	No	No	No
3935	HQE0499	Female	Indian	2019	22	Single	Tertiary	Unemployed	Rural	Yes	No	Yes	No	No	No	No	No	No	No
3936	HQE0500	Female	Others	2020	21	Single	Primary	Private	Rural	Yes	Yes	No	No	No	No	No	No	No	No
3937	HQE0501	Female	Others	2019	22	Single	No formal education	Unemployed	Rural	Yes	Yes	Yes	No	Yes	No	No	No	No	No
3938	HQE0503	Female	Others	2019	23	Single	Tertiary	Private	Rural	Yes	Yes	No	No	No	No	No	No	No	No
3939	HQE0504	Female	Others	2019	20	Single	Primary	Private	Rural	Yes	Yes	Yes	No	No	No	No	No	No	No
3940	HQE0506	Female	Chinese	2019	18	Single	Secondary	Private	Rural	Yes	No	Yes	No	No	No	No	No	No	No
3941	HQE0507	Female	Chinese	2019	43	Others	Tertiary	Private	Urban	Yes	Yes	Yes	No	No	No	No	No	No	No
3942	HQE0508	Female	Others	2019	45	Married	Secondary	Self-employed	Urban	Yes	Yes	No	No	No	No	No	No	No	No
3943	HQE0509	Female	Others	2018	46	Others	No formal education	Unemployed	Rural	Yes	Yes	Yes	No	No	No	No	No	No	No
3944	HQE0511	Male	Others	2018	32	Single	No formal education	Self-employed	Rural	Yes	No	Yes	No	No	No	No	No	No	No
3945	HQE0512	Male	Chinese	2019	55	Married	No formal education	Unemployed	Rural	Yes	No	Yes	No	No	No	No	No	No	No
3946	HQE0513	Male	Others	2020	73	Married	No formal education	Others	Rural	Yes	No	Yes	No	No	No	No	No	No	No
3947	HQE0515	Male	Others	2019	31	Married	Tertiary	Government	Rural	Yes	Yes	No	No	Yes	No	No	No	No	No
3948	HQE0516	Male	Chinese	2021	43	Single	No formal education	Private	Rural	Yes	Yes	Yes	No	Yes	No	No	No	No	No
3949	HQE0517	Male	Others	2018	44	Single	No formal education	Self-employed	Rural	Yes	Yes	Yes	No	No	No	No	No	No	No
3950	HQE0518	Male	Others	2020	33	Others	Secondary	Private	Rural	Yes	No	Yes	No	No	No	No	No	No	No
3951	HQE0520	Male	Others	2021	32	Single	Secondary	Unemployed	Rural	Yes	Yes	Yes	No	No	No	No	No	No	No
3952	HQE0523	Male	Malay	2018	28	Single	Tertiary	Government	Urban	Yes	Yes	No	No	No	No	No	No	No	No
3953	HQE0524	Male	Others	2020	38	Single	Secondary	Self-employed	Rural	Yes	Yes	Yes	No	No	No	No	No	No	No
3954	HQE0525	Male	Others	2019	32	Single	Secondary	Others	Urban	Yes	Yes	No	No	No	No	No	No	No	No
3955	HQE0526	Male	Others	2021	33	Married	Primary	Self-employed	Rural	Yes	Yes	Yes	No	No	No	No	No	No	No
3956	HQE0527	Male	Others	2020	22	Single	Primary	Private	Rural	Yes	Yes	No	No	No	No	No	No	No	No
3957	HQE0528	Male	Others	2020	56	Married	No formal education	Self-employed	Rural	Yes	No	Yes	No	No	No	No	No	No	No
3958	HQE0529	Male	Others	2019	24	Single	Tertiary	Unemployed	Rural	Yes	Yes	No	No	Yes	No	No	No	No	No
3959	HQE0530	Male	Others	2020	53	Single	Secondary	Unemployed	Rural	Yes	Yes	Yes	No	Yes	No	No	No	No	No
3960	HQE0531	Male	Others	2018	17	Single	Primary	Private	Rural	Yes	No	Yes	No	No	No	No	No	No	No
3961	HQE0532	Male	Others	2020	36	Single	Tertiary	Private	Rural	Yes	Yes	No	No	Yes	No	No	No	No	No
3962	HQE0533	Male	Others	2021	57	Married	No formal education	Self-employed	Rural	Yes	Yes	Yes	No	No	No	No	No	No	No
3963	HQE0534	Male	Chinese	2019	65	Married	No formal education	Unemployed	Rural	Yes	Yes	Yes	No	No	No	No	No	No	No
3964	HQE0535	Male	Others	2018	50	Single	Secondary	Self-employed	Rural	Yes	Yes	No	No	No	No	No	No	No	No
3965	HQE0536	Male	Others	2019	24	Single	Primary	Private	Rural	Yes	Yes	No	No	No	No	No	No	No	No

No	Patient ID	Gender	Ethnic group	Year of diagnosis	Age	Marital status	Education level	Occupation	Place of residence	History of psy illness	Tobacco	Alcohol	Cannabis	ATS	Inhalant	Sedative	Hallucinogens	Opioids	Kratom
3966	HQE0537	Male	Malay	2020	23	Single	Tertiary	Unemployed	Rural	Yes	No	Yes	No	Yes	No	No	No	No	No
3967	HQE0538	Male	Chinese	2019	34	Single	Secondary	Private	Urban	Yes	Yes	Yes	No	No	No	No	No	No	No
3968	HQE0539	Male	Others	2019	35	Married	Secondary	Government	Rural	Yes	Yes	No	No	No	No	No	No	No	No
3969	HQE0540	Male	Others	2019	49	Married	Secondary	Private	Rural	Yes	Yes	Yes	No	No	No	No	No	No	No
3970	HQE0541	Male	Others	2020	33	Single	Secondary	Government	Rural	Yes	Yes	Yes	Yes	No	No	No	No	No	No
3971	HQE0542	Male	Others	2018	25	Single	Tertiary	Unemployed	Rural	Yes	Yes	Yes	No	No	No	No	No	No	No
3972	HQE0543	Male	Others	2020	31	Single	Primary	Self-employed	Rural	Yes	No	Yes	No	No	No	No	No	No	No
3973	HQE0544	Male	Others	2019	46	Married	Secondary	Unemployed	Rural	Yes	No	Yes	No	No	No	No	No	No	No
3974	HQE0545	Male	Others	2018	72	Married	Primary	Unemployed	Rural	Yes	Yes	No	No	No	No	No	No	No	No
3975	HQE0546	Male	Others	2019	37	Single	Secondary	Private	Rural	Yes	Yes	Yes	No	No	No	No	No	No	No
3976	HQE0547	Male	Others	2019	43	Married	Primary	Private	Rural	Yes	No	Yes	No	No	No	No	No	No	No
3977	HQE0548	Male	Chinese	2019	67	Married	Secondary	Unemployed	Rural	Yes	No	Yes	No	No	No	No	No	No	No
3978	HQE0549	Male	Others	2020	25	Married	No formal education	Private	Rural	Yes	Yes	No	No	Yes	No	No	No	No	No
3979	HQE0549	Male	Others	2020	50	Others	No formal education	Self-employed	Rural	Yes	Yes	Yes	No	No	No	No	No	No	No
3980	HQE0550	Male	Others	2021	24	Single	Tertiary	Government	Rural	Yes	Yes	No	Yes	Yes	No	No	No	No	No
3981	HQE0551	Male	Others	2018	28	Married	No formal education	Self-employed	Rural	Yes	Yes	Yes	No	No	No	No	No	No	No
3982	HQE0552	Male	Others	2020	34	Married	Primary	Private	Rural	Yes	Yes	Yes	No	No	No	No	No	No	No
3983	HQE0553	Male	Chinese	2019	39	Single	Secondary	Self-employed	Urban	Yes	Yes	Yes	No	No	No	No	No	No	No
3984	HQE0554	Male	Others	2021	43	Married	Primary	Self-employed	Rural	Yes	Yes	Yes	No	No	No	No	No	No	No
3985	HQE0555	Male	Malay	2021	50	Others	Tertiary	Self-employed	Urban	Yes	No	Yes	No	No	No	No	No	No	No
3986	HQE0556	Male	Others	2020	18	Single	Primary	Unemployed	Rural	Yes	Yes	Yes	No	Yes	No	No	No	No	No
3987	HQE0557	Male	Chinese	2018	62	Others	No formal education	Unemployed	Rural	Yes	Yes	Yes	No	No	No	No	No	No	No
3988	HQE0558	Male	Others	2019	27	Single	Tertiary	Government	Rural	Yes	Yes	No	No	No	No	No	No	No	No
3989	HQE0559	Male	Chinese	2019	31	Married	Tertiary	Self-employed	Urban	Yes	Yes	Yes	No	No	No	No	No	No	No
3990	HQE0560	Male	Others	2021	45	Single	No formal education	Self-employed	Rural	Yes	Yes	No	No	Yes	No	No	No	No	No
3991	HQE0561	Male	Others	2018	29	Married	Secondary	Unemployed	Rural	Yes	Yes	No	No	Yes	No	No	No	No	No
3992	HQE0562	Male	Others	2020	33	Single	Tertiary	Private	Rural	Yes	Yes	Yes	No	No	No	No	No	No	No
3993	HQE0563	Male	Chinese	2019	34	Married	Secondary	Private	Rural	Yes	Yes	Yes	No	Yes	No	No	No	No	No
3994	HQE0566	Male	Others	2018	43	Married	Secondary	Government	Rural	Yes	Yes	Yes	No	No	Yes	No	No	No	No
3995	HQE0567	Male	Others	2018	31	Others	Primary	Private	Rural	Yes	Yes	Yes	No	Yes	No	No	No	No	No
3996	HQE0570	Male	Malay	2019	30	Single	Tertiary	Government	Rural	Yes	Yes	No	No	Yes	No	No	No	No	No
3997	HQE0571	Male	Malay	2019	45	Others	Tertiary	Private	Rural	Yes	Yes	No	No	No	No	No	No	No	No
3998	HQE0573	Male	Others	2019	25	Single	No formal education	Private	Rural	Yes	Yes	Yes	No	No	No	No	No	No	No
3999	HQE0574	Male	Others	2019	52	Married	No formal education	Self-employed	Rural	Yes	No	Yes	No	No	No	No	No	No	No
4000	HQE0575	Male	Others	2018	39	Married	No formal education	Private	Rural	Yes	Yes	Yes	No	Yes	No	No	No	No	No

No	Patient ID	Gender	Ethnic group	Year of diagnosis	Age	Marital status	Education level	Occupation	Place of residence	History of psy illness	Tobacco	Alcohol	Cannabis	ATS	Inhalant	Sedative	Hallucinogens	Opioids	Kratom
4001	HQE0576	Male	Others	2018	25	Others	Primary	Unemployed	Rural	Yes	Yes	Yes	No	No	No	No	No	No	No
4002	HQE0577	Male	Others	2019	43	Others	No formal education	Unemployed	Rural	Yes	Yes	No	No	No	No	No	No	No	No
4003	HQE0579	Male	Others	2020	26	Single	Secondary	Unemployed	Rural	Yes	Yes	No	No	No	No	No	No	No	No
4004	HQE0580	Male	Others	2018	24	Others	Primary	Private	Rural	Yes	Yes	Yes	Yes	No	No	No	No	No	No
4005	HQE0581	Male	Others	2020	23	Others	Secondary	Private	Rural	Yes	Yes	Yes	No	No	No	No	No	No	No
4006	HQE0583	Male	Chinese	2019	34	Married	Secondary	Self-employed	Rural	Yes	No	Yes	No	No	No	No	No	No	No
4007	HQE0584	Male	Others	2021	42	Single	No formal education	Self-employed	Rural	Yes	Yes	Yes	No	Yes	No	No	No	No	No
4008	HQE0586	Male	Others	2018	28	Single	Secondary	Self-employed	Rural	Yes	Yes	No	No	No	No	No	No	No	No
4009	HQE0587	Male	Others	2018	17	Single	Primary	Unemployed	Rural	Yes	Yes	No	No	No	No	No	No	No	No
4010	HQE0588	Male	Others	2018	29	Single	No formal education	Self-employed	Rural	Yes	Yes	Yes	No	No	No	No	No	No	No
4011	HQE0590	Male	Others	2018	11	Single	Primary	Unemployed	Urban	Yes	Yes	No	No	No	Yes	No	No	No	No
4012	HQE0591	Male	Others	2020	33	Married	Primary	Private	Rural	Yes	Yes	Yes	No	No	No	No	No	No	No
4013	HQE0592	Female	Chinese	2020	16	Single	Primary	Unemployed	Rural	Yes	Yes	No	No	No	No	No	No	No	No
4014	HQE0593	Male	Others	2018	20	Single	Secondary	Self-employed	Rural	Yes	No	Yes	No	No	No	No	No	No	No
4015	HQE0594	Male	Others	2018	44	Single	No formal education	Self-employed	Rural	Yes	Yes	Yes	No	Yes	No	No	No	No	No
4016	HQE0595	Male	Chinese	2018	47	Single	Secondary	Private	Urban	Yes	No	Yes	No	No	No	No	No	No	No
4017	HQE0596	Male	Others	2018	70	Married	No formal education	Others	Rural	Yes	No	Yes	No	No	No	No	No	No	No
4018	HQE0598	Male	Others	2019	45	Single	Primary	Private	Rural	Yes	Yes	No	No	No	No	No	No	No	No
4019	HQE0599	Female	Others	2018	26	Others	Secondary	Private	Rural	Yes	Yes	Yes	No	No	No	No	No	No	No
4020	HQE0600	Male	Others	2021	37	Married	Primary	Private	Rural	Yes	Yes	Yes	No	Yes	No	No	No	No	No
4021	HQE0601	Male	Others	2021	66	Married	Secondary	Self-employed	Rural	Yes	Yes	No	No	No	No	No	No	No	No
4022	HQE0602	Male	Chinese	2018	37	Married	Secondary	Private	Rural	Yes	Yes	Yes	No	Yes	No	No	No	No	No
4023	HQE0603	Male	Others	2018	35	Others	Secondary	Private	Rural	Yes	Yes	No	No	No	No	No	No	No	No
4024	HQE0604	Male	Chinese	2019	26	Single	Primary	Private	Rural	Yes	Yes	Yes	No	No	No	No	No	No	No
4025	HQE0605	Male	Others	2021	37	Single	Secondary	Private	Rural	No	Yes	Yes	No	No	No	No	No	No	No
4026	HQE0606	Male	Others	2020	28	Single	Tertiary	Self-employed	Rural	Yes	Yes	No	No	No	No	No	No	No	No
4027	HQE0607	Male	Others	2019	23	Single	No formal education	Self-employed	Rural	Yes	Yes	No	No	No	No	No	No	No	No
4028	HQE0608	Male	Malay	2018	29	Single	Secondary	Private	Urban	Yes	Yes	No	No	No	No	No	No	No	No
4029	HQE0610	Male	Chinese	2021	64	Married	Primary	Private	Rural	Yes	Yes	Yes	No	No	No	No	No	No	No
4030	HQE0611	Male	Others	2021	27	Single	Secondary	Private	Rural	Yes	No	Yes	No	No	No	No	No	No	No
4031	HQE0612	Male	Others	2019	31	Single	Tertiary	Private	Rural	Yes	Yes	Yes	No	No	No	No	No	No	No
4032	HQE0613	Male	Others	2019	48	Married	Primary	Government	Rural	Yes	Yes	Yes	No	No	No	No	No	No	No
4033	HQE0614	Male	Others	2019	41	Single	Tertiary	Private	Rural	Yes	Yes	Yes	No	No	No	No	No	No	No
4034	HQE0615	Male	Others	2019	36	Single	Primary	Private	Rural	Yes	Yes	Yes	No	Yes	No	No	No	No	No
4035	HQE0616	Male	Others	2020	34	Single	Primary	Self-employed	Rural	Yes	Yes	Yes	No	No	No	No	No	No	No
4036	HQE0617	Male	Others	2019	30	Married	Primary	Self-employed	Rural	Yes	Yes	Yes	No	No	No	No	No	No	No
4037	HQE0619	Male	Others	2019	24	Single	No formal education	Self-employed	Rural	Yes	No	Yes	No	No	No	No	No	No	No

No	Patient ID	Gender	Ethnic group	Year of diagnosis	Age	Marital status	Education level	Occupation	Place of residence	History of psy illness	Tobacco	Alcohol	Cannabis	ATS	Inhalant	Sedative	Hallucinogens	Opioids	Kratom
4038	HQE0620	Male	Others	2019	25	Single	No formal education	Unemployed	Rural	Yes	Yes	Yes	No	Yes	No	No	No	No	No
4039	HQE0621	Male	Others	2018	38	Single	No formal education	Unemployed	Urban	No	No	No	No	No	Yes	No	No	No	No
4040	HQE0622	Male	Chinese	2019	21	Married	Secondary	Private	Rural	Yes	No	Yes	No	No	No	No	No	No	No
4041	HQE0624	Male	Others	2019	69	Married	Secondary	Others	Rural	No	Yes	No	No	No	No	No	No	No	No
4042	HQE0625	Male	Others	2018	39	Married	No formal education	Self-employed	Rural	Yes	Yes	Yes	No	No	No	No	No	No	No
4043	HQE0626	Male	Others	2020	18	Single	Tertiary	Unemployed	Rural	Yes	Yes	Yes	No	No	No	No	No	No	No
4044	HQE0627	Male	Others	2020	43	Single	Secondary	Self-employed	Rural	No	No	Yes	No	No	No	No	No	No	No
4045	HQE0628	Male	Others	2018	51	Married	Primary	Government	Rural	Yes	Yes	No	No	No	No	No	No	No	No
4046	HQE0630	Male	Others	2019	29	Married	No formal education	Self-employed	Urban	No	Yes	Yes	Yes	No	No	No	No	No	No
4047	HQE0631	Male	Others	2021	42	Single	Primary	Private	Rural	Yes	Yes	Yes	No	No	No	No	No	No	No
4048	HQE0632	Male	Chinese	2020	58	Married	Secondary	Unemployed	Urban	Yes	Yes	Yes	No	No	No	No	No	No	No
4049	HQE0633	Male	Chinese	2018	40	Single	Secondary	Private	Rural	Yes	No	Yes	No	No	No	No	No	No	No
4050	HQE0634	Male	Others	2020	29	Single	Secondary	Private	Rural	Yes	Yes	Yes	No	Yes	No	No	No	No	No
4051	HQE0635	Male	Others	2019	37	Single	Tertiary	Private	Rural	Yes	Yes	No	No	No	No	No	No	No	No
4052	HQE0636	Male	Others	2020	46	Married	Secondary	Government	Rural	Yes	Yes	Yes	No	No	No	No	No	No	No
4053	HQE0637	Male	Others	2021	23	Single	No formal education	Unemployed	Urban	Yes	Yes	Yes	No	No	No	No	No	No	No
4054	HQE0639	Male	Others	2020	24	Single	Primary	Private	Rural	Yes	Yes	Yes	No	Yes	No	No	No	No	No
4055	HQE0640	Male	Others	2021	47	Others	Primary	Unemployed	Rural	Yes	Yes	Yes	No	No	No	No	No	No	No
4056	HQE0641	Male	Chinese	2019	40	Married	Secondary	Private	Urban	Yes	No	Yes	No	No	No	No	No	No	No
4057	HQE0642	Male	Others	2020	22	Single	Primary	Private	Rural	Yes	Yes	Yes	No	No	No	No	No	No	No
4058	HQE0643	Male	Others	2018	50	Married	Primary	Self-employed	Rural	Yes	No	Yes	No	No	No	No	No	No	No
4059	HQE0644	Male	Others	2019	53	Married	No formal education	Self-employed	Rural	Yes	Yes	No	No	No	No	No	No	No	No
4060	HQE0645	Male	Others	2020	70	Married	Primary	Unemployed	Rural	Yes	Yes	Yes	No	No	No	No	No	No	No
4061	HQE0646	Male	Chinese	2020	63	Married	Secondary	Self-employed	Urban	Yes	No	Yes	No	No	No	No	No	No	No
4062	HQE0647	Male	Chinese	2018	20	Single	Secondary	Private	Urban	Yes	Yes	Yes	No	No	No	No	No	No	No
4063	HQE0648	Male	Others	2019	31	Married	Secondary	Private	Urban	Yes	Yes	Yes	No	Yes	No	No	No	No	No
4064	HQE0649	Male	Others	2020	30	Married	Secondary	Private	Rural	Yes	No	Yes	No	No	No	No	No	No	No
4065	HQE0650	Male	Others	2019	65	Married	Tertiary	Private	Rural	Yes	Yes	Yes	No	No	No	No	No	No	No
4066	HQE0652	Female	Others	2020	50	Others	No formal education	Private	Rural	Yes	Yes	Yes	No	No	No	No	No	No	No
4067	HQE0653	Male	Others	2018	49	Married	Secondary	Private	Urban	Yes	Yes	No	No	No	No	No	No	No	No
4068	HQE0654	Male	Others	2020	35	Married	No formal education	Private	Rural	Yes	Yes	No	No	No	No	No	No	No	No
4069	HQE0655	Male	Others	2018	30	Married	Tertiary	Self-employed	Urban	Yes	Yes	Yes	No	No	No	No	No	No	No
4070	HQE0656	Female	Others	2018	27	Married	No formal education	Unemployed	Rural	Yes	Yes	Yes	No	Yes	No	No	No	No	No
4071	HQE0657	Male	Chinese	2019	42	Married	Tertiary	Private	Urban	Yes	No	Yes	No	No	No	No	No	No	No

No	Patient ID	Gender	Ethnic group	Year of diagnosis	Age	Marital status	Education level	Occupation	Place of residence	History of psy illness	Tobacco	Alcohol	Cannabis	ATS	Inhalant	Sedative	Hallucinogens	Opioids	Kratom
4072	HQE0658	Female	Others	2019	28	Married	No formal education	Private	Rural	Yes	Yes	No	No	Yes	No	No	No	No	No
4073	HQE0659	Male	Others	2020	23	Single	Secondary	Unemployed	Urban	Yes	Yes	Yes	No	No	No	No	No	No	No
4074	HQE0661	Male	Others	2018	22	Single	No formal education	Private	Rural	Yes	No	No	No	Yes	No	No	No	No	No
4075	HQE0662	Female	Others	2020	40	Married	No formal education	Unemployed	Rural	Yes	Yes	No	No	No	No	No	No	No	No
4076	HQE0663	Female	Others	2020	21	Single	Primary	Private	Rural	Yes	No	Yes	No	No	No	No	No	No	No
4077	HQE0665	Female	Others	2021	20	Single	Primary	Private	Urban	Yes	Yes	Yes	No	No	No	No	No	No	No
4078	HQE0666	Female	Others	2020	26	Others	No formal education	Private	Urban	Yes	Yes	Yes	No	No	No	No	No	No	No
4079	HQE0667	Female	Others	2019	39	Others	No formal education	Unemployed	Rural	Yes	No	No	No	Yes	No	No	No	No	No
4080	HQE0668	Female	Others	2019	27	Single	Secondary	Private	Urban	Yes	No	Yes	No	No	No	No	No	No	No
4081	HQE0669	Female	Others	2020	20	Single	No formal education	Unemployed	Rural	Yes	Yes	Yes	No	No	No	No	No	No	No
4082	HQE0670	Female	Others	2020	34	Married	Primary	Private	Rural	Yes	No	Yes	No	No	No	No	No	No	No
4083	HQE0671	Male	Others	2019	22	Single	Secondary	Self-employed	Rural	Yes	Yes	Yes	No	No	No	No	No	No	No
4084	HQE0672	Male	Others	2020	18	Single	Primary	Unemployed	Rural	Yes	Yes	No	No	No	No	No	No	No	No
4085	HQE0673	Male	Others	2020	32	Married	No formal education	Private	Urban	Yes	Yes	Yes	No	Yes	No	No	No	No	No
4086	HQE0674	Male	Others	2019	18	Single	Tertiary	Unemployed	Rural	Yes	No	Yes	No	No	No	No	No	No	No
4087	HQE0675	Male	Others	2020	42	Married	No formal education	Private	Rural	Yes	Yes	Yes	No	No	No	No	No	No	No
4088	HQE0676	Male	Others	2020	37	Single	No formal education	Others	Rural	Yes	Yes	Yes	No	Yes	No	No	No	No	No
4089	HQE0678	Male	Others	2018	17	Single	No formal education	Private	Urban	Yes	Yes	Yes	No	No	No	No	No	No	No
4090	HQE0679	Male	Others	2019	25	Single	Tertiary	Private	Rural	Yes	Yes	Yes	No	No	No	No	No	No	No
4091	HQE0680	Male	Others	2019	20	Single	No formal education	Unemployed	Rural	Yes	Yes	Yes	No	Yes	No	No	No	No	No
4092	HQE0681	Male	Chinese	2019	47	Married	Tertiary	Self-employed	Rural	No	Yes	No	No	No	No	No	No	No	No
4093	HQE0682	Female	Others	2018	19	Single	Secondary	Private	Rural	Yes	Yes	Yes	No	Yes	No	No	No	No	No
4094	HQE0683	Male	Others	2018	32	Married	Secondary	Self-employed	Rural	Yes	Yes	Yes	No	Yes	No	No	No	No	No
4095	HQE0684	Male	Others	2018	25	Single	No formal education	Unemployed	Rural	Yes	Yes	No	No	Yes	No	No	No	No	No
4096	HQE0686	Male	Others	2019	25	Single	No formal education	Unemployed	Rural	Yes	Yes	Yes	No	Yes	Yes	No	No	No	No
4097	HQE0687	Male	Others	2018	39	Married	No formal education	Unemployed	Rural	Yes	Yes	Yes	No	No	No	No	No	No	No
4098	HQE0689	Female	Others	2018	36	Married	Tertiary	Private	Rural	Yes	No	Yes	No	No	No	No	No	No	No
4099	HQE0690	Male	Others	2019	46	Married	No formal education	Self-employed	Rural	Yes	Yes	Yes	No	No	No	No	No	No	No
4100	HQE0693	Male	Others	2020	24	Single	Primary	Private	Rural	Yes	Yes	No	No	Yes	No	No	No	No	No

No	Patient ID	Gender	Ethnic group	Year of diagnosis	Age	Marital status	Education level	Occupation	Place of residence	History of psy illness	Tobacco	Alcohol	Cannabis	ATS	Inhalant	Sedative	Hallucinogens	Opioids	Kratom
4101	HQE0694	Male	Others	2018	42	Married	Tertiary	Self-employed	Urban	Yes	Yes	Yes	No	No	No	No	No	No	No
4102	HQE0696	Male	Others	2019	47	Single	Primary	Unemployed	Urban	Yes	Yes	Yes	No	No	No	No	No	No	No
4103	HRPB0001	Male	Indian	2018	33	Married	No formal education	Private	Rural	Yes	Yes	Yes	No	No	No	No	No	No	No
4104	HRPB0002	Male	Others	2018	46	Single	Secondary	Unemployed	Rural	Yes	No	Yes	Yes	No	No	No	No	Yes	No
4105	HRPB0004	Male	Indian	2018	50	Married	No formal education	Others	Urban	Yes	No	Yes	No	No	No	No	No	No	No
4106	HRPB0005	Male	Indian	2018	43	Married	No formal education	Government	Rural	Yes	No	Yes	No	No	No	No	No	Yes	No
4107	HRPB0006	Male	Indian	2019	59	Others	Primary	Private	Rural	Yes	Yes	Yes	No	No	No	No	No	No	No
4108	HRPB0007	Male	Indian	2018	51	Married	Secondary	Self-employed	Rural	Yes	Yes	Yes	No	No	No	No	No	No	No
4109	HRPB0011	Male	Indian	2018	40	Single	No formal education	Others	Urban	Yes	No	Yes	No	Yes	No	No	No	No	No
4110	HRPB0012	Male	Indian	2020	35	Married	No formal education	Others	Urban	Yes	No	Yes	No	No	No	No	No	No	No
4111	HRPB0013	Male	Malay	2020	32	Others	No formal education	Unemployed	Urban	Yes	No	No	Yes	Yes	No	No	No	No	No
4112	HRPB0014	Male	Malay	2020	21	Others	No formal education	Others	Urban	Yes	Yes	No	Yes	No	No	No	No	No	No
4113	HRPB0015	Male	Malay	2020	29	Single	No formal education	Others	Rural	Yes	No	No	No	Yes	No	No	No	Yes	No
4114	HRPB0016	Male	Indian	2021	59	Married	No formal education	Private	Urban	Yes	No	Yes	No	No	No	No	No	No	No
4115	HRPB0017	Male	Chinese	2020	42	Married	No formal education	Others	Urban	Yes	No	No	No	Yes	No	No	No	No	No
4116	HRPB0018	Male	Indian	2021	54	Others	No formal education	Others	Urban	Yes	Yes	Yes	No	No	No	No	No	No	No
4117	HRPB0019	Male	Malay	2021	37	Others	No formal education	Unemployed	Urban	Yes	No	No	Yes	Yes	No	Yes	No	No	No
4118	HRPB0020	Male	Indian	2020	31	Single	No formal education	Private	Urban	Yes	No	No	No	No	No	No	No	Yes	No
4119	HRPB0021	Male	Malay	2020	31	Others	No formal education	Others	Urban	Yes	No	No	No	Yes	No	No	No	Yes	No
4120	HRPB0022	Male	Indian	2020	42	Others	No formal education	Self-employed	Urban	Yes	No	Yes	No	No	No	No	No	No	No
4121	HRPB0023	Male	Chinese	2021	55	Married	No formal education	Private	Urban	Yes	No	Yes	No	No	No	No	No	No	No
4122	HRPB0024	Male	Malay	2019	32	Others	Tertiary	Unemployed	Urban	Yes	Yes	No	No	No	No	No	No	No	No

No	Patient ID	Gender	Ethnic group	Year of diagnosis	Age	Marital status	Education level	Occupation	Place of residence	History of psy illness	Tobacco	Alcohol	Cannabis	ATS	Inhalant	Sedative	Hallucinogens	Opioids	Kratom
4123	HRPB0025	Male	Malay	2019	32	Single	Secondary	Unemployed	Urban	Yes	No	No	No	Yes	No	No	No	Yes	No
4124	HRPB0026	Male	Indian	2018	56	Married	Secondary	Private	Urban	Yes	Yes	Yes	No	No	No	No	No	No	No
4125	HRPB0027	Male	Chinese	2019	43	Others	No formal education	Unemployed	Urban	Yes	Yes	Yes	No	No	No	No	No	No	No
4126	HRPB0028	Male	Indian	2020	39	Others	No formal education	Others	Urban	Yes	No	No	No	Yes	No	No	No	No	No
4127	HRPB0030	Male	Indian	2020	46	Married	No formal education	Others	Urban	Yes	No	Yes	No	No	No	No	No	No	No
4128	HRPB0031	Male	Indian	2019	47	Married	Primary	Self-employed	Urban	Yes	Yes	Yes	No	No	No	No	No	No	No
4129	HRPB0032	Male	Others	2021	44	Others	No formal education	Private	Rural	Yes	No	Yes	No	No	No	No	No	No	No
4130	HRPB0033	Male	Indian	2019	36	Others	Secondary	Self-employed	Urban	Yes	Yes	Yes	No	No	No	No	No	No	No
4131	HRPB0035	Male	Indian	2018	39	Married	Secondary	Private	Urban	Yes	Yes	Yes	No	No	No	No	No	No	No
4132	HRPB0037	Male	Malay	2020	58	Others	Secondary	Others	Urban	Yes	Yes	No	No	Yes	No	Yes	No	Yes	No
4133	HRPB0038	Male	Chinese	2020	35	Others	No formal education	Others	Urban	Yes	No	No	No	Yes	No	No	No	No	No
4134	HRPB0039	Male	Malay	2019	31	Married	No formal education	Others	Rural	Yes	No	No	No	No	No	No	No	No	Yes
4135	HRPB0041	Male	Malay	2020	26	Single	No formal education	Private	Urban	Yes	No	No	Yes	Yes	No	No	No	Yes	No
4136	HRPB0042	Male	Malay	2019	29	Others	No formal education	Self-employed	Urban	Yes	No	No	Yes	No	No	No	No	Yes	Yes
4137	HRPB0043	Male	Chinese	2018	42	Others	No formal education	Others	Urban	Yes	No	No	No	Yes	No	No	No	No	No
4138	HRPB0044	Male	Malay	2018	33	Others	No formal education	Private	Urban	Yes	No	No	No	Yes	No	No	No	No	No
4139	HRPB0045	Male	Chinese	2019	48	Single	Secondary	Private	Urban	Yes	No	Yes	No	No	No	No	No	No	No
4140	HRPB0046	Male	Indian	2019	46	Married	No formal education	Others	Rural	Yes	No	Yes	No	No	No	No	No	No	No
4141	HRPB0047	Male	Chinese	2019	36	Others	No formal education	Private	Urban	Yes	No	No	No	No	No	No	No	No	No
4142	HRPB0048	Male	Indian	2018	50	Married	Secondary	Private	Urban	Yes	Yes	Yes	No	No	No	No	No	No	No
4143	HRPB0051	Male	Chinese	2019	25	Single	Secondary	Others	Urban	Yes	Yes	No	No	Yes	No	No	No	No	No

No	Patient ID	Gender	Ethnic group	Year of diagnosis	Age	Marital status	Education level	Occupation	Place of residence	History of psy illness	Tobacco	Alcohol	Cannabis	ATS	Inhalant	Sedative	Hallucinogens	Opioids	Kratom
4144	HRPB0052	Male	Indian	2019	50	Others	No formal education	Unemployed	Urban	Yes	Yes	Yes	No	No	No	No	No	No	No
4145	HRPB0053	Male	Malay	2019	25	Others	No formal education	Private	Urban	Yes	No	No	Yes	Yes	No	No	No	No	Yes
4146	HRPB0054	Male	Indian	2018	61	Married	No formal education	Others	Urban	Yes	No	Yes	No	No	No	No	No	No	No
4147	HRPB0055	Male	Malay	2020	23	Single	Secondary	Private	Rural	Yes	No	No	Yes	Yes	No	No	No	No	Yes
4148	HRPB0059	Female	Malay	2019	26	Married	Secondary	Unemployed	Urban	Yes	Yes	No	No	Yes	No	No	No	No	No
4149	HRPB0060	Male	Chinese	2018	48	Married	No formal education	Private	Urban	Yes	No	Yes	No	No	No	No	No	No	No
4150	HRPB0061	Female	Indian	2019	42	Married	Secondary	Unemployed	Urban	Yes	Yes	No	No	No	No	No	No	No	No
4151	HRPB0062	Male	Indian	2019	34	Single	No formal education	Private	Urban	Yes	No	Yes	No	No	No	No	No	No	No
4152	HRPB0063	Male	Indian	2020	16	Others	No formal education	Others	Urban	Yes	No	No	No	Yes	No	No	No	No	No
4153	HRPB0064	Male	Chinese	2020	25	Others	Tertiary	Others	Urban	Yes	No	No	No	Yes	No	No	No	No	No
4154	HRPB0065	Male	Indian	2019	39	Married	No formal education	Others	Urban	Yes	No	Yes	No	No	No	No	No	No	No
4155	HRPB0068	Male	Malay	2019	27	Single	Secondary	Unemployed	Urban	Yes	Yes	No	No	Yes	No	No	No	No	No
4156	HRPB0070	Male	Chinese	2018	42	Single	No formal education	Self-employed	Urban	Yes	No	Yes	No	No	No	No	No	No	No
4157	HRPB0071	Male	Indian	2018	59	Married	Primary	Private	Urban	Yes	No	Yes	No	No	No	No	No	No	No
4158	HRPB0073	Male	Indian	2019	56	Others	No formal education	Unemployed	Urban	Yes	Yes	Yes	No	No	No	No	No	No	No
4159	HRPB0074	Male	Chinese	2019	49	Others	No formal education	Unemployed	Urban	Yes	No	No	No	Yes	No	No	No	No	No
4160	HRPB0075	Male	Malay	2021	36	Married	Secondary	Self-employed	Urban	Yes	No	No	Yes	Yes	Yes	No	No	No	No
4161	HRPB0077	Female	Malay	2019	32	Single	No formal education	Private	Urban	Yes	No	No	No	No	No	Yes	No	No	No
4162	HRPB0079	Female	Malay	2019	29	Others	No formal education	Others	Urban	Yes	Yes	No	No	Yes	No	No	No	No	No
4163	HRPB0080	Female	Indian	2019	37	Others	No formal education	Private	Urban	Yes	No	Yes	No	No	No	No	No	No	No
4164	HRPB0083	Male	Indian	2020	55	Others	No formal education	Private	Urban	Yes	Yes	Yes	No	No	No	No	No	No	No

No	Patient ID	Gender	Ethnic group	Year of diagnosis	Age	Marital status	Education level	Occupation	Place of residence	History of psy illness	Tobacco	Alcohol	Cannabis	ATS	Inhalant	Sedative	Hallucinogens	Opioids	Kratom
4165	HRPB0085	Male	Malay	2018	21	Single	Secondary	Unemployed	Rural	Yes	No	No	No	Yes	No	No	No	No	No
4166	HRPB0086	Male	Chinese	2018	49	Single	Secondary	Private	Urban	Yes	No	Yes	No	No	No	No	No	No	No
4167	HRPB0092	Male	Malay	2018	31	Single	No formal education	Self-employed	Urban	Yes	No	No	No	Yes	No	Yes	No	No	No
4168	HRPB0093	Male	Chinese	2021	38	Others	No formal education	Private	Urban	Yes	No	No	No	Yes	No	No	No	No	No
4169	HRPB0094	Male	Malay	2019	22	Single	Secondary	Others	Urban	Yes	No	No	No	Yes	No	No	No	No	No
4170	HRPB0095	Male	Indian	2021	46	Married	Primary	Unemployed	Urban	Yes	Yes	Yes	No	No	No	No	No	No	No
4171	HRPB0096	Male	Chinese	2019	34	Others	No formal education	Private	Urban	Yes	No	No	No	Yes	No	Yes	No	No	No
4172	HRPB0097	Male	Malay	2019	20	Single	Secondary	Unemployed	Urban	Yes	Yes	No	No	Yes	No	No	No	No	No
4173	HRPB0098	Male	Malay	2018	30	Single	No formal education	Others	Rural	Yes	Yes	No	Yes	Yes	No	No	No	No	No
4174	HRPB0099	Male	Chinese	2020	29	Others	Tertiary	Private	Urban	Yes	No	No	No	Yes	No	No	No	No	No
4175	HRPB0104	Male	Others	2018	46	Married	No formal education	Others	Urban	Yes	Yes	Yes	No	No	No	No	No	No	No
4176	HRPB0106	Male	Malay	2018	54	Single	No formal education	Others	Urban	Yes	No	No	No	No	No	No	No	Yes	No
4177	HRPB0107	Male	Chinese	2018	44	Single	Secondary	Private	Urban	Yes	No	Yes	No	No	No	No	No	No	No
4178	HRPB0108	Male	Malay	2018	28	Single	No formal education	Others	Rural	Yes	No	No	Yes	No	No	No	No	No	No
4179	HRPB0109	Male	Malay	2018	28	Married	Tertiary	Private	Urban	Yes	No	Yes	No	No	No	No	No	No	No
4180	HRPB0110	Male	Indian	2018	44	Single	No formal education	Others	Rural	Yes	No	Yes	No	No	No	No	No	No	No
4181	HRPB0111	Male	Indian	2018	50	Single	No formal education	Others	Urban	Yes	Yes	Yes	Yes	Yes	No	Yes	No	No	No
4182	HRPB0112	Male	Malay	2018	31	Married	No formal education	Others	Urban	Yes	Yes	Yes	No	Yes	No	No	No	No	No
4183	HRPB0113	Male	Malay	2018	51	Single	Primary	Others	Rural	Yes	No	No	No	No	No	No	No	Yes	No
4184	HRPB0115	Male	Malay	2021	17	Single	Secondary	Unemployed	Urban	Yes	No	No	No	Yes	No	No	No	No	Yes
4185	HRPB0116	Male	Indian	2021	31	Married	No formal education	Private	Urban	Yes	No	Yes	No	Yes	No	No	No	No	No

No	Patient ID	Gender	Ethnic group	Year of diagnosis	Age	Marital status	Education level	Occupation	Place of residence	History of psy illness	Tobacco	Alcohol	Cannabis	ATS	Inhalant	Sedative	Hallucinogens	Opioids	Kratom
4186	HRPB0117	Male	Malay	2021	58	Others	No formal education	Others	Urban	Yes	No	No	No	Yes	No	No	No	No	No
4187	HRPB0118	Male	Indian	2021	34	Others	No formal education	Private	Urban	Yes	Yes	Yes	No	No	No	No	No	No	No
4188	HRPB0119	Male	Chinese	2021	64	Others	No formal education	Unemployed	Urban	Yes	No	No	No	No	No	Yes	No	No	No
4189	HRPB0120	Male	Indian	2021	54	Married	No formal education	Unemployed	Urban	Yes	Yes	Yes	No	No	No	No	No	No	No
4190	HRPB0121	Male	Indian	2020	31	Single	No formal education	Private	Urban	Yes	No	No	No	No	No	No	No	Yes	No
4191	HRPB0122	Male	Malay	2020	37	Others	No formal education	Others	Urban	Yes	Yes	No	No	Yes	No	No	No	No	No
4192	HRPB0123	Male	Indian	2020	24	Others	No formal education	Others	Urban	Yes	No	No	Yes	No	No	No	No	No	No
4193	HRPB0124	Male	Chinese	2020	78	Others	No formal education	Others	Rural	Yes	Yes	Yes	No	No	No	No	No	No	No
4194	HRPB0125	Male	Malay	2020	34	Single	No formal education	Unemployed	Urban	Yes	No	No	No	Yes	No	No	No	No	No
4195	HRPB0126	Male	Malay	2020	26	Single	Secondary	Private	Urban	Yes	No	No	No	No	No	No	No	No	Yes
4196	HRPB0127	Male	Indian	2020	47	Others	No formal education	Others	Urban	Yes	No	No	No	No	No	No	No	Yes	No
4197	HRPB0128	Male	Malay	2020	23	Single	Primary	Self-employed	Urban	Yes	No	No	No	Yes	No	No	No	Yes	No
4198	HRPB0129	Male	Malay	2019	32	Married	No formal education	Private	Rural	Yes	No	No	No	No	No	No	No	No	Yes
4199	HRPB0131	Male	Indian	2019	53	Married	No formal education	Unemployed	Urban	Yes	Yes	No	Yes	Yes	No	No	No	No	No
4200	HRPB0132	Male	Indian	2019	33	Married	Secondary	Private	Urban	Yes	No	Yes	No	No	No	No	No	No	No
4201	HRPB0133	Male	Indian	2018	27	Single	Tertiary	Others	Urban	Yes	No	No	No	Yes	Yes	No	No	No	No
4202	HRPB0134	Male	Indian	2018	61	Single	No formal education	Others	Urban	Yes	No	Yes	No	No	No	No	No	No	No
4203	HRPB0136	Male	Malay	2020	35	Single	Secondary	Private	Urban	Yes	Yes	No	No	Yes	No	No	No	No	No
4204	HRPB0137	Male	Indian	2020	34	Others	Primary	Others	Rural	Yes	No	Yes	Yes	No	No	No	No	No	No
4205	HRPB0138	Male	Indian	2020	37	Married	No formal education	Others	Urban	Yes	Yes	Yes	No	No	No	No	No	No	No
4206	HRPB0139	Male	Malay	2020	36	Married	No formal education	Others	Urban	Yes	Yes	No	Yes	No	No	No	No	No	No

No	Patient ID	Gender	Ethnic group	Year of diagnosis	Age	Marital status	Education level	Occupation	Place of residence	History of psy illness	Tobacco	Alcohol	Cannabis	ATS	Inhalant	Sedative	Hallucinogens	Opioids	Kratom
4207	HRPB0141	Female	Malay	2019	33	Married	Secondary	Private	Rural	Yes	No	No	No	Yes	No	No	No	Yes	No
4208	HRPB0142	Male	Malay	2019	36	Married	Primary	Self-employed	Urban	Yes	No	No	No	No	No	No	No	No	Yes
4209	HRPB0143	Male	Malay	2019	18	Others	No formal education	Others	Urban	Yes	No	No	No	Yes	Yes	No	No	No	No
4210	HRPB0144	Female	Malay	2019	33	Married	No formal education	Others	Rural	Yes	No	No	No	Yes	No	No	No	Yes	No
4211	HRPB0145	Male	Chinese	2020	47	Others	Secondary	Private	Urban	Yes	Yes	Yes	No	No	No	No	No	No	No
4212	HRPB0146	Female	Malay	2019	28	Others	Tertiary	Private	Urban	Yes	Yes	Yes	No	Yes	No	No	No	No	No
4213	HRPB0147	Male	Malay	2021	29	Others	Tertiary	Private	Urban	Yes	No	No	No	Yes	No	No	No	No	No
4214	HRPB0148	Male	Indian	2020	27	Single	Secondary	Unemployed	Urban	Yes	No	Yes	No	Yes	No	No	No	No	No
4215	HRPB0149	Male	Indian	2021	64	Others	No formal education	Others	Urban	Yes	No	No	No	No	No	No	No	Yes	No
4216	HRPB0150	Male	Indian	2021	33	Others	No formal education	Others	Urban	Yes	No	No	No	Yes	No	No	No	No	No
4217	HRPB0151	Male	Indian	2018	49	Others	No formal education	Others	Urban	Yes	No	No	Yes	No	No	No	No	No	No
4218	HRPB0152	Male	Indian	2020	28	Single	Tertiary	Unemployed	Urban	Yes	Yes	No	Yes	No	No	No	No	No	No
4219	HRPB0153	Male	Indian	2020	67	Married	No formal education	Others	Urban	Yes	No	No	No	No	No	Yes	No	Yes	No
4220	HRPB0154	Male	Others	2020	17	Single	Secondary	Unemployed	Urban	Yes	No	No	No	Yes	No	No	No	No	No
4221	HRPB0156	Male	Chinese	2021	51	Others	No formal education	Private	Rural	Yes	No	Yes	No	No	No	No	No	No	No
4222	HRPB0157	Male	Malay	2019	35	Single	Secondary	Private	Urban	Yes	No	No	No	Yes	No	Yes	No	Yes	No
4223	HRPB0158	Male	Indian	2018	40	Married	Primary	Private	Urban	Yes	Yes	No	No	Yes	No	No	No	Yes	No
4224	HRPB0159	Male	Indian	2021	49	Married	No formal education	Private	Urban	Yes	Yes	No	No	No	No	No	No	Yes	No
4225	HRPB0161	Female	Chinese	2019	68	Married	Primary	Private	Rural	Yes	No	No	No	No	No	Yes	No	No	No
4226	HRPB0162	Female	Malay	2018	23	Others	No formal education	Others	Urban	Yes	No	No	No	Yes	No	No	No	No	No
4227	HRPB0163	Female	Malay	2020	18	Others	No formal education	Others	Urban	Yes	No	No	No	Yes	No	No	No	No	No

No	Patient ID	Gender	Ethnic group	Year of diagnosis	Age	Marital status	Education level	Occupation	Place of residence	History of psy illness	Tobacco	Alcohol	Cannabis	ATS	Inhalant	Sedative	Hallucinogens	Opioids	Kratom
4228	HRPB0164	Female	Malay	2019	28	Others	No formal education	Unemployed	Urban	Yes	No	No	No	Yes	No	No	No	No	No
4229	HRPB0167	Male	Malay	2021	54	Others	No formal education	Others	Urban	Yes	No	No	No	No	No	No	No	Yes	No
4230	HRPB0168	Male	Malay	2019	34	Others	No formal education	Others	Urban	Yes	No	No	No	Yes	No	No	No	No	No
4231	HRPB0169	Male	Malay	2018	36	Married	No formal education	Others	Urban	Yes	No	No	No	Yes	No	No	No	No	Yes
4232	HRPB0170	Male	Malay	2019	48	Others	Secondary	Others	Urban	Yes	No	No	Yes	No	No	No	Yes	No	No
4233	HRPB0171	Male	Malay	2020	70	Others	No formal education	Private	Urban	Yes	No	No	No	No	No	No	No	Yes	No
4234	HRPB0173	Male	Malay	2019	36	Others	Tertiary	Private	Rural	Yes	No	No	Yes	Yes	No	No	No	Yes	No
4235	HRPB0174	Male	Malay	2021	22	Others	No formal education	Others	Rural	Yes	No	No	No	Yes	No	No	No	No	Yes
4236	HRPB0177	Male	Malay	2020	27	Married	No formal education	Private	Urban	Yes	No	No	Yes	No	No	No	No	Yes	Yes
4237	HRPB0178	Male	Malay	2018	31	Single	Secondary	Unemployed	Rural	Yes	Yes	No	No	Yes	No	No	No	No	No
4238	HRPB0179	Female	Malay	2019	20	Others	Primary	Unemployed	Rural	Yes	No	No	No	Yes	No	No	No	No	No
4239	HRPB0184	Male	Malay	2019	59	Married	No formal education	Others	Rural	Yes	Yes	Yes	No	No	No	No	No	No	Yes
4240	HRPB0185	Male	Malay	2020	29	Single	Secondary	Self-employed	Urban	Yes	Yes	No	No	No	No	No	No	No	No
4241	HRPB0187	Male	Malay	2021	25	Single	No formal education	Unemployed	Rural	Yes	No	No	No	Yes	No	No	No	No	No
4242	HRPB0189	Male	Malay	2020	27	Single	No formal education	Others	Urban	Yes	No	No	No	Yes	No	No	No	No	No
4243	HRPB0191	Male	Malay	2018	60	Married	Tertiary	Government	Urban	Yes	No	No	No	No	No	Yes	No	No	No
4244	HRPB0192	Male	Malay	2019	69	Married	No formal education	Others	Urban	Yes	Yes	No	No	No	No	No	No	No	No
4245	HRPB0194	Male	Malay	2020	39	Others	No formal education	Others	Urban	Yes	No	No	No	No	No	No	No	Yes	No
4246	HRPB0195	Male	Malay	2019	38	Others	No formal education	Others	Urban	Yes	No	No	No	No	No	No	No	No	Yes
4247	HRPB0197	Female	Indian	2019	46	Married	Secondary	Private	Urban	Yes	Yes	No	No	No	No	No	No	No	No
4248	HRPB0200	Male	Malay	2019	27	Single	No formal education	Unemployed	Urban	Yes	No	No	Yes	No	No	No	No	Yes	Yes

No	Patient ID	Gender	Ethnic group	Year of diagnosis	Age	Marital status	Education level	Occupation	Place of residence	History of psy illness	Tobacco	Alcohol	Cannabis	ATS	Inhalant	Sedative	Hallucinogens	Opioids	Kratom
4249	HRPB0202	Male	Malay	2019	44	Married	No formal education	Private	Urban	Yes	Yes	No	No	No	No	No	No	No	No
4250	HRPB0203	Male	Others	2018	24	Single	Secondary	Private	Urban	Yes	Yes	No	No	Yes	No	No	No	No	No
4251	HRPB0204	Male	Chinese	2018	22	Others	No formal education	Others	Urban	Yes	No	Yes	No	No	No	No	No	No	No
4252	HRPB0207	Male	Chinese	2019	57	Others	No formal education	Private	Urban	Yes	No	No	No	No	No	Yes	No	No	No
4253	HRPB0209	Male	Indian	2020	50	Others	No formal education	Others	Urban	Yes	Yes	Yes	No	No	No	No	No	No	No
4254	HRPB0210	Male	Indian	2020	51	Others	No formal education	Unemployed	Urban	Yes	Yes	Yes	No	No	No	No	No	No	No
4255	HRPB0211	Male	Indian	2020	32	Married	No formal education	Others	Rural	Yes	No	Yes	No	No	No	No	No	No	No
4256	HRPB0212	Male	Indian	2020	62	Married	No formal education	Others	Urban	Yes	Yes	Yes	No	No	No	No	No	No	No
4257	HRPB0213	Male	Indian	2021	47	Married	No formal education	Unemployed	Urban	Yes	Yes	Yes	No	No	No	No	No	No	No
4258	HRPB0214	Male	Indian	2021	39	Married	Primary	Private	Urban	Yes	Yes	Yes	No	No	No	No	No	No	No
4259	HRPB0215	Male	Chinese	2020	42	Others	No formal education	Others	Urban	Yes	Yes	Yes	No	No	No	No	No	No	No
4260	HRPB0216	Male	Chinese	2018	46	Others	No formal education	Others	Urban	Yes	No	No	No	Yes	No	No	No	No	No
4261	HRPB0217	Male	Chinese	2019	33	Married	No formal education	Others	Urban	Yes	Yes	Yes	No	No	No	No	No	No	No
4262	HRPB0218	Male	Chinese	2019	33	Single	No formal education	Unemployed	Urban	Yes	Yes	Yes	No	No	No	No	No	No	No
4263	HRPB0219	Male	Chinese	2020	42	Others	No formal education	Others	Urban	Yes	No	No	No	Yes	No	No	No	No	No
4264	HRPB0220	Male	Chinese	2019	45	Others	No formal education	Others	Urban	Yes	No	No	Yes	Yes	No	No	No	No	No
4265	HRPB0221	Male	Indian	2021	29	Married	No formal education	Others	Urban	Yes	No	Yes	No	No	No	No	No	No	No
4266	HRPB0223	Male	Indian	2020	60	Married	Primary	Private	Urban	Yes	No	Yes	No	No	No	No	No	No	No
4267	HRPB0224	Male	Indian	2018	36	Married	No formal education	Others	Urban	Yes	No	Yes	No	Yes	No	No	No	No	No
4268	HRPB0225	Male	Indian	2021	46	Single	No formal education	Unemployed	Urban	Yes	No	Yes	No	No	No	No	No	No	No
4269	HRPB0226	Male	Indian	2019	30	Single	No formal education	Others	Urban	Yes	Yes	Yes	No	No	No	No	No	No	No

No	Patient ID	Gender	Ethnic group	Year of diagnosis	Age	Marital status	Education level	Occupation	Place of residence	History of psy illness	Tobacco	Alcohol	Cannabis	ATS	Inhalant	Sedative	Hallucinogens	Opioids	Kratom
4270	HRPB0227	Male	Indian	2021	43	Single	Tertiary	Private	Urban	Yes	Yes	Yes	No	No	No	No	No	No	Yes
4271	HRPB0228	Male	Indian	2021	46	Married	No formal education	Unemployed	Urban	Yes	No	No	No	Yes	No	No	No	Yes	No
4272	HRPB0229	Male	Indian	2019	32	Others	No formal education	Others	Urban	Yes	No	No	No	Yes	No	No	No	No	No
4273	HRPB0230	Male	Indian	2018	52	Others	No formal education	Others	Urban	Yes	No	No	No	Yes	No	No	No	No	No
4274	HRPB0231	Male	Indian	2020	33	Married	No formal education	Private	Urban	Yes	Yes	No	No	No	No	No	No	No	No
4275	HRPB0232	Male	Indian	2019	52	Others	Secondary	Unemployed	Rural	Yes	Yes	Yes	No	No	No	No	No	No	No
4276	HRPB0233	Male	Indian	2019	37	Others	No formal education	Others	Urban	Yes	No	Yes	No	No	No	No	No	No	No
4277	HRPB0234	Male	Indian	2018	61	Others	No formal education	Others	Urban	Yes	Yes	Yes	No	No	No	No	No	No	No
4278	HRPB0235	Male	Indian	2020	63	Married	No formal education	Private	Urban	Yes	No	Yes	No	No	No	No	No	No	No
4279	HRPB0237	Male	Indian	2018	31	Single	Secondary	Self-employed	Urban	Yes	Yes	Yes	No	Yes	No	No	No	No	No
4280	HRPB0238	Male	Malay	2018	33	Others	Secondary	Private	Urban	Yes	Yes	No	No	Yes	No	No	No	No	No
4281	HRPB0239	Male	Malay	2018	24	Single	Secondary	Private	Urban	Yes	Yes	No	No	Yes	No	No	No	No	No
4282	HRPB0240	Male	Malay	2021	44	Others	Secondary	Private	Urban	Yes	Yes	No	No	No	No	No	No	No	No
4283	HRPB0241	Female	Malay	2021	36	Others	No formal education	Others	Urban	Yes	Yes	No	No	Yes	No	No	No	No	No
4284	HRPB0242	Male	Malay	2018	36	Married	Primary	Private	Urban	Yes	Yes	No	No	No	No	No	No	Yes	No
4285	HRPB0243	Male	Malay	2020	50	Married	No formal education	Others	Urban	Yes	No	No	No	Yes	No	No	No	No	No
4286	HRPB0244	Male	Malay	2020	35	Single	No formal education	Self-employed	Urban	Yes	No	No	Yes	Yes	No	No	No	No	Yes
4287	HRPB0246	Male	Indian	2019	59	Married	No formal education	Others	Urban	Yes	Yes	No	No	No	No	No	No	No	No
4288	HRPB0248	Male	Chinese	2018	66	Single	Secondary	Private	Urban	Yes	No	Yes	No	No	No	No	No	No	No
4289	HRPB0250	Male	Malay	2018	21	Single	Tertiary	Unemployed	Urban	Yes	Yes	No	Yes	No	No	No	No	No	No
4290	HRPB0251	Male	Others	2018	35	Others	No formal education	Others	Urban	Yes	No	Yes	No	No	No	No	No	No	No

No	Patient ID	Gender	Ethnic group	Year of diagnosis	Age	Marital status	Education level	Occupation	Place of residence	History of psy illness	Tobacco	Alcohol	Cannabis	ATS	Inhalant	Sedative	Hallucinogens	Opioids	Kratom
4291	HRPB0252	Male	Indian	2020	41	Married	Secondary	Private	Urban	Yes	Yes	Yes	No	No	No	No	No	No	No
4292	HRPB0253	Male	Indian	2019	27	Others	No formal education	Others	Urban	Yes	No	Yes	No	Yes	No	No	No	No	No
4293	HRPB0254	Male	Indian	2019	22	Others	No formal education	Others	Urban	Yes	No	Yes	No	No	No	No	No	No	No
4294	HRPB0255	Male	Indian	2021	41	Married	Secondary	Unemployed	Urban	Yes	Yes	Yes	No	Yes	No	No	No	Yes	No
4295	HRPB0256	Male	Indian	2018	36	Others	No formal education	Others	Urban	Yes	No	Yes	Yes	No	No	No	No	Yes	No
4296	HRPB0257	Male	Chinese	2019	55	Married	Primary	Unemployed	Urban	Yes	No	No	No	Yes	No	No	No	No	No
4297	HRPB0258	Male	Chinese	2021	39	Single	No formal education	Others	Urban	Yes	Yes	Yes	No	Yes	No	No	No	No	No
4298	HRPB0260	Male	Chinese	2021	66	Married	No formal education	Others	Urban	Yes	No	Yes	No	No	No	No	No	No	No
4299	HRPB0261	Male	Chinese	2020	56	Married	No formal education	Others	Urban	Yes	Yes	No	No	No	No	No	No	No	No
4300	HRPB0262	Female	Chinese	2018	26	Others	No formal education	Others	Urban	Yes	Yes	Yes	No	No	No	No	No	No	No
4301	HRPB0263	Female	Chinese	2018	37	Married	No formal education	Private	Urban	Yes	No	Yes	No	No	Yes	No	No	No	No
4302	HRPB0264	Male	Chinese	2018	39	Others	No formal education	Others	Urban	Yes	No	No	No	Yes	No	Yes	No	No	No
4303	HRPB0265	Male	Chinese	2021	41	Married	Secondary	Self-employed	Urban	Yes	Yes	No	No	No	No	No	No	No	No
4304	HRPB0266	Male	Malay	2021	32	Single	No formal education	Unemployed	Rural	Yes	No	No	No	Yes	No	No	No	Yes	No
4305	HRPB0267	Male	Malay	2021	36	Single	No formal education	Private	Urban	Yes	No	No	No	No	Yes	No	No	No	No
4306	HRPB0268	Male	Malay	2019	53	Married	No formal education	Private	Urban	Yes	Yes	No	No	No	No	No	No	No	No
4307	HRPB0269	Male	Malay	2021	35	Single	No formal education	Unemployed	Urban	Yes	No	No	Yes	No	No	No	No	No	No
4308	HRPB0270	Male	Malay	2020	31	Single	No formal education	Private	Urban	No	Yes	Yes	No	Yes	No	No	No	No	Yes
4309	HRPB0271	Female	Indian	2019	53	Married	Tertiary	Government	Urban	Yes	No	Yes	No	No	No	No	No	No	No
4310	HRPB0272	Male	Indian	2019	33	Single	No formal education	Self-employed	Urban	Yes	No	No	No	No	No	No	No	Yes	No
4311	HRPB0273	Female	Indian	2018	75	Married	No formal education	Others	Urban	Yes	No	No	No	No	No	Yes	No	No	No

No	Patient ID	Gender	Ethnic group	Year of diagnosis	Age	Marital status	Education level	Occupation	Place of residence	History of psy illness	Tobacco	Alcohol	Cannabis	ATS	Inhalant	Sedative	Hallucinogens	Opioids	Kratom
4312	HRPB0274	Male	Indian	2018	62	Married	Primary	Others	Urban	Yes	No	Yes	No	No	No	No	No	No	No
4313	HRPB0275	Male	Chinese	2018	46	Others	Secondary	Others	Urban	Yes	Yes	No	No	Yes	No	No	No	No	No
4314	HRPB0276	Male	Chinese	2019	58	Married	No formal education	Unemployed	Urban	Yes	No	No	No	No	No	No	No	Yes	No
4315	HRPB0277	Male	Indian	2018	17	Single	Secondary	Others	Urban	Yes	No	No	No	Yes	No	No	No	No	No
4316	HRPB0278	Male	Chinese	2018	48	Others	Secondary	Unemployed	Urban	Yes	Yes	Yes	No	Yes	No	No	No	No	No
4317	HRPB0279	Male	Chinese	2018	59	Others	No formal education	Others	Urban	Yes	No	No	No	No	No	No	No	Yes	No
4318	HRPB0280	Male	Chinese	2020	33	Others	No formal education	Private	Urban	Yes	Yes	No	No	No	No	No	No	No	No
4319	HRPB0281	Male	Malay	2020	26	Others	No formal education	Others	Urban	Yes	No	No	No	Yes	No	Yes	No	Yes	No
4320	HRPB0282	Male	Malay	2021	28	Married	No formal education	Government	Urban	Yes	No	No	No	Yes	No	No	No	No	Yes
4321	HRPB0283	Male	Malay	2018	20	Others	No formal education	Others	Rural	Yes	No	No	No	Yes	No	No	No	No	No
4322	HRPB0284	Male	Malay	2018	33	Single	No formal education	Private	Urban	Yes	Yes	No	No	No	No	No	No	No	No
4323	HRPB0285	Male	Malay	2021	36	Single	No formal education	Others	Urban	Yes	Yes	Yes	No	No	No	No	No	No	No
4324	HRPB0291	Female	Malay	2020	38	Married	No formal education	Private	Urban	Yes	No	No	No	Yes	No	No	No	No	No
4325	HRPB0292	Male	Malay	2019	32	Married	Tertiary	Private	Urban	Yes	Yes	No	No	No	No	No	No	No	Yes
4326	HRPB0295	Male	Malay	2020	49	Married	No formal education	Others	Urban	Yes	Yes	No	No	Yes	No	No	No	No	No
4327	HRPB0296	Male	Malay	2020	38	Single	Secondary	Unemployed	Urban	Yes	Yes	No	No	No	No	No	No	No	No
4328	HRPB0297	Male	Malay	2020	28	Others	No formal education	Others	Urban	Yes	Yes	No	No	No	No	No	No	No	No
4329	HRPB0298	Female	Malay	2020	30	Others	No formal education	Private	Urban	Yes	Yes	Yes	No	No	No	No	No	No	No
4330	HRPB0300	Male	Chinese	2019	51	Others	Secondary	Private	Urban	Yes	Yes	No	Yes	No	No	No	No	No	No
4331	HRPB0302	Female	Chinese	2021	48	Married	No formal education	Others	Urban	Yes	No	Yes	No	No	No	No	No	No	No
4332	HRPB0303	Male	Indian	2020	55	Single	No formal education	Unemployed	Urban	Yes	No	No	No	Yes	No	No	No	Yes	No

No	Patient ID	Gender	Ethnic group	Year of diagnosis	Age	Marital status	Education level	Occupation	Place of residence	History of psy illness	Tobacco	Alcohol	Cannabis	ATS	Inhalant	Sedative	Hallucinogens	Opioids	Kratom
4333	HRPB0304	Male	Chinese	2019	35	Others	No formal education	Others	Urban	Yes	Yes	No	No	No	No	No	No	No	No
4334	HRPB0305	Male	Indian	2018	39	Married	Tertiary	Government	Urban	Yes	No	Yes	No	No	No	No	No	No	No
4335	HRPB0306	Male	Indian	2020	30	Single	Secondary	Unemployed	Urban	Yes	Yes	Yes	No	No	No	No	No	No	No
4336	HRPB0307	Male	Indian	2019	35	Others	Secondary	Private	Urban	Yes	Yes	No	No	Yes	No	No	No	No	No
4337	HRPB0308	Male	Indian	2018	46	Married	Secondary	Others	Urban	Yes	Yes	Yes	No	No	No	No	No	No	No
4338	HRPB0309	Male	Indian	2021	39	Others	No formal education	Unemployed	Urban	Yes	No	Yes	No	Yes	No	No	No	No	No
4339	HRPB0312	Male	Indian	2018	53	Married	No formal education	Private	Urban	Yes	Yes	Yes	No	No	No	No	No	No	No
4340	HRPB0313	Male	Malay	2019	62	Single	Secondary	Private	Urban	Yes	No	No	No	No	No	No	No	Yes	No
4341	HRPB0314	Male	Malay	2019	21	Others	No formal education	Others	Urban	Yes	Yes	No	No	No	No	No	No	No	No
4342	HRPB0315	Male	Malay	2020	33	Others	No formal education	Private	Urban	Yes	Yes	No	No	No	No	No	No	No	No
4343	HRPB0316	Male	Malay	2021	53	Married	No formal education	Unemployed	Rural	Yes	No	No	Yes	Yes	No	No	No	Yes	No
4344	HRPB0318	Male	Malay	2019	56	Married	No formal education	Self-employed	Urban	Yes	Yes	Yes	No	No	No	No	No	No	No
4345	HRPB0319	Male	Malay	2019	30	Others	No formal education	Unemployed	Urban	Yes	No	No	No	Yes	No	No	No	No	No
4346	HRPB0320	Male	Malay	2020	17	Single	No formal education	Others	Urban	Yes	Yes	No	No	No	No	No	No	No	No
4347	HRPB0321	Male	Malay	2019	33	Married	No formal education	Others	Urban	Yes	Yes	No	No	Yes	No	No	No	No	No
4348	HRPB0322	Male	Chinese	2021	63	Others	Secondary	Unemployed	Urban	Yes	No	Yes	No	No	No	No	No	No	No
4349	HRPB0323	Male	Chinese	2019	49	Single	No formal education	Private	Urban	Yes	Yes	No	Yes	No	No	No	No	No	No
4350	HRPB0324	Male	Others	2021	48	Married	No formal education	Private	Rural	Yes	No	Yes	No	No	No	No	No	No	No
4351	HRPB0325	Male	Malay	2021	34	Others	Secondary	Unemployed	Urban	Yes	No	No	No	Yes	No	No	No	No	No
4352	HRPB0326	Male	Malay	2021	70	Married	Secondary	Self-employed	Urban	Yes	Yes	No	No	No	No	No	No	No	No
4353	HRPB0327	Male	Malay	2019	47	Married	Secondary	Private	Rural	Yes	Yes	No	No	No	No	No	No	Yes	No

No	Patient ID	Gender	Ethnic group	Year of diagnosis	Age	Marital status	Education level	Occupation	Place of residence	History of psy illness	Tobacco	Alcohol	Cannabis	ATS	Inhalant	Sedative	Hallucinogens	Opioids	Kratom
4354	HRPB0328	Male	Chinese	2019	49	Single	No formal education	Unemployed	Urban	Yes	Yes	No	No	No	No	No	No	No	No
4355	HRPB0329	Male	Chinese	2019	86	Others	No formal education	Others	Urban	Yes	Yes	No	No	No	No	Yes	No	No	No
4356	HRPB0330	Male	Chinese	2020	38	Married	No formal education	Self-employed	Urban	Yes	No	No	No	Yes	No	No	No	Yes	No
4357	HRPB0331	Male	Chinese	2021	41	Others	No formal education	Others	Urban	Yes	No	No	Yes	Yes	No	No	No	No	No
4358	HRPB0332	Male	Chinese	2019	57	Married	Secondary	Others	Urban	Yes	No	Yes	No	No	No	No	No	No	No
4359	HRPB0333	Male	Chinese	2018	35	Others	No formal education	Others	Urban	Yes	No	Yes	No	No	No	No	No	No	No
4360	HRPB0335	Male	Malay	2020	36	Married	No formal education	Others	Urban	Yes	No	No	Yes	Yes	No	Yes	No	No	No
4361	HRPB0336	Male	Malay	2021	36	Single	No formal education	Unemployed	Urban	Yes	No	No	Yes	No	No	No	No	No	No
4362	HRPB0337	Male	Malay	2019	29	Others	Secondary	Self-employed	Urban	Yes	Yes	No	No	No	No	No	No	No	No
4363	HRPB0338	Male	Malay	2018	38	Others	Tertiary	Government	Urban	Yes	Yes	No	No	No	No	No	No	No	No
4364	HRPB0340	Male	Malay	2020	26	Others	No formal education	Others	Urban	Yes	No	No	Yes	No	No	No	No	No	No
4365	HRPB0342	Male	Malay	2019	27	Others	No formal education	Others	Urban	Yes	No	No	Yes	Yes	No	No	No	Yes	No
4366	HRPB0344	Female	Malay	2021	33	Others	Secondary	Unemployed	Urban	Yes	Yes	No	No	Yes	No	No	No	No	No
4367	HRPB0346	Male	Malay	2018	20	Single	Tertiary	Others	Urban	Yes	Yes	Yes	No	Yes	No	No	No	No	No
4368	HRPB0348	Male	Others	2021	36	Married	No formal education	Government	Urban	Yes	No	No	No	Yes	No	No	No	No	No
4369	HRPB0349	Male	Malay	2018	19	Others	No formal education	Others	Rural	Yes	Yes	No	No	No	No	No	No	No	No
4370	HRPB0358	Male	Chinese	2019	38	Married	Tertiary	Private	Urban	Yes	Yes	Yes	No	No	No	No	No	No	No
4371	HRPB0359	Male	Indian	2019	20	Single	Tertiary	Others	Urban	Yes	No	No	No	Yes	No	No	No	No	No
4372	HRPB0360	Male	Others	2020	22	Single	No formal education	Others	Urban	Yes	Yes	Yes	No	No	No	No	No	No	No
4373	HRPB0361	Male	Indian	2020	23	Others	No formal education	Others	Urban	Yes	No	Yes	Yes	No	No	No	No	No	No
4374	HRPB0362	Female	Malay	2020	25	Married	Secondary	Private	Urban	Yes	No	Yes	No	No	No	No	No	No	No

No	Patient ID	Gender	Ethnic group	Year of diagnosis	Age	Marital status	Education level	Occupation	Place of residence	History of psy illness	Tobacco	Alcohol	Cannabis	ATS	Inhalant	Sedative	Hallucinogens	Opioids	Kratom
4375	HRPB0363	Male	Malay	2018	40	Married	No formal education	Others	Urban	Yes	No	No	No	Yes	No	No	No	No	Yes
4376	HRPB0364	Male	Malay	2021	41	Married	No formal education	Private	Urban	Yes	Yes	No	No	No	No	No	No	No	No
4377	HRPB0365	Female	Chinese	2018	52	Others	No formal education	Others	Urban	Yes	Yes	Yes	No	No	No	No	No	No	No
4378	HRPB0366	Female	Chinese	2018	24	Married	Secondary	Self-employed	Urban	Yes	Yes	Yes	No	Yes	No	No	No	No	No
4379	HRPB0367	Male	Malay	2020	25	Others	No formal education	Others	Urban	Yes	No	No	No	Yes	No	No	No	No	No
4380	HRPB0368	Female	Chinese	2018	49	Married	No formal education	Others	Urban	Yes	No	No	No	No	No	Yes	No	No	No
4381	HRPB0369	Male	Chinese	2019	35	Others	No formal education	Others	Urban	Yes	Yes	No	No	Yes	No	No	No	No	No
4382	HRPB0370	Male	Indian	2018	20	Single	Secondary	Others	Urban	Yes	Yes	No	No	No	No	No	No	No	No
4383	HRPB0371	Male	Chinese	2018	35	Single	Secondary	Unemployed	Urban	Yes	No	No	No	No	No	Yes	No	No	No
4384	HRPB0372	Male	Indian	2018	19	Others	No formal education	Others	Urban	Yes	No	Yes	No	No	No	Yes	No	No	No
4385	HRPB0373	Male	Indian	2021	47	Others	Secondary	Private	Urban	Yes	Yes	Yes	No	No	No	No	No	No	No
4386	HRPB0374	Male	Indian	2019	23	Others	No formal education	Unemployed	Urban	Yes	Yes	Yes	No	No	No	No	No	No	No
4387	HRPB0375	Male	Indian	2019	22	Single	Secondary	Unemployed	Urban	Yes	No	Yes	Yes	No	No	No	No	No	No
4388	HRPB0378	Female	Chinese	2020	25	Single	No formal education	Self-employed	Urban	Yes	No	Yes	No	No	No	No	No	No	No
4389	HRPB0383	Male	Indian	2018	27	Single	Secondary	Private	Urban	Yes	No	No	No	No	No	No	No	Yes	No
4390	HRPB0386	Male	Indian	2018	30	Others	Primary	Private	Urban	Yes	No	Yes	Yes	Yes	No	No	No	No	No
4391	HRPB0399	Female	Chinese	2020	40	Married	No formal education	Others	Urban	Yes	No	Yes	No	No	No	Yes	No	No	No
4392	HRPB0400	Male	Chinese	2018	54	Others	No formal education	Private	Urban	Yes	No	No	No	No	No	Yes	No	No	No
4393	HRPB0406	Male	Indian	2020	56	Married	Secondary	Private	Urban	Yes	Yes	Yes	No	No	No	No	No	No	No
4394	HRPB0407	Male	Indian	2018	34	Married	Secondary	Private	Urban	No	Yes	Yes	Yes	No	No	No	No	Yes	No
4395	HRPB0409	Male	Indian	2021	39	Married	No formal education	Private	Urban	Yes	Yes	Yes	No	No	No	No	No	No	No

No	Patient ID	Gender	Ethnic group	Year of diagnosis	Age	Marital status	Education level	Occupation	Place of residence	History of psy illness	Tobacco	Alcohol	Cannabis	ATS	Inhalant	Sedative	Hallucinogens	Opioids	Kratom
4396	HRPB0410	Male	Chinese	2020	63	Others	No formal education	Others	Urban	Yes	No	No	No	No	No	Yes	No	No	No
4397	HRPB0411	Male	Indian	2021	32	Married	No formal education	Unemployed	Urban	Yes	No	No	No	Yes	No	No	No	No	No
4398	HRPB0412	Male	Indian	2020	29	Others	No formal education	Others	Urban	Yes	Yes	Yes	No	No	No	No	No	No	No
4399	HRPB0413	Male	Indian	2021	57	Married	Secondary	Private	Urban	Yes	No	Yes	Yes	No	No	No	No	No	No
4400	HRPB0414	Male	Indian	2018	26	Others	Secondary	Others	Urban	Yes	No	Yes	No	Yes	No	No	No	No	No
4401	HRPB0416	Male	Indian	2020	39	Others	No formal education	Others	Urban	Yes	No	Yes	No	No	No	No	No	No	No
4402	HRPB0417	Male	Indian	2021	41	Married	No formal education	Private	Urban	Yes	No	Yes	No	No	No	No	No	No	No
4403	HRPB0418	Male	Indian	2021	32	Single	Secondary	Self-employed	Urban	Yes	Yes	Yes	Yes	Yes	No	No	No	No	No
4404	HRPB0419	Male	Indian	2021	55	Married	No formal education	Others	Urban	Yes	Yes	No	No	No	No	No	No	No	No
4405	HRPB0420	Male	Indian	2018	36	Married	No formal education	Private	Urban	Yes	No	Yes	No	Yes	No	No	No	No	No
4406	HRPB0421	Male	Indian	2020	53	Married	No formal education	Others	Urban	Yes	Yes	Yes	No	No	No	No	No	No	No
4407	HRPB0422	Male	Indian	2021	29	Others	No formal education	Others	Urban	Yes	Yes	Yes	No	No	No	No	No	No	No
4408	HRPB0423	Male	Indian	2019	32	Married	Primary	Unemployed	Urban	Yes	No	No	No	Yes	No	No	No	No	No
4409	HRPB0424	Male	Indian	2018	65	Married	No formal education	Others	Urban	Yes	Yes	Yes	No	No	No	No	No	No	No
4410	HRPB0425	Male	Indian	2021	45	Married	Secondary	Unemployed	Urban	Yes	Yes	No	No	Yes	No	No	No	No	No
4411	HRPB0426	Male	Chinese	2020	47	Single	Secondary	Unemployed	Urban	Yes	No	Yes	No	No	No	No	No	No	No
4412	HRPB0427	Male	Indian	2020	35	Others	No formal education	Others	Urban	Yes	No	No	No	Yes	No	No	No	No	No
4413	HRPB0428	Male	Chinese	2021	64	Married	No formal education	Others	Urban	Yes	No	Yes	No	No	No	No	No	No	No
4414	HRPB0429	Male	Chinese	2021	50	Others	No formal education	Others	Urban	Yes	Yes	No	No	No	No	No	No	No	No
4415	HRPB0430	Female	Chinese	2018	64	Married	Primary	Unemployed	Urban	Yes	No	Yes	No	No	No	No	No	No	No
4416	HRPB0431	Male	Indian	2020	52	Others	No formal education	Others	Rural	Yes	Yes	No	No	No	No	No	No	No	No

No	Patient ID	Gender	Ethnic group	Year of diagnosis	Age	Marital status	Education level	Occupation	Place of residence	History of psy illness	Tobacco	Alcohol	Cannabis	ATS	Inhalant	Sedative	Hallucinogens	Opioids	Kratom
4417	HRPB0436	Female	Chinese	2019	61	Married	Primary	Private	Urban	Yes	Yes	No	No	No	No	No	No	No	No
4418	HRPB0437	Male	Indian	2019	28	Married	Secondary	Private	Urban	Yes	No	No	No	Yes	No	No	No	No	No
4419	HRPB0438	Male	Indian	2019	38	Single	Primary	Others	Urban	Yes	Yes	Yes	No	No	No	No	No	No	No
4420	HRPB0439	Male	Indian	2018	32	Others	No formal education	Others	Urban	Yes	Yes	No	Yes	Yes	No	No	No	No	No
4421	HRPB0440	Male	Chinese	2018	24	Single	Tertiary	Private	Urban	Yes	Yes	No	Yes	No	No	No	No	No	No
4422	HRPB0441	Male	Malay	2021	26	Single	No formal education	Private	Urban	Yes	Yes	Yes	No	No	No	No	No	No	No
4423	HRPB0442	Female	Indian	2020	19	Single	Secondary	Unemployed	Urban	Yes	No	Yes	No	Yes	No	No	No	No	No
4424	HRPB0443	Female	Indian	2018	27	Others	Tertiary	Unemployed	Urban	Yes	Yes	Yes	Yes	No	No	No	No	No	No
4425	HRPB0444	Male	Malay	2021	41	Single	Secondary	Private	Rural	Yes	Yes	No	No	No	No	No	No	Yes	No
4426	HRPB0445	Female	Malay	2019	33	Married	No formal education	Others	Urban	Yes	No	No	No	No	No	No	No	Yes	No
4427	HRPB0446	Female	Malay	2019	23	Others	No formal education	Others	Rural	Yes	Yes	No	No	No	No	No	No	No	No
4428	HRPB0447	Female	Chinese	2018	24	Others	No formal education	Private	Urban	Yes	Yes	Yes	No	No	No	No	No	No	No
4429	HRPB0449	Male	Malay	2019	31	Others	Secondary	Private	Rural	Yes	Yes	Yes	Yes	Yes	No	No	No	No	No
4430	HRPB0450	Male	Malay	2018	37	Others	Secondary	Private	Urban	Yes	Yes	No	Yes	Yes	No	No	No	No	No
4431	HRPB0451	Male	Malay	2018	60	Married	No formal education	Private	Urban	Yes	Yes	No	No	No	No	No	No	No	No
4432	HRPB0452	Male	Malay	2020	22	Others	No formal education	Others	Urban	Yes	Yes	No	No	No	No	No	No	No	No
4433	HRPB0453	Male	Malay	2018	35	Others	No formal education	Others	Urban	Yes	Yes	Yes	No	Yes	No	No	No	No	No
4434	HRPB0454	Male	Malay	2020	27	Others	No formal education	Others	Urban	Yes	No	No	No	Yes	No	No	No	No	No
4435	HRPB0455	Male	Chinese	2019	45	Others	Primary	Unemployed	Urban	Yes	No	No	No	Yes	No	No	No	No	No
4436	HRPB0456	Female	Chinese	2019	67	Others	Primary	Others	Urban	Yes	No	No	No	No	No	Yes	No	No	No
4437	HRPB0457	Male	Chinese	2020	39	Single	No formal education	Unemployed	Urban	Yes	No	No	No	Yes	No	No	No	No	No

No	Patient ID	Gender	Ethnic group	Year of diagnosis	Age	Marital status	Education level	Occupation	Place of residence	History of psy illness	Tobacco	Alcohol	Cannabis	ATS	Inhalant	Sedative	Hallucinogens	Opioids	Kratom
4438	HRPB0458	Female	Chinese	2018	53	Single	No formal education	Others	Urban	Yes	No	Yes	No	No	No	No	No	No	No
4439	HRPB0460	Female	Chinese	2019	66	Others	Primary	Self-employed	Urban	Yes	No	No	No	No	No	Yes	No	No	No
4440	HRPB0462	Male	Malay	2020	52	Married	No formal education	Others	Urban	Yes	Yes	No	No	No	No	No	No	Yes	No
4441	HRPB0463	Male	Malay	2020	24	Single	No formal education	Unemployed	Urban	Yes	No	No	Yes	Yes	No	No	No	No	No
4442	HRPB0464	Male	Malay	2020	53	Married	Secondary	Unemployed	Urban	Yes	Yes	No	No	No	No	No	No	No	No
4443	HRPB0466	Male	Malay	2021	25	Single	No formal education	Others	Urban	Yes	No	Yes	No	Yes	No	No	No	Yes	No
4444	HRPB0467	Male	Malay	2018	40	Single	Secondary	Unemployed	Urban	Yes	Yes	No	No	No	No	No	No	Yes	No
4445	HRPB0469	Male	Malay	2019	75	Married	Secondary	Others	Urban	Yes	Yes	No	No	No	No	No	No	No	No
4446	HRPB0470	Male	Malay	2020	23	Single	Tertiary	Unemployed	Urban	Yes	Yes	No	Yes	No	No	No	No	No	No
4447	HRPB0471	Male	Chinese	2020	60	Single	No formal education	Private	Urban	Yes	No	Yes	No	No	No	No	No	No	No
4448	HRPB0472	Male	Chinese	2020	29	Single	Secondary	Unemployed	Urban	Yes	Yes	No	No	Yes	No	No	No	No	No
4449	HRPB0473	Male	Chinese	2020	42	Single	No formal education	Private	Urban	Yes	No	No	No	No	No	No	No	Yes	No
4450	HRPB0474	Male	Malay	2019	32	Others	No formal education	Unemployed	Urban	Yes	No	No	No	Yes	No	No	No	No	No
4451	HRPB0475	Female	Malay	2018	23	Others	No formal education	Others	Urban	Yes	Yes	No	No	Yes	No	No	No	No	No
4452	HRPB0476	Male	Malay	2020	24	Single	Tertiary	Unemployed	Urban	Yes	Yes	No	No	Yes	No	No	No	No	No
4453	HRPB0479	Male	Others	2018	24	Others	No formal education	Others	Rural	Yes	No	No	No	Yes	No	No	No	No	Yes
4454	HRPB0480	Female	Malay	2018	58	Others	Secondary	Private	Urban	Yes	No	No	No	No	No	Yes	No	No	No
4455	HRPB0481	Female	Chinese	2019	39	Married	Secondary	Private	Urban	Yes	Yes	No	No	No	No	Yes	No	No	No
4456	HRPB0482	Female	Chinese	2020	53	Married	No formal education	Others	Urban	Yes	No	Yes	No	No	No	No	No	No	No
4457	HRPB0484	Female	Chinese	2021	39	Others	Secondary	Unemployed	Urban	Yes	Yes	Yes	No	Yes	No	No	No	No	No
4458	HRPB0485	Female	Malay	2021	22	Single	Secondary	Private	Urban	Yes	Yes	No	No	No	No	No	No	No	No

No	Patient ID	Gender	Ethnic group	Year of diagnosis	Age	Marital status	Education level	Occupation	Place of residence	History of psy illness	Tobacco	Alcohol	Cannabis	ATS	Inhalant	Sedative	Hallucinogens	Opioids	Kratom
4459	HRPB0486	Male	Indian	2018	51	Married	No formal education	Private	Urban	Yes	Yes	Yes	Yes	No	No	No	No	No	No
4460	HRPB0487	Male	Indian	2019	25	Others	Secondary	Private	Urban	Yes	Yes	No	No	No	No	No	No	No	No
4461	HRPB0488	Male	Indian	2018	57	Married	Secondary	Private	Urban	Yes	No	Yes	No	No	No	No	No	No	No
4462	HRPB0490	Male	Chinese	2020	44	Married	Secondary	Unemployed	Urban	Yes	Yes	No	No	No	No	No	No	No	No
4463	HRPB0491	Male	Chinese	2019	45	Single	Primary	Others	Urban	Yes	No	No	No	Yes	No	No	No	No	No
4464	HRPB0492	Female	Malay	2020	21	Others	No formal education	Others	Urban	Yes	Yes	No	No	No	No	No	No	No	No
4465	HRPB0493	Female	Malay	2021	20	Single	No formal education	Unemployed	Urban	Yes	Yes	No	No	No	No	No	No	No	No
4466	HRPB0494	Female	Malay	2021	34	Married	No formal education	Others	Urban	Yes	Yes	No	No	No	No	No	No	No	No
4467	HRPB0495	Male	Malay	2019	45	Single	Secondary	Self-employed	Urban	Yes	Yes	No	No	No	No	No	No	No	No
4468	HRPB0496	Male	Malay	2020	28	Others	No formal education	Others	Urban	Yes	No	No	Yes	Yes	No	No	No	Yes	No
4469	HRPB0498	Male	Chinese	2021	49	Others	No formal education	Others	Urban	Yes	Yes	No	No	No	No	No	No	No	No
4470	HRPB0499	Male	Malay	2020	25	Married	No formal education	Self-employed	Urban	Yes	Yes	No	No	Yes	No	No	No	No	No
4471	HRPB0500	Male	Malay	2018	42	Others	No formal education	Others	Urban	Yes	Yes	No	No	Yes	Yes	No	No	Yes	No
4472	HRPB0501	Female	Indian	2018	42	Married	No formal education	Private	Urban	Yes	No	Yes	No	No	No	No	No	No	No
4473	HRPB0502	Male	Chinese	2020	57	Others	Primary	Private	Urban	Yes	Yes	No	No	No	No	No	No	No	No
4474	HRPB0503	Male	Chinese	2021	34	Others	Secondary	Private	Urban	Yes	Yes	Yes	No	No	No	No	No	No	No
4475	HRPB0504	Male	Chinese	2018	38	Single	No formal education	Private	Urban	Yes	Yes	Yes	No	No	No	No	No	No	No
4476	HRPB0507	Male	Chinese	2021	41	Married	Secondary	Private	Urban	Yes	Yes	No	No	No	No	No	No	No	No
4477	HRPB0509	Male	Indian	2019	58	Others	No formal education	Private	Urban	Yes	No	Yes	No	No	No	No	No	No	No
4478	HRPB0510	Male	Indian	2020	58	Married	Secondary	Private	Urban	Yes	No	Yes	No	No	No	No	No	No	No
4479	HRPB0511	Male	Indian	2020	70	Married	Primary	Unemployed	Urban	No	Yes	Yes	No	No	No	No	No	No	No

No	Patient ID	Gender	Ethnic group	Year of diagnosis	Age	Marital status	Education level	Occupation	Place of residence	History of psy illness	Tobacco	Alcohol	Cannabis	ATS	Inhalant	Sedative	Hallucinogens	Opioids	Kratom
4480	HRPB0512	Male	Indian	2018	25	Others	Tertiary	Private	Urban	Yes	Yes	Yes	No	No	No	No	No	No	No
4481	HRPB0513	Male	Malay	2018	46	Others	No formal education	Others	Urban	Yes	Yes	No	No	Yes	No	No	No	No	No
4482	HRPB0514	Male	Malay	2021	35	Married	No formal education	Private	Urban	Yes	Yes	No	No	No	No	No	No	No	No
4483	HRPB0515	Male	Malay	2020	30	Single	No formal education	Unemployed	Urban	Yes	Yes	No	No	No	No	No	No	No	No
4484	HRPB0516	Male	Chinese	2020	48	Others	No formal education	Others	Urban	Yes	No	No	No	Yes	No	No	No	No	No
4485	HRPB0517	Male	Indian	2019	33	Single	No formal education	Private	Urban	Yes	No	Yes	No	No	No	No	No	No	No
4486	HRPB0518	Male	Chinese	2018	46	Others	No formal education	Others	Urban	Yes	No	Yes	No	No	No	No	No	No	No
4487	HRPB0519	Male	Chinese	2019	47	Others	No formal education	Others	Urban	Yes	Yes	No	No	No	No	No	No	No	No
4488	HRPB0520	Male	Malay	2018	31	Married	Tertiary	Government	Urban	Yes	Yes	No	No	No	No	No	No	No	No
4489	HRPB0521	Male	Indian	2020	47	Married	Secondary	Private	Urban	No	Yes	Yes	Yes	No	No	No	No	No	No
4490	HRPB0522	Male	Chinese	2021	35	Single	No formal education	Unemployed	Urban	Yes	No	No	No	Yes	No	No	No	No	No
4491	HRPB0523	Male	Malay	2020	38	Single	No formal education	Unemployed	Urban	Yes	No	No	No	No	No	No	No	Yes	No
4492	HRPB0524	Male	Indian	2020	37	Married	No formal education	Private	Urban	Yes	No	Yes	No	No	No	No	No	No	No
4493	HRPB0525	Male	Malay	2020	42	Single	No formal education	Unemployed	Urban	Yes	Yes	Yes	No	Yes	No	No	No	No	No
4494	HRPB0526	Male	Chinese	2020	75	Married	No formal education	Others	Urban	Yes	No	No	No	No	No	Yes	No	No	No
4495	HRPB0527	Male	Chinese	2019	35	Married	No formal education	Private	Urban	Yes	Yes	Yes	No	Yes	No	No	No	No	No
4496	HRPB0529	Female	Chinese	2019	40	Others	No formal education	Others	Urban	Yes	Yes	Yes	No	No	No	No	No	No	No
4497	HRPB0530	Female	Chinese	2018	69	Married	No formal education	Unemployed	Urban	Yes	No	Yes	No	No	No	No	No	No	No
4498	HRPB0532	Male	Chinese	2018	36	Married	Secondary	Self-employed	Urban	Yes	Yes	No	No	Yes	No	No	No	No	No
4499	HRPB0533	Male	Chinese	2021	75	Single	No formal education	Others	Urban	Yes	No	Yes	No	No	No	No	No	No	No
4500	HRPB0534	Male	Chinese	2021	51	Single	Secondary	Unemployed	Urban	Yes	Yes	No	Yes	No	No	No	No	No	No

No	Patient ID	Gender	Ethnic group	Year of diagnosis	Age	Marital status	Education level	Occupation	Place of residence	History of psy illness	Tobacco	Alcohol	Cannabis	ATS	Inhalant	Sedative	Hallucinogens	Opioids	Kratom
4501	HRPB0536	Female	Chinese	2019	38	Married	Secondary	Private	Urban	Yes	Yes	No	No	No	No	No	No	No	No
4502	HRPB0538	Male	Chinese	2020	32	Single	No formal education	Unemployed	Urban	Yes	No	Yes	No	No	No	No	No	No	No
4503	HRPB0539	Male	Malay	2019	33	Others	No formal education	Others	Rural	Yes	No	No	No	Yes	No	No	No	Yes	No
4504	HRPB0542	Male	Malay	2018	32	Married	Secondary	Private	Urban	Yes	Yes	No	No	No	No	No	No	No	No
4505	HRPB0555	Male	Chinese	2019	52	Single	Secondary	Unemployed	Urban	Yes	No	No	Yes	Yes	No	No	No	Yes	No
4506	HRPB0559	Male	Chinese	2020	68	Others	Secondary	Self-employed	Urban	Yes	Yes	No	No	No	No	No	No	No	No
4507	HRPB0560	Male	Indian	2021	34	Married	No formal education	Private	Urban	Yes	No	Yes	No	No	No	No	No	No	No
4508	HRPB0562	Male	Chinese	2020	48	Others	No formal education	Others	Urban	Yes	Yes	No	No	No	No	No	No	No	No
4509	HRPB0563	Female	Indian	2019	22	Others	No formal education	Others	Urban	Yes	No	No	No	Yes	No	No	No	No	No
4510	HRPB0564	Male	Indian	2020	30	Married	No formal education	Private	Urban	Yes	No	No	Yes	No	No	No	No	No	No
4511	HRPB0565	Male	Malay	2019	19	Single	Primary	Self-employed	Urban	Yes	No	No	No	No	No	No	No	No	Yes
4512	HRPB0566	Male	Malay	2019	36	Married	No formal education	Government	Urban	Yes	No	No	No	Yes	No	No	No	No	No
4513	HRPB0567	Male	Malay	2019	29	Others	No formal education	Private	Urban	Yes	Yes	No	No	No	No	No	No	No	No
4514	HRPB0568	Male	Malay	2019	24	Others	No formal education	Government	Urban	Yes	Yes	No	No	No	No	No	No	No	No
4515	HRPB0569	Male	Indian	2020	51	Married	Secondary	Private	Urban	Yes	Yes	Yes	No	No	No	No	No	No	No
4516	HRPB0570	Male	Chinese	2018	73	Married	No formal education	Others	Urban	Yes	Yes	No	No	No	No	No	No	No	No
4517	HRPB0572	Male	Malay	2021	33	Single	No formal education	Unemployed	Urban	Yes	No	No	No	Yes	No	No	No	No	No
4518	HRPB0573	Male	Chinese	2020	38	Single	No formal education	Unemployed	Urban	Yes	Yes	No	No	No	No	No	No	No	No
4519	HRPB0574	Female	Indian	2020	29	Others	No formal education	Others	Urban	Yes	No	Yes	No	No	No	No	No	No	No
4520	HRPB0575	Male	Chinese	2021	51	Others	No formal education	Others	Urban	Yes	Yes	No	No	No	No	No	No	No	No
4521	HRPB0576	Male	Chinese	2019	54	Others	No formal education	Unemployed	Urban	Yes	Yes	No	No	No	No	No	No	No	No

No	Patient ID	Gender	Ethnic group	Year of diagnosis	Age	Marital status	Education level	Occupation	Place of residence	History of psy illness	Tobacco	Alcohol	Cannabis	ATS	Inhalant	Sedative	Hallucinogens	Opioids	Kratom
4522	HRPB0577	Male	Indian	2020	37	Others	No formal education	Others	Urban	Yes	No	No	Yes	No	No	No	No	No	No
4523	HRPB0578	Male	Indian	2019	45	Others	No formal education	Others	Urban	Yes	No	Yes	No	No	No	No	No	Yes	No
4524	HRPB0579	Male	Chinese	2018	60	Others	No formal education	Others	Urban	Yes	No	Yes	No	No	No	No	No	Yes	No
4525	HRPB0580	Male	Malay	2018	31	Married	Secondary	Self-employed	Urban	Yes	Yes	No	Yes	No	No	No	No	No	No
4526	HRPB0581	Male	Malay	2020	37	Others	No formal education	Others	Urban	Yes	Yes	No	No	No	No	No	No	No	No
4527	HRPB0582	Male	Others	2019	33	Married	No formal education	Others	Urban	Yes	Yes	Yes	No	No	No	No	No	No	No
4528	HRPB0583	Male	Malay	2018	56	Married	No formal education	Others	Urban	Yes	Yes	No	No	No	No	Yes	No	No	No
4529	HRPB0584	Male	Indian	2021	32	Single	No formal education	Unemployed	Urban	Yes	Yes	Yes	Yes	Yes	No	No	No	Yes	No
4530	HRPB0585	Male	Malay	2018	28	Others	No formal education	Others	Urban	Yes	No	No	No	Yes	No	No	No	No	No
4531	HRPB0586	Male	Indian	2021	54	Married	Secondary	Private	Urban	Yes	No	Yes	No	No	No	No	No	No	No
4532	HRPB0594	Female	Chinese	2020	39	Married	Primary	Unemployed	Urban	Yes	No	Yes	No	No	No	No	No	No	No
4533	HRPB0595	Female	Chinese	2018	75	Married	Primary	Self-employed	Urban	Yes	No	No	No	No	No	Yes	No	No	No
4534	HRPB0599	Female	Chinese	2021	32	Others	No formal education	Others	Urban	Yes	No	Yes	No	No	No	No	No	No	No
4535	HRPB0600	Male	Malay	2018	28	Others	Tertiary	Unemployed	Urban	Yes	Yes	No	Yes	No	No	No	No	No	No
4536	HRPB0601	Female	Malay	2021	21	Others	No formal education	Others	Urban	Yes	Yes	Yes	No	No	No	No	No	No	No
4537	HRPB0603	Female	Indian	2020	39	Others	No formal education	Others	Urban	Yes	Yes	Yes	No	No	No	No	No	No	No
4538	HRPB0604	Male	Indian	2020	38	Married	Tertiary	Private	Urban	Yes	Yes	Yes	No	No	No	No	No	No	No
4539	HRPB0605	Female	Chinese	2020	74	Married	No formal education	Others	Urban	Yes	No	Yes	No	No	No	Yes	No	No	No
4540	HRPB0612	Male	Chinese	2020	55	Others	No formal education	Others	Urban	Yes	No	Yes	No	No	No	No	No	No	No
4541	HRPB0613	Male	Indian	2018	32	Single	No formal education	Self-employed	Urban	Yes	Yes	Yes	No	No	No	No	No	No	No
4542	HRPB0614	Male	Malay	2019	67	Married	Secondary	Government	Urban	Yes	Yes	No	No	No	No	No	No	No	No

No	Patient ID	Gender	Ethnic group	Year of diagnosis	Age	Marital status	Education level	Occupation	Place of residence	History of psy illness	Tobacco	Alcohol	Cannabis	ATS	Inhalant	Sedative	Hallucinogens	Opioids	Kratom
4543	HRPB0615	Male	Chinese	2018	70	Married	No formal education	Others	Urban	Yes	No	No	No	No	No	Yes	No	No	No
4544	HRPB0617	Male	Chinese	2020	55	Single	No formal education	Private	Urban	Yes	No	No	No	Yes	No	No	No	Yes	No
4545	HRPB0618	Male	Indian	2018	23	Married	Secondary	Others	Urban	Yes	No	Yes	Yes	Yes	No	No	No	No	No
4546	HRPB0619	Male	Malay	2019	22	Others	No formal education	Others	Urban	Yes	No	No	No	Yes	No	No	No	No	No
4547	HRPB0620	Male	Indian	2020	67	Married	No formal education	Private	Urban	Yes	Yes	Yes	No	No	No	No	No	No	No
4548	HRPB0621	Male	Chinese	2019	70	Married	Primary	Unemployed	Urban	Yes	Yes	No	No	No	No	No	No	No	No
4549	HRPB0622	Male	Chinese	2018	60	Others	No formal education	Others	Urban	Yes	No	No	No	No	No	Yes	No	No	No
4550	HRPB0623	Male	Malay	2021	24	Others	No formal education	Others	Urban	Yes	Yes	No	Yes	No	No	No	No	No	No
4551	HRPB0624	Male	Indian	2018	61	Married	No formal education	Unemployed	Urban	Yes	Yes	Yes	No	No	No	No	No	No	No
4552	HRPB0625	Male	Malay	2019	26	Others	No formal education	Others	Urban	Yes	Yes	No	No	No	No	No	No	No	No
4553	HRPB0627	Male	Chinese	2019	60	Others	No formal education	Others	Urban	Yes	No	Yes	No	No	No	No	No	No	No
4554	HRPB0628	Male	Malay	2020	25	Married	No formal education	Unemployed	Urban	Yes	Yes	No	No	No	No	No	No	No	Yes
4555	HRPB0629	Male	Malay	2018	30	Married	Tertiary	Private	Urban	Yes	No	No	Yes	Yes	No	No	No	No	No
4556	HRPB0630	Male	Indian	2020	31	Single	Secondary	Unemployed	Urban	Yes	Yes	Yes	No	No	No	No	No	No	No
4557	HRPB0631	Male	Malay	2019	21	Others	No formal education	Others	Urban	Yes	Yes	No	No	No	No	No	No	No	No
4558	HRPB0632	Male	Malay	2020	30	Others	No formal education	Others	Urban	Yes	Yes	No	No	Yes	No	No	No	No	No
4559	HRPB0633	Male	Indian	2018	35	Others	Secondary	Others	Urban	Yes	Yes	Yes	No	No	No	No	No	No	No
4560	HRPB0634	Male	Indian	2018	50	Married	No formal education	Self-employed	Urban	Yes	No	Yes	No	No	No	No	No	No	No
4561	HRPB0635	Male	Chinese	2020	64	Others	No formal education	Others	Urban	Yes	No	No	No	No	No	No	No	Yes	No
4562	HRPB0638	Male	Indian	2021	28	Others	No formal education	Others	Urban	Yes	No	Yes	No	No	No	No	No	No	No
4563	HRPB0639	Male	Indian	2020	74	Others	Secondary	Others	Urban	Yes	No	Yes	No	No	No	No	No	No	No

No	Patient ID	Gender	Ethnic group	Year of diagnosis	Age	Marital status	Education level	Occupation	Place of residence	History of psy illness	Tobacco	Alcohol	Cannabis	ATS	Inhalant	Sedative	Hallucinogens	Opioids	Kratom
4564	HRPB0640	Male	Indian	2018	53	Others	No formal education	Others	Urban	Yes	No	Yes	No	No	No	No	No	No	No
4565	HRPB0642	Male	Indian	2020	36	Married	Tertiary	Unemployed	Urban	Yes	No	Yes	No	No	No	No	No	No	No
4566	HRPB0643	Male	Indian	2018	65	Others	No formal education	Others	Urban	Yes	No	Yes	No	No	No	No	No	No	No
4567	HRPB0645	Male	Malay	2021	35	Married	No formal education	Others	Urban	Yes	No	No	No	Yes	No	No	No	No	No
4568	HRPB0676	Male	Malay	2019	55	Single	No formal education	Private	Urban	Yes	No	No	No	Yes	No	No	No	Yes	No
4569	HRPB0677	Male	Malay	2019	25	Others	No formal education	Others	Urban	Yes	No	No	No	No	No	No	No	Yes	No
4570	HRPB0687	Male	Malay	2019	41	Married	Secondary	Private	Urban	Yes	No	No	Yes	Yes	No	No	No	Yes	No
4571	HRPB0689	Male	Malay	2021	48	Others	No formal education	Others	Urban	Yes	No	No	No	Yes	No	Yes	No	Yes	No
4572	HRPB0704	Male	Indian	2019	41	Others	No formal education	Others	Urban	Yes	No	No	No	No	No	No	No	Yes	No
4573	HRPB0707	Male	Malay	2021	44	Single	Secondary	Self-employed	Urban	Yes	No	No	No	No	No	Yes	No	Yes	No
4574	HRPB0712	Male	Malay	2021	43	Married	No formal education	Others	Urban	Yes	No	No	No	Yes	No	No	No	Yes	No
4575	HRPB0724	Male	Malay	2021	26	Married	No formal education	Private	Rural	Yes	No	No	No	Yes	No	No	No	Yes	No
4576	HRPB0730	Male	Malay	2018	16	Married	Secondary	Private	Urban	Yes	No	No	No	No	No	No	No	Yes	No
4577	HRPB0731	Male	Malay	2020	39	Others	No formal education	Others	Urban	Yes	No	No	No	Yes	No	No	No	Yes	No
4578	HRPB0739	Male	Indian	2019	35	Others	No formal education	Private	Urban	Yes	No	Yes	No	Yes	No	No	No	No	No
4579	HRPB0740	Male	Malay	2019	35	Others	No formal education	Others	Urban	Yes	No	No	No	Yes	No	No	No	No	No
4580	HRPB0741	Male	Malay	2019	31	Single	Secondary	Unemployed	Urban	No	Yes	No	No	Yes	No	Yes	No	Yes	No
4581	HRPB0746	Male	Indian	2019	47	Others	No formal education	Others	Urban	Yes	No	No	No	No	No	No	No	Yes	No
4582	HRPB0747	Male	Malay	2019	32	Others	Secondary	Others	Urban	Yes	No	No	No	No	No	No	No	Yes	No
4583	HRPB0751	Male	Malay	2018	30	Single	Secondary	Others	Urban	Yes	No	No	No	No	No	No	No	Yes	No
4584	HRPB0752	Male	Indian	2019	38	Single	Primary	Private	Urban	Yes	No	No	No	Yes	No	No	No	Yes	No

No	Patient ID	Gender	Ethnic group	Year of diagnosis	Age	Marital status	Education level	Occupation	Place of residence	History of psy illness	Tobacco	Alcohol	Cannabis	ATS	Inhalant	Sedative	Hallucinogens	Opioids	Kratom
4585	HRPB0753	Male	Malay	2019	40	Others	No formal education	Others	Urban	Yes	No	No	No	No	No	Yes	No	No	No
4586	HRPB0754	Male	Malay	2020	53	Others	No formal education	Private	Urban	Yes	No	No	No	No	No	Yes	No	No	No
4587	HRPB0755	Male	Malay	2018	36	Single	Secondary	Unemployed	Urban	Yes	No	No	No	Yes	No	No	No	Yes	No
4588	HRPB0761	Male	Malay	2018	39	Others	No formal education	Others	Urban	Yes	No	No	No	Yes	No	No	No	Yes	No
4589	HRPB0763	Male	Malay	2019	24	Single	Secondary	Unemployed	Urban	Yes	No	No	Yes	No	No	No	No	Yes	No
4590	HSA0001	Male	Indian	2019	28	Married	Secondary	Private	Urban	Yes	No	Yes	No	No	No	No	No	No	No
4591	HSA0002	Male	Malay	2019	37	Married	Tertiary	Government	Urban	No	No	No	No	Yes	No	No	No	No	No
4592	HSA0004	Male	Chinese	2020	37	Single	No formal education	Others	Urban	No	No	No	No	Yes	No	No	No	No	No
4593	HSA0005	Male	Malay	2021	49	Others	No formal education	Unemployed	Urban	Yes	No	No	No	Yes	Yes	No	No	No	No
4594	HSA0006	Male	Indian	2018	35	Others	No formal education	Others	Urban	Yes	No	Yes	No	No	No	No	No	No	No
4595	HSA0007	Male	Chinese	2018	30	Single	Tertiary	Unemployed	Urban	Yes	No	No	No	Yes	No	No	No	No	No
4596	HSA0008	Male	Malay	2019	37	Married	No formal education	Government	Urban	No	No	No	No	Yes	No	No	No	No	No
4597	HSA0009	Male	Malay	2021	51	Married	Secondary	Private	Rural	Yes	Yes	Yes	Yes	No	Yes	No	No	Yes	No
4598	HSA0010	Male	Indian	2020	34	Single	Secondary	Private	Urban	No	No	Yes	No	No	No	No	No	No	No
4599	HSA0011	Male	Indian	2021	55	Married	No formal education	Private	Urban	No	No	Yes	No	No	No	No	No	No	No
4600	HSA0012	Male	Indian	2021	27	Single	Secondary	Self-employed	Urban	No	Yes	Yes	Yes	No	No	No	No	No	No
4601	HSA0013	Male	Malay	2021	25	Others	Tertiary	Private	Urban	Yes	Yes	No	Yes	No	No	No	No	No	No
4602	HSA0014	Male	Malay	2021	37	Others	Secondary	Self-employed	Urban	Yes	Yes	No	No	No	No	No	No	No	No
4603	HSA0016	Female	Chinese	2020	55	Married	Secondary	Unemployed	Urban	Yes	No	Yes	No	No	No	No	No	No	No
4604	HSA0019	Male	Malay	2020	30	Married	Tertiary	Private	Urban	Yes	No	No	Yes	Yes	No	No	No	No	No
4605	HSA0020	Male	Malay	2021	27	Single	Tertiary	Private	Rural	No	Yes	No	No	No	No	No	No	No	No
4606	HSA0022	Female	Malay	2021	21	Single	Primary	Unemployed	Urban	Yes	No	No	No	Yes	No	No	No	No	No
4607	HSA0023	Male	Malay	2018	20	Single	Tertiary	Unemployed	Urban	Yes	Yes	No	Yes	Yes	No	No	No	No	No
4608	HSA0024	Male	Malay	2020	28	Married	Tertiary	Government	Rural	Yes	Yes	No	No	No	No	No	No	No	No
4609	HSA0025	Male	Malay	2020	31	Married	No formal education	Private	Rural	Yes	Yes	No	No	Yes	No	No	No	No	No
4610	HSA0027	Male	Chinese	2020	31	Single	Secondary	Unemployed	Urban	Yes	No	No	No	Yes	No	No	No	No	No
4611	HSA0029	Male	Malay	2018	44	Married	No formal education	Others	Urban	Yes	Yes	No	No	No	No	No	No	No	No
4612	HSA0030	Male	Chinese	2020	27	Single	Secondary	Unemployed	Urban	Yes	Yes	Yes	No	No	No	No	No	No	No
4613	HSA0031	Male	Indian	2020	36	Married	Secondary	Private	Urban	Yes	Yes	Yes	No	No	No	No	No	No	No
4614	HSA0032	Male	Malay	2018	25	Single	Secondary	Unemployed	Urban	Yes	Yes	No	No	Yes	No	No	No	No	No
4615	HSA0033	Male	Chinese	2019	29	Single	Secondary	Unemployed	Urban	Yes	Yes	Yes	No	Yes	No	No	No	No	No
4616	HSA0034	Male	Malay	2021	27	Married	Tertiary	Government	Urban	No	Yes	No	No	Yes	No	No	No	No	No

No	Patient ID	Gender	Ethnic group	Year of diagnosis	Age	Marital status	Education level	Occupation	Place of residence	History of psy illness	Tobacco	Alcohol	Cannabis	ATS	Inhalant	Sedative	Hallucinogens	Opioids	Kratom
4617	HSA0036	Male	Malay	2019	43	Single	No formal education	Private	Urban	Yes	Yes	No	No	No	No	No	No	No	No
4618	HSA0037	Male	Indian	2019	23	Single	Secondary	Private	Urban	Yes	Yes	Yes	No	Yes	No	No	No	No	No
4619	HSA0039	Male	Malay	2021	45	Single	No formal education	Others	Urban	Yes	No	Yes	No	Yes	No	No	No	No	No
4620	HSA0040	Male	Malay	2020	26	Single	Secondary	Unemployed	Urban	Yes	Yes	No	Yes	Yes	No	No	No	No	No
4621	HSA0041	Male	Chinese	2019	66	Married	No formal education	Unemployed	Urban	Yes	Yes	No	No	No	No	No	No	No	No
4622	HSA0042	Male	Indian	2021	20	Single	Secondary	Unemployed	Urban	No	Yes	No	No	No	No	No	No	No	No
4623	HSA0043	Male	Malay	2019	39	Married	Secondary	Government	Urban	No	Yes	Yes	No	Yes	No	No	No	No	No
4624	HSA0044	Male	Malay	2020	20	Single	Secondary	Unemployed	Urban	Yes	Yes	No	No	Yes	No	No	No	No	No
4625	HSA0045	Male	Chinese	2019	22	Single	No formal education	Unemployed	Urban	Yes	No	No	No	Yes	No	No	No	No	No
4626	HSA0047	Male	Indian	2018	28	Single	Secondary	Unemployed	Urban	Yes	Yes	Yes	No	Yes	No	No	No	No	No
4627	HSA0048	Male	Chinese	2019	31	Single	No formal education	Unemployed	Urban	Yes	No	Yes	No	No	No	No	No	No	No
4628	HSA0049	Male	Malay	2020	28	Married	No formal education	Private	Urban	Yes	Yes	No	No	No	No	No	No	No	No
4629	HSA0052	Male	Malay	2021	30	Single	Secondary	Private	Urban	Yes	Yes	No	No	No	No	No	No	No	No
4630	HSA0053	Male	Indian	2019	52	Married	No formal education	Private	Urban	No	No	Yes	No	No	No	No	No	No	No
4631	HSA0055	Male	Malay	2020	41	Married	No formal education	Unemployed	Rural	Yes	Yes	Yes	No	Yes	No	No	No	No	No
4632	HSA0057	Male	Malay	2021	29	Single	Tertiary	Private	Urban	Yes	No	No	No	Yes	No	No	No	No	No
4633	HSA0058	Male	Chinese	2019	44	Single	Secondary	Private	Urban	Yes	No	Yes	No	No	No	No	No	No	No
4634	HSA0059	Male	Malay	2021	25	Single	Tertiary	Government	Urban	Yes	Yes	No	No	No	No	No	No	No	No
4635	HSA0060	Male	Chinese	2020	27	Single	Tertiary	Government	Urban	Yes	No	Yes	No	No	No	No	No	No	No
4636	HSA0061	Male	Chinese	2018	30	Married	No formal education	Self-employed	Urban	Yes	Yes	Yes	No	No	No	No	No	No	No
4637	HSA0062	Male	Malay	2020	31	Single	Secondary	Self-employed	Urban	Yes	Yes	No	No	No	No	No	No	No	No
4638	HSA0063	Male	Malay	2018	29	Married	Secondary	Private	Urban	Yes	Yes	No	No	No	No	No	No	No	No
4639	HSA0064	Male	Malay	2019	31	Married	Tertiary	Unemployed	Urban	Yes	Yes	No	No	No	No	No	No	No	No
4640	HSA0065	Male	Malay	2019	15	Single	Secondary	Unemployed	Urban	Yes	Yes	No	No	Yes	No	No	No	No	No
4641	HSA0066	Male	Indian	2019	61	Married	No formal education	Others	Urban	Yes	Yes	Yes	No	No	No	No	No	No	No
4642	HSA0067	Male	Chinese	2019	29	Married	Secondary	Private	Urban	Yes	Yes	Yes	No	No	No	No	No	No	No
4643	HSA0068	Male	Malay	2019	30	Single	Tertiary	Government	Urban	No	Yes	No	No	Yes	No	No	No	No	No
4644	HSA0069	Male	Indian	2020	44	Others	Tertiary	Private	Urban	Yes	Yes	Yes	No	No	No	No	No	No	No
4645	HSA0070	Male	Malay	2021	21	Single	Tertiary	Unemployed	Urban	No	No	Yes	Yes	No	No	No	No	No	No
4646	HSA0071	Male	Chinese	2018	66	Married	No formal education	Unemployed	Urban	Yes	No	Yes	No	No	No	No	No	No	No
4647	HSA0072	Male	Malay	2020	22	Single	Tertiary	Private	Urban	Yes	Yes	No	Yes	No	No	No	No	No	No
4648	HSA0073	Male	Malay	2019	29	Others	Tertiary	Private	Rural	Yes	Yes	Yes	No	Yes	No	No	No	No	No
4649	HSA0074	Male	Malay	2019	72	Others	Secondary	Unemployed	Urban	Yes	Yes	No	No	No	No	No	No	No	No

No	Patient ID	Gender	Ethnic group	Year of diagnosis	Age	Marital status	Education level	Occupation	Place of residence	History of psy illness	Tobacco	Alcohol	Cannabis	ATS	Inhalant	Sedative	Hallucinogens	Opioids	Kratom
4650	HSA0075	Male	Chinese	2021	74	Married	Tertiary	Unemployed	Urban	Yes	No	Yes	No	No	No	No	No	No	No
4651	HSA0077	Male	Malay	2020	27	Married	No formal education	Private	Urban	Yes	Yes	No	No	No	No	No	No	No	No
4652	HSA0078	Male	Others	2020	28	Single	Tertiary	Government	Urban	Yes	No	Yes	No	No	No	No	No	No	No
4653	HSA0079	Male	Chinese	2020	62	Others	Secondary	Unemployed	Urban	Yes	No	Yes	No	No	No	No	No	No	No
4654	HSA0080	Male	Chinese	2021	46	Married	No formal education	Unemployed	Urban	Yes	Yes	No	No	Yes	No	No	No	No	No
4655	HSA0081	Male	Malay	2021	30	Single	Secondary	Private	Urban	Yes	No	No	No	Yes	No	No	No	No	No
4656	HSA0082	Male	Others	2021	59	Single	No formal education	Unemployed	Rural	No	Yes	No	No	No	No	No	No	No	No
4657	HSA0083	Male	Indian	2018	36	Married	No formal education	Others	Urban	Yes	No	No	Yes	Yes	No	No	No	Yes	No
4658	HSA0084	Male	Chinese	2020	34	Single	Secondary	Unemployed	Urban	Yes	Yes	No	No	Yes	No	No	No	No	No
4659	HSA0085	Male	Malay	2018	40	Married	Secondary	Private	Urban	Yes	Yes	No	Yes	No	No	No	No	No	No
4660	HSA0086	Male	Malay	2020	35	Others	No formal education	Private	Urban	Yes	Yes	No	No	Yes	No	No	No	No	No
4661	HSA0087	Male	Indian	2019	51	Others	Secondary	Unemployed	Urban	Yes	Yes	Yes	No	No	No	No	No	No	No
4662	HSA0088	Male	Malay	2018	25	Married	Secondary	Private	Urban	Yes	No	Yes	No	No	No	No	No	No	No
4663	HSA0089	Male	Malay	2019	28	Single	Secondary	Unemployed	Urban	Yes	Yes	No	No	Yes	No	No	No	No	No
4664	HSA0090	Male	Chinese	2020	25	Single	No formal education	Private	Urban	Yes	No	No	No	Yes	No	No	No	No	No
4665	HSA0091	Male	Malay	2020	36	Others	No formal education	Unemployed	Urban	Yes	No	No	No	Yes	No	No	No	Yes	No
4666	HSA0092	Male	Malay	2020	38	Single	Tertiary	Unemployed	Rural	No	Yes	No	No	No	No	No	No	No	No
4667	HSA0093	Male	Indian	2018	59	Married	No formal education	Unemployed	Rural	Yes	Yes	Yes	No	No	No	No	No	No	No
4668	HSA0094	Male	Chinese	2020	38	Single	Secondary	Unemployed	Urban	No	No	No	No	Yes	No	No	No	No	No
4669	HSA0095	Male	Malay	2018	31	Others	No formal education	Private	Rural	Yes	Yes	No	No	No	No	No	No	No	No
4670	HSA0097	Male	Others	2019	28	Single	Tertiary	Unemployed	Urban	Yes	Yes	Yes	No	Yes	No	No	No	No	No
4671	HSA0098	Male	Malay	2019	20	Single	Secondary	Unemployed	Urban	Yes	Yes	No	No	No	No	No	No	No	No
4672	HSA0099	Male	Chinese	2020	46	Single	Secondary	Unemployed	Urban	Yes	Yes	Yes	No	No	No	No	No	No	No
4673	HSA0100	Male	Malay	2020	17	Single	Secondary	Unemployed	Urban	No	Yes	Yes	No	No	No	No	No	No	No
4674	HSA0102	Male	Malay	2021	37	Married	No formal education	Government	Urban	Yes	Yes	No	No	No	No	No	No	No	No
4675	HSA0103	Male	Malay	2020	52	Others	No formal education	Unemployed	Urban	Yes	Yes	No	No	Yes	No	No	No	Yes	No
4676	HSA0104	Male	Malay	2019	22	Single	Tertiary	Unemployed	Urban	Yes	No	Yes	Yes	Yes	No	No	No	No	No
4677	HSA0105	Male	Malay	2021	43	Others	Tertiary	Private	Urban	Yes	Yes	No	No	No	Yes	No	No	No	No
4678	HSA0106	Male	Others	2020	29	Single	No formal education	Private	Urban	Yes	Yes	No	No	No	No	No	No	No	No
4679	HSA0107	Male	Malay	2021	38	Married	No formal education	Government	Urban	No	Yes	No	No	Yes	No	No	No	No	No
4680	HSA0108	Male	Malay	2018	24	Single	Tertiary	Private	Urban	No	No	Yes	Yes	No	No	No	No	No	No

No	Patient ID	Gender	Ethnic group	Year of diagnosis	Age	Marital status	Education level	Occupation	Place of residence	History of psy illness	Tobacco	Alcohol	Cannabis	ATS	Inhalant	Sedative	Hallucinogens	Opioids	Kratom
4681	HSA0109	Male	Malay	2019	39	Single	Tertiary	Private	Urban	Yes	Yes	No	No	No	No	No	No	No	No
4682	HSA0110	Male	Indian	2020	30	Others	Tertiary	Private	Urban	Yes	Yes	Yes	No	Yes	No	No	No	No	No
4683	HSA0111	Male	Malay	2021	55	Married	Secondary	Unemployed	Rural	Yes	No	No	No	Yes	No	No	No	Yes	No
4684	HSA0112	Male	Malay	2020	28	Single	No formal education	Self-employed	Urban	Yes	No	No	Yes	Yes	No	No	No	No	No
4685	HSA0113	Male	Chinese	2018	31	Single	No formal education	Private	Urban	Yes	Yes	Yes	No	No	No	No	No	No	No
4686	HSA0116	Male	Chinese	2018	20	Single	Tertiary	Unemployed	Urban	Yes	No	Yes	No	No	No	No	No	No	No
4687	HSA0117	Male	Chinese	2020	36	Married	No formal education	Self-employed	Urban	Yes	No	Yes	No	No	No	No	No	No	No
4688	HSA0118	Male	Malay	2020	54	Married	Tertiary	Private	Urban	Yes	Yes	No	No	No	No	No	No	No	No
4689	HSA0119	Male	Chinese	2020	71	Married	Tertiary	Unemployed	Urban	Yes	Yes	No	No	No	No	No	No	No	No
4690	HSA0120	Male	Chinese	2019	47	Single	Secondary	Private	Urban	Yes	Yes	No	No	No	No	No	No	No	No
4691	HSA0121	Male	Malay	2020	26	Married	Secondary	Private	Rural	No	Yes	No	No	No	No	No	No	No	No
4692	HSA0122	Male	Malay	2018	44	Married	Tertiary	Private	Urban	No	Yes	No	No	No	No	No	No	No	No
4693	HSA0123	Male	Chinese	2020	30	Single	Tertiary	Unemployed	Urban	Yes	Yes	No	No	No	No	No	No	No	No
4694	HSA0124	Male	Malay	2019	24	Married	Secondary	Private	Urban	Yes	Yes	No	No	No	No	No	No	No	Yes
4695	HSA0126	Male	Malay	2021	43	Married	Secondary	Self-employed	Rural	Yes	Yes	Yes	Yes	No	No	No	No	No	No
4696	HSA0127	Male	Malay	2021	28	Single	Tertiary	Private	Rural	Yes	Yes	Yes	Yes	Yes	No	No	No	No	No
4697	HSA0128	Male	Malay	2020	38	Married	Secondary	Self-employed	Rural	Yes	Yes	No	Yes	No	No	No	No	No	No
4698	HSA0129	Male	Chinese	2019	34	Others	Tertiary	Private	Rural	No	No	No	No	Yes	No	No	No	No	No
4699	HSA0130	Male	Malay	2018	34	Others	No formal education	Private	Urban	Yes	Yes	Yes	Yes	Yes	No	No	No	No	No
4700	HSA0131	Male	Indian	2020	31	Others	Secondary	Unemployed	Urban	Yes	No	No	Yes	Yes	No	No	No	No	No
4701	HSA0132	Male	Malay	2020	25	Single	Secondary	Government	Urban	No	No	No	No	Yes	No	No	No	No	No
4702	HSA0133	Male	Chinese	2021	46	Single	Secondary	Self-employed	Urban	Yes	No	Yes	No	No	No	No	No	No	No
4703	HSA0134	Male	Indian	2020	34	Married	Secondary	Private	Urban	Yes	Yes	No	No	Yes	No	No	No	No	No
4704	HSA0135	Male	Chinese	2020	29	Others	No formal education	Private	Urban	Yes	No	No	No	No	No	No	No	No	No
4705	HSA0136	Male	Indian	2021	44	Married	No formal education	Unemployed	Urban	No	No	Yes	No	No	No	No	No	No	No
4706	HSA0137	Male	Chinese	2020	70	Married	Primary	Unemployed	Urban	Yes	No	Yes	No	No	No	No	No	No	No
4707	HSA0138	Male	Malay	2020	19	Single	Secondary	Private	Urban	Yes	Yes	Yes	No	No	No	No	No	No	No
4708	HSA0139	Male	Malay	2021	29	Single	Secondary	Unemployed	Rural	Yes	No	No	No	Yes	No	No	No	No	No
4709	HSA0140	Male	Chinese	2020	22	Single	Secondary	Unemployed	Urban	Yes	No	Yes	No	No	No	No	No	No	No
4710	HSA0142	Male	Malay	2020	30	Single	Secondary	Private	Urban	Yes	No	No	No	Yes	No	No	No	No	No
4711	HSA0143	Male	Malay	2019	28	Married	No formal education	Private	Urban	Yes	No	No	Yes	No	No	No	No	No	No
4712	HSA0144	Male	Malay	2021	29	Single	Tertiary	Private	Urban	Yes	No	No	No	Yes	No	No	No	No	No
4713	HSA0145	Male	Chinese	2019	24	Single	Secondary	Unemployed	Urban	Yes	Yes	Yes	No	No	No	No	No	No	No
4714	HSA0146	Male	Malay	2019	22	Single	Tertiary	Unemployed	Urban	Yes	No	No	Yes	No	No	No	No	No	No
4715	HSA0147	Male	Chinese	2021	24	Single	Tertiary	Unemployed	Urban	Yes	No	Yes	No	No	No	No	No	No	No
4716	HSA0148	Male	Chinese	2019	32	Married	Tertiary	Private	Urban	Yes	Yes	Yes	No	No	No	No	No	No	No

No	Patient ID	Gender	Ethnic group	Year of diagnosis	Age	Marital status	Education level	Occupation	Place of residence	History of psy illness	Tobacco	Alcohol	Cannabis	ATS	Inhalant	Sedative	Hallucinogens	Opioids	Kratom
4717	HSA0150	Male	Chinese	2020	38	Single	Secondary	Self-employed	Urban	Yes	No	Yes	No	Yes	No	No	No	No	No
4718	HSA0151	Male	Indian	2020	57	Married	No formal education	Unemployed	Urban	Yes	No	Yes	No	Yes	No	No	No	No	No
4719	HSA0152	Male	Others	2021	31	Married	No formal education	Government	Urban	No	Yes	No	No	Yes	No	No	No	No	No
4720	HSA0154	Male	Malay	2021	39	Others	Secondary	Self-employed	Urban	Yes	Yes	No	Yes	Yes	No	No	No	No	No
4721	HSA0155	Male	Chinese	2019	30	Married	Secondary	Self-employed	Urban	Yes	Yes	Yes	No	No	No	No	No	No	No
4722	HSA0156	Male	Indian	2018	60	Married	No formal education	Unemployed	Urban	Yes	Yes	Yes	No	No	No	No	No	No	No
4723	HSA0158	Male	Malay	2021	20	Single	Tertiary	Unemployed	Urban	Yes	No	No	Yes	No	No	No	No	No	No
4724	HSA0159	Male	Malay	2021	25	Single	Tertiary	Private	Urban	Yes	No	Yes	Yes	Yes	No	No	No	No	No
4725	HSA0160	Male	Indian	2020	60	Married	No formal education	Private	Urban	No	Yes	Yes	No	No	No	No	No	No	No
4726	HSA0161	Male	Indian	2020	62	Married	Primary	Private	Urban	Yes	No	Yes	No	No	No	No	No	No	No
4727	HSA0162	Male	Indian	2021	26	Single	Secondary	Private	Urban	Yes	Yes	Yes	No	No	No	No	No	No	No
4728	HSA0163	Male	Malay	2020	42	Married	No formal education	Self-employed	Urban	Yes	Yes	No	Yes	Yes	No	No	No	No	No
4729	HSA0164	Male	Chinese	2020	22	Single	Secondary	Private	Urban	Yes	No	Yes	No	No	No	No	No	No	No
4730	HSA0165	Male	Others	2020	39	Others	No formal education	Unemployed	Urban	Yes	No	Yes	No	No	No	No	No	No	No
4731	HSA0166	Male	Indian	2020	25	Single	Secondary	Private	Rural	No	Yes	No	No	No	No	No	No	No	Yes
4732	HSA0167	Male	Malay	2020	31	Married	Tertiary	Private	Urban	Yes	Yes	No	Yes	No	No	No	No	No	No
4733	HSA0168	Female	Chinese	2021	39	Married	Secondary	Private	Urban	No	No	Yes	No	No	No	No	No	No	No
4734	HSA0169	Male	Malay	2021	31	Single	Secondary	Unemployed	Urban	Yes	Yes	No	Yes	Yes	No	No	No	No	No
4735	HSA0170	Male	Malay	2021	37	Married	No formal education	Self-employed	Urban	Yes	Yes	No	No	Yes	No	No	No	No	No
4736	HSA0171	Male	Malay	2020	41	Married	Secondary	Government	Urban	Yes	Yes	No	No	Yes	No	No	No	No	No
4737	HSA0172	Male	Malay	2020	34	Others	Secondary	Unemployed	Urban	Yes	No	No	No	Yes	No	No	No	No	Yes
4738	HSA0173	Male	Malay	2019	19	Single	Secondary	Others	Urban	No	No	No	Yes	Yes	No	No	No	No	No
4739	HSA0174	Male	Malay	2020	26	Single	Tertiary	Unemployed	Rural	Yes	No	Yes	No	Yes	No	No	No	No	No
4740	HSA0175	Male	Chinese	2020	71	Married	No formal education	Unemployed	Urban	Yes	No	Yes	No	No	No	No	No	No	No
4741	HSA0176	Male	Indian	2020	23	Single	Secondary	Private	Urban	Yes	No	Yes	No	No	No	No	No	No	No
4742	HSA0177	Male	Malay	2020	30	Married	No formal education	Private	Rural	Yes	No	Yes	Yes	Yes	Yes	No	No	Yes	No
4743	HSA0178	Male	Chinese	2020	44	Married	No formal education	Self-employed	Urban	Yes	Yes	Yes	No	No	No	No	No	No	No
4744	HSA0179	Male	Malay	2020	22	Married	Tertiary	Unemployed	Urban	Yes	No	No	No	No	No	No	No	No	Yes
4745	HSA0180	Male	Chinese	2020	44	Married	No formal education	Private	Urban	Yes	Yes	Yes	No	No	No	No	No	No	No
4746	HSA0181	Male	Chinese	2021	60	Married	Secondary	Private	Urban	Yes	No	Yes	No	No	No	No	No	No	No
4747	HSA0182	Male	Malay	2021	36	Others	No formal education	Private	Rural	Yes	No	No	No	Yes	No	No	No	No	No

No	Patient ID	Gender	Ethnic group	Year of diagnosis	Age	Marital status	Education level	Occupation	Place of residence	History of psy illness	Tobacco	Alcohol	Cannabis	ATS	Inhalant	Sedative	Hallucinogens	Opioids	Kratom
4748	HSA0183	Male	Chinese	2019	58	Married	Primary	Unemployed	Urban	Yes	No	Yes	No	No	No	No	No	No	No
4749	HSA0184	Female	Others	2019	37	Married	Secondary	Unemployed	Urban	Yes	No	Yes	No	No	No	No	No	No	No
4750	HSA0185	Male	Chinese	2019	51	Married	No formal education	Private	Urban	No	No	Yes	No	No	No	No	No	No	No
4751	HSA0187	Male	Indian	2019	62	Married	Primary	Private	Urban	Yes	No	Yes	No	No	No	No	No	No	No
4752	HSA0188	Male	Malay	2020	61	Married	Tertiary	Unemployed	Urban	Yes	Yes	No	No	No	No	No	No	No	Yes
4753	HSA0189	Male	Malay	2020	25	Single	Secondary	Private	Urban	Yes	Yes	Yes	No	Yes	No	No	No	No	No
4754	HSA0190	Male	Indian	2021	58	Married	Primary	Unemployed	Urban	No	No	Yes	No	No	No	No	No	No	No
4755	HSA0191	Male	Malay	2021	36	Married	Secondary	Unemployed	Rural	No	Yes	No	Yes	No	No	No	No	No	No
4756	HSA0192	Male	Indian	2018	21	Single	Secondary	Others	Urban	Yes	No	Yes	Yes	No	No	No	No	No	No
4757	HSA0193	Male	Indian	2021	28	Single	Secondary	Private	Urban	Yes	No	Yes	Yes	No	No	No	No	No	No
4758	HSA0194	Male	Others	2021	36	Married	Secondary	Others	Urban	Yes	No	Yes	No	Yes	No	No	No	No	No
4759	HSA0195	Male	Chinese	2021	40	Married	No formal education	Private	Urban	Yes	No	Yes	No	No	No	No	No	No	No
4760	HSA0196	Male	Malay	2020	22	Single	Secondary	Others	Urban	Yes	Yes	Yes	No	No	No	No	No	No	No
4761	HSA0197	Male	Chinese	2021	50	Single	No formal education	Unemployed	Urban	Yes	No	Yes	No	No	No	No	No	No	No
4762	HSA0198	Male	Malay	2021	27	Married	No formal education	Government	Urban	No	No	No	No	Yes	No	No	No	No	No
4763	HSA0199	Male	Indian	2019	56	Single	No formal education	Government	Urban	Yes	No	Yes	No	No	No	No	No	Yes	No
4764	HSA0200	Male	Malay	2020	20	Single	No formal education	Private	Rural	Yes	Yes	Yes	No	Yes	No	No	No	No	No
4765	HSA0201	Male	Malay	2021	28	Single	Secondary	Private	Rural	Yes	No	Yes	No	No	No	No	No	No	No
4766	HSA0202	Male	Chinese	2020	56	Married	Primary	Unemployed	Urban	Yes	Yes	Yes	No	No	No	No	No	No	No
4767	HSA0203	Male	Malay	2019	26	Single	Secondary	Unemployed	Urban	Yes	Yes	No	No	Yes	No	No	No	No	No
4768	HSA0204	Male	Chinese	2018	47	Single	No formal education	Private	Urban	No	Yes	Yes	No	No	No	No	No	No	No
4769	HSA0207	Male	Indian	2021	57	Married	No formal education	Unemployed	Urban	No	Yes	Yes	No	No	No	No	No	No	No
4770	HSA0208	Female	Others	2020	23	Single	Tertiary	Private	Urban	Yes	Yes	Yes	No	No	No	No	No	No	No
4771	HSA0209	Male	Indian	2021	24	Single	No formal education	Unemployed	Urban	Yes	Yes	Yes	No	No	No	No	No	No	No
4772	HSA0210	Female	Malay	2020	31	Married	Tertiary	Private	Rural	Yes	Yes	Yes	No	No	No	No	No	No	No
4773	HSA0211	Female	Malay	2021	23	Single	Secondary	Private	Urban	Yes	Yes	Yes	No	No	No	No	No	No	No
4774	HSA0212	Female	Chinese	2020	36	Others	Secondary	Private	Urban	Yes	No	Yes	No	No	No	No	No	No	No
4775	HSA0213	Female	Malay	2020	33	Married	No formal education	Unemployed	Rural	Yes	No	No	No	Yes	No	No	No	No	No
4776	HSA0214	Female	Indian	2021	34	Married	No formal education	Unemployed	Urban	Yes	No	No	No	Yes	No	No	No	No	No
4777	HSA0215	Female	Malay	2021	24	Single	Secondary	Private	Urban	Yes	Yes	No	Yes	Yes	No	No	No	No	No
4778	HSA0216	Female	Malay	2021	21	Married	Secondary	Unemployed	Rural	Yes	Yes	Yes	No	No	No	No	No	No	No
4779	HSA0217	Female	Chinese	2020	69	Married	No formal education	Unemployed	Urban	Yes	No	Yes	No	No	No	No	No	No	No

No	Patient ID	Gender	Ethnic group	Year of diagnosis	Age	Marital status	Education level	Occupation	Place of residence	History of psy illness	Tobacco	Alcohol	Cannabis	ATS	Inhalant	Sedative	Hallucinogens	Opioids	Kratom
4780	HSA0218	Female	Malay	2021	24	Single	Tertiary	Unemployed	Urban	Yes	Yes	No	Yes	No	No	No	No	No	No
4781	HSA0219	Female	Malay	2020	26	Single	Secondary	Private	Urban	Yes	No	No	No	Yes	No	No	No	No	No
4782	HSA0220	Female	Malay	2020	21	Single	Tertiary	Unemployed	Urban	Yes	Yes	Yes	No	No	No	No	No	No	No
4783	HSA0221	Female	Malay	2020	24	Single	Tertiary	Private	Rural	Yes	Yes	Yes	Yes	Yes	No	No	No	No	No
4784	HSA0222	Female	Chinese	2020	29	Married	No formal education	Unemployed	Urban	Yes	Yes	Yes	No	No	No	No	No	No	No
4785	HSA0223	Female	Chinese	2019	43	Married	No formal education	Unemployed	Urban	Yes	No	Yes	No	No	No	No	No	No	No
4786	HSA0224	Female	Malay	2020	25	Single	Tertiary	Unemployed	Urban	Yes	No	No	Yes	No	No	No	No	No	No
4787	HSA0225	Female	Malay	2020	34	Married	No formal education	Unemployed	Urban	Yes	No	No	No	Yes	No	No	No	No	No
4788	HSA0226	Female	Malay	2020	26	Others	Primary	Unemployed	Rural	Yes	No	Yes	No	Yes	No	No	No	No	No
4789	HSA0227	Female	Indian	2019	25	Single	Tertiary	Private	Urban	Yes	No	Yes	No	No	No	No	No	No	No
4790	HSA0228	Female	Chinese	2019	44	Married	No formal education	Unemployed	Urban	Yes	No	No	No	Yes	No	No	No	No	No
4791	HSA0230	Female	Malay	2020	20	Single	Secondary	Unemployed	Urban	Yes	No	Yes	No	Yes	No	No	No	No	No
4792	HSA0231	Female	Chinese	2019	28	Single	Tertiary	Government	Urban	Yes	No	Yes	No	No	No	No	No	No	No
4793	HSA0232	Female	Malay	2020	36	Single	Tertiary	Private	Rural	Yes	Yes	Yes	No	No	No	No	No	No	No
4794	HSA0233	Female	Chinese	2020	51	Others	No formal education	Unemployed	Urban	Yes	Yes	Yes	No	No	No	No	No	No	No
4795	HSA0234	Female	Chinese	2019	69	Others	Tertiary	Unemployed	Urban	Yes	No	Yes	No	No	No	No	No	No	No
4796	HSA0235	Female	Chinese	2019	43	Others	Secondary	Private	Urban	Yes	No	Yes	No	No	No	No	No	No	No
4797	HSA0236	Female	Malay	2020	19	Single	No formal education	Private	Rural	No	No	Yes	No	Yes	No	No	No	No	No
4798	HSA0237	Female	Others	2020	24	Others	Secondary	Unemployed	Rural	Yes	No	No	No	No	No	No	No	No	No
4799	HSA0238	Female	Chinese	2019	21	Single	Secondary	Private	Rural	Yes	No	Yes	No	No	No	No	No	No	No
4800	HSA0239	Female	Chinese	2019	57	Others	No formal education	Private	Urban	Yes	No	Yes	No	No	No	No	No	No	No
4801	HSA0240	Female	Chinese	2019	37	Married	Tertiary	Private	Urban	Yes	Yes	Yes	No	No	No	No	No	No	No
4802	HSA0241	Female	Indian	2020	26	Married	Tertiary	Government	Urban	Yes	No	Yes	No	No	No	No	No	No	No
4803	HSA0242	Female	Malay	2018	23	Single	Tertiary	Unemployed	Urban	Yes	No	No	Yes	No	No	No	No	No	No
4804	HSA0243	Female	Malay	2021	30	Married	No formal education	Unemployed	Urban	Yes	Yes	No	No	Yes	No	No	No	No	No
4805	HSA0244	Female	Indian	2019	24	Single	Tertiary	Private	Urban	Yes	Yes	Yes	No	No	No	No	No	No	No
4806	HSA0245	Female	Malay	2019	27	Single	Secondary	Unemployed	Urban	Yes	Yes	No	No	Yes	No	No	No	No	No
4807	HSA0246	Female	Indian	2020	59	Others	Primary	Unemployed	Urban	Yes	No	Yes	No	No	No	No	No	No	No
4808	HSA0247	Female	Chinese	2020	59	Others	Primary	Unemployed	Urban	No	No	Yes	No	No	No	No	No	No	No
4809	HSA0248	Female	Malay	2021	22	Married	No formal education	Private	Rural	Yes	No	No	No	No	No	No	No	No	No
4810	HSA0249	Female	Chinese	2020	14	Single	Secondary	Unemployed	Urban	Yes	No	Yes	No	No	No	No	No	No	No
4811	HSA0250	Female	Malay	2021	17	Single	Secondary	Private	Urban	Yes	Yes	No	No	Yes	No	No	No	No	No
4812	HSA0251	Female	Malay	2021	30	Married	No formal education	Unemployed	Urban	Yes	No	No	No	No	No	No	No	No	No
4813	HSA0252	Female	Malay	2019	20	Single	Tertiary	Unemployed	Urban	Yes	Yes	Yes	Yes	Yes	No	No	No	No	No

No	Patient ID	Gender	Ethnic group	Year of diagnosis	Age	Marital status	Education level	Occupation	Place of residence	History of psy illness	Tobacco	Alcohol	Cannabis	ATS	Inhalant	Sedative	Hallucinogens	Opioids	Kratom
4814	HSA0253	Female	Chinese	2020	28	Single	Tertiary	Unemployed	Urban	Yes	No	Yes	No	No	No	No	No	No	No
4815	HSA0254	Female	Chinese	2019	37	Others	Primary	Self-employed	Urban	Yes	Yes	Yes	No	No	No	No	No	No	No
4816	HSA0255	Female	Chinese	2020	40	Others	Secondary	Private	Urban	Yes	Yes	No	No	Yes	No	No	No	No	No
4817	HSA0256	Female	Indian	2020	28	Single	Secondary	Private	Urban	Yes	Yes	Yes	No	No	No	No	No	No	No
4818	HSA0257	Female	Malay	2020	37	Single	Secondary	Unemployed	Urban	Yes	No	No	No	Yes	No	No	No	No	No
4819	HSA0258	Female	Malay	2021	30	Married	No formal education	Unemployed	Rural	Yes	Yes	No	No	Yes	No	No	No	No	No
4820	HSA0259	Female	Others	2021	33	Others	No formal education	Unemployed	Urban	Yes	No	No	Yes	Yes	No	No	No	No	No
4821	HSA0260	Female	Malay	2020	33	Others	Primary	Unemployed	Urban	Yes	Yes	Yes	No	No	No	No	No	No	No
4822	HSA0261	Female	Chinese	2020	33	Single	Primary	Unemployed	Urban	Yes	Yes	No	No	Yes	No	No	No	No	No
4823	HSA0262	Female	Others	2021	22	Single	Tertiary	Government	Urban	Yes	Yes	Yes	No	No	No	No	No	No	No
4824	HSA0263	Female	Chinese	2021	42	Others	No formal education	Others	Urban	Yes	No	No	No	Yes	No	No	No	No	No
4825	HSA0264	Female	Chinese	2018	51	Married	Secondary	Unemployed	Urban	Yes	Yes	Yes	No	No	No	No	No	No	No
4826	HSA0265	Female	Malay	2021	35	Single	Tertiary	Unemployed	Urban	Yes	No	No	No	Yes	No	No	No	No	No
4827	HSA0266	Female	Malay	2020	19	Single	Primary	Private	Urban	Yes	Yes	Yes	No	No	No	No	No	No	No
4828	HSA0267	Female	Malay	2021	24	Single	Tertiary	Unemployed	Urban	Yes	No	Yes	No	No	No	No	No	No	No
4829	HSA0268	Female	Chinese	2021	21	Single	Secondary	Unemployed	Urban	Yes	Yes	Yes	Yes	No	No	No	No	No	No
4830	HSA0269	Female	Chinese	2018	42	Single	No formal education	Private	Urban	Yes	No	Yes	No	No	No	No	No	No	No
4831	HSA0270	Female	Chinese	2019	42	Others	No formal education	Self-employed	Urban	Yes	Yes	Yes	No	No	No	No	No	No	No
4832	HSA0271	Female	Malay	2020	47	Single	Secondary	Unemployed	Urban	Yes	No	Yes	No	No	No	No	No	No	No
4833	HSA0272	Female	Chinese	2021	38	Married	Tertiary	Private	Rural	Yes	No	Yes	No	No	No	No	No	No	No
4834	HSA0273	Female	Chinese	2020	43	Others	Primary	Unemployed	Urban	Yes	No	Yes	No	No	No	No	No	No	No
4835	HSA0274	Female	Malay	2020	26	Single	Secondary	Private	Urban	Yes	Yes	No	No	Yes	No	No	No	No	No
4836	HSA0275	Female	Malay	2020	37	Single	No formal education	Unemployed	Rural	Yes	No	No	No	Yes	No	No	No	No	No
4837	HSA0276	Female	Others	2020	20	Single	Secondary	Private	Urban	Yes	Yes	Yes	No	No	No	No	No	No	No
4838	HSA0277	Female	Chinese	2019	14	Single	Primary	Unemployed	Urban	Yes	No	Yes	No	No	No	No	No	No	No
4839	HSA0278	Male	Malay	2018	40	Single	No formal education	Private	Urban	Yes	No	No	Yes	Yes	No	No	No	No	No
4840	HSA0279	Male	Malay	2018	34	Married	Tertiary	Unemployed	Urban	Yes	Yes	No	No	Yes	No	No	No	No	No
4841	HSA0280	Male	Malay	2018	33	Single	No formal education	Unemployed	Urban	Yes	No	No	No	No	No	No	No	Yes	No
4842	HSA0281	Male	Indian	2018	41	Married	No formal education	Others	Urban	Yes	No	Yes	No	No	No	No	No	No	No
4843	HSA0282	Male	Malay	2018	31	Single	No formal education	Others	Urban	Yes	No	Yes	Yes	Yes	No	No	No	Yes	No
4844	HSA0283	Female	Chinese	2018	29	Married	No formal education	Unemployed	Urban	Yes	Yes	No	No	No	No	No	No	No	No
4845	HSA0284	Male	Malay	2018	31	Others	No formal education	Private	Rural	Yes	No	No	No	Yes	No	No	No	No	No

No	Patient ID	Gender	Ethnic group	Year of diagnosis	Age	Marital status	Education level	Occupation	Place of residence	History of psy illness	Tobacco	Alcohol	Cannabis	ATS	Inhalant	Sedative	Hallucinogens	Opioids	Kratom
4846	HSA0285	Male	Indian	2018	20	Single	Primary	Unemployed	Urban	Yes	Yes	Yes	No	No	No	No	No	No	No
4847	HSA0286	Male	Malay	2018	38	Married	No formal education	Unemployed	Rural	No	No	No	Yes	Yes	No	No	No	No	No
4848	HSA0287	Male	Malay	2018	53	Married	No formal education	Private	Rural	Yes	Yes	No	No	No	No	No	No	Yes	No
4849	HSA0288	Male	Malay	2018	30	Single	Secondary	Private	Urban	Yes	Yes	No	No	Yes	No	No	No	No	No
4850	HSA0289	Male	Others	2018	31	Single	No formal education	Private	Urban	No	Yes	No	No	Yes	No	No	No	No	No
4851	HSA0290	Male	Malay	2018	34	Others	No formal education	Private	Rural	Yes	No	No	Yes	Yes	No	No	No	No	No
4852	HSA0291	Female	Malay	2018	35	Married	No formal education	Unemployed	Urban	Yes	No	No	No	No	No	No	No	No	No
4853	HSA0292	Male	Malay	2018	29	Single	Primary	Unemployed	Urban	Yes	No	No	No	Yes	No	No	No	Yes	No
4854	HSA0293	Male	Others	2018	22	Married	No formal education	Others	Urban	Yes	No	Yes	Yes	No	No	No	No	No	No
4855	HSA0294	Male	Malay	2018	34	Single	Primary	Private	Rural	Yes	No	No	Yes	Yes	No	No	No	No	No
4856	HSA0295	Male	Indian	2018	62	Married	No formal education	Private	Urban	Yes	No	Yes	No	No	No	No	No	No	No
4857	HSA0296	Male	Malay	2018	25	Single	No formal education	Others	Urban	Yes	No	No	Yes	Yes	No	No	No	Yes	No
4858	HSA0297	Male	Malay	2018	23	Single	Tertiary	Others	Urban	No	No	No	No	Yes	No	No	No	No	No
4859	HSA0298	Male	Malay	2018	38	Others	Secondary	Others	Urban	Yes	No	No	No	Yes	No	No	No	Yes	No
4860	HSA0299	Male	Malay	2018	45	Married	No formal education	Others	Urban	Yes	Yes	No	No	Yes	No	No	No	Yes	No
4861	HSA0300	Male	Malay	2018	32	Single	No formal education	Private	Urban	Yes	No	No	No	Yes	No	No	No	No	No
4862	HSA0301	Male	Malay	2018	34	Single	No formal education	Private	Urban	Yes	No	No	No	No	No	No	No	Yes	No
4863	HSA0302	Male	Malay	2018	34	Single	No formal education	Others	Urban	Yes	No	No	No	Yes	No	No	No	No	No
4864	HSA0303	Male	Malay	2018	46	Married	No formal education	Private	Urban	Yes	Yes	No	Yes	Yes	No	No	No	No	No
4865	HSA0304	Male	Malay	2018	28	Married	Primary	Others	Urban	Yes	No	No	No	Yes	No	No	No	No	No
4866	HSA0305	Male	Indian	2018	40	Married	Primary	Private	Urban	Yes	Yes	Yes	No	No	No	No	No	No	No
4867	HSA0306	Male	Malay	2018	32	Single	No formal education	Others	Urban	Yes	No	Yes	Yes	No	No	No	No	No	No
4868	HSA0307	Male	Malay	2018	41	Married	No formal education	Unemployed	Urban	Yes	No	No	No	Yes	No	No	No	No	No
4869	HSA0308	Male	Indian	2018	53	Married	Primary	Private	Urban	Yes	Yes	Yes	No	No	No	No	No	No	No
4870	HSA0309	Male	Malay	2018	24	Married	No formal education	Private	Urban	Yes	No	Yes	No	No	No	No	No	No	No
4871	HSA0310	Male	Malay	2018	20	Single	No formal education	Others	Urban	Yes	No	No	No	Yes	No	No	No	No	No

No	Patient ID	Gender	Ethnic group	Year of diagnosis	Age	Marital status	Education level	Occupation	Place of residence	History of psy illness	Tobacco	Alcohol	Cannabis	ATS	Inhalant	Sedative	Hallucinogens	Opioids	Kratom
4872	HSA0311	Male	Malay	2018	57	Others	No formal education	Unemployed	Urban	Yes	No	No	No	No	No	No	No	Yes	No
4873	HSA0312	Male	Indian	2018	38	Married	No formal education	Others	Urban	Yes	No	Yes	No	No	No	No	No	No	No
4874	HSA0313	Female	Others	2018	33	Married	No formal education	Others	Urban	Yes	No	No	Yes	Yes	No	No	No	No	No
4875	HSA0314	Male	Indian	2018	20	Single	Primary	Private	Urban	Yes	No	Yes	No	No	No	No	No	No	No
4876	HSA0315	Male	Malay	2018	35	Single	Secondary	Others	Urban	Yes	Yes	No	No	No	No	No	No	Yes	No
4877	HSA0316	Male	Malay	2018	38	Married	Tertiary	Private	Rural	Yes	Yes	No	No	No	No	No	No	Yes	No
4878	HSA0317	Male	Others	2018	28	Others	No formal education	Others	Urban	Yes	No	Yes	No	No	No	No	No	No	No
4879	HSA0318	Male	Malay	2018	23	Single	Tertiary	Unemployed	Urban	Yes	No	No	Yes	No	No	No	No	No	No
4880	HSA0319	Male	Indian	2018	22	Single	No formal education	Others	Urban	Yes	No	Yes	No	No	No	No	No	No	No
4881	HSA0320	Male	Malay	2018	77	Married	No formal education	Others	Urban	Yes	No	Yes	No	No	No	No	No	No	No
4882	HSA0321	Female	Indian	2018	44	Married	Secondary	Private	Urban	Yes	No	Yes	No	No	No	No	No	No	No
4883	HSA0322	Male	Malay	2018	26	Others	Secondary	Others	Urban	Yes	Yes	No	No	Yes	No	No	No	No	No
4884	HSA0323	Male	Chinese	2018	37	Others	No formal education	Others	Urban	Yes	Yes	No	No	Yes	No	No	No	No	No
4885	HSA0324	Male	Malay	2018	47	Married	Primary	Private	Urban	Yes	No	No	No	No	No	No	No	No	Yes
4886	HSA0325	Male	Indian	2018	36	Single	Secondary	Private	Urban	Yes	Yes	Yes	No	No	No	No	No	No	No
4887	HSA0326	Male	Malay	2018	38	Married	No formal education	Self-employed	Urban	Yes	No	No	No	Yes	No	No	No	No	No
4888	HSA0327	Male	Chinese	2018	39	Others	Secondary	Private	Urban	Yes	Yes	Yes	No	Yes	No	No	No	No	No
4889	HSA0328	Male	Malay	2018	46	Others	No formal education	Others	Urban	Yes	No	No	No	Yes	No	No	No	No	No
4890	HSA0329	Male	Chinese	2018	40	Single	Secondary	Private	Urban	Yes	Yes	Yes	No	No	No	No	No	No	No
4891	HSA0330	Female	Malay	2018	32	Married	No formal education	Unemployed	Rural	No	No	No	No	Yes	No	No	No	Yes	No
4892	HSA0331	Female	Chinese	2018	28	Others	Tertiary	Unemployed	Urban	Yes	Yes	Yes	No	No	No	No	No	No	No
4893	HSA0332	Female	Others	2018	20	Others	No formal education	Private	Urban	Yes	No	No	No	Yes	No	No	No	No	No
4894	HSA0333	Female	Chinese	2018	22	Single	Secondary	Unemployed	Urban	No	Yes	Yes	No	No	No	No	No	No	No
4895	HSA0334	Male	Indian	2018	22	Single	Primary	Others	Urban	Yes	No	No	Yes	Yes	No	No	No	No	No
4896	HSA0335	Female	Chinese	2018	46	Others	No formal education	Unemployed	Urban	Yes	Yes	Yes	No	Yes	No	No	No	No	No
4897	HSA0336	Male	Malay	2018	44	Married	No formal education	Others	Urban	Yes	Yes	No	No	No	No	No	No	Yes	No
4898	HSA0337	Male	Malay	2018	49	Others	No formal education	Unemployed	Rural	Yes	No	No	No	No	No	No	No	Yes	No
4899	HSA0338	Male	Indian	2018	42	Married	No formal education	Self-employed	Urban	Yes	Yes	Yes	No	No	No	No	No	No	No
4900	HSA0339	Male	Chinese	2018	43	Married	Secondary	Private	Urban	Yes	Yes	Yes	No	No	No	No	No	No	No

No	Patient ID	Gender	Ethnic group	Year of diagnosis	Age	Marital status	Education level	Occupation	Place of residence	History of psy illness	Tobacco	Alcohol	Cannabis	ATS	Inhalant	Sedative	Hallucinogens	Opioids	Kratom
4901	HSA0340	Female	Malay	2018	23	Others	Secondary	Self-employed	Urban	Yes	No	No	No	Yes	No	No	No	No	No
4902	HSA0341	Female	Chinese	2018	30	Single	No formal education	Private	Urban	Yes	Yes	No	No	Yes	No	No	No	No	No
4903	HSA0342	Female	Others	2018	27	Others	No formal education	Others	Urban	Yes	No	No	No	Yes	No	No	No	No	No
4904	HSA0343	Female	Indian	2018	55	Others	No formal education	Unemployed	Urban	Yes	Yes	Yes	No	No	No	No	No	Yes	No
4905	HSA0344	Male	Others	2018	17	Single	Secondary	Unemployed	Urban	Yes	No	Yes	No	Yes	No	No	No	No	No
4906	HSA0345	Female	Chinese	2018	34	Others	Secondary	Others	Urban	Yes	Yes	Yes	No	No	No	No	No	No	No
4907	HSA0346	Female	Malay	2018	29	Others	No formal education	Unemployed	Rural	Yes	No	No	No	Yes	No	No	No	No	No
4908	HSA0347	Female	Chinese	2018	36	Single	Secondary	Unemployed	Urban	Yes	No	Yes	No	No	No	No	No	No	No
4909	HSA0348	Female	Malay	2018	27	Others	No formal education	Unemployed	Urban	Yes	Yes	No	No	Yes	No	No	No	No	No
4910	HSA0349	Male	Indian	2018	49	Single	No formal education	Unemployed	Urban	Yes	No	Yes	No	No	No	No	No	No	No
4911	HSA0350	Male	Indian	2018	26	Others	No formal education	Unemployed	Urban	No	No	Yes	No	No	No	No	No	No	No
4912	HSA0351	Male	Indian	2018	32	Married	Secondary	Others	Urban	Yes	Yes	Yes	No	No	No	No	No	No	No
4913	HSA0352	Female	Malay	2018	30	Married	No formal education	Unemployed	Urban	Yes	No	Yes	Yes	Yes	No	No	No	No	No
4914	HSA0353	Male	Malay	2018	38	Married	No formal education	Others	Urban	Yes	No	No	No	Yes	No	No	No	No	No
4915	HSA0354	Female	Malay	2018	27	Single	Secondary	Private	Urban	Yes	Yes	No	No	Yes	No	No	No	No	No
4916	HSA0355	Male	Indian	2018	39	Married	Secondary	Unemployed	Urban	Yes	No	No	No	No	No	No	No	No	No
4917	HSA0356	Female	Malay	2018	16	Single	Secondary	Others	Urban	Yes	Yes	Yes	No	Yes	No	No	No	No	No
4918	HSA0357	Male	Others	2018	36	Single	No formal education	Private	Urban	Yes	No	Yes	No	No	No	No	No	No	No
4919	HSA0358	Female	Others	2018	20	Others	No formal education	Private	Urban	Yes	No	No	No	Yes	No	No	No	No	No
4920	HSA0359	Male	Malay	2018	45	Married	Tertiary	Private	Urban	Yes	No	No	Yes	Yes	No	No	No	No	No
4921	HSA0360	Female	Malay	2018	31	Others	No formal education	Others	Rural	Yes	No	No	No	Yes	No	No	No	No	No
4922	HSA0361	Female	Chinese	2018	48	Others	No formal education	Self-employed	Urban	Yes	Yes	Yes	No	No	No	No	No	No	No
4923	HSA0362	Male	Malay	2018	23	Single	Tertiary	Private	Urban	No	Yes	No	No	No	No	No	No	No	No
4924	HSA0363	Female	Others	2018	26	Others	No formal education	Others	Urban	Yes	No	No	No	Yes	No	No	No	No	No
4925	HSA0364	Female	Malay	2018	19	Single	Secondary	Private	Urban	Yes	Yes	Yes	No	Yes	No	No	No	No	No
4926	HSA0365	Male	Indian	2018	56	Others	No formal education	Unemployed	Urban	Yes	No	No	No	No	No	No	No	Yes	No
4927	HSA0366	Male	Chinese	2018	28	Others	No formal education	Self-employed	Urban	Yes	Yes	No	No	Yes	No	No	No	No	No
4928	HSA0367	Male	Indian	2018	30	Married	Secondary	Unemployed	Urban	Yes	Yes	Yes	No	Yes	No	No	No	No	No

No	Patient ID	Gender	Ethnic group	Year of diagnosis	Age	Marital status	Education level	Occupation	Place of residence	History of psy illness	Tobacco	Alcohol	Cannabis	ATS	Inhalant	Sedative	Hallucinogens	Opioids	Kratom
4929	HSA0368	Female	Malay	2018	13	Single	Secondary	Unemployed	Urban	No	No	No	No	Yes	No	No	No	No	No
4930	HSA0369	Male	Malay	2018	48	Others	No formal education	Others	Rural	Yes	No	No	Yes	No	No	No	No	No	No
4931	HSA0370	Male	Malay	2018	40	Single	Secondary	Unemployed	Rural	No	No	No	No	Yes	No	No	No	No	No
4932	HSA0371	Male	Malay	2018	53	Single	No formal education	Unemployed	Urban	Yes	Yes	No	No	No	No	No	No	No	No
4933	HSA0372	Male	Malay	2018	47	Single	No formal education	Unemployed	Urban	Yes	No	No	No	Yes	No	No	No	Yes	No
4934	HSA0373	Male	Malay	2018	54	Married	No formal education	Unemployed	Urban	Yes	No	No	No	Yes	No	No	No	Yes	No
4935	HSA0374	Male	Malay	2018	35	Single	No formal education	Unemployed	Urban	Yes	No	No	No	Yes	No	No	No	No	No
4936	HSA0375	Male	Chinese	2018	39	Married	Secondary	Unemployed	Urban	Yes	No	No	No	Yes	No	No	No	No	No
4937	HSA0376	Male	Indian	2018	24	Single	Secondary	Unemployed	Urban	Yes	No	Yes	No	Yes	No	No	No	No	No
4938	HSA0377	Male	Malay	2018	40	Others	No formal education	Private	Rural	Yes	No	Yes	No	No	No	No	No	No	No
4939	HSA0378	Male	Malay	2018	53	Single	Secondary	Unemployed	Urban	Yes	Yes	No	No	No	No	No	No	No	No
4940	HSA0379	Male	Indian	2018	38	Others	Secondary	Unemployed	Urban	Yes	Yes	No	No	No	No	No	No	Yes	No
4941	HSA0380	Male	Chinese	2019	63	Married	Primary	Self-employed	Urban	No	No	No	No	No	No	No	No	Yes	No
4942	HSA0381	Female	Indian	2018	33	Others	No formal education	Unemployed	Urban	Yes	No	No	Yes	Yes	No	No	No	Yes	No
4943	HSA0382	Male	Indian	2018	25	Others	No formal education	Others	Urban	Yes	No	No	Yes	No	No	No	No	No	No
4944	HSA0383	Male	Malay	2018	58	Married	No formal education	Government	Urban	Yes	No	No	No	Yes	No	No	No	Yes	No
4945	HSA0384	Male	Chinese	2018	43	Others	No formal education	Others	Urban	Yes	No	No	No	Yes	No	No	No	No	No
4946	HSA0385	Male	Chinese	2018	61	Single	No formal education	Private	Urban	Yes	No	No	No	No	No	No	No	Yes	No
4947	HSA0386	Male	Indian	2018	43	Married	Secondary	Private	Urban	Yes	No	Yes	No	No	No	No	No	No	No
4948	HSA0387	Female	Chinese	2018	62	Married	No formal education	Private	Urban	No	No	No	No	No	No	No	No	No	No
4949	HSA0388	Male	Chinese	2018	34	Others	Secondary	Private	Urban	No	No	Yes	No	Yes	No	No	No	No	No
4950	HSA0389	Male	Chinese	2018	62	Married	No formal education	Others	Urban	Yes	No	Yes	No	No	No	No	No	Yes	No
4951	HSA0390	Female	Chinese	2018	25	Others	Secondary	Private	Urban	Yes	Yes	Yes	No	Yes	No	No	No	No	No
4952	HSA0391	Male	Indian	2018	22	Others	No formal education	Others	Urban	Yes	No	Yes	No	Yes	No	No	No	No	No
4953	HSA0392	Male	Indian	2018	30	Single	Secondary	Private	Urban	Yes	Yes	Yes	No	Yes	No	No	No	No	No
4954	HSA0393	Female	Others	2018	26	Others	Secondary	Private	Urban	Yes	Yes	Yes	No	Yes	No	No	No	No	No
4955	HSA0394	Male	Malay	2018	41	Married	No formal education	Private	Urban	Yes	No	No	No	Yes	No	No	No	No	No
4956	HSA0395	Female	Chinese	2018	29	Others	No formal education	Unemployed	Urban	Yes	No	No	No	Yes	No	No	No	No	No

No	Patient ID	Gender	Ethnic group	Year of diagnosis	Age	Marital status	Education level	Occupation	Place of residence	History of psy illness	Tobacco	Alcohol	Cannabis	ATS	Inhalant	Sedative	Hallucinogens	Opioids	Kratom
4957	HSA0396	Male	Chinese	2018	31	Others	No formal education	Private	Urban	Yes	No	No	No	Yes	No	No	No	No	No
4958	HSA0397	Female	Others	2018	23	Single	Primary	Others	Urban	Yes	No	Yes	No	Yes	No	No	No	No	No
4959	HSA0398	Male	Malay	2018	29	Others	No formal education	Unemployed	Urban	Yes	No	No	No	Yes	No	No	No	Yes	No
4960	HSA0399	Female	Chinese	2018	19	Others	No formal education	Private	Urban	Yes	No	No	No	Yes	No	No	No	No	No
4961	HSA0400	Male	Malay	2018	25	Single	Secondary	Unemployed	Rural	Yes	Yes	No	No	Yes	No	No	No	Yes	No
4962	HSA0401	Male	Chinese	2018	36	Others	Secondary	Unemployed	Urban	Yes	Yes	Yes	No	Yes	No	No	No	Yes	No
4963	HSA0403	Male	Chinese	2018	34	Married	No formal education	Private	Urban	Yes	Yes	No	No	Yes	No	No	No	No	No
4964	HSA0404	Male	Malay	2018	46	Single	Primary	Unemployed	Rural	Yes	Yes	Yes	No	No	No	No	No	Yes	No
4965	HSA0405	Male	Others	2018	54	Single	No formal education	Unemployed	Urban	Yes	No	No	No	Yes	No	No	No	Yes	No
4966	HSA0406	Male	Chinese	2018	54	Single	Primary	Private	Urban	Yes	Yes	No	No	No	No	No	No	Yes	No
4967	HSA0407	Male	Chinese	2018	29	Single	No formal education	Others	Urban	Yes	No	No	No	Yes	No	No	No	Yes	No
4968	HSA0408	Male	Malay	2018	33	Married	Secondary	Government	Urban	Yes	No	No	No	Yes	No	No	No	No	No
4969	HSA0409	Male	Malay	2019	40	Single	Secondary	Private	Rural	Yes	Yes	No	No	Yes	No	No	No	Yes	No
4970	HSA0410	Male	Malay	2018	40	Married	No formal education	Private	Urban	Yes	No	No	No	Yes	No	No	No	Yes	No
4971	HSA0411	Male	Malay	2019	31	Single	Secondary	Unemployed	Urban	Yes	Yes	No	No	Yes	No	No	No	No	No
4972	HSA0412	Female	Malay	2018	39	Others	No formal education	Others	Rural	Yes	Yes	No	No	No	No	No	No	No	No
4973	HSA0413	Male	Malay	2019	40	Single	Primary	Private	Urban	Yes	Yes	Yes	No	Yes	No	No	No	No	No
4974	HSA0414	Male	Malay	2018	54	Married	Tertiary	Private	Urban	Yes	Yes	No	No	No	No	No	No	No	No
4975	HSA0415	Male	Malay	2019	20	Single	Secondary	Private	Urban	Yes	Yes	No	No	Yes	No	No	No	No	No
4976	HSA0416	Male	Malay	2018	48	Others	No formal education	Unemployed	Rural	Yes	No	No	No	No	No	No	No	Yes	No
4977	HSA0417	Male	Malay	2019	26	Single	Secondary	Unemployed	Urban	Yes	Yes	No	Yes	Yes	No	No	No	No	No
4978	HSA0418	Female	Malay	2019	17	Single	Secondary	Unemployed	Urban	No	No	No	No	Yes	No	No	No	No	No
4979	HSA0419	Male	Malay	2019	29	Single	No formal education	Private	Rural	Yes	Yes	No	No	Yes	No	No	No	No	No
4980	HSA0420	Male	Chinese	2019	22	Others	Secondary	Private	Urban	Yes	Yes	Yes	No	No	No	No	No	No	No
4981	HSA0421	Male	Malay	2019	31	Married	No formal education	Private	Urban	Yes	No	No	No	Yes	No	No	No	No	No
4982	HSA0422	Male	Malay	2019	48	Single	No formal education	Others	Rural	Yes	Yes	No	No	Yes	No	No	No	Yes	No
4983	HSA0423	Male	Malay	2019	20	Single	Tertiary	Unemployed	Urban	Yes	Yes	No	No	Yes	No	No	No	No	No
4984	HSA0424	Male	Chinese	2019	59	Married	Secondary	Others	Urban	Yes	Yes	No	No	No	No	No	No	Yes	No
4985	HSA0425	Male	Malay	2019	35	Others	Tertiary	Private	Urban	Yes	No	No	No	Yes	No	No	No	No	No
4986	HSA0426	Female	Chinese	2019	31	Others	Secondary	Unemployed	Urban	Yes	No	No	Yes	No	No	No	No	No	No
4987	HSA0427	Male	Malay	2019	24	Single	Secondary	Unemployed	Urban	Yes	Yes	No	No	No	No	No	No	No	No
4988	HSA0428	Male	Chinese	2019	37	Married	Secondary	Private	Urban	Yes	Yes	No	No	Yes	No	No	No	No	No

No	Patient ID	Gender	Ethnic group	Year of diagnosis	Age	Marital status	Education level	Occupation	Place of residence	History of psy illness	Tobacco	Alcohol	Cannabis	ATS	Inhalant	Sedative	Hallucinogens	Opioids	Kratom
4989	HSA0429	Male	Malay	2019	19	Single	Secondary	Private	Urban	Yes	Yes	No	No	Yes	No	No	No	No	No
4990	HSA0430	Male	Malay	2019	41	Single	No formal education	Self-employed	Urban	Yes	No	Yes	No	No	No	No	No	No	No
4991	HSA0431	Male	Malay	2019	32	Others	No formal education	Others	Urban	No	No	No	Yes	No	No	No	No	No	Yes
4992	HSA0432	Female	Chinese	2019	34	Others	Secondary	Private	Urban	Yes	Yes	No	No	Yes	No	No	No	No	No
4993	HSA0433	Male	Indian	2019	36	Others	No formal education	Others	Urban	Yes	No	No	No	Yes	No	No	No	Yes	No
4994	HSA0434	Male	Chinese	2019	34	Others	No formal education	Unemployed	Urban	Yes	No	Yes	No	Yes	No	No	No	Yes	No
4995	HSA0435	Male	Malay	2019	37	Others	Secondary	Private	Rural	No	Yes	Yes	Yes	No	No	No	No	No	No
4996	HSA0436	Male	Chinese	2019	27	Others	Secondary	Private	Urban	No	No	Yes	No	No	No	No	No	No	No
4997	HSA0437	Male	Malay	2019	30	Married	Secondary	Private	Urban	Yes	No	No	No	Yes	No	No	No	Yes	No
4998	HSA0438	Female	Chinese	2019	29	Others	Secondary	Others	Urban	Yes	No	No	No	Yes	No	No	No	No	No
4999	HSA0439	Male	Malay	2019	29	Single	Secondary	Unemployed	Urban	Yes	No	No	No	Yes	No	No	No	Yes	No
5000	HSA0440	Female	Chinese	2019	24	Others	Secondary	Others	Urban	Yes	No	Yes	Yes	No	No	No	No	No	No
5001	HSA0441	Male	Malay	2019	26	Single	Secondary	Unemployed	Rural	Yes	No	No	Yes	No	No	No	No	No	No
5002	HSA0442	Male	Malay	2019	27	Single	Primary	Others	Urban	Yes	Yes	No	No	Yes	No	No	No	No	No
5003	HSA0443	Male	Malay	2019	27	Single	Tertiary	Private	Urban	Yes	No	No	No	Yes	No	No	No	No	No
5004	HSA0444	Male	Others	2019	45	Single	Primary	Private	Urban	Yes	Yes	No	No	Yes	No	No	No	No	No
5005	HSA0445	Male	Malay	2019	36	Others	No formal education	Unemployed	Rural	Yes	No	No	No	No	No	No	No	Yes	No
5006	HSA0446	Male	Chinese	2019	31	Single	No formal education	Unemployed	Urban	Yes	No	No	No	Yes	No	No	No	No	No
5007	HSA0447	Male	Malay	2019	21	Single	Secondary	Unemployed	Urban	Yes	Yes	No	No	Yes	No	No	No	No	No
5008	HSA0448	Female	Chinese	2019	28	Others	No formal education	Unemployed	Urban	Yes	Yes	No	No	Yes	No	No	No	No	No
5009	HSA0449	Male	Malay	2019	18	Single	Tertiary	Unemployed	Urban	Yes	Yes	No	No	No	No	No	No	No	No
5010	HSA0450	Female	Malay	2019	36	Married	No formal education	Others	Urban	Yes	No	No	No	Yes	No	No	No	No	No
5011	HSA0451	Male	Malay	2019	47	Single	No formal education	Unemployed	Urban	Yes	No	Yes	No	No	No	No	No	Yes	No
5012	HSA0452	Female	Others	2019	32	Married	No formal education	Others	Rural	Yes	No	No	No	Yes	No	No	No	No	No
5013	HSA0453	Male	Malay	2019	39	Single	No formal education	Unemployed	Urban	Yes	Yes	No	No	No	No	No	No	No	No
5014	HSA0454	Male	Malay	2019	40	Married	No formal education	Others	Urban	Yes	No	No	No	No	No	No	No	Yes	No
5015	HSA0455	Male	Malay	2019	42	Others	No formal education	Unemployed	Urban	Yes	Yes	No	No	No	No	No	No	Yes	No
5016	HSA0456	Female	Malay	2019	40	Others	Secondary	Unemployed	Urban	Yes	Yes	No	No	No	No	No	No	Yes	No
5017	HSA0457	Male	Malay	2019	48	Others	No formal education	Unemployed	Urban	Yes	No	No	Yes	No	No	No	No	No	No

No	Patient ID	Gender	Ethnic group	Year of diagnosis	Age	Marital status	Education level	Occupation	Place of residence	History of psy illness	Tobacco	Alcohol	Cannabis	ATS	Inhalant	Sedative	Hallucinogens	Opioids	Kratom
5018	HSA0458	Female	Malay	2019	31	Married	No formal education	Others	Urban	No	Yes	No	No	No	No	No	No	No	No
5019	HSA0459	Male	Malay	2019	63	Married	Tertiary	Unemployed	Urban	Yes	Yes	No	Yes	Yes	No	No	No	Yes	No
5020	HSA0460	Male	Chinese	2019	41	Others	No formal education	Others	Rural	Yes	No	No	No	Yes	No	No	No	Yes	No
5021	HSA0461	Female	Malay	2019	34	Married	No formal education	Others	Urban	Yes	Yes	No	No	Yes	No	No	No	No	No
5022	HSA0463	Male	Malay	2019	41	Single	Secondary	Private	Urban	No	Yes	No	No	No	No	No	No	Yes	No
5023	HSA0463	Male	Indian	2019	36	Others	Secondary	Private	Urban	No	Yes	Yes	No	No	No	No	No	No	No
5024	HSA0464	Male	Malay	2019	28	Single	Secondary	Unemployed	Rural	Yes	Yes	No	No	No	No	No	No	No	No
5025	HSA0465	Male	Others	2019	38	Married	No formal education	Private	Urban	Yes	No	Yes	Yes	No	No	No	No	No	No
5026	HSA0466	Female	Malay	2019	30	Others	Tertiary	Unemployed	Urban	Yes	No	No	No	Yes	No	No	No	No	No
5027	HSA0467	Male	Malay	2019	42	Others	Secondary	Unemployed	Urban	Yes	Yes	No	No	Yes	No	No	No	No	No
5028	HSA0468	Male	Chinese	2019	63	Married	Primary	Self-employed	Urban	Yes	Yes	Yes	No	No	No	No	No	No	No
5029	HSA0469	Female	Others	2019	35	Married	No formal education	Others	Urban	Yes	No	No	No	Yes	No	No	No	No	No
5030	HSA0470	Male	Chinese	2019	39	Married	Secondary	Others	Urban	Yes	Yes	No	Yes	Yes	No	No	No	No	No
5031	HSA0471	Male	Malay	2019	26	Single	Secondary	Others	Urban	Yes	No	No	Yes	No	No	No	No	No	No
5032	HSA0472	Male	Others	2019	36	Married	Secondary	Private	Urban	Yes	Yes	No	No	No	No	No	No	No	No
5033	HSA0473	Male	Malay	2019	18	Others	Secondary	Unemployed	Urban	Yes	No	No	Yes	Yes	No	No	No	No	No
5034	HSA0474	Male	Others	2019	21	Single	Tertiary	Unemployed	Urban	Yes	No	Yes	No	No	No	No	No	No	No
5035	HSA0475	Male	Malay	2019	34	Others	Secondary	Unemployed	Rural	Yes	Yes	No	Yes	Yes	No	No	No	No	No
5036	HSA0476	Male	Malay	2019	36	Single	Secondary	Private	Urban	Yes	Yes	Yes	Yes	No	No	No	No	No	No
5037	HSA0477	Female	Malay	2019	32	Married	Secondary	Unemployed	Urban	Yes	Yes	No	No	No	No	No	No	Yes	No
5038	HSA0478	Female	Malay	2019	39	Married	Secondary	Unemployed	Urban	Yes	No	No	No	No	No	No	No	Yes	No
5039	HSA0479	Male	Chinese	2019	29	Single	Secondary	Unemployed	Urban	Yes	Yes	No	No	Yes	No	No	No	No	No
5040	HSA0480	Male	Indian	2019	39	Married	Secondary	Unemployed	Urban	Yes	Yes	No	No	Yes	No	No	No	Yes	No
5041	HSA0481	Female	Malay	2019	34	Married	Tertiary	Private	Urban	Yes	No	Yes	No	No	No	No	No	No	No
5042	HSA0482	Female	Malay	2019	28	Others	No formal education	Others	Urban	Yes	Yes	No	No	Yes	No	No	No	No	No
5043	HSA0483	Female	Malay	2019	30	Single	Secondary	Unemployed	Rural	Yes	No	No	No	Yes	No	No	No	No	No
5044	HSA0484	Male	Indian	2019	53	Others	Secondary	Private	Urban	Yes	No	Yes	No	Yes	No	No	No	No	No
5045	HSA0485	Female	Malay	2019	35	Others	Tertiary	Unemployed	Urban	Yes	Yes	No	No	Yes	No	No	No	No	No
5046	HSA0486	Male	Malay	2019	51	Single	Secondary	Unemployed	Urban	No	No	No	No	Yes	No	No	No	Yes	No
5047	HSA0487	Female	Malay	2019	26	Others	Secondary	Others	Rural	No	No	No	No	Yes	No	No	No	No	No
5048	HSA0488	Female	Malay	2019	26	Others	Secondary	Unemployed	Rural	Yes	Yes	No	No	Yes	No	No	No	No	No
5049	HSA0489	Female	Malay	2019	24	Married	No formal education	Unemployed	Urban	No	Yes	No	No	No	No	No	No	No	No
5050	HSA0490	Male	Malay	2019	62	Married	No formal education	Self-employed	Rural	No	Yes	No	No	No	No	No	No	Yes	No
5051	HSA0491	Male	Malay	2019	31	Single	Secondary	Unemployed	Urban	Yes	No	No	No	Yes	No	No	No	No	No
5052	HSA0492	Female	Malay	2019	20	Single	Tertiary	Unemployed	Urban	Yes	No	Yes	Yes	Yes	No	No	No	No	No
5053	HSA0493	Female	Malay	2019	19	Single	Secondary	Others	Urban	Yes	No	No	No	Yes	No	No	No	No	No

No	Patient ID	Gender	Ethnic group	Year of diagnosis	Age	Marital status	Education level	Occupation	Place of residence	History of psy illness	Tobacco	Alcohol	Cannabis	ATS	Inhalant	Sedative	Hallucinogens	Opioids	Kratom
5054	HSA0494	Male	Indian	2019	36	Others	Secondary	Others	Urban	Yes	Yes	Yes	No	No	No	No	No	No	No
5055	HSA0495	Female	Malay	2019	23	Married	No formal education	Others	Urban	Yes	No	No	No	Yes	No	No	No	No	No
5056	HSA0496	Male	Malay	2019	37	Others	Secondary	Unemployed	Rural	Yes	Yes	No	No	Yes	No	No	No	No	No
5057	HSA0497	Male	Chinese	2019	29	Single	No formal education	Unemployed	Urban	Yes	Yes	Yes	No	Yes	No	No	No	No	No
5058	HSA0498	Female	Indian	2019	24	Single	Secondary	Unemployed	Urban	Yes	Yes	Yes	No	No	No	No	No	No	No
5059	HSA0499	Female	Others	2019	33	Married	No formal education	Unemployed	Urban	Yes	Yes	Yes	No	Yes	No	No	No	No	No
5060	HSA0500	Female	Malay	2019	35	Others	No formal education	Unemployed	Urban	Yes	Yes	No	No	Yes	No	No	No	No	No
5061	HSA0501	Female	Malay	2019	24	Single	No formal education	Private	Urban	Yes	No	Yes	No	No	No	No	No	No	No
5062	HSA0502	Male	Indian	2019	44	Others	Primary	Unemployed	Urban	No	Yes	Yes	No	No	No	No	No	No	No
5063	HSA0503	Male	Chinese	2019	32	Others	Secondary	Private	Urban	Yes	Yes	No	No	No	No	No	No	No	No
5064	HSA0504	Male	Indian	2019	37	Married	No formal education	Private	Urban	No	Yes	Yes	No	No	No	No	No	No	No
5065	HSA0505	Male	Indian	2019	39	Others	Secondary	Private	Urban	Yes	No	Yes	No	No	No	No	No	No	No
5066	HSA0506	Female	Others	2019	23	Others	No formal education	Others	Urban	No	Yes	No	No	Yes	No	No	No	No	No
5067	HSA0507	Male	Indian	2019	30	Married	Secondary	Private	Urban	Yes	Yes	Yes	No	No	No	No	No	No	No
5068	HSA0508	Male	Malay	2019	53	Others	No formal education	Self-employed	Urban	No	Yes	No	No	Yes	No	No	No	No	No
5069	HSA0509	Male	Chinese	2019	44	Others	Secondary	Private	Urban	Yes	Yes	Yes	No	No	No	No	No	No	No
5070	HSA0510	Male	Chinese	2019	25	Single	Secondary	Private	Urban	Yes	No	No	No	Yes	No	No	No	No	No
5071	HSA0511	Male	Malay	2019	54	Others	No formal education	Others	Urban	Yes	Yes	No	No	No	No	No	No	Yes	No
5072	HSA0512	Female	Malay	2019	25	Others	Secondary	Private	Urban	No	Yes	No	No	No	No	No	No	No	No
5073	HSA0513	Male	Indian	2019	29	Married	No formal education	Private	Urban	Yes	No	Yes	No	No	No	No	No	No	No
5074	HSA0514	Male	Indian	2019	38	Others	No formal education	Others	Urban	No	Yes	No	No	No	No	No	No	No	No
5075	HSA0515	Male	Chinese	2019	38	Others	No formal education	Self-employed	Urban	Yes	No	Yes	No	No	No	No	No	No	No
5076	HSA0516	Female	Malay	2019	39	Others	No formal education	Unemployed	Rural	Yes	Yes	No	No	No	No	No	No	No	No
5077	HSA0517	Male	Malay	2019	40	Single	No formal education	Unemployed	Urban	No	No	No	No	Yes	No	No	No	No	No
5078	HSA0518	Male	Indian	2019	33	Others	No formal education	Private	Urban	No	Yes	No	Yes	Yes	No	No	No	No	No
5079	HSA0519	Male	Malay	2019	50	Married	Primary	Private	Urban	No	No	No	No	Yes	No	No	No	No	No
5080	HSA0520	Male	Indian	2019	76	Others	Primary	Unemployed	Urban	Yes	No	Yes	No	No	No	No	No	No	No
5081	HSA0521	Male	Malay	2019	28	Single	Secondary	Unemployed	Urban	Yes	No	Yes	No	No	No	No	No	No	No

No	Patient ID	Gender	Ethnic group	Year of diagnosis	Age	Marital status	Education level	Occupation	Place of residence	History of psy illness	Tobacco	Alcohol	Cannabis	ATS	Inhalant	Sedative	Hallucinogens	Opioids	Kratom
5082	HSA0522	Male	Indian	2019	34	Others	No formal education	Others	Urban	Yes	Yes	Yes	No	No	No	No	No	No	No
5083	HSA0523	Female	Indian	2019	49	Married	No formal education	Unemployed	Urban	Yes	Yes	No	No	No	No	No	No	No	No
5084	HSA0524	Male	Chinese	2019	64	Others	No formal education	Unemployed	Urban	No	Yes	Yes	No	Yes	No	No	No	No	No
5085	HSA0525	Female	Others	2019	40	Married	No formal education	Private	Urban	Yes	Yes	Yes	No	No	No	No	No	No	No
5086	HSA0526	Male	Chinese	2019	52	Others	No formal education	Others	Urban	Yes	Yes	Yes	No	No	No	No	No	No	No
5087	HSA0527	Female	Indian	2019	25	Others	No formal education	Private	Urban	No	Yes	No	No	No	No	No	No	No	No
5088	HSA0528	Male	Chinese	2019	28	Single	Secondary	Unemployed	Urban	Yes	Yes	No	No	Yes	No	No	Yes	No	No
5089	HSA0529	Female	Malay	2019	33	Others	No formal education	Private	Urban	Yes	Yes	No	No	Yes	No	No	No	No	No
5090	HSA0530	Male	Indian	2019	26	Single	Tertiary	Private	Urban	Yes	No	Yes	No	No	No	No	No	No	No
5091	HSA0531	Female	Malay	2019	39	Married	No formal education	Others	Urban	Yes	Yes	No	No	No	No	No	No	No	No
5092	HSA0532	Male	Chinese	2019	37	Married	Secondary	Private	Urban	Yes	No	Yes	No	No	No	No	No	No	No
5093	HSA0533	Male	Malay	2019	56	Others	No formal education	Unemployed	Urban	No	Yes	No	No	No	No	No	No	No	No
5094	HSA0534	Male	Chinese	2019	45	Married	No formal education	Private	Urban	Yes	Yes	Yes	No	No	No	No	No	No	No
5095	HSA0535	Male	Indian	2019	41	Others	No formal education	Others	Urban	Yes	No	No	No	Yes	No	No	No	No	No
5096	HSA0536	Male	Chinese	2019	77	Others	No formal education	Unemployed	Urban	No	No	Yes	No	No	No	No	No	No	No
5097	HSA0537	Male	Indian	2019	37	Single	Secondary	Unemployed	Urban	Yes	Yes	Yes	No	No	No	No	No	No	No
5098	HSA0538	Male	Others	2019	35	Others	Secondary	Private	Urban	Yes	No	No	No	Yes	No	No	No	No	No
5099	HSA0539	Male	Indian	2019	58	Others	No formal education	Unemployed	Urban	Yes	Yes	No	No	No	No	No	No	No	No
5100	HSA0540	Male	Indian	2019	21	Single	Secondary	Unemployed	Urban	Yes	Yes	Yes	No	No	No	No	No	No	No
5101	HSA0541	Male	Indian	2019	37	Married	Secondary	Self-employed	Urban	Yes	No	Yes	No	No	No	No	No	No	No
5102	HSA0542	Male	Indian	2019	28	Single	Secondary	Private	Urban	Yes	Yes	Yes	No	No	No	No	No	No	No
5103	HSA0543	Male	Indian	2019	57	Married	Secondary	Unemployed	Urban	Yes	No	Yes	No	No	No	No	No	No	No
5104	HSA0544	Male	Chinese	2019	33	Single	No formal education	Unemployed	Urban	Yes	No	No	No	Yes	No	No	No	No	No
5105	HSA0545	Male	Malay	2019	60	Others	No formal education	Unemployed	Rural	Yes	Yes	No	No	No	No	No	No	No	No
5106	HSA0546	Female	Indian	2019	20	Married	Secondary	Others	Urban	Yes	No	Yes	No	No	No	No	No	No	No
5107	HSA0547	Male	Others	2019	35	Single	No formal education	Unemployed	Urban	Yes	Yes	No	No	No	No	No	No	No	No
5108	HSA0548	Male	Indian	2019	27	Single	No formal education	Private	Urban	No	Yes	Yes	No	No	No	No	No	No	No

No	Patient ID	Gender	Ethnic group	Year of diagnosis	Age	Marital status	Education level	Occupation	Place of residence	History of psy illness	Tobacco	Alcohol	Cannabis	ATS	Inhalant	Sedative	Hallucinogens	Opioids	Kratom
5109	HSA0549	Male	Indian	2019	59	Married	No formal education	Private	Urban	Yes	Yes	Yes	No	No	No	No	No	No	No
5110	HSA0550	Male	Chinese	2019	35	Married	No formal education	Others	Urban	Yes	Yes	Yes	No	No	No	No	No	No	No
5111	HSA0551	Male	Indian	2019	44	Others	Secondary	Unemployed	Urban	Yes	No	Yes	No	No	No	No	No	No	No
5112	HSA0552	Female	Chinese	2019	39	Single	No formal education	Unemployed	Urban	Yes	Yes	No	No	No	No	No	No	No	No
5113	HSA0553	Female	Chinese	2019	33	Single	No formal education	Private	Urban	Yes	Yes	Yes	No	No	No	No	No	No	No
5114	HSA0554	Male	Malay	2019	34	Single	No formal education	Private	Urban	Yes	Yes	No	No	Yes	No	No	No	No	No
5115	HSA0555	Male	Indian	2019	28	Others	No formal education	Others	Urban	Yes	No	Yes	No	No	No	No	No	No	No
5117	HSA0556	Female	Malay	2019	25	Single	Secondary	Others	Rural	Yes	Yes	No	No	No	No	No	No	No	No
5116	HSA0556	Male	Chinese	2019	34	Others	No formal education	Others	Urban	Yes	No	No	No	Yes	No	No	No	Yes	No
5118	HSA0557	Male	Indian	2019	25	Others	Secondary	Unemployed	Urban	No	Yes	Yes	No	No	No	No	No	No	No
5119	HSA0558	Male	Chinese	2019	46	Others	Primary	Unemployed	Urban	Yes	Yes	No	No	No	No	No	No	No	No
5120	HSA0559	Male	Indian	2019	34	Married	Secondary	Unemployed	Urban	Yes	Yes	No	No	No	No	No	No	No	No
5121	HSA0560	Male	Others	2019	42	Married	No formal education	Unemployed	Urban	Yes	No	Yes	Yes	No	No	No	No	No	No
5122	HSA0561	Male	Indian	2019	51	Married	Secondary	Private	Urban	No	No	Yes	No	No	No	No	No	No	No
5123	HSA0562	Male	Chinese	2019	50	Others	No formal education	Others	Urban	Yes	No	No	No	Yes	No	No	No	No	No
5124	HSA0563	Male	Indian	2019	34	Others	Secondary	Private	Urban	Yes	No	Yes	Yes	No	No	No	No	No	No
5125	HSA0564	Male	Malay	2019	36	Single	No formal education	Private	Urban	Yes	No	No	No	Yes	No	No	No	Yes	No
5126	HSA0565	Male	Malay	2019	23	Single	Secondary	Others	Urban	Yes	Yes	No	No	Yes	No	No	No	No	No
5127	HSA0567	Male	Malay	2019	28	Married	Tertiary	Others	Urban	Yes	Yes	No	No	No	No	No	No	No	No
5128	HSA0568	Male	Malay	2019	59	Others	No formal education	Others	Rural	Yes	Yes	No	No	No	No	No	No	No	No
5129	HSA0569	Female	Malay	2020	31	Others	Secondary	Others	Urban	Yes	Yes	No	No	No	No	No	No	No	No
5130	HSA0570	Male	Malay	2019	45	Others	No formal education	Others	Urban	No	No	No	Yes	No	No	No	No	No	No
5131	HSA0571	Male	Indian	2020	66	Others	No formal education	Private	Urban	Yes	No	Yes	No	No	No	No	No	No	No
5132	HSA0572	Male	Indian	2019	23	Single	Tertiary	Private	Urban	Yes	Yes	Yes	No	No	No	No	No	No	No
5133	HSA0573	Male	Others	2020	37	Married	Primary	Private	Urban	Yes	Yes	Yes	No	No	No	No	No	No	No
5134	HSA0574	Male	Indian	2019	22	Single	No formal education	Others	Urban	Yes	Yes	Yes	No	No	No	No	No	No	Yes
5135	HSA0575	Male	Indian	2020	22	Single	Tertiary	Private	Urban	Yes	Yes	Yes	No	No	No	No	No	No	No
5136	HSA0576	Male	Malay	2020	52	Others	Primary	Self-employed	Urban	Yes	No	No	No	Yes	No	No	No	No	No
5137	HSA0577	Male	Indian	2020	62	Others	No formal education	Unemployed	Urban	Yes	Yes	Yes	No	No	No	No	No	No	No

No	Patient ID	Gender	Ethnic group	Year of diagnosis	Age	Marital status	Education level	Occupation	Place of residence	History of psy illness	Tobacco	Alcohol	Cannabis	ATS	Inhalant	Sedative	Hallucinogens	Opioids	Kratom
5138	HSA0578	Male	Malay	2020	22	Single	Tertiary	Self-employed	Rural	Yes	No	No	Yes	No	No	No	No	No	No
5139	HSA0579	Female	Indian	2020	36	Single	Secondary	Private	Urban	Yes	No	Yes	No	No	No	No	No	No	No
5140	HSA0580	Male	Indian	2020	23	Single	Primary	Private	Urban	No	Yes	No	No	No	No	No	No	No	No
5141	HSA0582	Male	Malay	2020	66	Married	No formal education	Unemployed	Urban	Yes	Yes	No	No	No	No	No	No	No	No
5142	HSA0583	Male	Malay	2020	35	Single	No formal education	Private	Urban	Yes	No	No	No	No	No	No	No	Yes	No
5143	HSA0584	Male	Indian	2020	15	Single	Secondary	Unemployed	Urban	Yes	No	Yes	No	No	No	No	No	No	No
5144	HSA0585	Female	Others	2020	25	Single	No formal education	Private	Urban	Yes	No	No	No	No	No	No	No	No	No
5145	HSA0586	Male	Malay	2020	51	Married	Secondary	Private	Urban	Yes	Yes	No	No	No	No	No	No	No	No
5146	HSA0587	Male	Chinese	2020	30	Married	Primary	Private	Urban	Yes	Yes	No	No	Yes	No	No	No	No	No
5147	HSA0588	Male	Indian	2020	42	Single	Secondary	Unemployed	Urban	No	No	Yes	No	No	No	No	No	No	No
5148	HSA0589	Female	Indian	2020	42	Married	No formal education	Unemployed	Urban	Yes	Yes	Yes	No	No	No	No	No	No	No
5149	HSA0590	Male	Malay	2020	43	Single	Secondary	Private	Urban	Yes	Yes	No	No	No	No	No	No	Yes	Yes
5150	HSA0591	Male	Chinese	2020	44	Single	Secondary	Private	Urban	Yes	Yes	Yes	No	Yes	No	No	No	No	No
5151	HSA0592	Male	Malay	2020	23	Single	No formal education	Unemployed	Rural	Yes	Yes	No	No	No	No	No	No	No	No
5152	HSA0593	Male	Chinese	2020	46	Others	No formal education	Others	Urban	No	No	Yes	No	Yes	No	No	No	No	No
5153	HSA0594	Male	Malay	2020	38	Others	Secondary	Unemployed	Rural	Yes	No	Yes	No	No	No	No	No	No	No
5154	HSA0595	Female	Chinese	2020	29	Others	Tertiary	Private	Urban	Yes	Yes	Yes	No	No	No	No	No	No	No
5155	HSA0597	Male	Chinese	2020	40	Others	Secondary	Private	Urban	Yes	Yes	No	No	No	No	No	No	No	No
5156	HSA0598	Male	Malay	2020	17	Single	Secondary	Unemployed	Rural	Yes	Yes	No	No	No	No	No	No	No	No
5157	HSA0599	Female	Chinese	2020	31	Single	Secondary	Unemployed	Urban	No	Yes	No	No	No	No	No	No	No	No
5158	HSA0600	Male	Malay	2020	48	Married	Secondary	Private	Rural	Yes	Yes	No	No	No	No	No	No	No	No
5159	HSA0601	Male	Chinese	2020	60	Married	No formal education	Private	Urban	Yes	Yes	No	No	No	No	No	No	No	No
5160	HSA0602	Male	Malay	2020	62	Others	No formal education	Private	Urban	Yes	Yes	No	No	No	No	No	No	No	No
5161	HSA0603	Female	Chinese	2020	37	Single	No formal education	Unemployed	Urban	Yes	Yes	No	No	No	No	No	No	No	No
5162	HSA0604	Male	Others	2020	34	Single	Secondary	Others	Rural	Yes	No	Yes	No	No	No	No	No	No	No
5163	HSA0605	Female	Chinese	2020	64	Others	Primary	Others	Urban	Yes	No	Yes	No	No	No	No	No	No	No
5164	HSA0606	Female	Others	2020	23	Others	Secondary	Unemployed	Urban	Yes	No	No	No	Yes	No	No	No	No	No
5165	HSA0607	Male	Chinese	2020	55	Single	Secondary	Private	Urban	Yes	Yes	Yes	No	Yes	No	No	No	Yes	No
5166	HSA0608	Female	Others	2020	35	Married	No formal education	Unemployed	Urban	Yes	No	No	No	Yes	No	No	No	No	No
5167	HSA0609	Male	Chinese	2020	45	Others	No formal education	Others	Urban	Yes	Yes	Yes	No	No	No	No	No	No	No
5168	HSA0610	Male	Malay	2020	44	Married	Tertiary	Private	Urban	Yes	Yes	No	No	No	No	No	No	No	No
5169	HSA0611	Female	Chinese	2020	37	Married	Primary	Self-employed	Urban	Yes	Yes	Yes	No	No	No	No	No	No	No

No	Patient ID	Gender	Ethnic group	Year of diagnosis	Age	Marital status	Education level	Occupation	Place of residence	History of psy illness	Tobacco	Alcohol	Cannabis	ATS	Inhalant	Sedative	Hallucinogens	Opioids	Kratom
5170	HSA0612	Male	Malay	2020	28	Single	No formal education	Self-employed	Rural	Yes	Yes	No	Yes	Yes	No	No	No	No	No
5171	HSA0613	Male	Chinese	2020	34	Others	Secondary	Private	Urban	Yes	Yes	Yes	No	No	No	No	No	No	No
5172	HSA0614	Male	Indian	2020	21	Married	Secondary	Private	Urban	Yes	Yes	Yes	No	No	No	No	No	No	No
5173	HSA0615	Male	Chinese	2020	68	Married	Secondary	Unemployed	Urban	Yes	Yes	No	No	No	No	No	No	No	No
5174	HSA0616	Male	Indian	2020	31	Others	Secondary	Others	Urban	Yes	No	No	No	Yes	No	No	No	Yes	No
5175	HSA0617	Female	Chinese	2020	32	Others	No formal education	Private	Urban	Yes	Yes	Yes	No	Yes	No	No	No	No	No
5176	HSA0618	Male	Malay	2020	34	Married	Secondary	Others	Urban	No	Yes	No	No	No	No	No	No	No	No
5177	HSA0619	Male	Others	2020	71	Married	No formal education	Others	Urban	Yes	Yes	Yes	No	No	No	No	No	No	No
5178	HSA0620	Male	Malay	2020	17	Single	Primary	Unemployed	Urban	Yes	Yes	No	No	No	No	No	No	No	No
5179	HSA0621	Male	Indian	2020	39	Others	No formal education	Unemployed	Urban	Yes	Yes	Yes	No	Yes	No	No	No	No	No
5180	HSA0622	Male	Malay	2020	42	Single	Primary	Private	Urban	Yes	Yes	No	No	Yes	No	No	No	Yes	No
5181	HSA0623	Female	Malay	2020	23	Others	Secondary	Private	Urban	Yes	No	No	Yes	Yes	No	No	No	Yes	No
5182	HSA0624	Male	Malay	2020	37	Single	No formal education	Self-employed	Rural	Yes	No	No	No	Yes	No	No	No	No	No
5183	HSA0625	Male	Indian	2020	36	Married	No formal education	Private	Urban	Yes	No	Yes	No	No	No	No	No	No	No
5184	HSA0626	Male	Malay	2020	22	Married	Tertiary	Private	Urban	Yes	Yes	No	No	Yes	No	No	No	No	No
5185	HSA0627	Male	Indian	2020	33	Married	Tertiary	Self-employed	Urban	Yes	No	Yes	No	No	No	No	No	No	No
5186	HSA0628	Male	Chinese	2020	39	Others	No formal education	Others	Urban	Yes	No	No	No	Yes	No	No	No	No	No
5187	HSA0629	Female	Indian	2019	26	Others	Tertiary	Private	Urban	Yes	No	Yes	No	No	No	No	No	No	No
5188	HSA0630	Male	Malay	2020	32	Others	Secondary	Private	Rural	Yes	No	No	Yes	Yes	No	No	No	No	No
5189	HSA0632	Female	Malay	2020	29	Single	Secondary	Private	Urban	Yes	Yes	No	No	No	No	No	No	No	No
5190	HSA0633	Male	Indian	2020	24	Married	Secondary	Private	Urban	Yes	Yes	Yes	No	No	No	No	No	No	No
5191	HSA0634	Female	Malay	2020	28	Single	Secondary	Private	Urban	Yes	Yes	No	No	Yes	No	No	No	No	No
5192	HSA0635	Male	Indian	2020	21	Others	Secondary	Private	Urban	Yes	No	Yes	No	No	No	No	No	No	No
5193	HSA0636	Female	Others	2020	40	Others	No formal education	Unemployed	Urban	Yes	No	No	No	Yes	No	No	No	No	No
5194	HSA0637	Male	Malay	2020	33	Others	No formal education	Unemployed	Urban	Yes	No	Yes	No	No	No	No	No	No	No
5195	HSA0638	Male	Indian	2020	36	Married	Secondary	Private	Urban	No	No	Yes	No	No	No	No	No	No	No
5196	HSA0639	Male	Malay	2020	70	Married	Primary	Private	Urban	Yes	Yes	No	No	No	No	No	No	No	No
5197	HSA0640	Male	Indian	2020	46	Married	Primary	Private	Urban	No	Yes	Yes	No	No	No	No	No	No	No
5198	HSA0641	Female	Malay	2020	35	Others	Primary	Unemployed	Urban	No	Yes	Yes	No	Yes	No	No	No	Yes	No
5199	HSA0642	Male	Indian	2020	18	Single	Secondary	Unemployed	Urban	Yes	Yes	No	No	No	No	No	No	No	No
5200	HSA0643	Female	Malay	2020	37	Others	Secondary	Unemployed	Urban	Yes	No	Yes	No	Yes	No	No	No	No	No
5201	HSA0644	Male	Indian	2020	28	Married	Primary	Unemployed	Urban	Yes	Yes	No	No	No	No	No	No	No	No
5202	HSA0645	Male	Malay	2020	28	Others	Secondary	Unemployed	Urban	Yes	No	No	No	Yes	No	No	No	No	No
5203	HSA0646	Male	Chinese	2020	45	Others	Secondary	Unemployed	Urban	No	Yes	Yes	No	No	No	No	No	No	No

No	Patient ID	Gender	Ethnic group	Year of diagnosis	Age	Marital status	Education level	Occupation	Place of residence	History of psy illness	Tobacco	Alcohol	Cannabis	ATS	Inhalant	Sedative	Hallucinogens	Opioids	Kratom
5204	HSA0647	Male	Indian	2020	67	Others	No formal education	Others	Urban	Yes	No	Yes	No	No	No	No	No	No	No
5205	HSA0648	Male	Malay	2020	38	Single	No formal education	Unemployed	Urban	Yes	No	No	No	Yes	No	No	No	No	No
5206	HSA0649	Male	Others	2020	40	Married	No formal education	Others	Urban	Yes	No	No	No	Yes	No	No	No	No	No
5207	HSA0650	Female	Malay	2020	34	Others	Secondary	Unemployed	Rural	Yes	No	No	No	Yes	No	No	No	No	No
5208	HSA0651	Male	Chinese	2020	46	Single	Secondary	Private	Urban	Yes	Yes	Yes	No	No	No	No	No	No	No
5209	HSA0652	Male	Others	2020	39	Married	Secondary	Private	Urban	Yes	Yes	Yes	No	No	No	No	No	No	No
5210	HSA0653	Male	Others	2020	51	Others	Secondary	Private	Urban	No	No	Yes	No	No	No	No	No	No	No
5211	HSA0654	Male	Indian	2020	28	Married	Primary	Private	Urban	Yes	No	Yes	No	No	No	No	No	No	No
5212	HSA0655	Male	Chinese	2020	23	Single	Secondary	Private	Urban	Yes	Yes	Yes	No	No	No	No	No	No	No
5213	HSA0657	Female	Others	2020	33	Others	No formal education	Private	Urban	Yes	No	No	No	Yes	No	No	No	No	No
5214	HSA0658	Female	Malay	2020	31	Married	No formal education	Unemployed	Rural	Yes	Yes	No	No	No	No	No	No	No	No
5215	HSA0659	Female	Chinese	2020	39	Married	No formal education	Unemployed	Urban	Yes	Yes	No	No	No	No	No	No	No	No
5216	HSA0660	Male	Indian	2020	38	Married	No formal education	Private	Urban	No	Yes	Yes	No	No	No	No	No	No	No
5217	HSA0661	Female	Chinese	2020	21	Single	Secondary	Unemployed	Urban	Yes	Yes	No	No	Yes	No	No	No	No	No
5218	HSA0662	Male	Indian	2020	59	Married	Secondary	Unemployed	Urban	Yes	Yes	Yes	No	No	No	Yes	No	No	No
5219	HSA0663	Male	Chinese	2020	30	Single	Tertiary	Private	Urban	Yes	No	No	No	Yes	No	No	No	No	No
5220	HSA0664	Male	Chinese	2020	61	Single	No formal education	Unemployed	Urban	No	Yes	No	No	No	No	No	No	No	No
5221	HSA0665	Male	Chinese	2020	43	Married	No formal education	Private	Urban	Yes	Yes	No	No	No	No	No	No	No	No
5222	HSA0666	Female	Indian	2020	52	Married	No formal education	Unemployed	Urban	Yes	Yes	No	No	No	No	No	No	No	No
5223	HSA0667	Female	Chinese	2020	38	Married	Secondary	Unemployed	Urban	No	Yes	No	No	No	No	No	No	No	No
5224	HSA0668	Male	Indian	2020	44	Married	Secondary	Private	Urban	Yes	No	Yes	No	No	No	No	No	No	No
5225	HSA0669	Male	Indian	2020	56	Others	Primary	Private	Urban	Yes	No	Yes	No	No	No	No	No	No	No
5226	HSA0670	Female	Chinese	2020	37	Married	No formal education	Unemployed	Urban	Yes	Yes	No	No	No	No	No	No	No	No
5227	HSA0671	Male	Chinese	2020	66	Others	No formal education	Unemployed	Urban	Yes	Yes	No	No	Yes	No	No	No	Yes	No
5228	HSA0672	Female	Chinese	2020	41	Married	Secondary	Self-employed	Urban	No	Yes	Yes	No	No	No	No	No	No	No
5229	HSA0673	Male	Chinese	2020	39	Married	Secondary	Private	Urban	Yes	No	Yes	No	No	No	No	No	No	No
5230	HSA0674	Male	Malay	2020	43	Others	Secondary	Private	Urban	Yes	Yes	No	No	Yes	No	No	No	Yes	No
5231	HSA0675	Male	Chinese	2020	22	Others	Tertiary	Unemployed	Urban	Yes	Yes	Yes	No	No	No	No	No	No	No
5232	HSA0676	Male	Indian	2020	33	Married	No formal education	Others	Urban	Yes	No	Yes	No	No	No	No	No	No	No
5233	HSA0677	Female	Chinese	2020	42	Single	No formal education	Private	Urban	Yes	Yes	No	No	No	No	No	No	Yes	No

No	Patient ID	Gender	Ethnic group	Year of diagnosis	Age	Marital status	Education level	Occupation	Place of residence	History of psy illness	Tobacco	Alcohol	Cannabis	ATS	Inhalant	Sedative	Hallucinogens	Opioids	Kratom
5234	HSA0678	Female	Indian	2020	36	Single	Secondary	Private	Urban	Yes	Yes	Yes	No	No	No	No	No	No	No
5235	HSA0679	Male	Chinese	2020	26	Others	Tertiary	Private	Urban	Yes	Yes	Yes	No	No	No	No	No	No	No
5236	HSA0680	Male	Malay	2020	25	Others	Secondary	Private	Urban	Yes	Yes	No	No	No	No	No	No	No	No
5237	HSA0681	Male	Chinese	2020	55	Single	No formal education	Unemployed	Urban	Yes	Yes	Yes	No	No	No	No	No	No	No
5238	HSA0682	Male	Malay	2020	23	Single	No formal education	Others	Urban	Yes	No	No	No	Yes	No	No	No	No	No
5239	HSA0683	Female	Chinese	2020	25	Single	Tertiary	Private	Urban	Yes	No	Yes	No	No	No	No	No	No	No
5240	HSA0684	Male	Malay	2020	57	Married	Secondary	Unemployed	Urban	No	Yes	No	No	No	No	No	No	No	No
5241	HSA0685	Female	Chinese	2020	29	Single	Secondary	Unemployed	Urban	Yes	No	Yes	No	No	No	No	No	No	No
5242	HSA0686	Male	Malay	2020	53	Others	Secondary	Private	Urban	No	No	Yes	Yes	No	No	No	Yes	No	Yes
5243	HSA0687	Male	Chinese	2020	57	Married	Primary	Private	Urban	Yes	No	Yes	No	No	No	No	No	No	No
5244	HSA0688	Male	Malay	2020	40	Others	Secondary	Private	Urban	No	Yes	No	No	No	No	No	No	Yes	No
5245	HSA0689	Female	Indian	2020	60	Others	No formal education	Unemployed	Urban	Yes	No	Yes	No	Yes	No	No	No	No	No
5246	HSA0690	Male	Malay	2020	23	Single	Tertiary	Private	Urban	Yes	Yes	No	No	No	No	No	No	No	Yes
5247	HSA0691	Male	Chinese	2020	36	Married	Secondary	Private	Urban	Yes	Yes	Yes	No	No	No	No	No	No	No
5248	HSA0692	Male	Malay	2020	29	Single	No formal education	Private	Urban	Yes	Yes	No	No	Yes	No	No	No	No	No
5249	HSA0693	Female	Malay	2020	48	Married	No formal education	Unemployed	Urban	Yes	Yes	No	No	Yes	No	No	No	No	No
5250	HSA0694	Female	Others	2020	42	Married	No formal education	Private	Urban	Yes	Yes	Yes	No	No	No	No	No	No	No
5251	HSA0695	Male	Chinese	2020	40	Others	Tertiary	Others	Urban	Yes	No	Yes	No	No	No	No	No	Yes	No
5252	HSA0696	Male	Malay	2020	33	Married	No formal education	Unemployed	Urban	Yes	No	Yes	No	No	No	No	No	No	No
5253	HSA0697	Female	Chinese	2020	31	Others	Primary	Private	Urban	Yes	Yes	No	No	Yes	No	No	No	No	No
5254	HSA0698	Male	Indian	2020	38	Married	Primary	Self-employed	Urban	Yes	No	Yes	No	No	No	No	No	No	No
5255	HSA0699	Male	Chinese	2020	40	Married	Secondary	Private	Urban	Yes	Yes	Yes	Yes	Yes	No	No	No	No	No
5256	HSA0700	Male	Malay	2020	33	Others	No formal education	Government	Rural	No	Yes	No	No	No	No	No	No	No	No
5257	HSA0701	Male	Chinese	2020	38	Others	Tertiary	Private	Urban	Yes	No	No	No	Yes	No	No	No	No	No
5258	HSA0702	Male	Indian	2020	47	Married	No formal education	Others	Urban	Yes	Yes	No	No	Yes	No	No	No	Yes	No
5259	HSA0703	Male	Chinese	2020	67	Others	No formal education	Private	Urban	Yes	No	Yes	No	No	No	No	No	No	No
5260	HSA0704	Male	Malay	2020	26	Single	No formal education	Unemployed	Rural	Yes	Yes	No	No	Yes	No	No	No	No	No
5261	HSA0705	Male	Indian	2020	40	Single	Tertiary	Private	Urban	Yes	Yes	Yes	No	No	No	No	No	No	No
5262	HSA0706	Male	Chinese	2020	63	Single	Secondary	Private	Urban	Yes	Yes	Yes	Yes	Yes	No	No	No	No	No
5263	HSA0707	Male	Chinese	2020	57	Single	No formal education	Private	Urban	Yes	Yes	No	No	No	No	No	No	No	No
5264	HSA0708	Male	Malay	2020	19	Single	Tertiary	Unemployed	Urban	Yes	Yes	No	Yes	No	No	No	No	No	No
5265	HSA0709	Male	Chinese	2020	44	Married	Secondary	Self-employed	Urban	Yes	Yes	Yes	No	No	No	No	No	No	No

No	Patient ID	Gender	Ethnic group	Year of diagnosis	Age	Marital status	Education level	Occupation	Place of residence	History of psy illness	Tobacco	Alcohol	Cannabis	ATS	Inhalant	Sedative	Hallucinogens	Opioids	Kratom
5266	HSA0710	Male	Indian	2020	28	Married	No formal education	Private	Urban	Yes	Yes	No	No	No	No	No	No	Yes	No
5267	HSA0711	Male	Malay	2020	35	Single	No formal education	Others	Urban	Yes	No	No	No	Yes	No	No	No	No	No
5268	HSA0712	Male	Malay	2020	21	Others	Tertiary	Private	Rural	Yes	No	Yes	No	Yes	No	No	No	No	No
5269	HSA0713	Male	Malay	2020	40	Married	Secondary	Self-employed	Urban	No	Yes	No	No	No	No	No	No	No	No
5270	HSA0714	Male	Malay	2020	24	Single	Secondary	Private	Urban	Yes	Yes	No	No	No	No	No	No	No	No
5271	HSA0715	Male	Indian	2020	29	Others	No formal education	Unemployed	Urban	Yes	No	Yes	No	No	No	No	No	No	No
5272	HSA0716	Male	Malay	2020	39	Single	Secondary	Others	Rural	Yes	Yes	No	No	No	No	No	No	Yes	No
5273	HSA0717	Female	Malay	2020	38	Married	Primary	Unemployed	Urban	Yes	No	Yes	No	No	No	No	No	No	No
5274	HSA0718	Male	Malay	2020	31	Single	Secondary	Unemployed	Rural	Yes	Yes	Yes	No	Yes	No	No	No	Yes	No
5275	HSA0719	Male	Malay	2020	37	Others	Secondary	Unemployed	Urban	Yes	No	No	Yes	Yes	No	No	No	Yes	No
5276	HSA0720	Male	Malay	2020	36	Others	Secondary	Private	Urban	Yes	Yes	Yes	No	No	No	No	No	No	No
5277	HSA0721	Male	Indian	2020	32	Others	Primary	Unemployed	Urban	Yes	No	No	Yes	Yes	No	No	No	Yes	No
5278	HSA0722	Male	Malay	2020	39	Others	No formal education	Self-employed	Urban	Yes	No	Yes	No	Yes	No	No	No	No	No
5279	HSA0723	Male	Malay	2020	33	Others	No formal education	Others	Urban	Yes	No	No	No	Yes	No	No	No	No	No
5280	HSA0724	Male	Malay	2020	19	Single	Secondary	Unemployed	Urban	No	Yes	No	Yes	No	No	No	No	No	No
5281	HSA0725	Female	Malay	2020	21	Single	No formal education	Private	Urban	Yes	Yes	No	No	No	No	No	No	No	No
5282	HSA0726	Male	Malay	2020	23	Others	No formal education	Others	Urban	Yes	Yes	No	No	No	No	No	No	No	Yes
5283	HSA0727	Male	Indian	2020	40	Others	Secondary	Private	Urban	Yes	No	Yes	No	No	No	No	No	No	No
5284	HSA0728	Male	Malay	2020	23	Single	Primary	Others	Urban	Yes	Yes	No	No	Yes	No	No	No	Yes	No
5285	HSA0729	Male	Malay	2020	40	Single	Secondary	Private	Urban	Yes	Yes	No	No	Yes	No	No	No	No	No
5286	HSA0730	Male	Malay	2020	27	Single	No formal education	Unemployed	Urban	No	Yes	No	No	No	No	No	No	No	No
5287	HSA0731	Male	Malay	2020	34	Married	No formal education	Private	Urban	Yes	Yes	No	No	Yes	No	No	No	Yes	No
5288	HSA0732	Male	Malay	2020	24	Single	No formal education	Private	Urban	Yes	Yes	No	No	Yes	No	No	No	No	No
5289	HSA0733	Male	Malay	2020	35	Married	Secondary	Private	Urban	Yes	Yes	No	No	No	No	No	No	No	No
5290	HSA0734	Female	Indian	2020	16	Single	Secondary	Unemployed	Urban	Yes	Yes	Yes	No	No	No	No	No	No	No
5291	HSA0735	Male	Malay	2020	27	Married	Secondary	Private	Urban	Yes	Yes	No	No	No	No	No	No	No	No
5292	HSA0736	Male	Indian	2020	45	Married	Primary	Unemployed	Urban	Yes	No	Yes	No	No	No	No	No	No	No
5293	HSA0737	Male	Malay	2020	46	Others	No formal education	Others	Urban	No	No	No	No	No	No	No	No	Yes	No
5294	HSA0738	Male	Indian	2020	49	Others	Secondary	Unemployed	Urban	Yes	No	Yes	No	No	No	No	No	No	No
5295	HSA0739	Male	Malay	2020	18	Single	Secondary	Private	Urban	Yes	Yes	No	No	No	No	No	No	No	No
5296	HSA0740	Male	Malay	2020	25	Single	Tertiary	Private	Rural	Yes	Yes	No	Yes	No	No	No	No	No	Yes
5297	HSA0741	Male	Malay	2020	22	Single	Tertiary	Private	Urban	Yes	Yes	No	Yes	No	No	No	No	No	No
5298	HSA0742	Male	Malay	2020	30	Single	Secondary	Unemployed	Urban	Yes	Yes	Yes	No	No	No	No	No	No	No

No	Patient ID	Gender	Ethnic group	Year of diagnosis	Age	Marital status	Education level	Occupation	Place of residence	History of psy illness	Tobacco	Alcohol	Cannabis	ATS	Inhalant	Sedative	Hallucinogens	Opioids	Kratom
5299	HSA0743	Male	Malay	2020	37	Others	Secondary	Private	Rural	No	Yes	No	Yes	No	No	No	No	Yes	No
5300	HSA0744	Male	Malay	2020	33	Single	Secondary	Unemployed	Urban	Yes	Yes	No	No	No	No	No	No	No	No
5301	HSA0745	Male	Malay	2020	32	Others	Secondary	Others	Urban	Yes	Yes	No	No	Yes	No	No	No	No	No
5302	HSA0746	Male	Malay	2020	34	Single	No formal education	Unemployed	Rural	Yes	No	No	No	No	No	No	No	Yes	No
5303	HSA0747	Male	Malay	2020	34	Others	Secondary	Private	Urban	Yes	Yes	No	No	No	No	No	No	Yes	No
5304	HSA0748	Male	Malay	2020	28	Others	Secondary	Unemployed	Urban	Yes	Yes	No	No	Yes	No	No	No	No	No
5305	HSA0749	Male	Malay	2020	32	Others	Secondary	Government	Urban	No	Yes	No	No	No	No	No	No	No	Yes
5306	HSA0750	Male	Chinese	2020	49	Married	No formal education	Private	Urban	Yes	Yes	Yes	No	No	No	No	No	No	No
5307	HSA0751	Male	Malay	2020	33	Married	No formal education	Unemployed	Urban	Yes	No	Yes	No	No	No	No	No	No	No
5308	HSA0752	Male	Others	2020	46	Married	No formal education	Private	Urban	Yes	No	Yes	No	No	No	No	No	No	No
5309	HSA0753	Male	Malay	2020	32	Single	No formal education	Unemployed	Urban	Yes	Yes	No	No	No	No	No	No	No	No
5310	HSA0754	Male	Indian	2020	53	Single	No formal education	Private	Urban	Yes	Yes	No	No	No	No	No	No	No	No
5311	HSA0755	Male	Malay	2020	33	Single	Tertiary	Private	Urban	Yes	Yes	No	No	No	No	No	No	No	No
5312	HSA0756	Male	Malay	2020	30	Married	Secondary	Private	Urban	Yes	Yes	No	No	No	No	No	No	No	No
5313	HSA0757	Male	Malay	2020	30	Married	Tertiary	Private	Urban	Yes	Yes	No	No	Yes	No	No	No	No	No
5314	HSA0758	Female	Malay	2020	30	Others	No formal education	Unemployed	Urban	Yes	No	No	No	Yes	No	No	No	No	No
5315	HSA0759	Female	Indian	2020	24	Single	Tertiary	Private	Urban	Yes	Yes	Yes	No	No	No	No	No	No	No
5316	HSA0760	Female	Others	2020	34	Married	No formal education	Unemployed	Urban	No	Yes	Yes	No	No	No	No	No	No	No
5317	HSA0761	Male	Malay	2020	22	Single	Secondary	Private	Urban	Yes	Yes	No	No	No	No	No	No	No	No
5318	HSA0762	Female	Malay	2020	28	Others	Secondary	Others	Urban	Yes	No	No	No	Yes	No	No	No	No	No
5319	HSA0763	Male	Malay	2020	23	Others	Tertiary	Unemployed	Urban	Yes	Yes	Yes	No	Yes	No	No	No	No	No
5320	HSA0764	Female	Malay	2020	20	Others	No formal education	Others	Rural	Yes	No	No	No	Yes	No	No	No	No	No
5321	HSA0765	Male	Malay	2020	36	Single	Secondary	Private	Urban	Yes	No	No	Yes	No	No	No	No	No	No
5322	HSA0766	Female	Malay	2020	17	Single	Secondary	Unemployed	Urban	Yes	Yes	No	No	No	No	No	No	No	No
5323	HSA0767	Male	Malay	2020	34	Others	Secondary	Private	Urban	Yes	Yes	No	No	Yes	No	No	No	No	No
5324	HSA0768	Male	Indian	2020	21	Single	No formal education	Private	Urban	Yes	No	Yes	No	No	No	No	No	No	No
5325	HSA0769	Male	Malay	2020	38	Single	No formal education	Unemployed	Urban	Yes	No	No	No	Yes	No	No	No	Yes	No
5326	HSA0770	Female	Malay	2020	27	Others	No formal education	Private	Urban	Yes	Yes	No	No	No	No	No	No	No	No
5327	HSA0771	Male	Indian	2020	33	Single	Secondary	Unemployed	Urban	Yes	Yes	No	Yes	Yes	No	No	No	No	No
5328	HSA0772	Female	Malay	2020	22	Single	Secondary	Unemployed	Urban	Yes	Yes	No	No	No	No	No	No	No	No
5329	HSA0773	Male	Malay	2020	47	Others	No formal education	Private	Urban	Yes	No	No	No	Yes	No	No	No	Yes	No

No	Patient ID	Gender	Ethnic group	Year of diagnosis	Age	Marital status	Education level	Occupation	Place of residence	History of psy illness	Tobacco	Alcohol	Cannabis	ATS	Inhalant	Sedative	Hallucinogens	Opioids	Kratom
5330	HSA0774	Female	Malay	2020	30	Married	Primary	Unemployed	Urban	Yes	Yes	No	No	Yes	No	No	No	No	No
5331	HSA0775	Male	Malay	2020	53	Others	Secondary	Unemployed	Urban	No	Yes	No	No	No	No	No	No	No	No
5332	HSA0776	Female	Malay	2020	29	Others	No formal education	Unemployed	Urban	Yes	Yes	No	No	No	No	No	No	No	No
5333	HSA0777	Male	Malay	2020	26	Others	No formal education	Others	Urban	Yes	No	No	No	Yes	No	No	No	No	No
5334	HSA0778	Female	Malay	2020	32	Married	Secondary	Unemployed	Urban	Yes	Yes	No	No	No	No	No	No	No	No
5335	HSA0779	Male	Malay	2020	47	Married	No formal education	Self-employed	Urban	No	No	Yes	Yes	Yes	No	No	No	No	No
5336	HSA0780	Male	Malay	2020	39	Others	Secondary	Unemployed	Urban	Yes	Yes	No	No	No	No	No	No	No	No
5337	HSA0781	Male	Malay	2020	30	Others	No formal education	Unemployed	Urban	Yes	No	No	No	Yes	No	No	No	No	No
5338	HSA0782	Female	Malay	2020	18	Single	Secondary	Others	Urban	No	Yes	No	Yes	No	No	No	No	No	No
5339	HSA0783	Female	Malay	2020	54	Married	No formal education	Unemployed	Urban	Yes	Yes	No	Yes	No	No	No	No	No	No
5340	HSA0784	Female	Malay	2020	27	Married	No formal education	Unemployed	Rural	Yes	No	No	No	Yes	No	No	No	No	No
5341	HSA0785	Female	Malay	2020	19	Others	Secondary	Unemployed	Urban	Yes	No	No	No	Yes	No	No	No	No	No
5342	HSA0786	Female	Malay	2020	30	Married	Secondary	Private	Urban	Yes	Yes	No	No	No	No	No	No	No	No
5343	HSA0787	Female	Malay	2020	34	Single	No formal education	Others	Urban	Yes	No	No	No	Yes	No	No	No	No	No
5344	HSA0788	Male	Malay	2020	25	Single	Secondary	Private	Urban	No	No	Yes	No	No	No	No	No	No	No
5345	HSA0789	Female	Others	2020	37	Others	Secondary	Unemployed	Urban	Yes	Yes	No	No	Yes	No	No	No	No	No
5346	HSA0790	Female	Malay	2020	23	Married	Tertiary	Private	Urban	Yes	Yes	No	No	No	No	No	No	No	No
5347	HSA0791	Female	Malay	2020	36	Others	Secondary	Private	Urban	Yes	Yes	No	No	Yes	No	No	No	No	No
5348	HSA0792	Female	Others	2020	40	Others	No formal education	Private	Urban	Yes	No	No	No	Yes	No	No	No	No	No
5349	HSA0793	Male	Chinese	2020	57	Single	Primary	Unemployed	Urban	Yes	Yes	No	No	Yes	No	No	No	No	No
5350	HSA0794	Male	Indian	2020	19	Single	Secondary	Private	Urban	No	Yes	Yes	No	No	No	No	No	No	No
5351	HSA0796	Male	Chinese	2020	61	Others	Secondary	Private	Urban	No	Yes	Yes	No	No	No	No	No	No	No
5352	HSA0796	Male	Indian	2020	48	Others	No formal education	Private	Urban	Yes	No	Yes	No	No	No	No	No	No	No
5353	HSA0797	Male	Malay	2020	77	Married	No formal education	Others	Urban	Yes	Yes	No	No	No	No	No	No	No	No
5354	HSA0798	Female	Malay	2020	40	Married	Secondary	Unemployed	Urban	Yes	Yes	No	No	No	No	No	No	No	No
5355	HSA0799	Male	Others	2020	38	Single	No formal education	Private	Urban	Yes	Yes	No	No	Yes	No	No	No	No	No
5356	HSA0800	Female	Malay	2020	27	Others	No formal education	Unemployed	Rural	Yes	No	No	No	No	No	No	No	Yes	No
5357	HSA0801	Female	Malay	2020	33	Married	Primary	Unemployed	Urban	Yes	No	No	No	Yes	No	No	No	No	No
5358	HSA0802	Female	Malay	2020	27	Others	Primary	Private	Urban	Yes	No	No	No	Yes	No	No	No	No	No
5359	HSA0803	Male	Indian	2020	44	Single	Secondary	Private	Urban	Yes	Yes	No	Yes	Yes	No	No	No	No	No
5360	HSA0804	Female	Malay	2020	53	Married	No formal education	Unemployed	Urban	Yes	No	No	Yes	No	No	No	No	No	No

No	Patient ID	Gender	Ethnic group	Year of diagnosis	Age	Marital status	Education level	Occupation	Place of residence	History of psy illness	Tobacco	Alcohol	Cannabis	ATS	Inhalant	Sedative	Hallucinogens	Opioids	Kratom
5361	HSA0805	Male	Malay	2020	49	Others	Secondary	Private	Urban	Yes	Yes	No	No	Yes	No	No	No	Yes	No
5362	HSA0806	Female	Malay	2020	23	Married	Secondary	Private	Urban	No	Yes	No	No	Yes	No	No	No	No	No
5363	HSA0807	Male	Others	2020	60	Married	Secondary	Private	Urban	Yes	No	Yes	No	No	No	No	No	No	No
5364	HSA0808	Female	Malay	2020	27	Others	Secondary	Unemployed	Urban	No	Yes	No	No	Yes	No	No	No	No	No
5365	HSA0809	Male	Malay	2020	51	Married	No formal education	Private	Urban	Yes	Yes	No	No	Yes	No	No	No	No	No
5366	HSA0810	Female	Malay	2020	24	Others	No formal education	Private	Urban	Yes	Yes	No	No	Yes	No	No	No	No	No
5367	HSA0811	Male	Indian	2020	28	Others	No formal education	Private	Urban	Yes	No	Yes	No	Yes	No	No	No	No	No
5368	HSA0812	Female	Malay	2020	29	Others	Secondary	Unemployed	Urban	Yes	Yes	No	No	No	No	No	No	No	No
5369	HSA0813	Female	Indian	2020	46	Others	Primary	Private	Urban	Yes	Yes	Yes	No	No	No	No	No	No	No
5370	HSA0814	Female	Malay	2020	33	Married	No formal education	Others	Urban	Yes	Yes	Yes	No	Yes	No	No	No	Yes	No
5371	HSA0815	Male	Malay	2020	42	Others	No formal education	Others	Urban	No	No	No	No	Yes	No	No	No	No	No
5372	HSA0816	Female	Malay	2020	20	Single	Tertiary	Private	Urban	Yes	Yes	Yes	No	No	No	No	No	No	No
5373	HSA0817	Male	Indian	2020	35	Others	No formal education	Private	Urban	Yes	No	Yes	No	No	No	No	No	No	No
5374	HSA0819	Male	Malay	2020	56	Single	No formal education	Private	Urban	Yes	Yes	No	No	No	No	No	No	Yes	No
5375	HSA0820	Male	Chinese	2020	36	Single	Tertiary	Unemployed	Urban	Yes	Yes	No	No	No	No	No	No	No	No
5376	HSA0821	Female	Indian	2020	25	Married	Secondary	Unemployed	Urban	No	No	Yes	No	No	No	No	No	No	No
5377	HSA0822	Female	Malay	2020	16	Single	Secondary	Unemployed	Urban	Yes	Yes	No	No	No	No	No	No	Yes	No
5378	HSA0823	Male	Malay	2020	43	Married	No formal education	Private	Urban	Yes	Yes	No	No	No	No	No	No	No	No
5379	HSA0824	Female	Malay	2020	23	Others	Secondary	Private	Urban	Yes	Yes	Yes	Yes	Yes	No	No	No	No	No
5380	HSA0825	Male	Malay	2020	37	Single	Secondary	Others	Urban	Yes	Yes	No	No	No	No	No	No	No	No
5381	HSA0826	Male	Malay	2020	29	Married	Secondary	Others	Urban	Yes	No	No	No	Yes	No	No	No	No	No
5382	HSA0827	Female	Malay	2020	51	Single	No formal education	Unemployed	Urban	Yes	No	No	No	No	No	No	No	Yes	No
5383	HSA0828	Male	Malay	2020	23	Others	Secondary	Unemployed	Urban	Yes	Yes	No	No	Yes	Yes	No	No	No	No
5384	HSA0829	Male	Malay	2020	47	Married	Primary	Private	Urban	Yes	Yes	Yes	No	No	No	No	No	No	Yes
5385	HSA0830	Female	Malay	2020	29	Married	Secondary	Private	Urban	Yes	No	No	No	Yes	No	No	No	No	No
5386	HSA0831	Female	Malay	2020	28	Others	Secondary	Private	Urban	Yes	Yes	No	No	Yes	No	No	No	No	No
5387	HSA0832	Female	Others	2020	63	Married	No formal education	Unemployed	Urban	Yes	No	Yes	No	No	No	No	No	No	No
5388	HSA0833	Male	Indian	2020	18	Single	Secondary	Private	Urban	Yes	Yes	Yes	Yes	No	No	No	No	No	No
5389	HSA0834	Female	Malay	2020	37	Others	Secondary	Private	Rural	Yes	Yes	No	No	No	No	No	No	No	No
5390	HSA0835	Male	Malay	2020	41	Others	Secondary	Self-employed	Rural	Yes	Yes	No	No	No	No	No	No	No	No
5391	HSA0836	Male	Indian	2020	32	Others	Secondary	Private	Urban	Yes	Yes	Yes	Yes	Yes	No	No	No	No	No
5392	HSA0837	Male	Indian	2020	34	Married	No formal education	Private	Urban	Yes	No	Yes	No	No	No	No	No	No	No
5393	HSA0838	Male	Indian	2020	49	Married	Secondary	Self-employed	Urban	Yes	Yes	Yes	No	No	No	No	No	No	No

No	Patient ID	Gender	Ethnic group	Year of diagnosis	Age	Marital status	Education level	Occupation	Place of residence	History of psy illness	Tobacco	Alcohol	Cannabis	ATS	Inhalant	Sedative	Hallucinogens	Opioids	Kratom
5394	HSA0839	Male	Malay	2020	43	Single	Tertiary	Others	Urban	Yes	Yes	No	No	No	No	No	No	No	No
5395	HSA0840	Female	Indian	2020	18	Single	Secondary	Others	Urban	No	No	No	No	No	No	No	No	Yes	No
5396	HSA0841	Male	Indian	2020	47	Others	No formal education	Others	Urban	Yes	Yes	Yes	No	No	No	No	No	No	No
5397	HSA0842	Male	Indian	2020	58	Married	No formal education	Others	Urban	Yes	Yes	Yes	No	No	No	No	No	No	No
5398	HSA0843	Male	Malay	2020	59	Others	No formal education	Private	Urban	Yes	Yes	No	No	No	No	No	No	No	No
5399	HSA0844	Male	Malay	2020	37	Others	Primary	Private	Urban	Yes	Yes	No	No	Yes	No	No	No	No	No
5400	HSA0845	Male	Indian	2020	37	Married	No formal education	Unemployed	Urban	Yes	No	Yes	No	Yes	No	No	No	No	No
5401	HSA0846	Male	Malay	2020	32	Others	No formal education	Private	Urban	Yes	Yes	No	No	Yes	No	No	No	No	No
5402	HSA0847	Female	Malay	2020	22	Married	Secondary	Private	Urban	No	Yes	No	No	No	No	No	No	No	No
5403	HSA0848	Female	Indian	2020	40	Others	Secondary	Private	Urban	Yes	No	Yes	No	No	No	No	No	No	No
5404	HSA0849	Female	Indian	2020	29	Married	Secondary	Unemployed	Urban	Yes	No	Yes	No	No	No	No	No	No	No
5405	HSA0850	Male	Indian	2020	43	Others	No formal education	Private	Urban	Yes	No	Yes	Yes	Yes	No	No	No	No	No
5406	HSA0851	Male	Indian	2020	32	Married	Primary	Unemployed	Urban	Yes	Yes	Yes	No	Yes	No	No	No	No	No
5407	HSA0852	Female	Indian	2020	24	Married	Secondary	Unemployed	Urban	Yes	No	Yes	No	No	No	No	No	No	No
5408	HSA0853	Female	Malay	2020	18	Single	Secondary	Private	Urban	Yes	Yes	Yes	No	No	No	No	No	No	No
5409	HSA0854	Male	Malay	2020	31	Single	Secondary	Unemployed	Urban	Yes	Yes	No	No	Yes	No	No	No	No	No
5410	HSA0855	Female	Malay	2020	21	Others	Secondary	Self-employed	Urban	Yes	Yes	No	No	Yes	No	No	No	No	No
5411	HSA0856	Male	Malay	2020	44	Single	No formal education	Private	Urban	Yes	Yes	No	Yes	No	No	No	No	No	No
5412	HSA0857	Male	Indian	2020	39	Single	Secondary	Private	Urban	No	Yes	Yes	No	No	No	No	No	No	No
5413	HSA0858	Male	Chinese	2020	57	Married	Primary	Private	Urban	No	Yes	No	No	No	No	No	No	No	No
5414	HSA0859	Male	Indian	2020	45	Single	No formal education	Unemployed	Urban	Yes	Yes	Yes	No	No	No	No	No	No	No
5415	HSA0860	Male	Indian	2020	40	Married	Secondary	Private	Urban	Yes	Yes	Yes	No	Yes	No	No	No	No	No
5416	HSA0861	Male	Indian	2020	38	Single	No formal education	Unemployed	Urban	Yes	Yes	Yes	No	No	No	No	No	No	No
5417	HSA0862	Male	Indian	2020	43	Married	Secondary	Private	Urban	Yes	Yes	Yes	Yes	Yes	No	No	No	Yes	No
5418	HSA0863	Male	Indian	2020	24	Single	No formal education	Private	Urban	Yes	No	Yes	No	No	No	No	No	No	No
5419	HSA0864	Male	Indian	2020	44	Married	Primary	Private	Urban	No	Yes	Yes	No	Yes	No	No	No	No	No
5420	HSA0865	Female	Malay	2020	65	Others	No formal education	Others	Urban	No	Yes	No	No	No	No	No	No	No	No
5421	HSA0866	Male	Indian	2020	24	Married	No formal education	Private	Urban	Yes	No	Yes	No	No	No	No	No	No	No
5422	HSA0867	Female	Malay	2020	33	Married	No formal education	Unemployed	Urban	Yes	No	No	No	Yes	No	No	No	No	No
5423	HSA0868	Male	Chinese	2020	38	Married	Secondary	Self-employed	Urban	Yes	Yes	Yes	No	No	No	No	No	No	No

No	Patient ID	Gender	Ethnic group	Year of diagnosis	Age	Marital status	Education level	Occupation	Place of residence	History of psy illness	Tobacco	Alcohol	Cannabis	ATS	Inhalant	Sedative	Hallucinogens	Opioids	Kratom
5424	HSA0869	Male	Malay	2020	41	Married	No formal education	Unemployed	Rural	Yes	Yes	No	Yes	Yes	No	No	No	No	No
5425	HSA0870	Male	Indian	2020	67	Married	Primary	Others	Rural	No	No	Yes	No	No	No	No	No	No	No
5426	HSA0871	Female	Malay	2020	31	Others	Primary	Others	Urban	Yes	Yes	No	No	Yes	No	No	No	No	No
5427	HSA0872	Male	Indian	2020	26	Married	No formal education	Self-employed	Urban	Yes	Yes	Yes	No	No	No	No	No	No	No
5428	HSA0873	Female	Malay	2020	28	Married	Primary	Private	Urban	Yes	Yes	No	No	No	No	No	No	No	No
5429	HSA0874	Male	Indian	2020	42	Single	Tertiary	Private	Urban	Yes	Yes	Yes	No	No	No	No	No	No	No
5430	HSA0875	Male	Indian	2020	36	Others	Secondary	Unemployed	Urban	Yes	Yes	No	No	No	No	No	No	No	No
5431	HSA0876	Female	Chinese	2020	31	Others	Secondary	Self-employed	Urban	Yes	No	Yes	No	No	No	No	No	No	No
5432	HSA0877	Male	Chinese	2020	31	Single	Tertiary	Private	Urban	Yes	Yes	No	No	No	No	No	No	No	No
5433	HSA0878	Male	Indian	2020	24	Single	Secondary	Private	Urban	Yes	No	Yes	No	No	No	No	No	No	No
5434	HSA0879	Male	Others	2020	40	Others	No formal education	Private	Urban	Yes	Yes	No	No	No	No	No	No	No	No
5435	HSA0880	Male	Chinese	2020	47	Married	Secondary	Private	Urban	Yes	Yes	Yes	No	No	No	No	No	No	No
5436	HSA0881	Male	Malay	2020	38	Single	Tertiary	Unemployed	Urban	Yes	No	No	No	Yes	No	No	No	No	No
5437	HSA0882	Male	Indian	2020	43	Others	Primary	Unemployed	Urban	Yes	Yes	Yes	No	No	No	No	No	No	No
5438	HSA0883	Female	Malay	2020	38	Others	No formal education	Others	Rural	Yes	Yes	No	No	No	No	No	No	No	No
5439	HSA0884	Male	Chinese	2020	33	Married	No formal education	Others	Urban	No	Yes	No	No	No	No	No	No	No	No
5440	HSA0885	Female	Malay	2020	43	Single	No formal education	Others	Urban	Yes	No	No	No	Yes	No	No	No	Yes	No
5441	HSA0886	Male	Indian	2020	40	Married	Secondary	Private	Urban	Yes	No	Yes	No	No	No	No	No	No	No
5442	HSA0887	Male	Malay	2020	29	Single	Primary	Private	Urban	Yes	Yes	No	No	No	No	No	No	No	No
5443	HSA0888	Male	Chinese	2020	63	Others	No formal education	Unemployed	Urban	Yes	Yes	No	No	No	No	No	Yes	No	No
5444	HSA0889	Male	Indian	2020	19	Single	Secondary	Private	Urban	Yes	Yes	Yes	No	No	No	No	No	No	No
5445	HSA0890	Male	Chinese	2020	64	Others	Primary	Private	Urban	Yes	No	No	No	Yes	No	No	No	Yes	No
5446	HSA0891	Male	Indian	2020	39	Others	No formal education	Private	Urban	Yes	No	Yes	No	No	No	No	No	No	No
5447	HSA0892	Male	Chinese	2020	58	Single	No formal education	Self-employed	Urban	No	No	No	No	Yes	No	No	No	Yes	No
5448	HSA0893	Male	Indian	2020	67	Married	No formal education	Others	Urban	Yes	No	Yes	No	No	No	No	No	No	No
5449	HSA0894	Female	Chinese	2020	43	Married	No formal education	Unemployed	Urban	No	Yes	Yes	No	No	No	No	No	No	No
5450	HSA0895	Male	Indian	2020	48	Others	No formal education	Unemployed	Urban	Yes	No	Yes	No	No	No	No	No	No	No
5451	HSA0896	Male	Chinese	2020	45	Married	Secondary	Private	Urban	No	Yes	No	No	No	No	No	No	No	No
5452	HSA0897	Female	Indian	2020	52	Others	No formal education	Private	Urban	Yes	Yes	Yes	No	No	No	No	No	No	No
5453	HSA0898	Male	Chinese	2020	50	Single	No formal education	Private	Urban	Yes	No	No	No	Yes	No	No	No	No	No

No	Patient ID	Gender	Ethnic group	Year of diagnosis	Age	Marital status	Education level	Occupation	Place of residence	History of psy illness	Tobacco	Alcohol	Cannabis	ATS	Inhalant	Sedative	Hallucinogens	Opioids	Kratom
5454	HSA0899	Female	Others	2020	32	Married	No formal education	Unemployed	Urban	Yes	No	No	No	Yes	No	No	No	No	No
5455	HSA0900	Male	Chinese	2020	32	Others	Tertiary	Private	Urban	Yes	Yes	Yes	No	No	No	No	No	No	No
5456	HSA0901	Male	Malay	2020	31	Single	Secondary	Private	Urban	Yes	Yes	No	No	Yes	No	No	No	No	No
5457	HSA0902	Male	Indian	2020	45	Married	Primary	Private	Urban	Yes	Yes	Yes	No	No	No	No	No	No	No
5458	HSA0903	Female	Chinese	2020	32	Single	No formal education	Private	Urban	Yes	Yes	Yes	No	No	No	No	No	No	No
5459	HSA0904	Female	Chinese	2020	19	Married	Secondary	Private	Urban	Yes	No	Yes	No	No	No	No	No	No	No
5460	HSA0905	Male	Chinese	2020	26	Others	Secondary	Private	Urban	Yes	Yes	No	No	Yes	No	No	No	No	No
5461	HSA0906	Male	Others	2020	44	Married	Tertiary	Private	Urban	Yes	No	Yes	No	No	No	No	No	No	No
5462	HSA0907	Male	Chinese	2020	68	Others	No formal education	Private	Urban	Yes	Yes	No	No	No	No	No	No	No	No
5463	HSA0908	Female	Chinese	2020	16	Single	Secondary	Private	Urban	No	Yes	No	No	No	No	No	No	No	No
5464	HSA0909	Male	Indian	2020	27	Single	No formal education	Unemployed	Urban	Yes	Yes	Yes	No	No	No	No	No	No	No
5465	HSA0910	Female	Chinese	2020	38	Married	Secondary	Unemployed	Urban	Yes	Yes	Yes	No	No	No	No	No	No	No
5466	HSA0911	Male	Chinese	2020	37	Others	Secondary	Private	Urban	No	No	Yes	No	No	No	No	No	No	No
5467	HSA0912	Male	Indian	2020	32	Others	Secondary	Private	Urban	Yes	Yes	Yes	No	No	No	No	No	No	No
5468	HSA0913	Male	Indian	2020	34	Married	Secondary	Private	Urban	Yes	Yes	Yes	No	No	No	No	No	No	No
5469	HSA0914	Male	Chinese	2020	61	Single	No formal education	Unemployed	Urban	No	Yes	No	No	No	No	No	No	No	No
5470	HSA0915	Female	Others	2020	31	Others	No formal education	Others	Urban	Yes	No	No	No	Yes	No	No	No	No	No
5471	HSA0916	Male	Chinese	2020	43	Married	Tertiary	Private	Urban	Yes	Yes	Yes	No	No	No	No	No	No	No
5472	HSA0917	Male	Indian	2020	39	Married	Secondary	Self-employed	Urban	Yes	Yes	Yes	No	No	No	No	No	No	No
5473	HSA0918	Male	Chinese	2020	39	Others	Secondary	Private	Urban	Yes	No	No	No	Yes	No	No	No	No	No
5474	HSA0919	Male	Indian	2020	17	Others	Tertiary	Private	Urban	Yes	Yes	Yes	No	No	No	No	No	No	No
5475	HSA0920	Female	Chinese	2020	24	Others	Primary	Self-employed	Urban	Yes	No	No	No	Yes	No	No	No	No	No
5476	HSA0921	Male	Indian	2020	47	Others	No formal education	Unemployed	Urban	Yes	Yes	Yes	No	No	No	No	No	No	No
5477	HSA0922	Female	Chinese	2020	59	Married	No formal education	Unemployed	Urban	No	Yes	Yes	No	No	No	No	No	No	No
5478	HSA0923	Female	Indian	2020	29	Single	Secondary	Private	Urban	Yes	No	No	No	Yes	No	No	No	No	No
5479	HSA0924	Male	Others	2020	28	Single	No formal education	Unemployed	Urban	Yes	Yes	Yes	No	No	No	No	No	No	No
5480	HSA0925	Male	Malay	2020	50	Others	Secondary	Private	Urban	Yes	Yes	No	No	No	No	No	No	No	No
5481	HSA0926	Male	Malay	2020	56	Others	No formal education	Private	Urban	Yes	Yes	No	No	No	No	No	No	No	No
5482	HSA0927	Male	Indian	2020	55	Married	No formal education	Private	Urban	Yes	Yes	Yes	No	No	No	No	No	No	No
5483	HSA0928	Male	Chinese	2020	41	Others	No formal education	Others	Urban	No	No	No	No	Yes	No	No	No	No	No
5484	HSA0929	Male	Indian	2020	38	Others	Secondary	Private	Urban	Yes	Yes	Yes	No	No	No	No	No	No	No
5485	HSA0930	Female	Chinese	2020	25	Others	Secondary	Private	Urban	No	Yes	Yes	No	No	No	No	No	No	No

No	Patient ID	Gender	Ethnic group	Year of diagnosis	Age	Marital status	Education level	Occupation	Place of residence	History of psy illness	Tobacco	Alcohol	Cannabis	ATS	Inhalant	Sedative	Hallucinogens	Opioids	Kratom
5486	HSA0931	Female	Malay	2020	40	Others	Secondary	Self-employed	Urban	Yes	Yes	Yes	No	No	No	No	No	No	No
5487	HSA0932	Male	Chinese	2020	36	Others	Secondary	Unemployed	Urban	Yes	Yes	No	No	No	No	No	No	No	No
5488	HSA0933	Male	Indian	2020	30	Others	Secondary	Private	Urban	Yes	No	Yes	No	No	No	No	No	No	No
5489	HSA0934	Male	Indian	2020	30	Single	Secondary	Private	Urban	Yes	Yes	Yes	No	Yes	No	No	No	No	No
5490	HSA0935	Male	Indian	2020	23	Married	No formal education	Private	Urban	Yes	No	Yes	No	No	No	No	No	No	No
5491	HSA0936	Female	Others	2020	41	Married	Secondary	Unemployed	Urban	Yes	Yes	Yes	No	No	No	No	No	No	No
5492	HSA0937	Male	Indian	2020	35	Married	Secondary	Private	Urban	Yes	Yes	Yes	No	No	No	No	No	No	No
5493	HSA0938	Male	Others	2020	33	Single	No formal education	Private	Urban	Yes	Yes	No	No	No	No	No	No	No	No
5494	HSA0940	Male	Malay	2020	32	Married	Secondary	Private	Urban	Yes	Yes	Yes	No	No	No	No	No	No	No
5495	HSA0941	Male	Indian	2020	30	Married	Primary	Unemployed	Urban	Yes	Yes	Yes	No	No	No	No	No	No	No
5496	HSA0942	Female	Chinese	2020	14	Single	Secondary	Unemployed	Urban	No	Yes	Yes	No	No	No	No	No	No	No
5497	HSA0943	Female	Others	2020	50	Others	No formal education	Private	Urban	Yes	No	No	No	Yes	No	No	No	No	No
5498	HSA0944	Male	Malay	2020	27	Single	Secondary	Private	Urban	Yes	No	No	No	Yes	No	No	No	No	No
5499	HSA0945	Female	Others	2020	25	Married	Secondary	Unemployed	Urban	Yes	Yes	No	No	Yes	No	No	No	No	No
5500	HSA0946	Female	Indian	2020	24	Married	No formal education	Others	Urban	Yes	Yes	Yes	No	No	No	No	No	No	No
5501	HSA0947	Female	Chinese	2020	23	Others	No formal education	Unemployed	Urban	Yes	Yes	Yes	No	No	No	No	No	No	No
5502	HSA0948	Male	Indian	2020	27	Single	Secondary	Private	Urban	No	Yes	Yes	No	No	No	No	No	No	No
5503	HSA0949	Male	Others	2020	32	Others	No formal education	Private	Urban	No	No	No	No	Yes	No	No	No	No	No
5504	HSA0950	Male	Indian	2020	20	Others	Secondary	Private	Urban	Yes	Yes	Yes	No	No	No	No	No	No	No
5505	HSA0951	Male	Malay	2021	47	Others	Secondary	Private	Urban	Yes	Yes	No	No	No	No	No	No	No	Yes
5506	HSA0952	Male	Malay	2021	36	Others	Secondary	Unemployed	Urban	Yes	No	No	No	Yes	No	No	No	Yes	No
5507	HSA0953	Male	Indian	2021	43	Single	Secondary	Private	Urban	Yes	No	Yes	No	No	No	No	No	No	No
5508	HSA0954	Male	Indian	2021	26	Single	Tertiary	Private	Urban	Yes	No	Yes	No	Yes	No	No	No	Yes	No
5509	HSA0955	Female	Indian	2021	52	Single	Secondary	Unemployed	Urban	Yes	No	Yes	No	No	No	No	No	No	No
5510	HSA0956	Male	Indian	2021	34	Married	Secondary	Private	Urban	Yes	No	Yes	No	No	No	No	No	No	No
5511	HSA0957	Male	Chinese	2021	62	Others	No formal education	Unemployed	Urban	Yes	No	No	No	No	No	No	No	Yes	No
5512	HSA0958	Male	Indian	2021	21	Single	Secondary	Private	Urban	Yes	Yes	Yes	No	No	No	No	No	No	No
5513	HSA0959	Male	Malay	2021	43	Others	Secondary	Private	Urban	Yes	Yes	No	No	Yes	No	No	No	No	No
5514	HSA0960	Male	Indian	2021	77	Others	Secondary	Others	Urban	No	Yes	Yes	No	No	No	No	No	No	No
5515	HSA0961	Male	Indian	2021	40	Married	No formal education	Unemployed	Urban	Yes	No	Yes	No	Yes	No	No	No	Yes	No
5516	HSA0962	Male	Chinese	2021	31	Single	Secondary	Private	Urban	No	Yes	Yes	No	No	No	No	No	No	Yes
5517	HSA0963	Male	Malay	2021	40	Married	Secondary	Self-employed	Urban	Yes	Yes	No	No	Yes	No	No	No	No	No
5518	HSA0964	Male	Malay	2021	38	Others	No formal education	Unemployed	Urban	Yes	No	No	No	Yes	No	No	No	No	No
5519	HSA0965	Female	Malay	2021	34	Married	Secondary	Unemployed	Urban	Yes	Yes	No	No	No	No	No	No	No	No
5520	HSA0966	Male	Chinese	2021	37	Single	Secondary	Private	Urban	No	Yes	Yes	No	No	No	No	No	No	No

No	Patient ID	Gender	Ethnic group	Year of diagnosis	Age	Marital status	Education level	Occupation	Place of residence	History of psy illness	Tobacco	Alcohol	Cannabis	ATS	Inhalant	Sedative	Hallucinogens	Opioids	Kratom
5521	HSA0967	Male	Chinese	2021	46	Single	Primary	Private	Urban	Yes	Yes	Yes	No	No	No	No	No	No	No
5522	HSA0969	Male	Malay	2021	25	Single	Tertiary	Private	Rural	Yes	Yes	No	No	No	No	No	No	No	No
5523	HSA0970	Male	Malay	2021	35	Single	No formal education	Private	Urban	No	Yes	No	No	No	No	No	No	No	No
5524	HSA0971	Female	Chinese	2021	31	Married	Secondary	Private	Urban	Yes	Yes	Yes	No	No	No	No	No	No	No
5525	HSA0972	Female	Chinese	2021	25	Married	Secondary	Self-employed	Urban	Yes	Yes	Yes	No	No	No	No	No	No	No
5526	HSA0973	Male	Indian	2021	40	Single	Primary	Private	Urban	Yes	No	Yes	No	No	No	No	No	No	No
5527	HSA0974	Female	Malay	2021	28	Others	Secondary	Unemployed	Urban	Yes	No	No	No	Yes	No	No	No	No	No
5528	HSA0975	Male	Indian	2021	30	Others	Secondary	Private	Urban	Yes	Yes	Yes	No	Yes	No	No	No	No	No
5529	HSA0976	Male	Malay	2021	59	Others	Secondary	Others	Urban	No	Yes	No	No	No	No	No	No	No	No
5530	HSA0977	Male	Indian	2021	43	Married	Tertiary	Private	Urban	Yes	Yes	Yes	No	No	No	No	No	No	No
5531	HSA0978	Male	Malay	2021	52	Others	Primary	Unemployed	Urban	Yes	No	No	No	Yes	No	No	No	Yes	No
5532	HSA0979	Male	Indian	2021	40	Others	Secondary	Unemployed	Urban	Yes	Yes	Yes	No	No	No	No	No	No	No
5533	HSA0980	Female	Chinese	2021	16	Single	Secondary	Unemployed	Urban	No	Yes	Yes	No	No	No	No	No	No	No
5534	HSA0981	Female	Malay	2021	28	Married	Primary	Unemployed	Urban	Yes	No	Yes	No	No	No	No	No	No	No
5535	HSA0982	Male	Chinese	2021	47	Single	No formal education	Unemployed	Urban	Yes	Yes	No	No	Yes	No	No	No	Yes	No
5536	HSA0983	Male	Indian	2021	20	Single	Secondary	Private	Urban	No	Yes	No	No	No	No	No	No	No	No
5537	HSA0984	Male	Chinese	2021	20	Single	Secondary	Unemployed	Urban	No	No	Yes	No	No	No	No	No	No	No
5538	HSA0985	Male	Indian	2021	41	Others	Secondary	Private	Urban	Yes	Yes	No	No	No	No	No	No	No	No
5539	HSA0986	Female	Indian	2021	30	Married	Tertiary	Self-employed	Urban	Yes	No	Yes	No	No	No	No	No	No	No
5540	HSA0987	Male	Indian	2021	55	Others	No formal education	Others	Urban	Yes	No	Yes	No	No	No	No	No	No	No
5541	HSA0988	Female	Chinese	2021	23	Married	Secondary	Unemployed	Urban	Yes	No	Yes	No	No	No	No	No	No	No
5542	HSA0989	Female	Indian	2021	35	Single	No formal education	Unemployed	Urban	Yes	No	Yes	No	No	No	No	No	No	No
5543	HSA0990	Female	Chinese	2021	62	Married	No formal education	Unemployed	Urban	Yes	No	Yes	No	No	No	No	No	No	No
5544	HSA0991	Female	Malay	2021	39	Others	Secondary	Unemployed	Urban	Yes	Yes	No	No	Yes	No	No	No	No	No
5545	HSA0992	Male	Malay	2021	62	Others	Primary	Private	Urban	No	Yes	No	No	No	No	No	No	No	No
5546	HSA0993	Male	Indian	2021	40	Others	No formal education	Others	Urban	Yes	No	No	No	No	No	No	No	Yes	No
5547	HSA0994	Male	Malay	2021	49	Married	No formal education	Private	Urban	No	Yes	No	No	No	No	No	No	No	No
5548	HSA0995	Male	Indian	2021	27	Single	No formal education	Unemployed	Urban	Yes	Yes	Yes	No	Yes	No	No	No	Yes	No
5549	HSA0996	Female	Others	2021	31	Married	No formal education	Unemployed	Rural	Yes	Yes	No	No	Yes	No	No	No	No	No
5550	HSA0997	Male	Chinese	2021	77	Married	No formal education	Others	Urban	Yes	Yes	Yes	No	No	No	No	No	No	No
5551	HSA0998	Male	Chinese	2021	27	Others	Tertiary	Private	Urban	Yes	No	Yes	No	No	No	No	No	Yes	No
5552	HSA0999	Female	Chinese	2021	36	Married	Secondary	Private	Urban	No	No	Yes	No	No	No	No	No	No	No
5553	HSA1000	Male	Malay	2021	24	Single	Secondary	Unemployed	Urban	Yes	Yes	No	No	Yes	No	No	No	No	No

No	Patient ID	Gender	Ethnic group	Year of diagnosis	Age	Marital status	Education level	Occupation	Place of residence	History of psy illness	Tobacco	Alcohol	Cannabis	ATS	Inhalant	Sedative	Hallucinogens	Opioids	Kratom
5554	HSA1001	Male	Malay	2021	49	Others	No formal education	Others	Urban	Yes	No	No	No	Yes	No	No	No	No	No
5555	HSA1002	Male	Malay	2021	64	Married	Secondary	Unemployed	Urban	Yes	Yes	No	No	No	No	No	No	No	No
5556	HSA1003	Male	Indian	2021	38	Single	Secondary	Unemployed	Urban	Yes	Yes	Yes	No	Yes	No	No	No	No	No
5557	HSA1004	Male	Others	2021	42	Married	Tertiary	Private	Urban	Yes	Yes	No	No	No	No	No	No	No	No
5558	HSA1005	Male	Indian	2021	66	Others	Secondary	Unemployed	Urban	Yes	No	No	No	Yes	No	No	No	No	No
5559	HSA1006	Male	Chinese	2021	19	Single	Tertiary	Private	Urban	Yes	No	Yes	No	No	Yes	No	No	No	No
5560	HSA1007	Male	Indian	2021	39	Others	No formal education	Unemployed	Urban	Yes	No	Yes	No	No	No	No	No	No	No
5561	HSA1008	Male	Indian	2021	67	Others	Tertiary	Unemployed	Urban	No	No	Yes	No	No	No	No	No	No	No
5562	HSA1009	Male	Chinese	2021	43	Others	No formal education	Unemployed	Rural	Yes	Yes	Yes	No	No	No	No	No	No	No
5563	HSA1010	Female	Chinese	2021	50	Married	No formal education	Unemployed	Urban	No	Yes	Yes	No	No	No	No	No	No	No
5564	HSA1011	Female	Malay	2021	26	Married	Tertiary	Private	Urban	Yes	Yes	No	No	No	No	No	No	No	No
5565	HSA1012	Male	Chinese	2021	24	Married	No formal education	Private	Urban	Yes	No	No	No	No	No	No	No	No	No
5566	HSA1013	Male	Chinese	2021	56	Others	No formal education	Unemployed	Urban	Yes	No	Yes	No	No	No	No	No	No	No
5567	HSA1014	Male	Chinese	2021	42	Married	Secondary	Unemployed	Urban	Yes	Yes	No	No	Yes	Yes	No	No	No	No
5568	HSA1015	Female	Malay	2021	36	Others	Tertiary	Private	Urban	Yes	Yes	No	No	No	No	No	No	No	No
5569	HSA1016	Female	Indian	2021	25	Single	No formal education	Unemployed	Urban	Yes	Yes	Yes	No	Yes	No	No	No	No	No
5570	HSA1017	Male	Indian	2021	65	Married	No formal education	Others	Urban	Yes	Yes	No	No	No	No	No	No	No	No
5571	HSA1018	Male	Chinese	2021	63	Married	No formal education	Private	Urban	No	Yes	No	No	No	No	No	No	No	No
5572	HSA1019	Female	Malay	2021	53	Others	No formal education	Unemployed	Urban	Yes	Yes	No	No	Yes	No	No	No	Yes	No
5573	HSA1020	Male	Chinese	2021	29	Single	No formal education	Unemployed	Urban	Yes	No	No	No	No	No	No	No	No	No
5574	HSA1021	Male	Malay	2021	55	Others	Secondary	Others	Urban	Yes	No	No	No	Yes	No	No	No	No	No
5575	HSA1022	Female	Indian	2021	23	Married	Secondary	Others	Urban	Yes	Yes	No	No	Yes	No	Yes	No	No	No
5576	HSA1023	Male	Indian	2021	45	Others	No formal education	Unemployed	Urban	No	Yes	Yes	No	No	No	No	No	No	No
5577	HSA1024	Female	Indian	2021	22	Single	Secondary	Unemployed	Urban	Yes	Yes	Yes	No	No	No	No	No	No	No
5578	HSA1025	Female	Malay	2021	40	Others	No formal education	Others	Urban	Yes	No	No	No	Yes	No	No	No	No	No
5579	HSA1026	Male	Malay	2021	32	Single	Secondary	Private	Urban	Yes	Yes	Yes	Yes	No	No	No	No	No	Yes
5580	HSA1027	Male	Indian	2021	68	Others	No formal education	Private	Urban	Yes	No	No	Yes	Yes	No	No	No	No	No
5581	HSA1028	Male	Indian	2021	23	Married	Secondary	Private	Urban	Yes	Yes	Yes	No	No	No	No	No	No	No
5582	HSA1029	Female	Malay	2021	36	Married	No formal education	Unemployed	Urban	No	Yes	No	No	No	No	No	No	No	No

No	Patient ID	Gender	Ethnic group	Year of diagnosis	Age	Marital status	Education level	Occupation	Place of residence	History of psy illness	Tobacco	Alcohol	Cannabis	ATS	Inhalant	Sedative	Hallucinogens	Opioids	Kratom
5583	HSA1030	Male	Malay	2021	34	Married	No formal education	Private	Urban	Yes	No	No	No	Yes	No	No	No	No	No
5584	HSA1031	Male	Malay	2021	28	Married	Secondary	Private	Rural	Yes	Yes	No	No	Yes	No	No	No	No	No
5585	HSA1032	Male	Indian	2021	48	Married	Primary	Private	Urban	Yes	Yes	Yes	No	No	No	No	No	No	No
5586	HSA1033	Male	Indian	2021	43	Single	No formal education	Unemployed	Urban	Yes	Yes	Yes	No	No	No	No	No	No	No
5587	HSA1035	Female	Malay	2021	44	Others	Tertiary	Private	Urban	Yes	Yes	No	No	Yes	No	No	No	No	No
5588	HSA1036	Male	Malay	2021	45	Others	Secondary	Self-employed	Urban	Yes	Yes	Yes	Yes	Yes	No	No	No	No	No
5589	HSA1037	Female	Malay	2021	27	Others	Secondary	Private	Urban	Yes	Yes	Yes	No	No	No	No	No	No	No
5590	HSA1038	Male	Indian	2021	55	Single	No formal education	Private	Urban	Yes	No	Yes	No	No	No	No	No	No	No
5591	HSA1039	Female	Malay	2021	23	Single	Tertiary	Self-employed	Urban	Yes	No	Yes	Yes	No	No	No	No	No	No
5592	HSA1040	Female	Malay	2021	31	Others	Secondary	Unemployed	Rural	Yes	Yes	No	No	Yes	No	Yes	No	No	No
5593	HSA1041	Male	Indian	2021	44	Others	No formal education	Unemployed	Urban	Yes	No	Yes	No	No	No	No	No	No	No
5594	HSA1042	Female	Chinese	2021	25	Married	Secondary	Unemployed	Urban	Yes	No	Yes	No	No	No	No	No	No	No
5595	HSA1043	Female	Malay	2021	23	Others	Secondary	Unemployed	Urban	Yes	Yes	Yes	No	No	No	No	No	No	No
5596	HSA1044	Male	Malay	2021	44	Single	No formal education	Private	Rural	No	Yes	Yes	No	No	No	No	No	Yes	No
5597	HSA1045	Male	Malay	2021	31	Single	Tertiary	Private	Urban	Yes	No	No	No	Yes	No	No	No	No	No
5598	HSA1046	Male	Indian	2021	32	Others	Secondary	Private	Urban	No	Yes	Yes	No	No	No	No	No	No	No
5599	HSA1047	Female	Malay	2021	38	Others	No formal education	Unemployed	Urban	Yes	Yes	No	No	No	No	No	No	No	No
5600	HSA1048	Female	Malay	2021	34	Married	Secondary	Unemployed	Urban	Yes	Yes	No	Yes	Yes	No	No	No	No	No
5601	HSA1050	Male	Indian	2021	25	Single	No formal education	Unemployed	Urban	Yes	No	Yes	No	No	No	No	No	No	No
5602	HSA1051	Female	Malay	2021	13	Single	Secondary	Unemployed	Urban	Yes	Yes	No	No	No	No	No	No	No	No
5603	HSA1052	Female	Malay	2021	29	Single	Secondary	Private	Urban	No	Yes	Yes	No	No	No	No	No	No	No
5604	HSA1053	Male	Indian	2021	26	Single	No formal education	Unemployed	Urban	No	Yes	Yes	No	No	No	No	No	No	No
5605	HSA1054	Female	Malay	2021	42	Others	Secondary	Private	Urban	Yes	Yes	Yes	No	Yes	No	No	No	No	No
5606	HSA1055	Female	Malay	2021	34	Married	Tertiary	Others	Urban	Yes	Yes	No	No	No	No	No	No	Yes	No
5607	HSA1056	Male	Malay	2021	28	Others	Secondary	Unemployed	Urban	Yes	No	Yes	Yes	Yes	No	No	No	No	Yes
5608	HSA1057	Female	Indian	2021	24	Single	Secondary	Private	Urban	Yes	Yes	Yes	No	No	No	No	No	No	No
5609	HSA1058	Male	Malay	2021	45	Married	No formal education	Self-employed	Urban	Yes	Yes	No	No	Yes	No	No	No	No	No
5610	HSA1059	Female	Malay	2021	28	Others	No formal education	Private	Urban	Yes	Yes	Yes	No	Yes	No	No	No	No	No
5611	HSA1060	Male	Malay	2021	38	Single	Secondary	Self-employed	Urban	Yes	Yes	Yes	No	No	No	No	No	No	No
5612	HSA1061	Male	Malay	2021	35	Others	No formal education	Unemployed	Urban	Yes	Yes	No	No	Yes	No	No	No	Yes	No
5613	HSA1062	Male	Malay	2021	39	Others	Tertiary	Self-employed	Urban	Yes	No	No	Yes	Yes	No	No	Yes	No	No
5614	HSA1063	Male	Malay	2021	33	Married	Secondary	Private	Urban	No	Yes	No	No	No	No	No	No	No	No
5615	HSA1064	Male	Malay	2021	18	Single	Secondary	Unemployed	Urban	No	Yes	No	No	No	No	No	No	No	No

No	Patient ID	Gender	Ethnic group	Year of diagnosis	Age	Marital status	Education level	Occupation	Place of residence	History of psy illness	Tobacco	Alcohol	Cannabis	ATS	Inhalant	Sedative	Hallucinogens	Opioids	Kratom
5616	HSA1065	Male	Malay	2021	29	Single	No formal education	Private	Urban	Yes	No	Yes	Yes	Yes	Yes	No	No	No	No
5617	HSA1066	Male	Malay	2021	39	Married	Secondary	Private	Urban	Yes	Yes	No	No	No	No	No	No	No	No
5618	HSA1067	Female	Malay	2021	33	Married	Secondary	Unemployed	Urban	Yes	Yes	No	No	Yes	No	No	No	Yes	No
5619	HSA1068	Male	Malay	2021	28	Single	Secondary	Unemployed	Urban	Yes	No	No	Yes	Yes	No	No	No	No	Yes
5620	HSA1069	Male	Malay	2021	36	Married	No formal education	Others	Urban	Yes	No	No	Yes	Yes	No	No	No	Yes	No
5621	HSA1070	Male	Malay	2021	31	Single	No formal education	Private	Urban	Yes	Yes	No	No	Yes	No	No	No	No	No
5622	HSA1071	Male	Malay	2021	27	Others	No formal education	Government	Urban	No	Yes	No	No	No	No	No	No	No	No
5623	HSA1072	Male	Malay	2021	40	Single	No formal education	Unemployed	Urban	Yes	No	No	No	Yes	No	No	No	No	No
5624	HSA1073	Male	Malay	2021	39	Single	Secondary	Private	Urban	Yes	Yes	No	No	No	No	No	No	No	No
5625	HSA1074	Male	Malay	2021	22	Single	No formal education	Unemployed	Urban	Yes	Yes	No	No	No	No	No	No	No	No
5626	HSA1075	Male	Malay	2021	28	Married	Secondary	Private	Urban	Yes	Yes	Yes	No	No	No	No	No	No	No
5627	HSA1076	Male	Malay	2021	19	Single	Tertiary	Unemployed	Urban	Yes	Yes	No	Yes	No	No	No	No	No	No
5628	HSA1077	Female	Chinese	2021	38	Single	Secondary	Self-employed	Urban	Yes	No	Yes	No	No	No	No	No	No	No
5629	HSA1078	Male	Malay	2021	46	Married	No formal education	Self-employed	Urban	No	Yes	No	Yes	No	No	No	No	No	No
5630	HSA1079	Male	Indian	2021	58	Single	No formal education	Unemployed	Urban	Yes	No	Yes	No	No	No	No	No	No	No
5631	HSA1080	Male	Others	2021	33	Single	Secondary	Unemployed	Urban	Yes	No	Yes	Yes	Yes	No	No	No	No	No
5632	HSA1081	Male	Malay	2021	26	Single	No formal education	Unemployed	Urban	Yes	Yes	Yes	No	Yes	No	No	No	No	No
5633	HSA1082	Male	Malay	2021	50	Married	No formal education	Private	Urban	Yes	No	No	No	No	No	No	No	Yes	No
5634	HSA1083	Male	Malay	2021	20	Single	Secondary	Unemployed	Urban	No	Yes	No	Yes	Yes	No	No	No	No	No
5635	HSA1084	Male	Malay	2021	23	Single	Secondary	Unemployed	Urban	Yes	Yes	No	Yes	Yes	No	No	No	No	No
5636	HSA1085	Male	Malay	2021	28	Others	Secondary	Private	Urban	Yes	Yes	Yes	Yes	Yes	No	No	No	Yes	No
5637	HSA1086	Male	Malay	2021	26	Single	Secondary	Government	Urban	Yes	Yes	No	No	Yes	No	Yes	No	No	No
5638	HSA1087	Male	Malay	2021	34	Married	Secondary	Unemployed	Urban	Yes	Yes	No	No	Yes	No	No	No	No	No
5639	HSA1088	Male	Malay	2021	27	Single	Tertiary	Private	Urban	Yes	Yes	Yes	No	Yes	No	No	No	No	No
5640	HSA1089	Female	Malay	2021	45	Married	No formal education	Unemployed	Urban	Yes	Yes	No	No	No	No	No	No	No	No
5641	HSA1090	Male	Malay	2021	47	Others	Secondary	Private	Urban	Yes	Yes	Yes	No	No	No	No	No	No	No
5642	HSA1091	Male	Malay	2021	25	Single	No formal education	Unemployed	Urban	Yes	No	No	No	Yes	No	No	No	No	No
5643	HSA1092	Male	Indian	2021	31	Others	No formal education	Private	Urban	No	No	Yes	No	No	No	No	No	No	No
5644	HSA1093	Male	Malay	2021	47	Married	No formal education	Private	Rural	Yes	No	No	No	No	No	No	No	Yes	No
5645	HSA1094	Male	Indian	2021	51	Married	Secondary	Self-employed	Urban	Yes	Yes	Yes	No	No	No	No	No	No	No

No	Patient ID	Gender	Ethnic group	Year of diagnosis	Age	Marital status	Education level	Occupation	Place of residence	History of psy illness	Tobacco	Alcohol	Cannabis	ATS	Inhalant	Sedative	Hallucinogens	Opioids	Kratom
5646	HSA1095	Male	Malay	2021	28	Others	Secondary	Unemployed	Urban	Yes	Yes	No	No	No	No	No	No	No	No
5647	HSA1096	Male	Malay	2021	41	Single	Secondary	Unemployed	Urban	No	Yes	No	No	No	No	No	No	Yes	No
5648	HSA1097	Male	Malay	2021	28	Others	Secondary	Unemployed	Urban	Yes	Yes	No	No	No	No	No	No	No	No
5649	HSA1098	Male	Malay	2021	44	Others	No formal education	Unemployed	Urban	Yes	Yes	No	No	Yes	No	No	No	Yes	No
5650	HSA1099	Male	Malay	2021	25	Married	Primary	Unemployed	Urban	No	Yes	No	No	Yes	No	No	No	No	No
5651	HSA1100	Male	Malay	2021	40	Others	No formal education	Unemployed	Urban	Yes	Yes	Yes	No	Yes	No	No	No	No	No
5652	HSA1101	Male	Malay	2021	42	Others	No formal education	Others	Urban	No	No	No	No	No	No	No	No	Yes	No
5653	HSA1102	Male	Malay	2021	38	Single	No formal education	Private	Urban	No	No	No	No	Yes	No	No	No	No	No
5654	HSA1103	Female	Malay	2021	38	Married	Secondary	Private	Urban	No	Yes	No	Yes	Yes	No	No	No	No	No
5655	HSA1104	Female	Malay	2021	20	Single	Secondary	Others	Urban	Yes	Yes	No	No	Yes	No	No	No	No	No
5656	HSA1105	Male	Malay	2021	37	Others	Secondary	Private	Urban	Yes	Yes	No	No	No	No	No	No	No	No
5657	HSA1106	Male	Malay	2021	43	Married	Tertiary	Private	Rural	No	Yes	No	No	No	No	No	No	No	No
5658	HSA1107	Male	Malay	2021	22	Single	Secondary	Private	Urban	No	Yes	No	No	No	No	No	No	No	No
5659	HSA1108	Female	Malay	2021	28	Married	No formal education	Others	Urban	Yes	Yes	No	No	Yes	No	No	No	No	No
5660	HSA1109	Male	Malay	2021	23	Married	Tertiary	Private	Urban	Yes	Yes	No	No	No	No	No	No	No	No
5661	HSA1110	Male	Malay	2021	23	Single	Secondary	Private	Urban	Yes	Yes	No	No	No	No	No	No	No	No
5662	HSA1111	Male	Malay	2021	34	Single	Secondary	Unemployed	Urban	Yes	Yes	No	No	No	No	No	No	No	No
5663	HSA1112	Male	Malay	2021	26	Others	Secondary	Private	Urban	Yes	Yes	Yes	Yes	Yes	No	No	No	No	No
5664	HSA1113	Male	Malay	2021	16	Single	No formal education	Self-employed	Urban	Yes	Yes	No	No	No	No	No	No	No	No
5665	HSA1114	Male	Malay	2021	64	Others	No formal education	Unemployed	Rural	Yes	No	No	No	Yes	No	No	No	Yes	No
5666	HSA1115	Male	Chinese	2021	27	Married	Secondary	Self-employed	Urban	Yes	Yes	Yes	No	No	No	No	No	No	No
5667	HSA1116	Male	Malay	2021	20	Single	Secondary	Private	Urban	Yes	Yes	No	No	Yes	No	No	No	No	No
5668	HSA1117	Male	Malay	2021	31	Single	Secondary	Unemployed	Urban	Yes	Yes	No	No	No	No	No	No	No	No
5669	HSA1118	Male	Malay	2021	43	Others	No formal education	Unemployed	Urban	No	No	Yes	No	No	No	No	No	Yes	No
5670	HSA1119	Male	Malay	2021	40	Single	Tertiary	Unemployed	Urban	Yes	Yes	No	No	Yes	No	No	Yes	Yes	No
5671	HSA1120	Male	Indian	2021	25	Single	Secondary	Unemployed	Urban	No	Yes	No	No	Yes	No	No	No	No	No
5672	HSA1121	Male	Malay	2021	31	Married	Tertiary	Self-employed	Urban	Yes	Yes	No	No	No	No	No	No	No	No
5673	HSA1122	Male	Chinese	2021	57	Married	No formal education	Private	Urban	Yes	Yes	No	No	No	No	No	No	No	No
5674	HSA1123	Male	Malay	2021	36	Married	Primary	Private	Rural	Yes	Yes	No	No	No	No	No	No	No	No
5675	HSA1125	Male	Chinese	2021	47	Single	Secondary	Others	Urban	Yes	Yes	No	No	No	No	No	No	No	No
5676	HSA1126	Male	Chinese	2021	38	Single	Secondary	Private	Urban	Yes	No	Yes	No	No	No	No	No	No	No
5677	HSA1126	Male	Chinese	2021	51	Married	Secondary	Unemployed	Urban	No	Yes	No	No	No	No	No	No	No	No
5678	HSA1127	Male	Malay	2021	32	Others	No formal education	Private	Urban	Yes	Yes	No	No	Yes	No	No	No	No	No
5679	HSA1128	Male	Chinese	2021	40	Single	Secondary	Private	Urban	Yes	Yes	No	No	No	No	No	No	No	No

No	Patient ID	Gender	Ethnic group	Year of diagnosis	Age	Marital status	Education level	Occupation	Place of residence	History of psy illness	Tobacco	Alcohol	Cannabis	ATS	Inhalant	Sedative	Hallucinogens	Opioids	Kratom
5680	HSA1129	Female	Malay	2021	34	Others	Secondary	Others	Urban	Yes	Yes	No	No	Yes	No	No	No	No	No
5681	HSA1130	Male	Chinese	2021	51	Married	Secondary	Private	Urban	Yes	Yes	Yes	No	No	No	No	No	Yes	No
5682	HSA1131	Male	Others	2021	23	Single	Secondary	Unemployed	Urban	Yes	Yes	Yes	No	Yes	No	No	No	No	No
5683	HSA1132	Male	Chinese	2021	30	Single	Secondary	Private	Urban	Yes	No	No	No	No	No	No	No	No	No
5684	HSA1133	Female	Malay	2021	39	Married	Tertiary	Self-employed	Urban	No	Yes	No	No	No	No	No	No	No	No
5685	HSA1134	Male	Chinese	2021	89	Others	Primary	Others	Urban	Yes	No	Yes	No	No	No	No	No	No	No
5686	HSA1135	Male	Indian	2021	28	Married	No formal education	Private	Urban	Yes	Yes	No	No	Yes	No	No	No	No	No
5687	HSA1136	Male	Chinese	2021	45	Others	Secondary	Others	Urban	Yes	No	No	No	Yes	No	No	No	No	No
5688	HSA1137	Male	Indian	2021	47	Married	Primary	Private	Urban	Yes	Yes	Yes	Yes	Yes	No	No	No	No	No
5689	HSA1138	Male	Chinese	2021	35	Others	Primary	Unemployed	Urban	Yes	Yes	Yes	No	No	No	No	No	No	No
5690	HSA1139	Male	Others	2021	37	Single	Secondary	Unemployed	Urban	Yes	No	Yes	No	No	Yes	No	No	Yes	No
5691	HSA1140	Male	Chinese	2021	55	Others	No formal education	Unemployed	Urban	Yes	Yes	No	No	Yes	No	No	No	Yes	No
5692	HSA1141	Male	Indian	2021	32	Single	Tertiary	Unemployed	Urban	Yes	No	Yes	No	No	No	No	No	No	No
5693	HSA1142	Female	Chinese	2021	38	Married	Tertiary	Private	Urban	No	No	Yes	No	No	No	No	No	No	No
5694	HSA1143	Male	Indian	2021	49	Others	Secondary	Private	Urban	Yes	Yes	Yes	No	No	No	No	No	No	No
5695	HSA1144	Female	Chinese	2021	25	Single	Secondary	Private	Urban	No	Yes	Yes	No	No	No	No	No	No	No
5696	HSA1145	Male	Malay	2021	57	Single	No formal education	Private	Rural	Yes	Yes	No	No	Yes	No	No	No	Yes	No
5697	HSA1146	Male	Others	2021	39	Others	Secondary	Private	Urban	Yes	Yes	Yes	Yes	Yes	No	No	No	No	Yes
5698	HSA1147	Male	Others	2021	51	Others	No formal education	Private	Urban	Yes	No	Yes	No	No	No	No	No	No	No
5699	HSA1148	Female	Malay	2021	33	Others	Tertiary	Private	Urban	Yes	Yes	No	No	Yes	No	No	No	No	No
5700	HSA1149	Female	Chinese	2021	23	Others	Secondary	Unemployed	Urban	Yes	Yes	No	No	No	No	No	No	No	No
5701	HSA1150	Male	Indian	2021	39	Single	No formal education	Unemployed	Urban	Yes	Yes	No	No	No	No	No	No	No	No
5702	HSA1151	Female	Indian	2021	61	Others	No formal education	Unemployed	Urban	No	No	Yes	No	No	No	No	No	No	No
5703	HSA1152	Male	Others	2021	42	Married	Secondary	Private	Urban	No	Yes	Yes	No	No	No	No	No	No	No
5704	HSA1153	Male	Indian	2021	45	Others	No formal education	Unemployed	Urban	Yes	No	Yes	No	No	No	No	No	No	No
5705	HSA1154	Female	Others	2021	27	Others	Primary	Self-employed	Urban	Yes	Yes	Yes	No	No	No	Yes	No	No	No
5706	HSA1155	Male	Indian	2021	79	Married	No formal education	Unemployed	Urban	Yes	No	No	No	Yes	No	No	No	No	No
5707	HSA1156	Female	Others	2021	19	Single	Tertiary	Others	Urban	Yes	No	Yes	No	No	No	No	No	No	No
5708	HSA1157	Female	Others	2021	49	Others	No formal education	Private	Urban	Yes	Yes	Yes	No	No	No	No	No	No	No
5709	HSA1158	Female	Malay	2021	19	Single	Tertiary	Unemployed	Urban	Yes	Yes	Yes	No	No	No	No	No	No	No
5710	HSA1159	Female	Chinese	2021	26	Others	Secondary	Private	Urban	Yes	No	Yes	No	No	No	No	No	No	No
5711	HSA1160	Female	Malay	2021	25	Married	No formal education	Self-employed	Urban	No	Yes	Yes	No	No	No	No	No	No	No
5712	HSA1161	Female	Indian	2021	33	Married	No formal education	Private	Urban	Yes	No	Yes	No	No	No	No	No	No	No

No	Patient ID	Gender	Ethnic group	Year of diagnosis	Age	Marital status	Education level	Occupation	Place of residence	History of psy illness	Tobacco	Alcohol	Cannabis	ATS	Inhalant	Sedative	Hallucinogens	Opioids	Kratom
5713	HSA1162	Male	Malay	2021	42	Married	Secondary	Private	Urban	Yes	Yes	No	No	No	No	No	No	No	No
5714	HSA1163	Female	Chinese	2021	25	Others	Secondary	Private	Urban	Yes	No	Yes	No	No	No	No	No	No	No
5715	HSA1164	Male	Malay	2021	69	Married	Secondary	Unemployed	Urban	Yes	Yes	No	No	No	No	No	Yes	No	No
5716	HSA1165	Male	Malay	2021	61	Married	Secondary	Self-employed	Urban	Yes	Yes	No	No	No	No	No	No	No	No
5717	HSA1166	Male	Chinese	2021	70	Married	Primary	Unemployed	Urban	No	Yes	No	No	No	No	No	No	No	No
5718	HSA1167	Male	Indian	2021	45	Others	No formal education	Others	Urban	Yes	Yes	Yes	No	No	No	No	No	No	No
5719	HSA1168	Male	Malay	2021	33	Single	No formal education	Private	Urban	Yes	No	No	No	Yes	No	No	No	No	No
5720	HSA1169	Male	Indian	2021	59	Married	No formal education	Others	Urban	Yes	No	Yes	No	No	No	No	No	No	No
5721	HSA1170	Male	Chinese	2021	32	Single	Tertiary	Private	Urban	Yes	Yes	No	Yes	Yes	No	No	No	No	No
5722	HSA1171	Male	Malay	2021	39	Married	Primary	Self-employed	Urban	Yes	Yes	Yes	No	No	No	No	No	No	No
5723	HSA1172	Male	Indian	2021	38	Married	Primary	Self-employed	Urban	Yes	Yes	No	No	No	No	No	No	No	No
5724	HSA1173	Male	Indian	2021	25	Single	Secondary	Private	Urban	Yes	Yes	Yes	No	No	No	No	No	No	No
5725	HSA1174	Female	Chinese	2021	32	Married	Secondary	Private	Urban	Yes	Yes	Yes	No	Yes	No	No	No	No	No
5726	HSA1175	Male	Others	2021	40	Married	No formal education	Others	Urban	Yes	Yes	Yes	No	No	No	No	No	No	No
5727	HSA1176	Male	Indian	2021	31	Single	Secondary	Private	Urban	Yes	Yes	Yes	No	No	No	No	No	No	No
5728	HSA1177	Male	Chinese	2021	57	Single	Tertiary	Private	Urban	Yes	Yes	Yes	No	No	No	No	No	No	No
5729	HSA1178	Male	Malay	2021	27	Others	Secondary	Private	Urban	Yes	Yes	No	No	No	No	No	No	No	No
5730	HSA1179	Female	Chinese	2021	16	Single	Secondary	Self-employed	Urban	Yes	Yes	Yes	No	No	No	No	No	No	No
5731	HSA1180	Male	Indian	2021	62	Others	No formal education	Others	Urban	No	No	Yes	No	No	No	No	No	No	No
5732	HSA1181	Female	Malay	2021	27	Others	Secondary	Private	Urban	Yes	Yes	No	No	No	No	No	No	No	No
5733	HSA1182	Male	Malay	2021	76	Married	No formal education	Others	Urban	Yes	Yes	No	No	No	No	No	No	No	No
5734	HSA1183	Male	Chinese	2021	84	Married	No formal education	Self-employed	Urban	Yes	No	Yes	No	No	No	No	No	No	No
5735	HSA1184	Male	Malay	2021	53	Others	Secondary	Others	Urban	Yes	No	No	No	No	No	No	No	Yes	No
5736	HSA1185	Male	Malay	2021	23	Single	Secondary	Unemployed	Urban	Yes	No	No	Yes	Yes	No	No	No	No	No
5737	HSA1186	Male	Malay	2021	38	Single	Secondary	Private	Urban	Yes	Yes	No	No	Yes	No	No	No	Yes	No
5738	HSA1187	Male	Malay	2021	59	Single	Secondary	Private	Urban	Yes	Yes	Yes	No	No	No	No	No	Yes	No
5739	HSA1188	Male	Chinese	2021	30	Single	No formal education	Unemployed	Urban	No	Yes	Yes	No	No	No	No	No	No	No
5740	HSA1189	Male	Indian	2021	88	Married	No formal education	Others	Urban	Yes	No	Yes	No	No	No	No	No	No	No
5741	HSA1190	Female	Malay	2021	44	Others	No formal education	Private	Urban	Yes	No	No	No	Yes	No	No	No	No	No
5742	HSA1191	Male	Chinese	2021	39	Married	No formal education	Private	Urban	Yes	No	No	No	Yes	No	No	No	No	No
5743	HSA1192	Male	Malay	2021	34	Married	Secondary	Private	Rural	No	Yes	No	No	No	No	No	Yes	No	No
5744	HSA1193	Male	Others	2021	23	Single	Secondary	Private	Urban	Yes	No	Yes	No	No	No	No	No	No	No
5745	HSA1194	Female	Others	2021	24	Single	Tertiary	Private	Urban	No	No	Yes	No	No	No	No	No	No	No

No	Patient ID	Gender	Ethnic group	Year of diagnosis	Age	Marital status	Education level	Occupation	Place of residence	History of psy illness	Tobacco	Alcohol	Cannabis	ATS	Inhalant	Sedative	Hallucinogens	Opioids	Kratom
5746	HSA1195	Female	Chinese	2021	23	Others	Secondary	Private	Urban	Yes	No	Yes	No	Yes	No	No	No	No	No
5747	HSA1196	Male	Malay	2021	53	Others	No formal education	Private	Urban	No	No	No	No	No	No	No	No	Yes	No
5748	HSA1197	Male	Indian	2021	21	Single	No formal education	Private	Rural	Yes	No	Yes	No	No	No	No	No	No	No
5749	HSA1198	Male	Malay	2021	29	Single	Secondary	Unemployed	Urban	Yes	Yes	No	Yes	Yes	No	No	No	No	No
5750	HSA1199	Male	Chinese	2021	28	Others	Tertiary	Government	Urban	Yes	Yes	Yes	Yes	No	No	No	No	No	No
5751	HSA1200	Male	Malay	2021	55	Others	No formal education	Unemployed	Urban	No	Yes	No	No	No	No	No	No	No	No
5752	HSA1201	Male	Chinese	2021	55	Others	No formal education	Private	Urban	Yes	No	No	No	No	No	No	No	Yes	No
5753	HSA1202	Male	Others	2021	45	Married	Tertiary	Unemployed	Urban	Yes	Yes	No	No	Yes	No	No	No	No	No
5754	HSA1203	Female	Indian	2021	22	Others	Secondary	Private	Urban	Yes	Yes	No	No	No	No	No	No	No	No
5755	HSA1204	Male	Malay	2021	46	Married	Secondary	Private	Urban	Yes	Yes	No	No	Yes	No	No	No	No	No
5756	HSA1205	Female	Others	2021	36	Married	Secondary	Private	Urban	Yes	No	Yes	No	No	No	No	No	No	No
5757	HSA1206	Male	Malay	2021	45	Others	Secondary	Private	Urban	No	Yes	No	No	No	No	No	No	No	No
5758	HSA1207	Male	Others	2021	27	Married	Secondary	Others	Urban	Yes	No	Yes	Yes	No	No	No	No	No	No
5759	HSA1208	Male	Malay	2021	51	Single	No formal education	Private	Urban	Yes	Yes	No	No	No	No	No	No	No	No
5760	HSA1209	Female	Chinese	2021	62	Married	Secondary	Unemployed	Urban	No	No	Yes	No	No	No	No	No	No	No
5761	HSA1210	Male	Malay	2021	36	Single	Primary	Private	Urban	Yes	Yes	No	No	No	No	No	No	No	No
5762	HSA1212	Female	Others	2021	33	Others	No formal education	Others	Urban	No	No	Yes	No	No	No	No	No	No	No
5763	HSA1213	Male	Malay	2021	34	Others	Secondary	Private	Urban	Yes	No	Yes	Yes	Yes	No	No	No	No	No
5764	HSA1214	Male	Malay	2021	47	Single	Secondary	Private	Urban	Yes	Yes	Yes	No	No	No	No	No	Yes	No
5765	HSA1216	Male	Others	2021	33	Others	Secondary	Unemployed	Urban	Yes	No	No	No	Yes	No	No	No	No	No
5766	HSA1217	Male	Indian	2021	21	Single	Secondary	Unemployed	Urban	Yes	Yes	No	Yes	Yes	No	No	No	No	No
5767	HSA1218	Male	Malay	2021	26	Married	Secondary	Private	Urban	Yes	Yes	No	No	No	No	No	No	No	No
5768	HSA1219	Male	Indian	2021	17	Single	Secondary	Unemployed	Urban	Yes	Yes	Yes	No	No	No	No	No	No	No
5769	HSA1220	Male	Malay	2021	35	Others	No formal education	Others	Urban	Yes	No	No	Yes	Yes	No	No	No	No	No
5770	HSA1221	Male	Malay	2021	53	Married	Secondary	Unemployed	Urban	Yes	No	Yes	No	No	No	No	No	No	No
5771	HSA1222	Male	Malay	2021	64	Others	Primary	Others	Urban	Yes	Yes	No	No	No	No	No	No	No	No
5772	HSA1223	Male	Indian	2021	27	Single	Secondary	Private	Urban	No	No	Yes	No	No	No	No	No	No	No
5773	HSA1224	Male	Malay	2021	31	Single	No formal education	Unemployed	Urban	Yes	No	No	No	Yes	No	No	No	No	No
5774	HSA1225	Male	Malay	2021	36	Others	No formal education	Unemployed	Urban	Yes	No	No	No	Yes	No	No	No	Yes	No
5775	HSA1226	Male	Malay	2018	41	Others	Secondary	Private	Urban	Yes	Yes	No	No	No	No	No	No	No	No
5776	HSA1228	Male	Indian	2019	29	Single	Tertiary	Private	Urban	Yes	Yes	Yes	No	No	No	No	No	No	No
5777	HSA1229	Male	Malay	2019	28	Single	Secondary	Private	Urban	Yes	Yes	No	No	No	No	No	No	No	No
5778	HSA1230	Male	Others	2021	35	Single	Secondary	Private	Urban	Yes	Yes	No	No	No	No	No	No	No	No
5779	HSA1231	Male	Indian	2018	40	Married	Secondary	Unemployed	Urban	Yes	Yes	Yes	No	Yes	No	No	No	No	No

No	Patient ID	Gender	Ethnic group	Year of diagnosis	Age	Marital status	Education level	Occupation	Place of residence	History of psy illness	Tobacco	Alcohol	Cannabis	ATS	Inhalant	Sedative	Hallucinogens	Opioids	Kratom
5780	HSA1232	Male	Malay	2021	28	Single	No formal education	Unemployed	Urban	Yes	No	No	Yes	No	No	No	No	No	No
5781	HSA1233	Male	Malay	2018	41	Others	Primary	Unemployed	Rural	Yes	Yes	No	No	No	No	No	No	No	No
5782	HSA1234	Male	Malay	2021	25	Single	No formal education	Private	Urban	No	Yes	Yes	No	No	No	No	No	No	No
5783	HSA1235	Male	Indian	2019	41	Married	No formal education	Private	Urban	Yes	Yes	Yes	No	No	No	No	No	No	No
5784	HSA1236	Male	Indian	2018	17	Single	Secondary	Unemployed	Urban	Yes	Yes	Yes	No	No	No	No	No	No	No
5785	HSA1237	Male	Malay	2018	44	Married	Secondary	Self-employed	Urban	Yes	Yes	No	No	No	No	No	No	No	No
5786	HSA1238	Male	Chinese	2018	41	Married	Primary	Private	Urban	Yes	Yes	Yes	No	Yes	No	No	No	No	No
5787	HSA1239	Male	Malay	2019	16	Single	Secondary	Unemployed	Urban	Yes	Yes	No	No	No	No	No	No	No	No
5788	HSA1240	Male	Malay	2018	21	Single	Secondary	Unemployed	Urban	Yes	Yes	No	No	No	No	No	No	No	No
5789	HSA1242	Male	Malay	2021	25	Single	No formal education	Unemployed	Urban	Yes	Yes	Yes	No	No	No	No	No	No	No
5790	HSA1243	Male	Indian	2020	45	Single	No formal education	Unemployed	Urban	Yes	No	Yes	No	Yes	No	No	No	No	No
5791	HSA1244	Male	Chinese	2019	43	Others	Secondary	Others	Urban	Yes	Yes	Yes	No	No	No	No	No	No	No
5792	HSA1245	Male	Malay	2019	29	Single	Tertiary	Government	Urban	Yes	Yes	No	No	No	No	No	No	No	Yes
5793	HSA1247	Male	Malay	2020	67	Married	Primary	Unemployed	Urban	Yes	Yes	No	No	No	No	No	No	No	No
5794	HSA1248	Male	Malay	2019	45	Others	Secondary	Unemployed	Urban	Yes	Yes	No	Yes	Yes	No	No	No	No	No
5795	HSA1249	Male	Malay	2020	35	Married	Tertiary	Government	Urban	Yes	No	No	No	Yes	No	No	No	No	No
5796	HSA1250	Male	Others	2019	32	Single	No formal education	Unemployed	Urban	Yes	No	Yes	No	No	No	No	No	No	No
5797	HSA1251	Male	Chinese	2019	28	Single	No formal education	Unemployed	Urban	Yes	Yes	Yes	No	No	No	No	No	No	No
5798	HSA1252	Male	Others	2019	24	Single	Secondary	Unemployed	Rural	Yes	Yes	No	No	No	No	No	No	No	No
5799	HSA1253	Male	Chinese	2019	70	Others	No formal education	Others	Urban	Yes	No	Yes	No	No	No	No	No	No	No
5800	HSA1254	Male	Chinese	2019	38	Single	Secondary	Unemployed	Urban	Yes	Yes	No	No	No	No	No	No	No	No
5801	HSA1255	Male	Malay	2018	55	Married	No formal education	Private	Urban	No	No	No	Yes	No	No	No	No	No	No
5802	HSA1256	Male	Indian	2018	25	Single	Tertiary	Private	Urban	Yes	Yes	No	Yes	No	No	No	No	No	No
5803	HSA1257	Male	Malay	2019	21	Single	Secondary	Unemployed	Rural	Yes	Yes	No	No	No	No	No	No	No	No
5804	HSA1258	Male	Chinese	2018	68	Married	No formal education	Unemployed	Urban	No	No	No	No	No	No	No	No	Yes	No
5805	HSA1259	Male	Malay	2019	51	Single	Secondary	Private	Urban	Yes	Yes	No	No	No	No	No	No	No	No
5806	HSA1260	Male	Indian	2018	28	Single	No formal education	Unemployed	Urban	No	Yes	No	No	No	No	No	No	No	No
5807	HSA1261	Male	Indian	2019	35	Married	Tertiary	Private	Urban	Yes	Yes	No	No	No	No	No	No	No	No
5808	HSA1262	Female	Malay	2018	16	Single	Secondary	Unemployed	Urban	No	Yes	No	No	No	No	No	No	No	No
5809	HSA1263	Male	Malay	2019	29	Single	Tertiary	Private	Urban	No	Yes	Yes	Yes	Yes	No	No	No	Yes	No
5810	HSA1264	Male	Malay	2018	36	Married	Secondary	Government	Urban	Yes	Yes	No	No	No	No	No	No	No	No
5811	HSA1265	Male	Chinese	2019	35	Married	Secondary	Private	Urban	Yes	Yes	Yes	No	No	No	No	Yes	No	No
5812	HSA1266	Male	Indian	2018	28	Single	Secondary	Private	Urban	Yes	Yes	No	No	No	No	No	No	No	No

No	Patient ID	Gender	Ethnic group	Year of diagnosis	Age	Marital status	Education level	Occupation	Place of residence	History of psy illness	Tobacco	Alcohol	Cannabis	ATS	Inhalant	Sedative	Hallucinogens	Opioids	Kratom
5813	HSA1267	Male	Malay	2018	53	Married	No formal education	Others	Urban	No	Yes	No	No	No	No	No	No	No	No
5814	HSA1268	Male	Malay	2018	24	Single	Secondary	Unemployed	Urban	Yes	Yes	No	No	No	No	No	No	No	No
5815	HSA1269	Male	Malay	2019	42	Married	No formal education	Private	Urban	Yes	Yes	No	No	No	No	No	No	No	No
5816	HSA1270	Male	Malay	2018	31	Married	Secondary	Private	Urban	Yes	Yes	No	No	Yes	No	No	No	No	No
5817	HSA1272	Male	Malay	2018	33	Married	Secondary	Self-employed	Rural	Yes	Yes	No	No	No	No	No	No	No	No
5818	HSA1273	Male	Malay	2019	18	Single	Secondary	Self-employed	Rural	Yes	Yes	No	Yes	Yes	No	No	No	No	No
5819	HSA1274	Male	Chinese	2019	46	Married	Secondary	Private	Urban	Yes	Yes	Yes	No	No	No	No	No	No	No
5820	HSA1275	Male	Chinese	2018	41	Others	Primary	Unemployed	Urban	Yes	No	No	Yes	Yes	No	No	No	No	No
5821	HSA1276	Male	Malay	2019	51	Married	Primary	Government	Urban	Yes	Yes	No	No	No	No	No	No	No	No
5822	HSA1277	Male	Chinese	2019	30	Single	Tertiary	Private	Urban	Yes	Yes	No	No	No	No	No	No	No	No
5823	HSA1278	Male	Malay	2019	30	Single	No formal education	Private	Urban	Yes	Yes	Yes	No	No	No	No	No	Yes	No
5824	HSA1279	Female	Malay	2018	19	Single	Secondary	Private	Urban	Yes	Yes	Yes	No	No	No	No	No	No	No
5825	HSA1280	Male	Indian	2018	38	Single	Secondary	Private	Urban	Yes	No	Yes	No	No	No	No	No	No	No
5826	HSA1281	Male	Malay	2019	24	Single	No formal education	Unemployed	Urban	Yes	Yes	No	No	Yes	No	No	No	Yes	No
5827	HSA1282	Male	Chinese	2019	58	Married	No formal education	Private	Urban	Yes	Yes	No	No	No	No	No	No	No	No
5828	HSA1283	Male	Malay	2019	49	Married	Secondary	Private	Urban	Yes	Yes	No	No	No	No	No	No	No	No
5829	HSA1284	Male	Malay	2019	22	Single	Secondary	Unemployed	Urban	Yes	Yes	No	No	No	No	No	No	No	No
5830	HSA1285	Male	Indian	2019	19	Single	Secondary	Government	Urban	No	Yes	Yes	No	No	No	No	No	No	No
5831	HSA1286	Male	Malay	2018	22	Single	Secondary	Unemployed	Urban	Yes	No	Yes	Yes	No	No	No	No	No	No
5832	HSA1287	Male	Malay	2019	18	Single	Tertiary	Unemployed	Urban	Yes	Yes	Yes	Yes	No	No	No	No	No	No
5833	HSA1288	Male	Malay	2018	23	Single	Secondary	Private	Urban	Yes	Yes	No	No	Yes	No	No	No	No	No
5834	HSA1289	Male	Malay	2019	23	Single	Secondary	Private	Urban	Yes	Yes	Yes	No	No	No	No	No	No	No
5835	HSA1290	Male	Malay	2020	37	Single	Tertiary	Private	Urban	Yes	No	Yes	No	No	No	No	No	No	No
5836	HSA1291	Male	Malay	2019	36	Single	Secondary	Government	Urban	No	Yes	No	No	No	No	Yes	No	No	No
5837	HSA1292	Male	Chinese	2019	81	Married	Secondary	Unemployed	Urban	Yes	No	Yes	No	No	No	No	No	No	No
5838	HSA1293	Male	Malay	2019	18	Single	Tertiary	Unemployed	Urban	Yes	Yes	No	No	No	No	No	No	No	No
5839	HSA1294	Male	Malay	2018	42	Married	No formal education	Government	Urban	Yes	Yes	No	No	No	No	No	No	No	No
5840	HSA1295	Male	Indian	2020	40	Single	Secondary	Private	Rural	Yes	Yes	No	No	No	No	No	No	No	No
5841	HSA1296	Male	Indian	2018	27	Others	Secondary	Others	Urban	Yes	Yes	Yes	No	No	No	No	No	No	No
5842	HSA1297	Male	Malay	2019	31	Others	No formal education	Private	Urban	Yes	No	Yes	Yes	Yes	No	No	No	No	No
5843	HSA1298	Male	Malay	2018	23	Single	No formal education	Government	Urban	Yes	Yes	No	No	No	No	No	No	No	No
5844	HSA1299	Male	Chinese	2019	23	Single	Secondary	Private	Urban	Yes	Yes	No	No	No	No	No	No	No	No
5845	HSA1300	Male	Indian	2019	37	Married	Secondary	Private	Urban	Yes	No	Yes	No	No	No	No	No	No	No
5846	HSA1301	Male	Indian	2021	24	Single	Tertiary	Unemployed	Rural	Yes	Yes	Yes	No	No	No	No	No	No	No
5847	HSA1302	Male	Indian	2018	68	Married	No formal education	Private	Urban	Yes	No	Yes	No	No	No	No	Yes	No	No

No	Patient ID	Gender	Ethnic group	Year of diagnosis	Age	Marital status	Education level	Occupation	Place of residence	History of psy illness	Tobacco	Alcohol	Cannabis	ATS	Inhalant	Sedative	Hallucinogens	Opioids	Kratom
5848	HSA1303	Male	Malay	2019	41	Single	No formal education	Unemployed	Urban	Yes	Yes	No	No	No	No	No	No	No	No
5849	HSA1304	Female	Malay	2019	22	Single	Tertiary	Private	Urban	Yes	Yes	No	Yes	No	No	No	No	No	No
5850	HSA1305	Male	Malay	2018	44	Married	Secondary	Government	Urban	No	Yes	No	Yes	No	No	No	No	No	No
5851	HSA1306	Male	Malay	2018	41	Married	No formal education	Unemployed	Urban	Yes	Yes	No	No	No	No	No	No	No	No
5852	HSA1307	Male	Malay	2018	35	Single	Secondary	Self-employed	Urban	Yes	Yes	Yes	No	No	No	No	No	No	No
5853	HSA1308	Male	Indian	2018	26	Single	Secondary	Unemployed	Urban	Yes	Yes	No	No	Yes	No	No	No	Yes	No
5854	HSA1309	Male	Malay	2019	28	Single	Tertiary	Private	Urban	Yes	Yes	No	No	No	No	Yes	No	No	No
5855	HSA1310	Male	Indian	2019	25	Single	Tertiary	Private	Urban	Yes	No	No	No	No	No	No	No	No	No
5856	HSA1311	Male	Chinese	2018	67	Married	No formal education	Private	Urban	Yes	Yes	Yes	No	No	No	No	No	Yes	No
5857	HSA1312	Male	Malay	2018	54	Married	Primary	Private	Urban	Yes	Yes	No	Yes	No	No	No	No	No	No
5858	HSA1313	Male	Malay	2018	20	Single	Secondary	Unemployed	Urban	Yes	Yes	No	Yes	Yes	No	No	No	No	No
5859	HSA1314	Male	Malay	2020	39	Married	Secondary	Unemployed	Urban	Yes	Yes	No	No	No	No	No	No	No	No
5860	HSA1315	Male	Malay	2019	26	Married	Secondary	Private	Urban	No	Yes	No	No	No	No	No	No	No	No
5861	HSA1316	Male	Chinese	2019	73	Married	No formal education	Unemployed	Urban	Yes	Yes	No	No	No	No	No	No	No	No
5862	HSA1317	Male	Malay	2018	40	Married	No formal education	Private	Urban	Yes	No	No	Yes	No	No	No	No	No	No
5863	HSA1318	Male	Indian	2018	39	Married	Primary	Unemployed	Urban	Yes	Yes	Yes	Yes	No	No	No	No	No	No
5864	HSA1319	Male	Chinese	2018	42	Married	Secondary	Private	Urban	No	Yes	No	No	No	No	No	No	No	No
5865	HSA1320	Male	Chinese	2020	27	Others	Tertiary	Government	Urban	Yes	No	Yes	No	No	No	No	No	No	No
5866	HSA1321	Male	Indian	2018	68	Married	No formal education	Others	Urban	Yes	Yes	No	No	No	No	No	Yes	No	No
5867	HSA1322	Male	Malay	2020	74	Married	Secondary	Unemployed	Urban	Yes	Yes	No	No	No	No	No	No	No	No
5868	HSA1323	Male	Indian	2019	32	Single	Secondary	Private	Urban	Yes	Yes	No	No	No	No	No	No	No	No
5869	HSA1324	Male	Chinese	2018	31	Single	Secondary	Private	Urban	Yes	Yes	Yes	No	No	No	No	No	No	No
5870	HSA1325	Female	Others	2019	24	Single	Primary	Private	Urban	Yes	No	Yes	No	No	No	No	No	No	No
5871	HSA1326	Male	Malay	2018	36	Single	No formal education	Others	Urban	Yes	Yes	No	Yes	Yes	No	No	No	Yes	No
5872	HSA1327	Male	Chinese	2018	65	Married	No formal education	Unemployed	Urban	Yes	Yes	Yes	No	No	No	No	No	No	No
5873	HSA1328	Female	Chinese	2020	34	Others	Secondary	Unemployed	Urban	No	Yes	Yes	No	No	No	No	No	No	No
5874	HSA1329	Female	Chinese	2019	37	Others	No formal education	Unemployed	Urban	Yes	Yes	No	No	No	No	No	No	No	No
5875	HSA1330	Male	Malay	2019	34	Married	No formal education	Private	Urban	Yes	Yes	No	No	No	No	No	No	No	No
5876	HSA1331	Female	Chinese	2019	81	Married	No formal education	Unemployed	Urban	Yes	Yes	No	No	No	No	No	No	No	No
5877	HSA1332	Female	Chinese	2020	19	Others	Secondary	Unemployed	Urban	Yes	Yes	No	No	No	No	No	No	No	No
5878	HSA1333	Male	Indian	2019	60	Married	No formal education	Unemployed	Urban	No	Yes	No	No	Yes	No	No	No	No	No

No	Patient ID	Gender	Ethnic group	Year of diagnosis	Age	Marital status	Education level	Occupation	Place of residence	History of psy illness	Tobacco	Alcohol	Cannabis	ATS	Inhalant	Sedative	Hallucinogens	Opioids	Kratom
5879	HSA1334	Female	Malay	2020	20	Single	No formal education	Private	Urban	Yes	Yes	No	No	Yes	No	No	No	No	No
5880	HSA1335	Male	Malay	2018	40	Single	No formal education	Others	Urban	Yes	Yes	No	No	No	No	No	No	No	No
5881	HSA1336	Male	Malay	2019	42	Others	No formal education	Unemployed	Urban	Yes	Yes	No	No	Yes	No	No	No	No	No
5882	HSA1337	Male	Malay	2018	25	Single	Secondary	Private	Urban	Yes	Yes	No	No	No	No	No	No	No	No
5883	HSA1338	Male	Chinese	2018	33	Single	Secondary	Unemployed	Urban	Yes	No	Yes	No	No	No	No	No	No	No
5884	HSA1339	Female	Chinese	2021	16	Single	Secondary	Unemployed	Urban	No	Yes	Yes	No	No	No	No	No	No	No
5885	HSA1340	Male	Malay	2018	59	Married	Secondary	Self-employed	Urban	Yes	No	Yes	Yes	No	No	No	No	No	No
5886	HSA1341	Male	Indian	2020	30	Single	Secondary	Unemployed	Urban	Yes	Yes	No	No	No	No	No	No	No	No
5887	HSA1342	Male	Malay	2020	58	Single	No formal education	Unemployed	Urban	Yes	Yes	Yes	No	No	No	No	No	No	No
5888	HSA1343	Male	Indian	2019	60	Others	Secondary	Unemployed	Urban	Yes	No	Yes	No	No	No	No	No	No	No
5889	HSA1344	Male	Chinese	2018	27	Single	Secondary	Private	Urban	Yes	Yes	Yes	No	No	No	No	No	No	No
5890	HSA1345	Male	Indian	2019	33	Others	Tertiary	Unemployed	Urban	Yes	Yes	No	No	No	No	No	No	No	No
5891	HSA1346	Male	Indian	2020	41	Married	Secondary	Private	Urban	Yes	Yes	Yes	No	No	No	No	No	No	No
5892	HSA1347	Male	Chinese	2018	72	Married	Tertiary	Others	Urban	No	Yes	No	No	No	No	No	No	No	No
5893	HSA1348	Male	Malay	2019	22	Single	Secondary	Private	Urban	Yes	Yes	No	No	No	No	No	No	No	No
5894	HSA1349	Male	Indian	2020	48	Others	Primary	Unemployed	Urban	No	Yes	Yes	No	No	No	No	No	No	No
5895	HSA1350	Male	Others	2018	35	Single	Primary	Unemployed	Urban	Yes	Yes	Yes	No	Yes	No	No	No	No	No
5896	HSA1351	Male	Chinese	2018	88	Others	Secondary	Private	Urban	Yes	Yes	No	No	No	No	No	No	No	No
5897	HSA1352	Male	Chinese	2018	34	Others	Secondary	Private	Urban	Yes	Yes	Yes	No	No	No	No	No	No	No
5898	HSA1353	Male	Indian	2018	49	Married	Primary	Unemployed	Urban	Yes	Yes	Yes	No	No	No	No	No	No	No
5899	HSA1354	Male	Malay	2018	58	Married	No formal education	Unemployed	Urban	Yes	Yes	No	No	No	No	No	No	No	No
5900	HSA1355	Male	Malay	2019	26	Others	Tertiary	Unemployed	Urban	No	Yes	No	No	No	No	No	No	No	No
5901	HSA1356	Male	Malay	2020	36	Single	No formal education	Private	Urban	Yes	Yes	No	No	No	No	No	No	No	No
5902	HSA1357	Male	Indian	2020	63	Married	Secondary	Unemployed	Urban	Yes	No	Yes	No	No	No	No	No	No	No
5903	HSA1358	Male	Chinese	2019	41	Single	Secondary	Unemployed	Urban	Yes	Yes	No	No	No	No	No	No	No	No
5904	HSA1359	Male	Chinese	2019	15	Single	Secondary	Unemployed	Urban	Yes	Yes	No	No	No	No	No	No	No	No
5905	HSA1360	Male	Indian	2019	38	Married	No formal education	Private	Urban	Yes	No	Yes	No	No	No	No	No	No	No
5906	HSA1361	Male	Chinese	2019	79	Married	No formal education	Unemployed	Urban	Yes	No	Yes	No	No	No	No	No	No	No
5907	HSA1362	Male	Malay	2019	22	Single	Tertiary	Private	Urban	Yes	Yes	No	No	No	No	No	No	No	No
5908	HSA1363	Male	Malay	2018	30	Married	Tertiary	Private	Urban	Yes	Yes	No	No	No	No	No	No	No	No
5909	HSA1364	Male	Malay	2018	25	Married	Tertiary	Private	Urban	Yes	Yes	No	No	No	No	No	No	No	No
5910	HSA1365	Male	Chinese	2019	50	Single	Secondary	Private	Urban	No	No	Yes	No	No	No	No	No	No	No
5911	HSA1366	Male	Chinese	2019	57	Others	No formal education	Private	Urban	Yes	Yes	Yes	No	No	No	No	No	No	No

No	Patient ID	Gender	Ethnic group	Year of diagnosis	Age	Marital status	Education level	Occupation	Place of residence	History of psy illness	Tobacco	Alcohol	Cannabis	ATS	Inhalant	Sedative	Hallucinogens	Opioids	Kratom
5912	HSA1367	Male	Indian	2019	64	Others	No formal education	Unemployed	Urban	No	Yes	No	No	No	No	No	No	No	No
5913	HSA1368	Male	Malay	2018	28	Single	Secondary	Unemployed	Rural	Yes	Yes	No	Yes	Yes	No	No	No	No	No
5914	HSA1369	Male	Malay	2018	29	Others	Tertiary	Unemployed	Urban	Yes	Yes	No	Yes	Yes	No	No	No	No	No
5915	HSA1370	Male	Chinese	2018	32	Others	Secondary	Private	Urban	No	Yes	No	No	No	No	No	No	No	No
5916	HSA1372	Male	Indian	2020	76	Married	No formal education	Unemployed	Urban	Yes	No	Yes	No	No	No	No	No	No	No
5917	HSA1373	Male	Indian	2018	26	Single	Secondary	Private	Urban	No	Yes	Yes	No	Yes	No	No	No	No	No
5918	HSA1374	Male	Chinese	2018	39	Single	Secondary	Private	Urban	Yes	Yes	Yes	No	No	No	No	No	No	No
5919	HSA1375	Male	Chinese	2018	79	Married	Secondary	Others	Urban	No	No	Yes	No	No	No	No	No	No	No
5920	HSA1376	Male	Chinese	2019	48	Married	Tertiary	Private	Urban	Yes	Yes	Yes	No	No	No	No	No	No	No
5921	HSA1377	Male	Indian	2020	78	Married	Primary	Unemployed	Urban	No	Yes	No	No	No	No	No	No	No	No
5922	HSA1378	Male	Malay	2018	25	Single	Tertiary	Private	Urban	Yes	Yes	No	No	No	No	No	No	No	No
5923	HSA1379	Male	Malay	2019	31	Married	Tertiary	Private	Rural	Yes	No	Yes	No	No	No	No	No	No	No
5924	HSA1380	Male	Malay	2020	19	Single	Tertiary	Unemployed	Urban	No	No	No	Yes	No	No	No	No	No	No
5925	HSA1381	Male	Malay	2020	20	Single	Secondary	Private	Rural	Yes	Yes	Yes	Yes	Yes	No	No	No	No	Yes
5926	HSA1382	Male	Others	2019	31	Married	No formal education	Private	Urban	Yes	No	Yes	No	No	No	No	No	No	No
5927	HSA1383	Male	Malay	2018	63	Married	No formal education	Others	Urban	Yes	Yes	No	No	No	No	No	No	No	No
5928	HSA1384	Male	Indian	2019	35	Others	No formal education	Private	Urban	No	No	No	No	Yes	Yes	No	No	Yes	No
5929	HSA1385	Male	Indian	2018	41	Married	No formal education	Private	Urban	Yes	Yes	Yes	No	No	No	No	No	No	No
5930	HSA1386	Male	Chinese	2019	59	Married	Primary	Unemployed	Urban	Yes	Yes	Yes	No	No	Yes	No	No	No	No
5931	HSA1387	Male	Malay	2018	41	Single	No formal education	Self-employed	Urban	Yes	Yes	No	No	No	No	No	No	No	No
5932	HSA1388	Male	Chinese	2018	76	Married	Secondary	Unemployed	Urban	No	Yes	Yes	No	No	No	No	No	No	No
5933	HSA1389	Male	Malay	2018	38	Single	Secondary	Unemployed	Urban	Yes	Yes	No	Yes	Yes	No	No	No	No	No
5934	HSA1390	Male	Malay	2018	39	Single	No formal education	Others	Urban	Yes	Yes	No	No	No	No	No	No	No	No
5935	HSA1391	Male	Indian	2018	35	Others	Secondary	Private	Urban	Yes	Yes	Yes	No	No	No	No	No	No	No
5936	HSA1392	Male	Indian	2018	30	Married	Tertiary	Private	Urban	Yes	Yes	Yes	No	No	No	No	No	Yes	No
5937	HSA1393	Male	Indian	2019	29	Single	No formal education	Unemployed	Urban	Yes	No	No	No	Yes	No	No	No	Yes	No
5938	HSA1394	Male	Malay	2018	54	Married	No formal education	Private	Urban	No	Yes	No	Yes	No	No	No	No	No	No
5939	HSA1395	Male	Malay	2018	26	Single	No formal education	Private	Urban	Yes	Yes	No	No	Yes	No	No	No	No	No
5940	HSA1396	Male	Malay	2018	24	Single	No formal education	Private	Urban	Yes	No	No	Yes	No	Yes	No	No	No	No
5941	HSA1397	Male	Indian	2018	30	Married	Secondary	Private	Urban	Yes	No	Yes	No	Yes	No	No	No	No	No
5942	HSA1398	Male	Malay	2019	27	Married	Secondary	Private	Urban	Yes	Yes	No	No	No	No	No	No	No	Yes
5943	HSA1399	Male	Malay	2019	26	Single	Secondary	Private	Urban	Yes	Yes	No	No	Yes	No	No	No	No	No

No	Patient ID	Gender	Ethnic group	Year of diagnosis	Age	Marital status	Education level	Occupation	Place of residence	History of psy illness	Tobacco	Alcohol	Cannabis	ATS	Inhalant	Sedative	Hallucinogens	Opioids	Kratom
5944	HSA1400	Male	Indian	2018	70	Married	No formal education	Private	Urban	Yes	No	Yes	No	No	No	No	No	No	No
5945	HSA1401	Male	Malay	2018	30	Married	Secondary	Private	Urban	Yes	Yes	No	Yes	No	No	No	No	No	No
5946	HSA1402	Male	Others	2019	55	Married	No formal education	Unemployed	Rural	Yes	Yes	No	No	Yes	No	Yes	No	No	No
5947	HSA1403	Male	Malay	2018	21	Single	Tertiary	Unemployed	Urban	Yes	Yes	Yes	No	No	No	No	Yes	No	No
5948	HSA1404	Male	Chinese	2018	22	Single	Secondary	Unemployed	Urban	Yes	Yes	No	No	Yes	No	No	No	Yes	No
5949	HSA1405	Female	Indian	2018	15	Single	Secondary	Unemployed	Urban	No	Yes	No	No	No	No	No	No	No	No
5950	HSA1406	Male	Malay	2018	38	Single	No formal education	Unemployed	Rural	Yes	Yes	No	No	No	No	No	No	No	No
5951	HSA1407	Male	Chinese	2018	41	Single	No formal education	Unemployed	Urban	Yes	Yes	No	No	No	No	No	No	No	No
5952	HSA1408	Female	Malay	2020	19	Single	Secondary	Unemployed	Urban	Yes	Yes	No	No	No	No	No	No	No	No
5953	HSA1409	Female	Indian	2018	43	Married	No formal education	Private	Urban	Yes	No	Yes	No	No	No	No	No	No	No
5954	HSA1410	Male	Malay	2019	43	Others	No formal education	Unemployed	Urban	Yes	Yes	No	No	Yes	No	No	No	No	No
5955	HSA1411	Female	Indian	2020	26	Single	No formal education	Unemployed	Urban	Yes	Yes	No	No	No	No	No	No	No	No
5956	HSA1412	Male	Indian	2018	32	Married	No formal education	Others	Urban	No	No	Yes	No	No	No	Yes	No	No	No
5957	HSA1413	Female	Indian	2020	31	Married	Tertiary	Unemployed	Urban	Yes	Yes	No	No	No	No	No	No	No	No
5958	HSA1414	Female	Chinese	2018	66	Married	Secondary	Unemployed	Urban	No	No	Yes	No	No	No	No	No	No	No
5959	HSA1415	Female	Malay	2019	33	Others	Secondary	Private	Urban	Yes	Yes	Yes	Yes	Yes	No	No	No	No	Yes
5960	HSA1416	Male	Indian	2019	66	Married	No formal education	Unemployed	Urban	No	Yes	Yes	No	No	No	No	No	No	No
5961	HSA1417	Male	Malay	2018	45	Single	Secondary	Unemployed	Urban	Yes	Yes	Yes	No	No	No	Yes	No	No	No
5962	HSA1418	Male	Malay	2018	62	Married	No formal education	Unemployed	Rural	Yes	Yes	No	No	No	No	No	No	No	No
5963	HSA1419	Male	Indian	2018	45	Married	No formal education	Private	Urban	Yes	Yes	No	No	No	No	No	No	No	No
5964	HSA1420	Female	Indian	2018	29	Married	No formal education	Unemployed	Urban	Yes	No	No	No	Yes	No	No	No	No	No
5965	HSA1421	Male	Malay	2018	40	Married	Tertiary	Private	Urban	Yes	Yes	No	No	No	No	Yes	No	Yes	No
5966	HSA1422	Male	Malay	2019	37	Married	No formal education	Private	Urban	No	Yes	No	No	No	No	No	No	No	No
5967	HSA1423	Male	Malay	2019	29	Single	No formal education	Unemployed	Urban	Yes	No	No	Yes	No	No	No	No	No	No
5968	HSA1424	Male	Others	2018	22	Single	No formal education	Private	Urban	Yes	Yes	Yes	No	No	No	No	No	No	No
5969	HSA1425	Female	Indian	2019	39	Married	No formal education	Private	Urban	Yes	Yes	Yes	No	Yes	No	No	No	No	No
5970	HSA1426	Female	Chinese	2019	35	Others	Secondary	Private	Urban	Yes	Yes	Yes	No	Yes	No	No	No	No	No
5971	HSA1427	Female	Malay	2018	22	Single	Tertiary	Unemployed	Urban	Yes	Yes	Yes	Yes	No	No	No	No	No	No

No	Patient ID	Gender	Ethnic group	Year of diagnosis	Age	Marital status	Education level	Occupation	Place of residence	History of psy illness	Tobacco	Alcohol	Cannabis	ATS	Inhalant	Sedative	Hallucinogens	Opioids	Kratom
5972	HSA1428	Female	Indian	2018	21	Single	Tertiary	Private	Urban	Yes	Yes	Yes	No	No	No	No	No	No	No
5973	HSA1429	Female	Malay	2018	25	Single	Tertiary	Government	Urban	Yes	Yes	Yes	No	No	No	Yes	No	No	No
5974	HSA1430	Female	Indian	2019	78	Married	Primary	Unemployed	Urban	Yes	Yes	Yes	No	No	No	No	No	No	No
5975	HSA1432	Female	Indian	2020	34	Single	Tertiary	Private	Urban	Yes	Yes	No	Yes	No	No	No	No	No	Yes
5976	HSA1433	Female	Indian	2019	24	Single	No formal education	Unemployed	Urban	Yes	Yes	Yes	No	No	No	No	No	No	No
5977	HSA1434	Female	Indian	2019	28	Married	No formal education	Unemployed	Urban	Yes	No	Yes	No	No	No	No	No	No	No
5978	HSA1435	Female	Malay	2019	26	Married	No formal education	Private	Urban	Yes	No	Yes	No	No	No	No	No	No	No
5979	HSA1436	Female	Malay	2019	19	Single	Secondary	Private	Urban	Yes	Yes	No	No	No	No	No	No	Yes	No
5980	HSA1437	Female	Chinese	2020	27	Married	Tertiary	Private	Urban	Yes	No	Yes	No	No	No	No	No	No	No
5981	HSA1438	Female	Malay	2020	19	Single	Secondary	Private	Urban	Yes	Yes	Yes	No	No	No	No	No	No	No
5982	HSA1439	Female	Indian	2018	58	Others	No formal education	Unemployed	Urban	Yes	No	Yes	No	No	No	No	No	No	No
5983	HSA1440	Female	Malay	2018	35	Married	Secondary	Private	Urban	Yes	Yes	No	No	No	No	No	No	No	No
5984	HSA1441	Female	Malay	2018	31	Married	No formal education	Private	Urban	Yes	Yes	No	No	No	No	No	No	No	No
5985	HSA1442	Female	Indian	2020	37	Married	No formal education	Unemployed	Urban	Yes	Yes	No	No	No	No	No	No	No	No
5986	HSA1443	Female	Malay	2018	26	Others	No formal education	Others	Urban	Yes	Yes	No	No	No	No	No	No	No	No
5987	HSA1444	Female	Malay	2019	16	Single	Secondary	Unemployed	Urban	No	Yes	No	No	No	No	No	No	No	No
5988	HSA1447	Female	Malay	2020	26	Single	Tertiary	Private	Urban	Yes	Yes	No	No	No	No	No	No	No	No
5989	HSA1448	Female	Chinese	2020	39	Married	Secondary	Unemployed	Urban	Yes	Yes	No	No	No	No	No	Yes	No	No
5990	HSA1449	Female	Indian	2020	55	Others	Primary	Private	Urban	Yes	Yes	Yes	No	No	No	No	No	No	No
5991	HSA1450	Female	Malay	2019	24	Married	Secondary	Unemployed	Urban	Yes	No	No	No	Yes	No	No	No	No	No
5992	HSA1451	Female	Malay	2018	32	Single	Tertiary	Private	Urban	Yes	Yes	No	No	No	No	No	No	No	No
5993	HSA1453	Female	Malay	2020	25	Single	Secondary	Private	Urban	Yes	Yes	No	No	No	No	No	No	No	No
5994	HSA1454	Female	Malay	2018	23	Married	Secondary	Unemployed	Urban	Yes	No	No	No	Yes	No	No	No	No	No
5995	HSA1455	Female	Chinese	2021	17	Single	Secondary	Others	Urban	No	Yes	Yes	No	No	No	No	No	No	No
5996	HSA1456	Female	Malay	2019	16	Single	Secondary	Unemployed	Urban	Yes	Yes	No	No	No	No	No	No	No	No
5997	HSA1458	Female	Chinese	2021	20	Single	Secondary	Private	Urban	Yes	Yes	Yes	No	No	No	No	No	No	No
5998	HSA1460	Female	Chinese	2019	16	Single	Secondary	Unemployed	Urban	No	Yes	Yes	No	No	No	No	No	No	No
5999	HSA1461	Female	Indian	2018	20	Single	Secondary	Private	Urban	Yes	No	Yes	No	No	No	No	No	No	No
6000	HSA1462	Female	Malay	2019	24	Married	Tertiary	Private	Urban	Yes	Yes	No	No	No	No	No	No	No	Yes
6001	HSA1463	Female	Malay	2019	28	Married	No formal education	Private	Urban	Yes	Yes	Yes	No	No	No	No	No	No	No
6002	HSA1464	Female	Indian	2018	22	Single	No formal education	Private	Urban	Yes	Yes	No	No	No	No	No	No	No	No
6003	HSA1465	Female	Malay	2019	32	Others	Primary	Unemployed	Urban	Yes	Yes	No	No	Yes	No	No	No	Yes	No
6004	HSA1466	Female	Chinese	2020	37	Single	Primary	Unemployed	Urban	Yes	No	Yes	No	No	No	No	No	No	No
6005	HSA1467	Female	Chinese	2018	39	Married	No formal education	Private	Urban	Yes	No	Yes	No	No	No	No	No	No	No

No	Patient ID	Gender	Ethnic group	Year of diagnosis	Age	Marital status	Education level	Occupation	Place of residence	History of psy illness	Tobacco	Alcohol	Cannabis	ATS	Inhalant	Sedative	Hallucinogens	Opioids	Kratom
6006	HSA1468	Female	Malay	2020	19	Single	Secondary	Private	Urban	Yes	Yes	Yes	No	No	No	No	No	No	No
6007	HSA1469	Female	Malay	2020	24	Married	Secondary	Others	Rural	Yes	Yes	No	No	No	No	No	No	No	No
6008	HSA1470	Female	Indian	2018	25	Married	No formal education	Private	Urban	Yes	Yes	Yes	No	No	No	No	No	No	No
6009	HSA1471	Female	Others	2020	30	Married	No formal education	Others	Urban	Yes	No	Yes	No	No	No	No	No	No	No
6010	HSA1472	Female	Chinese	2018	63	Married	No formal education	Others	Urban	Yes	No	Yes	No	No	No	No	No	No	No
6011	HSA1473	Female	Malay	2019	28	Married	Primary	Others	Urban	Yes	Yes	No	No	No	No	No	No	No	No
6012	HSA1474	Female	Malay	2019	55	Married	No formal education	Private	Urban	Yes	No	No	No	Yes	No	No	No	No	No
6013	HSA1475	Female	Indian	2019	27	Single	Secondary	Unemployed	Urban	Yes	No	No	Yes	Yes	Yes	No	No	No	No
6014	HSA1476	Female	Chinese	2018	48	Others	Primary	Private	Urban	Yes	Yes	Yes	No	No	No	No	No	No	No
6015	HSA1477	Male	Indian	2019	41	Married	Secondary	Private	Urban	Yes	No	Yes	No	No	No	No	No	No	No
6016	HSA1479	Female	Malay	2020	17	Single	Secondary	Unemployed	Urban	Yes	Yes	No	No	No	No	No	No	No	No
6017	HSA1480	Male	Malay	2019	34	Others	Tertiary	Private	Urban	Yes	Yes	No	No	Yes	No	No	No	No	No
6018	HSA1481	Female	Chinese	2018	26	Single	Secondary	Unemployed	Urban	Yes	No	Yes	No	No	No	No	No	No	No
6019	HSA1482	Male	Malay	2020	23	Single	Tertiary	Private	Urban	Yes	Yes	No	No	No	No	No	No	No	No
6020	HSA1483	Male	Chinese	2020	44	Single	Secondary	Private	Urban	Yes	No	Yes	No	No	No	No	No	No	No
6021	HSA1484	Male	Chinese	2020	32	Others	Secondary	Private	Urban	Yes	Yes	No	No	No	No	No	No	No	No
6022	HSA1485	Female	Malay	2018	15	Single	Secondary	Unemployed	Rural	No	No	Yes	No	No	No	No	No	No	No
6023	HSA1486	Female	Indian	2019	20	Single	Tertiary	Private	Urban	Yes	Yes	Yes	No	No	No	No	No	No	No
6024	HSA1487	Male	Indian	2020	64	Married	No formal education	Others	Urban	Yes	No	Yes	No	No	No	No	No	No	No
6025	HSA1488	Male	Chinese	2020	27	Single	Secondary	Private	Urban	Yes	Yes	Yes	No	No	No	No	No	No	No
6026	HSA1489	Female	Indian	2018	49	Married	No formal education	Others	Urban	Yes	No	Yes	No	No	No	No	No	No	No
6027	HSA1490	Female	Chinese	2020	34	Single	No formal education	Private	Urban	Yes	Yes	Yes	No	No	No	No	No	No	No
6028	HSA1491	Male	Malay	2020	26	Single	Tertiary	Private	Urban	Yes	Yes	No	No	No	No	No	No	No	No
6029	HSA1492	Female	Malay	2018	16	Single	Secondary	Unemployed	Urban	No	Yes	No	No	No	No	No	No	No	No
6030	HSA1493	Female	Malay	2020	27	Married	Tertiary	Others	Urban	No	Yes	No	No	No	No	No	No	No	No
6031	HSA1494	Female	Chinese	2018	23	Single	Tertiary	Unemployed	Urban	Yes	Yes	No	No	No	No	No	No	No	No
6032	HSA1495	Female	Malay	2019	22	Others	Tertiary	Private	Urban	Yes	Yes	No	Yes	No	No	No	No	No	No
6033	HSA1496	Male	Chinese	2020	34	Single	No formal education	Private	Urban	Yes	Yes	No	No	No	No	No	No	No	No
6034	HSA1498	Female	Chinese	2019	48	Others	No formal education	Private	Urban	Yes	Yes	Yes	No	No	No	No	No	No	No
6035	HSA1499	Female	Chinese	2018	31	Married	Tertiary	Private	Urban	No	No	Yes	No	No	No	No	No	No	No
6036	HSA1500	Male	Chinese	2020	44	Single	Secondary	Private	Urban	Yes	Yes	Yes	No	No	No	No	No	No	No
6037	HSA1502	Male	Indian	2020	65	Married	Secondary	Unemployed	Urban	No	No	Yes	No	No	No	No	No	No	No
6038	HSA1503	Male	Malay	2019	36	Others	No formal education	Private	Urban	Yes	Yes	No	No	No	No	No	No	No	No

No	Patient ID	Gender	Ethnic group	Year of diagnosis	Age	Marital status	Education level	Occupation	Place of residence	History of psy illness	Tobacco	Alcohol	Cannabis	ATS	Inhalant	Sedative	Hallucinogens	Opioids	Kratom
6039	HSA1504	Male	Malay	2020	26	Married	Tertiary	Private	Urban	Yes	Yes	No	No	No	No	No	No	No	No
6040	HSA1505	Female	Indian	2019	26	Single	Secondary	Private	Urban	No	Yes	No	No	No	No	No	No	No	No
6041	HSA1506	Female	Chinese	2019	33	Single	No formal education	Private	Urban	Yes	No	Yes	No	No	No	No	No	No	No
6042	HSA1507	Female	Malay	2019	16	Single	Secondary	Unemployed	Urban	No	No	Yes	No	Yes	No	No	No	No	No
6043	HSA1508	Female	Others	2019	55	Married	Secondary	Others	Urban	Yes	No	Yes	No	No	No	No	No	No	No
6044	HSA1509	Female	Indian	2018	70	Others	No formal education	Others	Rural	No	Yes	No	No	No	No	No	No	No	No
6045	HSA1510	Female	Malay	2018	38	Married	Secondary	Unemployed	Urban	Yes	Yes	Yes	No	No	No	No	No	No	No
6046	HSA1511	Female	Indian	2019	33	Married	Tertiary	Unemployed	Urban	Yes	Yes	No	No	No	No	No	No	No	No
6047	HSA1512	Female	Chinese	2019	84	Married	No formal education	Others	Urban	No	Yes	Yes	No	No	No	No	No	No	No
6048	HSA1513	Female	Chinese	2018	38	Married	Secondary	Others	Urban	Yes	Yes	No	No	No	No	No	No	No	No
6049	HSA1514	Female	Malay	2019	25	Others	Secondary	Unemployed	Urban	Yes	No	No	No	Yes	No	No	No	No	No
6050	HSA1515	Female	Indian	2018	37	Married	No formal education	Unemployed	Urban	Yes	No	Yes	No	No	No	No	No	No	No
6051	HSA1516	Female	Others	2018	39	Married	No formal education	Unemployed	Urban	Yes	No	Yes	No	No	No	No	No	No	No
6052	HSA1517	Female	Malay	2018	15	Single	Secondary	Unemployed	Urban	No	Yes	No	No	No	No	No	No	No	No
6053	HSA1518	Female	Malay	2019	39	Others	Tertiary	Private	Urban	Yes	Yes	No	No	No	No	No	No	No	No
6054	HSA1519	Female	Others	2018	32	Single	No formal education	Private	Urban	Yes	No	Yes	No	No	No	No	No	No	No
6055	HSA1520	Female	Malay	2020	21	Single	Tertiary	Unemployed	Urban	Yes	Yes	No	No	No	No	No	No	No	No
6056	HSA1521	Male	Chinese	2020	26	Single	Secondary	Private	Urban	Yes	No	Yes	No	No	No	No	No	No	No
6057	HSA1522	Female	Malay	2018	17	Single	Primary	Unemployed	Urban	Yes	No	Yes	No	Yes	No	No	No	No	No
6058	HSA1523	Female	Chinese	2019	32	Others	Tertiary	Others	Urban	No	No	Yes	No	No	No	No	No	No	No
6059	HSA1525	Female	Indian	2019	22	Single	Tertiary	Unemployed	Urban	Yes	Yes	Yes	No	No	No	No	No	No	No
6060	HSA1526	Female	Chinese	2018	31	Single	Tertiary	Unemployed	Urban	Yes	No	Yes	No	No	No	No	No	No	No
6061	HSA1527	Male	Indian	2018	45	Married	Secondary	Private	Urban	Yes	Yes	Yes	No	No	No	No	No	No	No
6062	HSA1528	Female	Chinese	2018	33	Single	Secondary	Private	Urban	Yes	Yes	Yes	No	No	No	No	No	No	No
6063	HSA1529	Male	Indian	2018	31	Married	Secondary	Private	Urban	Yes	Yes	Yes	No	No	No	No	No	No	No
6064	HSA1530	Male	Chinese	2020	51	Married	No formal education	Private	Urban	Yes	Yes	No	No	No	No	No	No	No	No
6065	HSA1531	Female	Indian	2020	17	Single	Secondary	Unemployed	Urban	Yes	No	Yes	No	No	No	No	No	No	No
6066	HSA1532	Female	Malay	2018	17	Single	Secondary	Unemployed	Urban	No	Yes	No	No	No	No	No	No	No	No
6067	HSB0001	Male	Malay	2018	26	Single	Tertiary	Private	Rural	No	Yes	Yes	No	Yes	Yes	Yes	Yes	Yes	Yes
6068	HSB0002	Male	Malay	2018	26	Married	Secondary	Private	Rural	No	Yes	Yes	Yes	Yes	Yes	Yes	Yes	Yes	Yes
6069	HSB0003	Male	Malay	2018	37	Married	Secondary	Unemployed	Rural	No	Yes	Yes	Yes	Yes	Yes	Yes	Yes	Yes	Yes
6070	HSB0004	Male	Malay	2018	32	Single	Secondary	Unemployed	Rural	Yes	No	Yes	Yes	Yes	Yes	Yes	Yes	Yes	Yes
6071	HSB0005	Male	Malay	2018	34	Married	No formal education	Others	Rural	No	Yes	Yes	Yes	Yes	Yes	Yes	Yes	Yes	Yes
6072	HSB0006	Male	Malay	2018	38	Single	Secondary	Private	Rural	No	Yes	Yes	Yes	Yes	Yes	Yes	Yes	Yes	Yes
6073	HSB0007	Male	Malay	2018	48	Others	No formal education	Unemployed	Rural	No	Yes	Yes	Yes	Yes	Yes	Yes	Yes	Yes	Yes

No	Patient ID	Gender	Ethnic group	Year of diagnosis	Age	Marital status	Education level	Occupation	Place of residence	History of psy illness	Tobacco	Alcohol	Cannabis	ATS	Inhalant	Sedative	Hallucinogens	Opioids	Kratom
6074	HSB0008	Male	Malay	2018	42	Others	Secondary	Private	Rural	Yes	Yes	Yes	Yes	Yes	Yes	Yes	Yes	Yes	Yes
6075	HSB0009	Male	Malay	2018	30	Single	Tertiary	Private	Rural	No	Yes	Yes	Yes	Yes	Yes	Yes	Yes	Yes	Yes
6076	HSB0010	Male	Malay	2018	34	Single	Tertiary	Unemployed	Rural	No	Yes	Yes	Yes	Yes	Yes	Yes	Yes	Yes	Yes
6077	HSB0011	Male	Malay	2018	20	Single	Secondary	Private	Rural	No	Yes	Yes	Yes	Yes	Yes	Yes	Yes	Yes	Yes
6078	HSB0012	Male	Malay	2018	31	Single	Secondary	Unemployed	Rural	Yes	Yes	Yes	Yes	Yes	Yes	Yes	Yes	Yes	Yes
6079	HSB0013	Male	Malay	2019	20	Single	Secondary	Unemployed	Rural	No	Yes	Yes	Yes	Yes	Yes	Yes	Yes	Yes	Yes
6080	HSB0014	Male	Malay	2019	19	Single	Secondary	Unemployed	Rural	No	Yes	Yes	Yes	Yes	Yes	Yes	Yes	Yes	Yes
6081	HSB0015	Male	Malay	2019	43	Married	Secondary	Private	Rural	No	Yes	Yes	Yes	Yes	Yes	Yes	Yes	Yes	Yes
6082	HSB0016	Female	Malay	2019	23	Married	Tertiary	Government	Urban	No	Yes	Yes	Yes	Yes	Yes	Yes	Yes	Yes	Yes
6083	HSB0017	Male	Malay	2019	25	Single	Secondary	Self-employed	Rural	No	Yes	Yes	Yes	Yes	Yes	Yes	Yes	Yes	Yes
6084	HSB0018	Male	Malay	2019	28	Married	Tertiary	Government	Urban	No	Yes	Yes	Yes	Yes	Yes	Yes	Yes	Yes	Yes
6085	HSB0019	Male	Malay	2019	21	Single	Tertiary	Unemployed	Rural	No	Yes	Yes	Yes	Yes	Yes	Yes	Yes	Yes	Yes
6086	HSB0020	Male	Malay	2019	19	Single	Tertiary	Unemployed	Rural	No	Yes	Yes	Yes	Yes	Yes	Yes	Yes	Yes	Yes
6087	HSB0021	Male	Malay	2019	32	Married	Primary	Private	Rural	No	Yes	Yes	Yes	Yes	Yes	Yes	Yes	Yes	Yes
6088	HSB0022	Male	Others	2019	45	Single	Primary	Self-employed	Rural	No	Yes	Yes	Yes	Yes	Yes	Yes	Yes	Yes	Yes
6089	HSB0023	Male	Malay	2019	37	Single	Secondary	Private	Rural	No	Yes	Yes	Yes	Yes	Yes	Yes	Yes	Yes	Yes
6090	HSB0024	Male	Malay	2019	31	Others	Tertiary	Private	Rural	No	Yes	Yes	Yes	Yes	Yes	Yes	Yes	Yes	Yes
6091	HSB0025	Male	Malay	2019	39	Others	Secondary	Government	Rural	Yes	Yes	Yes	Yes	Yes	Yes	Yes	Yes	Yes	Yes
6092	HSB0026	Male	Malay	2019	31	Single	Secondary	Private	Rural	No	Yes	Yes	Yes	Yes	Yes	Yes	Yes	Yes	Yes
6093	HSB0027	Male	Malay	2019	54	Married	No formal education	Self-employed	Rural	No	Yes	Yes	Yes	Yes	Yes	Yes	Yes	Yes	Yes
6094	HSB0028	Female	Others	2019	27	Married	Secondary	Others	Rural	No	Yes	Yes	Yes	Yes	Yes	Yes	Yes	Yes	Yes
6095	HSB0029	Male	Malay	2019	25	Single	Secondary	Self-employed	Rural	No	Yes	Yes	Yes	Yes	Yes	Yes	Yes	Yes	Yes
6096	HSB0030	Male	Malay	2019	28	Single	Secondary	Unemployed	Rural	No	Yes	Yes	Yes	Yes	Yes	Yes	Yes	Yes	Yes
6097	HSB0031	Male	Malay	2020	40	Single	Secondary	Self-employed	Rural	No	No	No	No	Yes	No	No	No	Yes	No
6098	HSB0032	Male	Chinese	2020	35	Married	No formal education	Private	Urban	No	Yes	Yes	Yes	Yes	Yes	Yes	Yes	Yes	Yes
6099	HSB0033	Female	Malay	2020	25	Married	Tertiary	Self-employed	Rural	No	Yes	Yes	Yes	Yes	Yes	Yes	Yes	Yes	Yes
6100	HSB0034	Male	Malay	2020	42	Others	Secondary	Unemployed	Rural	No	Yes	Yes	Yes	Yes	Yes	Yes	Yes	Yes	Yes
6101	HSB0035	Male	Chinese	2020	27	Single	Secondary	Private	Rural	No	Yes	Yes	Yes	Yes	Yes	Yes	Yes	Yes	Yes
6102	HSB0036	Female	Malay	2020	38	Single	Tertiary	Private	Urban	No	Yes	Yes	Yes	Yes	Yes	Yes	Yes	Yes	Yes
6103	HSB0037	Male	Malay	2020	31	Single	Secondary	Unemployed	Rural	No	Yes	Yes	Yes	Yes	Yes	Yes	Yes	Yes	Yes
6104	HSB0038	Female	Malay	2020	27	Married	Secondary	Private	Rural	No	Yes	Yes	Yes	Yes	Yes	Yes	Yes	Yes	Yes
6105	HSB0039	Male	Malay	2020	26	Others	Secondary	Self-employed	Rural	No	Yes	Yes	Yes	Yes	Yes	Yes	Yes	Yes	Yes
6106	HSB0040	Male	Malay	2020	22	Single	Secondary	Unemployed	Rural	No	Yes	Yes	Yes	Yes	Yes	Yes	Yes	Yes	Yes
6107	HSB0041	Male	Malay	2020	31	Others	No formal education	Private	Rural	No	Yes	Yes	Yes	Yes	Yes	Yes	Yes	Yes	Yes
6108	HSB0042	Male	Malay	2018	29	Single	No formal education	Government	Rural	No	Yes	Yes	Yes	Yes	Yes	Yes	Yes	Yes	Yes
6109	HSB0043	Male	Malay	2018	23	Single	Secondary	Unemployed	Rural	No	Yes	Yes	Yes	Yes	Yes	Yes	Yes	Yes	Yes
6110	HSB0044	Male	Malay	2018	39	Married	Primary	Self-employed	Rural	No	No	Yes	Yes	Yes	Yes	Yes	Yes	Yes	Yes
6111	HSB0045	Male	Malay	2018	55	Married	Secondary	Self-employed	Rural	No	No	Yes	Yes	Yes	Yes	Yes	Yes	Yes	Yes

No	Patient ID	Gender	Ethnic group	Year of diagnosis	Age	Marital status	Education level	Occupation	Place of residence	History of psy illness	Tobacco	Alcohol	Cannabis	ATS	Inhalant	Sedative	Hallucinogens	Opioids	Kratom
6112	HSB0047	Male	Malay	2018	21	Single	Secondary	Unemployed	Rural	No	Yes	Yes	Yes	Yes	Yes	Yes	Yes	Yes	Yes
6113	HSB0049	Male	Malay	2018	65	Others	No formal education	Others	Rural	Yes	Yes	Yes	Yes	Yes	Yes	Yes	Yes	Yes	Yes
6114	HSB0050	Male	Malay	2018	32	Single	Secondary	Private	Rural	No	Yes	Yes	Yes	Yes	Yes	Yes	Yes	Yes	Yes
6115	HSB0051	Male	Malay	2018	31	Others	Secondary	Private	Rural	No	Yes	Yes	Yes	Yes	Yes	Yes	Yes	Yes	Yes
6116	HSB0052	Female	Malay	2018	18	Single	No formal education	Others	Rural	No	No	Yes	Yes	Yes	Yes	Yes	Yes	Yes	Yes
6117	HSB0053	Male	Malay	2018	37	Others	Secondary	Private	Rural	No	No	Yes	Yes	Yes	Yes	Yes	Yes	Yes	Yes
6118	HSB0054	Male	Malay	2018	34	Married	Tertiary	Government	Urban	No	No	Yes	Yes	Yes	Yes	Yes	Yes	Yes	Yes
6119	HSB0055	Male	Malay	2018	42	Others	Secondary	Others	Rural	No	No	Yes	Yes	Yes	Yes	Yes	Yes	Yes	Yes
6120	HSB0056	Male	Malay	2018	22	Single	Tertiary	Private	Rural	No	Yes	Yes	Yes	Yes	Yes	Yes	Yes	Yes	Yes
6121	HSB0057	Male	Chinese	2018	45	Others	No formal education	Government	Rural	No	No	Yes	Yes	Yes	Yes	Yes	Yes	Yes	Yes
6122	HSB0058	Male	Malay	2018	43	Single	Secondary	Private	Rural	Yes	Yes	Yes	Yes	Yes	Yes	Yes	Yes	Yes	Yes
6123	HSB0058	Male	Malay	2018	40	Single	Secondary	Self-employed	Rural	No	Yes	Yes	Yes	Yes	Yes	Yes	Yes	Yes	Yes
6124	HSB0059	Male	Others	2018	24	Single	Secondary	Self-employed	Rural	No	Yes	Yes	Yes	Yes	Yes	Yes	Yes	Yes	Yes
6125	HSB0060	Male	Malay	2019	28	Single	Tertiary	Self-employed	Rural	No	Yes	Yes	Yes	Yes	Yes	Yes	Yes	Yes	Yes
6126	HSB0061	Male	Malay	2019	30	Others	Tertiary	Government	Urban	No	Yes	Yes	Yes	Yes	Yes	Yes	Yes	Yes	Yes
6127	HSB0062	Male	Malay	2019	28	Single	Tertiary	Government	Rural	Yes	Yes	Yes	Yes	Yes	Yes	Yes	Yes	Yes	Yes
6128	HSB0063	Male	Indian	2019	49	Married	Secondary	Unemployed	Urban	No	Yes	Yes	Yes	Yes	Yes	Yes	Yes	Yes	Yes
6129	HSB0064	Male	Malay	2019	34	Married	Secondary	Private	Urban	Yes	Yes	Yes	Yes	Yes	Yes	Yes	Yes	Yes	Yes
6130	HSB0065	Male	Malay	2019	37	Single	No formal education	Private	Rural	No	Yes	Yes	Yes	Yes	Yes	Yes	Yes	Yes	Yes
6131	HSB0066	Male	Malay	2019	59	Married	Secondary	Others	Rural	Yes	Yes	Yes	Yes	Yes	Yes	Yes	Yes	Yes	Yes
6132	HSB0067	Male	Others	2019	35	Others	No formal education	Self-employed	Rural	No	Yes	Yes	Yes	Yes	Yes	Yes	Yes	Yes	Yes
6133	HSB0068	Male	Malay	2019	29	Single	Secondary	Government	Urban	No	Yes	Yes	Yes	Yes	Yes	Yes	Yes	Yes	Yes
6134	HSB0070	Male	Malay	2019	35	Others	Secondary	Unemployed	Rural	No	Yes	Yes	Yes	Yes	Yes	Yes	Yes	Yes	Yes
6135	HSB0071	Male	Malay	2019	37	Married	No formal education	Government	Rural	No	Yes	Yes	Yes	Yes	Yes	Yes	Yes	Yes	Yes
6136	HSB0072	Male	Malay	2019	30	Single	Secondary	Self-employed	Rural	No	Yes	Yes	Yes	Yes	Yes	Yes	Yes	Yes	Yes
6137	HSB0073	Female	Malay	2019	24	Married	Secondary	Unemployed	Rural	No	Yes	Yes	Yes	Yes	Yes	Yes	Yes	Yes	Yes
6138	HSB0074	Male	Malay	2019	29	Others	No formal education	Unemployed	Urban	No	Yes	Yes	Yes	Yes	Yes	Yes	Yes	Yes	Yes
6139	HSB0075	Female	Malay	2019	35	Married	Secondary	Unemployed	Urban	No	Yes	Yes	Yes	Yes	Yes	Yes	Yes	Yes	Yes
6140	HSB0076	Male	Malay	2019	41	Single	Secondary	Self-employed	Rural	No	Yes	Yes	Yes	Yes	Yes	Yes	Yes	Yes	Yes
6141	HSB0077	Male	Malay	2019	42	Others	No formal education	Unemployed	Urban	Yes	Yes	Yes	Yes	Yes	Yes	Yes	Yes	Yes	Yes

No	Patient ID	Gender	Ethnic group	Year of diagnosis	Age	Marital status	Education level	Occupation	Place of residence	History of psy illness	Tobacco	Alcohol	Cannabis	ATS	Inhalant	Sedative	Hallucinogens	Opioids	Kratom
6142	HSB0078	Male	Malay	2019	16	Single	Secondary	Unemployed	Rural	No	Yes	Yes	Yes	Yes	Yes	Yes	Yes	Yes	Yes
6143	HSB0079	Male	Others	2019	15	Single	Secondary	Unemployed	Rural	No	Yes	Yes	Yes	Yes	Yes	Yes	Yes	Yes	Yes
6144	HSB0080	Male	Malay	2020	34	Married	Secondary	Self-employed	Rural	No	Yes	Yes	Yes	Yes	Yes	Yes	Yes	Yes	Yes
6145	HSB0081	Male	Indian	2020	48	Single	Secondary	Unemployed	Rural	No	Yes	Yes	Yes	Yes	Yes	Yes	Yes	Yes	Yes
6146	HSB0082	Male	Malay	2020	45	Married	Secondary	Government	Rural	No	Yes	No	No	No	No	No	No	No	Yes
6147	HSB0083	Male	Malay	2020	36	Single	No formal education	Unemployed	Rural	No	No	No	No	Yes	No	No	No	No	No
6148	HSB0084	Male	Malay	2020	38	Others	Tertiary	Private	Rural	No	Yes	Yes	Yes	Yes	Yes	Yes	Yes	Yes	Yes
6149	HSB0085	Male	Malay	2020	27	Single	Secondary	Unemployed	Rural	No	Yes	Yes	Yes	Yes	Yes	Yes	Yes	Yes	Yes
6150	HSB0086	Male	Malay	2020	32	Others	Secondary	Unemployed	Rural	No	Yes	Yes	Yes	Yes	Yes	Yes	Yes	Yes	Yes
6151	HSB0087	Male	Malay	2020	27	Others	No formal education	Unemployed	Rural	Yes	Yes	Yes	Yes	Yes	Yes	Yes	Yes	Yes	Yes
6152	HSB0088	Male	Malay	2020	21	Single	Secondary	Self-employed	Rural	No	Yes	Yes	Yes	Yes	Yes	Yes	Yes	Yes	Yes
6153	HSB0090	Male	Chinese	2020	54	Single	Secondary	Self-employed	Urban	No	Yes	Yes	Yes	Yes	Yes	Yes	Yes	Yes	Yes
6154	HSB0091	Male	Malay	2020	29	Single	Secondary	Unemployed	Rural	No	Yes	Yes	Yes	Yes	Yes	Yes	Yes	Yes	Yes
6155	HSB0092	Male	Malay	2020	31	Single	Secondary	Unemployed	Urban	Yes	Yes	Yes	Yes	Yes	Yes	Yes	Yes	Yes	Yes
6156	HSB0093	Male	Malay	2020	29	Married	Secondary	Others	Rural	Yes	Yes	Yes	Yes	Yes	Yes	Yes	Yes	Yes	Yes
6157	HSB0094	Male	Malay	2020	34	Single	Secondary	Self-employed	Rural	No	Yes	No	No	No	No	No	No	No	Yes
6158	HSB0095	Male	Malay	2020	33	Married	Secondary	Self-employed	Rural	No	Yes	No	No	Yes	No	No	No	No	No
6159	HSB0096	Male	Malay	2020	33	Single	Tertiary	Unemployed	Urban	Yes	No	No	No	No	No	No	No	No	Yes
6160	HSB0097	Male	Malay	2018	41	Married	Secondary	Unemployed	Rural	No	Yes	No	No	Yes	No	No	No	No	Yes
6161	HSB0098	Male	Chinese	2020	25	Single	Secondary	Unemployed	Urban	No	No	No	No	Yes	No	No	No	Yes	No
6162	HSB0099	Male	Malay	2020	48	Others	Secondary	Unemployed	Rural	Yes	Yes	No	No	Yes	No	No	No	No	No
6163	HSB0100	Male	Malay	2020	28	Single	Secondary	Unemployed	Rural	No	Yes	Yes	Yes	Yes	Yes	Yes	Yes	Yes	Yes
6164	HSB0101	Male	Malay	2020	42	Others	Tertiary	Private	Rural	No	Yes	Yes	Yes	Yes	Yes	Yes	Yes	Yes	Yes
6165	HSB0102	Male	Malay	2020	41	Others	No formal education	Others	Rural	No	Yes	Yes	Yes	Yes	Yes	Yes	Yes	Yes	Yes
6166	HSB0103	Male	Indian	2020	17	Single	Secondary	Private	Urban	No	Yes	Yes	Yes	Yes	Yes	Yes	Yes	Yes	Yes
6167	HSB0104	Female	Malay	2019	53	Others	No formal education	Unemployed	Urban	No	No	Yes	Yes	Yes	Yes	Yes	Yes	Yes	Yes
6168	HSB0105	Male	Malay	2020	40	Others	No formal education	Private	Urban	No	Yes	Yes	Yes	Yes	Yes	Yes	Yes	Yes	Yes
6169	HSB0106	Male	Malay	2020	31	Others	Tertiary	Unemployed	Urban	Yes	Yes	Yes	Yes	Yes	Yes	Yes	Yes	Yes	Yes
6170	HSB0107	Male	Malay	2020	37	Others	Secondary	Unemployed	Urban	Yes	Yes	Yes	Yes	Yes	Yes	Yes	Yes	Yes	Yes
6171	HSB0108	Male	Malay	2020	20	Single	Secondary	Unemployed	Rural	No	Yes	Yes	Yes	Yes	Yes	Yes	Yes	Yes	Yes
6172	HSB0109	Male	Indian	2020	47	Others	Tertiary	Unemployed	Urban	Yes	Yes	Yes	Yes	Yes	Yes	Yes	Yes	Yes	Yes
6173	HSB0110	Male	Malay	2020	27	Single	Primary	Private	Rural	No	Yes	Yes	Yes	Yes	Yes	Yes	Yes	Yes	Yes
6174	HSB0111	Male	Malay	2018	35	Married	Tertiary	Government	Urban	No	Yes	No	Yes	No	No	No	No	No	No
6175	HSB0112	Male	Malay	2020	20	Single	Tertiary	Private	Urban	No	Yes	Yes	Yes	Yes	Yes	Yes	Yes	Yes	Yes
6176	HSB0113	Male	Malay	2020	19	Single	Secondary	Unemployed	Rural	No	Yes	Yes	Yes	Yes	Yes	Yes	Yes	Yes	Yes

No	Patient ID	Gender	Ethnic group	Year of diagnosis	Age	Marital status	Education level	Occupation	Place of residence	History of psy illness	Tobacco	Alcohol	Cannabis	ATS	Inhalant	Sedative	Hallucinogens	Opioids	Kratom
6177	HSB0114	Male	Malay	2020	36	Single	No formal education	Unemployed	Rural	No	Yes	Yes	Yes	Yes	Yes	Yes	Yes	Yes	Yes
6178	HSB0115	Male	Malay	2020	41	Married	Tertiary	Private	Urban	No	Yes	Yes	Yes	Yes	Yes	Yes	Yes	Yes	Yes
6179	HSB0116	Male	Malay	2021	29	Single	Tertiary	Unemployed	Rural	No	Yes	Yes	Yes	Yes	Yes	Yes	Yes	Yes	Yes
6180	HSB0117	Male	Malay	2020	36	Married	Secondary	Self-employed	Rural	No	Yes	Yes	Yes	Yes	Yes	Yes	Yes	Yes	Yes
6181	HSB0118	Male	Indian	2020	42	Married	Secondary	Private	Rural	No	Yes	Yes	Yes	Yes	Yes	Yes	Yes	Yes	Yes
6182	HSB0119	Male	Malay	2020	54	Others	Secondary	Self-employed	Rural	No	Yes	Yes	Yes	Yes	Yes	Yes	Yes	Yes	Yes
6183	HSB0121	Male	Malay	2020	28	Single	Tertiary	Self-employed	Rural	No	Yes	Yes	Yes	Yes	Yes	Yes	Yes	Yes	Yes
6184	HSB0122	Male	Malay	2020	60	Married	Tertiary	Government	Urban	No	Yes	Yes	Yes	Yes	Yes	Yes	Yes	Yes	Yes
6185	HSB0123	Male	Malay	2020	32	Single	Secondary	Private	Rural	No	Yes	Yes	Yes	Yes	Yes	Yes	Yes	Yes	Yes
6186	HSB0125	Male	Malay	2018	27	Single	Secondary	Unemployed	Urban	No	Yes	No	Yes	Yes	No	No	No	Yes	No
6187	HSB0126	Male	Malay	2018	31	Others	No formal education	Private	Rural	No	No	Yes	Yes	Yes	Yes	Yes	Yes	Yes	Yes
6188	HSB0127	Male	Malay	2018	18	Single	Secondary	Unemployed	Rural	Yes	Yes	Yes	Yes	Yes	Yes	Yes	Yes	Yes	Yes
6189	HSB0128	Male	Others	2018	52	Single	No formal education	Self-employed	Rural	No	Yes	Yes	Yes	Yes	Yes	Yes	Yes	Yes	Yes
6190	HSB0129	Male	Malay	2018	34	Married	No formal education	Unemployed	Rural	No	Yes	No	No	Yes	No	Yes	No	No	No
6191	HSB0130	Male	Malay	2018	35	Married	No formal education	Self-employed	Urban	No	No	Yes	Yes	Yes	Yes	Yes	Yes	Yes	Yes
6192	HSB0131	Male	Malay	2018	29	Married	Tertiary	Private	Rural	No	Yes	Yes	Yes	Yes	Yes	Yes	Yes	Yes	Yes
6193	HSB0132	Male	Indian	2018	47	Others	No formal education	Unemployed	Urban	Yes	No	Yes	No	No	No	No	No	No	Yes
6194	HSB0133	Male	Others	2018	43	Married	No formal education	Self-employed	Rural	No	No	Yes	Yes	Yes	Yes	Yes	Yes	Yes	Yes
6195	HSB0134	Male	Malay	2018	36	Others	Tertiary	Unemployed	Urban	No	No	Yes	Yes	Yes	Yes	Yes	Yes	Yes	Yes
6196	HSB0135	Male	Malay	2018	29	Others	Secondary	Self-employed	Rural	No	No	Yes	Yes	Yes	Yes	Yes	Yes	Yes	Yes
6197	HSB0136	Male	Malay	2018	69	Married	Tertiary	Private	Urban	Yes	No	Yes	Yes	Yes	Yes	Yes	Yes	Yes	Yes
6198	HSB0137	Male	Malay	2018	24	Single	Tertiary	Private	Urban	No	No	No	No	Yes	No	No	No	No	No
6199	HSB0138	Male	Malay	2019	28	Single	No formal education	Unemployed	Urban	No	Yes	Yes	Yes	Yes	Yes	Yes	Yes	Yes	Yes
6200	HSB0139	Male	Malay	2018	40	Married	No formal education	Private	Urban	No	No	Yes	Yes	Yes	Yes	Yes	Yes	Yes	Yes
6201	HSB0140	Male	Chinese	2018	44	Married	Secondary	Private	Urban	No	No	Yes	Yes	Yes	Yes	Yes	Yes	Yes	Yes
6202	HSB0141	Male	Chinese	2018	39	Others	No formal education	Others	Rural	No	No	Yes	Yes	Yes	Yes	Yes	Yes	Yes	Yes
6203	HSB0142	Male	Malay	2018	37	Married	Secondary	Self-employed	Urban	No	No	Yes	Yes	Yes	Yes	Yes	Yes	Yes	Yes
6204	HSB0143	Male	Malay	2018	42	Married	Tertiary	Government	Urban	Yes	No	Yes	Yes	Yes	Yes	Yes	Yes	Yes	Yes
6205	HSB0144	Male	Chinese	2018	38	Single	Secondary	Self-employed	Urban	No	Yes	Yes	Yes	Yes	Yes	Yes	Yes	Yes	Yes
6206	HSB0145	Male	Indian	2018	42	Single	Primary	Others	Rural	No	Yes	Yes	Yes	Yes	Yes	Yes	Yes	Yes	Yes
6207	HSB0146	Male	Malay	2018	30	Single	Secondary	Private	Urban	Yes	Yes	Yes	Yes	Yes	Yes	Yes	Yes	Yes	Yes
6208	HSB0147	Male	Malay	2018	22	Single	Tertiary	Unemployed	Rural	Yes	Yes	Yes	Yes	Yes	Yes	Yes	Yes	Yes	Yes

No	Patient ID	Gender	Ethnic group	Year of diagnosis	Age	Marital status	Education level	Occupation	Place of residence	History of psy illness	Tobacco	Alcohol	Cannabis	ATS	Inhalant	Sedative	Hallucinogens	Opioids	Kratom
6209	HSB0148	Male	Malay	2018	39	Single	No formal education	Unemployed	Rural	Yes	Yes	No	No	No	No	No	No	No	No
6210	HSB0150	Male	Malay	2018	19	Single	Secondary	Unemployed	Urban	Yes	Yes	No	No	Yes	No	Yes	No	No	No
6211	HSB0151	Male	Malay	2018	32	Married	Secondary	Unemployed	Rural	No	Yes	No	Yes	Yes	No	No	No	Yes	Yes
6212	HSB0152	Male	Malay	2018	29	Single	Tertiary	Self-employed	Rural	No	No	No	No	Yes	No	No	No	Yes	No
6213	HSB0153	Male	Malay	2019	24	Single	Secondary	Private	Rural	No	Yes	Yes	No	Yes	No	No	No	No	No
6214	HSB0154	Male	Malay	2019	30	Married	No formal education	Government	Rural	No	No	No	No	No	No	No	No	No	Yes
6215	HSB0155	Male	Malay	2019	36	Others	Secondary	Government	Urban	No	Yes	No	No	Yes	No	No	No	No	No
6216	HSB0156	Male	Malay	2019	29	Married	Tertiary	Government	Rural	No	No	No	Yes	No	No	No	No	No	No
6217	HSB0157	Male	Malay	2019	27	Others	Tertiary	Government	Rural	No	Yes	Yes	No	No	No	No	No	No	No
6218	HSB0159	Male	Malay	2019	35	Others	No formal education	Self-employed	Rural	No	No	No	No	No	No	No	No	Yes	No
6219	HSB0160	Male	Malay	2019	39	Single	Secondary	Unemployed	Rural	No	Yes	No	No	Yes	No	No	No	Yes	Yes
6220	HSB0161	Male	Malay	2019	28	Married	Tertiary	Government	Rural	No	Yes	No	No	Yes	No	No	No	No	Yes
6221	HSB0162	Male	Malay	2019	20	Single	No formal education	Private	Rural	No	Yes	No	No	No	No	No	No	No	Yes
6222	HSB0163	Male	Malay	2019	29	Single	Secondary	Self-employed	Rural	No	No	No	No	Yes	No	No	No	Yes	Yes
6223	HSB0164	Male	Malay	2019	21	Single	Tertiary	Unemployed	Urban	No	No	No	No	Yes	No	No	No	No	No
6224	HSB0165	Male	Chinese	2019	35	Single	Secondary	Private	Rural	Yes	Yes	Yes	No	No	No	Yes	No	No	No
6225	HSB0166	Male	Chinese	2020	40	Single	No formal education	Private	Urban	No	Yes	Yes	No	No	No	Yes	No	No	No
6226	HSB0167	Female	Malay	2020	34	Married	No formal education	Unemployed	Rural	No	No	No	No	No	No	Yes	No	No	No
6227	HSB0168	Male	Malay	2020	29	Single	Secondary	Private	Urban	No	No	No	No	Yes	No	No	No	No	Yes
6228	HSB0169	Male	Malay	2020	27	Single	Secondary	Private	Rural	No	Yes	No	No	Yes	No	No	No	No	Yes
6229	HSB0170	Male	Malay	2020	31	Others	Secondary	Unemployed	Rural	No	Yes	No	No	No	No	Yes	No	No	No
6230	HSB0171	Male	Chinese	2020	25	Single	Secondary	Private	Urban	No	Yes	No	No	No	No	No	No	No	Yes
6231	HSB0172	Male	Malay	2018	34	Single	Secondary	Private	Urban	Yes	Yes	Yes	Yes	Yes	Yes	Yes	Yes	Yes	Yes
6232	HSB0172	Male	Malay	2018	31	Single	Secondary	Unemployed	Urban	No	Yes	Yes	Yes	Yes	Yes	Yes	Yes	Yes	Yes
6233	HSB0173	Female	Malay	2021	34	Single	Secondary	Unemployed	Urban	No	Yes	Yes	Yes	Yes	Yes	Yes	Yes	Yes	Yes
6234	HSB0174	Female	Malay	2021	28	Married	Secondary	Self-employed	Urban	No	Yes	Yes	Yes	Yes	Yes	Yes	Yes	Yes	Yes
6235	HSB0175	Female	Malay	2021	24	Others	Secondary	Unemployed	Urban	No	Yes	Yes	Yes	Yes	Yes	Yes	Yes	Yes	Yes
6236	HSB0176	Female	Malay	2021	41	Single	No formal education	Unemployed	Urban	No	Yes	No	No	Yes	No	No	No	No	No
6237	HSB0177	Female	Malay	2021	31	Married	No formal education	Unemployed	Urban	No	Yes	No	No	No	No	No	No	No	No
6238	HSB0178	Female	Malay	2020	36	Married	Secondary	Private	Rural	No	Yes	No	No	Yes	No	No	No	No	No
6239	HSB0179	Female	Malay	2021	35	Others	Secondary	Unemployed	Urban	No	Yes	No	No	Yes	No	No	No	No	No
6240	HSB0180	Female	Malay	2020	23	Others	No formal education	Unemployed	Urban	No	Yes	No	No	Yes	No	No	No	No	No

No	Patient ID	Gender	Ethnic group	Year of diagnosis	Age	Marital status	Education level	Occupation	Place of residence	History of psy illness	Tobacco	Alcohol	Cannabis	ATS	Inhalant	Sedative	Hallucinogens	Opioids	Kratom
6241	HSB0181	Female	Malay	2020	22	Others	Secondary	Private	Rural	No	No	No	No	Yes	No	No	No	No	No
6242	HSB0182	Female	Malay	2020	18	Others	Secondary	Unemployed	Urban	No	Yes	No	No	No	No	No	No	No	No
6243	HSB0183	Female	Malay	2020	28	Others	Secondary	Unemployed	Rural	No	Yes	No	No	Yes	No	No	No	No	No
6244	HSB0184	Female	Malay	2020	21	Married	Secondary	Unemployed	Rural	No	Yes	No	No	Yes	No	No	No	No	No
6245	HSB0185	Female	Malay	2020	28	Others	Secondary	Unemployed	Urban	No	Yes	No	No	Yes	No	No	No	No	No
6246	HSB0186	Female	Others	2020	23	Others	Secondary	Private	Rural	No	No	No	No	Yes	No	No	No	No	No
6247	HSB0187	Female	Malay	2019	31	Married	Primary	Unemployed	Rural	No	Yes	No	No	Yes	No	No	No	Yes	No
6248	HSB0188	Male	Malay	2019	39	Married	Secondary	Private	Rural	Yes	Yes	No	No	Yes	No	No	No	Yes	No
6249	HSB0189	Male	Malay	2020	27	Single	Secondary	Self-employed	Rural	No	Yes	No	No	Yes	No	No	No	Yes	Yes
6250	HSB0190	Female	Malay	2020	21	Others	Secondary	Unemployed	Rural	No	Yes	No	No	Yes	No	No	No	No	No
6251	HSB0191	Female	Malay	2020	23	Single	Tertiary	Private	Rural	No	Yes	No	Yes	Yes	No	No	No	No	No
6252	HSB0192	Male	Malay	2020	31	Others	Secondary	Unemployed	Rural	No	No	No	No	No	No	No	No	Yes	Yes
6253	HSB0193	Female	Malay	2020	18	Others	Secondary	Private	Rural	No	Yes	No	No	Yes	No	No	No	No	No
6254	HSB0194	Female	Malay	2020	25	Single	Primary	Others	Urban	No	Yes	No	No	No	Yes	No	No	No	No
6255	HSB0196	Male	Malay	2018	33	Single	Secondary	Private	Rural	No	No	No	Yes	Yes	No	No	No	Yes	No
6256	HSB0199	Male	Malay	2020	22	Single	Tertiary	Unemployed	Rural	No	No	No	No	Yes	No	No	No	No	No
6257	HSB0200	Male	Malay	2018	30	Married	Primary	Self-employed	Rural	No	Yes	No	No	No	No	No	No	No	Yes
6258	HSB0201	Female	Malay	2021	34	Single	Secondary	Others	Rural	No	Yes	No	No	Yes	No	No	No	No	Yes
6259	HSB0202	Male	Malay	2018	41	Others	Tertiary	Government	Urban	No	Yes	No	No	No	No	No	No	Yes	Yes
6260	HSB0203	Male	Malay	2018	22	Others	Tertiary	Unemployed	Rural	No	Yes	No	No	No	No	No	No	Yes	Yes
6261	HSB0204	Male	Malay	2018	35	Others	Tertiary	Government	Rural	Yes	Yes	No	Yes	Yes	No	No	No	No	Yes
6262	HSB0206	Male	Malay	2018	30	Single	Secondary	Self-employed	Urban	Yes	Yes	No	Yes	Yes	No	No	No	No	Yes
6263	HSB0207	Male	Malay	2018	40	Single	Secondary	Self-employed	Urban	No	No	No	No	Yes	No	No	No	Yes	No
6264	HSB0208	Male	Malay	2018	33	Married	Primary	Private	Rural	No	Yes	No	No	No	No	No	No	No	Yes
6265	HSB0209	Female	Malay	2018	20	Married	Tertiary	Private	Rural	No	No	No	No	Yes	No	No	No	Yes	No
6266	HSB0210	Male	Malay	2018	27	Single	Secondary	Unemployed	Urban	No	Yes	No	No	Yes	No	No	No	No	Yes
6267	HSB0211	Male	Chinese	2018	51	Single	Secondary	Private	Rural	No	No	No	Yes	No	No	No	No	Yes	No
6268	HSB0213	Male	Chinese	2018	59	Others	No formal education	Others	Rural	No	No	No	No	Yes	No	No	No	Yes	No
6269	HSB0214	Male	Malay	2018	37	Single	Secondary	Self-employed	Rural	No	Yes	No	No	No	No	No	No	Yes	No
6270	HSB0215	Male	Malay	2018	36	Others	Secondary	Private	Rural	Yes	No	No	No	Yes	No	No	No	Yes	No
6271	HSB0216	Male	Malay	2018	47	Married	Secondary	Self-employed	Rural	No	Yes	No	No	Yes	No	No	No	Yes	No
6272	HSB0217	Male	Malay	2018	24	Others	No formal education	Self-employed	Rural	No	Yes	No	No	No	No	No	No	No	Yes
6273	HSB0219	Male	Chinese	2018	34	Married	Secondary	Private	Urban	No	No	No	No	Yes	No	No	No	No	No
6274	HSB0220	Male	Malay	2018	36	Single	Secondary	Others	Rural	No	No	No	No	Yes	No	No	No	Yes	No
6275	HSB0221	Male	Malay	2018	53	Others	Primary	Self-employed	Rural	No	Yes	No	Yes	No	No	No	No	Yes	No
6276	HSB0222	Male	Malay	2018	37	Others	Secondary	Self-employed	Rural	No	No	No	No	Yes	No	No	No	No	No
6277	HSB0223	Male	Chinese	2018	39	Single	Primary	Self-employed	Urban	Yes	No	Yes	No	No	No	No	No	No	No
6278	HSB0224	Female	Malay	2019	25	Single	Tertiary	Private	Urban	Yes	Yes	No	No	No	No	No	No	No	No

No	Patient ID	Gender	Ethnic group	Year of diagnosis	Age	Marital status	Education level	Occupation	Place of residence	History of psy illness	Tobacco	Alcohol	Cannabis	ATS	Inhalant	Sedative	Hallucinogens	Opioids	Kratom
6279	HSB0225	Male	Chinese	2019	34	Married	Secondary	Private	Urban	No	No	Yes	No	No	No	No	No	No	No
6280	HSB0226	Male	Malay	2020	45	Married	No formal education	Unemployed	Rural	Yes	No	No	No	Yes	No	No	No	No	No
6281	HSB0227	Male	Malay	2019	35	Married	Secondary	Private	Rural	No	Yes	No	No	No	No	No	No	No	Yes
6282	HSB0228	Male	Malay	2020	19	Others	Secondary	Others	Rural	No	No	No	No	Yes	No	No	No	No	No
6283	HSB0229	Male	Malay	2019	28	Single	Secondary	Unemployed	Urban	Yes	Yes	No	Yes	Yes	No	No	No	Yes	No
6284	HSB0230	Male	Malay	2019	34	Others	No formal education	Unemployed	Rural	No	No	No	No	Yes	No	No	No	No	No
6285	HSB0231	Male	Malay	2020	41	Single	No formal education	Private	Rural	No	No	No	Yes	Yes	No	No	No	Yes	No
6286	HSB0232	Male	Malay	2019	37	Single	Secondary	Private	Urban	Yes	Yes	No	No	Yes	No	No	No	Yes	No
6287	HSB0233	Male	Malay	2019	44	Married	Secondary	Self-employed	Rural	No	No	No	No	Yes	No	No	No	No	Yes
6288	HSB0234	Male	Malay	2020	33	Single	Secondary	Private	Urban	No	No	No	No	No	No	No	No	Yes	No
6289	HSB0235	Male	Malay	2021	43	Others	No formal education	Others	Urban	No	No	No	No	Yes	No	No	No	No	No
6290	HSB0236	Male	Malay	2019	33	Others	Tertiary	Private	Rural	No	No	No	No	No	No	Yes	No	Yes	No
6291	HSB0237	Female	Malay	2019	32	Married	Secondary	Others	Rural	Yes	No	No	No	No	No	No	No	No	No
6292	HSB0238	Male	Chinese	2019	41	Others	No formal education	Others	Urban	Yes	No	Yes	No	No	No	Yes	No	No	No
6293	HSB0239	Male	Malay	2018	40	Married	No formal education	Private	Rural	No	No	No	No	Yes	No	No	No	No	No
6294	HSB0240	Male	Chinese	2018	49	Others	No formal education	Unemployed	Urban	Yes	Yes	Yes	Yes	Yes	No	No	No	No	No
6295	HSB0241	Male	Malay	2019	31	Single	Secondary	Self-employed	Rural	No	Yes	No	Yes	Yes	No	No	No	No	Yes
6296	HSB0242	Male	Malay	2018	34	Single	Secondary	Private	Rural	No	No	No	Yes	Yes	No	No	No	No	No
6297	HSB0243	Male	Chinese	2020	58	Others	No formal education	Unemployed	Urban	No	No	No	No	Yes	No	No	No	No	No
6298	HSB0244	Male	Malay	2020	33	Married	Primary	Private	Urban	No	Yes	No	No	No	No	No	No	No	Yes
6299	HSB0245	Male	Malay	2020	64	Married	No formal education	Private	Rural	No	No	No	No	No	No	No	No	No	Yes
6300	HSB0246	Male	Indian	2018	40	Married	Secondary	Self-employed	Urban	No	Yes	Yes	No	No	No	No	No	No	No
6301	HSB0247	Male	Indian	2018	33	Married	No formal education	Private	Urban	No	No	Yes	No	No	No	No	No	No	No
6302	HSB0248	Male	Malay	2020	32	Married	No formal education	Government	Rural	No	No	No	No	Yes	No	No	No	No	No
6303	HSB0249	Male	Malay	2018	27	Single	Tertiary	Unemployed	Rural	Yes	Yes	No	No	No	No	No	No	No	No
6304	HSB0250	Male	Others	2020	55	Married	Tertiary	Self-employed	Rural	No	Yes	Yes	No	No	No	No	No	No	No
6305	HSB0251	Male	Others	2020	47	Married	No formal education	Self-employed	Rural	Yes	Yes	No	No	Yes	No	No	No	No	No
6306	HSB0252	Male	Malay	2020	29	Others	Secondary	Unemployed	Rural	No	No	No	No	Yes	No	No	No	No	No
6307	HSB0253	Male	Chinese	2020	46	Single	Primary	Private	Rural	No	Yes	No	No	No	No	No	No	No	Yes
6308	HSB0254	Female	Malay	2019	38	Married	Tertiary	Private	Urban	No	No	No	No	No	No	Yes	No	No	No
6309	HSB0255	Female	Malay	2019	25	Married	Secondary	Others	Rural	No	No	No	No	Yes	No	No	No	No	No

No	Patient ID	Gender	Ethnic group	Year of diagnosis	Age	Marital status	Education level	Occupation	Place of residence	History of psy illness	Tobacco	Alcohol	Cannabis	ATS	Inhalant	Sedative	Hallucinogens	Opioids	Kratom
6310	HSB0256	Male	Malay	2020	24	Single	Secondary	Unemployed	Rural	No	Yes	No	No	Yes	No	No	No	No	No
6311	HSB0257	Male	Malay	2020	26	Single	Secondary	Private	Rural	No	Yes	No	No	Yes	No	No	No	No	Yes
6312	HSB0258	Female	Malay	2020	25	Single	Secondary	Unemployed	Rural	No	No	No	No	Yes	No	No	No	No	No
6313	HSB0259	Male	Malay	2019	35	Others	Secondary	Private	Rural	No	No	No	No	Yes	No	No	No	Yes	Yes
6314	HSB0260	Female	Malay	2019	49	Married	Secondary	Unemployed	Rural	Yes	Yes	No	Yes	No	No	No	No	No	No
6315	HSB0262	Male	Malay	2019	35	Married	Tertiary	Private	Rural	No	No	No	Yes	No	No	No	No	No	No
6316	HSB0263	Male	Malay	2020	21	Single	Secondary	Self-employed	Rural	No	Yes	No	No	Yes	No	No	No	No	No
6317	HSB0264	Male	Malay	2018	24	Single	No formal education	Unemployed	Rural	No	No	No	No	Yes	No	No	No	Yes	Yes
6318	HSB0265	Male	Chinese	2020	46	Married	Secondary	Unemployed	Rural	No	Yes	No	No	No	No	No	No	No	No
6319	HSB0266	Male	Malay	2018	23	Single	Tertiary	Private	Urban	No	Yes	No	No	Yes	No	No	No	Yes	No
6320	HSB0268	Male	Malay	2019	31	Single	No formal education	Unemployed	Rural	No	Yes	No	No	Yes	No	Yes	No	No	Yes
6321	HSB0269	Male	Indian	2018	38	Others	Secondary	Self-employed	Rural	No	Yes	Yes	No	Yes	No	No	No	Yes	No
6322	HSB0270	Male	Malay	2020	34	Single	Tertiary	Private	Urban	No	No	No	No	Yes	Yes	No	No	No	No
6323	HSB0271	Male	Malay	2020	45	Single	Secondary	Unemployed	Rural	No	No	Yes	Yes	Yes	No	No	No	Yes	No
6324	HSB0272	Male	Malay	2020	55	Married	Secondary	Self-employed	Rural	No	Yes	No	No	No	No	No	No	No	No
6325	HSB0273	Male	Malay	2020	28	Single	Secondary	Unemployed	Urban	No	No	No	Yes	Yes	No	No	No	No	Yes
6326	HSB0274	Female	Chinese	2020	18	Single	Secondary	Private	Urban	No	Yes	Yes	No	No	No	No	No	No	No
6327	HSB0275	Male	Malay	2020	22	Single	Secondary	Private	Urban	No	No	No	No	Yes	No	No	No	Yes	No
6328	HSB0276	Male	Malay	2020	26	Single	No formal education	Government	Rural	Yes	Yes	No	No	No	No	No	No	No	No
6329	HSB0277	Male	Malay	2020	30	Others	No formal education	Government	Urban	No	Yes	No	No	No	No	No	No	No	No
6330	HSB0278	Male	Malay	2020	31	Others	No formal education	Others	Rural	No	Yes	No	No	No	No	No	No	No	No
6331	HSB0279	Male	Malay	2020	22	Others	Secondary	Self-employed	Rural	No	No	No	No	Yes	No	No	No	Yes	No
6332	HSB0280	Male	Malay	2019	24	Single	Secondary	Self-employed	Rural	No	No	No	No	Yes	No	No	No	Yes	No
6333	HSB0281	Male	Malay	2019	43	Others	No formal education	Others	Rural	No	No	No	No	No	No	No	No	Yes	No
6334	HSB0282	Female	Malay	2020	24	Single	Tertiary	Unemployed	Urban	No	No	No	Yes	Yes	No	No	No	No	No
6335	HSB0283	Male	Chinese	2020	62	Married	Primary	Unemployed	Rural	Yes	Yes	No	No	No	No	No	No	No	No
6336	HSB0284	Male	Malay	2019	41	Married	Secondary	Government	Rural	No	Yes	No	No	No	No	No	No	No	Yes
6337	HSB0285	Male	Malay	2020	47	Single	Secondary	Unemployed	Rural	Yes	No	Yes	Yes	Yes	No	No	No	No	No
6338	HSB0286	Male	Malay	2020	31	Single	Secondary	Self-employed	Rural	Yes	No	No	Yes	Yes	No	No	No	No	Yes
6339	HSB0287	Male	Malay	2020	40	Married	Primary	Unemployed	Rural	No	No	No	No	Yes	No	No	No	No	No
6340	HSB0288	Male	Malay	2019	29	Married	Tertiary	Private	Urban	No	No	No	No	Yes	No	No	No	No	No
6341	HSB0289	Male	Chinese	2019	92	Married	Primary	Unemployed	Urban	No	Yes	Yes	No	No	No	No	No	No	No
6342	HSB0290	Male	Malay	2019	32	Married	Secondary	Self-employed	Rural	Yes	No	No	No	Yes	No	No	No	No	Yes
6343	HSB0292	Male	Malay	2019	19	Single	Tertiary	Self-employed	Urban	No	Yes	No	No	No	No	No	No	No	No
6344	HSB0293	Male	Malay	2019	41	Others	Secondary	Self-employed	Rural	Yes	No	Yes	Yes	Yes	No	No	No	No	Yes

No	Patient ID	Gender	Ethnic group	Year of diagnosis	Age	Marital status	Education level	Occupation	Place of residence	History of psy illness	Tobacco	Alcohol	Cannabis	ATS	Inhalant	Sedative	Hallucinogens	Opioids	Kratom
6345	HSB0294	Female	Malay	2020	23	Married	Secondary	Unemployed	Urban	No	No	No	No	Yes	No	No	No	No	No
6346	HSB0295	Male	Malay	2019	32	Others	Secondary	Unemployed	Rural	No	Yes	No	No	No	No	No	No	No	No
6347	HSB0296	Male	Malay	2019	21	Others	Tertiary	Private	Rural	No	Yes	No	No	No	No	No	No	No	No
6348	HSB0297	Male	Malay	2019	42	Married	Secondary	Unemployed	Rural	No	Yes	No	No	No	No	No	No	No	No
6349	HSB0298	Male	Malay	2020	41	Married	Tertiary	Government	Urban	No	Yes	No	No	No	No	No	No	No	No
6350	HSB0299	Male	Malay	2020	23	Single	Tertiary	Unemployed	Rural	No	Yes	No	No	No	No	No	No	No	No
6351	HSB0300	Male	Malay	2020	20	Single	Secondary	Private	Rural	No	Yes	No	No	Yes	No	No	No	No	No
6352	HSB0301	Male	Malay	2020	29	Others	Tertiary	Unemployed	Urban	Yes	No	No	No	Yes	No	No	No	No	No
6353	HSB0302	Male	Malay	2020	59	Single	Secondary	Self-employed	Rural	Yes	Yes	No	No	No	No	No	No	No	No
6354	HSB0303	Male	Malay	2020	27	Single	Tertiary	Private	Rural	No	No	No	No	No	No	No	No	Yes	No
6355	HSB0305	Male	Chinese	2020	41	Single	Secondary	Private	Urban	No	Yes	No	No	Yes	No	No	No	Yes	No
6356	HSB0306	Male	Malay	2020	35	Married	Secondary	Unemployed	Rural	No	Yes	No	No	Yes	No	No	No	No	No
6357	HSB0307	Male	Malay	2020	39	Single	No formal education	Self-employed	Rural	No	No	No	No	Yes	No	No	No	Yes	Yes
6358	HSB0308	Male	Malay	2020	33	Married	Tertiary	Unemployed	Rural	Yes	Yes	No	No	No	No	No	No	No	No
6359	HSB0309	Male	Malay	2019	42	Others	No formal education	Government	Urban	No	No	No	No	Yes	No	No	No	No	No
6360	HSB0310	Male	Indian	2019	29	Single	Tertiary	Unemployed	Urban	No	No	Yes	Yes	Yes	No	Yes	No	Yes	No
6361	HSB0312	Male	Malay	2019	48	Others	Secondary	Private	Rural	No	No	No	No	Yes	No	No	No	Yes	No
6362	HSB0314	Male	Malay	2019	38	Others	No formal education	Self-employed	Urban	No	No	No	No	No	No	No	No	Yes	No
6363	HSB0315	Male	Chinese	2019	42	Others	Secondary	Unemployed	Urban	No	No	No	No	Yes	No	No	No	No	No
6364	HSB0316	Male	Malay	2020	38	Others	No formal education	Others	Rural	Yes	No	Yes	No	Yes	No	No	No	Yes	No
6365	HSB0317	Male	Malay	2020	32	Married	Tertiary	Government	Rural	No	Yes	No	No	No	No	No	No	No	No
6366	HSB0318	Male	Chinese	2020	75	Married	Secondary	Others	Urban	Yes	No	Yes	No	No	No	No	No	No	No
6367	HSB0319	Male	Malay	2020	37	Married	Tertiary	Unemployed	Urban	Yes	No	No	No	No	No	Yes	No	Yes	No
6368	HSB0320	Male	Malay	2020	37	Single	Secondary	Self-employed	Rural	No	Yes	No	No	No	No	No	No	No	No
6369	HSB0323	Male	Malay	2020	22	Single	Tertiary	Unemployed	Urban	No	Yes	No	Yes	No	No	No	No	Yes	Yes
6370	HSB0324	Male	Malay	2020	28	Single	Secondary	Self-employed	Rural	No	Yes	No	No	No	No	No	No	No	Yes
6371	HSB0325	Male	Malay	2020	53	Married	Secondary	Unemployed	Urban	No	Yes	No	No	No	No	No	No	No	No
6372	HSB0326	Male	Malay	2020	33	Married	Secondary	Self-employed	Rural	No	No	No	No	Yes	No	No	No	Yes	Yes
6373	HSB0327	Male	Chinese	2020	46	Others	Secondary	Private	Rural	Yes	Yes	Yes	No	No	No	No	No	No	No
6374	HSB0329	Male	Malay	2019	20	Single	Tertiary	Unemployed	Urban	No	Yes	No	Yes	No	No	No	No	No	No
6375	HSB0330	Male	Malay	2019	33	Married	Primary	Self-employed	Rural	No	No	No	No	No	No	Yes	No	No	No
6376	HSB0331	Male	Malay	2019	49	Married	Tertiary	Self-employed	Rural	Yes	Yes	No	No	No	No	No	No	No	No
6377	HSB0334	Male	Malay	2019	38	Married	Secondary	Self-employed	Urban	No	Yes	No	No	No	No	No	No	No	Yes
6378	HSB0336	Male	Chinese	2020	54	Single	Secondary	Private	Rural	No	Yes	No	No	No	No	No	No	No	No
6379	HSB0337	Female	Others	2020	37	Married	Primary	Others	Rural	No	No	Yes	No	No	No	No	No	No	No
6380	HSB0339	Male	Malay	2019	40	Married	No formal education	Unemployed	Rural	Yes	No	No	No	No	No	Yes	No	Yes	Yes
6381	HSB0340	Male	Malay	2019	33	Married	Secondary	Private	Rural	No	Yes	No	No	No	No	No	No	No	No
6382	HSB0341	Male	Malay	2019	28	Single	Secondary	Unemployed	Rural	No	Yes	No	No	Yes	No	No	No	Yes	No
6383	HSB0342	Male	Malay	2019	44	Others	Secondary	Private	Urban	No	Yes	No	No	No	No	No	No	No	Yes

No	Patient ID	Gender	Ethnic group	Year of diagnosis	Age	Marital status	Education level	Occupation	Place of residence	History of psy illness	Tobacco	Alcohol	Cannabis	ATS	Inhalant	Sedative	Hallucinogens	Opioids	Kratom
6384	HSB0343	Male	Malay	2019	30	Single	Tertiary	Others	Rural	No	No	No	No	Yes	No	No	No	No	No
6385	HSB0344	Male	Malay	2019	27	Single	Secondary	Private	Rural	No	Yes	No	No	Yes	No	Yes	No	No	No
6386	HSB0345	Male	Malay	2019	29	Single	Secondary	Unemployed	Rural	No	No	No	No	Yes	No	No	No	No	Yes
6387	HSB0346	Male	Malay	2019	38	Single	Secondary	Unemployed	Rural	No	No	No	No	Yes	No	No	No	No	No
6388	HSB0348	Male	Malay	2019	33	Single	Secondary	Self-employed	Rural	No	Yes	No	No	No	No	No	No	No	No
6389	HSB0349	Male	Malay	2019	48	Single	Secondary	Private	Rural	No	Yes	No	No	No	No	No	No	No	No
6390	HSB0351	Male	Chinese	2019	63	Married	Primary	Self-employed	Rural	No	Yes	Yes	No	No	No	No	No	No	No
6391	HSB0352	Female	Malay	2020	29	Single	Secondary	Unemployed	Rural	No	No	No	No	Yes	No	No	No	No	No
6392	HSB0353	Male	Malay	2020	75	Others	Tertiary	Others	Rural	No	Yes	No	No	No	No	No	No	No	No
6393	HSB0354	Male	Malay	2020	60	Married	Secondary	Unemployed	Rural	No	No	No	Yes	No	No	Yes	No	No	No
6394	HSB0355	Female	Malay	2020	27	Others	Secondary	Unemployed	Urban	No	Yes	No	No	Yes	No	No	No	No	No
6395	HSB0356	Male	Chinese	2020	48	Married	Secondary	Private	Urban	No	Yes	Yes	Yes	No	No	No	No	No	No
6396	HSB0357	Male	Chinese	2020	44	Married	Tertiary	Private	Rural	Yes	Yes	No	No	No	No	No	No	No	No
6397	HSB0359	Male	Malay	2020	22	Single	Secondary	Unemployed	Rural	Yes	No	No	No	No	No	No	No	No	Yes
6398	HSB0360	Male	Malay	2019	19	Single	Tertiary	Unemployed	Rural	No	Yes	No	No	No	No	No	No	No	No
6399	HSB0361	Male	Malay	2019	33	Married	No formal education	Private	Urban	No	No	No	No	Yes	No	No	No	Yes	Yes
6400	HSB0362	Male	Malay	2019	38	Married	Tertiary	Self-employed	Urban	No	Yes	No	Yes	No	No	No	No	No	No
6401	HSB0364	Male	Chinese	2019	46	Others	Secondary	Unemployed	Rural	No	Yes	No	No	Yes	No	No	No	No	Yes
6402	HSB0365	Male	Malay	2019	30	Others	Secondary	Others	Rural	No	Yes	No	No	Yes	No	No	No	No	Yes
6403	HSB0366	Male	Malay	2019	23	Single	Secondary	Others	Rural	No	No	No	No	No	No	No	No	No	Yes
6404	HSB0367	Female	Malay	2019	31	Married	Secondary	Private	Urban	No	No	No	No	Yes	No	No	No	No	No
6405	HSB0368	Male	Chinese	2019	31	Others	Secondary	Private	Urban	Yes	Yes	No	No	Yes	No	No	No	No	No
6406	HSB0369	Male	Chinese	2019	36	Single	Secondary	Private	Rural	Yes	Yes	No	No	No	No	Yes	No	No	Yes
6407	HSB0370	Male	Malay	2020	26	Single	Secondary	Self-employed	Rural	No	Yes	No	No	No	No	No	No	No	No
6408	HSB0371	Female	Malay	2020	53	Married	Secondary	Unemployed	Urban	No	No	No	No	No	No	No	No	No	Yes
6409	HSB0373	Female	Chinese	2020	26	Others	Tertiary	Private	Urban	Yes	Yes	No	No	No	No	No	No	No	No
6410	HSB0374	Male	Malay	2020	43	Single	Secondary	Self-employed	Rural	No	No	No	No	Yes	No	No	No	No	Yes
6411	HSB0375	Male	Chinese	2020	40	Single	Secondary	Private	Rural	Yes	Yes	Yes	Yes	Yes	No	Yes	No	No	Yes
6412	HSB0376	Male	Malay	2019	30	Married	Tertiary	Private	Urban	No	Yes	No	No	No	No	No	No	No	No
6413	HSB0378	Male	Chinese	2019	37	Others	Primary	Unemployed	Rural	No	Yes	No	Yes	No	No	No	No	No	No
6414	HSB0379	Female	Others	2020	33	Others	Primary	Self-employed	Rural	No	No	No	No	Yes	No	No	No	No	No
6415	HSB0380	Male	Chinese	2019	60	Single	No formal education	Unemployed	Rural	No	Yes	No	No	No	No	No	No	No	No
6416	HSB0381	Male	Malay	2019	52	Married	No formal education	Government	Urban	No	Yes	No	No	No	No	No	No	No	No
6417	HSB0382	Male	Malay	2019	37	Married	Secondary	Unemployed	Rural	Yes	Yes	No	No	No	No	No	No	No	No
6418	HSB0383	Male	Others	2020	32	Married	Secondary	Self-employed	Rural	No	Yes	No	No	No	No	No	No	No	No
6419	HSB0384	Male	Malay	2020	26	Others	Tertiary	Self-employed	Rural	No	Yes	No	Yes	No	No	No	No	No	No
6420	HSB0385	Male	Malay	2019	27	Married	Tertiary	Unemployed	Urban	No	Yes	No	No	No	No	No	No	No	No
6421	HSB0386	Male	Malay	2019	27	Single	No formal education	Unemployed	Rural	No	Yes	Yes	Yes	Yes	No	No	No	No	Yes
6422	HSB0387	Male	Malay	2020	25	Single	Secondary	Private	Rural	No	Yes	No	No	No	No	No	No	No	Yes

No	Patient ID	Gender	Ethnic group	Year of diagnosis	Age	Marital status	Education level	Occupation	Place of residence	History of psy illness	Tobacco	Alcohol	Cannabis	ATS	Inhalant	Sedative	Hallucinogens	Opioids	Kratom
6423	HSB0388	Male	Malay	2019	31	Married	Tertiary	Unemployed	Rural	No	No	No	No	Yes	No	No	No	Yes	No
6424	HSB0389	Male	Indian	2019	31	Others	Secondary	Self-employed	Urban	No	Yes	No	No	No	No	No	No	No	No
6425	HSB0390	Male	Malay	2019	27	Others	Tertiary	Private	Rural	No	Yes	No	No	No	No	No	No	No	No
6426	HSB0392	Male	Malay	2020	31	Single	No formal education	Private	Urban	Yes	No	No	No	No	No	No	No	No	Yes
6427	HSB0393	Male	Malay	2019	30	Married	No formal education	Self-employed	Rural	No	No	No	No	No	No	No	No	Yes	No
6428	HSB0394	Male	Malay	2019	36	Others	Secondary	Self-employed	Rural	No	Yes	No	Yes	Yes	No	No	No	No	No
6429	HSB0395	Male	Malay	2019	25	Others	No formal education	Unemployed	Rural	No	No	No	No	Yes	No	No	No	No	Yes
6430	HSB0397	Male	Malay	2020	18	Single	Secondary	Self-employed	Rural	No	Yes	No	No	No	No	No	No	No	No
6431	HSB0398	Female	Malay	2020	20	Single	Tertiary	Unemployed	Rural	No	No	Yes	No	No	No	No	No	No	No
6432	HSB0399	Female	Malay	2020	34	Married	Secondary	Unemployed	Urban	No	No	No	No	Yes	No	No	No	No	No
6433	HSB0400	Male	Malay	2020	35	Single	Tertiary	Self-employed	Urban	Yes	No	No	No	Yes	No	No	No	Yes	No
6434	HSB0401	Female	Malay	2020	32	Others	Primary	Private	Rural	No	Yes	No	No	No	No	No	No	No	No
6435	HSB0403	Male	Malay	2020	59	Married	Tertiary	Private	Urban	No	Yes	Yes	No	No	No	No	No	No	No
6436	HSB0404	Male	Malay	2020	42	Married	Secondary	Unemployed	Rural	No	No	No	No	Yes	No	No	No	No	No
6437	HSB0405	Male	Malay	2020	50	Married	Secondary	Unemployed	Urban	No	No	No	No	Yes	No	No	No	Yes	No
6438	HSB0406	Male	Malay	2020	29	Single	Secondary	Self-employed	Rural	No	Yes	No	No	Yes	No	No	No	No	Yes
6439	HSB0407	Male	Malay	2020	18	Single	Secondary	Others	Urban	Yes	Yes	No	No	No	No	No	No	No	No
6440	HSB0408	Female	Malay	2019	35	Others	Primary	Unemployed	Rural	No	No	No	No	Yes	No	No	No	No	No
6441	HSB0409	Male	Malay	2019	29	Married	Tertiary	Government	Rural	Yes	Yes	No	No	No	No	No	No	No	No
6442	HSB0411	Male	Malay	2019	34	Single	Tertiary	Unemployed	Rural	No	Yes	No	No	No	No	No	No	No	No
6443	HSB0412	Male	Indian	2019	39	Others	No formal education	Self-employed	Rural	No	Yes	Yes	No	Yes	No	No	No	No	No
6444	HSB0413	Male	Malay	2019	33	Married	No formal education	Self-employed	Rural	No	No	No	No	No	No	No	No	Yes	No
6445	HSB0414	Male	Malay	2019	40	Others	No formal education	Unemployed	Rural	No	No	No	No	No	No	No	No	Yes	No
6446	HSB0415	Male	Malay	2019	46	Married	Secondary	Private	Rural	No	Yes	No	No	No	No	No	No	No	No
6447	HSB0416	Male	Malay	2019	48	Married	Primary	Private	Rural	No	Yes	No	No	No	No	No	No	No	No
6448	HSB0418	Male	Malay	2019	31	Married	No formal education	Others	Rural	No	Yes	No	No	No	No	No	No	No	No
6449	HSB0419	Male	Malay	2019	22	Single	Secondary	Unemployed	Rural	No	Yes	No	No	Yes	No	No	No	No	Yes
6450	HSB0421	Male	Malay	2019	62	Others	Secondary	Self-employed	Rural	No	Yes	No	No	No	No	No	No	No	No
6451	HSB0422	Male	Malay	2020	21	Single	Secondary	Unemployed	Urban	Yes	Yes	No	No	Yes	No	No	No	No	No
6452	HSB0423	Male	Malay	2020	20	Single	Tertiary	Private	Urban	No	Yes	No	No	No	No	No	No	No	No
6453	HSB0424	Male	Malay	2020	22	Single	Tertiary	Government	Rural	No	Yes	No	No	No	No	No	No	No	No
6454	HSB0425	Male	Malay	2020	35	Single	Tertiary	Unemployed	Rural	No	Yes	No	No	No	No	No	No	No	Yes
6455	HSB0426	Male	Malay	2020	30	Single	Tertiary	Self-employed	Rural	No	Yes	No	No	No	No	No	No	No	Yes
6456	HSB0427	Female	Malay	2019	22	Single	Tertiary	Unemployed	Rural	Yes	No	Yes	No	No	No	No	No	No	No
6457	HSB0428	Male	Chinese	2019	39	Single	Tertiary	Government	Urban	No	Yes	Yes	No	No	No	No	No	No	No

No	Patient ID	Gender	Ethnic group	Year of diagnosis	Age	Marital status	Education level	Occupation	Place of residence	History of psy illness	Tobacco	Alcohol	Cannabis	ATS	Inhalant	Sedative	Hallucinogens	Opioids	Kratom
6458	HSB0429	Male	Chinese	2019	35	Others	No formal education	Others	Rural	No	No	Yes	No	Yes	No	No	No	No	No
6459	HSB0430	Male	Malay	2019	19	Single	Tertiary	Private	Rural	No	Yes	No	No	No	No	No	No	No	No
6460	HSB0433	Male	Malay	2019	53	Married	Primary	Self-employed	Urban	No	Yes	No	Yes	No	No	No	No	No	No
6461	HSB0434	Male	Chinese	2020	35	Others	Tertiary	Private	Urban	No	Yes	Yes	No	No	No	No	No	No	No
6462	HSB0435	Male	Malay	2020	41	Others	No formal education	Unemployed	Rural	No	No	No	No	Yes	No	No	No	No	No
6463	HSB0436	Male	Malay	2020	18	Single	Secondary	Others	Rural	No	Yes	No	No	Yes	No	No	No	Yes	Yes
6464	HSB0437	Female	Malay	2020	30	Married	Secondary	Others	Urban	No	Yes	No	No	Yes	No	No	No	Yes	No
6465	HSB0438	Male	Indian	2020	21	Others	Tertiary	Unemployed	Urban	No	No	Yes	No	No	No	No	No	No	No
6466	HSB0439	Male	Malay	2019	35	Married	Tertiary	Government	Rural	Yes	Yes	No	Yes	No	No	No	Yes	No	No
6467	HSB0440	Male	Malay	2020	33	Single	Tertiary	Unemployed	Rural	No	Yes	No	No	No	No	No	No	No	Yes
6468	HSB0442	Male	Others	2020	25	Single	Secondary	Others	Rural	No	Yes	No	No	Yes	No	No	No	Yes	Yes
6469	HSB0443	Male	Malay	2020	54	Married	Secondary	Self-employed	Rural	No	Yes	No	No	No	No	No	No	Yes	No
6470	HSB0444	Male	Malay	2020	30	Others	Tertiary	Unemployed	Urban	No	No	Yes	No	Yes	No	No	No	No	No
6471	HSB0446	Male	Malay	2020	22	Single	Secondary	Private	Rural	No	Yes	No	No	No	No	No	No	No	Yes
6472	HSB0447	Female	Malay	2020	28	Others	Secondary	Private	Rural	No	No	No	No	Yes	No	No	No	No	No
6473	HSB0448	Male	Malay	2020	24	Single	Tertiary	Self-employed	Rural	Yes	Yes	No	No	No	No	No	No	No	No
6474	HSB0450	Male	Malay	2020	35	Others	No formal education	Others	Rural	No	No	No	No	Yes	No	No	No	No	No
6475	HSB0451	Male	Malay	2020	73	Married	No formal education	Others	Rural	No	Yes	No	No	No	No	No	No	No	No
6476	HSB0453	Male	Malay	2020	26	Single	No formal education	Self-employed	Rural	No	No	No	Yes	Yes	No	Yes	No	No	No
6477	HSB0454	Male	Malay	2020	23	Single	Tertiary	Unemployed	Rural	No	Yes	No	No	No	No	No	No	No	No
6478	HSB0456	Male	Others	2020	31	Others	Tertiary	Private	Urban	No	Yes	No	No	No	No	No	No	No	No
6479	HSB0458	Male	Chinese	2020	36	Married	No formal education	Private	Urban	No	Yes	Yes	No	No	No	No	No	No	No
6480	HSB0459	Male	Malay	2020	19	Single	Tertiary	Self-employed	Rural	No	Yes	No	No	No	No	No	No	No	No
6481	HSB0460	Female	Chinese	2019	57	Married	No formal education	Others	Rural	Yes	No	Yes	No	No	No	No	No	No	No
6482	HSB0461	Male	Malay	2020	51	Married	Tertiary	Self-employed	Rural	No	Yes	No	No	No	No	No	No	No	No
6483	HSB0462	Male	Malay	2020	32	Married	Tertiary	Private	Rural	No	Yes	No	No	No	No	No	No	No	No
6484	HSB0463	Male	Malay	2020	43	Married	Secondary	Others	Urban	Yes	Yes	No	No	No	No	No	No	No	Yes
6485	HSB0464	Male	Malay	2019	29	Single	Secondary	Unemployed	Urban	Yes	No	No	No	Yes	No	No	No	No	No
6486	HSB0465	Male	Malay	2020	33	Single	Tertiary	Self-employed	Rural	No	Yes	No	No	Yes	No	No	No	Yes	Yes
6487	HSB0466	Male	Malay	2020	41	Married	Secondary	Self-employed	Urban	Yes	Yes	No	No	No	No	No	No	Yes	No
6488	HSB0467	Male	Malay	2020	23	Others	Tertiary	Private	Urban	No	No	No	No	No	No	No	No	No	Yes
6489	HSB0468	Female	Chinese	2019	70	Married	No formal education	Others	Rural	Yes	No	No	No	No	No	Yes	No	No	No
6490	HSB0469	Male	Malay	2019	56	Married	Secondary	Private	Rural	No	Yes	No	No	No	No	No	No	No	No
6491	HSB0470	Female	Malay	2021	32	Others	Secondary	Self-employed	Urban	No	Yes	No	No	Yes	No	No	No	No	No
6492	HSB0471	Male	Chinese	2019	24	Single	Tertiary	Self-employed	Urban	Yes	No	No	No	Yes	No	No	No	No	No

No	Patient ID	Gender	Ethnic group	Year of diagnosis	Age	Marital status	Education level	Occupation	Place of residence	History of psy illness	Tobacco	Alcohol	Cannabis	ATS	Inhalant	Sedative	Hallucinogens	Opioids	Kratom
6493	HSB0473	Male	Malay	2019	36	Married	Tertiary	Self-employed	Rural	No	Yes	No	No	Yes	No	No	No	No	No
6494	HSB0475	Male	Malay	2019	31	Single	Secondary	Unemployed	Rural	No	Yes	No	No	No	No	No	No	Yes	No
6495	HSB0476	Male	Malay	2020	30	Married	Tertiary	Self-employed	Rural	No	Yes	No	No	No	No	Yes	No	No	Yes
6496	HSB0477	Male	Chinese	2020	45	Others	Secondary	Self-employed	Rural	No	No	Yes	No	No	No	Yes	No	No	No
6497	HSB0478	Male	Indian	2020	31	Single	Tertiary	Private	Urban	Yes	No	Yes	Yes	No	No	No	No	Yes	No
6498	HSB0479	Male	Malay	2019	50	Married	Tertiary	Self-employed	Urban	No	Yes	No	No	No	No	No	No	No	No
6499	HSB0480	Female	Malay	2019	41	Others	Secondary	Unemployed	Urban	No	No	No	No	Yes	No	Yes	No	Yes	No
6500	HSB0482	Male	Malay	2019	41	Others	Secondary	Others	Rural	No	Yes	No	No	No	No	No	No	No	No
6501	HSB0483	Male	Malay	2019	36	Others	Secondary	Private	Urban	Yes	Yes	No	No	No	No	No	No	No	Yes
6502	HSB0484	Male	Malay	2019	27	Single	Tertiary	Unemployed	Rural	No	Yes	No	No	No	No	No	No	No	No
6503	HSB0485	Male	Malay	2018	41	Others	Secondary	Private	Rural	No	No	No	No	Yes	No	No	No	Yes	No
6504	HSB0486	Female	Malay	2020	34	Married	Secondary	Self-employed	Rural	Yes	No	No	No	Yes	No	No	No	No	No
6505	HSB0487	Male	Indian	2020	35	Married	Secondary	Unemployed	Rural	Yes	No	Yes	No	No	No	No	No	No	No
6506	HSB0488	Male	Indian	2020	56	Married	Primary	Self-employed	Urban	Yes	Yes	Yes	No	No	No	No	No	No	No
6507	HSB0489	Male	Malay	2020	41	Others	Tertiary	Government	Rural	Yes	No	No	Yes	Yes	No	No	No	No	No
6508	HSB0490	Male	Malay	2020	28	Single	Secondary	Self-employed	Rural	Yes	No	No	No	Yes	No	No	No	No	No
6509	HSB0492	Male	Malay	2020	28	Single	Secondary	Unemployed	Rural	Yes	No	No	No	Yes	No	No	No	No	No
6510	HSB0493	Male	Malay	2020	36	Single	No formal education	Unemployed	Rural	No	No	No	Yes	Yes	No	No	No	No	No
6511	HSB0494	Male	Malay	2020	33	Single	No formal education	Self-employed	Rural	No	Yes	No	No	Yes	No	No	No	Yes	No
6512	HSB0495	Male	Malay	2020	46	Others	No formal education	Others	Urban	No	No	No	No	Yes	No	No	No	No	Yes
6513	HSB0498	Male	Malay	2020	39	Others	No formal education	Unemployed	Rural	Yes	No	No	Yes	Yes	No	No	No	No	Yes
6514	HSB0500	Male	Chinese	2020	36	Married	Tertiary	Private	Rural	No	No	Yes	No	No	No	No	No	No	No
6515	HSB0501	Male	Malay	2021	32	Single	Tertiary	Government	Rural	Yes	Yes	No	No	Yes	No	No	No	No	No
6516	HSB0502	Male	Malay	2021	39	Others	No formal education	Others	Rural	No	Yes	No	No	Yes	No	No	No	No	No
6517	HSB0503	Male	Malay	2021	24	Single	No formal education	Others	Rural	No	No	No	No	Yes	No	No	No	No	No
6518	HSB0504	Male	Malay	2021	41	Others	No formal education	Others	Rural	No	No	No	No	Yes	No	No	No	No	No
6519	HSB0505	Male	Malay	2021	31	Others	No formal education	Others	Rural	No	No	No	No	Yes	No	No	No	No	No
6520	HSB0506	Male	Indian	2021	40	Married	Secondary	Unemployed	Urban	Yes	Yes	No	No	Yes	No	No	No	Yes	No
6521	HSB0507	Female	Malay	2021	32	Married	No formal education	Others	Rural	No	Yes	No	No	Yes	No	No	No	No	No
6522	HSB0508	Male	Others	2021	30	Single	Secondary	Private	Rural	No	Yes	Yes	No	No	No	No	No	No	Yes
6523	HSB0509	Male	Malay	2021	54	Others	Secondary	Self-employed	Urban	No	Yes	No	Yes	Yes	No	Yes	No	Yes	No
6524	HSB0510	Male	Malay	2021	25	Single	No formal education	Others	Urban	No	Yes	No	No	No	Yes	No	No	No	No

No	Patient ID	Gender	Ethnic group	Year of diagnosis	Age	Marital status	Education level	Occupation	Place of residence	History of psy illness	Tobacco	Alcohol	Cannabis	ATS	Inhalant	Sedative	Hallucinogens	Opioids	Kratom
6525	HSB0511	Female	Chinese	2021	32	Others	Secondary	Private	Urban	No	Yes	No	No	Yes	No	No	No	No	No
6526	HSB0513	Male	Indian	2021	50	Single	No formal education	Unemployed	Rural	No	Yes	Yes	No	No	No	No	No	No	No
6527	HSB0514	Male	Malay	2021	29	Others	No formal education	Unemployed	Rural	No	Yes	No	Yes	Yes	No	No	No	No	Yes
6528	HSB0516	Female	Malay	2021	30	Married	Secondary	Unemployed	Urban	No	No	No	No	Yes	No	No	No	No	No
6529	HSB0517	Male	Malay	2020	24	Single	Secondary	Self-employed	Rural	No	No	No	No	Yes	No	No	No	Yes	No
6530	HSB0520	Female	Indian	2020	24	Others	Tertiary	Others	Urban	No	No	No	No	No	No	Yes	No	No	No
6531	HSB0522	Male	Malay	2020	19	Single	Secondary	Unemployed	Rural	No	No	No	No	Yes	No	No	No	No	No
6532	HSB0523	Male	Malay	2020	34	Others	Secondary	Self-employed	Rural	No	No	No	No	Yes	No	No	No	Yes	No
6533	HSB0525	Male	Indian	2020	27	Married	Secondary	Private	Rural	No	No	No	No	Yes	No	No	No	No	No
6534	HSB0527	Male	Malay	2020	32	Married	No formal education	Self-employed	Rural	No	No	No	Yes	Yes	No	No	No	No	Yes
6535	HSB0529	Male	Malay	2020	27	Single	Tertiary	Unemployed	Rural	No	Yes	No	Yes	Yes	No	No	No	No	Yes
6536	HSB0530	Male	Malay	2020	24	Single	Secondary	Unemployed	Rural	No	No	No	No	Yes	No	No	No	No	No
6537	HSB0531	Male	Malay	2020	25	Single	Tertiary	Government	Urban	No	Yes	No	No	No	No	Yes	No	Yes	No
6538	HSB0532	Male	Malay	2020	46	Single	Tertiary	Unemployed	Rural	Yes	Yes	No	No	No	No	No	No	No	No
6539	HSB0533	Male	Malay	2020	27	Married	No formal education	Self-employed	Rural	No	Yes	No	No	Yes	No	No	No	No	No
6540	HSB0535	Male	Malay	2020	27	Single	Tertiary	Unemployed	Urban	Yes	Yes	No	No	No	No	No	No	No	No
6541	HSB0536	Male	Malay	2018	26	Single	Secondary	Unemployed	Urban	No	No	No	No	Yes	No	No	No	No	No
6542	HSB0537	Male	Malay	2020	39	Single	Secondary	Unemployed	Rural	Yes	Yes	Yes	No	Yes	Yes	No	No	Yes	Yes
6543	HSB0539	Male	Indian	2018	37	Single	No formal education	Others	Rural	No	No	No	No	Yes	No	No	No	No	No
6544	HSB0541	Male	Malay	2020	40	Others	Secondary	Self-employed	Urban	No	No	No	No	Yes	No	No	No	Yes	No
6545	HSB0544	Male	Malay	2020	33	Single	Secondary	Unemployed	Rural	No	Yes	No	No	Yes	No	No	No	No	Yes
6546	HSB0547	Male	Malay	2020	28	Single	Secondary	Self-employed	Rural	No	Yes	No	No	Yes	No	No	No	Yes	No
6547	HSB0548	Male	Malay	2020	40	Others	No formal education	Unemployed	Rural	No	No	No	No	Yes	No	No	No	No	No
6548	HSB0549	Male	Malay	2020	28	Single	Secondary	Private	Rural	No	Yes	No	No	Yes	No	No	No	No	Yes
6549	HSB0552	Male	Malay	2020	30	Married	Secondary	Private	Rural	No	Yes	No	No	Yes	No	No	No	No	Yes
6550	HSB0555	Male	Malay	2020	28	Others	Secondary	Unemployed	Rural	Yes	Yes	No	No	Yes	No	No	No	No	No
6551	HSB0556	Male	Malay	2020	29	Married	Secondary	Others	Rural	Yes	No	No	No	Yes	No	No	No	No	Yes
6552	HSB0557	Male	Chinese	2020	47	Married	Primary	Private	Rural	No	Yes	Yes	No	No	No	No	No	No	No
6553	HSB0558	Female	Malay	2020	27	Others	No formal education	Self-employed	Rural	No	Yes	No	No	Yes	No	No	No	No	No
6554	HSB0559	Male	Malay	2020	40	Others	Secondary	Self-employed	Rural	No	Yes	No	No	Yes	No	No	No	Yes	No
6555	HSB0561	Male	Malay	2020	68	Married	Secondary	Others	Rural	No	Yes	No	No	No	No	No	No	No	No
6556	HSB0562	Male	Malay	2020	38	Married	No formal education	Others	Rural	Yes	No	No	No	No	No	No	No	Yes	No

No	Patient ID	Gender	Ethnic group	Year of diagnosis	Age	Marital status	Education level	Occupation	Place of residence	History of psy illness	Tobacco	Alcohol	Cannabis	ATS	Inhalant	Sedative	Hallucinogens	Opioids	Kratom
6557	HSB0563	Male	Malay	2020	39	Single	Secondary	Others	Rural	No	Yes	No	Yes	Yes	No	No	No	No	No
6558	HSB0564	Male	Malay	2020	30	Single	Primary	Self-employed	Rural	No	Yes	No	Yes	Yes	No	Yes	No	Yes	Yes
6559	HSB0565	Male	Malay	2020	40	Married	No formal education	Private	Rural	Yes	Yes	No	No	Yes	No	No	No	Yes	No
6560	HSB0566	Male	Malay	2020	57	Married	Tertiary	Unemployed	Rural	No	Yes	No	No	No	No	No	No	No	No
6561	HSB0567	Male	Malay	2020	38	Single	Primary	Unemployed	Rural	No	Yes	No	No	No	No	No	No	No	No
6562	HSB0568	Male	Chinese	2020	67	Married	Primary	Self-employed	Rural	No	No	No	No	No	No	Yes	No	No	No
6563	HSB0569	Male	Malay	2020	30	Married	Tertiary	Government	Rural	No	Yes	No	No	No	No	No	No	No	No
6564	HSB0570	Male	Malay	2020	41	Single	Secondary	Unemployed	Rural	No	No	No	No	No	No	Yes	No	No	No
6565	HSB0571	Male	Malay	2019	37	Married	Secondary	Self-employed	Urban	No	No	Yes	No	No	No	No	No	No	No
6566	HSB0572	Male	Malay	2019	27	Married	Tertiary	Government	Rural	No	No	No	No	Yes	No	No	No	No	No
6567	HSB0573	Male	Malay	2019	46	Married	No formal education	Self-employed	Rural	No	No	No	No	Yes	No	No	No	No	Yes
6568	HSB0574	Male	Chinese	2019	58	Others	Secondary	Unemployed	Rural	Yes	No	No	No	Yes	No	No	No	No	No
6569	HSB0576	Male	Malay	2019	33	Single	Secondary	Self-employed	Rural	No	Yes	No	No	No	No	No	No	No	No
6570	HSB0577	Male	Malay	2019	63	Others	No formal education	Self-employed	Rural	No	No	No	Yes	Yes	No	No	No	Yes	Yes
6571	HSB0578	Male	Malay	2019	39	Others	Tertiary	Government	Rural	No	Yes	No	No	No	No	No	No	No	No
6572	HSB0579	Male	Malay	2019	37	Single	Secondary	Self-employed	Rural	No	No	No	No	Yes	No	No	No	No	No
6573	HSB0580	Male	Malay	2019	37	Others	Secondary	Private	Rural	Yes	No	No	No	No	Yes	No	No	No	No
6574	HSB0581	Male	Malay	2019	30	Single	Secondary	Private	Rural	No	Yes	No	No	No	No	No	No	No	No
6575	HSB0582	Male	Malay	2020	47	Single	Tertiary	Unemployed	Urban	No	Yes	No	No	No	No	No	No	No	No
6576	HSB0583	Male	Chinese	2020	54	Single	Secondary	Unemployed	Urban	Yes	Yes	No	No	No	No	No	No	No	No
6577	HSB0584	Male	Malay	2020	25	Others	Secondary	Others	Rural	No	No	No	Yes	Yes	No	No	No	No	No
6578	HSB0585	Male	Chinese	2020	81	Married	Primary	Unemployed	Urban	No	Yes	No	No	No	No	No	No	No	No
6579	HSB0586	Male	Malay	2020	28	Married	No formal education	Government	Rural	No	Yes	No	No	No	No	No	No	No	No
6580	HSB0586	Male	Malay	2020	29	Others	No formal education	Government	Rural	Yes	Yes	No	No	No	No	No	No	No	No
6581	HSB0588	Male	Malay	2019	36	Married	Tertiary	Government	Rural	No	Yes	No	No	No	No	No	No	No	No
6582	HSB0589	Male	Malay	2019	23	Single	Secondary	Unemployed	Rural	No	No	No	No	No	No	Yes	No	No	No
6583	HSB0590	Male	Malay	2019	20	Single	Secondary	Unemployed	Urban	No	Yes	No	No	No	No	No	No	No	No
6584	HSB0591	Male	Malay	2019	37	Others	Secondary	Private	Rural	No	Yes	No	No	No	No	No	No	Yes	Yes
6585	HSB0595	Male	Indian	2020	49	Married	No formal education	Self-employed	Urban	No	Yes	Yes	No	No	No	No	No	No	No
6586	HSB0597	Male	Malay	2020	35	Others	Secondary	Self-employed	Urban	No	No	No	No	Yes	No	No	No	Yes	No
6587	HSB0598	Male	Malay	2020	35	Married	Secondary	Self-employed	Urban	No	Yes	No	No	No	No	No	No	No	No
6588	HSB0599	Male	Malay	2020	55	Single	Secondary	Self-employed	Urban	No	Yes	No	No	No	No	No	No	No	Yes
6589	HSB0600	Male	Others	2020	32	Married	Tertiary	Self-employed	Rural	No	No	Yes	No	Yes	No	No	No	No	No
6590	HSB0601	Male	Malay	2020	44	Others	Secondary	Self-employed	Rural	No	No	No	No	No	No	No	No	Yes	No
6591	HSB0602	Male	Malay	2020	40	Single	Secondary	Private	Rural	No	No	No	No	No	No	Yes	No	Yes	Yes
6592	HSB0603	Female	Malay	2019	35	Married	Secondary	Unemployed	Rural	No	Yes	No	No	Yes	No	No	No	No	No

No	Patient ID	Gender	Ethnic group	Year of diagnosis	Age	Marital status	Education level	Occupation	Place of residence	History of psy illness	Tobacco	Alcohol	Cannabis	ATS	Inhalant	Sedative	Hallucinogens	Opioids	Kratom
6593	HSB0605	Male	Malay	2019	32	Single	No formal education	Self-employed	Urban	No	No	No	No	Yes	No	No	No	No	No
6594	HSB0606	Female	Malay	2019	23	Others	Secondary	Unemployed	Rural	No	Yes	Yes	No	Yes	No	No	No	No	No
6595	HSB0607	Male	Malay	2019	24	Others	Primary	Private	Rural	No	Yes	No	No	No	No	No	No	No	No
6596	HSB0608	Male	Indian	2019	60	Married	Primary	Private	Urban	No	No	Yes	No	No	No	No	No	No	No
6597	HSB0609	Male	Malay	2019	57	Others	Secondary	Private	Rural	No	No	Yes	No	Yes	No	No	No	No	No
6598	HSB0610	Male	Malay	2019	32	Single	Secondary	Unemployed	Rural	No	Yes	No	No	No	No	No	No	No	Yes
6599	HSB0612	Male	Others	2018	36	Single	No formal education	Self-employed	Rural	No	Yes	Yes	No	No	No	No	No	No	No
6600	HSB0613	Male	Malay	2019	34	Single	Secondary	Unemployed	Rural	No	Yes	No	No	No	No	No	No	No	No
6601	HSB0614	Male	Malay	2019	31	Single	Secondary	Unemployed	Rural	No	No	No	No	Yes	No	No	No	No	Yes
6602	HSB0615	Male	Chinese	2019	43	Single	Tertiary	Private	Urban	No	Yes	No	No	No	No	Yes	No	No	No
6603	HSB0616	Male	Malay	2019	34	Single	No formal education	Private	Rural	No	No	No	No	No	No	No	No	Yes	No
6604	HSB0617	Male	Malay	2020	40	Married	Tertiary	Government	Rural	No	Yes	No	No	No	No	No	No	No	No
6605	HSB0619	Male	Malay	2019	59	Married	Secondary	Unemployed	Rural	No	Yes	No	No	No	No	No	No	No	No
6606	HSB0620	Male	Malay	2019	33	Single	Tertiary	Government	Rural	No	Yes	Yes	No	No	No	No	No	No	No
6607	HSB0621	Female	Malay	2019	55	Married	Primary	Unemployed	Urban	No	No	No	No	No	No	Yes	No	No	No
6608	HSB0623	Male	Malay	2019	43	Married	Tertiary	Unemployed	Urban	No	Yes	No	No	No	No	No	No	No	Yes
6609	HSB0624	Male	Indian	2020	57	Married	No formal education	Private	Rural	No	Yes	Yes	No	No	No	No	No	No	No
6610	HSB0625	Male	Malay	2020	33	Single	Tertiary	Unemployed	Rural	No	Yes	No	No	Yes	No	No	No	Yes	Yes
6611	HSB0626	Male	Malay	2020	30	Single	Tertiary	Government	Urban	No	Yes	No	No	No	No	No	No	No	No
6612	HSB0627	Female	Chinese	2020	53	Single	Tertiary	Government	Rural	No	No	Yes	No	No	No	No	No	No	No
6613	HSB0628	Female	Chinese	2020	38	Married	Secondary	Unemployed	Urban	No	Yes	Yes	No	No	No	No	No	No	No
6614	HSB0629	Male	Malay	2020	60	Married	Primary	Private	Rural	No	Yes	No	No	No	No	No	No	No	No
6615	HSB0631	Male	Malay	2021	24	Single	Secondary	Unemployed	Rural	No	Yes	Yes	Yes	Yes	No	No	No	Yes	Yes
6616	HSB0632	Male	Malay	2021	26	Single	No formal education	Unemployed	Urban	No	Yes	No	No	No	No	No	No	No	No
6617	HSB0633	Male	Malay	2021	33	Others	Tertiary	Self-employed	Rural	No	Yes	No	Yes	Yes	No	No	No	Yes	No
6618	HSB0635	Male	Malay	2020	50	Single	Secondary	Unemployed	Rural	Yes	Yes	No	No	No	No	No	No	No	Yes
6619	HSB0636	Male	Others	2020	61	Married	No formal education	Others	Rural	No	No	Yes	No	No	No	Yes	No	No	No
6620	HSB0637	Male	Indian	2020	30	Married	Secondary	Unemployed	Rural	No	No	Yes	No	Yes	No	No	No	No	No
6621	HSB0639	Male	Malay	2020	39	Married	Tertiary	Unemployed	Rural	No	Yes	No	No	Yes	No	No	No	No	No
6622	HSB0642	Male	Malay	2020	53	Married	Secondary	Private	Rural	Yes	Yes	No	No	No	No	No	No	No	No
6623	HSB0643	Male	Chinese	2020	31	Others	Secondary	Private	Rural	No	No	Yes	No	No	No	No	No	No	No
6624	HSB0644	Male	Malay	2020	43	Married	No formal education	Self-employed	Rural	Yes	No	No	No	Yes	No	No	No	Yes	Yes
6625	HSB0645	Male	Malay	2020	29	Single	No formal education	Unemployed	Rural	No	No	No	Yes	Yes	No	No	No	Yes	No
6626	HSB0646	Female	Malay	2020	31	Married	Primary	Unemployed	Rural	No	No	No	No	Yes	No	No	No	No	No
6627	HSB0648	Male	Malay	2020	36	Married	Secondary	Government	Rural	Yes	Yes	No	No	No	No	No	No	No	Yes

No	Patient ID	Gender	Ethnic group	Year of diagnosis	Age	Marital status	Education level	Occupation	Place of residence	History of psy illness	Tobacco	Alcohol	Cannabis	ATS	Inhalant	Sedative	Hallucinogens	Opioids	Kratom
6628	HSB0651	Male	Malay	2020	46	Single	Secondary	Others	Rural	No	No	No	No	Yes	No	No	No	Yes	No
6629	HSB0652	Male	Malay	2020	21	Others	Secondary	Others	Rural	No	No	No	No	Yes	No	No	No	Yes	No
6630	HSB0653	Male	Malay	2020	18	Single	Tertiary	Self-employed	Rural	No	Yes	No	No	No	No	No	No	No	No
6631	HSB0655	Male	Indian	2020	38	Married	Primary	Unemployed	Rural	No	Yes	Yes	No	Yes	No	No	No	No	No
6632	HSB0657	Male	Malay	2020	38	Others	Secondary	Self-employed	Rural	No	Yes	No	No	No	No	No	No	No	Yes
6633	HSB0658	Male	Malay	2020	45	Single	No formal education	Private	Rural	No	Yes	No	No	No	No	No	No	No	Yes
6634	HSB0659	Male	Chinese	2020	65	Married	Primary	Self-employed	Rural	No	No	Yes	No	No	No	No	No	No	No
6635	HSB0660	Male	Malay	2020	18	Others	Secondary	Self-employed	Rural	No	No	Yes	Yes	Yes	No	No	No	No	No
6636	HSB0661	Male	Malay	2020	22	Single	Secondary	Others	Rural	No	No	No	No	Yes	No	No	No	No	No
6637	HSB0662	Male	Malay	2020	27	Single	Primary	Private	Rural	No	No	No	No	Yes	No	No	No	No	Yes
6638	HSB0665	Male	Malay	2020	42	Others	Tertiary	Private	Rural	Yes	Yes	No	Yes	No	No	No	No	No	No
6639	HSB0666	Female	Malay	2020	26	Married	Secondary	Private	Rural	No	Yes	No	No	No	No	No	No	No	No
6640	HSB0667	Male	Malay	2020	30	Single	Tertiary	Unemployed	Rural	Yes	No	No	No	Yes	No	No	No	No	No
6641	HSB0668	Male	Malay	2020	36	Single	Tertiary	Self-employed	Rural	Yes	Yes	No	No	Yes	No	No	No	No	No
6642	HSB0669	Male	Malay	2020	22	Others	Secondary	Unemployed	Urban	No	No	No	No	Yes	No	No	No	No	No
6643	HSB0670	Male	Malay	2020	33	Single	No formal education	Others	Rural	Yes	No	No	No	Yes	No	No	No	No	No
6644	HSB0673	Male	Malay	2020	41	Others	No formal education	Unemployed	Rural	No	Yes	No	Yes	Yes	No	Yes	No	Yes	No
6645	HSB0674	Male	Malay	2020	28	Single	Secondary	Unemployed	Rural	No	Yes	No	No	Yes	No	No	No	No	No
6646	HSB0677	Male	Malay	2020	44	Others	No formal education	Self-employed	Rural	No	Yes	No	Yes	No	No	No	No	Yes	No
6647	HSB0678	Male	Indian	2020	36	Others	No formal education	Self-employed	Rural	No	Yes	No	Yes	Yes	No	No	No	Yes	No
6648	HSB0680	Male	Malay	2020	43	Single	Secondary	Unemployed	Rural	No	No	No	No	Yes	No	No	No	No	No
6649	HSB0681	Male	Malay	2020	36	Single	Secondary	Self-employed	Rural	No	Yes	No	No	No	No	No	No	No	Yes
6650	HSB0682	Male	Malay	2018	36	Single	No formal education	Unemployed	Rural	No	Yes	No	No	No	Yes	No	No	No	Yes
6651	HSB0684	Male	Malay	2021	22	Single	Tertiary	Self-employed	Rural	No	Yes	No	No	Yes	No	No	No	No	No
6652	HSB0685	Male	Malay	2021	43	Married	Secondary	Unemployed	Rural	No	Yes	No	Yes	Yes	No	No	No	No	Yes
6653	HSB0686	Male	Malay	2021	46	Single	No formal education	Unemployed	Urban	No	No	No	No	Yes	No	No	No	Yes	No
6654	HSB0690	Male	Malay	2021	36	Single	Secondary	Unemployed	Rural	No	No	No	No	Yes	No	No	No	No	No
6655	HSB0691	Male	Malay	2021	42	Married	Tertiary	Private	Rural	No	Yes	No	Yes	Yes	No	No	No	No	Yes
6656	HSB0692	Female	Malay	2021	29	Married	Primary	Private	Rural	No	No	Yes	No	Yes	No	No	No	No	No
6657	HSB0694	Male	Malay	2021	34	Others	Secondary	Self-employed	Rural	No	Yes	No	No	No	No	No	No	No	Yes
6658	HSB0696	Male	Indian	2021	38	Others	Tertiary	Unemployed	Urban	No	Yes	Yes	No	Yes	No	No	No	Yes	No
6659	HSB0697	Male	Malay	2020	30	Single	Secondary	Self-employed	Rural	Yes	Yes	No	No	Yes	No	No	No	Yes	Yes
6660	HSB0698	Male	Malay	2021	27	Others	Tertiary	Unemployed	Urban	No	Yes	No	No	Yes	No	No	No	No	No
6661	HSB0699	Male	Malay	2021	33	Single	Secondary	Self-employed	Rural	No	No	No	No	No	No	No	No	No	Yes
6662	HSB0700	Male	Malay	2021	40	Single	Primary	Unemployed	Rural	No	No	No	Yes	Yes	No	No	No	Yes	No
6663	HSB0701	Male	Malay	2021	35	Single	Secondary	Private	Urban	No	Yes	No	No	Yes	No	No	No	No	No

No	Patient ID	Gender	Ethnic group	Year of diagnosis	Age	Marital status	Education level	Occupation	Place of residence	History of psy illness	Tobacco	Alcohol	Cannabis	ATS	Inhalant	Sedative	Hallucinogens	Opioids	Kratom
6664	HSB0704	Male	Malay	2021	41	Single	Secondary	Self-employed	Rural	Yes	Yes	No	No	Yes	No	No	No	No	Yes
6665	HSB0705	Female	Chinese	2018	45	Married	Secondary	Unemployed	Urban	Yes	No	No	No	No	No	Yes	No	No	No
6666	HSB0712	Male	Malay	2021	55	Married	Secondary	Private	Rural	No	No	No	Yes	Yes	No	No	No	No	Yes
6667	HSB0713	Male	Malay	2021	40	Married	No formal education	Self-employed	Urban	No	No	No	No	Yes	No	No	No	No	Yes
6668	HSB0715	Male	Indian	2019	31	Married	Tertiary	Government	Urban	No	Yes	Yes	No	No	No	No	No	No	No
6669	HSB0717	Male	Malay	2018	33	Single	Secondary	Private	Urban	No	Yes	No	No	Yes	No	Yes	No	No	Yes
6670	HSB0719	Male	Malay	2021	37	Others	No formal education	Private	Rural	No	Yes	No	No	Yes	No	Yes	No	No	No
6671	HSB0720	Male	Chinese	2021	41	Single	No formal education	Private	Urban	Yes	No	No	No	No	No	Yes	No	No	No
6672	HSB0723	Male	Malay	2021	32	Single	Secondary	Unemployed	Urban	No	Yes	No	No	Yes	No	No	No	No	Yes
6673	HSB0726	Male	Malay	2021	34	Single	Tertiary	Self-employed	Rural	No	Yes	No	Yes	Yes	No	No	No	No	Yes
6674	HSB0727	Male	Malay	2021	31	Single	Secondary	Others	Rural	No	No	No	No	Yes	No	No	No	No	Yes
6675	HSB0728	Male	Malay	2021	37	Single	Primary	Unemployed	Urban	No	No	No	No	Yes	No	No	No	Yes	Yes
6676	HSB0730	Male	Malay	2021	27	Married	Tertiary	Self-employed	Urban	Yes	Yes	No	Yes	No	No	No	No	No	No
6677	HSB0732	Male	Malay	2021	48	Others	No formal education	Unemployed	Rural	Yes	Yes	No	No	Yes	No	No	No	No	No
6678	HSB0733	Male	Malay	2021	32	Others	Tertiary	Self-employed	Rural	No	No	No	Yes	Yes	No	No	No	No	No
6679	HSB0734	Male	Malay	2021	43	Married	Secondary	Unemployed	Rural	Yes	No	No	No	No	No	No	No	No	Yes
6680	HSB0735	Male	Malay	2021	48	Others	Secondary	Unemployed	Rural	No	No	No	Yes	Yes	No	No	No	Yes	Yes
6681	HSB0736	Male	Indian	2021	41	Single	Primary	Unemployed	Urban	No	No	No	Yes	Yes	No	No	No	Yes	No
6682	HSB0738	Male	Malay	2021	25	Single	Secondary	Private	Rural	No	Yes	No	No	Yes	No	No	No	No	No
6683	HSB0739	Male	Malay	2021	43	Married	No formal education	Unemployed	Rural	No	No	No	No	No	No	Yes	No	Yes	No
6684	HSB0742	Male	Malay	2021	32	Single	Tertiary	Unemployed	Rural	No	No	No	No	Yes	No	No	No	No	Yes
6685	HSB0748	Male	Malay	2021	29	Single	Secondary	Unemployed	Urban	No	Yes	No	Yes	Yes	No	No	No	Yes	Yes
6686	HSB0749	Female	Malay	2021	21	Others	Tertiary	Unemployed	Urban	No	No	No	No	Yes	No	Yes	No	No	No
6687	HSB0751	Male	Malay	2021	25	Single	Secondary	Self-employed	Rural	No	Yes	No	No	Yes	No	No	No	No	Yes
6688	HSB0752	Male	Malay	2021	33	Single	Secondary	Unemployed	Rural	No	Yes	No	No	No	No	No	No	No	Yes
6689	HSB0755	Male	Malay	2021	40	Single	Secondary	Unemployed	Rural	No	Yes	No	No	No	No	No	No	No	No
6690	HSB0756	Male	Malay	2021	40	Single	No formal education	Unemployed	Rural	No	No	Yes	No	Yes	No	No	No	No	No
6691	HSB0757	Male	Malay	2021	36	Married	Secondary	Self-employed	Rural	No	No	No	No	Yes	No	Yes	No	Yes	Yes
6692	HSB0758	Male	Malay	2021	40	Single	Primary	Unemployed	Rural	Yes	Yes	No	No	Yes	No	No	No	No	Yes
6693	HSB0759	Male	Malay	2021	45	Single	Secondary	Private	Rural	No	Yes	No	Yes	Yes	No	No	No	Yes	Yes
6694	HSB0761	Female	Malay	2021	23	Single	Tertiary	Unemployed	Urban	No	Yes	No	No	No	No	No	No	No	No
6695	HSB0762	Male	Malay	2021	24	Single	No formal education	Private	Rural	No	Yes	No	No	Yes	No	No	No	No	No
6696	HSB0763	Male	Malay	2021	21	Single	Secondary	Unemployed	Rural	Yes	Yes	No	Yes	Yes	No	No	No	Yes	Yes
6697	HSB0764	Male	Malay	2021	46	Others	Secondary	Unemployed	Urban	No	Yes	No	No	Yes	No	Yes	No	No	Yes
6698	HSB0765	Female	Chinese	2021	23	Single	Tertiary	Unemployed	Urban	Yes	No	Yes	No	No	No	No	No	No	No
6699	HSB0766	Female	Malay	2021	27	Others	Secondary	Unemployed	Urban	No	No	No	No	Yes	No	Yes	No	No	No
6700	HSB0768	Male	Malay	2021	37	Others	Primary	Self-employed	Rural	Yes	No	Yes	No	Yes	No	No	No	Yes	Yes

No	Patient ID	Gender	Ethnic group	Year of diagnosis	Age	Marital status	Education level	Occupation	Place of residence	History of psy illness	Tobacco	Alcohol	Cannabis	ATS	Inhalant	Sedative	Hallucinogens	Opioids	Kratom
6701	HSB0770	Male	Malay	2021	33	Single	Secondary	Self-employed	Rural	No	Yes	No	No	Yes	No	No	No	No	No
6702	HSB0771	Male	Indian	2021	52	Others	No formal education	Unemployed	Urban	No	Yes	No	No	No	No	No	No	No	No
6703	HSNZ0001	Male	Malay	2021	22	Married	Secondary	Self-employed	Rural	No	Yes	No	No	Yes	Yes	Yes	No	Yes	No
6704	HSNZ0002	Male	Malay	2021	22	Single	Secondary	Others	Urban	Yes	Yes	No	No	Yes	No	Yes	No	No	No
6705	HSNZ0003	Male	Malay	2021	35	Married	Tertiary	Private	Urban	Yes	Yes	No	No	Yes	No	No	No	No	Yes
6706	HSNZ0004	Male	Malay	2021	31	Single	Secondary	Others	Urban	Yes	Yes	Yes	Yes	Yes	Yes	No	Yes	Yes	Yes
6707	HSNZ0005	Male	Malay	2021	37	Married	Secondary	Self-employed	Urban	Yes	Yes	No	No	Yes	No	No	No	No	No
6708	HSNZ0007	Male	Malay	2019	30	Single	Secondary	Unemployed	Urban	Yes	Yes	No	No	Yes	No	No	No	No	Yes
6709	HSNZ0009	Male	Malay	2018	42	Single	Secondary	Others	Urban	Yes	Yes	No	No	Yes	No	No	No	No	No
6710	HSNZ0010	Male	Malay	2019	33	Single	Secondary	Self-employed	Urban	Yes	Yes	No	No	Yes	No	No	No	No	No
6711	HSNZ0011	Male	Malay	2020	37	Single	Secondary	Others	Urban	Yes	Yes	No	No	Yes	No	No	No	Yes	No
6712	HSNZ0012	Male	Malay	2020	29	Single	Secondary	Private	Urban	Yes	Yes	No	No	Yes	No	No	No	No	No
6713	HSNZ0013	Male	Malay	2019	21	Single	Secondary	Unemployed	Urban	No	Yes	No	No	Yes	No	No	No	No	No
6714	HSNZ0014	Male	Malay	2019	33	Single	Secondary	Unemployed	Urban	Yes	No	No	No	Yes	No	No	No	No	No
6715	HSNZ0015	Male	Malay	2021	34	Others	Secondary	Unemployed	Urban	Yes	Yes	No	No	Yes	No	No	No	No	No
6716	HSNZ0016	Male	Malay	2019	28	Single	Secondary	Private	Urban	No	Yes	No	No	Yes	No	No	No	No	No
6717	HSNZ0017	Male	Malay	2018	26	Single	Secondary	Unemployed	Urban	Yes	No	No	No	Yes	No	No	No	No	No
6718	HSNZ0018	Male	Malay	2021	57	Married	Primary	Unemployed	Urban	Yes	Yes	No	No	Yes	No	No	No	No	No
6719	HSNZ0019	Male	Malay	2018	31	Single	Secondary	Self-employed	Urban	Yes	Yes	No	No	No	No	No	No	No	No
6720	HSNZ0020	Male	Malay	2018	35	Single	Secondary	Unemployed	Urban	Yes	Yes	No	No	Yes	No	No	No	No	No
6721	HSNZ0021	Male	Malay	2019	29	Single	Secondary	Unemployed	Urban	No	Yes	No	No	Yes	No	No	No	Yes	No
6722	HSNZ0022	Female	Malay	2018	30	Others	Secondary	Unemployed	Urban	Yes	Yes	No	No	Yes	No	No	No	No	No

No	Patient ID	Gender	Ethnic group	Year of diagnosis	Age	Marital status	Education level	Occupation	Place of residence	History of psy illness	Tobacco	Alcohol	Cannabis	ATS	Inhalant	Sedative	Hallucinogens	Opioids	Kratom
6723	HSNZ0023	Male	Malay	2021	38	Others	Secondary	Unemployed	Urban	No	Yes	No	No	No	No	No	No	No	No
6724	HSNZ0024	Male	Malay	2020	21	Single	Secondary	Unemployed	Urban	Yes	Yes	Yes	Yes	No	No	No	No	No	Yes
6725	HSNZ0026	Male	Malay	2021	24	Single	Secondary	Unemployed	Urban	Yes	Yes	No	No	Yes	No	No	No	No	No
6726	HSNZ0027	Female	Malay	2021	39	Others	Secondary	Unemployed	Urban	Yes	Yes	No	No	Yes	No	No	No	No	No
6727	HSNZ0028	Male	Malay	2018	28	Single	Secondary	Unemployed	Urban	Yes	Yes	No	No	Yes	No	No	No	No	No
6728	HSNZ0030	Male	Malay	2018	51	Married	Secondary	Self-employed	Urban	No	No	No	No	Yes	No	No	No	No	No
6729	HSNZ0031	Male	Malay	2019	37	Single	Secondary	Private	Urban	No	No	No	No	Yes	No	No	No	No	No
6730	HSNZ0035	Male	Malay	2018	22	Single	Secondary	Private	Urban	No	Yes	No	No	Yes	No	No	No	No	No
6731	HSNZ0036	Male	Malay	2021	48	Single	Secondary	Self-employed	Urban	No	Yes	No	Yes	Yes	No	No	Yes	No	Yes
6732	HSNZ0038	Male	Malay	2019	32	Single	Secondary	Unemployed	Rural	Yes	Yes	No	No	Yes	No	No	No	No	No
6733	HSNZ0040	Male	Malay	2018	30	Single	Secondary	Unemployed	Rural	No	Yes	No	Yes	Yes	No	No	No	No	No
6734	HSNZ0042	Male	Malay	2020	31	Single	Secondary	Unemployed	Rural	No	Yes	No	No	Yes	No	No	No	No	No
6735	HSNZ0043	Male	Malay	2020	45	Others	Secondary	Unemployed	Rural	Yes	Yes	No	No	Yes	No	No	No	No	No
6736	HSNZ0044	Male	Malay	2018	36	Single	Secondary	Unemployed	Rural	No	Yes	No	No	Yes	No	No	No	No	No
6737	HSNZ0048	Male	Malay	2021	43	Married	Secondary	Self-employed	Rural	No	Yes	No	No	Yes	No	No	No	No	No
6738	HSNZ0051	Male	Malay	2020	25	Single	Secondary	Unemployed	Rural	No	Yes	No	No	Yes	No	No	No	No	No
6739	HSNZ0053	Female	Malay	2021	36	Single	Secondary	Unemployed	Rural	No	Yes	No	No	Yes	No	No	No	No	No
6740	HSNZ0054	Male	Malay	2020	38	Others	Secondary	Unemployed	Rural	No	Yes	No	No	Yes	No	No	No	No	No
6741	HSNZ0055	Male	Malay	2019	23	Single	Secondary	Unemployed	Rural	Yes	Yes	No	No	No	No	No	No	No	No
6742	HSNZ0057	Male	Malay	2018	42	Others	No formal education	Unemployed	Urban	No	No	No	No	No	No	No	No	Yes	No
6743	HSNZ0061	Male	Malay	2018	29	Single	Secondary	Unemployed	Urban	Yes	No	No	No	Yes	No	No	No	No	No

No	Patient ID	Gender	Ethnic group	Year of diagnosis	Age	Marital status	Education level	Occupation	Place of residence	History of psy illness	Tobacco	Alcohol	Cannabis	ATS	Inhalant	Sedative	Hallucinogens	Opioids	Kratom
6744	HSNZ0062	Male	Malay	2018	38	Single	No formal education	Self-employed	Urban	Yes	No	No	No	Yes	No	No	No	No	No
6745	HSNZ0063	Male	Malay	2018	54	Others	Secondary	Self-employed	Urban	No	Yes	No	No	No	No	No	No	Yes	No
6746	HSNZ0065	Male	Malay	2018	50	Married	No formal education	Self-employed	Urban	No	No	No	No	No	No	No	No	Yes	No
6747	HSNZ0066	Male	Malay	2018	53	Single	No formal education	Others	Urban	Yes	No	No	No	Yes	No	No	No	Yes	No
6748	HSNZ0067	Male	Malay	2018	59	Married	No formal education	Self-employed	Urban	Yes	Yes	No	No	Yes	No	No	No	Yes	No
6749	HSNZ0069	Male	Malay	2018	48	Single	No formal education	Others	Urban	No	No	No	No	No	No	No	No	Yes	No
6750	HSNZ0070	Male	Malay	2018	41	Single	Secondary	Unemployed	Urban	Yes	No	No	No	Yes	No	No	No	Yes	No
6751	HSNZ0072	Male	Malay	2018	29	Single	Secondary	Unemployed	Urban	Yes	No	No	No	Yes	No	No	No	No	No
6752	HSNZ0074	Male	Malay	2018	37	Single	No formal education	Private	Urban	Yes	No	No	No	No	No	No	No	Yes	No
6753	HSNZ0075	Male	Malay	2018	44	Single	Secondary	Unemployed	Urban	No	No	No	No	No	No	No	No	No	No
6754	HSNZ0077	Male	Malay	2018	33	Others	No formal education	Unemployed	Urban	No	No	No	No	Yes	No	Yes	No	Yes	No
6755	HSNZ0081	Male	Malay	2018	36	Single	No formal education	Unemployed	Urban	Yes	No	No	No	Yes	No	No	No	Yes	No
6756	HSNZ0082	Female	Malay	2018	31	Single	Tertiary	Unemployed	Urban	No	No	No	Yes	Yes	No	No	No	Yes	No
6757	HSNZ0083	Male	Malay	2018	33	Single	Secondary	Private	Urban	No	No	No	No	Yes	No	No	No	Yes	No
6758	HSNZ0084	Male	Malay	2018	34	Single	No formal education	Private	Urban	No	No	No	No	No	No	No	No	Yes	No
6759	HSNZ0085	Male	Malay	2018	49	Single	No formal education	Unemployed	Urban	No	No	No	No	No	No	No	No	Yes	No
6760	HSNZ0086	Male	Malay	2018	39	Single	No formal education	Unemployed	Urban	Yes	No	No	No	No	No	No	No	Yes	No
6761	HSNZ0087	Male	Malay	2018	36	Single	No formal education	Self-employed	Urban	Yes	No	No	Yes	No	No	Yes	No	Yes	No
6762	HSNZ0091	Male	Malay	2018	44	Married	No formal education	Unemployed	Urban	No	No	No	No	No	No	No	No	Yes	No
6763	HSNZ0094	Male	Malay	2019	39	Single	No formal education	Self-employed	Urban	No	No	No	No	Yes	No	No	No	Yes	No
6764	HSNZ0095	Male	Malay	2019	49	Others	No formal education	Unemployed	Urban	No	No	No	No	No	No	No	No	Yes	No

No	Patient ID	Gender	Ethnic group	Year of diagnosis	Age	Marital status	Education level	Occupation	Place of residence	History of psy illness	Tobacco	Alcohol	Cannabis	ATS	Inhalant	Sedative	Hallucinogens	Opioids	Kratom
6765	HSNZ0098	Male	Malay	2019	53	Others	No formal education	Self-employed	Urban	Yes	No	No	No	Yes	No	No	No	Yes	No
6766	HSNZ0099	Male	Malay	2019	33	Single	No formal education	Unemployed	Urban	No	No	No	No	No	No	No	No	No	No
6767	HSNZ0103	Male	Malay	2019	42	Married	No formal education	Self-employed	Urban	Yes	No	No	Yes	Yes	No	No	No	Yes	No
6768	HSNZ0105	Male	Malay	2019	43	Single	No formal education	Unemployed	Urban	No	No	No	No	Yes	No	No	No	Yes	No
6769	HSNZ0106	Female	Malay	2019	27	Married	Secondary	Self-employed	Urban	No	Yes	No	No	Yes	No	No	No	No	No
6770	HSNZ0107	Male	Malay	2019	43	Single	No formal education	Unemployed	Urban	No	No	No	No	No	No	No	No	Yes	No
6771	HSNZ0108	Male	Malay	2019	30	Single	Secondary	Unemployed	Urban	No	No	No	No	Yes	No	No	No	Yes	No
6772	HSNZ0109	Male	Malay	2019	42	Married	Secondary	Unemployed	Urban	No	No	No	No	No	No	No	No	Yes	No
6773	HSNZ0110	Male	Malay	2019	43	Single	No formal education	Unemployed	Urban	No	No	No	No	No	No	No	No	Yes	No
6774	HSNZ0115	Male	Malay	2019	21	Single	Secondary	Self-employed	Urban	No	No	No	No	Yes	No	No	No	Yes	No
6775	HSNZ0119	Male	Malay	2019	35	Single	Primary	Unemployed	Urban	No	No	No	Yes	No	No	Yes	No	Yes	No
6776	HSNZ0123	Male	Malay	2019	46	Others	No formal education	Others	Rural	No	Yes	No	No	No	No	No	No	Yes	No
6777	HSNZ0124	Male	Malay	2019	40	Single	Secondary	Unemployed	Urban	No	No	No	Yes	Yes	No	Yes	No	Yes	No
6778	HSNZ0125	Male	Malay	2019	51	Single	No formal education	Self-employed	Rural	No	No	No	Yes	No	No	No	No	Yes	No
6779	HSNZ0131	Male	Malay	2019	23	Single	No formal education	Private	Urban	No	No	No	No	Yes	No	No	No	No	No
6780	HSNZ0134	Male	Malay	2019	45	Others	No formal education	Unemployed	Urban	Yes	No	Yes	No	Yes	Yes	No	No	Yes	No
6781	HSNZ0135	Female	Malay	2019	35	Others	No formal education	Unemployed	Rural	No	No	No	No	Yes	No	No	No	Yes	No
6782	HSNZ0136	Male	Malay	2019	37	Married	No formal education	Private	Urban	No	Yes	No	No	No	No	No	No	No	No
6783	HSNZ0137	Female	Malay	2019	33	Married	No formal education	Unemployed	Urban	No	No	No	No	Yes	No	No	No	No	No
6784	HSNZ0139	Male	Malay	2019	37	Single	Secondary	Unemployed	Urban	No	No	No	No	Yes	No	No	No	Yes	No
6785	HSNZ0141	Male	Malay	2019	39	Others	No formal education	Unemployed	Urban	Yes	Yes	No	No	Yes	No	No	No	No	No

No	Patient ID	Gender	Ethnic group	Year of diagnosis	Age	Marital status	Education level	Occupation	Place of residence	History of psy illness	Tobacco	Alcohol	Cannabis	ATS	Inhalant	Sedative	Hallucinogens	Opioids	Kratom
6786	HSNZ0142	Male	Malay	2019	48	Married	No formal education	Others	Urban	No	No	No	No	No	No	No	No	Yes	Yes
6787	HSNZ0149	Female	Malay	2020	37	Others	No formal education	Unemployed	Urban	No	No	No	No	Yes	No	No	No	No	No
6788	HSNZ0153	Male	Malay	2020	46	Others	No formal education	Private	Rural	No	No	No	No	No	No	No	No	Yes	No
6789	HSNZ0154	Male	Malay	2020	48	Others	No formal education	Others	Urban	No	No	No	No	Yes	No	No	No	No	No
6790	HSNZ0156	Male	Malay	2020	20	Single	Secondary	Private	Urban	Yes	Yes	No	No	Yes	No	No	No	No	No
6791	HSNZ0157	Male	Malay	2020	47	Others	No formal education	Unemployed	Urban	No	No	No	No	Yes	No	No	No	Yes	No
6792	HSNZ0159	Male	Malay	2020	21	Single	No formal education	Unemployed	Urban	No	No	No	No	Yes	No	No	No	Yes	No
6793	HSNZ0164	Male	Malay	2020	31	Single	No formal education	Unemployed	Urban	Yes	Yes	No	No	Yes	No	No	No	No	No
6794	HSNZ0165	Male	Malay	2019	31	Others	No formal education	Self-employed	Urban	No	No	No	No	Yes	No	No	No	No	No
6795	HSNZ0166	Male	Malay	2019	24	Single	Secondary	Unemployed	Urban	No	Yes	No	No	Yes	No	No	No	No	No
6796	HSNZ0167	Male	Malay	2020	24	Single	Primary	Self-employed	Rural	No	No	No	No	Yes	No	No	No	No	No
6797	HSNZ0169	Male	Malay	2019	33	Married	Secondary	Unemployed	Urban	Yes	Yes	No	No	Yes	No	No	No	Yes	No
6798	HSNZ0170	Male	Malay	2020	44	Single	Secondary	Self-employed	Rural	No	No	No	Yes	Yes	No	No	No	No	No
6799	HSNZ0171	Male	Malay	2020	29	Single	Secondary	Self-employed	Urban	No	No	No	No	Yes	No	No	No	No	No
6800	HSNZ0172	Male	Malay	2020	40	Single	Secondary	Self-employed	Rural	No	No	No	No	Yes	No	No	No	Yes	No
6801	HSNZ0173	Male	Malay	2020	51	Married	No formal education	Others	Rural	No	Yes	No	No	No	No	No	No	Yes	No
6802	HSNZ0174	Male	Malay	2018	36	Single	No formal education	Unemployed	Urban	Yes	Yes	No	No	Yes	No	No	No	No	No
6803	HSNZ0180	Male	Malay	2020	25	Single	No formal education	Private	Urban	No	Yes	No	No	No	No	No	Yes	No	No
6804	HSNZ0182	Male	Malay	2020	45	Single	No formal education	Private	Urban	No	No	No	No	No	No	No	No	Yes	No
6805	HSNZ0186	Male	Malay	2020	33	Single	Secondary	Unemployed	Urban	Yes	No	No	No	Yes	No	No	No	No	No
6806	HSNZ0194	Male	Malay	2018	42	Others	Secondary	Self-employed	Rural	No	No	No	No	No	No	No	No	Yes	No

No	Patient ID	Gender	Ethnic group	Year of diagnosis	Age	Marital status	Education level	Occupation	Place of residence	History of psy illness	Tobacco	Alcohol	Cannabis	ATS	Inhalant	Sedative	Hallucinogens	Opioids	Kratom
6807	HSNZ0196	Female	Malay	2018	35	Married	No formal education	Others	Rural	No	No	No	No	Yes	No	No	No	No	No
6808	HSNZ0197	Male	Malay	2018	55	Others	No formal education	Self-employed	Rural	No	Yes	No	No	Yes	No	No	No	No	No
6809	HSNZ0200	Male	Malay	2018	38	Others	Secondary	Self-employed	Rural	No	Yes	No	No	Yes	No	No	No	No	No
6810	HSNZ0201	Male	Malay	2018	35	Married	No formal education	Self-employed	Rural	No	No	No	No	Yes	No	No	No	No	No
6811	HSNZ0205	Male	Malay	2018	30	Single	Primary	Unemployed	Rural	No	Yes	No	No	Yes	No	No	No	No	No
6812	HSNZ0210	Male	Malay	2018	41	Single	No formal education	Others	Rural	No	Yes	No	No	No	No	No	No	Yes	No
6813	HSNZ0214	Male	Malay	2018	30	Single	Tertiary	Unemployed	Rural	No	Yes	No	No	Yes	No	No	No	No	No
6814	HSNZ0216	Male	Malay	2018	48	Single	No formal education	Private	Urban	No	Yes	No	No	Yes	No	No	No	No	No
6815	HSNZ0217	Male	Malay	2018	32	Single	Secondary	Unemployed	Rural	No	No	No	No	No	No	No	No	Yes	No
6816	HSNZ0218	Male	Malay	2018	23	Single	Tertiary	Unemployed	Rural	Yes	Yes	No	Yes	No	No	No	No	No	No
6817	HSNZ0219	Male	Malay	2018	44	Others	No formal education	Private	Urban	No	Yes	No	No	No	No	No	No	Yes	No
6818	HSNZ0221	Male	Malay	2018	41	Single	Secondary	Unemployed	Rural	No	No	No	No	Yes	No	No	No	Yes	No
6819	HSNZ0224	Male	Malay	2018	36	Married	No formal education	Self-employed	Rural	No	Yes	No	No	Yes	No	No	No	No	No
6820	HSNZ0227	Male	Malay	2018	18	Single	Secondary	Private	Rural	No	Yes	Yes	No	Yes	No	No	No	No	No
6821	HSNZ0228	Male	Others	2018	44	Married	No formal education	Self-employed	Urban	No	Yes	No	No	Yes	No	No	No	Yes	No
6822	HSNZ0232	Male	Malay	2018	48	Married	No formal education	Self-employed	Rural	Yes	Yes	No	No	Yes	No	No	No	No	No
6823	HSNZ0234	Male	Malay	2018	30	Single	No formal education	Private	Rural	Yes	No	No	Yes	No	No	No	No	No	No
6824	HSNZ0235	Male	Malay	2018	33	Single	No formal education	Private	Rural	No	Yes	No	No	Yes	No	No	No	No	No
6825	HSNZ0239	Male	Malay	2018	29	Married	Secondary	Unemployed	Urban	No	No	No	No	Yes	No	No	No	No	No
6826	HSNZ0242	Male	Malay	2018	28	Single	No formal education	Unemployed	Urban	No	No	No	No	No	No	No	No	Yes	No
6827	HSNZ0243	Male	Chinese	2018	32	Married	No formal education	Self-employed	Urban	No	No	No	No	Yes	No	No	No	No	No

No	Patient ID	Gender	Ethnic group	Year of diagnosis	Age	Marital status	Education level	Occupation	Place of residence	History of psy illness	Tobacco	Alcohol	Cannabis	ATS	Inhalant	Sedative	Hallucinogens	Opioids	Kratom
6828	HSNZ0244	Male	Malay	2018	25	Single	Tertiary	Self-employed	Urban	No	Yes	No	Yes	No	No	No	No	No	No
6829	HSNZ0245	Male	Malay	2018	30	Single	Secondary	Unemployed	Rural	No	Yes	No	No	Yes	No	No	No	No	No
6830	HSNZ0247	Male	Malay	2018	33	Others	Secondary	Unemployed	Rural	No	No	No	No	Yes	No	No	No	No	No
6831	HSNZ0248	Male	Malay	2018	63	Single	No formal education	Unemployed	Rural	No	Yes	No	No	No	No	No	No	No	No
6832	HSNZ0249	Male	Malay	2018	36	Single	Secondary	Unemployed	Rural	No	Yes	No	No	Yes	No	No	No	No	No
6833	HSNZ0251	Female	Malay	2018	21	Married	No formal education	Unemployed	Rural	No	Yes	No	No	Yes	No	No	No	No	No
6834	HSNZ0252	Male	Malay	2018	42	Married	No formal education	Unemployed	Rural	No	Yes	No	No	No	No	No	No	No	No
6835	HSNZ0253	Male	Malay	2018	31	Single	No formal education	Self-employed	Rural	No	No	No	No	Yes	No	No	No	No	No
6836	HSNZ0254	Female	Malay	2018	20	Single	No formal education	Private	Urban	No	No	No	No	Yes	No	No	No	No	No
6837	HSNZ0255	Male	Malay	2018	24	Others	Secondary	Self-employed	Rural	No	No	No	No	Yes	No	Yes	No	No	No
6838	HSNZ0258	Male	Malay	2018	26	Single	Secondary	Unemployed	Rural	No	Yes	No	No	Yes	No	No	No	No	No
6839	HSNZ0259	Male	Malay	2018	38	Single	Secondary	Self-employed	Rural	No	Yes	No	No	Yes	No	No	No	No	Yes
6840	HSNZ0261	Male	Malay	2018	46	Single	No formal education	Self-employed	Rural	No	Yes	No	No	Yes	No	No	No	No	No
6841	HSNZ0263	Male	Malay	2019	35	Single	Primary	Unemployed	Rural	No	No	No	No	Yes	No	No	No	No	No
6842	HSNZ0264	Female	Chinese	2019	19	Single	Tertiary	Unemployed	Urban	Yes	No	No	No	Yes	No	No	No	No	No
6843	HSNZ0265	Male	Malay	2018	28	Single	Secondary	Unemployed	Rural	No	Yes	No	No	No	No	No	No	No	No
6844	HSNZ0266	Female	Malay	2019	31	Married	Secondary	Unemployed	Rural	No	Yes	No	No	Yes	No	No	No	No	No
6845	HSNZ0267	Male	Malay	2019	32	Others	No formal education	Self-employed	Rural	No	No	No	No	Yes	No	No	No	No	Yes
6846	HSNZ0268	Male	Malay	2019	38	Married	No formal education	Self-employed	Rural	Yes	Yes	No	No	Yes	No	No	No	Yes	No
6847	HSNZ0269	Male	Malay	2019	30	Married	No formal education	Unemployed	Rural	No	No	No	No	Yes	No	No	No	No	No
6848	HSNZ0274	Male	Malay	2019	37	Others	No formal education	Self-employed	Urban	No	No	No	No	Yes	No	No	No	No	No

No	Patient ID	Gender	Ethnic group	Year of diagnosis	Age	Marital status	Education level	Occupation	Place of residence	History of psy illness	Tobacco	Alcohol	Cannabis	ATS	Inhalant	Sedative	Hallucinogens	Opioids	Kratom
6849	HSNZ0275	Male	Malay	2020	30	Single	Secondary	Unemployed	Urban	No	Yes	No	No	Yes	No	No	No	No	No
6850	HSNZ0277	Female	Malay	2019	37	Married	No formal education	Unemployed	Urban	No	Yes	No	No	Yes	No	No	No	No	No
6851	HSNZ0279	Male	Malay	2018	38	Married	Secondary	Unemployed	Urban	Yes	Yes	No	Yes	Yes	No	No	No	No	No
6852	HSNZ0281	Male	Malay	2020	30	Others	Secondary	Unemployed	Urban	No	No	No	No	Yes	No	No	No	No	No
6853	HSNZ0283	Male	Malay	2019	37	Single	No formal education	Self-employed	Urban	No	No	No	No	Yes	No	No	No	No	No
6854	HSNZ0284	Male	Malay	2019	25	Single	Secondary	Unemployed	Urban	No	No	No	No	Yes	No	No	No	No	No
6855	HSNZ0287	Male	Malay	2019	55	Married	No formal education	Self-employed	Rural	No	Yes	No	No	Yes	No	No	No	No	No
6856	HSNZ0288	Male	Indian	2020	21	Single	Secondary	Private	Urban	No	No	Yes	No	No	No	No	No	No	No
6857	HSNZ0291	Male	Malay	2019	27	Single	Tertiary	Unemployed	Urban	No	Yes	No	Yes	Yes	No	No	No	No	No
6858	HSNZ0293	Male	Malay	2019	27	Single	Secondary	Unemployed	Urban	No	No	No	No	No	No	Yes	No	No	No
6859	HSNZ0294	Male	Malay	2019	33	Single	No formal education	Self-employed	Urban	Yes	No	No	No	Yes	No	No	No	Yes	No
6860	HSNZ0297	Male	Malay	2020	33	Single	Secondary	Private	Urban	No	No	No	Yes	No	No	No	No	No	No
6861	HSNZ0298	Male	Malay	2019	22	Single	Secondary	Unemployed	Urban	No	Yes	No	No	Yes	No	No	No	No	No
6862	HSNZ0301	Male	Malay	2020	31	Married	No formal education	Others	Urban	Yes	No	No	No	No	No	No	No	No	No
6863	HSNZ0307	Male	Malay	2019	42	Married	Secondary	Private	Urban	No	No	No	No	Yes	No	No	No	No	No
6864	HSNZ0311	Male	Malay	2018	42	Single	Secondary	Private	Urban	No	Yes	No	No	Yes	No	No	No	No	No
6865	HSNZ0314	Female	Malay	2019	26	Others	Secondary	Unemployed	Urban	Yes	Yes	No	No	No	No	No	No	No	No
6866	HSNZ0318	Male	Malay	2019	39	Married	No formal education	Self-employed	Urban	No	Yes	No	No	Yes	No	No	No	Yes	No
6867	HSNZ0320	Female	Malay	2019	57	Married	No formal education	Unemployed	Urban	No	Yes	No	No	No	No	No	No	Yes	No
6868	HSNZ0321	Male	Malay	2020	38	Married	No formal education	Others	Rural	No	No	No	No	Yes	No	No	No	No	No
6869	HSNZ0322	Male	Malay	2019	58	Single	No formal education	Self-employed	Rural	Yes	Yes	No	No	No	No	No	No	No	No

No	Patient ID	Gender	Ethnic group	Year of diagnosis	Age	Marital status	Education level	Occupation	Place of residence	History of psy illness	Tobacco	Alcohol	Cannabis	ATS	Inhalant	Sedative	Hallucinogens	Opioids	Kratom
6870	HSNZ0324	Male	Malay	2019	33	Single	No formal education	Unemployed	Urban	No	No	No	No	Yes	No	No	No	Yes	No
6871	HSNZ0325	Male	Malay	2020	27	Single	Tertiary	Unemployed	Urban	No	Yes	No	Yes	Yes	No	No	No	No	No
6872	HSNZ0326	Male	Malay	2020	26	Married	Secondary	Self-employed	Urban	Yes	No	No	No	Yes	No	No	No	No	No
6873	HSNZ0327	Male	Malay	2020	19	Single	Secondary	Others	Urban	No	No	No	No	Yes	No	No	No	No	No
6874	HSNZ0328	Male	Malay	2019	29	Married	Tertiary	Self-employed	Urban	No	Yes	No	No	Yes	No	No	No	No	No
6875	HSNZ0330	Male	Malay	2019	26	Single	Secondary	Self-employed	Urban	Yes	Yes	No	No	Yes	No	No	No	Yes	No
6876	HSNZ0334	Male	Malay	2020	41	Others	No formal education	Unemployed	Urban	No	No	No	No	No	Yes	No	No	No	No
6877	HSNZ0335	Male	Malay	2019	54	Single	No formal education	Unemployed	Urban	No	No	No	No	Yes	No	No	No	Yes	No
6878	HSNZ0336	Male	Others	2019	35	Married	No formal education	Self-employed	Urban	No	Yes	Yes	Yes	No	No	No	No	No	No
6879	HSNZ0337	Female	Malay	2018	21	Others	Secondary	Private	Urban	Yes	Yes	No	No	No	No	No	No	No	No
6880	HSNZ0338	Male	Malay	2019	31	Single	Tertiary	Self-employed	Urban	No	No	No	No	Yes	No	No	No	No	No
6881	HSNZ0339	Male	Malay	2019	36	Single	No formal education	Unemployed	Urban	Yes	No	No	No	Yes	No	No	No	No	No
6882	HSNZ0343	Male	Malay	2019	19	Single	Tertiary	Unemployed	Urban	Yes	Yes	No	No	No	No	No	No	No	No
6883	HSNZ0345	Female	Malay	2020	30	Others	No formal education	Private	Urban	No	No	No	No	Yes	No	No	No	No	No
6884	HSNZ0346	Male	Others	2020	47	Others	No formal education	Unemployed	Urban	Yes	No	Yes	No	Yes	No	No	No	No	No
6885	HSNZ0348	Male	Malay	2019	34	Married	No formal education	Self-employed	Urban	Yes	Yes	No	No	No	No	No	No	No	No
6886	HSNZ0349	Male	Malay	2019	29	Single	Secondary	Self-employed	Urban	Yes	Yes	No	No	Yes	No	No	No	No	No
6887	HSNZ0351	Male	Malay	2020	34	Single	Secondary	Others	Urban	No	No	No	No	Yes	No	No	No	No	Yes
6888	HSNZ0352	Male	Malay	2019	33	Married	No formal education	Self-employed	Urban	No	Yes	No	No	No	No	No	No	No	No
6889	HSNZ0353	Female	Malay	2019	25	Married	No formal education	Unemployed	Urban	Yes	No	No	No	Yes	No	No	No	No	No
6890	HSNZ0354	Male	Malay	2019	28	Single	Secondary	Self-employed	Urban	No	Yes	Yes	Yes	Yes	No	No	No	Yes	No

No	Patient ID	Gender	Ethnic group	Year of diagnosis	Age	Marital status	Education level	Occupation	Place of residence	History of psy illness	Tobacco	Alcohol	Cannabis	ATS	Inhalant	Sedative	Hallucinogens	Opioids	Kratom
6891	HSNZ0355	Male	Malay	2019	42	Single	Secondary	Unemployed	Urban	Yes	Yes	No	No	No	No	No	No	No	No
6892	HSNZ0356	Female	Malay	2019	38	Others	Secondary	Private	Urban	No	Yes	Yes	No	No	No	No	No	No	No
6893	HSNZ0359	Male	Malay	2019	32	Single	Secondary	Unemployed	Urban	No	No	No	No	Yes	No	No	No	No	Yes
6894	HSNZ0360	Female	Malay	2019	15	Single	Secondary	Unemployed	Urban	No	Yes	No	No	No	No	No	No	No	No
6895	HSNZ0361	Male	Malay	2019	16	Single	Secondary	Unemployed	Rural	Yes	Yes	No	No	No	No	No	No	No	No
6896	HSNZ0362	Male	Malay	2019	46	Married	Secondary	Private	Urban	Yes	Yes	No	No	Yes	No	No	No	No	No
6897	HSNZ0363	Male	Malay	2019	23	Single	No formal education	Self-employed	Rural	No	No	No	No	Yes	No	No	No	No	No
6898	HSNZ0365	Male	Malay	2019	46	Single	No formal education	Self-employed	Urban	No	Yes	No	No	Yes	No	No	No	No	No
6899	HSNZ0366	Male	Malay	2018	53	Single	No formal education	Unemployed	Urban	Yes	Yes	No	No	No	No	No	No	No	No
6900	HSNZ0367	Male	Malay	2019	48	Single	Secondary	Self-employed	Urban	No	No	No	No	Yes	No	No	No	No	No
6901	HSNZ0369	Male	Others	2018	37	Others	Primary	Self-employed	Urban	No	No	No	No	Yes	No	No	No	No	No
6902	HSNZ0372	Male	Malay	2020	37	Others	Secondary	Unemployed	Rural	No	Yes	No	No	Yes	No	No	No	No	No
6903	HSNZ0374	Male	Malay	2020	42	Married	Primary	Others	Urban	Yes	No	No	No	Yes	No	No	No	No	Yes
6904	HSNZ0376	Male	Malay	2019	27	Married	Tertiary	Others	Rural	Yes	No	No	No	Yes	No	No	No	No	No
6905	HSNZ0378	Male	Malay	2019	34	Single	No formal education	Others	Urban	No	Yes	No	No	No	No	No	No	No	No
6906	HSNZ0379	Male	Malay	2021	29	Others	Tertiary	Private	Urban	No	Yes	No	No	Yes	No	No	No	No	No
6907	HSNZ0380	Male	Malay	2020	40	Others	Secondary	Unemployed	Urban	Yes	No	No	Yes	Yes	Yes	No	No	No	No
6908	HSNZ0382	Male	Malay	2020	21	Single	No formal education	Unemployed	Urban	Yes	Yes	No	No	Yes	No	No	No	No	No
6909	HSNZ0383	Male	Malay	2021	22	Others	Tertiary	Private	Rural	No	Yes	No	No	Yes	No	No	No	Yes	Yes
6910	HSNZ0384	Male	Malay	2019	28	Single	Secondary	Private	Urban	No	No	No	No	Yes	No	No	No	No	No
6911	HSNZ0386	Male	Malay	2020	43	Single	No formal education	Unemployed	Rural	Yes	Yes	No	Yes	Yes	No	No	No	No	No

No	Patient ID	Gender	Ethnic group	Year of diagnosis	Age	Marital status	Education level	Occupation	Place of residence	History of psy illness	Tobacco	Alcohol	Cannabis	ATS	Inhalant	Sedative	Hallucinogens	Opioids	Kratom
6912	HSNZ0387	Male	Malay	2021	39	Married	No formal education	Unemployed	Rural	Yes	No	No	No	Yes	No	No	No	No	No
6913	HSNZ0388	Male	Malay	2020	40	Others	No formal education	Unemployed	Rural	Yes	No	No	No	Yes	No	No	No	No	No
6914	HSNZ0389	Female	Malay	2020	30	Others	No formal education	Unemployed	Urban	Yes	Yes	No	No	Yes	No	No	No	No	No
6915	HSNZ0390	Male	Malay	2021	30	Single	Secondary	Unemployed	Urban	No	Yes	No	No	Yes	No	No	No	No	No
6916	HSNZ0391	Male	Malay	2020	36	Married	No formal education	Unemployed	Urban	Yes	No	No	No	Yes	No	No	No	No	No
6917	HSNZ0392	Male	Malay	2021	15	Single	Secondary	Unemployed	Rural	No	Yes	No	Yes	Yes	No	No	No	No	Yes
6918	HSNZ0394	Male	Malay	2021	34	Others	Secondary	Unemployed	Urban	No	Yes	No	No	Yes	No	No	No	No	No
6919	HSNZ0395	Male	Malay	2021	22	Married	Secondary	Private	Urban	No	No	No	No	Yes	No	Yes	No	No	No
6920	HSNZ0400	Male	Malay	2020	38	Others	Secondary	Private	Urban	Yes	No	No	No	Yes	No	No	No	No	No
6921	HSNZ0401	Male	Malay	2021	51	Single	No formal education	Self-employed	Rural	Yes	No	No	No	Yes	No	No	No	Yes	No
6922	HSNZ0402	Male	Malay	2020	33	Single	No formal education	Unemployed	Urban	Yes	Yes	No	No	Yes	No	No	No	No	No
6923	HSNZ0404	Male	Malay	2021	35	Married	Secondary	Unemployed	Urban	Yes	Yes	No	No	Yes	No	No	No	No	No
6924	HSNZ0405	Male	Malay	2021	47	Others	Tertiary	Private	Rural	Yes	Yes	No	No	Yes	No	Yes	No	Yes	Yes
6925	HSNZ0408	Male	Malay	2021	37	Others	Secondary	Private	Urban	No	Yes	No	No	Yes	No	No	No	No	No
6926	HSNZ0409	Male	Malay	2021	23	Single	No formal education	Private	Urban	No	Yes	No	Yes	No	No	No	No	No	No
6927	HSNZ0413	Male	Malay	2021	37	Married	Secondary	Government	Urban	No	Yes	No	No	Yes	No	No	No	No	No
6928	HSNZ0414	Female	Malay	2021	36	Single	No formal education	Private	Urban	Yes	No	No	Yes	Yes	No	No	No	No	Yes
6929	HSNZ0416	Male	Malay	2021	18	Single	Primary	Unemployed	Rural	Yes	Yes	No	Yes	Yes	No	No	No	No	No
6930	HSNZ0418	Female	Malay	2021	22	Married	Secondary	Others	Rural	Yes	Yes	No	No	Yes	No	No	No	No	No
6931	HSNZ0419	Female	Malay	2020	21	Single	Tertiary	Unemployed	Rural	Yes	No	No	No	No	No	Yes	No	No	No
6932	HSNZ0420	Female	Malay	2021	29	Married	No formal education	Others	Rural	No	No	No	No	Yes	No	No	No	No	No

No	Patient ID	Gender	Ethnic group	Year of diagnosis	Age	Marital status	Education level	Occupation	Place of residence	History of psy illness	Tobacco	Alcohol	Cannabis	ATS	Inhalant	Sedative	Hallucinogens	Opioids	Kratom
6933	HSNZ0421	Female	Malay	2020	53	Married	No formal education	Others	Urban	Yes	No	No	No	No	No	No	No	Yes	No
6934	HSNZ0424	Female	Malay	2020	30	Others	No formal education	Others	Urban	Yes	No	No	No	Yes	No	No	No	No	No
6935	HSNZ0425	Male	Malay	2021	24	Single	Tertiary	Others	Urban	Yes	No	No	No	Yes	No	No	No	No	No
6936	HSNZ0426	Female	Malay	2020	43	Others	No formal education	Others	Rural	Yes	No	No	No	Yes	No	No	No	Yes	No
6937	HSNZ0427	Male	Malay	2020	38	Others	No formal education	Self-employed	Urban	Yes	No	No	No	Yes	No	No	No	No	No
6938	HSNZ0428	Male	Malay	2020	46	Others	No formal education	Others	Urban	No	No	No	No	Yes	No	No	No	No	No
6939	HSNZ0429	Female	Others	2021	19	Married	Tertiary	Unemployed	Urban	Yes	No	No	No	Yes	No	No	No	No	No
6940	HSNZ0430	Male	Malay	2020	41	Single	No formal education	Unemployed	Urban	No	No	No	No	Yes	No	No	No	No	No
6941	HSNZ0431	Female	Malay	2021	22	Single	Tertiary	Private	Rural	Yes	Yes	No	No	No	No	No	Yes	No	No
6942	HSNZ0432	Female	Malay	2021	17	Single	Secondary	Unemployed	Rural	Yes	No	No	No	Yes	No	No	No	No	No
6943	HSNZ0434	Female	Chinese	2021	37	Married	Secondary	Private	Rural	Yes	Yes	No	No	No	No	No	No	No	No
6944	HSNZ0435	Female	Others	2021	48	Married	No formal education	Unemployed	Rural	No	Yes	No	No	No	No	No	No	No	No
6945	HSNZ0437	Male	Malay	2020	27	Others	No formal education	Self-employed	Rural	Yes	No	No	No	Yes	No	No	No	No	No
6946	HSNZ0438	Male	Malay	2021	25	Single	Secondary	Unemployed	Urban	Yes	No	No	No	Yes	No	No	No	No	No
6947	HSNZ0439	Male	Malay	2021	46	Married	Secondary	Private	Rural	Yes	Yes	No	No	No	No	Yes	No	No	No
6948	HSNZ0441	Male	Malay	2021	41	Others	No formal education	Unemployed	Rural	Yes	No	No	No	No	No	No	No	Yes	No
6949	HSNZ0442	Male	Malay	2021	42	Others	No formal education	Private	Rural	Yes	Yes	No	No	No	No	No	No	Yes	Yes
6950	HSNZ0443	Male	Malay	2021	55	Married	Primary	Unemployed	Rural	Yes	Yes	No	No	No	No	No	No	No	No
6951	HSNZ0444	Male	Malay	2021	19	Single	Primary	Private	Urban	No	No	No	No	Yes	No	No	No	No	Yes
6952	HSNZ0445	Male	Malay	2021	29	Single	Secondary	Unemployed	Urban	Yes	Yes	No	No	Yes	No	No	No	No	No
6953	HSNZ0446	Male	Malay	2021	17	Single	Secondary	Unemployed	Urban	Yes	Yes	No	No	No	No	No	No	No	No

No	Patient ID	Gender	Ethnic group	Year of diagnosis	Age	Marital status	Education level	Occupation	Place of residence	History of psy illness	Tobacco	Alcohol	Cannabis	ATS	Inhalant	Sedative	Hallucinogens	Opioids	Kratom
6954	HSNZ0447	Male	Malay	2021	35	Single	No formal education	Others	Rural	No	No	No	No	Yes	No	No	No	No	No
6955	HSNZ0448	Male	Malay	2021	38	Others	No formal education	Private	Urban	Yes	No	No	No	Yes	No	No	No	No	No
6956	HSNZ0449	Male	Malay	2021	33	Single	Tertiary	Unemployed	Rural	Yes	Yes	No	No	Yes	No	No	No	No	No
6957	HSNZ0450	Male	Malay	2021	40	Single	Secondary	Private	Rural	Yes	Yes	No	Yes	Yes	No	No	No	No	No
6958	HSNZ0451	Male	Malay	2021	43	Others	Secondary	Unemployed	Rural	No	No	No	Yes	Yes	No	No	No	Yes	No
6959	HSNZ0452	Male	Malay	2018	27	Single	No formal education	Unemployed	Urban	No	No	No	Yes	Yes	No	No	No	No	No
6960	HSNZ0453	Male	Malay	2021	48	Single	Secondary	Private	Urban	Yes	No	No	No	Yes	No	No	No	No	Yes
6961	HSNZ0455	Male	Malay	2021	25	Single	Secondary	Private	Rural	Yes	No	No	No	Yes	No	No	No	No	No
6962	HSNZ0457	Male	Malay	2019	31	Others	Secondary	Unemployed	Rural	Yes	Yes	No	Yes	Yes	No	No	No	No	Yes
6963	HSNZ0460	Male	Malay	2021	42	Others	Secondary	Unemployed	Urban	No	No	No	No	Yes	No	No	No	No	No
6964	HSNZ0462	Male	Malay	2021	22	Single	Secondary	Private	Urban	Yes	Yes	Yes	Yes	Yes	No	No	Yes	No	No
6965	HSNZ0464	Male	Malay	2021	35	Single	Tertiary	Unemployed	Urban	No	No	No	Yes	Yes	No	No	No	No	No
6966	HSNZ0465	Male	Malay	2021	28	Single	Secondary	Unemployed	Rural	Yes	No	No	No	Yes	No	No	No	No	No
6967	HSNZ0466	Male	Malay	2021	28	Single	Secondary	Unemployed	Rural	No	No	No	No	Yes	No	No	No	No	No
6968	HSNZ0468	Male	Malay	2021	22	Single	No formal education	Unemployed	Urban	No	Yes	No	No	Yes	No	No	No	No	No
6969	HSNZ0469	Female	Malay	2021	28	Single	Secondary	Private	Urban	Yes	Yes	No	Yes	Yes	No	No	No	No	No
6970	HSNZ0471	Male	Malay	2021	43	Married	No formal education	Others	Rural	No	Yes	No	No	Yes	No	No	No	No	No
6971	HSNZ0472	Male	Malay	2021	36	Single	Secondary	Private	Rural	No	Yes	No	No	Yes	No	No	No	No	No
6972	HSNZ0474	Male	Malay	2021	31	Single	Secondary	Unemployed	Urban	No	Yes	No	No	Yes	No	No	No	No	No
6973	HSNZ0476	Male	Malay	2021	33	Others	Primary	Self-employed	Rural	No	No	No	No	Yes	No	No	No	No	No
6974	HSNZ0480	Male	Malay	2021	21	Single	Secondary	Unemployed	Rural	No	Yes	No	No	No	No	No	Yes	No	No

No	Patient ID	Gender	Ethnic group	Year of diagnosis	Age	Marital status	Education level	Occupation	Place of residence	History of psy illness	Tobacco	Alcohol	Cannabis	ATS	Inhalant	Sedative	Hallucinogens	Opioids	Kratom
6975	HSNZ0481	Male	Malay	2021	44	Married	Secondary	Unemployed	Rural	Yes	No	No	No	Yes	No	No	No	No	No
6976	HSNZ0482	Male	Malay	2021	28	Single	Tertiary	Private	Rural	Yes	Yes	No	Yes	Yes	No	No	No	No	No
6977	HSNZ0483	Male	Malay	2021	24	Married	Secondary	Self-employed	Urban	Yes	Yes	No	No	No	No	No	No	No	Yes
6978	HSNZ0484	Male	Malay	2021	29	Single	Secondary	Self-employed	Rural	Yes	No	No	No	Yes	No	No	No	No	No
6979	HSNZ0485	Male	Malay	2021	21	Single	Secondary	Unemployed	Rural	Yes	Yes	No	No	Yes	No	No	No	No	No
6980	HSNZ0486	Male	Malay	2021	25	Single	No formal education	Self-employed	Rural	Yes	Yes	No	No	Yes	No	No	No	No	No
6981	HSNZ0487	Male	Malay	2021	40	Married	Tertiary	Unemployed	Urban	Yes	Yes	No	No	Yes	No	No	No	No	No
6982	HSNZ0488	Male	Malay	2021	18	Single	Secondary	Self-employed	Rural	Yes	No	No	No	Yes	No	No	Yes	No	No
6983	HSNZ0489	Male	Malay	2021	46	Single	No formal education	Unemployed	Rural	Yes	No	No	Yes	Yes	No	No	No	Yes	No
6984	HSNZ0490	Male	Malay	2021	46	Single	No formal education	Self-employed	Rural	Yes	Yes	No	No	Yes	No	Yes	No	No	No
6985	HSNZ0492	Male	Malay	2021	32	Single	Secondary	Unemployed	Rural	Yes	No	No	Yes	Yes	No	No	No	No	No
6986	HSNZ0494	Male	Malay	2021	16	Single	Secondary	Unemployed	Urban	Yes	Yes	No	Yes	Yes	No	No	Yes	No	Yes
6987	HSNZ0495	Male	Malay	2021	32	Single	Secondary	Unemployed	Urban	Yes	No	No	No	Yes	No	No	No	No	No
6988	HSNZ0496	Male	Malay	2021	30	Single	No formal education	Unemployed	Rural	No	No	No	No	Yes	No	No	No	No	No
6989	HSNZ0497	Male	Malay	2021	38	Single	Primary	Unemployed	Rural	No	Yes	No	No	Yes	No	No	No	No	No
6990	HSNZ0498	Male	Malay	2021	15	Single	Secondary	Private	Rural	Yes	Yes	No	Yes	No	No	No	Yes	No	Yes
6991	HSNZ0499	Male	Malay	2021	26	Single	No formal education	Private	Rural	Yes	Yes	No	Yes	No	No	No	Yes	No	Yes
6992	HSNZ0503	Male	Malay	2021	22	Others	Secondary	Private	Rural	Yes	Yes	No	No	No	No	No	Yes	No	No
6993	HSNZ0505	Male	Malay	2021	35	Married	Tertiary	Unemployed	Urban	Yes	Yes	Yes	No	No	No	No	No	No	Yes
6994	HSNZ0506	Male	Malay	2021	56	Married	No formal education	Self-employed	Urban	Yes	Yes	No	No	No	No	Yes	No	No	No
6995	HSNZ0507	Male	Malay	2021	35	Single	Secondary	Unemployed	Rural	Yes	No	No	No	Yes	No	No	No	Yes	Yes

No	Patient ID	Gender	Ethnic group	Year of diagnosis	Age	Marital status	Education level	Occupation	Place of residence	History of psy illness	Tobacco	Alcohol	Cannabis	ATS	Inhalant	Sedative	Hallucinogens	Opioids	Kratom
6996	HSNZ0508	Male	Malay	2021	28	Married	Secondary	Private	Urban	No	Yes	No	No	Yes	No	No	No	No	No
6997	HSNZ0509	Male	Malay	2021	45	Single	No formal education	Others	Rural	Yes	Yes	No	No	No	No	No	No	No	No
6998	HSNZ0510	Male	Malay	2021	42	Others	No formal education	Self-employed	Rural	Yes	No	No	No	Yes	No	No	No	No	No
6999	HSNZ0511	Male	Malay	2021	49	Married	No formal education	Private	Urban	Yes	Yes	No	No	No	No	No	No	No	No
7000	HSNZ0512	Male	Malay	2021	51	Single	No formal education	Unemployed	Urban	Yes	No	No	Yes	Yes	No	No	No	No	No
7001	HSNZ0514	Male	Malay	2021	24	Single	Secondary	Others	Urban	Yes	No	No	No	Yes	No	No	No	No	No
7002	HSNZ0515	Male	Malay	2021	43	Married	No formal education	Private	Urban	No	Yes	No	No	Yes	No	No	No	No	No
7003	HSNZ0516	Male	Malay	2021	27	Others	Secondary	Private	Rural	No	No	No	No	Yes	No	No	No	No	No
7004	HSNZ0517	Male	Malay	2021	38	Single	Secondary	Unemployed	Rural	No	No	No	No	Yes	No	No	No	No	Yes
7005	HSNZ0519	Male	Malay	2021	37	Single	Tertiary	Private	Urban	Yes	No	No	Yes	Yes	No	No	No	No	No
7006	HSNZ0522	Male	Malay	2021	27	Married	No formal education	Others	Rural	No	No	No	No	Yes	No	No	No	No	No
7007	HSNZ0523	Male	Malay	2021	28	Married	Secondary	Self-employed	Rural	No	No	No	No	Yes	No	No	No	No	No
7008	HSNZ0526	Male	Malay	2021	25	Single	Secondary	Unemployed	Rural	Yes	No	No	Yes	Yes	No	No	No	Yes	No
7009	HSNZ0527	Male	Malay	2021	40	Single	No formal education	Unemployed	Rural	Yes	No	No	No	Yes	No	No	No	No	No
7010	HSNZ0528	Male	Malay	2021	17	Single	Secondary	Self-employed	Rural	No	Yes	No	Yes	Yes	No	No	No	No	Yes
7011	HSNZ0529	Male	Malay	2021	41	Others	Tertiary	Self-employed	Rural	Yes	No	No	Yes	Yes	No	No	No	No	No
7012	HSNZ0530	Male	Malay	2021	27	Single	Secondary	Unemployed	Rural	Yes	Yes	Yes	No	No	No	No	No	No	No
7013	HSNZ0532	Male	Malay	2021	33	Others	Secondary	Private	Urban	Yes	Yes	No	No	Yes	No	No	No	No	No
7014	HSNZ0533	Male	Malay	2021	53	Married	Tertiary	Unemployed	Urban	Yes	Yes	No	No	No	No	No	No	No	No
7015	HSNZ0534	Male	Malay	2021	39	Single	No formal education	Unemployed	Urban	Yes	Yes	No	No	No	No	No	No	No	No
7016	HSNZ0535	Male	Malay	2020	30	Single	Secondary	Self-employed	Urban	No	No	No	No	Yes	No	No	No	Yes	No

No	Patient ID	Gender	Ethnic group	Year of diagnosis	Age	Marital status	Education level	Occupation	Place of residence	History of psy illness	Tobacco	Alcohol	Cannabis	ATS	Inhalant	Sedative	Hallucinogens	Opioids	Kratom
7017	HSNZ0537	Male	Malay	2021	32	Single	No formal education	Self-employed	Rural	Yes	Yes	No	No	No	No	No	No	No	No
7018	HSNZ0539	Male	Malay	2020	33	Single	Secondary	Unemployed	Rural	Yes	No	No	No	Yes	No	No	No	No	No
7019	HSNZ0540	Male	Malay	2020	25	Single	Secondary	Self-employed	Rural	Yes	No	No	No	No	No	No	Yes	No	Yes
7020	HSNZ0542	Male	Malay	2020	37	Others	No formal education	Private	Rural	No	No	No	No	Yes	No	No	No	Yes	No
7021	HSNZ0544	Male	Malay	2020	41	Single	Primary	Unemployed	Rural	Yes	Yes	No	No	No	No	No	No	No	No
7022	HSNZ0547	Male	Malay	2020	42	Single	Tertiary	Unemployed	Rural	Yes	No	No	No	Yes	No	No	No	No	Yes
7023	HSNZ0550	Male	Malay	2020	37	Single	No formal education	Unemployed	Rural	Yes	No	No	No	Yes	No	No	No	No	Yes
7024	HSNZ0552	Male	Malay	2019	33	Married	No formal education	Private	Urban	Yes	No	No	Yes	Yes	Yes	No	No	Yes	No
7025	HSNZ0553	Male	Malay	2021	22	Single	Secondary	Self-employed	Rural	Yes	Yes	Yes	Yes	Yes	No	No	No	No	No
7026	HSNZ0554	Male	Malay	2021	34	Others	Tertiary	Self-employed	Urban	No	No	Yes	No	Yes	No	Yes	No	Yes	No
7027	HSNZ0555	Male	Malay	2021	30	Others	No formal education	Unemployed	Rural	No	No	No	No	Yes	No	No	No	No	No
7028	HSNZ0562	Male	Malay	2020	54	Married	No formal education	Self-employed	Urban	Yes	Yes	No	No	No	No	No	No	No	No
7029	HSNZ0567	Male	Malay	2021	26	Single	Secondary	Others	Urban	Yes	No	No	No	Yes	No	No	No	No	No
7030	HSNZ0568	Male	Malay	2021	42	Married	Secondary	Self-employed	Urban	Yes	Yes	No	No	No	No	No	No	No	No
7031	HSNZ0569	Male	Malay	2021	33	Married	Tertiary	Government	Urban	Yes	Yes	No	No	Yes	No	No	No	No	No
7032	HSNZ0570	Male	Malay	2020	36	Married	Tertiary	Self-employed	Urban	Yes	Yes	No	No	No	No	No	No	No	No
7033	HSNZ0571	Male	Malay	2020	44	Single	Tertiary	Others	Rural	Yes	Yes	No	Yes	Yes	No	Yes	No	Yes	No
7034	HSNZ0574	Male	Malay	2019	44	Married	No formal education	Unemployed	Urban	Yes	Yes	No	No	No	No	No	No	No	No
7035	HSNZ0575	Male	Malay	2021	39	Single	Secondary	Self-employed	Urban	Yes	Yes	Yes	No	No	No	No	No	Yes	Yes
7036	HSNZ0577	Male	Malay	2021	23	Single	Tertiary	Self-employed	Urban	Yes	Yes	No	No	No	No	No	Yes	No	No
7037	HSNZ0578	Male	Malay	2021	27	Single	Secondary	Private	Urban	Yes	Yes	No	No	Yes	No	No	No	No	No

No	Patient ID	Gender	Ethnic group	Year of diagnosis	Age	Marital status	Education level	Occupation	Place of residence	History of psy illness	Tobacco	Alcohol	Cannabis	ATS	Inhalant	Sedative	Hallucinogens	Opioids	Kratom
7038	HSNZ0580	Male	Malay	2020	29	Others	No formal education	Unemployed	Urban	Yes	Yes	No	No	Yes	No	No	No	No	No
7039	HSNZ0583	Male	Malay	2019	25	Single	No formal education	Unemployed	Urban	No	Yes	No	No	Yes	Yes	No	No	No	No
7040	HTAA0001	Male	Malay	2020	24	Single	Tertiary	Unemployed	Urban	Yes	Yes	No	Yes	No	No	No	No	No	No
7041	HTAA0004	Male	Malay	2018	26	Single	Secondary	Unemployed	Rural	Yes	No	No	No	Yes	No	No	No	No	No
7042	HTAA0005	Male	Chinese	2018	38	Others	Tertiary	Others	Urban	No	No	No	No	Yes	No	Yes	No	No	No
7043	HTAA0007	Male	Malay	2019	17	Single	Secondary	Unemployed	Urban	Yes	Yes	No	Yes	Yes	Yes	Yes	No	No	No
7044	HTAA0009	Male	Others	2020	23	Single	Tertiary	Others	Rural	Yes	Yes	Yes	Yes	Yes	No	No	No	Yes	Yes
7045	HTAA0010	Male	Malay	2019	39	Married	Secondary	Self-employed	Urban	Yes	Yes	No	No	Yes	No	No	No	No	No
7046	HTAA0011	Female	Malay	2019	22	Single	Secondary	Self-employed	Urban	No	Yes	Yes	Yes	Yes	No	No	No	No	Yes
7047	HTAA0012	Male	Malay	2021	31	Single	No formal education	Self-employed	Urban	Yes	No	No	No	Yes	No	No	No	No	No
7048	HTAA0013	Male	Chinese	2019	49	Single	Primary	Unemployed	Urban	No	Yes	Yes	Yes	Yes	Yes	Yes	Yes	Yes	Yes
7049	HTAA0015	Male	Malay	2020	23	Single	Secondary	Government	Urban	No	Yes	No	No	Yes	No	No	No	No	No
7050	HTAA0016	Male	Malay	2020	38	Married	Tertiary	Unemployed	Urban	No	Yes	Yes	Yes	Yes	Yes	Yes	Yes	Yes	Yes
7051	HTAA0017	Male	Malay	2021	28	Single	Primary	Unemployed	Urban	No	Yes	No	Yes	Yes	Yes	Yes	No	No	No
7052	HTAA0018	Male	Malay	2021	26	Married	Secondary	Others	Urban	Yes	Yes	Yes	Yes	Yes	Yes	Yes	Yes	Yes	Yes
7053	HTAA0020	Female	Malay	2018	30	Married	Secondary	Self-employed	Urban	Yes	No	No	No	Yes	No	No	No	No	No
7054	HTAA0021	Male	Malay	2018	18	Single	Primary	Private	Urban	Yes	No	No	No	Yes	No	No	No	No	No
7055	HTAA0022	Male	Malay	2019	31	Others	Secondary	Private	Urban	Yes	No	Yes	No	No	No	No	No	No	No
7056	HTAA0024	Male	Malay	2019	36	Single	Secondary	Unemployed	Rural	Yes	Yes	Yes	Yes	Yes	No	No	Yes	Yes	Yes
7057	HTAA0025	Female	Malay	2020	36	Others	Secondary	Unemployed	Urban	Yes	No	No	No	Yes	No	No	No	Yes	No
7058	HTAA0026	Male	Malay	2020	28	Single	Primary	Unemployed	Urban	Yes	Yes	Yes	Yes	No	No	No	No	No	No

No	Patient ID	Gender	Ethnic group	Year of diagnosis	Age	Marital status	Education level	Occupation	Place of residence	History of psy illness	Tobacco	Alcohol	Cannabis	ATS	Inhalant	Sedative	Hallucinogens	Opioids	Kratom
7059	HTAA0027	Male	Malay	2018	34	Single	Secondary	Private	Urban	Yes	No	No	Yes	Yes	No	No	No	Yes	No
7060	HTAA0029	Male	Malay	2019	17	Single	Secondary	Unemployed	Urban	Yes	Yes	No	Yes	Yes	No	No	No	No	No
7061	HTAA0030	Male	Malay	2020	27	Single	Secondary	Unemployed	Rural	Yes	No	No	No	Yes	No	No	No	No	No
7062	HTAA0032	Female	Malay	2019	34	Others	Primary	Private	Urban	No	Yes	Yes	No	Yes	No	Yes	No	No	No
7063	HTAA0034	Male	Malay	2021	45	Others	Secondary	Self-employed	Urban	Yes	No	Yes	No	Yes	No	No	No	Yes	Yes
7064	HTAA0036	Male	Chinese	2018	34	Married	Secondary	Self-employed	Urban	No	No	No	Yes	No	No	No	No	No	No
7065	HTAA0038	Male	Malay	2019	26	Single	Secondary	Private	Urban	Yes	Yes	No	Yes	Yes	No	No	No	Yes	Yes
7066	HTAA0039	Male	Malay	2019	23	Single	Primary	Self-employed	Urban	Yes	No	Yes	Yes	Yes	No	No	No	No	No
7067	HTAA0041	Male	Malay	2019	39	Single	Primary	Unemployed	Rural	Yes	Yes	No	Yes	Yes	No	No	No	Yes	No
7068	HTAA0043	Male	Indian	2019	24	Married	Secondary	Self-employed	Rural	Yes	Yes	Yes	No	Yes	No	No	No	No	Yes
7069	HTAA0044	Male	Malay	2018	31	Single	Primary	Unemployed	Rural	Yes	No	No	No	Yes	No	No	No	No	No
7070	HTAA0046	Male	Malay	2019	35	Single	Secondary	Unemployed	Urban	Yes	No	No	Yes	Yes	No	No	No	No	No
7071	HTAA0047	Male	Malay	2020	26	Single	Secondary	Self-employed	Urban	Yes	No	Yes	No	Yes	No	No	No	No	No
7072	HTAA0049	Female	Others	2018	47	Married	Primary	Private	Urban	Yes	Yes	Yes	No	No	No	Yes	No	No	No
7073	HTAA0050	Male	Malay	2018	25	Single	Tertiary	Others	Urban	Yes	No	No	Yes	No	No	No	No	No	No
7074	HTAA0053	Male	Chinese	2018	34	Single	Primary	Self-employed	Urban	Yes	Yes	No	Yes	Yes	No	No	No	No	No
7075	HTAA0054	Male	Malay	2020	37	Single	Secondary	Unemployed	Urban	Yes	No	No	No	Yes	No	No	No	Yes	No
7076	HTAA0058	Male	Malay	2019	25	Single	Secondary	Unemployed	Urban	Yes	Yes	No	No	Yes	No	No	No	No	No
7077	HTAA0059	Male	Malay	2020	22	Single	Tertiary	Unemployed	Urban	No	Yes	No	Yes	Yes	No	No	No	No	No
7078	HTAA0060	Male	Malay	2020	39	Others	No formal education	Unemployed	Rural	Yes	Yes	No	Yes	Yes	No	No	No	Yes	No
7079	HTAA0061	Male	Malay	2018	29	Married	Secondary	Government	Urban	Yes	No	No	No	No	No	No	No	No	Yes

No	Patient ID	Gender	Ethnic group	Year of diagnosis	Age	Marital status	Education level	Occupation	Place of residence	History of psy illness	Tobacco	Alcohol	Cannabis	ATS	Inhalant	Sedative	Hallucinogens	Opioids	Kratom
7080	HTAA0062	Male	Malay	2020	20	Single	Primary	Self-employed	Rural	Yes	No	No	No	Yes	No	No	No	No	Yes
7081	HTAA0063	Male	Malay	2021	26	Single	Tertiary	Unemployed	Rural	No	Yes	No	Yes	Yes	No	No	No	No	No
7082	HTAA0064	Male	Malay	2020	46	Single	Tertiary	Private	Urban	Yes	Yes	No	Yes	Yes	No	No	No	No	No
7083	HTAA0066	Male	Malay	2021	34	Single	Secondary	Unemployed	Urban	Yes	Yes	No	Yes	Yes	No	No	No	No	No
7084	HTAA0068	Male	Malay	2018	26	Single	Tertiary	Private	Rural	No	Yes	No	Yes	Yes	No	No	No	No	No
7085	HTAA0070	Male	Malay	2018	28	Single	No formal education	Unemployed	Urban	Yes	Yes	No	Yes	Yes	No	No	No	Yes	No
7086	HTAA0072	Male	Malay	2018	28	Single	Secondary	Unemployed	Rural	Yes	Yes	No	Yes	Yes	No	Yes	No	No	No
7087	HTAA0073	Male	Chinese	2020	56	Single	Primary	Unemployed	Urban	Yes	Yes	Yes	No	Yes	No	No	No	Yes	No
7088	HTAA0074	Male	Malay	2019	35	Single	Secondary	Unemployed	Urban	Yes	Yes	No	No	Yes	No	No	No	Yes	No
7089	HTAA0075	Male	Malay	2021	21	Single	Primary	Unemployed	Urban	Yes	No	No	No	Yes	No	No	No	Yes	No
7090	HTAA0076	Male	Malay	2019	42	Single	Secondary	Unemployed	Rural	Yes	Yes	No	Yes	Yes	No	No	No	Yes	No
7091	HTAA0077	Male	Malay	2019	31	Single	Primary	Self-employed	Rural	Yes	No	No	No	Yes	No	No	No	No	No
7092	HTAA0078	Male	Malay	2020	35	Single	Secondary	Private	Urban	Yes	Yes	No	Yes	Yes	No	No	No	Yes	No
7093	HTAA0079	Male	Malay	2019	38	Others	Primary	Unemployed	Rural	Yes	Yes	Yes	Yes	Yes	No	No	No	No	No
7094	HTAA0081	Male	Others	2019	23	Single	Secondary	Unemployed	Urban	Yes	Yes	No	Yes	No	No	No	No	No	No
7095	HTAA0082	Female	Malay	2020	42	Married	Secondary	Unemployed	Urban	Yes	Yes	No	No	Yes	No	No	No	No	No
7096	HTAA0083	Male	Malay	2020	32	Others	Secondary	Unemployed	Urban	Yes	Yes	No	Yes	Yes	No	No	No	No	No
7097	HTAA0084	Male	Malay	2020	41	Others	Secondary	Unemployed	Rural	Yes	No	No	No	Yes	No	No	No	No	No
7098	HTAA0085	Male	Malay	2019	25	Married	Tertiary	Private	Urban	No	Yes	No	No	Yes	No	No	No	Yes	No
7100	HTAA0086	Female	Malay	2019	29	Single	Primary	Private	Urban	Yes	Yes	No	No	Yes	No	No	No	No	No
7099	HTAA0086	Male	Chinese	2021	42	Married	Primary	Self-employed	Urban	Yes	Yes	Yes	No	No	No	No	No	No	No

No	Patient ID	Gender	Ethnic group	Year of diagnosis	Age	Marital status	Education level	Occupation	Place of residence	History of psy illness	Tobacco	Alcohol	Cannabis	ATS	Inhalant	Sedative	Hallucinogens	Opioids	Kratom
7101	HTAA0088	Male	Malay	2019	18	Single	Secondary	Unemployed	Urban	Yes	Yes	No	Yes	Yes	No	Yes	No	No	No
7102	HTAA0090	Male	Malay	2020	49	Married	Primary	Self-employed	Urban	Yes	No	No	No	Yes	No	No	No	No	No
7103	HTAA0091	Male	Malay	2019	32	Single	Secondary	Unemployed	Rural	Yes	Yes	No	Yes	Yes	No	No	No	Yes	Yes
7104	HTAA0092	Male	Malay	2021	28	Single	Secondary	Private	Urban	Yes	Yes	No	No	No	No	No	No	No	Yes
7105	HTAA0093	Male	Malay	2020	47	Single	Primary	Unemployed	Rural	Yes	Yes	Yes	No	No	No	No	No	Yes	No
7106	HTAA0095	Male	Malay	2019	25	Single	Tertiary	Unemployed	Urban	Yes	Yes	No	No	Yes	No	No	No	No	No
7107	HTAA0096	Male	Malay	2018	27	Single	Secondary	Unemployed	Rural	Yes	Yes	No	No	Yes	No	No	No	Yes	No
7108	HTAA0098	Male	Malay	2019	29	Single	Secondary	Self-employed	Rural	Yes	Yes	No	No	Yes	No	No	No	No	No
7109	HTAA0099	Male	Malay	2021	24	Single	Tertiary	Unemployed	Urban	Yes	Yes	No	Yes	Yes	No	Yes	No	No	No
7110	HTAA0100	Male	Malay	2020	24	Married	Tertiary	Unemployed	Urban	Yes	Yes	No	Yes	No	No	No	No	No	No
7111	HTAA0101	Female	Malay	2021	35	Others	Primary	Self-employed	Rural	Yes	Yes	No	No	Yes	No	No	No	No	No
7112	HTAA0102	Male	Malay	2020	41	Single	No formal education	Unemployed	Urban	Yes	Yes	No	No	Yes	Yes	No	No	No	Yes
7113	HTAA0103	Female	Chinese	2019	41	Married	Primary	Unemployed	Urban	Yes	Yes	No	No	Yes	No	No	No	No	No
7114	HTAA0104	Male	Malay	2018	33	Single	Primary	Unemployed	Rural	Yes	Yes	No	No	Yes	No	No	No	Yes	No
7115	HTAA0105	Female	Malay	2019	28	Single	Secondary	Unemployed	Urban	Yes	Yes	Yes	Yes	Yes	No	No	No	Yes	No
7116	HTAA0106	Male	Malay	2018	41	Single	Primary	Self-employed	Urban	Yes	Yes	No	No	Yes	No	No	No	No	No
7117	HTAA0107	Male	Malay	2020	42	Single	Secondary	Unemployed	Urban	No	Yes	No	Yes	Yes	No	No	No	Yes	Yes
7118	HTAA0108	Female	Malay	2020	21	Single	Secondary	Unemployed	Urban	Yes	Yes	No	No	Yes	No	No	No	Yes	No
7119	HTAA0109	Male	Malay	2021	29	Others	Secondary	Self-employed	Urban	Yes	Yes	No	Yes	Yes	No	No	No	Yes	Yes
7120	HTAA0110	Male	Malay	2021	32	Single	Secondary	Unemployed	Urban	Yes	Yes	No	Yes	Yes	No	No	No	No	No

No	Patient ID	Gender	Ethnic group	Year of diagnosis	Age	Marital status	Education level	Occupation	Place of residence	History of psy illness	Tobacco	Alcohol	Cannabis	ATS	Inhalant	Sedative	Hallucinogens	Opioids	Kratom
7121	HTAA0111	Female	Malay	2020	31	Single	Secondary	Self-employed	Urban	No	Yes	No	No	Yes	No	No	No	No	No
7122	HTAA0112	Male	Malay	2020	42	Married	No formal education	Unemployed	Urban	Yes	No	No	No	Yes	No	No	No	No	No
7123	HTAA0116	Male	Chinese	2020	43	Single	Secondary	Unemployed	Urban	Yes	No	Yes	No	Yes	No	No	No	No	No
7124	HTAA0118	Male	Malay	2020	29	Single	Tertiary	Unemployed	Urban	Yes	Yes	No	Yes	Yes	No	No	No	No	No
7125	HTAA0119	Male	Malay	2019	21	Single	Secondary	Unemployed	Urban	Yes	Yes	Yes	Yes	No	No	No	No	No	Yes
7126	HTAA0120	Male	Malay	2020	26	Single	Secondary	Unemployed	Urban	Yes	Yes	No	Yes	Yes	No	No	No	No	No
7127	HTAA0121	Male	Malay	2020	37	Others	Primary	Self-employed	Rural	Yes	Yes	No	No	Yes	No	No	No	No	No
7128	HTAA0122	Male	Malay	2019	36	Married	Primary	Private	Rural	Yes	Yes	No	Yes	Yes	No	No	No	Yes	Yes
7129	HTAA0123	Male	Malay	2021	37	Single	No formal education	Unemployed	Rural	Yes	Yes	No	No	Yes	No	No	No	Yes	No
7130	HTAA0124	Male	Malay	2021	42	Single	Primary	Unemployed	Urban	Yes	Yes	No	Yes	Yes	No	No	No	Yes	No
7131	HTAA0126	Male	Chinese	2020	47	Single	Primary	Unemployed	Urban	Yes	Yes	Yes	No	Yes	No	No	No	No	No
7132	HTAA0129	Male	Malay	2018	26	Married	Primary	Private	Urban	Yes	Yes	No	Yes	Yes	No	No	No	No	No
7133	HTAA0131	Male	Malay	2019	20	Single	Secondary	Unemployed	Urban	Yes	No	No	No	Yes	No	No	No	No	No
7134	HTAA0134	Male	Chinese	2021	72	Married	No formal education	Unemployed	Urban	Yes	Yes	Yes	No	No	No	No	No	No	No
7135	HTAA0138	Male	Malay	2019	24	Single	Tertiary	Government	Urban	Yes	Yes	No	No	Yes	No	No	No	No	No
7136	HTAA0142	Male	Malay	2021	35	Single	Primary	Self-employed	Rural	Yes	Yes	No	No	Yes	No	No	No	No	No
7137	HTAA0148	Male	Malay	2020	34	Single	Secondary	Unemployed	Rural	Yes	Yes	No	Yes	Yes	No	No	No	No	No
7138	HTAA0153	Male	Malay	2019	22	Married	Secondary	Unemployed	Urban	No	Yes	No	No	No	No	No	No	No	Yes
7139	HTAA0157	Male	Malay	2018	29	Married	Tertiary	Government	Urban	No	Yes	No	No	No	No	No	No	No	No
7140	HTAA0162	Male	Malay	2021	46	Others	No formal education	Unemployed	Rural	Yes	Yes	No	No	No	No	No	No	No	No

No	Patient ID	Gender	Ethnic group	Year of diagnosis	Age	Marital status	Education level	Occupation	Place of residence	History of psy illness	Tobacco	Alcohol	Cannabis	ATS	Inhalant	Sedative	Hallucinogens	Opioids	Kratom
7141	HTAA0163	Male	Malay	2018	41	Single	Secondary	Unemployed	Rural	Yes	Yes	No	No	No	No	No	No	No	No
7142	HTAA0164	Male	Malay	2020	43	Married	Secondary	Government	Urban	Yes	Yes	No	No	No	No	No	No	No	No
7143	HTAA0167	Male	Malay	2020	33	Single	Secondary	Unemployed	Urban	Yes	Yes	No	No	Yes	No	No	No	Yes	No
7144	HTAA0168	Male	Malay	2020	18	Single	Primary	Private	Urban	Yes	Yes	No	No	Yes	No	No	No	No	No
7145	HTAA0170	Male	Malay	2019	26	Single	No formal education	Unemployed	Urban	Yes	Yes	No	No	Yes	No	No	No	Yes	No
7146	HTAA0173	Male	Malay	2020	34	Married	Tertiary	Government	Urban	Yes	Yes	No	No	No	No	No	No	No	No
7147	HTAA0177	Male	Malay	2019	30	Single	Primary	Unemployed	Rural	Yes	Yes	Yes	No	Yes	No	No	No	No	No
7148	HTAA0180	Male	Malay	2021	33	Single	Tertiary	Private	Urban	Yes	Yes	No	No	Yes	No	No	No	No	No
7149	HTAA0182	Male	Malay	2018	21	Single	Secondary	Private	Urban	Yes	Yes	No	No	No	No	No	No	No	No
7150	HTAA0187	Female	Malay	2018	34	Others	Primary	Unemployed	Rural	Yes	No	No	No	Yes	No	No	No	No	No
7151	HTAA0188	Male	Malay	2020	39	Single	Primary	Unemployed	Rural	Yes	No	No	No	No	No	No	No	Yes	No
7152	HTAA0190	Male	Malay	2020	26	Single	Tertiary	Unemployed	Urban	Yes	Yes	No	Yes	No	No	No	No	No	No
7153	HTAA0192	Male	Malay	2018	28	Single	Secondary	Private	Urban	Yes	Yes	No	Yes	Yes	No	No	No	No	No
7154	HTAA0198	Female	Malay	2018	17	Single	Secondary	Private	Urban	Yes	No	No	No	No	No	Yes	No	No	No
7155	HTAA0200	Male	Malay	2018	34	Single	Secondary	Government	Urban	Yes	Yes	No	No	Yes	No	No	No	No	No
7156	HTAA0201	Male	Malay	2021	33	Others	Primary	Unemployed	Urban	Yes	Yes	No	No	Yes	No	No	No	No	No
7157	HTAA0202	Male	Chinese	2020	47	Others	No formal education	Unemployed	Urban	Yes	Yes	Yes	No	Yes	No	No	No	No	No
7158	HTAA0210	Male	Malay	2018	25	Single	Tertiary	Unemployed	Urban	Yes	Yes	No	No	No	No	No	No	No	No
7159	HTAA0211	Male	Malay	2021	32	Single	Secondary	Self-employed	Urban	Yes	Yes	Yes	Yes	Yes	No	No	No	No	No
7160	HTAA0212	Male	Malay	2019	39	Married	Secondary	Self-employed	Urban	Yes	Yes	No	No	No	No	No	No	No	No
7161	HTAA0213	Female	Indian	2020	28	Single	Tertiary	Unemployed	Urban	No	Yes	Yes	No	No	No	No	No	No	No

No	Patient ID	Gender	Ethnic group	Year of diagnosis	Age	Marital status	Education level	Occupation	Place of residence	History of psy illness	Tobacco	Alcohol	Cannabis	ATS	Inhalant	Sedative	Hallucinogens	Opioids	Kratom
7162	HTAA0217	Female	Malay	2021	22	Single	Secondary	Unemployed	Urban	Yes	Yes	No	No	No	No	No	No	No	No
7163	HTAA0219	Female	Indian	2021	41	Married	Tertiary	Unemployed	Urban	Yes	Yes	No	No	No	No	No	No	No	No
7164	HTAA0222	Male	Chinese	2020	57	Others	Primary	Self-employed	Urban	Yes	Yes	No	No	Yes	No	No	No	No	No
7165	HTAA0223	Male	Chinese	2020	31	Single	Secondary	Private	Rural	Yes	Yes	Yes	No	No	No	No	No	No	No
7166	HTAA0227	Male	Malay	2018	40	Others	No formal education	Unemployed	Urban	Yes	No	No	No	Yes	Yes	No	No	No	No
7167	HTAA0228	Male	Others	2021	27	Single	Primary	Unemployed	Rural	Yes	No	No	Yes	Yes	No	No	No	No	No
7168	HTAA0229	Male	Chinese	2018	40	Single	Secondary	Private	Urban	Yes	Yes	Yes	Yes	Yes	No	No	No	No	No
7169	HTAA0233	Male	Malay	2018	23	Single	Primary	Private	Rural	Yes	Yes	No	No	Yes	No	No	No	No	No
7170	HTAA0234	Male	Malay	2019	21	Single	Secondary	Private	Urban	Yes	Yes	No	No	Yes	No	No	No	No	No
7171	HTAA0240	Male	Malay	2021	54	Single	Secondary	Unemployed	Urban	Yes	No	No	No	Yes	No	No	No	No	No
7172	HTAA0244	Male	Malay	2020	30	Married	Primary	Self-employed	Urban	Yes	No	No	No	Yes	No	No	No	No	No
7173	HTAA0246	Male	Malay	2018	23	Single	Secondary	Private	Urban	Yes	Yes	No	Yes	Yes	Yes	No	No	No	Yes
7174	HTAA0249	Male	Chinese	2019	44	Others	Primary	Self-employed	Urban	Yes	Yes	Yes	No	Yes	No	Yes	No	No	No
7175	HTAA0252	Male	Malay	2020	46	Single	Secondary	Private	Rural	Yes	Yes	No	No	No	No	No	No	No	No
7176	HTAA0253	Male	Malay	2018	28	Single	Secondary	Unemployed	Urban	Yes	Yes	No	Yes	Yes	No	No	No	No	No
7177	HTAA0254	Male	Malay	2021	44	Others	Primary	Private	Urban	Yes	Yes	No	No	Yes	No	No	No	Yes	No
7178	HTAA0255	Male	Chinese	2020	44	Single	Secondary	Unemployed	Urban	Yes	No	Yes	No	No	No	No	No	No	No
7179	HTAA0267	Male	Malay	2018	22	Single	Secondary	Unemployed	Urban	Yes	Yes	No	Yes	Yes	No	No	No	No	No
7180	HTAA0268	Male	Malay	2018	34	Others	Secondary	Private	Urban	Yes	Yes	No	No	Yes	No	No	No	No	No
7181	HTAA0271	Male	Malay	2018	38	Married	Secondary	Others	Rural	Yes	Yes	Yes	Yes	Yes	No	No	No	No	No
7182	HTAA0273	Male	Malay	2021	22	Single	Secondary	Unemployed	Urban	Yes	No	No	No	Yes	No	No	No	No	No

No	Patient ID	Gender	Ethnic group	Year of diagnosis	Age	Marital status	Education level	Occupation	Place of residence	History of psy illness	Tobacco	Alcohol	Cannabis	ATS	Inhalant	Sedative	Hallucinogens	Opioids	Kratom
7183	HTAA0276	Male	Malay	2019	31	Married	Tertiary	Private	Rural	Yes	Yes	No	Yes	Yes	No	No	No	No	No
7184	HTAA0281	Male	Malay	2019	29	Married	Tertiary	Private	Urban	Yes	Yes	No	Yes	No	No	No	No	No	No
7185	HTAA0283	Male	Malay	2019	39	Others	Secondary	Unemployed	Urban	Yes	Yes	No	No	No	No	No	No	No	No
7186	HTAA0285	Male	Malay	2020	32	Others	Secondary	Unemployed	Urban	Yes	Yes	No	No	Yes	No	No	No	No	No
7187	HTAA0289	Male	Chinese	2018	22	Single	Secondary	Unemployed	Rural	Yes	Yes	No	No	No	No	No	No	No	No
7188	HTAA0290	Male	Chinese	2020	32	Single	Secondary	Unemployed	Urban	Yes	Yes	Yes	Yes	Yes	No	No	Yes	No	No
7189	HTAA0294	Male	Malay	2020	40	Others	Secondary	Unemployed	Rural	Yes	Yes	No	Yes	Yes	No	No	No	No	No
7190	HTAA0298	Male	Malay	2018	19	Single	Tertiary	Unemployed	Urban	Yes	Yes	No	Yes	No	No	No	No	No	No
7191	HTAA0299	Male	Malay	2018	26	Single	Secondary	Unemployed	Rural	Yes	Yes	No	Yes	No	Yes	No	No	No	Yes
7192	HTAA0302	Male	Malay	2019	32	Single	Secondary	Unemployed	Rural	Yes	Yes	No	Yes	Yes	No	No	No	Yes	Yes
7193	HTAA0304	Male	Malay	2018	32	Others	Primary	Self-employed	Rural	Yes	Yes	No	No	Yes	No	No	No	No	No
7194	HTAA0310	Male	Malay	2021	24	Single	Secondary	Unemployed	Urban	Yes	Yes	No	Yes	Yes	No	Yes	No	No	Yes
7195	HTAA0312	Male	Malay	2021	28	Married	Secondary	Private	Rural	Yes	Yes	Yes	No	Yes	No	No	No	No	No
7196	HTAA0316	Male	Malay	2019	28	Single	Secondary	Self-employed	Urban	Yes	Yes	Yes	No	Yes	No	No	No	No	No
7197	HTAA0317	Male	Malay	2021	34	Others	Secondary	Unemployed	Rural	Yes	Yes	No	No	Yes	No	No	No	No	No
7198	HTAA0320	Male	Malay	2019	39	Married	Primary	Government	Urban	Yes	Yes	No	No	Yes	No	No	No	No	No
7199	HTAA0325	Male	Malay	2019	33	Single	Secondary	Unemployed	Urban	Yes	Yes	No	No	Yes	No	No	No	No	No
7200	HTAA0330	Male	Malay	2021	28	Married	No formal education	Unemployed	Urban	Yes	Yes	No	No	Yes	No	Yes	No	No	No

No	Patient ID	Gender	Ethnic group	Year of diagnosis	Age	Marital status	Education level	Occupation	Place of residence	History of psy illness	Tobacco	Alcohol	Cannabis	ATS	Inhalant	Sedative	Hallucinogens	Opioids	Kratom
7201	HTAA0334	Male	Malay	2018	21	Single	Tertiary	Private	Urban	Yes	Yes	No	Yes	Yes	No	No	No	No	No
7202	HTAA0335	Male	Malay	2020	42	Single	Primary	Unemployed	Rural	Yes	Yes	No	No	Yes	No	No	No	No	No
7203	HTAA0338	Male	Malay	2019	50	Single	Tertiary	Unemployed	Rural	Yes	Yes	No	No	Yes	No	No	No	Yes	No
7204	HTAA0339	Male	Malay	2020	28	Single	Secondary	Unemployed	Urban	Yes	Yes	No	No	Yes	No	No	No	No	No
7205	HTAA0340	Male	Malay	2020	33	Single	Secondary	Self-employed	Urban	Yes	Yes	No	No	Yes	No	No	No	No	No
7206	HTAA0341	Male	Malay	2021	56	Married	Secondary	Private	Urban	Yes	Yes	No	No	No	No	No	No	No	No
7207	HTAA0344	Male	Malay	2021	38	Single	Secondary	Unemployed	Urban	Yes	Yes	No	No	Yes	No	No	No	No	No
7208	HTAA0345	Male	Malay	2021	36	Single	Secondary	Unemployed	Rural	Yes	Yes	No	No	Yes	No	No	No	No	No
7209	HTAA0353	Male	Malay	2020	35	Single	Secondary	Unemployed	Rural	Yes	Yes	No	Yes	Yes	No	No	No	No	Yes
7210	HTAA0355	Male	Malay	2018	40	Married	Primary	Private	Urban	Yes	Yes	No	No	Yes	No	No	No	No	No
7211	HTAA0356	Male	Malay	2019	43	Single	Secondary	Unemployed	Rural	No	Yes	No	No	No	No	No	No	No	Yes
7212	HTAA0358	Male	Malay	2020	47	Married	No formal education	Self-employed	Urban	Yes	Yes	No	No	No	No	No	No	No	No
7213	HTAA0360	Male	Malay	2021	35	Others	Secondary	Unemployed	Urban	Yes	Yes	No	Yes	Yes	No	No	No	Yes	No
7214	HTAA0361	Male	Malay	2019	44	Single	Secondary	Unemployed	Urban	Yes	Yes	No	Yes	Yes	No	No	No	Yes	No
7215	HTAA0365	Male	Malay	2018	36	Single	Primary	Unemployed	Urban	Yes	Yes	No	No	No	No	No	No	No	No
7216	HTAA0366	Male	Malay	2020	38	Married	Secondary	Others	Rural	Yes	Yes	No	No	Yes	No	No	No	No	Yes
7217	HTAA0367	Male	Malay	2018	25	Single	Secondary	Private	Urban	Yes	Yes	No	No	No	No	No	No	No	No
7218	HTAA0368	Male	Malay	2021	31	Married	Secondary	Self-employed	Rural	Yes	Yes	No	No	Yes	No	No	No	No	Yes
7219	HTAA0370	Male	Malay	2018	39	Single	Secondary	Self-employed	Urban	Yes	Yes	No	No	No	No	No	No	No	No

No	Patient ID	Gender	Ethnic group	Year of diagnosis	Age	Marital status	Education level	Occupation	Place of residence	History of psy illness	Tobacco	Alcohol	Cannabis	ATS	Inhalant	Sedative	Hallucinogens	Opioids	Kratom
7220	HTAA0373	Male	Others	2018	32	Single	Secondary	Unemployed	Rural	Yes	Yes	No	Yes	Yes	No	No	No	Yes	No
7221	HTAA0378	Male	Malay	2020	43	Married	No formal education	Unemployed	Urban	Yes	Yes	No	No	Yes	No	No	No	Yes	Yes
7222	HTAA0385	Male	Malay	2020	37	Single	Secondary	Self-employed	Urban	Yes	Yes	No	No	Yes	No	No	No	No	No
7223	HTAA0390	Male	Malay	2018	30	Single	Secondary	Unemployed	Rural	No	Yes	No	No	No	No	No	No	No	No
7224	HTAA0391	Male	Malay	2019	21	Single	Tertiary	Unemployed	Urban	Yes	Yes	No	Yes	No	No	No	No	No	No
7225	HTAA0392	Male	Chinese	2020	28	Single	Secondary	Unemployed	Urban	Yes	Yes	Yes	No	Yes	No	No	No	No	No
7226	HTAA0393	Male	Malay	2019	44	Married	Primary	Self-employed	Rural	Yes	Yes	No	No	Yes	No	No	No	Yes	Yes
7227	HTAA0396	Male	Malay	2021	23	Single	Secondary	Private	Urban	Yes	Yes	No	Yes	Yes	No	No	Yes	No	No
7228	HTAA0397	Male	Malay	2020	27	Single	Tertiary	Unemployed	Rural	Yes	Yes	No	No	No	No	No	No	No	No
7229	HTAA0400	Male	Malay	2021	48	Others	Secondary	Self-employed	Urban	Yes	Yes	No	No	Yes	No	No	No	Yes	No
7230	HTAA0406	Male	Malay	2020	23	Single	Secondary	Self-employed	Urban	Yes	Yes	No	No	No	No	Yes	No	No	Yes
7231	HTAA0410	Male	Malay	2019	30	Single	Secondary	Unemployed	Urban	Yes	Yes	No	No	Yes	No	No	No	No	No
7232	HTAA0411	Male	Malay	2018	29	Single	Primary	Unemployed	Rural	Yes	No	Yes	No	No	Yes	No	No	No	No
7233	HTAA0416	Male	Malay	2019	28	Others	Tertiary	Unemployed	Urban	Yes	Yes	No	Yes	Yes	No	No	No	No	Yes
7234	HTAA0417	Male	Malay	2021	33	Married	Secondary	Unemployed	Rural	Yes	Yes	No	No	Yes	No	No	Yes	No	No
7235	HTAA0418	Male	Malay	2018	30	Others	Secondary	Self-employed	Rural	Yes	Yes	No	No	Yes	No	No	No	No	Yes
7236	HTAA0419	Male	Malay	2020	41	Single	Primary	Unemployed	Urban	Yes	Yes	Yes	Yes	Yes	No	No	No	No	No
7237	HTAA0423	Male	Malay	2020	33	Single	Tertiary	Private	Urban	Yes	Yes	No	No	Yes	No	No	No	No	No

No	Patient ID	Gender	Ethnic group	Year of diagnosis	Age	Marital status	Education level	Occupation	Place of residence	History of psy illness	Tobacco	Alcohol	Cannabis	ATS	Inhalant	Sedative	Hallucinogens	Opioids	Kratom
7238	HTAA0424	Male	Malay	2019	26	Single	Tertiary	Self-employed	Urban	Yes	Yes	No	No	Yes	No	No	No	Yes	No
7239	HTAA0425	Male	Malay	2018	22	Single	Secondary	Unemployed	Urban	Yes	Yes	Yes	No	No	No	No	No	No	No
7240	HTAA0429	Male	Malay	2020	49	Others	Primary	Unemployed	Urban	Yes	Yes	No	No	Yes	No	No	No	No	No
7241	HTAA0430	Male	Malay	2018	28	Single	Secondary	Unemployed	Rural	Yes	Yes	No	No	Yes	No	No	No	No	No
7242	HTAA0433	Male	Malay	2019	28	Single	Secondary	Self-employed	Urban	Yes	Yes	No	Yes	Yes	No	No	No	No	No
7243	HTAA0434	Male	Malay	2019	33	Single	Primary	Unemployed	Rural	Yes	Yes	Yes	No	Yes	No	No	No	Yes	No
7244	HTAA0436	Male	Malay	2021	23	Single	Secondary	Private	Rural	Yes	Yes	No	No	Yes	Yes	No	No	No	No
7245	HTAA0438	Male	Malay	2019	35	Married	Tertiary	Others	Rural	Yes	Yes	No	No	No	No	Yes	No	No	No
7246	HTAA0440	Male	Others	2021	32	Others	No formal education	Private	Urban	Yes	No	Yes	No	Yes	No	No	No	No	No
7247	HTAA0441	Male	Malay	2019	30	Married	Tertiary	Unemployed	Urban	No	No	No	No	No	No	Yes	No	No	No
7248	HTAA0443	Female	Chinese	2020	57	Others	No formal education	Unemployed	Urban	Yes	No	Yes	No	No	No	Yes	No	No	No
7249	HTAA0448	Male	Malay	2018	36	Single	Secondary	Unemployed	Rural	Yes	Yes	No	No	Yes	No	No	No	No	No
7250	HTAA0449	Female	Chinese	2018	24	Single	Tertiary	Government	Rural	Yes	No	Yes	No	No	No	Yes	No	No	No
7251	HTAA0450	Male	Malay	2020	29	Single	Secondary	Unemployed	Rural	Yes	No	No	No	Yes	No	No	No	No	No
7252	HTAA0451	Female	Chinese	2019	57	Married	Secondary	Unemployed	Urban	Yes	No	Yes	No	No	No	Yes	No	No	No
7253	HTAA0452	Male	Indian	2021	39	Married	Primary	Unemployed	Urban	Yes	Yes	Yes	No	Yes	No	No	No	No	No
7254	HTAA0453	Female	Malay	2019	40	Others	Secondary	Self-employed	Urban	Yes	No	Yes	No	No	No	No	No	No	No
7255	HTAA0455	Female	Malay	2018	29	Married	Secondary	Government	Urban	Yes	No	No	No	No	No	Yes	No	No	No
7256	HTAA0457	Female	Malay	2020	22	Single	Secondary	Private	Urban	Yes	Yes	Yes	Yes	No	No	No	No	No	No
7257	HTAA0458	Male	Malay	2018	29	Single	Secondary	Government	Urban	Yes	Yes	No	Yes	No	No	No	No	No	No
7258	HTAA0459	Female	Chinese	2019	19	Single	Tertiary	Unemployed	Urban	Yes	No	Yes	No	No	No	No	No	No	No

No	Patient ID	Gender	Ethnic group	Year of diagnosis	Age	Marital status	Education level	Occupation	Place of residence	History of psy illness	Tobacco	Alcohol	Cannabis	ATS	Inhalant	Sedative	Hallucinogens	Opioids	Kratom
7259	HTAA0461	Female	Malay	2018	45	Married	Secondary	Self-employed	Urban	Yes	No	No	No	No	No	Yes	No	No	No
7260	HTAA0463	Female	Others	2018	31	Married	No formal education	Unemployed	Urban	Yes	No	No	No	No	No	Yes	No	No	No
7261	HTAA0464	Male	Malay	2020	37	Others	Primary	Others	Urban	No	No	No	Yes	Yes	No	No	No	No	No
7262	HTAA0465	Male	Malay	2019	17	Single	Secondary	Unemployed	Urban	Yes	Yes	No	Yes	Yes	No	No	No	No	No
7263	HTAA0466	Female	Malay	2019	41	Married	No formal education	Others	Rural	Yes	No	No	No	No	No	Yes	No	No	No
7264	HTAA0467	Male	Malay	2018	48	Others	Secondary	Others	Urban	Yes	Yes	No	No	Yes	No	No	No	No	No
7265	HTAA0471	Male	Malay	2018	40	Single	Secondary	Unemployed	Urban	No	Yes	No	No	Yes	No	No	No	Yes	No
7266	HTAA0472	Female	Malay	2018	31	Others	No formal education	Unemployed	Urban	Yes	No	No	No	Yes	No	No	No	No	No
7267	HTAA0473	Male	Malay	2019	29	Married	Tertiary	Government	Urban	Yes	Yes	No	Yes	No	No	No	No	No	No
7268	HTAA0476	Female	Malay	2019	19	Others	Secondary	Unemployed	Urban	Yes	No	No	No	No	No	Yes	No	No	No
7269	HTAA0477	Male	Malay	2021	28	Single	Secondary	Private	Urban	Yes	Yes	No	No	No	No	No	No	No	Yes
7270	HTAA0479	Male	Malay	2020	44	Others	Secondary	Self-employed	Urban	Yes	Yes	No	No	No	No	No	No	No	No
7271	HTAA0480	Male	Chinese	2018	37	Single	Secondary	Private	Rural	Yes	Yes	No	No	No	No	Yes	No	No	No
7272	HTAA0482	Male	Malay	2019	29	Single	Secondary	Private	Urban	Yes	Yes	No	No	No	No	Yes	No	No	No
7273	HTAA0483	Male	Malay	2019	19	Single	Tertiary	Unemployed	Rural	Yes	Yes	No	No	No	No	No	No	No	No
7274	HTAA0484	Male	Malay	2018	35	Married	Secondary	Private	Urban	Yes	Yes	No	No	No	No	Yes	No	No	No
7275	HTAA0485	Male	Malay	2018	26	Single	Secondary	Self-employed	Rural	Yes	Yes	No	No	No	No	No	No	No	No
7276	HTAA0487	Female	Malay	2019	18	Single	Secondary	Unemployed	Urban	Yes	Yes	No	No	No	No	Yes	No	No	No
7277	HTAA0489	Female	Malay	2019	17	Single	Secondary	Unemployed	Urban	Yes	Yes	No	No	No	No	Yes	No	No	No
7278	HTAA0490	Male	Indian	2020	31	Single	Tertiary	Self-employed	Urban	Yes	Yes	No	No	Yes	No	No	No	No	No

No	Patient ID	Gender	Ethnic group	Year of diagnosis	Age	Marital status	Education level	Occupation	Place of residence	History of psy illness	Tobacco	Alcohol	Cannabis	ATS	Inhalant	Sedative	Hallucinogens	Opioids	Kratom
7279	HTAA0491	Female	Chinese	2019	52	Others	Secondary	Self-employed	Urban	Yes	Yes	Yes	No	No	No	No	No	No	No
7280	HTAA0493	Female	Malay	2019	40	Others	Tertiary	Unemployed	Urban	Yes	No	No	No	No	No	Yes	No	No	No
7281	HTAA0494	Male	Others	2020	35	Single	No formal education	Unemployed	Rural	Yes	No	No	No	Yes	No	No	No	No	No
7282	HTAA0495	Female	Malay	2019	23	Others	Tertiary	Unemployed	Urban	Yes	Yes	No	Yes	No	No	Yes	No	No	No
7283	HTAA0497	Female	Malay	2019	38	Married	Secondary	Self-employed	Urban	Yes	No	No	No	No	No	Yes	No	No	No
7284	HTAA0498	Female	Malay	2019	18	Others	Secondary	Unemployed	Rural	Yes	No	No	No	No	No	Yes	No	No	No
7285	HTAA0500	Female	Malay	2019	23	Married	Secondary	Private	Urban	Yes	No	No	No	No	No	Yes	No	No	No
7286	HTAA0501	Male	Malay	2018	24	Single	Tertiary	Private	Urban	Yes	Yes	No	No	No	No	No	No	No	No
7287	HTAA0503	Male	Malay	2019	23	Single	Tertiary	Unemployed	Urban	Yes	Yes	No	No	No	No	No	No	No	No
7288	HTAA0504	Male	Malay	2018	58	Others	Secondary	Unemployed	Rural	Yes	Yes	No	No	Yes	No	No	No	No	No
7289	HTAA0505	Male	Malay	2020	25	Single	Secondary	Unemployed	Rural	Yes	Yes	No	No	Yes	No	No	No	Yes	No
7290	HTAA0507	Male	Malay	2018	36	Others	Secondary	Self-employed	Urban	Yes	Yes	No	Yes	Yes	No	No	No	No	Yes
7291	HTAA0510	Male	Malay	2019	27	Single	Primary	Unemployed	Rural	Yes	Yes	No	No	Yes	No	No	No	No	No
7292	HTAA0512	Male	Chinese	2019	44	Others	No formal education	Unemployed	Urban	Yes	No	No	No	No	No	Yes	No	No	No
7293	HTAA0513	Male	Malay	2019	35	Others	Secondary	Others	Urban	Yes	Yes	No	Yes	No	No	Yes	No	No	No
7294	HTAA0515	Male	Malay	2018	25	Single	Tertiary	Government	Urban	Yes	No	No	No	No	No	Yes	No	No	No
7295	HTAA0516	Female	Malay	2019	32	Others	Tertiary	Government	Urban	Yes	No	No	No	Yes	No	No	No	No	No
7296	HTAA0518	Female	Malay	2020	28	Single	Tertiary	Unemployed	Rural	Yes	No	No	No	No	No	Yes	No	No	No
7297	HTAA0520	Male	Malay	2019	26	Single	Primary	Private	Urban	Yes	Yes	No	No	Yes	No	No	No	No	No
7298	HTAA0521	Male	Chinese	2018	51	Married	Secondary	Self-employed	Urban	Yes	Yes	Yes	No	No	No	Yes	No	No	No
7299	HTAA0524	Male	Malay	2019	32	Single	Primary	Others	Urban	Yes	Yes	No	No	No	No	No	No	No	No

No	Patient ID	Gender	Ethnic group	Year of diagnosis	Age	Marital status	Education level	Occupation	Place of residence	History of psy illness	Tobacco	Alcohol	Cannabis	ATS	Inhalant	Sedative	Hallucinogens	Opioids	Kratom
7300	HTAA0526	Male	Malay	2020	32	Others	Primary	Private	Urban	Yes	Yes	Yes	No	Yes	No	No	No	Yes	No
7301	HTAA0528	Male	Malay	2019	32	Married	No formal education	Others	Urban	No	Yes	Yes	No	No	No	Yes	No	No	No
7302	HTAA0529	Male	Malay	2021	33	Single	Secondary	Unemployed	Urban	Yes	Yes	No	Yes	Yes	No	No	No	No	No
7303	HTAA0530	Female	Malay	2019	23	Others	Secondary	Unemployed	Urban	Yes	No	No	No	No	No	Yes	No	No	No
7304	HTAA0531	Male	Malay	2018	38	Others	Secondary	Unemployed	Urban	No	Yes	Yes	Yes	Yes	No	No	No	Yes	No
7305	HTAA0532	Female	Chinese	2018	51	Married	Secondary	Self-employed	Urban	Yes	No	No	No	No	No	Yes	No	No	No
7306	HTAA0534	Female	Malay	2018	24	Married	Secondary	Others	Rural	Yes	Yes	No	No	No	No	Yes	No	No	No
7307	HTAA0540	Female	Chinese	2019	26	Single	Tertiary	Unemployed	Urban	Yes	Yes	No	No	No	No	Yes	No	No	No
7308	HTAA0542	Female	Indian	2018	42	Married	No formal education	Private	Urban	Yes	No	No	No	No	No	Yes	No	No	No
7309	HTAA0544	Female	Malay	2019	44	Others	Secondary	Self-employed	Rural	Yes	Yes	No	No	No	No	No	No	No	No
7310	HTAA0545	Male	Chinese	2019	53	Single	Secondary	Unemployed	Rural	Yes	Yes	No	No	No	No	No	No	No	No
7311	HTAA0546	Male	Malay	2021	27	Single	Secondary	Self-employed	Rural	Yes	Yes	No	No	Yes	No	No	No	No	No
7312	HTAA0547	Male	Malay	2019	50	Single	Secondary	Unemployed	Urban	Yes	Yes	No	No	Yes	No	No	No	No	No
7313	HTAA0548	Male	Malay	2019	35	Single	Secondary	Unemployed	Urban	Yes	No	No	No	No	No	Yes	No	No	No
7314	HTAA0549	Male	Malay	2019	25	Single	Secondary	Private	Urban	Yes	Yes	No	Yes	No	No	No	No	No	No
7315	HTAA0550	Female	Malay	2019	39	Married	Secondary	Unemployed	Urban	Yes	No	No	No	No	No	Yes	No	No	No
7316	HTAA0552	Female	Malay	2019	26	Single	Secondary	Private	Urban	Yes	Yes	No	Yes	No	No	No	Yes	No	No
7317	HTAA0553	Male	Malay	2020	36	Single	Secondary	Unemployed	Rural	Yes	Yes	No	No	Yes	No	No	No	No	No
7318	HTAA0555	Male	Malay	2018	45	Single	Secondary	Unemployed	Urban	Yes	Yes	No	No	No	No	No	No	No	No
7319	HTAA0556	Male	Malay	2020	57	Married	Primary	Self-employed	Urban	Yes	Yes	No	No	No	No	Yes	No	No	No

No	Patient ID	Gender	Ethnic group	Year of diagnosis	Age	Marital status	Education level	Occupation	Place of residence	History of psy illness	Tobacco	Alcohol	Cannabis	ATS	Inhalant	Sedative	Hallucinogens	Opioids	Kratom
7320	HTAA0557	Male	Indian	2018	50	Married	Secondary	Unemployed	Urban	Yes	Yes	Yes	No	Yes	No	No	No	No	No
7321	HTAA0559	Male	Malay	2018	21	Single	Tertiary	Unemployed	Urban	Yes	Yes	No	Yes	No	No	No	No	No	Yes
7322	HTAA0560	Male	Malay	2018	29	Single	Primary	Unemployed	Urban	Yes	Yes	No	No	No	No	Yes	No	No	No
7323	HTAA0560	Female	Indian	2019	17	Single	Secondary	Unemployed	Urban	Yes	No	No	Yes	No	No	Yes	No	No	No
7324	HTAA0563	Male	Chinese	2019	22	Single	Secondary	Unemployed	Urban	Yes	Yes	Yes	No	No	No	No	No	No	No
7325	HTAA0566	Female	Malay	2018	24	Others	Tertiary	Private	Rural	Yes	No	Yes	No	No	No	Yes	No	No	No
7326	HTAA0567	Male	Malay	2020	39	Others	Primary	Unemployed	Rural	Yes	Yes	No	No	Yes	No	No	No	No	No
7327	HTAA0568	Female	Malay	2019	27	Single	Secondary	Unemployed	Urban	Yes	No	No	No	No	No	Yes	No	No	No
7328	HTAA0569	Male	Malay	2021	24	Single	Tertiary	Unemployed	Urban	Yes	Yes	No	Yes	Yes	No	No	No	No	No
7329	HTAA0570	Female	Malay	2019	26	Others	Secondary	Unemployed	Urban	Yes	No	No	No	No	No	Yes	No	No	No
7330	HTAA0571	Male	Malay	2019	25	Single	Secondary	Self-employed	Rural	Yes	Yes	No	No	Yes	No	No	No	No	No
7331	HTAA0573	Male	Malay	2019	18	Single	Secondary	Unemployed	Rural	No	Yes	No	No	No	No	No	No	No	No
7332	HTAA0574	Female	Malay	2019	34	Others	Secondary	Unemployed	Rural	Yes	No	No	No	No	No	Yes	No	No	No
7333	HTAA0575	Male	Malay	2018	46	Single	Secondary	Unemployed	Rural	Yes	Yes	No	No	Yes	No	No	No	No	Yes
7334	HTAA0577	Male	Malay	2018	21	Single	Tertiary	Unemployed	Urban	Yes	Yes	No	No	Yes	No	No	No	No	Yes
7335	HTAA0578	Female	Chinese	2019	18	Single	Secondary	Unemployed	Urban	Yes	No	No	No	No	No	Yes	No	No	No
7336	HTAA0581	Male	Malay	2020	30	Married	Tertiary	Private	Urban	Yes	Yes	No	Yes	No	No	No	No	No	No
7337	HTAA0583	Male	Malay	2018	48	Others	Secondary	Unemployed	Urban	Yes	Yes	No	No	Yes	No	No	No	No	No
7338	HTAA0584	Male	Malay	2019	57	Single	Secondary	Others	Rural	Yes	Yes	No	No	No	No	No	No	No	No
7339	HTAA0585	Male	Chinese	2019	25	Single	Tertiary	Private	Urban	Yes	Yes	Yes	Yes	Yes	No	No	No	No	No
7340	HTAA0587	Male	Malay	2020	38	Married	Secondary	Self-employed	Rural	Yes	Yes	No	No	Yes	No	No	No	No	No

No	Patient ID	Gender	Ethnic group	Year of diagnosis	Age	Marital status	Education level	Occupation	Place of residence	History of psy illness	Tobacco	Alcohol	Cannabis	ATS	Inhalant	Sedative	Hallucinogens	Opioids	Kratom
7341	HTAA0588	Male	Malay	2019	21	Single	Secondary	Unemployed	Rural	Yes	Yes	No	Yes	No	No	No	No	No	No
7342	HTAA0589	Male	Malay	2018	28	Single	Secondary	Unemployed	Urban	Yes	Yes	No	No	Yes	No	No	No	No	No
7343	HTAA0590	Male	Chinese	2018	37	Single	Primary	Unemployed	Urban	Yes	Yes	Yes	No	Yes	No	No	No	No	No
7344	HTAA0591	Female	Malay	2019	26	Single	Tertiary	Government	Urban	Yes	No	No	No	No	No	Yes	No	No	No
7345	HTAA0592	Male	Chinese	2019	36	Married	No formal education	Self-employed	Urban	Yes	No	No	No	Yes	No	No	No	No	No
7346	HTAA0593	Female	Malay	2019	37	Married	Secondary	Unemployed	Urban	Yes	No	No	No	No	No	Yes	No	No	No
7347	HTAA0594	Male	Malay	2019	32	Single	Secondary	Unemployed	Urban	Yes	Yes	No	No	Yes	No	No	No	No	No
7348	HTAA0595	Female	Chinese	2020	47	Married	Secondary	Unemployed	Urban	Yes	No	Yes	No	No	No	Yes	No	No	No
7349	HTAA0597	Female	Malay	2019	19	Single	Tertiary	Unemployed	Urban	Yes	Yes	No	No	No	No	Yes	No	No	No
7350	HTAA0598	Male	Malay	2020	25	Single	Tertiary	Unemployed	Urban	Yes	Yes	No	No	No	No	Yes	No	No	No
7351	HTAA0599	Female	Malay	2019	23	Single	Secondary	Private	Urban	Yes	No	No	No	No	No	Yes	No	No	No
7352	HTAA0603	Female	Malay	2019	24	Single	Secondary	Private	Urban	Yes	No	No	No	No	No	Yes	No	No	No
7353	HTAA0604	Male	Indian	2018	48	Others	Primary	Unemployed	Urban	Yes	No	No	No	Yes	No	Yes	No	No	No
7354	HTAA0606	Male	Malay	2019	32	Others	No formal education	Unemployed	Rural	Yes	Yes	No	No	Yes	No	Yes	No	No	No
7355	HTAA0607	Female	Chinese	2019	20	Single	Tertiary	Unemployed	Urban	Yes	No	Yes	No	No	No	Yes	No	No	No
7356	HTAA0609	Female	Malay	2019	31	Others	Tertiary	Private	Urban	Yes	No	No	No	No	No	Yes	No	No	No
7357	HTAA0611	Female	Malay	2021	32	Single	Secondary	Unemployed	Urban	Yes	No	No	No	No	No	Yes	No	No	No
7358	HTAA0612	Male	Malay	2020	29	Single	Secondary	Unemployed	Urban	Yes	Yes	Yes	No	Yes	No	No	No	No	No
7359	HTAA0613	Female	Malay	2021	33	Single	Tertiary	Self-employed	Urban	Yes	No	No	No	No	No	Yes	No	No	No
7360	HTAA0614	Male	Malay	2018	34	Married	Primary	Unemployed	Rural	Yes	Yes	No	No	Yes	No	Yes	No	No	No
7361	HTAA0617	Male	Malay	2018	33	Single	Secondary	Unemployed	Urban	No	Yes	No	No	No	No	No	No	No	No

No	Patient ID	Gender	Ethnic group	Year of diagnosis	Age	Marital status	Education level	Occupation	Place of residence	History of psy illness	Tobacco	Alcohol	Cannabis	ATS	Inhalant	Sedative	Hallucinogens	Opioids	Kratom
7362	HTAA0619	Male	Malay	2018	24	Single	Tertiary	Unemployed	Urban	Yes	Yes	No	Yes	Yes	No	No	No	No	No
7363	HTAA0622	Male	Malay	2018	25	Single	Primary	Self-employed	Rural	Yes	No	No	No	Yes	No	No	No	Yes	No
7364	HTAA0623	Male	Malay	2019	31	Single	Secondary	Unemployed	Rural	Yes	Yes	No	No	No	No	No	No	No	No
7365	HTAA0624	Male	Chinese	2019	27	Others	No formal education	Unemployed	Urban	No	Yes	No	No	No	No	No	No	No	No
7366	HTAA0626	Male	Malay	2020	37	Others	Primary	Others	Rural	Yes	No	No	No	Yes	No	No	No	Yes	No
7367	HTAA0627	Male	Malay	2018	45	Single	Secondary	Unemployed	Urban	Yes	Yes	No	No	Yes	No	No	No	No	Yes
7368	HTAA0629	Male	Malay	2018	22	Single	Secondary	Self-employed	Urban	Yes	Yes	No	No	No	No	No	No	No	No
7369	HTAA0631	Male	Malay	2018	26	Single	Tertiary	Others	Urban	Yes	Yes	No	Yes	No	No	No	No	No	No
7370	HTAA0635	Female	Malay	2019	19	Single	Tertiary	Unemployed	Urban	Yes	No	No	No	No	No	Yes	No	No	No
7371	HTAA0637	Female	Malay	2019	26	Married	Secondary	Self-employed	Rural	Yes	No	No	No	No	No	Yes	No	No	No
7372	HTAA0644	Male	Malay	2018	31	Others	Primary	Unemployed	Urban	Yes	Yes	No	No	No	No	Yes	No	No	No
7373	HTAA0645	Female	Malay	2019	17	Single	Secondary	Unemployed	Rural	Yes	No	No	No	No	No	Yes	No	No	No
7374	HTAA0646	Male	Malay	2019	25	Single	Secondary	Unemployed	Urban	No	Yes	No	No	No	No	No	No	No	No
7375	HTAA0647	Female	Malay	2019	26	Single	Tertiary	Unemployed	Urban	Yes	Yes	No	Yes	No	No	Yes	No	No	No
7376	HTAA0648	Male	Malay	2018	54	Single	Tertiary	Unemployed	Urban	Yes	Yes	No	No	No	No	Yes	No	No	No
7377	HTAA0652	Male	Malay	2018	28	Single	Secondary	Unemployed	Rural	Yes	No	No	Yes	Yes	No	No	No	No	Yes
7378	HTAA0653	Female	Malay	2019	26	Single	Tertiary	Unemployed	Urban	Yes	No	No	No	No	No	Yes	No	No	No
7379	HTAA0654	Male	Malay	2019	21	Single	Primary	Unemployed	Rural	Yes	Yes	No	No	Yes	No	No	No	No	No
7380	HTAA0656	Female	Malay	2021	43	Others	Secondary	Unemployed	Urban	No	No	No	No	Yes	No	No	No	No	No
7381	HTAA0657	Female	Malay	2018	37	Married	Secondary	Self-employed	Urban	Yes	No	No	No	No	No	Yes	No	No	No
7382	HTAA0658	Female	Malay	2018	22	Single	Tertiary	Unemployed	Urban	Yes	No	No	No	No	No	Yes	No	No	No

No	Patient ID	Gender	Ethnic group	Year of diagnosis	Age	Marital status	Education level	Occupation	Place of residence	History of psy illness	Tobacco	Alcohol	Cannabis	ATS	Inhalant	Sedative	Hallucinogens	Opioids	Kratom
7383	HTAA0659	Female	Chinese	2018	22	Single	Tertiary	Unemployed	Urban	Yes	No	Yes	No	No	No	Yes	No	No	No
7384	HTAA0660	Female	Malay	2018	33	Married	Secondary	Others	Urban	No	No	No	No	No	No	Yes	No	No	No
7385	HTAA0662	Female	Malay	2021	54	Others	Secondary	Unemployed	Urban	Yes	No	No	No	No	No	Yes	No	No	No
7386	HTAA0663	Female	Malay	2018	40	Others	Secondary	Private	Urban	Yes	No	No	No	No	No	Yes	No	No	No
7387	HTAA0664	Female	Malay	2019	49	Married	No formal education	Others	Urban	Yes	No	No	No	No	No	Yes	No	No	No
7388	HTAA0665	Female	Malay	2019	23	Single	Secondary	Self-employed	Urban	No	No	No	No	No	No	Yes	No	No	No
7389	HTAA0668	Female	Malay	2018	39	Married	Secondary	Unemployed	Urban	Yes	No	No	No	No	No	Yes	No	No	No
7390	HTAA0669	Female	Malay	2019	37	Married	Secondary	Unemployed	Urban	Yes	No	No	No	No	No	Yes	No	No	No
7391	HTAA0670	Male	Malay	2018	23	Single	Secondary	Private	Urban	Yes	Yes	No	Yes	No	No	No	No	No	No
7392	HTAA0672	Male	Malay	2021	33	Single	Tertiary	Unemployed	Rural	Yes	Yes	No	No	Yes	No	No	No	No	No
7393	HTAA0673	Female	Malay	2018	54	Married	Secondary	Unemployed	Urban	Yes	No	No	No	No	No	Yes	No	No	No
7394	HTAA0674	Male	Malay	2020	31	Single	Tertiary	Private	Urban	Yes	No	No	No	No	No	Yes	Yes	No	No
7395	HTAA0675	Female	Malay	2019	21	Single	Tertiary	Private	Rural	Yes	No	No	No	No	No	Yes	No	No	No
7396	HTAA0676	Male	Malay	2018	54	Married	Primary	Unemployed	Urban	Yes	Yes	No	No	No	No	No	No	No	No
7397	HTAA0677	Female	Malay	2020	40	Others	Secondary	Private	Urban	Yes	Yes	No	Yes	Yes	No	No	No	No	No
7398	HTAA0678	Male	Malay	2019	30	Married	Secondary	Government	Urban	Yes	Yes	No	Yes	No	No	No	No	No	Yes
7399	HTAA0679	Female	Malay	2019	19	Single	Tertiary	Unemployed	Urban	Yes	No	No	No	No	No	Yes	No	No	No
7400	HTAA0680	Male	Chinese	2019	36	Married	No formal education	Unemployed	Rural	No	No	No	No	No	No	Yes	No	No	No
7401	HTAA0681	Female	Malay	2019	28	Single	Secondary	Private	Urban	Yes	Yes	No	Yes	Yes	No	No	No	No	No
7402	HTAA0684	Male	Malay	2019	30	Married	Secondary	Government	Urban	Yes	No	No	No	Yes	No	Yes	No	No	No
7403	HTAA0689	Female	Malay	2018	27	Married	Tertiary	Private	Urban	Yes	No	No	No	No	No	Yes	No	No	No

No	Patient ID	Gender	Ethnic group	Year of diagnosis	Age	Marital status	Education level	Occupation	Place of residence	History of psy illness	Tobacco	Alcohol	Cannabis	ATS	Inhalant	Sedative	Hallucinogens	Opioids	Kratom
7404	HTAA0690	Male	Malay	2019	55	Married	Secondary	Private	Rural	Yes	Yes	No	No	No	No	No	No	No	No
7405	HTAA0693	Female	Others	2019	25	Married	Secondary	Unemployed	Urban	Yes	No	No	No	No	No	Yes	No	No	No
7406	HTAA0694	Female	Malay	2018	36	Married	No formal education	Others	Urban	No	Yes	No	No	No	No	Yes	No	No	No
7407	HTAA0695	Female	Malay	2018	25	Married	Secondary	Unemployed	Urban	No	No	No	No	No	No	Yes	No	No	No
7408	HTAA0696	Female	Chinese	2019	31	Single	Tertiary	Private	Urban	Yes	Yes	Yes	No	No	No	Yes	No	No	No
7409	HTAA0699	Female	Indian	2019	40	Married	Tertiary	Government	Urban	Yes	No	No	No	No	No	Yes	No	No	No
7410	HTAA0700	Male	Malay	2019	24	Single	Secondary	Self-employed	Rural	Yes	Yes	No	No	No	No	Yes	No	No	No
7411	HTAA0702	Female	Malay	2018	33	Married	No formal education	Self-employed	Rural	Yes	Yes	No	No	No	No	Yes	No	No	Yes
7412	HTAA0704	Male	Malay	2018	28	Single	Secondary	Government	Urban	Yes	No	Yes	No	Yes	No	Yes	No	No	No
7413	HTAA0710	Male	Chinese	2019	34	Single	Primary	Unemployed	Urban	Yes	No	No	No	No	No	Yes	No	No	No
7414	HTAR0018	Male	Malay	2018	50	Others	Secondary	Self-employed	Urban	No	No	No	No	No	No	No	No	Yes	No
7415	HTAR0020	Male	Chinese	2018	64	Married	Secondary	Private	Urban	No	No	No	No	Yes	No	No	No	Yes	No
7416	HTAR0021	Male	Indian	2018	41	Single	Secondary	Others	Urban	No	No	No	No	Yes	No	No	No	Yes	No
7417	HTAR0022	Male	Malay	2018	45	Others	No formal education	Others	Urban	No	No	No	No	Yes	No	No	No	Yes	No
7418	HTAR0024	Male	Malay	2019	38	Others	Secondary	Unemployed	Urban	No	No	No	No	Yes	No	No	No	Yes	No
7419	HTAR0025	Male	Malay	2019	42	Single	Secondary	Private	Urban	No	No	No	No	Yes	No	Yes	No	Yes	No
7420	HTAR0026	Male	Malay	2020	36	Single	Secondary	Self-employed	Urban	Yes	No	No	No	No	No	No	No	Yes	No
7421	HTAR0027	Male	Malay	2020	39	Married	Secondary	Private	Urban	Yes	No	No	No	No	No	No	No	Yes	No
7422	HTAR0028	Male	Malay	2021	41	Others	Secondary	Unemployed	Urban	Yes	No	No	No	Yes	No	No	No	Yes	No
7423	HTAR0029	Male	Malay	2021	32	Married	Secondary	Private	Urban	Yes	No	No	No	No	No	No	No	Yes	No
7424	HTAR0030	Male	Indian	2021	49	Single	Tertiary	Unemployed	Urban	No	No	No	No	Yes	No	No	No	Yes	No

No	Patient ID	Gender	Ethnic group	Year of diagnosis	Age	Marital status	Education level	Occupation	Place of residence	History of psy illness	Tobacco	Alcohol	Cannabis	ATS	Inhalant	Sedative	Hallucinogens	Opioids	Kratom
7425	HTAR0031	Male	Malay	2019	41	Single	Secondary	Unemployed	Urban	No	No	No	No	Yes	No	No	Yes	Yes	No
7426	HTAR0032	Male	Malay	2021	40	Married	Secondary	Unemployed	Urban	Yes	No	No	No	Yes	No	No	No	Yes	No
7427	HTAR0033	Male	Indian	2021	45	Single	Secondary	Private	Urban	Yes	No	No	Yes	Yes	No	No	No	Yes	No
7428	HTAR0034	Male	Malay	2021	29	Married	Secondary	Unemployed	Urban	Yes	No	No	No	No	No	No	No	Yes	No
7429	HTAR0035	Male	Malay	2021	44	Single	Secondary	Unemployed	Urban	Yes	No	No	No	Yes	No	No	No	Yes	No
7430	HTAR0037	Male	Malay	2020	51	Married	Secondary	Unemployed	Rural	Yes	No	No	No	Yes	No	No	No	Yes	No
7431	HTAR0038	Male	Chinese	2020	39	Married	Secondary	Self-employed	Urban	Yes	No	No	No	Yes	No	No	No	Yes	No
7432	HTAR0039	Female	Malay	2020	36	Single	Primary	Unemployed	Urban	Yes	No	No	No	Yes	No	No	No	Yes	No
7433	HTAR0041	Male	Chinese	2019	50	Single	Primary	Private	Urban	Yes	No	No	No	Yes	No	No	No	Yes	No
7434	HTAR0043	Male	Malay	2020	43	Single	Secondary	Private	Urban	Yes	No	No	No	No	No	No	No	Yes	No
7435	HTAR0047	Male	Malay	2018	41	Married	Tertiary	Private	Urban	Yes	No	No	No	No	No	No	No	Yes	No
7436	HTAR0048	Male	Indian	2018	35	Married	No formal education	Self-employed	Urban	Yes	No	No	Yes	Yes	No	No	No	Yes	No
7437	HTAR0049	Female	Indian	2020	29	Single	Secondary	Unemployed	Urban	No	No	No	No	No	No	Yes	No	Yes	No
7438	HTAR0054	Male	Malay	2019	37	Others	No formal education	Unemployed	Urban	Yes	No	No	No	No	No	No	No	Yes	No
7439	HTAR0064	Male	Malay	2020	44	Married	Secondary	Unemployed	Urban	Yes	No	No	No	Yes	No	No	No	No	No
7440	HTAR0066	Male	Malay	2019	39	Single	Primary	Self-employed	Urban	Yes	No	No	No	Yes	No	No	No	Yes	No
7441	HTAR0067	Female	Malay	2019	31	Married	Secondary	Unemployed	Urban	Yes	Yes	No	No	No	No	No	No	Yes	No
7442	HTAR0068	Male	Malay	2020	31	Married	Primary	Private	Urban	Yes	Yes	No	No	Yes	No	No	No	Yes	No
7443	HTAR0070	Male	Malay	2019	41	Married	Tertiary	Government	Urban	No	No	No	No	Yes	No	No	No	No	No
7444	HTAR0071	Male	Indian	2019	53	Married	No formal education	Private	Urban	Yes	Yes	Yes	No	No	No	No	No	No	No
7445	HTAR0074	Male	Chinese	2019	54	Married	No formal education	Private	Urban	Yes	No	No	No	No	No	No	No	Yes	No

No	Patient ID	Gender	Ethnic group	Year of diagnosis	Age	Marital status	Education level	Occupation	Place of residence	History of psy illness	Tobacco	Alcohol	Cannabis	ATS	Inhalant	Sedative	Hallucinogens	Opioids	Kratom
7446	HTAR0079	Male	Indian	2019	47	Married	Primary	Unemployed	Urban	Yes	No	Yes	No	No	No	No	No	No	No
7447	HTAR0083	Male	Indian	2018	43	Married	No formal education	Private	Urban	Yes	No	Yes	No	No	No	No	No	No	No
7448	HTAR0084	Male	Indian	2020	35	Single	No formal education	Others	Urban	Yes	No	Yes	No	Yes	No	No	No	No	No
7449	HTAR0087	Male	Malay	2018	26	Single	Tertiary	Unemployed	Urban	Yes	No	No	No	No	No	No	No	Yes	No
7450	HTAR0088	Male	Indian	2018	64	Married	Secondary	Private	Urban	Yes	No	Yes	No	No	No	No	No	No	No
7451	HTAR0089	Female	Others	2019	22	Single	Tertiary	Unemployed	Urban	Yes	No	No	Yes	No	No	No	No	No	No
7452	HTAR0090	Male	Chinese	2018	57	Married	No formal education	Unemployed	Urban	Yes	No	Yes	No	No	No	No	No	No	No
7453	HTAR0093	Male	Indian	2018	67	Others	No formal education	Others	Urban	Yes	No	Yes	No	No	No	No	No	No	No
7454	HTAR0095	Female	Indian	2018	25	Others	Secondary	Unemployed	Urban	Yes	No	Yes	No	No	No	No	No	No	No
7455	HTAR0096	Male	Chinese	2019	23	Single	Tertiary	Unemployed	Urban	Yes	No	Yes	No	No	No	No	No	No	No
7456	HTAR0098	Male	Indian	2018	25	Single	Tertiary	Unemployed	Urban	Yes	Yes	Yes	Yes	Yes	No	No	No	No	No
7457	HTAR0100	Male	Chinese	2020	43	Married	Secondary	Unemployed	Urban	Yes	Yes	Yes	No	No	No	No	No	No	No
7458	HTAR0101	Male	Malay	2019	29	Single	Primary	Unemployed	Urban	Yes	No	No	No	Yes	No	No	No	Yes	No
7459	HTAR0102	Male	Indian	2020	31	Single	Secondary	Unemployed	Urban	Yes	Yes	Yes	No	No	No	No	No	No	No
7460	HTAR0104	Male	Indian	2019	36	Married	Secondary	Self-employed	Urban	Yes	Yes	Yes	No	No	No	No	No	No	No
7461	HTAR0105	Male	Indian	2019	49	Single	Secondary	Private	Urban	Yes	Yes	Yes	No	No	No	No	No	No	No
7462	HTAR0106	Male	Chinese	2018	36	Married	Secondary	Unemployed	Urban	Yes	No	Yes	No	Yes	No	No	No	No	No
7463	HTAR0107	Female	Others	2018	31	Married	Secondary	Unemployed	Urban	Yes	No	No	No	Yes	No	No	No	No	No
7464	HTAR0108	Male	Malay	2018	43	Single	Secondary	Unemployed	Urban	Yes	No	No	No	Yes	No	No	No	Yes	No
7465	HTAR0110	Female	Malay	2019	57	Others	Tertiary	Self-employed	Urban	Yes	Yes	Yes	No	No	No	No	No	No	No
7466	HTAR0111	Male	Chinese	2019	60	Single	Primary	Private	Urban	Yes	Yes	No	No	Yes	No	No	No	Yes	No

No	Patient ID	Gender	Ethnic group	Year of diagnosis	Age	Marital status	Education level	Occupation	Place of residence	History of psy illness	Tobacco	Alcohol	Cannabis	ATS	Inhalant	Sedative	Hallucinogens	Opioids	Kratom
7467	HTAR0113	Male	Indian	2020	47	Married	Primary	Private	Urban	Yes	Yes	Yes	No	No	No	No	No	No	No
7468	HTAR0115	Male	Malay	2019	34	Single	Tertiary	Unemployed	Urban	Yes	Yes	No	No	No	No	No	No	No	No
7469	HTAR0116	Male	Malay	2020	30	Single	Secondary	Unemployed	Urban	Yes	Yes	No	No	Yes	No	No	No	No	No
7470	HTAR0118	Male	Indian	2020	47	Married	Secondary	Private	Urban	Yes	Yes	Yes	No	No	No	No	No	No	No
7471	HTAR0119	Female	Malay	2020	44	Married	Tertiary	Self-employed	Urban	Yes	Yes	No	No	Yes	No	No	No	No	No
7472	HTAR0120	Male	Malay	2019	32	Single	Secondary	Self-employed	Urban	Yes	No	No	Yes	No	No	No	No	No	Yes
7473	HTAR0121	Male	Malay	2019	23	Single	Secondary	Government	Urban	Yes	Yes	No	Yes	No	No	No	No	No	No
7474	HTAR0123	Female	Indian	2018	54	Married	Primary	Unemployed	Urban	Yes	No	Yes	No	No	No	No	No	No	No
7475	HTAR0124	Male	Indian	2018	38	Single	No formal education	Private	Urban	Yes	Yes	Yes	No	No	No	No	No	No	No
7476	HTAR0125	Male	Indian	2019	50	Others	Secondary	Unemployed	Urban	Yes	Yes	Yes	No	No	No	No	No	No	No
7477	HTAR0126	Male	Malay	2020	23	Single	Secondary	Unemployed	Urban	Yes	No	No	Yes	Yes	No	No	No	No	No
7478	HTAR0127	Male	Malay	2018	34	Single	Primary	Self-employed	Urban	Yes	Yes	No	Yes	Yes	No	No	No	No	Yes
7479	HTAR0128	Male	Indian	2019	41	Married	No formal education	Private	Urban	Yes	No	Yes	No	No	No	No	No	No	No
7480	HTAR0132	Male	Malay	2018	54	Married	No formal education	Private	Urban	Yes	Yes	Yes	No	No	No	No	No	No	No
7481	HTAR0133	Male	Others	2020	25	Others	Secondary	Unemployed	Rural	Yes	Yes	No	No	Yes	No	No	No	No	No
7482	HTAR0134	Male	Malay	2020	33	Single	Secondary	Unemployed	Urban	Yes	Yes	Yes	Yes	Yes	No	No	No	No	No
7483	HTAR0135	Male	Malay	2018	54	Married	Tertiary	Unemployed	Urban	Yes	Yes	No	No	No	No	No	No	No	No
7484	HTAR0136	Male	Others	2020	49	Single	Primary	Unemployed	Urban	Yes	Yes	Yes	No	Yes	No	No	No	No	No
7485	HTAR0137	Male	Malay	2019	52	Married	Secondary	Government	Urban	Yes	Yes	Yes	No	No	No	No	No	No	Yes
7486	HTAR0139	Male	Malay	2020	40	Married	Secondary	Private	Urban	Yes	No	No	No	Yes	No	No	No	No	No
7487	HTAR0140	Male	Malay	2019	43	Married	Secondary	Private	Urban	Yes	Yes	No	Yes	Yes	No	No	No	No	No

No	Patient ID	Gender	Ethnic group	Year of diagnosis	Age	Marital status	Education level	Occupation	Place of residence	History of psy illness	Tobacco	Alcohol	Cannabis	ATS	Inhalant	Sedative	Hallucinogens	Opioids	Kratom
7488	HTAR0142	Female	Malay	2018	40	Single	Primary	Unemployed	Rural	Yes	No	No	No	Yes	No	No	No	Yes	No
7489	HTAR0149	Male	Malay	2019	42	Married	Tertiary	Government	Urban	Yes	Yes	No	No	Yes	No	No	No	No	No
7490	HTAR0150	Male	Malay	2020	30	Single	Primary	Self-employed	Urban	Yes	Yes	No	Yes	No	No	No	No	Yes	No
7491	HTAR0151	Male	Malay	2020	30	Others	Secondary	Private	Urban	Yes	No	No	No	No	No	Yes	No	No	No
7492	HTAR0152	Male	Others	2018	30	Others	No formal education	Others	Urban	Yes	No	No	No	Yes	No	No	No	No	No
7493	HTAR0153	Male	Malay	2019	26	Single	Secondary	Private	Urban	Yes	No	No	No	Yes	No	No	No	No	No
7494	HTAR0156	Male	Malay	2020	32	Others	Secondary	Unemployed	Urban	Yes	No	No	No	No	No	No	No	No	No
7495	HTAR0157	Female	Malay	2019	29	Single	No formal education	Others	Urban	Yes	No	No	No	Yes	No	No	No	No	No
7496	HTAR0159	Male	Indian	2018	27	Others	Secondary	Private	Urban	Yes	No	No	No	Yes	No	No	No	Yes	No
7497	HTAR0161	Male	Malay	2019	42	Others	Tertiary	Unemployed	Urban	Yes	Yes	No	No	No	No	No	No	Yes	No
7498	HTAR0162	Male	Malay	2018	24	Others	Secondary	Self-employed	Urban	Yes	Yes	No	No	Yes	No	No	No	No	No
7499	HTAR0165	Female	Malay	2018	25	Others	Tertiary	Others	Urban	Yes	No	Yes	Yes	No	No	No	No	No	No
7500	HTAR0166	Male	Chinese	2018	32	Single	Primary	Unemployed	Urban	Yes	No	No	No	Yes	No	No	No	No	No
7501	HTAR0169	Male	Malay	2019	28	Single	Secondary	Private	Urban	Yes	Yes	No	Yes	Yes	No	No	No	Yes	No
7502	HTAR0170	Male	Malay	2019	24	Single	Secondary	Private	Rural	Yes	Yes	No	No	Yes	No	No	No	No	No
7503	HTAR0171	Male	Indian	2018	42	Others	Primary	Self-employed	Rural	Yes	Yes	Yes	No	No	No	No	No	No	No
7504	HTAR0172	Male	Indian	2018	29	Married	Secondary	Private	Urban	Yes	Yes	Yes	No	No	No	No	No	No	No
7505	HTAR0173	Male	Indian	2018	62	Married	Primary	Others	Rural	Yes	Yes	Yes	No	No	No	No	No	No	No
7506	HTAR0175	Male	Malay	2018	33	Others	No formal education	Others	Urban	No	No	No	No	Yes	No	No	No	No	No
7507	HTAR0176	Male	Indian	2018	40	Others	Primary	Private	Urban	No	Yes	No	Yes	No	No	No	No	No	No
7508	HTAR0178	Male	Indian	2018	66	Married	Primary	Private	Urban	No	Yes	Yes	No	No	No	No	No	No	No

No	Patient ID	Gender	Ethnic group	Year of diagnosis	Age	Marital status	Education level	Occupation	Place of residence	History of psy illness	Tobacco	Alcohol	Cannabis	ATS	Inhalant	Sedative	Hallucinogens	Opioids	Kratom
7509	HTAR0179	Male	Indian	2018	44	Single	Secondary	Private	Urban	Yes	No	Yes	No	No	No	No	No	No	No
7510	HTAR0181	Male	Indian	2019	46	Single	Tertiary	Private	Urban	No	Yes	Yes	No	No	No	No	No	No	No
7511	HTAR0182	Male	Others	2019	35	Single	Secondary	Private	Urban	No	Yes	No	Yes	Yes	No	No	No	No	No
7512	HTAR0183	Male	Malay	2018	23	Others	Secondary	Private	Urban	Yes	Yes	No	No	No	No	No	No	No	No
7513	HTAR0184	Male	Malay	2019	26	Single	Secondary	Private	Urban	Yes	Yes	No	Yes	Yes	Yes	No	No	No	No
7514	HTAR0188	Male	Chinese	2018	37	Single	Secondary	Self-employed	Urban	No	Yes	No	No	Yes	No	No	No	No	No
7515	HTAR0190	Male	Indian	2020	32	Married	Secondary	Private	Urban	No	Yes	Yes	Yes	No	No	No	No	No	No
7516	HTAR0191	Male	Malay	2019	64	Married	Primary	Self-employed	Urban	Yes	Yes	No	No	No	No	Yes	No	No	No
7517	HTAR0192	Male	Malay	2018	27	Single	Secondary	Unemployed	Urban	No	Yes	No	No	Yes	No	No	No	Yes	No
7518	HTAR0194	Male	Malay	2018	26	Single	Secondary	Private	Urban	Yes	Yes	No	No	Yes	No	No	No	No	No
7519	HTAR0195	Male	Chinese	2018	61	Single	Secondary	Unemployed	Urban	No	Yes	No	No	No	No	No	No	Yes	No
7520	HTAR0197	Male	Malay	2020	21	Single	Secondary	Unemployed	Urban	No	No	No	Yes	No	No	No	No	No	No
7521	HTAR0198	Male	Indian	2018	35	Married	Secondary	Unemployed	Urban	No	No	No	No	Yes	No	No	No	No	No
7522	HTAR0199	Male	Malay	2019	37	Married	Tertiary	Private	Urban	No	No	Yes	No	Yes	No	No	No	No	No
7523	HTAR0201	Female	Malay	2019	26	Single	Tertiary	Private	Urban	No	Yes	No	Yes	No	No	Yes	No	No	No
7524	HTAR0203	Male	Indian	2019	38	Single	Secondary	Private	Urban	No	Yes	No	No	No	No	No	No	Yes	No
7525	HTAR0205	Male	Malay	2019	40	Married	Secondary	Private	Urban	No	Yes	No	No	Yes	No	No	No	No	No
7526	HTAR0207	Female	Malay	2018	33	Others	Tertiary	Unemployed	Urban	No	No	No	No	Yes	No	No	No	No	No
7527	HTAR0208	Male	Indian	2019	31	Single	Secondary	Private	Urban	Yes	No	No	No	No	No	No	No	Yes	No
7528	HTAR0209	Male	Indian	2020	50	Others	No formal education	Others	Urban	No	Yes	Yes	No	Yes	No	No	No	No	No
7529	HTAR0210	Male	Malay	2018	25	Single	Secondary	Unemployed	Urban	No	Yes	No	No	Yes	No	No	No	No	No

No	Patient ID	Gender	Ethnic group	Year of diagnosis	Age	Marital status	Education level	Occupation	Place of residence	History of psy illness	Tobacco	Alcohol	Cannabis	ATS	Inhalant	Sedative	Hallucinogens	Opioids	Kratom
7530	HTAR0211	Male	Malay	2018	32	Married	Secondary	Private	Urban	No	Yes	Yes	Yes	Yes	No	No	No	No	No
7531	HTAR0212	Male	Indian	2019	41	Single	Tertiary	Private	Urban	Yes	No	No	No	Yes	No	No	No	No	No
7532	HTAR0216	Male	Malay	2018	46	Married	Secondary	Private	Urban	Yes	Yes	Yes	Yes	Yes	No	No	No	Yes	No
7533	HTAR0217	Male	Indian	2019	46	Married	Secondary	Unemployed	Urban	No	No	No	No	Yes	No	No	No	Yes	No
7534	HTAR0220	Female	Others	2018	31	Others	No formal education	Private	Urban	No	Yes	Yes	No	Yes	No	No	No	No	No
7535	HTAR0221	Male	Indian	2018	46	Single	Primary	Private	Urban	No	Yes	Yes	Yes	Yes	No	No	No	No	No
7536	HTAR0222	Male	Malay	2019	40	Others	Secondary	Self-employed	Urban	Yes	Yes	No	No	Yes	No	No	No	No	No
7537	HTAR0225	Female	Malay	2018	38	Single	Tertiary	Private	Urban	No	Yes	Yes	No	Yes	No	No	No	No	No
7538	HTAR0227	Male	Malay	2020	25	Single	No formal education	Private	Urban	No	Yes	No	Yes	No	No	No	No	No	No
7539	HTAR0229	Female	Malay	2018	35	Others	Secondary	Private	Urban	No	Yes	No	No	Yes	No	No	No	No	No
7540	HTAR0231	Male	Malay	2018	41	Single	Secondary	Private	Urban	No	No	No	Yes	Yes	No	No	No	No	No
7541	HTAR0232	Male	Malay	2020	24	Single	Tertiary	Private	Urban	Yes	Yes	Yes	Yes	Yes	No	No	No	No	No
7542	HTAR0234	Male	Indian	2020	68	Married	Primary	Self-employed	Urban	No	Yes	Yes	No	No	No	No	No	No	No
7543	HTAR0235	Male	Indian	2020	40	Married	Primary	Private	Urban	No	No	Yes	Yes	Yes	No	No	No	No	No
7544	HTAR0236	Male	Malay	2018	42	Single	Primary	Self-employed	Urban	No	No	Yes	No	Yes	No	No	No	No	No
7545	HTAR0237	Male	Indian	2020	60	Married	Primary	Private	Urban	No	Yes	Yes	No	No	No	No	No	No	No
7546	HTAR0241	Male	Malay	2018	36	Others	Secondary	Private	Urban	Yes	No	No	No	Yes	No	No	No	No	No
7547	HTAR0242	Male	Indian	2018	67	Single	Secondary	Private	Urban	No	No	Yes	No	Yes	No	No	No	Yes	No
7548	HTAR0246	Male	Chinese	2018	38	Married	Tertiary	Self-employed	Urban	Yes	Yes	Yes	Yes	Yes	No	No	No	Yes	No
7549	HTAR0247	Male	Indian	2019	57	Married	Secondary	Self-employed	Urban	No	Yes	Yes	No	No	No	No	No	No	No
7550	HTAR0248	Male	Malay	2018	35	Married	Secondary	Self-employed	Urban	No	No	No	No	Yes	No	No	No	No	No

No	Patient ID	Gender	Ethnic group	Year of diagnosis	Age	Marital status	Education level	Occupation	Place of residence	History of psy illness	Tobacco	Alcohol	Cannabis	ATS	Inhalant	Sedative	Hallucinogens	Opioids	Kratom
7551	HTAR0249	Male	Indian	2019	45	Married	Tertiary	Private	Urban	No	No	Yes	No	No	No	No	No	No	No
7552	HTAR0250	Male	Others	2019	29	Single	Secondary	Unemployed	Urban	Yes	Yes	No	No	Yes	No	No	No	Yes	No
7553	HTAR0253	Male	Indian	2020	55	Others	No formal education	Others	Urban	No	Yes	No	No	Yes	No	No	No	Yes	No
7554	HTAR0255	Female	Malay	2019	21	Others	Secondary	Private	Urban	Yes	Yes	No	No	Yes	No	No	No	No	No
7555	HTAR0256	Male	Indian	2018	45	Married	Tertiary	Private	Urban	Yes	No	No	Yes	No	No	No	No	No	No
7556	HTAR0257	Male	Malay	2018	38	Others	Secondary	Government	Urban	Yes	Yes	No	No	Yes	No	No	No	No	No
7557	HTAR0261	Male	Malay	2020	30	Others	Secondary	Private	Urban	Yes	Yes	No	No	Yes	No	No	No	No	No
7558	HTAR0262	Male	Chinese	2018	27	Single	Tertiary	Unemployed	Urban	Yes	Yes	No	No	Yes	No	No	No	No	No
7559	HTAR0263	Male	Others	2018	24	Single	Secondary	Unemployed	Urban	Yes	No	No	No	Yes	No	No	No	No	No
7560	HTAR0264	Male	Malay	2020	47	Married	Secondary	Self-employed	Urban	Yes	No	No	Yes	Yes	No	No	No	No	No
7561	HTAR0265	Female	Malay	2018	30	Others	Secondary	Private	Urban	No	No	No	Yes	Yes	No	No	No	No	No
7562	HTAR0267	Male	Indian	2019	55	Married	Secondary	Unemployed	Urban	Yes	No	Yes	No	No	No	No	No	No	No
7563	HTAR0268	Male	Chinese	2019	49	Others	Secondary	Unemployed	Urban	Yes	No	No	No	No	No	No	No	Yes	No
7564	HTAR0269	Male	Malay	2020	36	Single	Secondary	Unemployed	Urban	Yes	No	No	No	Yes	No	No	No	No	No
7565	HTAR0271	Male	Others	2019	37	Single	Secondary	Unemployed	Urban	Yes	Yes	No	No	Yes	No	No	No	Yes	No
7566	HTAR0273	Male	Indian	2018	51	Married	Primary	Unemployed	Urban	Yes	No	Yes	No	No	No	No	No	No	No
7567	HTAR0275	Female	Malay	2018	31	Others	Secondary	Private	Urban	Yes	Yes	No	No	Yes	No	No	No	No	No
7568	HTAR0276	Male	Indian	2018	60	Married	Primary	Unemployed	Urban	Yes	No	Yes	No	No	No	No	No	No	No
7569	HTAR0276	Male	Indian	2018	60	Married	Secondary	Unemployed	Urban	Yes	No	Yes	No	No	No	No	No	No	No
7570	HTAR0277	Male	Malay	2020	42	Others	No formal education	Unemployed	Urban	Yes	Yes	No	No	Yes	No	No	No	Yes	No
7571	HTAR0279	Male	Malay	2019	40	Married	Primary	Private	Urban	Yes	No	No	No	No	No	No	No	Yes	No

No	Patient ID	Gender	Ethnic group	Year of diagnosis	Age	Marital status	Education level	Occupation	Place of residence	History of psy illness	Tobacco	Alcohol	Cannabis	ATS	Inhalant	Sedative	Hallucinogens	Opioids	Kratom
7572	HTAR0280	Female	Malay	2018	36	Single	Tertiary	Unemployed	Urban	Yes	No	Yes	No	No	No	No	No	No	No
7573	HTAR0283	Female	Malay	2018	35	Others	Secondary	Private	Urban	Yes	No	No	No	Yes	No	No	No	No	No
7574	HTAR0285	Male	Malay	2020	40	Married	Secondary	Private	Urban	Yes	Yes	No	No	Yes	No	No	No	No	No
7575	HTAR0287	Male	Malay	2018	25	Single	Secondary	Private	Urban	Yes	No	No	Yes	No	No	No	No	No	No
7576	HTAR0289	Male	Malay	2019	27	Single	Secondary	Private	Urban	Yes	Yes	No	No	No	No	No	No	No	Yes
7577	HTAR0291	Male	Chinese	2019	24	Single	Tertiary	Unemployed	Urban	Yes	No	No	Yes	No	No	No	No	No	No
7578	HTAR0292	Male	Malay	2020	26	Others	Secondary	Private	Urban	Yes	Yes	No	Yes	No	No	No	No	No	Yes
7579	HTAR0293	Male	Indian	2019	46	Others	Secondary	Unemployed	Urban	Yes	No	Yes	No	No	No	No	No	No	No
7580	HTAR0295	Male	Chinese	2019	23	Single	Secondary	Unemployed	Urban	Yes	Yes	Yes	Yes	Yes	No	No	No	No	No
7581	HTAR0298	Male	Others	2019	25	Single	Tertiary	Private	Urban	Yes	Yes	Yes	Yes	Yes	No	No	No	No	No
7582	HTAR0300	Male	Chinese	2018	72	Married	Secondary	Others	Urban	No	Yes	Yes	No	No	No	No	No	No	No
7583	HTAR0301	Male	Malay	2019	19	Single	Secondary	Unemployed	Urban	No	Yes	No	No	Yes	No	No	No	No	No
7584	HTAR0302	Female	Malay	2019	25	Others	Tertiary	Private	Urban	Yes	Yes	Yes	Yes	No	No	No	No	No	No
7585	HTAR0303	Male	Malay	2019	34	Single	Secondary	Private	Urban	Yes	Yes	No	No	Yes	No	No	No	No	No
7586	HTAR0304	Male	Malay	2018	25	Single	Secondary	Unemployed	Urban	No	Yes	No	No	No	No	No	No	No	Yes
7587	HTAR0305	Male	Indian	2018	31	Single	Secondary	Private	Urban	No	No	No	No	Yes	No	No	No	No	Yes
7588	HTAR0307	Male	Indian	2018	47	Single	Primary	Private	Urban	No	Yes	Yes	No	No	No	No	No	No	No
7589	HTAR0308	Male	Malay	2018	30	Others	Primary	Private	Urban	No	Yes	No	No	Yes	No	No	No	No	No
7590	HTAR0310	Male	Indian	2019	61	Married	Tertiary	Self-employed	Urban	Yes	No	Yes	No	No	No	No	No	No	No
7591	HTAR0311	Male	Indian	2018	47	Married	Secondary	Private	Urban	Yes	No	Yes	No	No	No	No	No	No	No
7592	HTAR0312	Male	Malay	2020	21	Single	Secondary	Private	Urban	No	No	No	No	Yes	No	No	No	No	Yes

No	Patient ID	Gender	Ethnic group	Year of diagnosis	Age	Marital status	Education level	Occupation	Place of residence	History of psy illness	Tobacco	Alcohol	Cannabis	ATS	Inhalant	Sedative	Hallucinogens	Opioids	Kratom
7593	HTAR0315	Male	Indian	2018	61	Married	No formal education	Unemployed	Urban	No	No	Yes	No	No	No	No	No	No	No
7594	HTAR0316	Male	Chinese	2018	26	Single	Secondary	Private	Urban	No	Yes	Yes	No	Yes	No	No	No	No	No
7595	HTAR0317	Male	Chinese	2018	43	Married	Primary	Self-employed	Urban	No	Yes	Yes	No	Yes	No	No	No	No	No
7596	HTAR0318	Male	Indian	2018	55	Married	Primary	Private	Urban	No	No	Yes	No	Yes	No	No	No	No	No
7597	HTAR0319	Male	Indian	2018	31	Married	No formal education	Private	Urban	No	Yes	Yes	No	No	No	No	No	No	No
7598	HTAR0321	Female	Malay	2019	31	Married	Tertiary	Others	Urban	No	Yes	No	No	No	No	No	No	Yes	No
7599	HTAR0322	Male	Malay	2018	41	Married	Secondary	Private	Urban	No	Yes	Yes	No	Yes	No	No	No	Yes	No
7600	HTAR0323	Male	Malay	2019	40	Married	Secondary	Private	Urban	No	Yes	No	Yes	Yes	No	No	No	No	No
7601	HTAR0327	Male	Indian	2018	26	Single	Primary	Unemployed	Urban	No	No	Yes	No	No	No	No	No	No	No
7602	HTAR0329	Male	Others	2019	28	Married	Secondary	Private	Urban	No	No	No	No	Yes	No	No	No	Yes	No
7603	HTAR0332	Male	Indian	2019	43	Single	Secondary	Private	Urban	No	Yes	Yes	No	No	No	No	No	No	No
7604	HTAR0333	Male	Indian	2018	33	Single	Secondary	Private	Urban	No	No	Yes	No	No	No	No	No	No	No
7605	HTAR0335	Male	Indian	2018	32	Others	Primary	Private	Urban	No	Yes	Yes	No	No	No	No	No	No	No
7606	HTAR0338	Male	Indian	2018	33	Single	Secondary	Self-employed	Urban	No	Yes	Yes	No	No	No	No	No	Yes	No
7607	HTAR0339	Male	Indian	2018	42	Others	Tertiary	Self-employed	Urban	No	No	Yes	No	No	No	No	No	No	No
7608	HTAR0340	Male	Chinese	2018	58	Single	Secondary	Unemployed	Urban	No	Yes	Yes	No	No	No	No	No	No	No
7609	HTAR0341	Female	Chinese	2018	28	Others	Tertiary	Private	Urban	No	No	No	No	Yes	No	No	No	No	No
7610	HTAR0342	Male	Malay	2018	20	Single	Secondary	Unemployed	Urban	No	No	No	Yes	Yes	No	No	No	No	No
7611	HTAR0343	Male	Indian	2018	41	Single	Secondary	Private	Urban	No	Yes	Yes	No	No	No	No	No	No	No
7612	HTAR0344	Male	Malay	2018	27	Single	Tertiary	Private	Urban	No	Yes	No	No	Yes	No	No	No	No	No
7613	HTAR0345	Male	Indian	2018	32	Single	Tertiary	Private	Urban	No	Yes	Yes	Yes	No	No	No	No	No	No

No	Patient ID	Gender	Ethnic group	Year of diagnosis	Age	Marital status	Education level	Occupation	Place of residence	History of psy illness	Tobacco	Alcohol	Cannabis	ATS	Inhalant	Sedative	Hallucinogens	Opioids	Kratom
7614	HTAR0346	Male	Malay	2018	33	Single	Tertiary	Private	Urban	Yes	Yes	No	No	No	No	No	No	No	No
7615	HTAR0347	Male	Malay	2019	41	Single	Secondary	Private	Urban	No	Yes	No	No	Yes	No	No	No	Yes	No
7616	HTAR0348	Male	Indian	2019	48	Others	Secondary	Private	Urban	No	Yes	No	Yes	Yes	No	No	No	No	No
7617	HTAR0351	Male	Chinese	2018	27	Single	Tertiary	Unemployed	Urban	Yes	Yes	Yes	No	Yes	No	No	No	No	No
7618	HTAR0352	Male	Malay	2018	35	Single	Secondary	Self-employed	Urban	Yes	Yes	No	No	Yes	Yes	No	No	Yes	No
7619	HTAR0353	Male	Indian	2018	62	Married	Secondary	Private	Urban	Yes	Yes	Yes	No	No	No	No	No	No	No
7620	HTAR0355	Male	Malay	2019	30	Married	Secondary	Self-employed	Urban	Yes	Yes	No	Yes	Yes	No	No	No	Yes	No
7621	HTAR0357	Male	Indian	2018	39	Single	Secondary	Private	Urban	Yes	No	Yes	No	No	No	No	No	No	No
7622	HTAR0358	Male	Malay	2020	28	Single	Tertiary	Private	Urban	Yes	No	No	No	Yes	No	No	No	No	No
7623	HTAR0361	Male	Malay	2019	25	Others	Secondary	Private	Urban	Yes	Yes	No	Yes	No	No	No	No	No	No
7624	HTAR0362	Female	Indian	2018	25	Single	Secondary	Others	Urban	Yes	Yes	No	No	Yes	No	No	No	No	No
7625	HTAR0363	Male	Indian	2018	33	Others	Secondary	Private	Urban	Yes	Yes	No	No	Yes	No	No	No	No	No
7626	HTAR0364	Male	Malay	2018	31	Single	Secondary	Private	Urban	Yes	Yes	No	No	Yes	No	No	No	No	No
7627	HTAR0365	Female	Others	2018	35	Others	Secondary	Unemployed	Urban	Yes	Yes	Yes	Yes	Yes	No	No	No	Yes	No
7628	HTAR0366	Male	Malay	2020	20	Others	Secondary	Unemployed	Urban	Yes	No	No	Yes	No	No	No	No	No	No
7629	HTAR0367	Male	Others	2018	27	Others	Secondary	Private	Urban	Yes	Yes	No	No	Yes	No	No	No	No	No
7630	HTAR0368	Male	Malay	2020	54	Others	No formal education	Unemployed	Urban	Yes	No	No	No	No	No	No	No	No	Yes
7631	HTAR0373	Female	Indian	2018	39	Others	Secondary	Self-employed	Urban	Yes	Yes	Yes	No	Yes	No	No	No	No	No
7632	HTAR0377	Male	Malay	2018	23	Single	Tertiary	Unemployed	Urban	Yes	No	No	Yes	No	No	No	No	No	No
7633	HTAR0379	Male	Others	2018	42	Others	No formal education	Private	Urban	Yes	Yes	Yes	No	No	No	No	No	No	No
7634	HTAR0380	Female	Malay	2018	39	Others	Secondary	Private	Urban	Yes	No	No	No	Yes	No	No	No	No	No

No	Patient ID	Gender	Ethnic group	Year of diagnosis	Age	Marital status	Education level	Occupation	Place of residence	History of psy illness	Tobacco	Alcohol	Cannabis	ATS	Inhalant	Sedative	Hallucinogens	Opioids	Kratom
7635	HTAR0381	Male	Indian	2020	55	Married	Secondary	Self-employed	Urban	Yes	Yes	Yes	No	No	No	No	No	No	No
7636	HTAR0383	Male	Malay	2018	41	Others	Secondary	Private	Urban	Yes	No	No	No	Yes	No	No	No	Yes	No
7637	HTAR0384	Male	Malay	2019	38	Single	Secondary	Private	Urban	Yes	Yes	No	Yes	Yes	No	No	No	No	No
7638	HTAR0385	Female	Malay	2020	32	Married	Secondary	Private	Urban	Yes	Yes	No	No	Yes	No	No	No	No	No
7639	HTAR0387	Female	Indian	2018	39	Married	Secondary	Others	Urban	Yes	No	No	No	No	No	No	No	Yes	No
7640	HTAR0388	Male	Indian	2019	37	Single	Tertiary	Self-employed	Urban	Yes	Yes	No	Yes	Yes	No	No	No	No	No
7641	HTAR0390	Male	Indian	2018	30	Single	Secondary	Private	Urban	Yes	No	Yes	No	Yes	No	No	No	No	No
7642	HTAR0392	Male	Chinese	2018	30	Single	Secondary	Private	Urban	Yes	No	Yes	No	No	No	No	No	Yes	No
7643	HTAR0393	Male	Indian	2020	42	Married	Secondary	Private	Urban	Yes	No	Yes	No	No	No	No	No	No	No
7644	HTAR0394	Male	Malay	2018	34	Married	Secondary	Others	Urban	Yes	Yes	Yes	No	Yes	No	No	No	No	No
7645	HTAR0395	Male	Malay	2018	53	Married	Secondary	Self-employed	Urban	Yes	Yes	No	No	Yes	No	Yes	No	No	No
7646	HTAR0397	Male	Indian	2020	23	Single	Secondary	Unemployed	Urban	Yes	Yes	Yes	Yes	No	No	No	No	No	No
7647	HTAR0398	Female	Malay	2019	39	Others	Secondary	Private	Urban	Yes	No	No	No	Yes	No	No	No	No	No
7648	HTAR0399	Male	Indian	2019	53	Married	Secondary	Private	Urban	Yes	No	Yes	No	No	No	No	No	No	No
7649	HTAR0400	Male	Indian	2018	42	Married	Secondary	Private	Urban	No	Yes	Yes	Yes	Yes	No	No	No	No	No
7650	HTAR0401	Male	Chinese	2020	41	Married	Secondary	Unemployed	Urban	No	No	No	No	Yes	No	No	No	No	No
7651	HTAR0402	Male	Indian	2020	38	Married	Primary	Private	Urban	No	No	No	No	Yes	No	No	No	No	No
7652	HTAR0403	Male	Others	2019	44	Married	Secondary	Unemployed	Urban	No	Yes	Yes	No	No	No	No	No	No	No
7653	HTAR0404	Male	Malay	2020	36	Single	Secondary	Private	Urban	No	Yes	No	No	Yes	No	No	No	No	No
7654	HTAR0407	Male	Indian	2019	42	Others	No formal education	Others	Urban	No	Yes	No	No	No	No	No	No	Yes	No
7655	HTAR0408	Male	Malay	2019	38	Single	Secondary	Unemployed	Urban	No	Yes	No	No	No	No	No	No	No	No

No	Patient ID	Gender	Ethnic group	Year of diagnosis	Age	Marital status	Education level	Occupation	Place of residence	History of psy illness	Tobacco	Alcohol	Cannabis	ATS	Inhalant	Sedative	Hallucinogens	Opioids	Kratom
7656	HTAR0410	Male	Malay	2019	23	Others	Secondary	Others	Urban	No	No	No	No	Yes	No	No	No	No	No
7657	HTAR0411	Male	Indian	2018	36	Married	Secondary	Unemployed	Urban	No	No	Yes	No	No	No	No	No	No	No
7658	HTAR0413	Female	Malay	2018	28	Others	Secondary	Unemployed	Urban	No	Yes	Yes	No	Yes	No	No	No	Yes	No
7659	HTAR0416	Male	Indian	2019	28	Single	Secondary	Unemployed	Urban	No	No	Yes	No	No	No	No	No	No	No
7660	HTAR0417	Female	Chinese	2019	33	Single	No formal education	Self-employed	Urban	No	Yes	Yes	Yes	Yes	No	No	No	No	No
7661	HTAR0418	Male	Indian	2019	32	Married	Secondary	Private	Urban	No	Yes	Yes	No	Yes	No	No	No	No	No
7662	HTAR0419	Male	Malay	2019	22	Single	Secondary	Unemployed	Urban	No	Yes	No	No	Yes	No	No	No	No	No
7663	HTAR0420	Male	Indian	2018	44	Married	Tertiary	Private	Urban	No	No	No	No	Yes	No	No	No	No	No
7664	HTAR0421	Male	Malay	2018	29	Single	Tertiary	Unemployed	Urban	Yes	Yes	No	No	Yes	No	No	No	No	No
7665	HTAR0424	Male	Others	2018	31	Others	Secondary	Private	Urban	No	Yes	Yes	No	Yes	No	No	No	No	No
7666	HTAR0425	Male	Indian	2018	38	Married	Secondary	Private	Urban	No	Yes	Yes	No	Yes	No	No	No	No	No
7667	HTAR0427	Male	Malay	2019	30	Single	Secondary	Unemployed	Urban	No	Yes	No	Yes	Yes	No	No	No	No	No
7668	HTAR0430	Male	Indian	2019	57	Married	Primary	Unemployed	Urban	No	Yes	Yes	No	No	No	No	No	No	No
7669	HTAR0432	Male	Malay	2018	27	Single	Secondary	Unemployed	Urban	No	Yes	No	No	Yes	No	No	No	Yes	No
7670	HTAR0434	Male	Chinese	2019	63	Single	No formal education	Unemployed	Urban	No	Yes	No	No	No	No	No	No	Yes	No
7671	HTAR0435	Male	Indian	2020	42	Married	Secondary	Private	Urban	No	Yes	Yes	No	No	No	No	No	No	No
7672	HTAR0437	Male	Malay	2020	36	Others	No formal education	Others	Urban	Yes	No	No	No	Yes	No	No	No	No	No
7673	HTAR0439	Female	Malay	2019	21	Others	Secondary	Others	Urban	No	No	No	No	Yes	No	No	No	No	No
7674	HTAR0441	Male	Others	2019	31	Others	Secondary	Unemployed	Urban	Yes	Yes	Yes	No	Yes	No	No	No	No	No
7675	HTAR0442	Male	Malay	2018	45	Married	Secondary	Private	Urban	Yes	Yes	No	No	Yes	No	No	No	Yes	No
7676	HTAR0443	Male	Indian	2018	53	Others	Secondary	Unemployed	Urban	Yes	Yes	Yes	No	No	No	No	No	No	No

No	Patient ID	Gender	Ethnic group	Year of diagnosis	Age	Marital status	Education level	Occupation	Place of residence	History of psy illness	Tobacco	Alcohol	Cannabis	ATS	Inhalant	Sedative	Hallucinogens	Opioids	Kratom
7677	HTAR0445	Male	Indian	2018	57	Married	Primary	Unemployed	Urban	Yes	Yes	Yes	No	No	No	No	No	No	No
7678	HTAR0446	Male	Indian	2020	39	Others	Secondary	Unemployed	Urban	Yes	No	No	No	Yes	No	No	No	No	No
7679	HTAR0448	Male	Malay	2018	33	Married	Secondary	Private	Urban	Yes	Yes	No	No	Yes	No	No	No	No	No
7680	HTAR0449	Male	Indian	2020	39	Married	Secondary	Self-employed	Urban	Yes	Yes	Yes	No	No	No	No	No	No	No
7681	HTAR0451	Male	Malay	2018	28	Others	Secondary	Self-employed	Urban	Yes	Yes	No	Yes	Yes	No	No	No	No	No
7682	HTAR0452	Male	Indian	2019	26	Single	Secondary	Unemployed	Urban	Yes	Yes	Yes	No	Yes	No	No	No	No	No
7683	HTAR0456	Male	Malay	2019	32	Single	Secondary	Private	Urban	Yes	Yes	No	Yes	Yes	No	No	No	No	No
7684	HTAR0458	Male	Indian	2019	34	Married	Tertiary	Private	Urban	Yes	Yes	Yes	Yes	Yes	No	No	No	No	No
7685	HTAR0460	Male	Malay	2019	46	Others	Secondary	Unemployed	Urban	Yes	No	No	No	No	No	No	No	Yes	No
7686	HTAR0463	Male	Malay	2018	29	Single	Secondary	Private	Urban	Yes	Yes	Yes	Yes	Yes	No	No	No	No	No
7687	HTAR0464	Male	Indian	2019	45	Married	Secondary	Private	Urban	Yes	Yes	Yes	No	No	No	No	No	No	No
7688	HTAR0467	Female	Indian	2018	47	Married	Tertiary	Unemployed	Urban	Yes	No	No	No	Yes	No	No	No	No	No
7689	HTAR0470	Male	Indian	2020	46	Others	Secondary	Private	Urban	Yes	Yes	Yes	No	No	No	No	No	No	No
7690	HTAR0472	Male	Indian	2018	60	Others	No formal education	Others	Urban	Yes	No	Yes	No	No	No	No	No	No	No
7691	HTAR0473	Male	Indian	2018	42	Others	No formal education	Others	Urban	Yes	No	Yes	No	No	No	No	No	No	No
7692	HTAR0474	Male	Indian	2019	52	Married	Tertiary	Private	Urban	Yes	Yes	Yes	No	No	No	No	No	No	No
7693	HTAR0478	Male	Chinese	2019	34	Single	Secondary	Private	Urban	Yes	Yes	Yes	No	No	No	No	No	No	No
7694	HTAR0481	Male	Malay	2018	30	Single	Tertiary	Private	Urban	Yes	Yes	No	No	Yes	No	No	No	No	No
7695	HTAR0483	Male	Indian	2018	47	Married	Primary	Self-employed	Urban	Yes	Yes	Yes	No	No	No	No	No	No	No
7696	HTAR0484	Male	Indian	2020	27	Others	Secondary	Self-employed	Urban	Yes	Yes	Yes	No	No	No	No	No	No	No
7697	HTAR0485	Male	Indian	2020	49	Married	Primary	Private	Urban	No	Yes	Yes	No	No	No	No	No	No	No

No	Patient ID	Gender	Ethnic group	Year of diagnosis	Age	Marital status	Education level	Occupation	Place of residence	History of psy illness	Tobacco	Alcohol	Cannabis	ATS	Inhalant	Sedative	Hallucinogens	Opioids	Kratom
7698	HTAR0486	Male	Indian	2019	37	Single	No formal education	Private	Urban	Yes	Yes	Yes	No	No	No	No	No	No	No
7699	HTAR0487	Male	Indian	2020	43	Married	Secondary	Unemployed	Urban	No	No	Yes	No	No	No	No	No	No	No
7700	HTAR0490	Male	Indian	2018	49	Married	Secondary	Private	Urban	Yes	Yes	Yes	No	No	No	No	No	No	No
7701	HTAR0491	Male	Indian	2019	52	Married	Secondary	Private	Urban	Yes	No	Yes	No	No	No	No	No	No	No
7702	HTAR0494	Female	Malay	2019	36	Single	Secondary	Private	Urban	Yes	Yes	No	No	Yes	No	No	No	No	No
7703	HTAR0495	Male	Malay	2021	28	Married	Tertiary	Private	Urban	Yes	Yes	No	Yes	No	No	No	No	No	No
7704	HTAR0497	Male	Indian	2018	36	Married	Tertiary	Private	Urban	Yes	Yes	Yes	No	No	No	No	No	No	No
7705	HTAR0499	Male	Malay	2021	31	Married	Secondary	Private	Urban	Yes	Yes	No	Yes	No	No	No	No	No	Yes
7706	HTAR0501	Male	Indian	2018	49	Others	No formal education	Self-employed	Urban	Yes	Yes	Yes	Yes	No	No	No	No	No	No
7707	HTAR0502	Male	Malay	2019	37	Others	Secondary	Others	Urban	Yes	Yes	No	No	Yes	No	No	No	No	No
7708	HTAR0505	Male	Indian	2020	34	Single	Tertiary	Private	Urban	Yes	Yes	Yes	No	No	No	No	No	No	No
7709	HTAR0506	Male	Indian	2018	47	Others	Tertiary	Private	Urban	Yes	Yes	Yes	No	No	No	No	No	No	No
7710	HTAR0507	Male	Malay	2018	24	Single	Tertiary	Unemployed	Urban	Yes	Yes	No	Yes	No	No	No	No	No	No
7711	HTAR0508	Male	Malay	2021	29	Married	Tertiary	Private	Urban	Yes	No	No	Yes	Yes	No	No	No	No	No
7712	HTAR0509	Male	Malay	2020	31	Single	Tertiary	Unemployed	Urban	Yes	Yes	No	Yes	No	No	No	No	No	No
7713	HTAR0510	Male	Malay	2021	30	Single	Tertiary	Self-employed	Urban	Yes	No	No	No	Yes	No	No	No	No	No
7714	HTAR0511	Male	Indian	2021	45	Others	Tertiary	Unemployed	Urban	Yes	Yes	Yes	No	No	No	No	No	No	No
7715	HTAR0512	Female	Others	2021	35	Others	No formal education	Private	Urban	Yes	No	No	No	Yes	No	No	No	No	No
7716	HTAR0513	Male	Indian	2021	32	Single	Secondary	Unemployed	Urban	Yes	No	Yes	No	No	No	No	No	No	No
7717	HTAR0514	Male	Indian	2021	38	Others	Secondary	Private	Urban	Yes	Yes	Yes	No	No	No	No	No	No	No
7718	HTAR0515	Male	Chinese	2021	38	Single	Primary	Unemployed	Urban	Yes	No	No	Yes	No	No	No	No	No	No

No	Patient ID	Gender	Ethnic group	Year of diagnosis	Age	Marital status	Education level	Occupation	Place of residence	History of psy illness	Tobacco	Alcohol	Cannabis	ATS	Inhalant	Sedative	Hallucinogens	Opioids	Kratom
7719	HTAR0516	Male	Indian	2021	37	Married	Secondary	Private	Urban	Yes	Yes	Yes	Yes	Yes	No	No	No	No	No
7720	HTAR0517	Female	Malay	2021	38	Others	Secondary	Unemployed	Urban	Yes	Yes	Yes	No	Yes	No	No	No	No	No
7721	HTAR0518	Male	Chinese	2021	44	Married	No formal education	Private	Urban	Yes	No	Yes	No	No	No	No	No	No	No
7722	HTAR0519	Male	Malay	2021	28	Single	Secondary	Self-employed	Urban	Yes	Yes	No	No	Yes	No	No	No	No	No
7723	HTAR0520	Male	Chinese	2020	38	Married	Secondary	Unemployed	Urban	Yes	No	No	Yes	Yes	No	No	No	No	No
7724	HTAR0521	Male	Indian	2021	40	Single	Secondary	Others	Urban	Yes	Yes	Yes	No	No	No	No	No	No	No
7725	HTAR0522	Male	Malay	2021	45	Others	Secondary	Unemployed	Urban	Yes	Yes	Yes	Yes	Yes	No	No	No	No	No
7726	HTAR0523	Male	Chinese	2018	39	Single	Primary	Self-employed	Urban	No	No	Yes	No	No	No	No	No	No	No
7727	HTAR0524	Male	Malay	2018	24	Single	Secondary	Unemployed	Urban	Yes	Yes	No	Yes	Yes	No	No	No	No	No
7728	HTAR0525	Male	Malay	2020	24	Single	Secondary	Private	Urban	Yes	No	No	Yes	Yes	No	No	No	No	No
7729	HTAR0526	Male	Indian	2020	32	Married	Tertiary	Private	Urban	Yes	Yes	Yes	No	No	No	No	No	No	No
7730	HTAR0527	Female	Indian	2020	35	Others	No formal education	Unemployed	Urban	Yes	Yes	Yes	No	No	No	No	No	No	No
7731	HTAR0528	Male	Indian	2020	49	Married	Secondary	Others	Urban	Yes	Yes	Yes	No	No	No	No	No	No	No
7732	HTAR0529	Male	Indian	2021	46	Married	No formal education	Unemployed	Urban	Yes	No	Yes	No	No	No	No	No	No	No
7733	HTAR0530	Male	Indian	2021	37	Married	Secondary	Private	Urban	Yes	Yes	Yes	No	Yes	No	No	No	No	No
7734	HTAR0531	Male	Malay	2019	36	Married	Secondary	Unemployed	Urban	Yes	Yes	No	No	Yes	No	No	No	No	No
7735	HTAR0532	Male	Indian	2021	46	Single	Secondary	Others	Urban	Yes	Yes	Yes	No	No	No	No	No	No	No
7736	HTAR0533	Male	Malay	2021	45	Single	Secondary	Unemployed	Urban	Yes	Yes	No	Yes	Yes	No	No	No	No	No
7737	HTAR0535	Male	Malay	2021	28	Married	Tertiary	Private	Urban	Yes	Yes	No	Yes	No	No	No	No	No	No
7738	HTAR0536	Female	Others	2021	32	Married	Secondary	Private	Urban	Yes	Yes	No	No	Yes	No	No	No	No	No
7739	HTAR0537	Male	Malay	2021	35	Married	Secondary	Private	Urban	Yes	Yes	No	Yes	Yes	No	No	No	No	No

No	Patient ID	Gender	Ethnic group	Year of diagnosis	Age	Marital status	Education level	Occupation	Place of residence	History of psy illness	Tobacco	Alcohol	Cannabis	ATS	Inhalant	Sedative	Hallucinogens	Opioids	Kratom
7740	HTAR0538	Male	Others	2021	37	Married	No formal education	Private	Urban	No	No	Yes	No	No	No	No	No	No	No
7741	HTAR0539	Female	Malay	2020	39	Others	No formal education	Private	Urban	Yes	No	No	Yes	No	No	No	No	No	No
7742	HTAR0540	Male	Chinese	2020	44	Single	Secondary	Private	Urban	Yes	No	No	No	Yes	No	No	No	Yes	No
7743	HTAR0541	Male	Malay	2021	20	Single	Secondary	Others	Urban	No	Yes	No	No	Yes	No	No	No	No	No
7744	HTAR0542	Male	Chinese	2020	50	Others	Tertiary	Unemployed	Urban	Yes	Yes	No	No	Yes	No	No	No	No	No
7745	HTAR0543	Female	Indian	2020	32	Single	Tertiary	Private	Urban	Yes	Yes	Yes	Yes	Yes	No	No	No	No	No
7746	HTAR0544	Male	Malay	2019	42	Married	Tertiary	Private	Urban	Yes	Yes	No	No	Yes	No	No	No	No	No
7747	HTAR0545	Male	Malay	2021	22	Single	Tertiary	Unemployed	Urban	Yes	Yes	No	Yes	No	No	No	No	No	No
7748	HTAR0547	Male	Indian	2021	43	Others	Secondary	Private	Urban	Yes	Yes	Yes	No	No	No	No	No	No	No
7749	HTAR0548	Male	Malay	2021	31	Single	Tertiary	Private	Urban	Yes	Yes	No	Yes	No	No	No	No	No	No
7750	HTAR0549	Male	Malay	2021	25	Single	Secondary	Self-employed	Urban	Yes	Yes	No	Yes	Yes	No	No	No	No	No
7751	HTAR0550	Male	Malay	2021	35	Others	Secondary	Private	Urban	Yes	Yes	No	Yes	Yes	No	No	No	No	No
7752	HTAR0551	Male	Malay	2020	36	Single	Secondary	Unemployed	Urban	Yes	Yes	No	No	Yes	No	No	No	No	No
7753	HTAR0552	Male	Malay	2021	28	Single	Secondary	Unemployed	Urban	Yes	Yes	Yes	No	No	No	No	No	No	No
7754	HTAR0553	Male	Indian	2021	69	Married	Tertiary	Others	Urban	Yes	No	Yes	No	No	No	No	No	No	No
7755	HTAR0554	Male	Malay	2021	30	Married	Secondary	Unemployed	Urban	Yes	Yes	Yes	No	Yes	No	No	No	No	No
7756	HTAR0555	Female	Malay	2021	45	Married	Secondary	Unemployed	Urban	Yes	No	No	No	Yes	No	No	No	No	No
7757	HTAR0556	Male	Indian	2021	38	Married	Tertiary	Unemployed	Urban	Yes	No	Yes	No	No	No	No	No	No	No
7758	HTAR0558	Female	Malay	2021	21	Single	Tertiary	Unemployed	Urban	Yes	No	No	No	Yes	No	No	No	No	No
7759	HTAR0560	Male	Malay	2021	26	Single	Secondary	Unemployed	Urban	Yes	Yes	No	No	Yes	No	No	No	No	No
7760	HTAR0561	Female	Malay	2021	43	Others	Secondary	Unemployed	Urban	Yes	Yes	No	No	Yes	No	No	No	No	No

No	Patient ID	Gender	Ethnic group	Year of diagnosis	Age	Marital status	Education level	Occupation	Place of residence	History of psy illness	Tobacco	Alcohol	Cannabis	ATS	Inhalant	Sedative	Hallucinogens	Opioids	Kratom
7761	HTAR0562	Female	Chinese	2021	55	Married	No formal education	Others	Urban	Yes	No	No	No	No	No	Yes	No	No	No
7762	HTAR0563	Male	Indian	2021	38	Others	Tertiary	Unemployed	Urban	Yes	No	No	Yes	Yes	No	No	No	No	No
7763	HTAR0564	Male	Malay	2021	29	Single	Secondary	Unemployed	Urban	Yes	Yes	No	No	Yes	No	No	No	No	No
7764	HTAR0567	Male	Indian	2020	51	Married	Primary	Private	Urban	Yes	No	Yes	No	No	No	No	No	No	No
7765	HTAR0568	Female	Indian	2021	24	Single	Secondary	Unemployed	Urban	Yes	No	No	Yes	Yes	No	No	No	No	No
7766	HTAR0569	Male	Chinese	2020	41	Single	No formal education	Unemployed	Urban	Yes	No	Yes	Yes	Yes	No	No	No	No	No
7767	HTAR0570	Male	Indian	2021	24	Single	Secondary	Unemployed	Urban	Yes	Yes	Yes	Yes	Yes	No	No	No	No	No
7768	HTAR0571	Male	Indian	2021	39	Others	No formal education	Unemployed	Urban	Yes	No	Yes	No	No	No	No	No	No	No
7769	HTAR0572	Male	Indian	2021	39	Others	Secondary	Private	Urban	Yes	Yes	Yes	No	No	No	No	No	No	No
7770	HTAR0573	Male	Indian	2021	20	Single	Tertiary	Unemployed	Urban	Yes	Yes	No	Yes	No	No	No	No	No	No
7771	HTAR0574	Male	Malay	2021	35	Single	Secondary	Private	Urban	No	No	No	No	Yes	No	No	No	No	No
7772	HTAR0575	Male	Malay	2020	39	Others	Secondary	Unemployed	Urban	Yes	Yes	No	No	Yes	No	No	No	No	No
7773	HTAR0576	Male	Malay	2020	31	Married	Primary	Unemployed	Urban	Yes	Yes	Yes	No	No	No	No	No	No	No
7774	HTAR0577	Male	Malay	2021	27	Single	Secondary	Private	Urban	Yes	Yes	No	No	Yes	No	No	No	No	No
7775	HTAR0578	Female	Malay	2021	30	Others	Secondary	Private	Urban	Yes	Yes	Yes	No	Yes	No	No	No	No	No
7776	HTAR0579	Male	Indian	2020	34	Single	Secondary	Unemployed	Urban	Yes	No	Yes	No	No	No	No	No	No	No
7777	HTAR0580	Male	Chinese	2021	26	Single	Secondary	Private	Urban	Yes	Yes	Yes	No	Yes	No	No	No	No	No
7778	HTAR0581	Male	Malay	2020	35	Single	Secondary	Self-employed	Urban	Yes	Yes	Yes	No	No	No	No	No	Yes	No
7779	HTAR0582	Male	Indian	2020	31	Others	Tertiary	Private	Urban	Yes	Yes	No	Yes	Yes	No	No	No	No	No
7780	HTAR0583	Male	Malay	2021	46	Others	Secondary	Private	Urban	Yes	Yes	No	Yes	No	No	No	No	No	No
7781	HTAR0584	Male	Indian	2021	32	Single	Secondary	Private	Urban	Yes	No	Yes	No	No	No	No	No	No	No

No	Patient ID	Gender	Ethnic group	Year of diagnosis	Age	Marital status	Education level	Occupation	Place of residence	History of psy illness	Tobacco	Alcohol	Cannabis	ATS	Inhalant	Sedative	Hallucinogens	Opioids	Kratom
7782	HTAR0585	Male	Malay	2021	25	Single	Secondary	Unemployed	Urban	Yes	No	No	Yes	No	No	No	No	No	No
7783	HTAR0586	Male	Indian	2020	32	Married	Secondary	Private	Urban	Yes	No	Yes	No	No	No	No	No	No	No
7784	HTAR0587	Male	Indian	2020	61	Married	Secondary	Unemployed	Urban	Yes	No	Yes	No	No	No	No	No	No	No
7785	HTAR0588	Female	Chinese	2021	44	Married	Tertiary	Private	Urban	Yes	Yes	Yes	No	Yes	No	No	No	No	No
7786	HTAR0589	Female	Malay	2020	30	Married	Secondary	Unemployed	Urban	Yes	No	Yes	Yes	Yes	No	No	No	No	No
7787	HTAR0590	Male	Malay	2020	42	Married	Secondary	Private	Urban	Yes	Yes	No	No	No	No	No	No	Yes	No
7788	HTAR0591	Female	Chinese	2020	41	Married	Secondary	Unemployed	Urban	Yes	No	No	No	Yes	No	No	No	No	No
7789	HTAR0592	Male	Malay	2021	35	Single	Secondary	Unemployed	Urban	Yes	No	No	No	Yes	No	No	No	No	No
7790	HTAR0593	Female	Indian	2020	45	Others	Secondary	Unemployed	Urban	Yes	No	No	No	Yes	No	No	No	Yes	No
7791	HTAR0594	Male	Malay	2021	29	Single	Secondary	Unemployed	Urban	Yes	No	No	No	Yes	No	No	No	No	No
7792	HTAR0595	Male	Malay	2021	24	Single	Secondary	Private	Urban	Yes	Yes	No	Yes	No	No	No	No	No	Yes
7793	HTAR0596	Male	Malay	2020	20	Married	Secondary	Unemployed	Urban	Yes	No	No	No	Yes	No	No	No	No	No
7794	HTAR0597	Male	Malay	2020	30	Single	Secondary	Unemployed	Urban	Yes	No	No	No	Yes	No	No	No	No	No
7795	HTAR0598	Male	Chinese	2020	42	Single	Secondary	Unemployed	Urban	Yes	No	No	No	Yes	No	No	No	No	No
7796	HTAR0599	Male	Malay	2020	55	Single	Secondary	Unemployed	Urban	Yes	Yes	No	No	Yes	No	No	No	Yes	No
7797	HTAR0600	Male	Malay	2021	33	Others	No formal education	Private	Urban	Yes	No	No	No	Yes	No	No	No	No	No
7798	HTAR0601	Male	Indian	2020	38	Others	Secondary	Private	Urban	Yes	Yes	Yes	Yes	No	No	No	No	No	No
7799	HTAR0602	Male	Malay	2021	26	Single	Secondary	Private	Urban	Yes	Yes	No	No	Yes	No	No	No	No	No
7800	HTAR0603	Male	Malay	2020	25	Single	Tertiary	Private	Urban	Yes	Yes	Yes	No	No	No	No	No	No	Yes
7801	HTAR0604	Male	Malay	2021	29	Single	Tertiary	Government	Urban	Yes	Yes	No	No	No	No	No	No	No	No
7802	HTAR0605	Male	Malay	2021	22	Single	Tertiary	Unemployed	Urban	Yes	Yes	No	Yes	No	No	No	No	No	No

No	Patient ID	Gender	Ethnic group	Year of diagnosis	Age	Marital status	Education level	Occupation	Place of residence	History of psy illness	Tobacco	Alcohol	Cannabis	ATS	Inhalant	Sedative	Hallucinogens	Opioids	Kratom
7803	HTAR0606	Female	Indian	2021	26	Others	Secondary	Unemployed	Urban	Yes	Yes	No	No	Yes	No	No	No	No	No
7804	HTAR0607	Male	Indian	2020	38	Others	Primary	Self-employed	Urban	Yes	No	Yes	No	No	No	No	No	No	No
7805	HTAR0608	Male	Malay	2020	22	Single	Tertiary	Unemployed	Urban	Yes	No	No	No	Yes	No	No	No	No	No
7806	HTAR0609	Male	Malay	2020	36	Married	Tertiary	Private	Urban	Yes	Yes	No	Yes	Yes	No	No	No	No	Yes
7807	HTAR0610	Female	Malay	2021	25	Others	Secondary	Private	Urban	Yes	Yes	No	Yes	Yes	No	No	No	No	No
7808	HTAR0611	Male	Indian	2021	29	Single	Secondary	Private	Urban	Yes	No	Yes	Yes	Yes	No	No	No	No	No
7809	HTAR0612	Female	Indian	2020	64	Single	Secondary	Unemployed	Urban	Yes	Yes	Yes	No	No	No	No	No	No	No
7810	HTAR0614	Male	Malay	2020	22	Single	Tertiary	Unemployed	Urban	Yes	Yes	No	No	Yes	No	No	No	No	No
7811	HTAR0615	Male	Indian	2020	61	Married	Secondary	Unemployed	Urban	Yes	No	Yes	No	No	No	No	No	No	No
7812	HTAR0616	Male	Others	2021	20	Others	No formal education	Private	Urban	Yes	Yes	No	Yes	Yes	No	No	No	No	No
7813	HTAR0617	Male	Malay	2020	39	Single	Secondary	Unemployed	Urban	Yes	Yes	No	No	Yes	No	No	No	No	No
7814	HTAR0618	Male	Malay	2021	40	Married	Secondary	Others	Urban	Yes	No	No	Yes	Yes	No	No	No	Yes	No
7815	HTAR0619	Male	Malay	2020	28	Others	Secondary	Unemployed	Urban	Yes	Yes	No	No	Yes	No	No	No	No	No
7816	HTAR0620	Male	Indian	2021	27	Others	Secondary	Unemployed	Urban	Yes	Yes	Yes	No	No	No	No	No	No	No
7817	HTAR0621	Male	Indian	2021	29	Married	Secondary	Private	Urban	Yes	Yes	Yes	Yes	Yes	No	No	No	No	No
7818	HTAR0622	Male	Indian	2021	45	Single	Secondary	Private	Urban	Yes	Yes	Yes	Yes	No	No	No	No	No	No
7819	HTAR0623	Male	Indian	2021	42	Married	Secondary	Private	Urban	Yes	No	Yes	No	No	No	No	No	No	No
7820	HTAR0624	Male	Others	2021	24	Others	No formal education	Private	Urban	Yes	Yes	No	No	Yes	No	No	No	No	No
7821	HTAR0625	Male	Malay	2020	24	Others	Secondary	Private	Urban	Yes	Yes	No	No	No	No	No	No	No	Yes
7822	HTAR0626	Male	Indian	2021	33	Single	Secondary	Unemployed	Urban	Yes	No	Yes	No	No	No	No	No	No	No
7823	HTAR0627	Male	Malay	2020	38	Single	Tertiary	Unemployed	Urban	Yes	No	No	No	Yes	No	No	No	No	No

No	Patient ID	Gender	Ethnic group	Year of diagnosis	Age	Marital status	Education level	Occupation	Place of residence	History of psy illness	Tobacco	Alcohol	Cannabis	ATS	Inhalant	Sedative	Hallucinogens	Opioids	Kratom
7824	HTAR0628	Male	Indian	2021	29	Married	Secondary	Private	Urban	Yes	No	No	No	Yes	No	No	No	No	No
7825	HTAR0629	Male	Malay	2021	24	Others	Secondary	Private	Urban	Yes	No	No	Yes	No	No	Yes	No	No	Yes
7826	HTAR0630	Male	Chinese	2020	40	Single	Secondary	Unemployed	Urban	Yes	No	No	No	Yes	No	No	No	Yes	No
7827	HTAR0631	Male	Malay	2020	43	Single	Secondary	Unemployed	Urban	Yes	No	No	No	Yes	No	No	No	Yes	No
7828	HTAR0632	Male	Indian	2021	34	Married	Secondary	Private	Urban	Yes	No	Yes	No	No	No	No	No	No	No
7829	HTAR0634	Male	Malay	2020	34	Married	Secondary	Government	Urban	Yes	Yes	No	No	No	No	Yes	No	No	No
7830	HTAR0635	Male	Indian	2020	40	Single	Secondary	Private	Urban	Yes	Yes	No	Yes	Yes	No	No	No	Yes	No
7831	HTAR0636	Female	Malay	2021	29	Single	Secondary	Unemployed	Urban	Yes	Yes	Yes	No	Yes	No	No	No	No	No
7832	HTAR0637	Male	Malay	2021	31	Others	No formal education	Unemployed	Urban	Yes	Yes	No	No	Yes	No	No	No	No	No
7833	HTAR0638	Male	Others	2020	34	Single	No formal education	Private	Urban	Yes	Yes	Yes	No	No	No	No	No	No	No
7834	HTAR0639	Male	Chinese	2020	65	Others	Secondary	Private	Urban	Yes	No	Yes	No	No	No	No	No	No	No
7835	HTAR0640	Male	Indian	2021	36	Married	Secondary	Unemployed	Urban	Yes	No	Yes	No	No	No	No	No	No	No
7836	HTAR0641	Male	Malay	2020	23	Single	No formal education	Unemployed	Urban	Yes	No	No	No	Yes	No	No	No	No	No
7837	HTAR0642	Male	Indian	2020	45	Married	Secondary	Private	Urban	Yes	No	Yes	No	No	No	No	No	No	No
7838	HTAR0643	Male	Malay	2020	37	Others	Secondary	Unemployed	Urban	Yes	No	No	No	Yes	No	No	No	No	No
7839	HTAR0644	Female	Malay	2021	29	Single	Secondary	Unemployed	Urban	Yes	No	No	No	Yes	No	No	No	No	No
7840	HTAR0645	Male	Indian	2020	40	Single	Secondary	Unemployed	Urban	Yes	Yes	No	No	Yes	No	No	No	No	No
7841	HTAR0646	Male	Indian	2021	42	Others	No formal education	Unemployed	Urban	Yes	No	Yes	No	No	No	No	No	No	No
7842	HTAR0647	Male	Indian	2021	51	Married	Secondary	Private	Urban	Yes	Yes	Yes	No	No	No	No	No	No	No
7843	HTAR0648	Male	Malay	2021	31	Others	Secondary	Unemployed	Urban	Yes	Yes	No	No	Yes	No	No	No	No	No
7844	HTAR0649	Male	Indian	2020	48	Married	Secondary	Unemployed	Urban	Yes	No	Yes	No	No	No	No	No	No	No

No	Patient ID	Gender	Ethnic group	Year of diagnosis	Age	Marital status	Education level	Occupation	Place of residence	History of psy illness	Tobacco	Alcohol	Cannabis	ATS	Inhalant	Sedative	Hallucinogens	Opioids	Kratom
7845	HTAR0650	Male	Chinese	2021	42	Others	Secondary	Unemployed	Urban	Yes	No	Yes	No	No	No	No	No	No	No
7846	HTAR0651	Male	Chinese	2020	38	Married	Tertiary	Private	Urban	Yes	No	Yes	No	No	No	No	No	No	No
7847	HTAR0652	Male	Malay	2020	26	Single	Secondary	Private	Urban	Yes	Yes	No	Yes	No	No	No	No	No	No
7848	HTAR0653	Male	Indian	2020	40	Others	No formal education	Private	Urban	Yes	No	Yes	No	No	No	No	No	No	No
7849	HTAR0654	Male	Indian	2020	36	Married	Secondary	Others	Urban	Yes	No	No	No	Yes	No	No	No	No	No
7850	HTAR0655	Male	Others	2021	36	Married	Secondary	Private	Urban	Yes	No	No	No	Yes	No	No	No	No	No
7851	HTAR0657	Male	Malay	2021	21	Married	Secondary	Self-employed	Urban	Yes	Yes	No	No	Yes	No	No	No	No	No
7852	HTAR0658	Male	Indian	2021	38	Single	Secondary	Unemployed	Urban	Yes	Yes	No	No	Yes	No	No	No	No	No
7853	HTAR0659	Male	Malay	2021	28	Others	Secondary	Private	Urban	Yes	Yes	No	No	Yes	No	No	No	No	No
7854	HTAR0660	Male	Indian	2020	48	Single	Tertiary	Private	Urban	Yes	Yes	No	No	Yes	No	No	No	No	No
7855	HTAR0661	Male	Chinese	2020	38	Married	Secondary	Unemployed	Urban	Yes	No	Yes	No	Yes	No	No	No	No	No
7856	HTAR0662	Male	Malay	2020	28	Single	Secondary	Private	Urban	Yes	No	No	Yes	Yes	No	No	No	No	No
7857	HTAR0663	Male	Malay	2020	29	Others	Secondary	Private	Urban	Yes	Yes	Yes	No	Yes	No	No	No	No	No
7858	HTAR0664	Male	Malay	2020	26	Single	Secondary	Unemployed	Urban	Yes	Yes	No	Yes	No	No	No	No	No	Yes
7859	HTAR0665	Male	Chinese	2021	32	Single	Secondary	Self-employed	Urban	Yes	No	No	No	Yes	No	No	No	No	No
7860	HTAR0666	Male	Malay	2020	34	Single	Secondary	Private	Urban	Yes	Yes	No	No	Yes	No	No	No	Yes	No
7861	HTAR0667	Male	Chinese	2020	58	Married	No formal education	Others	Urban	Yes	No	Yes	No	No	No	No	No	No	No
7862	HTAR0668	Male	Indian	2021	62	Married	Primary	Private	Urban	Yes	No	Yes	No	No	No	No	No	No	No
7863	HTAR0669	Male	Indian	2021	36	Married	No formal education	Unemployed	Urban	Yes	Yes	No	No	Yes	No	No	No	No	No
7864	HTAR0671	Male	Malay	2021	25	Single	Tertiary	Unemployed	Urban	Yes	Yes	No	Yes	No	No	No	No	Yes	No
7865	HTAR0672	Male	Indian	2021	30	Single	Secondary	Unemployed	Urban	Yes	Yes	No	No	Yes	No	No	No	No	No

No	Patient ID	Gender	Ethnic group	Year of diagnosis	Age	Marital status	Education level	Occupation	Place of residence	History of psy illness	Tobacco	Alcohol	Cannabis	ATS	Inhalant	Sedative	Hallucinogens	Opioids	Kratom
7866	HTAR0673	Male	Malay	2020	18	Single	Secondary	Unemployed	Urban	Yes	No	No	No	Yes	No	No	No	No	No
7867	HTAR0674	Male	Malay	2021	36	Others	Secondary	Private	Urban	Yes	No	No	No	Yes	No	No	No	No	No
7868	HTAR0675	Male	Malay	2021	21	Single	Secondary	Unemployed	Urban	Yes	No	No	Yes	No	No	No	No	No	No
7869	HTAR0676	Male	Malay	2021	18	Single	Secondary	Unemployed	Urban	Yes	Yes	Yes	No	No	No	No	No	No	No
7870	HTAR0677	Male	Chinese	2021	41	Married	Secondary	Self-employed	Urban	Yes	No	No	No	Yes	No	No	No	No	No
7871	HTAR0678	Male	Indian	2020	32	Married	Secondary	Unemployed	Urban	Yes	No	No	Yes	Yes	No	No	No	No	No
7872	HTAR0679	Male	Indian	2021	38	Married	Secondary	Unemployed	Urban	Yes	No	Yes	No	Yes	No	No	No	No	No
7873	HTAR0680	Male	Malay	2020	36	Married	Secondary	Unemployed	Urban	Yes	No	No	No	Yes	No	No	No	No	No
7874	HTAR0681	Female	Malay	2020	35	Married	Secondary	Unemployed	Urban	Yes	No	No	No	Yes	No	No	No	Yes	No
7875	HTAR0682	Male	Indian	2020	43	Single	Tertiary	Unemployed	Urban	Yes	No	No	No	Yes	No	No	No	No	No
7876	HTAR0683	Male	Chinese	2020	31	Single	Primary	Unemployed	Urban	Yes	No	No	No	Yes	No	No	No	No	No
7877	HTAR0684	Male	Chinese	2019	55	Others	No formal education	Others	Urban	Yes	No	No	No	Yes	No	No	No	No	No
7878	HTAR0685	Female	Others	2021	24	Others	Secondary	Unemployed	Urban	Yes	Yes	Yes	Yes	Yes	No	No	No	No	No
7879	HTAR0686	Male	Chinese	2020	44	Single	Secondary	Unemployed	Urban	Yes	Yes	No	No	Yes	No	No	No	No	No
7880	HTAR0687	Male	Indian	2020	44	Married	Secondary	Others	Urban	Yes	Yes	Yes	No	Yes	No	No	No	No	No
7881	HTAR0688	Female	Indian	2020	24	Single	Tertiary	Unemployed	Urban	Yes	No	No	Yes	Yes	No	No	No	No	No
7882	HTAR0689	Male	Others	2020	37	Others	No formal education	Unemployed	Urban	Yes	No	Yes	No	No	No	No	No	No	No
7883	HTAR0690	Male	Indian	2020	44	Others	No formal education	Unemployed	Urban	Yes	No	No	Yes	Yes	No	No	No	Yes	No
7884	HTAR0691	Male	Indian	2021	23	Single	Secondary	Unemployed	Urban	Yes	No	No	Yes	Yes	No	No	No	No	No
7885	HTAR0692	Male	Others	2021	43	Married	Tertiary	Private	Urban	Yes	No	No	No	Yes	No	No	No	Yes	No
7886	HTAR0693	Male	Malay	2021	38	Others	Secondary	Private	Urban	Yes	No	No	No	Yes	No	No	No	No	No

No	Patient ID	Gender	Ethnic group	Year of diagnosis	Age	Marital status	Education level	Occupation	Place of residence	History of psy illness	Tobacco	Alcohol	Cannabis	ATS	Inhalant	Sedative	Hallucinogens	Opioids	Kratom
7888	HTAR0694	Female	Others	2020	17	Single	Secondary	Unemployed	Urban	Yes	No	No	No	Yes	No	No	No	No	No
7887	HTAR0694	Female	Others	2020	17	Others	Secondary	Unemployed	Urban	Yes	No	No	No	Yes	No	No	No	No	No
7889	HTAR0695	Male	Malay	2020	30	Married	Secondary	Private	Urban	Yes	No	No	No	Yes	No	No	No	Yes	No
7890	HTAR0696	Male	Indian	2020	45	Married	Primary	Private	Urban	Yes	No	Yes	No	No	No	No	No	No	No
7891	HTAR0697	Male	Indian	2020	39	Others	Primary	Private	Urban	Yes	No	Yes	No	No	No	No	No	No	No
7892	HTAR0698	Male	Malay	2020	32	Others	Secondary	Unemployed	Urban	Yes	Yes	No	No	Yes	No	No	No	No	No
7893	HTAR0699	Male	Malay	2020	24	Married	Secondary	Unemployed	Urban	Yes	Yes	Yes	Yes	Yes	No	No	No	No	No
7894	HTAR0700	Male	Malay	2021	33	Others	No formal education	Unemployed	Urban	Yes	No	No	Yes	Yes	No	No	No	No	No
7895	HTAR0701	Male	Malay	2020	23	Single	Tertiary	Private	Urban	Yes	Yes	Yes	No	No	No	No	No	No	No
7896	HTAR0702	Male	Malay	2020	29	Single	Tertiary	Unemployed	Urban	Yes	Yes	No	No	Yes	No	No	No	No	No
7897	HTAR0703	Female	Malay	2021	38	Others	Secondary	Unemployed	Urban	Yes	No	No	No	Yes	No	No	No	No	No
7898	HTAR0704	Male	Indian	2018	58	Married	Primary	Private	Urban	Yes	Yes	Yes	No	No	No	No	No	No	No
7899	HTAR0705	Male	Indian	2021	43	Married	No formal education	Unemployed	Urban	Yes	Yes	Yes	No	No	No	No	No	No	No
7900	HTAR0706	Male	Indian	2021	41	Single	Secondary	Unemployed	Urban	Yes	Yes	Yes	No	No	No	No	No	No	No
7901	HTAR0707	Male	Indian	2021	36	Others	Secondary	Unemployed	Urban	Yes	Yes	No	Yes	No	No	No	No	No	No
7902	HTAR0708	Male	Malay	2020	37	Single	Secondary	Private	Urban	Yes	Yes	No	Yes	Yes	No	No	No	No	No
7903	HTAR0709	Male	Malay	2021	37	Others	Secondary	Private	Urban	Yes	Yes	No	No	Yes	No	No	No	No	Yes
7904	HTAR0710	Male	Indian	2021	25	Single	Tertiary	Private	Urban	Yes	Yes	Yes	Yes	No	No	No	No	No	No
7905	HTAR0711	Male	Indian	2021	28	Single	No formal education	Unemployed	Urban	Yes	No	No	No	Yes	No	No	No	No	No
7906	HTAR0712	Male	Malay	2021	31	Others	No formal education	Self-employed	Urban	Yes	No	No	Yes	No	No	No	No	No	No
7907	HTAR0713	Male	Indian	2021	36	Others	Secondary	Private	Urban	Yes	Yes	No	No	Yes	No	No	No	No	No

No	Patient ID	Gender	Ethnic group	Year of diagnosis	Age	Marital status	Education level	Occupation	Place of residence	History of psy illness	Tobacco	Alcohol	Cannabis	ATS	Inhalant	Sedative	Hallucinogens	Opioids	Kratom
7908	HTAR0714	Male	Malay	2021	45	Single	Secondary	Unemployed	Urban	Yes	No	No	Yes	Yes	No	No	No	No	No
7909	HTAR0715	Male	Malay	2021	32	Married	Secondary	Others	Urban	Yes	No	No	No	Yes	No	No	No	No	No
7910	HTAR0716	Male	Malay	2020	47	Others	No formal education	Unemployed	Urban	Yes	No	No	No	Yes	No	No	No	Yes	No
7911	HTAR0717	Male	Malay	2021	40	Single	Secondary	Unemployed	Urban	Yes	No	No	No	Yes	No	No	No	Yes	No
7912	HTAR0718	Male	Malay	2021	27	Others	Secondary	Unemployed	Urban	Yes	Yes	No	No	Yes	Yes	No	No	Yes	Yes
7913	HTAR0719	Male	Indian	2021	30	Others	No formal education	Private	Urban	Yes	No	No	No	Yes	No	No	No	No	No
7914	HTAR0720	Male	Indian	2021	28	Married	Tertiary	Unemployed	Urban	Yes	Yes	Yes	Yes	No	No	No	No	No	No
7915	HTAR0721	Male	Indian	2021	34	Single	Secondary	Unemployed	Urban	Yes	No	No	Yes	Yes	No	No	No	Yes	No
7916	HTAR0722	Male	Malay	2021	38	Others	Tertiary	Unemployed	Urban	Yes	Yes	No	No	Yes	No	No	No	No	No
7917	HTAR0723	Male	Malay	2021	45	Married	No formal education	Private	Urban	Yes	No	No	No	Yes	No	No	No	No	No
7918	HTAR0724	Male	Malay	2021	43	Single	Primary	Unemployed	Urban	Yes	No	No	No	Yes	No	No	No	Yes	No
7919	HTAR0725	Male	Malay	2021	32	Single	Secondary	Unemployed	Urban	Yes	Yes	No	Yes	No	No	No	No	No	No
7920	HTAR0726	Male	Indian	2021	35	Married	Primary	Private	Urban	Yes	Yes	No	Yes	No	No	No	No	No	No
7921	HTAR0727	Male	Malay	2021	22	Single	No formal education	Unemployed	Urban	Yes	No	No	Yes	No	No	No	No	No	No
7922	HTAR0728	Male	Malay	2020	27	Others	Secondary	Unemployed	Urban	Yes	Yes	No	No	Yes	No	No	No	No	No
7923	HTAR0729	Male	Malay	2021	50	Married	No formal education	Private	Urban	Yes	No	No	Yes	No	No	No	No	No	No
7924	HTAR0731	Male	Malay	2021	35	Single	Tertiary	Private	Urban	Yes	Yes	Yes	No	Yes	No	No	No	No	No
7925	HTAR0732	Male	Malay	2021	45	Single	Primary	Unemployed	Urban	Yes	Yes	Yes	No	Yes	No	No	No	Yes	No
7926	HTAR0733	Male	Malay	2021	29	Married	Secondary	Unemployed	Urban	Yes	Yes	No	No	Yes	No	No	No	No	No
7927	HTAR0734	Male	Indian	2019	46	Married	Secondary	Private	Urban	Yes	Yes	Yes	No	No	No	No	No	No	No
7928	HTAR0735	Male	Malay	2021	33	Others	Primary	Others	Urban	Yes	Yes	No	No	No	No	No	No	No	No

No	Patient ID	Gender	Ethnic group	Year of diagnosis	Age	Marital status	Education level	Occupation	Place of residence	History of psy illness	Tobacco	Alcohol	Cannabis	ATS	Inhalant	Sedative	Hallucinogens	Opioids	Kratom
7929	HTAR0736	Male	Malay	2021	34	Others	Tertiary	Unemployed	Urban	Yes	No	Yes	No	Yes	No	No	No	No	No
7930	HTAR0737	Male	Malay	2021	21	Others	Secondary	Self-employed	Urban	Yes	Yes	No	Yes	Yes	No	No	No	No	No
7931	HTAR0738	Male	Indian	2021	37	Others	Secondary	Unemployed	Urban	Yes	No	No	Yes	No	No	No	No	No	No
7932	HTAR0740	Male	Malay	2021	32	Others	No formal education	Unemployed	Urban	Yes	Yes	No	No	Yes	No	No	No	Yes	No
7933	HTAR0741	Male	Indian	2021	36	Single	Tertiary	Unemployed	Urban	Yes	Yes	Yes	Yes	No	No	No	No	No	No
7934	HTAR0743	Male	Malay	2021	37	Single	Tertiary	Private	Urban	Yes	Yes	No	No	Yes	No	No	No	No	No
7935	HTAR0744	Male	Malay	2021	29	Single	Tertiary	Unemployed	Urban	Yes	Yes	No	Yes	Yes	No	No	No	No	No
7936	HTAR0745	Male	Malay	2021	22	Single	No formal education	Others	Urban	Yes	No	No	Yes	No	No	No	No	No	No
7937	HTAR0746	Male	Malay	2021	44	Married	No formal education	Unemployed	Urban	Yes	Yes	Yes	No	Yes	No	No	No	No	No
7938	HTAR0747	Male	Malay	2021	27	Others	Tertiary	Self-employed	Urban	Yes	No	No	Yes	Yes	No	No	No	No	No
7939	HTAR0748	Male	Malay	2021	29	Single	Primary	Private	Urban	Yes	Yes	Yes	Yes	Yes	No	No	No	No	No
7940	HTAR0749	Male	Others	2021	29	Married	No formal education	Others	Urban	Yes	No	No	No	Yes	No	No	No	No	No
7941	HTAR0750	Male	Malay	2021	33	Others	Tertiary	Unemployed	Urban	Yes	No	No	No	Yes	No	No	No	No	No
7942	HTAR0751	Male	Indian	2021	63	Others	No formal education	Private	Urban	Yes	Yes	Yes	No	No	No	No	No	Yes	No
7943	HTAR0752	Male	Malay	2021	29	Others	No formal education	Others	Urban	Yes	No	No	No	Yes	No	No	No	Yes	No
7944	HTAR0753	Male	Malay	2021	30	Single	Secondary	Private	Urban	Yes	Yes	No	No	Yes	No	No	No	No	No
7945	HTAR0754	Female	Others	2021	29	Others	No formal education	Unemployed	Urban	Yes	No	No	No	Yes	No	No	No	No	No
7946	HTAR0755	Male	Indian	2021	57	Others	Primary	Private	Urban	Yes	Yes	Yes	No	No	No	No	No	No	No
7947	HTAR0756	Male	Malay	2020	21	Single	Tertiary	Unemployed	Urban	Yes	Yes	No	No	Yes	No	No	No	No	No
7948	HTAR0757	Male	Indian	2020	27	Single	Tertiary	Self-employed	Urban	Yes	Yes	Yes	Yes	No	No	No	No	No	No
7949	HTAR0758	Male	Malay	2020	36	Single	Secondary	Private	Urban	Yes	Yes	No	No	Yes	No	No	No	No	No

No	Patient ID	Gender	Ethnic group	Year of diagnosis	Age	Marital status	Education level	Occupation	Place of residence	History of psy illness	Tobacco	Alcohol	Cannabis	ATS	Inhalant	Sedative	Hallucinogens	Opioids	Kratom
7950	HTAR0759	Male	Chinese	2020	28	Single	Tertiary	Private	Urban	Yes	No	No	No	Yes	No	No	No	No	No
7951	HTAR0760	Male	Malay	2020	17	Single	Secondary	Unemployed	Urban	Yes	No	No	No	Yes	No	No	No	No	No
7952	HTAR0761	Male	Malay	2021	38	Others	Tertiary	Unemployed	Urban	Yes	Yes	No	Yes	Yes	Yes	No	No	No	No
7953	HTAR0762	Male	Malay	2021	35	Single	Secondary	Private	Urban	Yes	Yes	No	Yes	Yes	No	No	No	No	No
7954	HTAR0763	Female	Malay	2020	25	Single	Tertiary	Private	Urban	Yes	Yes	No	Yes	No	No	No	No	No	No
7955	HTAR0764	Male	Malay	2020	42	Single	Secondary	Private	Urban	Yes	Yes	Yes	No	No	No	No	No	No	No
7956	HTAR0765	Male	Indian	2021	25	Married	Secondary	Private	Urban	Yes	Yes	Yes	No	Yes	No	No	No	No	No
7957	HTAR0766	Male	Indian	2020	53	Married	No formal education	Unemployed	Urban	Yes	Yes	Yes	No	No	No	No	No	No	No
7958	HTAR0767	Male	Chinese	2021	54	Married	Secondary	Self-employed	Urban	Yes	Yes	Yes	No	No	No	No	No	Yes	No
7959	HTAR0768	Male	Indian	2021	36	Single	Tertiary	Private	Urban	Yes	Yes	Yes	No	Yes	No	No	No	No	No
7960	HTAR0769	Male	Indian	2021	36	Married	Primary	Private	Urban	Yes	Yes	No	No	Yes	No	No	No	No	No
7961	HTAR0770	Male	Indian	2021	24	Single	Tertiary	Unemployed	Urban	Yes	No	Yes	Yes	No	No	No	No	No	No
7962	HTAR0771	Male	Malay	2020	33	Married	Secondary	Unemployed	Urban	Yes	No	No	Yes	Yes	No	No	No	No	No
7963	HTAR0772	Female	Chinese	2021	35	Others	Secondary	Private	Urban	Yes	Yes	Yes	Yes	Yes	No	No	No	No	No
7964	HTAR0773	Male	Malay	2021	50	Others	Secondary	Private	Urban	Yes	No	No	No	Yes	No	No	No	No	No
7965	HTAR0774	Male	Malay	2021	35	Married	Secondary	Self-employed	Urban	Yes	Yes	No	No	Yes	No	No	No	No	No
7966	HTAR0775	Male	Indian	2021	33	Others	Primary	Unemployed	Urban	No	Yes	Yes	No	No	No	No	No	Yes	No
7967	HTAR0776	Male	Indian	2020	43	Others	Secondary	Unemployed	Urban	Yes	Yes	No	No	Yes	No	No	No	No	No
7968	HTAR0777	Male	Malay	2020	31	Married	Tertiary	Government	Urban	Yes	No	Yes	No	Yes	No	No	No	No	No
7969	HTAR0779	Male	Malay	2021	40	Married	Secondary	Private	Urban	Yes	Yes	No	No	No	No	No	No	No	Yes
7970	HTAR0780	Male	Indian	2020	41	Married	Primary	Private	Urban	Yes	Yes	Yes	No	No	No	No	No	No	No

No	Patient ID	Gender	Ethnic group	Year of diagnosis	Age	Marital status	Education level	Occupation	Place of residence	History of psy illness	Tobacco	Alcohol	Cannabis	ATS	Inhalant	Sedative	Hallucinogens	Opioids	Kratom
7971	HTAR0781	Male	Malay	2020	39	Others	Primary	Unemployed	Urban	Yes	No	No	No	Yes	No	No	No	No	No
7972	HTAR0782	Male	Chinese	2020	38	Single	No formal education	Unemployed	Urban	Yes	No	No	Yes	Yes	No	No	No	No	No
7973	HTAR0783	Male	Indian	2021	31	Single	Tertiary	Private	Urban	Yes	Yes	No	No	Yes	No	No	No	No	No
7974	HTAR0784	Male	Malay	2020	31	Single	Secondary	Unemployed	Urban	Yes	No	No	No	Yes	No	No	No	No	No
7975	HTAR0785	Male	Indian	2021	21	Single	Secondary	Unemployed	Urban	Yes	No	Yes	Yes	Yes	No	No	No	No	No
7976	HTAR0786	Male	Chinese	2021	26	Single	Tertiary	Others	Urban	Yes	No	No	No	Yes	No	No	No	No	No
7977	HTAR0787	Male	Malay	2019	29	Single	Secondary	Unemployed	Urban	Yes	Yes	No	Yes	Yes	No	No	No	No	Yes
7978	HTAR0788	Male	Indian	2021	39	Married	Secondary	Private	Urban	Yes	Yes	Yes	No	No	No	No	No	No	No
7979	HTAR0789	Female	Malay	2020	51	Others	No formal education	Unemployed	Urban	Yes	Yes	No	No	Yes	No	No	No	No	No
7980	HTAR0790	Female	Malay	2020	32	Married	No formal education	Unemployed	Urban	Yes	Yes	No	No	Yes	No	No	No	No	No
7981	HTAR0791	Male	Malay	2020	31	Single	No formal education	Unemployed	Urban	Yes	Yes	No	No	No	No	No	No	Yes	No
7982	HTAR0792	Male	Malay	2020	33	Single	No formal education	Others	Urban	Yes	Yes	Yes	Yes	Yes	No	No	No	Yes	No
7983	HTAR0793	Male	Malay	2020	37	Others	No formal education	Self-employed	Urban	Yes	No	No	No	Yes	No	No	No	No	No
7984	HTAR0794	Male	Malay	2020	44	Others	No formal education	Unemployed	Urban	Yes	No	No	No	Yes	No	No	No	Yes	No
7985	HTAR0795	Male	Malay	2020	31	Others	Primary	Private	Urban	Yes	No	No	No	Yes	No	No	No	Yes	Yes
7986	HTAR0796	Male	Malay	2020	39	Others	No formal education	Others	Urban	Yes	Yes	No	Yes	Yes	No	No	No	No	No
7987	HTAR0797	Male	Malay	2020	36	Married	Secondary	Unemployed	Urban	Yes	Yes	No	Yes	Yes	No	No	No	No	No
7988	HTAR0798	Male	Malay	2020	21	Others	No formal education	Others	Urban	Yes	No	No	No	Yes	No	No	No	No	No
7989	HTAR0799	Male	Malay	2020	32	Others	Secondary	Private	Urban	Yes	No	No	Yes	Yes	No	No	No	No	No
7990	HTAR0850	Male	Malay	2020	25	Single	Secondary	Unemployed	Urban	Yes	Yes	No	No	Yes	No	No	No	Yes	Yes
7991	HTAR0851	Male	Malay	2020	25	Others	Secondary	Private	Urban	Yes	Yes	No	No	No	No	No	No	No	No

No	Patient ID	Gender	Ethnic group	Year of diagnosis	Age	Marital status	Education level	Occupation	Place of residence	History of psy illness	Tobacco	Alcohol	Cannabis	ATS	Inhalant	Sedative	Hallucinogens	Opioids	Kratom
7992	HTAR0852	Male	Malay	2020	29	Others	No formal education	Private	Urban	Yes	No	No	Yes	Yes	No	No	No	No	No
7993	HTAR0854	Male	Malay	2020	27	Others	No formal education	Private	Urban	Yes	No	No	No	Yes	No	No	No	No	No
7994	HTAR0855	Male	Malay	2020	25	Single	Secondary	Unemployed	Urban	Yes	Yes	Yes	No	Yes	No	No	No	No	No
7995	HTAR0856	Male	Malay	2020	25	Others	Tertiary	Private	Urban	Yes	No	No	No	No	No	No	No	No	Yes
7996	HTAR0857	Male	Malay	2020	33	Married	Secondary	Self-employed	Urban	Yes	Yes	No	No	No	No	No	No	No	Yes
7997	HTAR0858	Male	Malay	2020	30	Single	Primary	Self-employed	Urban	Yes	No	No	No	Yes	No	No	No	Yes	No
7998	HTAR0859	Female	Chinese	2021	33	Married	No formal education	Unemployed	Urban	Yes	No	No	No	Yes	No	No	No	No	No
7999	HTAR0860	Female	Malay	2020	37	Others	No formal education	Unemployed	Urban	Yes	Yes	No	No	Yes	No	No	No	No	No
8000	HTAR0861	Male	Indian	2020	44	Others	No formal education	Self-employed	Urban	Yes	Yes	Yes	No	Yes	No	No	No	Yes	No
8001	HTAR0862	Male	Indian	2020	33	Others	Secondary	Private	Urban	Yes	Yes	Yes	Yes	Yes	No	No	No	No	No
8002	HTAR0863	Female	Indian	2021	25	Single	Tertiary	Private	Urban	Yes	Yes	Yes	Yes	No	No	No	No	No	No
8003	HTAR0864	Male	Malay	2020	47	Married	No formal education	Self-employed	Urban	Yes	Yes	No	Yes	Yes	No	No	No	No	No
8004	HTAR0866	Female	Others	2020	37	Married	No formal education	Unemployed	Urban	Yes	No	No	No	Yes	No	No	No	No	No
8005	HTAR0867	Female	Others	2020	32	Others	No formal education	Private	Urban	Yes	No	No	No	Yes	No	No	No	No	No
8006	HTAR0868	Female	Others	2021	19	Others	No formal education	Unemployed	Urban	Yes	No	No	No	Yes	No	No	No	No	No
8007	HTAR0869	Female	Others	2021	25	Single	No formal education	Private	Urban	Yes	No	No	No	Yes	No	No	No	No	No
8008	HTAR0870	Female	Indian	2021	35	Married	Tertiary	Unemployed	Urban	Yes	Yes	Yes	No	Yes	No	No	No	Yes	No
8009	HTAR0871	Male	Indian	2021	32	Single	Secondary	Unemployed	Urban	Yes	Yes	Yes	No	No	No	No	No	No	No
8010	HTAR0872	Male	Chinese	2020	58	Married	No formal education	Unemployed	Urban	Yes	No	Yes	No	No	No	No	No	No	No
8011	HTAR0873	Female	Malay	2021	43	Others	Secondary	Private	Urban	Yes	No	No	No	Yes	No	No	No	No	No
8012	HTAR0874	Male	Indian	2021	34	Others	No formal education	Private	Urban	No	Yes	Yes	No	No	No	No	No	No	No

No	Patient ID	Gender	Ethnic group	Year of diagnosis	Age	Marital status	Education level	Occupation	Place of residence	History of psy illness	Tobacco	Alcohol	Cannabis	ATS	Inhalant	Sedative	Hallucinogens	Opioids	Kratom
8013	HTAR0875	Male	Indian	2020	37	Others	No formal education	Others	Urban	Yes	No	Yes	No	No	No	No	No	No	No
8014	HTAR0876	Male	Malay	2020	36	Married	No formal education	Others	Urban	Yes	Yes	No	Yes	No	No	No	No	No	Yes
8015	HTAR0878	Male	Indian	2020	41	Single	No formal education	Unemployed	Urban	Yes	Yes	No	No	Yes	No	No	No	No	No
8016	HTAR0879	Male	Malay	2020	40	Married	No formal education	Others	Urban	Yes	No	No	No	Yes	No	No	No	No	No
8017	HTAR0880	Male	Malay	2020	37	Married	Secondary	Private	Urban	Yes	No	No	No	Yes	No	No	No	No	No
8018	HTAR0881	Male	Malay	2020	38	Married	No formal education	Private	Urban	Yes	Yes	Yes	No	Yes	No	No	No	Yes	No
8019	HTAR0882	Female	Malay	2020	39	Married	No formal education	Private	Urban	Yes	No	No	No	Yes	No	No	No	No	No
8020	HTAR0883	Male	Chinese	2020	41	Married	No formal education	Others	Urban	Yes	Yes	No	No	Yes	No	No	No	No	No
8021	HTAR0884	Female	Indian	2021	26	Others	No formal education	Private	Urban	Yes	No	No	No	Yes	No	No	No	No	No
8022	HTAR0885	Male	Malay	2020	47	Others	No formal education	Others	Urban	Yes	No	No	No	No	No	No	No	No	Yes
8023	HTAR0886	Female	Malay	2021	20	Married	No formal education	Others	Urban	Yes	Yes	No	No	Yes	No	No	No	No	No
8024	HTAR0887	Female	Indian	2020	34	Single	No formal education	Unemployed	Urban	Yes	Yes	No	Yes	Yes	No	No	No	No	No
8025	HTAR0890	Male	Others	2020	30	Others	No formal education	Private	Urban	Yes	Yes	No	No	Yes	No	No	No	No	No
8026	HTAR0892	Male	Chinese	2020	55	Others	No formal education	Others	Urban	Yes	No	No	No	Yes	No	No	No	Yes	No
8027	HTAR0893	Male	Malay	2020	27	Others	No formal education	Others	Urban	Yes	No	No	No	Yes	No	No	No	No	No
8028	HTAR0894	Male	Chinese	2020	44	Single	No formal education	Unemployed	Urban	Yes	No	No	Yes	Yes	No	No	No	No	No
8029	HTAR0895	Female	Others	2021	35	Single	No formal education	Unemployed	Urban	Yes	No	No	No	Yes	No	No	No	No	No
8030	HTAR0896	Female	Others	2021	31	Others	No formal education	Private	Urban	Yes	No	No	No	No	No	No	No	Yes	No
8031	HTAR0897	Male	Malay	2020	21	Single	Secondary	Private	Urban	Yes	No	No	Yes	No	No	No	Yes	No	No
8032	HTAR0899	Male	Malay	2020	35	Others	No formal education	Unemployed	Urban	Yes	No	No	No	Yes	No	No	No	No	No
8033	HTAR0900	Male	Others	2020	52	Others	No formal education	Unemployed	Urban	Yes	No	No	No	No	No	No	No	Yes	No

No	Patient ID	Gender	Ethnic group	Year of diagnosis	Age	Marital status	Education level	Occupation	Place of residence	History of psy illness	Tobacco	Alcohol	Cannabis	ATS	Inhalant	Sedative	Hallucinogens	Opioids	Kratom
8034	HTAR0901	Male	Indian	2020	34	Single	No formal education	Unemployed	Urban	Yes	No	Yes	No	Yes	No	No	No	No	No
8035	HTAR0902	Male	Chinese	2020	50	Married	No formal education	Private	Urban	Yes	No	No	No	Yes	No	No	No	No	No
8036	HTAR0903	Male	Malay	2018	40	Married	No formal education	Unemployed	Urban	Yes	No	No	No	Yes	No	No	No	Yes	No
8037	HTAR0904	Male	Malay	2020	24	Single	No formal education	Private	Urban	Yes	Yes	No	No	Yes	No	No	No	No	No
8038	HTAR0905	Male	Malay	2020	31	Others	No formal education	Private	Urban	Yes	No	No	No	Yes	No	No	No	No	No
8039	HTAR0907	Male	Malay	2021	32	Married	No formal education	Self-employed	Urban	Yes	Yes	No	Yes	No	No	No	No	No	No
8040	HTAR0908	Male	Indian	2021	40	Single	Secondary	Self-employed	Urban	Yes	Yes	Yes	No	No	No	No	No	No	No
8041	HTAR0909	Male	Chinese	2020	44	Single	Secondary	Unemployed	Urban	Yes	Yes	Yes	No	Yes	No	No	No	Yes	No
8042	HTAR0911	Male	Indian	2021	24	Single	No formal education	Others	Urban	Yes	No	Yes	Yes	No	No	No	No	No	No
8043	HTAR0912	Male	Malay	2021	32	Others	No formal education	Others	Urban	Yes	No	No	No	Yes	No	No	No	No	No
8044	HTAR0913	Male	Chinese	2020	38	Others	Tertiary	Self-employed	Urban	Yes	Yes	Yes	No	Yes	No	No	No	No	No
8045	HTAR0914	Female	Malay	2018	28	Married	Secondary	Unemployed	Urban	Yes	Yes	No	No	Yes	No	No	No	No	No
8046	HTAR0915	Male	Malay	2019	21	Others	Tertiary	Unemployed	Urban	Yes	Yes	Yes	Yes	Yes	No	No	No	No	No
8047	HTAR0916	Male	Indian	2018	47	Married	No formal education	Others	Urban	Yes	Yes	No	Yes	No	No	No	No	Yes	No
8048	HTAR0917	Male	Indian	2019	50	Married	Primary	Others	Urban	Yes	Yes	Yes	No	No	No	No	No	No	No
8049	HTAR0920	Female	Malay	2019	26	Married	Tertiary	Unemployed	Urban	Yes	Yes	No	No	Yes	No	No	No	No	No
8050	HTAR0922	Male	Malay	2018	23	Single	Secondary	Private	Urban	Yes	Yes	No	No	Yes	No	No	No	No	No
8051	HTAR0924	Male	Malay	2018	36	Single	No formal education	Unemployed	Urban	Yes	No	No	No	Yes	No	No	No	No	No
8052	HTAR0928	Female	Others	2018	36	Others	No formal education	Others	Urban	Yes	No	No	No	Yes	No	No	No	No	No
8053	HTAR0929	Male	Malay	2019	28	Single	No formal education	Others	Urban	Yes	Yes	No	Yes	No	No	No	No	No	No
8054	HTAR0930	Male	Malay	2018	34	Others	Tertiary	Unemployed	Urban	Yes	Yes	Yes	Yes	Yes	No	No	No	No	No

No	Patient ID	Gender	Ethnic group	Year of diagnosis	Age	Marital status	Education level	Occupation	Place of residence	History of psy illness	Tobacco	Alcohol	Cannabis	ATS	Inhalant	Sedative	Hallucinogens	Opioids	Kratom
8055	HTAR0931	Female	Malay	2018	27	Married	Secondary	Unemployed	Urban	Yes	No	No	No	Yes	No	No	No	No	No
8056	HTAR0932	Male	Malay	2019	27	Single	Tertiary	Unemployed	Urban	Yes	Yes	Yes	Yes	No	No	No	No	No	No
8057	HTAR0933	Male	Others	2018	26	Single	Tertiary	Unemployed	Urban	Yes	No	No	Yes	No	No	No	No	No	No
8058	HTAR0934	Male	Malay	2019	31	Married	Secondary	Private	Urban	Yes	Yes	No	No	Yes	No	No	No	No	No
8059	HTAR0935	Male	Indian	2018	35	Married	No formal education	Private	Urban	Yes	Yes	No	No	No	No	No	No	Yes	No
8060	HTAR0936	Male	Malay	2018	42	Others	No formal education	Private	Urban	Yes	Yes	No	Yes	No	No	No	No	No	No
8061	HTAR0937	Male	Chinese	2019	41	Married	No formal education	Unemployed	Urban	Yes	No	No	Yes	No	No	No	No	No	No
8062	HTAR0938	Male	Indian	2019	41	Married	No formal education	Private	Urban	Yes	No	Yes	No	Yes	No	No	No	No	No
8063	HTAR0939	Male	Chinese	2019	43	Others	No formal education	Private	Urban	Yes	No	No	No	Yes	No	No	No	No	No
8064	HTAR0940	Male	Indian	2018	23	Single	Primary	Unemployed	Urban	Yes	Yes	Yes	Yes	No	No	No	No	No	No
8065	HTAR0941	Male	Indian	2019	29	Single	Secondary	Unemployed	Urban	Yes	No	Yes	Yes	No	No	No	No	No	No
8066	HTAR0943	Female	Malay	2019	22	Married	Primary	Unemployed	Urban	Yes	No	No	No	Yes	No	No	No	Yes	No
8067	HTAR0944	Male	Indian	2019	31	Single	No formal education	Private	Urban	Yes	No	No	No	Yes	No	No	No	No	No
8068	HTAR0945	Male	Malay	2018	37	Single	Tertiary	Private	Urban	Yes	No	No	No	Yes	No	No	No	No	No
8069	HTAR0946	Male	Malay	2018	34	Married	No formal education	Unemployed	Urban	Yes	No	No	No	Yes	No	No	No	No	No
8070	HTAR0947	Male	Indian	2019	31	Married	No formal education	Private	Urban	Yes	Yes	Yes	Yes	No	No	No	No	No	No
8071	HTAR0948	Male	Chinese	2019	33	Single	Secondary	Unemployed	Urban	Yes	No	Yes	No	No	No	No	No	No	No
8072	HTAR0949	Male	Indian	2018	29	Single	No formal education	Private	Urban	Yes	Yes	Yes	Yes	No	No	No	No	No	No
8073	HTAR0949	Male	Indian	2018	29	Single	No formal education	Unemployed	Urban	Yes	Yes	Yes	Yes	Yes	No	No	No	No	No
8074	HTAR0950	Female	Indian	2018	33	Married	Secondary	Unemployed	Urban	Yes	No	No	No	Yes	No	No	No	No	No
8075	HTAR0951	Male	Others	2018	40	Single	No formal education	Unemployed	Urban	Yes	No	No	No	Yes	No	No	No	No	No

No	Patient ID	Gender	Ethnic group	Year of diagnosis	Age	Marital status	Education level	Occupation	Place of residence	History of psy illness	Tobacco	Alcohol	Cannabis	ATS	Inhalant	Sedative	Hallucinogens	Opioids	Kratom
8076	HTAR0952	Female	Others	2018	31	Others	No formal education	Unemployed	Urban	Yes	No	No	No	Yes	No	No	No	No	No
8077	HTAR0953	Male	Others	2018	23	Single	No formal education	Private	Urban	Yes	No	No	No	Yes	No	No	No	No	No
8078	HTAR0954	Male	Indian	2019	29	Single	Secondary	Private	Urban	Yes	No	No	Yes	No	No	No	No	No	No
8079	HTAR0955	Male	Chinese	2019	42	Others	No formal education	Unemployed	Urban	Yes	No	Yes	No	Yes	No	No	No	No	No
8080	HTAR0956	Male	Indian	2018	27	Single	Secondary	Private	Urban	Yes	No	Yes	Yes	No	No	No	No	No	No
8081	HTAR0957	Female	Malay	2018	44	Others	No formal education	Unemployed	Urban	Yes	No	No	No	Yes	No	No	No	No	No
8082	HTAR0958	Male	Malay	2019	62	Married	No formal education	Unemployed	Urban	Yes	No	No	No	Yes	No	No	No	No	No
8083	HTAR0959	Male	Malay	2019	35	Others	No formal education	Private	Urban	Yes	Yes	No	No	Yes	No	No	No	No	Yes
8084	HTAR0960	Male	Indian	2019	46	Single	Secondary	Private	Urban	Yes	No	No	No	Yes	No	No	No	No	No
8085	HTAR0961	Male	Others	2018	52	Married	No formal education	Private	Urban	Yes	No	Yes	No	Yes	No	No	No	No	No
8086	HTAR0962	Male	Malay	2018	28	Single	Primary	Unemployed	Urban	Yes	Yes	No	No	No	No	No	No	No	No
8087	HTAR0964	Male	Malay	2018	25	Single	No formal education	Unemployed	Urban	Yes	No	No	No	No	Yes	No	No	No	No
8088	HTAR0965	Male	Malay	2019	31	Single	No formal education	Unemployed	Urban	Yes	No	Yes	No	Yes	No	No	No	No	No
8089	HTAR0967	Male	Malay	2019	30	Single	No formal education	Self-employed	Urban	Yes	Yes	No	No	Yes	No	No	No	No	No
8090	HTAR0969	Male	Malay	2018	23	Single	Secondary	Private	Urban	Yes	No	No	Yes	No	No	No	No	No	No
8091	HTAR0970	Male	Indian	2018	39	Single	No formal education	Unemployed	Urban	Yes	No	No	No	Yes	No	No	No	No	No
8092	HTAR0971	Male	Malay	2018	31	Married	No formal education	Private	Urban	Yes	Yes	No	Yes	Yes	No	No	No	No	No
8093	HTAR0972	Male	Malay	2018	30	Single	No formal education	Unemployed	Urban	Yes	No	No	No	Yes	No	No	No	No	No
8094	HTAR0973	Male	Malay	2018	34	Single	Secondary	Unemployed	Urban	Yes	No	No	No	Yes	Yes	No	No	No	No
8095	HTAR0974	Male	Malay	2019	23	Single	Secondary	Private	Urban	Yes	No	No	No	Yes	No	No	No	No	No
8096	HTAR0975	Male	Malay	2018	33	Single	Secondary	Private	Urban	Yes	No	No	Yes	Yes	No	No	No	No	No

No	Patient ID	Gender	Ethnic group	Year of diagnosis	Age	Marital status	Education level	Occupation	Place of residence	History of psy illness	Tobacco	Alcohol	Cannabis	ATS	Inhalant	Sedative	Hallucinogens	Opioids	Kratom
8097	HTAR0976	Male	Malay	2018	41	Others	Secondary	Unemployed	Urban	Yes	No	No	No	Yes	No	No	No	No	No
8098	HTAR0977	Male	Malay	2019	29	Single	Secondary	Unemployed	Urban	Yes	No	No	No	Yes	No	No	No	No	No
8099	HTAR0978	Male	Malay	2018	22	Single	Secondary	Unemployed	Urban	Yes	No	No	Yes	Yes	No	No	No	No	No
8100	HTAR0980	Male	Malay	2018	31	Single	Secondary	Unemployed	Urban	Yes	No	No	Yes	Yes	No	No	No	No	No
8101	HTAR0981	Male	Others	2018	48	Married	Tertiary	Private	Urban	Yes	No	No	No	Yes	No	No	No	No	No
8102	HTAR0982	Male	Indian	2019	26	Married	Secondary	Private	Urban	Yes	Yes	No	No	No	No	No	No	No	No
8103	HTAR0983	Female	Malay	2019	51	Others	No formal education	Unemployed	Urban	Yes	Yes	No	No	Yes	No	No	No	No	No
8104	HTAR0984	Male	Chinese	2018	37	Single	No formal education	Unemployed	Urban	Yes	No	No	No	Yes	No	No	No	No	No
8105	HTAR0985	Female	Others	2019	32	Others	No formal education	Others	Urban	Yes	No	No	No	Yes	No	No	No	No	No
8106	HTAR0986	Female	Malay	2018	28	Others	Primary	Private	Urban	Yes	No	No	No	Yes	No	No	No	No	No
8107	HTAR0986	Female	Malay	2018	28	Others	Primary	Private	Urban	Yes	Yes	Yes	No	Yes	No	No	No	No	No
8108	HTAR0988	Female	Malay	2018	32	Others	Tertiary	Unemployed	Urban	Yes	No	No	No	Yes	No	No	No	No	No
8109	HTAR0989	Female	Malay	2019	28	Married	Secondary	Unemployed	Urban	Yes	No	No	No	Yes	No	No	No	No	No
8110	HTAR0990	Male	Others	2018	26	Single	Primary	Private	Urban	Yes	No	No	No	Yes	No	No	No	No	No
8111	HTAR0991	Female	Malay	2019	20	Single	Secondary	Unemployed	Urban	Yes	No	No	No	Yes	No	No	No	No	No
8112	HTAR0992	Male	Indian	2018	24	Married	No formal education	Unemployed	Urban	Yes	No	Yes	No	Yes	No	No	No	No	No
8113	HTAR0993	Male	Indian	2019	26	Single	No formal education	Private	Rural	Yes	No	No	No	Yes	No	No	No	No	No
8114	HTAR0996	Male	Malay	2018	41	Single	Secondary	Private	Urban	Yes	Yes	No	No	Yes	No	No	No	No	No
8115	HTAR0997	Male	Malay	2019	47	Married	No formal education	Private	Urban	Yes	No	No	No	Yes	No	No	No	Yes	No
8116	HTAR0998	Male	Others	2018	38	Single	No formal education	Self-employed	Urban	Yes	No	No	Yes	Yes	No	No	No	No	No
8117	HTAR0998	Male	Others	2018	38	Single	No formal education	Unemployed	Urban	Yes	No	No	No	Yes	No	No	No	No	No

No	Patient ID	Gender	Ethnic group	Year of diagnosis	Age	Marital status	Education level	Occupation	Place of residence	History of psy illness	Tobacco	Alcohol	Cannabis	ATS	Inhalant	Sedative	Hallucinogens	Opioids	Kratom
8118	HTAR0998	Male	Others	2018	38	Single	No formal education	Unemployed	Urban	Yes	No	No	No	No	No	No	No	No	No
8119	HTAR0999	Female	Malay	2019	41	Married	Secondary	Unemployed	Urban	Yes	No	No	No	Yes	No	No	No	No	No
8120	HTAR1000	Male	Indian	2019	42	Single	No formal education	Unemployed	Urban	Yes	No	No	Yes	No	No	No	No	No	No
8121	HTAR1001	Male	Others	2019	30	Single	No formal education	Private	Urban	Yes	No	No	No	Yes	No	No	No	No	No
8122	HTAR1002	Male	Indian	2018	38	Married	No formal education	Private	Urban	Yes	No	Yes	No	No	No	No	No	No	No
8123	HTAR1003	Female	Others	2018	33	Married	Secondary	Self-employed	Urban	Yes	Yes	No	No	Yes	No	No	No	No	No
8124	HTAR1004	Male	Indian	2018	54	Others	No formal education	Self-employed	Urban	Yes	No	Yes	No	Yes	No	No	No	No	No
8125	HTAR1005	Male	Chinese	2018	50	Married	No formal education	Unemployed	Urban	Yes	No	No	No	Yes	No	No	No	No	No
8126	HTAR1006	Male	Chinese	2019	40	Others	No formal education	Self-employed	Urban	Yes	No	No	No	No	No	No	No	No	No
8127	HTAR1007	Male	Malay	2019	28	Single	Secondary	Self-employed	Urban	Yes	No	Yes	No	Yes	No	No	No	No	No
8128	HTF0003	Male	Malay	2018	30	Single	Secondary	Unemployed	Rural	No	No	No	No	Yes	No	No	No	No	No
8129	HTF0009	Male	Malay	2018	23	Single	Secondary	Unemployed	Rural	Yes	Yes	No	No	Yes	No	No	No	No	Yes
8130	HTF0024	Male	Malay	2018	21	Single	Secondary	Unemployed	Rural	Yes	No	No	No	No	No	No	No	No	Yes
8131	HTF0041	Female	Malay	2018	17	Others	Secondary	Unemployed	Urban	Yes	No	No	No	Yes	No	No	No	No	No
8132	HTF0057	Male	Malay	2018	34	Single	Secondary	Self-employed	Rural	Yes	Yes	No	No	Yes	No	No	No	No	Yes
8133	HTF0067	Male	Malay	2021	27	Others	Tertiary	Self-employed	Urban	Yes	Yes	No	Yes	No	No	Yes	No	No	No
8134	HTF0094	Male	Malay	2018	30	Married	Secondary	Self-employed	Rural	Yes	No	No	No	Yes	No	No	No	No	Yes
8135	HTF0118	Male	Malay	2018	21	Single	Primary	Unemployed	Rural	No	Yes	No	No	No	No	No	No	No	Yes
8136	HTF0124	Male	Malay	2018	33	Others	Primary	Unemployed	Rural	Yes	No	No	No	Yes	No	No	No	Yes	No
8137	HTF0127	Male	Others	2018	28	Single	Secondary	Unemployed	Rural	Yes	Yes	No	No	No	No	No	No	No	No
8138	HTF0132	Male	Malay	2018	26	Single	Secondary	Self-employed	Rural	Yes	Yes	No	No	Yes	No	No	No	No	Yes
8139	HTF0133	Male	Malay	2019	23	Single	Secondary	Private	Rural	No	Yes	No	No	Yes	No	No	No	Yes	Yes
8140	HTF0134	Male	Malay	2018	32	Single	Primary	Unemployed	Rural	No	No	No	No	Yes	No	No	No	No	Yes
8141	HTF0137	Male	Malay	2019	32	Married	Secondary	Self-employed	Rural	Yes	No	No	No	Yes	No	No	No	No	Yes
8142	HTF0138	Male	Malay	2019	29	Others	Primary	Self-employed	Rural	Yes	No	No	No	Yes	No	No	No	No	No
8143	HTF0139	Male	Malay	2020	25	Single	Secondary	Government	Rural	Yes	Yes	No	No	Yes	No	No	No	Yes	Yes
8144	HTF0140	Male	Malay	2019	40	Married	Primary	Private	Rural	Yes	No	No	No	Yes	No	No	No	No	Yes
8145	HTF0142	Male	Malay	2019	43	Married	No formal education	Private	Rural	No	No	No	No	Yes	No	No	No	No	No
8146	HTF0145	Male	Malay	2019	33	Single	Tertiary	Unemployed	Rural	Yes	No	No	Yes	No	No	No	No	No	Yes

No	Patient ID	Gender	Ethnic group	Year of diagnosis	Age	Marital status	Education level	Occupation	Place of residence	History of psy illness	Tobacco	Alcohol	Cannabis	ATS	Inhalant	Sedative	Hallucinogens	Opioids	Kratom
8147	HTF0146	Male	Malay	2020	27	Single	Secondary	Unemployed	Rural	Yes	No	No	No	Yes	No	No	No	Yes	No
8148	HTF0147	Male	Malay	2020	41	Others	Primary	Unemployed	Rural	Yes	No	No	No	Yes	No	Yes	No	No	No
8149	HTF0149	Male	Malay	2020	40	Married	Secondary	Self-employed	Rural	Yes	Yes	No	No	Yes	No	No	No	No	Yes
8150	HTF0156	Female	Malay	2020	27	Married	Tertiary	Unemployed	Rural	Yes	Yes	No	No	Yes	No	No	No	No	No
8151	HTF0157	Male	Malay	2020	25	Single	Secondary	Unemployed	Rural	Yes	Yes	No	No	Yes	No	Yes	No	No	Yes
8152	HTF0158	Male	Malay	2020	31	Married	Secondary	Self-employed	Rural	Yes	No	No	No	Yes	No	Yes	No	No	No
8153	HTF0160	Male	Others	2020	46	Married	Secondary	Self-employed	Rural	Yes	No	No	No	Yes	No	No	No	No	Yes
8154	HTF0162	Female	Malay	2018	35	Married	Secondary	Unemployed	Rural	Yes	No	No	No	Yes	No	No	No	No	No
8155	HTF0168	Male	Malay	2018	29	Others	Secondary	Private	Rural	Yes	No	No	No	Yes	No	No	No	No	No
8156	HTF0169	Male	Malay	2018	46	Married	Secondary	Unemployed	Rural	Yes	No	No	No	Yes	No	No	No	No	Yes
8157	HTF0184	Male	Malay	2018	51	Others	Secondary	Self-employed	Rural	Yes	Yes	No	No	Yes	No	No	No	No	No
8158	HTF0188	Male	Malay	2020	20	Single	Secondary	Unemployed	Rural	No	Yes	No	No	Yes	No	No	No	No	Yes
8159	HTF0200	Male	Malay	2018	23	Single	Secondary	Private	Rural	Yes	No	No	No	No	No	No	No	No	Yes
8160	HTF0201	Male	Malay	2018	25	Single	Secondary	Unemployed	Urban	Yes	Yes	No	No	Yes	No	No	No	No	No
8161	HTF0208	Male	Malay	2019	26	Others	Secondary	Unemployed	Rural	Yes	Yes	No	No	Yes	No	No	No	No	Yes
8162	HTF0210	Male	Malay	2019	32	Married	Secondary	Self-employed	Rural	Yes	Yes	No	No	Yes	No	No	No	No	Yes
8163	HTF0215	Male	Malay	2019	29	Single	Secondary	Unemployed	Rural	No	Yes	No	No	Yes	No	No	No	No	No
8164	HTF0217	Male	Malay	2018	27	Single	Primary	Self-employed	Rural	Yes	Yes	No	No	Yes	No	No	No	No	No
8165	HTF0222	Male	Malay	2018	20	Single	Secondary	Unemployed	Rural	Yes	No	No	No	Yes	No	No	No	No	No
8166	HTF0225	Male	Malay	2019	34	Married	Secondary	Self-employed	Rural	No	Yes	No	No	Yes	No	No	No	No	No
8167	HTF0227	Male	Malay	2019	37	Married	Secondary	Self-employed	Rural	Yes	Yes	No	No	Yes	No	No	No	No	No
8168	HTF0228	Female	Malay	2019	34	Others	Secondary	Unemployed	Rural	Yes	No	No	No	Yes	No	No	No	No	No
8169	HTF0229	Male	Malay	2019	25	Single	Secondary	Unemployed	Rural	Yes	No	No	No	Yes	No	No	No	No	No
8170	HTF0231	Male	Malay	2019	25	Single	Tertiary	Private	Rural	Yes	Yes	No	No	Yes	No	No	No	No	Yes
8171	HTF0233	Male	Malay	2019	29	Single	Secondary	Private	Rural	Yes	No	No	No	Yes	No	No	No	No	No
8172	HTF0234	Male	Malay	2019	31	Married	Secondary	Self-employed	Rural	Yes	No	No	No	Yes	No	No	No	Yes	Yes
8173	HTF0235	Male	Chinese	2019	48	Married	Secondary	Self-employed	Rural	Yes	Yes	No	No	Yes	No	No	No	No	No
8174	HTF0238	Male	Malay	2019	24	Single	Secondary	Self-employed	Rural	Yes	Yes	No	No	Yes	No	No	No	No	No
8175	HTF0241	Male	Malay	2019	28	Others	Secondary	Private	Rural	Yes	No	No	No	Yes	No	No	No	No	No
8176	HTF0248	Male	Malay	2019	24	Single	Secondary	Unemployed	Rural	No	No	No	Yes	No	No	No	No	No	Yes
8177	HTF0251	Male	Malay	2019	27	Single	Secondary	Unemployed	Rural	Yes	Yes	No	No	Yes	No	No	No	Yes	Yes
8178	HTF0265	Male	Malay	2019	39	Married	Secondary	Private	Rural	No	Yes	No	No	No	No	No	No	No	Yes
8179	HTF0266	Male	Malay	2018	33	Single	Tertiary	Self-employed	Rural	Yes	No	No	No	Yes	No	No	No	Yes	No
8180	HTF0284	Male	Malay	2020	24	Single	Secondary	Self-employed	Rural	Yes	No	No	No	Yes	No	Yes	No	No	Yes
8181	HTF0285	Male	Malay	2020	22	Single	Secondary	Self-employed	Rural	Yes	Yes	No	No	No	No	No	No	No	No
8182	HTF0291	Male	Malay	2020	36	Single	Secondary	Self-employed	Rural	Yes	No	No	No	Yes	No	Yes	No	Yes	No
8183	HTF0295	Male	Malay	2020	26	Single	Secondary	Self-employed	Rural	Yes	Yes	No	No	Yes	No	No	No	No	No
8184	HTF0296	Male	Malay	2019	27	Single	Primary	Unemployed	Rural	Yes	Yes	No	No	Yes	No	No	No	Yes	Yes

No	Patient ID	Gender	Ethnic group	Year of diagnosis	Age	Marital status	Education level	Occupation	Place of residence	History of psy illness	Tobacco	Alcohol	Cannabis	ATS	Inhalant	Sedative	Hallucinogens	Opioids	Kratom
8185	HTF0299	Male	Malay	2020	23	Single	Secondary	Unemployed	Urban	Yes	Yes	No	No	Yes	No	No	No	No	No
8186	HTF0303	Female	Malay	2020	17	Married	Primary	Unemployed	Rural	Yes	No	No	No	Yes	No	No	No	No	No
8187	HTF0304	Male	Malay	2020	36	Single	Secondary	Unemployed	Rural	No	No	No	No	Yes	No	Yes	No	No	No
8188	HTF0308	Male	Malay	2020	23	Single	Secondary	Unemployed	Rural	Yes	Yes	No	No	Yes	No	No	No	No	No
8189	HTF0314	Male	Malay	2020	27	Single	Secondary	Unemployed	Rural	Yes	Yes	No	No	No	No	No	No	No	No
8190	HTF0323	Male	Malay	2018	37	Others	No formal education	Others	Rural	Yes	No	No	No	Yes	No	No	No	No	No
8191	HTF0329	Male	Malay	2020	24	Single	Secondary	Private	Rural	Yes	No	No	No	Yes	No	No	No	No	Yes
8192	HTF0333	Male	Malay	2019	25	Single	Secondary	Unemployed	Rural	Yes	No	No	No	Yes	No	No	No	No	No
8193	HTF0344	Male	Malay	2018	43	Others	No formal education	Others	Rural	Yes	No	No	No	Yes	No	No	No	No	No
8194	HTF0346	Male	Malay	2018	30	Others	No formal education	Others	Rural	Yes	No	No	No	Yes	No	No	No	No	No
8195	HTF0348	Female	Malay	2018	20	Others	No formal education	Others	Rural	Yes	No	No	No	Yes	No	No	No	No	No
8196	HTF0349	Male	Malay	2018	19	Others	No formal education	Others	Urban	Yes	No	No	No	Yes	No	No	No	No	No
8197	HTF0350	Male	Malay	2018	33	Others	No formal education	Self-employed	Rural	Yes	No	No	No	Yes	No	No	No	No	No
8198	HTF0351	Male	Malay	2018	25	Others	No formal education	Others	Rural	Yes	No	No	No	Yes	No	No	No	No	No
8199	HTF0352	Male	Malay	2018	22	Others	No formal education	Others	Rural	Yes	No	No	No	Yes	No	No	No	No	No
8200	HTF0353	Male	Malay	2018	23	Others	No formal education	Others	Rural	Yes	No	No	No	Yes	No	No	No	No	No
8201	HTF0354	Male	Malay	2018	22	Others	No formal education	Others	Rural	Yes	No	No	No	Yes	No	No	No	No	No
8202	HTF0356	Male	Malay	2018	36	Others	No formal education	Others	Rural	Yes	No	No	No	Yes	No	No	No	No	No
8203	HTF0357	Male	Malay	2018	25	Others	No formal education	Others	Rural	Yes	No	No	No	Yes	No	No	No	No	No
8204	HTF0358	Male	Malay	2018	24	Others	No formal education	Others	Urban	No	No	No	No	Yes	No	No	No	No	No
8205	HTF0359	Male	Malay	2018	29	Married	No formal education	Others	Rural	Yes	No	No	No	Yes	No	No	No	No	No
8206	HTF0362	Female	Others	2018	26	Others	No formal education	Others	Rural	Yes	No	No	No	Yes	No	No	No	No	No
8207	HTF0363	Male	Malay	2018	28	Others	No formal education	Others	Urban	Yes	No	No	No	Yes	No	No	No	No	No
8208	HTF0364	Male	Malay	2018	31	Others	No formal education	Others	Rural	No	No	No	No	Yes	No	No	No	No	No
8209	HTF0365	Male	Malay	2018	40	Others	No formal education	Others	Rural	Yes	No	No	Yes	Yes	No	No	No	No	No

No	Patient ID	Gender	Ethnic group	Year of diagnosis	Age	Marital status	Education level	Occupation	Place of residence	History of psy illness	Tobacco	Alcohol	Cannabis	ATS	Inhalant	Sedative	Hallucinogens	Opioids	Kratom
8210	HTF0366	Male	Malay	2018	31	Others	No formal education	Others	Rural	Yes	No	No	No	Yes	No	No	No	No	No
8211	HTF0367	Male	Malay	2018	24	Others	No formal education	Others	Rural	Yes	No	No	No	Yes	No	No	No	No	No
8212	HTF0370	Male	Malay	2018	39	Others	No formal education	Others	Rural	Yes	No	No	No	Yes	No	No	No	No	No
8213	HTF0371	Male	Malay	2018	26	Others	No formal education	Others	Rural	Yes	No	No	No	Yes	No	No	No	No	No
8214	HTF0372	Male	Malay	2018	20	Single	No formal education	Others	Rural	Yes	No	No	No	Yes	No	No	No	No	No
8215	HTF0373	Male	Malay	2018	33	Others	No formal education	Others	Rural	Yes	No	No	No	Yes	No	No	No	No	No
8216	HTF0374	Male	Malay	2018	24	Others	No formal education	Others	Rural	Yes	No	No	No	Yes	No	No	No	No	No
8217	HTF0375	Male	Malay	2018	22	Others	No formal education	Others	Rural	Yes	No	No	No	Yes	No	No	No	No	No
8218	HTF0376	Male	Malay	2018	24	Others	No formal education	Others	Rural	Yes	No	No	No	Yes	No	No	No	No	No
8219	HTF0378	Male	Malay	2018	36	Others	No formal education	Others	Rural	Yes	No	No	No	Yes	No	No	No	No	No
8220	HTF0379	Male	Malay	2018	38	Others	No formal education	Others	Rural	Yes	No	No	No	Yes	No	No	No	No	No
8221	HTF0380	Male	Malay	2018	36	Others	No formal education	Others	Rural	Yes	No	No	No	Yes	No	No	No	No	No
8222	HTF0381	Male	Malay	2018	21	Others	No formal education	Others	Rural	Yes	No	No	No	Yes	No	No	No	No	No
8223	HTF0382	Male	Malay	2018	41	Others	No formal education	Others	Rural	Yes	No	No	No	Yes	No	No	No	Yes	No
8224	HTF0383	Male	Malay	2018	26	Others	No formal education	Others	Rural	Yes	No	No	No	Yes	No	No	No	No	No
8225	HTF0385	Male	Malay	2018	36	Others	No formal education	Others	Rural	Yes	No	No	No	Yes	No	No	No	No	No
8226	HTF0386	Male	Malay	2019	37	Others	No formal education	Others	Rural	Yes	No	No	No	Yes	No	No	No	No	No
8227	HTF0387	Male	Malay	2019	50	Others	No formal education	Others	Rural	Yes	No	No	Yes	No	No	No	No	No	No
8228	HTF0388	Male	Malay	2019	29	Others	No formal education	Others	Rural	Yes	No	No	No	Yes	No	No	No	Yes	No
8229	HTF0389	Male	Malay	2019	28	Others	No formal education	Others	Rural	Yes	No	No	No	Yes	No	No	No	Yes	No
8230	HTF0390	Male	Malay	2019	36	Others	No formal education	Others	Rural	Yes	No	No	No	No	No	No	No	Yes	No

No	Patient ID	Gender	Ethnic group	Year of diagnosis	Age	Marital status	Education level	Occupation	Place of residence	History of psy illness	Tobacco	Alcohol	Cannabis	ATS	Inhalant	Sedative	Hallucinogens	Opioids	Kratom
8231	HTF0391	Male	Malay	2019	30	Others	No formal education	Others	Rural	Yes	No	No	No	No	No	No	No	Yes	No
8232	HTF0392	Male	Malay	2019	25	Others	No formal education	Others	Rural	Yes	No	No	No	Yes	No	No	No	No	No
8233	HTF0393	Male	Malay	2019	26	Others	No formal education	Others	Rural	Yes	No	No	No	Yes	No	No	No	No	No
8234	HTF0394	Male	Malay	2019	28	Others	No formal education	Others	Rural	Yes	No	No	No	Yes	No	No	No	No	No
8235	HTF0395	Male	Malay	2019	53	Others	No formal education	Others	Rural	Yes	No	No	Yes	No	No	No	No	No	No
8236	HTF0396	Male	Malay	2019	45	Others	No formal education	Others	Rural	Yes	No	No	No	Yes	No	No	No	No	No
8237	HTF0398	Male	Others	2019	52	Others	No formal education	Others	Rural	Yes	No	No	No	Yes	No	No	No	Yes	No
8238	HTF0399	Male	Malay	2019	38	Others	No formal education	Others	Rural	Yes	No	No	No	Yes	No	No	No	No	No
8239	HTF0400	Male	Malay	2019	43	Others	No formal education	Others	Rural	Yes	No	No	No	No	No	No	No	Yes	No
8240	HTF0401	Male	Malay	2019	28	Others	No formal education	Others	Rural	Yes	No	No	No	Yes	No	No	No	No	No
8241	HTF0402	Male	Malay	2019	35	Others	No formal education	Others	Rural	Yes	No	No	No	Yes	No	No	No	No	No
8242	HTF0403	Male	Malay	2019	46	Others	No formal education	Others	Rural	Yes	No	No	No	Yes	No	No	No	No	No
8243	HTF0404	Male	Malay	2019	30	Others	No formal education	Others	Rural	Yes	No	No	No	Yes	No	No	No	No	No
8244	HTF0405	Male	Malay	2019	19	Others	No formal education	Others	Rural	Yes	No	No	No	Yes	No	No	No	No	No
8245	HTF0406	Male	Malay	2019	20	Others	No formal education	Others	Rural	Yes	No	No	No	Yes	No	No	No	No	No
8246	HTF0407	Male	Malay	2019	37	Others	No formal education	Others	Rural	Yes	No	No	No	Yes	No	No	No	No	No
8247	HTF0408	Male	Malay	2019	47	Others	No formal education	Others	Rural	Yes	No	No	No	Yes	No	No	No	No	No
8248	HTF0409	Female	Malay	2019	39	Others	No formal education	Others	Rural	Yes	No	No	No	Yes	No	No	No	No	No
8249	HTF0410	Male	Malay	2019	31	Others	No formal education	Others	Rural	Yes	No	No	No	Yes	No	No	No	No	No
8250	HTF0412	Male	Malay	2019	18	Others	No formal education	Others	Rural	Yes	No	No	No	Yes	No	No	No	No	No
8251	HTF0413	Male	Malay	2019	23	Others	No formal education	Others	Rural	Yes	No	No	No	Yes	No	No	No	Yes	Yes

No	Patient ID	Gender	Ethnic group	Year of diagnosis	Age	Marital status	Education level	Occupation	Place of residence	History of psy illness	Tobacco	Alcohol	Cannabis	ATS	Inhalant	Sedative	Hallucinogens	Opioids	Kratom
8252	HTF0414	Male	Malay	2019	28	Others	No formal education	Others	Rural	Yes	No	No	No	Yes	No	No	No	No	No
8253	HTF0416	Male	Malay	2019	24	Others	No formal education	Others	Rural	Yes	No	No	No	Yes	No	No	No	No	No
8254	HTF0417	Male	Malay	2019	22	Others	Secondary	Others	Rural	Yes	No	No	No	Yes	No	No	No	No	No
8255	HTF0418	Male	Malay	2019	21	Others	No formal education	Others	Rural	Yes	No	No	Yes	No	No	No	No	No	Yes
8256	HTF0419	Male	Malay	2019	18	Others	No formal education	Others	Rural	Yes	No	No	No	Yes	No	No	No	No	No
8257	HTF0420	Male	Malay	2019	42	Others	No formal education	Others	Rural	Yes	No	No	No	Yes	No	No	No	No	No
8258	HTF0423	Male	Malay	2018	36	Single	Primary	Unemployed	Rural	No	No	No	Yes	Yes	No	No	No	No	No
8259	HTF0424	Male	Malay	2018	26	Others	Primary	Others	Rural	No	No	No	No	Yes	No	No	No	No	No
8260	HTF0425	Male	Chinese	2021	26	Single	Secondary	Private	Rural	Yes	No	No	No	Yes	No	No	No	No	No
8261	HTF0427	Male	Malay	2020	30	Single	Secondary	Private	Rural	Yes	No	No	No	Yes	No	No	No	No	No
8262	HTF0428	Male	Malay	2018	39	Married	Secondary	Self-employed	Rural	No	Yes	No	No	Yes	No	No	No	Yes	Yes
8263	HTF0429	Male	Malay	2020	23	Single	Secondary	Others	Rural	Yes	Yes	No	Yes	Yes	No	No	No	No	No
8264	HTF0430	Male	Malay	2020	42	Single	Secondary	Unemployed	Rural	Yes	No	No	No	No	No	Yes	No	No	Yes
8265	HTF0431	Female	Malay	2018	28	Others	Secondary	Unemployed	Rural	No	No	No	No	Yes	No	No	No	No	No
8266	HTF0432	Male	Malay	2020	27	Others	Secondary	Unemployed	Rural	No	Yes	No	Yes	Yes	No	Yes	No	Yes	No
8267	HTF0436	Male	Others	2021	38	Single	Secondary	Unemployed	Rural	Yes	No	Yes	No	Yes	No	No	No	No	No
8268	HTF0437	Male	Malay	2021	28	Others	Secondary	Self-employed	Rural	No	No	No	No	Yes	No	No	No	No	Yes
8269	HTF0438	Male	Malay	2021	35	Single	Secondary	Self-employed	Rural	No	Yes	No	No	Yes	No	No	No	No	No
8270	HTF0439	Male	Malay	2021	32	Single	Secondary	Unemployed	Rural	No	No	No	No	Yes	No	No	No	No	No
8271	HTF0440	Male	Malay	2021	30	Single	Secondary	Self-employed	Rural	Yes	No	No	No	Yes	No	No	No	No	No
8272	HTF0441	Male	Malay	2021	30	Others	Secondary	Unemployed	Rural	Yes	No	No	No	Yes	No	No	No	No	No
8273	HTF0442	Male	Malay	2021	34	Single	Primary	Unemployed	Rural	No	No	No	No	Yes	No	No	No	No	No
8274	HTF0447	Male	Malay	2020	36	Married	Secondary	Unemployed	Rural	Yes	No	No	No	No	No	No	No	No	Yes
8275	HTF0456	Female	Malay	2020	27	Others	No formal education	Others	Rural	Yes	No	No	No	Yes	No	No	No	No	No
8276	HTF0457	Female	Malay	2020	32	Married	Secondary	Others	Rural	Yes	No	No	No	Yes	No	No	No	No	No
8277	HTF0458	Female	Malay	2020	35	Married	No formal education	Others	Rural	Yes	No	No	No	Yes	No	No	No	No	No
8278	HTF0459	Male	Others	2020	42	Married	No formal education	Others	Rural	Yes	No	No	No	Yes	No	No	No	No	No
8279	HTF0460	Male	Malay	2020	32	Married	No formal education	Others	Rural	Yes	No	No	No	Yes	No	No	No	Yes	No
8280	HTF0461	Male	Malay	2020	35	Others	No formal education	Others	Rural	Yes	No	No	No	Yes	No	No	No	Yes	No
8281	HTF0462	Male	Malay	2020	52	Married	No formal education	Others	Rural	Yes	No	No	No	Yes	No	No	No	No	No
8282	HTF0463	Male	Malay	2020	39	Others	Secondary	Government	Rural	Yes	No	No	No	Yes	No	No	No	No	No
8283	HTF0465	Male	Malay	2020	22	Others	No formal education	Others	Rural	Yes	No	No	No	Yes	No	No	No	No	No

No	Patient ID	Gender	Ethnic group	Year of diagnosis	Age	Marital status	Education level	Occupation	Place of residence	History of psy illness	Tobacco	Alcohol	Cannabis	ATS	Inhalant	Sedative	Hallucinogens	Opioids	Kratom
8284	HTF0466	Male	Malay	2020	35	Others	No formal education	Others	Rural	Yes	No	No	No	No	No	No	No	Yes	No
8285	HTF0467	Male	Malay	2020	40	Others	No formal education	Others	Rural	Yes	No	No	No	Yes	No	Yes	No	Yes	No
8286	HTF0468	Male	Malay	2020	35	Others	No formal education	Others	Rural	Yes	No	No	No	Yes	No	No	No	No	No
8287	HTF0469	Male	Malay	2020	23	Others	No formal education	Others	Rural	Yes	No	No	No	Yes	No	No	No	Yes	Yes
8288	HTF0470	Male	Malay	2020	22	Others	No formal education	Others	Rural	Yes	No	No	No	Yes	No	No	No	No	No
8289	HTF0471	Male	Malay	2020	53	Others	No formal education	Others	Rural	Yes	No	No	No	Yes	No	Yes	No	Yes	No
8290	HTF0472	Male	Malay	2020	26	Others	No formal education	Others	Rural	Yes	No	No	No	Yes	No	No	No	No	No
8291	HTF0473	Male	Malay	2020	37	Others	No formal education	Others	Rural	Yes	No	No	No	Yes	No	No	No	No	No
8292	HTF0474	Male	Malay	2020	58	Married	No formal education	Others	Rural	Yes	No	No	No	Yes	No	No	No	No	No
8293	HTF0475	Female	Malay	2020	42	Married	No formal education	Others	Rural	Yes	No	No	No	Yes	No	No	No	No	No
8294	HTF0476	Male	Malay	2020	35	Married	Secondary	Others	Rural	Yes	No	No	No	Yes	No	No	No	No	No
8295	HTF0477	Male	Malay	2020	35	Others	No formal education	Others	Rural	Yes	No	No	No	Yes	No	No	No	No	No
8296	HTF0478	Male	Malay	2020	40	Others	No formal education	Private	Rural	Yes	No	No	No	Yes	No	No	No	No	No
8297	HTF0479	Male	Malay	2020	27	Others	No formal education	Others	Rural	Yes	No	No	No	Yes	No	No	No	No	No
8298	HTF0480	Male	Malay	2020	28	Others	No formal education	Others	Rural	Yes	No	No	No	Yes	No	No	No	No	No
8299	HTF0481	Male	Malay	2020	53	Others	No formal education	Others	Rural	Yes	No	No	No	Yes	No	No	No	No	No
8300	HTF0482	Male	Malay	2020	36	Others	No formal education	Others	Rural	Yes	Yes	No	No	Yes	No	No	No	No	No
8301	HTF0483	Male	Malay	2020	28	Others	No formal education	Others	Rural	Yes	No	No	No	Yes	No	No	No	No	No
8302	HTF0484	Male	Malay	2020	25	Others	No formal education	Others	Rural	Yes	No	No	No	Yes	No	No	No	No	No
8303	HTF0485	Male	Others	2020	38	Others	No formal education	Others	Rural	Yes	No	No	No	Yes	No	No	No	No	No
8304	HTF0486	Male	Malay	2020	24	Others	No formal education	Others	Rural	Yes	No	No	No	Yes	No	No	No	No	No
8305	HTF0487	Male	Malay	2020	32	Others	No formal education	Others	Rural	Yes	No	No	No	Yes	No	No	No	No	No

No	Patient ID	Gender	Ethnic group	Year of diagnosis	Age	Marital status	Education level	Occupation	Place of residence	History of psy illness	Tobacco	Alcohol	Cannabis	ATS	Inhalant	Sedative	Hallucinogens	Opioids	Kratom
8306	HTF0488	Male	Malay	2020	20	Others	No formal education	Others	Rural	Yes	No	No	No	Yes	No	No	No	No	No
8307	HTF0490	Male	Malay	2020	52	Married	No formal education	Others	Rural	Yes	No	No	No	Yes	No	No	No	No	No
8308	HTF0491	Male	Malay	2020	46	Others	No formal education	Others	Rural	Yes	No	No	No	No	No	No	No	Yes	No
8309	HTF0492	Male	Malay	2020	20	Single	No formal education	Others	Rural	Yes	No	No	No	Yes	No	No	No	No	No
8310	HTF0493	Male	Malay	2020	35	Others	No formal education	Others	Rural	Yes	No	No	No	Yes	No	No	No	No	No
8311	HTF0494	Male	Malay	2020	25	Others	No formal education	Others	Rural	Yes	Yes	No	No	Yes	No	No	No	No	No
8312	HTF0495	Male	Malay	2020	18	Single	No formal education	Others	Rural	Yes	No	No	No	Yes	No	No	No	No	No
8313	HTF0496	Male	Malay	2020	35	Others	No formal education	Others	Rural	Yes	No	No	No	Yes	No	No	No	No	No
8314	HTF0498	Male	Malay	2020	27	Others	No formal education	Others	Rural	Yes	No	No	No	Yes	No	No	No	No	No
8315	HTF0499	Male	Malay	2020	22	Single	Secondary	Unemployed	Rural	Yes	No	No	No	Yes	No	No	No	No	No
8316	HTF0500	Male	Malay	2020	25	Others	No formal education	Others	Rural	Yes	No	No	No	Yes	No	No	No	No	No
8317	HTF0501	Male	Malay	2020	18	Others	No formal education	Others	Rural	Yes	No	No	No	Yes	No	No	No	No	No
8318	HTF0502	Male	Malay	2020	31	Others	No formal education	Others	Rural	Yes	No	No	No	Yes	No	No	No	No	No
8319	HTF0503	Male	Malay	2020	28	Others	No formal education	Others	Rural	Yes	Yes	No	No	Yes	No	No	No	Yes	No
8320	HTF0504	Male	Malay	2020	23	Others	No formal education	Others	Rural	Yes	Yes	No	No	Yes	No	No	No	No	No
8321	HTF0505	Male	Malay	2020	38	Others	No formal education	Others	Rural	Yes	No	No	No	No	No	No	No	Yes	No
8322	HTF0506	Male	Malay	2020	26	Others	No formal education	Others	Rural	Yes	No	No	No	Yes	No	No	No	No	No
8323	HTF0507	Male	Malay	2020	33	Others	No formal education	Others	Rural	Yes	No	No	No	Yes	No	No	No	No	No
8324	HTF0508	Male	Malay	2020	23	Others	No formal education	Others	Rural	Yes	No	No	No	Yes	No	No	No	No	No
8325	HTF0509	Male	Malay	2020	23	Others	No formal education	Others	Rural	Yes	Yes	No	No	Yes	No	No	No	No	No
8326	HTF0510	Male	Malay	2020	20	Single	Secondary	Unemployed	Rural	Yes	No	No	No	Yes	No	No	No	No	No
8327	HTF0511	Male	Malay	2020	21	Others	No formal education	Unemployed	Rural	Yes	No	No	No	Yes	No	No	No	No	No

No	Patient ID	Gender	Ethnic group	Year of diagnosis	Age	Marital status	Education level	Occupation	Place of residence	History of psy illness	Tobacco	Alcohol	Cannabis	ATS	Inhalant	Sedative	Hallucinogens	Opioids	Kratom
8328	HTF0512	Male	Malay	2020	39	Others	No formal education	Others	Rural	Yes	No	No	No	Yes	No	No	No	No	No
8329	HTF0513	Male	Malay	2020	31	Others	No formal education	Others	Rural	Yes	Yes	No	No	Yes	No	No	No	No	No
8330	HTF0514	Male	Malay	2020	30	Others	No formal education	Others	Rural	Yes	Yes	No	No	Yes	No	No	No	No	No
8331	HTF0515	Male	Malay	2020	33	Others	No formal education	Others	Rural	Yes	No	No	No	Yes	No	No	No	No	No
8332	HTF0516	Male	Malay	2020	41	Others	No formal education	Unemployed	Rural	Yes	No	No	No	Yes	No	No	No	No	No
8333	HTF0517	Male	Malay	2020	35	Others	No formal education	Others	Rural	Yes	No	No	No	Yes	No	No	No	No	No
8334	HTF0518	Male	Malay	2020	41	Others	No formal education	Others	Rural	Yes	Yes	No	No	Yes	No	No	No	No	No
8335	HTF0519	Male	Malay	2020	25	Others	No formal education	Others	Rural	Yes	No	No	No	Yes	No	No	No	No	No
8336	HTF0520	Male	Malay	2020	48	Married	No formal education	Others	Rural	Yes	No	No	No	Yes	No	No	No	No	No
8337	HTF0521	Male	Malay	2020	34	Others	No formal education	Others	Rural	Yes	No	No	No	Yes	No	No	No	No	No
8338	HTF0522	Male	Malay	2020	36	Others	No formal education	Others	Rural	Yes	Yes	No	No	Yes	No	No	No	No	No
8339	HTF0523	Male	Malay	2020	36	Others	Secondary	Others	Rural	No	No	No	No	Yes	No	No	No	No	No
8340	HTF0524	Male	Malay	2021	22	Single	No formal education	Others	Rural	Yes	No	No	No	Yes	No	No	No	No	No
8341	HTF0525	Male	Malay	2021	32	Single	No formal education	Others	Rural	Yes	No	No	No	Yes	No	No	No	No	No
8342	HTF0526	Male	Malay	2021	25	Others	No formal education	Others	Rural	Yes	No	No	No	Yes	No	No	No	No	No
8343	HTF0527	Male	Malay	2021	22	Others	No formal education	Others	Rural	Yes	No	No	No	Yes	No	No	No	No	No
8344	HTF0528	Male	Malay	2021	34	Others	No formal education	Others	Rural	Yes	No	No	No	Yes	No	No	No	No	No
8345	HTF0530	Male	Malay	2021	36	Others	No formal education	Others	Rural	Yes	No	No	No	Yes	No	No	No	No	No
8346	HTF0531	Male	Indian	2021	33	Others	No formal education	Others	Rural	Yes	No	No	No	Yes	No	No	No	No	No
8347	HTF0532	Male	Malay	2021	21	Others	No formal education	Others	Rural	Yes	Yes	No	No	Yes	No	No	No	No	No
8348	HTF0533	Male	Malay	2021	21	Others	No formal education	Others	Rural	Yes	Yes	No	No	Yes	No	No	No	No	No
8349	HTF0534	Male	Malay	2021	17	Single	Primary	Unemployed	Rural	Yes	No	No	No	Yes	No	No	No	No	No
8350	HTF0535	Male	Malay	2021	22	Single	Primary	Others	Rural	Yes	No	No	No	Yes	No	No	No	No	No

No	Patient ID	Gender	Ethnic group	Year of diagnosis	Age	Marital status	Education level	Occupation	Place of residence	History of psy illness	Tobacco	Alcohol	Cannabis	ATS	Inhalant	Sedative	Hallucinogens	Opioids	Kratom
8351	HTF0536	Male	Malay	2021	32	Others	No formal education	Others	Rural	Yes	No	No	No	Yes	No	No	No	No	No
8352	HTF0537	Male	Malay	2021	42	Married	No formal education	Self-employed	Rural	Yes	No	No	No	Yes	No	No	No	No	No
8353	HTF0538	Male	Malay	2021	34	Others	No formal education	Others	Rural	Yes	No	No	No	Yes	No	No	No	No	No
8354	HTF0540	Male	Malay	2021	32	Married	Secondary	Private	Rural	Yes	Yes	No	No	Yes	No	No	No	No	No
8355	HTF0541	Male	Malay	2021	18	Single	No formal education	Unemployed	Rural	Yes	No	No	No	Yes	No	No	No	No	No
8356	HTF0542	Male	Malay	2021	30	Married	No formal education	Others	Rural	Yes	No	No	No	Yes	No	No	No	No	No
8357	HTF0542	Male	Malay	2021	32	Others	No formal education	Others	Rural	Yes	Yes	No	No	Yes	No	No	No	No	Yes
8358	HTF0545	Male	Malay	2021	34	Others	No formal education	Others	Rural	Yes	No	No	No	Yes	No	No	No	No	No
8359	HTF0546	Male	Malay	2021	28	Others	No formal education	Others	Rural	Yes	Yes	No	No	Yes	No	No	No	No	No
8360	HTF0547	Male	Malay	2021	31	Others	No formal education	Others	Rural	Yes	No	No	No	Yes	No	No	No	No	No
8361	HTF0548	Female	Malay	2021	35	Others	No formal education	Others	Rural	Yes	No	No	No	Yes	No	No	No	No	No
8362	HTF0549	Female	Malay	2021	35	Others	No formal education	Others	Rural	Yes	No	No	No	Yes	No	No	No	No	No
8363	HTF0550	Male	Malay	2021	22	Others	No formal education	Others	Rural	Yes	No	No	No	Yes	No	No	No	No	No
8364	HTF0551	Male	Malay	2021	23	Others	No formal education	Others	Rural	Yes	No	No	No	Yes	No	No	No	No	No
8365	HTF0552	Male	Malay	2021	59	Others	No formal education	Unemployed	Rural	Yes	Yes	No	No	Yes	No	No	No	No	No
8366	HTF0553	Male	Malay	2021	22	Single	Secondary	Unemployed	Rural	Yes	No	No	No	Yes	No	No	No	No	No
8367	HTJ0001	Male	Chinese	2021	34	Married	No formal education	Private	Urban	Yes	Yes	No	Yes	Yes	No	No	No	No	No
8368	HTJ0002	Female	Chinese	2019	34	Others	Primary	Private	Urban	Yes	Yes	Yes	Yes	Yes	Yes	Yes	Yes	Yes	Yes
8369	HTJ0006	Male	Indian	2020	25	Single	Tertiary	Unemployed	Urban	No	Yes	Yes	Yes	No	No	No	No	No	No
8370	HTJ0007	Male	Indian	2020	24	Single	Secondary	Self-employed	Urban	Yes	Yes	Yes	No	No	No	No	No	No	No
8371	HTJ0009	Male	Indian	2021	46	Married	No formal education	Private	Urban	Yes	Yes	Yes	Yes	No	No	No	No	No	No
8372	HTJ0010	Male	Malay	2020	22	Others	Secondary	Unemployed	Urban	Yes	Yes	No	No	Yes	No	No	No	No	No
8373	HTJ0011	Male	Indian	2021	28	Single	Secondary	Private	Urban	Yes	Yes	No	Yes	Yes	No	No	No	No	No
8374	HTJ0012	Male	Indian	2021	48	Married	Primary	Private	Urban	Yes	Yes	Yes	No	No	No	No	No	No	No

No	Patient ID	Gender	Ethnic group	Year of diagnosis	Age	Marital status	Education level	Occupation	Place of residence	History of psy illness	Tobacco	Alcohol	Cannabis	ATS	Inhalant	Sedative	Hallucinogens	Opioids	Kratom
8375	HTJ0014	Male	Indian	2021	37	Single	Primary	Unemployed	Urban	No	Yes	Yes	Yes	Yes	Yes	Yes	Yes	Yes	Yes
8376	HTJ0015	Male	Malay	2020	32	Single	Secondary	Unemployed	Urban	No	Yes	No	No	Yes	No	No	No	No	No
8377	HTJ0017	Male	Malay	2021	18	Single	Primary	Private	Rural	Yes	Yes	Yes	Yes	Yes	Yes	Yes	Yes	Yes	Yes
8378	HTJ0018	Female	Malay	2020	16	Single	Primary	Unemployed	Rural	Yes	Yes	Yes	Yes	Yes	Yes	Yes	Yes	Yes	Yes
8379	HTJ0020	Male	Indian	2021	28	Married	Secondary	Private	Rural	No	Yes	Yes	Yes	Yes	Yes	Yes	Yes	Yes	Yes
8380	HTJ0021	Male	Chinese	2021	54	Married	Primary	Private	Rural	Yes	Yes	Yes	Yes	Yes	Yes	Yes	Yes	Yes	Yes
8381	HTJ0022	Male	Indian	2020	28	Single	No formal education	Unemployed	Urban	Yes	Yes	Yes	Yes	Yes	Yes	Yes	Yes	Yes	Yes
8382	HTJ0023	Male	Indian	2021	34	Married	No formal education	Private	Urban	No	Yes	Yes	No	No	No	No	No	No	No
8383	HTJ0024	Male	Indian	2021	48	Married	Primary	Private	Rural	Yes	Yes	Yes	Yes	Yes	Yes	Yes	Yes	Yes	Yes
8384	HTJ0025	Male	Indian	2020	49	Single	No formal education	Private	Urban	No	Yes	Yes	No	No	No	No	No	No	No
8385	HTJ0027	Male	Indian	2020	59	Married	No formal education	Private	Rural	Yes	Yes	Yes	Yes	Yes	Yes	Yes	Yes	Yes	Yes
8386	HTJ0028	Male	Indian	2020	44	Married	No formal education	Private	Urban	Yes	Yes	Yes	No	No	No	No	No	No	No
8387	HTJ0030	Male	Indian	2018	35	Married	Primary	Private	Rural	Yes	No	Yes	Yes	Yes	Yes	Yes	Yes	Yes	Yes
8388	HTJ0031	Male	Malay	2018	37	Single	Secondary	Unemployed	Urban	No	Yes	No	Yes	Yes	No	No	No	No	No
8389	HTJ0032	Male	Indian	2018	51	Others	Tertiary	Government	Urban	Yes	No	Yes	Yes	Yes	Yes	Yes	Yes	Yes	Yes
8390	HTJ0035	Male	Indian	2020	24	Others	Primary	Private	Rural	Yes	Yes	Yes	Yes	Yes	Yes	Yes	Yes	Yes	Yes
8391	HTJ0036	Male	Chinese	2020	37	Married	No formal education	Unemployed	Rural	Yes	Yes	Yes	Yes	Yes	Yes	Yes	Yes	Yes	Yes
8392	HTJ0038	Female	Malay	2020	48	Married	No formal education	Others	Urban	Yes	No	No	No	No	No	No	No	Yes	No
8393	HTJ0039	Male	Indian	2018	47	Married	No formal education	Private	Urban	Yes	No	Yes	Yes	Yes	Yes	Yes	Yes	Yes	Yes
8394	HTJ0040	Male	Chinese	2021	48	Others	Secondary	Private	Rural	Yes	Yes	Yes	Yes	Yes	Yes	Yes	Yes	Yes	Yes
8395	HTJ0042	Male	Malay	2020	28	Single	Secondary	Unemployed	Urban	No	Yes	No	Yes	Yes	No	No	No	No	No
8396	HTJ0045	Male	Malay	2020	26	Single	Tertiary	Private	Urban	No	Yes	No	Yes	No	No	No	No	No	No
8397	HTJ0046	Male	Indian	2020	39	Married	Secondary	Unemployed	Rural	Yes	No	Yes	No	Yes	No	No	No	Yes	No
8398	HTJ0047	Male	Indian	2018	47	Married	Primary	Private	Urban	Yes	No	Yes	No	No	No	No	No	No	No
8399	HTJ0048	Male	Others	2020	28	Single	Tertiary	Private	Rural	Yes	No	Yes	No	Yes	No	Yes	No	No	No
8400	HTJ0049	Female	Malay	2020	48	Married	No formal education	Unemployed	Rural	Yes	No	No	No	No	No	Yes	No	Yes	No
8401	HTJ0057	Male	Malay	2020	44	Others	Secondary	Unemployed	Rural	Yes	No	No	No	Yes	No	Yes	No	No	No
8402	HTJ0058	Male	Malay	2020	26	Single	Tertiary	Government	Rural	Yes	No	No	Yes	No	No	No	No	No	No

No	Patient ID	Gender	Ethnic group	Year of diagnosis	Age	Marital status	Education level	Occupation	Place of residence	History of psy illness	Tobacco	Alcohol	Cannabis	ATS	Inhalant	Sedative	Hallucinogens	Opioids	Kratom
8403	HTJ0059	Male	Chinese	2018	53	Single	No formal education	Self-employed	Rural	Yes	Yes	Yes	No	Yes	No	Yes	No	Yes	No
8404	HTJ0060	Male	Chinese	2019	53	Others	Primary	Self-employed	Rural	Yes	No	Yes	No	No	No	Yes	No	No	Yes
8405	HTJ0061	Male	Indian	2020	50	Married	Secondary	Private	Rural	No	No	Yes	No	No	No	Yes	No	No	No
8406	HTJ0062	Male	Indian	2020	45	Married	Primary	Private	Urban	Yes	No	Yes	No	No	No	Yes	No	No	No
8407	HTJ0066	Male	Malay	2020	46	Single	Secondary	Unemployed	Rural	No	No	No	No	Yes	No	No	No	Yes	Yes
8408	HTJ0067	Male	Malay	2019	20	Single	Secondary	Private	Rural	Yes	No	No	No	Yes	No	No	No	No	Yes
8409	HTJ0068	Male	Malay	2021	41	Others	Tertiary	Unemployed	Rural	Yes	No	Yes	No	Yes	No	No	No	No	No
8410	HTJ0069	Male	Indian	2020	57	Married	Primary	Private	Rural	Yes	No	Yes	No	No	No	No	No	No	No
8411	HTJ0070	Male	Malay	2020	32	Married	Secondary	Private	Rural	Yes	No	No	Yes	Yes	No	No	No	No	No
8412	HTJ0073	Male	Malay	2020	17	Single	Primary	Private	Urban	No	No	No	No	No	No	No	No	No	Yes
8413	HTJ0076	Male	Malay	2020	39	Others	Secondary	Unemployed	Urban	Yes	No	No	No	Yes	No	No	No	Yes	Yes
8414	HTJ0077	Male	Malay	2021	22	Single	Tertiary	Private	Rural	Yes	No	Yes	No	Yes	No	No	No	No	No
8415	HTJ0081	Male	Malay	2021	31	Single	Tertiary	Private	Urban	Yes	No	No	No	Yes	No	Yes	No	Yes	No
8416	HTJ0084	Male	Indian	2020	29	Others	No formal education	Unemployed	Urban	Yes	No	No	Yes	Yes	No	No	No	No	No
8417	HTJ0090	Male	Indian	2020	46	Married	Primary	Unemployed	Rural	Yes	No	Yes	No	No	No	No	No	No	No
8418	HTJ0091	Female	Malay	2019	32	Others	Tertiary	Unemployed	Urban	No	No	No	No	Yes	No	No	No	No	No
8419	HTJ0096	Male	Malay	2019	32	Married	Tertiary	Unemployed	Urban	No	No	No	No	No	No	No	No	No	No
8420	HTJ0097	Male	Malay	2020	34	Married	Secondary	Private	Urban	No	No	No	No	No	No	No	No	No	Yes
8421	HTJ0098	Male	Indian	2020	38	Single	Secondary	Private	Rural	Yes	No	Yes	No	No	No	No	No	No	No
8422	HTJ0099	Male	Chinese	2020	64	Others	No formal education	Self-employed	Rural	No	No	Yes	No	No	No	No	No	No	No
8423	HTJ0100	Male	Indian	2020	35	Single	No formal education	Private	Rural	Yes	No	Yes	No	No	No	No	No	No	No
8424	HTJ0101	Male	Chinese	2020	37	Single	Primary	Self-employed	Rural	Yes	Yes	Yes	No	No	No	Yes	No	No	No
8425	HTJ0102	Male	Malay	2021	28	Others	Tertiary	Private	Rural	Yes	No	No	No	Yes	No	No	No	No	No
8426	HTJ0103	Male	Indian	2020	25	Single	Tertiary	Others	Urban	No	No	No	Yes	No	No	No	No	No	Yes
8427	HTJ0104	Male	Others	2019	44	Married	No formal education	Self-employed	Urban	No	No	Yes	No	No	No	No	No	No	No
8428	HTJ0105	Male	Malay	2020	27	Single	Tertiary	Government	Rural	No	No	No	No	Yes	No	No	No	No	No

No	Patient ID	Gender	Ethnic group	Year of diagnosis	Age	Marital status	Education level	Occupation	Place of residence	History of psy illness	Tobacco	Alcohol	Cannabis	ATS	Inhalant	Sedative	Hallucinogens	Opioids	Kratom
8429	HTJ0106	Male	Indian	2020	61	Married	No formal education	Others	Urban	No	No	Yes	No	No	No	No	No	No	No
8430	HTJ0109	Male	Indian	2020	49	Single	Primary	Self-employed	Rural	No	No	Yes	No	No	No	No	No	No	No
8431	HTJ0110	Male	Indian	2021	29	Married	Primary	Private	Rural	No	No	Yes	No	No	No	Yes	No	No	No
8432	HTJ0111	Male	Indian	2018	47	Married	Primary	Private	Rural	No	Yes	Yes	No	No	No	Yes	No	No	Yes
8433	HTJ0113	Male	Chinese	2019	56	Single	Primary	Self-employed	Rural	Yes	No	Yes	Yes	No	No	Yes	No	No	No
8434	HTJ0114	Female	Others	2021	40	Married	No formal education	Unemployed	Rural	No	Yes	No	No	Yes	No	Yes	No	Yes	No
8435	HTJ0115	Male	Malay	2020	56	Others	No formal education	Self-employed	Urban	No	Yes	Yes	No	Yes	No	No	No	No	No
8436	HTJ0116	Male	Malay	2021	35	Married	Primary	Unemployed	Rural	Yes	Yes	No	No	Yes	No	Yes	No	No	No
8437	HTJ0117	Female	Malay	2018	29	Others	Secondary	Self-employed	Urban	Yes	No	Yes	Yes	Yes	No	No	Yes	No	No
8438	HTJ0119	Male	Chinese	2021	57	Married	No formal education	Self-employed	Rural	No	No	No	No	No	No	No	No	Yes	No
8439	HTJ0120	Male	Indian	2019	47	Married	Primary	Self-employed	Rural	No	No	Yes	No	No	No	No	No	No	No
8440	HTJ0121	Male	Indian	2021	62	Married	No formal education	Unemployed	Urban	No	No	Yes	No	No	No	No	No	No	No
8441	HTJ0122	Male	Indian	2020	38	Single	No formal education	Private	Rural	No	Yes	Yes	No	No	No	No	No	No	No
8442	HTJ0123	Male	Malay	2021	21	Single	Secondary	Unemployed	Urban	No	Yes	Yes	Yes	Yes	No	No	No	No	No
8443	HTJ0125	Male	Malay	2021	47	Married	Tertiary	Unemployed	Urban	No	No	No	No	Yes	No	No	No	No	No
8444	HTJ0126	Male	Chinese	2021	56	Others	No formal education	Others	Urban	No	Yes	No	No	No	No	Yes	No	No	No
8445	HTJ0128	Male	Malay	2018	33	Single	Secondary	Unemployed	Rural	Yes	Yes	No	Yes	Yes	No	Yes	No	Yes	No
8446	HTJ0129	Male	Indian	2020	32	Others	Primary	Private	Rural	Yes	Yes	Yes	No	Yes	No	Yes	No	No	No
8447	HTJ0130	Female	Malay	2020	24	Single	Secondary	Unemployed	Urban	Yes	Yes	No	No	No	No	No	No	No	No
8448	HTJ0132	Male	Malay	2019	49	Others	Secondary	Self-employed	Rural	No	Yes	Yes	No	Yes	No	No	No	Yes	No
8449	HTJ0134	Male	Malay	2019	29	Single	Primary	Unemployed	Rural	Yes	Yes	Yes	No	Yes	Yes	No	No	Yes	No
8450	HTJ0135	Male	Indian	2020	41	Married	Secondary	Private	Rural	No	Yes	Yes	No	No	No	No	No	No	No
8451	HTJ0136	Male	Malay	2020	28	Others	Secondary	Unemployed	Rural	No	Yes	No	Yes	Yes	No	No	No	No	No
8452	HTJ0137	Male	Malay	2019	59	Single	Secondary	Unemployed	Urban	Yes	No	No	No	Yes	No	No	No	Yes	Yes
8453	HTJ0138	Male	Indian	2021	36	Single	Tertiary	Private	Rural	No	Yes	Yes	No	No	No	No	No	Yes	No
8454	HTJ0139	Female	Indian	2020	54	Single	Primary	Private	Rural	No	No	Yes	No	No	No	No	No	No	No
8455	HTJ0140	Male	Malay	2021	31	Others	Secondary	Private	Urban	No	Yes	No	No	Yes	No	No	No	No	Yes

No	Patient ID	Gender	Ethnic group	Year of diagnosis	Age	Marital status	Education level	Occupation	Place of residence	History of psy illness	Tobacco	Alcohol	Cannabis	ATS	Inhalant	Sedative	Hallucinogens	Opioids	Kratom
8456	HTJ0142	Female	Indian	2020	55	Married	No formal education	Unemployed	Urban	No	No	Yes	No	No	No	No	No	No	No
8457	HTJ0143	Male	Malay	2020	42	Single	Tertiary	Private	Urban	Yes	No	No	No	Yes	No	No	No	No	No
8458	HTJ0147	Male	Chinese	2020	47	Single	Primary	Unemployed	Rural	No	Yes	Yes	No	Yes	No	No	No	No	No
8459	HTJ0148	Male	Malay	2020	34	Others	Secondary	Unemployed	Rural	No	No	No	No	Yes	No	No	No	Yes	No
8460	HTJ0149	Male	Indian	2020	28	Others	Primary	Private	Rural	Yes	Yes	Yes	No	No	No	No	No	No	No
8461	HTJ0150	Male	Indian	2020	33	Married	Tertiary	Private	Rural	No	No	No	Yes	Yes	No	No	No	No	No
8462	HTJ0152	Male	Indian	2021	49	Married	No formal education	Private	Urban	No	No	Yes	No	No	No	No	No	No	No
8463	HTJ0153	Male	Chinese	2018	50	Single	Primary	Unemployed	Rural	Yes	Yes	No	Yes	Yes	No	Yes	No	No	No
8464	HTJ0159	Female	Indian	2021	34	Married	Secondary	Unemployed	Urban	No	No	Yes	No	No	No	No	No	No	No
8465	HTJ0160	Male	Indian	2020	33	Married	Primary	Others	Urban	No	No	Yes	No	No	No	No	No	No	No
8466	HTJ0162	Male	Indian	2021	35	Married	Primary	Self-employed	Urban	No	Yes	Yes	No	Yes	No	No	No	No	No
8467	HTJ0167	Male	Indian	2020	53	Others	Primary	Private	Urban	No	No	Yes	No	No	No	No	No	No	No
8468	HTJ0168	Male	Malay	2021	32	Single	Secondary	Private	Urban	No	Yes	No	No	Yes	No	No	No	No	No
8469	HTJ0169	Male	Chinese	2021	59	Married	No formal education	Unemployed	Urban	No	No	Yes	No	No	No	No	No	No	No
8470	HTJ0170	Male	Indian	2020	41	Married	No formal education	Private	Rural	No	No	No	No	Yes	No	No	No	Yes	No
8471	HTJ0171	Male	Indian	2018	34	Others	Secondary	Private	Rural	No	Yes	Yes	Yes	Yes	No	No	No	No	No
8472	HTJ0172	Male	Indian	2019	45	Others	No formal education	Private	Rural	No	No	Yes	No	No	No	No	No	No	No
8473	HTJ0173	Male	Malay	2021	27	Married	Secondary	Unemployed	Rural	Yes	Yes	No	No	Yes	No	No	No	No	Yes
8474	HTJ0174	Male	Indian	2020	31	Single	Primary	Self-employed	Rural	Yes	Yes	Yes	No	No	No	No	No	No	No
8475	HTJ0175	Male	Indian	2020	46	Single	No formal education	Self-employed	Rural	Yes	No	Yes	No	No	No	No	No	No	No
8476	HTJ0176	Male	Indian	2021	39	Married	Tertiary	Private	Urban	No	Yes	Yes	No	No	No	No	No	No	No
8477	HTJ0179	Male	Indian	2020	38	Married	No formal education	Private	Rural	No	Yes	Yes	No	No	No	No	No	No	No
8478	HTJ0180	Male	Indian	2019	44	Married	No formal education	Private	Urban	Yes	Yes	Yes	No	No	No	No	No	No	No
8479	HTJ0181	Male	Indian	2020	48	Single	Primary	Unemployed	Urban	Yes	No	Yes	No	No	No	No	No	No	No
8480	HTJ0182	Male	Malay	2021	45	Married	No formal education	Unemployed	Urban	No	Yes	No	No	No	No	No	No	No	No
8481	HTJ0183	Female	Indian	2020	68	Married	Primary	Self-employed	Urban	No	No	No	No	No	No	No	No	Yes	No
8482	HTJ0184	Female	Others	2020	49	Married	No formal education	Private	Urban	No	No	No	Yes	No	No	No	No	No	No
8483	HTJ0186	Male	Malay	2020	58	Married	Secondary	Unemployed	Urban	No	Yes	No	Yes	Yes	No	No	No	Yes	No

No	Patient ID	Gender	Ethnic group	Year of diagnosis	Age	Marital status	Education level	Occupation	Place of residence	History of psy illness	Tobacco	Alcohol	Cannabis	ATS	Inhalant	Sedative	Hallucinogens	Opioids	Kratom
8484	HTJ0188	Male	Chinese	2019	31	Others	Primary	Private	Urban	Yes	Yes	Yes	No	Yes	No	No	No	No	No
8485	HTJ0189	Male	Malay	2021	43	Married	Tertiary	Unemployed	Rural	No	No	Yes	No	Yes	No	No	No	Yes	No
8486	HTJ0191	Male	Malay	2018	30	Married	Secondary	Unemployed	Urban	Yes	Yes	No	No	Yes	Yes	Yes	No	No	No
8487	HTJ0193	Male	Indian	2021	48	Married	Primary	Private	Rural	No	Yes	Yes	No	No	No	No	No	No	No
8488	HTJ0196	Male	Malay	2021	45	Others	Primary	Self-employed	Urban	No	Yes	No	No	Yes	No	No	No	Yes	No
8489	HTJ0197	Male	Indian	2020	35	Married	Primary	Self-employed	Urban	No	Yes	Yes	No	No	No	No	No	Yes	No
8490	HTJ0198	Male	Malay	2020	30	Others	Primary	Unemployed	Urban	No	No	No	No	Yes	No	No	No	No	No
8491	HTJ0199	Male	Malay	2021	28	Others	Tertiary	Unemployed	Urban	No	Yes	No	No	No	No	No	No	No	No
8492	HTJ0200	Male	Malay	2020	29	Married	Secondary	Unemployed	Rural	Yes	Yes	Yes	Yes	Yes	No	Yes	No	Yes	Yes
8493	HTJ0202	Male	Malay	2021	32	Single	Tertiary	Self-employed	Rural	Yes	Yes	No	Yes	Yes	No	Yes	No	Yes	Yes
8494	HTJ0203	Male	Malay	2021	27	Single	Tertiary	Unemployed	Rural	No	Yes	Yes	Yes	Yes	No	No	Yes	No	Yes
8495	HTJ0204	Male	Malay	2021	31	Single	Secondary	Unemployed	Rural	No	Yes	Yes	Yes	Yes	No	No	No	No	No
8496	HTJ0205	Male	Chinese	2020	55	Others	No formal education	Private	Rural	No	Yes	Yes	No	No	No	No	No	No	No
8497	HTJ0206	Male	Malay	2021	31	Single	Tertiary	Private	Rural	No	Yes	No	Yes	Yes	No	No	No	Yes	Yes
8498	HTJ0207	Male	Malay	2021	30	Single	Secondary	Unemployed	Rural	No	Yes	No	No	Yes	No	No	No	Yes	No
8499	HTJ0209	Male	Chinese	2020	35	Single	Primary	Unemployed	Rural	No	Yes	Yes	Yes	Yes	No	No	No	No	Yes
8500	HTJ0211	Male	Others	2020	44	Others	No formal education	Self-employed	Urban	No	Yes	No	No	Yes	No	Yes	No	No	No
8501	HTJ0212	Male	Indian	2021	46	Others	No formal education	Unemployed	Urban	No	Yes	Yes	No	No	No	No	No	No	No
8502	HTJ0213	Male	Chinese	2019	53	Single	No formal education	Self-employed	Urban	No	Yes	Yes	No	No	No	No	No	No	No
8503	HTJ0214	Male	Malay	2019	21	Single	Secondary	Others	Urban	No	Yes	Yes	Yes	Yes	No	No	No	No	Yes
8504	HTJ0216	Female	Others	2019	44	Married	No formal education	Private	Urban	No	No	Yes	No	No	No	No	No	No	No
8505	HTJ0217	Male	Indian	2020	55	Married	Primary	Private	Urban	No	No	Yes	No	No	No	No	No	No	No
8506	HTJ0219	Male	Malay	2021	36	Single	No formal education	Unemployed	Urban	No	Yes	No	No	Yes	No	No	No	No	No
8507	HTJ0220	Male	Indian	2020	45	Others	Primary	Private	Rural	No	No	Yes	No	No	No	No	No	No	No
8508	HTJ0221	Male	Indian	2020	25	Others	Secondary	Unemployed	Rural	No	Yes	Yes	No	Yes	No	No	No	No	No
8509	HTJ0222	Male	Indian	2018	37	Others	No formal education	Self-employed	Rural	No	No	Yes	No	No	No	No	No	No	No
8510	HTJ0223	Male	Indian	2020	66	Married	No formal education	Self-employed	Rural	No	No	Yes	No	No	No	No	No	No	No
8511	HTJ0224	Male	Indian	2019	46	Married	Primary	Unemployed	Rural	No	Yes	Yes	No	No	No	No	No	No	No
8512	HTJ0225	Male	Indian	2020	48	Married	Primary	Private	Urban	No	Yes	Yes	No	No	No	No	No	No	No
8513	HTJ0226	Male	Indian	2021	67	Married	No formal education	Unemployed	Rural	No	No	Yes	No	No	No	No	No	No	No
8514	HTJ0229	Male	Indian	2020	48	Married	Secondary	Private	Rural	No	Yes	Yes	No	No	No	No	No	No	No

No	Patient ID	Gender	Ethnic group	Year of diagnosis	Age	Marital status	Education level	Occupation	Place of residence	History of psy illness	Tobacco	Alcohol	Cannabis	ATS	Inhalant	Sedative	Hallucinogens	Opioids	Kratom
8515	HTJ0233	Male	Malay	2020	25	Single	Secondary	Self-employed	Urban	No	No	No	No	No	Yes	No	No	No	No
8516	HTJ0234	Male	Chinese	2020	69	Married	No formal education	Self-employed	Urban	No	No	Yes	No	No	No	No	No	No	No
8517	HTJ0236	Male	Malay	2020	18	Single	Tertiary	Unemployed	Urban	No	No	No	Yes	Yes	No	No	No	No	No
8518	HTJ0237	Male	Malay	2020	31	Married	Primary	Self-employed	Urban	No	Yes	No	No	Yes	No	No	No	Yes	No
8519	HTJ0238	Female	Malay	2020	36	Married	No formal education	Unemployed	Urban	No	No	No	No	No	No	No	No	Yes	No
8520	HTJ0239	Male	Indian	2021	56	Married	Primary	Others	Urban	Yes	Yes	Yes	No	No	No	No	No	No	No
8521	HTJ0240	Male	Indian	2021	30	Married	No formal education	Unemployed	Rural	Yes	Yes	Yes	No	No	No	No	No	No	No
8522	HTJ0242	Male	Malay	2021	21	Single	Primary	Private	Rural	No	Yes	No	Yes	No	No	No	No	No	No
8523	HTJ0244	Male	Malay	2019	25	Others	Primary	Private	Rural	No	Yes	Yes	No	Yes	No	No	No	No	Yes
8524	HTJ0247	Male	Malay	2020	25	Married	Primary	Private	Rural	No	Yes	No	No	Yes	No	No	No	No	No
8525	HTJ0248	Male	Indian	2021	62	Married	Primary	Others	Rural	No	Yes	Yes	No	No	No	No	No	No	No
8526	HTJ0249	Male	Malay	2020	22	Single	Secondary	Self-employed	Rural	Yes	Yes	Yes	Yes	Yes	No	Yes	Yes	No	No
8527	HTJ0251	Male	Indian	2020	43	Married	Primary	Self-employed	Urban	No	No	Yes	No	No	No	No	No	No	No
8528	HTJ0252	Male	Indian	2020	39	Others	Primary	Unemployed	Urban	Yes	Yes	Yes	No	No	No	No	No	No	No
8529	HTJ0255	Male	Malay	2018	32	Married	Tertiary	Unemployed	Urban	No	No	No	No	Yes	No	No	No	No	No
8530	HTJ0256	Male	Indian	2020	39	Married	Secondary	Private	Urban	No	No	No	No	Yes	No	No	No	No	No
8531	HTJ0258	Male	Indian	2020	27	Single	Primary	Self-employed	Urban	No	No	Yes	No	No	No	No	No	No	No
8532	HTJ0259	Male	Indian	2020	29	Single	Secondary	Private	Urban	No	No	Yes	No	Yes	No	No	No	No	No
8533	HTJ0260	Male	Indian	2021	40	Single	Primary	Private	Rural	Yes	Yes	Yes	Yes	Yes	No	No	No	Yes	No
8534	HTJ0261	Male	Indian	2020	66	Married	Primary	Others	Rural	No	Yes	Yes	No	No	No	No	No	No	No
8535	HTJ0262	Male	Malay	2021	41	Single	Tertiary	Self-employed	Urban	No	Yes	Yes	No	No	No	No	No	No	Yes
8536	HTJ0263	Male	Malay	2021	29	Single	Secondary	Private	Urban	Yes	No	No	No	Yes	No	No	No	No	No
8537	HTJ0265	Male	Indian	2020	42	Married	Primary	Unemployed	Rural	No	No	Yes	No	No	No	No	No	No	No
8538	HTJ0266	Male	Malay	2020	27	Single	Secondary	Private	Rural	No	Yes	No	No	Yes	No	No	No	No	No
8539	HTJ0267	Male	Malay	2019	23	Single	Primary	Self-employed	Rural	Yes	Yes	Yes	Yes	Yes	No	No	No	No	No
8540	HTJ0268	Male	Indian	2021	36	Married	No formal education	Unemployed	Rural	No	Yes	Yes	No	No	No	No	No	No	No
8541	HTJ0269	Male	Indian	2020	25	Single	Tertiary	Private	Rural	No	Yes	Yes	Yes	Yes	No	No	No	No	No
8542	HTJ0270	Male	Indian	2020	16	Single	Primary	Unemployed	Rural	Yes	Yes	Yes	No	Yes	No	No	No	No	No
8543	HTJ0272	Male	Indian	2020	43	Married	Secondary	Private	Rural	No	Yes	Yes	No	No	No	No	No	No	No
8544	HTJ0277	Male	Indian	2020	43	Others	No formal education	Private	Urban	No	No	Yes	No	No	No	No	No	No	No
8545	HTJ0278	Male	Indian	2021	42	Married	No formal education	Unemployed	Urban	No	No	Yes	No	No	No	No	No	No	No
8546	HTJ0280	Male	Indian	2020	34	Others	Primary	Private	Urban	No	No	Yes	No	No	No	No	No	No	No

No	Patient ID	Gender	Ethnic group	Year of diagnosis	Age	Marital status	Education level	Occupation	Place of residence	History of psy illness	Tobacco	Alcohol	Cannabis	ATS	Inhalant	Sedative	Hallucinogens	Opioids	Kratom
8547	HTJ0282	Male	Chinese	2018	56	Single	Primary	Unemployed	Rural	Yes	Yes	No	No	No	No	Yes	No	No	No
8548	HTJ0283	Male	Indian	2021	24	Single	Secondary	Private	Rural	Yes	Yes	Yes	Yes	Yes	No	No	No	No	No
8549	HTJ0284	Male	Indian	2018	49	Others	Primary	Private	Rural	No	Yes	Yes	No	No	No	No	No	No	No
8550	HTJ0286	Male	Indian	2020	22	Single	Tertiary	Private	Urban	No	Yes	Yes	Yes	Yes	No	No	No	No	No
8551	HTJ0289	Male	Indian	2020	39	Married	No formal education	Private	Rural	Yes	Yes	Yes	Yes	Yes	No	No	No	Yes	No
8552	HTJ0291	Male	Indian	2020	56	Married	Primary	Unemployed	Urban	Yes	Yes	Yes	No	No	No	No	No	No	No
8553	HTJ0292	Male	Indian	2020	48	Married	Secondary	Private	Rural	No	Yes	Yes	No	No	No	No	No	No	No
8554	HTJ0293	Male	Indian	2021	24	Married	Secondary	Private	Rural	No	Yes	Yes	Yes	No	No	No	No	Yes	No
8555	HTJ0294	Male	Indian	2019	36	Others	Tertiary	Self-employed	Rural	Yes	Yes	Yes	No	No	No	No	No	No	No
8556	HTJ0295	Male	Chinese	2018	31	Single	Tertiary	Private	Urban	Yes	No	No	No	Yes	No	No	No	No	No
8557	HTJ0296	Male	Malay	2019	36	Married	Primary	Private	Urban	No	Yes	No	Yes	Yes	Yes	Yes	No	Yes	No
8558	HTJ0300	Male	Malay	2021	24	Single	No formal education	Private	Urban	No	Yes	No	No	No	No	No	No	No	No
8559	HTJ0301	Male	Indian	2019	20	Single	Primary	Private	Urban	No	Yes	No	No	No	Yes	No	No	No	No
8560	HTJ0303	Male	Indian	2019	33	Married	Primary	Self-employed	Urban	No	Yes	Yes	No	Yes	No	Yes	No	No	No
8561	HTJ0304	Male	Indian	2021	33	Married	Primary	Government	Urban	No	No	Yes	No	No	No	No	No	No	No
8562	HTJ0306	Male	Indian	2020	56	Married	No formal education	Self-employed	Urban	No	No	Yes	No	No	No	No	No	No	No
8563	HTJ0307	Male	Malay	2020	27	Married	Tertiary	Unemployed	Urban	No	Yes	No	No	Yes	No	No	No	No	No
8564	HTJ0308	Female	Indian	2019	55	Married	No formal education	Unemployed	Urban	No	No	Yes	No	No	No	No	No	No	No
8565	HTJ0309	Male	Malay	2018	42	Married	Secondary	Private	Urban	No	Yes	No	No	Yes	No	No	No	No	No
8566	HTJ0311	Male	Malay	2018	27	Single	No formal education	Government	Urban	No	Yes	No	No	Yes	No	No	No	No	No
8567	HTJ0314	Male	Indian	2019	28	Single	Primary	Unemployed	Urban	No	Yes	Yes	No	No	No	No	No	No	No
8568	HTJ0315	Male	Others	2020	22	Married	No formal education	Private	Urban	No	No	Yes	No	No	No	No	No	No	No
8569	HTJ0316	Male	Malay	2020	32	Others	No formal education	Others	Urban	No	Yes	No	No	Yes	No	No	No	No	No
8570	HTJ0317	Male	Malay	2021	40	Single	No formal education	Self-employed	Urban	No	No	No	No	Yes	No	No	No	Yes	No
8571	HTJ0318	Male	Malay	2019	27	Single	Tertiary	Private	Urban	No	Yes	No	No	Yes	No	No	No	No	No
8572	HTJ0319	Male	Indian	2019	53	Married	Primary	Unemployed	Urban	No	Yes	Yes	No	No	No	No	No	No	No
8573	HTJ0325	Male	Malay	2019	25	Married	Secondary	Private	Urban	No	Yes	No	No	No	No	Yes	No	No	Yes
8574	HTJ0326	Male	Malay	2018	25	Single	Tertiary	Private	Rural	No	No	No	No	Yes	No	Yes	Yes	No	No
8575	HTJ0327	Male	Indian	2019	64	Married	Primary	Unemployed	Rural	Yes	Yes	Yes	No	No	No	No	No	No	No
8576	HTJ0328	Male	Indian	2019	39	Single	No formal education	Self-employed	Rural	No	Yes	Yes	No	No	Yes	No	No	No	No

No	Patient ID	Gender	Ethnic group	Year of diagnosis	Age	Marital status	Education level	Occupation	Place of residence	History of psy illness	Tobacco	Alcohol	Cannabis	ATS	Inhalant	Sedative	Hallucinogens	Opioids	Kratom
8577	HTJ0329	Male	Chinese	2018	52	Married	Secondary	Unemployed	Urban	Yes	Yes	No	No	No	No	Yes	No	Yes	No
8578	HTJ0330	Female	Indian	2019	25	Others	Secondary	Self-employed	Urban	Yes	No	Yes	No	Yes	No	No	No	No	No
8579	HTJ0331	Male	Others	2018	28	Single	Primary	Self-employed	Rural	No	No	Yes	No	No	No	Yes	No	No	No
8580	HTJ0332	Male	Indian	2019	39	Married	No formal education	Private	Rural	No	Yes	Yes	No	No	No	No	No	No	No
8581	HTJ0334	Male	Malay	2019	19	Single	Secondary	Private	Rural	No	Yes	No	No	Yes	No	No	No	No	No
8582	HTJ0335	Male	Indian	2019	40	Married	Primary	Self-employed	Rural	Yes	Yes	Yes	No	No	No	No	No	No	No
8583	HTJ0339	Male	Malay	2019	34	Single	Primary	Unemployed	Rural	No	Yes	No	No	Yes	No	No	No	Yes	No
8584	HTJ0340	Male	Chinese	2019	45	Others	No formal education	Unemployed	Urban	No	Yes	No	No	Yes	No	No	No	No	No
8585	HTJ0341	Male	Chinese	2019	46	Others	Primary	Self-employed	Urban	No	Yes	Yes	No	Yes	No	No	No	No	No
8586	HTJ0342	Male	Malay	2019	27	Single	Tertiary	Government	Urban	No	Yes	No	No	Yes	No	No	No	No	No
8587	HTJ0344	Male	Indian	2019	25	Married	Primary	Unemployed	Urban	No	Yes	Yes	No	No	No	No	No	No	No
8588	HTJ0346	Male	Malay	2019	34	Married	Secondary	Government	Urban	No	Yes	No	No	No	No	No	No	No	No
8589	HTJ0347	Male	Indian	2019	56	Married	Primary	Self-employed	Urban	No	No	Yes	No	No	No	No	No	No	No
8590	HTJ0348	Male	Others	2019	47	Others	Tertiary	Private	Urban	No	No	Yes	No	No	No	No	No	No	No
8591	HTJ0350	Male	Malay	2019	34	Others	Tertiary	Private	Urban	Yes	No	No	No	No	No	No	No	No	No
8592	HTJ0353	Male	Chinese	2018	45	Married	Primary	Unemployed	Urban	No	Yes	Yes	No	Yes	No	No	No	No	No
8593	HTJ0356	Male	Chinese	2018	42	Single	Secondary	Unemployed	Rural	No	Yes	No	No	Yes	No	No	No	No	No
8594	HTJ0357	Male	Malay	2018	38	Single	Primary	Private	Urban	No	Yes	No	Yes	Yes	No	Yes	No	Yes	No
8595	HTJ0359	Male	Malay	2018	18	Single	Secondary	Unemployed	Urban	Yes	Yes	No	No	Yes	No	No	No	No	No
8596	HTJ0367	Male	Indian	2019	44	Married	Secondary	Private	Rural	No	Yes	Yes	No	No	No	No	No	No	No
8597	HTJ0369	Male	Indian	2018	50	Married	Secondary	Self-employed	Rural	No	Yes	Yes	No	No	No	No	No	No	No
8598	HTJ0370	Male	Indian	2019	45	Married	Primary	Private	Rural	No	Yes	Yes	No	No	No	No	No	No	No
8599	HTJ0371	Male	Malay	2019	56	Others	Tertiary	Self-employed	Rural	Yes	Yes	Yes	Yes	Yes	No	No	No	Yes	No
8600	HTJ0373	Male	Indian	2018	39	Married	Primary	Private	Rural	No	Yes	Yes	No	No	No	No	No	No	No
8601	HTJ0374	Male	Malay	2019	37	Married	Tertiary	Unemployed	Rural	Yes	Yes	Yes	Yes	Yes	Yes	Yes	No	Yes	No
8602	HTJ0376	Female	Malay	2019	29	Married	Tertiary	Self-employed	Rural	Yes	Yes	No	No	Yes	No	No	No	No	No
8603	HTJ0377	Male	Indian	2019	61	Married	Secondary	Private	Urban	No	Yes	Yes	No	Yes	No	No	No	No	No
8604	HTJ0378	Male	Chinese	2019	30	Married	No formal education	Unemployed	Rural	Yes	No	No	No	No	No	Yes	No	No	No
8605	HTJ0379	Female	Chinese	2019	33	Others	Primary	Private	Rural	No	Yes	Yes	Yes	Yes	No	No	No	No	No
8606	HTJ0381	Male	Malay	2018	28	Others	Secondary	Private	Urban	No	Yes	No	Yes	No	No	No	No	No	No
8607	HTJ0382	Female	Malay	2020	24	Single	Secondary	Private	Urban	No	No	No	No	Yes	No	No	No	No	No
8608	HTJ0384	Male	Malay	2021	52	Others	No formal education	Unemployed	Urban	No	Yes	Yes	No	No	No	No	No	No	No
8609	HTJ0385	Male	Indian	2021	39	Others	No formal education	Private	Urban	No	Yes	No	No	No	No	No	No	No	No
8610	HTJ0387	Male	Indian	2021	40	Married	Primary	Self-employed	Urban	No	Yes	Yes	No	No	No	No	No	No	No

No	Patient ID	Gender	Ethnic group	Year of diagnosis	Age	Marital status	Education level	Occupation	Place of residence	History of psy illness	Tobacco	Alcohol	Cannabis	ATS	Inhalant	Sedative	Hallucinogens	Opioids	Kratom
8611	HTJ0388	Male	Indian	2021	56	Others	No formal education	Unemployed	Urban	No	Yes	Yes	No	No	No	No	No	No	No
8612	HTJ0389	Male	Indian	2021	27	Single	Primary	Self-employed	Urban	No	No	Yes	No	No	No	No	No	No	No
8613	HTJ0390	Male	Indian	2021	46	Married	Primary	Unemployed	Rural	No	No	Yes	No	No	No	No	No	No	No
8614	HTJ0391	Male	Malay	2021	42	Others	Primary	Self-employed	Urban	No	Yes	No	Yes	Yes	No	No	No	No	No
8615	HTJ0392	Male	Malay	2021	45	Others	Tertiary	Self-employed	Urban	No	Yes	No	No	Yes	No	No	No	No	No
8616	HTJ0393	Male	Indian	2021	42	Others	Tertiary	Self-employed	Urban	No	Yes	Yes	No	No	No	No	No	No	No
8617	HTJ0394	Male	Indian	2021	35	Others	No formal education	Unemployed	Urban	No	No	Yes	No	No	No	No	No	No	No
8618	HTJ0395	Male	Indian	2021	58	Married	No formal education	Self-employed	Urban	No	Yes	Yes	No	No	No	No	No	No	No
8619	HTJ0396	Male	Indian	2020	42	Married	Primary	Private	Rural	No	Yes	Yes	No	No	No	No	No	No	No
8620	HTJ0401	Male	Malay	2019	37	Married	Tertiary	Government	Urban	No	Yes	Yes	Yes	Yes	No	No	No	No	No
8621	HTJ0402	Male	Malay	2018	58	Married	Secondary	Private	Rural	Yes	Yes	Yes	No	No	No	No	No	No	No
8622	HTJ0404	Male	Indian	2018	51	Married	Primary	Unemployed	Rural	No	Yes	Yes	No	No	No	No	No	No	No
8623	HTJ0405	Male	Chinese	2018	28	Married	Secondary	Self-employed	Rural	No	No	Yes	No	Yes	No	Yes	No	No	No
8624	HTJ0406	Male	Indian	2019	35	Married	No formal education	Unemployed	Urban	No	No	Yes	No	No	No	No	No	No	No
8625	HTJ0407	Male	Indian	2019	38	Married	Primary	Self-employed	Urban	No	Yes	Yes	No	Yes	No	No	No	No	No
8626	HTJ0408	Male	Indian	2018	32	Single	No formal education	Self-employed	Urban	No	No	No	Yes	No	No	No	No	No	No
8627	HTJ0409	Male	Indian	2019	64	Married	Primary	Others	Urban	No	No	Yes	No	No	No	No	No	No	No
8628	HTJ0410	Male	Malay	2019	22	Single	Tertiary	Private	Rural	No	Yes	Yes	Yes	Yes	No	No	No	No	No
8629	HTJ0412	Male	Indian	2019	18	Single	Primary	Unemployed	Rural	Yes	Yes	Yes	Yes	Yes	No	No	No	No	No
8630	HTJ0413	Male	Malay	2019	31	Single	Secondary	Unemployed	Rural	No	Yes	No	No	Yes	No	No	No	No	No
8631	HTJ0414	Male	Malay	2021	38	Married	Secondary	Unemployed	Rural	No	Yes	Yes	No	Yes	No	No	No	No	No
8632	HTJ0415	Male	Indian	2020	55	Married	Primary	Self-employed	Rural	No	No	Yes	No	No	No	No	No	No	No
8633	HTJ0416	Male	Indian	2020	40	Others	Primary	Private	Rural	Yes	Yes	Yes	No	No	No	No	No	No	No
8634	HTJ0417	Male	Indian	2019	44	Married	Primary	Private	Rural	No	No	Yes	No	No	No	No	No	No	No
8635	HTJ0418	Male	Malay	2020	65	Others	Primary	Unemployed	Urban	No	Yes	No	Yes	No	No	No	No	No	No
8636	HTJ0419	Male	Indian	2020	21	Single	No formal education	Unemployed	Rural	No	Yes	No	No	Yes	No	No	No	No	No
8637	HTJ0421	Male	Malay	2021	34	Single	Primary	Unemployed	Urban	Yes	Yes	No	No	No	Yes	No	No	No	No
8638	HTJ0425	Male	Malay	2020	27	Married	Secondary	Private	Rural	No	Yes	Yes	Yes	Yes	No	No	No	No	No
8639	HTJ0426	Male	Malay	2021	27	Married	No formal education	Self-employed	Urban	No	Yes	Yes	No	Yes	No	No	No	No	No
8640	HTJ0427	Male	Indian	2020	36	Others	Primary	Private	Urban	No	No	Yes	No	No	No	No	No	No	No
8641	HTJ0429	Male	Malay	2019	42	Single	Primary	Self-employed	Rural	Yes	Yes	Yes	Yes	Yes	Yes	Yes	No	Yes	No
8642	HTJ0431	Male	Indian	2019	55	Married	No formal education	Self-employed	Rural	No	Yes	Yes	No	No	No	No	No	No	No

No	Patient ID	Gender	Ethnic group	Year of diagnosis	Age	Marital status	Education level	Occupation	Place of residence	History of psy illness	Tobacco	Alcohol	Cannabis	ATS	Inhalant	Sedative	Hallucinogens	Opioids	Kratom
8643	HTJ0432	Male	Others	2019	30	Others	Tertiary	Private	Urban	Yes	No	Yes	No	Yes	No	No	No	No	No
8644	HTJ0433	Male	Malay	2018	31	Others	Tertiary	Private	Urban	Yes	No	No	No	Yes	No	Yes	No	No	No
8645	HTJ0434	Male	Indian	2018	53	Single	Primary	Unemployed	Rural	No	Yes	Yes	No	No	No	No	No	No	No
8646	HTJ0435	Male	Chinese	2018	49	Others	Primary	Self-employed	Rural	Yes	No	No	No	No	No	Yes	No	No	No
8647	HTJ0436	Male	Malay	2020	44	Others	Primary	Unemployed	Rural	Yes	Yes	Yes	No	Yes	No	No	No	Yes	No
8648	HTJ0437	Male	Malay	2020	48	Married	Secondary	Private	Urban	Yes	Yes	No	Yes	No	No	No	No	No	No
8649	HTJ0438	Male	Malay	2018	31	Single	Primary	Private	Rural	No	Yes	No	No	No	No	No	No	No	No
8650	HTJ0439	Male	Malay	2020	36	Single	Primary	Unemployed	Rural	Yes	Yes	No	No	No	No	No	No	Yes	No
8651	HTJ0440	Male	Malay	2020	32	Married	No formal education	Unemployed	Urban	No	Yes	No	No	Yes	No	No	No	No	No
8652	HTJ0441	Female	Malay	2019	21	Single	Secondary	Unemployed	Rural	Yes	Yes	Yes	No	Yes	No	No	No	No	No
8653	HTJ0442	Female	Chinese	2019	42	Others	Primary	Private	Rural	No	Yes	Yes	No	No	No	No	No	No	No
8654	HTJ0443	Female	Chinese	2018	56	Married	Primary	Self-employed	Rural	No	No	Yes	No	No	No	Yes	No	No	No
8655	HTJ0444	Female	Malay	2019	17	Married	Primary	Self-employed	Rural	No	No	No	No	Yes	No	No	No	No	No
8656	HTJ0445	Male	Malay	2018	23	Others	Secondary	Private	Rural	Yes	Yes	Yes	Yes	Yes	No	No	No	No	No
8657	HTJ0446	Male	Indian	2018	47	Single	No formal education	Unemployed	Rural	Yes	Yes	Yes	No	No	No	No	No	No	No
8658	HTJ0447	Male	Malay	2018	34	Others	Secondary	Self-employed	Rural	No	No	Yes	No	No	No	No	No	No	Yes
8659	HTJ0448	Male	Indian	2020	44	Married	Primary	Unemployed	Urban	No	No	Yes	No	No	No	No	No	No	No
8660	HTJ0449	Female	Malay	2020	47	Others	Primary	Unemployed	Rural	No	No	No	No	Yes	No	No	No	No	No
8661	HTJ0450	Male	Others	2019	24	Married	No formal education	Unemployed	Urban	No	Yes	No	Yes	No	No	No	No	No	No
8662	HTJ0451	Male	Indian	2018	69	Others	No formal education	Private	Urban	No	No	Yes	No	No	No	No	No	No	No
8663	HTJ0452	Male	Indian	2018	34	Others	No formal education	Others	Urban	No	Yes	Yes	No	No	No	No	No	No	No
8664	HTJ0453	Male	Chinese	2018	25	Single	Secondary	Self-employed	Urban	No	Yes	No	No	Yes	No	No	No	No	No
8665	HTJ0454	Male	Others	2020	32	Single	Primary	Unemployed	Rural	No	Yes	Yes	Yes	Yes	Yes	No	No	No	Yes
8666	HTJ0455	Female	Malay	2018	21	Single	Secondary	Self-employed	Urban	No	Yes	No	No	Yes	No	No	No	No	No
8667	HTJ0456	Female	Malay	2020	43	Married	Secondary	Unemployed	Rural	No	No	No	No	Yes	No	No	No	No	No
8668	HTJ0457	Female	Chinese	2018	46	Married	Primary	Self-employed	Rural	Yes	No	Yes	Yes	Yes	No	No	No	No	No
8669	HTJ0458	Male	Indian	2019	44	Single	Primary	Self-employed	Rural	No	Yes	Yes	Yes	No	No	No	No	Yes	No
8670	HTJ0459	Male	Chinese	2019	46	Single	No formal education	Private	Urban	No	No	Yes	No	Yes	No	No	No	No	No
8671	HTJ0460	Male	Indian	2020	36	Married	Primary	Self-employed	Rural	No	Yes	Yes	No	No	No	No	No	Yes	No
8672	HTJ0461	Male	Malay	2018	38	Single	Secondary	Unemployed	Urban	No	Yes	No	No	Yes	No	No	No	No	No
8673	HTJ0462	Male	Chinese	2020	28	Single	Secondary	Unemployed	Rural	Yes	Yes	Yes	No	No	No	No	No	No	No
8674	HTJ0463	Male	Indian	2019	47	Married	Primary	Private	Rural	No	Yes	Yes	No	No	No	No	No	No	No
8675	HTJ0464	Male	Chinese	2018	36	Single	Primary	Private	Rural	Yes	No	Yes	Yes	Yes	No	Yes	No	Yes	No
8676	HTJ0465	Male	Others	2018	46	Married	Secondary	Private	Rural	No	No	Yes	No	Yes	No	No	No	No	No

No	Patient ID	Gender	Ethnic group	Year of diagnosis	Age	Marital status	Education level	Occupation	Place of residence	History of psy illness	Tobacco	Alcohol	Cannabis	ATS	Inhalant	Sedative	Hallucinogens	Opioids	Kratom
8677	HTJ0466	Male	Indian	2018	52	Married	Primary	Self-employed	Rural	No	Yes	Yes	No	No	No	No	No	No	No
8678	HTJ0467	Male	Malay	2018	27	Married	Tertiary	Unemployed	Rural	No	Yes	Yes	Yes	Yes	No	No	No	Yes	Yes
8679	HTJ0469	Male	Malay	2018	42	Others	No formal education	Others	Rural	No	No	No	No	No	No	Yes	No	Yes	No
8680	HTJ0470	Male	Malay	2020	20	Single	No formal education	Unemployed	Rural	No	Yes	No	No	Yes	No	No	No	No	No
8681	HTJ0471	Male	Malay	2019	37	Single	Secondary	Private	Urban	No	Yes	No	No	Yes	No	No	No	No	No
8682	HTJ0472	Male	Indian	2019	33	Single	Primary	Unemployed	Rural	No	No	No	No	Yes	No	No	No	Yes	No
8683	HTJ0473	Male	Indian	2018	28	Married	Secondary	Unemployed	Rural	No	Yes	Yes	No	Yes	No	No	No	No	No
8684	HTJ0474	Male	Indian	2018	40	Married	Primary	Private	Rural	No	Yes	Yes	No	Yes	No	No	No	No	No
8685	HTJ0476	Male	Chinese	2019	24	Single	Tertiary	Unemployed	Urban	No	Yes	Yes	Yes	No	No	No	No	No	No
8686	HTJ0478	Male	Indian	2018	50	Married	Secondary	Private	Rural	No	Yes	Yes	No	No	No	No	No	No	No
8687	HTJ0480	Male	Malay	2019	35	Single	Primary	Unemployed	Urban	No	Yes	No	No	Yes	No	No	No	No	No
8688	HTJ0485	Male	Chinese	2018	33	Others	Secondary	Unemployed	Rural	No	Yes	No	No	Yes	No	No	No	No	No
8689	HTJ0486	Male	Indian	2018	37	Single	Primary	Self-employed	Rural	No	No	Yes	No	No	No	No	No	Yes	No
8690	HTJ0487	Male	Malay	2019	24	Others	Secondary	Others	Rural	No	Yes	No	Yes	Yes	No	Yes	No	No	No
8691	HTJ0488	Male	Others	2020	44	Married	Secondary	Private	Rural	No	No	Yes	No	No	No	No	No	Yes	No
8692	HTJ0489	Male	Chinese	2019	44	Others	Primary	Unemployed	Rural	No	Yes	Yes	No	No	No	No	No	No	No
8693	HTJ0490	Male	Malay	2018	36	Single	Secondary	Unemployed	Rural	Yes	Yes	No	Yes	Yes	No	No	No	Yes	No
8694	HTJ0491	Male	Indian	2018	60	Married	Primary	Private	Urban	No	No	Yes	No	No	No	No	No	No	No
8695	HTJ0492	Male	Indian	2018	31	Single	Primary	Private	Rural	No	Yes	Yes	No	No	No	No	No	No	No
8696	HTJ0493	Male	Indian	2019	69	Married	Primary	Self-employed	Urban	No	No	Yes	No	No	No	No	No	No	No
8697	HTJ0494	Male	Indian	2018	26	Married	Primary	Unemployed	Rural	No	Yes	Yes	No	No	No	No	No	No	No
8698	HTJ0495	Male	Malay	2018	27	Married	Primary	Self-employed	Rural	Yes	Yes	No	No	No	No	No	No	No	Yes
8699	HTJ0496	Male	Malay	2018	22	Single	Secondary	Unemployed	Rural	No	Yes	No	Yes	Yes	No	Yes	No	No	Yes
8700	HTJ0497	Male	Malay	2020	55	Married	Secondary	Private	Urban	No	Yes	Yes	No	No	No	No	No	No	No
8701	HTJ0498	Male	Indian	2020	48	Married	Primary	Private	Rural	Yes	Yes	Yes	No	No	No	No	No	No	No
8702	HTJ0500	Male	Malay	2018	43	Others	Primary	Self-employed	Rural	No	Yes	Yes	No	No	No	No	No	No	No
8703	HTJ0501	Male	Malay	2018	29	Single	Tertiary	Unemployed	Rural	No	Yes	No	Yes	No	No	No	No	No	No
8704	HTJ0503	Male	Indian	2019	51	Married	Primary	Unemployed	Rural	No	Yes	Yes	No	No	No	No	No	No	No
8705	HTJ0504	Male	Malay	2019	30	Married	Secondary	Unemployed	Rural	No	Yes	No	No	Yes	No	No	No	No	No
8706	HTJ0505	Male	Indian	2018	63	Married	Primary	Private	Urban	No	Yes	Yes	No	No	No	No	No	No	No
8707	HTJ0506	Male	Malay	2018	52	Married	No formal education	Others	Urban	No	No	No	No	Yes	No	No	No	No	No
8708	HTJ0507	Male	Malay	2018	32	Single	No formal education	Unemployed	Rural	No	Yes	No	No	Yes	No	No	No	No	No
8709	HTJ0511	Female	Malay	2018	24	Others	Secondary	Unemployed	Rural	No	No	No	No	Yes	No	No	No	No	No
8710	HTJ0512	Male	Indian	2018	57	Married	Primary	Unemployed	Rural	No	Yes	Yes	No	No	No	No	No	No	No
8711	HTJ0513	Male	Malay	2019	44	Married	Primary	Private	Rural	No	Yes	Yes	No	No	No	No	No	No	No

No	Patient ID	Gender	Ethnic group	Year of diagnosis	Age	Marital status	Education level	Occupation	Place of residence	History of psy illness	Tobacco	Alcohol	Cannabis	ATS	Inhalant	Sedative	Hallucinogens	Opioids	Kratom
8712	HTJ0514	Male	Malay	2019	42	Others	No formal education	Unemployed	Urban	No	Yes	Yes	Yes	No	No	No	No	No	No
8713	HTJ0515	Male	Chinese	2018	53	Others	Primary	Private	Rural	No	Yes	Yes	Yes	No	No	Yes	No	Yes	No
8714	HTJ0516	Male	Malay	2020	40	Married	Tertiary	Private	Rural	No	Yes	Yes	Yes	No	No	No	No	No	Yes
8715	HTJ0517	Male	Indian	2019	46	Married	Secondary	Private	Rural	No	No	Yes	No	No	No	No	No	No	No
8716	HTJ0518	Male	Indian	2019	40	Married	Secondary	Private	Rural	No	Yes	Yes	No	Yes	No	No	No	No	No
8717	HTJ0519	Male	Malay	2019	26	Married	Secondary	Private	Urban	No	Yes	Yes	Yes	Yes	No	Yes	No	Yes	No
8718	HTJ0520	Male	Malay	2020	38	Single	Primary	Unemployed	Urban	No	Yes	Yes	Yes	Yes	No	No	No	Yes	No
8719	HTJ0521	Male	Malay	2019	23	Single	Primary	Unemployed	Rural	No	Yes	No	No	Yes	No	No	No	No	No
8720	HTJ0522	Female	Malay	2018	29	Married	Secondary	Private	Rural	No	Yes	No	No	Yes	No	No	No	No	No
8721	HTJ0523	Female	Malay	2018	22	Single	Tertiary	Unemployed	Rural	Yes	Yes	No	Yes	No	No	No	No	No	No
8722	HTJ0524	Male	Malay	2018	42	Married	Secondary	Unemployed	Rural	Yes	Yes	No	Yes	No	No	No	No	No	No
8723	HTJ0525	Male	Malay	2018	32	Married	Primary	Private	Rural	No	Yes	No	No	No	No	No	No	Yes	No
8724	HTJ0527	Male	Malay	2018	27	Single	No formal education	Unemployed	Rural	No	Yes	Yes	No	Yes	No	No	No	Yes	No
8725	HTJ0528	Male	Chinese	2020	28	Single	Primary	Unemployed	Rural	No	Yes	Yes	No	Yes	No	No	No	No	No
8726	HTJ0529	Male	Indian	2019	52	Married	Primary	Unemployed	Rural	No	No	Yes	No	No	No	No	No	No	No
8727	HTJ0530	Male	Indian	2019	21	Single	Primary	Private	Rural	Yes	Yes	Yes	No	Yes	No	No	No	No	No
8728	HTJ0533	Male	Indian	2020	40	Married	Primary	Private	Rural	No	Yes	Yes	No	Yes	No	No	No	Yes	No
8729	HTJ0534	Male	Indian	2020	46	Married	Secondary	Unemployed	Rural	No	Yes	Yes	No	No	No	No	No	No	No
8730	HTJ0536	Male	Chinese	2019	46	Married	Secondary	Private	Rural	No	Yes	Yes	No	No	No	Yes	No	No	No
8731	HTJ0537	Male	Indian	2018	37	Married	Tertiary	Unemployed	Urban	No	Yes	No	No	Yes	No	No	No	No	No
8732	HTJ0538	Male	Malay	2018	34	Married	Primary	Self-employed	Rural	Yes	Yes	Yes	No	Yes	No	No	No	Yes	No
8733	HTJ0539	Male	Others	2018	23	Single	No formal education	Unemployed	Urban	Yes	No	No	No	Yes	No	No	No	No	No
8734	HTJ0540	Male	Chinese	2018	34	Single	Primary	Unemployed	Urban	No	Yes	No	No	Yes	No	No	No	No	No
8735	HTJ0542	Male	Indian	2019	53	Married	Primary	Unemployed	Urban	No	Yes	Yes	No	No	No	No	No	No	No
8736	HTJ0543	Male	Indian	2018	73	Married	No formal education	Unemployed	Rural	No	Yes	Yes	No	No	No	No	No	No	No
8737	HTJ0544	Female	Indian	2018	60	Others	No formal education	Self-employed	Rural	No	No	Yes	No	No	No	No	No	No	No
8738	HTJ0545	Male	Indian	2018	46	Married	No formal education	Private	Urban	No	Yes	Yes	No	No	No	No	No	No	No
8739	HTJ0546	Male	Indian	2019	53	Married	Primary	Private	Rural	No	Yes	Yes	No	No	No	No	No	No	No
8740	HTJ0549	Male	Malay	2018	33	Married	Primary	Private	Urban	Yes	Yes	Yes	Yes	Yes	Yes	No	No	No	No
8741	HTJ0550	Male	Chinese	2018	25	Single	Secondary	Private	Rural	No	Yes	Yes	Yes	No	No	No	No	No	No
8742	HTJ0551	Female	Chinese	2018	55	Single	Primary	Unemployed	Rural	No	No	Yes	No	No	No	Yes	No	No	No
8743	HTJ0552	Male	Malay	2018	35	Single	Primary	Self-employed	Rural	No	Yes	Yes	No	No	No	No	No	Yes	No
8744	HTJ0553	Male	Indian	2020	36	Others	Primary	Private	Rural	No	Yes	Yes	No	No	No	No	No	No	No
8745	HTJ0554	Male	Malay	2018	28	Single	Tertiary	Private	Urban	Yes	Yes	No	No	Yes	No	No	No	No	No
8746	HTJ0555	Male	Malay	2019	29	Single	Primary	Unemployed	Rural	Yes	Yes	Yes	Yes	No	No	No	No	No	Yes

No	Patient ID	Gender	Ethnic group	Year of diagnosis	Age	Marital status	Education level	Occupation	Place of residence	History of psy illness	Tobacco	Alcohol	Cannabis	ATS	Inhalant	Sedative	Hallucinogens	Opioids	Kratom
8747	HTJ0556	Male	Indian	2018	18	Single	Primary	Unemployed	Rural	No	Yes	Yes	No	No	No	No	No	No	No
8748	HTJ0557	Male	Malay	2018	51	Married	Primary	Private	Rural	No	Yes	No	Yes	Yes	No	No	No	Yes	No
8749	HTJ0558	Male	Indian	2020	39	Married	Primary	Private	Rural	No	Yes	Yes	No	No	No	No	No	No	No
8750	HTJ0559	Male	Malay	2018	27	Single	Tertiary	Private	Rural	No	Yes	No	Yes	Yes	No	Yes	No	No	No
8751	HTJ0560	Male	Malay	2018	34	Married	Secondary	Private	Rural	No	Yes	No	No	No	No	No	No	Yes	No
8752	HTJ0561	Male	Malay	2018	27	Single	Secondary	Private	Rural	Yes	No	No	No	Yes	No	No	No	No	No
8753	HTJ0562	Male	Malay	2019	25	Single	Secondary	Private	Urban	No	Yes	No	No	Yes	No	Yes	No	No	No
8754	HTJ0564	Female	Malay	2019	34	Married	Secondary	Unemployed	Rural	No	Yes	No	No	Yes	No	No	No	Yes	No
8755	HTJ0565	Male	Malay	2020	33	Single	No formal education	Unemployed	Urban	No	No	No	No	Yes	No	No	No	Yes	No
8756	HTJ0566	Male	Malay	2018	30	Single	Primary	Unemployed	Urban	No	No	No	No	No	Yes	No	No	No	No
8757	HTJ0569	Male	Indian	2019	24	Single	Primary	Private	Urban	No	Yes	No	No	No	No	No	No	No	No
8758	HTJ0570	Male	Chinese	2019	40	Married	Primary	Private	Rural	Yes	Yes	Yes	No	Yes	No	No	No	No	No
8759	HTJ0571	Male	Indian	2018	54	Others	Primary	Unemployed	Urban	No	Yes	Yes	No	No	No	No	No	No	No
8760	HTJ0572	Female	Malay	2020	48	Married	Primary	Unemployed	Rural	No	Yes	Yes	Yes	Yes	No	No	No	No	No
8761	HTJ0573	Female	Chinese	2019	28	Married	Secondary	Private	Rural	No	Yes	No	No	Yes	No	No	No	No	No
8762	HTJ0574	Female	Indian	2018	50	Married	Primary	Unemployed	Urban	No	No	Yes	No	No	No	No	No	No	No
8763	HTJ0575	Male	Chinese	2018	37	Single	Secondary	Private	Rural	No	Yes	Yes	No	Yes	No	No	No	No	No
8764	HTJ0576	Male	Malay	2018	20	Others	Primary	Self-employed	Rural	No	Yes	Yes	No	Yes	No	No	No	No	No
8765	HTJ0577	Male	Malay	2018	27	Single	Tertiary	Private	Rural	Yes	Yes	No	Yes	Yes	No	No	Yes	No	No
8766	HTJ0578	Male	Malay	2019	38	Single	Primary	Private	Rural	Yes	Yes	Yes	No	Yes	No	No	No	No	No
8767	HTJ0579	Male	Chinese	2018	34	Single	Primary	Unemployed	Rural	Yes	No	Yes	No	No	No	No	No	Yes	No
8768	HTJ0581	Male	Indian	2018	54	Others	No formal education	Unemployed	Urban	No	No	No	Yes	No	No	No	No	No	No
8769	HTJ0582	Male	Malay	2018	33	Others	No formal education	Unemployed	Urban	No	Yes	Yes	No	Yes	No	No	No	Yes	No
8770	HTJ0583	Male	Indian	2018	51	Married	No formal education	Unemployed	Urban	No	Yes	Yes	No	No	No	No	No	No	No
8771	HTJ0584	Male	Malay	2018	25	Single	Secondary	Private	Urban	No	Yes	Yes	No	No	No	No	No	No	No
8772	HTJ0585	Male	Malay	2019	32	Single	No formal education	Others	Urban	Yes	No	No	No	Yes	No	No	No	No	No
8773	HTJ0586	Male	Malay	2019	25	Single	No formal education	Private	Urban	No	Yes	Yes	No	Yes	No	Yes	No	No	No
8774	HTJ0587	Male	Indian	2019	44	Married	Primary	Self-employed	Urban	No	Yes	Yes	No	No	No	No	No	No	No
8775	HTJ0588	Male	Chinese	2019	37	Married	No formal education	Self-employed	Urban	No	Yes	No	No	Yes	No	Yes	No	No	No
8776	HTJ0589	Male	Indian	2018	37	Others	Primary	Private	Urban	No	Yes	Yes	No	Yes	No	No	No	No	No
8777	HTJ0590	Male	Malay	2019	31	Single	Primary	Unemployed	Rural	Yes	Yes	No	No	Yes	No	No	No	No	No
8778	HTJ0591	Male	Indian	2019	40	Married	Primary	Private	Urban	No	Yes	Yes	No	No	No	No	No	No	No
8779	HTJ0592	Male	Indian	2019	59	Married	Primary	Unemployed	Rural	No	Yes	Yes	No	No	No	No	No	No	No

No	Patient ID	Gender	Ethnic group	Year of diagnosis	Age	Marital status	Education level	Occupation	Place of residence	History of psy illness	Tobacco	Alcohol	Cannabis	ATS	Inhalant	Sedative	Hallucinogens	Opioids	Kratom
8780	HTJ0593	Male	Indian	2019	44	Married	Primary	Unemployed	Rural	No	No	Yes	No	No	No	No	No	No	No
8781	HTJ0594	Male	Malay	2019	40	Married	Primary	Private	Rural	No	Yes	Yes	No	Yes	No	No	No	Yes	No
8782	HTJ0595	Male	Indian	2019	32	Married	Primary	Self-employed	Rural	No	Yes	Yes	No	No	No	No	No	No	No
8783	HTJ0596	Male	Indian	2020	40	Single	No formal education	Self-employed	Rural	No	Yes	Yes	No	No	No	No	No	No	No
8784	HTJ0597	Male	Malay	2018	55	Married	Secondary	Private	Urban	No	Yes	Yes	No	No	No	No	No	No	No
8785	HTJ0598	Male	Malay	2019	39	Single	Secondary	Private	Rural	No	Yes	Yes	No	No	No	No	No	Yes	No
8786	HTJ0599	Male	Malay	2020	23	Single	Tertiary	Private	Rural	No	Yes	No	No	No	No	No	No	No	Yes
8787	HTJ0600	Male	Chinese	2018	34	Single	Primary	Self-employed	Urban	No	No	No	No	Yes	No	No	No	No	No
8788	HTJ0601	Male	Others	2020	23	Single	Secondary	Private	Urban	No	No	Yes	No	No	No	No	No	Yes	No
8789	HTJ0602	Male	Indian	2019	51	Married	Primary	Unemployed	Rural	No	Yes	Yes	No	Yes	No	No	No	No	No
8790	HTJ0603	Female	Malay	2020	23	Single	Tertiary	Private	Urban	No	Yes	No	Yes	Yes	No	Yes	No	No	No
8791	HTJ0604	Female	Chinese	2018	73	Married	No formal education	Unemployed	Rural	No	No	Yes	No	No	No	No	No	No	No
8792	HTJ0607	Male	Malay	2020	21	Single	Secondary	Unemployed	Urban	Yes	Yes	Yes	Yes	Yes	No	No	No	No	No
8793	HTJ0608	Male	Chinese	2018	40	Married	Primary	Private	Urban	Yes	No	Yes	No	No	No	No	No	No	No
8794	HTJ0609	Female	Indian	2018	22	Single	Tertiary	Unemployed	Urban	Yes	Yes	No	Yes	Yes	No	No	No	No	No
8795	HTJ0610	Male	Malay	2020	23	Single	Secondary	Unemployed	Urban	No	No	No	Yes	No	No	No	No	No	No
8796	HTJ0611	Male	Malay	2020	39	Others	No formal education	Unemployed	Urban	No	Yes	No	No	No	No	No	No	No	No
8797	HTJ0612	Male	Chinese	2019	52	Married	No formal education	Unemployed	Urban	No	No	Yes	No	No	No	No	No	No	No
8798	HTJ0613	Male	Indian	2018	49	Others	Primary	Others	Urban	No	No	Yes	No	No	No	No	No	No	No
8799	HTJ0614	Male	Malay	2018	55	Single	No formal education	Unemployed	Urban	No	No	Yes	No	No	No	No	No	No	No
8800	HTJ0615	Female	Chinese	2018	36	Married	Tertiary	Self-employed	Urban	No	No	Yes	No	No	No	No	No	No	No
8801	HTJ0616	Male	Indian	2020	24	Single	Tertiary	Self-employed	Urban	No	Yes	Yes	Yes	No	No	No	No	No	No
8802	HTJ0617	Male	Malay	2020	44	Single	Secondary	Unemployed	Urban	Yes	No	No	Yes	No	No	No	No	No	No
8803	HTJ0624	Male	Malay	2020	37	Others	Secondary	Unemployed	Urban	No	No	No	No	Yes	No	No	No	No	Yes
8804	HTJ0630	Male	Indian	2020	57	Others	Primary	Unemployed	Rural	No	Yes	Yes	No	No	No	No	No	Yes	No
8805	HTJ0631	Male	Malay	2018	20	Single	Primary	Unemployed	Urban	No	Yes	No	Yes	No	No	No	No	No	No
8806	HTJ0633	Male	Indian	2021	31	Married	Secondary	Private	Rural	No	Yes	Yes	No	No	No	No	No	No	No
8807	HTJ0634	Male	Chinese	2018	24	Others	Tertiary	Private	Urban	No	Yes	Yes	No	Yes	No	No	No	No	No
8808	HTJ0636	Male	Indian	2021	47	Married	Primary	Private	Rural	No	Yes	Yes	No	No	No	No	No	No	No
8809	HTJ0638	Male	Malay	2020	28	Single	Secondary	Private	Urban	Yes	Yes	No	Yes	Yes	No	No	Yes	No	No
8810	HTJ0639	Male	Malay	2018	31	Single	Secondary	Unemployed	Rural	Yes	Yes	No	No	Yes	No	No	No	No	No
8811	HTJ0640	Male	Indian	2018	32	Others	Secondary	Private	Rural	No	Yes	No	No	Yes	No	Yes	No	No	No
8812	HTJ0641	Male	Chinese	2018	28	Others	Secondary	Private	Urban	Yes	Yes	Yes	No	Yes	No	No	No	No	No
8813	HTJ0642	Male	Malay	2018	23	Single	Primary	Unemployed	Rural	No	Yes	No	No	Yes	Yes	No	No	No	No
8814	HTJ0643	Male	Indian	2021	30	Married	Primary	Private	Rural	Yes	Yes	Yes	No	No	No	Yes	No	No	No
8815	HTJ0644	Male	Chinese	2021	37	Single	Secondary	Private	Rural	Yes	Yes	No	No	Yes	No	No	No	No	No
8816	HTJ0646	Male	Malay	2018	23	Married	Primary	Self-employed	Rural	Yes	Yes	No	Yes	Yes	No	No	No	No	No
8817	HTJ0647	Female	Malay	2018	50	Married	Secondary	Unemployed	Rural	No	No	No	No	No	No	Yes	No	No	No

No	Patient ID	Gender	Ethnic group	Year of diagnosis	Age	Marital status	Education level	Occupation	Place of residence	History of psy illness	Tobacco	Alcohol	Cannabis	ATS	Inhalant	Sedative	Hallucinogens	Opioids	Kratom
8818	HTJ0649	Male	Malay	2020	21	Single	Tertiary	Unemployed	Urban	Yes	Yes	No	Yes	No	No	No	No	No	No
8819	HTJ0650	Male	Indian	2020	70	Married	No formal education	Unemployed	Rural	No	No	Yes	No	No	No	Yes	No	No	No
8820	HTJ0652	Male	Malay	2020	35	Single	Primary	Unemployed	Rural	Yes	Yes	Yes	Yes	Yes	No	No	No	No	No
8821	HTJ0653	Male	Chinese	2018	25	Single	Primary	Private	Rural	Yes	Yes	Yes	No	Yes	No	Yes	No	No	No
8822	HTJ0656	Male	Malay	2018	28	Single	Secondary	Unemployed	Urban	Yes	Yes	No	Yes	Yes	No	No	No	No	No
8823	HTJ0660	Male	Malay	2019	26	Others	Primary	Self-employed	Rural	No	Yes	No	Yes	Yes	No	Yes	No	Yes	No
8824	HTJ0662	Female	Malay	2020	23	Others	Tertiary	Unemployed	Urban	No	Yes	Yes	Yes	No	No	No	No	No	No
8825	HTJ0668	Male	Malay	2021	33	Others	Primary	Private	Rural	Yes	Yes	No	Yes	Yes	No	No	No	Yes	No
8826	HTJ0669	Male	Malay	2018	30	Married	Tertiary	Private	Rural	Yes	Yes	Yes	Yes	Yes	No	No	No	Yes	No
8827	HTJ0671	Male	Malay	2019	33	Others	Tertiary	Private	Urban	No	Yes	No	No	No	No	No	No	No	No
8828	HTJ0672	Male	Malay	2018	24	Single	Secondary	Unemployed	Urban	No	Yes	No	Yes	No	No	No	No	No	Yes
8829	HTJ0678	Male	Chinese	2018	22	Single	Secondary	Unemployed	Urban	No	No	No	No	Yes	No	No	No	No	No
8830	HTJ0679	Male	Indian	2020	29	Single	Primary	Private	Rural	Yes	Yes	Yes	Yes	No	No	No	No	No	No
8831	HTJ0681	Male	Chinese	2019	40	Single	Primary	Unemployed	Rural	Yes	Yes	Yes	Yes	Yes	No	Yes	No	Yes	No
8832	HTJ0682	Male	Malay	2019	36	Others	Tertiary	Private	Rural	Yes	Yes	No	Yes	No	No	No	No	No	No
8833	HTJ0685	Male	Malay	2018	24	Single	Primary	Unemployed	Rural	Yes	Yes	Yes	Yes	Yes	Yes	No	No	Yes	No
8834	HTJ0687	Male	Indian	2018	29	Single	Primary	Private	Rural	Yes	Yes	Yes	Yes	Yes	No	No	No	No	No
8835	HTJ0688	Male	Chinese	2018	26	Others	Secondary	Private	Rural	No	Yes	No	No	Yes	No	No	No	No	No
8836	HTJ0689	Male	Indian	2020	39	Others	Primary	Private	Rural	No	Yes	Yes	No	No	No	No	No	No	No
8837	HTJ0690	Male	Malay	2018	32	Others	Secondary	Private	Rural	Yes	Yes	Yes	No	Yes	Yes	No	No	Yes	Yes
8838	HTJ0691	Male	Chinese	2020	43	Married	Primary	Self-employed	Rural	Yes	No	No	No	Yes	No	Yes	No	No	No
8839	HTJ0693	Female	Malay	2018	31	Single	Secondary	Unemployed	Urban	Yes	Yes	Yes	No	No	No	Yes	No	No	No
8840	HTJ0695	Male	Chinese	2018	52	Others	Primary	Private	Rural	Yes	Yes	Yes	No	No	No	No	No	No	No
8841	HTJ0696	Male	Malay	2018	19	Others	Tertiary	Unemployed	Rural	Yes	Yes	No	Yes	No	No	No	No	No	No
8842	HTJ0697	Male	Malay	2018	37	Single	Primary	Unemployed	Rural	Yes	Yes	No	No	No	No	No	No	Yes	No
8843	HTJ0699	Male	Chinese	2019	45	Married	Primary	Private	Rural	No	Yes	No	Yes	Yes	No	No	No	Yes	No
8844	HTJ0701	Male	Malay	2021	28	Single	Secondary	Unemployed	Rural	No	Yes	Yes	Yes	Yes	Yes	No	Yes	No	No
8845	HTJ0702	Female	Indian	2019	41	Married	No formal education	Unemployed	Rural	Yes	Yes	No	No	Yes	No	No	No	No	No
8846	HTJ0705	Male	Chinese	2019	32	Single	Primary	Self-employed	Rural	Yes	Yes	Yes	Yes	Yes	No	No	Yes	No	No
8847	HTJ0706	Male	Malay	2020	31	Single	Primary	Unemployed	Rural	Yes	Yes	No	Yes	Yes	No	No	No	Yes	No
8848	HTJ0707	Male	Indian	2020	50	Others	Primary	Private	Rural	No	Yes	Yes	No	Yes	No	No	No	Yes	No
8849	HTJ0710	Male	Malay	2019	51	Others	Secondary	Unemployed	Urban	No	Yes	No	No	No	No	No	No	Yes	No
8850	HTJ0712	Male	Malay	2019	38	Married	Secondary	Others	Urban	No	Yes	No	No	No	No	No	No	No	No
8851	HTJ0715	Male	Malay	2020	33	Married	Secondary	Unemployed	Rural	Yes	Yes	No	No	Yes	No	No	No	No	No
8852	HTJ0716	Male	Malay	2018	24	Single	Tertiary	Private	Rural	No	Yes	No	Yes	No	No	No	No	No	No
8853	HTJ0719	Male	Indian	2018	74	Married	Primary	Unemployed	Rural	Yes	No	No	No	No	No	Yes	No	No	No

No	Patient ID	Gender	Ethnic group	Year of diagnosis	Age	Marital status	Education level	Occupation	Place of residence	History of psy illness	Tobacco	Alcohol	Cannabis	ATS	Inhalant	Sedative	Hallucinogens	Opioids	Kratom
8854	HTJ0721	Male	Malay	2020	24	Single	Tertiary	Private	Urban	No	Yes	No	No	No	No	No	No	No	No
8855	HTJ0722	Male	Indian	2019	20	Single	Secondary	Unemployed	Urban	No	Yes	Yes	Yes	No	No	No	Yes	No	No
8856	HTJ0723	Female	Chinese	2019	47	Single	No formal education	Unemployed	Urban	No	No	Yes	No	No	No	No	No	No	No
8857	HTJ0724	Female	Chinese	2018	65	Married	No formal education	Self-employed	Urban	Yes	No	No	No	No	No	Yes	No	No	No
8858	HTJ0729	Female	Indian	2018	38	Married	Primary	Unemployed	Rural	Yes	No	Yes	No	No	No	No	No	No	No
8859	HTJ0730	Male	Malay	2019	27	Single	Secondary	Private	Rural	Yes	Yes	Yes	Yes	Yes	Yes	Yes	No	Yes	Yes
8860	HTJ0731	Male	Malay	2021	36	Single	Primary	Self-employed	Rural	No	Yes	Yes	Yes	Yes	No	No	No	No	No
8861	HTJ0732	Male	Malay	2019	27	Single	Tertiary	Self-employed	Rural	Yes	Yes	No	Yes	No	No	No	No	Yes	No
8862	HTJ0735	Male	Chinese	2019	32	Single	Tertiary	Private	Rural	Yes	Yes	Yes	No	Yes	No	No	No	No	No
8863	HTJ0739	Male	Malay	2021	35	Single	Tertiary	Unemployed	Urban	No	No	No	No	Yes	No	No	No	No	No
8864	HTJ0743	Male	Malay	2020	31	Married	Tertiary	Private	Urban	Yes	Yes	No	Yes	Yes	No	No	No	No	Yes
8865	HTJ0746	Male	Malay	2020	32	Single	Tertiary	Self-employed	Urban	No	Yes	No	No	Yes	No	No	No	No	No
8866	HTJ0747	Male	Malay	2021	45	Married	Secondary	Self-employed	Urban	No	Yes	No	No	Yes	No	No	No	No	No
8867	HTJ0748	Male	Indian	2021	31	Married	No formal education	Unemployed	Urban	No	No	Yes	No	No	No	No	No	No	No
8868	HTJ0750	Male	Malay	2020	30	Single	No formal education	Unemployed	Urban	No	Yes	No	No	No	Yes	No	No	No	No
8869	HTJ0751	Male	Malay	2020	37	Married	Tertiary	Self-employed	Urban	No	Yes	Yes	Yes	Yes	No	Yes	No	No	No
8870	HTJ0752	Male	Indian	2020	17	Single	No formal education	Unemployed	Urban	No	Yes	Yes	Yes	Yes	No	No	No	No	No
8871	HTJ0753	Male	Others	2021	34	Married	No formal education	Private	Urban	No	No	Yes	No	No	No	No	No	No	No
8872	HTJ0755	Male	Malay	2021	21	Single	Primary	Private	Urban	No	Yes	Yes	No	Yes	No	No	No	No	No
8873	HTJ0756	Male	Malay	2021	21	Single	Secondary	Private	Urban	No	Yes	No	No	No	No	No	No	No	Yes
8874	HTJ0758	Male	Malay	2021	59	Others	No formal education	Self-employed	Urban	Yes	Yes	No	No	No	No	No	No	Yes	No
8875	HTJ0760	Male	Malay	2021	44	Married	Primary	Unemployed	Rural	Yes	Yes	No	No	No	No	No	No	Yes	No
8876	HTJ0761	Male	Malay	2021	27	Single	Secondary	Unemployed	Urban	Yes	Yes	No	Yes	No	No	No	No	No	No
8877	HTJ0762	Male	Malay	2021	42	Married	Secondary	Private	Urban	No	Yes	No	No	Yes	No	No	No	Yes	No
8878	HTJ0763	Male	Malay	2020	35	Single	Secondary	Unemployed	Rural	Yes	Yes	No	No	Yes	No	No	No	No	No
8879	HTJ0764	Male	Indian	2020	58	Single	No formal education	Self-employed	Rural	No	Yes	Yes	No	No	No	No	No	No	No
8880	HTJ0765	Male	Indian	2021	47	Single	No formal education	Self-employed	Urban	No	Yes	Yes	No	Yes	No	No	No	Yes	No
8881	HTJ0766	Male	Others	2021	27	Single	Primary	Private	Rural	No	No	Yes	Yes	Yes	No	No	No	No	No
8882	HTJ0767	Male	Malay	2020	36	Single	Primary	Self-employed	Rural	Yes	Yes	Yes	Yes	Yes	No	No	No	No	No
8883	HTJ0768	Male	Malay	2021	43	Married	Secondary	Private	Urban	Yes	Yes	No	No	Yes	No	Yes	No	No	Yes
8884	HTJ0769	Male	Chinese	2020	54	Married	Tertiary	Private	Urban	Yes	Yes	No	No	Yes	No	No	No	No	No
8885	HTJ0770	Male	Malay	2021	30	Others	Primary	Private	Urban	No	Yes	Yes	Yes	Yes	No	No	No	Yes	No
8886	HTJ0771	Male	Malay	2021	45	Single	Primary	Unemployed	Rural	No	Yes	No	No	Yes	No	No	No	Yes	No
8887	HTJ0772	Male	Malay	2021	56	Single	No formal education	Unemployed	Rural	Yes	Yes	Yes	Yes	Yes	No	No	No	Yes	No

No	Patient ID	Gender	Ethnic group	Year of diagnosis	Age	Marital status	Education level	Occupation	Place of residence	History of psy illness	Tobacco	Alcohol	Cannabis	ATS	Inhalant	Sedative	Hallucinogens	Opioids	Kratom
8888	HTJ0773	Male	Malay	2020	58	Married	Primary	Private	Rural	Yes	Yes	No	No	No	No	No	No	Yes	No
8889	HTJ0774	Male	Indian	2021	51	Married	Primary	Private	Rural	No	Yes	Yes	No	No	No	No	No	No	No
8890	HTJ0775	Male	Chinese	2021	63	Single	Primary	Self-employed	Rural	No	Yes	No	No	Yes	No	No	No	Yes	No
8891	HTJ0776	Male	Malay	2021	27	Single	Secondary	Private	Rural	Yes	Yes	No	Yes	No	No	No	No	No	No
8892	HTJ0777	Male	Malay	2021	24	Single	Primary	Private	Urban	No	Yes	No	Yes	Yes	No	No	No	No	No
8893	HTJ0778	Male	Indian	2020	26	Single	Secondary	Self-employed	Rural	Yes	Yes	No	Yes	No	No	No	No	No	No
8894	HTJ0779	Male	Malay	2021	34	Others	Primary	Private	Rural	No	Yes	No	No	Yes	No	No	No	No	No
8895	HTJ0780	Male	Malay	2021	23	Single	Tertiary	Self-employed	Urban	No	Yes	No	Yes	Yes	No	Yes	No	No	No
8896	HTJ0781	Male	Malay	2021	25	Single	Secondary	Unemployed	Rural	Yes	Yes	Yes	No	No	No	No	No	No	Yes
8897	HTJ0782	Male	Malay	2021	31	Single	Primary	Unemployed	Rural	No	Yes	No	Yes	Yes	No	No	No	Yes	No
8898	HTJ0783	Male	Malay	2021	28	Others	Secondary	Private	Rural	Yes	Yes	No	No	No	No	No	No	No	Yes
8899	HTJ0784	Male	Malay	2021	28	Married	Tertiary	Private	Urban	No	Yes	Yes	No	No	No	No	No	No	Yes
8900	HTJ0785	Male	Indian	2021	42	Others	Primary	Private	Rural	No	Yes	Yes	No	No	No	No	No	No	No
8901	HTJ0786	Male	Malay	2021	31	Single	Primary	Unemployed	Rural	Yes	Yes	No	No	Yes	No	No	No	Yes	No
8902	HTJ0787	Male	Malay	2021	31	Single	Primary	Self-employed	Rural	No	Yes	Yes	No	Yes	No	No	No	No	No
8903	HTJ0788	Male	Malay	2019	31	Married	Primary	Private	Rural	No	Yes	Yes	No	No	No	No	No	Yes	No
8904	HTJ0789	Male	Others	2020	30	Married	Tertiary	Self-employed	Urban	No	Yes	No	Yes	No	No	No	No	No	No
8905	HTJ0790	Female	Malay	2020	30	Others	Secondary	Unemployed	Rural	No	No	Yes	No	Yes	No	No	No	No	No
8906	HTJ0791	Female	Malay	2020	40	Single	Secondary	Self-employed	Rural	Yes	No	Yes	No	Yes	No	Yes	No	Yes	No
8907	HTJ0792	Male	Malay	2021	28	Single	Primary	Private	Rural	Yes	Yes	No	Yes	Yes	No	No	No	Yes	No
8908	HTJ0793	Female	Others	2021	35	Others	Primary	Self-employed	Rural	Yes	Yes	No	No	Yes	No	No	No	No	No
8909	HTJ0794	Male	Others	2021	24	Single	Primary	Private	Rural	No	Yes	Yes	Yes	No	No	No	No	No	No
8910	HTJ0795	Male	Indian	2021	51	Married	Primary	Self-employed	Urban	Yes	Yes	Yes	No	No	No	No	No	No	No
8911	HTJ0796	Male	Malay	2021	26	Others	Secondary	Private	Rural	No	Yes	Yes	Yes	Yes	No	No	No	No	No
8912	HTJ0797	Male	Malay	2020	27	Single	Tertiary	Unemployed	Urban	Yes	Yes	No	Yes	No	No	No	No	No	No
8913	HTJ0798	Male	Others	2021	53	Married	No formal education	Self-employed	Urban	No	No	Yes	No	No	No	No	No	No	No
8914	HTJ0799	Male	Malay	2020	27	Others	Secondary	Private	Urban	No	Yes	Yes	No	Yes	No	No	No	No	No
8915	HTJ0800	Male	Chinese	2020	64	Married	No formal education	Unemployed	Urban	No	Yes	Yes	No	No	No	No	No	No	No
8916	HTJ0801	Male	Chinese	2021	34	Others	Primary	Private	Urban	No	Yes	Yes	No	Yes	No	No	No	No	No
8917	HTJ0802	Male	Malay	2021	31	Single	Secondary	Private	Urban	Yes	Yes	No	No	No	No	No	No	No	No
8918	HTJ0804	Male	Indian	2020	30	Married	Secondary	Government	Urban	Yes	Yes	Yes	No	No	No	No	No	No	No
8919	HTJ0806	Male	Malay	2020	22	Single	Tertiary	Unemployed	Urban	No	No	Yes	Yes	No	No	No	No	No	No
8920	HTJ0809	Male	Chinese	2021	32	Others	No formal education	Unemployed	Urban	No	Yes	No	No	Yes	No	No	No	No	No
8921	HTJ0811	Female	Malay	2021	20	Single	Tertiary	Private	Urban	No	No	Yes	Yes	No	No	No	No	No	No
8922	HTJ0812	Male	Malay	2021	33	Others	Secondary	Unemployed	Rural	Yes	Yes	No	Yes	Yes	Yes	No	No	No	No
8923	HTJ0813	Male	Malay	2020	18	Single	Secondary	Unemployed	Urban	No	Yes	No	Yes	No	No	No	No	No	No

No	Patient ID	Gender	Ethnic group	Year of diagnosis	Age	Marital status	Education level	Occupation	Place of residence	History of psy illness	Tobacco	Alcohol	Cannabis	ATS	Inhalant	Sedative	Hallucinogens	Opioids	Kratom
8924	HTJ0814	Male	Malay	2021	31	Single	Tertiary	Unemployed	Rural	Yes	Yes	No	Yes	Yes	No	No	No	Yes	No
8925	HTJ0815	Male	Malay	2021	22	Single	Tertiary	Unemployed	Urban	Yes	Yes	Yes	Yes	No	No	No	No	No	No
8926	HTJ0816	Male	Malay	2021	39	Married	Primary	Private	Rural	No	Yes	Yes	No	No	No	No	No	Yes	No
8927	HTJ0817	Female	Chinese	2021	43	Single	Primary	Private	Urban	Yes	No	Yes	Yes	Yes	No	No	No	No	No
8928	HTJ0818	Male	Malay	2021	34	Married	Primary	Government	Urban	No	Yes	No	No	Yes	No	No	No	No	No
8929	HTJ0819	Male	Indian	2021	39	Married	Primary	Private	Rural	No	Yes	Yes	No	No	No	No	No	No	No
8930	HTJ0820	Male	Malay	2021	18	Others	Secondary	Private	Rural	No	Yes	No	Yes	No	No	No	No	No	No
8931	HTJ0821	Male	Indian	2021	39	Married	Primary	Private	Rural	Yes	Yes	Yes	No	No	No	No	No	No	No
8932	HTJ0822	Male	Indian	2021	20	Single	Tertiary	Unemployed	Urban	No	Yes	Yes	Yes	Yes	No	No	Yes	No	No
8933	HTJ0823	Male	Malay	2020	31	Single	Primary	Unemployed	Rural	Yes	Yes	No	Yes	Yes	No	No	No	No	No
8934	HTJ0824	Female	Others	2021	24	Others	Secondary	Unemployed	Rural	Yes	Yes	Yes	No	Yes	No	No	No	No	No
8935	HTJ0825	Male	Malay	2021	40	Others	Primary	Unemployed	Rural	Yes	Yes	Yes	Yes	Yes	No	Yes	No	Yes	No
8936	HTJ0826	Male	Malay	2021	41	Single	Primary	Self-employed	Rural	Yes	Yes	No	No	Yes	No	No	No	Yes	Yes
8937	HTJ0827	Male	Indian	2021	53	Married	Primary	Private	Rural	No	Yes	Yes	No	No	No	No	No	No	No
8938	HTJ0828	Male	Malay	2021	31	Single	Primary	Private	Rural	Yes	Yes	Yes	No	Yes	No	No	No	No	No
8939	HTJ0829	Female	Others	2021	35	Married	Primary	Self-employed	Rural	No	No	Yes	No	Yes	No	No	No	No	No
8940	HTJ0830	Female	Chinese	2021	38	Others	No formal education	Unemployed	Urban	No	Yes	Yes	No	No	No	No	No	No	No
8941	HTJ0831	Male	Indian	2021	45	Married	No formal education	Private	Rural	No	Yes	Yes	No	No	No	No	No	No	No
8942	HTJ0832	Male	Indian	2019	34	Single	Secondary	Private	Rural	No	Yes	Yes	Yes	Yes	No	No	No	Yes	No
8943	HTJ0833	Male	Malay	2021	36	Single	Primary	Self-employed	Urban	No	Yes	No	No	No	No	No	No	No	Yes
8944	HTJ0834	Male	Malay	2019	26	Single	Tertiary	Unemployed	Urban	No	Yes	Yes	Yes	No	No	No	No	No	No
8945	HTJ0835	Male	Malay	2021	36	Single	Primary	Unemployed	Urban	No	Yes	No	No	No	No	No	No	No	Yes
8946	HTJ0836	Male	Others	2021	42	Single	No formal education	Self-employed	Rural	No	No	Yes	No	No	No	No	No	No	No
8947	HTJ0837	Male	Malay	2021	35	Married	No formal education	Private	Urban	No	Yes	No	No	Yes	No	No	No	No	No
8948	HTJ0838	Male	Chinese	2021	66	Married	Primary	Others	Urban	No	No	No	No	No	No	Yes	No	No	No
8949	HTJ0839	Male	Chinese	2021	27	Single	No formal education	Unemployed	Urban	No	Yes	No	Yes	Yes	No	No	No	Yes	No
8950	HTJ0840	Female	Malay	2020	26	Married	Secondary	Self-employed	Rural	Yes	Yes	Yes	No	Yes	No	No	No	Yes	No
8951	HTJ0841	Male	Malay	2019	39	Married	Tertiary	Unemployed	Rural	Yes	Yes	No	No	Yes	No	No	No	No	No
8952	HTJ0842	Male	Malay	2020	36	Single	Tertiary	Private	Rural	Yes	Yes	No	Yes	Yes	No	No	No	Yes	Yes
8953	HTJ0843	Male	Indian	2021	47	Married	Primary	Private	Rural	No	Yes	Yes	No	No	No	No	No	No	No
8954	HTJ0844	Male	Malay	2021	19	Single	Tertiary	Private	Rural	Yes	Yes	No	Yes	Yes	No	No	No	No	No
8955	HTJ0845	Male	Indian	2021	44	Married	Primary	Unemployed	Urban	No	Yes	Yes	No	No	No	No	No	No	No
8956	HTJ0846	Male	Malay	2021	22	Single	Tertiary	Self-employed	Urban	No	Yes	No	Yes	No	No	No	No	No	No
8957	HTJ0847	Female	Chinese	2021	43	Single	Secondary	Unemployed	Urban	Yes	Yes	No	No	Yes	No	Yes	No	No	No
8958	HTJ0848	Female	Malay	2020	22	Married	Secondary	Self-employed	Urban	Yes	Yes	No	Yes	No	No	No	No	No	No
8959	HTJ0849	Male	Indian	2021	41	Married	Primary	Self-employed	Rural	No	Yes	Yes	No	Yes	No	No	No	No	No

No	Patient ID	Gender	Ethnic group	Year of diagnosis	Age	Marital status	Education level	Occupation	Place of residence	History of psy illness	Tobacco	Alcohol	Cannabis	ATS	Inhalant	Sedative	Hallucinogens	Opioids	Kratom
8960	HTJ0850	Male	Indian	2020	30	Single	No formal education	Unemployed	Rural	No	Yes	Yes	No	No	No	No	No	No	No
8961	HTJ0851	Male	Indian	2021	20	Single	Primary	Unemployed	Urban	No	Yes	Yes	Yes	Yes	No	No	No	No	No
8962	HTJ0852	Male	Chinese	2019	55	Single	No formal education	Unemployed	Rural	Yes	No	Yes	No	No	No	No	No	No	No
8963	HTJ0853	Female	Malay	2019	58	Married	Secondary	Unemployed	Urban	No	No	No	No	No	No	No	No	No	Yes
8964	HTJ0854	Female	Chinese	2019	23	Single	Tertiary	Unemployed	Urban	Yes	Yes	No	Yes	No	No	No	No	No	No
8965	HTJ0855	Male	Malay	2018	23	Single	Secondary	Private	Rural	Yes	Yes	Yes	Yes	Yes	No	No	No	No	No
8966	HTJ0856	Female	Malay	2019	17	Single	Primary	Unemployed	Urban	No	Yes	No	No	Yes	No	No	No	No	No
8967	HTJ0857	Male	Indian	2018	27	Married	Tertiary	Private	Urban	No	Yes	Yes	No	Yes	No	Yes	No	No	No
8968	HTJ0858	Male	Malay	2018	33	Single	Primary	Private	Rural	No	Yes	No	No	Yes	No	Yes	No	No	No
8969	HTJ0859	Male	Malay	2018	42	Single	Primary	Unemployed	Rural	No	Yes	Yes	No	Yes	No	No	No	Yes	No
8970	HTJ0860	Male	Malay	2018	31	Single	Primary	Unemployed	Rural	No	Yes	Yes	No	Yes	No	Yes	No	Yes	No
8971	HTJ0861	Male	Chinese	2018	41	Others	Primary	Self-employed	Rural	No	Yes	Yes	No	Yes	No	No	No	No	No
8972	HTJ0862	Male	Malay	2018	20	Single	Primary	Private	Rural	Yes	Yes	Yes	Yes	No	No	No	No	No	No
8973	HTJ0863	Male	Malay	2018	32	Single	Primary	Private	Rural	No	Yes	Yes	No	Yes	No	No	No	Yes	No
8974	HTJ0864	Male	Chinese	2018	33	Single	No formal education	Private	Urban	No	Yes	No	No	Yes	No	No	No	No	No
8975	HTJ0865	Male	Malay	2020	27	Others	Tertiary	Private	Urban	No	Yes	Yes	Yes	Yes	No	No	No	No	No
8976	HTJ0866	Female	Chinese	2020	37	Married	Primary	Unemployed	Rural	Yes	Yes	Yes	No	Yes	No	Yes	No	Yes	No
8977	HTJ0867	Female	Malay	2018	31	Married	Primary	Unemployed	Urban	Yes	Yes	No	Yes	Yes	No	Yes	No	Yes	No
8978	HTJ0868	Male	Malay	2018	56	Married	Primary	Others	Urban	Yes	Yes	No	No	No	No	Yes	No	No	No
8979	HTJ0869	Male	Malay	2020	33	Single	Primary	Unemployed	Urban	Yes	Yes	No	No	Yes	Yes	No	No	No	No
8980	HTJ0871	Female	Malay	2019	21	Married	Primary	Others	Urban	No	Yes	No	No	Yes	No	No	No	No	No
8981	HTJ0872	Female	Malay	2018	28	Married	No formal education	Unemployed	Urban	No	No	No	No	Yes	No	No	No	No	No
8982	HTJ0877	Male	Malay	2019	32	Others	Primary	Self-employed	Urban	No	Yes	No	No	Yes	No	No	No	No	No
8983	HTJ0878	Male	Malay	2019	23	Single	Secondary	Self-employed	Urban	No	No	No	Yes	No	No	No	No	No	No
8984	HTJ0880	Male	Malay	2019	37	Married	Secondary	Private	Urban	Yes	Yes	No	Yes	Yes	No	No	No	Yes	No
8985	HTJ0881	Female	Malay	2018	34	Others	Primary	Private	Rural	No	Yes	Yes	No	Yes	No	No	No	Yes	No
8986	HTJ0882	Female	Chinese	2018	24	Others	Secondary	Private	Urban	No	Yes	Yes	No	Yes	No	No	No	No	No
8987	HTJ0883	Male	Others	2019	38	Married	No formal education	Private	Urban	No	Yes	Yes	No	No	No	No	No	No	No
8988	HTJ0885	Male	Malay	2021	54	Single	Primary	Unemployed	Rural	No	Yes	No	Yes	Yes	No	No	No	Yes	No
8989	HTJ0886	Male	Malay	2018	30	Single	Primary	Unemployed	Rural	No	Yes	Yes	No	Yes	No	No	No	Yes	No
8990	HTJ0887	Male	Indian	2019	42	Married	Secondary	Private	Rural	No	Yes	Yes	No	No	No	No	No	No	No
8991	HTJ0888	Male	Malay	2018	57	Married	Tertiary	Government	Urban	Yes	Yes	No	Yes	No	No	No	No	No	No
8992	HTJ0889	Male	Indian	2019	34	Single	Secondary	Private	Rural	No	Yes	Yes	No	No	No	No	No	No	No
8993	HTJ0890	Female	Indian	2019	62	Married	Primary	Private	Rural	No	No	Yes	No	No	No	No	No	No	No
8994	HTJ0891	Female	Malay	2021	40	Married	Secondary	Unemployed	Urban	No	No	No	Yes	Yes	No	No	No	Yes	No
8995	HTJ0892	Male	Malay	2018	23	Single	Tertiary	Unemployed	Urban	Yes	Yes	No	Yes	No	No	No	No	No	No
8996	HTJ0893	Male	Indian	2021	57	Married	Primary	Unemployed	Rural	No	Yes	Yes	No	No	No	No	No	No	No
8997	HTJ0894	Male	Malay	2020	45	Married	Primary	Self-employed	Rural	No	Yes	No	Yes	No	No	No	No	Yes	No

No	Patient ID	Gender	Ethnic group	Year of diagnosis	Age	Marital status	Education level	Occupation	Place of residence	History of psy illness	Tobacco	Alcohol	Cannabis	ATS	Inhalant	Sedative	Hallucinogens	Opioids	Kratom
8998	HTJ0896	Male	Malay	2020	25	Single	Tertiary	Private	Rural	Yes	Yes	Yes	Yes	No	No	No	No	No	No
8999	HTJ0898	Male	Malay	2020	49	Others	No formal education	Unemployed	Rural	Yes	Yes	No	Yes	Yes	Yes	No	No	Yes	No
9000	HTJ0899	Male	Malay	2020	32	Single	Tertiary	Private	Urban	No	Yes	No	No	Yes	No	No	No	Yes	No
9001	HTJ0904	Male	Malay	2019	19	Single	Secondary	Private	Urban	No	Yes	No	No	Yes	No	No	No	No	No
9002	HTJ0905	Male	Malay	2019	15	Single	Primary	Unemployed	Rural	No	Yes	No	No	No	Yes	No	No	No	No
9003	HTJ0906	Male	Chinese	2018	54	Married	Secondary	Private	Urban	No	Yes	No	No	Yes	No	No	No	Yes	No
9004	HTJ0907	Male	Malay	2019	31	Married	Primary	Unemployed	Rural	Yes	Yes	Yes	No	Yes	No	Yes	No	No	No
9005	HTJ0908	Male	Malay	2019	21	Single	Secondary	Unemployed	Rural	Yes	Yes	Yes	Yes	Yes	Yes	No	No	Yes	No
9006	HTJ0909	Male	Indian	2019	28	Others	Primary	Private	Urban	Yes	Yes	Yes	No	Yes	No	No	No	No	No
9007	HTJ0910	Male	Malay	2018	53	Married	Primary	Private	Urban	Yes	Yes	No	Yes	No	No	No	No	No	No
9008	HTJ0911	Male	Others	2019	28	Single	No formal education	Private	Urban	Yes	No	Yes	Yes	No	No	No	No	No	No
9009	HTJ0912	Male	Indian	2018	37	Single	Primary	Self-employed	Rural	No	Yes	Yes	No	No	No	No	No	No	No
9010	HTJ0915	Male	Indian	2018	37	Married	Tertiary	Private	Urban	No	Yes	Yes	No	No	No	No	No	No	No
9011	HTJ0916	Male	Indian	2018	35	Single	Tertiary	Government	Urban	No	Yes	No	No	Yes	No	Yes	No	No	No
9012	HTJ0917	Male	Chinese	2019	29	Single	Secondary	Unemployed	Rural	Yes	Yes	No	Yes	Yes	No	Yes	No	No	No
9013	HTJ0918	Male	Indian	2018	40	Married	Tertiary	Private	Urban	No	Yes	Yes	No	No	No	No	No	No	No
9014	HTJ0919	Male	Indian	2019	60	Others	Primary	Unemployed	Urban	Yes	No	Yes	No	No	No	No	No	No	No
9015	HTJ0920	Male	Indian	2018	37	Others	Primary	Private	Urban	No	Yes	Yes	No	No	No	No	No	No	No
9016	HTJ0921	Male	Indian	2020	44	Married	Secondary	Private	Urban	No	Yes	Yes	No	No	No	No	No	No	No
9017	HTJ0922	Male	Malay	2018	34	Others	Primary	Private	Rural	No	Yes	Yes	No	No	No	Yes	No	No	No
9018	HTJ0923	Male	Malay	2018	21	Single	Secondary	Self-employed	Rural	Yes	Yes	No	No	Yes	No	No	No	No	No
9019	HTJ0924	Male	Indian	2021	37	Married	Primary	Private	Rural	No	Yes	Yes	No	Yes	No	No	No	Yes	No
9020	HTJ0925	Male	Malay	2018	54	Others	Primary	Unemployed	Rural	Yes	Yes	Yes	No	Yes	No	No	No	Yes	No
9021	HTJ0926	Male	Indian	2018	47	Married	Secondary	Private	Rural	No	Yes	Yes	No	No	No	No	No	No	No
9022	HTJ0927	Male	Chinese	2018	24	Single	Secondary	Unemployed	Urban	No	Yes	No	No	Yes	No	No	No	No	No
9023	HTJ0928	Male	Indian	2018	69	Others	Primary	Private	Urban	No	Yes	Yes	No	No	No	No	No	No	No
9024	HTJ0929	Male	Others	2019	24	Married	No formal education	Unemployed	Rural	No	Yes	No	Yes	No	No	No	No	No	No
9025	HTJ0930	Male	Indian	2018	47	Married	Primary	Unemployed	Urban	No	Yes	Yes	No	No	No	No	No	No	No
9026	HTJ0931	Male	Malay	2019	25	Single	Secondary	Unemployed	Urban	No	Yes	No	No	Yes	No	No	No	No	Yes
9027	HTJ0932	Male	Indian	2019	25	Others	Secondary	Private	Rural	No	Yes	Yes	No	No	No	No	No	No	No
9028	HTJ0933	Male	Malay	2019	35	Married	Primary	Private	Urban	Yes	Yes	No	No	Yes	No	No	No	Yes	No
9029	HTJ0934	Male	Malay	2018	32	Single	Tertiary	Private	Urban	Yes	Yes	No	No	Yes	No	No	No	No	No
9030	HTJ0935	Male	Indian	2018	44	Others	Primary	Others	Urban	No	Yes	Yes	No	No	No	No	No	No	No
9031	HTJ0936	Male	Malay	2019	52	Married	Primary	Private	Rural	No	Yes	No	No	Yes	No	No	No	Yes	No
9032	HTJ0937	Male	Malay	2018	39	Single	No formal education	Unemployed	Rural	No	Yes	No	No	No	No	No	No	Yes	No

No	Patient ID	Gender	Ethnic group	Year of diagnosis	Age	Marital status	Education level	Occupation	Place of residence	History of psy illness	Tobacco	Alcohol	Cannabis	ATS	Inhalant	Sedative	Hallucinogens	Opioids	Kratom
9033	HTJ0938	Male	Chinese	2018	48	Single	No formal education	Unemployed	Urban	Yes	Yes	Yes	Yes	Yes	No	No	No	Yes	No
9034	HTJ0939	Male	Indian	2020	57	Married	Primary	Unemployed	Urban	No	Yes	Yes	No	No	No	No	No	No	No
9035	HTJ0940	Male	Malay	2019	21	Single	No formal education	Others	Urban	No	Yes	No	No	Yes	No	No	No	No	No
9036	HTJ0941	Male	Malay	2019	52	Single	No formal education	Unemployed	Urban	No	Yes	No	No	Yes	No	No	No	No	No
9037	HTJ0942	Male	Malay	2019	32	Single	Secondary	Private	Urban	Yes	Yes	No	No	Yes	No	No	No	No	No
9038	HTJ0945	Male	Indian	2018	46	Others	No formal education	Unemployed	Urban	No	Yes	Yes	No	No	No	No	No	No	No
9039	HTJ0947	Male	Malay	2018	27	Married	Secondary	Unemployed	Urban	No	Yes	Yes	No	No	No	No	No	No	No
9040	HTJ0948	Male	Indian	2019	55	Others	No formal education	Others	Urban	No	No	Yes	No	No	No	No	No	No	No
9041	HTJ0949	Male	Malay	2018	40	Others	No formal education	Unemployed	Urban	No	No	No	No	Yes	No	No	No	Yes	No
9042	HTJ0950	Male	Malay	2020	33	Married	Primary	Unemployed	Urban	Yes	Yes	Yes	Yes	Yes	No	Yes	Yes	No	Yes
9043	HTJ0951	Male	Chinese	2019	32	Others	Secondary	Unemployed	Urban	No	Yes	Yes	No	Yes	No	No	No	No	No
9044	HTJ0952	Male	Malay	2019	21	Single	Secondary	Private	Urban	No	Yes	No	Yes	Yes	No	No	No	No	Yes
9045	HTJ0953	Male	Malay	2020	40	Single	Secondary	Private	Urban	No	Yes	No	Yes	Yes	No	No	No	Yes	No
9046	HTJ0954	Male	Malay	2018	24	Others	Primary	Private	Urban	Yes	Yes	Yes	No	Yes	No	No	No	No	Yes
9047	HTJ0955	Male	Chinese	2018	41	Single	Primary	Self-employed	Rural	Yes	Yes	Yes	No	Yes	No	Yes	No	Yes	No
9048	HTJ0956	Male	Chinese	2020	42	Single	No formal education	Self-employed	Rural	No	Yes	Yes	No	No	No	No	No	No	No
9049	HTJ0957	Male	Malay	2020	33	Married	Primary	Unemployed	Rural	Yes	Yes	No	No	Yes	No	Yes	No	No	No
9050	HTJ0958	Male	Malay	2020	28	Married	Secondary	Private	Rural	No	Yes	Yes	No	No	No	No	No	No	Yes
9051	HTJ0959	Male	Malay	2018	20	Others	Primary	Private	Rural	Yes	Yes	No	No	Yes	No	No	No	No	No
9052	HTJ0960	Male	Chinese	2018	29	Others	No formal education	Unemployed	Rural	No	Yes	No	Yes	Yes	No	Yes	No	No	No
9053	HTJ0961	Male	Malay	2019	20	Single	Primary	Unemployed	Rural	No	Yes	No	Yes	Yes	Yes	No	No	No	Yes
9054	HTJ0962	Male	Malay	2018	34	Married	Secondary	Unemployed	Urban	No	Yes	No	Yes	Yes	No	No	No	No	No
9055	HTJ0963	Male	Indian	2018	25	Single	Secondary	Private	Rural	Yes	Yes	Yes	No	No	No	No	No	No	No
9056	HTJ0964	Male	Indian	2018	38	Single	No formal education	Private	Rural	No	Yes	Yes	No	No	No	No	No	No	No
9057	HTJ0965	Male	Chinese	2019	72	Married	No formal education	Unemployed	Rural	Yes	No	Yes	No	No	No	No	No	No	No
9058	HTJ0966	Male	Indian	2019	32	Married	No formal education	Private	Rural	Yes	Yes	Yes	No	No	No	Yes	No	No	No
9059	HTJ0967	Male	Malay	2019	31	Married	Secondary	Self-employed	Urban	No	Yes	No	No	Yes	No	No	No	No	No
9060	HTJ0968	Male	Malay	2018	42	Single	Primary	Unemployed	Rural	No	Yes	No	Yes	Yes	No	No	No	Yes	No
9061	HTJ0969	Male	Indian	2018	36	Married	Primary	Private	Urban	No	Yes	Yes	No	No	No	No	No	No	No

No	Patient ID	Gender	Ethnic group	Year of diagnosis	Age	Marital status	Education level	Occupation	Place of residence	History of psy illness	Tobacco	Alcohol	Cannabis	ATS	Inhalant	Sedative	Hallucinogens	Opioids	Kratom
9062	HTJ0970	Male	Malay	2018	49	Others	No formal education	Unemployed	Urban	No	No	No	Yes	No	No	No	No	Yes	Yes
9063	HTJ0971	Male	Indian	2019	28	Single	No formal education	Self-employed	Urban	No	No	Yes	No	No	No	No	No	No	No
9064	HTJ0972	Male	Indian	2019	29	Others	No formal education	Unemployed	Urban	Yes	Yes	Yes	No	No	No	No	No	No	No
9065	HTJ0973	Male	Chinese	2019	52	Single	Primary	Private	Urban	No	Yes	Yes	No	No	No	No	No	No	No
9066	HTJ0974	Male	Malay	2018	35	Single	No formal education	Private	Urban	No	No	Yes	No	Yes	No	No	No	No	No
9067	HTJ0975	Male	Chinese	2018	35	Married	No formal education	Others	Urban	No	Yes	Yes	No	No	No	No	No	No	No
9068	HTJ0979	Male	Others	2019	37	Others	No formal education	Private	Urban	No	Yes	Yes	No	No	No	No	No	No	No
9069	HTJ0980	Male	Indian	2018	29	Others	No formal education	Private	Urban	No	No	Yes	No	No	No	Yes	No	No	No
9070	HTJ0981	Male	Indian	2018	24	Single	Secondary	Unemployed	Urban	No	Yes	No	No	Yes	No	No	No	No	No
9071	HTJ0983	Male	Indian	2021	27	Married	Secondary	Others	Urban	No	Yes	Yes	No	No	No	No	No	No	No
9072	HTJ0985	Male	Malay	2018	33	Single	No formal education	Unemployed	Rural	Yes	Yes	Yes	No	Yes	No	No	No	No	No
9073	HTJ0986	Male	Malay	2019	43	Others	Primary	Self-employed	Rural	No	Yes	Yes	No	Yes	No	No	No	No	No
9074	HTJ0987	Female	Malay	2018	27	Others	No formal education	Self-employed	Urban	No	Yes	Yes	No	Yes	No	Yes	No	No	No
9075	HTJ0988	Female	Malay	2020	47	Others	Primary	Self-employed	Urban	No	No	No	No	Yes	No	No	No	No	No
9076	HTJ0989	Male	Indian	2019	41	Married	Primary	Private	Rural	No	Yes	Yes	No	No	No	No	No	No	No
9077	HTJ0990	Female	Malay	2018	53	Others	Primary	Self-employed	Urban	No	Yes	Yes	Yes	Yes	No	No	No	Yes	No
9078	HTJ0991	Male	Indian	2018	49	Married	Primary	Private	Rural	No	Yes	Yes	No	No	No	No	No	No	No
9079	HTJ0992	Male	Others	2019	31	Others	No formal education	Private	Rural	No	No	Yes	No	No	No	No	No	No	No
9080	HTJ0993	Male	Indian	2018	23	Single	Primary	Self-employed	Rural	Yes	Yes	Yes	No	Yes	No	No	No	No	No
9081	HTJ0995	Male	Chinese	2018	47	Single	Primary	Self-employed	Urban	Yes	Yes	Yes	No	No	No	No	No	Yes	No
9082	HTJ0996	Female	Malay	2019	27	Married	Primary	Self-employed	Rural	No	Yes	No	No	Yes	No	No	No	Yes	Yes
9083	HTJ0997	Male	Malay	2019	27	Single	No formal education	Private	Urban	No	Yes	No	No	Yes	No	No	No	No	Yes
9084	HTJ0998	Male	Malay	2018	24	Others	No formal education	Self-employed	Urban	No	Yes	No	Yes	No	No	Yes	No	No	No
9085	HTJ1000	Female	Malay	2018	22	Single	Tertiary	Unemployed	Urban	No	Yes	No	Yes	No	No	No	No	No	No
9086	HTJ1001	Male	Malay	2018	24	Single	Secondary	Self-employed	Urban	No	Yes	No	Yes	No	No	No	No	No	No
9087	HTJ1002	Male	Chinese	2020	37	Others	No formal education	Self-employed	Urban	No	Yes	Yes	No	Yes	No	No	No	No	No
9088	HTJ1003	Female	Chinese	2018	56	Married	Primary	Self-employed	Urban	No	No	Yes	No	No	No	No	No	No	No
9089	HTJ1004	Female	Chinese	2018	48	Others	No formal education	Private	Urban	No	Yes	Yes	No	No	No	No	No	No	No
9090	HTJ1005	Male	Chinese	2018	33	Single	Secondary	Unemployed	Urban	No	Yes	Yes	No	Yes	No	No	No	No	No

No	Patient ID	Gender	Ethnic group	Year of diagnosis	Age	Marital status	Education level	Occupation	Place of residence	History of psy illness	Tobacco	Alcohol	Cannabis	ATS	Inhalant	Sedative	Hallucinogens	Opioids	Kratom
9091	HTJ1006	Male	Malay	2020	32	Married	Primary	Self-employed	Urban	No	Yes	No	No	Yes	No	No	No	No	No
9092	HTJ1007	Male	Malay	2018	39	Others	Primary	Self-employed	Urban	No	Yes	No	No	Yes	No	No	No	No	No
9093	HUS0002	Male	Malay	2020	32	Others	No formal education	Private	Rural	Yes	No	No	No	Yes	No	No	No	No	No
9094	HUS0005	Male	Malay	2018	28	Others	No formal education	Others	Urban	Yes	No	No	No	Yes	No	No	No	No	No
9095	HUS0008	Female	Malay	2018	35	Married	No formal education	Private	Rural	Yes	No	No	No	Yes	No	No	No	No	No
9096	HUS0010	Male	Others	2018	39	Married	Secondary	Government	Rural	Yes	No	Yes	No	No	No	No	No	No	No
9097	HUS0011	Male	Malay	2018	28	Others	No formal education	Others	Rural	Yes	No	No	No	Yes	No	No	No	No	No
9098	HUS0012	Female	Malay	2018	46	Others	No formal education	Unemployed	Rural	Yes	No	No	No	Yes	No	No	No	No	No
9099	HUS0013	Male	Others	2018	29	Others	Secondary	Private	Rural	Yes	No	Yes	No	No	No	No	No	No	No
9100	HUS0014	Male	Malay	2018	31	Others	No formal education	Others	Rural	Yes	No	Yes	No	No	No	No	No	No	No
9101	HUS0015	Male	Malay	2018	28	Others	No formal education	Others	Rural	Yes	No	No	No	Yes	No	No	No	No	No
9102	HUS0016	Female	Others	2018	25	Single	Tertiary	Private	Urban	Yes	No	No	No	Yes	No	No	No	No	No
9103	HUS0017	Male	Malay	2018	27	Single	Secondary	Private	Rural	Yes	No	No	No	Yes	No	No	No	No	No
9104	HUS0019	Male	Others	2018	26	Single	Secondary	Private	Rural	Yes	No	Yes	No	No	No	No	No	No	No
9105	HUS0020	Female	Malay	2018	29	Married	Secondary	Unemployed	Rural	Yes	No	No	No	Yes	No	No	No	No	No
9106	HUS0021	Male	Others	2018	28	Married	Secondary	Private	Urban	Yes	No	No	No	Yes	No	No	No	No	No
9107	HUS0022	Male	Chinese	2018	46	Others	Secondary	Unemployed	Rural	Yes	No	No	Yes	Yes	No	No	No	No	No
9108	HUS0023	Male	Others	2018	51	Married	No formal education	Self-employed	Rural	Yes	No	Yes	No	No	No	No	No	No	No
9109	HUS0026	Male	Chinese	2018	42	Single	Secondary	Private	Rural	Yes	No	Yes	No	No	No	No	No	No	No
9110	HUS0029	Male	Malay	2018	29	Married	Secondary	Self-employed	Urban	Yes	No	Yes	No	No	No	No	No	No	No
9111	HUS0032	Male	Others	2018	24	Single	Secondary	Unemployed	Rural	Yes	No	Yes	No	No	No	No	No	No	No
9112	HUS0035	Male	Others	2018	31	Single	No formal education	Unemployed	Rural	Yes	No	Yes	No	No	No	No	No	No	No
9113	HUS0040	Male	Chinese	2018	42	Married	No formal education	Others	Urban	Yes	No	Yes	Yes	No	No	No	No	No	No
9114	HUS0041	Male	Malay	2018	20	Single	Secondary	Unemployed	Rural	Yes	No	No	Yes	No	No	No	No	No	No
9115	HUS0045	Male	Malay	2019	24	Single	Secondary	Unemployed	Rural	Yes	No	No	No	Yes	No	No	No	No	No
9116	HUS0046	Male	Malay	2019	27	Married	Secondary	Private	Rural	Yes	Yes	Yes	No	No	No	No	No	No	No

No	Patient ID	Gender	Ethnic group	Year of diagnosis	Age	Marital status	Education level	Occupation	Place of residence	History of psy illness	Tobacco	Alcohol	Cannabis	ATS	Inhalant	Sedative	Hallucinogens	Opioids	Kratom
9117	HUS0047	Male	Others	2018	33	Single	No formal education	Private	Rural	Yes	No	No	Yes	Yes	No	No	No	No	No
9118	HUS0048	Male	Malay	2019	33	Married	No formal education	Private	Rural	Yes	No	Yes	No	No	No	No	No	No	No
9119	HUS0049	Male	Others	2018	33	Single	No formal education	Unemployed	Rural	Yes	No	Yes	No	No	No	No	No	No	No
9120	HUS0050	Male	Malay	2018	23	Single	No formal education	Unemployed	Rural	Yes	No	No	Yes	No	No	No	No	No	No
9121	HUS0051	Female	Malay	2018	23	Others	Primary	Unemployed	Rural	Yes	No	No	Yes	Yes	No	No	No	No	No
9122	HUS0052	Male	Others	2018	52	Others	No formal education	Self-employed	Rural	Yes	No	Yes	No	No	No	No	No	No	No
9123	HUS0054	Male	Malay	2018	28	Others	No formal education	Private	Urban	Yes	No	No	No	Yes	No	No	No	No	No
9124	HUS0055	Male	Malay	2018	29	Single	No formal education	Unemployed	Rural	Yes	No	No	No	Yes	No	No	No	No	No
9125	HUS0057	Male	Others	2018	29	Single	Primary	Unemployed	Rural	Yes	No	Yes	No	No	No	No	No	No	No
9126	HUS0058	Male	Malay	2018	29	Married	Tertiary	Government	Urban	Yes	No	No	No	Yes	No	No	No	No	No
9127	HUS0059	Male	Malay	2018	33	Married	Tertiary	Private	Rural	Yes	No	No	No	Yes	No	No	No	No	No
9128	HUS0061	Female	Others	2018	19	Married	Primary	Unemployed	Rural	Yes	No	No	No	Yes	No	No	No	No	No
9129	HUS0063	Male	Malay	2018	26	Single	Secondary	Unemployed	Rural	Yes	No	No	No	Yes	No	No	No	No	No
9130	HUS0064	Male	Malay	2019	34	Single	Secondary	Private	Rural	Yes	No	No	Yes	Yes	No	No	No	No	No
9131	HUS0065	Male	Chinese	2019	22	Single	Secondary	Unemployed	Urban	Yes	No	No	No	Yes	No	No	No	No	No
9132	HUS0067	Male	Others	2019	29	Married	Secondary	Government	Urban	Yes	No	No	No	Yes	No	No	No	No	No
9133	HUS0068	Male	Malay	2019	37	Single	Secondary	Self-employed	Rural	Yes	No	Yes	Yes	Yes	No	No	No	No	No
9134	HUS0069	Male	Others	2018	30	Single	No formal education	Self-employed	Rural	Yes	Yes	Yes	No	No	No	No	No	No	No
9135	HUS0070	Female	Others	2019	16	Single	Secondary	Unemployed	Rural	Yes	Yes	Yes	No	Yes	No	No	No	No	No
9136	HUS0071	Male	Malay	2019	35	Single	Primary	Self-employed	Rural	Yes	Yes	Yes	Yes	Yes	No	No	No	No	No
9137	HUS0072	Male	Malay	2019	33	Married	Tertiary	Government	Urban	Yes	No	No	No	Yes	No	No	No	No	No
9138	HUS0073	Male	Malay	2019	26	Married	Tertiary	Others	Urban	Yes	No	No	No	Yes	No	No	No	No	No
9139	HUS0074	Male	Malay	2020	35	Single	No formal education	Self-employed	Rural	Yes	No	No	Yes	Yes	No	No	No	No	No
9140	HUS0075	Male	Malay	2019	43	Married	Secondary	Unemployed	Rural	Yes	Yes	Yes	No	Yes	No	No	No	No	No
9141	HUS0076	Male	Others	2019	42	Others	Secondary	Self-employed	Rural	Yes	Yes	Yes	No	Yes	No	No	No	No	No
9142	HUS0077	Female	Malay	2019	30	Single	Secondary	Private	Rural	Yes	Yes	Yes	No	No	No	No	No	Yes	No
9143	HUS0078	Male	Malay	2019	23	Single	Secondary	Private	Urban	Yes	Yes	Yes	No	Yes	No	No	No	No	No

No	Patient ID	Gender	Ethnic group	Year of diagnosis	Age	Marital status	Education level	Occupation	Place of residence	History of psy illness	Tobacco	Alcohol	Cannabis	ATS	Inhalant	Sedative	Hallucinogens	Opioids	Kratom
9144	HUS0079	Female	Malay	2019	23	Others	Secondary	Unemployed	Rural	Yes	No	No	No	Yes	No	No	No	No	No
9145	HUS0080	Female	Malay	2019	45	Married	No formal education	Unemployed	Rural	Yes	Yes	No	No	Yes	No	No	No	No	No
9146	HUS0081	Male	Malay	2019	25	Single	Secondary	Self-employed	Rural	Yes	No	No	No	Yes	No	No	No	No	No
9147	HUS0082	Female	Malay	2019	19	Married	No formal education	Unemployed	Rural	Yes	No	No	No	Yes	No	No	No	No	No
9148	HUS0083	Male	Chinese	2018	25	Single	Secondary	Unemployed	Urban	Yes	No	Yes	Yes	Yes	No	No	No	No	No
9149	HUS0084	Female	Others	2019	33	Others	Secondary	Private	Rural	Yes	Yes	Yes	No	Yes	No	No	No	No	No
9150	HUS0088	Male	Malay	2018	31	Single	Secondary	Private	Urban	Yes	Yes	No	No	Yes	No	No	No	No	No
9151	HUS0090	Male	Others	2019	25	Single	Secondary	Unemployed	Urban	Yes	No	No	No	Yes	No	No	No	No	No
9152	HUS0091	Male	Malay	2019	37	Married	Tertiary	Private	Rural	Yes	No	No	Yes	Yes	No	No	No	No	No
9153	HUS0092	Male	Malay	2020	35	Married	Primary	Self-employed	Rural	Yes	Yes	Yes	Yes	Yes	Yes	No	No	No	No
9154	HUS0093	Female	Malay	2020	18	Single	No formal education	Unemployed	Urban	Yes	Yes	Yes	No	Yes	No	No	No	No	No
9155	HUS0094	Male	Malay	2019	28	Married	Secondary	Private	Urban	Yes	No	No	Yes	Yes	No	No	No	No	No
9156	HUS0096	Male	Chinese	2020	22	Single	Secondary	Unemployed	Urban	Yes	Yes	Yes	Yes	Yes	No	No	No	No	No
9157	HUS0097	Male	Malay	2020	24	Single	Secondary	Unemployed	Rural	Yes	No	Yes	Yes	Yes	No	No	No	No	No
9158	HUS0098	Male	Malay	2019	30	Others	Tertiary	Unemployed	Rural	Yes	No	No	No	Yes	No	No	No	No	No
9159	HUS0099	Male	Malay	2020	22	Single	Secondary	Unemployed	Rural	Yes	Yes	Yes	Yes	Yes	No	Yes	No	No	No
9160	HUS0100	Male	Others	2020	25	Single	Tertiary	Unemployed	Urban	Yes	No	No	No	Yes	No	No	No	No	No
9161	HUS0101	Male	Malay	2019	32	Single	No formal education	Unemployed	Urban	Yes	No	No	Yes	Yes	No	No	No	No	No
9162	HUS0103	Male	Others	2020	40	Married	Secondary	Unemployed	Rural	Yes	Yes	No	No	Yes	No	Yes	No	No	No
9163	HUS0104	Male	Others	2020	40	Others	Secondary	Private	Rural	Yes	Yes	Yes	No	Yes	No	No	No	No	No
9164	HUS0105	Male	Malay	2020	30	Single	Secondary	Self-employed	Rural	Yes	Yes	No	No	Yes	No	No	No	No	No
9165	HUS0106	Male	Others	2020	22	Single	No formal education	Private	Rural	Yes	Yes	Yes	No	Yes	No	No	No	No	No
9166	HUS0107	Male	Malay	2020	37	Married	Secondary	Government	Urban	Yes	Yes	Yes	No	Yes	No	No	No	No	No
9167	HUS0108	Male	Malay	2020	28	Married	Primary	Self-employed	Rural	Yes	Yes	No	No	Yes	No	No	No	No	No
9168	HUS0109	Male	Malay	2020	29	Others	Secondary	Private	Urban	Yes	No	No	No	Yes	No	No	No	No	No
9169	HUS0110	Male	Others	2020	34	Married	No formal education	Unemployed	Rural	Yes	No	Yes	No	No	No	No	No	No	No
9170	HUS0112	Male	Chinese	2020	25	Single	Tertiary	Self-employed	Urban	Yes	No	No	Yes	No	No	No	No	No	No
9171	HUS0113	Male	Others	2020	30	Single	Secondary	Private	Rural	Yes	No	Yes	No	No	No	No	No	No	No
9172	HUS0114	Male	Malay	2020	36	Single	No formal education	Self-employed	Urban	Yes	No	No	No	Yes	No	No	No	No	No
9173	HUS0115	Male	Chinese	2020	35	Married	No formal education	Unemployed	Rural	Yes	No	Yes	No	No	No	No	No	No	No
9174	HUS0116	Male	Malay	2020	38	Others	Secondary	Unemployed	Urban	Yes	No	Yes	Yes	Yes	No	No	No	No	No

No	Patient ID	Gender	Ethnic group	Year of diagnosis	Age	Marital status	Education level	Occupation	Place of residence	History of psy illness	Tobacco	Alcohol	Cannabis	ATS	Inhalant	Sedative	Hallucinogens	Opioids	Kratom
9175	HUS0117	Male	Others	2020	55	Married	Primary	Private	Rural	Yes	Yes	Yes	No	No	No	No	No	No	No
9176	HUS0118	Male	Malay	2020	25	Married	Tertiary	Unemployed	Urban	Yes	No	Yes	No	Yes	No	No	No	No	No
9177	HUS0119	Male	Malay	2020	40	Single	Secondary	Private	Rural	Yes	No	Yes	No	No	No	No	No	No	No
9178	HUS0121	Male	Malay	2019	27	Married	Secondary	Private	Urban	Yes	Yes	Yes	No	Yes	No	No	No	No	No
9179	HUS0122	Male	Malay	2021	32	Single	No formal education	Unemployed	Rural	Yes	No	No	No	Yes	No	No	No	No	No
9180	HUS0124	Male	Others	2019	39	Married	Secondary	Private	Urban	Yes	No	Yes	No	No	No	No	No	No	No
9181	HUS0125	Male	Others	2019	25	Single	Secondary	Unemployed	Urban	Yes	No	Yes	No	No	No	No	No	No	No
9182	HUS0129	Male	Malay	2021	20	Single	Secondary	Unemployed	Urban	Yes	No	No	No	Yes	No	No	No	No	No
9183	HUS0130	Female	Malay	2019	18	Married	Secondary	Unemployed	Rural	Yes	Yes	Yes	No	Yes	No	No	No	No	No
9184	HUS0131	Male	Malay	2018	29	Single	No formal education	Self-employed	Rural	Yes	No	No	No	Yes	No	No	No	No	No
9185	HUS0132	Male	Others	2019	52	Married	No formal education	Unemployed	Rural	Yes	Yes	Yes	No	No	No	No	No	No	No
9186	HUS0133	Male	Others	2019	38	Married	Tertiary	Government	Urban	Yes	Yes	Yes	No	No	No	No	No	No	No
9187	HUS0134	Male	Malay	2019	25	Married	Secondary	Private	Rural	Yes	Yes	No	No	Yes	No	No	No	No	No
9188	HUS0135	Male	Others	2019	25	Single	Primary	Unemployed	Urban	Yes	No	Yes	No	No	No	No	No	No	No
9189	HUS0136	Male	Others	2018	50	Married	Secondary	Others	Rural	Yes	No	Yes	No	No	No	No	No	No	No
9190	HUS0137	Female	Malay	2019	34	Married	Tertiary	Unemployed	Urban	No	No	Yes	No	Yes	No	No	No	No	No
9191	HUS0138	Male	Malay	2019	30	Single	Tertiary	Unemployed	Rural	Yes	Yes	Yes	No	Yes	No	No	No	No	No
9192	HUS0139	Male	Malay	2019	41	Others	Secondary	Self-employed	Rural	Yes	Yes	Yes	No	Yes	No	No	No	No	No
9193	HUS0140	Female	Chinese	2019	44	Married	Secondary	Unemployed	Urban	Yes	No	Yes	No	No	No	No	No	No	No
9194	HUS0141	Male	Others	2019	33	Single	Secondary	Private	Rural	Yes	No	Yes	No	No	No	No	No	No	No
9195	HUS0142	Male	Others	2019	59	Married	Secondary	Others	Rural	Yes	No	Yes	No	No	No	No	No	No	No
9196	HUS0144	Male	Malay	2021	30	Others	Secondary	Unemployed	Rural	Yes	No	No	No	Yes	No	No	No	No	No
9197	HUS0146	Male	Malay	2019	35	Married	Secondary	Private	Rural	Yes	No	No	No	Yes	No	No	No	No	No
9198	HUS0147	Male	Malay	2019	38	Married	Secondary	Government	Urban	Yes	Yes	Yes	No	Yes	No	No	No	No	No
9199	HUS0152	Male	Others	2021	31	Married	Secondary	Unemployed	Rural	Yes	No	Yes	Yes	Yes	No	No	No	No	No
9200	HUS0153	Male	Others	2018	40	Single	Secondary	Unemployed	Rural	Yes	Yes	Yes	No	No	No	No	No	No	No
9201	HUS0156	Male	Malay	2020	19	Single	Secondary	Unemployed	Urban	Yes	No	No	Yes	Yes	No	No	No	No	No
9202	HUS0157	Male	Others	2018	29	Married	Secondary	Private	Rural	Yes	No	Yes	No	No	No	No	No	No	No
9203	HUS0158	Male	Malay	2020	18	Single	Secondary	Unemployed	Rural	Yes	No	No	No	Yes	No	No	No	No	No
9204	HUS0159	Male	Others	2020	27	Single	Tertiary	Private	Urban	Yes	No	No	Yes	Yes	No	No	No	No	No
9205	HUS0161	Male	Malay	2021	25	Single	Secondary	Unemployed	Urban	Yes	No	No	No	Yes	No	No	No	No	No
9206	HUS0162	Male	Others	2019	29	Single	Secondary	Unemployed	Rural	Yes	Yes	Yes	No	No	No	No	No	No	No
9207	HUS0163	Male	Others	2019	32	Single	Secondary	Private	Rural	Yes	Yes	Yes	No	No	No	No	No	No	No
9208	HUS0164	Male	Malay	2020	34	Single	Tertiary	Private	Rural	Yes	No	No	No	Yes	No	No	No	No	No
9209	HUS0165	Male	Others	2020	47	Single	Secondary	Private	Urban	Yes	No	Yes	No	No	No	No	No	No	No
9210	HUS0166	Male	Others	2019	49	Married	Secondary	Private	Rural	Yes	Yes	Yes	No	No	No	No	No	No	No
9211	HUS0167	Male	Chinese	2018	43	Single	Secondary	Private	Urban	Yes	Yes	Yes	No	No	No	No	No	No	No

No	Patient ID	Gender	Ethnic group	Year of diagnosis	Age	Marital status	Education level	Occupation	Place of residence	History of psy illness	Tobacco	Alcohol	Cannabis	ATS	Inhalant	Sedative	Hallucinogens	Opioids	Kratom
9212	HUS0168	Male	Malay	2020	17	Single	Secondary	Unemployed	Rural	Yes	Yes	Yes	Yes	Yes	No	No	No	No	No
9213	HUS0170	Male	Others	2019	27	Single	Tertiary	Private	Rural	Yes	No	Yes	No	Yes	No	No	No	No	No
9214	HUS0171	Male	Malay	2020	26	Single	Secondary	Unemployed	Rural	Yes	No	No	No	Yes	No	No	No	No	No
9215	HUS0172	Male	Others	2020	23	Single	Secondary	Unemployed	Rural	Yes	No	No	No	Yes	No	No	No	No	No
9216	HUS0173	Male	Malay	2019	24	Single	Secondary	Unemployed	Rural	Yes	Yes	No	No	Yes	No	No	No	No	No
9217	HUS0174	Male	Malay	2020	21	Single	Secondary	Unemployed	Urban	Yes	No	Yes	Yes	Yes	No	No	No	No	No
9218	HUS0175	Male	Others	2020	27	Single	Tertiary	Private	Urban	Yes	Yes	Yes	No	No	No	No	No	No	No
9219	HUS0176	Male	Indian	2018	56	Married	Secondary	Others	Rural	Yes	Yes	Yes	No	No	No	No	No	No	No
9220	HUS0177	Male	Others	2018	50	Single	No formal education	Others	Rural	Yes	No	Yes	No	No	No	No	No	No	No
9221	HUS0178	Male	Others	2018	28	Married	Secondary	Unemployed	Urban	Yes	Yes	No	No	Yes	No	No	No	No	No
9222	HUS0179	Male	Others	2018	18	Single	Secondary	Unemployed	Urban	Yes	No	Yes	No	No	No	No	No	No	No
9223	HUS0180	Male	Malay	2018	38	Others	Primary	Self-employed	Rural	Yes	No	Yes	No	Yes	No	No	No	No	No
9224	HUS0181	Male	Others	2018	42	Married	Secondary	Government	Urban	Yes	No	Yes	No	No	No	No	No	No	No
9225	HUS0182	Male	Malay	2018	32	Others	Tertiary	Unemployed	Rural	Yes	Yes	Yes	Yes	Yes	No	No	No	No	No
9226	HUS0184	Male	Malay	2019	25	Single	Tertiary	Unemployed	Urban	Yes	Yes	No	No	Yes	No	No	No	No	No
9227	HUS0187	Male	Malay	2019	45	Married	Secondary	Others	Rural	Yes	Yes	Yes	Yes	Yes	No	No	No	No	No
9228	HUS0187	Male	Malay	2019	47	Married	Secondary	Others	Rural	Yes	Yes	Yes	Yes	Yes	No	No	No	No	No
9229	HUS0189	Male	Others	2021	48	Married	Secondary	Unemployed	Rural	Yes	No	Yes	No	No	No	No	No	No	No
9230	HUS0192	Male	Malay	2021	40	Others	No formal education	Private	Urban	Yes	No	No	No	Yes	No	No	No	No	No
9231	HUS0193	Female	Chinese	2020	34	Married	No formal education	Self-employed	Urban	Yes	No	Yes	No	Yes	No	No	No	No	No
9232	HUS0194	Female	Malay	2019	28	Single	Secondary	Private	Rural	Yes	Yes	Yes	No	No	No	No	No	No	No
9233	HUS0195	Male	Others	2020	27	Married	Secondary	Unemployed	Rural	Yes	Yes	Yes	No	No	No	No	No	No	No
9234	HUS0196	Male	Others	2021	32	Married	Secondary	Private	Urban	Yes	No	Yes	No	No	No	No	No	No	No
9235	HUS0197	Male	Malay	2021	26	Single	Secondary	Private	Rural	Yes	Yes	No	No	Yes	No	No	No	No	No
9236	HUS0198	Male	Malay	2020	23	Single	Primary	Unemployed	Urban	Yes	No	Yes	Yes	Yes	No	No	No	No	Yes
9237	HUS0199	Male	Others	2021	32	Married	Secondary	Private	Urban	Yes	No	Yes	No	No	No	No	No	No	No
9238	HUS0200	Male	Malay	2018	47	Others	Secondary	Private	Urban	Yes	Yes	No	Yes	Yes	No	No	No	No	No
9239	HUS0201	Male	Others	2019	32	Married	Primary	Unemployed	Rural	Yes	Yes	Yes	Yes	Yes	No	No	No	No	No
9240	HUS0202	Male	Malay	2020	40	Single	Secondary	Unemployed	Rural	Yes	Yes	Yes	No	No	No	No	No	No	No
9241	HUS0204	Female	Others	2018	33	Others	No formal education	Unemployed	Rural	Yes	No	Yes	No	No	No	No	No	No	No
9242	HUS0205	Male	Malay	2020	40	Married	Secondary	Self-employed	Urban	Yes	No	No	No	Yes	No	No	No	No	No
9243	HUS0206	Male	Malay	2019	23	Single	Tertiary	Self-employed	Urban	Yes	Yes	No	Yes	Yes	No	No	No	No	No

No	Patient ID	Gender	Ethnic group	Year of diagnosis	Age	Marital status	Education level	Occupation	Place of residence	History of psy illness	Tobacco	Alcohol	Cannabis	ATS	Inhalant	Sedative	Hallucinogens	Opioids	Kratom
9244	HUS0207	Male	Malay	2021	25	Single	Secondary	Private	Rural	Yes	Yes	No	No	Yes	No	No	No	No	No
9245	HUS0210	Female	Others	2021	25	Married	Secondary	Unemployed	Rural	Yes	No	No	No	Yes	No	No	No	No	No
9246	HUS0211	Female	Malay	2021	36	Married	No formal education	Unemployed	Rural	Yes	No	No	No	Yes	No	No	No	No	No
9247	HUS0212	Female	Malay	2021	18	Single	Secondary	Unemployed	Rural	No	No	No	Yes	Yes	No	No	No	No	No
9248	HUS0213	Male	Malay	2018	22	Single	Secondary	Private	Rural	Yes	No	No	Yes	Yes	No	No	No	No	No
9249	HUS0214	Male	Malay	2021	41	Others	Secondary	Private	Urban	Yes	No	No	No	Yes	No	No	No	No	No
9250	HUS0215	Male	Malay	2021	34	Single	Secondary	Self-employed	Urban	Yes	No	No	No	Yes	No	No	No	No	No
9251	HUS0216	Male	Malay	2020	44	Others	Secondary	Others	Rural	Yes	No	No	No	Yes	No	No	No	No	No
9252	HUS0217	Male	Others	2019	36	Single	Secondary	Private	Rural	Yes	No	Yes	No	No	No	No	No	No	No
9253	HUS0218	Male	Chinese	2021	48	Married	Secondary	Others	Rural	Yes	No	Yes	No	No	No	No	No	No	No
9254	HUS0219	Male	Others	2020	32	Single	Tertiary	Unemployed	Rural	Yes	Yes	Yes	No	Yes	No	No	No	No	No
9255	HUS0221	Female	Malay	2021	26	Single	Secondary	Private	Rural	Yes	No	Yes	No	Yes	No	No	No	No	No
9256	HUS0222	Male	Malay	2019	25	Single	Secondary	Private	Rural	Yes	Yes	Yes	No	Yes	No	No	No	No	No
9257	HUS0223	Male	Malay	2018	20	Single	Tertiary	Unemployed	Rural	Yes	Yes	Yes	Yes	Yes	No	No	No	No	No
9258	HUS0224	Male	Malay	2019	29	Single	Tertiary	Unemployed	Rural	Yes	No	No	Yes	Yes	No	No	No	No	No
9259	HUS0225	Male	Chinese	2020	44	Married	No formal education	Self-employed	Urban	Yes	No	Yes	No	No	No	No	No	No	No
9260	HUS0227	Male	Malay	2021	26	Married	Secondary	Private	Urban	Yes	No	No	No	Yes	No	No	No	No	Yes
9261	HUS0228	Male	Malay	2020	48	Married	Tertiary	Self-employed	Urban	Yes	Yes	Yes	No	No	No	No	No	No	No
9262	HUS0229	Male	Others	2021	24	Others	Secondary	Unemployed	Rural	Yes	Yes	Yes	No	Yes	No	No	No	No	No
9263	HUS0230	Male	Others	2020	50	Single	Secondary	Unemployed	Rural	Yes	No	Yes	No	No	No	No	No	No	No
9264	HUS0231	Female	Malay	2021	32	Married	Secondary	Unemployed	Rural	Yes	No	No	No	Yes	No	No	No	No	No
9265	HUS0232	Male	Chinese	2021	34	Single	Secondary	Unemployed	Urban	Yes	No	No	No	Yes	No	No	No	No	No
9266	HUS0233	Male	Malay	2021	26	Single	Secondary	Unemployed	Urban	Yes	No	No	No	Yes	No	No	No	No	No
9267	HUS0234	Female	Others	2019	28	Single	No formal education	Unemployed	Urban	Yes	Yes	Yes	No	No	No	No	No	No	No
9268	HUS0235	Male	Others	2021	45	Married	Secondary	Private	Rural	Yes	Yes	Yes	No	No	No	No	No	No	No
9269	HUS0237	Male	Malay	2018	39	Single	Secondary	Self-employed	Rural	Yes	Yes	Yes	Yes	No	Yes	No	No	No	No
9270	HUS0238	Male	Others	2018	56	Married	No formal education	Self-employed	Rural	Yes	Yes	Yes	No	No	No	No	No	No	No
9271	HUS0239	Male	Chinese	2018	43	Others	Secondary	Private	Urban	Yes	Yes	No	No	Yes	No	No	No	No	No
9272	HUS0240	Male	Malay	2020	30	Married	Tertiary	Private	Rural	Yes	Yes	Yes	No	Yes	No	No	No	No	No
9273	HUS0242	Male	Others	2021	32	Married	Secondary	Private	Urban	Yes	Yes	Yes	No	No	No	No	No	No	No
9274	HUS0243	Male	Others	2018	36	Married	Secondary	Private	Urban	Yes	Yes	Yes	No	No	No	No	No	No	No
9275	HUS0244	Male	Malay	2018	30	Single	Secondary	Unemployed	Rural	Yes	No	No	No	Yes	No	No	No	No	No

No	Patient ID	Gender	Ethnic group	Year of diagnosis	Age	Marital status	Education level	Occupation	Place of residence	History of psy illness	Tobacco	Alcohol	Cannabis	ATS	Inhalant	Sedative	Hallucinogens	Opioids	Kratom
9276	HUS0245	Male	Malay	2020	38	Others	Tertiary	Private	Urban	Yes	Yes	No	No	Yes	No	No	No	No	No
9277	HUS0246	Male	Others	2018	33	Married	Secondary	Others	Rural	Yes	Yes	Yes	No	No	No	No	No	No	No
9278	HUS0247	Male	Chinese	2018	31	Single	Tertiary	Unemployed	Urban	Yes	Yes	Yes	Yes	No	No	No	No	No	No
9279	HUS0248	Male	Malay	2020	34	Single	Tertiary	Unemployed	Rural	Yes	Yes	No	No	Yes	No	No	No	No	No
9280	HUS0249	Male	Malay	2018	31	Married	Secondary	Self-employed	Rural	Yes	Yes	No	No	Yes	No	No	No	No	No
9281	HUS0250	Female	Others	2018	21	Single	Tertiary	Unemployed	Urban	Yes	Yes	Yes	No	No	No	No	No	No	No
9282	HUS0252	Male	Malay	2021	36	Married	Tertiary	Unemployed	Rural	No	No	No	No	Yes	No	No	No	No	No
9283	HUS0254	Male	Malay	2020	48	Single	No formal education	Unemployed	Urban	Yes	Yes	No	No	Yes	No	No	No	No	No
9284	HUS0256	Female	Chinese	2019	38	Single	Secondary	Unemployed	Urban	Yes	No	No	No	Yes	No	No	No	No	No
9285	HUS0258	Male	Chinese	2021	44	Others	Tertiary	Others	Urban	Yes	Yes	No	No	Yes	No	No	No	No	No
9286	HUS0262	Male	Malay	2019	21	Single	No formal education	Unemployed	Rural	Yes	No	Yes	No	Yes	No	No	No	No	No
9287	HUS0263	Male	Malay	2020	28	Single	Tertiary	Private	Urban	Yes	Yes	Yes	Yes	Yes	No	No	No	No	No
9288	HUS0264	Male	Others	2021	42	Single	No formal education	Private	Rural	Yes	No	Yes	No	No	No	No	No	No	No
9289	HUS0268	Male	Chinese	2020	40	Others	No formal education	Private	Urban	Yes	No	No	No	Yes	No	No	Yes	No	No
9290	HUS0269	Male	Malay	2021	27	Single	Tertiary	Unemployed	Urban	Yes	No	No	Yes	Yes	No	No	No	No	No
9291	HUS0270	Male	Malay	2020	42	Married	Secondary	Government	Urban	Yes	No	No	No	Yes	No	No	No	No	No
9292	HUS0271	Male	Others	2018	36	Single	Primary	Unemployed	Rural	Yes	No	Yes	No	No	No	No	No	No	No
9293	HUS0272	Male	Malay	2018	30	Others	Secondary	Self-employed	Rural	Yes	Yes	Yes	No	Yes	No	No	No	No	No
9294	HUS0273	Male	Malay	2020	44	Others	No formal education	Private	Rural	Yes	No	No	No	Yes	No	No	No	No	No
9295	HUS0274	Male	Malay	2019	23	Single	Secondary	Private	Rural	Yes	No	Yes	Yes	Yes	No	No	No	No	No
9296	HUS0275	Male	Malay	2019	28	Others	Secondary	Private	Urban	Yes	No	No	Yes	Yes	No	No	No	No	No
9297	HUS0276	Male	Malay	2019	23	Single	Tertiary	Self-employed	Urban	Yes	No	Yes	No	Yes	No	No	No	No	No
9298	HUS0277	Male	Malay	2018	36	Single	Secondary	Unemployed	Urban	Yes	Yes	No	No	Yes	No	No	No	No	No
9299	HUS0278	Male	Others	2021	24	Single	Secondary	Private	Urban	Yes	No	Yes	No	Yes	No	No	No	No	No
9300	HUS0280	Male	Others	2020	34	Single	Tertiary	Unemployed	Rural	Yes	Yes	No	No	Yes	No	No	No	No	No
9301	HUS0281	Male	Malay	2021	24	Single	Secondary	Unemployed	Rural	Yes	No	No	No	Yes	No	No	No	No	No
9302	HUS0282	Male	Malay	2018	19	Single	Secondary	Private	Rural	Yes	No	No	No	Yes	No	No	No	No	No
9303	HUS0283	Male	Indian	2021	22	Single	Tertiary	Unemployed	Rural	Yes	No	No	Yes	No	No	No	No	No	No
9304	HUS0284	Male	Others	2021	41	Married	Tertiary	Private	Urban	Yes	Yes	Yes	Yes	No	No	No	No	No	No
9305	HUS0285	Male	Others	2020	27	Single	Tertiary	Private	Urban	Yes	Yes	Yes	No	No	No	No	No	No	No
9306	HUS0287	Male	Malay	2020	34	Married	Secondary	Private	Rural	Yes	Yes	Yes	Yes	Yes	No	No	No	No	No
9307	HUS0289	Male	Malay	2021	17	Single	Primary	Unemployed	Rural	Yes	No	No	No	Yes	No	No	No	No	No
9308	HUS0291	Male	Malay	2020	41	Married	Secondary	Unemployed	Urban	Yes	No	No	No	Yes	No	No	No	No	No
9309	HUS0292	Male	Malay	2021	51	Married	Secondary	Unemployed	Rural	Yes	Yes	Yes	No	No	No	No	No	No	No
9310	HUS0293	Male	Others	2021	41	Married	Secondary	Government	Rural	Yes	No	Yes	No	No	No	No	No	No	No
9311	HUS0294	Female	Others	2021	23	Single	Tertiary	Unemployed	Urban	Yes	No	Yes	No	No	No	No	No	No	No

No	Patient ID	Gender	Ethnic group	Year of diagnosis	Age	Marital status	Education level	Occupation	Place of residence	History of psy illness	Tobacco	Alcohol	Cannabis	ATS	Inhalant	Sedative	Hallucinogens	Opioids	Kratom
9312	HUS0295	Male	Malay	2021	46	Single	Secondary	Unemployed	Rural	Yes	No	Yes	No	No	No	No	No	No	No
9313	HUS0296	Female	Chinese	2021	21	Single	Tertiary	Private	Urban	Yes	Yes	No	Yes	No	No	No	No	No	No
9314	HUS0297	Male	Malay	2020	21	Single	Secondary	Private	Urban	Yes	Yes	Yes	No	No	No	No	No	No	No
9315	HUS0298	Male	Others	2021	31	Single	Secondary	Private	Urban	Yes	No	No	No	Yes	No	No	No	No	No
9316	HUS0300	Male	Malay	2018	20	Single	Secondary	Unemployed	Rural	Yes	Yes	Yes	Yes	Yes	No	No	No	No	No
9317	HUS0302	Male	Others	2018	29	Single	Secondary	Unemployed	Rural	Yes	Yes	Yes	No	Yes	No	No	No	No	No
9318	HUS0304	Male	Malay	2019	44	Married	No formal education	Private	Rural	Yes	Yes	No	No	No	No	No	No	No	No
9319	HUS0306	Male	Malay	2018	31	Single	Tertiary	Private	Rural	Yes	Yes	No	No	Yes	No	No	No	No	No
9320	HUS0308	Male	Malay	2019	34	Married	Tertiary	Private	Urban	Yes	Yes	Yes	No	No	No	No	No	No	No
9321	HUS0309	Female	Malay	2019	18	Single	Secondary	Unemployed	Rural	Yes	Yes	Yes	No	No	No	No	No	No	No
9322	HUS0310	Male	Others	2018	29	Single	Secondary	Self-employed	Rural	Yes	Yes	Yes	No	No	No	No	No	No	No
9323	HUS0311	Male	Others	2018	18	Single	Secondary	Unemployed	Urban	Yes	No	Yes	Yes	Yes	Yes	No	No	No	No
9324	HUS0312	Male	Others	2018	46	Married	Secondary	Unemployed	Rural	Yes	Yes	Yes	No	No	No	No	No	No	No
9325	HUS0313	Female	Chinese	2018	18	Single	Secondary	Unemployed	Urban	Yes	No	Yes	No	Yes	No	No	No	No	No
9326	HUS0315	Male	Malay	2021	24	Single	Tertiary	Private	Urban	Yes	No	No	Yes	Yes	No	No	No	No	No
9327	HUS0316	Male	Malay	2021	39	Married	Secondary	Private	Rural	Yes	Yes	Yes	Yes	Yes	No	No	No	No	No
9328	HUS0317	Male	Malay	2018	26	Single	Secondary	Others	Rural	Yes	Yes	Yes	No	Yes	No	No	No	No	No
9329	HUS0318	Male	Others	2020	31	Single	Secondary	Unemployed	Rural	Yes	Yes	Yes	No	No	No	No	No	No	No
9330	HUS0319	Male	Malay	2019	46	Married	Secondary	Self-employed	Urban	Yes	No	No	No	No	No	No	No	No	Yes
9331	HUS0320	Male	Malay	2020	23	Single	Tertiary	Unemployed	Urban	Yes	No	No	Yes	Yes	No	No	No	No	No
9332	HUS0323	Male	Malay	2019	34	Married	Secondary	Government	Urban	Yes	Yes	Yes	No	Yes	No	No	No	No	No
9333	HUS0324	Male	Others	2020	35	Married	Secondary	Private	Urban	Yes	No	Yes	No	No	No	No	No	No	No
9334	HUS0325	Male	Malay	2018	42	Single	Primary	Others	Rural	Yes	Yes	Yes	No	No	No	No	No	No	No
9335	HUS0326	Male	Malay	2019	26	Single	Tertiary	Unemployed	Rural	Yes	Yes	No	No	Yes	No	No	No	No	No
9336	HUS0327	Male	Malay	2020	18	Single	Tertiary	Unemployed	Rural	Yes	Yes	No	No	No	No	No	No	No	No
9337	HUS0328	Male	Malay	2019	26	Married	Tertiary	Private	Urban	Yes	No	No	No	Yes	No	No	No	No	No
9338	HUS0329	Male	Malay	2019	24	Single	Secondary	Unemployed	Urban	Yes	No	No	No	Yes	No	No	No	No	No
9339	HUS0331	Female	Malay	2019	25	Married	Secondary	Unemployed	Rural	Yes	Yes	Yes	Yes	Yes	No	No	No	No	No
9340	HUS0332	Male	Malay	2018	26	Married	Secondary	Private	Rural	Yes	Yes	Yes	Yes	Yes	No	No	No	No	No
9341	HUS0334	Male	Malay	2021	47	Married	No formal education	Private	Urban	Yes	Yes	No	No	No	No	No	No	No	No
9342	HUS0335	Female	Chinese	2020	21	Single	Secondary	Private	Urban	Yes	Yes	Yes	No	No	No	No	No	No	No
9343	HUS0338	Female	Chinese	2020	28	Married	Secondary	Others	Urban	Yes	No	Yes	No	No	No	No	No	No	No
9344	HUS0339	Male	Malay	2019	19	Single	Secondary	Unemployed	Rural	Yes	Yes	No	No	No	No	No	No	No	No
9345	HUS0341	Male	Malay	2018	22	Single	Secondary	Unemployed	Rural	Yes	No	Yes	Yes	Yes	No	No	No	No	No
9346	HUS0342	Male	Malay	2019	45	Married	No formal education	Self-employed	Rural	Yes	No	Yes	No	No	No	No	No	No	No
9347	HUS0343	Female	Malay	2018	20	Single	Tertiary	Unemployed	Urban	Yes	No	No	No	No	No	No	No	No	No
9348	HUS0345	Male	Malay	2020	39	Married	Secondary	Government	Urban	Yes	Yes	Yes	No	Yes	No	No	No	No	No

No	Patient ID	Gender	Ethnic group	Year of diagnosis	Age	Marital status	Education level	Occupation	Place of residence	History of psy illness	Tobacco	Alcohol	Cannabis	ATS	Inhalant	Sedative	Hallucinogens	Opioids	Kratom
9349	HUS0346	Male	Chinese	2018	46	Others	No formal education	Unemployed	Urban	Yes	Yes	Yes	No	No	No	No	No	No	No
9350	HUS0347	Male	Malay	2018	24	Single	Tertiary	Unemployed	Urban	Yes	Yes	Yes	No	Yes	No	No	No	No	No
9351	HUS0348	Female	Malay	2021	32	Married	Tertiary	Government	Rural	Yes	Yes	No	No	Yes	No	No	No	No	No
9352	HUS0349	Male	Malay	2020	35	Married	No formal education	Private	Rural	Yes	No	Yes	No	Yes	No	No	No	No	No
9353	HUS0351	Male	Malay	2021	40	Others	Tertiary	Self-employed	Urban	Yes	No	No	No	Yes	No	No	No	No	No
9354	HUS0352	Female	Malay	2019	34	Married	Secondary	Unemployed	Urban	Yes	No	No	No	Yes	No	No	No	No	No
9355	HUS0353	Male	Others	2018	22	Single	Secondary	Private	Rural	Yes	Yes	Yes	No	No	No	No	No	No	No
9356	HUS0354	Male	Chinese	2019	25	Single	Secondary	Self-employed	Urban	Yes	No	No	No	Yes	No	No	No	No	No
9357	HUS0355	Male	Others	2018	26	Single	Tertiary	Private	Urban	Yes	Yes	Yes	No	No	No	No	No	No	No
9358	HUS0356	Male	Malay	2021	46	Single	Secondary	Unemployed	Urban	No	Yes	Yes	No	No	No	No	No	No	No
9359	HUS0359	Female	Chinese	2018	25	Married	Tertiary	Private	Urban	Yes	No	Yes	No	No	No	No	No	No	No
9360	HUS0360	Male	Malay	2018	54	Others	Secondary	Self-employed	Urban	Yes	No	Yes	Yes	Yes	No	No	No	No	No
9361	HUS0363	Male	Others	2019	27	Single	Primary	Private	Rural	Yes	Yes	Yes	No	Yes	No	No	No	No	No
9362	HUS0364	Female	Chinese	2021	32	Married	Secondary	Unemployed	Rural	Yes	Yes	Yes	No	No	No	No	No	No	No
9363	HUS0365	Male	Others	2019	20	Single	Secondary	Unemployed	Rural	Yes	Yes	Yes	No	No	No	No	No	No	No
9364	HUS0366	Male	Others	2020	25	Single	Secondary	Unemployed	Rural	Yes	Yes	Yes	No	No	No	No	No	No	No
9365	HUS0367	Male	Others	2021	70	Married	Primary	Unemployed	Rural	Yes	No	Yes	No	No	No	No	No	No	No
9366	HUS0368	Female	Others	2019	28	Married	Tertiary	Others	Urban	Yes	Yes	Yes	No	No	No	No	No	No	No
9367	HUS0369	Male	Others	2019	27	Single	Secondary	Private	Rural	Yes	No	Yes	No	No	No	No	No	No	No
9368	HUS0372	Male	Chinese	2018	45	Single	Secondary	Unemployed	Rural	Yes	Yes	Yes	No	No	No	No	No	No	No
9369	HUS0374	Male	Malay	2018	38	Single	Tertiary	Unemployed	Urban	Yes	Yes	Yes	Yes	No	No	No	No	No	No
9370	HUS0376	Male	Others	2021	32	Married	Secondary	Private	Rural	Yes	No	Yes	No	No	No	No	No	No	No
9371	HUS0377	Male	Others	2018	43	Others	No formal education	Private	Rural	Yes	No	Yes	No	No	No	No	No	No	No
9372	HUS0378	Male	Malay	2019	33	Single	Tertiary	Others	Urban	Yes	Yes	No	Yes	No	No	No	No	No	No
9373	HUS0379	Male	Others	2019	25	Married	Secondary	Private	Urban	Yes	Yes	Yes	No	No	No	No	No	No	No
9374	HUS0380	Male	Malay	2018	36	Married	No formal education	Private	Rural	Yes	No	No	No	Yes	No	No	No	No	No
9375	HUS0381	Female	Malay	2021	25	Single	Tertiary	Unemployed	Rural	Yes	No	Yes	No	No	No	No	No	No	No
9376	HUS0382	Female	Malay	2019	20	Single	Tertiary	Private	Urban	Yes	Yes	Yes	No	No	No	No	No	No	No
9377	HUS0383	Male	Others	2018	40	Others	Secondary	Private	Rural	Yes	Yes	Yes	No	No	No	No	No	No	No
9378	HUS0384	Female	Malay	2019	17	Single	Primary	Unemployed	Rural	Yes	No	No	No	Yes	No	No	No	No	No
9379	HUS0385	Male	Chinese	2018	28	Single	Tertiary	Private	Urban	Yes	Yes	Yes	No	No	No	No	No	No	No
9380	HUS0386	Male	Others	2020	42	Married	Secondary	Private	Urban	Yes	Yes	Yes	No	No	No	No	No	No	No
9381	HUS0387	Male	Others	2020	58	Married	Secondary	Unemployed	Rural	Yes	No	Yes	No	No	No	No	No	No	No
9382	HUS0388	Male	Others	2020	20	Single	Tertiary	Unemployed	Urban	Yes	No	Yes	No	No	No	No	No	No	No
9383	HUS0389	Female	Others	2018	21	Single	Tertiary	Unemployed	Rural	Yes	Yes	Yes	No	No	No	No	No	No	No
9384	HUS0390	Male	Malay	2019	25	Single	Tertiary	Others	Urban	Yes	Yes	No	Yes	No	No	No	No	No	No
9385	HUS0391	Male	Chinese	2021	28	Single	Secondary	Self-employed	Urban	Yes	Yes	Yes	Yes	Yes	No	No	No	No	No
9386	HUS0393	Male	Malay	2019	26	Single	Secondary	Unemployed	Rural	Yes	Yes	No	No	No	No	No	No	No	No
9387	HUS0394	Male	Chinese	2020	43	Married	Secondary	Self-employed	Rural	Yes	No	No	No	Yes	No	No	No	No	No
9388	HUS0395	Male	Others	2020	31	Single	Secondary	Private	Rural	Yes	Yes	Yes	No	No	No	No	No	No	No

No	Patient ID	Gender	Ethnic group	Year of diagnosis	Age	Marital status	Education level	Occupation	Place of residence	History of psy illness	Tobacco	Alcohol	Cannabis	ATS	Inhalant	Sedative	Hallucinogens	Opioids	Kratom
9389	HUS0396	Male	Malay	2020	26	Single	Tertiary	Unemployed	Rural	Yes	Yes	No	No	Yes	No	No	No	No	No
9390	HUS0397	Male	Malay	2019	39	Married	No formal education	Unemployed	Rural	Yes	Yes	No	No	Yes	No	No	No	No	No
9391	HUS0398	Male	Malay	2020	23	Single	Tertiary	Private	Urban	Yes	Yes	Yes	No	No	No	No	No	No	No
9392	HUS0400	Female	Malay	2018	28	Others	Secondary	Unemployed	Rural	Yes	Yes	Yes	No	Yes	No	No	No	No	No
9393	HUS0402	Male	Malay	2021	41	Others	No formal education	Unemployed	Rural	Yes	No	No	No	Yes	No	No	No	No	No
9394	HUS0403	Male	Malay	2020	21	Single	Tertiary	Unemployed	Rural	Yes	Yes	Yes	Yes	No	No	No	No	No	No
9395	HUS0404	Female	Malay	2018	24	Single	Primary	Unemployed	Rural	Yes	Yes	No	Yes	Yes	No	No	No	No	No
9396	HUS0405	Female	Others	2019	25	Single	Tertiary	Unemployed	Urban	Yes	Yes	Yes	No	No	No	No	No	No	No
9397	HUS0406	Female	Malay	2021	23	Married	No formal education	Unemployed	Rural	Yes	Yes	Yes	No	No	No	No	No	No	No
9398	HUS0407	Male	Others	2018	27	Married	Secondary	Private	Urban	Yes	Yes	No	No	Yes	No	No	No	No	No
9399	HUS0408	Female	Chinese	2020	17	Single	Secondary	Unemployed	Urban	Yes	No	No	No	No	No	No	No	Yes	No
9400	HUS0409	Male	Others	2019	23	Single	No formal education	Unemployed	Rural	Yes	Yes	Yes	No	Yes	No	No	No	No	No
9401	HUS0410	Male	Malay	2019	34	Others	Secondary	Private	Urban	Yes	Yes	Yes	Yes	Yes	No	No	No	No	No
9402	HUS0411	Female	Malay	2021	31	Single	Tertiary	Government	Urban	Yes	Yes	Yes	Yes	No	No	No	No	Yes	No
9403	HUS0412	Female	Chinese	2020	49	Married	Primary	Unemployed	Urban	Yes	No	Yes	No	No	No	No	No	No	No
9404	HUS0413	Male	Chinese	2018	43	Others	Secondary	Others	Urban	Yes	Yes	Yes	No	Yes	No	No	No	No	No
9405	HUS0414	Male	Malay	2018	26	Others	Secondary	Others	Rural	Yes	Yes	No	No	Yes	No	No	No	No	No
9406	HUS0415	Male	Malay	2020	21	Single	Tertiary	Private	Rural	Yes	Yes	Yes	No	No	No	No	No	No	No
9407	HUS0416	Male	Malay	2020	29	Single	Secondary	Private	Rural	Yes	No	No	Yes	Yes	No	No	No	No	No
9408	HUS0417	Male	Malay	2019	29	Single	Secondary	Private	Urban	Yes	Yes	No	No	Yes	No	No	No	No	No
9409	HUS0418	Male	Malay	2020	36	Single	Tertiary	Private	Urban	Yes	No	Yes	Yes	No	No	No	No	No	No
9410	HUS0419	Male	Malay	2018	39	Single	Secondary	Unemployed	Rural	Yes	Yes	Yes	Yes	No	Yes	No	No	No	No
9411	HUS0423	Female	Others	2021	28	Others	Secondary	Private	Urban	Yes	Yes	Yes	No	No	No	No	No	No	No
9412	HUS0426	Male	Malay	2020	40	Married	No formal education	Self-employed	Urban	Yes	No	No	No	Yes	No	No	No	No	No
9413	HUS0427	Male	Malay	2018	26	Married	Secondary	Unemployed	Urban	Yes	No	No	Yes	Yes	No	No	No	No	No
9414	HUS0428	Male	Malay	2020	22	Single	Tertiary	Unemployed	Urban	Yes	Yes	Yes	No	No	No	No	No	No	No
9415	HUS0430	Male	Malay	2021	28	Single	Tertiary	Private	Rural	Yes	Yes	No	Yes	No	No	No	No	No	No
9416	HUS0431	Male	Chinese	2019	56	Married	No formal education	Unemployed	Urban	Yes	Yes	Yes	No	No	No	No	No	No	No
9417	HUS0435	Male	Malay	2019	31	Others	Secondary	Unemployed	Rural	Yes	Yes	No	No	Yes	No	No	No	No	No
9418	HUS0436	Female	Others	2019	20	Single	Tertiary	Unemployed	Urban	Yes	Yes	Yes	Yes	No	No	No	No	No	No
9419	HUS0438	Male	Malay	2018	30	Single	Tertiary	Unemployed	Urban	Yes	Yes	Yes	No	Yes	No	No	No	No	No
9420	HUS0439	Male	Malay	2019	21	Single	Tertiary	Unemployed	Rural	Yes	Yes	No	Yes	Yes	No	No	No	No	No
9421	HUS0440	Male	Others	2018	18	Single	Primary	Unemployed	Rural	Yes	No	No	No	Yes	No	No	No	No	No
9422	HUS0441	Male	Others	2020	35	Single	Secondary	Private	Urban	Yes	No	Yes	No	Yes	No	No	No	No	No
9423	HUS0443	Male	Others	2018	22	Single	Secondary	Private	Rural	Yes	Yes	Yes	Yes	Yes	No	No	No	No	No
9424	HUS0444	Female	Malay	2021	19	Single	Secondary	Unemployed	Rural	No	Yes	Yes	No	Yes	No	No	No	No	No
9425	HUS0446	Female	Malay	2018	23	Single	Secondary	Self-employed	Rural	Yes	Yes	No	Yes	Yes	No	No	No	No	No

No	Patient ID	Gender	Ethnic group	Year of diagnosis	Age	Marital status	Education level	Occupation	Place of residence	History of psy illness	Tobacco	Alcohol	Cannabis	ATS	Inhalant	Sedative	Hallucinogens	Opioids	Kratom
9426	HUS0449	Male	Others	2021	58	Married	No formal education	Others	Urban	Yes	Yes	Yes	No	No	No	No	No	No	No
9427	HUS0450	Female	Malay	2020	24	Single	Tertiary	Unemployed	Urban	Yes	Yes	Yes	No	No	No	No	No	No	No
9428	HUS0452	Male	Malay	2018	40	Single	Secondary	Unemployed	Rural	Yes	No	Yes	Yes	Yes	No	No	No	No	No
9429	HUS0453	Male	Malay	2018	33	Married	Secondary	Private	Rural	Yes	No	Yes	No	Yes	No	No	No	No	No
9430	HUS0454	Male	Others	2018	21	Single	Secondary	Private	Rural	Yes	Yes	Yes	Yes	Yes	No	No	No	No	No
9431	HUS0455	Male	Chinese	2019	24	Single	Tertiary	Private	Urban	Yes	Yes	Yes	No	No	No	No	No	No	No
9432	HUS0457	Male	Malay	2019	44	Married	Secondary	Unemployed	Rural	No	Yes	No	No	Yes	No	No	No	No	No
9433	HUS0458	Male	Others	2018	36	Married	Secondary	Private	Urban	Yes	Yes	Yes	No	No	No	No	No	No	No
9434	HUS0461	Male	Malay	2021	39	Single	Secondary	Private	Urban	Yes	No	No	Yes	Yes	No	No	No	No	No
9435	HUS0463	Male	Malay	2019	40	Married	Tertiary	Self-employed	Urban	Yes	Yes	No	Yes	Yes	No	No	No	No	No
9436	HUS0464	Female	Malay	2019	18	Married	Secondary	Unemployed	Rural	Yes	Yes	Yes	No	Yes	No	No	No	No	No
9437	HUS0465	Male	Malay	2018	19	Single	Secondary	Unemployed	Rural	Yes	Yes	Yes	Yes	No	No	No	No	No	No
9438	HUS0466	Male	Malay	2021	27	Single	Secondary	Private	Rural	Yes	No	No	No	Yes	No	No	No	No	No
9439	HUS0468	Male	Chinese	2021	41	Married	Secondary	Self-employed	Urban	Yes	No	Yes	No	Yes	No	No	No	No	No
9440	HUS0469	Male	Malay	2021	47	Single	Secondary	Unemployed	Rural	Yes	No	Yes	No	No	No	No	No	No	No
9441	HUS0470	Male	Chinese	2018	31	Single	Tertiary	Private	Urban	Yes	Yes	Yes	Yes	No	No	No	No	No	No
9442	HUS0472	Female	Others	2020	21	Single	Tertiary	Unemployed	Rural	Yes	Yes	Yes	No	No	No	No	No	No	No
9443	HUS0477	Male	Others	2020	33	Married	Tertiary	Government	Urban	Yes	Yes	Yes	No	No	No	No	No	No	No
9444	HUS0479	Male	Others	2019	22	Single	Tertiary	Unemployed	Urban	Yes	No	Yes	No	No	No	No	No	No	No
9445	HUS0480	Male	Others	2019	30	Single	Tertiary	Government	Urban	Yes	Yes	No	Yes	Yes	No	No	No	No	No
9446	HUS0485	Male	Chinese	2021	57	Married	Secondary	Private	Urban	Yes	Yes	Yes	No	No	No	No	No	No	No
9447	HUS0486	Female	Others	2021	34	Others	Secondary	Private	Urban	Yes	Yes	Yes	No	No	No	No	No	No	No
9448	HUS0491	Male	Others	2018	38	Married	Secondary	Private	Rural	Yes	No	Yes	No	Yes	No	No	No	No	No
9449	HUS0492	Female	Others	2018	23	Single	Tertiary	Unemployed	Urban	Yes	Yes	Yes	No	No	No	No	No	No	No
9450	HUS0493	Male	Others	2020	43	Married	Secondary	Private	Rural	Yes	Yes	Yes	No	No	No	No	No	No	No
9451	HUS0494	Female	Malay	2020	22	Single	Secondary	Unemployed	Urban	Yes	Yes	Yes	No	No	No	No	No	No	No
9452	HUS0497	Male	Malay	2021	39	Married	Secondary	Private	Rural	Yes	Yes	Yes	No	Yes	No	No	No	No	No
9453	HUS0498	Female	Malay	2021	36	Married	Secondary	Unemployed	Rural	Yes	Yes	Yes	No	No	No	No	No	No	No
9454	HUS0501	Female	Chinese	2018	33	Single	Tertiary	Unemployed	Urban	Yes	Yes	Yes	Yes	Yes	No	No	No	No	No
9455	HUS0503	Male	Chinese	2020	36	Single	Secondary	Self-employed	Urban	Yes	Yes	Yes	No	No	No	No	No	No	No
9456	HUS0505	Male	Malay	2021	36	Married	Primary	Private	Rural	Yes	Yes	Yes	No	Yes	No	No	No	No	No
9457	HUS0506	Male	Chinese	2019	23	Single	Secondary	Private	Urban	Yes	Yes	No	No	Yes	No	No	No	No	No
9458	HUS0507	Male	Chinese	2020	42	Single	No formal education	Private	Urban	Yes	Yes	No	No	Yes	No	No	No	No	No
9459	HUS0508	Male	Others	2020	28	Single	Secondary	Private	Urban	Yes	Yes	Yes	No	Yes	No	No	No	No	No
9460	HUS0510	Male	Others	2021	35	Married	Tertiary	Private	Urban	Yes	Yes	Yes	No	No	No	No	No	No	No
9461	HUS0513	Male	Others	2020	18	Single	Tertiary	Unemployed	Urban	Yes	Yes	Yes	No	No	No	No	No	No	No

No	Patient ID	Gender	Ethnic group	Year of diagnosis	Age	Marital status	Education level	Occupation	Place of residence	History of psy illness	Tobacco	Alcohol	Cannabis	ATS	Inhalant	Sedative	Hallucinogens	Opioids	Kratom
9462	HUS0516	Male	Malay	2018	19	Single	Secondary	Unemployed	Urban	Yes	Yes	No	Yes	No	No	No	No	No	No
9463	HUS0517	Male	Others	2019	24	Single	Secondary	Private	Urban	Yes	Yes	Yes	No	No	No	No	No	No	No
9464	HUS0518	Male	Malay	2019	22	Single	Secondary	Unemployed	Urban	Yes	Yes	Yes	Yes	Yes	Yes	No	No	No	No
9465	HUS0519	Female	Malay	2021	22	Single	Secondary	Unemployed	Urban	Yes	Yes	Yes	No	No	No	No	No	No	No
9466	HUS0520	Male	Others	2021	23	Single	Secondary	Private	Rural	Yes	Yes	Yes	No	No	No	No	No	No	No
9467	HUS0521	Male	Chinese	2020	34	Single	Secondary	Private	Urban	Yes	Yes	Yes	No	No	No	No	No	No	No
9468	HUS0522	Male	Malay	2020	47	Married	Secondary	Unemployed	Rural	Yes	Yes	Yes	No	No	No	No	No	No	No
9469	HUS0523	Male	Malay	2021	24	Single	Secondary	Others	Rural	Yes	Yes	Yes	No	No	No	No	No	No	No
9470	HUS0524	Male	Malay	2021	34	Married	Secondary	Private	Rural	Yes	Yes	Yes	No	No	No	No	No	No	No
9471	HUS0525	Male	Chinese	2021	51	Single	Tertiary	Private	Urban	Yes	No	Yes	No	No	No	No	No	No	No
9472	HUS0526	Male	Others	2021	49	Married	Secondary	Private	Urban	Yes	Yes	Yes	No	No	No	No	No	No	No
9473	HUS0527	Male	Malay	2021	26	Single	Secondary	Government	Rural	Yes	Yes	No	Yes	No	No	No	No	No	No
9474	HUS0528	Male	Malay	2021	28	Single	Secondary	Private	Rural	Yes	Yes	Yes	No	Yes	No	No	No	No	No
9475	HUS0529	Male	Chinese	2020	49	Married	Secondary	Private	Urban	Yes	Yes	Yes	No	Yes	No	No	No	No	No
9476	HUS0530	Male	Malay	2020	35	Married	Tertiary	Government	Rural	Yes	Yes	Yes	No	No	No	No	No	No	No
9477	HUS0531	Male	Malay	2018	26	Others	Tertiary	Unemployed	Rural	Yes	Yes	Yes	No	No	No	No	No	No	No
9478	HUS0534	Male	Others	2020	35	Married	Secondary	Private	Urban	Yes	No	Yes	No	No	No	No	No	No	No
9479	HUS0535	Male	Malay	2018	40	Single	Secondary	Others	Rural	Yes	Yes	Yes	No	No	No	No	No	No	No
9480	HUS0536	Male	Others	2021	28	Single	Secondary	Private	Rural	Yes	Yes	Yes	No	Yes	No	No	No	No	No
9481	HUS0539	Male	Malay	2021	42	Married	Secondary	Others	Rural	Yes	Yes	No	No	Yes	Yes	No	No	No	No
9482	HUS0540	Male	Others	2018	34	Single	Tertiary	Private	Urban	Yes	Yes	Yes	No	No	No	No	No	No	No
9483	HUS0542	Male	Others	2018	39	Married	Tertiary	Government	Urban	Yes	Yes	Yes	No	No	No	No	No	No	No
9484	HUS0543	Female	Malay	2021	22	Married	Tertiary	Unemployed	Rural	Yes	No	Yes	No	No	No	No	No	No	No
9485	HUS0544	Male	Malay	2021	20	Single	Secondary	Unemployed	Rural	Yes	Yes	No	Yes	No	No	No	No	No	No
9486	HUS0545	Male	Malay	2020	24	Single	Secondary	Unemployed	Rural	Yes	No	Yes	Yes	Yes	No	No	No	No	No
9487	HUS0546	Female	Others	2021	35	Married	Secondary	Unemployed	Urban	Yes	No	Yes	No	No	No	No	No	No	No
9488	HUS0547	Male	Malay	2021	18	Single	Secondary	Unemployed	Urban	Yes	Yes	No	Yes	No	No	No	No	No	No
9489	HUS0549	Male	Chinese	2018	59	Single	Secondary	Unemployed	Urban	Yes	Yes	Yes	No	No	No	No	No	No	No
9490	HUS0550	Female	Malay	2021	25	Single	Tertiary	Unemployed	Urban	Yes	Yes	No	Yes	No	No	No	No	No	No
9491	HUS0551	Male	Malay	2021	47	Married	Tertiary	Self-employed	Rural	Yes	Yes	Yes	No	No	No	No	No	No	No
9492	HUS0552	Male	Others	2021	35	Single	Secondary	Private	Urban	Yes	Yes	Yes	No	Yes	No	No	No	No	No
9493	HUS0553	Male	Malay	2021	26	Single	Secondary	Self-employed	Rural	Yes	Yes	No	No	Yes	No	No	No	No	No
9494	HUS0554	Male	Malay	2021	57	Single	No formal education	Unemployed	Rural	Yes	Yes	Yes	No	Yes	No	No	No	No	No
9495	HUS0555	Male	Malay	2021	30	Single	Secondary	Others	Rural	Yes	No	Yes	Yes	Yes	No	No	No	No	Yes
9496	HUS0556	Male	Malay	2021	36	Married	Tertiary	Self-employed	Rural	Yes	No	No	Yes	Yes	No	No	No	No	No
9497	HUS0558	Male	Malay	2018	25	Single	Primary	Others	Rural	Yes	Yes	Yes	No	Yes	No	No	No	No	No
9498	HUS0559	Female	Chinese	2019	38	Single	Secondary	Private	Urban	Yes	No	No	No	Yes	No	No	No	No	No
9499	HUS0561	Male	Malay	2018	23	Single	Secondary	Unemployed	Rural	Yes	Yes	Yes	No	Yes	No	No	No	No	No
9500	HUS0563	Male	Malay	2021	26	Single	Secondary	Private	Urban	Yes	No	No	No	Yes	No	No	No	No	Yes
9501	HUS0565	Male	Malay	2018	28	Others	Secondary	Private	Rural	Yes	Yes	No	No	Yes	No	No	No	No	No
9502	HUS0566	Male	Malay	2018	31	Single	Secondary	Unemployed	Rural	Yes	Yes	No	No	Yes	No	No	No	No	No

No	Patient ID	Gender	Ethnic group	Year of diagnosis	Age	Marital status	Education level	Occupation	Place of residence	History of psy illness	Tobacco	Alcohol	Cannabis	ATS	Inhalant	Sedative	Hallucinogens	Opioids	Kratom
9503	HUS0567	Male	Malay	2018	45	Single	Secondary	Unemployed	Urban	Yes	Yes	Yes	No	No	No	No	No	No	No
9504	HUS0568	Male	Malay	2018	35	Single	Secondary	Private	Urban	Yes	Yes	Yes	No	No	No	No	No	No	No
9505	HUS0571	Male	Chinese	2018	42	Others	Secondary	Unemployed	Urban	Yes	Yes	Yes	No	Yes	No	No	No	No	No
9506	HUS0574	Male	Chinese	2019	37	Single	Secondary	Private	Urban	Yes	No	No	Yes	Yes	No	No	No	No	No
9507	HUS0575	Female	Others	2018	38	Married	Primary	Unemployed	Rural	Yes	No	Yes	No	No	No	No	No	No	No
9508	HUS0577	Male	Others	2019	22	Single	Primary	Unemployed	Urban	Yes	Yes	Yes	No	Yes	No	No	No	No	No
9509	HUS0578	Male	Others	2019	23	Married	Secondary	Private	Rural	Yes	Yes	Yes	No	No	No	No	No	No	No
9510	HUS0579	Female	Malay	2021	18	Single	Secondary	Unemployed	Urban	Yes	Yes	Yes	No	No	No	No	No	No	No
9511	HUS0580	Male	Malay	2021	20	Single	Tertiary	Unemployed	Urban	Yes	Yes	Yes	No	No	No	No	No	No	No
9512	HUS0581	Male	Malay	2021	22	Single	Tertiary	Private	Rural	Yes	Yes	Yes	Yes	No	No	No	No	No	No
9513	HUS0582	Male	Malay	2018	38	Others	Primary	Others	Rural	Yes	Yes	Yes	No	No	Yes	No	No	No	No
9514	HUS0584	Female	Malay	2018	25	Single	Tertiary	Unemployed	Urban	Yes	No	Yes	Yes	Yes	No	No	No	No	No
9515	HUS0585	Female	Malay	2019	32	Married	Secondary	Unemployed	Urban	Yes	Yes	No	No	Yes	No	No	No	No	No
9516	HUS0587	Male	Malay	2020	35	Married	Secondary	Private	Urban	Yes	No	Yes	Yes	Yes	No	No	No	No	No
9517	HUS0588	Male	Malay	2021	36	Married	Secondary	Government	Rural	Yes	Yes	No	Yes	Yes	No	No	No	No	No
9518	HUS0589	Female	Malay	2021	23	Married	Secondary	Unemployed	Urban	Yes	Yes	No	No	Yes	No	No	No	No	No
9519	HUS0590	Female	Malay	2020	24	Single	Tertiary	Government	Urban	Yes	Yes	Yes	No	No	No	No	No	No	No
9520	HUS0591	Male	Malay	2019	27	Single	Tertiary	Private	Urban	Yes	Yes	Yes	Yes	Yes	No	No	No	No	No
9521	HUS0592	Male	Malay	2020	34	Single	Tertiary	Private	Urban	Yes	No	No	No	Yes	No	No	No	No	No
9522	HUS0593	Male	Others	2021	35	Married	Tertiary	Unemployed	Urban	Yes	Yes	Yes	No	No	No	No	No	No	No
9523	HUS0594	Male	Malay	2019	35	Single	Primary	Others	Rural	Yes	Yes	Yes	No	No	No	No	No	No	No
9524	HUS0595	Male	Malay	2021	40	Others	Tertiary	Self-employed	Urban	Yes	Yes	No	No	Yes	No	No	No	No	No
9525	HUS0596	Male	Malay	2019	37	Married	Tertiary	Private	Rural	Yes	Yes	No	Yes	Yes	No	No	No	No	No
9526	HUS0597	Male	Others	2021	32	Married	Secondary	Private	Urban	Yes	Yes	Yes	No	No	No	No	No	No	No
9527	HUS0598	Male	Others	2020	29	Single	Tertiary	Private	Rural	Yes	Yes	Yes	No	No	No	No	No	No	No
9528	HUS0599	Male	Malay	2021	31	Married	Secondary	Private	Urban	Yes	Yes	Yes	Yes	Yes	No	No	No	No	No
9529	HUS0600	Male	Chinese	2020	20	Single	Secondary	Unemployed	Urban	Yes	No	Yes	No	No	No	No	No	No	No
9530	HUS0601	Male	Others	2021	31	Married	Secondary	Self-employed	Urban	Yes	Yes	Yes	No	No	No	No	No	No	No
9531	HUS0602	Male	Others	2021	21	Single	Secondary	Unemployed	Rural	Yes	Yes	Yes	No	No	No	No	No	No	No
9532	HUS0603	Male	Malay	2018	22	Married	Secondary	Unemployed	Rural	Yes	Yes	Yes	Yes	Yes	No	No	No	No	No
9533	HUS0604	Male	Malay	2021	40	Others	Secondary	Unemployed	Rural	Yes	Yes	Yes	No	Yes	Yes	No	No	No	No
9534	HUS0605	Male	Malay	2019	46	Married	Secondary	Unemployed	Rural	Yes	Yes	Yes	No	No	No	No	No	No	No
9535	HUS0606	Male	Malay	2021	23	Single	Secondary	Self-employed	Rural	Yes	Yes	Yes	No	No	No	No	No	No	No
9536	HUS0607	Male	Malay	2021	25	Married	No formal education	Self-employed	Urban	Yes	No	No	Yes	Yes	No	No	No	No	No
9537	HUS0609	Male	Malay	2019	34	Married	Secondary	Government	Rural	Yes	Yes	Yes	No	Yes	No	No	No	No	No
9538	HUS0610	Male	Malay	2021	25	Single	Secondary	Self-employed	Rural	Yes	No	No	Yes	Yes	No	No	No	No	No
9539	HUS0611	Female	Malay	2021	18	Single	Secondary	Unemployed	Urban	Yes	Yes	Yes	No	Yes	No	No	No	No	No
9540	HUS0612	Female	Chinese	2020	18	Single	Secondary	Unemployed	Urban	Yes	Yes	Yes	No	No	No	No	No	No	No
9541	HUS0613	Female	Malay	2020	22	Others	Tertiary	Unemployed	Rural	Yes	Yes	Yes	No	No	No	No	No	No	No
9542	HUS0614	Male	Malay	2019	40	Others	Secondary	Others	Rural	Yes	Yes	Yes	No	No	No	No	No	No	No
9543	HUS0615	Male	Chinese	2020	30	Single	Tertiary	Private	Urban	Yes	No	No	No	Yes	No	No	No	No	No

No	Patient ID	Gender	Ethnic group	Year of diagnosis	Age	Marital status	Education level	Occupation	Place of residence	History of psy illness	Tobacco	Alcohol	Cannabis	ATS	Inhalant	Sedative	Hallucinogens	Opioids	Kratom
9544	HUS0616	Male	Others	2021	18	Single	Secondary	Private	Urban	Yes	Yes	Yes	No	No	No	No	No	No	No
9545	HUS0618	Male	Chinese	2018	40	Single	Secondary	Others	Urban	Yes	No	Yes	No	Yes	Yes	No	No	No	No
9546	HUS0619	Female	Others	2021	22	Single	Tertiary	Unemployed	Urban	Yes	Yes	Yes	No	No	No	No	No	No	No
9547	HUS0620	Male	Malay	2018	33	Married	Secondary	Government	Urban	Yes	Yes	No	No	Yes	No	No	No	No	No
9548	HUS0621	Male	Malay	2018	22	Single	Tertiary	Unemployed	Urban	Yes	Yes	No	No	Yes	Yes	No	No	No	No
9549	HUS0622	Male	Others	2020	39	Others	No formal education	Unemployed	Rural	Yes	No	Yes	No	No	No	No	No	No	No
9550	HUS0623	Female	Malay	2021	26	Single	Tertiary	Private	Urban	Yes	Yes	Yes	No	No	No	No	No	No	No
9551	HUS0625	Male	Chinese	2021	56	Married	Secondary	Private	Urban	Yes	Yes	Yes	No	Yes	No	No	No	No	No
9552	HUS0626	Male	Chinese	2019	27	Single	Tertiary	Unemployed	Urban	Yes	Yes	Yes	Yes	Yes	No	No	No	No	No
9553	HUS0627	Male	Chinese	2020	45	Single	Secondary	Private	Urban	No	No	No	No	Yes	No	No	No	No	No
9554	HUS0628	Male	Chinese	2020	34	Married	Secondary	Self-employed	Rural	Yes	No	Yes	No	Yes	No	No	No	No	No
9555	HUS0629	Female	Others	2019	20	Single	Secondary	Unemployed	Rural	Yes	No	No	No	Yes	No	No	No	No	No
9556	HUS0631	Male	Chinese	2019	36	Married	Tertiary	Unemployed	Rural	Yes	Yes	Yes	No	Yes	No	No	No	No	No
9557	HUS0635	Male	Others	2021	33	Married	Secondary	Private	Urban	Yes	No	Yes	No	Yes	No	No	No	No	No
9558	HUS0636	Male	Others	2020	41	Others	Secondary	Private	Urban	Yes	Yes	Yes	No	No	No	No	No	No	No
9559	HUS0637	Male	Others	2019	40	Married	Secondary	Government	Urban	No	Yes	Yes	No	No	No	No	No	No	No
9560	HUS0640	Male	Malay	2018	28	Single	Tertiary	Unemployed	Urban	Yes	Yes	Yes	Yes	No	No	No	No	No	No
9561	HUS0643	Male	Malay	2020	49	Married	Tertiary	Self-employed	Urban	Yes	Yes	Yes	No	No	No	No	No	No	No
9562	HUS0644	Male	Malay	2021	51	Married	Primary	Unemployed	Rural	Yes	Yes	Yes	No	No	No	No	No	No	No
9563	HUS0645	Male	Chinese	2020	54	Married	No formal education	Unemployed	Urban	Yes	Yes	No	No	Yes	No	No	No	No	No
9564	HUS0646	Male	Others	2018	38	Married	Primary	Private	Rural	Yes	Yes	Yes	No	Yes	No	No	No	No	No
9565	HUS0647	Female	Malay	2018	35	Single	Secondary	Private	Rural	Yes	No	Yes	No	Yes	No	No	No	No	No
9566	HUS0648	Male	Others	2021	55	Married	Secondary	Unemployed	Rural	Yes	Yes	Yes	No	No	No	No	No	No	No
9567	HUS0652	Male	Chinese	2021	45	Married	Secondary	Private	Urban	Yes	Yes	Yes	No	No	No	No	No	No	No
9568	HUS0653	Male	Chinese	2019	39	Married	Tertiary	Private	Urban	Yes	Yes	Yes	No	No	No	No	No	No	No
9569	HUS0655	Male	Chinese	2020	37	Single	Tertiary	Private	Urban	Yes	Yes	Yes	No	No	No	No	No	No	No
9570	HUS0656	Male	Chinese	2018	31	Single	Tertiary	Private	Urban	Yes	Yes	Yes	Yes	No	No	No	No	No	No
9571	HUS0657	Male	Malay	2019	28	Single	Secondary	Unemployed	Urban	Yes	Yes	Yes	No	No	No	No	No	No	No
9572	HUS0658	Female	Others	2021	20	Single	Tertiary	Unemployed	Urban	Yes	Yes	Yes	Yes	No	No	No	No	No	No
9573	HUS0660	Male	Chinese	2018	49	Single	Secondary	Private	Urban	Yes	Yes	Yes	No	Yes	No	No	No	No	No
9574	HUS0662	Male	Chinese	2020	32	Married	Secondary	Government	Rural	Yes	Yes	Yes	No	No	No	No	No	No	No
9575	HUS0665	Female	Malay	2020	26	Single	Secondary	Unemployed	Rural	Yes	Yes	Yes	No	Yes	No	No	No	No	No
9576	HUS0671	Female	Others	2020	29	Single	Tertiary	Government	Urban	Yes	Yes	Yes	No	No	No	No	No	No	No
9577	HUS0672	Female	Malay	2019	20	Single	Secondary	Unemployed	Rural	Yes	Yes	No	No	Yes	No	No	No	No	No
9578	HUS0673	Female	Others	2021	23	Married	Secondary	Unemployed	Urban	Yes	Yes	Yes	No	No	No	No	No	No	No
9579	HUS0675	Male	Chinese	2019	25	Single	Secondary	Unemployed	Urban	Yes	No	No	No	Yes	No	No	No	No	No
9580	HUS0676	Male	Chinese	2020	20	Single	Secondary	Private	Urban	Yes	Yes	Yes	No	No	No	No	No	No	No
9581	HUS0677	Female	Chinese	2020	22	Single	Secondary	Private	Rural	Yes	No	Yes	No	No	No	No	No	No	No
9582	HUS0678	Male	Malay	2019	40	Single	Secondary	Unemployed	Rural	Yes	Yes	Yes	Yes	No	No	No	No	No	No

No	Patient ID	Gender	Ethnic group	Year of diagnosis	Age	Marital status	Education level	Occupation	Place of residence	History of psy illness	Tobacco	Alcohol	Cannabis	ATS	Inhalant	Sedative	Hallucinogens	Opioids	Kratom
9583	HUS0679	Male	Others	2020	47	Married	Secondary	Private	Urban	Yes	Yes	Yes	No	No	No	No	No	No	No
9584	HUS0681	Male	Chinese	2018	43	Single	Secondary	Private	Urban	Yes	Yes	Yes	No	No	No	No	No	No	No
9585	HUS0682	Male	Others	2018	27	Single	Secondary	Private	Urban	Yes	Yes	Yes	No	No	No	No	No	No	No
9586	HUS0683	Male	Malay	2020	34	Married	No formal education	Others	Rural	Yes	Yes	Yes	No	Yes	No	No	No	No	No
9587	HUS0684	Male	Others	2018	18	Single	Tertiary	Unemployed	Urban	Yes	Yes	Yes	No	Yes	No	No	No	No	No
9588	HUS0685	Male	Malay	2020	39	Married	Secondary	Government	Urban	Yes	Yes	Yes	No	Yes	No	No	No	No	No
9589	HUS0686	Male	Malay	2019	47	Married	Secondary	Government	Rural	Yes	Yes	Yes	No	No	No	No	No	No	No
9590	HUS0688	Male	Malay	2019	37	Single	No formal education	Others	Urban	Yes	No	No	Yes	Yes	No	No	No	No	No
9591	HUS0689	Male	Others	2021	50	Married	Secondary	Government	Urban	Yes	Yes	Yes	No	No	No	No	No	No	No
9592	HUS0690	Male	Chinese	2021	22	Single	Tertiary	Unemployed	Urban	Yes	Yes	No	Yes	No	No	No	No	No	No
9593	HUS0691	Male	Chinese	2021	29	Single	Secondary	Unemployed	Urban	Yes	Yes	Yes	No	No	No	No	No	No	No
9594	HUS0693	Male	Others	2020	37	Married	Secondary	Government	Urban	Yes	Yes	Yes	No	No	No	No	No	No	No
9595	HUS0696	Male	Others	2019	25	Single	Tertiary	Unemployed	Rural	Yes	No	No	Yes	No	Yes	No	No	No	No
9596	HUS0697	Male	Others	2018	30	Single	Secondary	Private	Urban	Yes	Yes	Yes	No	No	No	No	No	No	No
9597	HUS0701	Male	Malay	2020	22	Single	No formal education	Unemployed	Urban	Yes	Yes	Yes	No	No	No	No	No	No	No
9598	HUS0702	Female	Malay	2018	25	Single	Tertiary	Self-employed	Urban	Yes	No	No	Yes	No	No	No	No	No	No
9599	HUS0704	Male	Malay	2018	26	Others	Secondary	Private	Rural	Yes	No	Yes	No	Yes	No	No	No	No	No
9600	HUS0705	Male	Malay	2019	34	Others	Primary	Unemployed	Rural	Yes	Yes	Yes	No	Yes	No	No	No	No	No
9601	HUS0706	Male	Others	2018	50	Married	Primary	Private	Rural	Yes	Yes	Yes	No	No	No	No	No	No	No
9602	HUS0707	Male	Malay	2020	18	Single	Primary	Unemployed	Rural	Yes	Yes	Yes	No	No	No	No	No	No	No
9603	HUS0708	Female	Others	2020	20	Single	Tertiary	Unemployed	Urban	Yes	Yes	Yes	No	No	No	No	No	No	No
9604	HUS0711	Male	Chinese	2018	33	Single	Tertiary	Unemployed	Urban	Yes	Yes	Yes	No	No	No	No	No	No	No
9605	HUS0712	Male	Malay	2021	39	Married	Secondary	Private	Rural	No	Yes	Yes	No	Yes	No	No	No	No	No
9606	HUS0714	Male	Malay	2018	41	Single	Secondary	Unemployed	Urban	Yes	No	Yes	Yes	Yes	No	No	No	No	No

No	Patient ID	Other substances	No of substances abused at a time	Reason for substance use initiation
1	HKB0004	No	1	Peer pressure
2	HKB0008	No	1	-
3	HKB0011	No	1	-
4	HKB0045	Yes	4	Job problem, Relaxation
5	HKB0046	Yes	5	Relationship, Peer pressure
6	HKB0048	No	5	Relaxation
7	HKB0049	No	3	Peer pressure
8	HKB0050	No	4	-
9	HKB0053	No	3	Peer pressure
10	HKB0054	No	2	Job problem, Peer pressure
11	HKB0055	No	2	Family issue
12	HKB0062	Yes	5	Peer pressure
13	HKB0063	No	5	Job problem
14	HKB0066	No	1	Relationship
15	HKB0068	No	3	Peer pressure
16	HKB0070	No	3	-
17	HKB0071	Yes	2	-
18	HKB0072	No	3	Peer pressure
19	HKB0073	No	1	Others
20	HKB0074	No	2	Relaxation
21	HKB0075	Yes	2	-
22	HKB0076	Yes	2	Joy seeking
23	HKB0079	No	1	Peer pressure
24	HKB0080	No	2	Peer pressure
25	HKB0081	No	1	-
26	HKB0082	No	4	Peer pressure
27	HKB0083	No	2	-
28	HKB0084	No	2	Joy seeking
29	HKB0085	No	1	-
30	HKB0086	No	1	Peer pressure
31	HKB0088	No	3	Job problem
32	HKB0091	No	3	-

No	Patient ID	Other substances	No of substances abused at a time	Reason for substance use initiation
33	HKB0092	No	3	Job problem
34	HKB0093	No	1	-
35	HKB0095	No	2	-
36	HKB0096	No	1	Relationship
37	HKB0097	No	1	Peer pressure
38	HKB0098	No	3	Family issue, Relaxation, Peer pressure
39	HKB0099	No	4	Relaxation
40	HKB0100	No	3	-
41	HKB0101	No	3	Others
42	HKB0102	No	2	Peer pressure
43	HKB0103	No	1	-
44	HKB0104	No	3	-
45	HKB0105	No	5	-
46	HKB0110	No	4	Job problem, Joy seeking
47	HKB0111	No	5	Family issue
48	HKB0112	No	1	-
49	HKB0113	No	3	Others
50	HKB0114	No	2	-
51	HKB0115	No	3	Relaxation, Peer pressure
52	HKB0119	No	4	Relaxation
53	HKB0120	No	2	Peer pressure
54	HKB0121	No	4	-
55	HKB0122	Yes	4	Health
56	HKB0123	No	4	Family issue, Others
57	HKB0124	No	2	Peer pressure
58	HKB0125	No	2	Relaxation
59	HKB0126	No	4	-
60	HKB0130	No	2	Others

No	Patient ID	Other substances	No of substances abused at a time	Reason for substance use initiation
61	HKB0133	No	1	-
62	HKB0136	No	4	-
63	HKB0137	No	5	-
64	HKB0138	No	4	-
65	HKB0141	No	1	-
66	HKB0142	Yes	2	-
67	HKB0144	No	4	-
68	HKB0145	No	2	Others
69	HKB0146	No	1	-
70	HKB0148	No	1	-
71	HKB0149	No	1	-
72	HKB0151	No	4	-
73	HKB0152	No	3	Health
74	HKB0153	No	4	Health, Relaxation
75	HKB0154	No	4	Peer pressure
76	HKB0157	No	3	Peer pressure
77	HKB0158	No	3	-
78	HKB0159	No	2	Peer pressure
79	HKB0160	No	2	Peer pressure
80	HKB0161	No	2	Joy seeking
81	HKB0164	No	1	-
82	HKB0166	No	1	-
83	HKB0168	No	1	Relaxation
84	HKB0169	No	1	Others
85	HKB0171	No	7	Peer pressure, Financial problem
86	HKB0173	No	1	Health
87	HKB0174	No	1	-
88	HKB0175	No	6	Peer pressure
89	HKB0176	No	1	-
90	HKB0177	No	2	-
91	HKB0179	No	4	-
92	HKB0180	No	2	Others
93	HKB0181	No	4	Peer pressure
94	HKB0185	No	2	-

No	Patient ID	Other substances	No of substances abused at a time	Reason for substance use initiation
95	HKB0186	Yes	3	Health, Peer pressure
96	HKB0187	No	3	Joy seeking, Peer pressure
97	HKB0188	No	2	Peer pressure
98	HKB0189	No	4	-
99	HKB0190	No	4	-
100	HKB0192	No	3	Others
101	HKB0193	No	2	Peer pressure
102	HKB0194	No	2	-
103	HKB0195	No	5	-
104	HKB0196	No	4	Peer pressure
105	HKB0197	No	2	-
106	HKB0198	No	4	Peer pressure
107	HKB0199	No	3	Relaxation
108	HKB0200	No	2	Others
109	HKB0202	No	2	-
110	HKB0207	No	1	-
111	HKB0212	No	5	-
112	HKB0222	No	2	-
113	HKB0225	No	2	-
114	HKB0226	No	2	-
115	HKB0228	No	1	-
116	HKB0240	No	2	-
117	HKB0241	No	2	Relaxation
118	HKB0244	No	2	Peer pressure
119	HKB0246	No	2	-
120	HKB0247	No	2	Job problem, Peer pressure
121	HKB0250	No	1	-
122	HKB0251	No	2	-
123	HKB0257	No	1	-
124	HKB0260	No	1	-

No	Patient ID	Other substances	No of substances abused at a time	Reason for substance use initiation
125	HKB0264	No	1	-
126	HKB0266	No	2	Peer pressure
127	HKB0272	No	2	-
128	HKB0273	No	1	-
129	HKB0278	No	1	-
130	HKB0280	No	2	-
131	HKB0282	No	2	Others
132	HKB0286	No	2	Relaxation
133	HKB0295	No	3	-
134	HKB0300	No	2	-
135	HKB0302	No	1	-
136	HKB0303	No	1	Peer pressure
137	HKB0305	No	1	-
138	HKB0311	No	2	Peer pressure
139	HKB0313	No	1	-
140	HKB0315	No	1	-
141	HKB0316	No	1	-
142	HKB0327	No	2	-
143	HKB0328	No	1	-
144	HKB0338	No	1	-
145	HKB0339	No	1	Health
146	HKB0347	No	4	Family issue
147	HKB0348	No	1	-
148	HKB0359	No	3	Peer pressure
149	HKB0362	No	1	-
150	HKB0365	No	3	Joy seeking
151	HKB0370	No	1	-
152	HKB0383	No	1	-

No	Patient ID	Other substances	No of substances abused at a time	Reason for substance use initiation
153	HKB0388	No	1	-
154	HKB0389	No	1	Joy seeking
155	HKB0394	No	3	-
156	HKB0395	No	1	-
157	HKB0396	No	1	-
158	HKB0402	No	1	-
159	HKB0403	No	1	-
160	HKB0408	No	2	Peer pressure
161	HKB0410	No	2	-
162	HKB0412	No	1	-
163	HKB0414	No	1	-
164	HKB0415	No	1	-
165	HKB0416	No	2	-
166	HKB0419	No	1	-
167	HKB0420	No	1	-
168	HKB0422	No	1	-
169	HKB0427	No	1	-
170	HKB0433	No	1	-
171	HKB0436	No	1	-
172	HKB0437	No	1	-
173	HKB0438	No	1	-
174	HKB0441	No	2	Peer pressure
175	HKB0454	No	2	-
176	HKB0455	No	1	Relaxation
177	HKB0457	No	1	-
178	HKB0460	No	1	-
179	HKB0462	No	1	-

No	Patient ID	Other substances	No of substances abused at a time	Reason for substance use initiation
180	HKB0465	No	3	Family issue
181	HKB0468	No	1	Peer pressure
182	HKB0476	No	2	-
183	HKB0477	No	1	-
184	HKB0481	No	1	-
185	HKB0485	No	2	-
186	HKB0486	No	1	Others
187	HKB0489	No	1	Others
188	HKB0489	No	3	Others
189	HKB0491	No	2	-
190	HKB0493	No	2	Others
191	HKB0494	No	1	-
192	HKB0496	Yes	2	-
193	HKB0497	No	1	-
194	HKB0498	No	1	Family issue
195	HKB0503	No	4	-
196	HKB0505	No	2	Health, Joy seeking
197	HKB0509	No	1	-
198	HKB0510	No	1	-
199	HKB0512	No	1	-
200	HKB0516	No	1	-
201	HKB0519	No	1	-
202	HKB0521	No	1	-
203	HKB0526	No	1	-
204	HKB0528	No	1	-
205	HKB0529	No	1	-
206	HKB0530	No	1	-
207	HKB0532	No	1	Relaxation
208	HKB0536	No	3	-
209	HKB0537	No	3	-
210	HKB0542	No	1	Relationship
211	HKB0543	No	1	-

No	Patient ID	Other substances	No of substances abused at a time	Reason for substance use initiation
212	HKB0549	No	3	Relaxation
213	HKB0552	No	1	-
214	HKB0554	No	1	Others
215	HKB0555	No	1	-
216	HKB0556	No	3	-
217	HKB0558	No	3	Others
218	HKB0559	No	2	-
219	HKB0560	No	2	-
220	HKB0561	No	1	-
221	HKB0564	No	1	-
222	HKB0566	No	1	-
223	HKB0567	No	2	-
224	HKB0568	No	1	Health, Peer pressure
225	HKB0569	No	3	-
226	HKB0573	No	3	-
227	HKB0574	Yes	1	Peer pressure
228	HKB0582	No	3	-
229	HKB0591	No	1	Others
230	HKB0592	No	1	-
231	HKB0593	No	3	Others
232	HKB0598	No	1	-
233	HKB0599	No	2	-
234	HKB0599	No	3	-
235	HKB0600	No	1	-
236	HKB0601	No	3	-
237	HKB0605	No	1	-
238	HKB0612	No	1	-
239	HKB0615	No	2	Peer pressure
240	HKB0617	No	4	-
241	HKB0619	No	1	-
242	HKB0622	No	1	-

No	Patient ID	Other substances	No of substances abused at a time	Reason for substance use initiation
243	HKB0623	No	2	Peer pressure
244	HKB0628	No	2	Peer pressure, Others
245	HKB0632	No	1	Relaxation
246	HKB0634	No	1	Others
247	HKB0637	No	1	-
248	HKB0639	No	2	-
249	HKB0642	No	1	-
250	HKB0646	No	1	-
251	HKB0648	No	2	-
252	HKB0649	No	1	Relaxation
253	HKB0654	No	2	-
254	HKB0656	No	1	-
255	HKB0658	No	2	-
256	HKB0659	No	1	-
257	HKB0660	No	1	Health
258	HKB0665	No	1	-
259	HKB0667	No	2	Peer pressure
260	HKB0668	No	2	-
261	HKB0669	No	1	-
262	HKB0670	No	1	-
263	HKB0670	No	3	-
264	HKB0672	No	4	Others
265	HKB0673	No	1	-
266	HKB0682	No	2	Joy seeking
267	HKB0683	No	1	-
268	HKB0688	No	1	-
269	HKB0691	No	3	Peer pressure
270	HKB0693	No	1	-
271	HKB0695	No	1	-
272	HKB0700	No	2	Peer pressure
273	HKB0701	No	3	Others
274	HKB0706	No	1	-
275	HKB0707	No	4	-

No	Patient ID	Other substances	No of substances abused at a time	Reason for substance use initiation
276	HKB0711	No	1	-
277	HKB0715	No	2	-
278	HKB0717	No	2	-
279	HKB0718	No	1	-
280	HKB0722	No	2	-
281	HKB0723	No	1	-
282	HKB0726	No	1	-
283	HKB0733	No	1	-
284	HKB0735	No	4	Peer pressure
285	HKB0737	No	2	-
286	HKB0738	No	1	-
287	HKB0739	No	2	Others
288	HKB0741	No	1	Peer pressure
289	HKB0742	No	1	-
290	HKB0744	No	1	Others
291	HKB0751	No	1	-
292	HKB0752	No	1	-
293	HKB0753	No	2	Peer pressure
294	HKB0758	No	1	-
295	HKB0759	No	1	Peer pressure
296	HKB0766	No	1	-
297	HKB0776	No	1	-
298	HKB0778	No	1	-
299	HKB0779	No	4	Joy seeking
300	HKB0780	No	1	Others
301	HKB0781	No	1	-
302	HKB0787	No	3	-
303	HKB0790	No	1	-
304	HKB0791	No	3	-
305	HKB0793	No	2	-

No	Patient ID	Other substances	No of substances abused at a time	Reason for substance use initiation
306	HKB0796	No	1	Peer pressure
307	HKB0810	No	2	-
308	HKB0811	No	1	-
309	HKB0816	No	3	Job problem
310	HKB0817	No	2	-
311	HKB0818	No	1	-
312	HKB0821	No	2	-
313	HKB0825	No	2	-
314	HKB0827	No	3	-
315	HKB0831	No	1	Job problem
316	HKB0834	No	2	Health
317	HKB0835	No	1	-
318	HKB0837	No	1	Health, Job problem
319	HKB0840	No	1	-
320	HKB0842	No	5	-
321	HKB0850	No	1	Others
322	HKB0851	No	1	Peer pressure
323	HKB0852	No	2	-
324	HKB0853	No	3	Peer pressure
325	HKB0854	No	3	Peer pressure
326	HKB0855	No	1	-
327	HKB0856	No	3	Joy seeking
328	HKB0857	No	1	-
329	HKB0865	No	1	-
330	HKB0866	No	1	-
331	HKB0868	No	1	-
332	HKB0869	No	1	-
333	HKB0874	No	1	-
334	HKB0881	No	1	Relaxation

No	Patient ID	Other substances	No of substances abused at a time	Reason for substance use initiation
335	HKB0882	No	3	Health
336	HKB0884	No	1	-
337	HKB0887	No	1	-
338	HKB0890	No	1	-
339	HKB0891	No	1	-
340	HKB0893	No	2	-
341	HKB0894	No	3	Peer pressure
342	HKB0895	No	2	-
343	HKB0897	No	1	-
344	HKB0898	No	1	-
345	HKB0900	No	2	Others
346	HKB0901	No	1	Others
347	HKB0903	No	1	-
348	HKB0904	No	1	-
349	HKB0905	No	1	Others
350	HKB0908	No	1	-
351	HKB0910	No	1	-
352	HKB0913	No	3	Peer pressure
353	HKB0916	No	1	-
354	HKB0917	No	1	-
355	HKB0921	No	1	-
356	HKB0923	No	1	Health
357	HKB0925	No	4	-
358	HKB0927	No	2	Family issue, Job problem, Relaxation, Financial problem
359	HKB0934	No	1	-
360	HKB0937	No	1	Health
361	HKB0939	No	1	-
362	HKB0940	No	1	-
363	HKB0943	No	1	Peer pressure

No	Patient ID	Other substances	No of substances abused at a time	Reason for substance use initiation
364	HKB0944	No	1	-
365	HKB0949	No	3	Joy seeking
366	HKB0950	No	1	-
367	HKB0955	No	1	Health
368	HKB0956	No	1	-
369	HKB0958	No	1	-
370	HKB0960	No	2	-
371	HKB0961	No	1	-
372	HKB0962	No	4	Peer pressure
373	HKB0963	No	1	-
374	HKB0974	No	3	-
375	HKB0979	No	4	Health
376	HKB0981	No	1	-
377	HKB0987	No	2	-
378	HKB0989	No	1	-
379	HKB1000	No	2	-
380	HKB1003	No	1	-
381	HKB1005	No	4	-
382	HKB1006	Yes	3	-
383	HKB1011	No	1	-
384	HKB1015	No	1	-
385	HKB1016	No	2	-
386	HKB1017	No	2	-
387	HKB1020	No	2	-
388	HKB1024	No	1	-
389	HKB1025	No	2	-
390	HKB1026	No	1	-
391	HKB1027	No	1	-

No	Patient ID	Other substances	No of substances abused at a time	Reason for substance use initiation
392	HKB1035	No	2	-
393	HKB1036	No	3	Health, Joy seeking
394	HKB1038	No	1	-
395	HKB1042	No	1	Peer pressure
396	HKB1043	No	1	-
397	HKB1050	No	1	-
398	HKB1057	Yes	2	Peer pressure
399	HKB1059	No	1	-
400	HKB1061	No	1	-
401	HKB1062	No	1	-
402	HKB1064	No	3	-
403	HKB1069	No	2	-
404	HKB1070	No	1	-
405	HKB1071	No	2	-
406	HKB1072	No	2	Peer pressure
407	HKB1074	No	1	Others
408	HKB1079	No	1	-
409	HKB1080	No	3	Job problem
410	HKB1081	No	1	-
411	HKB1082	No	1	-
412	HKB1090	No	1	-
413	HKB1091	No	4	Health
414	HKB1092	No	3	Peer pressure
415	HKB1093	No	1	-
416	HKB1098	No	1	-
417	HKB1099	No	2	Relationship
418	HKB1100	No	1	Joy seeking
419	HKB1101	No	2	Joy seeking
420	HKB1102	No	1	-
421	HKJ0008	No	1	-
422	HKJ0013	No	1	-

No	Patient ID	Other substances	No of substances abused at a time	Reason for substance use initiation
423	HKJ0016	No	1	-
424	HKJ0024	No	1	-
425	HKJ0031	No	1	-
426	HKJ0035	No	1	-
427	HKJ0040	No	1	-
428	HKJ0041	No	1	-
429	HKJ0042	No	1	-
430	HKJ0044	No	1	-
431	HKJ0047	No	1	-
432	HKJ0049	No	1	Joy seeking
433	HKJ0050	No	4	-
434	HKJ0052	No	1	-
435	HKJ0062	No	1	-
436	HKJ0067	No	1	-
437	HKJ0069	No	1	-
438	HKJ0071	No	2	-
439	HKJ0073	No	2	-
440	HKJ0074	No	1	-
441	HKJ0076	Yes	1	-
442	HKJ0079	No	1	-
443	HKJ0080	No	1	-
444	HKJ0081	Yes	3	-
445	HKJ0082	No	1	Peer pressure
446	HKJ0083	No	2	-
447	HKJ0085	No	1	-
448	HKJ0087	No	1	-
449	HKJ0090	No	2	-
450	HKJ0091	No	1	-
451	HKJ0092	No	1	-
452	HKJ0093	No	2	-
453	HKJ0096	No	2	-
454	HKJ0097	No	1	-
455	HKJ0098	No	1	-
456	HKJ0103	No	1	-

No	Patient ID	Other substances	No of substances abused at a time	Reason for substance use initiation
457	HKJ0104	No	2	-
458	HKJ0106	No	2	-
459	HKJ0107	No	2	-
460	HKJ0108	No	1	-
461	HKJ0109	No	1	-
462	HKJ0111	No	1	-
463	HKJ0112	No	4	Others
464	HKJ0115	No	3	-
465	HKJ0116	No	1	-
466	HKJ0118	No	2	-
467	HKJ0127	No	1	-
468	HKJ0128	No	1	-
469	HKJ0129	No	2	-
470	HKJ0130	No	1	-
471	HKJ0131	No	1	-
472	HKJ0131	No	2	-
473	HKJ0134	No	2	-
474	HKJ0135	No	1	-
475	HKJ0138	No	2	-
476	HKJ0140	No	1	-
477	HKJ0141	No	1	-
478	HKJ0143	No	1	-
479	HKJ0144	No	1	-
480	HKJ0145	No	1	-
481	HKJ0146	No	2	-
482	HKJ0148	No	1	-
483	HKJ0151	No	2	-
484	HKJ0157	No	1	-
485	HKJ0158	No	1	-
486	HKJ0160	No	2	-
487	HKJ0161	No	1	-
488	HKJ0162	No	1	-
489	HKJ0165	No	2	-
490	HKJ0166	No	1	-
491	HKJ0170	No	1	-
492	HKJ0172	No	1	-

No	Patient ID	Other substances	No of substances abused at a time	Reason for substance use initiation
493	HKJ0175	No	1	-
494	HKJ0177	No	2	-
495	HKJ0178	No	1	-
496	HKJ0179	No	1	-
497	HKJ0182	No	1	-
498	HKJ0183	No	1	-
499	HKJ0184	No	1	-
500	HKJ0185	No	1	-
501	HKJ0187	No	3	-
502	HKJ0191	No	1	-
503	HKJ0192	No	1	-
504	HKJ0193	No	1	-
505	HKJ0195	No	2	-
506	HKJ0199	No	2	-
507	HKJ0203	No	1	-
508	HKJ0204	No	2	-
509	HKJ0206	No	1	-
510	HKJ0207	No	2	-
511	HKJ0208	No	2	-
512	HKJ0210	No	3	-
513	HKJ0212	No	1	-
514	HKJ0213	No	2	-
515	HKJ0214	No	1	-
516	HKJ0215	No	2	-
517	HKJ0216	No	2	-
518	HKJ0217	No	3	-
519	HKJ0218	No	1	-
520	HKJ0221	No	2	-
521	HKJ0224	No	1	-
522	HKJ0225	No	2	-
523	HKJ0227	No	2	-
524	HKJ0228	No	3	-
525	HKJ0229	No	3	-
526	HKJ0233	No	2	-
527	HKJ0236	No	2	-
528	HKJ0237	Yes	4	-
529	HKJ0238	No	1	-
530	HKJ0240	No	1	-

No	Patient ID	Other substances	No of substances abused at a time	Reason for substance use initiation
531	HKJ0241	No	3	-
532	HKJ0242	No	1	-
533	HKJ0243	Yes	3	-
534	HKJ0244	No	1	-
535	HKJ0245	No	2	-
536	HKJ0246	No	2	-
537	HKJ0248	No	2	-
538	HKJ0249	No	1	-
539	HKJ0251	No	1	-
540	HKJ0252	No	2	-
541	HKJ0253	No	1	-
542	HKJ0254	No	1	-
543	HKJ0255	No	1	-
544	HKJ0256	No	1	-
545	HKJ0257	No	1	-
546	HKJ0258	No	1	-
547	HKJ0260	No	1	-
548	HKJ0261	No	1	-
549	HKJ0264	No	1	-
550	HKJ0265	No	1	-
551	HKJ0266	No	1	-
552	HKJ0267	No	1	-
553	HKJ0268	No	1	-
554	HKJ0270	No	2	-
555	HKJ0271	No	1	-
556	HKJ0274	No	1	-
557	HKJ0277	No	1	-
558	HKJ0278	No	2	-
559	HKJ0280	No	1	-
560	HKJ0281	No	1	-
561	HKJ0283	No	1	-
562	HKJ0284	No	1	-
563	HKJ0287	No	1	-
564	HKJ0288	No	1	-
565	HKJ0289	No	3	-
566	HKJ0291	No	1	-
567	HKJ0293	Yes	4	-
568	HKJ0297	No	1	-

No	Patient ID	Other substances	No of substances abused at a time	Reason for substance use initiation
569	HKJ0298	No	1	-
570	HKJ0299	No	2	-
571	HKJ0300	No	3	-
572	HKJ0301	No	1	-
573	HKJ0303	No	1	-
574	HKJ0304	No	1	-
575	HKJ0305	No	1	-
576	HKJ0306	No	1	-
577	HKJ0308	Yes	2	-
578	HKJ0310	No	1	-
579	HKJ0311	No	1	-
580	HKJ0312	No	2	-
581	HKJ0314	No	3	-
582	HKJ0315	No	1	-
583	HKJ0316	No	1	-
584	HKJ0317	No	1	-
585	HKJ0318	No	4	-
586	HKJ0319	No	1	-
587	HKJ0320	No	2	-
588	HKJ0321	No	1	-
589	HKJ0322	No	1	-
590	HKJ0324	No	1	-
591	HKJ0326	No	1	-
592	HKJ0327	No	1	-
593	HKJ0328	No	3	-
594	HKJ0329	No	1	-
595	HKJ0330	No	1	-
596	HKJ0331	No	1	-
597	HKJ0332	No	1	-
598	HKJ0334	No	1	-
599	HKJ0335	No	1	-
600	HKJ0336	No	4	-
601	HKJ0337	No	1	-
602	HKJ0338	No	1	-
603	HKJ0339	No	3	-
604	HKJ0340	No	1	-
605	HKJ0342	No	1	-
606	HKJ0343	No	1	-
607	HKJ0345	No	5	-
608	HKJ0348	No	1	-
609	HKJ0349	No	1	-
610	HKJ0353	No	2	-
611	HKJ0354	No	1	-
612	HKJ0355	No	1	-

No	Patient ID	Other substances	No of substances abused at a time	Reason for substance use initiation
613	HKJ0358	No	1	-
614	HKJ0359	No	3	-
615	HKJ0360	No	1	-
616	HKJ0362	No	1	-
617	HKJ0364	No	1	-
618	HKJ0365	No	4	-
619	HKJ0366	No	1	-
620	HKJ0367	No	2	-
621	HKJ0368	No	1	-
622	HKJ0369	No	1	-
623	HKJ0372	No	1	-
624	HKJ0374	No	1	-
625	HKJ0375	No	1	-
626	HKJ0376	No	1	-
627	HKJ0378	No	2	-
628	HKJ0379	No	2	-
629	HKJ0381	No	1	-
630	HKJ0383	No	1	-
631	HKJ0384	No	1	-
632	HKJ0385	No	1	-
633	HKJ0387	No	1	-
634	HKJ0389	No	1	-
635	HKJ0391	No	1	-
636	HKJ0392	No	1	-
637	HKJ0393	No	1	-
638	HKJ0394	No	1	-
639	HKJ0395	No	2	-
640	HKJ0396	No	2	-
641	HKJ0397	No	2	-
642	HKJ0398	No	1	-
643	HKJ0399	No	2	-
644	HKJ0400	No	1	-
645	HKJ0401	No	1	-
646	HKJ0403	No	2	-
647	HKJ0404	No	2	-
648	HKJ0405	No	3	-
649	HKJ0407	No	2	-
650	HKJ0408	No	2	-
651	HKJ0409	No	1	-
652	HKJ0410	No	1	-
653	HKJ0411	No	1	-
654	HKJ0412	No	1	-

No	Patient ID	Other substances	No of substances abused at a time	Reason for substance use initiation
655	HKJ0413	No	1	-
656	HKJ0414	No	1	-
657	HKJ0415	No	2	-
658	HKJ0416	No	1	-
659	HKJ0417	No	4	-
660	HKJ0418	No	1	-
661	HKJ0419	No	2	Family issue, Relationship
662	HKJ0420	No	1	Peer pressure
663	HKJ0421	No	1	-
664	HKJ0422	No	1	-
665	HKJ0423	No	1	-
666	HKJ0424	No	2	-
667	HKJ0425	No	1	-
668	HKJ0426	No	1	-
669	HKJ0429	No	1	-
670	HKJ0429	No	1	-
671	HKJ0430	No	2	-
672	HKJ0431	No	1	-
673	HKJ0433	No	1	-
674	HKJ0434	No	1	-
675	HKJ0435	No	1	-
676	HKJ0436	No	1	-
677	HKJ0437	No	2	-
678	HKJ0438	No	1	-
679	HKJ0440	No	1	-
680	HKJ0441	No	1	-
681	HKJ0442	No	3	-
682	HKJ0443	No	1	-
683	HKJ0444	No	4	-
684	HKJ0445	No	1	-
685	HKJ0446	No	1	-
686	HKJ0447	No	1	-
687	HKJ0448	No	1	-
688	HKJ0449	No	2	-
689	HKJ0450	No	1	-
691	HKJ0452	No	4	-
690	HKJ0452	No	1	-
692	HKJ0453	No	1	-
693	HKJ0454	No	1	-
694	HKJ0455	No	1	-
695	HKJ0456	No	1	-

No	Patient ID	Other substances	No of substances abused at a time	Reason for substance use initiation
696	HKJ0457	No	2	-
697	HKJ0458	No	1	-
698	HKJ0459	No	1	-
699	HKJ0460	No	2	-
700	HKJ0461	No	1	-
702	HKJ0463	No	1	-
701	HKJ0463	No	1	-
703	HKJ0464	No	2	-
704	HKJ0466	No	2	-
705	HKJ0467	No	1	-
706	HKJ0468	No	1	-
707	HKJ0469	No	1	-
708	HKJ0470	No	1	-
709	HKJ0471	No	1	-
710	HKJ0472	No	1	-
711	HKJ0473	No	1	-
712	HKJ0474	No	1	-
713	HKJ0475	Yes	5	-
714	HKJ0476	No	1	-
715	HKJ0477	No	1	-
716	HKJ0478	No	2	-
717	HKJ0479	No	1	-
718	HKJ0480	No	2	-
719	HKJ0481	No	1	-
720	HKJ0482	No	1	-
721	HKJ0483	No	2	-
722	HKJ0484	No	1	-
723	HKJ0485	No	3	-
724	HKJ0486	No	1	-
725	HKJ0487	No	1	-
726	HKJ0488	No	1	-
727	HKJ0489	No	1	-
728	HKJ0490	No	1	-
729	HKJ0491	No	1	-
730	HKJ0492	No	1	-
731	HKJ0493	No	1	-
732	HKJ0494	No	1	-
733	HKJ0495	No	1	-
734	HKJ0496	No	1	-
735	HKJ0497	No	1	-
736	HKJ0498	No	1	-
737	HKJ0499	No	1	-

No	Patient ID	Other substances	No of substances abused at a time	Reason for substance use initiation
738	HKJ0500	No	1	-
739	HKJ0501	No	1	-
740	HKJ0502	No	1	-
741	HKJ0503	No	3	-
742	HKJ0504	No	1	-
743	HKJ0505	No	1	-
744	HKJ0506	No	2	-
745	HKJ0507	No	1	-
746	HKJ0508	No	1	-
747	HKJ0509	No	1	-
748	HKJ0510	No	1	-
749	HKJ0511	No	1	-
750	HKJ0512	No	1	-
751	HKJ0513	No	1	-
752	HKJ0516	No	1	-
753	HKJ0522	No	2	-
754	HKJ0523	No	2	-
755	HKJ0524	No	1	-
756	HKJ0525	No	1	-
757	HKJ0526	No	1	-
758	HKJ0527	No	1	-
759	HKJ0528	No	1	-
760	HKJ0529	No	1	-
761	HKJ0530	No	3	-
762	HKJ0531	No	2	-
763	HKJ0532	No	1	-
764	HKJ0533	No	1	-
765	HKJ0534	No	1	-
766	HKJ0535	No	1	-
767	HKJ0536	No	1	-
768	HKJ0537	No	1	-
769	HKJ0538	No	1	-
770	HKJ0539	No	1	-
771	HKJ0540	No	1	-
772	HKJ0541	No	1	-
773	HKJ0542	No	1	-
774	HKJ0554	No	1	-
775	HKJ0560	No	1	-
776	HKJ0562	No	1	-
777	HKJ0563	Yes	2	-
778	HKJ0564	No	1	-

No	Patient ID	Other substances	No of substances abused at a time	Reason for substance use initiation
779	HKJ0565	No	1	-
780	HKJ0566	No	1	-
781	HKJ0567	No	1	-
782	HKJ0568	Yes	3	-
783	HKJ0569	No	1	-
784	HKJ0570	No	1	-
785	HKJ0571	No	1	-
786	HKJ0572	No	1	-
787	HKJ0573	No	2	-
788	HKJ0574	No	1	-
789	HKJ0575	No	1	-
790	HKJ0576	No	1	-
791	HKJ0577	No	1	-
792	HKJ0578	No	3	-
793	HKJ0579	No	1	-
794	HKJ0580	No	1	-
795	HKJ0581	No	1	-
796	HKJ0585	No	1	-
797	HKJ0586	No	1	-
798	HKJ0587	No	1	-
799	HKJ0588	No	1	-
800	HKJ0589	No	1	-
801	HKJ0590	No	2	-
802	HKJ0591	No	1	-
803	HKJ0592	No	2	-
804	HKJ0593	No	1	-
805	HKJ0594	No	1	-
806	HKJ0603	No	1	-
807	HKJ0604	No	1	-
808	HKJ0605	No	1	-
809	HKJ0606	No	1	-
810	HKJ0607	No	1	-
811	HKJ0608	No	2	-
812	HKJ0609	No	1	-
813	HKJ0610	No	1	-
814	HKJ0611	No	1	-
815	HKJ0613	No	1	-
816	HKJ0614	No	1	-
817	HKJ0615	No	1	-
818	HKJ0616	Yes	4	-
819	HKJ0621	No	1	-
820	HKJ0622	No	1	-

No	Patient ID	Other substances	No of substances abused at a time	Reason for substance use initiation
821	HKJ0623	No	1	-
822	HKJ0624	No	1	-
823	HKJ0625	No	1	-
824	HKJ0626	No	2	-
825	HKJ0627	No	1	-
826	HKJ0628	No	1	-
827	HKJ0629	No	1	-
828	HKJ0632	No	1	-
829	HKJ0633	No	1	-
830	HKJ0634	No	1	-
831	HKJ0636	No	1	-
832	HKJ0637	No	1	-
833	HKJ0638	No	2	-
834	HKJ0639	No	1	-
835	HKJ0640	No	1	-
836	HKJ0641	No	2	-
837	HKJ0642	No	1	-
838	HKJ0643	No	1	-
839	HKJ0644	No	1	-
840	HKJ0645	No	1	-
841	HKJ0646	No	1	-
842	HKJ0647	No	1	-
843	HKJ0648	No	1	-
844	HKJ0649	No	1	-
845	HKJ0650	No	2	-
846	HKJ0673	No	1	-
847	HKJ0687	No	2	-
848	HKJ0688	No	1	-
849	HKJ0689	No	1	-
850	HKJ0690	No	1	-
851	HKJ0691	No	1	-
852	HKJ0694	No	1	-
853	HKJ0695	No	1	-
854	HKJ0696	No	1	-
855	HKJ0697	No	1	-
856	HKJ0698	No	2	-
857	HKJ0699	No	2	-
858	HKJ0700	No	2	-
859	HKJ0701	No	2	Relaxation
860	HKJ0702	No	1	-
861	HKJ0703	No	1	-

No	Patient ID	Other substances	No of substances abused at a time	Reason for substance use initiation
862	HKJ0704	No	1	-
863	HKJ0705	No	3	-
864	HKJ0706	No	1	-
865	HKJ0707	No	1	-
866	HKJ0708	No	1	-
867	HKJ0709	No	4	-
868	HKJ0710	No	1	-
869	HKJ0711	No	1	-
870	HKJ0712	No	2	-
871	HKJ0713	No	1	-
872	HKJ0714	No	2	-
873	HKJ0715	No	1	-
874	HKJ0719	No	1	-
875	HKJ0720	No	1	-
876	HKJ0721	No	2	-
877	HKJ0722	No	2	-
878	HKJ0723	No	3	-
879	HKJ0724	No	2	-
880	HKJ0725	No	3	-
881	HKJ0726	No	1	-
882	HKJ0727	No	1	-
883	HKJ0728	No	1	-
884	HKJ0729	No	1	-
885	HKJ0742	No	1	-
886	HKJ0743	No	2	-
887	HKJ0744	No	1	-
888	HKJ0745	No	1	-
889	HKJ0746	No	1	-
890	HKJ0747	No	1	-
891	HKJ0748	No	2	-
892	HKJ0749	No	1	-
893	HKJ0750	No	2	-
894	HKJ0751	No	3	-
895	HKJ0752	No	1	-
896	HKJ0753	No	2	-
897	HKJ0754	No	3	-
898	HKJ0755	No	1	-
899	HKJ0756	No	2	-
900	HKJ0758	No	1	-

No	Patient ID	Other substances	No of substances abused at a time	Reason for substance use initiation
901	HKJ0759	No	1	-
902	HKJ0760	No	1	-
903	HKJ0761	No	1	-
904	HKJ0762	No	1	-
905	HKJ0763	No	1	-
906	HKJ0764	No	1	-
907	HKJ0766	No	2	-
908	HKJ0767	No	1	-
909	HKJ0768	No	1	-
910	HKJ0770	No	1	-
911	HKJ0771	No	1	-
912	HKJ0772	No	2	-
913	HKJ0774	No	1	-
914	HKJ0775	No	1	-
915	HKJ0776	Yes	1	-
916	HKJ0777	No	1	-
917	HKJ0778	No	2	-
918	HKJ0779	No	2	-
919	HKJ0780	No	2	-
920	HKJ0782	No	1	-
921	HKJ0784	No	2	-
922	HKJ0785	No	1	-
923	HKJ0786	No	1	-
924	HKJ0787	No	1	-
925	HKJ0788	No	1	-
926	HKJ0789	No	2	-
927	HKJ0791	No	1	-
928	HKJ0792	No	2	-
929	HKJ0793	No	1	-
930	HKJ0794	No	2	-
931	HKJ0795	No	1	-
932	HKJ0796	No	1	-
933	HKJ0797	No	1	-
934	HKJ0798	No	1	-
935	HKJ0799	No	1	-
936	HKJ0800	No	2	-
937	HKJ0801	No	3	-
938	HKJ0802	No	2	-
939	HKJ0803	No	2	-

No	Patient ID	Other substances	No of substances abused at a time	Reason for substance use initiation
940	HKJ0804	No	1	-
941	HKJ0805	No	1	-
942	HKJ0806	No	1	-
943	HKJ0807	No	1	-
944	HKJ0808	Yes	3	-
945	HKJ0809	No	1	-
946	HKJ0810	Yes	1	-
947	HKJ0811	No	1	-
948	HKJ0812	No	1	-
949	HKJ0813	No	2	-
950	HKJ0814	No	1	-
951	HKJ0815	No	2	-
952	HKJ0816	No	2	-
953	HKJ0819	No	3	-
954	HKJ0820	No	1	-
955	HKJ0821	No	1	-
956	HKJ0822	No	2	-
957	HKJ0823	No	1	-
958	HKJ0824	No	2	-
959	HKJ0825	No	2	-
960	HKJ0826	No	1	-
961	HKJ0827	No	2	-
962	HKJ0828	Yes	3	-
963	HKJ0829	No	1	-
964	HKJ0830	No	1	-
965	HKJ0831	No	2	-
966	HKJ0832	No	1	-
967	HKJ0833	No	1	-
968	HKJ0834	No	2	-
969	HKJ0835	No	1	-
970	HKJ0836	No	1	-
971	HKJ0837	No	1	-
972	HKJ0838	No	2	-
973	HKJ0839	No	1	-
974	HKJ0840	No	2	-
975	HKL0004	No	1	Relaxation

No	Patient ID	Other substances	No of substances abused at a time	Reason for substance use initiation
976	HKL0009	No	1	Health
977	HKL0010	No	1	-
978	HKL0012	No	1	Health
979	HKL0017	No	2	Relaxation, Peer pressure, Others
980	HKL0030	No	3	Job problem, Peer pressure
981	HKL0049	No	3	-
982	HKL0055	No	1	-
983	HKL0056	No	2	-
984	HKL0058	No	2	Others
985	HKL0062	No	1	-
986	HKL0068	No	2	Job problem
987	HKL0081	No	2	Health, Relaxation
988	HKL0082	No	5	Peer pressure
989	HKL0084	No	2	Peer pressure
990	HKL0087	No	2	Job problem, Relaxation
991	HKL0105	No	3	-
992	HKL0108	No	4	Peer pressure, Others
993	HKL0112	Yes	10	Peer pressure
994	HKL0113	No	1	Peer pressure
995	HKL0121	No	1	-
996	HKL0144	No	3	Others
997	HKL0154	No	2	Family issue, Others
998	HKL0170	No	3	Peer pressure
999	HKL0173	No	1	Health
1000	HKL0175	Yes	3	Relaxation
1001	HKL0176	No	1	-
1002	HKL0186	No	2	Others
1003	HKL0187	No	1	-
1004	HKL0188	No	1	-

No	Patient ID	Other substances	No of substances abused at a time	Reason for substance use initiation
1005	HKL0189	No	2	Peer pressure
1006	HKL0191	No	5	-
1007	HKL0192	No	2	-
1008	HKL0193	No	3	Peer pressure
1009	HKL0194	No	2	-
1010	HKL0197	No	1	Peer pressure
1011	HKL0198	No	3	Peer pressure
1012	HKL0199	No	3	-
1013	HKL0203	No	3	Relaxation
1014	HKL0204	No	1	-
1015	HKL0209	No	4	-
1016	HKL0211	No	2	-
1017	HKL0214	No	4	Relaxation
1018	HKL0215	No	3	Joy seeking
1019	HKL0217	No	1	Peer pressure
1020	HKL0218	No	1	-
1021	HKL0219	No	2	Others
1022	HKL0222	No	2	Peer pressure
1023	HKL0224	No	2	-
1024	HKL0225	No	1	Peer pressure
1025	HKL0226	No	2	-
1026	HKL0227	No	3	-
1027	HKL0228	No	1	-
1028	HKL0229	No	3	-
1029	HKL0230	No	2	-
1030	HKL0231	No	2	-
1031	HKL0238	No	3	-
1032	HKL0240	No	1	Peer pressure
1033	HKL0241	No	2	-
1034	HKL0242	No	2	Others
1035	HKL0243	No	1	-
1036	HKL0244	No	1	-
1037	HKL0246	No	1	Peer pressure
1038	HKL0247	No	2	-

No	Patient ID	Other substances	No of substances abused at a time	Reason for substance use initiation
1039	HKL0248	No	1	-
1040	HKL0252	No	1	Joy seeking
1041	HKL0253	No	1	Relaxation
1042	HKL0254	No	3	Relaxation
1043	HKL0256	No	2	-
1044	HKL0257	No	3	-
1045	HKL0259	No	1	-
1046	HKL0262	No	2	Peer pressure
1047	HKL0267	No	1	Health
1048	HKL0268	No	3	Relaxation, Others
1049	HKL0272	No	1	-
1050	HKL0273	No	1	Peer pressure, Others
1051	HKL0275	No	1	Family issue
1052	HKL0276	No	3	-
1053	HKL0277	No	2	Peer pressure
1054	HKL0278	No	3	Peer pressure
1055	HKL0279	No	3	Others
1056	HKL0280	No	1	-
1057	HKL0282	No	1	-
1058	HKL0284	No	1	Peer pressure
1059	HKL0285	No	2	-
1060	HKL0286	No	1	Relationship
1061	HKL0287	No	2	-
1062	HKL0288	No	2	-
1063	HKL0289	No	2	Peer pressure
1064	HKL0290	Yes	5	Relaxation
1065	HKL0291	No	2	-
1066	HKL0292	No	1	Others
1067	HKL0293	No	2	Job problem, Others
1068	HKL0294	No	3	Joy seeking
1069	HKL0295	No	1	Relationship, Others
1070	HKL0296	No	1	-

No	Patient ID	Other substances	No of substances abused at a time	Reason for substance use initiation
1071	HKL0300	No	2	Job problem, Relaxation, Peer pressure
1072	HKL0301	Yes	2	Relationship
1073	HKL0302	No	2	Job problem
1074	HKL0319	No	2	Peer pressure
1075	HKL0320	No	3	Others
1076	HKL0321	No	1	-
1077	HKL0333	No	1	-
1078	HKL0334	No	2	Peer pressure
1079	HKL0335	No	1	Job problem
1080	HKL0338	No	1	Peer pressure
1081	HKL0339	No	1	Family issue, Relaxation, Peer pressure
1082	HKL0340	Yes	3	Others
1083	HKL0342	No	1	-
1084	HKL0349	No	2	-
1085	HKL0351	No	2	-
1086	HKL0353	No	1	-
1087	HKL0355	No	1	-
1088	HKL0366	No	1	-
1089	HKL0367	No	3	-
1090	HKL0368	No	2	Peer pressure
1091	HKL0370	No	3	-
1092	HKL0383	No	3	Peer pressure
1093	HKL0386	No	1	Peer pressure
1094	HKL0387	No	1	Others
1095	HKL0388	No	1	-
1096	HKL0390	No	3	Others
1097	HKL0391	No	2	Others
1098	HKL0392	No	1	Health

No	Patient ID	Other substances	No of substances abused at a time	Reason for substance use initiation
1099	HKL0393	No	1	Peer pressure
1100	HKL0402	No	1	-
1101	HKL0404	No	1	Peer pressure
1102	HKL0406	No	4	-
1103	HKL0407	No	2	Others
1104	HKL0410	No	3	Relaxation, Others
1105	HKL0424	No	1	Health
1106	HKL0427	No	2	Peer pressure
1107	HKL0428	No	2	-
1108	HKL0430	No	3	-
1109	HKL0433	No	1	-
1110	HKL0435	No	1	-
1111	HKL0436	No	1	-
1112	HKL0437	No	1	-
1113	HKL0446	No	2	Job problem, Relationship
1114	HKL0448	No	1	Others
1115	HKL0450	No	1	-
1116	HKL0452	No	1	-
1117	HKL0453	No	2	-
1118	HKL0455	No	1	-
1119	HKL0468	No	2	Peer pressure
1120	HKL0469	No	1	Others
1121	HKL0472	No	1	-
1122	HKL0475	No	1	-
1123	HKL0486	No	4	Peer pressure
1124	HKL0487	No	2	-
1125	HKL0488	No	2	Peer pressure, Others
1126	HKL0489	No	1	-
1127	HKL0490	No	2	-
1128	HKL0491	No	1	Others
1129	HKL0493	No	1	-

No	Patient ID	Other substances	No of substances abused at a time	Reason for substance use initiation
1130	HKL0494	No	2	-
1131	HKL0496	Yes	2	-
1132	HKL0497	No	3	-
1133	HKL0498	No	1	Joy seeking
1134	HKL0501	No	2	Peer pressure
1135	HKL0502	No	1	-
1136	HKL0503	No	3	Relaxation
1137	HKL0505	No	1	-
1138	HKL0507	No	1	-
1139	HKL0509	No	1	Job problem, Relationship
1140	HKL0511	No	3	-
1141	HKL0513	No	1	-
1142	HKL0514	No	3	-
1143	HKL0515	No	2	-
1144	HKL0516	No	3	-
1145	HKL0518	No	2	-
1146	HKL0521	No	2	-
1147	HKL0522	No	1	-
1148	HKL0525	No	1	-
1149	HKL0527	No	1	-
1150	HKL0528	No	1	-
1151	HKL0529	No	1	-
1152	HKL0530	No	3	-
1153	HKL0533	No	2	-
1154	HKL0534	No	1	-
1155	HKL0535	No	1	Others
1156	HKL0536	No	4	-
1157	HKL0540	No	2	-
1158	HKL0541	No	1	Relaxation, Others
1159	HKL0542	No	2	-
1160	HKL0543	No	2	Joy seeking, Peer pressure

No	Patient ID	Other substances	No of substances abused at a time	Reason for substance use initiation
1161	HKL0545	No	1	-
1162	HKL0546	No	3	Others
1163	HKL0548	No	4	Job problem, Relaxation
1164	HKL0550	No	1	-
1165	HKL0552	No	2	-
1166	HKL0553	No	2	-
1167	HKL0554	No	1	-
1168	HKL0555	No	2	-
1169	HKL0556	No	1	-
1170	HKL0559	No	1	-
1171	HKL0560	No	1	-
1172	HKL0561	No	1	-
1173	HKL0562	No	1	Peer pressure
1174	HKL0563	No	2	Peer pressure
1175	HKL0564	No	2	Peer pressure
1176	HKL0565	No	1	-
1177	HKL0566	No	2	Peer pressure
1178	HKL0567	No	1	-
1179	HKL0568	No	1	-
1180	HKL0569	No	2	-
1181	HKL0573	No	1	Others
1182	HKL0576	No	2	-
1183	HKL0584	No	1	-
1184	HKL0588	No	2	-
1185	HKL0590	No	2	-
1186	HKL0593	No	2	-
1187	HKL0594	No	4	-
1188	HKL0595	No	3	-

No	Patient ID	Other substances	No of substances abused at a time	Reason for substance use initiation
1189	HKL0596	No	2	Relaxation
1190	HKL0597	No	3	-
1191	HKL0599	No	4	-
1192	HKL0600	No	1	-
1193	HKL0601	No	1	-
1194	HKL0602	No	1	-
1195	HKL0605	No	1	-
1196	HKL0607	No	2	Peer pressure
1197	HKL0609	No	2	-
1198	HKL0611	No	2	-
1199	HKL0613	No	4	Family issue, Others
1200	HKL0614	No	1	-
1201	HKL0616	No	2	-
1202	HKL0618	No	2	-
1203	HKL0619	No	3	-
1204	HKL0622	No	3	-
1205	HKL0625	No	1	-
1206	HKL0629	No	4	-
1207	HKL0630	No	1	-
1208	HKL0631	No	2	-
1209	HKL0632	No	2	Family issue
1210	HKL0634	No	1	-
1211	HKL0636	No	1	Others
1212	HKL0637	No	1	Health
1213	HKL0638	No	1	Health
1214	HKL0642	No	1	-
1215	HKL0643	No	1	-

No	Patient ID	Other substances	No of substances abused at a time	Reason for substance use initiation
1216	HKL0646	No	2	-
1217	HKL0647	No	1	-
1218	HKL0649	No	3	-
1219	HKL0653	No	2	-
1220	HKL0656	No	2	-
1221	HKL0657	No	4	-
1222	HKL0658	No	3	-
1223	HKL0659	No	3	-
1224	HKL0660	No	2	-
1225	HKL0661	No	1	-
1226	HKL0663	No	1	-
1227	HKL0664	No	1	-
1228	HKL0666	No	1	-
1229	HKL0669	No	4	-
1230	HKL0670	No	2	-
1231	HKL0671	No	2	-
1232	HKL0672	No	5	-
1233	HKL0675	No	3	-
1234	HKL0677	No	2	-
1235	HKL0678	No	1	-
1236	HKL0680	No	2	-
1237	HKL0681	No	2	-
1238	HKL0682	No	1	-
1239	HKL0683	No	2	-
1240	HKL0685	No	3	-
1241	HKL0687	No	2	-
1242	HKL0688	No	1	-
1243	HKL0690	No	1	-
1244	HKL0693	No	2	Peer pressure
1245	HKL0696	No	1	-
1246	HKL0697	No	2	-

No	Patient ID	Other substances	No of substances abused at a time	Reason for substance use initiation
1247	HKL0698	No	3	Peer pressure
1248	HKL0699	Yes	4	-
1249	HKL0700	No	2	-
1250	HKL0702	No	3	-
1251	HKL0707	No	2	-
1252	HKL0708	No	3	-
1253	HKL0709	No	1	-
1254	HKL0710	No	2	-
1255	HKL0711	No	2	-
1256	HKL0712	No	1	-
1257	HKL0715	No	1	-
1258	HKL0716	No	3	-
1259	HKL0717	No	2	-
1260	HKL0719	No	2	-
1261	HKL0720	No	1	-
1262	HKL0721	No	2	-
1263	HKL0723	No	1	-
1264	HKL0725	No	1	-
1265	HKL0726	No	3	-
1266	HKL0727	No	1	-
1267	HKL0728	No	2	-
1268	HKL0729	No	2	Peer pressure
1269	HKL0731	No	1	-
1270	HKL0732	No	1	Job problem
1271	HKL0736	No	1	-
1272	HKL0742	No	2	Job problem, Joy seeking
1273	HKL0746	No	3	Family issue
1274	HKL0747	No	2	-
1275	HKL0748	No	1	-

No	Patient ID	Other substances	No of substances abused at a time	Reason for substance use initiation
1276	HKL0750	No	1	-
1277	HKL0751	No	1	-
1278	HKL0755	No	2	-
1279	HKL0759	No	1	-
1280	HKL0761	No	2	-
1281	HKL0762	No	1	-
1282	HKL0763	No	2	-
1283	HKL0764	No	2	-
1284	HKL0766	No	1	-
1285	HKL0768	No	1	-
1286	HKL0769	No	1	-
1287	HKL0770	No	3	-
1288	HKL0771	Yes	4	-
1289	HKL0774	No	1	-
1290	HKL0776	No	3	-
1291	HKL0777	No	1	-
1292	HKL0778	No	2	-
1293	HKL0779	No	2	-
1294	HKL0781	No	2	-
1295	HKL0783	No	2	-
1296	HKL0784	No	1	-
1297	HKL0786	No	2	-
1298	HKL0794	No	1	-
1299	HKL0795	No	3	-
1300	HKL0797	No	2	-
1301	HKL0801	No	2	-
1302	HKL0806	No	1	Peer pressure
1303	HKL0807	No	2	-
1304	HKL0811	No	1	-
1305	HKL0816	No	1	-
1306	HKL0817	No	2	-
1307	HKL0820	No	3	Peer pressure
1308	HKL0829	No	2	-
1309	HKL0835	No	2	-

No	Patient ID	Other substances	No of substances abused at a time	Reason for substance use initiation
1310	HKL0840	No	2	-
1311	HKL0849	No	3	-
1312	HKL0853	No	2	-
1313	HKL0854	No	1	-
1314	HKL0855	No	2	-
1315	HKL0859	No	1	-
1316	HKL0861	No	2	-
1317	HKL0865	No	3	-
1318	HKL0874	No	1	-
1319	HKL0877	No	1	-
1320	HKL0879	No	2	-
1321	HKL0881	No	4	-
1322	HKL0882	No	2	-
1323	HKL0884	No	2	-
1324	HKL0886	No	1	-
1325	HKL0891	No	3	-
1326	HKL0896	No	2	-
1327	HKL0900	No	2	-
1328	HKL0903	No	2	-
1329	HKL0904	No	2	-
1330	HKL0907	No	2	-
1331	HKL0914	No	1	-
1332	HKL0915	No	1	-
1333	HKL0916	No	3	-
1334	HKL0919	No	1	-
1335	HKL0922	No	2	-
1336	HKL0933	No	2	-
1337	HKL0934	No	1	-
1338	HKL0936	No	3	-
1339	HKL0940	No	1	-

No	Patient ID	Other substances	No of substances abused at a time	Reason for substance use initiation
1340	HKL0945	No	1	-
1341	HKL0958	No	2	-
1342	HKL0959	No	3	-
1343	HKL0960	No	2	-
1344	HKL0961	No	2	-
1345	HKL0962	No	3	-
1346	HKL0964	No	1	-
1347	HKL0965	No	1	-
1348	HKL0969	No	2	-
1349	HKL0970	No	2	-
1350	HKL0977	No	1	-
1351	HKL0980	No	2	-
1352	HKL0981	No	2	-
1353	HKL0982	No	4	-
1354	HKL0983	No	1	-
1355	HKL0986	No	4	-
1356	HKL0988	No	2	Relaxation
1357	HKL0995	No	3	-
1358	HKL1003	No	1	-
1359	HKL1004	No	2	-
1360	HKL1017	No	2	-
1361	HKL1018	No	3	-
1362	HKL1023	Yes	6	-
1363	HKL1024	No	3	-
1364	HKL1025	No	1	-
1365	HKL1026	No	2	-
1366	HKL1027	No	2	-

No	Patient ID	Other substances	No of substances abused at a time	Reason for substance use initiation
1367	HKL1028	No	2	-
1368	HKL1030	No	2	-
1369	HKL1031	No	2	-
1370	HKL1032	No	1	-
1371	HKL1033	No	3	-
1372	HKL1034	No	2	-
1373	HKL1035	No	2	-
1374	HKL1037	No	1	Relaxation, Peer pressure
1375	HKL1039	No	2	-
1376	HKL1042	Yes	5	-
1377	HKL1043	No	3	-
1378	HKL1045	No	3	-
1379	HKL1047	No	1	-
1380	HKL1052	No	3	-
1381	HKL1053	No	3	-
1382	HKL1055	No	2	-
1383	HKL1057	No	3	-
1384	HKL1063	No	2	-
1385	HKL1066	No	2	-
1386	HKL1067	No	2	-
1387	HKL1068	No	1	-
1388	HKL1069	No	4	-
1389	HKL1071	No	2	-
1390	HKL1073	No	2	-
1391	HKL1076	No	2	-
1392	HKL1077	No	3	-
1393	HKL1078	No	2	-
1394	HKL1079	No	3	-
1395	HKL1080	No	2	-

No	Patient ID	Other substances	No of substances abused at a time	Reason for substance use initiation
1396	HKL1084	No	4	-
1397	HKL1088	No	1	-
1398	HKL1094	No	2	-
1399	HKL1095	No	3	Peer pressure
1400	HKL1096	No	3	-
1401	HKL1097	No	2	-
1402	HKL1098	No	2	Joy seeking
1403	HKL1100	No	1	-
1404	HKL1101	No	1	-
1405	HKL1104	No	2	-
1406	HKL1105	No	1	-
1407	HKL1107	No	1	-
1408	HKL1108	No	1	Others
1409	HKL1109	No	3	-
1410	HKL1115	No	1	-
1411	HKL1116	No	1	Family issue
1412	HKL1117	No	2	-
1413	HKL1118	No	3	Relaxation
1414	HKL1121	No	2	-
1415	HKL1122	No	1	-
1416	HKL1123	No	2	-
1417	HKL1135	No	2	-
1418	HM0001	No	1	-
1419	HM0002	No	1	-
1420	HM0003	No	3	-
1421	HM0004	No	1	-
1422	HM0005	No	1	Others
1423	HM0006	No	1	-
1424	HM0008	No	7	Relaxation, Joy seeking, Peer pressure
1425	HM0009	No	1	-
1426	HM0010	No	1	Others
1427	HM0011	No	1	-
1428	HM0012	No	2	Peer pressure

No	Patient ID	Other substances	No of substances abused at a time	Reason for substance use initiation
1429	HM0014	No	1	-
1430	HM0015	No	4	-
1431	HM0016	No	1	-
1432	HM0017	No	1	-
1433	HM0019	No	1	-
1434	HM0020	No	1	-
1435	HM0021	No	4	Relaxation, Peer pressure
1436	HM0022	No	3	Peer pressure
1437	HM0023	No	2	-
1438	HM0024	No	1	-
1439	HM0025	No	2	Relaxation, Joy seeking, Others
1440	HM0026	No	2	-
1441	HM0027	No	1	-
1442	HM0028	No	1	-
1443	HM0029	No	1	-
1444	HM0030	No	1	-
1445	HM0031	No	1	-
1446	HM0032	No	2	Relationship, Others
1447	HM0033	No	1	-
1448	HM0034	No	4	Health, Joy seeking
1449	HM0035	No	1	-
1450	HM0036	No	1	Joy seeking, Peer pressure
1451	HM0037	No	2	-
1452	HM0038	No	1	-
1453	HM0039	No	1	-
1454	HM0040	No	1	-
1455	HM0041	Yes	3	-
1456	HM0042	No	1	-
1457	HM0043	No	2	Relaxation
1458	HM0044	No	2	-
1459	HM0045	No	2	-
1460	HM0046	No	2	Relaxation, Joy seeking, Peer pressure
1461	HM0047	No	1	-
1462	HM0048	No	2	-
1463	HM0049	No	1	Others
1464	HM0050	No	2	-
1465	HM0051	No	1	Relaxation, Joy seeking, Peer pressure
1466	HM0052	No	2	-

No	Patient ID	Other substances	No of substances abused at a time	Reason for substance use initiation
1467	HM0053	Yes	4	-
1468	HM0054	No	1	Peer pressure
1469	HM0055	No	1	-
1470	HM0056	No	1	-
1471	HM0057	No	4	-
1472	HM0058	No	2	Joy seeking
1473	HM0059	No	1	-
1474	HM0060	No	2	-
1475	HM0061	No	2	-
1476	HM0062	No	3	Relaxation, Joy seeking
1477	HM0063	No	1	-
1478	HM0065	No	1	-
1479	HM0066	No	4	Peer pressure
1480	HM0067	No	2	-
1481	HM0068	No	1	Peer pressure
1482	HM0069	No	2	-
1483	HM0070	No	2	-
1484	HM0071	No	2	-
1485	HM0072	No	3	-
1486	HM0073	No	1	-
1487	HM0074	No	1	-
1488	HM0075	No	1	-
1489	HM0076	No	2	-
1490	HM0077	No	2	-
1491	HM0078	No	1	-
1492	HM0079	No	2	-
1493	HM0080	No	1	-
1494	HM0081	No	1	-
1495	HM0084	No	1	-
1496	HM0085	No	1	-
1497	HM0087	No	2	-
1498	HM0088	No	2	-
1499	HM0090	No	2	-
1500	HM0091	No	1	-
1501	HM0092	No	1	-
1502	HM0094	No	2	-
1503	HM0095	No	1	-
1504	HM0096	No	4	-
1505	HM0097	No	1	-
1506	HM0098	No	1	-

No	Patient ID	Other substances	No of substances abused at a time	Reason for substance use initiation
1507	HM0099	No	1	-
1508	HM0100	No	2	-
1509	HM0101	No	2	-
1510	HM0102	No	1	-
1511	HM0103	No	1	-
1512	HM0105	No	1	-
1513	HM0107	Yes	2	-
1514	HM0108	No	1	-
1515	HM0109	No	2	-
1516	HM0111	No	1	Relationship
1517	HM0112	No	1	-
1518	HM0113	No	1	-
1519	HM0114	No	1	-
1520	HM0115	No	2	-
1521	HM0116	No	1	-
1522	HM0117	No	1	-
1523	HM0119	No	2	-
1524	HM0120	No	1	-
1525	HM0121	No	1	-
1526	HM0122	No	2	Relaxation
1527	HM0123	No	1	-
1528	HM0124	No	3	-
1529	HM0125	No	1	-
1530	HM0128	No	1	-
1531	HM0129	No	1	-
1532	HM0130	No	1	-
1533	HM0133	No	2	Relaxation
1534	HM0135	No	1	-
1535	HM0138	No	3	Job problem
1536	HM0139	No	3	-
1537	HM0140	No	1	-
1538	HM0141	No	3	-
1539	HM0142	No	1	-
1540	HM0143	No	1	Others
1541	HM0145	No	1	-

No	Patient ID	Other substances	No of substances abused at a time	Reason for substance use initiation
1542	HM0146	No	1	-
1543	HM0147	No	1	Joy seeking
1544	HM0148	No	1	-
1545	HM0153	No	1	-
1546	HM0154	No	2	-
1547	HM0156	No	1	-
1548	HM0159	No	1	-
1549	HM0160	No	1	Peer pressure
1550	HM0162	No	2	Health
1551	HM0163	No	2	-
1552	HM0164	No	4	-
1553	HM0165	No	3	-
1554	HM0167	No	1	-
1555	HM0168	No	3	-
1556	HM0170	No	1	-
1557	HM0171	No	1	-
1558	HM0172	No	1	-
1559	HM0173	No	1	-
1560	HM0174	No	2	-
1561	HM0175	No	1	-
1562	HM0177	No	1	-
1563	HM0178	No	1	-
1564	HM0179	No	2	-
1565	HM0180	No	1	-
1566	HM0182	No	1	-
1567	HM0183	No	2	-
1568	HM0184	No	2	-
1569	HM0185	No	2	-
1570	HM0186	No	2	-
1571	HM0187	No	1	-
1572	HM0188	No	1	-
1573	HM0189	No	2	-
1574	HM0190	No	1	-
1575	HM0191	No	1	-
1576	HM0192	No	4	-
1577	HM0193	No	1	-
1578	HM0197	No	1	-
1579	HM0198	Yes	3	-
1580	HM0199	No	1	-

No	Patient ID	Other substances	No of substances abused at a time	Reason for substance use initiation
1581	HM0204	No	3	-
1582	HM0205	No	1	-
1583	HM0206	No	1	-
1584	HM0208	No	2	-
1585	HM0209	No	1	-
1586	HM0211	No	6	-
1587	HM0212	No	1	Peer pressure
1588	HM0213	No	2	-
1589	HM0214	No	1	-
1590	HM0215	No	1	-
1591	HM0217	No	1	-
1592	HM0218	No	3	-
1593	HM0219	No	2	-
1594	HM0221	No	2	-
1595	HM0222	No	1	-
1596	HM0222	No	1	-
1597	HM0223	No	1	Health
1598	HM0225	No	1	-
1599	HM0226	No	2	Relaxation
1600	HM0227	No	1	-
1601	HM0230	No	1	Relaxation
1602	HM0234	No	3	-
1603	HM0236	No	2	-
1604	HM0237	No	1	-
1605	HM0238	No	4	-
1606	HM0241	No	2	-
1607	HM0242	No	1	-
1608	HM0243	No	1	-
1609	HM0245	No	4	-
1610	HM0247	No	1	-
1611	HM0248	Yes	5	-
1612	HM0249	No	1	Others
1613	HM0250	No	1	-
1614	HM0251	No	1	-
1615	HM0252	No	4	-
1616	HM0253	Yes	3	-

No	Patient ID	Other substances	No of substances abused at a time	Reason for substance use initiation
1617	HM0254	No	3	-
1618	HM0255	No	1	-
1619	HM0257	No	1	-
1620	HM0258	No	2	-
1621	HM0260	No	1	-
1622	HM0261	No	2	-
1623	HM0263	No	2	-
1624	HM0265	No	1	-
1625	HM0266	No	1	-
1626	HM0269	No	2	-
1627	HM0270	No	1	-
1628	HM0271	No	3	-
1629	HM0272	No	1	-
1630	HM0273	No	1	-
1631	HM0274	No	1	-
1632	HM0275	No	2	Peer pressure
1633	HM0276	No	1	Others
1634	HM0277	No	1	Relaxation
1635	HM0278	No	1	Joy seeking
1636	HM0279	No	1	-
1637	HM0280	No	3	-
1638	HM0281	Yes	3	-
1639	HM0282	No	1	-
1640	HM0283	No	3	-
1641	HM0284	No	1	-
1642	HM0285	No	2	-
1643	HM0286	No	2	Health
1644	HM0287	No	2	-
1645	HM0288	No	1	-
1646	HM0289	No	1	-
1647	HM0290	No	1	-
1648	HM0292	No	2	-
1649	HM0293	No	1	Joy seeking, Peer pressure
1650	HM0296	No	3	-
1651	HM0297	No	2	-
1652	HM0298	No	1	-
1653	HM0299	No	1	-
1654	HM0300	No	2	-
1655	HM0301	No	1	-
1656	HM0302	No	2	-

No	Patient ID	Other substances	No of substances abused at a time	Reason for substance use initiation
1657	HM0303	No	2	-
1658	HM0304	No	3	Health, Job problem, Relationship
1659	HM0305	No	1	-
1660	HM0306	No	1	-
1661	HM0308	No	2	-
1662	HM0309	No	3	-
1663	HM0310	No	1	Health
1664	HM0312	No	1	-
1665	HM0313	No	1	-
1666	HM0315	No	3	-
1667	HM0317	No	3	Relaxation
1668	HM0319	No	2	-
1669	HM0320	No	1	Relaxation, Joy seeking
1670	HM0321	No	1	-
1671	HM0323	No	1	-
1672	HM0324	No	1	-
1673	HM0325	No	2	-
1674	HM0327	No	1	Family issue, Relaxation, Joy seeking, Peer pressure
1675	HM0328	No	1	-
1676	HM0329	No	1	-
1677	HM0330	No	4	Family issue, Peer pressure
1678	HM0332	No	1	Family issue, Relaxation
1679	HM0333	No	1	Health
1680	HM0334	No	1	Relaxation, Joy seeking
1681	HM0335	No	1	-
1682	HM0336	No	1	Job problem, Relationship, Relaxation, Joy seeking, Peer pressure
1683	HM0338	No	2	-
1684	HM0339	No	2	-
1685	HM0340	No	1	-
1686	HM0340	No	2	-

No	Patient ID	Other substances	No of substances abused at a time	Reason for substance use initiation
1687	HM0342	No	1	-
1688	HM0344	No	1	-
1689	HM0345	No	1	-
1690	HM0346	No	2	Relaxation, Joy seeking
1691	HM0347	No	1	Relaxation, Joy seeking
1692	HM0348	No	2	-
1693	HM0350	No	1	-
1694	HM0351	No	1	Relaxation, Joy seeking
1695	HM0353	No	2	-
1696	HM0354	No	1	-
1697	HM0355	No	2	Relaxation, Joy seeking, Peer pressure
1698	HM0356	No	1	Job problem
1699	HM0357	No	2	-
1700	HM0360	No	1	-
1701	HM0361	No	2	-
1702	HM0363	No	2	-
1703	HM0364	No	4	-
1704	HM0366	Yes	5	-
1705	HM0368	No	1	-
1706	HM0369	No	2	-
1707	HM0370	No	1	-
1708	HM0371	No	1	-
1709	HM0372	No	1	-
1710	HM0373	No	1	-
1711	HM0374	No	2	-
1712	HM0375	No	1	-
1713	HM0377	No	1	-
1714	HM0381	No	2	-
1715	HM0382	No	2	-
1716	HM0384	No	1	-
1717	HM0385	No	1	-
1718	HM0386	No	4	-
1719	HM0387	Yes	2	-
1720	HM0388	No	3	-
1721	HM0391	No	2	-
1722	HM0392	No	2	-
1723	HM0393	No	2	-
1724	HM0394	No	5	-

No	Patient ID	Other substances	No of substances abused at a time	Reason for substance use initiation
1725	HM0395	No	3	-
1726	HM0396	No	2	-
1727	HM0397	No	2	-
1728	HM0398	No	1	Others
1729	HM0399	Yes	2	-
1730	HM0400	No	1	Health
1731	HM0401	No	2	-
1732	HM0402	No	3	-
1733	HM0406	No	2	-
1734	HM0407	No	2	-
1735	HM0408	No	3	-
1736	HM0409	No	2	Joy seeking
1737	HM0410	No	1	-
1738	HM0411	No	2	-
1739	HM0412	No	3	-
1740	HM0413	No	2	-
1741	HM0414	No	1	-
1742	HM0415	No	4	-
1743	HM0416	Yes	3	-
1744	HM0417	No	2	-
1745	HM0418	No	2	-
1746	HM0419	Yes	4	-
1747	HM0423	No	2	-
1748	HM0424	No	2	-
1749	HM0425	No	1	-
1750	HM0426	No	3	-
1751	HM0427	No	2	-
1752	HM0428	No	2	-
1753	HM0429	No	2	Relaxation, Joy seeking
1754	HM0430	No	3	-
1755	HM0431	No	2	-
1756	HM0432	No	2	-
1757	HM0433	No	2	-
1758	HM0434	No	5	-

No	Patient ID	Other substances	No of substances abused at a time	Reason for substance use initiation
1759	HM0437	No	1	Family issue, Relaxation, Peer pressure
1760	HM0439	No	2	-
1761	HM0441	Yes	3	-
1762	HM0442	No	1	-
1763	HM0443	No	2	-
1764	HM0445	No	2	-
1765	HM0447	No	2	-
1766	HM0448	No	1	-
1767	HM0449	No	3	-
1768	HM0452	No	1	-
1769	HM0453	No	2	-
1770	HM0455	No	3	Peer pressure
1771	HM0456	No	1	-
1772	HM0458	No	1	-
1773	HM0461	No	3	-
1774	HM0462	No	1	-
1775	HM0463	No	1	-
1776	HM0464	No	1	-
1777	HM0465	No	3	-
1778	HM0468	No	3	-
1779	HM0469	No	2	Peer pressure
1780	HM0470	No	1	-
1781	HM0471	No	3	-
1782	HM0472	No	1	-
1783	HM0473	No	1	-
1784	HM0475	No	2	Peer pressure
1785	HM0476	No	1	-
1786	HM0477	No	1	-
1787	HM0478	No	1	Family issue, Relaxation, Peer pressure
1788	HM0479	No	2	-
1789	HM0480	No	3	-
1790	HM0481	No	1	-
1791	HM0482	No	1	Family issue
1792	HM0483	No	4	-
1793	HM0484	No	1	-
1794	HM0485	No	2	-
1795	HM0486	No	1	Relaxation, Joy seeking
1796	HM0488	No	1	-

No	Patient ID	Other substances	No of substances abused at a time	Reason for substance use initiation
1797	HM0489	No	2	Relaxation
1798	HM0490	No	2	-
1799	HM0492	No	3	-
1800	HM0493	No	2	-
1801	HM0494	No	1	-
1802	HM0495	No	1	Relaxation, Joy seeking
1803	HM0496	No	1	Relaxation, Joy seeking
1804	HM0497	No	1	Family issue, Relaxation, Joy seeking, Peer pressure
1805	HM0499	No	1	Relaxation, Joy seeking
1806	HM0500	No	1	Relaxation, Joy seeking
1807	HM0501	No	3	Relaxation, Joy seeking
1808	HM0502	No	3	-
1809	HM0503	No	1	-
1810	HM0504	No	2	Relaxation, Joy seeking
1811	HM0505	No	1	-
1812	HM0506	No	2	-
1813	HM0507	No	2	-
1814	HM0508	No	2	-
1815	HM0510	No	2	-
1816	HM0511	No	1	-
1817	HM0514	No	1	Joy seeking, Peer pressure
1818	HM0515	No	2	Relaxation, Joy seeking
1819	HM0517	No	1	-
1820	HM0519	No	2	-
1821	HM0520	No	1	-
1822	HM0521	No	2	-
1823	HM0522	No	1	-
1824	HM0523	No	1	-
1825	HM0524	No	1	-
1826	HM0525	No	2	Relaxation, Joy seeking, Peer pressure
1827	HM0526	No	1	-
1828	HM0528	No	2	-
1829	HM0529	No	1	-

No	Patient ID	Other substances	No of substances abused at a time	Reason for substance use initiation
1830	HM0530	No	1	-
1831	HM0531	No	1	-
1832	HM0532	No	1	-
1833	HM0534	No	2	-
1834	HM0535	No	3	-
1835	HM0536	No	2	-
1836	HM0537	No	1	Relaxation, Joy seeking
1837	HM0538	No	1	Others
1838	HM0539	No	1	Relaxation, Joy seeking
1839	HM0540	No	1	-
1840	HM0541	No	1	-
1841	HM0543	No	2	-
1842	HM0544	No	1	Job problem
1843	HM0545	No	2	-
1844	HM0546	No	1	-
1845	HM0548	No	1	-
1846	HM0550	No	1	-
1847	HM0552	No	1	-
1848	HM0553	No	2	-
1849	HM0554	No	1	-
1850	HM0555	No	1	-
1851	HM0556	No	1	-
1852	HM0557	No	1	-
1853	HM0558	No	1	-
1854	HM0559	No	1	-
1855	HM0560	No	1	Relaxation, Joy seeking
1856	HM0561	No	2	-
1857	HM0562	No	2	-
1858	HM0563	No	1	-
1859	HM0564	No	3	-
1860	HM0565	No	3	-
1861	HM0566	No	2	-
1862	HM0567	No	1	-
1863	HM0568	No	2	-
1864	HM0569	No	1	-
1865	HM0570	No	2	-
1866	HM0571	No	1	-
1867	HM0572	No	1	-

No	Patient ID	Other substances	No of substances abused at a time	Reason for substance use initiation
1868	HM0574	No	1	Relaxation, Joy seeking
1869	HM0575	No	2	Relaxation, Joy seeking, Peer pressure
1870	HM0577	No	1	-
1871	HM0578	No	2	Relaxation, Joy seeking
1872	HM0579	No	1	-
1873	HM0580	No	2	Relaxation, Joy seeking
1874	HM0581	No	2	-
1875	HM0582	No	2	Relaxation, Joy seeking
1876	HM0583	No	1	Family issue
1877	HM0584	No	2	-
1878	HM0589	No	1	Relaxation, Joy seeking
1879	HM0592	No	1	Relaxation, Joy seeking
1880	HM0593	No	1	-
1881	HM0594	No	1	Relaxation, Joy seeking
1882	HM0595	No	2	-
1883	HM0597	No	1	Family issue, Relaxation, Joy seeking
1884	HM0598	No	1	-
1885	HM0600	No	1	-
1886	HM0601	No	1	-
1887	HM0604	No	1	-
1888	HM0605	No	1	Relaxation, Joy seeking, Peer pressure
1889	HM0606	No	2	-
1890	HM0607	No	1	Relaxation, Joy seeking
1891	HM0608	No	1	-
1892	HM0609	No	1	-
1893	HM0610	Yes	2	-
1894	HM0612	No	1	Relaxation, Joy seeking
1895	HM0613	No	2	-
1896	HM0616	No	1	-
1897	HM0618	No	1	-
1898	HM0619	No	2	-
1899	HM0621	No	1	Relaxation, Joy seeking
1900	HM0622	No	1	Relaxation, Joy seeking

No	Patient ID	Other substances	No of substances abused at a time	Reason for substance use initiation
1901	HM0623	No	1	Family issue, Job problem
1902	HM0624	No	1	Relaxation, Joy seeking
1903	HM0626	No	3	-
1904	HM0627	No	1	Health, Relaxation, Joy seeking
1905	HM0628	No	1	-
1906	HM0630	No	4	-
1907	HM0631	No	3	Family issue
1908	HM0632	No	1	Relaxation, Joy seeking
1909	HM0633	No	1	-
1910	HM0634	No	3	Relaxation, Joy seeking
1911	HM0635	No	1	Relaxation, Joy seeking
1912	HM0636	No	3	Family issue, Relaxation, Joy seeking
1913	HM0638	No	1	-
1914	HM0639	No	1	Relaxation, Joy seeking
1915	HM0641	No	1	-
1916	HM0642	No	1	Relaxation, Joy seeking
1917	HM0643	No	2	Relaxation, Joy seeking
1918	HM0644	No	1	Relaxation, Joy seeking
1919	HM0645	No	2	-
1920	HM0646	No	2	Relaxation, Joy seeking, Peer pressure
1921	HM0647	No	2	-
1922	HM0649	No	1	Relaxation, Joy seeking
1923	HM0650	No	2	-
1924	HM0652	No	3	-
1925	HM0653	No	2	-
1926	HM0655	No	3	-
1927	HM0656	No	1	-
1928	HM0657	No	2	-
1929	HM0658	No	2	-
1930	HM0659	No	2	Relaxation, Joy seeking
1931	HM0660	No	2	-
1932	HM0661	No	1	Relaxation, Joy seeking
1933	HM0663	No	1	-
1934	HM0664	No	2	Relaxation, Joy seeking

No	Patient ID	Other substances	No of substances abused at a time	Reason for substance use initiation
1935	HM0665	No	1	-
1936	HM0666	No	2	-
1937	HM0669	No	1	Relaxation, Joy seeking
1938	HM0670	No	2	-
1939	HM0671	No	1	Relaxation, Joy seeking
1940	HM0672	No	1	-
1941	HM0674	No	1	Family issue, Joy seeking, Peer pressure
1942	HM0675	No	3	-
1943	HM0676	No	3	-
1944	HM0678	No	3	Relaxation, Joy seeking
1945	HM0679	No	1	Relaxation, Joy seeking
1946	HM0680	No	1	-
1947	HM0681	No	2	Joy seeking, Peer pressure
1948	HM0683	No	2	-
1949	HM0685	No	2	Relaxation, Joy seeking
1950	HM0688	No	1	Relaxation, Joy seeking
1951	HM0690	No	2	-
1952	HM0691	No	2	Relaxation, Joy seeking
1953	HM0692	No	1	-
1954	HM0693	Yes	2	-
1955	HM0695	No	1	-
1956	HM0699	No	1	Relaxation, Joy seeking
1957	HM0700	Yes	8	-
1958	HM0701	No	1	Relaxation, Joy seeking
1959	HM0702	No	1	Relaxation, Joy seeking
1960	HM0703	No	2	-
1961	HM0704	No	1	-
1962	HM0707	No	2	-
1963	HM0708	No	2	-
1964	HM0709	No	4	Relaxation, Joy seeking
1965	HM0710	No	2	-
1966	HM0711	No	2	Relaxation, Joy seeking
1967	HM0712	No	3	-
1968	HM0713	No	2	Relaxation, Joy seeking
1969	HM0715	No	1	-
1970	HM0716	No	2	-
1971	HM0717	No	1	-
1972	HM0718	No	3	-
1973	HM0719	No	1	Relaxation, Joy seeking

No	Patient ID	Other substances	No of substances abused at a time	Reason for substance use initiation
1974	HM0721	No	2	Family issue, Job problem, Relationship, Peer pressure
1975	HM0723	No	2	Health, Relaxation, Joy seeking
1976	HM0724	No	1	-
1977	HM0725	No	1	Relaxation, Joy seeking
1978	HM0726	No	1	-
1979	HM0729	No	2	-
1980	HM0730	No	2	-
1981	HM0732	No	1	Relaxation, Joy seeking
1982	HM0733	No	2	Relaxation, Joy seeking
1983	HM0735	No	1	Relaxation, Joy seeking
1984	HM0736	No	1	Relaxation, Joy seeking
1985	HM0737	No	1	-
1986	HM0738	No	1	Relaxation, Joy seeking
1987	HM0739	No	3	Relaxation, Joy seeking
1988	HM0740	No	2	-
1989	HM0741	No	4	-
1990	HM0742	No	2	-
1991	HM0743	No	3	-
1992	HM0744	No	1	-
1993	HM0745	No	1	-
1994	HM0746	No	3	Relaxation, Joy seeking
1995	HM0747	No	2	-
1996	HM0749	No	1	-
1997	HM0750	No	1	-
1998	HM0752	No	6	-
1999	HM0753	No	1	-
2000	HM0754	No	1	Relaxation, Joy seeking
2001	HM0756	No	1	Relaxation, Joy seeking
2002	HM0757	No	1	Relaxation, Joy seeking
2003	HM0758	No	2	Family issue, Relaxation, Joy seeking, Peer pressure
2004	HM0760	No	1	Relaxation, Joy seeking
2005	HM0761	No	2	Relaxation, Joy seeking
2006	HM0762	No	1	Relaxation, Joy seeking
2007	HM0763	No	2	-
2008	HM0764	No	2	-
2009	HM0767	No	1	Relaxation, Joy seeking
2010	HM0768	No	2	-

No	Patient ID	Other substances	No of substances abused at a time	Reason for substance use initiation
2011	HM0769	No	1	-
2012	HM0770	No	5	-
2013	HM0771	No	3	-
2014	HM0772	No	3	-
2015	HM0774	No	2	-
2016	HM0775	No	1	-
2017	HM0776	No	1	Relaxation, Joy seeking
2018	HM0779	No	1	-
2019	HM0781	No	4	-
2020	HM0782	Yes	2	Relaxation, Joy seeking
2021	HM0783	No	2	-
2022	HM0784	No	2	-
2023	HM0785	No	1	-
2024	HM0786	No	1	-
2025	HM0787	No	3	-
2026	HM0788	No	1	-
2027	HM0789	No	3	-
2028	HM0790	No	1	-
2029	HM0791	No	1	Family issue, Relationship, Peer pressure
2030	HM0792	No	1	-
2031	HM0793	No	1	-
2032	HM0794	No	1	-
2033	HM0795	No	2	-
2034	HM0796	No	3	-
2035	HM0797	No	1	-
2036	HM0798	No	2	-
2037	HM0800	No	1	-
2038	HM0801	No	2	-
2039	HM0802	No	2	-
2040	HM0803	No	1	-
2041	HM0805	No	1	-
2042	HM0806	No	1	-
2043	HM0807	No	2	-
2044	HM0809	No	3	Relaxation, Joy seeking
2045	HM0811	No	2	-
2046	HM0812	No	3	Relaxation, Joy seeking
2047	HM0813	No	2	Relaxation, Joy seeking
2048	HM0814	No	3	-
2049	HM0815	No	1	Relaxation, Joy seeking
2050	HM0816	No	1	Joy seeking, Peer pressure

No	Patient ID	Other substances	No of substances abused at a time	Reason for substance use initiation
2051	HM0817	No	3	-
2052	HM0819	No	3	Relaxation, Joy seeking
2053	HM0820	No	1	Relaxation, Joy seeking
2054	HM0821	No	1	Relaxation, Joy seeking
2055	HM0823	No	2	-
2056	HM0824	No	1	Relaxation, Joy seeking
2057	HM0825	No	1	Relaxation, Joy seeking
2058	HM0826	No	3	Family issue, Relationship
2059	HM0827	No	1	Relaxation, Joy seeking
2060	HM0828	No	1	-
2061	HM0829	No	3	Relaxation, Joy seeking
2062	HM0830	No	2	Relaxation, Joy seeking
2063	HM0834	No	4	-
2064	HM0835	No	4	-
2065	HM0836	No	1	-
2066	HM0837	No	1	Relaxation, Joy seeking
2067	HM0838	No	1	Relaxation, Joy seeking
2068	HM0839	No	2	-
2069	HM0840	No	1	Family issue, Peer pressure
2070	HM0841	No	1	Family issue, Peer pressure
2071	HM0843	No	1	Family issue, Peer pressure
2072	HM0846	No	1	Relaxation, Joy seeking
2073	HM0847	No	3	-
2074	HM0848	No	1	Relaxation, Joy seeking
2075	HM0849	No	2	Relaxation
2076	HM0850	No	1	Relaxation, Joy seeking
2077	HM0851	No	1	Relaxation, Joy seeking
2078	HM0852	No	1	-
2079	HM0853	No	1	Relaxation, Joy seeking
2080	HM0854	No	2	-
2081	HM0856	No	1	-
2082	HM0857	Yes	3	-
2083	HM0858	No	1	Relaxation, Joy seeking
2084	HM0860	No	1	-
2085	HM0861	No	1	Relaxation, Joy seeking
2086	HM0862	No	5	Peer pressure
2087	HM0863	No	3	Relaxation, Joy seeking

No	Patient ID	Other substances	No of substances abused at a time	Reason for substance use initiation
2088	HM0864	No	2	Relaxation, Joy seeking
2089	HM0865	No	2	-
2090	HM0866	No	2	Relaxation, Joy seeking
2091	HM0867	No	3	-
2092	HM0868	No	1	Relaxation, Joy seeking
2093	HM0869	No	1	-
2094	HM0871	No	3	-
2095	HM0872	No	1	-
2096	HM0873	No	2	Relaxation, Joy seeking
2097	HM0874	No	1	-
2098	HM0875	No	2	Relaxation, Joy seeking
2099	HM0876	Yes	4	-
2100	HM0877	No	1	-
2101	HM0879	No	1	-
2102	HM0880	No	1	Relaxation, Joy seeking
2103	HM0881	No	1	Relaxation, Joy seeking
2104	HM0882	No	2	-
2105	HM0883	No	2	-
2106	HM0884	No	1	-
2107	HM0885	No	1	Relaxation, Joy seeking
2108	HM0886	No	3	Relaxation, Joy seeking
2109	HM0888	No	3	Family issue, Peer pressure
2110	HM0889	No	1	-
2111	HM0890	No	1	-
2112	HM0891	No	1	Relaxation, Joy seeking
2113	HM0892	No	1	-
2114	HM0893	No	1	Relaxation, Joy seeking
2115	HM0894	No	1	-
2116	HM0895	No	1	Relaxation, Joy seeking
2117	HM0896	No	1	-
2118	HM0897	Yes	2	-
2119	HM0898	No	1	-
2120	HM0899	No	3	-
2121	HM0900	No	1	-
2122	HM0901	No	3	-
2123	HM0903	No	1	Family issue, Peer pressure
2124	HM0904	No	2	-

No	Patient ID	Other substances	No of substances abused at a time	Reason for substance use initiation
2125	HM0905	No	1	-
2126	HM0907	No	1	Relaxation, Joy seeking
2127	HM0908	No	1	-
2128	HM0910	No	1	-
2129	HM0911	No	1	Relaxation, Joy seeking
2130	HM0912	No	2	-
2131	HM0913	No	1	-
2132	HM0914	No	1	Relaxation, Joy seeking
2133	HM0915	No	1	Relaxation, Joy seeking
2134	HM0918	No	1	-
2135	HM0919	No	2	-
2136	HM0920	No	1	-
2137	HM0921	No	3	-
2138	HM0922	No	1	-
2139	HM0923	No	5	-
2140	HM0924	No	1	-
2141	HM0925	No	1	-
2142	HM0926	No	1	-
2143	HM0927	No	2	-
2144	HM0928	No	2	-
2145	HM0929	No	5	-
2146	HM0930	No	1	-
2147	HM0931	No	1	-
2148	HM0932	No	2	-
2149	HM0933	No	1	-
2150	HM0935	No	2	-
2151	HM0936	No	1	Relaxation, Joy seeking
2152	HM0937	No	2	Relaxation, Joy seeking
2153	HM0938	No	4	-
2154	HM0939	No	3	-
2155	HM0940	No	2	Relaxation, Joy seeking
2156	HM0941	No	1	-
2157	HM0942	No	1	Relaxation, Joy seeking
2158	HM0943	No	1	Relaxation, Joy seeking
2159	HM0944	No	1	-
2160	HM0945	No	1	Relaxation, Joy seeking
2161	HM0946	No	1	-
2162	HM0947	No	1	-
2163	HM0948	No	1	-
2164	HM0949	No	1	-

No	Patient ID	Other substances	No of substances abused at a time	Reason for substance use initiation
2165	HM0950	No	1	Relaxation, Joy seeking
2166	HM0951	No	2	-
2167	HM0952	No	2	Relaxation, Joy seeking
2168	HM0953	No	2	-
2169	HM0954	No	1	Relaxation, Joy seeking
2170	HM0956	No	2	-
2171	HM0957	No	2	-
2172	HM0958	No	2	Relaxation, Joy seeking
2173	HM0959	No	1	-
2174	HM0960	No	1	Relaxation, Joy seeking
2175	HM0961	No	3	Relaxation, Joy seeking
2176	HM0962	No	1	Relaxation, Joy seeking
2177	HM0964	No	2	Relaxation, Joy seeking
2178	HM0965	No	2	Relaxation, Joy seeking
2179	HM0965	No	2	Relaxation, Joy seeking
2180	HM0966	No	1	-
2181	HM0967	No	1	Relaxation, Joy seeking
2182	HM0969	Yes	3	Relaxation, Joy seeking
2183	HM0970	No	2	-
2184	HM0971	No	1	Relaxation, Joy seeking
2185	HM0972	No	2	-
2186	HM0973	No	2	Relaxation, Joy seeking
2187	HM0974	Yes	3	Relaxation, Joy seeking
2188	HM0975	No	1	-
2189	HM0976	No	2	Relaxation, Joy seeking
2190	HM0977	No	3	Relaxation, Joy seeking
2191	HM0978	No	1	-
2192	HM0979	No	1	Relaxation, Joy seeking
2193	HM0980	No	1	-
2194	HM0981	No	1	Relaxation, Joy seeking
2195	HM0982	No	3	Relaxation, Joy seeking
2196	HM0983	No	3	-
2197	HM0984	No	2	Relaxation, Joy seeking
2198	HM0985	No	1	-
2199	HM0986	No	2	-
2200	HM0988	No	2	-
2201	HM0989	No	1	Relaxation, Joy seeking
2202	HM0990	No	1	-
2203	HM0991	No	3	-
2204	HM0992	No	1	-

No	Patient ID	Other substances	No of substances abused at a time	Reason for substance use initiation
2205	HM0993	No	1	-
2206	HM0994	No	3	-
2207	HM0996	No	2	Relaxation, Joy seeking
2208	HM0997	No	1	Relaxation, Joy seeking
2209	HM0998	No	2	-
2210	HM0999	No	3	Relaxation, Joy seeking
2211	HM1000	No	1	-
2212	HM1001	No	1	Relaxation, Joy seeking
2213	HM1002	No	2	-
2214	HM1003	No	1	Relaxation, Joy seeking
2215	HM1004	No	1	-
2216	HM1005	No	2	Relaxation, Joy seeking
2217	HM1006	No	1	-
2218	HM1007	No	1	Relaxation, Joy seeking
2219	HM1008	No	1	Relaxation, Joy seeking
2220	HM1009	No	3	-
2221	HM1010	No	1	Relaxation, Joy seeking
2222	HM1011	No	2	-
2223	HM1012	No	1	Relaxation, Joy seeking
2224	HM1013	No	1	-
2225	HM1014	No	3	-
2226	HM1015	No	3	-
2227	HM1016	No	2	Relaxation, Joy seeking
2228	HM1017	No	3	-
2229	HM1018	No	1	Relaxation, Joy seeking
2230	HM1019	No	2	Relaxation, Joy seeking
2231	HM1020	No	3	Family issue
2232	HM1021	No	1	-
2233	HM1022	No	3	Relaxation, Joy seeking
2234	HM1023	No	1	-
2235	HM1024	No	1	-
2236	HM1025	No	5	-
2237	HM1026	No	2	Relaxation, Joy seeking
2238	HM1027	No	1	Relaxation, Joy seeking
2239	HM1028	No	1	-
2240	HM1030	No	1	-
2241	HM1031	No	1	Relaxation, Joy seeking
2242	HM1032	No	1	-

No	Patient ID	Other substances	No of substances abused at a time	Reason for substance use initiation
2243	HM1033	No	2	-
2244	HM1034	No	3	-
2245	HM1035	No	2	-
2246	HM1036	No	2	-
2247	HM1037	No	1	-
2248	HM1038	No	4	-
2249	HM1039	No	2	Relaxation, Joy seeking
2250	HM1040	No	2	-
2251	HM1041	No	2	-
2252	HM1042	No	2	-
2253	HM1043	No	1	Relaxation, Joy seeking
2254	HM1044	No	3	-
2255	HM1045	No	2	-
2256	HM1046	No	2	-
2257	HM1047	No	1	-
2258	HM1048	No	2	Relaxation, Joy seeking
2259	HM1049	No	1	-
2260	HM1050	No	2	Relaxation, Joy seeking
2261	HM1051	No	1	-
2262	HM1052	No	1	Relaxation, Joy seeking
2263	HM1053	No	1	-
2264	HM1054	No	1	-
2265	HM1055	No	3	-
2266	HM1056	No	1	-
2267	HM1058	No	1	-
2268	HM1059	Yes	2	-
2269	HM1060	No	1	-
2270	HM1061	No	1	-
2271	HM1062	No	1	-
2272	HM1063	No	1	Joy seeking
2273	HM1064	No	1	-
2274	HM1065	No	2	-
2275	HM1066	No	1	-
2276	HM1068	No	1	-
2277	HM1070	No	1	-
2278	HM1080	No	1	-
2279	HM1093	No	1	Peer pressure
2280	HM1116	No	1	-
2281	HM1117	Yes	3	Peer pressure
2282	HM1120	No	3	-

No	Patient ID	Other substances	No of substances abused at a time	Reason for substance use initiation
2283	HM1122	No	1	Job problem, Peer pressure
2284	HM1159	No	2	-
2285	HM1160	No	2	-
2286	HM1161	No	2	Peer pressure
2287	HM1163	No	2	-
2288	HM1165	No	1	-
2289	HM1166	No	1	-
2290	HM1167	No	2	Relaxation, Joy seeking
2291	HM1168	No	1	Relaxation, Joy seeking
2292	HM1171	No	1	Relaxation, Joy seeking
2293	HM1172	No	2	Health, Relationship, Relaxation, Joy seeking
2294	HM1173	No	2	-
2295	HM1175	No	2	-
2296	HM1177	No	2	-
2297	HM1178	No	1	-
2298	HM1179	No	1	-
2299	HM1180	No	1	Relaxation, Joy seeking
2300	HM1181	No	1	-
2301	HM1182	No	1	Relaxation, Joy seeking
2302	HM1183	No	1	Relaxation, Joy seeking
2303	HM1184	No	1	Relaxation, Joy seeking
2304	HM1185	No	1	Relaxation, Joy seeking
2305	HM1186	No	1	Relaxation, Joy seeking
2306	HM1188	No	1	Relaxation, Joy seeking
2307	HM1189	No	2	Relaxation, Joy seeking
2308	HM1190	No	2	Relaxation, Joy seeking
2309	HM1191	No	1	Relaxation, Joy seeking
2310	HM1192	No	1	Relaxation, Joy seeking
2311	HM1193	No	1	Relaxation, Joy seeking
2312	HM1196	No	3	Relaxation, Joy seeking
2313	HM1198	Yes	2	-
2314	HM1199	No	1	-
2315	HM1200	No	1	-
2316	HM1201	No	2	Joy seeking, Peer pressure
2317	HM1202	No	1	-
2318	HM1203	No	1	-
2319	HM1204	No	4	-

No	Patient ID	Other substances	No of substances abused at a time	Reason for substance use initiation
2320	HM1205	No	2	Relaxation, Joy seeking
2321	HM1206	No	2	Relaxation, Joy seeking
2322	HM1208	No	1	-
2323	HM1209	No	1	Relaxation, Joy seeking
2324	HM1210	No	1	-
2325	HM1212	No	1	-
2326	HM1213	No	1	Relaxation, Joy seeking, Peer pressure
2327	HM1214	No	1	Relaxation, Joy seeking
2328	HM1215	No	1	-
2329	HM1216	No	3	Family issue, Relaxation, Joy seeking
2330	HM1217	No	1	-
2331	HM1221	No	2	Relaxation, Joy seeking
2332	HM1222	No	2	-
2333	HM1223	No	2	Relaxation, Joy seeking
2334	HM1224	No	2	Relaxation, Joy seeking
2335	HM1225	No	2	-
2336	HM1226	No	3	Relaxation, Joy seeking
2337	HM1227	No	1	-
2338	HM1229	No	1	-
2339	HM1230	No	2	Relaxation, Joy seeking
2340	HM1232	No	1	Joy seeking, Peer pressure
2341	HM1233	No	2	Relaxation, Joy seeking
2342	HM1234	No	1	Relaxation, Joy seeking
2343	HM1235	No	1	Relaxation, Joy seeking
2344	HM1236	No	1	Relaxation, Joy seeking
2345	HM1237	No	1	Relaxation, Joy seeking
2346	HM1238	No	1	Relaxation, Joy seeking
2347	HM1239	No	1	Relaxation, Joy seeking
2348	HM1240	No	1	Relaxation, Joy seeking
2349	HM1242	No	4	-
2350	HM1243	No	1	Joy seeking, Peer pressure
2351	HM1244	No	1	-
2352	HM1246	No	1	Relaxation, Joy seeking
2353	HM1247	No	1	Relaxation, Joy seeking
2354	HM1249	No	1	Relaxation, Joy seeking
2355	HM1250	No	1	Relaxation, Joy seeking

No	Patient ID	Other substances	No of substances abused at a time	Reason for substance use initiation
2356	HM1251	No	2	Joy seeking, Peer pressure
2357	HM1252	No	1	-
2358	HM1253	No	1	Relaxation, Joy seeking
2359	HM1254	No	1	Relaxation, Joy seeking
2360	HM1256	No	1	-
2361	HM1257	No	4	-
2362	HM1258	No	1	Relaxation, Joy seeking
2363	HM1259	No	2	Relaxation, Joy seeking
2364	HM1260	No	1	-
2365	HM1261	No	1	Relaxation, Joy seeking
2366	HM1262	No	3	-
2367	HM1263	No	1	Relaxation, Joy seeking
2368	HP0002	No	4	Peer pressure
2369	HP0004	No	2	Job problem
2370	HP0005	No	1	Others
2371	HP0006	No	2	-
2372	HP0007	No	2	Relaxation, Peer pressure
2373	HP0009	No	1	Relaxation
2374	HP0010	No	2	Others
2375	HP0011	Yes	4	Health, Relaxation
2376	HP0012	No	3	Peer pressure
2377	HP0014	No	3	-
2378	HP0015	No	2	Relaxation
2379	HP0016	No	2	-
2380	HP0018	No	1	Relaxation
2381	HP0019	No	1	Peer pressure
2382	HP0020	No	1	-
2383	HP0023	Yes	5	Peer pressure
2384	HP0024	No	1	-
2385	HP0025	No	3	Relationship, Relaxation
2386	HP0027	No	2	Job problem, Relaxation
2387	HP0028	No	3	-
2388	HP0029	No	2	Relaxation
2389	HP0030	No	2	-
2390	HP0031	No	1	Relaxation, Peer pressure
2391	HP0033	No	4	-

No	Patient ID	Other substances	No of substances abused at a time	Reason for substance use initiation
2392	HP0034	No	2	Peer pressure
2393	HP0035	No	1	-
2394	HP0036	No	1	Others
2395	HP0037	No	1	Others
2396	HP0037	No	2	Peer pressure
2397	HP0040	No	2	Job problem
2398	HP0041	No	2	Peer pressure
2399	HP0042	No	3	Relaxation
2400	HP0045	No	1	Peer pressure
2401	HP0046	No	3	-
2402	HP0048	No	1	Others
2403	HP0051	No	1	-
2404	HP0052	No	2	-
2405	HP0054	No	1	-
2406	HP0055	No	1	-
2407	HP0056	No	1	-
2408	HP0057	No	1	-
2409	HP0058	No	1	-
2410	HP0059	No	1	-
2411	HP0060	No	1	-
2412	HP0061	No	2	-
2413	HP0062	No	1	-
2414	HP0066	No	1	-
2415	HP0067	No	2	-
2416	HP0068	No	1	-
2417	HP0069	No	2	-
2418	HP0070	No	1	-
2419	HP0071	No	1	-
2420	HP0072	No	1	-
2421	HP0073	No	1	-
2422	HP0075	No	2	-
2423	HP0077	No	2	-
2424	HP0078	No	1	-
2425	HP0080	No	3	-
2426	HP0081	No	2	Others
2427	HP0082	No	1	-
2428	HP0083	No	1	-
2429	HP0084	No	1	-

No	Patient ID	Other substances	No of substances abused at a time	Reason for substance use initiation
2430	HP0085	No	1	Relaxation, Peer pressure
2431	HP0086	No	2	Job problem
2432	HP0087	No	1	-
2433	HP0088	No	1	-
2434	HP0089	No	1	-
2435	HP0090	No	1	-
2436	HP0091	No	2	-
2437	HP0092	No	2	-
2438	HP0093	No	2	Others
2439	HP0094	No	2	-
2440	HP0095	No	2	-
2441	HP0096	No	3	Others
2442	HP0097	No	2	Peer pressure
2443	HP0098	No	1	Peer pressure
2444	HP0099	No	1	-
2445	HP0100	No	1	-
2446	HP0102	No	1	Relaxation, Joy seeking, Peer pressure
2447	HP0105	No	1	-
2448	HP0108	No	1	-
2449	HP0109	No	2	-
2450	HP0110	No	1	-
2451	HP0111	No	1	-
2452	HP0118	No	1	-
2453	HP0119	No	1	-
2454	HP0120	No	2	Relationship, Relaxation
2455	HP0121	No	1	-
2456	HP0122	No	2	Others
2457	HP0123	No	2	-
2458	HP0124	No	2	Peer pressure
2459	HP0126	No	1	Relaxation
2460	HP0127	No	1	-
2461	HP0129	No	2	Relationship
2462	HP0130	No	1	-

No	Patient ID	Other substances	No of substances abused at a time	Reason for substance use initiation
2463	HP0136	No	1	-
2464	HP0140	No	1	-
2465	HP0141	No	1	-
2466	HP0142	No	2	-
2467	HP0143	No	1	-
2468	HP0145	No	2	-
2469	HP0146	No	1	-
2470	HP0148	No	1	-
2471	HP0149	No	1	-
2472	HPP0001	No	2	-
2473	HPP0002	No	1	-
2474	HPP0003	No	1	Relaxation
2475	HPP0004	No	1	Relaxation
2476	HPP0007	No	3	-
2477	HPP0008	No	1	-
2478	HPP0010	No	2	Job problem, Relationship, Others
2479	HPP0011	No	4	-
2480	HPP0013	No	2	-
2481	HPP0014	No	4	-
2482	HPP0016	No	4	-
2483	HPP0017	No	2	-
2484	HPP0019	No	2	Relaxation
2485	HPP0020	No	1	-
2486	HPP0022	No	3	Relationship
2487	HPP0024	No	1	-
2488	HPP0025	Yes	4	Job problem, Peer pressure
2489	HPP0026	No	4	-
2490	HPP0027	No	2	-
2491	HPP0028	No	2	Relationship
2492	HPP0029	No	1	-
2493	HPP0030	No	2	-
2494	HPP0031	No	1	Job problem, Relaxation

No	Patient ID	Other substances	No of substances abused at a time	Reason for substance use initiation
2495	HPP0032	No	1	Peer pressure
2496	HPP0040	No	3	-
2497	HPP0043	No	1	-
2498	HPP0048	No	1	-
2499	HPP0051	No	1	-
2500	HPP0052	No	1	-
2501	HPP0056	No	1	Relaxation, Joy seeking
2502	HPP0063	No	2	Job problem, Peer pressure
2503	HPP0064	No	2	-
2504	HPP0065	No	1	-
2505	HPP0066	No	2	-
2506	HPP0067	No	3	-
2507	HPP0068	No	1	-
2508	HPP0070	No	4	Joy seeking, Peer pressure
2509	HPP0074	No	3	-
2510	HPP0075	No	3	-
2511	HPP0077	Yes	5	Peer pressure
2512	HPP0079	No	5	-
2513	HPP0080	No	2	Joy seeking, Others
2514	HPP0081	No	2	Health, Relaxation
2515	HPP0083	No	3	Joy seeking, Peer pressure, Others
2516	HPP0085	No	3	-
2517	HPP0087	Yes	4	Joy seeking, Peer pressure, Others
2518	HPP0089	No	3	Joy seeking, Peer pressure
2519	HPP0090	Yes	3	Joy seeking, Others
2520	HPP0091	No	1	-
2521	HPP0092	No	5	Relaxation, Joy seeking, Peer pressure, Others
2522	HPP0093	No	1	-

No	Patient ID	Other substances	No of substances abused at a time	Reason for substance use initiation
2523	HPP0094	No	3	-
2524	HPP0095	No	2	Relaxation
2525	HPP0096	No	3	-
2526	HPP0097	No	1	-
2527	HPP0098	No	4	-
2528	HPP0099	No	2	Relaxation
2529	HPP0100	No	4	-
2530	HPP0101	No	2	Family issue, Relationship, Joy seeking, Peer pressure, Others
2531	HPP0102	Yes	5	-
2532	HPP0103	No	5	Peer pressure
2533	HPP0104	No	1	-
2534	HPP0107	Yes	6	Joy seeking, Peer pressure
2535	HPP0108	No	2	Relaxation
2536	HPP0109	No	4	-
2537	HPP0110	Yes	5	-
2538	HPP0111	No	3	Peer pressure
2539	HPP0112	No	2	-
2540	HPP0113	No	2	-
2541	HPP0114	No	1	-
2542	HPP0117	No	3	-
2543	HPP0118	No	3	-
2544	HPP0120	No	2	-
2545	HPP0121	No	4	Joy seeking, Peer pressure, Others
2546	HPP0122	No	2	-
2547	HPP0123	No	1	-
2548	HPP0124	No	1	-
2549	HPP0125	No	5	Peer pressure
2550	HPP0126	No	2	-
2551	HPP0127	No	6	-
2552	HPP0128	No	2	-
2553	HPP0129	No	3	-
2554	HPP0131	No	3	Relaxation
2555	HPP0132	No	3	-
2556	HPP0135	No	3	-
2557	HPP0138	Yes	4	-
2558	HPP0139	No	1	Peer pressure, Others

No	Patient ID	Other substances	No of substances abused at a time	Reason for substance use initiation
2559	HPP0140	Yes	3	Peer pressure
2560	HPP0141	No	3	-
2561	HPP0145	No	1	Family issue, Relaxation, Others
2562	HPP0146	No	2	-
2563	HPP0148	No	1	-
2564	HPP0149	No	3	Health
2565	HPP0151	No	4	-
2566	HPP0152	No	2	Joy seeking, Peer pressure
2567	HPP0154	No	2	-
2568	HPP0156	No	1	Family issue, Joy seeking
2569	HPP0157	No	3	-
2570	HPP0160	No	1	-
2571	HPP0162	No	3	Peer pressure
2572	HPP0163	No	3	Relaxation
2573	HPP0165	No	3	-
2574	HPP0167	Yes	4	-
2575	HPP0168	No	2	Relaxation
2576	HPP0169	No	2	Joy seeking, Peer pressure
2577	HPP0171	No	2	-
2578	HPP0172	No	3	-
2579	HPP0173	No	4	-
2580	HPP0175	No	2	Peer pressure
2581	HPP0179	No	3	-
2582	HPP0180	No	3	Peer pressure, Others
2583	HPP0181	No	2	Peer pressure
2584	HPP0182	No	3	-
2585	HPP0183	No	2	-
2586	HPP0184	No	1	Peer pressure, Others
2587	HPP0186	No	2	-
2588	HPP0188	No	2	Job problem, Peer pressure

No	Patient ID	Other substances	No of substances abused at a time	Reason for substance use initiation
2589	HPP0192	No	1	-
2590	HPP0193	No	2	Relaxation
2591	HPP0194	No	4	-
2592	HPP0196	Yes	4	-
2593	HPP0198	No	6	Peer pressure
2594	HPP0199	No	3	-
2595	HPP0201	No	3	-
2596	HPP0202	No	2	-
2597	HPP0203	No	2	Peer pressure
2598	HPP0204	No	1	-
2599	HPP0205	No	4	-
2600	HPP0206	No	1	-
2601	HPP0207	No	3	-
2602	HPP0208	No	1	-
2603	HPP0209	No	1	-
2604	HPP0210	No	1	-
2605	HPP0211	No	1	-
2606	HPP0212	No	3	-
2607	HPP0213	No	2	Job problem, Relationship
2608	HPP0215	No	1	Peer pressure
2609	HPP0216	No	2	-
2610	HPP0217	No	1	-
2611	HPP0218	No	4	-
2612	HPP0219	Yes	2	-
2613	HPP0221	No	1	Peer pressure
2614	HPP0222	No	1	-
2615	HPP0223	No	2	-
2616	HPP0224	No	2	-
2617	HPP0225	No	1	-
2618	HPP0226	No	1	Job problem, Relaxation
2619	HPP0228	No	2	Relaxation

No	Patient ID	Other substances	No of substances abused at a time	Reason for substance use initiation
2620	HPP0230	No	5	-
2621	HPP0231	No	1	-
2622	HPP0233	No	4	Family issue
2623	HPP0234	No	5	Peer pressure
2624	HPP0236	No	1	-
2625	HPP0237	No	2	-
2626	HPP0238	No	1	-
2627	HPP0239	No	2	Peer pressure
2628	HPP0240	No	1	-
2629	HPP0241	No	3	-
2630	HPP0244	No	3	-
2631	HPP0245	No	3	-
2632	HPP0246	Yes	3	Peer pressure
2633	HPP0247	No	1	-
2634	HPP0248	No	2	-
2635	HPP0250	No	1	-
2636	HPP0251	No	2	-
2637	HPP0252	No	1	-
2638	HPP0253	No	3	-
2639	HPP0256	No	4	-
2640	HPP0258	No	2	Peer pressure, Others
2641	HPP0259	No	3	-
2642	HPP0260	No	4	-
2643	HPP0261	No	2	-
2644	HPP0262	No	1	-
2645	HPP0264	No	1	-
2646	HPP0265	No	4	-
2647	HPP0266	No	2	Joy seeking
2648	HPP0268	No	2	Family issue
2649	HPP0269	No	2	-
2650	HPP0271	No	1	-
2651	HPP0272	No	1	-
2652	HPP0273	No	1	-
2653	HPP0276	No	4	-

No	Patient ID	Other substances	No of substances abused at a time	Reason for substance use initiation
2654	HPP0278	No	1	Relationship
2655	HPP0280	No	2	-
2656	HPP0281	No	3	-
2657	HPP0282	No	2	Peer pressure
2658	HPP0283	No	1	Others
2659	HPP0284	No	1	-
2660	HPP0287	No	1	-
2661	HPP0290	No	2	Peer pressure
2662	HPP0291	Yes	1	-
2663	HPP0294	No	4	Relationship, Peer pressure
2664	HPP0295	No	3	-
2665	HPP0296	No	2	-
2666	HPP0297	No	1	-
2667	HPP0298	No	4	-
2668	HPP0299	No	3	-
2669	HPP0300	No	2	Peer pressure
2670	HPP0301	No	3	Peer pressure
2671	HPP0303	No	2	-
2672	HPP0304	No	2	-
2673	HPP0305	Yes	1	Family issue, Others
2674	HPP0306	No	1	-
2675	HPP0308	No	1	-
2676	HPP0310	No	2	Relaxation
2677	HPP0311	No	2	Peer pressure
2678	HPP0316	No	1	-
2679	HPP0317	No	2	-
2680	HPP0320	No	2	-
2681	HPP0321	No	1	-
2682	HPP0322	No	3	Peer pressure
2683	HPP0323	No	1	-
2684	HPP0325	No	2	-
2685	HPP0326	No	1	Family issue
2686	HPP0328	Yes	4	-
2687	HPP0329	No	1	Family issue, Relationship, Relaxation, Joy seeking
2688	HPP0330	No	4	Relaxation

No	Patient ID	Other substances	No of substances abused at a time	Reason for substance use initiation
2689	HPP0331	No	5	Joy seeking, Peer pressure
2690	HPP0332	No	4	-
2691	HPP0334	No	1	Joy seeking
2692	HPP0335	No	2	Others
2693	HPP0336	No	2	-
2694	HPP0337	No	2	-
2695	HPP0338	No	1	Peer pressure
2696	HPP0339	No	5	-
2697	HPP0340	No	1	-
2698	HPP0341	No	3	-
2699	HPP0342	No	1	Family issue, Others
2700	HPP0343	No	2	Others
2701	HPP0344	Yes	3	-
2702	HPP0345	No	3	Peer pressure
2703	HPP0346	No	2	Relaxation
2704	HPP0348	No	1	-
2705	HPP0349	No	1	-
2706	HPP0352	No	2	Peer pressure
2707	HPP0354	No	2	-
2708	HPP0355	No	1	Others
2709	HPP0357	No	2	-
2710	HPP0358	Yes	7	-
2711	HPP0359	No	1	Others
2712	HPP0360	No	1	-
2713	HPP0361	No	1	-
2714	HPP0362	No	1	Peer pressure
2715	HPP0371	No	2	-
2716	HPP0372	No	1	Others
2717	HPP0391	Yes	3	Family issue, Relationship
2718	HPP0394	No	1	-
2719	HPP0395	Yes	2	-
2720	HPP0397	No	2	-
2721	HPP0398	No	1	Job problem
2722	HPP0399	No	2	Peer pressure
2723	HPP0400	No	1	Others
2724	HPP0401	No	2	-

No	Patient ID	Other substances	No of substances abused at a time	Reason for substance use initiation
2725	HPP0404	No	2	-
2726	HPP0405	No	2	Peer pressure
2727	HPP0406	No	1	-
2728	HPP0409	No	2	Peer pressure
2729	HPP0411	No	1	Relationship
2730	HPP0413	No	2	-
2731	HPP0414	No	4	-
2732	HPP0415	No	3	Peer pressure
2733	HPP0416	No	1	-
2734	HPP0417	No	2	Peer pressure
2735	HPP0421	No	5	-
2736	HPP0423	No	3	-
2737	HPP0424	No	4	-
2738	HPP0425	No	1	-
2739	HPP0426	No	2	-
2740	HPP0427	Yes	3	-
2741	HPP0430	No	2	Peer pressure
2742	HPP0431	No	1	-
2743	HPP0433	No	1	-
2744	HPP0435	No	3	-
2745	HPP0436	No	1	Family issue, Relationship
2746	HPP0437	No	2	-
2747	HPP0438	No	1	-
2748	HPP0440	No	1	-
2749	HPP0443	No	1	-
2750	HPP0444	No	2	Family issue, Peer pressure
2751	HPP0445	No	3	-
2752	HPP0448	No	2	-
2753	HPP0449	No	1	Peer pressure
2754	HPP0450	No	2	-
2755	HPP0454	No	1	-
2756	HPP0455	No	4	-
2757	HPP0457	No	2	Others
2758	HPP0460	Yes	6	-
2759	HPP0476	No	1	Joy seeking, Peer pressure
2760	HPP0478	No	2	-
2761	HPP0479	No	1	-
2762	HPP0480	No	3	-
2763	HPP0481	No	1	-

No	Patient ID	Other substances	No of substances abused at a time	Reason for substance use initiation
2764	HPP0482	No	1	-
2765	HPP0483	No	1	-
2766	HPP0484	No	1	-
2767	HPP0485	No	3	Relaxation, Peer pressure
2768	HPP0486	Yes	3	-
2769	HPP0487	No	2	-
2770	HPP0488	No	1	-
2771	HPP0490	No	1	-
2772	HPP0492	No	1	-
2773	HPP0494	No	2	-
2774	HPP0495	No	1	-
2775	HPP0496	No	2	-
2776	HPP0499	No	2	-
2777	HPP0502	No	2	-
2778	HPP0503	No	2	-
2779	HPP0504	No	2	Peer pressure
2780	HPP0505	No	1	-
2781	HPP0506	No	1	Relaxation
2782	HPP0508	No	1	-
2783	HPP0509	Yes	2	-
2784	HPP0511	No	3	-
2785	HPP0512	No	3	Peer pressure
2786	HPP0513	Yes	5	-
2787	HPP0514	No	2	-
2788	HPP0558	No	2	-
2789	HPP0561	No	2	-
2790	HPP0562	No	4	-
2791	HPP0563	No	2	Relationship
2792	HPP0566	No	2	Peer pressure
2793	HPP0567	No	1	Family issue
2794	HPP0568	No	1	-
2795	HPP0569	No	1	Relaxation
2796	HPP0571	No	4	Joy seeking
2797	HPP0572	No	1	-
2798	HPP0573	Yes	1	-
2799	HPP0575	No	2	-
2800	HPP0576	No	1	Peer pressure
2801	HPP0577	No	3	Relaxation
2802	HPP0578	No	2	-

No	Patient ID	Other substances	No of substances abused at a time	Reason for substance use initiation
2803	HPP0579	No	1	-
2804	HPP0581	No	1	-
2805	HPP0582	Yes	5	-
2806	HPP0583	No	1	-
2807	HPP0584	No	3	-
2808	HPP0585	No	1	-
2809	HPP0586	No	3	-
2810	HPP0587	No	1	-
2811	HPP0589	No	4	-
2812	HPP0590	No	3	-
2813	HPP0592	No	1	-
2814	HPP0593	No	1	Peer pressure
2815	HPP0595	No	1	-
2816	HPP0596	No	2	Peer pressure
2817	HPP0597	No	1	-
2818	HPP0598	No	2	-
2819	HPP0599	No	1	-
2820	HPP0600	No	1	-
2821	HPP0601	No	1	-
2822	HPP0603	No	1	Peer pressure
2823	HPP0605	No	4	Peer pressure
2824	HPP0607	No	1	Relationship
2825	HPP0609	No	1	-
2826	HPP0610	No	2	-
2827	HPP0611	No	3	-
2828	HPP0612	No	3	-
2829	HPP0613	No	2	-
2830	HPP0614	No	2	-
2831	HPP0615	No	2	-
2832	HPP0616	No	2	-
2833	HPP0617	No	3	-
2834	HPP0619	No	1	-
2835	HPP0620	No	1	Peer pressure
2836	HPP0622	No	2	Peer pressure
2837	HPP0624	No	1	-
2838	HPP0626	No	2	-
2839	HPP0627	No	1	-
2840	HPP0628	No	1	-
2841	HPP0629	No	2	-
2842	HPP0630	No	1	-

No	Patient ID	Other substances	No of substances abused at a time	Reason for substance use initiation
2843	HPP0631	No	1	-
2844	HPP0632	No	1	-
2845	HPP0636	No	2	-
2846	HPP0637	No	2	-
2847	HPP0639	Yes	4	-
2848	HPP0640	No	4	-
2849	HPP0641	No	1	-
2850	HPP0642	No	4	Family issue, Relationship, Financial problem
2851	HPP0643	No	1	-
2852	HPP0644	No	2	-
2853	HPP0647	No	3	-
2854	HPP0648	No	2	-
2855	HPP0649	No	1	-
2856	HPP0651	No	2	-
2857	HPP0652	No	1	Peer pressure
2858	HPP0653	No	2	-
2859	HPP0655	No	4	-
2860	HPP0659	No	2	Job problem
2861	HPP0661	No	1	-
2862	HPP0662	No	3	-
2863	HPP0664	No	2	Peer pressure
2864	HPP0666	No	1	-
2865	HPP0667	No	1	-
2866	HPP0669	No	3	-
2867	HPP0671	No	1	Relaxation
2868	HPP0674	No	1	-
2869	HPP0675	No	2	-
2870	HPP0676	No	2	-
2871	HPP0677	No	3	Peer pressure
2872	HPP0678	No	2	-
2873	HPP0679	No	1	Peer pressure
2874	HPP0691	No	2	Peer pressure
2875	HPP0699	No	4	Relaxation, Peer pressure
2876	HPP0700	Yes	4	Others
2877	HPP0701	No	3	-
2878	HPP0702	No	1	Peer pressure
2879	HPP0703	No	2	Peer pressure

No	Patient ID	Other substances	No of substances abused at a time	Reason for substance use initiation
2880	HPP0704	No	3	Joy seeking
2881	HPP0705	No	1	Others
2882	HPP0706	No	2	Peer pressure, Others
2883	HPP0707	No	5	Joy seeking, Peer pressure
2884	HPP0708	No	1	Peer pressure
2885	HPP0709	No	3	Peer pressure
2886	HPP0710	No	3	Peer pressure
2887	HPP0711	No	1	Peer pressure
2888	HPP0713	Yes	4	Peer pressure
2889	HPP0723	Yes	2	-
2890	HPP0727	No	3	-
2891	HPP0731	No	1	-
2892	HPP0733	No	1	-
2893	HPP0734	No	1	-
2894	HPP0735	No	1	-
2895	HPP0736	No	3	-
2896	HPP0737	No	1	-
2897	HPP0738	No	3	-
2898	HPP0739	No	1	-
2899	HPP0740	No	1	-
2900	HPP0741	Yes	4	-
2901	HPP0743	No	1	-
2902	HPP0744	No	1	-
2903	HPP0746	No	2	-
2904	HPP0748	No	1	-
2905	HPP0749	No	1	-
2906	HPP0750	No	3	-
2907	HPP0751	No	1	-
2908	HPP0754	No	4	-
2909	HPP0755	No	2	-
2910	HPP0756	No	2	-
2911	HPP0757	No	1	-
2912	HPP0758	Yes	4	Joy seeking, Peer pressure
2913	HPP0759	No	1	-
2914	HPP0760	No	1	-
2915	HPP0762	No	1	-
2916	HPP0763	No	1	-
2917	HPP0764	No	4	-

No	Patient ID	Other substances	No of substances abused at a time	Reason for substance use initiation
2918	HPP0765	No	1	-
2919	HPP0767	No	2	-
2920	HPP0769	No	1	-
2921	HPP0770	No	2	-
2922	HPP0771	No	1	-
2923	HPP0773	No	3	-
2924	HPP0774	No	1	-
2925	HPP0775	No	1	-
2926	HPP0776	No	2	-
2927	HPP0777	No	2	-
2928	HPP0780	No	1	-
2929	HPP0782	No	1	-
2930	HPP0783	Yes	3	Peer pressure
2931	HPP0784	No	1	Peer pressure
2932	HPP0786	No	1	-
2933	HPP0788	No	1	-
2934	HPP0789	No	2	-
2935	HPP0790	No	1	-
2936	HPP0791	No	1	-
2937	HPP0792	No	1	-
2938	HPP0793	No	1	-
2939	HPP0795	No	2	-
2940	HPP0796	No	2	-
2941	HPP0797	No	3	Peer pressure
2942	HPP0799	No	1	-
2943	HPP0800	No	2	-
2944	HPP0801	No	3	-
2945	HPP0802	No	1	-
2946	HPP0804	Yes	4	Peer pressure
2947	HPP0805	No	1	-
2948	HPP0807	No	2	-
2949	HPP0808	No	1	-
2950	HPP0809	No	3	-
2951	HPP0810	No	1	Peer pressure
2952	HPP0811	No	1	-
2953	HPP0812	No	4	-
2954	HPP0813	No	1	-
2955	HPP0816	No	3	-
2956	HPP0817	No	2	-
2957	HPP0819	No	1	-

No	Patient ID	Other substances	No of substances abused at a time	Reason for substance use initiation
2958	HPP0820	No	2	-
2959	HPP0821	No	2	-
2960	HPP0822	No	2	-
2961	HPP0823	No	2	-
2962	HPP0824	No	1	-
2963	HPP0826	No	1	-
2964	HPP0827	No	2	-
2965	HPP0829	No	2	-
2966	HPP0830	No	1	-
2967	HPP0832	No	2	-
2968	HPP0833	No	1	-
2969	HPP0834	No	1	Family issue
2970	HPP0836	Yes	2	-
2971	HPP0837	No	1	-
2972	HPP0838	No	2	-
2973	HPP0839	No	1	-
2974	HPP0840	No	2	-
2975	HPP0841	No	1	-
2976	HPP0842	No	4	-
2977	HPP0843	No	3	Peer pressure
2978	HPP0844	No	3	-
2979	HPP0845	No	5	-
2980	HPP0848	No	1	-
2981	HPP0849	No	1	-
2982	HPP0850	No	1	-
2983	HPP0851	No	1	-
2984	HPP0854	No	1	-
2985	HPP0855	No	1	-
2986	HPP0857	No	1	-
2987	HPP0858	No	1	-
2988	HPP0859	No	1	-
2989	HPP0860	No	2	-
2990	HPP0861	No	1	-
2991	HPP0862	No	1	-
2992	HPP0863	No	5	Relaxation
2993	HPP0864	No	2	-
2994	HPP0865	No	2	-
2995	HPP0866	No	1	-
2996	HPP0867	No	2	-
2997	HPP0868	Yes	2	-
2998	HPP0870	No	1	-

No	Patient ID	Other substances	No of substances abused at a time	Reason for substance use initiation
2999	HPP0871	No	3	-
3000	HPP0872	No	1	-
3001	HPP0873	No	4	-
3002	HPP0874	No	4	-
3003	HPP0875	No	1	-
3004	HPP0876	No	3	-
3005	HPP0877	No	1	-
3006	HPP0878	No	1	-
3007	HPP0879	No	1	-
3008	HPP0880	No	3	-
3009	HPP0881	Yes	4	-
3010	HPP0882	No	1	-
3011	HPP0886	No	1	-
3012	HPP0887	No	2	-
3013	HPP0888	No	1	-
3014	HPP0890	No	1	-
3015	HPP0891	No	1	-
3016	HPP0893	No	2	-
3017	HPP0895	No	1	-
3018	HPP0896	No	1	-
3019	HPP0897	No	1	-
3020	HPP0898	No	1	-
3021	HPP0900	No	1	-
3022	HPP0901	No	1	-
3023	HPP0902	No	1	-
3024	HPP0903	No	1	-
3025	HPP0906	No	1	-
3026	HPP0907	No	2	-
3027	HPP0908	No	1	Peer pressure
3028	HPP0909	No	2	-
3029	HPP0910	No	3	-
3030	HPP0911	No	1	-
3031	HPP0912	No	1	-
3032	HPP0913	No	1	-
3033	HPP0914	No	1	-
3034	HPP0915	No	1	-
3035	HPP0916	No	4	-
3036	HPP0918	No	2	-
3037	HPP0919	No	1	-
3038	HPP0920	No	1	-
3039	HPP0921	No	1	Job problem
3040	HPP0922	No	1	-

No	Patient ID	Other substances	No of substances abused at a time	Reason for substance use initiation
3041	HPP0927	No	1	-
3042	HPP0929	No	1	-
3043	HPP0930	No	3	Relationship
3044	HPP0932	No	1	-
3045	HPP0933	No	1	-
3046	HPP0934	No	1	-
3047	HPP0936	No	5	-
3048	HPP0937	No	2	-
3049	HPP0938	Yes	4	Joy seeking
3050	HPP0939	No	1	-
3051	HPP0940	No	1	-
3052	HPP0941	No	4	Peer pressure
3053	HPP0942	No	2	-
3054	HPP0943	No	1	-
3055	HPP0944	No	1	-
3056	HPP0945	No	2	-
3057	HPP0946	No	3	Relaxation
3058	HPP0947	Yes	4	-
3059	HPP0948	No	1	-
3060	HPP0949	No	1	-
3061	HPP0950	No	4	-
3062	HPP0952	No	1	Relationship
3063	HPP0953	No	1	-
3064	HPP0954	No	1	-
3065	HPP0955	No	3	Family issue
3066	HPP0956	No	1	-
3067	HPP0957	No	2	-
3068	HPP0958	No	1	-
3069	HPP0960	No	2	-
3070	HPP0961	No	2	Peer pressure
3071	HPP0962	No	3	-
3072	HPP0963	Yes	2	-
3073	HPP0964	No	2	-
3074	HPP0965	No	1	-
3075	HPP0966	No	2	-
3076	HPP0967	No	1	Family issue, Relationship
3077	HPP0968	No	2	-
3078	HPP0969	No	1	-
3079	HPP0970	No	1	Relaxation
3080	HPP0972	No	1	-
3081	HPP0973	No	1	-
3082	HPP0975	No	2	Relationship

No	Patient ID	Other substances	No of substances abused at a time	Reason for substance use initiation
3083	HPP0976	No	2	-
3084	HPP0977	No	2	Job problem
3085	HPP0978	No	2	-
3086	HPP0979	Yes	4	-
3087	HPP0980	No	1	-
3088	HPP0981	No	2	-
3089	HPP0982	No	1	-
3090	HPP0984	No	3	-
3091	HPP0986	Yes	5	-
3092	HPP0987	No	2	-
3093	HPP0988	No	3	-
3094	HPP0990	No	2	-
3095	HPP0991	No	4	-
3096	HPP0992	No	3	-
3097	HPP0993	No	1	-
3098	HPP0994	No	1	-
3099	HPP0996	No	4	-
3100	HPP0998	No	2	-
3101	HPP0999	No	1	-
3102	HPP1000	No	3	-
3103	HPP1001	No	2	-
3104	HPP1002	No	2	-
3105	HPP1003	No	2	-
3106	HPP1004	No	2	-
3107	HPP1005	No	1	-
3108	HPP1006	No	1	-
3109	HPP1007	No	1	-
3110	HPP1008	No	1	-
3111	HPP1009	No	3	-
3112	HPP1011	No	2	-
3113	HPP1012	No	2	-
3114	HPP1013	No	5	-
3115	HPP1014	No	3	-
3116	HPP1015	No	1	-
3117	HPP1016	No	3	-
3118	HPP1017	No	2	-
3119	HPP1018	No	1	Family issue, Job problem, Relationship
3120	HPP1019	No	2	-
3121	HPP1021	No	2	-
3122	HPP1022	No	2	-

No	Patient ID	Other substances	No of substances abused at a time	Reason for substance use initiation
3123	HPP1023	No	1	-
3124	HPP1024	No	3	-
3125	HPP1025	Yes	2	-
3126	HPP1027	No	1	-
3127	HPP1029	No	1	-
3128	HPP1031	No	2	-
3129	HPP1032	No	1	-
3130	HPP1033	No	1	-
3131	HPP1034	No	2	-
3132	HPP1035	No	2	-
3133	HPP1036	No	1	Relaxation, Joy seeking
3134	HPP1037	No	1	Others
3135	HPP1038	No	2	Peer pressure
3136	HPP1039	No	2	Job problem
3137	HPP1040	No	1	-
3138	HPP1041	No	1	-
3139	HPP1042	No	1	-
3140	HPP1043	No	1	-
3141	HPP1044	No	1	-
3142	HPP1047	No	3	-
3143	HPP1048	Yes	4	-
3144	HPP1049	No	3	-
3145	HPP1050	No	4	-
3146	HPP1052	No	1	-
3147	HPP1054	No	1	-
3148	HPP1055	No	1	-
3149	HPP1056	No	3	-
3150	HPP1057	No	1	-
3151	HPP1058	No	1	-
3152	HPP1059	No	2	-
3153	HPP1060	No	2	-
3154	HPP1062	No	1	-
3155	HPP1063	Yes	5	-
3156	HPP1064	No	1	-
3157	HPP1065	No	1	-
3158	HPP1067	No	1	-
3159	HPP1069	No	1	-
3160	HPP1070	No	2	-
3161	HPP1071	No	1	-
3162	HPP1073	No	3	-
3163	HPP1075	No	5	Peer pressure

No	Patient ID	Other substances	No of substances abused at a time	Reason for substance use initiation
3164	HPP1077	No	1	-
3165	HPP1079	No	2	-
3166	HPP1080	No	2	-
3167	HPP1081	No	2	Peer pressure
3168	HPP1082	No	1	-
3169	HPP1083	No	1	-
3170	HPP1084	Yes	5	-
3171	HPP1085	No	1	-
3172	HPP1086	No	1	Peer pressure
3173	HPP1087	No	1	-
3174	HPP1089	No	1	-
3175	HPP1091	No	2	-
3176	HPP1092	No	1	-
3177	HPP1094	No	2	-
3178	HPP1095	No	1	Relaxation
3179	HPP1096	No	1	-
3180	HPP1097	No	1	-
3181	HPP1098	No	1	-
3182	HPP1099	No	1	Job problem, Relationship, Relaxation
3183	HPP1100	No	2	-
3184	HPP1101	No	2	-
3185	HPP1102	No	1	-
3186	HPP1103	No	2	-
3187	HPP1104	No	1	-
3188	HPP1105	No	2	-
3189	HPP1106	No	1	-
3190	HPP1107	No	1	-
3191	HPP1108	No	2	-
3192	HPP1110	No	1	-
3193	HPP1111	No	1	-
3194	HPP1112	No	1	-
3195	HPP1113	No	2	-
3196	HPP1114	No	2	-
3197	HPP1115	No	1	-
3198	HPP1117	No	1	-
3199	HPP1118	No	3	-
3200	HPP1119	No	3	-
3201	HPP1120	No	1	-
3202	HPP1122	No	2	-
3203	HPP1123	No	1	-

No	Patient ID	Other substances	No of substances abused at a time	Reason for substance use initiation
3204	HPP1124	No	2	-
3205	HPP1125	No	2	-
3206	HPP1127	No	1	-
3207	HPP1128	No	2	-
3208	HPP1133	No	1	-
3209	HPP1134	No	1	-
3210	HPP1136	No	1	-
3211	HPP1137	No	3	Peer pressure
3212	HPP1139	No	2	-
3213	HPP1140	No	3	-
3214	HPP1143	No	1	-
3215	HPP1144	No	1	Family issue
3216	HPP1145	No	3	-
3217	HPP1146	No	2	-
3218	HPP1147	No	1	-
3219	HPP1149	No	2	-
3220	HPP1151	No	4	-
3221	HPP1155	No	1	-
3222	HPP11557	No	2	-
3223	HPP1156	No	1	-
3224	HPP1157	No	1	-
3225	HPP1160	No	3	-
3226	HPP1161	No	3	-
3227	HPP1164	No	1	-
3228	HPP1165	Yes	2	-
3229	HPP1166	No	2	-
3230	HPP1168	No	3	-
3231	HPP1169	No	2	-
3232	HPP1170	No	1	Job problem, Relaxation, Joy seeking
3233	HPP1171	Yes	5	-
3234	HPP1172	No	1	-
3235	HPP1173	No	1	Relaxation, Joy seeking
3236	HPP1174	Yes	4	-
3237	HPP1175	No	1	-
3238	HPP1176	No	3	Joy seeking
3239	HPP1177	No	1	-
3240	HPP1178	No	1	-
3241	HPP1179	No	2	Peer pressure

No	Patient ID	Other substances	No of substances abused at a time	Reason for substance use initiation
3242	HPP1180	No	1	-
3243	HPP1183	No	1	-
3244	HPP1185	No	1	-
3245	HPP1186	No	1	-
3246	HPP1187	No	1	-
3247	HPP1188	No	2	-
3248	HPP1189	No	3	Relationship
3249	HPP1190	No	2	-
3250	HPP1192	No	1	-
3251	HPP1195	No	2	-
3252	HPP1196	No	1	-
3253	HPP1198	No	1	-
3254	HPP1201	Yes	2	-
3255	HPP1202	No	2	-
3256	HPP1203	No	1	-
3257	HPP1205	No	1	Peer pressure
3258	HPP1206	Yes	2	-
3259	HPP1208	No	1	-
3260	HPP1209	No	1	-
3261	HPP1210	No	3	Family issue
3262	HPP1211	No	1	-
3263	HPP1213	No	1	-
3264	HPP1214	No	3	-
3265	HPP1218	No	1	-
3266	HPP1220	No	5	-
3267	HPP1222	No	2	Health, Peer pressure
3268	HPP1224	No	2	Job problem
3269	HPP1226	No	3	-
3270	HPP1228	No	1	-
3271	HPP1229	No	2	-
3272	HPP1230	No	1	-
3273	HPP1231	No	2	Relaxation
3274	HPP1232	No	1	Peer pressure
3275	HPP1233	No	2	-
3276	HPP1234	No	1	-
3277	HPP1236	No	1	Job problem, Joy seeking
3278	HPP1237	No	1	-
3279	HPP1238	No	1	-

No	Patient ID	Other substances	No of substances abused at a time	Reason for substance use initiation
3280	HPP1240	No	1	-
3281	HPP1241	No	2	-
3282	HPP1242	No	1	-
3283	HPP1243	No	2	-
3284	HPP1244	No	3	-
3285	HPP1245	No	4	-
3286	HPP1251	No	1	-
3287	HPP1252	No	1	-
3288	HPP1253	No	2	-
3289	HPP1256	No	2	-
3290	HPP1257	No	3	-
3291	HPP1259	No	3	Peer pressure
3292	HPP1260	No	2	-
3293	HPP1262	No	1	-
3294	HPP1265	No	2	Relationship, Peer pressure
3295	HPP1266	No	3	Relaxation, Peer pressure
3296	HPP1267	No	1	-
3297	HPP1268	No	4	Peer pressure
3298	HPP1270	No	1	-
3299	HPP1271	No	2	-
3300	HPP1273	No	1	-
3301	HPP1274	No	2	-
3302	HPP1275	No	1	-
3303	HPP1276	No	1	-
3304	HPP1277	No	1	-
3305	HPP1278	No	2	-
3306	HPP1279	No	2	-
3307	HPP1281	No	1	Relationship
3308	HPP1282	No	2	-
3309	HPP1283	No	2	-
3310	HPP1284	No	2	-
3311	HPP1285	No	1	-
3312	HPP1286	No	1	-
3313	HPP1288	No	1	-
3314	HPP1289	No	1	Joy seeking, Peer pressure
3315	HPP1290	No	1	-
3316	HPP1291	Yes	2	-
3317	HPP1292	No	1	-
3318	HPP1295	No	1	-
3319	HPP1296	No	3	-

No	Patient ID	Other substances	No of substances abused at a time	Reason for substance use initiation
3320	HPP1297	No	2	-
3321	HPP1298	No	1	-
3322	HPP1299	No	1	-
3323	HPP1300	No	1	-
3324	HPP1301	No	1	-
3325	HPP1302	No	1	-
3326	HPP1303	No	1	-
3327	HPP1304	No	2	Peer pressure
3328	HPP1305	No	1	-
3329	HPP1306	No	2	-
3330	HPP1307	No	1	-
3331	HPP1308	No	1	-
3332	HPP1309	No	1	-
3333	HPP1310	No	2	Family issue
3334	HPP1311	No	1	-
3335	HPP1312	No	1	-
3336	HPP1313	No	1	-
3337	HPP1314	No	1	-
3338	HPP1315	No	2	-
3339	HPP1318	No	1	-
3340	HPP1319	No	1	-
3341	HPP1320	No	1	-
3342	HPP1321	No	2	-
3343	HPP1324	No	1	-
3344	HPP1326	No	1	-
3345	HPP1327	Yes	4	Joy seeking, Peer pressure
3346	HPP1328	No	3	-
3347	HPP1329	No	1	-
3348	HPP1331	No	1	-
3349	HPP1332	No	5	-
3350	HPP1333	No	2	-
3351	HPP1334	No	1	Relationship
3352	HPP1335	No	1	-
3353	HPP1336	No	2	-
3354	HPP1337	No	1	-
3355	HPP1338	Yes	1	Relationship
3356	HPP1341	Yes	4	-

No	Patient ID	Other substances	No of substances abused at a time	Reason for substance use initiation
3357	HPP1343	No	1	-
3358	HPP1344	No	1	-
3359	HPP1345	No	1	-
3360	HPP1346	No	2	-
3361	HPP1347	No	1	-
3362	HPP1349	No	3	Family issue
3363	HPP1350	No	2	-
3364	HPP1352	No	3	Job problem, Relaxation
3365	HPP1354	No	2	-
3366	HPP1357	No	1	-
3367	HPP1358	No	2	-
3368	HPP1359	No	1	Relaxation, Joy seeking
3369	HPP1361	No	2	-
3370	HPP1362	No	1	-
3371	HPP1363	No	1	-
3372	HPP1364	No	1	-
3373	HPP1365	No	1	-
3374	HPP1367	No	2	-
3375	HPP1368	No	1	-
3376	HPP1369	No	1	-
3377	HPP1370	No	1	-
3378	HPP1372	No	1	-
3379	HPP1374	No	1	-
3380	HPP1375	No	2	-
3381	HPP1376	No	1	-
3382	HPP1377	No	1	-
3383	HPP1378	No	1	-
3384	HPP1379	No	1	-
3385	HPP1380	No	1	-
3386	HPP1381	No	2	-
3387	HPP1382	No	1	-
3388	HPP1384	No	1	-
3389	HPP1385	No	3	Family issue
3390	HPP1387	No	2	-
3391	HPP1388	No	1	-
3392	HPP1389	No	4	Peer pressure
3393	HPP1390	No	1	-
3394	HPP1391	No	3	-
3395	HPP1392	No	1	-

No	Patient ID	Other substances	No of substances abused at a time	Reason for substance use initiation
3396	HPP1394	Yes	3	Joy seeking
3397	HPP1395	No	5	-
3398	HPP1396	No	2	-
3399	HPP1397	No	2	-
3400	HPP1398	No	1	-
3401	HPP1399	No	1	-
3402	HPP1401	No	4	-
3403	HPP1402	No	2	-
3404	HPP1403	No	1	-
3405	HPP1404	No	1	-
3406	HPP1405	No	3	Relationship
3407	HPP1407	No	3	-
3408	HPP1408	No	2	-
3409	HPP1409	No	1	-
3410	HPP1411	No	4	Peer pressure
3411	HPP1412	No	1	-
3412	HPP1413	No	1	-
3413	HPP1414	No	1	-
3414	HPP1415	No	2	-
3415	HPP1416	No	2	-
3416	HPP1417	Yes	5	Peer pressure
3417	HPP1418	No	1	-
3418	HPP1419	No	2	-
3419	HPP1420	No	3	-
3420	HPP1421	No	2	-
3421	HPP1422	No	1	Peer pressure
3422	HPP1423	No	2	-
3423	HPP1424	No	1	-
3424	HPP1425	No	1	-
3425	HPP1426	No	1	-
3426	HPP1427	Yes	4	-
3427	HPP1429	No	2	-
3428	HPP1430	No	3	-
3429	HPP1431	No	2	-
3430	HPP1433	No	1	-
3431	HPP1435	No	1	-
3432	HPP1437	No	1	-
3433	HPP1438	No	4	-

No	Patient ID	Other substances	No of substances abused at a time	Reason for substance use initiation
3434	HPP1439	No	2	-
3435	HPP1441	No	3	-
3436	HPP1442	No	3	-
3437	HPP1443	No	2	-
3438	HPP1444	No	2	-
3439	HPP1445	No	1	-
3440	HPP1446	No	1	-
3441	HPP1447	No	1	-
3442	HPP1448	No	1	-
3443	HPP1449	No	1	-
3444	HPP1451	No	1	Relationship
3445	HPP1452	No	1	-
3446	HPP1453	No	2	Peer pressure
3447	HPP1454	No	1	-
3448	HPP1455	No	5	-
3449	HPP1456	No	1	-
3450	HPP1457	No	2	-
3451	HPP1460	No	2	-
3452	HPP1461	No	3	-
3453	HPP1462	No	7	-
3454	HPP1463	No	3	Peer pressure
3455	HPP1464	No	3	Peer pressure
3456	HPP1465	No	1	-
3457	HPP1466	No	2	-
3458	HPP1467	No	2	-
3459	HPP1468	No	1	-
3460	HPP1469	No	3	-
3461	HPP1470	Yes	4	-
3462	HPP1471	No	1	-
3463	HPP1473	No	1	-
3464	HPP1474	No	2	-
3465	HPP1475	No	2	-
3466	HPP1476	No	1	-
3467	HPP1477	No	2	-
3468	HPP1478	No	1	-
3469	HPP1479	No	4	-
3470	HPP1480	No	3	-
3471	HPP1481	No	1	-
3472	HPP1482	No	1	-
3473	HPP1483	No	2	-
3474	HPP1484	No	2	-
3475	HPP1485	No	5	-

No	Patient ID	Other substances	No of substances abused at a time	Reason for substance use initiation
3476	HPP1486	No	1	-
3477	HPP1487	No	1	-
3478	HPP1488	No	1	-
3479	HPP1489	No	1	-
3480	HPP1490	No	1	-
3481	HPP1491	No	2	-
3482	HPP1492	No	3	-
3483	HPP1494	No	1	-
3484	HPP1495	No	1	-
3485	HPP1497	No	1	-
3486	HPP1500	No	1	-
3487	HPP1501	No	2	-
3488	HPP1502	No	2	-
3489	HPP1503	No	2	Health
3490	HPP1504	No	3	-
3491	HPP1505	No	5	-
3492	HPP1506	No	2	-
3493	HPP1507	No	3	-
3494	HPP1510	No	1	-
3495	HPP1511	No	1	-
3496	HPP1512	No	2	-
3497	HPP1514	No	1	-
3498	HPP1515	No	1	-
3499	HPP1516	No	2	Relationship
3500	HPP1517	No	1	-
3501	HPP1518	No	1	-
3502	HPP1519	No	1	-
3503	HPP1521	No	1	-
3504	HPP1522	No	1	-
3505	HPP1523	No	2	-
3506	HPP1524	No	2	-
3507	HPP1526	No	1	-
3508	HPP1527	No	1	-
3509	HPP1530	No	1	-
3510	HPP1531	No	1	-
3511	HPP1532	No	1	Peer pressure
3512	HPP1533	No	1	-
3513	HPP1534	No	1	-
3514	HPP1535	No	2	-
3515	HPP1536	No	1	-

No	Patient ID	Other substances	No of substances abused at a time	Reason for substance use initiation
3516	HPP1538	No	2	-
3517	HPP1539	No	2	Peer pressure
3518	HPP1540	No	2	-
3519	HPP1541	Yes	7	-
3520	HPP1542	No	1	-
3521	HPP1543	No	1	-
3522	HPP1544	No	1	Family issue
3523	HPP1545	No	1	-
3524	HPP1546	No	1	-
3525	HPP1547	No	2	-
3526	HPP1548	No	1	-
3527	HPP1549	No	1	-
3528	HPP1550	No	2	-
3529	HPP1551	No	2	-
3530	HPP1552	No	3	-
3531	HPP1554	No	1	-
3532	HPP1555	No	1	-
3533	HPP1556	No	1	-
3534	HPP1558	No	3	-
3535	HPP1560	No	2	Peer pressure
3536	HPP1561	No	1	-
3537	HPP1562	No	1	-
3538	HPP1563	No	3	-
3539	HPP1564	No	2	-
3540	HPP1566	No	1	Relaxation, Joy seeking
3541	HPP1568	No	1	-
3542	HPP1569	No	1	-
3543	HPP1570	No	1	-
3544	HPP1571	No	1	-
3545	HPP1572	No	3	Peer pressure
3546	HPP1580	No	2	Peer pressure
3547	HPP1583	No	1	Peer pressure
3548	HPP1587	No	1	-
3549	HPP1590	No	1	Peer pressure
3550	HPP1597	No	3	Peer pressure
3551	HQE0013	No	2	Job problem, Relaxation
3552	HQE0014	No	2	Relationship

No	Patient ID	Other substances	No of substances abused at a time	Reason for substance use initiation
3553	HQE0017	No	1	Family issue
3554	HQE0022	No	3	-
3555	HQE0024	No	1	-
3556	HQE0025	No	1	-
3557	HQE0026	No	1	Health, Relationship
3558	HQE0027	No	1	-
3559	HQE0028	No	1	-
3560	HQE0029	No	3	Relationship, Peer pressure
3561	HQE0031	No	1	Relationship
3562	HQE0032	No	2	Health, Family issue, Relationship, Relaxation, Peer pressure
3563	HQE0033	No	2	Relaxation, Peer pressure, Others
3564	HQE0034	No	1	Relaxation, Peer pressure, Others
3565	HQE0035	No	1	-
3566	HQE0036	No	1	Relaxation, Peer pressure
3567	HQE0038	No	1	-
3568	HQE0039	No	1	-
3569	HQE0040	No	1	Peer pressure
3570	HQE0041	No	1	-
3571	HQE0042	No	2	Job problem
3572	HQE0043	No	2	-
3573	HQE0044	No	2	-
3574	HQE0045	No	2	-
3575	HQE0047	No	2	Relationship, Relaxation, Peer pressure
3576	HQE0051	No	3	Joy seeking
3577	HQE0053	No	4	Family issue, Relaxation, Others
3578	HQE0054	No	3	Relaxation
3579	HQE0055	No	3	Family issue, Job problem, Relaxation

No	Patient ID	Other substances	No of substances abused at a time	Reason for substance use initiation
3580	HQE0056	No	3	Health, Job problem, Joy seeking, Peer pressure
3581	HQE0057	No	2	-
3582	HQE0058	No	2	-
3583	HQE0059	No	2	-
3584	HQE0060	No	2	Relaxation, Peer pressure, Financial problem, Others
3585	HQE0061	No	2	Peer pressure
3586	HQE0062	No	1	Relaxation
3587	HQE0064	No	2	-
3588	HQE0065	No	3	Others
3589	HQE0066	No	2	-
3590	HQE0067	No	2	Joy seeking, Peer pressure
3591	HQE0068	No	2	Family issue, Relationship
3592	HQE0069	No	3	Relationship
3593	HQE0070	No	2	Relationship
3594	HQE0073	No	3	Job problem, Relaxation, Peer pressure
3595	HQE0075	No	2	Peer pressure
3596	HQE0076	No	2	Family issue
3597	HQE0077	No	2	Peer pressure
3598	HQE0078	No	2	Health
3599	HQE0079	No	2	Family issue
3600	HQE0080	No	2	Health
3601	HQE0081	No	2	-
3602	HQE0082	No	2	Family issue, Relationship, Relaxation, Peer pressure, Others
3603	HQE0083	No	2	-
3604	HQE0084	No	1	Health
3605	HQE0085	No	1	-

No	Patient ID	Other substances	No of substances abused at a time	Reason for substance use initiation
3606	HQE0086	No	4	Job problem
3607	HQE0087	No	2	-
3608	HQE0088	No	1	Joy seeking, Peer pressure
3609	HQE0089	No	2	Family issue
3610	HQE0091	No	1	-
3611	HQE0092	No	1	Health, Family issue
3612	HQE0093	No	3	Peer pressure
3613	HQE0094	No	3	Job problem, Relationship
3614	HQE0095	No	3	-
3615	HQE0096	No	2	Peer pressure
3616	HQE0097	No	3	Health, Family issue, Relaxation, Peer pressure
3617	HQE0098	No	3	Relationship
3618	HQE0099	No	2	Health
3619	HQE0100	No	2	-
3620	HQE0101	No	2	Health
3621	HQE0102	No	4	Health
3622	HQE0103	No	2	-
3623	HQE0104	No	2	Job problem, Financial problem
3624	HQE0106	No	2	Peer pressure
3625	HQE0107	No	2	Relationship
3626	HQE0108	No	3	-
3627	HQE0109	Yes	6	Health
3628	HQE0110	No	2	-
3629	HQE0111	No	2	Family issue, Relationship
3630	HQE0112	No	3	Family issue
3631	HQE0113	No	2	Health, Job problem
3632	HQE0114	No	2	-
3633	HQE0115	No	3	-
3634	HQE0116	No	2	Health, Peer pressure

No	Patient ID	Other substances	No of substances abused at a time	Reason for substance use initiation
3635	HQE0118	No	2	Job problem, Relaxation, Peer pressure
3636	HQE0119	No	1	Job problem, Relaxation, Peer pressure
3637	HQE0120	No	1	Family issue
3638	HQE0121	No	2	-
3639	HQE0122	No	1	Peer pressure
3640	HQE0122	No	2	Relaxation
3641	HQE0124	No	2	Relationship
3642	HQE0125	No	2	-
3643	HQE0126	No	2	-
3644	HQE0127	No	4	Peer pressure
3645	HQE0128	No	2	Relationship
3646	HQE0129	No	2	Joy seeking
3647	HQE0130	No	2	Family issue
3648	HQE0131	No	1	Relationship, Relaxation, Peer pressure
3649	HQE0133	No	2	-
3650	HQE0134	No	2	-
3651	HQE0135	No	2	-
3652	HQE0136	No	2	Health
3653	HQE0137	No	3	-
3654	HQE0143	No	2	-
3655	HQE0144	No	5	Family issue
3656	HQE0145	No	2	Family issue, Relationship
3657	HQE0146	No	1	Family issue
3658	HQE0147	No	2	Family issue
3659	HQE0148	No	1	Family issue
3660	HQE0149	No	2	Family issue, Relationship
3661	HQE0150	No	2	Family issue, Relationship
3662	HQE0151	No	1	-
3663	HQE0152	No	1	Health

No	Patient ID	Other substances	No of substances abused at a time	Reason for substance use initiation
3664	HQE0153	No	2	Family issue, Financial problem
3665	HQE0154	No	4	Peer pressure
3666	HQE0155	No	4	Peer pressure, Others
3667	HQE0156	No	1	Family issue
3668	HQE0157	No	2	Health, Financial problem, Others
3669	HQE0158	No	2	-
3670	HQE0160	No	2	Financial problem
3671	HQE0161	No	3	Family issue, Relationship
3672	HQE0162	No	2	Family issue
3673	HQE0163	No	2	Health
3674	HQE0165	No	2	Health
3675	HQE0166	No	1	Family issue
3676	HQE0167	Yes	5	Family issue, Relationship
3677	HQE0168	No	3	-
3678	HQE0169	No	4	-
3679	HQE0170	No	2	-
3680	HQE0171	No	3	Relationship
3681	HQE0172	No	3	Job problem
3682	HQE0174	No	2	Peer pressure
3683	HQE0175	No	3	-
3684	HQE0176	No	1	-
3685	HQE0177	No	3	Health, Family issue
3686	HQE0178	No	2	Family issue
3687	HQE0180	No	4	-
3688	HQE0181	No	1	-
3689	HQE0182	No	1	Peer pressure
3690	HQE0183	No	3	-
3691	HQE0184	No	2	Family issue, Relationship
3692	HQE0185	No	3	Family issue, Relationship
3693	HQE0186	No	2	Peer pressure
3694	HQE0188	No	3	Peer pressure
3695	HQE0189	No	3	Peer pressure

No	Patient ID	Other substances	No of substances abused at a time	Reason for substance use initiation
3696	HQE0190	No	1	Peer pressure
3697	HQE0191	No	3	Family issue
3698	HQE0192	No	3	-
3699	HQE0193	No	3	-
3700	HQE0195	No	2	Peer pressure
3701	HQE0196	No	2	Peer pressure
3702	HQE0197	No	3	Peer pressure
3703	HQE0198	No	2	Relationship
3704	HQE0199	No	3	Relationship, Peer pressure
3705	HQE0202	No	2	Peer pressure
3706	HQE0203	No	2	Job problem
3707	HQE0204	No	2	Peer pressure
3708	HQE0205	No	3	Peer pressure
3709	HQE0206	No	3	Peer pressure
3710	HQE0207	No	3	Peer pressure
3711	HQE0208	No	1	Family issue, Relationship
3712	HQE0209	No	2	Peer pressure
3713	HQE0210	No	3	Peer pressure
3714	HQE0211	No	3	-
3715	HQE0212	No	3	-
3716	HQE0214	No	3	-
3717	HQE0216	No	3	Peer pressure
3718	HQE0217	No	3	Family issue
3719	HQE0218	No	2	-
3720	HQE0219	No	3	Peer pressure
3721	HQE0220	No	2	Family issue, Relationship
3722	HQE0221	No	3	-
3723	HQE0224	No	2	Peer pressure
3724	HQE0225	No	3	Health, Family issue, Relationship
3725	HQE0226	No	1	-
3726	HQE0229	No	3	Peer pressure

No	Patient ID	Other substances	No of substances abused at a time	Reason for substance use initiation
3727	HQE0232	No	1	Family issue
3728	HQE0233	No	3	-
3729	HQE0234	No	1	Peer pressure
3730	HQE0235	No	3	Peer pressure
3731	HQE0236	No	2	Joy seeking
3732	HQE0237	No	2	-
3733	HQE0238	No	2	-
3734	HQE0240	No	2	-
3735	HQE0241	No	3	Family issue
3736	HQE0242	No	3	Peer pressure
3737	HQE0243	No	2	Health
3738	HQE0244	No	2	Others
3739	HQE0245	No	2	-
3740	HQE0248	No	3	Peer pressure
3741	HQE0251	No	2	Peer pressure
3742	HQE0252	No	3	Joy seeking, Peer pressure
3743	HQE0253	No	2	-
3744	HQE0255	No	3	Peer pressure
3745	HQE0256	No	2	Relationship
3746	HQE0257	No	2	Peer pressure
3747	HQE0258	No	3	Family issue, Peer pressure
3748	HQE0259	No	3	Family issue, Relationship, Peer pressure
3749	HQE0260	No	2	-
3750	HQE0262	No	1	Peer pressure
3751	HQE0263	No	1	-
3752	HQE0264	No	3	Peer pressure
3753	HQE0266	No	2	-
3754	HQE0268	No	2	-
3755	HQE0269	No	3	Peer pressure
3756	HQE0270	No	2	Family issue
3757	HQE0271	No	3	Joy seeking, Peer pressure
3758	HQE0272	No	3	Peer pressure

No	Patient ID	Other substances	No of substances abused at a time	Reason for substance use initiation
3759	HQE0273	No	3	Family issue
3760	HQE0274	No	3	-
3761	HQE0275	No	2	Peer pressure
3762	HQE0276	No	1	Health
3763	HQE0277	No	2	Family issue, Peer pressure
3764	HQE0278	No	2	Family issue, Relationship
3765	HQE0279	No	3	Peer pressure
3766	HQE0281	No	2	-
3767	HQE0282	No	2	Family issue
3768	HQE0283	No	1	Peer pressure
3769	HQE0285	No	2	Job problem
3770	HQE0286	No	3	Peer pressure
3771	HQE0287	No	2	Job problem
3772	HQE0289	No	2	-
3773	HQE0290	No	2	Health
3774	HQE0291	No	1	-
3775	HQE0292	No	2	Job problem, Financial problem
3776	HQE0293	No	2	-
3777	HQE0295	No	2	Family issue
3778	HQE0296	No	2	Financial problem
3779	HQE0299	No	2	Health
3780	HQE0300	No	2	-
3781	HQE0301	No	1	-
3782	HQE0303	No	2	Peer pressure
3783	HQE0304	No	2	-
3784	HQE0305	No	2	Peer pressure
3785	HQE0306	No	1	Relationship
3786	HQE0308	No	2	Family issue, Relationship
3787	HQE0309	No	1	Financial problem
3788	HQE0310	No	5	-
3789	HQE0311	No	2	Health, Job problem
3790	HQE0312	No	2	-

No	Patient ID	Other substances	No of substances abused at a time	Reason for substance use initiation
3791	HQE0314	No	1	-
3792	HQE0315	No	2	Family issue
3793	HQE0316	No	2	Family issue
3794	HQE0317	No	2	Relationship
3795	HQE0318	No	1	Relationship
3796	HQE0319	No	2	Family issue, Relationship
3797	HQE0321	No	1	Relationship
3798	HQE0322	No	2	Peer pressure
3799	HQE0324	No	2	Peer pressure
3800	HQE0325	No	1	Health
3801	HQE0328	No	1	Health
3802	HQE0331	No	4	Health, Family issue
3803	HQE0333	No	1	Family issue, Peer pressure
3804	HQE0335	No	2	Job problem
3805	HQE0336	No	2	-
3806	HQE0337	No	2	Job problem
3807	HQE0338	No	2	Job problem, Financial problem
3808	HQE0340	No	2	Relationship
3809	HQE0341	No	1	Family issue, Relationship, Financial problem
3810	HQE0343	No	2	Peer pressure
3811	HQE0344	No	3	Peer pressure
3812	HQE0345	No	2	Health
3813	HQE0348	No	1	Peer pressure
3814	HQE0349	No	2	Peer pressure
3815	HQE0350	No	2	Family issue, Relationship
3816	HQE0351	No	1	Family issue, Peer pressure
3817	HQE0352	No	2	Peer pressure
3818	HQE0353	No	2	Peer pressure
3819	HQE0354	No	3	Peer pressure
3820	HQE0355	No	2	Peer pressure
3821	HQE0356	No	2	Job problem
3822	HQE0358	No	1	Family issue

No	Patient ID	Other substances	No of substances abused at a time	Reason for substance use initiation
3823	HQE0359	No	1	Family issue
3824	HQE0361	No	1	-
3825	HQE0363	No	2	Relationship, Financial problem
3826	HQE0364	No	1	-
3827	HQE0365	No	2	-
3828	HQE0366	No	1	-
3829	HQE0367	No	2	-
3830	HQE0368	No	1	-
3831	HQE0369	No	1	-
3832	HQE0371	No	1	-
3833	HQE0374	No	2	-
3834	HQE0375	No	1	-
3835	HQE0382	No	1	-
3836	HQE0383	No	1	-
3837	HQE0385	No	1	-
3838	HQE0387	No	2	-
3839	HQE0388	No	2	-
3840	HQE0389	No	1	-
3841	HQE0390	No	1	-
3842	HQE0391	No	2	-
3843	HQE0392	No	2	-
3844	HQE0393	No	1	-
3845	HQE0394	No	2	Relationship
3846	HQE0395	No	2	-
3847	HQE0396	No	2	-
3848	HQE0397	No	1	-
3849	HQE0398	No	1	-
3850	HQE0399	No	1	-
3851	HQE0401	No	1	-
3852	HQE0402	No	2	-
3853	HQE0403	No	1	-
3854	HQE0404	No	1	-
3855	HQE0406	No	1	-
3856	HQE0407	No	1	-
3857	HQE0408	No	1	-
3858	HQE0409	No	1	-

No	Patient ID	Other substances	No of substances abused at a time	Reason for substance use initiation
3859	HQE0410	No	1	-
3860	HQE0412	No	1	-
3861	HQE0415	No	1	-
3862	HQE0416	No	1	-
3863	HQE0420	No	1	-
3864	HQE0421	No	2	Job problem, Financial problem
3865	HQE0423	No	2	Peer pressure
3866	HQE0424	No	2	-
3867	HQE0425	No	2	-
3868	HQE0426	No	1	-
3869	HQE0428	No	1	-
3870	HQE0429	No	2	Peer pressure
3871	HQE0430	No	1	-
3872	HQE0431	No	2	-
3873	HQE0432	No	1	-
3874	HQE0433	No	1	-
3875	HQE0434	No	2	-
3876	HQE0435	No	1	-
3877	HQE0436	No	1	-
3878	HQE0437	No	3	Peer pressure
3879	HQE0438	No	2	-
3880	HQE0439	No	4	Peer pressure
3881	HQE0440	No	2	-
3882	HQE0441	No	1	-
3883	HQE0442	No	2	-
3884	HQE0443	No	4	Peer pressure
3885	HQE0444	No	2	Family issue
3886	HQE0445	No	1	-
3887	HQE0446	No	4	Family issue
3888	HQE0447	No	2	-
3889	HQE0448	No	2	-
3890	HQE0449	No	2	-
3891	HQE0450	No	1	-

No	Patient ID	Other substances	No of substances abused at a time	Reason for substance use initiation
3892	HQE0451	No	3	Family issue
3893	HQE0452	No	3	-
3894	HQE0453	No	2	-
3895	HQE0455	No	1	-
3896	HQE0456	No	1	-
3897	HQE0457	No	1	-
3898	HQE0459	No	2	-
3899	HQE0460	No	1	-
3900	HQE0461	No	2	-
3901	HQE0462	No	1	Family issue
3902	HQE0463	No	1	-
3903	HQE0464	No	2	Relationship
3904	HQE0465	No	2	Joy seeking
3905	HQE0466	No	2	-
3906	HQE0467	No	2	-
3907	HQE0468	No	1	-
3908	HQE0469	No	1	Peer pressure
3909	HQE0470	No	2	-
3910	HQE0471	No	1	-
3911	HQE0472	No	2	Relationship
3912	HQE0473	No	2	-
3913	HQE0474	No	1	-
3914	HQE0475	No	1	-
3915	HQE0476	No	1	-
3916	HQE0477	No	1	-
3917	HQE0478	No	1	-
3918	HQE0479	No	1	-
3919	HQE0480	No	1	-
3920	HQE0481	No	2	-
3921	HQE0482	No	1	-
3922	HQE0483	No	2	-
3923	HQE0484	No	1	-
3924	HQE0485	No	1	-
3925	HQE0486	No	1	-
3926	HQE0487	No	1	-
3927	HQE0489	No	2	-
3928	HQE0492	No	1	-
3929	HQE0493	No	1	-
3930	HQE0494	No	2	Peer pressure
3931	HQE0495	No	1	-
3932	HQE0496	No	1	Family issue

No	Patient ID	Other substances	No of substances abused at a time	Reason for substance use initiation
3933	HQE0497	No	1	-
3934	HQE0498	No	2	-
3935	HQE0499	No	1	-
3936	HQE0500	No	1	-
3937	HQE0501	No	3	Peer pressure
3938	HQE0503	No	1	-
3939	HQE0504	No	2	-
3940	HQE0506	No	1	-
3941	HQE0507	No	2	-
3942	HQE0508	No	1	-
3943	HQE0509	No	2	-
3944	HQE0511	No	1	-
3945	HQE0512	No	1	-
3946	HQE0513	No	1	-
3947	HQE0515	No	2	-
3948	HQE0516	No	3	Peer pressure
3949	HQE0517	No	2	-
3950	HQE0518	No	1	Peer pressure
3951	HQE0520	No	2	-
3952	HQE0523	No	1	-
3953	HQE0524	No	2	-
3954	HQE0525	No	1	-
3955	HQE0526	No	2	-
3956	HQE0527	No	1	-
3957	HQE0528	No	1	-
3958	HQE0529	No	2	Peer pressure
3959	HQE0530	No	3	Peer pressure
3960	HQE0531	No	1	-
3961	HQE0532	No	2	Peer pressure
3962	HQE0533	No	2	-
3963	HQE0534	No	2	-
3964	HQE0535	No	1	Peer pressure
3965	HQE0536	No	1	-

No	Patient ID	Other substances	No of substances abused at a time	Reason for substance use initiation
3966	HQE0537	No	2	Peer pressure
3967	HQE0538	No	2	Peer pressure
3968	HQE0539	No	1	-
3969	HQE0540	No	2	-
3970	HQE0541	No	3	Peer pressure
3971	HQE0542	No	2	Family issue
3972	HQE0543	No	1	-
3973	HQE0544	No	1	-
3974	HQE0545	No	1	-
3975	HQE0546	No	2	Peer pressure
3976	HQE0547	No	1	Peer pressure
3977	HQE0548	No	1	-
3978	HQE0549	No	2	Family issue, Peer pressure, Financial problem
3979	HQE0549	No	2	-
3980	HQE0550	No	3	Peer pressure
3981	HQE0551	No	2	Peer pressure
3982	HQE0552	No	2	-
3983	HQE0553	No	2	-
3984	HQE0554	No	2	-
3985	HQE0555	No	1	-
3986	HQE0556	No	3	Peer pressure
3987	HQE0557	No	2	-
3988	HQE0558	No	1	-
3989	HQE0559	No	2	-
3990	HQE0560	No	2	Peer pressure
3991	HQE0561	No	2	Peer pressure
3992	HQE0562	No	2	-
3993	HQE0563	No	3	Peer pressure
3994	HQE0566	No	3	Peer pressure
3995	HQE0567	No	3	Peer pressure
3996	HQE0570	No	2	Peer pressure
3997	HQE0571	No	1	Peer pressure
3998	HQE0573	No	2	Peer pressure
3999	HQE0574	No	1	-
4000	HQE0575	No	3	Peer pressure

No	Patient ID	Other substances	No of substances abused at a time	Reason for substance use initiation
4001	HQE0576	No	2	Peer pressure
4002	HQE0577	No	1	-
4003	HQE0579	No	1	Peer pressure
4004	HQE0580	No	3	Peer pressure
4005	HQE0581	No	2	-
4006	HQE0583	No	1	Peer pressure
4007	HQE0584	No	3	Peer pressure
4008	HQE0586	No	1	Peer pressure
4009	HQE0587	No	1	-
4010	HQE0588	No	2	-
4011	HQE0590	No	2	Peer pressure
4012	HQE0591	No	2	Peer pressure
4013	HQE0592	No	1	-
4014	HQE0593	No	1	-
4015	HQE0594	No	3	Peer pressure
4016	HQE0595	No	1	-
4017	HQE0596	No	1	-
4018	HQE0598	No	1	Peer pressure
4019	HQE0599	No	2	-
4020	HQE0600	No	3	Peer pressure
4021	HQE0601	No	1	-
4022	HQE0602	No	3	Financial problem
4023	HQE0603	No	1	Peer pressure
4024	HQE0604	No	2	-
4025	HQE0605	No	2	Peer pressure
4026	HQE0606	No	1	-
4027	HQE0607	No	1	Peer pressure
4028	HQE0608	No	1	-
4029	HQE0610	No	2	-
4030	HQE0611	No	1	Peer pressure
4031	HQE0612	No	2	Peer pressure
4032	HQE0613	No	2	-
4033	HQE0614	No	2	Peer pressure
4034	HQE0615	No	3	Peer pressure
4035	HQE0616	No	2	Peer pressure
4036	HQE0617	No	2	-
4037	HQE0619	No	1	Peer pressure

No	Patient ID	Other substances	No of substances abused at a time	Reason for substance use initiation
4038	HQE0620	No	3	Peer pressure
4039	HQE0621	No	1	-
4040	HQE0622	No	1	-
4041	HQE0624	No	1	-
4042	HQE0625	No	2	-
4043	HQE0626	No	2	-
4044	HQE0627	No	1	-
4045	HQE0628	No	1	-
4046	HQE0630	No	3	Peer pressure
4047	HQE0631	No	2	-
4048	HQE0632	No	2	-
4049	HQE0633	No	1	-
4050	HQE0634	No	3	Peer pressure
4051	HQE0635	No	1	-
4052	HQE0636	No	2	-
4053	HQE0637	No	2	Peer pressure
4054	HQE0639	No	3	Peer pressure
4055	HQE0640	No	2	Peer pressure
4056	HQE0641	No	1	-
4057	HQE0642	No	2	-
4058	HQE0643	No	1	-
4059	HQE0644	No	1	-
4060	HQE0645	No	2	-
4061	HQE0646	No	1	-
4062	HQE0647	No	2	-
4063	HQE0648	No	3	Peer pressure
4064	HQE0649	No	1	-
4065	HQE0650	No	2	-
4066	HQE0652	No	2	-
4067	HQE0653	No	1	-
4068	HQE0654	No	1	-
4069	HQE0655	Yes	3	Peer pressure
4070	HQE0656	No	3	Peer pressure
4071	HQE0657	No	1	-

No	Patient ID	Other substances	No of substances abused at a time	Reason for substance use initiation
4072	HQE0658	No	2	Peer pressure
4073	HQE0659	No	2	-
4074	HQE0661	No	1	Peer pressure
4075	HQE0662	No	1	-
4076	HQE0663	No	1	-
4077	HQE0665	No	2	-
4078	HQE0666	No	2	-
4079	HQE0667	No	1	Others
4080	HQE0668	No	1	-
4081	HQE0669	No	2	Family issue
4082	HQE0670	No	1	-
4083	HQE0671	No	2	Relaxation
4084	HQE0672	No	1	-
4085	HQE0673	No	3	Peer pressure
4086	HQE0674	No	1	-
4087	HQE0675	No	2	-
4088	HQE0676	No	3	Peer pressure
4089	HQE0678	No	2	-
4090	HQE0679	No	2	-
4091	HQE0680	No	3	Peer pressure
4092	HQE0681	No	1	-
4093	HQE0682	No	3	Peer pressure
4094	HQE0683	No	3	Peer pressure
4095	HQE0684	No	2	Peer pressure
4096	HQE0686	No	4	Peer pressure
4097	HQE0687	No	2	Peer pressure
4098	HQE0689	No	1	-
4099	HQE0690	No	2	-
4100	HQE0693	No	2	Peer pressure

No	Patient ID	Other substances	No of substances abused at a time	Reason for substance use initiation
4101	HQE0694	No	2	-
4102	HQE0696	No	2	Peer pressure
4103	HRPB0001	No	2	Peer pressure
4104	HRPB0002	No	3	Peer pressure
4105	HRPB0004	No	1	-
4106	HRPB0005	No	2	Peer pressure
4107	HRPB0006	No	2	-
4108	HRPB0007	No	2	-
4109	HRPB0011	No	2	Family issue, Peer pressure
4110	HRPB0012	No	1	-
4111	HRPB0013	No	2	Relaxation, Joy seeking, Peer pressure
4112	HRPB0014	No	2	Family issue
4113	HRPB0015	No	2	Peer pressure
4114	HRPB0016	No	1	-
4115	HRPB0017	No	1	-
4116	HRPB0018	No	2	-
4117	HRPB0019	No	3	Peer pressure
4118	HRPB0020	No	1	Peer pressure
4119	HRPB0021	No	2	-
4120	HRPB0022	No	1	-
4121	HRPB0023	No	1	-
4122	HRPB0024	No	1	Family issue, Job problem, Financial problem

No	Patient ID	Other substances	No of substances abused at a time	Reason for substance use initiation
4123	HRPB0025	No	2	-
4124	HRPB0026	No	2	-
4125	HRPB0027	No	2	-
4126	HRPB0028	No	1	-
4127	HRPB0030	No	1	Health
4128	HRPB0031	No	2	-
4129	HRPB0032	No	1	-
4130	HRPB0033	No	2	-
4131	HRPB0035	No	2	-
4132	HRPB0037	No	4	Relaxation
4133	HRPB0038	No	1	-
4134	HRPB0039	No	1	-
4135	HRPB0041	No	3	Peer pressure
4136	HRPB0042	No	3	Peer pressure
4137	HRPB0043	No	1	Peer pressure
4138	HRPB0044	No	1	Others
4139	HRPB0045	No	1	Job problem
4140	HRPB0046	No	1	Peer pressure
4141	HRPB0047	Yes	1	Others
4142	HRPB0048	No	2	-
4143	HRPB0051	No	2	-

No	Patient ID	Other substances	No of substances abused at a time	Reason for substance use initiation
4144	HRPB0052	No	2	-
4145	HRPB0053	No	3	-
4146	HRPB0054	No	1	-
4147	HRPB0055	No	3	-
4148	HRPB0059	No	2	Relationship, Relaxation
4149	HRPB0060	No	1	-
4150	HRPB0061	No	1	Relaxation
4151	HRPB0062	No	1	Peer pressure
4152	HRPB0063	No	1	-
4153	HRPB0064	No	1	-
4154	HRPB0065	No	1	-
4155	HRPB0068	No	2	-
4156	HRPB0070	No	1	Health, Relationship, Peer pressure
4157	HRPB0071	No	1	-
4158	HRPB0073	No	2	-
4159	HRPB0074	No	1	-
4160	HRPB0075	No	3	-
4161	HRPB0077	No	1	Peer pressure
4162	HRPB0079	No	2	Peer pressure
4163	HRPB0080	No	1	-
4164	HRPB0083	No	2	-

No	Patient ID	Other substances	No of substances abused at a time	Reason for substance use initiation
4165	HRPB0085	No	1	Peer pressure
4166	HRPB0086	No	1	-
4167	HRPB0092	No	2	-
4168	HRPB0093	No	1	-
4169	HRPB0094	No	1	Peer pressure
4170	HRPB0095	No	2	Family issue, Financial problem
4171	HRPB0096	No	2	-
4172	HRPB0097	No	2	Peer pressure
4173	HRPB0098	No	3	-
4174	HRPB0099	No	1	-
4175	HRPB0104	No	2	-
4176	HRPB0106	No	1	Peer pressure
4177	HRPB0107	No	1	Peer pressure
4178	HRPB0108	No	1	-
4179	HRPB0109	No	1	-
4180	HRPB0110	No	1	-
4181	HRPB0111	No	5	-
4182	HRPB0112	No	3	Peer pressure
4183	HRPB0113	No	1	Peer pressure
4184	HRPB0115	No	2	Job problem, Joy seeking, Peer pressure
4185	HRPB0116	No	2	-

No	Patient ID	Other substances	No of substances abused at a time	Reason for substance use initiation
4186	HRPB0117	No	1	-
4187	HRPB0118	No	2	-
4188	HRPB0119	No	1	-
4189	HRPB0120	No	2	Peer pressure
4190	HRPB0121	No	1	Peer pressure
4191	HRPB0122	No	2	-
4192	HRPB0123	No	1	-
4193	HRPB0124	No	2	-
4194	HRPB0125	No	1	-
4195	HRPB0126	No	1	-
4196	HRPB0127	No	1	-
4197	HRPB0128	No	2	Job problem, Peer pressure
4198	HRPB0129	No	1	Peer pressure
4199	HRPB0131	No	3	Peer pressure
4200	HRPB0132	No	1	Financial problem
4201	HRPB0133	No	2	Peer pressure
4202	HRPB0134	No	1	Relaxation
4203	HRPB0136	No	2	Peer pressure
4204	HRPB0137	No	2	Peer pressure
4205	HRPB0138	No	2	Peer pressure
4206	HRPB0139	No	2	-

No	Patient ID	Other substances	No of substances abused at a time	Reason for substance use initiation
4207	HRPB0141	No	2	-
4208	HRPB0142	No	1	Peer pressure
4209	HRPB0143	No	2	Joy seeking
4210	HRPB0144	No	2	Family issue
4211	HRPB0145	No	2	-
4212	HRPB0146	No	3	Peer pressure
4213	HRPB0147	No	1	Peer pressure
4214	HRPB0148	No	2	Others
4215	HRPB0149	No	1	-
4216	HRPB0150	No	1	-
4217	HRPB0151	No	1	-
4218	HRPB0152	No	2	-
4219	HRPB0153	No	2	Peer pressure
4220	HRPB0154	No	1	Peer pressure
4221	HRPB0156	No	1	-
4222	HRPB0157	No	3	Joy seeking
4223	HRPB0158	No	3	Peer pressure
4224	HRPB0159	No	2	Peer pressure
4225	HRPB0161	No	1	-
4226	HRPB0162	No	1	-
4227	HRPB0163	No	1	-

No	Patient ID	Other substances	No of substances abused at a time	Reason for substance use initiation
4228	HRPB0164	No	1	-
4229	HRPB0167	No	1	-
4230	HRPB0168	No	1	-
4231	HRPB0169	No	2	-
4232	HRPB0170	No	2	-
4233	HRPB0171	No	1	-
4234	HRPB0173	No	3	-
4235	HRPB0174	No	2	-
4236	HRPB0177	No	3	-
4237	HRPB0178	No	2	-
4238	HRPB0179	No	1	Relationship
4239	HRPB0184	No	3	-
4240	HRPB0185	No	1	-
4241	HRPB0187	No	1	-
4242	HRPB0189	No	1	-
4243	HRPB0191	No	1	Health
4244	HRPB0192	No	1	-
4245	HRPB0194	No	1	-
4246	HRPB0195	No	1	-
4247	HRPB0197	No	1	-
4248	HRPB0200	No	3	Peer pressure

No	Patient ID	Other substances	No of substances abused at a time	Reason for substance use initiation
4249	HRPB0202	No	1	-
4250	HRPB0203	No	2	-
4251	HRPB0204	No	1	-
4252	HRPB0207	No	1	-
4253	HRPB0209	No	2	-
4254	HRPB0210	No	2	-
4255	HRPB0211	No	1	Family issue, Financial problem
4256	HRPB0212	No	2	-
4257	HRPB0213	No	2	Others
4258	HRPB0214	No	2	-
4259	HRPB0215	No	2	-
4260	HRPB0216	No	1	Job problem
4261	HRPB0217	No	2	Peer pressure
4262	HRPB0218	No	2	-
4263	HRPB0219	No	1	-
4264	HRPB0220	No	2	-
4265	HRPB0221	No	1	Peer pressure
4266	HRPB0223	No	1	Health
4267	HRPB0224	No	2	-
4268	HRPB0225	No	1	Job problem
4269	HRPB0226	No	2	-

No	Patient ID	Other substances	No of substances abused at a time	Reason for substance use initiation
4270	HRPB0227	No	3	-
4271	HRPB0228	No	2	-
4272	HRPB0229	No	1	-
4273	HRPB0230	No	1	-
4274	HRPB0231	No	1	-
4275	HRPB0232	No	2	-
4276	HRPB0233	No	1	-
4277	HRPB0234	No	2	-
4278	HRPB0235	No	1	-
4279	HRPB0237	No	3	Peer pressure
4280	HRPB0238	No	2	Peer pressure
4281	HRPB0239	No	2	-
4282	HRPB0240	No	1	-
4283	HRPB0241	No	2	-
4284	HRPB0242	No	2	Relaxation
4285	HRPB0243	No	1	Peer pressure
4286	HRPB0244	No	3	-
4287	HRPB0246	No	1	-
4288	HRPB0248	No	1	-
4289	HRPB0250	No	2	-
4290	HRPB0251	No	1	Peer pressure

No	Patient ID	Other substances	No of substances abused at a time	Reason for substance use initiation
4291	HRPB0252	No	2	-
4292	HRPB0253	No	2	Peer pressure
4293	HRPB0254	No	1	-
4294	HRPB0255	No	4	-
4295	HRPB0256	No	3	-
4296	HRPB0257	No	1	Peer pressure
4297	HRPB0258	No	3	-
4298	HRPB0260	No	1	-
4299	HRPB0261	No	1	-
4300	HRPB0262	No	2	-
4301	HRPB0263	No	2	Job problem
4302	HRPB0264	No	2	-
4303	HRPB0265	No	1	Family issue
4304	HRPB0266	No	2	-
4305	HRPB0267	No	1	-
4306	HRPB0268	No	1	-
4307	HRPB0269	No	1	-
4308	HRPB0270	No	4	-
4309	HRPB0271	No	1	-
4310	HRPB0272	No	1	-
4311	HRPB0273	No	1	-

No	Patient ID	Other substances	No of substances abused at a time	Reason for substance use initiation
4312	HRPB0274	No	1	-
4313	HRPB0275	No	2	-
4314	HRPB0276	No	1	-
4315	HRPB0277	No	1	-
4316	HRPB0278	No	3	Peer pressure
4317	HRPB0279	No	1	-
4318	HRPB0280	Yes	2	-
4319	HRPB0281	No	3	-
4320	HRPB0282	No	2	-
4321	HRPB0283	No	1	Peer pressure
4322	HRPB0284	No	1	-
4323	HRPB0285	No	2	-
4324	HRPB0291	No	1	Peer pressure
4325	HRPB0292	No	2	-
4326	HRPB0295	No	2	-
4327	HRPB0296	No	1	-
4328	HRPB0297	No	1	-
4329	HRPB0298	No	2	-
4330	HRPB0300	No	2	-
4331	HRPB0302	No	1	-
4332	HRPB0303	No	2	Peer pressure

No	Patient ID	Other substances	No of substances abused at a time	Reason for substance use initiation
4333	HRPB0304	No	1	-
4334	HRPB0305	No	1	-
4335	HRPB0306	No	2	Peer pressure
4336	HRPB0307	No	2	Peer pressure
4337	HRPB0308	No	2	Peer pressure
4338	HRPB0309	No	2	-
4339	HRPB0312	No	2	-
4340	HRPB0313	No	1	-
4341	HRPB0314	No	1	-
4342	HRPB0315	No	1	-
4343	HRPB0316	No	3	-
4344	HRPB0318	No	2	-
4345	HRPB0319	No	1	-
4346	HRPB0320	No	1	-
4347	HRPB0321	No	2	-
4348	HRPB0322	No	1	Relaxation
4349	HRPB0323	No	2	-
4350	HRPB0324	No	1	Peer pressure
4351	HRPB0325	No	1	Peer pressure
4352	HRPB0326	No	1	-
4353	HRPB0327	No	2	Family issue

No	Patient ID	Other substances	No of substances abused at a time	Reason for substance use initiation
4354	HRPB0328	No	1	Peer pressure
4355	HRPB0329	No	2	-
4356	HRPB0330	No	2	-
4357	HRPB0331	No	2	Peer pressure
4358	HRPB0332	No	1	-
4359	HRPB0333	No	1	-
4360	HRPB0335	No	3	-
4361	HRPB0336	No	1	-
4362	HRPB0337	No	1	-
4363	HRPB0338	No	1	-
4364	HRPB0340	No	1	-
4365	HRPB0342	No	3	-
4366	HRPB0344	No	2	-
4367	HRPB0346	No	3	-
4368	HRPB0348	No	1	Others
4369	HRPB0349	No	1	-
4370	HRPB0358	No	2	Peer pressure
4371	HRPB0359	No	1	Peer pressure
4372	HRPB0360	No	2	-
4373	HRPB0361	No	2	Family issue
4374	HRPB0362	No	1	-

No	Patient ID	Other substances	No of substances abused at a time	Reason for substance use initiation
4375	HRPB0363	No	2	Peer pressure
4376	HRPB0364	No	1	-
4377	HRPB0365	No	2	-
4378	HRPB0366	Yes	4	-
4379	HRPB0367	No	1	-
4380	HRPB0368	No	1	Health
4381	HRPB0369	No	2	-
4382	HRPB0370	No	1	-
4383	HRPB0371	No	1	Health
4384	HRPB0372	No	2	-
4385	HRPB0373	No	2	Job problem
4386	HRPB0374	No	2	-
4387	HRPB0375	No	2	-
4388	HRPB0378	No	1	-
4389	HRPB0383	No	1	-
4390	HRPB0386	No	3	Peer pressure
4391	HRPB0399	No	2	-
4392	HRPB0400	No	1	-
4393	HRPB0406	No	2	Health
4394	HRPB0407	No	4	Peer pressure
4395	HRPB0409	No	2	-

No	Patient ID	Other substances	No of substances abused at a time	Reason for substance use initiation
4396	HRPB0410	No	1	-
4397	HRPB0411	No	1	-
4398	HRPB0412	No	2	-
4399	HRPB0413	No	2	-
4400	HRPB0414	No	2	-
4401	HRPB0416	No	1	-
4402	HRPB0417	No	1	-
4403	HRPB0418	No	4	Peer pressure
4404	HRPB0419	No	1	-
4405	HRPB0420	No	2	-
4406	HRPB0421	No	2	-
4407	HRPB0422	No	2	-
4408	HRPB0423	No	1	Peer pressure
4409	HRPB0424	No	2	Relaxation
4410	HRPB0425	No	2	Peer pressure
4411	HRPB0426	No	1	-
4412	HRPB0427	No	1	-
4413	HRPB0428	No	1	-
4414	HRPB0429	No	1	-
4415	HRPB0430	No	1	-
4416	HRPB0431	No	1	-

No	Patient ID	Other substances	No of substances abused at a time	Reason for substance use initiation
4417	HRPB0436	No	1	-
4418	HRPB0437	No	1	Peer pressure
4419	HRPB0438	No	2	Peer pressure
4420	HRPB0439	No	3	Peer pressure
4421	HRPB0440	No	2	Job problem
4422	HRPB0441	No	2	-
4423	HRPB0442	No	2	-
4424	HRPB0443	No	3	-
4425	HRPB0444	No	2	-
4426	HRPB0445	No	1	Relationship
4427	HRPB0446	No	1	-
4428	HRPB0447	No	2	-
4429	HRPB0449	No	4	-
4430	HRPB0450	No	3	-
4431	HRPB0451	No	1	-
4432	HRPB0452	No	1	-
4433	HRPB0453	No	3	-
4434	HRPB0454	No	1	-
4435	HRPB0455	No	1	Peer pressure
4436	HRPB0456	No	1	-
4437	HRPB0457	No	1	-

No	Patient ID	Other substances	No of substances abused at a time	Reason for substance use initiation
4438	HRPB0458	No	1	-
4439	HRPB0460	No	1	Health
4440	HRPB0462	No	2	-
4441	HRPB0463	No	2	Peer pressure
4442	HRPB0464	No	1	-
4443	HRPB0466	No	3	-
4444	HRPB0467	No	2	-
4445	HRPB0469	No	1	-
4446	HRPB0470	No	2	-
4447	HRPB0471	No	1	-
4448	HRPB0472	No	2	-
4449	HRPB0473	No	1	-
4450	HRPB0474	No	1	-
4451	HRPB0475	No	2	Peer pressure
4452	HRPB0476	No	2	-
4453	HRPB0479	No	2	-
4454	HRPB0480	No	1	-
4455	HRPB0481	No	2	Health
4456	HRPB0482	No	1	-
4457	HRPB0484	No	3	Family issue
4458	HRPB0485	No	1	Relaxation

No	Patient ID	Other substances	No of substances abused at a time	Reason for substance use initiation
4459	HRPB0486	No	3	-
4460	HRPB0487	No	1	-
4461	HRPB0488	No	1	Joy seeking
4462	HRPB0490	No	1	-
4463	HRPB0491	No	1	-
4464	HRPB0492	No	1	Peer pressure
4465	HRPB0493	No	1	-
4466	HRPB0494	No	1	-
4467	HRPB0495	No	1	-
4468	HRPB0496	Yes	4	-
4469	HRPB0498	No	1	-
4470	HRPB0499	No	2	-
4471	HRPB0500	No	4	-
4472	HRPB0501	No	1	-
4473	HRPB0502	No	1	-
4474	HRPB0503	No	2	Health
4475	HRPB0504	No	2	-
4476	HRPB0507	No	1	-
4477	HRPB0509	No	1	-
4478	HRPB0510	No	1	-
4479	HRPB0511	No	2	-

No	Patient ID	Other substances	No of substances abused at a time	Reason for substance use initiation
4480	HRPB0512	No	2	-
4481	HRPB0513	No	2	-
4482	HRPB0514	No	1	-
4483	HRPB0515	No	1	-
4484	HRPB0516	Yes	2	-
4485	HRPB0517	No	1	Peer pressure
4486	HRPB0518	No	1	Peer pressure
4487	HRPB0519	Yes	2	-
4488	HRPB0520	No	1	-
4489	HRPB0521	No	3	-
4490	HRPB0522	No	1	Peer pressure
4491	HRPB0523	No	1	-
4492	HRPB0524	No	1	Family issue
4493	HRPB0525	No	3	-
4494	HRPB0526	No	1	-
4495	HRPB0527	No	3	-
4496	HRPB0529	No	2	-
4497	HRPB0530	No	1	-
4498	HRPB0532	No	2	Peer pressure
4499	HRPB0533	No	1	-
4500	HRPB0534	No	2	-

No	Patient ID	Other substances	No of substances abused at a time	Reason for substance use initiation
4501	HRPB0536	No	1	-
4502	HRPB0538	No	1	-
4503	HRPB0539	No	2	-
4504	HRPB0542	No	1	-
4505	HRPB0555	No	3	Peer pressure
4506	HRPB0559	No	1	-
4507	HRPB0560	No	1	-
4508	HRPB0562	No	1	-
4509	HRPB0563	No	1	-
4510	HRPB0564	No	1	-
4511	HRPB0565	No	1	Job problem
4512	HRPB0566	No	1	-
4513	HRPB0567	No	1	-
4514	HRPB0568	No	1	-
4515	HRPB0569	No	2	Relaxation
4516	HRPB0570	No	1	-
4517	HRPB0572	No	1	Peer pressure
4518	HRPB0573	No	1	-
4519	HRPB0574	No	1	-
4520	HRPB0575	No	1	-
4521	HRPB0576	No	1	-

No	Patient ID	Other substances	No of substances abused at a time	Reason for substance use initiation
4522	HRPB0577	No	1	-
4523	HRPB0578	No	2	-
4524	HRPB0579	No	2	-
4525	HRPB0580	No	2	-
4526	HRPB0581	No	1	-
4527	HRPB0582	No	2	-
4528	HRPB0583	No	2	-
4529	HRPB0584	No	5	-
4530	HRPB0585	No	1	-
4531	HRPB0586	No	1	-
4532	HRPB0594	No	1	-
4533	HRPB0595	No	1	-
4534	HRPB0599	No	1	-
4535	HRPB0600	No	2	Peer pressure
4536	HRPB0601	No	2	-
4537	HRPB0603	No	2	-
4538	HRPB0604	No	2	-
4539	HRPB0605	No	2	Health
4540	HRPB0612	No	1	-
4541	HRPB0613	No	2	-
4542	HRPB0614	No	1	-

No	Patient ID	Other substances	No of substances abused at a time	Reason for substance use initiation
4543	HRPB0615	No	1	-
4544	HRPB0617	No	2	-
4545	HRPB0618	No	3	-
4546	HRPB0619	No	1	-
4547	HRPB0620	No	2	-
4548	HRPB0621	No	1	-
4549	HRPB0622	No	1	-
4550	HRPB0623	No	2	-
4551	HRPB0624	No	2	-
4552	HRPB0625	No	1	-
4553	HRPB0627	No	1	-
4554	HRPB0628	No	2	-
4555	HRPB0629	No	2	-
4556	HRPB0630	No	2	-
4557	HRPB0631	No	1	-
4558	HRPB0632	No	2	-
4559	HRPB0633	No	2	-
4560	HRPB0634	No	1	-
4561	HRPB0635	No	1	-
4562	HRPB0638	No	1	-
4563	HRPB0639	No	1	-

No	Patient ID	Other substances	No of substances abused at a time	Reason for substance use initiation
4564	HRPB0640	No	1	-
4565	HRPB0642	No	1	-
4566	HRPB0643	No	1	-
4567	HRPB0645	No	1	-
4568	HRPB0676	No	2	Peer pressure
4569	HRPB0677	No	1	-
4570	HRPB0687	No	3	-
4571	HRPB0689	No	3	-
4572	HRPB0704	No	1	-
4573	HRPB0707	No	2	Peer pressure, Others
4574	HRPB0712	No	2	Peer pressure, Others
4575	HRPB0724	No	2	Peer pressure, Others
4576	HRPB0730	No	1	Peer pressure, Others
4577	HRPB0731	No	2	-
4578	HRPB0739	No	2	Peer pressure
4579	HRPB0740	No	1	-
4580	HRPB0741	No	4	-
4581	HRPB0746	No	1	-
4582	HRPB0747	No	1	-
4583	HRPB0751	No	1	-
4584	HRPB0752	No	2	-

No	Patient ID	Other substances	No of substances abused at a time	Reason for substance use initiation
4585	HRPB0753	No	1	-
4586	HRPB0754	No	1	Peer pressure
4587	HRPB0755	No	2	Peer pressure, Others
4588	HRPB0761	No	2	-
4589	HRPB0763	No	2	Peer pressure
4590	HSA0001	No	1	Relationship
4591	HSA0002	No	1	Relationship
4592	HSA0004	No	1	-
4593	HSA0005	No	2	-
4594	HSA0006	No	1	Relaxation
4595	HSA0007	No	1	-
4596	HSA0008	No	1	Health
4597	HSA0009	No	5	-
4598	HSA0010	No	1	Peer pressure
4599	HSA0011	No	1	Joy seeking
4600	HSA0012	No	3	Peer pressure
4601	HSA0013	No	2	-
4602	HSA0014	No	1	-
4603	HSA0016	No	1	-
4604	HSA0019	Yes	3	Peer pressure
4605	HSA0020	No	1	-
4606	HSA0022	No	1	-
4607	HSA0023	No	3	Relaxation
4608	HSA0024	No	1	-
4609	HSA0025	No	2	Joy seeking
4610	HSA0027	No	1	-
4611	HSA0029	No	1	-
4612	HSA0030	Yes	3	Peer pressure
4613	HSA0031	No	2	-
4614	HSA0032	No	2	-
4615	HSA0033	No	3	Relaxation
4616	HSA0034	No	2	Relaxation

No	Patient ID	Other substances	No of substances abused at a time	Reason for substance use initiation
4617	HSA0036	No	1	-
4618	HSA0037	No	3	Job problem
4619	HSA0039	No	2	Relaxation
4620	HSA0040	No	3	-
4621	HSA0041	No	1	-
4622	HSA0042	No	1	-
4623	HSA0043	No	3	-
4624	HSA0044	No	2	Relaxation
4625	HSA0045	Yes	2	-
4626	HSA0047	No	3	-
4627	HSA0048	No	1	-
4628	HSA0049	No	1	-
4629	HSA0052	No	1	-
4630	HSA0053	No	1	-
4631	HSA0055	No	3	Financial problem
4632	HSA0057	No	1	Relaxation
4633	HSA0058	No	1	-
4634	HSA0059	No	1	Family issue
4635	HSA0060	No	1	Relaxation
4636	HSA0061	No	2	-
4637	HSA0062	No	1	-
4638	HSA0063	No	1	Joy seeking
4639	HSA0064	No	1	-
4640	HSA0065	No	2	Peer pressure
4641	HSA0066	No	2	-
4642	HSA0067	Yes	3	Joy seeking
4643	HSA0068	No	2	Others
4644	HSA0069	No	2	-
4645	HSA0070	No	2	Relaxation
4646	HSA0071	No	1	-
4647	HSA0072	No	2	Health
4648	HSA0073	No	3	Relaxation
4649	HSA0074	No	1	-

No	Patient ID	Other substances	No of substances abused at a time	Reason for substance use initiation
4650	HSA0075	No	1	-
4651	HSA0077	No	1	-
4652	HSA0078	No	1	-
4653	HSA0079	No	1	-
4654	HSA0080	No	2	-
4655	HSA0081	Yes	2	-
4656	HSA0082	No	1	-
4657	HSA0083	No	3	-
4658	HSA0084	No	2	-
4659	HSA0085	No	2	-
4660	HSA0086	No	2	-
4661	HSA0087	No	2	-
4662	HSA0088	No	1	-
4663	HSA0089	No	2	-
4664	HSA0090	No	1	-
4665	HSA0091	Yes	3	-
4666	HSA0092	No	1	-
4667	HSA0093	No	2	-
4668	HSA0094	No	1	-
4669	HSA0095	No	1	-
4670	HSA0097	No	3	Joy seeking
4671	HSA0098	No	1	-
4672	HSA0099	No	2	-
4673	HSA0100	No	2	-
4674	HSA0102	No	1	-
4675	HSA0103	No	3	-
4676	HSA0104	Yes	4	Peer pressure
4677	HSA0105	No	2	-
4678	HSA0106	No	1	-
4679	HSA0107	No	2	Family issue, Relaxation
4680	HSA0108	No	2	Relaxation, Joy seeking

No	Patient ID	Other substances	No of substances abused at a time	Reason for substance use initiation
4681	HSA0109	No	1	-
4682	HSA0110	No	3	Relationship
4683	HSA0111	No	2	Relaxation, Peer pressure
4684	HSA0112	No	2	-
4685	HSA0113	No	2	-
4686	HSA0116	No	1	-
4687	HSA0117	No	1	-
4688	HSA0118	No	1	-
4689	HSA0119	No	1	-
4690	HSA0120	No	1	-
4691	HSA0121	No	1	-
4692	HSA0122	No	1	-
4693	HSA0123	No	1	-
4694	HSA0124	No	2	-
4695	HSA0126	No	3	Health
4696	HSA0127	No	4	Health
4697	HSA0128	No	2	-
4698	HSA0129	No	1	-
4699	HSA0130	No	4	-
4700	HSA0131	No	2	-
4701	HSA0132	No	1	Relaxation
4702	HSA0133	No	1	-
4703	HSA0134	No	2	Job problem
4704	HSA0135	Yes	1	-
4705	HSA0136	No	1	Job problem
4706	HSA0137	No	1	-
4707	HSA0138	No	2	-
4708	HSA0139	No	1	Joy seeking
4709	HSA0140	No	1	-
4710	HSA0142	No	1	-
4711	HSA0143	No	1	Family issue
4712	HSA0144	No	1	Others
4713	HSA0145	No	2	-
4714	HSA0146	No	1	-
4715	HSA0147	No	1	-
4716	HSA0148	No	2	-

No	Patient ID	Other substances	No of substances abused at a time	Reason for substance use initiation
4717	HSA0150	No	2	-
4718	HSA0151	No	2	-
4719	HSA0152	No	2	-
4720	HSA0154	No	3	-
4721	HSA0155	No	2	-
4722	HSA0156	No	2	-
4723	HSA0158	Yes	2	-
4724	HSA0159	No	3	-
4725	HSA0160	No	2	-
4726	HSA0161	No	1	-
4727	HSA0162	No	2	-
4728	HSA0163	No	3	-
4729	HSA0164	No	1	-
4730	HSA0165	No	1	-
4731	HSA0166	No	2	-
4732	HSA0167	No	2	-
4733	HSA0168	No	1	-
4734	HSA0169	No	3	Peer pressure
4735	HSA0170	No	2	-
4736	HSA0171	No	2	Joy seeking, Peer pressure
4737	HSA0172	No	2	-
4738	HSA0173	No	2	-
4739	HSA0174	No	2	Others
4740	HSA0175	No	1	-
4741	HSA0176	No	1	Relationship
4742	HSA0177	No	5	-
4743	HSA0178	No	2	-
4744	HSA0179	No	1	Family issue
4745	HSA0180	No	2	-
4746	HSA0181	No	1	-
4747	HSA0182	No	1	-

No	Patient ID	Other substances	No of substances abused at a time	Reason for substance use initiation
4748	HSA0183	No	1	-
4749	HSA0184	No	1	-
4750	HSA0185	No	1	-
4751	HSA0187	No	1	Health
4752	HSA0188	No	2	-
4753	HSA0189	No	3	Others
4754	HSA0190	No	1	-
4755	HSA0191	No	2	-
4756	HSA0192	No	2	-
4757	HSA0193	No	2	-
4758	HSA0194	No	2	-
4759	HSA0195	No	1	-
4760	HSA0196	No	2	-
4761	HSA0197	No	1	Health
4762	HSA0198	No	1	-
4763	HSA0199	No	2	-
4764	HSA0200	No	3	-
4765	HSA0201	No	1	Health, Joy seeking
4766	HSA0202	No	2	-
4767	HSA0203	No	2	-
4768	HSA0204	Yes	3	-
4769	HSA0207	No	2	-
4770	HSA0208	No	2	-
4771	HSA0209	No	2	-
4772	HSA0210	No	2	Relationship
4773	HSA0211	No	2	-
4774	HSA0212	No	1	-
4775	HSA0213	No	1	-
4776	HSA0214	No	1	-
4777	HSA0215	No	3	Peer pressure
4778	HSA0216	No	2	-
4779	HSA0217	No	1	-

No	Patient ID	Other substances	No of substances abused at a time	Reason for substance use initiation
4780	HSA0218	No	2	Health
4781	HSA0219	No	1	Relationship
4782	HSA0220	Yes	3	Peer pressure
4783	HSA0221	Yes	5	Relationship
4784	HSA0222	No	2	-
4785	HSA0223	No	1	Health
4786	HSA0224	No	1	-
4787	HSA0225	No	1	-
4788	HSA0226	No	2	-
4789	HSA0227	No	1	-
4790	HSA0228	No	1	-
4791	HSA0230	No	2	-
4792	HSA0231	No	1	-
4793	HSA0232	No	2	-
4794	HSA0233	No	2	Relaxation
4795	HSA0234	No	1	-
4796	HSA0235	No	1	Joy seeking
4797	HSA0236	No	2	Others
4798	HSA0237	Yes	1	-
4799	HSA0238	No	1	-
4800	HSA0239	No	1	-
4801	HSA0240	No	2	-
4802	HSA0241	No	1	Health
4803	HSA0242	No	1	-
4804	HSA0243	No	2	Relaxation, Joy seeking
4805	HSA0244	No	2	-
4806	HSA0245	No	2	Peer pressure
4807	HSA0246	No	1	Relationship
4808	HSA0247	No	1	-
4809	HSA0248	Yes	1	Others
4810	HSA0249	No	1	-
4811	HSA0250	Yes	3	-
4812	HSA0251	Yes	1	Peer pressure
4813	HSA0252	No	4	Health

No	Patient ID	Other substances	No of substances abused at a time	Reason for substance use initiation
4814	HSA0253	No	1	-
4815	HSA0254	No	2	-
4816	HSA0255	No	2	-
4817	HSA0256	No	2	Family issue
4818	HSA0257	No	1	Others
4819	HSA0258	No	2	-
4820	HSA0259	No	2	Family issue
4821	HSA0260	No	2	Peer pressure
4822	HSA0261	No	2	-
4823	HSA0262	No	2	-
4824	HSA0263	No	1	-
4825	HSA0264	No	2	-
4826	HSA0265	No	1	Health
4827	HSA0266	Yes	3	Joy seeking
4828	HSA0267	No	1	Family issue
4829	HSA0268	No	3	-
4830	HSA0269	No	1	Joy seeking
4831	HSA0270	No	2	-
4832	HSA0271	No	1	-
4833	HSA0272	No	1	-
4834	HSA0273	No	1	-
4835	HSA0274	Yes	3	-
4836	HSA0275	No	1	-
4837	HSA0276	No	2	-
4838	HSA0277	No	1	Peer pressure
4839	HSA0278	No	2	-
4840	HSA0279	No	2	-
4841	HSA0280	No	1	-
4842	HSA0281	No	1	-
4843	HSA0282	No	4	-
4844	HSA0283	Yes	2	-
4845	HSA0284	No	1	Job problem

No	Patient ID	Other substances	No of substances abused at a time	Reason for substance use initiation
4846	HSA0285	No	2	Others
4847	HSA0286	No	2	-
4848	HSA0287	Yes	3	-
4849	HSA0288	No	2	Peer pressure
4850	HSA0289	No	2	Peer pressure
4851	HSA0290	No	2	-
4852	HSA0291	Yes	1	-
4853	HSA0292	No	2	-
4854	HSA0293	No	2	-
4855	HSA0294	No	2	-
4856	HSA0295	No	1	-
4857	HSA0296	No	3	-
4858	HSA0297	No	1	Job problem, Peer pressure
4859	HSA0298	No	2	-
4860	HSA0299	No	3	-
4861	HSA0300	No	1	-
4862	HSA0301	No	1	-
4863	HSA0302	No	1	-
4864	HSA0303	No	3	-
4865	HSA0304	No	1	-
4866	HSA0305	No	2	-
4867	HSA0306	No	2	-
4868	HSA0307	No	1	-
4869	HSA0308	No	2	-
4870	HSA0309	No	1	-
4871	HSA0310	No	1	-

No	Patient ID	Other substances	No of substances abused at a time	Reason for substance use initiation
4872	HSA0311	No	1	-
4873	HSA0312	No	1	Health
4874	HSA0313	Yes	3	Peer pressure
4875	HSA0314	No	1	-
4876	HSA0315	No	2	Job problem
4877	HSA0316	No	2	-
4878	HSA0317	No	1	-
4879	HSA0318	No	1	Others
4880	HSA0319	No	1	-
4881	HSA0320	No	1	-
4882	HSA0321	No	1	-
4883	HSA0322	No	2	-
4884	HSA0323	No	2	Others
4885	HSA0324	No	1	Others
4886	HSA0325	No	2	-
4887	HSA0326	No	1	-
4888	HSA0327	Yes	4	-
4889	HSA0328	No	1	-
4890	HSA0329	No	2	Peer pressure, Others
4891	HSA0330	No	2	-
4892	HSA0331	No	2	-
4893	HSA0332	No	1	Job problem
4894	HSA0333	No	2	-
4895	HSA0334	No	2	-
4896	HSA0335	No	3	-
4897	HSA0336	No	2	-
4898	HSA0337	No	1	-
4899	HSA0338	No	2	-
4900	HSA0339	No	2	-

No	Patient ID	Other substances	No of substances abused at a time	Reason for substance use initiation
4901	HSA0340	No	1	-
4902	HSA0341	No	2	-
4903	HSA0342	No	1	Others
4904	HSA0343	No	3	Peer pressure
4905	HSA0344	Yes	3	-
4906	HSA0345	No	2	Relationship
4907	HSA0346	No	1	-
4908	HSA0347	No	1	-
4909	HSA0348	No	2	-
4910	HSA0349	No	1	-
4911	HSA0350	No	1	-
4912	HSA0351	No	2	-
4913	HSA0352	No	3	-
4914	HSA0353	No	1	-
4915	HSA0354	No	2	Job problem
4916	HSA0355	Yes	1	-
4917	HSA0356	No	3	Peer pressure
4918	HSA0357	No	1	-
4919	HSA0358	No	1	-
4920	HSA0359	No	2	Peer pressure
4921	HSA0360	No	1	-
4922	HSA0361	No	2	Joy seeking, Others
4923	HSA0362	No	1	Job problem
4924	HSA0363	No	1	-
4925	HSA0364	No	3	-
4926	HSA0365	No	1	-
4927	HSA0366	Yes	3	-
4928	HSA0367	No	3	-

No	Patient ID	Other substances	No of substances abused at a time	Reason for substance use initiation
4929	HSA0368	No	1	Peer pressure
4930	HSA0369	No	1	-
4931	HSA0370	No	1	Others
4932	HSA0371	No	1	-
4933	HSA0372	No	2	-
4934	HSA0373	No	2	-
4935	HSA0374	No	1	-
4936	HSA0375	No	1	-
4937	HSA0376	No	2	Peer pressure
4938	HSA0377	Yes	2	-
4939	HSA0378	No	1	-
4940	HSA0379	Yes	3	Peer pressure
4941	HSA0380	No	1	-
4942	HSA0381	No	3	-
4943	HSA0382	No	1	-
4944	HSA0383	No	2	-
4945	HSA0384	No	1	-
4946	HSA0385	No	1	-
4947	HSA0386	No	1	-
4948	HSA0387	Yes	1	-
4949	HSA0388	No	2	-
4950	HSA0389	No	2	-
4951	HSA0390	No	3	Joy seeking
4952	HSA0391	No	2	Peer pressure
4953	HSA0392	No	3	-
4954	HSA0393	Yes	4	-
4955	HSA0394	No	1	-
4956	HSA0395	No	1	-

No	Patient ID	Other substances	No of substances abused at a time	Reason for substance use initiation
4957	HSA0396	No	1	Job problem
4958	HSA0397	No	2	-
4959	HSA0398	No	2	-
4960	HSA0399	No	1	Peer pressure
4961	HSA0400	No	3	-
4962	HSA0401	No	4	Relaxation, Others
4963	HSA0403	No	2	-
4964	HSA0404	No	3	Peer pressure
4965	HSA0405	No	2	-
4966	HSA0406	No	2	-
4967	HSA0407	No	2	-
4968	HSA0408	No	1	-
4969	HSA0409	No	3	-
4970	HSA0410	No	2	-
4971	HSA0411	No	2	-
4972	HSA0412	No	1	-
4973	HSA0413	No	3	Others
4974	HSA0414	No	1	-
4975	HSA0415	Yes	3	-
4976	HSA0416	No	1	-
4977	HSA0417	No	3	Health
4978	HSA0418	No	1	Relationship
4979	HSA0419	No	2	-
4980	HSA0420	Yes	3	Peer pressure
4981	HSA0421	No	1	-
4982	HSA0422	No	3	Peer pressure
4983	HSA0423	No	2	-
4984	HSA0424	No	2	-
4985	HSA0425	No	1	Others
4986	HSA0426	No	1	-
4987	HSA0427	No	1	-
4988	HSA0428	No	2	Job problem

No	Patient ID	Other substances	No of substances abused at a time	Reason for substance use initiation
4989	HSA0429	Yes	3	-
4990	HSA0430	No	1	-
4991	HSA0431	No	2	-
4992	HSA0432	No	2	-
4993	HSA0433	No	2	-
4994	HSA0434	No	3	-
4995	HSA0435	No	3	-
4996	HSA0436	No	1	-
4997	HSA0437	No	2	Peer pressure
4998	HSA0438	Yes	2	-
4999	HSA0439	No	2	-
5000	HSA0440	Yes	3	Relationship
5001	HSA0441	No	1	-
5002	HSA0442	No	2	-
5003	HSA0443	No	1	Others
5004	HSA0444	No	2	-
5005	HSA0445	No	1	-
5006	HSA0446	No	1	-
5007	HSA0447	No	2	-
5008	HSA0448	No	2	Joy seeking
5009	HSA0449	No	1	-
5010	HSA0450	No	1	-
5011	HSA0451	No	2	-
5012	HSA0452	No	1	Joy seeking
5013	HSA0453	No	1	-
5014	HSA0454	No	1	-
5015	HSA0455	No	2	-
5016	HSA0456	No	2	-
5017	HSA0457	No	1	-

No	Patient ID	Other substances	No of substances abused at a time	Reason for substance use initiation
5018	HSA0458	No	1	-
5019	HSA0459	No	4	Peer pressure
5020	HSA0460	No	2	-
5021	HSA0461	No	2	Family issue
5022	HSA0463	No	2	-
5023	HSA0463	No	2	-
5024	HSA0464	No	1	-
5025	HSA0465	Yes	3	-
5026	HSA0466	No	1	-
5027	HSA0467	No	2	-
5028	HSA0468	No	2	-
5029	HSA0469	No	1	-
5030	HSA0470	No	3	-
5031	HSA0471	No	1	-
5032	HSA0472	No	1	-
5033	HSA0473	Yes	3	-
5034	HSA0474	No	1	Peer pressure
5035	HSA0475	No	3	-
5036	HSA0476	No	3	-
5037	HSA0477	No	2	Joy seeking
5038	HSA0478	No	1	-
5039	HSA0479	No	2	Peer pressure
5040	HSA0480	No	3	Relaxation
5041	HSA0481	No	1	Others
5042	HSA0482	No	2	-
5043	HSA0483	No	1	-
5044	HSA0484	Yes	3	-
5045	HSA0485	No	2	-
5046	HSA0486	No	2	-
5047	HSA0487	No	1	-
5048	HSA0488	No	2	-
5049	HSA0489	No	1	-
5050	HSA0490	No	2	-
5051	HSA0491	No	1	-
5052	HSA0492	No	3	-
5053	HSA0493	No	1	-

No	Patient ID	Other substances	No of substances abused at a time	Reason for substance use initiation
5054	HSA0494	No	2	-
5055	HSA0495	No	1	-
5056	HSA0496	No	2	Peer pressure
5057	HSA0497	No	3	-
5058	HSA0498	No	2	-
5059	HSA0499	No	3	-
5060	HSA0500	No	2	-
5061	HSA0501	No	1	Joy seeking
5062	HSA0502	No	2	-
5063	HSA0503	No	1	-
5064	HSA0504	No	2	Others
5065	HSA0505	No	1	Others
5066	HSA0506	No	2	Others
5067	HSA0507	No	2	-
5068	HSA0508	No	2	-
5069	HSA0509	No	2	-
5070	HSA0510	Yes	2	-
5071	HSA0511	No	2	-
5072	HSA0512	No	1	-
5073	HSA0513	No	1	Others
5074	HSA0514	No	1	Others
5075	HSA0515	No	1	Others
5076	HSA0516	No	1	-
5077	HSA0517	No	1	-
5078	HSA0518	Yes	4	-
5079	HSA0519	No	1	-
5080	HSA0520	No	1	-
5081	HSA0521	No	1	Others

No	Patient ID	Other substances	No of substances abused at a time	Reason for substance use initiation
5082	HSA0522	No	2	-
5083	HSA0523	No	1	Others
5084	HSA0524	No	3	Relationship
5085	HSA0525	No	2	Others
5086	HSA0526	No	2	-
5087	HSA0527	No	1	Others
5088	HSA0528	No	3	Peer pressure
5089	HSA0529	No	2	Others
5090	HSA0530	No	1	-
5091	HSA0531	No	1	-
5092	HSA0532	No	1	-
5093	HSA0533	No	1	Others
5094	HSA0534	No	2	Others
5095	HSA0535	No	1	-
5096	HSA0536	No	1	Others
5097	HSA0537	No	2	Others
5098	HSA0538	No	1	-
5099	HSA0539	No	1	Others
5100	HSA0540	No	2	Others
5101	HSA0541	No	1	Others
5102	HSA0542	No	2	Others
5103	HSA0543	No	1	Others
5104	HSA0544	Yes	2	-
5105	HSA0545	No	1	Others
5106	HSA0546	No	1	Others
5107	HSA0547	No	1	Others
5108	HSA0548	No	2	Others

No	Patient ID	Other substances	No of substances abused at a time	Reason for substance use initiation
5109	HSA0549	No	2	Others
5110	HSA0550	No	2	Others
5111	HSA0551	No	1	Others
5112	HSA0552	No	1	-
5113	HSA0553	No	2	Others
5114	HSA0554	No	2	Peer pressure
5115	HSA0555	No	1	Others
5117	HSA0556	No	1	Others
5116	HSA0556	No	2	-
5118	HSA0557	No	2	Others
5119	HSA0558	No	1	Others
5120	HSA0559	No	1	Others
5121	HSA0560	No	2	-
5122	HSA0561	No	1	Others
5123	HSA0562	No	1	-
5124	HSA0563	No	2	-
5125	HSA0564	No	2	-
5126	HSA0565	No	2	Peer pressure
5127	HSA0567	No	1	Others
5128	HSA0568	No	1	-
5129	HSA0569	No	1	Others
5130	HSA0570	No	1	-
5131	HSA0571	No	1	Others
5132	HSA0572	No	2	Others
5133	HSA0573	No	2	Others
5134	HSA0574	No	3	Others
5135	HSA0575	No	2	Others
5136	HSA0576	No	1	-
5137	HSA0577	No	2	Others

No	Patient ID	Other substances	No of substances abused at a time	Reason for substance use initiation
5138	HSA0578	No	1	-
5139	HSA0579	No	1	Others
5140	HSA0580	No	1	Others
5141	HSA0582	No	1	Others
5142	HSA0583	No	1	Others
5143	HSA0584	No	1	Others
5144	HSA0585	Yes	1	-
5145	HSA0586	No	1	Others
5146	HSA0587	No	2	Joy seeking
5147	HSA0588	No	1	Others
5148	HSA0589	No	2	Others
5149	HSA0590	No	3	-
5150	HSA0591	No	3	Joy seeking, Others
5151	HSA0592	No	1	Others
5152	HSA0593	No	2	-
5153	HSA0594	No	1	Others
5154	HSA0595	No	2	Others
5155	HSA0597	No	1	-
5156	HSA0598	No	1	-
5157	HSA0599	No	1	-
5158	HSA0600	No	1	Others
5159	HSA0601	No	1	-
5160	HSA0602	No	1	Others
5161	HSA0603	No	1	Others
5162	HSA0604	No	1	Others
5163	HSA0605	No	1	Others
5164	HSA0606	No	1	Peer pressure
5165	HSA0607	No	4	-
5166	HSA0608	Yes	2	-
5167	HSA0609	No	2	-
5168	HSA0610	No	1	-
5169	HSA0611	No	2	Relationship

No	Patient ID	Other substances	No of substances abused at a time	Reason for substance use initiation
5170	HSA0612	No	3	Peer pressure
5171	HSA0613	No	2	-
5172	HSA0614	No	2	-
5173	HSA0615	No	1	-
5174	HSA0616	No	2	Relaxation
5175	HSA0617	No	3	-
5176	HSA0618	No	1	-
5177	HSA0619	No	2	-
5178	HSA0620	No	1	-
5179	HSA0621	No	3	-
5180	HSA0622	No	3	Joy seeking
5181	HSA0623	No	3	-
5182	HSA0624	No	1	-
5183	HSA0625	No	1	-
5184	HSA0626	No	2	-
5185	HSA0627	No	1	-
5186	HSA0628	No	1	-
5187	HSA0629	No	1	-
5188	HSA0630	No	2	Relaxation
5189	HSA0632	No	1	-
5190	HSA0633	No	2	-
5191	HSA0634	No	2	-
5192	HSA0635	No	1	-
5193	HSA0636	No	1	-
5194	HSA0637	No	1	-
5195	HSA0638	No	1	-
5196	HSA0639	No	1	-
5197	HSA0640	No	2	-
5198	HSA0641	No	4	-
5199	HSA0642	No	1	-
5200	HSA0643	No	2	-
5201	HSA0644	No	1	-
5202	HSA0645	No	1	-
5203	HSA0646	No	2	-

No	Patient ID	Other substances	No of substances abused at a time	Reason for substance use initiation
5204	HSA0647	No	1	-
5205	HSA0648	No	1	-
5206	HSA0649	Yes	2	-
5207	HSA0650	No	1	Peer pressure
5208	HSA0651	No	2	-
5209	HSA0652	No	2	-
5210	HSA0653	No	1	-
5211	HSA0654	No	1	-
5212	HSA0655	No	2	-
5213	HSA0657	No	1	-
5214	HSA0658	No	1	-
5215	HSA0659	No	1	-
5216	HSA0660	No	2	-
5217	HSA0661	No	2	-
5218	HSA0662	No	3	-
5219	HSA0663	No	1	-
5220	HSA0664	No	1	-
5221	HSA0665	No	1	-
5222	HSA0666	No	1	-
5223	HSA0667	No	1	-
5224	HSA0668	No	1	-
5225	HSA0669	No	1	-
5226	HSA0670	No	1	-
5227	HSA0671	No	3	-
5228	HSA0672	No	2	-
5229	HSA0673	No	1	-
5230	HSA0674	No	3	Peer pressure
5231	HSA0675	No	2	-
5232	HSA0676	No	1	-
5233	HSA0677	No	2	-

No	Patient ID	Other substances	No of substances abused at a time	Reason for substance use initiation
5234	HSA0678	No	2	-
5235	HSA0679	No	2	-
5236	HSA0680	No	1	-
5237	HSA0681	No	2	-
5238	HSA0682	No	1	Peer pressure
5239	HSA0683	No	1	-
5240	HSA0684	No	1	-
5241	HSA0685	No	1	-
5242	HSA0686	No	4	Peer pressure
5243	HSA0687	No	1	-
5244	HSA0688	No	2	Peer pressure
5245	HSA0689	No	2	-
5246	HSA0690	Yes	3	Peer pressure
5247	HSA0691	No	2	-
5248	HSA0692	No	2	Peer pressure
5249	HSA0693	No	2	-
5250	HSA0694	No	2	-
5251	HSA0695	No	2	-
5252	HSA0696	No	1	-
5253	HSA0697	No	2	Peer pressure
5254	HSA0698	No	1	-
5255	HSA0699	No	4	-
5256	HSA0700	No	1	-
5257	HSA0701	No	1	Relationship
5258	HSA0702	No	3	-
5259	HSA0703	No	1	-
5260	HSA0704	No	2	Peer pressure
5261	HSA0705	No	2	-
5262	HSA0706	No	4	-
5263	HSA0707	No	1	-
5264	HSA0708	No	2	-
5265	HSA0709	No	2	-

No	Patient ID	Other substances	No of substances abused at a time	Reason for substance use initiation
5266	HSA0710	No	2	Peer pressure
5267	HSA0711	No	1	-
5268	HSA0712	No	2	Peer pressure
5269	HSA0713	No	1	-
5270	HSA0714	No	1	-
5271	HSA0715	No	1	-
5272	HSA0716	No	2	Peer pressure
5273	HSA0717	No	1	-
5274	HSA0718	No	4	Peer pressure
5275	HSA0719	No	3	-
5276	HSA0720	No	2	-
5277	HSA0721	No	3	-
5278	HSA0722	No	2	Peer pressure
5279	HSA0723	No	1	-
5280	HSA0724	No	2	Peer pressure
5281	HSA0725	No	1	-
5282	HSA0726	No	2	-
5283	HSA0727	No	1	-
5284	HSA0728	No	3	Peer pressure
5285	HSA0729	No	2	-
5286	HSA0730	No	1	-
5287	HSA0731	No	3	Peer pressure
5288	HSA0732	No	2	-
5289	HSA0733	No	1	-
5290	HSA0734	No	2	-
5291	HSA0735	No	1	-
5292	HSA0736	No	1	-
5293	HSA0737	No	1	-
5294	HSA0738	No	1	-
5295	HSA0739	No	1	-
5296	HSA0740	No	3	Relaxation
5297	HSA0741	No	2	Others
5298	HSA0742	No	2	-

No	Patient ID	Other substances	No of substances abused at a time	Reason for substance use initiation
5299	HSA0743	No	3	-
5300	HSA0744	No	1	-
5301	HSA0745	No	2	Others
5302	HSA0746	No	1	-
5303	HSA0747	No	2	-
5304	HSA0748	No	2	-
5305	HSA0749	No	2	Peer pressure
5306	HSA0750	Yes	3	-
5307	HSA0751	No	1	-
5308	HSA0752	No	1	-
5309	HSA0753	No	1	-
5310	HSA0754	No	1	-
5311	HSA0755	No	1	-
5312	HSA0756	No	1	-
5313	HSA0757	No	2	Others
5314	HSA0758	No	1	Relaxation
5315	HSA0759	No	2	-
5316	HSA0760	No	2	-
5317	HSA0761	No	1	-
5318	HSA0762	No	1	-
5319	HSA0763	No	3	Others
5320	HSA0764	No	1	-
5321	HSA0765	No	1	-
5322	HSA0766	No	1	-
5323	HSA0767	No	2	-
5324	HSA0768	No	1	-
5325	HSA0769	No	2	-
5326	HSA0770	No	1	-
5327	HSA0771	No	3	-
5328	HSA0772	No	1	-
5329	HSA0773	No	2	-

No	Patient ID	Other substances	No of substances abused at a time	Reason for substance use initiation
5330	HSA0774	No	2	Joy seeking
5331	HSA0775	No	1	-
5332	HSA0776	No	1	-
5333	HSA0777	No	1	-
5334	HSA0778	No	1	-
5335	HSA0779	No	3	Others
5336	HSA0780	Yes	2	-
5337	HSA0781	No	1	-
5338	HSA0782	No	2	-
5339	HSA0783	No	2	-
5340	HSA0784	No	1	-
5341	HSA0785	No	1	-
5342	HSA0786	No	1	-
5343	HSA0787	No	1	-
5344	HSA0788	No	1	-
5345	HSA0789	No	2	Joy seeking
5346	HSA0790	No	1	-
5347	HSA0791	No	2	Joy seeking
5348	HSA0792	No	1	-
5349	HSA0793	No	2	-
5350	HSA0794	No	2	-
5351	HSA0796	No	2	-
5352	HSA0796	No	1	-
5353	HSA0797	No	1	-
5354	HSA0798	No	1	Family issue
5355	HSA0799	No	2	-
5356	HSA0800	No	1	-
5357	HSA0801	No	1	Others
5358	HSA0802	No	1	Peer pressure
5359	HSA0803	No	3	-
5360	HSA0804	No	1	-

No	Patient ID	Other substances	No of substances abused at a time	Reason for substance use initiation
5361	HSA0805	No	3	-
5362	HSA0806	Yes	3	-
5363	HSA0807	No	1	-
5364	HSA0808	No	2	Peer pressure
5365	HSA0809	No	2	-
5366	HSA0810	No	2	Family issue, Relationship
5367	HSA0811	Yes	3	-
5368	HSA0812	No	1	-
5369	HSA0813	No	2	-
5370	HSA0814	No	4	-
5371	HSA0815	No	1	-
5372	HSA0816	No	2	-
5373	HSA0817	No	1	-
5374	HSA0819	No	2	-
5375	HSA0820	No	1	-
5376	HSA0821	No	1	-
5377	HSA0822	Yes	3	Peer pressure
5378	HSA0823	No	1	Others
5379	HSA0824	No	4	Peer pressure
5380	HSA0825	No	1	Peer pressure
5381	HSA0826	No	1	-
5382	HSA0827	No	1	-
5383	HSA0828	No	3	-
5384	HSA0829	No	3	-
5385	HSA0830	No	1	Joy seeking
5386	HSA0831	No	2	Others
5387	HSA0832	No	1	-
5388	HSA0833	No	3	Joy seeking
5389	HSA0834	No	1	-
5390	HSA0835	No	1	-
5391	HSA0836	No	4	-
5392	HSA0837	No	1	-
5393	HSA0838	No	2	-

No	Patient ID	Other substances	No of substances abused at a time	Reason for substance use initiation
5394	HSA0839	No	1	-
5395	HSA0840	No	1	-
5396	HSA0841	No	2	-
5397	HSA0842	No	2	-
5398	HSA0843	No	1	-
5399	HSA0844	No	2	-
5400	HSA0845	No	2	-
5401	HSA0846	No	2	-
5402	HSA0847	No	1	-
5403	HSA0848	No	1	-
5404	HSA0849	No	1	-
5405	HSA0850	No	3	-
5406	HSA0851	No	3	-
5407	HSA0852	No	1	-
5408	HSA0853	No	2	-
5409	HSA0854	No	2	-
5410	HSA0855	No	2	-
5411	HSA0856	No	2	Peer pressure
5412	HSA0857	No	2	-
5413	HSA0858	No	1	-
5414	HSA0859	No	2	-
5415	HSA0860	No	3	-
5416	HSA0861	No	2	-
5417	HSA0862	No	5	-
5418	HSA0863	No	1	-
5419	HSA0864	No	3	Peer pressure
5420	HSA0865	No	1	Family issue
5421	HSA0866	No	1	-
5422	HSA0867	No	1	-
5423	HSA0868	No	2	-

No	Patient ID	Other substances	No of substances abused at a time	Reason for substance use initiation
5424	HSA0869	No	3	-
5425	HSA0870	No	1	-
5426	HSA0871	No	2	-
5427	HSA0872	No	2	-
5428	HSA0873	No	1	-
5429	HSA0874	No	2	-
5430	HSA0875	No	1	-
5431	HSA0876	No	1	-
5432	HSA0877	No	1	-
5433	HSA0878	No	1	-
5434	HSA0879	No	1	-
5435	HSA0880	No	2	-
5436	HSA0881	No	1	-
5437	HSA0882	No	2	-
5438	HSA0883	No	1	-
5439	HSA0884	Yes	2	-
5440	HSA0885	No	2	-
5441	HSA0886	No	1	-
5442	HSA0887	No	1	-
5443	HSA0888	No	2	-
5444	HSA0889	No	2	-
5445	HSA0890	No	2	-
5446	HSA0891	No	1	-
5447	HSA0892	No	2	-
5448	HSA0893	No	1	-
5449	HSA0894	No	2	-
5450	HSA0895	No	1	-
5451	HSA0896	No	1	-
5452	HSA0897	No	2	-
5453	HSA0898	No	1	-

No	Patient ID	Other substances	No of substances abused at a time	Reason for substance use initiation
5454	HSA0899	No	1	-
5455	HSA0900	No	2	-
5456	HSA0901	Yes	3	-
5457	HSA0902	No	2	-
5458	HSA0903	No	2	-
5459	HSA0904	No	1	-
5460	HSA0905	No	2	-
5461	HSA0906	No	1	-
5462	HSA0907	No	1	-
5463	HSA0908	No	1	-
5464	HSA0909	No	2	Joy seeking
5465	HSA0910	No	2	-
5466	HSA0911	No	1	-
5467	HSA0912	No	2	-
5468	HSA0913	No	2	-
5469	HSA0914	No	1	-
5470	HSA0915	No	1	-
5471	HSA0916	No	2	-
5472	HSA0917	No	2	-
5473	HSA0918	No	1	-
5474	HSA0919	No	2	-
5475	HSA0920	No	1	Relationship
5476	HSA0921	No	2	-
5477	HSA0922	No	2	-
5478	HSA0923	No	1	-
5479	HSA0924	No	2	-
5480	HSA0925	No	1	-
5481	HSA0926	No	1	-
5482	HSA0927	No	2	-
5483	HSA0928	No	1	-
5484	HSA0929	No	2	-
5485	HSA0930	No	2	-

No	Patient ID	Other substances	No of substances abused at a time	Reason for substance use initiation
5486	HSA0931	No	2	-
5487	HSA0932	No	1	-
5488	HSA0933	No	1	-
5489	HSA0934	No	3	Peer pressure
5490	HSA0935	No	1	-
5491	HSA0936	No	2	-
5492	HSA0937	No	2	-
5493	HSA0938	No	1	-
5494	HSA0940	No	2	-
5495	HSA0941	No	2	-
5496	HSA0942	No	2	-
5497	HSA0943	Yes	2	-
5498	HSA0944	No	1	Peer pressure
5499	HSA0945	No	2	Peer pressure, Others
5500	HSA0946	No	2	-
5501	HSA0947	No	2	-
5502	HSA0948	No	2	-
5503	HSA0949	No	1	-
5504	HSA0950	No	2	-
5505	HSA0951	No	2	-
5506	HSA0952	No	2	-
5507	HSA0953	No	1	-
5508	HSA0954	No	3	-
5509	HSA0955	No	1	-
5510	HSA0956	No	1	-
5511	HSA0957	No	1	-
5512	HSA0958	No	2	-
5513	HSA0959	No	2	Others
5514	HSA0960	No	2	-
5515	HSA0961	No	3	-
5516	HSA0962	No	3	-
5517	HSA0963	No	2	Others
5518	HSA0964	No	1	Peer pressure
5519	HSA0965	No	1	Peer pressure
5520	HSA0966	No	2	-

No	Patient ID	Other substances	No of substances abused at a time	Reason for substance use initiation
5521	HSA0967	No	2	-
5522	HSA0969	No	1	-
5523	HSA0970	No	1	-
5524	HSA0971	No	2	-
5525	HSA0972	No	2	-
5526	HSA0973	No	1	-
5527	HSA0974	No	1	Peer pressure
5528	HSA0975	No	3	Others
5529	HSA0976	No	1	Peer pressure
5530	HSA0977	No	2	-
5531	HSA0978	No	2	-
5532	HSA0979	No	2	-
5533	HSA0980	No	2	-
5534	HSA0981	No	1	-
5535	HSA0982	No	3	Peer pressure
5536	HSA0983	No	1	-
5537	HSA0984	No	1	-
5538	HSA0985	No	1	-
5539	HSA0986	No	1	-
5540	HSA0987	No	1	-
5541	HSA0988	No	1	-
5542	HSA0989	No	1	-
5543	HSA0990	No	1	-
5544	HSA0991	No	2	Health, Peer pressure
5545	HSA0992	No	1	-
5546	HSA0993	No	1	-
5547	HSA0994	No	1	-
5548	HSA0995	No	4	-
5549	HSA0996	No	2	-
5550	HSA0997	No	2	-
5551	HSA0998	Yes	3	-
5552	HSA0999	No	1	-
5553	HSA1000	No	2	Peer pressure

No	Patient ID	Other substances	No of substances abused at a time	Reason for substance use initiation
5554	HSA1001	No	1	-
5555	HSA1002	No	1	-
5556	HSA1003	No	3	-
5557	HSA1004	No	1	-
5558	HSA1005	No	1	Peer pressure
5559	HSA1006	No	2	-
5560	HSA1007	No	1	-
5561	HSA1008	No	1	-
5562	HSA1009	No	2	-
5563	HSA1010	No	2	-
5564	HSA1011	No	1	-
5565	HSA1012	Yes	1	Peer pressure
5566	HSA1013	No	1	-
5567	HSA1014	No	3	-
5568	HSA1015	No	1	-
5569	HSA1016	No	3	-
5570	HSA1017	No	1	-
5571	HSA1018	No	1	-
5572	HSA1019	No	3	-
5573	HSA1020	Yes	1	-
5574	HSA1021	No	1	-
5575	HSA1022	No	3	Relationship
5576	HSA1023	No	2	-
5577	HSA1024	No	2	-
5578	HSA1025	No	1	-
5579	HSA1026	No	4	-
5580	HSA1027	No	2	-
5581	HSA1028	No	2	-
5582	HSA1029	No	1	-

No	Patient ID	Other substances	No of substances abused at a time	Reason for substance use initiation
5583	HSA1030	No	1	Peer pressure
5584	HSA1031	No	2	-
5585	HSA1032	No	2	-
5586	HSA1033	No	2	-
5587	HSA1035	No	2	-
5588	HSA1036	No	4	Relaxation
5589	HSA1037	No	2	-
5590	HSA1038	No	1	-
5591	HSA1039	Yes	3	Others
5592	HSA1040	No	3	-
5593	HSA1041	No	1	-
5594	HSA1042	No	1	-
5595	HSA1043	No	2	-
5596	HSA1044	No	3	-
5597	HSA1045	No	1	-
5598	HSA1046	No	2	-
5599	HSA1047	No	1	-
5600	HSA1048	No	3	-
5601	HSA1050	No	1	-
5602	HSA1051	No	1	-
5603	HSA1052	No	2	-
5604	HSA1053	No	2	-
5605	HSA1054	Yes	4	Peer pressure
5606	HSA1055	No	2	Peer pressure
5607	HSA1056	No	4	Relaxation
5608	HSA1057	No	2	-
5609	HSA1058	No	2	Others
5610	HSA1059	No	3	-
5611	HSA1060	No	2	-
5612	HSA1061	No	3	Others
5613	HSA1062	No	3	Peer pressure, Others
5614	HSA1063	No	1	-
5615	HSA1064	No	1	Peer pressure

No	Patient ID	Other substances	No of substances abused at a time	Reason for substance use initiation
5616	HSA1065	No	4	-
5617	HSA1066	No	1	-
5618	HSA1067	No	3	-
5619	HSA1068	No	3	-
5620	HSA1069	No	3	-
5621	HSA1070	No	2	-
5622	HSA1071	Yes	2	Others
5623	HSA1072	No	1	-
5624	HSA1073	No	1	-
5625	HSA1074	No	1	-
5626	HSA1075	No	2	-
5627	HSA1076	No	2	Peer pressure
5628	HSA1077	No	1	-
5629	HSA1078	No	2	Peer pressure
5630	HSA1079	No	1	-
5631	HSA1080	Yes	4	-
5632	HSA1081	No	3	Peer pressure
5633	HSA1082	No	1	-
5634	HSA1083	Yes	4	-
5635	HSA1084	Yes	4	-
5636	HSA1085	No	5	Peer pressure
5637	HSA1086	No	3	Others
5638	HSA1087	No	2	Others
5639	HSA1088	No	3	-
5640	HSA1089	No	1	-
5641	HSA1090	No	2	-
5642	HSA1091	No	1	-
5643	HSA1092	No	1	-
5644	HSA1093	No	1	-
5645	HSA1094	No	2	-

No	Patient ID	Other substances	No of substances abused at a time	Reason for substance use initiation
5646	HSA1095	No	1	Peer pressure
5647	HSA1096	No	2	-
5648	HSA1097	No	1	-
5649	HSA1098	No	3	-
5650	HSA1099	No	2	Others
5651	HSA1100	No	3	-
5652	HSA1101	No	1	-
5653	HSA1102	No	1	-
5654	HSA1103	No	3	-
5655	HSA1104	No	2	Peer pressure
5656	HSA1105	No	1	-
5657	HSA1106	No	1	-
5658	HSA1107	No	1	-
5659	HSA1108	No	2	-
5660	HSA1109	No	1	-
5661	HSA1110	No	1	-
5662	HSA1111	No	1	-
5663	HSA1112	Yes	5	Relaxation
5664	HSA1113	No	1	-
5665	HSA1114	No	2	Peer pressure
5666	HSA1115	No	2	-
5667	HSA1116	No	2	-
5668	HSA1117	No	1	-
5669	HSA1118	No	2	-
5670	HSA1119	No	4	-
5671	HSA1120	No	2	Peer pressure
5672	HSA1121	No	1	-
5673	HSA1122	No	1	-
5674	HSA1123	No	1	-
5675	HSA1125	No	1	-
5676	HSA1126	No	1	-
5677	HSA1126	No	1	-
5678	HSA1127	No	2	Others
5679	HSA1128	No	1	-

No	Patient ID	Other substances	No of substances abused at a time	Reason for substance use initiation
5680	HSA1129	No	2	-
5681	HSA1130	No	3	-
5682	HSA1131	No	3	-
5683	HSA1132	Yes	1	-
5684	HSA1133	No	1	-
5685	HSA1134	No	1	-
5686	HSA1135	No	2	-
5687	HSA1136	No	1	Relaxation
5688	HSA1137	No	4	-
5689	HSA1138	No	2	-
5690	HSA1139	No	3	-
5691	HSA1140	No	3	Relaxation
5692	HSA1141	No	1	-
5693	HSA1142	No	1	-
5694	HSA1143	No	2	-
5695	HSA1144	No	2	-
5696	HSA1145	No	3	-
5697	HSA1146	No	5	-
5698	HSA1147	No	1	-
5699	HSA1148	No	2	Relaxation
5700	HSA1149	No	1	-
5701	HSA1150	No	1	-
5702	HSA1151	No	1	-
5703	HSA1152	No	2	-
5704	HSA1153	No	1	-
5705	HSA1154	No	3	-
5706	HSA1155	No	1	Peer pressure
5707	HSA1156	No	1	-
5708	HSA1157	No	2	-
5709	HSA1158	No	2	-
5710	HSA1159	No	1	-
5711	HSA1160	No	2	-
5712	HSA1161	No	1	-

No	Patient ID	Other substances	No of substances abused at a time	Reason for substance use initiation
5713	HSA1162	No	1	-
5714	HSA1163	No	1	-
5715	HSA1164	No	2	-
5716	HSA1165	No	1	-
5717	HSA1166	No	1	-
5718	HSA1167	No	2	-
5719	HSA1168	No	1	Others
5720	HSA1169	No	1	-
5721	HSA1170	Yes	4	-
5722	HSA1171	No	2	-
5723	HSA1172	No	1	-
5724	HSA1173	No	2	-
5725	HSA1174	Yes	4	-
5726	HSA1175	No	2	-
5727	HSA1176	No	2	-
5728	HSA1177	No	2	-
5729	HSA1178	No	1	-
5730	HSA1179	No	2	-
5731	HSA1180	No	1	-
5732	HSA1181	No	1	-
5733	HSA1182	No	1	-
5734	HSA1183	No	1	-
5735	HSA1184	No	1	-
5736	HSA1185	No	2	-
5737	HSA1186	No	3	Peer pressure
5738	HSA1187	No	3	-
5739	HSA1188	No	2	-
5740	HSA1189	No	1	-
5741	HSA1190	No	1	-
5742	HSA1191	No	1	Relaxation, Joy seeking
5743	HSA1192	No	2	-
5744	HSA1193	No	1	-
5745	HSA1194	No	1	-

No	Patient ID	Other substances	No of substances abused at a time	Reason for substance use initiation
5746	HSA1195	No	2	-
5747	HSA1196	No	1	Peer pressure
5748	HSA1197	No	1	-
5749	HSA1198	No	3	-
5750	HSA1199	No	3	-
5751	HSA1200	No	1	-
5752	HSA1201	No	1	-
5753	HSA1202	No	2	Peer pressure
5754	HSA1203	No	1	-
5755	HSA1204	No	2	-
5756	HSA1205	No	1	-
5757	HSA1206	No	1	-
5758	HSA1207	No	2	Peer pressure
5759	HSA1208	No	1	-
5760	HSA1209	No	1	-
5761	HSA1210	No	1	-
5762	HSA1212	No	1	-
5763	HSA1213	No	3	-
5764	HSA1214	No	3	-
5765	HSA1216	No	1	Peer pressure
5766	HSA1217	No	3	-
5767	HSA1218	No	1	-
5768	HSA1219	No	2	-
5769	HSA1220	No	2	-
5770	HSA1221	No	1	-
5771	HSA1222	No	1	-
5772	HSA1223	No	1	-
5773	HSA1224	Yes	2	-
5774	HSA1225	No	2	-
5775	HSA1226	No	1	-
5776	HSA1228	No	2	-
5777	HSA1229	No	1	-
5778	HSA1230	No	1	-
5779	HSA1231	No	3	Others

No	Patient ID	Other substances	No of substances abused at a time	Reason for substance use initiation
5780	HSA1232	No	1	Relaxation
5781	HSA1233	No	1	-
5782	HSA1234	No	2	-
5783	HSA1235	No	2	-
5784	HSA1236	No	2	-
5785	HSA1237	No	1	-
5786	HSA1238	No	3	-
5787	HSA1239	No	1	-
5788	HSA1240	No	1	-
5789	HSA1242	No	2	-
5790	HSA1243	No	2	-
5791	HSA1244	No	2	-
5792	HSA1245	No	2	-
5793	HSA1247	No	1	-
5794	HSA1248	No	3	-
5795	HSA1249	No	1	-
5796	HSA1250	Yes	2	-
5797	HSA1251	No	2	-
5798	HSA1252	No	1	-
5799	HSA1253	No	1	-
5800	HSA1254	No	1	-
5801	HSA1255	No	1	-
5802	HSA1256	No	2	-
5803	HSA1257	No	1	-
5804	HSA1258	No	1	-
5805	HSA1259	No	1	-
5806	HSA1260	No	1	-
5807	HSA1261	No	1	Peer pressure
5808	HSA1262	No	1	-
5809	HSA1263	Yes	6	-
5810	HSA1264	No	1	-
5811	HSA1265	No	3	-
5812	HSA1266	No	1	Health

No	Patient ID	Other substances	No of substances abused at a time	Reason for substance use initiation
5813	HSA1267	No	1	-
5814	HSA1268	No	1	-
5815	HSA1269	No	1	-
5816	HSA1270	No	2	Peer pressure
5817	HSA1272	No	1	-
5818	HSA1273	No	3	-
5819	HSA1274	No	2	-
5820	HSA1275	No	2	-
5821	HSA1276	No	1	-
5822	HSA1277	No	1	-
5823	HSA1278	No	3	Others
5824	HSA1279	No	2	-
5825	HSA1280	No	1	-
5826	HSA1281	No	3	-
5827	HSA1282	No	1	-
5828	HSA1283	No	1	-
5829	HSA1284	No	1	-
5830	HSA1285	No	2	-
5831	HSA1286	No	2	-
5832	HSA1287	No	3	Relationship
5833	HSA1288	No	2	Family issue
5834	HSA1289	No	2	-
5835	HSA1290	No	1	-
5836	HSA1291	No	2	-
5837	HSA1292	No	1	-
5838	HSA1293	No	1	-
5839	HSA1294	No	1	-
5840	HSA1295	No	1	-
5841	HSA1296	No	2	-
5842	HSA1297	No	3	Joy seeking
5843	HSA1298	No	1	-
5844	HSA1299	No	1	-
5845	HSA1300	No	1	-
5846	HSA1301	No	2	-
5847	HSA1302	No	2	-

No	Patient ID	Other substances	No of substances abused at a time	Reason for substance use initiation
5848	HSA1303	No	1	-
5849	HSA1304	No	2	-
5850	HSA1305	No	2	Peer pressure
5851	HSA1306	No	1	-
5852	HSA1307	No	2	-
5853	HSA1308	No	3	-
5854	HSA1309	No	2	-
5855	HSA1310	Yes	1	Peer pressure
5856	HSA1311	No	3	-
5857	HSA1312	No	2	-
5858	HSA1313	No	3	Peer pressure
5859	HSA1314	No	1	-
5860	HSA1315	No	1	-
5861	HSA1316	No	1	-
5862	HSA1317	No	1	Peer pressure
5863	HSA1318	No	3	Peer pressure
5864	HSA1319	No	1	-
5865	HSA1320	No	1	-
5866	HSA1321	No	2	-
5867	HSA1322	No	1	-
5868	HSA1323	No	1	-
5869	HSA1324	No	2	-
5870	HSA1325	No	1	-
5871	HSA1326	Yes	5	-
5872	HSA1327	No	2	-
5873	HSA1328	No	2	-
5874	HSA1329	No	1	-
5875	HSA1330	No	1	-
5876	HSA1331	No	1	-
5877	HSA1332	No	1	-
5878	HSA1333	No	2	-

No	Patient ID	Other substances	No of substances abused at a time	Reason for substance use initiation
5879	HSA1334	No	2	-
5880	HSA1335	No	1	-
5881	HSA1336	No	2	Relaxation
5882	HSA1337	No	1	-
5883	HSA1338	No	1	-
5884	HSA1339	No	2	-
5885	HSA1340	No	2	-
5886	HSA1341	No	1	-
5887	HSA1342	No	2	-
5888	HSA1343	No	1	-
5889	HSA1344	Yes	3	Joy seeking, Peer pressure
5890	HSA1345	No	1	-
5891	HSA1346	No	2	-
5892	HSA1347	No	1	-
5893	HSA1348	No	1	-
5894	HSA1349	No	2	-
5895	HSA1350	No	3	-
5896	HSA1351	No	1	-
5897	HSA1352	No	2	-
5898	HSA1353	No	2	-
5899	HSA1354	No	1	-
5900	HSA1355	No	1	-
5901	HSA1356	No	1	-
5902	HSA1357	No	1	-
5903	HSA1358	No	1	-
5904	HSA1359	No	1	-
5905	HSA1360	No	1	-
5906	HSA1361	No	1	-
5907	HSA1362	No	1	-
5908	HSA1363	No	1	-
5909	HSA1364	No	1	-
5910	HSA1365	No	1	-
5911	HSA1366	No	2	-

No	Patient ID	Other substances	No of substances abused at a time	Reason for substance use initiation
5912	HSA1367	No	1	-
5913	HSA1368	No	3	-
5914	HSA1369	No	3	Relaxation
5915	HSA1370	No	1	-
5916	HSA1372	No	1	-
5917	HSA1373	No	3	-
5918	HSA1374	No	2	-
5919	HSA1375	No	1	-
5920	HSA1376	No	2	-
5921	HSA1377	No	1	-
5922	HSA1378	No	1	-
5923	HSA1379	No	1	-
5924	HSA1380	No	1	-
5925	HSA1381	Yes	6	-
5926	HSA1382	No	1	-
5927	HSA1383	No	1	-
5928	HSA1384	No	3	Relaxation
5929	HSA1385	No	2	-
5930	HSA1386	No	3	-
5931	HSA1387	No	1	-
5932	HSA1388	No	2	-
5933	HSA1389	No	3	Others
5934	HSA1390	No	1	-
5935	HSA1391	No	2	-
5936	HSA1392	No	3	-
5937	HSA1393	No	2	Health
5938	HSA1394	No	2	Peer pressure
5939	HSA1395	No	2	-
5940	HSA1396	No	2	-
5941	HSA1397	No	2	Peer pressure
5942	HSA1398	No	2	-
5943	HSA1399	No	2	-

No	Patient ID	Other substances	No of substances abused at a time	Reason for substance use initiation
5944	HSA1400	No	1	-
5945	HSA1401	No	2	-
5946	HSA1402	No	3	-
5947	HSA1403	No	3	-
5948	HSA1404	Yes	4	Peer pressure
5949	HSA1405	No	1	-
5950	HSA1406	No	1	-
5951	HSA1407	No	1	-
5952	HSA1408	No	1	-
5953	HSA1409	No	1	-
5954	HSA1410	No	2	-
5955	HSA1411	No	1	-
5956	HSA1412	No	2	-
5957	HSA1413	No	1	-
5958	HSA1414	No	1	-
5959	HSA1415	No	5	-
5960	HSA1416	No	2	-
5961	HSA1417	No	3	-
5962	HSA1418	No	1	-
5963	HSA1419	No	1	-
5964	HSA1420	No	1	Family issue
5965	HSA1421	No	3	-
5966	HSA1422	No	1	-
5967	HSA1423	No	1	-
5968	HSA1424	No	2	-
5969	HSA1425	No	3	Family issue
5970	HSA1426	No	3	Joy seeking
5971	HSA1427	No	3	-

No	Patient ID	Other substances	No of substances abused at a time	Reason for substance use initiation
5972	HSA1428	No	2	-
5973	HSA1429	No	3	-
5974	HSA1430	No	2	-
5975	HSA1432	No	3	-
5976	HSA1433	No	2	Peer pressure
5977	HSA1434	No	1	-
5978	HSA1435	No	1	-
5979	HSA1436	No	2	-
5980	HSA1437	No	1	-
5981	HSA1438	No	2	Peer pressure
5982	HSA1439	No	1	-
5983	HSA1440	No	1	-
5984	HSA1441	No	1	-
5985	HSA1442	No	1	-
5986	HSA1443	No	1	-
5987	HSA1444	No	1	-
5988	HSA1447	No	1	-
5989	HSA1448	No	2	-
5990	HSA1449	No	2	-
5991	HSA1450	No	1	-
5992	HSA1451	No	1	-
5993	HSA1453	No	1	-
5994	HSA1454	No	1	-
5995	HSA1455	No	2	-
5996	HSA1456	No	1	-
5997	HSA1458	No	2	-
5998	HSA1460	No	2	-
5999	HSA1461	No	1	-
6000	HSA1462	No	2	-
6001	HSA1463	No	2	-
6002	HSA1464	No	1	-
6003	HSA1465	No	3	-
6004	HSA1466	No	1	-
6005	HSA1467	No	1	-

No	Patient ID	Other substances	No of substances abused at a time	Reason for substance use initiation
6006	HSA1468	Yes	3	Relaxation, Peer pressure
6007	HSA1469	No	1	-
6008	HSA1470	No	2	Peer pressure
6009	HSA1471	No	1	-
6010	HSA1472	No	1	-
6011	HSA1473	No	1	-
6012	HSA1474	No	1	-
6013	HSA1475	No	3	-
6014	HSA1476	No	2	-
6015	HSA1477	No	1	-
6016	HSA1479	No	1	-
6017	HSA1480	No	2	-
6018	HSA1481	No	1	-
6019	HSA1482	No	1	-
6020	HSA1483	No	1	-
6021	HSA1484	No	1	-
6022	HSA1485	No	1	-
6023	HSA1486	No	2	-
6024	HSA1487	No	1	-
6025	HSA1488	No	2	-
6026	HSA1489	No	1	-
6027	HSA1490	No	2	-
6028	HSA1491	No	1	-
6029	HSA1492	No	1	-
6030	HSA1493	No	1	-
6031	HSA1494	No	1	-
6032	HSA1495	No	2	-
6033	HSA1496	No	1	-
6034	HSA1498	No	2	-
6035	HSA1499	No	1	-
6036	HSA1500	No	2	-
6037	HSA1502	No	1	-
6038	HSA1503	No	1	-

No	Patient ID	Other substances	No of substances abused at a time	Reason for substance use initiation
6039	HSA1504	No	1	Joy seeking
6040	HSA1505	No	1	-
6041	HSA1506	No	1	-
6042	HSA1507	No	2	-
6043	HSA1508	No	1	-
6044	HSA1509	No	1	-
6045	HSA1510	No	2	-
6046	HSA1511	No	1	-
6047	HSA1512	No	2	-
6048	HSA1513	No	1	-
6049	HSA1514	No	1	-
6050	HSA1515	No	1	-
6051	HSA1516	No	1	-
6052	HSA1517	No	1	-
6053	HSA1518	No	1	-
6054	HSA1519	No	1	-
6055	HSA1520	No	1	-
6056	HSA1521	No	1	-
6057	HSA1522	No	2	-
6058	HSA1523	No	1	-
6059	HSA1525	No	2	-
6060	HSA1526	No	1	-
6061	HSA1527	No	2	-
6062	HSA1528	No	2	-
6063	HSA1529	No	2	-
6064	HSA1530	No	1	-
6065	HSA1531	No	1	-
6066	HSA1532	No	1	-
6067	HSB0001	Yes	9	Job problem, Relaxation
6068	HSB0002	Yes	10	-
6069	HSB0003	Yes	10	Peer pressure
6070	HSB0004	Yes	9	-
6071	HSB0005	Yes	10	-
6072	HSB0006	Yes	10	Health
6073	HSB0007	Yes	10	-

No	Patient ID	Other substances	No of substances abused at a time	Reason for substance use initiation
6074	HSB0008	Yes	10	-
6075	HSB0009	Yes	10	-
6076	HSB0010	Yes	10	Relaxation
6077	HSB0011	Yes	10	-
6078	HSB0012	Yes	10	-
6079	HSB0013	Yes	10	-
6080	HSB0014	Yes	10	Joy seeking
6081	HSB0015	Yes	10	-
6082	HSB0016	Yes	10	-
6083	HSB0017	Yes	10	-
6084	HSB0018	Yes	10	-
6085	HSB0019	Yes	10	-
6086	HSB0020	Yes	10	Peer pressure
6087	HSB0021	Yes	10	Peer pressure
6088	HSB0022	Yes	10	-
6089	HSB0023	Yes	10	Peer pressure
6090	HSB0024	Yes	10	-
6091	HSB0025	Yes	10	-
6092	HSB0026	Yes	10	-
6093	HSB0027	Yes	10	-
6094	HSB0028	Yes	10	Joy seeking
6095	HSB0029	Yes	10	-
6096	HSB0030	Yes	10	Joy seeking
6097	HSB0031	No	2	-
6098	HSB0032	Yes	10	-
6099	HSB0033	Yes	10	Peer pressure
6100	HSB0034	Yes	10	-
6101	HSB0035	Yes	10	-
6102	HSB0036	Yes	10	Peer pressure
6103	HSB0037	Yes	10	Joy seeking
6104	HSB0038	Yes	10	Relationship, Peer pressure
6105	HSB0039	Yes	10	Job problem
6106	HSB0040	Yes	10	Peer pressure
6107	HSB0041	Yes	10	-
6108	HSB0042	Yes	10	Relaxation
6109	HSB0043	Yes	10	-
6110	HSB0044	Yes	9	Job problem, Peer pressure
6111	HSB0045	Yes	9	-

No	Patient ID	Other substances	No of substances abused at a time	Reason for substance use initiation
6112	HSB0047	Yes	10	-
6113	HSB0049	Yes	10	Family issue, Relationship, Peer pressure, Financial problem
6114	HSB0050	Yes	10	-
6115	HSB0051	Yes	10	Relaxation
6116	HSB0052	Yes	9	Family issue, Peer pressure
6117	HSB0053	Yes	9	-
6118	HSB0054	Yes	9	Joy seeking
6119	HSB0055	Yes	9	-
6120	HSB0056	Yes	10	Family issue, Job problem, Relaxation, Peer pressure
6121	HSB0057	Yes	9	Job problem
6122	HSB0058	Yes	10	Joy seeking
6123	HSB0058	Yes	10	Joy seeking
6124	HSB0059	Yes	10	Relaxation, Joy seeking, Peer pressure
6125	HSB0060	No	9	-
6126	HSB0061	Yes	10	Peer pressure
6127	HSB0062	Yes	10	-
6128	HSB0063	Yes	10	-
6129	HSB0064	Yes	10	-
6130	HSB0065	Yes	10	-
6131	HSB0066	Yes	10	-
6132	HSB0067	Yes	10	-
6133	HSB0068	Yes	10	Relationship
6134	HSB0070	Yes	10	Relaxation
6135	HSB0071	Yes	10	-
6136	HSB0072	Yes	10	-
6137	HSB0073	Yes	10	Relaxation, Joy seeking, Others
6138	HSB0074	Yes	10	-
6139	HSB0075	Yes	10	Family issue, Relaxation
6140	HSB0076	Yes	10	Peer pressure
6141	HSB0077	Yes	10	Relaxation

No	Patient ID	Other substances	No of substances abused at a time	Reason for substance use initiation
6142	HSB0078	Yes	10	Peer pressure
6143	HSB0079	Yes	10	Joy seeking, Peer pressure
6144	HSB0080	Yes	10	-
6145	HSB0081	Yes	10	-
6146	HSB0082	No	2	Others
6147	HSB0083	No	1	-
6148	HSB0084	Yes	10	Peer pressure
6149	HSB0085	Yes	10	Relaxation
6150	HSB0086	Yes	10	-
6151	HSB0087	Yes	10	Relaxation
6152	HSB0088	Yes	10	-
6153	HSB0090	Yes	10	-
6154	HSB0091	Yes	10	Relationship, Others
6155	HSB0092	Yes	10	Joy seeking, Peer pressure
6156	HSB0093	Yes	10	-
6157	HSB0094	No	2	Job problem, Relaxation
6158	HSB0095	No	2	Others
6159	HSB0096	No	1	-
6160	HSB0097	No	3	Relationship, Others
6161	HSB0098	No	2	-
6162	HSB0099	No	2	-
6163	HSB0100	Yes	10	Peer pressure
6164	HSB0101	Yes	10	Job problem
6165	HSB0102	Yes	10	-
6166	HSB0103	Yes	10	Peer pressure
6167	HSB0104	Yes	9	Family issue
6168	HSB0105	Yes	10	Job problem
6169	HSB0106	Yes	10	Peer pressure
6170	HSB0107	Yes	10	Family issue, Job problem, Relaxation, Financial problem
6171	HSB0108	Yes	10	Peer pressure
6172	HSB0109	Yes	10	-
6173	HSB0110	Yes	10	-
6174	HSB0111	No	2	-
6175	HSB0112	Yes	10	Relaxation, Joy seeking
6176	HSB0113	Yes	10	Peer pressure

No	Patient ID	Other substances	No of substances abused at a time	Reason for substance use initiation
6177	HSB0114	Yes	10	-
6178	HSB0115	Yes	10	Job problem, Financial problem
6179	HSB0116	Yes	10	-
6180	HSB0117	Yes	10	Job problem
6181	HSB0118	Yes	10	Family issue, Relaxation
6182	HSB0119	Yes	10	Job problem
6183	HSB0121	Yes	10	Peer pressure
6184	HSB0122	Yes	10	Health, Job problem
6185	HSB0123	Yes	10	-
6186	HSB0125	No	4	-
6187	HSB0126	Yes	9	Health, Job problem, Relationship, Relaxation
6188	HSB0127	Yes	10	-
6189	HSB0128	Yes	10	-
6190	HSB0129	No	3	Others
6191	HSB0130	Yes	9	Joy seeking
6192	HSB0131	Yes	10	Peer pressure
6193	HSB0132	Yes	3	Others
6194	HSB0133	Yes	9	-
6195	HSB0134	Yes	9	-
6196	HSB0135	Yes	9	Job problem
6197	HSB0136	Yes	9	Job problem, Relaxation
6198	HSB0137	No	1	-
6199	HSB0138	Yes	10	-
6200	HSB0139	Yes	9	Relaxation
6201	HSB0140	Yes	9	-
6202	HSB0141	Yes	9	-
6203	HSB0142	Yes	9	Job problem
6204	HSB0143	Yes	9	-
6205	HSB0144	Yes	10	Relaxation
6206	HSB0145	Yes	10	Family issue
6207	HSB0146	Yes	10	-
6208	HSB0147	Yes	10	-

No	Patient ID	Other substances	No of substances abused at a time	Reason for substance use initiation
6209	HSB0148	No	1	-
6210	HSB0150	Yes	4	Relaxation, Joy seeking, Peer pressure
6211	HSB0151	No	5	Peer pressure
6212	HSB0152	No	2	Joy seeking, Others
6213	HSB0153	No	3	Peer pressure
6214	HSB0154	No	1	-
6215	HSB0155	No	2	Joy seeking
6216	HSB0156	No	1	Peer pressure
6217	HSB0157	Yes	3	Peer pressure
6218	HSB0159	No	1	-
6219	HSB0160	No	4	-
6220	HSB0161	No	3	-
6221	HSB0162	No	2	-
6222	HSB0163	Yes	4	Peer pressure
6223	HSB0164	No	1	-
6224	HSB0165	No	3	Joy seeking
6225	HSB0166	No	3	-
6226	HSB0167	No	1	Relationship, Peer pressure
6227	HSB0168	No	2	Joy seeking
6228	HSB0169	No	3	Peer pressure, Others
6229	HSB0170	No	2	-
6230	HSB0171	Yes	3	Peer pressure
6231	HSB0172	Yes	10	Peer pressure
6232	HSB0172	Yes	10	Peer pressure
6233	HSB0173	Yes	10	Peer pressure
6234	HSB0174	Yes	10	Joy seeking, Peer pressure
6235	HSB0175	Yes	10	Relaxation, Financial problem
6236	HSB0176	No	2	Peer pressure
6237	HSB0177	No	1	-
6238	HSB0178	No	2	Others
6239	HSB0179	No	2	-
6240	HSB0180	No	2	-

No	Patient ID	Other substances	No of substances abused at a time	Reason for substance use initiation
6241	HSB0181	No	1	Peer pressure
6242	HSB0182	No	1	Peer pressure
6243	HSB0183	No	2	Relationship, Peer pressure
6244	HSB0184	No	2	Peer pressure
6245	HSB0185	No	2	Relationship, Peer pressure
6246	HSB0186	No	1	-
6247	HSB0187	No	3	Family issue, Relationship, Peer pressure
6248	HSB0188	No	3	-
6249	HSB0189	No	4	Joy seeking
6250	HSB0190	No	2	Peer pressure
6251	HSB0191	No	3	Family issue
6252	HSB0192	No	2	-
6253	HSB0193	No	2	Peer pressure
6254	HSB0194	No	2	Family issue
6255	HSB0196	No	3	-
6256	HSB0199	No	1	Relaxation
6257	HSB0200	No	2	-
6258	HSB0201	No	3	-
6259	HSB0202	No	3	-
6260	HSB0203	No	3	-
6261	HSB0204	No	4	Others
6262	HSB0206	Yes	5	-
6263	HSB0207	No	2	-
6264	HSB0208	No	2	Others
6265	HSB0209	No	2	Peer pressure
6266	HSB0210	Yes	4	-
6267	HSB0211	No	2	-
6268	HSB0213	No	2	-
6269	HSB0214	No	2	-
6270	HSB0215	No	2	-
6271	HSB0216	No	3	Joy seeking
6272	HSB0217	No	2	Job problem, Peer pressure
6273	HSB0219	No	1	-
6274	HSB0220	No	2	-
6275	HSB0221	No	3	-
6276	HSB0222	No	1	-
6277	HSB0223	No	1	Peer pressure
6278	HSB0224	No	1	Relationship

No	Patient ID	Other substances	No of substances abused at a time	Reason for substance use initiation
6279	HSB0225	No	1	Others
6280	HSB0226	No	1	-
6281	HSB0227	No	2	-
6282	HSB0228	No	1	Family issue
6283	HSB0229	No	4	-
6284	HSB0230	No	1	-
6285	HSB0231	No	3	Peer pressure, Others
6286	HSB0232	No	3	-
6287	HSB0233	No	2	-
6288	HSB0234	No	1	Peer pressure
6289	HSB0235	No	1	-
6290	HSB0236	No	2	Joy seeking
6291	HSB0237	Yes	1	-
6292	HSB0238	No	2	-
6293	HSB0239	No	1	Others
6294	HSB0240	Yes	5	-
6295	HSB0241	No	4	-
6296	HSB0242	No	2	-
6297	HSB0243	No	1	-
6298	HSB0244	No	2	Relaxation, Others
6299	HSB0245	No	1	Peer pressure, Others
6300	HSB0246	No	2	-
6301	HSB0247	No	1	-
6302	HSB0248	No	1	Peer pressure
6303	HSB0249	No	1	-
6304	HSB0250	No	2	-
6305	HSB0251	No	2	-
6306	HSB0252	No	1	-
6307	HSB0253	No	2	-
6308	HSB0254	No	1	Job problem
6309	HSB0255	No	1	Relationship, Peer pressure

No	Patient ID	Other substances	No of substances abused at a time	Reason for substance use initiation
6310	HSB0256	No	2	Job problem, Peer pressure
6311	HSB0257	No	3	-
6312	HSB0258	No	1	Peer pressure
6313	HSB0259	No	3	-
6314	HSB0260	No	2	-
6315	HSB0262	No	1	-
6316	HSB0263	No	2	Joy seeking, Others
6317	HSB0264	No	3	-
6318	HSB0265	No	1	-
6319	HSB0266	No	3	Peer pressure
6320	HSB0268	No	4	Peer pressure
6321	HSB0269	No	4	Others
6322	HSB0270	No	2	Peer pressure
6323	HSB0271	No	4	-
6324	HSB0272	No	1	-
6325	HSB0273	Yes	4	-
6326	HSB0274	No	2	-
6327	HSB0275	No	2	-
6328	HSB0276	No	1	-
6329	HSB0277	No	1	-
6330	HSB0278	No	1	-
6331	HSB0279	No	2	-
6332	HSB0280	No	2	-
6333	HSB0281	No	1	-
6334	HSB0282	No	2	Job problem, Joy seeking, Peer pressure
6335	HSB0283	No	1	-
6336	HSB0284	No	2	-
6337	HSB0285	No	3	-
6338	HSB0286	Yes	4	Family issue, Peer pressure
6339	HSB0287	No	1	Peer pressure
6340	HSB0288	No	1	Family issue
6341	HSB0289	No	2	-
6342	HSB0290	No	2	Relaxation
6343	HSB0292	No	1	-
6344	HSB0293	No	4	-

No	Patient ID	Other substances	No of substances abused at a time	Reason for substance use initiation
6345	HSB0294	No	1	Peer pressure
6346	HSB0295	No	1	-
6347	HSB0296	No	1	-
6348	HSB0297	No	1	-
6349	HSB0298	No	1	-
6350	HSB0299	No	1	-
6351	HSB0300	No	2	Peer pressure
6352	HSB0301	No	1	Job problem
6353	HSB0302	No	1	-
6354	HSB0303	No	1	Peer pressure
6355	HSB0305	No	3	-
6356	HSB0306	No	2	-
6357	HSB0307	Yes	4	-
6358	HSB0308	No	1	-
6359	HSB0309	No	1	Relationship, Relaxation
6360	HSB0310	No	5	-
6361	HSB0312	No	2	-
6362	HSB0314	No	1	-
6363	HSB0315	No	1	Peer pressure
6364	HSB0316	No	3	-
6365	HSB0317	No	1	-
6366	HSB0318	No	1	-
6367	HSB0319	No	2	-
6368	HSB0320	No	1	-
6369	HSB0323	No	4	-
6370	HSB0324	Yes	3	Peer pressure
6371	HSB0325	No	1	-
6372	HSB0326	No	3	-
6373	HSB0327	No	2	-
6374	HSB0329	No	2	-
6375	HSB0330	No	1	-
6376	HSB0331	No	1	-
6377	HSB0334	No	2	Others
6378	HSB0336	No	1	-
6379	HSB0337	No	1	-
6380	HSB0339	No	3	-
6381	HSB0340	No	1	-
6382	HSB0341	No	3	-
6383	HSB0342	No	2	-

No	Patient ID	Other substances	No of substances abused at a time	Reason for substance use initiation
6384	HSB0343	No	1	Joy seeking
6385	HSB0344	No	3	Peer pressure, Others
6386	HSB0345	Yes	3	-
6387	HSB0346	No	1	-
6388	HSB0348	No	1	-
6389	HSB0349	No	1	-
6390	HSB0351	No	2	-
6391	HSB0352	No	1	Peer pressure
6392	HSB0353	No	1	-
6393	HSB0354	No	2	-
6394	HSB0355	No	2	Family issue, Peer pressure
6395	HSB0356	No	3	-
6396	HSB0357	No	1	-
6397	HSB0359	No	1	-
6398	HSB0360	No	1	-
6399	HSB0361	No	3	-
6400	HSB0362	No	2	-
6401	HSB0364	No	3	-
6402	HSB0365	No	3	-
6403	HSB0366	Yes	2	-
6404	HSB0367	No	1	-
6405	HSB0368	No	2	-
6406	HSB0369	Yes	4	Peer pressure
6407	HSB0370	No	1	-
6408	HSB0371	No	1	Others
6409	HSB0373	No	1	-
6410	HSB0374	No	2	-
6411	HSB0375	No	6	-
6412	HSB0376	No	1	-
6413	HSB0378	No	2	-
6414	HSB0379	No	1	Family issue
6415	HSB0380	No	1	-
6416	HSB0381	No	1	-
6417	HSB0382	No	1	-
6418	HSB0383	No	1	-
6419	HSB0384	No	2	-
6420	HSB0385	No	1	-
6421	HSB0386	Yes	6	-
6422	HSB0387	No	2	Others

No	Patient ID	Other substances	No of substances abused at a time	Reason for substance use initiation
6423	HSB0388	No	2	Peer pressure
6424	HSB0389	No	1	-
6425	HSB0390	No	1	-
6426	HSB0392	No	1	-
6427	HSB0393	No	1	-
6428	HSB0394	No	3	-
6429	HSB0395	No	2	-
6430	HSB0397	No	1	-
6431	HSB0398	No	1	-
6432	HSB0399	No	1	-
6433	HSB0400	No	2	-
6434	HSB0401	No	1	-
6435	HSB0403	No	2	-
6436	HSB0404	No	1	Job problem, Peer pressure
6437	HSB0405	No	2	-
6438	HSB0406	No	3	-
6439	HSB0407	No	1	-
6440	HSB0408	No	1	Relationship
6441	HSB0409	No	1	-
6442	HSB0411	No	1	-
6443	HSB0412	No	3	-
6444	HSB0413	No	1	-
6445	HSB0414	No	1	-
6446	HSB0415	No	1	-
6447	HSB0416	No	1	-
6448	HSB0418	No	1	-
6449	HSB0419	No	3	-
6450	HSB0421	No	1	-
6451	HSB0422	No	2	Family issue
6452	HSB0423	No	1	-
6453	HSB0424	No	1	-
6454	HSB0425	Yes	3	Joy seeking
6455	HSB0426	Yes	3	-
6456	HSB0427	No	1	Relaxation
6457	HSB0428	No	2	-

No	Patient ID	Other substances	No of substances abused at a time	Reason for substance use initiation
6458	HSB0429	No	2	-
6459	HSB0430	No	1	-
6460	HSB0433	No	2	Peer pressure, Others
6461	HSB0434	No	2	Job problem, Others
6462	HSB0435	No	1	-
6463	HSB0436	No	4	-
6464	HSB0437	No	3	-
6465	HSB0438	No	1	-
6466	HSB0439	No	3	Joy seeking
6467	HSB0440	No	2	Family issue
6468	HSB0442	No	4	Peer pressure
6469	HSB0443	No	2	-
6470	HSB0444	No	2	-
6471	HSB0446	Yes	3	-
6472	HSB0447	No	1	-
6473	HSB0448	No	1	-
6474	HSB0450	No	1	-
6475	HSB0451	No	1	-
6476	HSB0453	No	3	Family issue
6477	HSB0454	No	1	-
6478	HSB0456	No	1	-
6479	HSB0458	No	2	-
6480	HSB0459	No	1	-
6481	HSB0460	No	1	-
6482	HSB0461	No	1	-
6483	HSB0462	No	1	Peer pressure
6484	HSB0463	No	2	-
6485	HSB0464	No	1	-
6486	HSB0465	No	4	Peer pressure
6487	HSB0466	No	2	Peer pressure
6488	HSB0467	Yes	2	Joy seeking, Peer pressure
6489	HSB0468	No	1	Others
6490	HSB0469	No	1	-
6491	HSB0470	No	2	Peer pressure
6492	HSB0471	No	1	Peer pressure, Others

No	Patient ID	Other substances	No of substances abused at a time	Reason for substance use initiation
6493	HSB0473	No	2	-
6494	HSB0475	No	2	-
6495	HSB0476	Yes	4	Others
6496	HSB0477	No	2	Peer pressure
6497	HSB0478	No	3	Peer pressure
6498	HSB0479	No	1	-
6499	HSB0480	No	3	-
6500	HSB0482	No	1	Others
6501	HSB0483	No	2	-
6502	HSB0484	No	1	-
6503	HSB0485	No	2	-
6504	HSB0486	No	1	Family issue
6505	HSB0487	No	1	Peer pressure
6506	HSB0488	No	2	-
6507	HSB0489	No	2	-
6508	HSB0490	No	1	-
6509	HSB0492	No	1	-
6510	HSB0493	No	2	-
6511	HSB0494	No	3	Relaxation
6512	HSB0495	No	2	-
6513	HSB0498	No	3	-
6514	HSB0500	No	1	-
6515	HSB0501	No	2	Joy seeking, Peer pressure
6516	HSB0502	No	2	-
6517	HSB0503	No	1	-
6518	HSB0504	No	1	-
6519	HSB0505	No	1	-
6520	HSB0506	No	3	-
6521	HSB0507	No	2	Family issue, Relationship, Peer pressure
6522	HSB0508	No	3	-
6523	HSB0509	No	5	-
6524	HSB0510	No	2	Joy seeking, Peer pressure

No	Patient ID	Other substances	No of substances abused at a time	Reason for substance use initiation
6525	HSB0511	No	2	Others
6526	HSB0513	No	2	Job problem, Relationship, Relaxation
6527	HSB0514	Yes	5	-
6528	HSB0516	No	1	-
6529	HSB0517	Yes	3	-
6530	HSB0520	No	1	Family issue, Job problem, Relationship
6531	HSB0522	No	1	Peer pressure
6532	HSB0523	No	2	Peer pressure
6533	HSB0525	No	1	-
6534	HSB0527	No	3	-
6535	HSB0529	Yes	5	Others
6536	HSB0530	No	1	Others
6537	HSB0531	No	3	Job problem
6538	HSB0532	No	1	-
6539	HSB0533	No	2	Peer pressure, Others
6540	HSB0535	No	1	-
6541	HSB0536	No	1	-
6542	HSB0537	No	6	-
6543	HSB0539	No	1	-
6544	HSB0541	No	2	-
6545	HSB0544	No	3	Joy seeking, Peer pressure
6546	HSB0547	No	3	-
6547	HSB0548	No	1	-
6548	HSB0549	Yes	4	Family issue, Peer pressure, Others
6549	HSB0552	Yes	4	Others
6550	HSB0555	No	2	-
6551	HSB0556	No	2	-
6552	HSB0557	No	2	-
6553	HSB0558	No	2	Peer pressure
6554	HSB0559	No	3	Family issue, Relationship
6555	HSB0561	No	1	-
6556	HSB0562	No	1	-

No	Patient ID	Other substances	No of substances abused at a time	Reason for substance use initiation
6557	HSB0563	No	3	-
6558	HSB0564	Yes	7	Others
6559	HSB0565	No	3	-
6560	HSB0566	No	1	-
6561	HSB0567	No	1	-
6562	HSB0568	No	1	Others
6563	HSB0569	No	1	-
6564	HSB0570	No	1	-
6565	HSB0571	No	1	Relaxation, Peer pressure
6566	HSB0572	No	1	-
6567	HSB0573	No	2	-
6568	HSB0574	No	1	-
6569	HSB0576	No	1	-
6570	HSB0577	No	4	-
6571	HSB0578	No	1	Peer pressure
6572	HSB0579	No	1	-
6573	HSB0580	No	1	Joy seeking
6574	HSB0581	No	1	-
6575	HSB0582	No	1	-
6576	HSB0583	No	1	-
6577	HSB0584	No	2	-
6578	HSB0585	No	1	-
6579	HSB0586	No	1	-
6580	HSB0586	No	1	-
6581	HSB0588	No	1	Others
6582	HSB0589	No	1	-
6583	HSB0590	No	1	Others
6584	HSB0591	No	3	-
6585	HSB0595	No	2	-
6586	HSB0597	No	2	-
6587	HSB0598	No	1	-
6588	HSB0599	No	2	-
6589	HSB0600	No	2	Peer pressure
6590	HSB0601	No	1	-
6591	HSB0602	Yes	4	-
6592	HSB0603	No	2	Relaxation

No	Patient ID	Other substances	No of substances abused at a time	Reason for substance use initiation
6593	HSB0605	No	1	Relaxation
6594	HSB0606	No	3	Relationship
6595	HSB0607	No	1	-
6596	HSB0608	No	1	-
6597	HSB0609	No	2	Peer pressure
6598	HSB0610	Yes	3	-
6599	HSB0612	No	2	-
6600	HSB0613	No	1	-
6601	HSB0614	No	2	-
6602	HSB0615	No	2	-
6603	HSB0616	No	1	-
6604	HSB0617	No	1	-
6605	HSB0619	No	1	-
6606	HSB0620	No	2	-
6607	HSB0621	No	1	Health
6608	HSB0623	No	2	-
6609	HSB0624	No	2	-
6610	HSB0625	No	4	Peer pressure
6611	HSB0626	No	1	-
6612	HSB0627	No	1	-
6613	HSB0628	No	2	-
6614	HSB0629	No	1	-
6615	HSB0631	No	6	-
6616	HSB0632	No	1	-
6617	HSB0633	No	4	-
6618	HSB0635	No	2	-
6619	HSB0636	No	2	-
6620	HSB0637	No	2	Joy seeking, Peer pressure
6621	HSB0639	No	2	-
6622	HSB0642	No	1	-
6623	HSB0643	No	1	-
6624	HSB0644	No	3	-
6625	HSB0645	No	3	-
6626	HSB0646	No	1	Relationship
6627	HSB0648	No	2	Others

No	Patient ID	Other substances	No of substances abused at a time	Reason for substance use initiation
6628	HSB0651	No	2	-
6629	HSB0652	No	2	-
6630	HSB0653	Yes	2	Relaxation, Others
6631	HSB0655	No	3	-
6632	HSB0657	No	2	Others
6633	HSB0658	No	2	Peer pressure
6634	HSB0659	No	1	-
6635	HSB0660	Yes	4	-
6636	HSB0661	No	1	-
6637	HSB0662	No	2	-
6638	HSB0665	No	2	-
6639	HSB0666	No	1	-
6640	HSB0667	No	1	Peer pressure
6641	HSB0668	No	2	Relationship, Others
6642	HSB0669	No	1	-
6643	HSB0670	No	1	-
6644	HSB0673	No	5	-
6645	HSB0674	No	2	Peer pressure
6646	HSB0677	No	3	-
6647	HSB0678	No	4	-
6648	HSB0680	No	1	-
6649	HSB0681	No	2	Others
6650	HSB0682	No	3	Joy seeking, Peer pressure
6651	HSB0684	No	2	Peer pressure
6652	HSB0685	No	4	-
6653	HSB0686	No	2	-
6654	HSB0690	No	1	Peer pressure
6655	HSB0691	No	4	Peer pressure
6656	HSB0692	No	2	Relationship, Peer pressure
6657	HSB0694	No	2	Peer pressure
6658	HSB0696	No	4	Others
6659	HSB0697	No	4	-
6660	HSB0698	No	2	Relaxation
6661	HSB0699	No	1	Peer pressure
6662	HSB0700	No	3	-
6663	HSB0701	No	2	-

No	Patient ID	Other substances	No of substances abused at a time	Reason for substance use initiation
6664	HSB0704	No	3	-
6665	HSB0705	No	1	Health, Others
6666	HSB0712	No	3	Peer pressure
6667	HSB0713	No	2	-
6668	HSB0715	No	2	-
6669	HSB0717	No	4	-
6670	HSB0719	No	3	-
6671	HSB0720	No	1	Others
6672	HSB0723	No	3	Job problem
6673	HSB0726	No	4	Others
6674	HSB0727	No	2	-
6675	HSB0728	No	3	-
6676	HSB0730	No	2	Joy seeking, Others
6677	HSB0732	No	2	-
6678	HSB0733	No	2	-
6679	HSB0734	No	1	-
6680	HSB0735	Yes	5	-
6681	HSB0736	No	3	-
6682	HSB0738	No	2	-
6683	HSB0739	No	2	-
6684	HSB0742	No	2	Peer pressure
6685	HSB0748	Yes	6	Peer pressure
6686	HSB0749	No	2	-
6687	HSB0751	No	3	Joy seeking
6688	HSB0752	No	2	-
6689	HSB0755	No	1	Joy seeking
6690	HSB0756	No	2	Peer pressure
6691	HSB0757	No	4	-
6692	HSB0758	No	3	Peer pressure
6693	HSB0759	No	5	-
6694	HSB0761	No	1	-
6695	HSB0762	No	2	-
6696	HSB0763	No	5	Peer pressure
6697	HSB0764	No	4	-
6698	HSB0765	No	1	-
6699	HSB0766	No	2	Family issue, Others
6700	HSB0768	Yes	5	-

No	Patient ID	Other substances	No of substances abused at a time	Reason for substance use initiation
6701	HSB0770	No	2	-
6702	HSB0771	No	1	-
6703	HSNZ0001	No	5	Health, Relaxation
6704	HSNZ0002	No	3	Relaxation, Joy seeking
6705	HSNZ0003	No	3	Relaxation, Others
6706	HSNZ0004	No	8	Relaxation, Peer pressure
6707	HSNZ0005	No	2	Relaxation, Peer pressure
6708	HSNZ0007	No	3	Peer pressure
6709	HSNZ0009	No	2	Peer pressure
6710	HSNZ0010	No	2	Relaxation, Peer pressure
6711	HSNZ0011	No	3	Peer pressure
6712	HSNZ0012	No	2	Others
6713	HSNZ0013	No	2	Peer pressure
6714	HSNZ0014	No	1	Peer pressure
6715	HSNZ0015	No	2	Joy seeking, Peer pressure
6716	HSNZ0016	No	2	Others
6717	HSNZ0017	No	1	Peer pressure
6718	HSNZ0018	No	2	Joy seeking, Others
6719	HSNZ0019	No	1	Peer pressure
6720	HSNZ0020	No	2	Relaxation, Peer pressure
6721	HSNZ0021	No	3	Peer pressure
6722	HSNZ0022	No	2	Relaxation, Peer pressure

No	Patient ID	Other substances	No of substances abused at a time	Reason for substance use initiation
6723	HSNZ0023	No	1	-
6724	HSNZ0024	No	4	Relaxation, Joy seeking
6725	HSNZ0026	No	2	Relaxation, Joy seeking, Peer pressure
6726	HSNZ0027	No	2	-
6727	HSNZ0028	No	2	Peer pressure
6728	HSNZ0030	No	1	Peer pressure
6729	HSNZ0031	No	1	Others
6730	HSNZ0035	No	2	Joy seeking, Peer pressure
6731	HSNZ0036	No	5	Others
6732	HSNZ0038	No	2	Peer pressure
6733	HSNZ0040	No	3	Peer pressure
6734	HSNZ0042	No	2	Peer pressure
6735	HSNZ0043	No	2	Relaxation, Joy seeking, Peer pressure, Others
6736	HSNZ0044	No	2	Peer pressure
6737	HSNZ0048	No	2	Job problem, Relaxation, Joy seeking, Peer pressure
6738	HSNZ0051	No	2	Joy seeking, Peer pressure, Others
6739	HSNZ0053	No	2	Relaxation
6740	HSNZ0054	No	2	Joy seeking, Peer pressure
6741	HSNZ0055	No	1	Joy seeking, Others
6742	HSNZ0057	No	1	-
6743	HSNZ0061	No	1	-

No	Patient ID	Other substances	No of substances abused at a time	Reason for substance use initiation
6744	HSNZ0062	No	1	Relaxation
6745	HSNZ0063	No	2	Peer pressure
6746	HSNZ0065	No	1	Peer pressure
6747	HSNZ0066	No	2	Joy seeking
6748	HSNZ0067	No	3	Relaxation
6749	HSNZ0069	No	1	-
6750	HSNZ0070	No	2	Health, Relaxation
6751	HSNZ0072	No	1	Peer pressure
6752	HSNZ0074	No	1	-
6753	HSNZ0075	Yes	1	Joy seeking
6754	HSNZ0077	No	3	Peer pressure
6755	HSNZ0081	No	2	-
6756	HSNZ0082	No	3	Relaxation, Others
6757	HSNZ0083	No	2	Health
6758	HSNZ0084	No	1	-
6759	HSNZ0085	No	1	-
6760	HSNZ0086	No	1	Relaxation
6761	HSNZ0087	No	3	Relaxation
6762	HSNZ0091	No	1	-
6763	HSNZ0094	No	2	-
6764	HSNZ0095	No	1	-

No	Patient ID	Other substances	No of substances abused at a time	Reason for substance use initiation
6765	HSNZ0098	No	2	Relaxation
6766	HSNZ0099	Yes	1	-
6767	HSNZ0103	No	3	-
6768	HSNZ0105	No	2	Relaxation
6769	HSNZ0106	No	2	Joy seeking
6770	HSNZ0107	No	1	-
6771	HSNZ0108	No	2	-
6772	HSNZ0109	No	1	-
6773	HSNZ0110	No	1	-
6774	HSNZ0115	No	2	-
6775	HSNZ0119	No	3	-
6776	HSNZ0123	No	2	-
6777	HSNZ0124	No	4	Relaxation
6778	HSNZ0125	No	2	-
6779	HSNZ0131	No	1	-
6780	HSNZ0134	No	4	-
6781	HSNZ0135	No	2	Relationship
6782	HSNZ0136	No	1	-
6783	HSNZ0137	No	1	Peer pressure
6784	HSNZ0139	No	2	Peer pressure
6785	HSNZ0141	No	2	-

No	Patient ID	Other substances	No of substances abused at a time	Reason for substance use initiation
6786	HSNZ0142	No	2	Others
6787	HSNZ0149	No	1	Family issue, Relaxation
6788	HSNZ0153	No	1	-
6789	HSNZ0154	No	1	-
6790	HSNZ0156	No	2	Peer pressure
6791	HSNZ0157	No	2	-
6792	HSNZ0159	No	2	-
6793	HSNZ0164	No	2	-
6794	HSNZ0165	No	1	-
6795	HSNZ0166	No	2	Peer pressure
6796	HSNZ0167	No	1	-
6797	HSNZ0169	No	3	Peer pressure
6798	HSNZ0170	No	2	-
6799	HSNZ0171	No	1	Peer pressure
6800	HSNZ0172	No	2	-
6801	HSNZ0173	No	2	Joy seeking
6802	HSNZ0174	No	2	-
6803	HSNZ0180	No	2	Relaxation
6804	HSNZ0182	No	1	-
6805	HSNZ0186	No	1	Family issue
6806	HSNZ0194	No	1	-

No	Patient ID	Other substances	No of substances abused at a time	Reason for substance use initiation
6807	HSNZ0196	No	1	Others
6808	HSNZ0197	No	2	Others
6809	HSNZ0200	No	2	Relaxation, Peer pressure
6810	HSNZ0201	No	1	-
6811	HSNZ0205	No	2	Relaxation, Others
6812	HSNZ0210	No	2	-
6813	HSNZ0214	No	2	Joy seeking, Peer pressure
6814	HSNZ0216	No	2	Peer pressure
6815	HSNZ0217	No	1	-
6816	HSNZ0218	No	2	-
6817	HSNZ0219	No	2	-
6818	HSNZ0221	No	2	-
6819	HSNZ0224	No	2	Peer pressure
6820	HSNZ0227	No	3	Peer pressure
6821	HSNZ0228	No	3	Others
6822	HSNZ0232	No	2	Relaxation
6823	HSNZ0234	No	1	-
6824	HSNZ0235	No	2	-
6825	HSNZ0239	No	1	-
6826	HSNZ0242	No	1	-
6827	HSNZ0243	No	1	Peer pressure

No	Patient ID	Other substances	No of substances abused at a time	Reason for substance use initiation
6828	HSNZ0244	No	2	-
6829	HSNZ0245	No	2	Joy seeking, Peer pressure
6830	HSNZ0247	No	1	Relaxation, Others
6831	HSNZ0248	No	1	-
6832	HSNZ0249	No	2	Peer pressure
6833	HSNZ0251	No	2	-
6834	HSNZ0252	No	1	-
6835	HSNZ0253	No	1	-
6836	HSNZ0254	No	1	Peer pressure
6837	HSNZ0255	No	2	Peer pressure
6838	HSNZ0258	No	2	Joy seeking
6839	HSNZ0259	No	3	Others
6840	HSNZ0261	No	2	Others
6841	HSNZ0263	No	1	-
6842	HSNZ0264	Yes	2	Joy seeking, Peer pressure
6843	HSNZ0265	No	1	-
6844	HSNZ0266	No	2	-
6845	HSNZ0267	No	2	Others
6846	HSNZ0268	No	3	-
6847	HSNZ0269	No	1	-
6848	HSNZ0274	No	1	Others

No	Patient ID	Other substances	No of substances abused at a time	Reason for substance use initiation
6849	HSNZ0275	No	2	Peer pressure
6850	HSNZ0277	No	2	Health, Relaxation, Joy seeking
6851	HSNZ0279	No	3	-
6852	HSNZ0281	No	1	-
6853	HSNZ0283	No	1	-
6854	HSNZ0284	No	1	Relaxation, Joy seeking, Peer pressure
6855	HSNZ0287	No	2	-
6856	HSNZ0288	No	1	Relationship
6857	HSNZ0291	No	3	Family issue, Relaxation
6858	HSNZ0293	Yes	2	Relaxation
6859	HSNZ0294	No	2	-
6860	HSNZ0297	No	1	-
6861	HSNZ0298	No	2	Peer pressure
6862	HSNZ0301	Yes	1	Relaxation
6863	HSNZ0307	No	1	Peer pressure
6864	HSNZ0311	No	2	-
6865	HSNZ0314	No	1	-
6866	HSNZ0318	No	3	Peer pressure
6867	HSNZ0320	No	2	-
6868	HSNZ0321	No	1	Joy seeking
6869	HSNZ0322	No	1	-

No	Patient ID	Other substances	No of substances abused at a time	Reason for substance use initiation
6870	HSNZ0324	No	2	-
6871	HSNZ0325	No	3	Relaxation
6872	HSNZ0326	No	1	Peer pressure
6873	HSNZ0327	No	1	Relaxation
6874	HSNZ0328	No	2	Others
6875	HSNZ0330	No	3	Relaxation, Peer pressure, Others
6876	HSNZ0334	No	1	Relaxation, Joy seeking
6877	HSNZ0335	No	2	-
6878	HSNZ0336	No	3	Joy seeking, Peer pressure
6879	HSNZ0337	No	1	-
6880	HSNZ0338	No	1	Job problem, Relaxation
6881	HSNZ0339	No	1	-
6882	HSNZ0343	No	1	-
6883	HSNZ0345	No	1	Family issue, Relaxation
6884	HSNZ0346	No	2	Family issue, Relaxation
6885	HSNZ0348	No	1	-
6886	HSNZ0349	No	2	-
6887	HSNZ0351	No	2	Joy seeking
6888	HSNZ0352	No	1	-
6889	HSNZ0353	No	1	-
6890	HSNZ0354	No	5	-

No	Patient ID	Other substances	No of substances abused at a time	Reason for substance use initiation
6891	HSNZ0355	No	1	-
6892	HSNZ0356	No	2	-
6893	HSNZ0359	No	2	-
6894	HSNZ0360	No	1	Joy seeking
6895	HSNZ0361	No	1	Peer pressure
6896	HSNZ0362	No	2	Relaxation
6897	HSNZ0363	No	1	-
6898	HSNZ0365	No	2	-
6899	HSNZ0366	No	1	-
6900	HSNZ0367	No	1	-
6901	HSNZ0369	No	1	-
6902	HSNZ0372	No	2	Others
6903	HSNZ0374	No	2	Peer pressure
6904	HSNZ0376	No	1	-
6905	HSNZ0378	No	1	-
6906	HSNZ0379	No	2	Others
6907	HSNZ0380	No	3	-
6908	HSNZ0382	No	2	Peer pressure
6909	HSNZ0383	No	4	-
6910	HSNZ0384	No	1	Joy seeking, Peer pressure
6911	HSNZ0386	No	3	-

No	Patient ID	Other substances	No of substances abused at a time	Reason for substance use initiation
6912	HSNZ0387	No	1	Others
6913	HSNZ0388	No	1	Peer pressure
6914	HSNZ0389	No	2	-
6915	HSNZ0390	No	2	Family issue, Peer pressure, Others
6916	HSNZ0391	No	1	-
6917	HSNZ0392	No	4	Family issue, Peer pressure
6918	HSNZ0394	No	2	Peer pressure
6919	HSNZ0395	No	2	Peer pressure
6920	HSNZ0400	No	1	-
6921	HSNZ0401	No	2	Job problem
6922	HSNZ0402	No	2	Peer pressure
6923	HSNZ0404	No	2	-
6924	HSNZ0405	No	5	Peer pressure
6925	HSNZ0408	No	2	Relaxation
6926	HSNZ0409	No	2	-
6927	HSNZ0413	No	2	Joy seeking
6928	HSNZ0414	No	3	Peer pressure
6929	HSNZ0416	No	3	Peer pressure, Others
6930	HSNZ0418	No	2	Peer pressure
6931	HSNZ0419	No	1	-
6932	HSNZ0420	No	1	-

No	Patient ID	Other substances	No of substances abused at a time	Reason for substance use initiation
6933	HSNZ0421	No	1	-
6934	HSNZ0424	No	1	-
6935	HSNZ0425	No	1	-
6936	HSNZ0426	No	2	-
6937	HSNZ0427	No	1	-
6938	HSNZ0428	No	1	Relaxation, Others
6939	HSNZ0429	No	1	Joy seeking, Peer pressure
6940	HSNZ0430	No	1	Job problem, Relationship
6941	HSNZ0431	No	2	Joy seeking, Peer pressure
6942	HSNZ0432	No	1	Joy seeking
6943	HSNZ0434	No	1	-
6944	HSNZ0435	No	1	-
6945	HSNZ0437	No	1	Joy seeking
6946	HSNZ0438	No	1	Peer pressure
6947	HSNZ0439	No	2	-
6948	HSNZ0441	No	1	-
6949	HSNZ0442	No	3	Job problem
6950	HSNZ0443	No	1	-
6951	HSNZ0444	No	2	Peer pressure
6952	HSNZ0445	No	2	Relationship, Peer pressure, Others
6953	HSNZ0446	No	1	-

No	Patient ID	Other substances	No of substances abused at a time	Reason for substance use initiation
6954	HSNZ0447	No	1	-
6955	HSNZ0448	No	1	-
6956	HSNZ0449	No	2	-
6957	HSNZ0450	No	3	-
6958	HSNZ0451	Yes	4	-
6959	HSNZ0452	No	2	Joy seeking, Peer pressure
6960	HSNZ0453	No	2	-
6961	HSNZ0455	No	1	-
6962	HSNZ0457	No	4	-
6963	HSNZ0460	No	1	-
6964	HSNZ0462	No	5	Family issue, Peer pressure
6965	HSNZ0464	No	2	Peer pressure
6966	HSNZ0465	No	1	Peer pressure
6967	HSNZ0466	No	1	-
6968	HSNZ0468	No	2	Job problem
6969	HSNZ0469	No	3	Peer pressure
6970	HSNZ0471	No	2	Family issue, Job problem
6971	HSNZ0472	No	2	-
6972	HSNZ0474	No	2	Peer pressure
6973	HSNZ0476	No	1	-
6974	HSNZ0480	No	2	Peer pressure

No	Patient ID	Other substances	No of substances abused at a time	Reason for substance use initiation
6975	HSNZ0481	No	1	-
6976	HSNZ0482	No	3	Joy seeking, Peer pressure
6977	HSNZ0483	No	2	-
6978	HSNZ0484	No	1	-
6979	HSNZ0485	No	2	Peer pressure
6980	HSNZ0486	No	2	Family issue, Others
6981	HSNZ0487	No	2	Health
6982	HSNZ0488	No	2	Family issue, Peer pressure, Others
6983	HSNZ0489	No	3	Others
6984	HSNZ0490	No	3	-
6985	HSNZ0492	No	2	-
6986	HSNZ0494	No	5	Relaxation, Others
6987	HSNZ0495	No	1	Family issue
6988	HSNZ0496	No	1	Joy seeking
6989	HSNZ0497	No	2	-
6990	HSNZ0498	No	4	Peer pressure
6991	HSNZ0499	No	4	Relaxation
6992	HSNZ0503	No	2	Job problem
6993	HSNZ0505	No	3	-
6994	HSNZ0506	No	2	-
6995	HSNZ0507	No	3	Others

No	Patient ID	Other substances	No of substances abused at a time	Reason for substance use initiation
6996	HSNZ0508	No	2	-
6997	HSNZ0509	No	1	-
6998	HSNZ0510	No	1	-
6999	HSNZ0511	No	1	-
7000	HSNZ0512	No	2	-
7001	HSNZ0514	No	1	-
7002	HSNZ0515	No	2	Job problem
7003	HSNZ0516	No	1	Job problem, Joy seeking, Others
7004	HSNZ0517	No	2	-
7005	HSNZ0519	No	2	-
7006	HSNZ0522	No	1	-
7007	HSNZ0523	No	1	-
7008	HSNZ0526	No	3	Others
7009	HSNZ0527	No	1	-
7010	HSNZ0528	No	4	-
7011	HSNZ0529	No	2	Peer pressure
7012	HSNZ0530	No	2	-
7013	HSNZ0532	No	2	Peer pressure
7014	HSNZ0533	No	1	-
7015	HSNZ0534	No	1	-
7016	HSNZ0535	No	2	Peer pressure

No	Patient ID	Other substances	No of substances abused at a time	Reason for substance use initiation
7017	HSNZ0537	No	1	-
7018	HSNZ0539	No	1	Peer pressure
7019	HSNZ0540	No	2	Joy seeking
7020	HSNZ0542	No	2	-
7021	HSNZ0544	No	1	-
7022	HSNZ0547	No	2	Peer pressure
7023	HSNZ0550	No	2	-
7024	HSNZ0552	No	4	-
7025	HSNZ0553	No	4	-
7026	HSNZ0554	No	4	Joy seeking, Peer pressure
7027	HSNZ0555	No	1	-
7028	HSNZ0562	No	1	-
7029	HSNZ0567	No	1	-
7030	HSNZ0568	No	1	-
7031	HSNZ0569	No	2	-
7032	HSNZ0570	No	1	-
7033	HSNZ0571	No	5	-
7034	HSNZ0574	No	1	-
7035	HSNZ0575	No	4	Peer pressure
7036	HSNZ0577	No	2	Joy seeking, Peer pressure
7037	HSNZ0578	No	2	-

No	Patient ID	Other substances	No of substances abused at a time	Reason for substance use initiation
7038	HSNZ0580	No	2	-
7039	HSNZ0583	No	3	Joy seeking
7040	HTAA0001	No	2	Health, Family issue, Joy seeking, Others
7041	HTAA0004	No	1	Family issue, Joy seeking, Peer pressure
7042	HTAA0005	Yes	3	Joy seeking, Financial problem
7043	HTAA0007	No	5	Peer pressure, Others
7044	HTAA0009	No	6	-
7045	HTAA0010	No	2	Job problem, Relaxation
7046	HTAA0011	No	5	Family issue, Relationship, Relaxation
7047	HTAA0012	No	1	-
7048	HTAA0013	Yes	10	-
7049	HTAA0015	No	2	Job problem
7050	HTAA0016	Yes	10	Joy seeking
7051	HTAA0017	No	5	Joy seeking, Peer pressure
7052	HTAA0018	Yes	10	Relaxation, Peer pressure, Others
7053	HTAA0020	No	1	Family issue, Relationship, Peer pressure
7054	HTAA0021	No	1	Peer pressure
7055	HTAA0022	No	1	Relationship
7056	HTAA0024	No	7	Relaxation, Joy seeking, Peer pressure
7057	HTAA0025	No	2	Relationship, Peer pressure
7058	HTAA0026	No	3	Health, Others

No	Patient ID	Other substances	No of substances abused at a time	Reason for substance use initiation
7059	HTAA0027	No	3	-
7060	HTAA0029	No	3	Health, Relaxation, Joy seeking, Peer pressure
7061	HTAA0030	No	1	Health, Job problem, Peer pressure
7062	HTAA0032	No	4	Health, Family issue, Relaxation, Others
7063	HTAA0034	No	4	Relationship
7064	HTAA0036	No	1	Relaxation, Joy seeking, Peer pressure
7065	HTAA0038	No	5	Joy seeking, Peer pressure, Others
7066	HTAA0039	No	3	-
7067	HTAA0041	No	4	-
7068	HTAA0043	No	4	Joy seeking, Peer pressure
7069	HTAA0044	No	1	Family issue, Relaxation
7070	HTAA0046	No	2	-
7071	HTAA0047	No	2	Joy seeking, Peer pressure
7072	HTAA0049	No	3	Relationship, Relaxation
7073	HTAA0050	No	1	Peer pressure
7074	HTAA0053	No	3	Peer pressure
7075	HTAA0054	No	2	-
7076	HTAA0058	No	2	Peer pressure
7077	HTAA0059	No	3	-
7078	HTAA0060	Yes	5	Relaxation, Peer pressure
7079	HTAA0061	No	1	Relaxation, Financial problem

No	Patient ID	Other substances	No of substances abused at a time	Reason for substance use initiation
7080	HTAA0062	No	2	Peer pressure
7081	HTAA0063	No	3	Relaxation, Peer pressure, Others
7082	HTAA0064	No	3	Job problem, Relaxation, Peer pressure
7083	HTAA0066	No	3	Joy seeking, Peer pressure
7084	HTAA0068	No	3	Relaxation, Joy seeking, Peer pressure, Others
7085	HTAA0070	No	4	-
7086	HTAA0072	No	4	Joy seeking, Peer pressure, Others
7087	HTAA0073	No	4	Peer pressure
7088	HTAA0074	No	3	Peer pressure, Others
7089	HTAA0075	No	2	-
7090	HTAA0076	No	4	Job problem, Peer pressure
7091	HTAA0077	No	1	Joy seeking, Peer pressure
7092	HTAA0078	No	4	-
7093	HTAA0079	No	4	Joy seeking, Peer pressure
7094	HTAA0081	Yes	3	-
7095	HTAA0082	No	2	Relationship
7096	HTAA0083	No	3	Job problem, Relaxation, Peer pressure, Others
7097	HTAA0084	No	1	-
7098	HTAA0085	No	3	Relaxation, Peer pressure, Others
7100	HTAA0086	No	2	-
7099	HTAA0086	No	2	Joy seeking

No	Patient ID	Other substances	No of substances abused at a time	Reason for substance use initiation
7101	HTAA0088	No	4	Family issue, Relaxation, Joy seeking, Others
7102	HTAA0090	No	1	-
7103	HTAA0091	No	5	Health, Job problem, Relaxation, Peer pressure, Financial problem
7104	HTAA0092	No	2	Peer pressure
7105	HTAA0093	No	3	-
7106	HTAA0095	No	2	-
7107	HTAA0096	No	3	Relaxation, Joy seeking, Peer pressure, Others
7108	HTAA0098	No	2	-
7109	HTAA0099	No	4	-
7110	HTAA0100	No	2	Joy seeking, Peer pressure
7111	HTAA0101	No	2	-
7112	HTAA0102	Yes	5	-
7113	HTAA0103	No	2	-
7114	HTAA0104	No	3	-
7115	HTAA0105	No	5	Relaxation, Joy seeking, Peer pressure, Others
7116	HTAA0106	No	2	Job problem, Joy seeking
7117	HTAA0107	No	5	Joy seeking, Peer pressure
7118	HTAA0108	No	3	-
7119	HTAA0109	No	5	Health, Family issue, Relaxation, Others
7120	HTAA0110	No	3	Joy seeking, Peer pressure

No	Patient ID	Other substances	No of substances abused at a time	Reason for substance use initiation
7121	HTAA0111	No	2	Relationship, Relaxation, Peer pressure
7122	HTAA0112	No	1	-
7123	HTAA0116	No	2	Joy seeking, Peer pressure
7124	HTAA0118	No	3	Relaxation, Joy seeking, Peer pressure, Others
7125	HTAA0119	No	4	Health, Others
7126	HTAA0120	No	3	Health, Relaxation, Financial problem
7127	HTAA0121	No	2	Peer pressure
7128	HTAA0122	No	5	Relaxation, Peer pressure, Others
7129	HTAA0123	No	3	-
7130	HTAA0124	No	4	Relaxation, Peer pressure, Others
7131	HTAA0126	No	3	Relaxation, Joy seeking, Peer pressure, Others
7132	HTAA0129	No	3	Joy seeking, Peer pressure, Others
7133	HTAA0131	No	1	-
7134	HTAA0134	No	2	-
7135	HTAA0138	No	2	Peer pressure, Others
7136	HTAA0142	No	2	-
7137	HTAA0148	No	3	Family issue, Peer pressure
7138	HTAA0153	No	2	Job problem, Relaxation
7139	HTAA0157	No	1	Family issue, Job problem, Financial problem
7140	HTAA0162	No	1	Joy seeking, Peer pressure

No	Patient ID	Other substances	No of substances abused at a time	Reason for substance use initiation
7141	HTAA0163	No	1	-
7142	HTAA0164	No	1	Job problem
7143	HTAA0167	No	3	Peer pressure
7144	HTAA0168	No	2	Health
7145	HTAA0170	No	3	Family issue, Peer pressure
7146	HTAA0173	No	1	Job problem
7147	HTAA0177	No	3	Joy seeking, Peer pressure
7148	HTAA0180	No	2	Peer pressure
7149	HTAA0182	No	1	Financial problem
7150	HTAA0187	No	1	Family issue, Relationship
7151	HTAA0188	No	1	-
7152	HTAA0190	No	2	Joy seeking, Peer pressure
7153	HTAA0192	No	3	Peer pressure
7154	HTAA0198	No	1	Family issue
7155	HTAA0200	No	2	Job problem
7156	HTAA0201	No	2	-
7157	HTAA0202	No	3	Financial problem
7158	HTAA0210	No	1	-
7159	HTAA0211	No	4	Family issue, Relaxation
7160	HTAA0212	No	1	Joy seeking, Peer pressure
7161	HTAA0213	No	2	Family issue, Relationship

No	Patient ID	Other substances	No of substances abused at a time	Reason for substance use initiation
7162	HTAA0217	No	1	Relaxation
7163	HTAA0219	No	1	-
7164	HTAA0222	No	2	-
7165	HTAA0223	No	2	Family issue, Peer pressure
7166	HTAA0227	No	2	-
7167	HTAA0228	No	2	-
7168	HTAA0229	No	4	Peer pressure, Financial problem
7169	HTAA0233	No	2	-
7170	HTAA0234	No	2	Job problem, Peer pressure
7171	HTAA0240	No	1	-
7172	HTAA0244	No	1	-
7173	HTAA0246	No	5	Relaxation, Joy seeking, Peer pressure
7174	HTAA0249	No	4	Job problem, Relaxation
7175	HTAA0252	No	1	-
7176	HTAA0253	No	3	Joy seeking, Peer pressure
7177	HTAA0254	No	3	-
7178	HTAA0255	No	1	-
7179	HTAA0267	No	3	Joy seeking, Peer pressure
7180	HTAA0268	No	2	Relaxation, Peer pressure, Financial problem
7181	HTAA0271	No	4	Family issue, Relationship, Relaxation
7182	HTAA0273	No	1	Relaxation, Joy seeking, Peer pressure

No	Patient ID	Other substances	No of substances abused at a time	Reason for substance use initiation
7183	HTAA0276	No	3	Relationship, Relaxation, Financial problem
7184	HTAA0281	No	2	Relationship, Relaxation, Peer pressure
7185	HTAA0283	No	1	Job problem, Financial problem
7186	HTAA0285	No	2	Relaxation, Peer pressure, Financial problem
7187	HTAA0289	No	1	-
7188	HTAA0290	Yes	6	Relaxation, Joy seeking, Peer pressure
7189	HTAA0294	No	3	Family issue, Relationship, Relaxation
7190	HTAA0298	No	2	Joy seeking, Peer pressure
7191	HTAA0299	No	4	Job problem, Joy seeking, Peer pressure
7192	HTAA0302	No	5	-
7193	HTAA0304	No	2	-
7194	HTAA0310	No	5	Relaxation, Joy seeking, Peer pressure
7195	HTAA0312	No	3	Job problem, Relationship, Relaxation, Financial problem
7196	HTAA0316	No	3	-
7197	HTAA0317	No	2	-
7198	HTAA0320	No	2	-
7199	HTAA0325	No	2	Job problem, Relaxation, Peer pressure
7200	HTAA0330	No	3	Relaxation, Peer pressure, Financial problem

No	Patient ID	Other substances	No of substances abused at a time	Reason for substance use initiation
7201	HTAA0334	No	3	Joy seeking, Peer pressure
7202	HTAA0335	No	2	Peer pressure
7203	HTAA0338	No	3	-
7204	HTAA0339	No	2	Relaxation, Joy seeking, Peer pressure
7205	HTAA0340	No	2	Family issue, Relaxation, Peer pressure, Financial problem
7206	HTAA0341	No	1	-
7207	HTAA0344	No	2	Relaxation, Peer pressure
7208	HTAA0345	No	2	-
7209	HTAA0353	No	4	Peer pressure
7210	HTAA0355	No	2	-
7211	HTAA0356	No	2	-
7212	HTAA0358	No	1	-
7213	HTAA0360	No	4	Job problem, Relationship, Relaxation
7214	HTAA0361	No	4	Relaxation, Peer pressure, Financial problem
7215	HTAA0365	No	1	-
7216	HTAA0366	No	3	Job problem, Relationship
7217	HTAA0367	No	1	-
7218	HTAA0368	No	3	Relationship, Peer pressure, Financial problem
7219	HTAA0370	No	1	Relaxation, Peer pressure, Financial problem

No	Patient ID	Other substances	No of substances abused at a time	Reason for substance use initiation
7220	HTAA0373	No	4	-
7221	HTAA0378	No	4	Family issue, Relationship, Relaxation, Financial problem
7222	HTAA0385	No	2	-
7223	HTAA0390	No	1	-
7224	HTAA0391	No	2	Relaxation, Joy seeking, Peer pressure
7225	HTAA0392	No	3	-
7226	HTAA0393	No	4	Joy seeking, Peer pressure
7227	HTAA0396	No	4	Family issue, Joy seeking, Peer pressure
7228	HTAA0397	No	1	Health
7229	HTAA0400	No	3	Job problem, Relationship, Peer pressure, Financial problem
7230	HTAA0406	No	3	Family issue, Relaxation, Joy seeking
7231	HTAA0410	No	2	Family issue, Joy seeking, Peer pressure
7232	HTAA0411	No	2	-
7233	HTAA0416	No	4	Job problem, Relaxation, Peer pressure
7234	HTAA0417	No	3	-
7235	HTAA0418	No	3	Relationship, Relaxation, Peer pressure, Financial problem
7236	HTAA0419	No	4	-
7237	HTAA0423	No	2	Job problem, Relaxation, Peer pressure

No	Patient ID	Other substances	No of substances abused at a time	Reason for substance use initiation
7238	HTAA0424	No	3	Relaxation, Joy seeking, Peer pressure
7239	HTAA0425	No	2	-
7240	HTAA0429	No	2	-
7241	HTAA0430	No	2	Family issue, Relaxation, Peer pressure
7242	HTAA0433	No	3	Health, Relaxation
7243	HTAA0434	No	4	-
7244	HTAA0436	No	3	Job problem, Relaxation, Peer pressure
7245	HTAA0438	No	2	Job problem, Relaxation
7246	HTAA0440	No	2	Family issue
7247	HTAA0441	No	1	Relationship
7248	HTAA0443	No	2	-
7249	HTAA0448	No	2	-
7250	HTAA0449	No	2	Job problem
7251	HTAA0450	No	1	-
7252	HTAA0451	No	2	Relationship
7253	HTAA0452	No	3	-
7254	HTAA0453	No	1	Family issue, Relaxation
7255	HTAA0455	No	1	Family issue
7256	HTAA0457	No	3	Family issue, Relationship, Relaxation
7257	HTAA0458	No	2	Relaxation
7258	HTAA0459	No	1	Job problem, Relationship

No	Patient ID	Other substances	No of substances abused at a time	Reason for substance use initiation
7259	HTAA0461	No	1	Relationship, Financial problem
7260	HTAA0463	No	1	-
7261	HTAA0464	No	2	-
7262	HTAA0465	No	3	Relaxation, Joy seeking, Peer pressure
7263	HTAA0466	No	1	Relaxation
7264	HTAA0467	No	2	Family issue, Job problem, Financial problem
7265	HTAA0471	No	3	Relaxation, Peer pressure, Financial problem
7266	HTAA0472	No	1	-
7267	HTAA0473	No	2	Job problem, Relaxation
7268	HTAA0476	No	1	Health
7269	HTAA0477	No	2	Job problem, Joy seeking, Peer pressure
7270	HTAA0479	No	1	Relationship, Peer pressure
7271	HTAA0480	No	2	-
7272	HTAA0482	No	2	Relaxation
7273	HTAA0483	No	1	Joy seeking, Peer pressure
7274	HTAA0484	No	2	Relaxation
7275	HTAA0485	No	1	Relaxation, Peer pressure
7276	HTAA0487	No	2	Family issue, Job problem
7277	HTAA0489	No	2	Family issue, Job problem
7278	HTAA0490	No	2	-

No	Patient ID	Other substances	No of substances abused at a time	Reason for substance use initiation
7279	HTAA0491	No	2	Family issue, Financial problem
7280	HTAA0493	No	1	Family issue, Financial problem
7281	HTAA0494	No	1	-
7282	HTAA0495	No	3	Relationship
7283	HTAA0497	No	1	Health, Relaxation
7284	HTAA0498	No	1	Relationship
7285	HTAA0500	No	1	Relaxation
7286	HTAA0501	No	1	Relaxation, Peer pressure
7287	HTAA0503	No	1	Relaxation, Peer pressure
7288	HTAA0504	No	2	Job problem
7289	HTAA0505	No	3	Joy seeking, Peer pressure
7290	HTAA0507	No	4	Job problem, Relationship, Peer pressure
7291	HTAA0510	No	2	-
7292	HTAA0512	No	1	Health
7293	HTAA0513	No	3	Health, Relationship
7294	HTAA0515	No	1	Family issue, Relaxation
7295	HTAA0516	No	1	Peer pressure
7296	HTAA0518	No	1	Health
7297	HTAA0520	No	2	Job problem
7298	HTAA0521	No	3	Job problem, Relaxation, Financial problem
7299	HTAA0524	No	1	-

No	Patient ID	Other substances	No of substances abused at a time	Reason for substance use initiation
7300	HTAA0526	No	4	Peer pressure
7301	HTAA0528	No	3	-
7302	HTAA0529	No	3	Job problem, Relaxation, Peer pressure
7303	HTAA0530	No	1	Health
7304	HTAA0531	No	5	Relaxation, Joy seeking, Peer pressure
7305	HTAA0532	No	1	Relaxation
7306	HTAA0534	No	2	Health
7307	HTAA0540	No	2	Health
7308	HTAA0542	No	1	Health
7309	HTAA0544	No	1	Health
7310	HTAA0545	No	1	Relaxation, Peer pressure, Financial problem
7311	HTAA0546	No	2	Peer pressure
7312	HTAA0547	No	2	Peer pressure, Financial problem
7313	HTAA0548	No	1	Relaxation
7314	HTAA0549	No	2	Relaxation, Joy seeking, Peer pressure
7315	HTAA0550	No	1	Relaxation
7316	HTAA0552	Yes	4	Health
7317	HTAA0553	No	2	Job problem, Relaxation, Peer pressure
7318	HTAA0555	No	1	Family issue, Relaxation, Peer pressure
7319	HTAA0556	No	2	Health

No	Patient ID	Other substances	No of substances abused at a time	Reason for substance use initiation
7320	HTAA0557	No	3	Family issue, Job problem, Relaxation
7321	HTAA0559	No	3	Job problem, Joy seeking, Peer pressure
7322	HTAA0560	No	2	-
7323	HTAA0560	No	2	Peer pressure
7324	HTAA0563	No	2	Joy seeking, Peer pressure
7325	HTAA0566	No	2	Health
7326	HTAA0567	No	2	Job problem, Relaxation, Peer pressure
7327	HTAA0568	No	1	Health
7328	HTAA0569	No	3	Job problem, Joy seeking, Peer pressure
7329	HTAA0570	No	1	Relationship
7330	HTAA0571	No	2	Family issue, Joy seeking, Peer pressure
7331	HTAA0573	No	1	Health, Job problem, Peer pressure
7332	HTAA0574	No	1	Health
7333	HTAA0575	No	3	Relaxation, Peer pressure
7334	HTAA0577	No	3	Job problem, Joy seeking, Peer pressure
7335	HTAA0578	No	1	Relaxation
7336	HTAA0581	No	2	Job problem, Relaxation, Peer pressure
7337	HTAA0583	No	2	Job problem, Relaxation
7338	HTAA0584	No	1	-
7339	HTAA0585	No	4	Relationship, Peer pressure
7340	HTAA0587	No	2	Job problem, Relaxation, Peer pressure

No	Patient ID	Other substances	No of substances abused at a time	Reason for substance use initiation
7341	HTAA0588	No	2	Relaxation
7342	HTAA0589	No	2	Joy seeking, Peer pressure
7343	HTAA0590	No	3	-
7344	HTAA0591	No	1	Job problem
7345	HTAA0592	No	1	Peer pressure
7346	HTAA0593	No	1	Relationship
7347	HTAA0594	No	2	-
7348	HTAA0595	No	2	Relationship
7349	HTAA0597	No	2	Job problem
7350	HTAA0598	No	2	-
7351	HTAA0599	No	1	Family issue
7352	HTAA0603	No	1	Family issue
7353	HTAA0604	No	2	-
7354	HTAA0606	No	3	Peer pressure
7355	HTAA0607	No	2	Job problem
7356	HTAA0609	No	1	Relationship
7357	HTAA0611	No	1	-
7358	HTAA0612	No	3	-
7359	HTAA0613	No	1	Family issue
7360	HTAA0614	No	3	-
7361	HTAA0617	No	1	Job problem, Relaxation

No	Patient ID	Other substances	No of substances abused at a time	Reason for substance use initiation
7362	HTAA0619	No	3	Joy seeking, Peer pressure
7363	HTAA0622	No	2	-
7364	HTAA0623	No	1	Relaxation, Peer pressure, Financial problem
7365	HTAA0624	No	1	Relationship
7366	HTAA0626	No	2	-
7367	HTAA0627	No	3	Job problem, Peer pressure
7368	HTAA0629	No	1	Job problem, Relaxation, Financial problem
7369	HTAA0631	No	2	Health, Relaxation
7370	HTAA0635	No	1	Job problem
7371	HTAA0637	No	1	Relationship
7372	HTAA0644	No	2	-
7373	HTAA0645	No	1	Job problem
7374	HTAA0646	No	1	Peer pressure
7375	HTAA0647	No	3	Relationship
7376	HTAA0648	No	2	Health
7377	HTAA0652	No	3	-
7378	HTAA0653	No	1	Job problem
7379	HTAA0654	No	2	-
7380	HTAA0656	No	1	-
7381	HTAA0657	No	1	Family issue
7382	HTAA0658	No	1	Health

No	Patient ID	Other substances	No of substances abused at a time	Reason for substance use initiation
7383	HTAA0659	No	2	Job problem
7384	HTAA0660	No	1	-
7385	HTAA0662	No	1	Health
7386	HTAA0663	No	1	Job problem, Relationship
7387	HTAA0664	No	1	-
7388	HTAA0665	No	1	Relaxation
7389	HTAA0668	No	1	Family issue
7390	HTAA0669	No	1	Family issue
7391	HTAA0670	No	2	-
7392	HTAA0672	No	2	Financial problem
7393	HTAA0673	No	1	Family issue
7394	HTAA0674	No	2	Joy seeking
7395	HTAA0675	No	1	Job problem, Relaxation
7396	HTAA0676	No	1	-
7397	HTAA0677	No	3	Health, Relaxation
7398	HTAA0678	No	3	Job problem
7399	HTAA0679	No	1	Peer pressure
7400	HTAA0680	No	1	-
7401	HTAA0681	No	3	Peer pressure
7402	HTAA0684	No	2	-
7403	HTAA0689	No	1	Relationship

No	Patient ID	Other substances	No of substances abused at a time	Reason for substance use initiation
7404	HTAA0690	No	1	-
7405	HTAA0693	No	1	Health
7406	HTAA0694	No	2	-
7407	HTAA0695	No	1	Family issue, Relaxation
7408	HTAA0696	No	3	Relaxation
7409	HTAA0699	No	1	Job problem
7410	HTAA0700	No	2	Relaxation
7411	HTAA0702	No	3	-
7412	HTAA0704	No	3	-
7413	HTAA0710	No	1	Health
7414	HTAR0018	No	1	Peer pressure
7415	HTAR0020	No	2	Peer pressure, Others
7416	HTAR0021	No	2	Peer pressure
7417	HTAR0022	No	2	Others
7418	HTAR0024	No	2	Relaxation, Peer pressure, Others
7419	HTAR0025	No	3	Others
7420	HTAR0026	No	1	Peer pressure, Others
7421	HTAR0027	No	1	Peer pressure, Others
7422	HTAR0028	No	2	Peer pressure, Others
7423	HTAR0029	No	1	Others
7424	HTAR0030	No	2	Peer pressure, Others

No	Patient ID	Other substances	No of substances abused at a time	Reason for substance use initiation
7425	HTAR0031	No	3	Others
7426	HTAR0032	No	2	Peer pressure, Others
7427	HTAR0033	No	3	Peer pressure
7428	HTAR0034	No	1	Relaxation, Others
7429	HTAR0035	No	2	Family issue, Peer pressure
7430	HTAR0037	No	2	Peer pressure
7431	HTAR0038	Yes	3	Peer pressure
7432	HTAR0039	No	2	Peer pressure, Others
7433	HTAR0041	No	2	Peer pressure
7434	HTAR0043	No	1	Others
7435	HTAR0047	No	1	Peer pressure
7436	HTAR0048	No	3	Health, Relaxation
7437	HTAR0049	No	2	Health
7438	HTAR0054	No	1	Peer pressure, Others
7439	HTAR0064	No	1	Peer pressure, Others
7440	HTAR0066	No	2	Family issue, Others
7441	HTAR0067	No	2	Peer pressure, Others
7442	HTAR0068	No	3	Peer pressure
7443	HTAR0070	No	1	Peer pressure
7444	HTAR0071	No	2	-
7445	HTAR0074	No	1	-

No	Patient ID	Other substances	No of substances abused at a time	Reason for substance use initiation
7446	HTAR0079	No	1	-
7447	HTAR0083	No	1	-
7448	HTAR0084	No	2	-
7449	HTAR0087	No	1	-
7450	HTAR0088	No	1	-
7451	HTAR0089	No	1	Health
7452	HTAR0090	No	1	-
7453	HTAR0093	No	1	-
7454	HTAR0095	No	1	Job problem
7455	HTAR0096	No	1	Joy seeking
7456	HTAR0098	No	4	Peer pressure
7457	HTAR0100	No	2	-
7458	HTAR0101	No	2	Peer pressure
7459	HTAR0102	No	2	Peer pressure
7460	HTAR0104	No	2	Peer pressure
7461	HTAR0105	No	2	Relaxation, Peer pressure
7462	HTAR0106	No	2	-
7463	HTAR0107	No	1	Relaxation, Peer pressure
7464	HTAR0108	Yes	3	-
7465	HTAR0110	No	2	Health
7466	HTAR0111	No	3	Peer pressure

No	Patient ID	Other substances	No of substances abused at a time	Reason for substance use initiation
7467	HTAR0113	No	2	Peer pressure
7468	HTAR0115	No	1	-
7469	HTAR0116	No	2	Peer pressure
7470	HTAR0118	No	2	Relaxation, Peer pressure
7471	HTAR0119	No	2	Peer pressure
7472	HTAR0120	No	2	-
7473	HTAR0121	No	2	-
7474	HTAR0123	No	1	-
7475	HTAR0124	No	2	-
7476	HTAR0125	No	2	-
7477	HTAR0126	No	2	Peer pressure
7478	HTAR0127	No	4	-
7479	HTAR0128	No	1	-
7480	HTAR0132	No	2	-
7481	HTAR0133	No	2	-
7482	HTAR0134	No	4	-
7483	HTAR0135	No	1	-
7484	HTAR0136	No	3	-
7485	HTAR0137	No	3	Health
7486	HTAR0139	Yes	2	Others
7487	HTAR0140	No	3	-

No	Patient ID	Other substances	No of substances abused at a time	Reason for substance use initiation
7488	HTAR0142	No	2	-
7489	HTAR0149	Yes	3	-
7490	HTAR0150	No	3	-
7491	HTAR0151	No	1	-
7492	HTAR0152	No	1	-
7493	HTAR0153	No	1	Relaxation, Joy seeking
7494	HTAR0156	Yes	1	-
7495	HTAR0157	Yes	2	Peer pressure
7496	HTAR0159	No	2	Peer pressure
7497	HTAR0161	No	2	Others
7498	HTAR0162	No	2	-
7499	HTAR0165	No	2	-
7500	HTAR0166	No	1	Peer pressure
7501	HTAR0169	No	4	Peer pressure
7502	HTAR0170	No	2	-
7503	HTAR0171	No	2	-
7504	HTAR0172	No	2	-
7505	HTAR0173	No	2	-
7506	HTAR0175	Yes	2	-
7507	HTAR0176	No	2	-
7508	HTAR0178	No	2	Others

No	Patient ID	Other substances	No of substances abused at a time	Reason for substance use initiation
7509	HTAR0179	No	1	-
7510	HTAR0181	No	2	-
7511	HTAR0182	Yes	4	Peer pressure
7512	HTAR0183	No	1	-
7513	HTAR0184	No	4	-
7514	HTAR0188	No	2	-
7515	HTAR0190	No	3	Peer pressure
7516	HTAR0191	No	2	Relaxation, Others
7517	HTAR0192	Yes	4	Peer pressure
7518	HTAR0194	No	2	Peer pressure
7519	HTAR0195	No	2	-
7520	HTAR0197	No	1	-
7521	HTAR0198	No	1	-
7522	HTAR0199	No	2	Peer pressure
7523	HTAR0201	No	3	Relationship
7524	HTAR0203	No	2	Peer pressure
7525	HTAR0205	Yes	3	Family issue, Peer pressure
7526	HTAR0207	No	1	-
7527	HTAR0208	No	1	-
7528	HTAR0209	No	3	-
7529	HTAR0210	Yes	3	Peer pressure

No	Patient ID	Other substances	No of substances abused at a time	Reason for substance use initiation
7530	HTAR0211	No	4	-
7531	HTAR0212	Yes	2	Job problem
7532	HTAR0216	No	5	-
7533	HTAR0217	No	2	-
7534	HTAR0220	No	3	-
7535	HTAR0221	Yes	5	-
7536	HTAR0222	No	2	Family issue
7537	HTAR0225	No	3	-
7538	HTAR0227	No	2	-
7539	HTAR0229	No	2	Peer pressure
7540	HTAR0231	No	2	-
7541	HTAR0232	Yes	5	Relationship, Others
7542	HTAR0234	No	2	Peer pressure
7543	HTAR0235	No	3	-
7544	HTAR0236	No	2	Peer pressure
7545	HTAR0237	No	2	-
7546	HTAR0241	No	1	-
7547	HTAR0242	No	3	Peer pressure
7548	HTAR0246	Yes	6	-
7549	HTAR0247	No	2	-
7550	HTAR0248	No	1	-

No	Patient ID	Other substances	No of substances abused at a time	Reason for substance use initiation
7551	HTAR0249	No	1	-
7552	HTAR0250	No	3	Family issue, Peer pressure
7553	HTAR0253	No	3	-
7554	HTAR0255	No	2	-
7555	HTAR0256	No	1	Joy seeking
7556	HTAR0257	No	2	-
7557	HTAR0261	No	2	Peer pressure
7558	HTAR0262	No	2	Peer pressure
7559	HTAR0263	No	1	-
7560	HTAR0264	Yes	3	-
7561	HTAR0265	No	2	-
7562	HTAR0267	No	1	-
7563	HTAR0268	No	1	Peer pressure
7564	HTAR0269	Yes	2	-
7565	HTAR0271	No	3	-
7566	HTAR0273	No	1	-
7567	HTAR0275	No	2	-
7568	HTAR0276	No	1	-
7569	HTAR0276	No	1	-
7570	HTAR0277	No	3	-
7571	HTAR0279	No	1	-

No	Patient ID	Other substances	No of substances abused at a time	Reason for substance use initiation
7572	HTAR0280	No	1	-
7573	HTAR0283	No	1	Others
7574	HTAR0285	No	2	Peer pressure, Others
7575	HTAR0287	No	1	-
7576	HTAR0289	Yes	3	Peer pressure
7577	HTAR0291	No	1	-
7578	HTAR0292	Yes	4	-
7579	HTAR0293	No	1	-
7580	HTAR0295	No	4	-
7581	HTAR0298	Yes	5	-
7582	HTAR0300	No	2	-
7583	HTAR0301	No	2	Peer pressure
7584	HTAR0302	No	3	Family issue
7585	HTAR0303	Yes	3	-
7586	HTAR0304	No	2	Peer pressure
7587	HTAR0305	No	2	-
7588	HTAR0307	No	2	-
7589	HTAR0308	No	2	Peer pressure
7590	HTAR0310	No	1	-
7591	HTAR0311	No	1	Financial problem
7592	HTAR0312	No	2	Peer pressure

No	Patient ID	Other substances	No of substances abused at a time	Reason for substance use initiation
7593	HTAR0315	No	1	-
7594	HTAR0316	No	3	-
7595	HTAR0317	No	3	-
7596	HTAR0318	No	2	-
7597	HTAR0319	No	2	-
7598	HTAR0321	No	2	Family issue
7599	HTAR0322	No	4	-
7600	HTAR0323	No	3	-
7601	HTAR0327	No	1	Peer pressure
7602	HTAR0329	No	2	-
7603	HTAR0332	No	2	-
7604	HTAR0333	No	1	Peer pressure
7605	HTAR0335	No	2	-
7606	HTAR0338	No	3	-
7607	HTAR0339	No	1	-
7608	HTAR0340	No	2	-
7609	HTAR0341	No	1	Relaxation, Peer pressure
7610	HTAR0342	No	2	-
7611	HTAR0343	No	2	-
7612	HTAR0344	No	2	Relaxation
7613	HTAR0345	No	3	-

No	Patient ID	Other substances	No of substances abused at a time	Reason for substance use initiation
7614	HTAR0346	No	1	-
7615	HTAR0347	No	3	Peer pressure
7616	HTAR0348	No	3	-
7617	HTAR0351	No	3	Peer pressure, Others
7618	HTAR0352	No	4	-
7619	HTAR0353	No	2	-
7620	HTAR0355	Yes	5	Peer pressure
7621	HTAR0357	No	1	-
7622	HTAR0358	No	1	-
7623	HTAR0361	No	2	-
7624	HTAR0362	No	2	-
7625	HTAR0363	Yes	3	Peer pressure
7626	HTAR0364	No	2	-
7627	HTAR0365	Yes	6	-
7628	HTAR0366	No	1	Peer pressure
7629	HTAR0367	No	2	Peer pressure
7630	HTAR0368	No	1	-
7631	HTAR0373	No	3	-
7632	HTAR0377	No	1	-
7633	HTAR0379	No	2	-
7634	HTAR0380	No	1	-

No	Patient ID	Other substances	No of substances abused at a time	Reason for substance use initiation
7635	HTAR0381	No	2	-
7636	HTAR0383	Yes	3	-
7637	HTAR0384	No	3	-
7638	HTAR0385	Yes	3	-
7639	HTAR0387	No	1	-
7640	HTAR0388	No	3	-
7641	HTAR0390	No	2	Peer pressure
7642	HTAR0392	No	2	-
7643	HTAR0393	No	1	Peer pressure
7644	HTAR0394	No	3	-
7645	HTAR0395	No	3	Relaxation, Joy seeking, Peer pressure
7646	HTAR0397	No	3	Peer pressure
7647	HTAR0398	Yes	2	-
7648	HTAR0399	No	1	-
7649	HTAR0400	Yes	5	-
7650	HTAR0401	No	1	-
7651	HTAR0402	No	1	-
7652	HTAR0403	No	2	-
7653	HTAR0404	No	2	Peer pressure
7654	HTAR0407	No	2	-
7655	HTAR0408	No	1	-

No	Patient ID	Other substances	No of substances abused at a time	Reason for substance use initiation
7656	HTAR0410	No	1	-
7657	HTAR0411	No	1	-
7658	HTAR0413	No	4	-
7659	HTAR0416	No	1	-
7660	HTAR0417	No	4	-
7661	HTAR0418	No	3	Peer pressure
7662	HTAR0419	Yes	3	Peer pressure
7663	HTAR0420	No	1	Peer pressure
7664	HTAR0421	No	2	-
7665	HTAR0424	No	3	-
7666	HTAR0425	No	3	-
7667	HTAR0427	No	3	-
7668	HTAR0430	No	2	-
7669	HTAR0432	No	3	-
7670	HTAR0434	No	2	-
7671	HTAR0435	No	2	-
7672	HTAR0437	No	1	-
7673	HTAR0439	Yes	2	Peer pressure
7674	HTAR0441	No	3	-
7675	HTAR0442	Yes	4	-
7676	HTAR0443	No	2	-

No	Patient ID	Other substances	No of substances abused at a time	Reason for substance use initiation
7677	HTAR0445	No	2	-
7678	HTAR0446	No	1	-
7679	HTAR0448	No	2	-
7680	HTAR0449	No	2	-
7681	HTAR0451	No	3	Peer pressure
7682	HTAR0452	No	3	-
7683	HTAR0456	No	3	-
7684	HTAR0458	No	4	-
7685	HTAR0460	No	1	-
7686	HTAR0463	No	4	-
7687	HTAR0464	No	2	-
7688	HTAR0467	No	1	-
7689	HTAR0470	No	2	-
7690	HTAR0472	No	1	-
7691	HTAR0473	No	1	-
7692	HTAR0474	No	2	-
7693	HTAR0478	No	2	-
7694	HTAR0481	No	2	Relaxation
7695	HTAR0483	No	2	-
7696	HTAR0484	No	2	-
7697	HTAR0485	No	2	-

No	Patient ID	Other substances	No of substances abused at a time	Reason for substance use initiation
7698	HTAR0486	No	2	-
7699	HTAR0487	No	1	-
7700	HTAR0490	No	2	-
7701	HTAR0491	No	1	-
7702	HTAR0494	No	2	-
7703	HTAR0495	No	2	Peer pressure
7704	HTAR0497	No	2	-
7705	HTAR0499	No	3	-
7706	HTAR0501	No	3	-
7707	HTAR0502	No	2	Peer pressure
7708	HTAR0505	No	2	Job problem, Peer pressure
7709	HTAR0506	No	2	-
7710	HTAR0507	Yes	3	-
7711	HTAR0508	No	2	Others
7712	HTAR0509	No	2	-
7713	HTAR0510	No	1	Others
7714	HTAR0511	No	2	Joy seeking, Peer pressure
7715	HTAR0512	No	1	-
7716	HTAR0513	No	1	-
7717	HTAR0514	No	2	-
7718	HTAR0515	No	1	-

No	Patient ID	Other substances	No of substances abused at a time	Reason for substance use initiation
7719	HTAR0516	No	4	-
7720	HTAR0517	No	3	-
7721	HTAR0518	No	1	-
7722	HTAR0519	Yes	3	-
7723	HTAR0520	No	2	Peer pressure
7724	HTAR0521	No	2	-
7725	HTAR0522	No	4	-
7726	HTAR0523	No	1	Family issue
7727	HTAR0524	No	3	-
7728	HTAR0525	No	2	-
7729	HTAR0526	No	2	Peer pressure
7730	HTAR0527	No	2	-
7731	HTAR0528	No	2	-
7732	HTAR0529	No	1	-
7733	HTAR0530	No	3	Peer pressure
7734	HTAR0531	No	2	-
7735	HTAR0532	No	2	-
7736	HTAR0533	No	3	-
7737	HTAR0535	Yes	3	-
7738	HTAR0536	No	2	Relationship
7739	HTAR0537	No	3	-

No	Patient ID	Other substances	No of substances abused at a time	Reason for substance use initiation
7740	HTAR0538	No	1	-
7741	HTAR0539	No	1	-
7742	HTAR0540	Yes	3	Peer pressure
7743	HTAR0541	No	2	Peer pressure
7744	HTAR0542	No	2	-
7745	HTAR0543	No	4	Peer pressure
7746	HTAR0544	No	2	-
7747	HTAR0545	No	2	-
7748	HTAR0547	No	2	-
7749	HTAR0548	No	2	-
7750	HTAR0549	No	3	Job problem
7751	HTAR0550	Yes	4	Family issue, Others
7752	HTAR0551	No	2	-
7753	HTAR0552	No	2	-
7754	HTAR0553	No	1	-
7755	HTAR0554	Yes	4	-
7756	HTAR0555	Yes	2	-
7757	HTAR0556	No	1	-
7758	HTAR0558	No	1	-
7759	HTAR0560	No	2	-
7760	HTAR0561	Yes	3	-

No	Patient ID	Other substances	No of substances abused at a time	Reason for substance use initiation
7761	HTAR0562	No	1	Health
7762	HTAR0563	Yes	3	-
7763	HTAR0564	No	2	Peer pressure
7764	HTAR0567	No	1	-
7765	HTAR0568	No	2	Peer pressure
7766	HTAR0569	Yes	4	-
7767	HTAR0570	No	4	-
7768	HTAR0571	No	1	-
7769	HTAR0572	No	2	-
7770	HTAR0573	No	2	-
7771	HTAR0574	No	1	-
7772	HTAR0575	No	2	-
7773	HTAR0576	Yes	3	-
7774	HTAR0577	No	2	-
7775	HTAR0578	No	3	-
7776	HTAR0579	No	1	-
7777	HTAR0580	No	3	-
7778	HTAR0581	No	3	-
7779	HTAR0582	No	3	-
7780	HTAR0583	No	2	-
7781	HTAR0584	No	1	-

No	Patient ID	Other substances	No of substances abused at a time	Reason for substance use initiation
7782	HTAR0585	No	1	-
7783	HTAR0586	No	1	-
7784	HTAR0587	No	1	-
7785	HTAR0588	No	3	-
7786	HTAR0589	No	3	-
7787	HTAR0590	No	2	-
7788	HTAR0591	Yes	2	-
7789	HTAR0592	Yes	2	-
7790	HTAR0593	Yes	3	-
7791	HTAR0594	No	1	-
7792	HTAR0595	No	3	-
7793	HTAR0596	No	1	Others
7794	HTAR0597	No	1	-
7795	HTAR0598	No	1	-
7796	HTAR0599	No	3	-
7797	HTAR0600	No	1	-
7798	HTAR0601	No	3	-
7799	HTAR0602	Yes	3	-
7800	HTAR0603	Yes	4	Peer pressure
7801	HTAR0604	No	1	-
7802	HTAR0605	No	2	Peer pressure

No	Patient ID	Other substances	No of substances abused at a time	Reason for substance use initiation
7803	HTAR0606	No	2	Peer pressure
7804	HTAR0607	No	1	Relaxation, Peer pressure
7805	HTAR0608	Yes	2	-
7806	HTAR0609	Yes	5	-
7807	HTAR0610	No	3	Peer pressure
7808	HTAR0611	Yes	4	-
7809	HTAR0612	No	2	-
7810	HTAR0614	Yes	3	-
7811	HTAR0615	No	1	-
7812	HTAR0616	No	3	-
7813	HTAR0617	No	2	-
7814	HTAR0618	No	3	-
7815	HTAR0619	No	2	-
7816	HTAR0620	No	2	Peer pressure
7817	HTAR0621	No	4	Peer pressure
7818	HTAR0622	No	3	-
7819	HTAR0623	No	1	-
7820	HTAR0624	No	2	-
7821	HTAR0625	No	2	Family issue, Others
7822	HTAR0626	No	1	-
7823	HTAR0627	No	1	Peer pressure

No	Patient ID	Other substances	No of substances abused at a time	Reason for substance use initiation
7824	HTAR0628	No	1	-
7825	HTAR0629	No	3	Health
7826	HTAR0630	No	2	-
7827	HTAR0631	Yes	3	-
7828	HTAR0632	No	1	-
7829	HTAR0634	No	2	Others
7830	HTAR0635	No	4	-
7831	HTAR0636	No	3	-
7832	HTAR0637	No	2	-
7833	HTAR0638	No	2	-
7834	HTAR0639	No	1	Peer pressure
7835	HTAR0640	No	1	-
7836	HTAR0641	No	1	-
7837	HTAR0642	No	1	-
7838	HTAR0643	No	1	-
7839	HTAR0644	Yes	2	-
7840	HTAR0645	Yes	3	-
7841	HTAR0646	No	1	-
7842	HTAR0647	No	2	-
7843	HTAR0648	No	2	-
7844	HTAR0649	No	1	-

No	Patient ID	Other substances	No of substances abused at a time	Reason for substance use initiation
7845	HTAR0650	No	1	-
7846	HTAR0651	No	1	-
7847	HTAR0652	No	2	Peer pressure
7848	HTAR0653	No	1	-
7849	HTAR0654	No	1	-
7850	HTAR0655	No	1	-
7851	HTAR0657	No	2	-
7852	HTAR0658	No	2	-
7853	HTAR0659	No	2	-
7854	HTAR0660	No	2	-
7855	HTAR0661	No	2	-
7856	HTAR0662	No	2	Peer pressure
7857	HTAR0663	No	3	-
7858	HTAR0664	No	3	-
7859	HTAR0665	No	1	-
7860	HTAR0666	No	3	-
7861	HTAR0667	No	1	-
7862	HTAR0668	No	1	Peer pressure, Others
7863	HTAR0669	No	2	-
7864	HTAR0671	No	3	-
7865	HTAR0672	No	2	-

No	Patient ID	Other substances	No of substances abused at a time	Reason for substance use initiation
7866	HTAR0673	Yes	2	Peer pressure
7867	HTAR0674	No	1	Peer pressure
7868	HTAR0675	No	1	-
7869	HTAR0676	No	2	Peer pressure
7870	HTAR0677	No	1	-
7871	HTAR0678	No	2	Others
7872	HTAR0679	No	2	-
7873	HTAR0680	No	1	-
7874	HTAR0681	No	2	-
7875	HTAR0682	No	1	-
7876	HTAR0683	No	1	Peer pressure
7877	HTAR0684	No	1	-
7878	HTAR0685	No	4	-
7879	HTAR0686	No	2	-
7880	HTAR0687	No	3	-
7881	HTAR0688	No	2	-
7882	HTAR0689	No	1	-
7883	HTAR0690	No	3	-
7884	HTAR0691	No	2	-
7885	HTAR0692	No	2	-
7886	HTAR0693	Yes	2	-

No	Patient ID	Other substances	No of substances abused at a time	Reason for substance use initiation
7888	HTAR0694	Yes	2	-
7887	HTAR0694	No	1	-
7889	HTAR0695	Yes	3	-
7890	HTAR0696	No	1	-
7891	HTAR0697	No	1	-
7892	HTAR0698	No	2	-
7893	HTAR0699	No	4	-
7894	HTAR0700	No	2	Peer pressure
7895	HTAR0701	No	2	-
7896	HTAR0702	No	2	Peer pressure
7897	HTAR0703	No	1	Relaxation
7898	HTAR0704	No	2	-
7899	HTAR0705	No	2	-
7900	HTAR0706	No	2	-
7901	HTAR0707	No	2	-
7902	HTAR0708	No	3	-
7903	HTAR0709	No	3	-
7904	HTAR0710	No	3	-
7905	HTAR0711	No	1	-
7906	HTAR0712	No	1	-
7907	HTAR0713	No	2	-

No	Patient ID	Other substances	No of substances abused at a time	Reason for substance use initiation
7908	HTAR0714	No	2	-
7909	HTAR0715	Yes	2	-
7910	HTAR0716	No	2	-
7911	HTAR0717	Yes	3	-
7912	HTAR0718	No	5	-
7913	HTAR0719	No	1	-
7914	HTAR0720	No	3	-
7915	HTAR0721	No	3	Peer pressure
7916	HTAR0722	No	2	Relaxation
7917	HTAR0723	No	1	-
7918	HTAR0724	No	2	Relaxation, Peer pressure
7919	HTAR0725	No	2	Relaxation, Peer pressure
7920	HTAR0726	No	2	Relaxation
7921	HTAR0727	No	1	-
7922	HTAR0728	Yes	3	Joy seeking, Peer pressure
7923	HTAR0729	No	1	Relaxation
7924	HTAR0731	No	3	Peer pressure
7925	HTAR0732	No	4	Family issue
7926	HTAR0733	No	2	Peer pressure
7927	HTAR0734	No	2	-
7928	HTAR0735	No	1	-

No	Patient ID	Other substances	No of substances abused at a time	Reason for substance use initiation
7929	HTAR0736	No	2	Peer pressure, Others
7930	HTAR0737	No	3	Peer pressure
7931	HTAR0738	Yes	2	Peer pressure
7932	HTAR0740	No	3	-
7933	HTAR0741	No	3	Peer pressure
7934	HTAR0743	No	2	Relaxation, Peer pressure
7935	HTAR0744	No	3	Peer pressure
7936	HTAR0745	No	1	Relaxation, Peer pressure
7937	HTAR0746	No	3	Peer pressure
7938	HTAR0747	No	2	Peer pressure
7939	HTAR0748	No	4	Peer pressure
7940	HTAR0749	No	1	-
7941	HTAR0750	Yes	2	-
7942	HTAR0751	Yes	4	-
7943	HTAR0752	No	2	-
7944	HTAR0753	No	2	Peer pressure
7945	HTAR0754	No	1	Peer pressure
7946	HTAR0755	No	2	-
7947	HTAR0756	No	2	Peer pressure
7948	HTAR0757	No	3	Relationship
7949	HTAR0758	No	2	Peer pressure

No	Patient ID	Other substances	No of substances abused at a time	Reason for substance use initiation
7950	HTAR0759	No	1	-
7951	HTAR0760	No	1	Peer pressure
7952	HTAR0761	No	4	-
7953	HTAR0762	No	3	-
7954	HTAR0763	No	2	Relaxation
7955	HTAR0764	No	2	-
7956	HTAR0765	No	3	Relationship
7957	HTAR0766	No	2	Relaxation
7958	HTAR0767	No	3	-
7959	HTAR0768	No	3	Peer pressure
7960	HTAR0769	No	2	-
7961	HTAR0770	No	2	Peer pressure
7962	HTAR0771	No	2	-
7963	HTAR0772	No	4	-
7964	HTAR0773	Yes	2	Joy seeking
7965	HTAR0774	No	2	-
7966	HTAR0775	No	3	-
7967	HTAR0776	Yes	3	-
7968	HTAR0777	No	2	Peer pressure
7969	HTAR0779	No	2	Health
7970	HTAR0780	No	2	-

No	Patient ID	Other substances	No of substances abused at a time	Reason for substance use initiation
7971	HTAR0781	No	1	Peer pressure
7972	HTAR0782	No	2	Relaxation
7973	HTAR0783	No	2	Peer pressure
7974	HTAR0784	No	1	Peer pressure
7975	HTAR0785	No	3	Peer pressure
7976	HTAR0786	No	1	Peer pressure, Financial problem
7977	HTAR0787	No	4	Joy seeking, Peer pressure
7978	HTAR0788	No	2	Peer pressure
7979	HTAR0789	No	2	-
7980	HTAR0790	No	2	Relationship
7981	HTAR0791	No	2	Peer pressure
7982	HTAR0792	No	5	Peer pressure
7983	HTAR0793	No	1	Peer pressure
7984	HTAR0794	Yes	3	Peer pressure
7985	HTAR0795	No	3	Peer pressure
7986	HTAR0796	No	3	Peer pressure
7987	HTAR0797	No	3	Peer pressure
7988	HTAR0798	Yes	2	Peer pressure
7989	HTAR0799	No	2	Relationship, Relaxation, Peer pressure
7990	HTAR0850	No	4	Relaxation, Peer pressure
7991	HTAR0851	No	1	Peer pressure

No	Patient ID	Other substances	No of substances abused at a time	Reason for substance use initiation
7992	HTAR0852	No	2	-
7993	HTAR0854	No	1	Peer pressure
7994	HTAR0855	No	3	Joy seeking, Peer pressure
7995	HTAR0856	No	1	Relaxation, Joy seeking, Peer pressure
7996	HTAR0857	Yes	3	Relaxation, Peer pressure
7997	HTAR0858	Yes	3	-
7998	HTAR0859	No	1	Relationship, Relaxation
7999	HTAR0860	No	2	-
8000	HTAR0861	No	4	Peer pressure
8001	HTAR0862	No	4	-
8002	HTAR0863	No	3	Relaxation, Peer pressure
8003	HTAR0864	Yes	4	Peer pressure
8004	HTAR0866	Yes	2	Relaxation, Peer pressure
8005	HTAR0867	No	1	Relaxation, Joy seeking
8006	HTAR0868	Yes	2	Family issue, Relationship
8007	HTAR0869	No	1	-
8008	HTAR0870	No	4	Relationship
8009	HTAR0871	No	2	Relaxation, Peer pressure
8010	HTAR0872	No	1	Relaxation, Joy seeking
8011	HTAR0873	No	1	Peer pressure
8012	HTAR0874	No	2	Peer pressure

No	Patient ID	Other substances	No of substances abused at a time	Reason for substance use initiation
8013	HTAR0875	No	1	-
8014	HTAR0876	No	3	-
8015	HTAR0878	No	2	-
8016	HTAR0879	No	1	-
8017	HTAR0880	No	1	-
8018	HTAR0881	No	4	-
8019	HTAR0882	No	1	-
8020	HTAR0883	No	2	-
8021	HTAR0884	Yes	2	-
8022	HTAR0885	No	1	-
8023	HTAR0886	No	2	Relationship
8024	HTAR0887	No	3	-
8025	HTAR0890	No	2	-
8026	HTAR0892	No	2	-
8027	HTAR0893	No	1	-
8028	HTAR0894	No	2	-
8029	HTAR0895	No	1	-
8030	HTAR0896	Yes	2	-
8031	HTAR0897	No	2	-
8032	HTAR0899	No	1	-
8033	HTAR0900	No	1	-

No	Patient ID	Other substances	No of substances abused at a time	Reason for substance use initiation
8034	HTAR0901	No	2	Family issue
8035	HTAR0902	Yes	2	Family issue
8036	HTAR0903	Yes	3	Joy seeking
8037	HTAR0904	No	2	Relaxation
8038	HTAR0905	Yes	2	-
8039	HTAR0907	Yes	3	-
8040	HTAR0908	No	2	Peer pressure
8041	HTAR0909	No	4	Peer pressure
8042	HTAR0911	No	2	-
8043	HTAR0912	Yes	2	-
8044	HTAR0913	No	3	-
8045	HTAR0914	No	2	-
8046	HTAR0915	No	4	Peer pressure
8047	HTAR0916	No	3	-
8048	HTAR0917	No	2	-
8049	HTAR0920	No	2	Family issue, Relationship
8050	HTAR0922	No	2	Peer pressure
8051	HTAR0924	No	1	-
8052	HTAR0928	Yes	2	-
8053	HTAR0929	No	2	-
8054	HTAR0930	Yes	5	-

No	Patient ID	Other substances	No of substances abused at a time	Reason for substance use initiation
8055	HTAR0931	No	1	-
8056	HTAR0932	Yes	4	-
8057	HTAR0933	No	1	Health, Family issue
8058	HTAR0934	No	2	-
8059	HTAR0935	No	2	-
8060	HTAR0936	No	2	-
8061	HTAR0937	No	1	-
8062	HTAR0938	No	2	Peer pressure
8063	HTAR0939	Yes	2	-
8064	HTAR0940	No	3	-
8065	HTAR0941	No	2	-
8066	HTAR0943	No	2	Peer pressure
8067	HTAR0944	Yes	2	-
8068	HTAR0945	No	1	Peer pressure
8069	HTAR0946	No	1	-
8070	HTAR0947	No	3	-
8071	HTAR0948	No	1	-
8072	HTAR0949	No	3	-
8073	HTAR0949	No	4	-
8074	HTAR0950	No	1	Relationship, Relaxation
8075	HTAR0951	No	1	-

No	Patient ID	Other substances	No of substances abused at a time	Reason for substance use initiation
8076	HTAR0952	No	1	-
8077	HTAR0953	No	1	-
8078	HTAR0954	No	1	Relaxation
8079	HTAR0955	Yes	3	-
8080	HTAR0956	No	2	-
8081	HTAR0957	No	1	-
8082	HTAR0958	Yes	2	Family issue
8083	HTAR0959	No	3	-
8084	HTAR0960	No	1	Peer pressure
8085	HTAR0961	No	2	Peer pressure
8086	HTAR0962	No	1	-
8087	HTAR0964	No	1	-
8088	HTAR0965	No	2	-
8089	HTAR0967	No	2	Relaxation
8090	HTAR0969	Yes	2	-
8091	HTAR0970	Yes	2	-
8092	HTAR0971	No	3	-
8093	HTAR0972	No	1	Relaxation, Peer pressure
8094	HTAR0973	No	2	Family issue
8095	HTAR0974	No	1	-
8096	HTAR0975	No	2	-

No	Patient ID	Other substances	No of substances abused at a time	Reason for substance use initiation
8097	HTAR0976	No	1	Joy seeking, Peer pressure
8098	HTAR0977	No	1	Relaxation, Joy seeking
8099	HTAR0978	Yes	3	-
8100	HTAR0980	No	2	-
8101	HTAR0981	No	1	-
8102	HTAR0982	No	1	-
8103	HTAR0983	No	2	-
8104	HTAR0984	No	1	-
8105	HTAR0985	Yes	2	-
8106	HTAR0986	No	1	-
8107	HTAR0986	No	3	-
8108	HTAR0988	No	1	-
8109	HTAR0989	No	1	Relationship, Peer pressure
8110	HTAR0990	No	1	Peer pressure
8111	HTAR0991	No	1	Peer pressure
8112	HTAR0992	Yes	3	-
8113	HTAR0993	No	1	-
8114	HTAR0996	No	2	Relaxation, Peer pressure
8115	HTAR0997	Yes	3	-
8116	HTAR0998	No	2	-
8117	HTAR0998	No	1	-

No	Patient ID	Other substances	No of substances abused at a time	Reason for substance use initiation
8118	HTAR0998	Yes	1	-
8119	HTAR0999	No	1	-
8120	HTAR1000	No	1	Peer pressure
8121	HTAR1001	No	1	-
8122	HTAR1002	No	1	-
8123	HTAR1003	Yes	3	Family issue
8124	HTAR1004	No	2	-
8125	HTAR1005	No	1	Relaxation, Joy seeking
8126	HTAR1006	Yes	1	Peer pressure
8127	HTAR1007	Yes	3	Peer pressure
8128	HTF0003	No	1	Peer pressure
8129	HTF0009	No	3	Family issue, Peer pressure
8130	HTF0024	No	1	Family issue
8131	HTF0041	No	1	Peer pressure
8132	HTF0057	No	3	-
8133	HTF0067	No	3	Relaxation
8134	HTF0094	No	2	-
8135	HTF0118	No	2	Peer pressure
8136	HTF0124	No	2	Family issue
8137	HTF0127	No	1	-
8138	HTF0132	No	3	-
8139	HTF0133	No	4	-
8140	HTF0134	No	2	-
8141	HTF0137	No	2	Family issue, Job problem, Peer pressure
8142	HTF0138	No	1	Family issue, Relationship, Peer pressure
8143	HTF0139	No	4	Peer pressure
8144	HTF0140	No	2	Family issue
8145	HTF0142	No	1	-
8146	HTF0145	No	2	-

No	Patient ID	Other substances	No of substances abused at a time	Reason for substance use initiation
8147	HTF0146	No	2	-
8148	HTF0147	No	2	-
8149	HTF0149	No	3	-
8150	HTF0156	No	2	-
8151	HTF0157	No	4	Peer pressure
8152	HTF0158	No	2	-
8153	HTF0160	No	2	-
8154	HTF0162	No	1	Family issue, Relaxation, Peer pressure
8155	HTF0168	No	1	Job problem, Relaxation
8156	HTF0169	No	2	-
8157	HTF0184	No	2	-
8158	HTF0188	No	3	-
8159	HTF0200	No	1	-
8160	HTF0201	No	2	Joy seeking, Peer pressure
8161	HTF0208	No	3	-
8162	HTF0210	No	3	Relaxation, Joy seeking, Peer pressure
8163	HTF0215	No	2	-
8164	HTF0217	No	2	-
8165	HTF0222	No	1	-
8166	HTF0225	No	2	Relaxation, Joy seeking
8167	HTF0227	No	2	Peer pressure
8168	HTF0228	No	1	Relaxation, Peer pressure
8169	HTF0229	No	1	-
8170	HTF0231	No	3	-
8171	HTF0233	No	1	Joy seeking
8172	HTF0234	No	3	Peer pressure
8173	HTF0235	No	2	Financial problem
8174	HTF0238	No	2	-
8175	HTF0241	No	1	-
8176	HTF0248	No	2	Joy seeking, Peer pressure
8177	HTF0251	No	4	-
8178	HTF0265	No	2	-
8179	HTF0266	No	2	-
8180	HTF0284	No	3	-
8181	HTF0285	No	1	-
8182	HTF0291	No	3	Peer pressure
8183	HTF0295	No	2	-
8184	HTF0296	No	4	Peer pressure

No	Patient ID	Other substances	No of substances abused at a time	Reason for substance use initiation
8185	HTF0299	No	2	Relaxation
8186	HTF0303	No	1	Peer pressure
8187	HTF0304	No	2	-
8188	HTF0308	No	2	-
8189	HTF0314	No	1	-
8190	HTF0323	No	1	-
8191	HTF0329	No	2	Family issue
8192	HTF0333	No	1	-
8193	HTF0344	No	1	-
8194	HTF0346	No	1	-
8195	HTF0348	No	1	-
8196	HTF0349	No	1	-
8197	HTF0350	No	1	-
8198	HTF0351	No	1	-
8199	HTF0352	No	1	-
8200	HTF0353	No	1	-
8201	HTF0354	No	1	-
8202	HTF0356	No	1	-
8203	HTF0357	No	1	-
8204	HTF0358	No	1	-
8205	HTF0359	No	1	-
8206	HTF0362	No	1	-
8207	HTF0363	No	1	-
8208	HTF0364	No	1	-
8209	HTF0365	No	2	-

No	Patient ID	Other substances	No of substances abused at a time	Reason for substance use initiation
8210	HTF0366	No	1	-
8211	HTF0367	No	1	-
8212	HTF0370	No	1	-
8213	HTF0371	No	1	-
8214	HTF0372	No	1	-
8215	HTF0373	No	1	-
8216	HTF0374	No	1	-
8217	HTF0375	No	1	-
8218	HTF0376	No	1	-
8219	HTF0378	No	1	-
8220	HTF0379	No	1	-
8221	HTF0380	No	1	-
8222	HTF0381	No	1	-
8223	HTF0382	No	2	-
8224	HTF0383	No	1	-
8225	HTF0385	No	1	-
8226	HTF0386	No	1	-
8227	HTF0387	No	1	-
8228	HTF0388	No	2	-
8229	HTF0389	No	2	-
8230	HTF0390	No	1	-

No	Patient ID	Other substances	No of substances abused at a time	Reason for substance use initiation
8231	HTF0391	No	1	-
8232	HTF0392	No	1	-
8233	HTF0393	No	1	-
8234	HTF0394	No	1	-
8235	HTF0395	No	1	-
8236	HTF0396	No	1	-
8237	HTF0398	No	2	-
8238	HTF0399	No	1	Peer pressure
8239	HTF0400	No	1	-
8240	HTF0401	No	1	-
8241	HTF0402	No	1	-
8242	HTF0403	No	1	-
8243	HTF0404	No	1	-
8244	HTF0405	No	1	-
8245	HTF0406	No	1	-
8246	HTF0407	No	1	-
8247	HTF0408	No	1	Peer pressure
8248	HTF0409	No	1	Peer pressure
8249	HTF0410	No	1	Job problem
8250	HTF0412	No	1	-
8251	HTF0413	No	3	-

No	Patient ID	Other substances	No of substances abused at a time	Reason for substance use initiation
8252	HTF0414	No	1	Family issue
8253	HTF0416	No	1	Peer pressure
8254	HTF0417	No	1	-
8255	HTF0418	No	2	-
8256	HTF0419	No	1	-
8257	HTF0420	No	1	-
8258	HTF0423	No	2	-
8259	HTF0424	No	1	-
8260	HTF0425	No	1	-
8261	HTF0427	No	1	Health, Job problem
8262	HTF0428	No	4	Health, Job problem
8263	HTF0429	No	3	-
8264	HTF0430	No	2	-
8265	HTF0431	No	1	-
8266	HTF0432	No	5	Peer pressure
8267	HTF0436	No	2	-
8268	HTF0437	No	2	-
8269	HTF0438	No	2	Peer pressure
8270	HTF0439	No	1	Joy seeking
8271	HTF0440	No	1	-
8272	HTF0441	No	1	-
8273	HTF0442	No	1	-
8274	HTF0447	No	1	-
8275	HTF0456	No	1	Joy seeking
8276	HTF0457	No	1	Relationship
8277	HTF0458	No	1	-
8278	HTF0459	No	1	Peer pressure
8279	HTF0460	No	2	-
8280	HTF0461	No	2	-
8281	HTF0462	No	1	Family issue
8282	HTF0463	No	1	-
8283	HTF0465	No	1	Peer pressure

No	Patient ID	Other substances	No of substances abused at a time	Reason for substance use initiation
8284	HTF0466	No	1	Family issue
8285	HTF0467	No	3	-
8286	HTF0468	No	1	-
8287	HTF0469	No	3	-
8288	HTF0470	No	1	Peer pressure
8289	HTF0471	No	3	-
8290	HTF0472	No	1	-
8291	HTF0473	No	1	-
8292	HTF0474	No	1	Relationship
8293	HTF0475	No	1	-
8294	HTF0476	No	1	-
8295	HTF0477	No	1	-
8296	HTF0478	No	1	-
8297	HTF0479	No	1	-
8298	HTF0480	No	1	-
8299	HTF0481	No	1	-
8300	HTF0482	No	2	-
8301	HTF0483	No	1	-
8302	HTF0484	No	1	-
8303	HTF0485	No	1	-
8304	HTF0486	No	1	Peer pressure
8305	HTF0487	No	1	-

No	Patient ID	Other substances	No of substances abused at a time	Reason for substance use initiation
8306	HTF0488	No	1	-
8307	HTF0490	No	1	-
8308	HTF0491	No	1	-
8309	HTF0492	No	1	Peer pressure
8310	HTF0493	No	1	-
8311	HTF0494	No	2	-
8312	HTF0495	No	1	-
8313	HTF0496	No	1	-
8314	HTF0498	No	1	Job problem
8315	HTF0499	No	1	-
8316	HTF0500	No	1	-
8317	HTF0501	No	1	-
8318	HTF0502	No	1	-
8319	HTF0503	No	3	-
8320	HTF0504	No	2	-
8321	HTF0505	No	1	-
8322	HTF0506	No	1	-
8323	HTF0507	No	1	-
8324	HTF0508	No	1	-
8325	HTF0509	No	2	Peer pressure
8326	HTF0510	No	1	-
8327	HTF0511	No	1	-

No	Patient ID	Other substances	No of substances abused at a time	Reason for substance use initiation
8328	HTF0512	No	1	-
8329	HTF0513	No	2	-
8330	HTF0514	No	2	-
8331	HTF0515	No	1	-
8332	HTF0516	No	1	Peer pressure
8333	HTF0517	No	1	-
8334	HTF0518	No	2	Peer pressure
8335	HTF0519	No	1	-
8336	HTF0520	No	1	-
8337	HTF0521	No	1	-
8338	HTF0522	No	2	-
8339	HTF0523	No	1	-
8340	HTF0524	No	1	Peer pressure
8341	HTF0525	No	1	-
8342	HTF0526	No	1	Peer pressure
8343	HTF0527	No	1	-
8344	HTF0528	No	1	-
8345	HTF0530	No	1	-
8346	HTF0531	No	1	Peer pressure
8347	HTF0532	No	2	Peer pressure
8348	HTF0533	No	2	-
8349	HTF0534	No	1	-
8350	HTF0535	No	1	-

No	Patient ID	Other substances	No of substances abused at a time	Reason for substance use initiation
8351	HTF0536	No	1	-
8352	HTF0537	No	1	Peer pressure
8353	HTF0538	No	1	-
8354	HTF0540	No	2	-
8355	HTF0541	No	1	-
8356	HTF0542	No	1	-
8357	HTF0542	No	3	Peer pressure
8358	HTF0545	No	1	-
8359	HTF0546	No	2	-
8360	HTF0547	No	1	-
8361	HTF0548	No	1	Peer pressure
8362	HTF0549	No	1	-
8363	HTF0550	No	1	Peer pressure
8364	HTF0551	No	1	-
8365	HTF0552	No	2	-
8366	HTF0553	No	1	Peer pressure
8367	HTJ0001	Yes	4	Peer pressure, Others
8368	HTJ0002	Yes	10	Family issue
8369	HTJ0006	No	3	Job problem, Relationship, Relaxation
8370	HTJ0007	No	2	Job problem, Financial problem
8371	HTJ0009	No	3	Job problem, Financial problem
8372	HTJ0010	No	2	Peer pressure, Others
8373	HTJ0011	No	3	Peer pressure
8374	HTJ0012	No	2	-

No	Patient ID	Other substances	No of substances abused at a time	Reason for substance use initiation
8375	HTJ0014	Yes	10	Relaxation, Peer pressure, Others
8376	HTJ0015	No	2	Peer pressure
8377	HTJ0017	Yes	10	Relaxation, Peer pressure
8378	HTJ0018	Yes	10	Relationship, Relaxation, Joy seeking, Peer pressure
8379	HTJ0020	Yes	10	-
8380	HTJ0021	Yes	10	Health, Relaxation, Peer pressure
8381	HTJ0022	Yes	10	Joy seeking, Peer pressure
8382	HTJ0023	No	2	-
8383	HTJ0024	Yes	10	-
8384	HTJ0025	No	2	-
8385	HTJ0027	Yes	10	-
8386	HTJ0028	No	2	-
8387	HTJ0030	Yes	9	-
8388	HTJ0031	No	3	Peer pressure
8389	HTJ0032	Yes	9	Family issue, Relaxation, Joy seeking
8390	HTJ0035	Yes	10	Relaxation, Peer pressure
8391	HTJ0036	Yes	10	-
8392	HTJ0038	No	1	Health, Family issue
8393	HTJ0039	Yes	9	-
8394	HTJ0040	Yes	10	Peer pressure, Others
8395	HTJ0042	No	3	-
8396	HTJ0045	No	2	Health
8397	HTJ0046	Yes	4	Family issue
8398	HTJ0047	No	1	Peer pressure
8399	HTJ0048	Yes	4	Peer pressure
8400	HTJ0049	No	2	Health
8401	HTJ0057	No	2	Peer pressure
8402	HTJ0058	No	1	Relaxation

No	Patient ID	Other substances	No of substances abused at a time	Reason for substance use initiation
8403	HTJ0059	No	5	Health, Family issue, Financial problem
8404	HTJ0060	Yes	4	Relaxation, Joy seeking, Peer pressure
8405	HTJ0061	No	2	Relaxation, Peer pressure
8406	HTJ0062	No	2	Peer pressure
8407	HTJ0066	No	3	Family issue, Peer pressure
8408	HTJ0067	No	2	Peer pressure
8409	HTJ0068	No	2	Family issue, Peer pressure
8410	HTJ0069	No	1	Health, Job problem
8411	HTJ0070	No	2	Job problem, Peer pressure
8412	HTJ0073	No	1	Others
8413	HTJ0076	No	3	Family issue, Peer pressure
8414	HTJ0077	No	2	Relaxation, Joy seeking, Peer pressure
8415	HTJ0081	Yes	4	Joy seeking, Peer pressure
8416	HTJ0084	No	2	-
8417	HTJ0090	No	1	Job problem, Peer pressure
8418	HTJ0091	No	1	-
8419	HTJ0096	Yes	1	Relaxation
8420	HTJ0097	No	1	Peer pressure
8421	HTJ0098	No	1	Relaxation, Peer pressure
8422	HTJ0099	No	1	-
8423	HTJ0100	No	1	Family issue, Peer pressure
8424	HTJ0101	No	3	Peer pressure
8425	HTJ0102	No	1	Relaxation, Peer pressure
8426	HTJ0103	No	2	Peer pressure, Others
8427	HTJ0104	No	1	-
8428	HTJ0105	No	1	Relaxation, Peer pressure, Others

No	Patient ID	Other substances	No of substances abused at a time	Reason for substance use initiation
8429	HTJ0106	No	1	Joy seeking, Peer pressure
8430	HTJ0109	No	1	Relaxation, Joy seeking
8431	HTJ0110	No	2	Peer pressure
8432	HTJ0111	No	4	Peer pressure
8433	HTJ0113	No	3	Others
8434	HTJ0114	No	4	Joy seeking, Peer pressure
8435	HTJ0115	No	3	Peer pressure
8436	HTJ0116	No	3	Peer pressure, Others
8437	HTJ0117	No	4	-
8438	HTJ0119	No	1	Relaxation, Peer pressure
8439	HTJ0120	No	1	-
8440	HTJ0121	No	1	-
8441	HTJ0122	No	2	-
8442	HTJ0123	No	4	Relationship, Relaxation, Peer pressure
8443	HTJ0125	No	1	Job problem, Relaxation, Joy seeking, Peer pressure
8444	HTJ0126	No	2	Others
8445	HTJ0128	No	5	Peer pressure
8446	HTJ0129	No	4	Family issue, Others
8447	HTJ0130	No	1	Relaxation, Joy seeking, Peer pressure
8448	HTJ0132	No	4	Joy seeking, Peer pressure
8449	HTJ0134	No	5	Relaxation, Peer pressure
8450	HTJ0135	No	2	Relaxation, Peer pressure
8451	HTJ0136	No	3	Peer pressure
8452	HTJ0137	No	3	Relaxation, Peer pressure
8453	HTJ0138	No	3	Peer pressure
8454	HTJ0139	No	1	Others
8455	HTJ0140	No	3	Peer pressure

No	Patient ID	Other substances	No of substances abused at a time	Reason for substance use initiation
8456	HTJ0142	No	1	Family issue, Others
8457	HTJ0143	No	1	-
8458	HTJ0147	No	3	Peer pressure
8459	HTJ0148	No	2	Family issue, Peer pressure
8460	HTJ0149	No	2	-
8461	HTJ0150	Yes	3	Peer pressure
8462	HTJ0152	No	1	Peer pressure
8463	HTJ0153	No	4	Health
8464	HTJ0159	No	1	-
8465	HTJ0160	No	1	Relationship
8466	HTJ0162	No	3	Relationship
8467	HTJ0167	No	1	Health, Joy seeking
8468	HTJ0168	No	2	Job problem, Relaxation
8469	HTJ0169	No	1	Joy seeking
8470	HTJ0170	No	2	Joy seeking, Peer pressure
8471	HTJ0171	No	4	Relaxation, Peer pressure
8472	HTJ0172	No	1	Peer pressure
8473	HTJ0173	Yes	4	Financial problem
8474	HTJ0174	No	2	Relationship, Peer pressure
8475	HTJ0175	No	1	Health
8476	HTJ0176	No	2	Relaxation, Peer pressure
8477	HTJ0179	No	2	Peer pressure
8478	HTJ0180	No	2	Joy seeking
8479	HTJ0181	No	1	-
8480	HTJ0182	No	1	Peer pressure, Others
8481	HTJ0183	No	1	Family issue, Financial problem
8482	HTJ0184	No	1	Job problem, Joy seeking
8483	HTJ0186	No	4	-

No	Patient ID	Other substances	No of substances abused at a time	Reason for substance use initiation
8484	HTJ0188	No	3	Joy seeking, Peer pressure
8485	HTJ0189	No	3	Peer pressure
8486	HTJ0191	Yes	5	Family issue, Peer pressure
8487	HTJ0193	No	2	Peer pressure
8488	HTJ0196	No	3	Job problem, Relaxation
8489	HTJ0197	No	3	Peer pressure
8490	HTJ0198	No	1	Relaxation, Joy seeking
8491	HTJ0199	No	1	Relaxation
8492	HTJ0200	Yes	8	Peer pressure
8493	HTJ0202	No	6	Peer pressure
8494	HTJ0203	No	6	Family issue, Relaxation, Peer pressure
8495	HTJ0204	No	4	Peer pressure
8496	HTJ0205	No	2	Joy seeking, Peer pressure
8497	HTJ0206	No	5	Peer pressure
8498	HTJ0207	No	3	Peer pressure
8499	HTJ0209	No	5	Family issue
8500	HTJ0211	No	3	-
8501	HTJ0212	No	2	Joy seeking
8502	HTJ0213	No	2	Relaxation
8503	HTJ0214	No	5	Others
8504	HTJ0216	No	1	Job problem
8505	HTJ0217	No	1	-
8506	HTJ0219	No	2	Peer pressure
8507	HTJ0220	No	1	-
8508	HTJ0221	No	3	Relaxation
8509	HTJ0222	No	1	-
8510	HTJ0223	No	1	Peer pressure
8511	HTJ0224	No	2	Peer pressure
8512	HTJ0225	No	2	Relaxation, Joy seeking
8513	HTJ0226	No	1	Peer pressure
8514	HTJ0229	No	2	Peer pressure

No	Patient ID	Other substances	No of substances abused at a time	Reason for substance use initiation
8515	HTJ0233	No	1	-
8516	HTJ0234	No	1	-
8517	HTJ0236	No	2	-
8518	HTJ0237	No	3	Peer pressure, Others
8519	HTJ0238	No	1	Job problem, Relationship
8520	HTJ0239	No	2	Health, Relaxation
8521	HTJ0240	No	2	Peer pressure
8522	HTJ0242	No	2	Peer pressure
8523	HTJ0244	No	4	Peer pressure
8524	HTJ0247	No	2	Peer pressure
8525	HTJ0248	No	2	Relaxation, Peer pressure
8526	HTJ0249	No	6	Peer pressure
8527	HTJ0251	No	1	Peer pressure
8528	HTJ0252	No	2	Relationship
8529	HTJ0255	No	1	Peer pressure
8530	HTJ0256	No	1	Joy seeking
8531	HTJ0258	No	1	Relationship
8532	HTJ0259	No	2	-
8533	HTJ0260	No	5	Peer pressure
8534	HTJ0261	No	2	Peer pressure
8535	HTJ0262	No	3	Job problem
8536	HTJ0263	No	1	Peer pressure
8537	HTJ0265	No	1	Joy seeking, Peer pressure
8538	HTJ0266	No	2	Peer pressure
8539	HTJ0267	No	4	Peer pressure
8540	HTJ0268	No	2	-
8541	HTJ0269	No	4	Joy seeking, Peer pressure
8542	HTJ0270	No	3	Family issue, Relationship, Peer pressure
8543	HTJ0272	No	2	Family issue, Peer pressure
8544	HTJ0277	No	1	-
8545	HTJ0278	No	1	-
8546	HTJ0280	No	1	-

No	Patient ID	Other substances	No of substances abused at a time	Reason for substance use initiation
8547	HTJ0282	No	2	Health
8548	HTJ0283	No	4	Peer pressure
8549	HTJ0284	No	2	Family issue, Peer pressure
8550	HTJ0286	Yes	5	Others
8551	HTJ0289	No	5	Peer pressure, Others
8552	HTJ0291	No	2	Relaxation, Peer pressure
8553	HTJ0292	No	2	Joy seeking, Peer pressure
8554	HTJ0293	No	4	Peer pressure
8555	HTJ0294	No	2	Family issue
8556	HTJ0295	No	1	Peer pressure
8557	HTJ0296	Yes	7	Health, Peer pressure
8558	HTJ0300	No	1	-
8559	HTJ0301	No	2	Family issue, Peer pressure
8560	HTJ0303	No	4	Peer pressure
8561	HTJ0304	No	1	Family issue, Job problem, Peer pressure
8562	HTJ0306	No	1	-
8563	HTJ0307	No	2	Peer pressure
8564	HTJ0308	No	1	Relaxation
8565	HTJ0309	No	2	Peer pressure
8566	HTJ0311	No	2	Peer pressure, Others
8567	HTJ0314	No	2	Others
8568	HTJ0315	No	1	Others
8569	HTJ0316	No	2	Job problem, Relationship
8570	HTJ0317	No	2	-
8571	HTJ0318	No	2	Job problem
8572	HTJ0319	No	2	Others
8573	HTJ0325	No	3	Job problem
8574	HTJ0326	No	3	Peer pressure
8575	HTJ0327	No	2	Peer pressure
8576	HTJ0328	No	3	Job problem, Peer pressure

No	Patient ID	Other substances	No of substances abused at a time	Reason for substance use initiation
8577	HTJ0329	No	3	Health, Family issue
8578	HTJ0330	No	2	Relationship
8579	HTJ0331	No	2	Health
8580	HTJ0332	No	2	Relaxation, Joy seeking
8581	HTJ0334	No	2	Peer pressure
8582	HTJ0335	No	2	Peer pressure
8583	HTJ0339	No	3	Peer pressure
8584	HTJ0340	No	2	Financial problem
8585	HTJ0341	No	3	Peer pressure
8586	HTJ0342	No	2	Job problem
8587	HTJ0344	No	2	Others
8588	HTJ0346	No	1	Peer pressure
8589	HTJ0347	No	1	Others
8590	HTJ0348	No	1	Others
8591	HTJ0350	Yes	1	Health
8592	HTJ0353	No	3	Relaxation, Peer pressure
8593	HTJ0356	No	2	Peer pressure
8594	HTJ0357	Yes	6	Peer pressure
8595	HTJ0359	No	2	Peer pressure
8596	HTJ0367	No	2	Relaxation, Joy seeking
8597	HTJ0369	No	2	Financial problem
8598	HTJ0370	No	2	Relaxation, Joy seeking
8599	HTJ0371	No	5	Peer pressure
8600	HTJ0373	No	2	Joy seeking, Peer pressure
8601	HTJ0374	Yes	8	Peer pressure
8602	HTJ0376	No	2	Family issue, Joy seeking
8603	HTJ0377	No	3	Relaxation, Peer pressure
8604	HTJ0378	Yes	2	Peer pressure
8605	HTJ0379	No	4	Relationship
8606	HTJ0381	No	2	Others
8607	HTJ0382	No	1	Health
8608	HTJ0384	No	2	Others
8609	HTJ0385	No	1	-
8610	HTJ0387	No	2	Others

No	Patient ID	Other substances	No of substances abused at a time	Reason for substance use initiation
8611	HTJ0388	No	2	Others
8612	HTJ0389	No	1	Others
8613	HTJ0390	No	1	Peer pressure
8614	HTJ0391	No	3	Relationship, Relaxation
8615	HTJ0392	No	2	Others
8616	HTJ0393	No	2	Others
8617	HTJ0394	No	1	Others
8618	HTJ0395	No	2	Others
8619	HTJ0396	No	2	Joy seeking
8620	HTJ0401	Yes	5	Relaxation
8621	HTJ0402	No	2	Relaxation, Peer pressure
8622	HTJ0404	No	2	Job problem
8623	HTJ0405	No	3	Peer pressure
8624	HTJ0406	No	1	Others
8625	HTJ0407	No	3	Joy seeking, Peer pressure
8626	HTJ0408	No	1	Family issue
8627	HTJ0409	No	1	Others
8628	HTJ0410	No	4	Peer pressure
8629	HTJ0412	No	4	Peer pressure
8630	HTJ0413	No	2	Job problem
8631	HTJ0414	No	3	Peer pressure
8632	HTJ0415	No	1	Peer pressure
8633	HTJ0416	No	2	Family issue, Relaxation
8634	HTJ0417	No	1	Family issue
8635	HTJ0418	No	2	Peer pressure
8636	HTJ0419	No	2	Peer pressure
8637	HTJ0421	No	2	-
8638	HTJ0425	No	4	Peer pressure
8639	HTJ0426	No	3	Relaxation, Joy seeking
8640	HTJ0427	No	1	Others
8641	HTJ0429	Yes	8	Peer pressure, Financial problem
8642	HTJ0431	No	2	Peer pressure

No	Patient ID	Other substances	No of substances abused at a time	Reason for substance use initiation
8643	HTJ0432	No	2	Family issue, Peer pressure
8644	HTJ0433	Yes	3	Health, Relationship
8645	HTJ0434	No	2	Relaxation, Joy seeking
8646	HTJ0435	No	1	Health
8647	HTJ0436	No	4	Peer pressure
8648	HTJ0437	No	2	Family issue, Job problem, Relaxation
8649	HTJ0438	Yes	2	Peer pressure
8650	HTJ0439	No	2	Peer pressure
8651	HTJ0440	No	2	Relationship, Financial problem
8652	HTJ0441	No	3	Family issue, Peer pressure
8653	HTJ0442	No	2	Relationship
8654	HTJ0443	No	2	Relaxation, Joy seeking
8655	HTJ0444	No	1	Family issue
8656	HTJ0445	No	4	Peer pressure
8657	HTJ0446	No	2	Relaxation, Peer pressure
8658	HTJ0447	No	2	Family issue
8659	HTJ0448	No	1	Family issue, Job problem
8660	HTJ0449	No	1	Peer pressure
8661	HTJ0450	No	2	Relaxation
8662	HTJ0451	No	1	Others
8663	HTJ0452	No	2	Others
8664	HTJ0453	No	2	Peer pressure
8665	HTJ0454	No	6	Peer pressure
8666	HTJ0455	No	2	Relaxation
8667	HTJ0456	No	1	Family issue
8668	HTJ0457	No	3	Family issue
8669	HTJ0458	No	4	Peer pressure
8670	HTJ0459	No	2	-
8671	HTJ0460	No	3	Peer pressure
8672	HTJ0461	No	2	Peer pressure
8673	HTJ0462	No	2	Relaxation
8674	HTJ0463	No	2	Peer pressure
8675	HTJ0464	Yes	6	Peer pressure
8676	HTJ0465	No	2	Relaxation

No	Patient ID	Other substances	No of substances abused at a time	Reason for substance use initiation
8677	HTJ0466	No	2	Relaxation, Joy seeking, Peer pressure
8678	HTJ0467	No	6	Peer pressure
8679	HTJ0469	No	2	Health, Relaxation
8680	HTJ0470	No	2	Peer pressure
8681	HTJ0471	No	2	Peer pressure
8682	HTJ0472	No	2	Health, Relaxation
8683	HTJ0473	No	3	Job problem
8684	HTJ0474	No	3	Job problem
8685	HTJ0476	No	3	Relaxation, Peer pressure
8686	HTJ0478	No	2	Relaxation
8687	HTJ0480	No	2	Peer pressure
8688	HTJ0485	No	2	Peer pressure
8689	HTJ0486	No	2	Peer pressure
8690	HTJ0487	No	4	Relaxation, Peer pressure
8691	HTJ0488	No	2	Peer pressure
8692	HTJ0489	No	2	Family issue, Job problem
8693	HTJ0490	No	4	Family issue, Peer pressure
8694	HTJ0491	No	1	Relaxation
8695	HTJ0492	No	2	Family issue
8696	HTJ0493	No	1	Others
8697	HTJ0494	No	2	Peer pressure
8698	HTJ0495	Yes	3	Health, Relaxation
8699	HTJ0496	No	5	Peer pressure
8700	HTJ0497	No	2	Job problem
8701	HTJ0498	No	2	Relaxation
8702	HTJ0500	No	2	Peer pressure
8703	HTJ0501	No	2	Peer pressure
8704	HTJ0503	No	2	Peer pressure
8705	HTJ0504	No	2	Relationship
8706	HTJ0505	No	2	Others
8707	HTJ0506	No	1	-
8708	HTJ0507	No	2	Job problem
8709	HTJ0511	No	1	Peer pressure
8710	HTJ0512	No	2	Relaxation
8711	HTJ0513	No	2	Job problem

No	Patient ID	Other substances	No of substances abused at a time	Reason for substance use initiation
8712	HTJ0514	No	3	Peer pressure
8713	HTJ0515	No	5	Peer pressure
8714	HTJ0516	No	4	Peer pressure
8715	HTJ0517	No	1	Peer pressure
8716	HTJ0518	No	3	Peer pressure
8717	HTJ0519	No	6	Peer pressure
8718	HTJ0520	No	5	Peer pressure
8719	HTJ0521	No	2	Peer pressure
8720	HTJ0522	No	2	Family issue
8721	HTJ0523	No	2	Peer pressure
8722	HTJ0524	No	2	Peer pressure
8723	HTJ0525	No	2	Peer pressure
8724	HTJ0527	No	4	Peer pressure
8725	HTJ0528	No	3	Peer pressure
8726	HTJ0529	No	1	Relaxation, Peer pressure
8727	HTJ0530	No	3	Relaxation
8728	HTJ0533	No	4	Relaxation, Joy seeking, Peer pressure
8729	HTJ0534	No	2	Job problem
8730	HTJ0536	No	3	Health, Relaxation
8731	HTJ0537	No	2	Job problem
8732	HTJ0538	No	4	Peer pressure
8733	HTJ0539	No	1	Relaxation, Joy seeking, Peer pressure
8734	HTJ0540	No	2	-
8735	HTJ0542	No	2	Others
8736	HTJ0543	No	2	Relaxation, Joy seeking
8737	HTJ0544	No	1	Family issue
8738	HTJ0545	No	2	Others
8739	HTJ0546	No	2	Relaxation
8740	HTJ0549	No	5	Peer pressure
8741	HTJ0550	No	3	Peer pressure
8742	HTJ0551	No	2	Health, Relaxation
8743	HTJ0552	No	3	Peer pressure
8744	HTJ0553	No	2	Family issue
8745	HTJ0554	No	2	Peer pressure
8746	HTJ0555	No	4	Peer pressure

No	Patient ID	Other substances	No of substances abused at a time	Reason for substance use initiation
8747	HTJ0556	No	2	Relaxation, Joy seeking, Peer pressure
8748	HTJ0557	No	4	Peer pressure
8749	HTJ0558	No	2	Family issue
8750	HTJ0559	No	4	Peer pressure
8751	HTJ0560	No	2	Peer pressure
8752	HTJ0561	No	1	Peer pressure
8753	HTJ0562	No	3	Peer pressure
8754	HTJ0564	No	3	Family issue
8755	HTJ0565	No	2	-
8756	HTJ0566	No	1	-
8757	HTJ0569	No	1	-
8758	HTJ0570	No	3	Relaxation, Joy seeking
8759	HTJ0571	No	2	Family issue, Relaxation
8760	HTJ0572	No	4	Peer pressure
8761	HTJ0573	No	2	Job problem
8762	HTJ0574	No	1	Family issue
8763	HTJ0575	No	3	Peer pressure
8764	HTJ0576	No	3	Family issue, Peer pressure
8765	HTJ0577	No	4	Peer pressure
8766	HTJ0578	No	3	Relaxation, Peer pressure
8767	HTJ0579	No	2	Relaxation, Peer pressure
8768	HTJ0581	No	1	Relaxation
8769	HTJ0582	No	4	Peer pressure
8770	HTJ0583	No	2	Others
8771	HTJ0584	No	2	Others
8772	HTJ0585	No	1	-
8773	HTJ0586	No	4	Peer pressure
8774	HTJ0587	No	2	Others
8775	HTJ0588	Yes	4	Peer pressure
8776	HTJ0589	No	3	Others
8777	HTJ0590	No	2	Peer pressure
8778	HTJ0591	No	2	Relaxation
8779	HTJ0592	No	2	Relaxation, Joy seeking

No	Patient ID	Other substances	No of substances abused at a time	Reason for substance use initiation
8780	HTJ0593	No	1	Peer pressure
8781	HTJ0594	No	4	Peer pressure
8782	HTJ0595	No	2	Job problem, Relaxation
8783	HTJ0596	No	2	Peer pressure
8784	HTJ0597	No	2	Job problem
8785	HTJ0598	No	3	Peer pressure
8786	HTJ0599	No	2	Peer pressure
8787	HTJ0600	No	1	Peer pressure
8788	HTJ0601	No	2	Peer pressure
8789	HTJ0602	No	3	Peer pressure
8790	HTJ0603	Yes	5	Peer pressure
8791	HTJ0604	No	1	Relaxation
8792	HTJ0607	No	4	Peer pressure
8793	HTJ0608	No	1	Relaxation, Joy seeking
8794	HTJ0609	No	3	Peer pressure
8795	HTJ0610	No	1	Peer pressure, Others
8796	HTJ0611	No	1	-
8797	HTJ0612	No	1	Others
8798	HTJ0613	No	1	Others
8799	HTJ0614	No	1	Others
8800	HTJ0615	No	1	Others
8801	HTJ0616	No	3	Peer pressure
8802	HTJ0617	No	1	-
8803	HTJ0624	No	2	Peer pressure
8804	HTJ0630	No	3	Peer pressure
8805	HTJ0631	No	2	Peer pressure
8806	HTJ0633	No	2	Joy seeking, Peer pressure
8807	HTJ0634	Yes	4	Relaxation, Joy seeking
8808	HTJ0636	No	2	Job problem
8809	HTJ0638	No	4	Peer pressure
8810	HTJ0639	No	2	Peer pressure
8811	HTJ0640	No	3	Relationship
8812	HTJ0641	Yes	4	Relationship
8813	HTJ0642	No	3	Peer pressure
8814	HTJ0643	No	3	Health, Others
8815	HTJ0644	No	2	Relationship
8816	HTJ0646	No	3	Peer pressure
8817	HTJ0647	No	1	Health

No	Patient ID	Other substances	No of substances abused at a time	Reason for substance use initiation
8818	HTJ0649	No	2	Peer pressure
8819	HTJ0650	No	2	Health
8820	HTJ0652	No	4	Peer pressure
8821	HTJ0653	No	4	Relaxation, Peer pressure
8822	HTJ0656	No	3	Peer pressure
8823	HTJ0660	No	5	Peer pressure
8824	HTJ0662	No	3	Joy seeking, Peer pressure
8825	HTJ0668	No	4	Peer pressure
8826	HTJ0669	Yes	6	Peer pressure
8827	HTJ0671	No	1	Health, Peer pressure
8828	HTJ0672	No	3	-
8829	HTJ0678	No	1	Joy seeking
8830	HTJ0679	No	3	Peer pressure
8831	HTJ0681	Yes	7	Peer pressure
8832	HTJ0682	No	2	Peer pressure
8833	HTJ0685	Yes	7	Peer pressure
8834	HTJ0687	No	4	Relaxation, Peer pressure
8835	HTJ0688	No	2	Peer pressure
8836	HTJ0689	No	2	Relationship
8837	HTJ0690	Yes	7	Peer pressure
8838	HTJ0691	No	2	Job problem, Financial problem
8839	HTJ0693	No	3	Peer pressure
8840	HTJ0695	No	2	Family issue
8841	HTJ0696	No	2	Peer pressure
8842	HTJ0697	No	2	Job problem, Peer pressure
8843	HTJ0699	No	4	Peer pressure
8844	HTJ0701	No	6	Peer pressure
8845	HTJ0702	No	2	Peer pressure
8846	HTJ0705	No	5	Joy seeking, Peer pressure
8847	HTJ0706	No	4	Peer pressure
8848	HTJ0707	No	4	Family issue, Relaxation
8849	HTJ0710	No	2	-
8850	HTJ0712	No	1	Health
8851	HTJ0715	No	2	Peer pressure
8852	HTJ0716	No	2	Peer pressure
8853	HTJ0719	No	1	Health

No	Patient ID	Other substances	No of substances abused at a time	Reason for substance use initiation
8854	HTJ0721	No	1	Peer pressure
8855	HTJ0722	No	4	-
8856	HTJ0723	No	1	Others
8857	HTJ0724	No	1	Health
8858	HTJ0729	No	1	Family issue, Relaxation
8859	HTJ0730	No	8	Peer pressure
8860	HTJ0731	No	4	Peer pressure
8861	HTJ0732	No	3	Peer pressure
8862	HTJ0735	No	3	Peer pressure
8863	HTJ0739	No	1	Relaxation
8864	HTJ0743	No	4	Peer pressure
8865	HTJ0746	No	2	-
8866	HTJ0747	No	2	Others
8867	HTJ0748	No	1	Others
8868	HTJ0750	No	2	-
8869	HTJ0751	No	5	-
8870	HTJ0752	No	4	-
8871	HTJ0753	No	1	Others
8872	HTJ0755	No	3	Peer pressure
8873	HTJ0756	No	2	Others
8874	HTJ0758	No	2	-
8875	HTJ0760	No	2	Peer pressure
8876	HTJ0761	No	2	Peer pressure
8877	HTJ0762	No	3	Peer pressure
8878	HTJ0763	No	2	Peer pressure
8879	HTJ0764	No	2	Joy seeking, Peer pressure
8880	HTJ0765	No	4	Peer pressure
8881	HTJ0766	No	3	Peer pressure
8882	HTJ0767	No	4	Job problem
8883	HTJ0768	No	4	Peer pressure
8884	HTJ0769	No	2	Job problem
8885	HTJ0770	No	5	Peer pressure
8886	HTJ0771	No	3	Peer pressure
8887	HTJ0772	No	5	Peer pressure

No	Patient ID	Other substances	No of substances abused at a time	Reason for substance use initiation
8888	HTJ0773	No	2	Job problem
8889	HTJ0774	No	2	Job problem, Peer pressure
8890	HTJ0775	No	3	Peer pressure
8891	HTJ0776	No	2	Peer pressure
8892	HTJ0777	No	3	Peer pressure
8893	HTJ0778	No	2	Peer pressure
8894	HTJ0779	No	2	Peer pressure
8895	HTJ0780	No	4	Relaxation, Joy seeking, Peer pressure
8896	HTJ0781	No	3	Joy seeking, Peer pressure
8897	HTJ0782	No	4	Peer pressure
8898	HTJ0783	No	2	Job problem
8899	HTJ0784	No	3	Peer pressure
8900	HTJ0785	No	2	Family issue
8901	HTJ0786	No	3	Peer pressure
8902	HTJ0787	No	3	Peer pressure
8903	HTJ0788	No	3	Peer pressure
8904	HTJ0789	No	2	Job problem
8905	HTJ0790	No	2	Peer pressure
8906	HTJ0791	Yes	5	Family issue, Peer pressure
8907	HTJ0792	No	4	Peer pressure
8908	HTJ0793	No	2	Relationship
8909	HTJ0794	No	3	Joy seeking, Peer pressure
8910	HTJ0795	No	2	Relationship
8911	HTJ0796	No	4	Peer pressure
8912	HTJ0797	No	2	-
8913	HTJ0798	No	1	Others
8914	HTJ0799	No	3	-
8915	HTJ0800	No	2	Others
8916	HTJ0801	No	3	-
8917	HTJ0802	No	1	Peer pressure
8918	HTJ0804	No	2	Peer pressure
8919	HTJ0806	No	2	Relaxation
8920	HTJ0809	No	2	-
8921	HTJ0811	No	2	Peer pressure
8922	HTJ0812	No	4	Peer pressure
8923	HTJ0813	No	2	Peer pressure

No	Patient ID	Other substances	No of substances abused at a time	Reason for substance use initiation
8924	HTJ0814	No	4	Peer pressure
8925	HTJ0815	No	3	Peer pressure
8926	HTJ0816	No	3	Peer pressure
8927	HTJ0817	No	3	Peer pressure
8928	HTJ0818	No	2	Family issue, Peer pressure
8929	HTJ0819	No	2	Peer pressure
8930	HTJ0820	No	2	Peer pressure
8931	HTJ0821	No	2	Family issue
8932	HTJ0822	Yes	6	Family issue
8933	HTJ0823	No	3	Peer pressure
8934	HTJ0824	No	3	Relationship
8935	HTJ0825	Yes	7	Peer pressure
8936	HTJ0826	Yes	5	Peer pressure
8937	HTJ0827	No	2	Relaxation, Joy seeking
8938	HTJ0828	No	3	Peer pressure
8939	HTJ0829	No	2	Peer pressure
8940	HTJ0830	No	2	Others
8941	HTJ0831	No	2	Relaxation, Joy seeking
8942	HTJ0832	No	5	Peer pressure
8943	HTJ0833	No	2	-
8944	HTJ0834	No	3	Relaxation
8945	HTJ0835	No	2	-
8946	HTJ0836	No	1	Others
8947	HTJ0837	No	2	-
8948	HTJ0838	No	1	Health
8949	HTJ0839	No	4	-
8950	HTJ0840	No	4	Peer pressure
8951	HTJ0841	No	2	Joy seeking, Peer pressure
8952	HTJ0842	No	5	Peer pressure
8953	HTJ0843	No	2	Relaxation, Joy seeking, Peer pressure
8954	HTJ0844	No	3	Peer pressure
8955	HTJ0845	No	2	Job problem, Relaxation
8956	HTJ0846	No	2	Peer pressure
8957	HTJ0847	No	3	Peer pressure
8958	HTJ0848	No	2	Peer pressure
8959	HTJ0849	No	3	Peer pressure

No	Patient ID	Other substances	No of substances abused at a time	Reason for substance use initiation
8960	HTJ0850	No	2	Peer pressure
8961	HTJ0851	No	4	Peer pressure
8962	HTJ0852	No	1	Relaxation, Peer pressure
8963	HTJ0853	No	1	Family issue
8964	HTJ0854	No	2	Peer pressure
8965	HTJ0855	No	4	Peer pressure
8966	HTJ0856	No	2	Peer pressure
8967	HTJ0857	No	4	Relaxation
8968	HTJ0858	No	3	Peer pressure
8969	HTJ0859	Yes	5	Peer pressure
8970	HTJ0860	Yes	6	Peer pressure
8971	HTJ0861	No	3	Family issue, Peer pressure
8972	HTJ0862	No	3	Peer pressure
8973	HTJ0863	No	4	Peer pressure
8974	HTJ0864	No	2	Family issue
8975	HTJ0865	No	4	Peer pressure
8976	HTJ0866	Yes	6	Family issue, Others
8977	HTJ0867	No	5	Peer pressure
8978	HTJ0868	No	2	Health
8979	HTJ0869	No	3	Peer pressure
8980	HTJ0871	No	2	Peer pressure, Others
8981	HTJ0872	No	1	Others
8982	HTJ0877	No	2	Relationship
8983	HTJ0878	No	1	Relaxation
8984	HTJ0880	No	4	Peer pressure
8985	HTJ0881	No	4	Peer pressure
8986	HTJ0882	No	3	Relationship
8987	HTJ0883	No	2	Relaxation, Peer pressure
8988	HTJ0885	No	4	Peer pressure
8989	HTJ0886	No	4	Peer pressure
8990	HTJ0887	No	2	Family issue
8991	HTJ0888	No	2	Peer pressure
8992	HTJ0889	No	2	Job problem
8993	HTJ0890	No	1	Job problem, Relaxation
8994	HTJ0891	No	3	Family issue
8995	HTJ0892	No	2	Peer pressure
8996	HTJ0893	No	2	Health, Relaxation
8997	HTJ0894	No	3	Peer pressure

No	Patient ID	Other substances	No of substances abused at a time	Reason for substance use initiation
8998	HTJ0896	No	3	Peer pressure
8999	HTJ0898	Yes	6	Peer pressure
9000	HTJ0899	No	3	Peer pressure
9001	HTJ0904	No	2	Peer pressure
9002	HTJ0905	No	2	Peer pressure
9003	HTJ0906	No	3	Peer pressure
9004	HTJ0907	Yes	5	Peer pressure
9005	HTJ0908	No	6	Peer pressure
9006	HTJ0909	No	3	Relationship
9007	HTJ0910	No	2	Joy seeking, Peer pressure
9008	HTJ0911	No	2	Peer pressure
9009	HTJ0912	No	2	Relationship, Relaxation
9010	HTJ0915	No	2	Job problem
9011	HTJ0916	No	3	Job problem, Relaxation
9012	HTJ0917	No	4	Peer pressure
9013	HTJ0918	No	2	Family issue
9014	HTJ0919	No	1	Joy seeking
9015	HTJ0920	No	2	Peer pressure
9016	HTJ0921	No	2	Peer pressure
9017	HTJ0922	No	3	Relaxation, Others
9018	HTJ0923	No	2	Peer pressure
9019	HTJ0924	No	4	Peer pressure
9020	HTJ0925	No	4	Peer pressure
9021	HTJ0926	No	2	Relaxation, Joy seeking
9022	HTJ0927	No	2	Joy seeking, Peer pressure
9023	HTJ0928	No	2	Financial problem
9024	HTJ0929	No	2	Peer pressure
9025	HTJ0930	No	2	-
9026	HTJ0931	No	3	Job problem, Peer pressure
9027	HTJ0932	No	2	Relationship
9028	HTJ0933	No	3	Peer pressure
9029	HTJ0934	No	2	Peer pressure
9030	HTJ0935	No	2	Relaxation, Peer pressure
9031	HTJ0936	Yes	4	Job problem, Peer pressure
9032	HTJ0937	No	2	Peer pressure

No	Patient ID	Other substances	No of substances abused at a time	Reason for substance use initiation
9033	HTJ0938	No	5	Peer pressure
9034	HTJ0939	No	2	Joy seeking, Peer pressure
9035	HTJ0940	No	2	-
9036	HTJ0941	No	2	-
9037	HTJ0942	No	2	Peer pressure
9038	HTJ0945	No	2	Others
9039	HTJ0947	No	2	Others
9040	HTJ0948	No	1	Others
9041	HTJ0949	No	2	-
9042	HTJ0950	No	7	Peer pressure
9043	HTJ0951	No	3	Family issue
9044	HTJ0952	No	4	Peer pressure
9045	HTJ0953	No	4	Peer pressure
9046	HTJ0954	No	4	Peer pressure
9047	HTJ0955	Yes	6	Peer pressure
9048	HTJ0956	No	2	Job problem, Relaxation
9049	HTJ0957	No	3	Peer pressure
9050	HTJ0958	No	3	Peer pressure
9051	HTJ0959	No	2	Family issue, Job problem, Financial problem
9052	HTJ0960	Yes	5	Family issue, Peer pressure
9053	HTJ0961	No	5	Peer pressure
9054	HTJ0962	No	3	Peer pressure
9055	HTJ0963	No	2	Family issue, Relaxation
9056	HTJ0964	No	2	Joy seeking, Peer pressure
9057	HTJ0965	No	1	Peer pressure
9058	HTJ0966	No	3	Relaxation
9059	HTJ0967	No	2	Peer pressure
9060	HTJ0968	No	4	Peer pressure
9061	HTJ0969	No	2	Job problem, Relaxation

No	Patient ID	Other substances	No of substances abused at a time	Reason for substance use initiation
9062	HTJ0970	No	3	-
9063	HTJ0971	No	1	Others
9064	HTJ0972	No	2	Others
9065	HTJ0973	No	2	Peer pressure
9066	HTJ0974	No	2	-
9067	HTJ0975	No	2	-
9068	HTJ0979	No	2	Others
9069	HTJ0980	No	2	-
9070	HTJ0981	No	2	Joy seeking
9071	HTJ0983	No	2	Others
9072	HTJ0985	No	3	Peer pressure
9073	HTJ0986	No	3	Peer pressure
9074	HTJ0987	No	4	Relaxation, Peer pressure
9075	HTJ0988	No	1	Peer pressure
9076	HTJ0989	No	2	Joy seeking
9077	HTJ0990	No	5	Peer pressure
9078	HTJ0991	No	2	Joy seeking
9079	HTJ0992	No	1	Peer pressure
9080	HTJ0993	No	3	Peer pressure
9081	HTJ0995	No	3	Peer pressure
9082	HTJ0996	No	4	Family issue, Peer pressure
9083	HTJ0997	No	3	Peer pressure
9084	HTJ0998	No	3	Joy seeking
9085	HTJ1000	No	2	Family issue
9086	HTJ1001	No	2	-
9087	HTJ1002	No	3	Others
9088	HTJ1003	No	1	Others
9089	HTJ1004	No	2	Others
9090	HTJ1005	No	3	Financial problem

No	Patient ID	Other substances	No of substances abused at a time	Reason for substance use initiation
9091	HTJ1006	No	2	Peer pressure
9092	HTJ1007	No	2	Peer pressure
9093	HUS0002	No	1	Relaxation
9094	HUS0005	No	1	Peer pressure
9095	HUS0008	No	1	Relaxation, Peer pressure
9096	HUS0010	No	1	Relaxation, Joy seeking, Peer pressure
9097	HUS0011	No	1	Job problem
9098	HUS0012	No	1	Health
9099	HUS0013	No	1	Joy seeking, Peer pressure
9100	HUS0014	No	1	-
9101	HUS0015	No	1	Relaxation, Peer pressure
9102	HUS0016	No	1	Others
9103	HUS0017	No	1	Relaxation, Joy seeking, Peer pressure
9104	HUS0019	No	1	Family issue, Joy seeking
9105	HUS0020	No	1	-
9106	HUS0021	No	1	Relaxation, Joy seeking
9107	HUS0022	No	2	-
9108	HUS0023	No	1	Health, Relaxation
9109	HUS0026	No	1	-
9110	HUS0029	No	1	Peer pressure
9111	HUS0032	No	1	Relaxation, Joy seeking, Peer pressure
9112	HUS0035	No	1	Peer pressure
9113	HUS0040	No	2	Peer pressure
9114	HUS0041	No	1	Relaxation, Joy seeking, Peer pressure
9115	HUS0045	No	1	Relaxation, Joy seeking, Peer pressure
9116	HUS0046	No	2	Relaxation, Joy seeking, Others

No	Patient ID	Other substances	No of substances abused at a time	Reason for substance use initiation
9117	HUS0047	No	2	-
9118	HUS0048	No	1	Relaxation, Others
9119	HUS0049	No	1	Joy seeking, Peer pressure
9120	HUS0050	No	1	Relaxation, Joy seeking, Peer pressure
9121	HUS0051	No	2	-
9122	HUS0052	No	1	Health, Relaxation
9123	HUS0054	No	1	Family issue, Relaxation, Joy seeking, Peer pressure
9124	HUS0055	No	1	Family issue, Relaxation, Joy seeking
9125	HUS0057	No	1	Health, Relaxation
9126	HUS0058	No	1	Family issue, Relaxation
9127	HUS0059	No	1	Relaxation, Joy seeking, Others
9128	HUS0061	No	1	-
9129	HUS0063	No	1	Relaxation, Joy seeking, Peer pressure
9130	HUS0064	No	2	Relaxation, Joy seeking, Peer pressure
9131	HUS0065	No	1	Joy seeking, Peer pressure
9132	HUS0067	No	1	Family issue, Relaxation
9133	HUS0068	No	3	Relaxation, Joy seeking, Peer pressure
9134	HUS0069	No	2	Peer pressure
9135	HUS0070	No	3	Relaxation, Joy seeking, Peer pressure
9136	HUS0071	No	4	-
9137	HUS0072	No	1	Job problem
9138	HUS0073	No	1	Relaxation
9139	HUS0074	No	2	Relaxation, Joy seeking
9140	HUS0075	No	3	Job problem
9141	HUS0076	No	3	Relaxation, Joy seeking, Peer pressure
9142	HUS0077	No	3	Job problem
9143	HUS0078	No	3	Relaxation

No	Patient ID	Other substances	No of substances abused at a time	Reason for substance use initiation
9144	HUS0079	No	1	Relationship, Relaxation, Joy seeking
9145	HUS0080	No	2	Peer pressure
9146	HUS0081	No	1	Others
9147	HUS0082	No	1	Peer pressure
9148	HUS0083	No	3	Relaxation, Joy seeking
9149	HUS0084	Yes	4	Peer pressure
9150	HUS0088	No	2	Relaxation, Joy seeking, Peer pressure
9151	HUS0090	No	1	-
9152	HUS0091	No	2	Relaxation, Joy seeking
9153	HUS0092	No	5	Peer pressure
9154	HUS0093	No	3	Family issue
9155	HUS0094	No	2	Family issue, Relaxation
9156	HUS0096	No	4	Peer pressure
9157	HUS0097	No	3	Joy seeking, Peer pressure
9158	HUS0098	No	1	Relaxation, Peer pressure
9159	HUS0099	No	5	Relaxation
9160	HUS0100	No	1	Job problem
9161	HUS0101	No	2	Joy seeking
9162	HUS0103	No	3	Peer pressure
9163	HUS0104	No	3	Relaxation
9164	HUS0105	No	2	Relaxation, Joy seeking
9165	HUS0106	No	3	Peer pressure, Others
9166	HUS0107	No	3	Relaxation, Financial problem
9167	HUS0108	No	2	Peer pressure
9168	HUS0109	No	1	Relaxation
9169	HUS0110	No	1	Relaxation, Peer pressure
9170	HUS0112	No	1	Family issue, Relaxation
9171	HUS0113	No	1	Peer pressure
9172	HUS0114	No	1	Relaxation, Joy seeking
9173	HUS0115	No	1	-
9174	HUS0116	No	3	Relaxation, Joy seeking

No	Patient ID	Other substances	No of substances abused at a time	Reason for substance use initiation
9175	HUS0117	No	2	Peer pressure
9176	HUS0118	No	2	Relaxation, Joy seeking, Peer pressure
9177	HUS0119	No	1	Relaxation, Joy seeking
9178	HUS0121	No	3	Relaxation
9179	HUS0122	No	1	Joy seeking, Peer pressure
9180	HUS0124	No	1	Relaxation, Peer pressure
9181	HUS0125	No	1	Relaxation
9182	HUS0129	No	1	Relaxation, Joy seeking, Peer pressure
9183	HUS0130	No	3	Relationship
9184	HUS0131	No	1	-
9185	HUS0132	No	2	Relaxation
9186	HUS0133	No	2	Relaxation, Joy seeking
9187	HUS0134	No	2	Family issue
9188	HUS0135	No	1	-
9189	HUS0136	No	1	-
9190	HUS0137	No	2	Relationship
9191	HUS0138	No	3	Peer pressure
9192	HUS0139	No	3	Family issue, Peer pressure
9193	HUS0140	No	1	Relaxation
9194	HUS0141	No	1	Relaxation
9195	HUS0142	No	1	Relaxation
9196	HUS0144	No	1	Relaxation
9197	HUS0146	No	1	-
9198	HUS0147	No	3	Job problem
9199	HUS0152	No	3	Others
9200	HUS0153	No	2	Peer pressure
9201	HUS0156	No	2	Peer pressure
9202	HUS0157	No	1	Relaxation
9203	HUS0158	No	1	Family issue
9204	HUS0159	No	2	Relaxation, Joy seeking
9205	HUS0161	No	1	Joy seeking
9206	HUS0162	No	2	Peer pressure
9207	HUS0163	No	2	Peer pressure
9208	HUS0164	No	1	Relaxation, Joy seeking
9209	HUS0165	No	1	Relaxation
9210	HUS0166	No	2	Peer pressure
9211	HUS0167	No	2	Relaxation

No	Patient ID	Other substances	No of substances abused at a time	Reason for substance use initiation
9212	HUS0168	No	4	Relaxation
9213	HUS0170	No	2	Peer pressure
9214	HUS0171	No	1	Joy seeking, Peer pressure
9215	HUS0172	No	1	Relaxation, Joy seeking, Peer pressure
9216	HUS0173	No	2	Job problem, Peer pressure
9217	HUS0174	No	3	Joy seeking, Peer pressure
9218	HUS0175	No	2	Peer pressure
9219	HUS0176	No	2	-
9220	HUS0177	No	1	-
9221	HUS0178	No	2	Others
9222	HUS0179	No	1	Peer pressure
9223	HUS0180	No	2	Joy seeking, Peer pressure
9224	HUS0181	No	1	-
9225	HUS0182	No	4	-
9226	HUS0184	No	2	Relaxation, Joy seeking
9227	HUS0187	No	4	Relaxation, Others
9228	HUS0187	No	4	Relaxation, Others
9229	HUS0189	No	1	Relaxation
9230	HUS0192	No	1	Relaxation, Joy seeking
9231	HUS0193	No	2	Peer pressure
9232	HUS0194	No	2	Peer pressure
9233	HUS0195	No	2	Relaxation
9234	HUS0196	No	1	Peer pressure
9235	HUS0197	No	2	Others
9236	HUS0198	No	4	Peer pressure
9237	HUS0199	No	1	Peer pressure
9238	HUS0200	No	3	Joy seeking, Peer pressure
9239	HUS0201	No	4	Relaxation
9240	HUS0202	No	2	Relaxation, Peer pressure
9241	HUS0204	No	1	-
9242	HUS0205	No	1	Relaxation, Joy seeking
9243	HUS0206	No	3	Peer pressure, Others

No	Patient ID	Other substances	No of substances abused at a time	Reason for substance use initiation
9244	HUS0207	No	2	Joy seeking, Peer pressure
9245	HUS0210	No	1	Relationship
9246	HUS0211	No	1	Relaxation
9247	HUS0212	No	2	Relaxation, Joy seeking
9248	HUS0213	No	2	-
9249	HUS0214	No	1	Peer pressure
9250	HUS0215	No	1	Relaxation, Joy seeking
9251	HUS0216	No	1	Others
9252	HUS0217	No	1	Relaxation
9253	HUS0218	No	1	Relaxation
9254	HUS0219	No	3	-
9255	HUS0221	No	2	Family issue, Job problem
9256	HUS0222	No	3	Joy seeking, Peer pressure
9257	HUS0223	Yes	5	Peer pressure
9258	HUS0224	No	2	Peer pressure
9259	HUS0225	Yes	2	Others
9260	HUS0227	No	2	Peer pressure
9261	HUS0228	No	2	-
9262	HUS0229	No	3	Relaxation, Peer pressure
9263	HUS0230	No	1	Peer pressure
9264	HUS0231	No	1	Family issue
9265	HUS0232	No	1	Relaxation
9266	HUS0233	No	1	Peer pressure
9267	HUS0234	No	2	-
9268	HUS0235	No	2	Relaxation, Peer pressure
9269	HUS0237	No	4	Joy seeking, Peer pressure
9270	HUS0238	No	2	Peer pressure
9271	HUS0239	No	2	Peer pressure
9272	HUS0240	No	3	Others
9273	HUS0242	No	2	-
9274	HUS0243	No	2	Family issue, Peer pressure
9275	HUS0244	No	1	Relaxation, Peer pressure

No	Patient ID	Other substances	No of substances abused at a time	Reason for substance use initiation
9276	HUS0245	No	2	Peer pressure
9277	HUS0246	No	2	-
9278	HUS0247	No	3	Peer pressure
9279	HUS0248	No	2	Peer pressure
9280	HUS0249	No	2	Relationship, Joy seeking, Others
9281	HUS0250	No	2	-
9282	HUS0252	No	1	Peer pressure
9283	HUS0254	No	2	Peer pressure
9284	HUS0256	No	1	-
9285	HUS0258	No	2	-
9286	HUS0262	No	2	Relaxation, Joy seeking
9287	HUS0263	No	4	Relaxation, Peer pressure
9288	HUS0264	No	1	Relaxation, Joy seeking
9289	HUS0268	No	2	Relaxation, Joy seeking
9290	HUS0269	No	2	Family issue, Relationship
9291	HUS0270	No	1	Relaxation
9292	HUS0271	No	1	Peer pressure
9293	HUS0272	No	3	Relaxation, Joy seeking
9294	HUS0273	No	1	Others
9295	HUS0274	No	3	Relaxation
9296	HUS0275	No	2	Relationship
9297	HUS0276	No	2	Peer pressure, Others
9298	HUS0277	No	2	Relaxation
9299	HUS0278	No	2	-
9300	HUS0280	No	2	Others
9301	HUS0281	No	1	Peer pressure
9302	HUS0282	No	1	Others
9303	HUS0283	No	1	Others
9304	HUS0284	No	3	Relaxation
9305	HUS0285	No	2	-
9306	HUS0287	No	4	Relaxation, Joy seeking
9307	HUS0289	No	1	-
9308	HUS0291	No	1	-
9309	HUS0292	No	2	-
9310	HUS0293	No	1	Peer pressure, Others
9311	HUS0294	No	1	Relaxation

No	Patient ID	Other substances	No of substances abused at a time	Reason for substance use initiation
9312	HUS0295	No	1	-
9313	HUS0296	No	2	Family issue
9314	HUS0297	No	2	Peer pressure
9315	HUS0298	No	1	Peer pressure, Others
9316	HUS0300	No	4	Peer pressure
9317	HUS0302	No	3	Peer pressure
9318	HUS0304	No	1	-
9319	HUS0306	No	2	-
9320	HUS0308	No	2	Relaxation, Peer pressure
9321	HUS0309	No	2	Relaxation
9322	HUS0310	No	2	Peer pressure, Others
9323	HUS0311	No	4	Peer pressure, Others
9324	HUS0312	No	2	Relaxation
9325	HUS0313	No	2	Relaxation
9326	HUS0315	No	2	-
9327	HUS0316	No	4	Peer pressure, Others
9328	HUS0317	No	3	Peer pressure
9329	HUS0318	No	2	Peer pressure
9330	HUS0319	No	1	Peer pressure
9331	HUS0320	No	2	Relaxation
9332	HUS0323	No	3	Peer pressure
9333	HUS0324	No	1	-
9334	HUS0325	No	2	-
9335	HUS0326	No	2	Peer pressure
9336	HUS0327	No	1	-
9337	HUS0328	No	1	Peer pressure
9338	HUS0329	No	1	Relaxation, Joy seeking, Peer pressure
9339	HUS0331	No	4	Others
9340	HUS0332	No	4	Peer pressure
9341	HUS0334	No	1	-
9342	HUS0335	No	2	Relaxation, Peer pressure
9343	HUS0338	No	1	-
9344	HUS0339	No	1	-
9345	HUS0341	No	3	-
9346	HUS0342	No	1	-
9347	HUS0343	Yes	1	-
9348	HUS0345	No	3	-

No	Patient ID	Other substances	No of substances abused at a time	Reason for substance use initiation
9349	HUS0346	No	2	-
9350	HUS0347	No	3	Others
9351	HUS0348	No	2	Family issue
9352	HUS0349	No	2	-
9353	HUS0351	No	1	Peer pressure
9354	HUS0352	No	1	Relationship
9355	HUS0353	No	2	Relaxation
9356	HUS0354	No	1	Peer pressure
9357	HUS0355	No	2	Peer pressure
9358	HUS0356	No	2	-
9359	HUS0359	No	1	Others
9360	HUS0360	No	3	Peer pressure
9361	HUS0363	No	3	Peer pressure
9362	HUS0364	No	2	Relaxation
9363	HUS0365	No	2	Peer pressure
9364	HUS0366	No	2	Peer pressure
9365	HUS0367	No	1	Relaxation
9366	HUS0368	No	2	-
9367	HUS0369	No	1	Peer pressure
9368	HUS0372	No	2	-
9369	HUS0374	No	3	-
9370	HUS0376	No	1	Peer pressure
9371	HUS0377	No	1	-
9372	HUS0378	No	2	Peer pressure
9373	HUS0379	No	2	-
9374	HUS0380	No	1	-
9375	HUS0381	No	1	-
9376	HUS0382	No	2	Peer pressure
9377	HUS0383	No	2	-
9378	HUS0384	No	1	Family issue
9379	HUS0385	No	2	-
9380	HUS0386	No	2	Relaxation
9381	HUS0387	No	1	Peer pressure
9382	HUS0388	No	1	-
9383	HUS0389	No	2	-
9384	HUS0390	No	2	Peer pressure
9385	HUS0391	No	4	Others
9386	HUS0393	No	1	-
9387	HUS0394	No	1	Peer pressure
9388	HUS0395	No	2	-

No	Patient ID	Other substances	No of substances abused at a time	Reason for substance use initiation
9389	HUS0396	No	2	-
9390	HUS0397	No	2	Joy seeking, Peer pressure
9391	HUS0398	No	2	-
9392	HUS0400	No	3	Relaxation, Peer pressure
9393	HUS0402	No	1	-
9394	HUS0403	No	3	-
9395	HUS0404	No	3	Peer pressure
9396	HUS0405	No	2	Relaxation
9397	HUS0406	No	2	Family issue
9398	HUS0407	No	2	Relaxation
9399	HUS0408	No	1	-
9400	HUS0409	No	3	Others
9401	HUS0410	No	4	-
9402	HUS0411	No	4	-
9403	HUS0412	No	1	-
9404	HUS0413	No	3	-
9405	HUS0414	No	2	-
9406	HUS0415	No	2	Relationship
9407	HUS0416	No	2	Peer pressure
9408	HUS0417	No	2	Peer pressure
9409	HUS0418	No	2	-
9410	HUS0419	No	4	Peer pressure
9411	HUS0423	No	2	Family issue
9412	HUS0426	No	1	Peer pressure
9413	HUS0427	No	2	Peer pressure, Others
9414	HUS0428	No	2	Others
9415	HUS0430	No	2	Relaxation
9416	HUS0431	No	2	-
9417	HUS0435	No	2	Peer pressure
9418	HUS0436	No	3	Peer pressure
9419	HUS0438	No	3	Others
9420	HUS0439	No	3	Peer pressure, Others
9421	HUS0440	No	1	Peer pressure
9422	HUS0441	No	2	Peer pressure
9423	HUS0443	No	4	Others
9424	HUS0444	No	3	Peer pressure, Others
9425	HUS0446	No	3	Others

No	Patient ID	Other substances	No of substances abused at a time	Reason for substance use initiation
9426	HUS0449	No	2	Relaxation
9427	HUS0450	No	2	Others
9428	HUS0452	No	3	Peer pressure
9429	HUS0453	No	2	Peer pressure
9430	HUS0454	No	4	Peer pressure
9431	HUS0455	No	2	Relaxation
9432	HUS0457	No	2	Peer pressure
9433	HUS0458	No	2	Relaxation, Peer pressure
9434	HUS0461	No	2	Joy seeking, Peer pressure
9435	HUS0463	No	3	Others
9436	HUS0464	No	3	Peer pressure
9437	HUS0465	No	3	Others
9438	HUS0466	No	1	Peer pressure
9439	HUS0468	No	2	Peer pressure
9440	HUS0469	No	1	Relaxation
9441	HUS0470	No	3	Relaxation, Peer pressure
9442	HUS0472	No	2	Peer pressure
9443	HUS0477	No	2	Relaxation, Peer pressure
9444	HUS0479	No	1	Family issue
9445	HUS0480	Yes	4	-
9446	HUS0485	No	2	Relaxation, Peer pressure
9447	HUS0486	No	2	Others
9448	HUS0491	No	2	Peer pressure
9449	HUS0492	No	2	Relaxation
9450	HUS0493	No	2	-
9451	HUS0494	No	2	Relaxation, Joy seeking
9452	HUS0497	No	3	Others
9453	HUS0498	No	2	Relaxation
9454	HUS0501	Yes	5	Joy seeking, Peer pressure
9455	HUS0503	No	2	Relaxation
9456	HUS0505	No	3	Peer pressure
9457	HUS0506	No	2	Others
9458	HUS0507	No	2	Relaxation
9459	HUS0508	No	3	Peer pressure
9460	HUS0510	No	2	-
9461	HUS0513	No	2	Peer pressure

No	Patient ID	Other substances	No of substances abused at a time	Reason for substance use initiation
9462	HUS0516	No	2	Joy seeking, Peer pressure
9463	HUS0517	No	2	Peer pressure
9464	HUS0518	No	5	Peer pressure
9465	HUS0519	No	2	Peer pressure
9466	HUS0520	No	2	Peer pressure
9467	HUS0521	No	2	Peer pressure
9468	HUS0522	No	2	-
9469	HUS0523	No	2	Peer pressure
9470	HUS0524	No	2	-
9471	HUS0525	No	1	-
9472	HUS0526	No	2	Peer pressure
9473	HUS0527	No	2	Others
9474	HUS0528	No	3	Peer pressure
9475	HUS0529	No	3	Others
9476	HUS0530	No	2	-
9477	HUS0531	No	2	Peer pressure
9478	HUS0534	No	1	Peer pressure
9479	HUS0535	No	2	Peer pressure
9480	HUS0536	No	3	Others
9481	HUS0539	No	3	Peer pressure
9482	HUS0540	No	2	-
9483	HUS0542	No	2	-
9484	HUS0543	No	1	Relaxation
9485	HUS0544	No	2	Peer pressure
9486	HUS0545	No	3	Others
9487	HUS0546	No	1	-
9488	HUS0547	No	2	Peer pressure
9489	HUS0549	No	2	-
9490	HUS0550	No	2	Peer pressure
9491	HUS0551	No	2	Peer pressure
9492	HUS0552	No	3	Peer pressure
9493	HUS0553	No	2	-
9494	HUS0554	No	3	Peer pressure
9495	HUS0555	No	4	Peer pressure
9496	HUS0556	No	2	Peer pressure
9497	HUS0558	No	3	Joy seeking, Peer pressure
9498	HUS0559	No	1	Relaxation
9499	HUS0561	No	3	-
9500	HUS0563	Yes	3	Peer pressure
9501	HUS0565	No	2	Others
9502	HUS0566	No	2	Peer pressure

No	Patient ID	Other substances	No of substances abused at a time	Reason for substance use initiation
9503	HUS0567	No	2	-
9504	HUS0568	No	2	Joy seeking
9505	HUS0571	No	3	-
9506	HUS0574	No	2	Peer pressure
9507	HUS0575	No	1	Peer pressure
9508	HUS0577	No	3	Peer pressure
9509	HUS0578	No	2	-
9510	HUS0579	No	2	Peer pressure
9511	HUS0580	No	2	Peer pressure
9512	HUS0581	No	3	Peer pressure
9513	HUS0582	No	3	Peer pressure
9514	HUS0584	No	3	Peer pressure
9515	HUS0585	No	2	Peer pressure
9516	HUS0587	No	3	Joy seeking, Peer pressure
9517	HUS0588	No	3	Peer pressure
9518	HUS0589	No	2	Relaxation
9519	HUS0590	No	2	Family issue
9520	HUS0591	Yes	5	Peer pressure
9521	HUS0592	No	1	Peer pressure
9522	HUS0593	No	2	Family issue
9523	HUS0594	No	2	Relaxation
9524	HUS0595	No	2	Relaxation
9525	HUS0596	No	3	Peer pressure
9526	HUS0597	No	2	Peer pressure
9527	HUS0598	No	2	Relaxation
9528	HUS0599	No	4	Joy seeking, Peer pressure
9529	HUS0600	No	1	Relaxation
9530	HUS0601	No	2	Peer pressure
9531	HUS0602	No	2	Peer pressure
9532	HUS0603	No	4	Others
9533	HUS0604	No	4	Peer pressure
9534	HUS0605	No	2	-
9535	HUS0606	No	2	Relaxation
9536	HUS0607	No	2	Peer pressure
9537	HUS0609	No	3	Peer pressure
9538	HUS0610	Yes	3	Others
9539	HUS0611	No	3	Family issue
9540	HUS0612	No	2	Relaxation, Joy seeking
9541	HUS0613	No	2	Relaxation, Joy seeking
9542	HUS0614	No	2	-
9543	HUS0615	No	1	Relaxation

No	Patient ID	Other substances	No of substances abused at a time	Reason for substance use initiation
9544	HUS0616	No	2	-
9545	HUS0618	No	3	Peer pressure
9546	HUS0619	No	2	Family issue
9547	HUS0620	No	2	Peer pressure
9548	HUS0621	No	3	Peer pressure
9549	HUS0622	No	1	Peer pressure
9550	HUS0623	No	2	Relaxation
9551	HUS0625	No	3	Peer pressure
9552	HUS0626	No	4	Peer pressure
9553	HUS0627	No	1	Family issue
9554	HUS0628	No	2	Peer pressure
9555	HUS0629	No	1	Peer pressure
9556	HUS0631	No	3	Peer pressure
9557	HUS0635	No	2	Others
9558	HUS0636	No	2	Relaxation
9559	HUS0637	No	2	Peer pressure
9560	HUS0640	No	3	Peer pressure
9561	HUS0643	No	2	Peer pressure
9562	HUS0644	No	2	-
9563	HUS0645	No	2	Relaxation, Joy seeking
9564	HUS0646	No	3	Peer pressure
9565	HUS0647	No	2	Joy seeking, Peer pressure
9566	HUS0648	No	2	Relaxation
9567	HUS0652	No	2	-
9568	HUS0653	No	2	Relaxation
9569	HUS0655	No	2	Relaxation
9570	HUS0656	No	3	Peer pressure
9571	HUS0657	No	2	Peer pressure
9572	HUS0658	No	3	Relaxation, Peer pressure
9573	HUS0660	No	3	Relaxation, Joy seeking
9574	HUS0662	No	2	Peer pressure
9575	HUS0665	No	3	Peer pressure
9576	HUS0671	No	2	-
9577	HUS0672	No	2	Family issue
9578	HUS0673	No	2	Family issue
9579	HUS0675	No	1	Relaxation, Peer pressure
9580	HUS0676	No	2	Relaxation
9581	HUS0677	No	1	Family issue
9582	HUS0678	No	3	Peer pressure

No	Patient ID	Other substances	No of substances abused at a time	Reason for substance use initiation
9583	HUS0679	No	2	Peer pressure
9584	HUS0681	No	2	Peer pressure
9585	HUS0682	No	2	-
9586	HUS0683	No	3	Peer pressure
9587	HUS0684	No	3	Peer pressure
9588	HUS0685	No	3	Peer pressure
9589	HUS0686	No	2	-
9590	HUS0688	No	2	Peer pressure
9591	HUS0689	No	2	Relaxation
9592	HUS0690	No	2	Job problem
9593	HUS0691	No	2	Peer pressure
9594	HUS0693	No	2	Peer pressure
9595	HUS0696	No	2	Peer pressure
9596	HUS0697	No	2	Peer pressure
9597	HUS0701	No	2	Peer pressure
9598	HUS0702	No	1	Family issue
9599	HUS0704	No	2	Peer pressure
9600	HUS0705	No	3	Peer pressure
9601	HUS0706	No	2	Relaxation
9602	HUS0707	No	2	Peer pressure
9603	HUS0708	No	2	Relaxation
9604	HUS0711	No	2	Relaxation
9605	HUS0712	No	3	Peer pressure
9606	HUS0714	No	3	Peer pressure
